# Machine Learning-Guided Identification of PET Hydrolases from Natural Diversity

**DOI:** 10.1021/acscatal.5c03460

**Published:** 2025-09-03

**Authors:** Brenna Norton-Baker, Evan Komp, Japheth E. Gado, Mackenzie C. R. Denton, Irimpan I. Mathews, Natasha P. Murphy, Erika Erickson, Olateju O. Storment, Ritimukta Sarangi, Nicholas P. Gauthier, John E. McGeehan, Gregg T. Beckham

**Affiliations:** † Renewable Resources and Enabling Sciences Center, 53405 National Renewable Energy Laboratory, Golden, Colorado 80401, United States; ‡ BOTTLE Consortium, Golden, Colorado 80401, United States; § Agile BioFoundry, Emeryville, California 94608, United States; ∥ SLAC National Accelerator Laboratory, Stanford Synchrotron Radiation Lightsource, Menlo Park, California 94025, United States; ⊥ Department of Systems Biology, 1811 Harvard Medical School, Boston, Massachusetts 02115, United States; # Department of Data Sciences, Dana-Farber Cancer Institute, Boston, Massachusetts 02115, United States

**Keywords:** biocatalysis, interfacial biocatalysis, high-throughput assay, machine learning, PET hydrolase

## Abstract

The enzymatic depolymerization of poly­(ethylene terephthalate) (PET) is emerging as a leading chemical recycling technology for waste polyester. As part of this endeavor, new candidate enzymes identified from natural diversity can serve as useful starting points for enzyme evolution and engineering. In this study, we improved upon HMM searches by applying an iterative machine learning strategy to identify 400 putative PET-degrading enzymes (PET hydrolases) from naturally occurring homologs. Using high-throughput (HTP) experimental techniques, we successfully expressed and purified >200 enzyme candidates and assayed them for PET hydrolysis activity as a function of pH, temperature, and substrate crystallinity. From this library, we discovered 91 previously unknown PET hydrolases, 35 of which retain activity at pH 4.5 on crystalline material, which are conditions relevant to developing more efficient commercial processes. Notably, four enzymes showed equal to or higher activity than LCC-ICCG, a benchmark PET hydrolase, at this challenging condition in our screening assay, and 11 of which have pH optima <7. Using these data, we identified regions of PETases statistically correlated to activity at lower pH. We additionally investigated the effect of condition-specific activity data on trained machine learning predictors and found a precision (putative hit rate) improvement of up to 30% compared to a Hidden Markov Model alone. Our findings show that by pointing enzyme discovery toward conditions of interest with multiple rounds of experimental and machine learning, we can discover large sets of active enzymes and explore factors associated with activity at those conditions.

## Graphical abstract

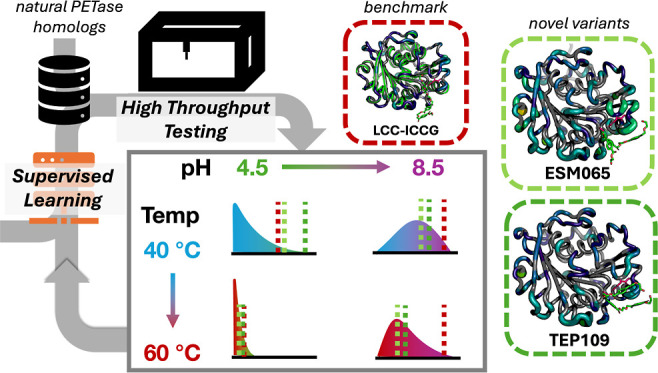

## Introduction

In recent years, biocatalysis has emerged as a promising recycling methodology for poly­(ethylene terephthalate) (PET), an abundantly produced plastic, due to its potential to selectively depolymerize the polymer under mild conditions.^1,2^ A number of naturally occurring hydrolase enzymes have been discovered that catalyze the conversion of PET to its constituent monomers, terephthalic acid (TPA) and ethylene glycol (EG).^3–11^ Most of these PET hydrolases exhibit promiscuous activity related to their native functions as cutinases, lipases, and carboxylesterases. In this study, we use the term ″PET hydrolase″ to denote enzymes with demonstrated PET hydrolytic activity, acknowledging that the observed activity of these enzymes is very likely promiscuous activity. At the time of writing, there are 127 PET hydrolases recorded in the PAZy database and on the order of 250 total known enzymes to have PET depolymerization activity.^12^

Significant efforts have been dedicated to understand and engineer these natural enzyme scaffolds to improve their performance under industrially relevant conditions, especially to operate at thermophilic temperatures (∼65–70 °C).^1,13,14^ Mechanistic studies of structure and dynamics have revealed key factors influencing enzyme performance, including active site binding, flexibility, and the role of individual amino acids in catalysis.^15–18^ Sequence databases and metagenomes have been mined and clustered for homologs, and ancestral sequences “revived” to discover new PET hydrolase scaffolds.^9–11,19,20^ For example, concurrently to this work, Seo et al. developed a natural sequence cluster framework that groups PET hydrolases by sequence similarity and systematically tests representatives from high-performing clusters.^11^ Additionally, engineering campaigns successfully increased the thermal stability and activity of PET hydrolases, achieving high conversion extents on amorphous PET.^11,21–25^ Despite these advancements, the effectiveness of biocatalytic PET recycling remains limited by several factors, including low depolymerization extents for crystalline PET and the acidic conditions generated by terephthalic acid release, requiring the addition of base, significantly increasing the cost of the process.^1,14,26,27^ Starting an engineering campaign from novel scaffolds with activity profiles targeting relevant conditions may help address these limitations.

In this study, we applied three consecutive rounds of machine learning and high-throughput (HTP) experimental characterization to discover PET-active hydrolases. In contrast to an HMM search alone, or the clustering approach of Seo et al., our work uses iterative, supervised machine learning predictors trained on experimental data from the peer-reviewed literature and from our assay to filter candidate sequences, with a focus on challenging conditions including acid tolerance and thermal tolerance. We used high-throughput experimental techniques and an automated liquid handling robot to express and purify over 200 enzymes and screen for activity across a range of conditions, including temperature, pH, and substrate crystallinity.^28^ From this screening, we identified 91 novel (115 total) PET hydrolases with diverse activity profiles, including some with pH optima <7, and with activity at or greater than LCC-ICCG^21^ at pH 4.5 on crystalline powder. Of the candidates tested, we achieved a hit rate of 55% active PET hydrolase and 22% with activity at pH 4.5. We used these data to identify regions of the sequence and surface with potential importance for low pH activity. We further demonstrate that models trained on all three rounds of data have higher predictive accuracy than those that use only literature data or our first two rounds, suggesting that future rounds of enzyme discovery may attain even higher hit rates. We expect that these findings may serve as a foundation for future PET hydrolase engineering efforts and enhance sequence mining strategies for enzymes with activities tailored to specific conditions.

## Methods

### Homolog Identification and Candidate Selection

A profile Hidden Markov Model (HMM) was constructed from a multiple sequence alignment of 61 experimentally verified PET hydrolases identified from PAZy (extracted Sept. 21, 2021). Multiple sequence alignment was performed using MAFFT.^12,29^ The resulting profile HMM was used to search against three sequence databases: NCBI nonredundant protein database, JGI combined hotsprings metagenome database, and MGnify.^30,31^ HMM searches were performed using HMMER (v3.2.1) with a bit score threshold of 100 for both the full sequence score (-T 100) and domain score (−domT 100). The same thresholds were applied to the inclusion thresholds (−incT 100 and – incdomT 100). This initial search retrieved 10,633 putative PET hydrolase sequences.^32^ For round two and three, we used a pool of candidates from more sensitive search using an HMM of 75 PAZy sequences (Mar. 7, 2023) and a search bit threshold modified to 160, yielding 8067 hits. HMMs are provided on Zenodo.

Candidate PET hydrolases were prioritized using a combination of machine learning-based activity and property predictors. In Round 1, a variational autoencoder (VAE) was trained on aligned candidate sequences to learn a continuous latent representation, followed by a supervised rank-based predictor of PET hydrolase activity. In Rounds 2, PET hydrolase activity was instead predicted using an ensemble predictor, which showed better performance than the Round 1 model. See Zenodo for details. In Round 2 and 3, predicted thermostability, PET hydrolase activity, and pH optimum were used for multiobjective selection. All property predictors were benchmarked using cross-validation. Candidate filtering included sequence identity thresholds and clustering to ensure novelty and diversity. Full methods for the models used in selection in Round 1−3 are given in Supporting Information Section S1.

### Protein Expression and Purification

Selected genes were codon optimized for *E. coli* expression, synthesized by Twist Biosciences, and cloned into a pCDB179 expression vector (Addgene #91960, gifted by Christopher Bahl) containing an N-terminal 10xHis-SUMO fusion. Proteins were expressed in *E. coli* C41­(DE3) using autoinduction media in 24-deep well plates. Expression, cell lysis, and nickel affinity purification were carried out using an OT-2 robotic platform as described previously.^28^ SUMO tags were cleaved using an in-house His-tagged SUMO protease. Protein concentration was measured using a BCA assay and normalized to 0.1 mg/mL for downstream applications. Full expression and purification protocols are available in Supporting Information Section S2.

### Thermostability Measurement

Differential scanning fluorimetry was performed on a Bio Rad CFX96 Touch Real-Time PCR machine. 45 μL of enzyme solution (from 0 to 0.3 mg/mL after concentration normalization) was combined with 5 μL of 5X Sypro Orange Dye (ThermoFisher Scientific S6650) in a PCR plate (Bio-Rad HSP9601), which was then sealed (ThermoFisher Scientific 4311971) and the following program was used: 1. 25 °C: 0:15; 2. 25 °C: 0:31; 3. 25 °C: 0:15 (+0.3 °C/cycle, ramp 0.3 °C/s); 4. Plateread, 5. Go to 3, 250X.

### Activity Assays

PET substrates, either amorphous film (Goodfellow ES30-FM-000145) or crystalline powder (Goodfellow ES30-PD-006031) were loaded into 96 deep-well plates (NEST 503501) with 3.5 mg in each well. Amorphous film was cut into 0.34 × 0.34 cm and loaded manually. Powder was dispensed using the Powdernium Automated Powder Dosing System (Symyx). 450 μL of buffer solution (prepared at room temperature) was added. The buffers used were 50 mM NaCitrate for pH 4.5 and 5.5 and 50 mM NaPhosphate for pH 6.5 and 7.5. The enzymes were reordered in a fresh plate and normalized to 0.1 mg/mL if necessary. 50 μL of enzyme solution (5 μg) was added to each well with blanks for each pH containing just the buffer of the enzyme solutions. Aluminum heat sealing foil (Azenta 4ti-0535) as well as autoclave tape around the foil edges was used to prevent evaporation. Plates were incubated at temperature (40 or 60 °C) for 48 h after which they were cooled at room temperature for 30 min then stored at −20 °C. The amount of aromatic product released was evaluated by using absorbance at 260 and 280 nm on 100 μL aliquots of the reaction supernatant. Reactions containing crystalline powder were filtered through MultiScreen_HTS_ GV Filter Plates (Millipore MSGVN2210) prior to analysis. The calculation of product was performed as previously described.^28^

### Crystallography

The purified protein was concentrated to 22 mg/mL and screened through approximately 600 crystallization conditions. The best crystals were obtained from Morpheus screen well A1 (0.06 M Divalents, 0.1 M Buffer System 1, 30% Precipitant Mix 1). Crystals were grown by sitting drops vapor diffusion at 16 °C using a 1:1 ratio of protein to well solution and grew in approximately 2 weeks. Crystals were transferred to a well solution supplemented with 20% glycerol and cryocooled in liquid nitrogen. Diffraction data was collected at the SSRL BL12–2 beamline using Dectris PILATUS EIGER 2XE 16 M PAD detector. The crystals belonged to space group *P*3_2_21 with dimensions *a* = 87.25 Å, 87.25 Å, 148.87 Å, α = 90°, β = 90°, γ = 90°. There are two monomers in the asymmetric unit. All data were processed with XDS.^33^ The structure was solved by molecular replacement using Phaser^34^ and by using a homology model developed from a PETase (PDB code: 8ETY) as the search models. Multiple rounds of manual model building using Coot^35^ and model building using the Buccaneer^36^ program were needed to successfully complete the model. The structures were refined by using Refmac^37^ and manually fitted using the Coot program.

### Sequence and Structure Analysis

The set of enzymes that exhibited nonzero activity in our assay at any condition were aligned with MUSCLE 5.1 with default parameters.^38^ This alignment is provided on Zenodo. The same procedure was used to align active training enzymes for HMMs. To identify determinants of condition-specific activity, we performed multiple sequence and structure-based analyses on enzymes with measurable activity. Conservation of physicochemical residue categories was assessed across performance groups using Jalview,^39^ highlighting alignment columns uniquely conserved under specific conditions (e.g., low pH). Structural models were generated via ColabFold^40^ (mean pLDDT > 90) and aligned using mTM-align. Surface properties—including electrostatics, hydrophobicity, and stickiness—were computed with SURFMAP^41^ and compared between groups at each mapped surface location. Additionally, predicted p*K*_a_ values of titratable residues were analyzed using PROPKA^42^ Significance for surface properties an p*K*_a_ was assessed via Mann–Whitney U tests with *p* < 0.05. Sequence positions prioritized by pretrained model attention (temBERTure, EpHod) were also mapped to the alignment, though not used in the analysis of significant factors presented here. A mapping of all factors computed as mean differences between groups and the values for individual candidates can be found in Appendix B and C^43,44^ Finally, we clustered additional noncatalytic domains by pairwise similarity, identifying a group of low-pH-active enzymes harboring a ricin B lectin-like module. Full analysis methods, statistical tests, and structure processing workflows are described in Supporting Information Section S3.

### Predictor Training and Cross Validation

Experimental data were split into 5 folds by random sampling. The maximum of the maximum observed percent identity between splits is 94%, with a mean of means of 42%. Note that due to differences in condition loading in our assay, some conditions have fewer experimental evaluations per split. This allows us to probe the effect of data size on trained models. Due to some splits for some conditions having few or zero active enzymes, we report scores over cross validated predictions as opposed to cross validated scores, such that the whole data set for each condition produces the score, however the model that made each individual prediction did not see that example during training. We tested HMMs with further training data by adding enzymes with nonzero activity at a particular experimental condition to an initial set of PET hydrolases and computing the score for the same experimental condition. We compared three starting points: HMM-17, HMM-61, and D1-Scraped-513. Scores were normalized to each HMM’s score for LCC-ICCG, to allow HMM scores across data splits to be combined. Sequences of variable length were embedded by the last layer of ESM3 open v1 and mean pooled and given to a random forest of size 100, with default scikit-learn parameters as of version 1.3.2.^45,46^ The supervised models use HMM scores as additional input features as in Hsu et al.^47^

## Results and Discussion

### Putative Thermo- and Acid Tolerant PET Hydrolases Identified Using Bioinformatics and Iterative Machine Learning

We conducted a PET hydrolase homolog screen consisting of three rounds (candidate ID per round: DP for Round 1, TEP for Round 2, and ESM for Round 3, vide infra), each augmented by machine learning on literature data or on previous round data (Figure 1A). A summary of the PET hydrolase data we used for this search is summarized in Table 1. Details for all models and parameters used for searching and filtering candidates can be found in the Methods Section, while an overview is given for each round below.

**Figure 1:**
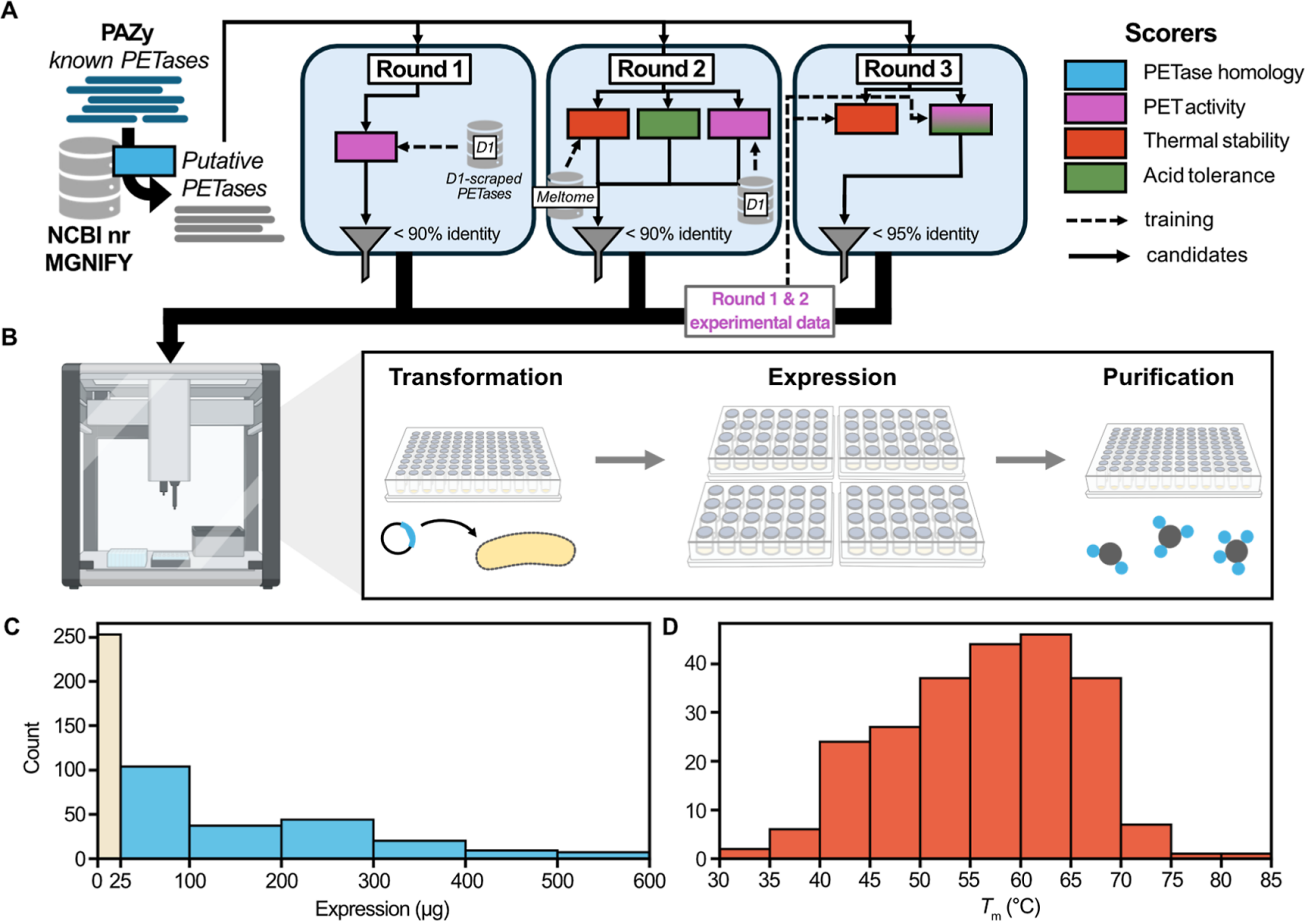
(A) Overview of the process for each round of sequence mining and filtering. Candidates were mined from reference sequences using an HMM of active PET hydrolases. In Round 1, candidates were filtered via a PET hydrolysis activity predictor trained on scraped literature data. In Round 2, the PET activity predictor was improved, and thermal stability and acid tolerance predictors were also leveraged to filter candidates. In Round 3, high quality and uniform experimental data from previous rounds was used to train predictors that we used to filter additional candidates. (B) Transformation, expression, and purification of the putative PET hydrolases assisted by the liquid handling system, OT-2, to achieve tagless purified enzymes via Ni-affinity separation and targeted proteolytic cleavages^28^ (C) Histogram showing the purified yields from a single well of the enzymes studied. Those below 25 μg (beige bar) were below our detection threshold. (D) Histogram showing the thermostability of the enzymes measured by differential scanning fluorimetry (DSF). If multiple inflection points were observed, the highest melting temperature (*T*_m_) is plotted. Portions of this figure were created using Biorender.com.

**Table 1:** Overview of PET Hydrolase Activity Data

name	description	source	supervised ML-trainable
HMM-17	17 characterized PET hydrolases used to construct HMM	Danso et al., 2018; Erikson et al. 2022	no
HMM-61/HMM-75	61/75 characterized PET hydrolases extracted from PAZy used to construct HMM (natural sequences)	PAZy database (Buchholz et al., 2022)	no
D1-scraped-513	513 proteins assayed for PET hydrolase activity scraped from published literature	26 studies	yes
D2-screened-212	212 proteins expressed with 115 active PET hydrolases in this work, 35 active at low pH	This work	yes, multiple conditions

For Round 1 (Enzymes DP001-DP098, 94 total), we first constructed a profile Hidden Markov Model (HMM-61) using 61 experimentally verified PET hydrolases from the PAZy database.^12^ Database searches using this HMM against MGnify and NCBI nonredundant (nr) database identified 10,633 candidate sequences, which were filtered to 8081 nonredundant sequences.^30,31^ We then developed a variational autoencoder (VAE) using the selected sequences and trained a supervised linear top model trained on the VAE latent space using 513 sequences with experimental PET-hydrolase activity from the literature (D1-Scraped-513). A summary of the works that were scraped to produce this data set is given in Table S1. With this approach, we selected 96 novel candidate sequences, identified as the top predictions of the linear model, with less than 90% identity to known PET hydrolases. We moved forward with these sequences for expression and experimental testing as described in the next section.

In Round 2 (Enzymes TEP001-TEP197, 191 total), we used a larger HMM with new additions from PAZy (75 total) and accepted more remote homologs in the search. We filtered these homologs with three machine learning models trained to predict PET hydrolysis activity, thermal stability, and acid tolerance, respectively. For PET activity, we developed a new model, an ensemble of supervised logistic regression and unsupervised scores that outperformed the evolutionary VAE from Round 1 in 5-fold cross-validation on the D-513 scraped data set. Homologs were also scored by a thermal stability predictor developed in house, trained on embedding outputs from the protein language model ProtT5 and melting temperatures from the Meltome Atlas, as well as for acid tolerance by EpHod (Figure S1).^43,48^ To ensure broad exploration, candidates for Round 2 were selected as those that maximized any individual score, or are above the 50th percentile for all three, and have less than 90% identity to known PET hydrolases and to each other.

In Round 3 (Enzymes ESM001-ESM191, 190 total), we incorporated activity data from Round 1 and 2 to create supervised models specifically trained to predict activity and melting temperature from our assay. Several embedding methods were tested, finding that ESM1v mean pool embeddings performed best in cross validation (Spearman correlation of 0.537 against max observed activity) with a ridge regressor. Candidates were selected based on the absolute value of predicted activity of 10 mM product/mg enzyme, > 55 °C predicted melting temperature, and sequence identity of <95% to each other and previously reported PET hydrolases.

### High-Throughput Experimentation Characterizes a Diverse Set of Active PET Hydrolases

The selected genes were commercially synthesized and cloned into the vector pCDB179 with an N-terminal His-tag and small ubiquitin-like modifier (SUMO) fusion to the target protein. The plasmids were transformed into chemically competent *E. coli* and expressed and purified using a HTP, semiautomated platform described previously.^28^ An Opentrons liquid handling robot (OT-2) was used to facilitate the expression and purification in multiwell plates (Figure 1B). Expression occurred in 2 mL autoinduction cultures in 24-deep well plates. The cultures were harvested and resuspended in a detergent-based lysis buffer and purified via affinity chromatography using Ni-charged magnetic beads. SUMO protease was added to cleave the target proteins from the magnetic beads, yielding the tagless protein into the supernatant. Purified protein concentrations were evaluated using the bicinchoninic acid (BCA) assay and wells containing sufficient yields were normalized to 0.1 mg/mL via dilution. We observed the threshold of detection above background to be 0.1 mg/mL using the BCA assay, and we considered samples at or above this threshold to be successfully purified. The purity of select enzyme samples was assessed using sodium dodecyl sulfate–polyacrylamide gel electrophoresis (SDS–PAGE) (Figure S2).

In total, 475 unique sequences were transformed, expressed, and purified. Of these, 221 (47%) successfully purified and achieved yields greater than or equal to a 0.1 mg/mL threshold (Figure 1C). Those that yielded below threshold (254 total, 53%) may have failed due to poor expression, poor solubility, or incomplete cleavage of the target protein from the magnetic beads. The highest yield reached was approximately 600 μg from a single well. The average yield of successfully purified enzymes was 167 μg or an 84 mg/L titer.

The thermostability of the purified enzymes was determined using differential scanning fluorimetry (DSF) using Sypro Orange as an unfolding indicator. Due to the high sensitivity of DSF, which requires minimal protein and can measure melting temperatures even below the concentration detection threshold, all expressed enzymes were evaluated for thermostability regardless of recorded yields. In total, 233 enzymes demonstrated measurable melting temperatures (*T*_m_), with observable *T*_m_ values for some samples with concentrations below 0.1 mg/mL (Figure 1D). The range of *T*_m_ values observed was 25.3–82.3 °C and the average *T*_m_ for all samples with measured values was 56.3 °C. A comparison of the expression yield as a function of *T*_m_ did not reveal a strong correlation (Figure S3).

Successfully purified enzymes were carried forward to determine their depolymerization activity on PET. Of the 221 successfully purified enzymes, 212 were tested for activity with several being excluded due to insufficient volume to meet the minimum number of test conditions. We loaded 96-deep well plates with amorphous PET film (aFilm) or crystalline PET powder (cryPow), both sourced commercially from Goodfellow with reported crystallinities of 4.0 ± 2.0%, and 39.3 ± 2.0%, respectively.^9,49,50^ Buffer was added for the pH levels tested as follows: 4.5 (NaCitrate), 5.5 (NaCitrate), 6.5 (NaPhosphate), 7.5 (NaPhosphate), and 8.5 (glycine). The PET solids loading was 3.5 mg in a 0.5 mL reaction (0.7 wt/v %). The enzyme was added (5 μg) to reach a final enzyme loading of 1.43 mg/g PET. The plates were mixed briefly via shaking, heat sealed with foil, and incubated at either 40 or 60 °C without shaking for 48 h.^51^

Enzymes were grouped by yield to determine the number of conditions possible to test. Those with high enough yields were tested in 32 conditions (4 pHs, 2 temperatures, 2 PET substrates, in duplicate) (Figure 2A). Enzymes yielding less were tested in 8 conditions (4 pHs, 2 temperatures, 1 PET substrate (cryPow)) (Figure 2B) and those with the lowest yields were tested in 4 conditions (4 pHs, 1 temperature (40 °C), 1 PET substrate (cryPow)) (Figure 2C). In Round 1, the pH levels tested were 5.5, 6.5, 7.5, and 8.5. In later rounds, lower pHs were targeted and the values tested were 4.5, 5.5, 6.5, and 7.5. The assays were analyzed using ultraviolet–visible (UV–Vis) spectroscopy of the aromatic products released: TPA, mono­(2-hydroxyethyl) terephthalate (MHET), and bis­(2-hydroxyethyl) terephthalate (BHET).^28,52^

**Figure 2:**
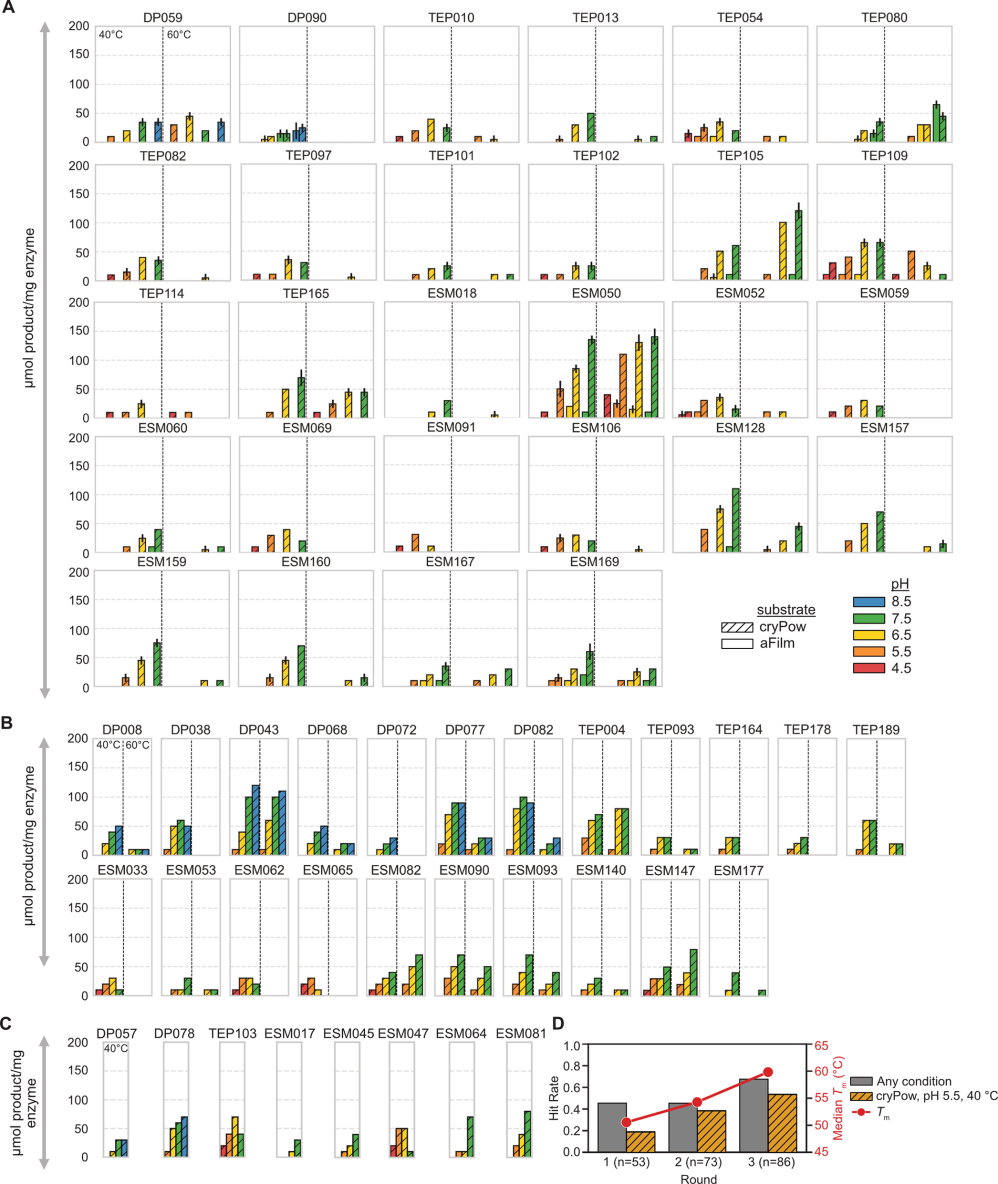
PET hydrolase activity in μmol aromatic products produced per mg of enzyme after 48 h added at varying pH (color of bar), temperature (left vs right for each enzyme), and substrate crystallinities–amorphous film (aFilm, solid bars) and crystalline powder (cryPow, hatched bars). Only enzymes with activity above 25 μmol product/mg of enzyme in at least one condition are shown. (A) Enzymes with yields sufficient to test in 32 conditions: 4 pHs, 2 temperatures, 2 PET substrates, in duplicate. Error bars represent the range between biological duplicates. (B) Enzymes with yields sufficient to test in 8 conditions: 4 pHs, 2 temperatures, 1 PET substrate (cryPow), based on single measurements. (C) Enzymes with yields sufficient to test in 4 conditions: 4 pHs, 1 temperature (40 °C), 1 PET substrate (cryPow), based on single measurements. (D) Observed hit rate for active PET hydrolases (the ratio of active PET hydrolases to the total number assayed for activity) and median *T*_m_ across the three rounds of mining, filtering, and testing. Gray bars represent the hit rate for enzymes showing activity under any condition tested; however, not all enzymes were tested under every condition. Orange bars represent the hit rate for active enzymes on crystalline powder at pH 5.5 and 40 °C, a targeted condition where all enzymes were tested.

Overall, 115 active enzymes were identified, or 54% of the 212 tested. Activity was defined as an aromatic absorbance reading at 260 nm of 0.05 above the blank in at least 1 condition. The number of active enzymes in different condition categories is given in Table 2. Figure 2A–C displays the 58 most active enzymes with activity in at least 1 condition above 25 μmol product/mg enzyme. The activity profiles for all active enzymes are shown in Figures S4–S6, as well as a comparison to a benchmark enzyme, LCC-ICCG, which was included as a positive control in all assay plates.^21^ Compared to LCC-ICCG, an engineered PET hydrolase with very high activity on amorphous film at high temperatures but limited activity at low pH conditions,^21^ the active enzymes identified in this study display moderate to low activity near LCC-ICCG’s optimal conditions. However, several showed superior performance at the challenging pH 4.5 condition, which we targeted for increased industrial relevance. Despite their comparable or superior performance to the benchmark at low pH, we anticipate that further engineering will be required to achieve activity levels sufficient for meaningful bioreactor studies. For PET hydrolases identified here that were tested on both substrates, they appear in most cases to be more active on crystalline PET powder than amorphous PET film. This observation may be due to the increased surface area, different surface properties, and availability of initial and easily cleavable noncrystalline regions in the powder versus the film.

**Table 2:** Distribution of Active Enzymes Across Varying Conditions

condition type	condition pair (cond. 1/cond. 2)	active only in cond. 1^a^	active in both^a^	active only in cond. 2^a^
pH	4.5/7.5	11	24	57
temperature	60/40	0	34	45
substrate	cryPow/aFilm	36	9	0

a Enzymes not tested at both conditions are excluded from the count.

Our iterative machine learning approach led to notable performance improvements across successive rounds, as reflected in increased hit rates for activity in any condition tested (44% in Round 1 to 67% in round 3), increased hit rates for activity specifically at low pH (crystalline powder pH = 5.5, 40 °C, 19% in round 1 to 59% in Round 3), and higher average thermal stability (52.8 °C in Round 1 to 59.0 °C in Round 3), as shown in Figure 2D. Full counts of number of enzymes tested and hit rates for all condition sets over each round are given in Table S2. We note that the interpretation of the hit rate for activity in any condition is complicated by the fact that not all enzymes were tested in all conditions. For low pH, the pronounced improvement in hit rate for Round 3 was likely due to the addition of supervised predictors trained on low-noise assay data from previous rounds, unlike earlier rounds which relied on globally trained predictors of PET hydrolase activity data from disparate experiments in literature (D1–513-scraped). The hit rate increase was more pronounced for crystalline powder activity, for which we tested the most homologues. In Rounds 2 and 3, the hit rate and average *T*_m_ increase is likely due to the addition of dedicated predictors for acid tolerance and thermal stability, including EpHod and *T*_m_ predictions.^43^ Many enzymes exhibited higher activity at the lower temperature tested (40 °C), likely due to their lower thermostability and destabilization at higher temperatures. For enzymes with higher thermostability, such as ESM050 (*T*_m_ = 82.3 °C), higher activities were observed at the higher temperature (60 °C). Generally, temperature enhances catalytic rate as long as the enzyme remains stable and active at those temperatures. As the median value of *T*_m_ in the enzymes studied was ∼ 57 °C, it is unsurprising that most showed reduced activity at 60 °C.

From the PET hydrolases that were successfully purified and assayed for PET hydrolytic activity, we analyzed the sequence diversity (Figure 3). A phylogenetic tree based on sequence similarity of all sequences that showed activity along with previously reported PET hydrolases is given in Appendix A, where many of the sequences tested fall in the phylum Actinomycetes. A subset of that tree is shown in Figure 3A, including a few commonly studied PET hydrolases and the candidates that we found with >20 μmol product/mg of enzyme conversion at pH 5.5, crystalline powder, 40 °C. We additionally computed a 2D uniform manifold approximation (UMAP) based on pairwise negative BLOSUM62 scores between active PET hydrolases in this study and those from previous studies in Figure 3B.^9–11^ Figure S7 depicts the space of this mapping for each previous study separately. We observe that the PET hydrolases from this work are generally within the manifold of known enzymes, although we saturate some areas that previously had few examples. Of the 115 PET hydrolases found to be active in this study, 23 were recently described by Seo et al.,^11^ and many but not all of our low pH active candidates fall within the cluster they identified as most performant. In general, candidates with nonzero activity at low pH appears to congregate. All sequences shared a catalytic domain with the Asp-His-Ser triad within the conserved α/β hydrolase fold, as shown by the alignment of Cα atoms for each Alphafold2 predicted structure against LCC-ICCG using mTm-align (Table S3).^53^

**Figure 3:**
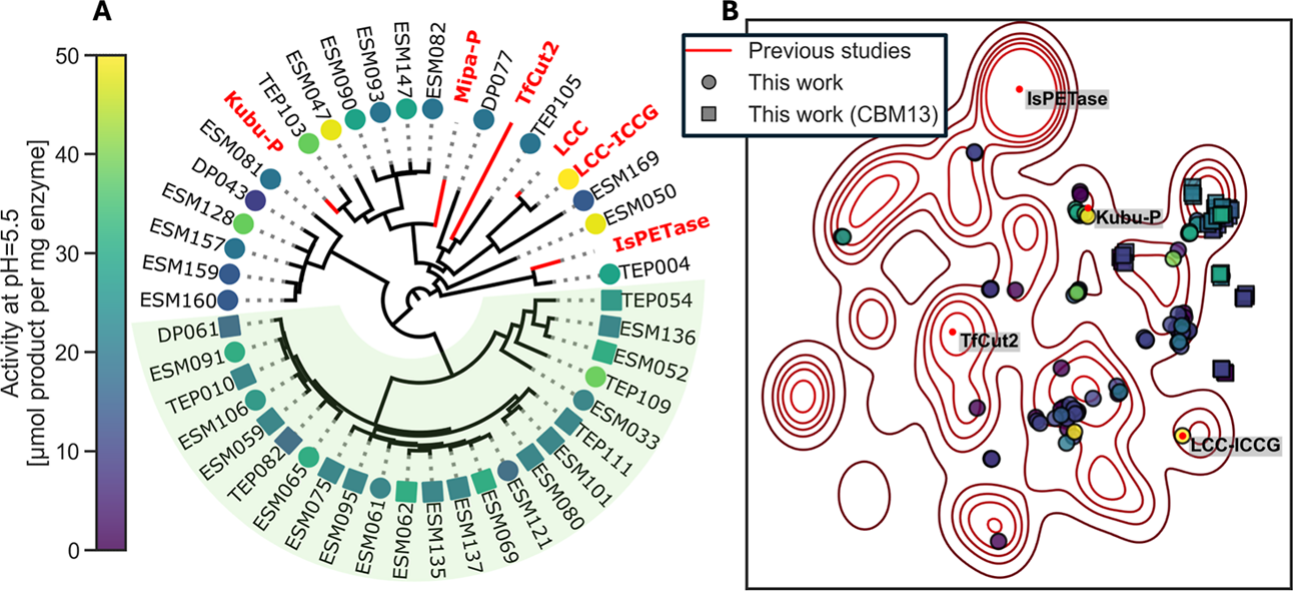
Sequence diversity of the PET hydrolases studied. (A) Minimum evolution phylogenetic tree computed using a multiple sequence alignment of all PET hydrolases with >50% gap columns removed. Extra domains were not included in the tree distance calculation due to the high gap content. Only candidates with greater than 20 μmol product/mg of enzyme conversion activity at pH 5.5 crystalline powder at 40 °C are shown, along with DP043. Enzyme activity is represented by marker color. Select enzymes from other studies are shown. Squares are enzymes with a Family 13 carbohydrate binding module (CBM13) identified by dbCAN2, and the background highlight shows a branch of the tree with many enzymes exhibiting a CBM13.^54^ (B) 2D UMAP plot using BLOSUM62 scores from an alignment of PET hydrolases from this work (circles) overlaid on the space of known PET hydrolases (red kernel density). Actives with CBM13 are depicted with squares. High-performing or widely studied PET hydrolases are depicted as red dots.^4–6,11,21^

A subset of enzymes were noted to contain additional carbohydrate binding domains (CBMs), which we labeled with dbCAN2.^54^ Interestingly, those with CBM13 were more likely to be active at pH 4.5, with 50% of CBM13 enzymes active in this condition compared to 17% of all tested enzymes active in this condition. These CBM13-containing enzymes are likely components of enzyme cocktails for the interaction with or breakdown of natural polyesters, such as cutin and suberin found in proximity to carbohydrates, for example in the plant cell wall.^55^ These are marked with squares in Figure 3A,B where they occupy a single branch on the tree (outlined) and cluster in 2D sequence space, based solely on the tree calculation using only the catalytic domain. A subcluster of the CBM13 domain, labeled CBM13­(Sub) and annotated as ricin B lectin, had an even higher hit rate at pH 4.5 with 11 out of 13 enzymes active (85%) as shown in Figure S8. The mechanism of this trend is unclear, but it may be influenced by evolutionary pressures on the CBM being coupled to other properties that contribute to pH tolerance. While acknowledging that the enzymes explored here are not the entirety of known sequence space, in conjunction with PET hydrolase searches in the literature to date, our findings suggest that moderate PET hydrolysis activity is prevalent across enzyme classes and considerable sequence distances. This is consistent with the hypothesis of diverse promiscuity for this substrate.

To provide a basis for structural investigations, we selected the most performant enzymes from our Round 1 candidates that also expressed well in the small-scale experiments. Expression of these enzymes was scaled-up, and one enzyme, DP043 from *Actinoplanes* sp. DH11, was successfully crystallized, with the crystal structure being determined using molecular replacement (Table S4 and Figure S9A). The crystal structure aligns closely with other known PET hydrolases with an all-atom RMSD of 0.827 Å from LCC-ICCG (PDB: 6THT) and 0.512 Å from the Alphafold structure of its closest identified homologue in the HMM search, enzyme 407 from Erickson et al. (Figure S9B).^9^ While multiple AlphaFold models have been shown to closely correlate with independent crystallographic models of these compact α/β folds,^9^ we wanted to validate this here. Alignment of the DP043 AlphaFold3 and crystallographic models shows close alignment, including side chains, with an all-atom RMSD of only 0.266 Å and accurate prediction via Alphafold3 of the location of the Ca^2+^ ion observed in the crystal structure (Figure S9C). The crystal structure revealed a Ca^2+^ ion, a feature known in other PET hydrolases which has been targeted for rational engineering due to its role in stabilizing the protein. The Ca^2+^ in the crystal structure of DP043 was not located in the same position as observed in the engineered examples, such as LCC and TfCut2 (Figure S9D) and further studies would be required to evaluate if the Ca^2+^ in DP043 serves a similar purpose.

### Sequence and Surface Properties Associated with Low pH Activity

Using the activity data collected at multiple pH conditions, we explored differences between enzymes active at low pH and those active only at higher pH levels. We split the enzymes into those two groups with 35 active at low pH (pH 4.5) and 57 active only at higher pHs to evaluate differences in sequence conservation, predicted p*K*_a_, and surface properties. For each residue in the alignment of all sequences, we determined any statistically significant (*p* < 0.05) differences for these properties (conservation, p*K*_a_, electrostatics, hydrophobicity, circular variance), with further details provided in the Methods. Table S5 contains all significant factors identified mapped back to LCC-ICCG and the alignment of all active sequences. Appendix B shows per-residue distributions of each property where a significant difference between performance groups was found.

For each candidate, we counted how many of these significant factors were observed and compared that number to its activity at pH 4.5 in Figure 4A, where we see a Spearman correlation of 0.53 (*p* < 0.05). Looking specifically at amino acid conservation, we found 9 sequence positions that were more conserved for enzymes with low pH activity than for those with only neutral activity (Figure 4B). The distribution of amino acids at these positions is given in Appendix B. Significant differences were also observed for hydrophobicity, measured using the Kyte-Doolittle method.^56^ Most of the surface tended to be more hydrophobic for low pH active candidates than neutral activity, seen in Figure 4C, where we show the average shift. This trend is exacerbated near the substrate binding site such as A62, V177 and I208 on LCC-ICCG which each had a shift of 1.0 units or more for low pH active enzymes. The observed properties for two enzymes with pH optima <6, ESM065 (which showed equivalent activity to LCC-ICCG at pH 4.5) and ESM091 are given in Figure 4D,E. Both exhibited more factors that were associated with low pH activity than the average for inactive candidates, have >6 out of 9 of the conserved residues identified, and have a hydrophobic region near the binding site. Mappings of all properties where differences are found and the observed properties for all candidates are shown in Appendix C. It is important to note that these trends are not absolute; for instance, although low pH activity was generally associated with a decrease in surface electrostatics, TEP109, exhibits a positive surface charge, and has a lower total count of factors than would be expected given the trend in Figure 4A. Further testing with, for example, adsorption assays may help elucidate mechanistic interpretations for these identified correlations.

**Figure 4:**
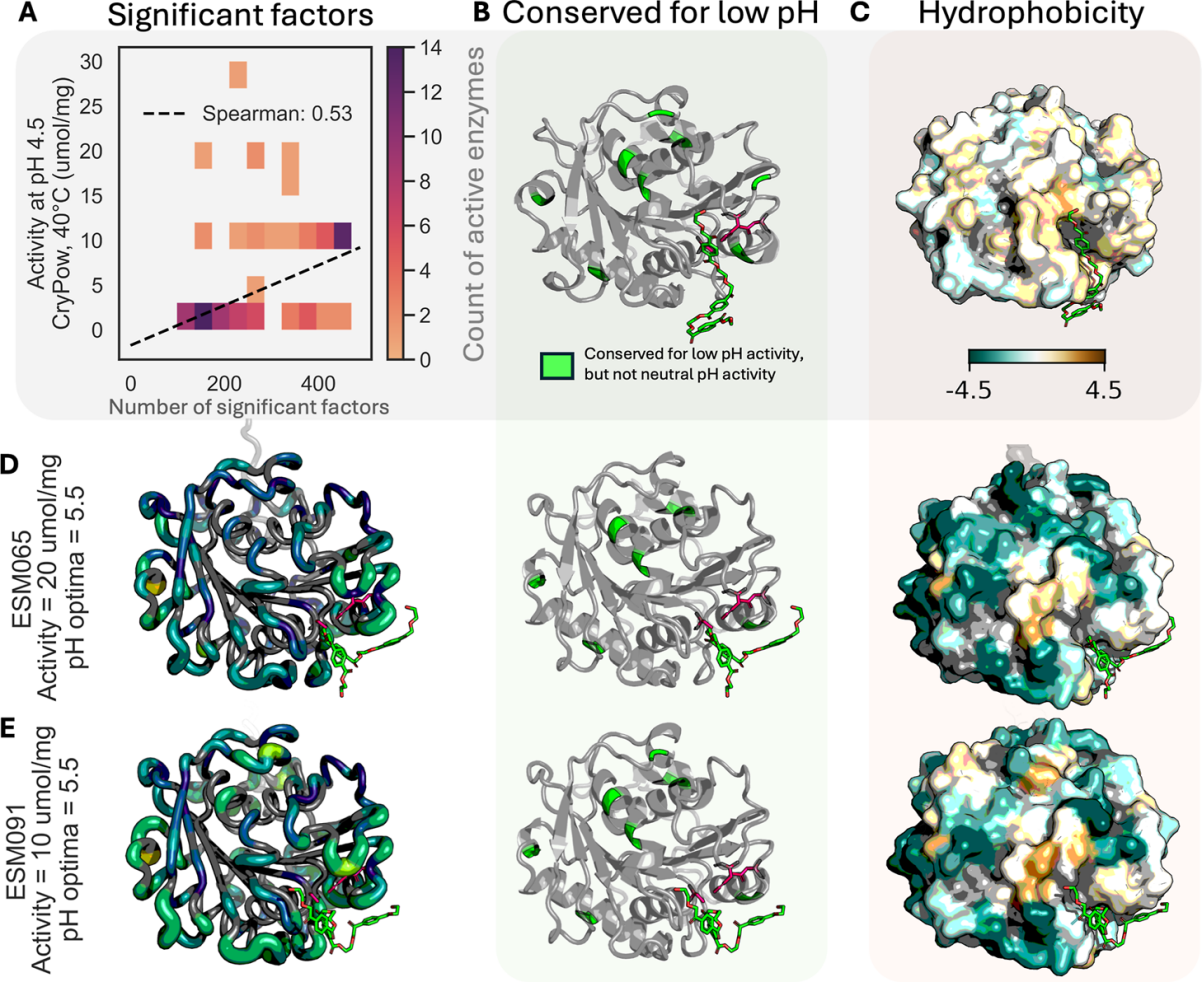
Significant sequence and surface properties associated with low pH activity. All 3D structures show a trimer of PET in green and the three catalytic residues (His, Asp, Ser) in pink. AlphaFold3 was used to produce the structures.^57^ (A) The relationship between the number of factors held by an enzyme as a function of its experimental activity for crystalline powder, pH = 4.5, *T* = 40 °C, where enzymes with more observed factors tend to have higher activity. (B) Nine residues mapped to LCC-ICCG that were more conserved for low pH activity than they were for neutral activity. (C) Mean difference in surface Kyte-Doolittle hydrophobicity, again mapped to LCC-ICCG, between the low pH active group and the neutral activity group, only colored for positions where statistical significance was observed. Most of the surface had a statistically significant shift toward more hydrophobic for acid tolerant candidates, most strongly near the binding site. (D) Factors exhibited by ESM065, which had comparable activity to LCC-ICCG at pH 4.5 and a pH optima <6. Left: counts of factors (conservation, p*K*_a_, electrostatics, hydrophobicity, circular variance, and stickiness) observed by ESM065 that were statistically significant when acid tolerant and neutral candidates were compared. Of all factors found to be significant when comparing these groups, those exhibited by ESM065 (if the ESM065 value is closer to the acid tolerant mean) are marked. Larger and brighter regions of the protein backbone indicate more factors (max 6). Middle: Residues that tended to be conserved for acid tolerance that we observed in ESM095 (6 out of 9). Right: hydrophobicity for ESM095, only colored for positions that where significant differences were found. (E) Same as in (D), but for ESM091, another representative from the 11 candidates with pH optima <6.

We additionally identified 13 positions in the catalytic domain with significantly different predicted p*K*_a_s between enzymes active at low pH (4.5 and 5.5) compared to those with activity only at higher pH (6.5, 7.5, 8.5), five of which occur in or in close proximity to the catalytic triad, as shown in Figure 5. For the low pH active enzymes, on average the catalytic histidine exhibited a negative shift of −0.37 p*K*_a_, suggesting more deprotonation at low pH for acid tolerant candidates, potentially facilitating an easier proton transfer from serine as it attacks the substrate nucleophile. The trend for the catalytic aspartate was an increase in average p*K*_a_ by 0.26, which differs from findings for serine proteases with a similar mechanism where it is suggested that the deprotonated aspartate helps position the histidine.^42,58^ It is possible that this is impacted by or offset by a number of other observations. First, the downstream residue from serine is a histidine in 94% of active enzymes (position 300 in the multiple sequence alignment), and when histidine it exhibits a large positive predicted p*K*_a_ shift of 1.5 for acid tolerant candidates. This histidine may be serving a similar purpose to the catalytic aspartate from the opposite side and protonation state. We also note three other residues within 15 Å (using the TEP109 predicted structure) of the catalytic histidine that are frequently charged in the PET hydrolases studied here. Given that electrostatic potential scales inversely with distance, these positions likely contribute to the p*K*_a_ of active site residues. The residue directly downstream of the catalytic histidine (aligned position 383) is aspartate in 80% of acid tolerant candidates compared to 13% in neutral active candidates. All enzymes with pH optima <6 have this aspartate. There is also a position about 9 Å from the active site (aligned position 224), and in close proximity to H300, that is leucine and uncharged in 94% of examples. The only exceptions are TEP109 (the top performer at pH 4.5), two other enzymes with activity comparable to LCC-ICCG at pH 4.5, and another two that were acid tolerant that have a charged glutamic acid in this position. It is possible that this glutamic acid (Glu224) is contributing to the positive shift of His300 and cascading to the negative shift of the catalytic proton transferring histidine.^59^ Finally, alignment position 378, about 14 Å away is nearly always glutamic acid for active candidates, except in a few examples, 4 out of 5 being acid tolerant, and including TEP109 where it is an uncharged glutamine. A depiction of the active site of TEP109, which exhibits all these features and demonstrated the highest activity at pH 4.5 is given in Figure 5. Further testing, such as with a mutational scan, may establish causation to the p*K*_a_ correlations identified in this data set.

**Figure 5:**
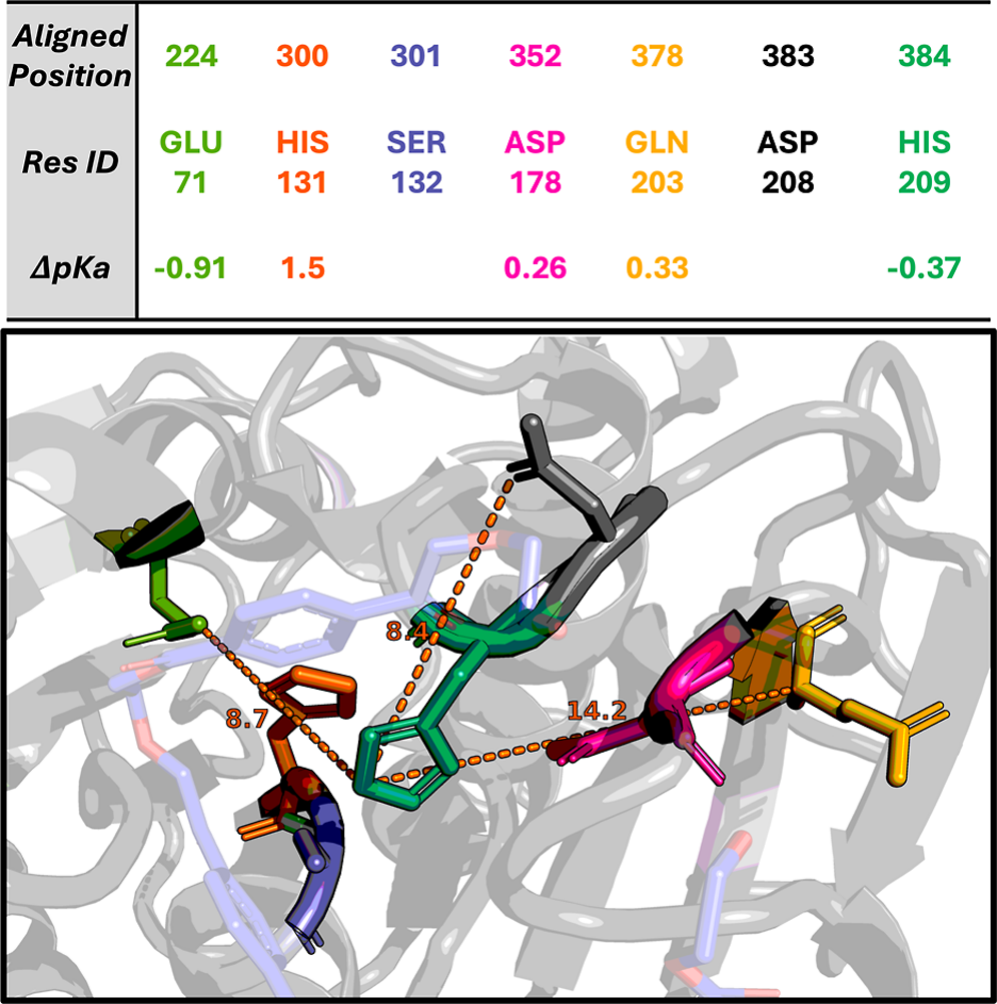
Residues in or near the active site with statistically significantly different predicted p*K*_a_ values between examples with low pH activity and those with activity at higher pH, mapped to the structure of TEP109. All residue positions are given relative to the MSA alignment. The difference in predicted p*K*_a_ mean values between those groups where it was found to be statistically significant is given in the third row. These include the proton transferring histidine (H384, teal), catalytic aspartate (D352, pink), and a histidine (H300, orange) directly adjacent to the catalytic serine (S301, purple). Three other residues within 15 Å that are often or sometimes charged are highlighted (alignment positions: E224 (green), D383 (black), Q378 (yellow)). AlphaFold3 prediction was used to “dock” a trimer of PET (blue), and all distances (Å) shown use the TEP109 predicted structure.^57^ TEP109 is the top performing candidate at pH 4.5 and one of a very small number to exhibit E224 and an uncharged amino acid at alignment position 378.

### PET Homolog Activity Predictors Applied to Substrate, pH, and Temperature

We next used our data to probe the ability of HMMs and supervised activity predictors like those used in experimental Rounds 1–3 as a function of condition and data set size. First, we tested three different HMMs (the “starting” HMMs) for their ability to distinguish active from nonactive candidates measured by area under the receiver operator curve (AUROC): HMM-17, which was used in the search in Erickson et al., HMM-61, which was used for the Round 1 search in this work, and an alignment of D1–513-Scraped, the largest set of known active PETases at the time. Results for select conditions are shown for in Table 3 below. We observed that performance varied across conditions and was sensitive to the PET hydrolases included in the search HMM, with values ranging from worse than random (<0.5) to >0.85 (Table S6). We found that HMM-17, which included mostly thermally stable PET hydrolases tested on amorphous and analog substrates tended to outperform the HMMs with larger sets of active PET hydrolases from the literature, particularly for high temperature activity on amorphous film. Performance of all HMMs is no better than random for crystalline powder and acidic conditions.

**Table 3:** AUROC Scores of Starting HMMs Against Data in This Study

test set	HMM-17	HMM-61	D1-scraped-513
D1-scraped-513	0.592	0.662	
cryPow, 40 °C, pH 5.5	0.470	0.474	0.473
aFilm, 60 °C, pH 7.5	0.871	0.770	0.795

We further looked to investigate the utility of our data collected across different conditions (in this study, various pHs, temperatures, and substrates) for enhancing our predictors. First, we tested “tuning” the HMMs by adding candidates that exhibited activity at the target condition, testing in 5-fold cross validation. We were able to increase performance by more than 0.05 AUROC for multiple conditions by tuning HMMs with data from our assay. The effect of tuning each HMM across conditions is shown in Figure S10. Notably, HMM-17 exhibited a pronounced improvement compared to the larger HMMs, which were saturated with PET hydrolases not necessarily active under the condition of interest. These results suggest that HMM searches could benefit from using a smaller, more targeted set of enzymes tested at the specific condition, rather than constructing large alignments based on data collected across varying conditions.

Next, we used the data collected in this study to train supervised models, like the activity predictor used in Round 3, in cross validation for each condition. We compared the performance of these models against HMM-17, tuned HMM-17, and the supervised model trained on literature data and used in Round 2. Figure 6A and Table S7 show the performance of all models for conditions where at least 10 active enzymes were found. Other conditions are not included as not enough data were available. In general, we see that for conditions where we collected more data (cryPow, 40 °C, pH 4.5–7.5), the tuned and supervised models performed better (at least 0.1 AUROC increase) compared to the starting HMM-17 or the Round 2 supervised predictor (“lit. supervised”). Furthermore, supervised training consistently outperformed HMM tuning. For some conditions, (such as cryPow and pH < 6), HMM-17 performs no better than random; however, the condition-specific supervised models can salvage AUROC of up to 0.8. There is a clear decrease in supervised model performance for conditions where fewer enzymes were tested, with some instances where performance was lower than the starting HMMs. These results suggest that for mining enzyme sequences for specific activities, experimental measurements at a condition of interest for a starting set of homologs should be used to evaluate supervised performance compared to the HMM. Once supervised performance is greater than the HMM alone, such predictors should be used to filter searches in a feedback loop.

**Figure 6:**
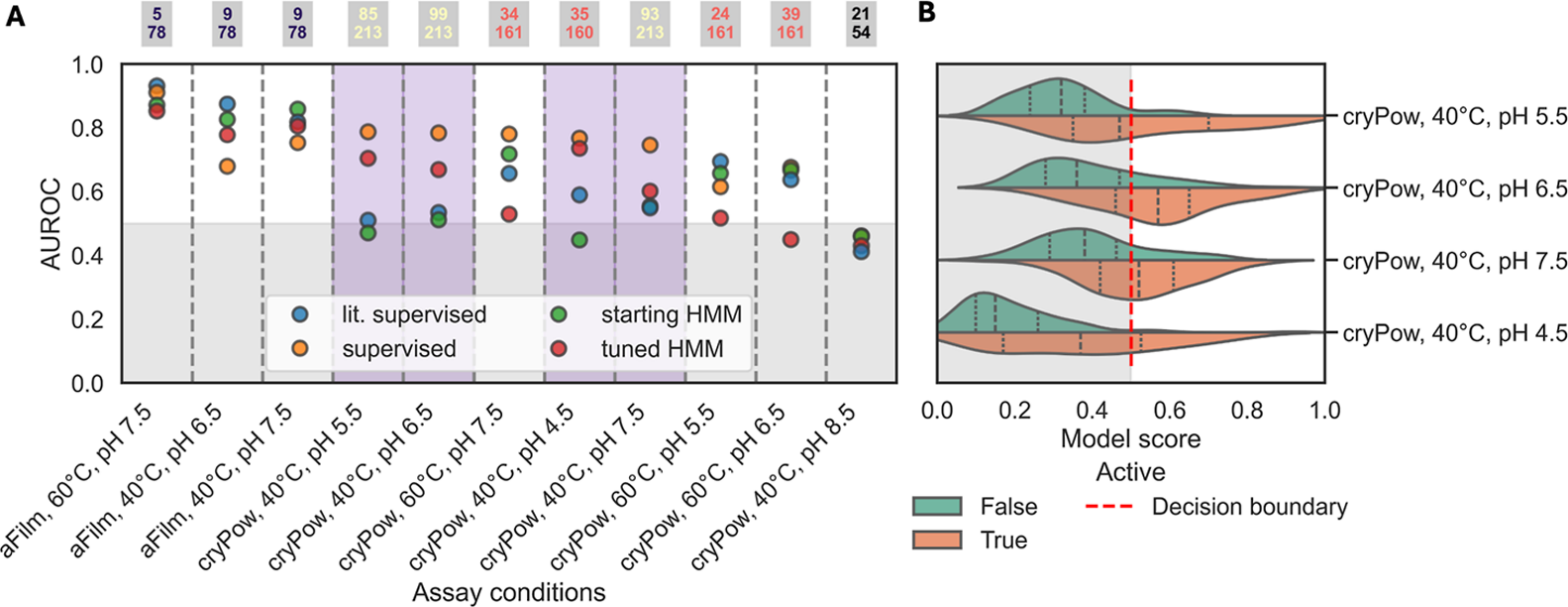
Performance of models across conditions. (A) Scores of supervised models compared in 5-fold cross validation to the starting HMM-17, tuned HMM-17, and the supervised model trained on D1–513-Scraped used in Round 2 (“lit. supervised”). The numbers above each condition show the number active (top) and number tested (bottom). Conditions where >0.1 AUROC improvement by the supervised models was observed are highlighted in purple. (B) Prediction by the supervised model as a function of measured enzyme activity at conditions where high performance was achieved. Decision boundary was set at 0.5, with enzymes above classified as “active” and those below as “inactive”. Enzymes that were experimentally determined as active are orange and those inactive are green.

Finally, we investigated whether these supervised models had the potential to improve hit rates if applied to search for novel enzyme candidates by evaluating their performance on the existing data set. Figure 6B presents the model predictions compared to observed activity in cross-validation for conditions with high-performing models (cryPow, 40 °C, 4.5–7.5). Figure S11 shows performance for all models. We set the decision boundary at the default threshold of 0.5, with those enzymes classified higher predicted as “active” and lower predicted as “inactive”. We observed for each condition a high precision score (the proportion of true positive predictions out of all positive predictions made by the model) of >75%. Specifically, for cryPow, 40 °C, pH 5.5, the precision score was 75%, which represents a putative increase in hit rate of +25% from the observed hit rate in Round 3, which used fewer training data and did not distinguish between conditions, and +55% compared to Round 1, which did not use condition-specific assay data (Figure 2D). The results indicate that supervised models have strong potential for identifying homologues while minimizing the screening of inactive enzymes.

## Conclusion

The expansion of genomic databases, along with significant advances in machine learning, bioinformatics, and HTP screening, offer the potential to drive major progress in enzyme discovery. This study showcases how these tools can accelerate the identification of new variants of PET-degrading enzymes with unique activity profiles across substrates, pH, and temperature from the promiscuous enzymes in nature. By leveraging both PET hydrolase specific data sets and large protein data sets, we showed that candidate enzymes can be saturated toward activity at a condition of interest—particularly those with enhanced thermostability and lower pH optima. Notably, we observed an average *T*_m_ near 60 °C and multiple PET hydrolases with pH optima <6 and activity at pH 4.5 on crystalline powder that is equal to or greater than the activity of LCC-ICCG. We further showed that once a sufficient number of candidates have been tested in a condition of interest, the hit rate can be progressively improved through informed selection for that condition. In contrast to traditional homologue clustering screens, we propose a condition-focused, targeted approach that combines iterative screening and supervised learning to guide the discovery of engineering targets tailored to industrially relevant environments. This approach not only increases efficiency but enables the generation of large uniform data sets suitable for analyzing sequence-function relationships. We expect this framework will support the discovery of more efficient biocatalysts for plastic recycling and other applications.

## Supplementary Material











## Data Availability

The Supporting Information contains Figures S1–10 and Tables S1–7. The Source Data file contains expression, melting temperature, and activity data for all candidates, source data for Figures 1–6, predicted structures for all candidates, and alignments for HMM-17, HMM-61, D1–513-Scraped. Jalview annotations for differential conservation on the alignment when comparing acid tolerant to neutral active candidates is also included. Appendix A provides a minimal distance taxonomic tree of all PET hydrolase sequences and variants shown in Figure 3. Appendix B provides distributions of amino acids, predicted p*K*_a_ values, and surface properties for acid tolerant versus neutral active candidates when found to be significant. Appendix C shows 3D structure renders for all active candidates with observed significant factors depicted. The scripts used to run metagenome searches, train global predictors, and use them for filtering putative PET hydrolases in Round 1–3 are provided on Zenodo including the VAE used in Round 1.^60^ A few of the components can be found independently from the overall workflow on GitHub, including the supervised PET hydrolase activity model trained on D1–513-Scraped and used in Round 2: https://github.com/jafetgado/PETML. The thermostability predictor trained on MeltomeAtlas using PLM embeddings can be found on GitHub at https://github.com/jafetgado/ThermoPalm. The analysis of the final data set for hit rate, conservation, and downstream learning capabilities, and resulting data are available in compressed form.^61^ The pipeline is tracked in Data Version Control (DVC). The entire process of analyzing performance groups, tuning HMMs, training and evaluating supervised predictors etc. can be executed in a single command after following installation instructions by calling ‘dvc reprò’.

## Notes

Supporting tables and figures, including sources for literature PETase data, experimental observations, machine learning performances, and supplemental expression and computational methods (PDF)
Expression, *T*_m_, and activity data for all enzymes tested (XLSX)
Sequence similarity taxonomic tree with phylum information for candidates in this study and from literature (PDF)
Observed distributions of features between low pH and neutral activity groups for all aligned residue positions with statistical significance (PDF)
3D structure renders for all active candidates with observed features (PDF)
