# Supplementary material for: Machine Learning-Guided Identification of PET Hydrolases from Natural Diversity
Source: ACS Catal. 2025 Sep 3;15(18):16070–83. doi: 10.1021/acscatal.5c03460 (PMC12455559; doi:10.1021/acscatal.5c03460)
Supplement: Supplementary file 1 [file cs5c03460_si_001.pdf]

Supporting Information

Machine learning-guided identification of PET hydrolases from natural diversity

Brenna Norton-Baker,[a,b,c,†] Evan Komp,[a,c,†] Japheth E. Gado,[a,b] Mackenzie C.R. Denton,[a,b] Irimpan I. Mathews,[d] Natasha P. Murphy,[a,b] Erika Erickson,[a,b] Olateju O. Storment,[a,b] Ritimukta Sarangi,[d] Nicholas P. Gauthier,[e,f] John E. McGeehan,[a,b] Gregg T. Beckham[a,b,c]*

[a]Renewable Resources and Enabling Sciences Center, National Renewable Energy Laboratory, Golden, CO 80401
[b]BOTTLE Consortium, Golden, CO 80401
[c]Agile BioFoundry, Emeryville, CA 94608
[d]SLAC National Accelerator Laboratory, Stanford Synchrotron Radiation Lightsource, Menlo Park, CA 94025
[e]Department of Systems Biology, Harvard Medical School, Boston, MA 02115
[f]Department of Data Sciences, Dana-Farber Cancer Institute, Boston, MA 02115

Table of Contents

Supporting Information 1

Supplementary Methods 2
Section S1. Predictors 2
Section S2. Protein Expression and Purification 3
Section S3. Sequence and Structure Analysis 4

Literature data used in D1-513-Scraped 6
Table S1 6
Figure S1 7

Experimental observations 8
Figure S2 8
Figure S3 9
Figure S4 10
Figure S5 11
Figure S6 12
Table S2 13
Figure S7 14
Table S3 15
Figure S8 19
Table S4 20
Figure S9 21

Significant factors observed for low pH active candidates 22
Table S5 22

Machine learning performance 32
Table S6 32
Figure S10 34
Table S7 35
Figure S11 36

References 37

Supplementary Methods

Section S1. Predictors

PETase activity predictor used in Round 1 selection

The HMM hits from the Round 1 search were used to train a PETase homolog VAE. The hits were redundancy reduced by clustering with mmseqs2 at sequence identity 0.9999: ` --min-seq-id 0.9999 -c 0.5 --cov-mode 1 --max-seqs 20 -e 1e-3 --seq-id-mode 1 -s 4.0` and taking the representative sequence from each cluster, producing 8,081 unique sequence.[1] These were split at approximately 80/20 train test by another clustering call with the same parameters as above except at 80% sequence identity, keeping sequences of a cluster together. All sequences were aligned using MAFFT, columns with >95% gaps were removed, resulting in an MSA with 437 position.[2] Sequence weights in the MSA were computed as the reciprocal of the number of sequences sharing >80% identity then normalized to mean 1, as developed elsewhere.[3]

The resulting MSA and sequence weights were used to train a Variational Autoencoder (VAE) to learn a continuous latent representation. Sequences in the MSA were one hot encoded including a gap character and given to a multi-layer perceptron with three dense layers of 512, 256, and 96 neurons and ELU activation functions. The 96 dimensional latent vector was decoded with a symmetrical decoder. For training, both dropout (0.2) and L2 norm (1e-6) was used with the evidence lower bound loss (KL divergence weight of 2.0), descended on by the Adam optimizer (initial learning rate 1e-3, gradient clipping at 10.0). A batch size of 256 was used with early stopping (patience 20 epochs, validation loss tolerance 1e-4). We used a learning rate reduction schedule of 0.5 per 10 epochs and a floor of 1e-7.

Latent embeddings from this pretrained VAE were used to train a supervised predictor of PETase activity using the D1-513-scraped dataset "DeepPETase". To address the heterogeneity in activity measurements across studies, a pairwise ranking approach was implemented. For each study, all possible n(n-1)/2 sequence pairs were generated, with the objective of predicting whether the second sequence exhibited higher activity than the first. Sample weights were assigned to each pair as $1 + (|\Delta_{activity}|/\sigma_{activity})$, where $\Delta_{activity}$ is the difference in activity between the pair and $\sigma_{activity}$ is the standard deviation of activity differences within the study. The top model used for training consisted of a linear "scorer" layer, followed by a "ranking" layer that takes scores of a pair of sequences from the scorer and returns sigmoid of the difference of scores, computing the probability that the second sequence has greater activity than the first. In training the scorer/rank model, we used no dropout, L2 regularization (λ=1e-6), a binary cross-entropy loss, a learning rate of 1e-2, and batch size of 256. The model employed early stopping with a patience of 20 epochs and minimum delta of 1e-4, with learning rate reduction by a factor of 0.5 (patience of 10 epochs, minimum learning rate of 1e-7). Both the VAE and the top model underwent hyperparameter optimization over layer sizes, learning rate, and more.

For inference, we used scores predicted from the linear scorer (i.e. without the ranker), avoiding the need for pairwise ranking. The 96 candidates with the highest predicted scores that had <90% identity to known PETases were selected, enforcing that the selections were <90% identity to each other.

Candidate selection for Round 2

For each candidate sequence, we predicted three properties: (1) thermostability using A Tm predictor (see below), (2) PETase activity using PETML (see below), and (3) optimal pH using EpHod.[4] Signal peptides were predicted and removed from the sequences prior to property prediction via SignalP.[5]

Candidate selection followed two parallel strategies. For focused exploitation, we selected sequences scoring above the 98th percentile in any of the three predicted properties (high thermostability, high activity, or low pH optimum). For exploration of diverse candidates, we selected sequences scoring above the 50th percentile in all three properties simultaneously. From

this combined pool we removed sequences with greater than 90% sequence identity to any known PETase in the PAZy database. The remaining 1,208 sequences were clustered at 90% sequence identity using MMseqs2 (coverage threshold 0.5, sensitivity 7.5), producing 448 clusters.[1] For each cluster, a discrete probability over candidates was created as the softmax of the max percentile of the three properties exhibited by the candidate, and one candidate per cluster sampled from these distributions. We went forward with the 197 of 448 candidates with the highest max percentile.

Round 2 thermal stability predictor
We trained an in house predictor of melting temperature for scoring candidates in Round 2. The model was not PETases specific, instead trained on all sequences in Meltome Atlas. The final model consisted of a Ridge regressor on ProtT5-xl-uniref50 mean pool embeddings, however we tested an extensive set of embeddings methods in cross validation.[6] Performance of these different embedding strategies is given in **Fig S1**.

Round 2 PETase activity predictor
PETML was trained to predict PETase activity of D1-Scraped-513. PETML was an ensemble of a logistic regression models with one-hot encodings, and four unsupervised traditional scores: profile-HMM score (HMM-61), family HMM score (full metagenomic alignment of homologs), active-site HMM (profile-HMM of HMM-61 using only active site residues), and the BLOSUM62 score with consensus sequence from alignment of PETases. The logistic regression was trained with the same ranking objective as the supervised mode trained for Round 1. The scores from these strategies were normalized and summed to produce a final score. The Round 2 model outperformed the Round 1 model in 5-fold cross validation with D1-Scraped-513 so was used. The code for this model is available at on Zenodo.

Candidate selection for Round 3
For Round 3, we incorporated our assay labels from previous rounds to train supervised models. We evaluated mean pool embeddings of four protein language models in 10 fold cross validation: ESM-1b (650M parameters), ESM-1v (650M parameters), ESM-2 (650M parameters), and ESM-2 (3B parameters).[7] The embeddings were used with ridge regressors (regularization strength 100.5) for melting temperature and max observed PET activity over all conditions. The best embeddings (ESM-1v) achieved a CV Spearman score of 0.537 for PET activity.

From the initial pool of 8,067 candidates, we first identified sequences predicted to have both high activity and thermal stability. We selected sequences with predicted log-transformed activity above 1.0 (corresponding to the 85th percentile) and predicted melting temperature above 55°C (86th percentile), yielding 763 sequences that met both criteria. To ensure novelty, we then removed sequences sharing more than 95% identity with any previously screened sequences from Rounds 1 and 2. The remaining sequences were further clustered at 95% identity using mmseqs2 to maintain diversity within our selection, keeping only one representative sequence from each cluster. Finally, we applied a length filter to remove sequences longer than 500 residues, producing a set of 191.

Section S2. Protein Expression and Purification
As reported previously,[8] C41(DE3) *E. coli* competent cells (prepared via the Zymo Mix and Go Transformation Kit, T3001) were transformed and grown at 37 °C, 300 rpm (19 mm orbit) over 40 h in 96-deep well plates (NEST 503501). These starter cultures were used to inoculate 2 mL autoinduction cultures supplemented 100 µg/mL kanamycin and 1X trace metals (Teknova T1001) grown in 24 deep-well plates with well volumes of 10.4 mL (Thomson Instrument Company 931568). Expression cultures were grown at 37 °C for 2 h then at 18 °C for 40 h at 300 rpm (19 mm orbit). Cells were harvested via centrifugation and resuspended in Lysis Buffer (20 mM TRIS pH 8.0, 300 mM NaCl, 5 mM imidazole, 1% *n*-octyl β-D-glucopyranoside supplemented with 0.1 mg/mL DNaseI and 1 mg/mL lysozyme) via shaking at 300 rpm (19 mm orbit) at 18 °C for 1 h. 70µL of a 25% slurry of Ni-charged magnetic beads (Genscript L00295) were added and binding occurred over 2 h at 250 rpm (19 mm orbit) and 18 °C. The magnetic beads were immobilized via

a magnetic block, the supernatant aspirated, and the beads resuspended in Wash Buffer (20 mM TRIS pH 8.0, 300 mM NaCl, 5 mM imidazole). The beads were transferred to a 96 deep-well plate, washed 3X with Wash Buffer and 2X with Cleavage Buffer (20 mM TRIS pH 8.0, 300 mM NaCl), then the SUMO protease (His-tagged *Cth* SUMO protease, produced in-house) was added and shaken at 1200 rpm (3 mm orbit) for 3-4 h at room temperature then stored static overnight at 4 °C. The supernatant was then aspirated off the magnetic beads and moved to a new plate. Concentration was determined using the Pierce Rapid Gold BCA Protein Assay Kit (A55862) and the concentration normalized via dilution to 0.1 mg/mL, unless this would exceed the volume of a 2 mL well, in which case it was brought to 0.3 mg/mL. If the concentration was under 0.1 mg/mL, then no dilution was performed.

Section S3. Sequence and Structure Analysis

Sequence conservation

Columns in the alignment of our enzymes were analyzed with Jalview.[9] Conservation factors such as "aliphatic," "tiny," "charged" for each column were compared between performance groups. See their documentation for a full list. Any columns for which there were at least 7 conservation factors in the positive group (e.g. pH 4.5) that were not observed to be conserved in the reference group (e.g. 7.5 pH) were marked as significant. This analysis differs from standard sequence conservation in that only conserved factors that were not necessary for activity in general but were for the challenge condition are marked. The Jalview annotation files including conservation differences are provided in on Zenodo.

Surface properties

Surface properties were compared between performance groups. To do this algorithmically for large sets of proteins we leveraged SURFMAP.[10] The software computes surface properties and projects those values onto the nearest point on discretized sphere surrounding the protein. This means that, after structurally aligned, proteins of different lengths can be compared on a fixed length vector of points as opposed to residue-wise comparisons. We first predicted the structures of each active homolog using ColabFold.[11] Each structure had a pLDDT > 0.9. We then removed any additional modules from proteins within the dataset. The start and the end of the main enzymatic domain were identified by the first and last block of 8 consecutive columns in the alignment with 80% occupancy. The resulting structures were aligned using mTM align with a mean pairwise TM score of 0.89 and a minimum of 0.81.[12] Raw predicted structures and cut aligned ones are provided on Zenodo. Kyte-Doolittle hydrophilicity, electrostatics, and stickiness were compute using SURFMAP, which uses APBS internally for electrostatics.[10,13] Values at each projected position were compared between performance groups, and positions with statistically different values for the positive performance group compared to the reference group according to a Mann-Whitney U test ($p<0.05$) were mapped back to the multiple sequence alignment and marked as significant for each surface property independently. When depicted in Figure 4, the count of significant factors is divided by three for surface properties such that a value of one indicates all three surface properties were significantly different.

Analysis of pKa and titratable residues

We predicted pKa values of each titratable residue in all active enzymes using PROPKA with a reference pH of 4.5.[14] Predicted structures from ColabFold were used.[11] For each column in the alignment, a Mann-Whitney U test was conducted over pKa values if present between the challenge group and the reference group. Significant residues were marked with $p<0.05$. We also considered simply the presence or absence of titratable residues at each column and marked significant columns according to $p<0.05$ with a Fisher Exact test, however were not used for the analysis in the main text.

Predictor attention

The attention mechanism of temBERTure and EpHod were considered as a means of identifying significant positions important for determining temperature tolerance and pH tolerance but were

not used for final conclusions in Figure 4. This was not conducted against performance groups independently but instead against the entire alignment of active homologs.[4,15] For each sequence, the attention matrix from the last layer of the model (L,L for temperature and L,d), where L is sequence length and d is the model dimension, was mean pooled over the attendee dimension and scaled to (0,1) producing a vector of the importance of each amino acid to prediction. These values were mapped back to the alignment of all active enzymes and mean averaged for each column. Columns were marked as significant if they exhibited a score greater than 0.7 times the interquartile range over the median score.

Clustering additional domains

Additional modules presented by some homologs were clustered. First, the main domain was removed from the MSA by identifying the first and last block of 8 consecutive columns in the alignment with 80% occupancy. Pairwise BLOSUM62 scores were then computed for the remaining columns. A minimum pairwise bitscore of 500 was set and an initial cluster was set by placing the two candidates with maximum observed pairwise score in a cluster. Clusters where iteratively expanded by identifying the max scoring pair among those remaining. If either of the pair is already a member of a cluster, the new member is added, else a new cluster is formed. This is repeated until no more pairwise scores meet the minimum bit score of 500. This process resulting in a cluster where candidates exhibited a ricin B lectin CBM and nearly all of them exhibited low pH activity.

Literature data used in D1-513-Scraped

Table S1: Breakdown of PETase activity measurements in D3-Scraped-513 dataset. This table details the D3-Scraped-513 dataset consisting of 513 PETase activity measurements for 449 unique proteins extracted from 26 studies.

	Study	Singles	Multiples	Naturals	Total
**1**	Bell et al, 2022[16]	0	5	1	6
**2**	Brott et al, 2021[17]	0	8	1	9
**3**	Chen et al, 2021[18]	4	9	9	22
**4.**	Cui et al, 2021[19]	86	64	1	151
**5**	Erickson et al, 2022[20]	0	3	40	43
**6**	Furukawa et al, 2019[21]	5	5	1	11
**7**	Guo et al, 2022[22]	12	1	1	14
**8**	Han et al, 2017[23]	11	0	1	12
**9**	Joo et al, 2018[24]	13	1	1	15
**10**	Li Q. et al, 2022[25]	1	6	1	8
**11**	Li Z. et al, 2022[26]	4	2	1	7
**12**	Liu et al, 2018[27]	16	1	1	18
**13**	Lu et al, 2022[28]	0	4	2	6
**14**	Ma et al, 2018[29]	8	0	1	9
**15**	Nakamura et al, 2021[30]	16	7	1	24
**16**	Pfaff et al, 2022[31]	26	3	1	30
**17**	Sagong et al, 2022[32]	6	0	1	7
**18**	Son et al, 2019[33]	10	6	1	17
**19**	Sonnendecker et al, 2021[34]	0	0	8	8
**20**	Then et al, 2016[35]	0	8	1	9
**21**	Tournier et al, 2020[36]	6	11	5	22
**22**	Wang et al, 2022[37]	8	4	1	13
**23**	Wei et al, 2016[38]	4	7	1	12
**24**	Xi et al, 2021[39]	0	0	3	3
**25**	Zeng et al, 2022[40]	0	34	0	34
**26**	Zhang et al, 2021[41]	0	0	3	3
	Total	236	189	88	513

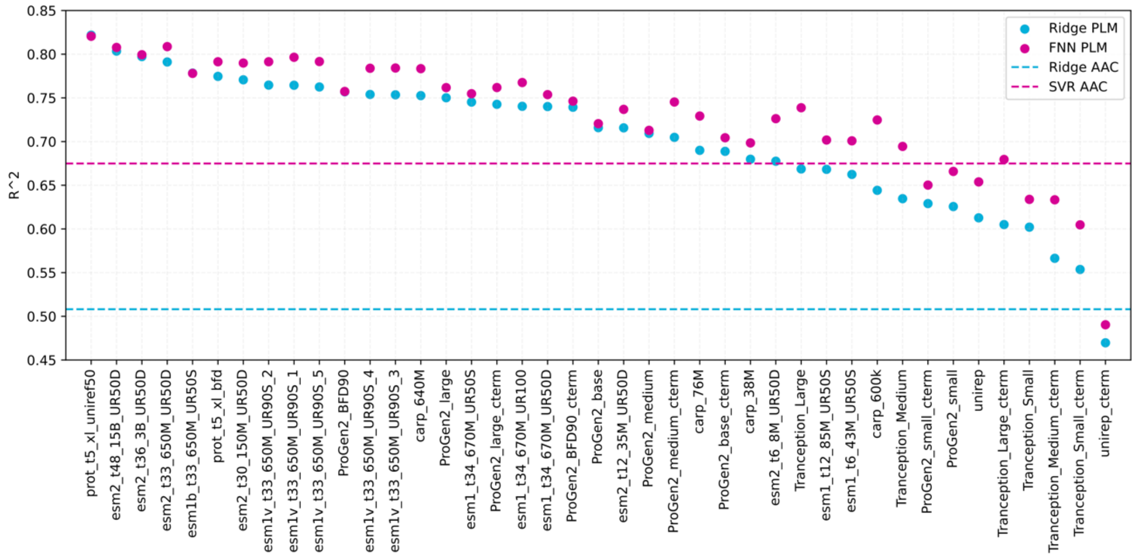

Figure S1: Performance of different protein language model (PLM) embeddings for predicting melting temperature (T_m) using the MeltomeAtlas dataset (n = 31,262 after preprocessing).[42] Ridge regression models and feed-forward neural networks (FNNs) with a single hidden layer were trained for each embedding, with hyperparameters optimized via random search. Model performance was evaluated on a held-out test set (n = 4,732) with less than 20% sequence identity to any training or validation sequence. As baselines, the performance of ridge regression and support vector regression (SVR) models trained on amino acid composition (AAC) are shown as dashed lines. The figure shows that masked/denoising language models (ESM, ProtT5, CARP) generally outperformed autoregressive models (ProGen2, Tranception, UniRep) with both mean-pooled embeddings and embeddings from the final C-terminal token (cterm) of the autoregressive models.[6,43–47] ProtT5-XL-U50 embeddings achieved the highest predictive performance for T_m. While FNN models largely outperformed ridge models, ProtT5-XL-U50 showed comparable performance with both ridge and FNN models. Hence, the ridge model trained with ProtT5-XL-U50 embeddings was selected as the final T_m predictor, named ThermoPalm. Code for ThermoPalm is available at https://github.com/jafetgado/ThermoPalm.

Experimental observations

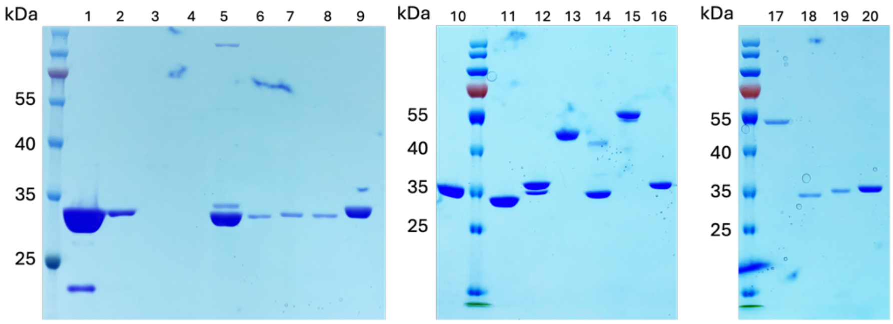

Lane	Sample	Expected MW (Da)
1	RFP	25424
2	DP001	27550
3	DP002	27991
4	DP005	31453
5	DP003	28336
6	DP006	28355
7	DP007	28306
8	DP008	27947
9	DP009	28164
10	DP079	27993
11	DP026	27998
12	DP090	29006
13	DP059	40611
14	DP047	40906
15	DP081	44641
16	DP022	27326
17	DP068	43312
18	DP082	27301
19	DP077	27669
20	DP029	28415

Figure S2: Sodium dodecyl sulfate–polyacrylamide gel electrophoresis (SDS-PAGE) analysis of a subset of purified enzymes. All enzymes evaluated showed a band at the expected molecular weight (MW) except for DP047 (Lane 14), which showed a faint band at the correct MW and a stronger band at a lower MW, and DP002 (Lane 3) and DP005 (Lane 4), which failed purification as evaluated by the BCA assay (measured concentration lower than 0.1 mg/mL) and showed no visible band.

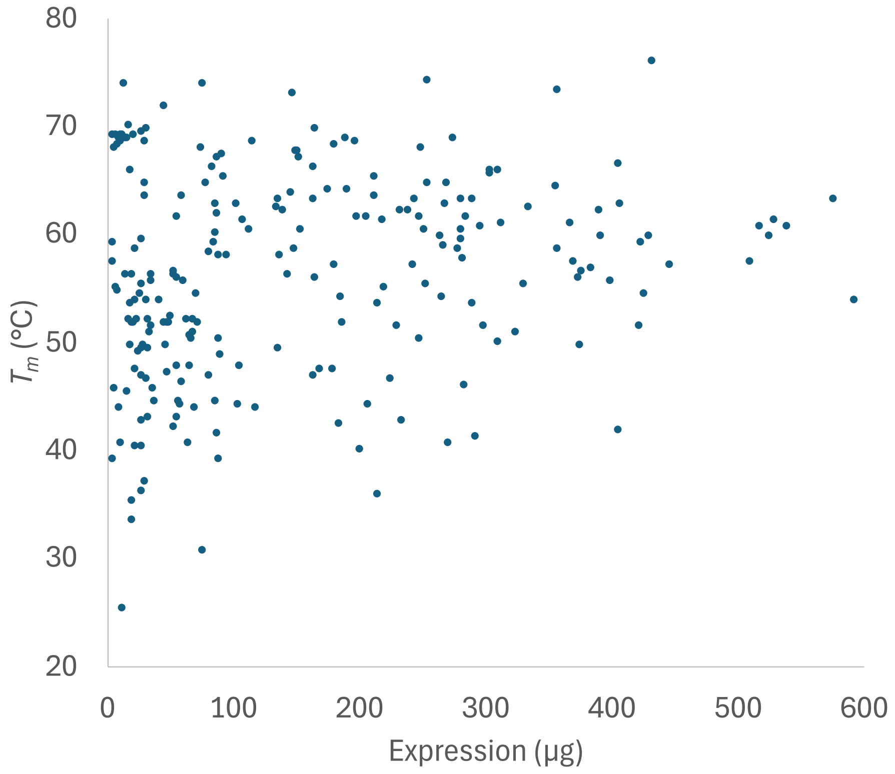

Figure S3: Thermostability versus expression yield in µg per single well for all enzymes that demonstrated a measurable T_m. If two inflection points were observed, the highest was used.

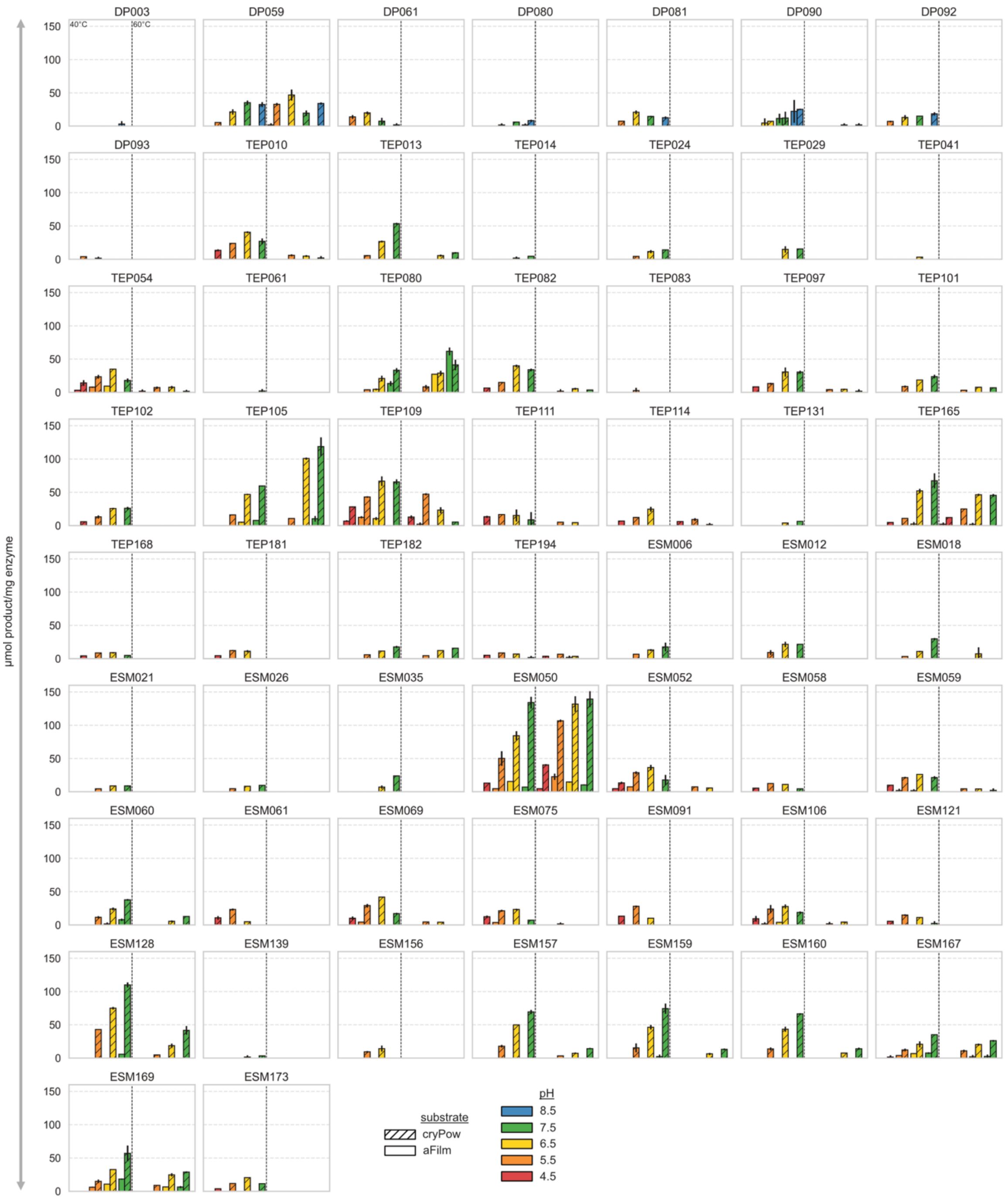

Figure S4: PET hydrolase activity in µmol aromatic products produced per mg of enzyme added at varying pH (color of bar), temperature (left vs. right for each enzyme), and substrate crystallinities – amorphous film (aFilm, solid bars) and crystalline powder (cryPow, hatched bars). Shown here are all enzymes tested with yields sufficient to test in 32 conditions: 4 pHs, 2 temperatures, 2 PET substrates, in duplicate. Error bars represent the range between biological duplicates.

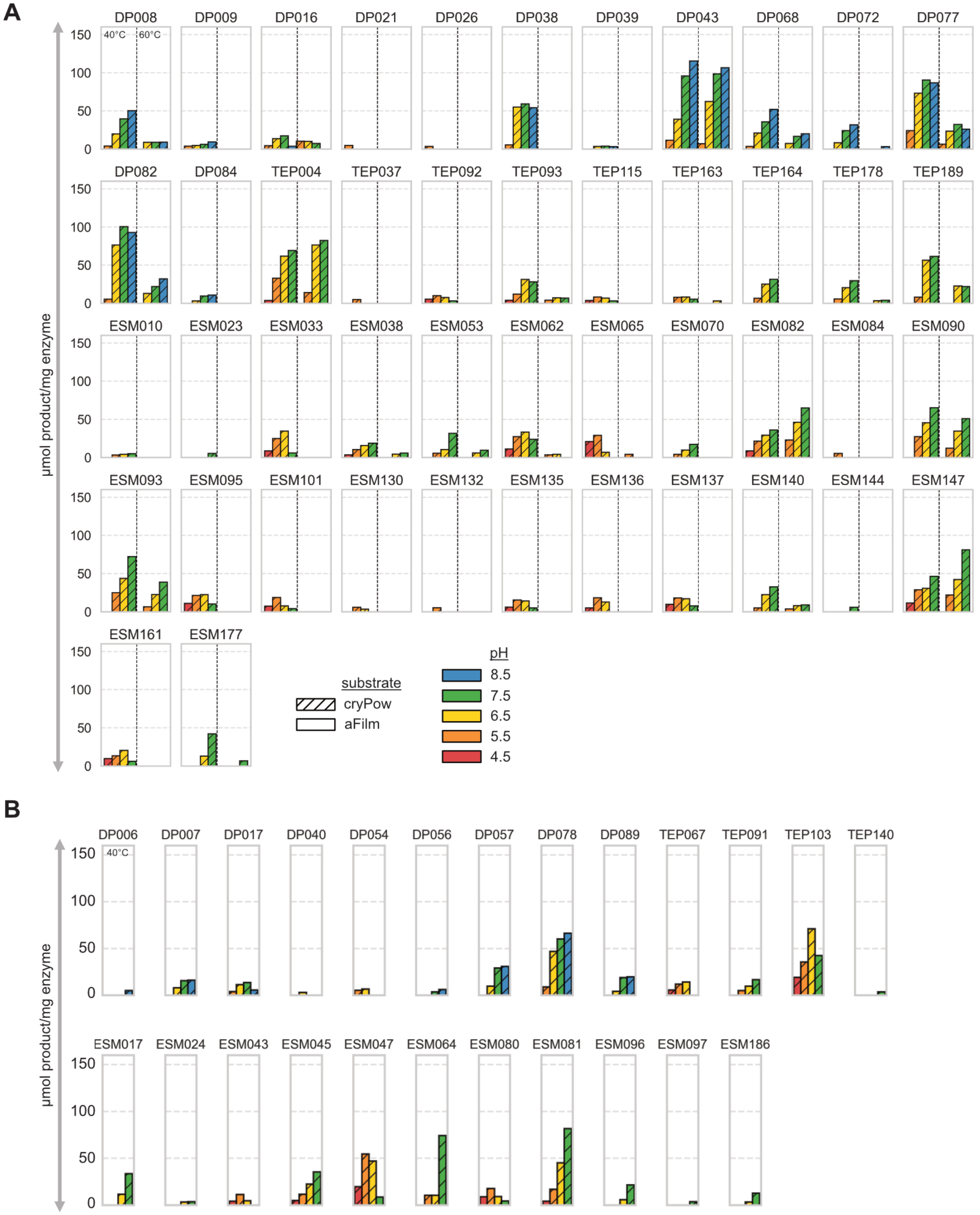

Figure S5: PET hydrolase activity in μmol aromatic products produced per mg of enzyme added at varying pH (color of bar), temperature (left vs. right for each enzyme), and substrate crystallinities – amorphous film (aFilm, solid bars) and crystalline powder (cryPow, hatched bars). **(A)** All enzymes tested with yields sufficient to test in 8 conditions: 4 pHs, 2 temperatures, 1 PET substrate (cryPow), based on single measurements. **(B)** All enzymes tested with yields sufficient to test in 4 conditions: 4 pHs, 1 temperature (40 °C), 1 PET substrate (cryPow), based on single measurements.

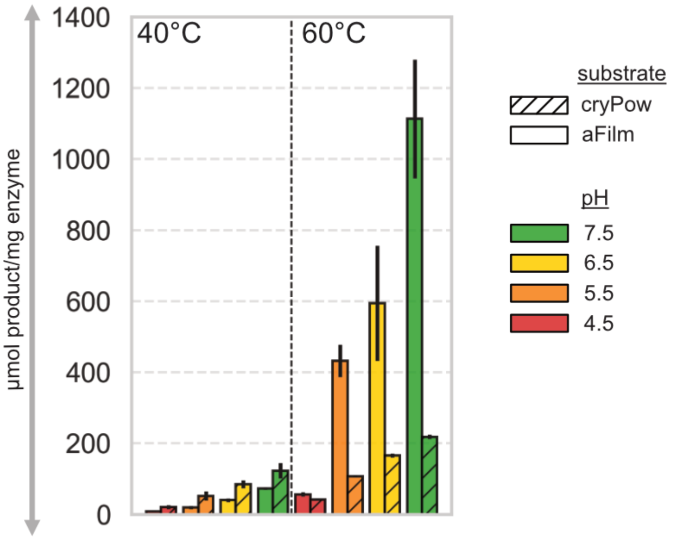

Figure S6: LCC-ICCG activity in µmol aromatic products produced per mg of enzyme added at varying pH (color of bar), temperature (left vs. right for each enzyme), and substrate crystallinities – amorphous film (aFilm, solid bars) and crystalline powder (cryPow, hatched bars).

Table S2: Observed hit rates over search rounds.

Substrate	T[C]	pH	R1 Count	R1 Hit Rate	R2 Count	R2 Hit Rate	R3 Count	R3 Hit Rate
**aFilm**	40	4.5	0		29	0.034	34	0.029
**aFilm**	40	5.5	14	0.000	29	0.069	34	0.059
**aFilm**	40	6.5	14	0.071	29	0.138	34	0.088
**aFilm**	40	7.5	14	0.071	29	0.069	34	0.147
**aFilm**	40	8.5	14	0.143	0		0	
**aFilm**	60	4.5	0		29	0.000	34	0.000
**aFilm**	60	5.5	14	0.000	29	0.000	34	0.029
**aFilm**	60	6.5	14	0.000	29	0.034	34	0.059
**aFilm**	60	7.5	14	0.000	29	0.069	34	0.059
**aFilm**	60	8.5	14	0.000	0		0	
**cryPow**	40	4.5	0		73	0.164	86	0.256
**cryPow**	40	5.5	53	0.189	73	0.384	86	0.535
**cryPow**	40	6.5	53	0.340	73	0.411	86	0.581
**cryPow**	40	7.5	53	0.396	73	0.356	86	0.523
**cryPow**	40	8.5	53	0.377	0		0	
**cryPow**	60	4.5	0		57	0.053	70	0.014
**cryPow**	60	5.5	33	0.121	57	0.175	70	0.129
**cryPow**	60	6.5	33	0.212	57	0.246	70	0.243
**cryPow**	60	7.5	33	0.212	57	0.175	70	0.229
**cryPow**	60	8.5	33	0.182	0		0	

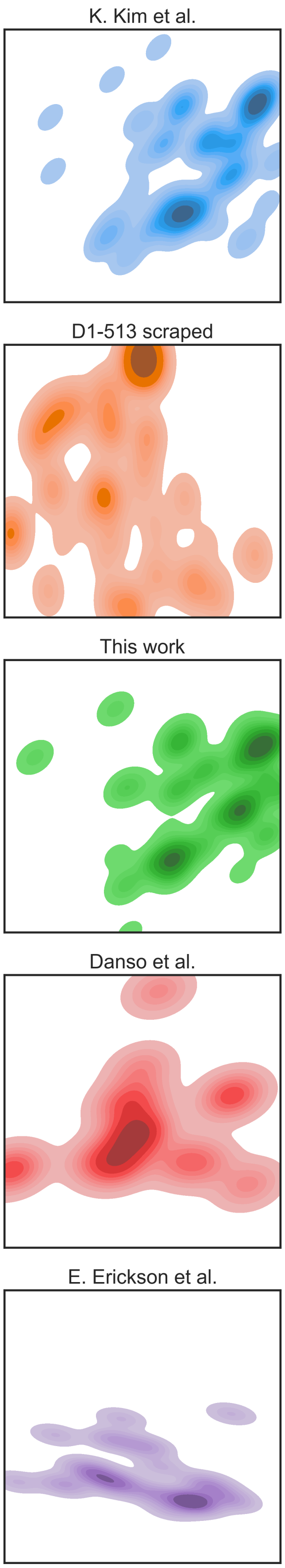

Figure S7: PETase space for previous studies showing a 2D uniform manifold approximation (UMAP) based on pairwise negative BLOSUM62 scores between PETases..[20,48,49]

Table S3: RMSD scores of catalytic domain predicted by AF2 of active candidates to LCC-ICCG.

	RMSD
**DP003**	1.122
**DP007**	1.117
**DP008**	1.094
**DP009**	1.346
**DP016**	1.195
**DP017**	1.031
**DP038**	1.099
**DP043**	1.294
**DP054**	1.108
**DP056**	0.881
**DP057**	0.986
**DP059**	1.339
**DP061**	1.213
**DP068**	1.221
**DP072**	1.36
**DP077**	0.988
**DP078**	1.573
**DP080**	0.943
**DP081**	1.189
**DP082**	0.913
**DP084**	0.877
**DP089**	1.095
**DP090**	0.908
**DP092**	1.398
**ESM006**	1.368
**ESM010**	1.379
**ESM012**	1.224
**ESM017**	1.55
**ESM018**	1.279
**ESM021**	1.45
**ESM023**	1.356
**ESM026**	1.311
**ESM033**	1.194
**ESM035**	1.381
**ESM038**	1.456

**ESM043**	1.361
**ESM045**	1.318
**ESM047**	1.127
**ESM050**	1.449
**ESM052**	1.169
**ESM053**	1.345
**ESM058**	1.222
**ESM059**	1.23
**ESM060**	1.188
**ESM061**	1.193
**ESM062**	1.218
**ESM064**	1.308
**ESM065**	1.191
**ESM069**	1.263
**ESM070**	1.318
**ESM075**	1.165
**ESM080**	1.204
**ESM081**	1.189
**ESM082**	1.027
**ESM084**	1.371
**ESM090**	1.002
**ESM091**	1.184
**ESM093**	0.986
**ESM095**	1.219
**ESM096**	1.564
**ESM101**	1.18
**ESM106**	1.188
**ESM121**	1.191
**ESM128**	1.191
**ESM130**	1.159
**ESM132**	1.235
**ESM135**	1.165
**ESM136**	1.178
**ESM137**	1.218
**ESM140**	1.128
**ESM144**	1
**ESM147**	1.005
**ESM156**	1.317
**ESM157**	1.144

**ESM159**	1.117
**ESM160**	1.168
**ESM161**	1.237
**ESM167**	1.138
**ESM169**	1.076
**ESM173**	1.223
**ESM177**	1.035
**ESM186**	1.234
**LCC-ICCG**	0
**TEP004**	1.404
**TEP010**	1.222
**TEP013**	1.376
**TEP014**	0.766
**TEP024**	1.302
**TEP029**	1.222
**TEP054**	1.2
**TEP067**	1.292
**TEP080**	1.428
**TEP082**	1.248
**TEP083**	1.225
**TEP091**	0.893
**TEP092**	1.156
**TEP093**	1.27
**TEP097**	1.332
**TEP101**	1.033
**TEP102**	1.177
**TEP103**	1.042
**TEP105**	1.166
**TEP109**	1.221
**TEP111**	1.27
**TEP114**	1.352
**TEP115**	1.229
**TEP131**	1.217
**TEP163**	1.286
**TEP164**	1.171
**TEP165**	1.27
**TEP168**	1.239
**TEP178**	1.087
**TEP181**	1.231

**TEP182**	1.101
**TEP189**	1.604
**TEP194**	0.986

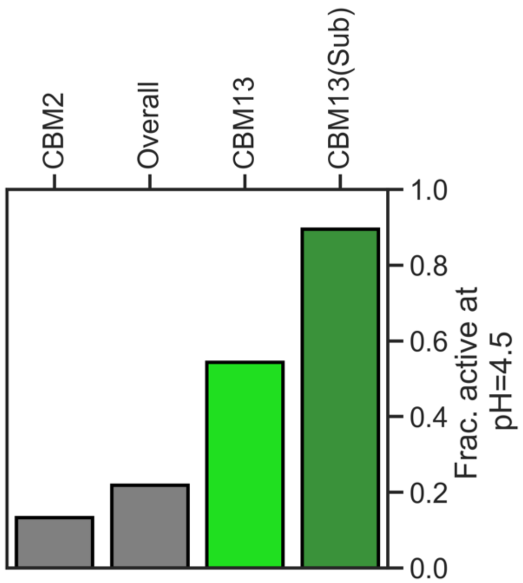

Figure S8: All carbohydrate binding modules observed for our active candidates, labeled by dbCAN2, with CBM13 showing an association with a high hit rate for pH 4.5 activity. A sub-cluster (pairwise bitscore >500 with BLOSUM62) labelled CBM13(Sub) demonstrated an even higher hit rate of 85%, with 11 of the 13 enzymes with this domain being active at pH 4.5.

Table S4: **Crystallographic parameters, data collection and refinement statistics.**

	DP043
**Crystallographic parameters**	
Space group	P $3_2 21$
Unit-cell dimensions	87.25, 87.25, 148.87Å<br>90.0, 107.2,90.0˚
**Data collection statistics**	
Resolution limits (outer shell) (Å)	37.78-1.76(1.81-1.76)
No: of observed reflections (outer shell)	890810 (67132)
No: of unique reflections (outer shell)	65682 (4789)
Completeness (outer shell)	98.6 (98.6)
CC1/2 (outer shell)	99.4 (73.7)
R_{sym}[a] (outer shell) (%)	12.8 (271.3)
Mean I/σ(I) (outer shell)	13.3 (1.5)
**Refinement statistics**	
Resolution limits (Å)	37.78-1.76
Number of reflections (%)	62371 (99.96)
Reflections used for R_{free}	3283
R_{factor}[b] (%)	16.1
R_{free} (%)	19.9
Model contents (average B($Å^2$))	
Protein atoms	3872 (33.5)
Ligand	0
Ion/bugger	14 (51.1)
Water molecules	508 (43.1)
RMS deviations	
Bond length (Å)	0.009
Bond angle (°)	1.64
Ramachandran (favored (%)/outliers)	99/0

[a] $R_{sym} = \Sigma|I_{avg}-I_i|/\Sigma I_i$

[b] R factor = $\Sigma|F_p - F_{pcalc}|/\Sigma F_p$, where F_p and F_{pcalc} are the observed and calculated structure factors; R_{free} is calculated with 5% of the data.

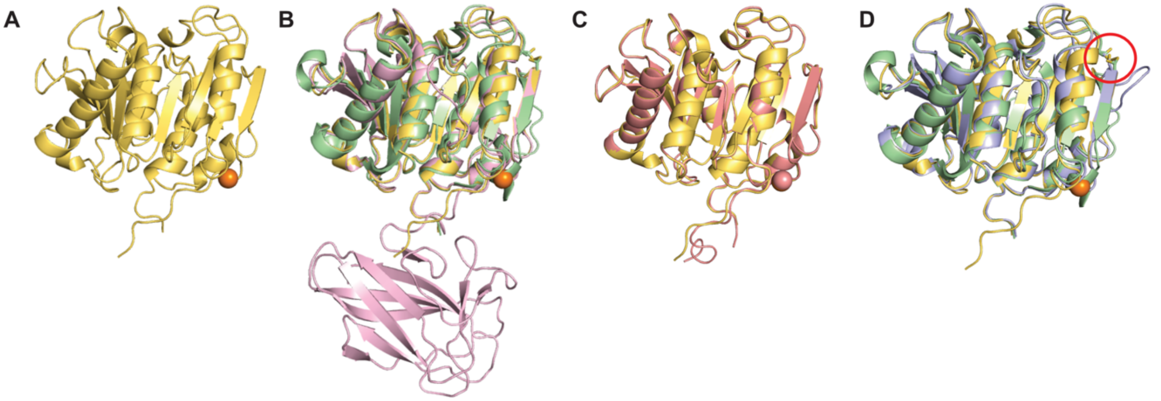

Figure S9: (**A**) DP043 crystal structure (yellow, PDB: 9O9W) with a Ca^{2+} bound (orange). (**B**) DP043 crystal structure aligned with LCC-ICCG (green, PDB: 6THT) with an all-atom RMSD of 0.827 Å and enzyme 407 (pink, Alphafold2) from Erickson et al.[20] with an all-atom RMSD of 0.512 Å. Enzyme 407 displays a second domain. (**C**) DP043 crystal structure aligned with the DP043 Alphafold3 structure with one predicted Ca^{2+} ion (dark pink) overlaying the observed Ca^{2+} in the crystal structure (**D**) DP043 crystal structure aligned with LCC-ICCG (green, PDB: 6THT) and $TfCut2_{D204C/E253C}$ (purple, Alphafold3) with the location of the disulfide bonds engineered to replace the native Ca^{2+} binding site shown (red circle).

Significant factors observed for low pH active candidates

Table S5: Mapping of all significant factors to LCC-ICCG. Reasons are space separated of <descriptor>:<values>, for conservation the value is the AA conserved in low pH activity. For continuous values it is <observed value on LCC-ICCG>:<low pH active mean>vs<neutral only active mean>.

Ali. Pos	LCC Pos	AA	Reasons
**154**	1	S	pH_electrostatics:2.8158181818181816:-0.4853238095238095vs0.45330681818181817 pH_circular_variance:0.41900000000000004:0.427290756302521vs0.46138480392156866 pH_stickiness:0.035500000000000004:-0.4213685714285714vs-0.29978333333333335
**155**	2	N	pH_circular_variance:0.458:0.42015714285714284vs0.448354166666667 pH_electrostatics:2.5995:-0.49177857142857145vs0.4351614583333333
**156**	3	P	pH_circular_variance:0.607:0.5684857142857143vs0.5492499999999999 pH_stickiness:-0.006:-0.3422857142857143vs-0.22743750000000004 pH_electrostatics:2.4934545454545454:-0.3360233766233766vs0.6080672348484849
**157**	4	Y	pH_circular_variance:0.6447142857142857:0.6190530612244898vs0.5914077380952382 pH_kyte_doolittle:-2.1526:-1.6224457142857145vs-1.4790750000000001 pH_stickiness:0.0965:0.1612857142857143vs-0.006593749999999885 pH_electrostatics:2.1695882352941176:-0.39834957983193275vs0.5328357843137255
**158**	5	Q	pH_circular_variance:0.49083333333333334:0.4714190476190476vs0.5012326388888889 pH_stickiness:-0.162875:-0.4144142857142857vs-0.3076822916666666 pH_electrostatics:3.2614705882352943:-0.6176420168067226vs0.3401188725490196
**159**	6	R	pH_electrostatics:3.0715000000000003:-0.30757142857142855vs0.6110520833333334
**160**	7	G	pH_electrostatics:3.4825714285714287:-0.38535102040816327vs0.5583095238095239
**161**	8	P	pH_electrostatics:2.7082592592592594:-0.09336402116402116vs0.7348479938271605 pH_kyte_doolittle:-2.62:-2.402114285714286vs-2.0074166666666664 pH_stickiness:-0.15433333333333332:-0.35662857142857146vs-0.26753819444444443
**162**	9	N	pH_kyte_doolittle:-3.2234285714285713:-3.042342857142857vs-2.623854166666667 pH_stickiness:-0.2254736842105263:-0.4358345864661654vs-0.30351644736842104 pH_electrostatics:2.626923076923077:-0.5830428571428572vs0.3272411858974359
**164**	11	T	pH_electrostatics:2.05925:0.20079761904761906vs0.7826180555555555 pH_kyte_doolittle:-1.767375:-1.5710071428571428vs-0.9521614583333333 pH_stickiness:0.078:0.04356190476190477vs-0.050364583333333345
**165**	12	R	pH_kyte_doolittle:-3.0885833333333337:-1.8452714285714287vs-1.0368680555555556 pH_stickiness:-0.027428571428571434:-0.036595918367346936vs-0.18363690476190478
**166**	13	S	pH_kyte_doolittle:-1.1125714285714285:-0.6786897959183673vs-0.9019761904761905 pH_electrostatics:1.5761666666666667:0.20365238095238095vs0.8722326388888889 pH_stickiness:0.09433333333333334:0.020440816326530616vs-0.1280436507936508 pH_circular_variance:0.524:0.5308571428571429vs0.5522291666666667
**167**	14	A	pH_electrostatics:1.8628888888888886:0.24544126984126985vs0.9470300925925925 pH_stickiness:0.026:0.03320000000000001vs-0.04850000000000001
**169**	16	T	pH_stickiness:0.049428571428571426:-0.0023836734693877507vs-0.1426488095238095 pH_circular_variance:0.574:0.6218571428571429vs0.5939166666666666 pH_electrostatics:1.6942857142857142:0.18634693877551017vs0.9462142857142857 pH_kyte_doolittle:-0.6036666666666667:-0.3907190476190476vs-1.0915937500000001
**170**	17	A	pH_circular_variance:0.5572727272727273:0.5535714285714286vs0.5598617424242425 pH_conserved:A pH_stickiness:-0.2092857142857143:-0.09183673469387754vs-0.048514880952380955 pH_electrostatics:1.58335:0.31317285714285714vs1.1374385416666666 pH_kyte_doolittle:-0.4082222222222222:-1.4795650793650796vs-1.2412916666666667
**172**	18	D	pH_kyte_doolittle:-1.5638999999999998:-2.4413vs-1.8047895833333332 pH_circular_variance:0.5643333333333334:0.523602380952381vs0.549748263888889

			pH_electrostatics:1.6420000000000001:0.29614444444444443vs1.223050925925926 pH_stickiness:-0.34933333333333333:-0.1496952380952381vs-0.03466145833333333
**173**	19	G	pH_electrostatics:1.47475:0.11889999999999999vs0.9914635416666666 pH_kyte_doolittle:-0.784:-0.9356285714285715vs-0.5963541666666666 pH_stickiness:-0.18766666666666665:-0.09561904761904762vs-0.025444444444444447 pH_circular_variance:0.56775:0.5339785714285714vs0.555625
**174**	20	P	pH_electrostatics:2.215:0.07415892857142857vs0.8767565104166667 pH_stickiness:-0.1535:-0.07567142857142858vs-0.022479166666666665 pH_kyte_doolittle:-1.562:-0.8199142857142857vs-1.074854166666667 pH_circular_variance:0.571:0.5520857142857143vs0.5662395833333334
**175**	21	F	pH_electrostatics:3.394:-0.26542857142857146vs0.6706041666666668
**176**	22	S	pH_kyte_doolittle:-0.9215:-0.37894285714285714vs-0.31562499999999993 pH_stickiness:-0.08199999999999999:-0.07128571428571429vs0.020763888888888887 pH_electrostatics:2.1354444444444445:0.3171047619047619vs1.0984652777777777 pH_circular_variance:0.5625:0.5268999999999999vs0.5504583333333333
**177**	23	V	pH_kyte_doolittle:-1.169:-0.9488857142857142vs-0.3556666666666666 pH_electrostatics:2.534333333333333:0.5171714285714286vs1.2951319444444445 pH_stickiness:0.16:-0.015057142857142856vs0.08648958333333333
**178**	24	A	pH_electrostatics:2.5886:0.6250971428571429vs1.2166062500000001 pH_kyte_doolittle:0.047:-1.4591714285714288vs-0.9868958333333335 pH_circular_variance:0.62:0.6005142857142857vs0.5916666666666667
**179**	25	T	pH_electrostatics:2.7483333333333335:0.7836190476190477vs1.2901666666666667 pH_kyte_doolittle:-1.3684615384615384:-2.788164835164835vs-1.9975160256410258 pH_circular_variance:0.5813571428571428:0.5988224489795918vs0.5740238095238095
**180**	26	Y	pH_kyte_doolittle:-1.073:-1.0065428571428572vs-0.6475000000000001 pH_stickiness:0.5844:0.05709142857142856vs0.1686166666666667 pH_electrostatics:2.5918:0.7587685714285713vs1.3102333333333334 pH_circular_variance:0.6056:0.5838571428571429vs0.5655854166666667
**181**	27	T	pH_electrostatics:2.458:0.6498857142857143vs1.0709375 pH_circular_variance:0.5372105263157895:0.5496646616541353vs0.5266831140350877 pH_kyte_doolittle:-0.2678888888888889:-2.503079365079365vs-1.7369004629629632
**183**	29	S	pH_kyte_doolittle:1.156:-1.1230571428571428vs-0.4409166666666667 pH_circular_variance:0.5284444444444445:0.5197936507936508vs0.5029537037037036 pH_stickiness:0.4515:-0.052457142857142855vs0.052770833333333336 pH_electrostatics:2.4613333333333336:0.4410444444444444vs0.9349606481481482
**184**	30	R	pH_electrostatics:2.44515:-0.14174857142857142vs0.600428125 pH_kyte_doolittle:-3.10375:-2.317207142857143vs-1.74378515625 pH_stickiness:0.09194444444444444:-0.09646825396825397vs0.06793634259259258 pH_circular_variance:0.558:0.5732128571428572vs0.5489677083333333
**186**	31	L	pH_circular_variance:0.4654:0.5090600000000001vs0.4853895833333334 pH_kyte_doolittle:1.95225:-1.0124499999999999vs-0.2539895833333333 pH_electrostatics:2.00675:-0.061280357142857136vs0.7200240885416667 pH_stickiness:0.432375:-0.029307142857142865vs0.07312760416666667
**187**	32	S	pH_stickiness:0.30833333333333335:0.17946666666666666vs0.05790509259259259 pH_kyte_doolittle:-0.23725000000000002:-1.1911357142857142vs-0.5993437500000001 pH_electrostatics:1.9587333333333334:0.1750704761904762vs1.0320902777777776 pH_circular_variance:0.4948:0.5143828571428573vs0.4885666666666667
**188**	33	V	pH_electrostatics:1.6465:-0.13522857142857142vs0.79365625 pH_circular_variance:0.474:0.5125857142857143vs0.49436458333333333 pH_stickiness:0.314:-0.08302857142857144vs0.07668749999999999
**189**	34	S	pH_electrostatics:1.5490384615384616:-0.033629670329670326vs0.7938397435897435 pH_circular_variance:0.45233333333333337:0.5115219047619048vs0.48933472222222224

			pH_kyte_doolittle:-1.204:-1.1458920634920635vs-0.7919166666666667<br>pH_stickiness:0.09507692307692307:-0.05509670329670329vs-0.013782051282051282
**190**	35	G	pH_circular_variance:0.5333333333333333:0.5538666666666666vs0.5535555555555556<br>pH_kyte_doolittle:-1.2625:-1.2704914285714286vs-0.99773125 pH_stickiness:-0.056499999999999995:-0.17499047619047617vs0.009079861111111117<br>pH_electrostatics:1.574:0.02154505494505495vs0.8145817307692307
**195**	40	V	pH_kyte_doolittle:0.8003333333333332:-2.741571428571428vs-1.7847222222222223
**197**	42	Y	pKa:11.32:11.15vs10.78
**198**	43	Y	pH_circular_variance:0.641:0.6501142857142858vs0.6304166666666666<br>pH_electrostatics:2.9501666666666666:0.8043809523809524vs1.3216909722222223
**200**	45	T	pH_electrostatics:2.6877333333333335:0.5310609523809524vs1.2145444444444444<br>pH_kyte_doolittle:-0.3626666666666667:-0.3713238095238095 6vs-0.7593194444444444
**201**	46	G	pH_kyte_doolittle:-0.2994:-0.7419428571428572vs-1.1158<br>pH_electrostatics:2.9114375:0.2471232142857143vs1.1196002604166666
**202**	47	T	pH_electrostatics:3.0389999999999997:-0.0720857142857143vs0.9004479166666668
**203**	48	S	pH_kyte_doolittle:-0.748:-0.7558vs-1.12296875 pH_stickiness:0.2395:0.027121428571428574vs-0.03950260416666666 pH_electrostatics:2.6230526315789473:-0.2452421052631579vs0.7334111842105263<br>pH_circular_variance:0.4625:0.4667642857142857vs0.4765390625
**205**	49	L	pH_electrostatics:3.0616363636363633:-0.34674025974025974vs0.5604545454545454<br>pH_circular_variance:0.513:0.4844857142857143vs0.49379166666666663<br>pH_stickiness:0.40149999999999997:0.035885714285714285vs-0.025364583333333333
**206**	50	T	pH_circular_variance:0.5475:0.5291vs0.5386666666666666<br>pH_electrostatics:2.3126666666666664:-0.39098285714285713vs0.6923611111111111<br>pH_kyte_doolittle:-1.154090909090909:-1.122781818181818vs-1.5914375<br>pH_stickiness:0.09445454545454547:0.01605714285714286vs-0.09285984848484849
**216**	60	Y	pH_electrostatics:0.9647142857142857:-0.5565142857142857vs0.3722251984126984<br>pH_circular_variance:0.593:0.5839333333333333vs0.5727222222222222<br>pH_kyte_doolittle:0.11066666666666664:0.8607142857142858vs-0.26328472222222227<br>pH_stickiness:0.748:0.9178857142857143vs0.6793981481481483
**217**	61	T	pH_kyte_doolittle:0.22662499999999994:-0.45831071428571424vs-0.2996666666666667<br>pH_electrostatics:1.1533125:-0.5944446428571428vs0.2320703125<br>pH_stickiness:0.052:0.04864285714285715vs0.261375<br>pH_circular_variance:0.5304166666666666:0.5806785714285714vs0.5590885416666667
**218**	62	A	pH_kyte_doolittle:-1.1660000000000001:0.7105619047619047vs-0.47643055555555547<br>pH_electrostatics:1.293:-0.4713333333333334vs0.3242777777777778
**219**	63	D	pH_kyte_doolittle:-3.3545:0.5856857142857144vs-0.5776666666666667 pH_stickiness:-0.221:0.2046vs-0.023020833333333327 pH_electrostatics:1.56475:-0.18778571428571428vs0.48248437499999997<br>pH_circular_variance:0.5912499999999999:0.5522357142857143vs0.52725
**221**	65	S	pH_kyte_doolittle:-1.77525:1.0695428571428571vs-0.14604687500000002 pH_stickiness:-0.029:0.09034285714285716vs-0.09202083333333333 pH_electrostatics:1.6209999999999998:-0.2371940476190476vs0.5681414930555556<br>pH_circular_variance:0.5467333333333334:0.5474609523809524vs0.5275791666666667
**223**	66	S	pH_electrostatics:1.134125:-0.6593107142857143vs0.28589583333333335<br>pH_circular_variance:0.619:0.5429999999999999vs0.5294791666666667 pH_kyte_doolittle:-0.3502:1.4745142857142857vs-0.13721250000000002
**224**	67	L	pKa:NA:10.55vs11.46
**225**	68	A	pH_kyte_doolittle:0.245:-0.4297714285714286vs-0.8498749999999999 pH_stickiness:0.209:-0.005657142857142856vs0.14127083333333335 pH_conserved:A

			pH_circular_variance:0.6517142857142858:0.6785795918367347vs0.6686636904761905 pH_electrostatics:2.4487272727272726:0.2127090909090909vs1.053810606060606
**226**	69	W	pH_circular_variance:0.6886666666666666:0.6692857142857144vs0.6750763888888889 pH_stickiness:0.209:-0.005657142857142856vs0.14127083333333335 pH_kyte_doolittle:-1.126:-0.8564285714285714vs-1.3797777777777778 pH_electrostatics:2.3804000000000003:0.14750857142857143vs1.1812458333333333
**229**	72	R	pH_conserved:P pH_kyte_doolittle:-2.6734166666666668:-2.229140476190476vs-1.9184826388888887 pH_circular_variance:0.6138:0.6359542857142857vs0.6155166666666666 pH_stickiness:0.0395:-0.06472857142857143vs0.064890625 pH_electrostatics:2.6695:0.6555017857142857vs1.4524440104166667
**230**	73	R	pH_electrostatics:2.0686:0.3328114285714286vs1.4484041666666667 pH_kyte_doolittle:-3.643:-0.6792857142857143vs-1.9372083333333332
**234**	77	H	pKa:4.45:4.17vs5.23
**242**	85	N	pH_stickiness:-0.06883333333333334:0.015695238095238106vs-0.17405555555555555 pH_kyte_doolittle:-3.403:-0.6211333333333334vs-1.7607152777777777 pH_circular_variance:0.6506666666666666:0.6328666666666667vs0.6143055555555555
**244**	87	N	pH_stickiness:-0.12283333333333334:-0.16698095238095237vs0.03597569444444444 pH_electrostatics:1.6828333333333332:-0.13519523809523806vs0.6828819444444444 pH_circular_variance:0.5756:0.57304vs0.570175 pH_kyte_doolittle:-2.3829166666666666:-2.224709523809524vs-1.239282986111111
**245**	88	S	pH_circular_variance:0.57775:0.5634761904761905vs0.5538211805555555 pH_electrostatics:1.8089411764705883:-0.023741176470588224vs0.5963075980392156 pH_stickiness:0.1775:-0.023919047619047618vs0.12706944444444443 pH_kyte_doolittle:-1.5522857142857143:-2.3560979591836735vs-1.3444285714285713
**246**	89	R	pH_electrostatics:1.5796999999999999:-0.28033714285714284vs0.3952333333333333 pH_circular_variance:0.5942000000000001:0.5955485714285714vs0.5758075 pH_kyte_doolittle:-3.3937037037037037:-0.6346084656084656vs-1.0023364197530864 pH_stickiness:-0.0492:0.14141714285714285vs0.13683125000000002
**247**	90	F	pH_electrostatics:1.4124666666666668:-0.13598285714285713vs0.5100486111111111 pH_stickiness:0.628375:0.3996821428571429vs0.3793385416666667 pH_kyte_doolittle:0.5165714285714286:-1.538716326530612vs-0.9014479166666668 pH_circular_variance:0.5283333333333333:0.5527126984126984vs0.5388414351851851
**248**	91	D	pH_circular_variance:0.6122:0.6020114285714285vs0.591254166666666 pH_kyte_doolittle:-0.52:-1.1352285714285715vs-1.7124097222222223 pH_stickiness:-0.0935:-0.18538571428571432vs-0.11553125 pH_electrostatics:1.3825714285714288:-0.21489387755102038vs0.536139880952381 pKa:3.06:3.12vs2.81
**249**	92	G	pH_electrostatics:1.1775:-0.37815714285714286vs0.5810104166666666 pH_stickiness:0.06354545454545456:0.7225688311688312vs0.2641912878787879 pH_kyte_doolittle:-0.7608333333333334:0.7134357142857143vs-1.4064583333333331 pH_circular_variance:0.60575:0.5854714285714285vs0.5684270833333334
**250**	93	P	pH_electrostatics:0.614:-0.7128857142857145vs0.4279375 pH_stickiness:-0.002:0.6385142857142858vs0.3091041666666667 pH_circular_variance:0.582:0.5641714285714287vs0.54775 pH_kyte_doolittle:-1.261:0.7034285714285715vs-0.7988125
**251**	94	D	pH_stickiness:-0.5275714285714286:0.22414489795918366vs-0.03835267857142858 pH_kyte_doolittle:-2.501:-0.15849761904761905vs-1.4441232638888888 pH_circular_variance:0.59275:0.5866428571428571vs0.5711979166666666 pH_electrostatics:0.39685000000000004:-0.4715142857142857vs0.5237302083333334
**252**	95	S	pH_circular_variance:0.5962:0.61332vs0.5951444444444445 pH_electrostatics:1.2027333333333334:-0.1134952380952381vs0.6931708333333333 pH_kyte_doolittle:-1.198:-0.44238333333333335vs-1.3778020833333333 pH_stickiness:-0.094625:-0.026610714285714297vs-0.04080989583333333

**255**	98	S	pH_stickiness:-0.036:-0.1293571428571429vs-0.0047708333333333335 pH_circular_variance:0.578:0.5994571428571429vs0.582984375 pH_electrostatics:1.0807857142857142:0.13688367346938776vs0.8163169642857142 pH_kyte_doolittle:-1.1007500000000001:-1.0412vs-1.4381458333333332
**258**	101	S	pH_kyte_doolittle:-0.86425:-2.0036vs-2.4479479166666667 pH_circular_variance:0.644:0.6341142857142857vs0.6231874999999999 pH_electrostatics:1.6260999999999999:0.13980571428571428vs0.7583604166666666
**259**	102	A	pH_circular_variance:0.6405000000000001:0.6388285714285715vs0.6272187499999999 pH_kyte_doolittle:-0.5796:-1.031685714285714vs-1.6595166666666663 pH_conserved:A pH_electrostatics:1.5462:0.024359999999999993vs0.7275291666666667
**262**	105	N	pH_electrostatics:1.9916470588235295:-0.06499495798319328vs0.6131997549019608 pH_kyte_doolittle:-2.219125:-2.6992142857142856vs-2.8843932291666667 pH_circular_variance:0.594:0.56725vs0.5751041666666666 pH_stickiness:-0.03:-0.39860952380952375vs-0.2549375
**263**	106	Y	pKa:11.6:11.12vs10.52
**265**	108	R	pH_stickiness:-0.06275:-0.23091071428571427vs-0.119640625 pH_kyte_doolittle:-3.658666666666667:-2.9656285714285717vs-2.4080291666666667 pH_circular_variance:0.5637500000000001:0.5241380952380952vs0.5434861111111111 pH_electrostatics:2.4639677419354835:-0.24871612903225807vs0.5847573924731183
**271**	109	T	pH_kyte_doolittle:-2.1695625:-3.4903392857142856vs-2.9671848958333333 pH_electrostatics:2.4332222222222222:-0.04693809523809524vs0.7645416666666667 pH_circular_variance:0.5615000000000001:0.5238571428571429vs0.5276458333333334 pH_stickiness:0.023266666666666665:-0.32307047619047613vs-0.2177875
**274**	110	S	pH_kyte_doolittle:-1.70275:-3.530207142857143vs-2.9784322916666666 pH_stickiness:0.09166666666666666:-0.18837460317460317vs-0.12187731481481483 pH_electrostatics:2.0307222222222222:0.2221031746031746vs1.0249166666666667 pH_circular_variance:0.5485:0.5417857142857142vs0.5279583333333333
**281**	112	P	pH_electrostatics:2.9298333333333333:0.8103142857142857vs1.3316631944444444 pH_circular_variance:0.5982000000000001:0.5675485714285713vs0.5359291666666668 pH_stickiness:0.22325:-0.09845vs0.050875000000000004 pH_kyte_doolittle:-1.731:-2.567457142857143vs-2.1811875
**282**	113	S	pH_stickiness:0.0505:-0.18528214285714284vs-0.05275130208333333 pH_circular_variance:0.5074000000000001:0.5643047619047619vs0.5374083333333334 pH_electrostatics:2.990782608695652:0.5275403726708074vs1.2508233695652173 pH_kyte_doolittle:-2.0491111111111113:-3.4007714285714283vs-2.807523148148148
**283**	114	A	pH_stickiness:0.010499999999999999:-0.2357285714285714vs-0.12539583333333332 pH_circular_variance:0.54925:0.6213857142857142vs0.5963333333333334 pH_electrostatics:3.0811333333333333:0.6791333333333333vs1.3604444444444443
**287**	116	R	pH_conserved:R pH_kyte_doolittle:-2.6666666666666665:-3.564742857142857vs-2.998420634920635 pH_electrostatics:2.7805909090909093:0.16387532467532467vs1.018720643939394 pH_circular_variance:0.5451428571428572:0.5443877551020408vs0.5273943452380953 pH_stickiness:0.0005714285714285709:-0.27819795918367346vs-0.1011235119047619 pKa:12.23:12.47vs12.01
**288**	117	A	pH_electrostatics:2.8642857142857143:-0.2343448979591837vs0.8364776785714286 pH_stickiness:-0.0031666666666666667:-0.35156190476190474vs-0.11347569444444444 pH_kyte_doolittle:-0.9893333333333333:-2.8319952380952382vs-2.152875 pH_circular_variance:0.609:0.5436285714285713vs0.5595
**289**	118	R	pH_stickiness:-0.0032857142857142855:-0.22219999999999998vs-0.09200297619047618 pH_electrostatics:3.0770714285714287:0.11281224489795916vs1.0642083333333334 pH_circular_variance:0.5865:0.5628571428571427vs0.5786458333333333 pH_kyte_doolittle:-0.396:-2.1071428571428577vs-1.6892083333333332

**290**	119	L	pH_stickiness:0.028999999999999998:-0.35544285714285717vs-0.1446875 pH_electrostatics:2.406:-0.6064285714285714vs0.6508055555555555
**291**	120	D	pH_stickiness:-0.127:-0.10217142857142857vs-0.19847916666666668 pH_kyte_doolittle:-1.43:-1.5663714285714285vs-2.0364166666666663 pH_electrostatics:2.242:-0.6103857142857143vs0.6172604166666666
**292**	121	A	pH_electrostatics:2.4556:-0.4931914285714286vs0.6738958333333334 pH_stickiness:-0.045000000000000005:-0.19228571428571428vs-0.10140972222222222 pH_kyte_doolittle:-1.5895000000000001:-1.9819999999999998vs-1.9726666666666663
**293**	122	N	pH_kyte_doolittle:-2.3878333333333335:-1.7651190476190477vs-1.902784722222222 pH_electrostatics:2.132903225806452:-0.34924608294930876vs0.5944509408602151 pH_stickiness:-0.23750000000000002:-0.10766666666666667vs-0.11711111111111111
**300**	129	H	pKa:3.89:6.62vs5.12
**302**	131	M	pH_stickiness:0.6288:0.9866685714285716vs0.6257083333333333 pH_kyte_doolittle:0.23339999999999997:1.6050114285714288vs-0.006458333333333366 pH_electrostatics:0.9762000000000001:-0.6735085714285715vs0.4187333333333333 pH_circular_variance:0.642:0.5895999999999999vs0.5783958333333333
**309**	138	R	pH_circular_variance:0.652:0.5771428571428572vs0.5976845238095239 pH_electrostatics:-0.3133076923076923:-0.7345076923076923vs0.32536217948717944 pH_stickiness:-0.205:-0.19982857142857138vs-0.08966666666666667 pH_kyte_doolittle:-3.379:-2.7938571428571426vs-2.128763888888889
**312**	141	E	pH_stickiness:-0.288:-0.14754857142857142vs-0.12249166666666667 pH_circular_variance:0.5883333333333334:0.5278857142857144vs0.5439236111111111 pH_electrostatics:0.035846153846153854:-0.47967032967032963vs0.44529166666666664 pH_kyte_doolittle:-3.532:-2.763785714285714vs-1.9876718750000002
**313**	142	Q	pH_circular_variance:0.5900000000000001:0.5867571428571428vs0.5885 pH_kyte_doolittle:-3.29875:-3.688642857142857vs-3.0381901041666666 pH_electrostatics:0.5761851851851852:-0.16813121693121694vs0.5788873456790123
**314**	143	N	pH_electrostatics:1.9065:0.1343257142857143vs0.7679666666666666 pH_kyte_doolittle:-2.958:-3.779542857142857vs-3.3058541666666663
**317**	144	P	pH_electrostatics:0.9451212121212121:-0.26065108225108224vs0.5287089646464646 pH_kyte_doolittle:-1.7064666666666668:-1.5501580952380953vs-1.8796347222222223 pH_stickiness:-0.15741666666666668:-0.027710714285714283vs-0.14705034722222224
**318**	145	S	pH_kyte_doolittle:-1.6529999999999998:-1.3068380952380951vs-1.3309166666666667 pH_circular_variance:0.5402857142857143:0.5580489795918367vs0.5478333333333334 pH_electrostatics:1.8222307692307693:-0.0786967032967033vs0.6440416666666666 pH_stickiness:-0.0986:-0.031428571428571424vs-0.056108333333333336
**320**	147	K	pH_kyte_doolittle:-3.2015000000000002:-2.632192857142857vs-2.157619791666667 pH_stickiness:-0.6214999999999999:-0.5661071428571429vs-0.43046875 pH_electrostatics:2.1044761904761904:-0.27869931972789114vs0.5195029761904761 pH_circular_variance:0.6757272727272727:0.6702831168831168vs0.6447727272727273
**330**	155	W	pH_stickiness:0.4957142857142857:0.8854938775510206vs0.6358095238095238 pH_circular_variance:0.6033333333333334:0.6423333333333333vs0.6191180555555555 pH_electrostatics:0.8775000000000001:-0.7877542857142857vs0.40196250000000006 pH_kyte_doolittle:-0.5953333333333333:0.9768158730158729vs0.12489814814814812
**332**	157	T	pH_electrostatics:0.57936:-0.8775314285714285vs0.3753133333333334 pH_circular_variance:0.5368095238095238:0.5958027210884353vs0.5637500000000001 pH_kyte_doolittle:-1.077:-0.38834065934065937vs-0.2977612179487179 pH_stickiness:-0.07615384615384616:0.37147252747252746vs0.2796201923076923
**334**	158	D	pH_electrostatics:-0.24911764705882355:-1.030418487394958vs0.24835049019607844 pH_kyte_doolittle:-2.2555:-1.9378714285714285vs-1.4097708333333334 pH_stickiness:-0.46900000000000003:-0.03387142857142857vs-0.031593750000000004 pH_circular_variance:0.5594444444444444:0.5582380952380953vs0.5300231481481482

**335**	159	K	pH_kyte_doolittle:-3.14575:-1.306vs-1.8573020833333334 pH_stickiness:-0.7543124999999999:0.28668392857142855vs-0.035110677083333326 pH_electrostatics:0.5835263157894737:-1.0487293233082706vs0.3885548245614035 pH_circular_variance:0.5863125:0.6185553571428571vs0.5782747395833333
**336**	160	T	pH_circular_variance:0.5662727272727273:0.549987012987013vs0.5350094696969697 pH_kyte_doolittle:-1.9572222222222224:-1.0295936507936507vs-1.7163472222222222 pH_electrostatics:0.043333333333333334:-0.8573292517006802vs0.35940873015873015 pH_stickiness:-0.3335:-0.029542857142857155vs-0.1627708333333333
**339**	162	N	pH_kyte_doolittle:-3.206285714285714:-2.595520408163265vs-2.110483630952381 pH_electrostatics:0.2571851851851852:-0.5158116402116403vs0.43075385802469135 pH_stickiness:-0.2271:-0.17625428571428572vs-0.09498958333333334 pH_circular_variance:0.5405000000000001:0.5223428571428572vs0.5236597222222222
**341**	164	S	pH_stickiness:0.033:-0.07148253968253969vs-0.19029861111111113 pH_circular_variance:0.5202:0.5286228571428572vs0.5134124999999999 pH_kyte_doolittle:-1.089:-3.077257142857143vs-2.693729166666667 pH_electrostatics:0.6541111111111111:-0.6894444444444444vs0.12216550925925923
**343**	166	P	pH_electrostatics:1.1372499999999999:-1.3254214285714285vs-0.383859375
**350**	173	E	pH_stickiness:-0.2242:-0.03623428571428572vs-0.2510833333333333 pH_electrostatics:-1.6577142857142857:-1.1797816326530612vs-0.39363690476190477 pH_circular_variance:0.5599000000000001:0.5656685714285714vs0.5703020833333333 pH_kyte_doolittle:-1.826857142857143:-1.6053918367346938vs-2.5490357142857145
**351**	174	A	pH_stickiness:-0.0498:-0.22111428571428574vs-0.07747083333333334 pH_electrostatics:-0.7545000000000001:-1.0917816326530614vs-0.11427529761904763 pH_circular_variance:0.49642857142857144:0.5391469387755102vs0.5228571428571429 pH_kyte_doolittle:-0.875625:-1.0770892857142857vs-1.7311510416666667
**352**	175	D	pKa:2.44:3.01vs2.75
**353**	176	T	pH_stickiness:0.060333333333333334:0.04907619047619049vs0.11175694444444446 pH_circular_variance:0.47633333333333333:0.49051746031746035vs0.4877037037037037 pH_kyte_doolittle:-0.21850000000000003:-0.21050476190476194vs-0.9343420138888888 pH_electrostatics:-0.21100000000000002:-1.1054125714285714vs-0.06956333333333334
**354**	177	V	pH_electrostatics:0.561:-0.9170928571428572vs0.19795833333333332 pH_kyte_doolittle:0.953:0.9277285714285715vs0.029208333333333336 pH_circular_variance:0.458:0.4756857142857142vs0.49416666666666664 pH_stickiness:0.5895:0.47174285714285713vs0.5002500000000001
**356**	179	P	pH_kyte_doolittle:-1.584:0.43057142857142855vs-0.037520833333333316 pH_circular_variance:0.5016666666666666:0.5225809523809525vs0.5076805555555556 pH_stickiness:-0.12:0.25014285714285717vs0.18216666666666667 pH_electrostatics:-0.17166666666666666:-1.0689968253968254vs-0.1419583333333333
**357**	180	V	pH_circular_variance:0.6126666666666667:0.6275238095238095vs0.6070069444444445 pH_electrostatics:-1.5883333333333332:-1.566857142857143vs-0.64625 pH_kyte_doolittle:-1.65:-1.7470857142857141vs-2.3194583333333334
**358**	181	S	pH_electrostatics:-0.2539:-0.9705114285714286vs-0.03389687499999999 pH_circular_variance:0.5406666666666666:0.5455714285714286vs0.5346898148148148
**359**	182	Q	pH_kyte_doolittle:-1.869:0.34609999999999996vs-0.08104166666666666 pH_electrostatics:0.45477272727272733:-0.9953870129870129vs0.1993285984848485 pH_circular_variance:0.5283:0.6040514285714286vs0.5820791666666667 pH_stickiness:-0.4308181818181818:0.4395844155844156vs0.23741287878787878
**360**	183	H	pH_conserved:Y
**362**	185	I	pH_electrostatics:-0.7485999999999999:-1.222897142857143vs-0.249775 pH_circular_variance:0.612:0.6290857142857142vs0.6069722222222222 pH_kyte_doolittle:-1.65:-1.7470857142857141vs-2.3194583333333334 pKa:NA:4.08vs9.42

**363**	186	P	pH_stickiness:-0.637:0.01848571428571428vs-0.11725000000000001 pH_circular_variance:0.526:0.4910571428571428vs0.5034791666666667 pH_kyte_doolittle:-2.919:-1.181790476190476vs-1.834270833333333 pH_electrostatics:0.29816666666666664:-0.8907428571428572vs0.31089930555555556
**365**	188	Y	pH_electrostatics:0.2538333333333333:-1.5134285714285716vs-0.32045138888888886 pH_kyte_doolittle:-2.9939999999999998:-3.7091142857142856vs-3.2589375 pH_circular_variance:0.6556666666666667:0.5727619047619048vs0.6174270833333334 pKa:11.65:11.20vs11.51
**366**	189	Q	pH_circular_variance:0.5523571428571429:0.5280306122448979vs0.5444360119047619 pH_stickiness:-0.211:-0.07485714285714286vs-0.1866875 pH_electrostatics:0.29888:-0.9229131428571428vs0.21296083333333332 pH_kyte_doolittle:-3.353:-3.841714285714286vs-3.5215416666666663
**367**	190	N	pH_stickiness:-0.3725:-0.03142142857142858vs-0.158859375 pH_kyte_doolittle:-2.6263636363636365:-1.0212363636363635vs-1.7654943181818181 pH_electrostatics:0.1618235294117647:-1.0418319327731091vs0.33877696078431374 pH_circular_variance:0.580375:0.5614392857142858vs0.5560494791666667
**369**	192	P	pH_circular_variance:0.5666666666666667:0.5528190476190477vs0.5370625 pH_kyte_doolittle:-1.747:-0.5585357142857144vs-1.336807291666667 pH_electrostatics:0.45333333333333337:-0.9028349206349205vs0.3062546296296296 pH_stickiness:-0.002:-0.037757142857142864vs-0.07161458333333333
**370**	193	S	pH_stickiness:0.0435:-0.01465714285714286vs-0.04963541666666667 pH_electrostatics:0.2758148148148148:-1.4961079365079366vs-0.28350077160493825 pH_kyte_doolittle:-1.4894:-0.4935371428571429vs-1.0474333333333334 pH_circular_variance:0.5262:0.4958114285714286vs0.5396625
**371**	194	T	pH_kyte_doolittle:-0.8520000000000001:-0.47838775510204073vs-1.0122470238095238 pH_stickiness:0.053714285714285714:-0.04960612244897959vs-0.07688392857142858 pH_circular_variance:0.44370588235294117:0.4796773109243697vs0.4545808823529412 pH_electrostatics:0.5681999999999999:-1.135389523809524vs-0.2330131944444445
**373**	196	P	pH_stickiness:-0.001:-0.2628857142857143vs-0.17802083333333335 pH_circular_variance:0.533:0.5239600000000001vs0.5110791666666666 pH_electrostatics:0.7278888888888889:-1.8964063492063492vs-0.990386574074074
**374**	197	K	pH_circular_variance:0.5740000000000001:0.5259714285714286vs0.5714270833333334 pH_electrostatics:0.34299999999999997:-1.6996vs-0.2868177083333333
**376**	199	Y	pKa:14.49:12.68vs13.46
**378**	201	E	pH_circular_variance:0.637:0.6437714285714286vs0.6272083333333334 pH_kyte_doolittle:-2.221:-1.8313571428571427vs-2.6523854166666663 pH_stickiness:-0.338:-0.12537142857142858vs-0.32014583333333335 pKa:5.23:4.76vs4.43 pH_electrostatics:-2.1855:-1.6428714285714285vs-0.7769791666666666
**380**	203	C	pH_stickiness:-0.132:-0.001228571428571432vs0.15377083333333333 pH_kyte_doolittle:-1.1980000000000002:0.6457047619047619vs-0.4421458333333333 pH_electrostatics:-0.5277499999999999:-0.3664928571428572vs0.24034375
**381**	204	N	pH_circular_variance:0.4891666666666667:0.5367428571428572vs0.5371458333333333 pH_electrostatics:-0.465551724137931:-0.589016748768473vs0.17917169540229885 pH_kyte_doolittle:-2.0664000000000002:-0.39337714285714287vs-1.0281479166666667 pH_stickiness:-0.1615294117647059:-0.17517815126050418vs-0.054098039215686275
**382**	205	A	pH_kyte_doolittle:-0.538:-2.009342857142857vs-1.0702916666666666 pH_conserved:A pH_electrostatics:0.9285:-0.2410428571428571vs0.5061822916666667 pH_circular_variance:0.5535:0.5515142857142857vs0.5778541666666667
**383**	206	S	pH_stickiness:0.301:-0.24461904761904762vs0.0004999999999999993 pH_circular_variance:0.5603636363636363:0.569612987012987vs0.571564393939394 pH_electrostatics:0.6046428571428571:-0.911704081632653vs0.33924404761904764 pH_kyte_doolittle:0.9864444444444445:-1.9761333333333333vs-0.9902476851851851

**384**	207	H	pKa:6.74:5.93vs6.30
**385**	208	I	pH_kyte_doolittle:2.217071428571429:-1.3343714285714285vs-0.7754627976190476 pH_electrostatics:0.9169411764705883:-0.7990168067226892vs0.27417034313725486 pH_stickiness:0.662:-0.18231999999999998vs0.10306250000000002 pH_circular_variance:0.5531818181818182:0.5761922077922077vs0.5789810606060606
**386**	209	A	pH_electrostatics:1.149:-0.3627714285714286vs0.4916666666666667 pH_circular_variance:0.622:0.5659142857142859vs0.5969375 pH_kyte_doolittle:1.104:-2.813942857142857vs-1.2558749999999999
**389**	211	N	pH_kyte_doolittle:-0.7037500000000001:1.4957714285714285vs-0.18860937500000002 pH_stickiness:0.122:0.226vs0.08775 pH_circular_variance:0.619:0.5429999999999999vs0.5294791666666667 pH_electrostatics:1.1795714285714285:-0.6570979591836734vs0.2917529761904762
**393**	212	S	pH_stickiness:0.209:-0.005657142857142856vs0.14127083333333335 pH_electrostatics:1.37645:-0.10864vs0.6327083333333333 pH_circular_variance:0.56575:0.5505142857142857vs0.543765625 pH_kyte_doolittle:-0.994736842105263:-2.858012030075188vs-1.3690076754385965
**396**	213	N	pH_stickiness:-0.14600000000000002:-0.12678571428571428vs-0.033624999999999995 pH_electrostatics:1.8494444444444444:0.1602198412698413vs1.1632216435185185 pH_kyte_doolittle:-2.71256:-1.4997645714285717vs-1.4802016666666666 pH_circular_variance:0.5237142857142858:0.5723115646258503vs0.5460714285714287
**398**	214	N	pH_electrostatics:1.4767142857142856:0.08600408163265308vs0.851842261904762 pH_kyte_doolittle:-1.83425:-2.2602714285714285vs-1.5218541666666667 pH_stickiness:-0.166:-0.11642857142857141vs-0.0255625
**400**	215	A	pH_kyte_doolittle:-1.205:-1.6105571428571426vs-1.2046354166666666 pH_circular_variance:0.614:0.5652142857142858vs0.5890625 pH_electrostatics:1.743090909090909:0.1814779220779221vs1.0300246212121211 pH_stickiness:-0.097:-0.12444571428571427vs-0.030266666666666664
**407**	219	V	pH_conserved:R
**416**	228	W	pH_circular_variance:0.591:0.5786285714285714vs0.5973333333333334 pH_kyte_doolittle:-2.7859999999999996:-2.3552714285714287vs-2.3287604166666664 pH_electrostatics:1.737769230769231:-1.3196703296703296vs-0.3618044871794872
**419**	231	N	pH_stickiness:0.04933333333333334:-0.04667619047619048vs-0.04746527777777778 pH_electrostatics:2.2306:-0.6664342857142856vs0.26721249999999996 pH_kyte_doolittle:-2.6239999999999997:-2.6935142857142855vs-2.26740625 pH_circular_variance:0.6082000000000001:0.5953428571428571vs0.5762083333333333
**421**	233	T	pH_electrostatics:2.656388888888889:-0.7628142857142857vs0.07991435185185185 pH_circular_variance:0.506:0.4595485714285714vs0.4849458333333333 pH_stickiness:0.00025000000000000001:-0.20839999999999997vs-0.11823697916666667
**422**	234	R	pH_circular_variance:0.546:0.49902857142857143vs0.5205 pH_kyte_doolittle:-3.4435000000000002:-3.3078000000000003vs-2.9231249999999998 pH_stickiness:-0.115:-0.31264897959183674vs-0.20769047619047618 pH_electrostatics:2.6274444444444445:-0.8123111111111112vs0.07058796296296296
**424**	236	R	pH_kyte_doolittle:-3.766:-0.923763492063492vs-1.6168541666666667 pH_conserved:S pH_stickiness:-0.008545454545454544:0.19789870129870132vs0.0955378787878788 pH_circular_variance:0.5555:0.5676714285714286vs0.5858958333333333 pH_electrostatics:2.21964:-1.1216445714285714vs-0.3043475
**425**	237	Q	pH_kyte_doolittle:-2.916166666666667:-2.3371666666666666vs-1.8237135416666668 pH_stickiness:-0.31666666666666665:-0.3137vs-0.2272951388888889 pH_electrostatics:2.0068333333333332:-0.6828095238095239vs-0.010381944444444452 pH_circular_variance:0.49:0.5394vs0.5236614583333334
**428**	240	C	pH_electrostatics:1.3463333333333332:-1.2252190476190474vs-0.5601666666666667 pH_circular_variance:0.5344285714285715:0.5218897959183674vs0.5466875

			pH_stickiness:0.222:0.6108571428571429vs0.45045833333333335 pH_kyte_doolittle:-1.3885:0.8195571428571429vs0.29309375
**429**	241	N	pH_electrostatics:0.865764705882353:-1.1204621848739496vs-0.5129154411764706 pH_kyte_doolittle:-3.396:-0.9897071428571428vs-1.3430208333333333 pH_stickiness:-0.269:0.00025714285714286035vs-0.07725 pH_circular_variance:0.4665882352941177:0.5053142857142857vs0.5097022058823529
**432**	243	N	pH_kyte_doolittle:-2.681142857142857:-0.8563795918367346vs-1.2002440476190477 pH_electrostatics:-0.562875:-0.5513821428571428vs-0.0020468750000000036 pH_circular_variance:0.45380000000000004:0.5500171428571429vs0.5195833333333334 pH_stickiness:-0.223375:-0.0014678571428571444vs0.171890625
**446**	245	P	pH_electrostatics:-0.2006:-0.3042095238095238vs0.23203611111111108 pH_kyte_doolittle:-1.4653333333333334:-0.4468666666666666vs-0.8441458333333333 pH_stickiness:-0.16283333333333333:0.03796904761904762vs0.19067534722222224
**449**	247	L	pH_kyte_doolittle:-0.701:0.3759428571428572vs-0.7917916666666667 pH_electrostatics:-1.456:-0.7867142857142856vs-0.1720625
**450**	248	C	pH_stickiness:0.3102307692307692:0.22073406593406594vs-0.05290865384615385 pH_electrostatics:-1.6018333333333334:-0.7448857142857143vs-0.11918402777777777 pH_circular_variance:0.5768888888888889:0.575720634920635vs0.5890555555555556 pH_kyte_doolittle:-0.146:0.19345357142857142vs-1.708859375
**451**	249	D	pH_electrostatics:-1.603:-0.8720857142857142vs-0.28029166666666666 pH_kyte_doolittle:-0.9891666666666667:-0.5866166666666667vs-2.078595486111111 pH_stickiness:0.16066666666666665:0.20584047619047618vs-0.08736979166666665 pH_circular_variance:0.6031428571428571:0.5935632653061224vs0.6078392857142857
**452**	250	F	pH_kyte_doolittle:0.245:-0.4390761904761904vs-1.4805624999999998 pH_stickiness:0.5125000000000001:0.3629047619047619vs0.1769791666666667 pH_circular_variance:0.67025:0.6404285714285715vs0.6294375
**453**	251	R	pH_stickiness:-0.162:-0.019876190476190474vs-0.24085069444444443 pH_electrostatics:-0.6571666666666667:-1.2359714285714285vs-0.3278344907407407 pH_kyte_doolittle:-3.158583333333333:-2.4410166666666666vs-3.1459774305555555 pH_circular_variance:0.6198823529411764:0.5956554621848739vs0.6082205882352941
**454**	252	T	pH_circular_variance:0.5465:0.5507071428571428vs0.5713567708333334 pH_kyte_doolittle:-0.7163999999999999:-0.29007428571428573vs-0.9443270833333333 pH_electrostatics:-0.43894736842105264:-2.1714135338345866vs-1.2904177631578946 pH_stickiness:0.09727272727272727:0.10816103896103896vs-0.019405303030303033
**536**		-	pKa::12.79vs11.69
**594**		-	pKa::11.83vs12.29
**598**		-	pKa::11.22vs9.96

Machine learning performance

Table S6: Performance of starting and tuned HMMs for each condition.

starting alignment	type	pH	T	substrate	AUROC
**D1-Scraped-513**	starting	7.5	40	aFilm	0.76167472
**D1-Scraped-513**	starting	7.5	60	aFilm	0.79452055
**D1-Scraped-513**	starting	6.5	40	aFilm	0.76167472
**D1-Scraped-513**	starting	7.5	60	cryPow	0.60074108
**D1-Scraped-513**	starting	6.5	60	cryPow	0.59709962
**D1-Scraped-513**	starting	5.5	60	cryPow	0.56173966
**D1-Scraped-513**	starting	7.5	40	cryPow	0.52997312
**D1-Scraped-513**	starting	6.5	40	cryPow	0.51280347
**D1-Scraped-513**	starting	4.5	40	cryPow	0.49108571
**D1-Scraped-513**	starting	5.5	40	cryPow	0.47325368
**D1-Scraped-513**	starting	8.5	40	cryPow	0.46464646
**D1-Scraped-513**	tuned	7.5	40	aFilm	0.80193237
**D1-Scraped-513**	tuned	7.5	60	aFilm	0.79452055
**D1-Scraped-513**	tuned	6.5	40	aFilm	0.74074074
**D1-Scraped-513**	tuned	6.5	40	cryPow	0.67827397
**D1-Scraped-513**	tuned	5.5	40	cryPow	0.63694853
**D1-Scraped-513**	tuned	7.5	40	cryPow	0.61818996
**D1-Scraped-513**	tuned	4.5	40	cryPow	0.59497143
**D1-Scraped-513**	tuned	7.5	60	cryPow	0.58869847
**D1-Scraped-513**	tuned	6.5	60	cryPow	0.51786465
**D1-Scraped-513**	tuned	5.5	60	cryPow	0.48935523
**D1-Scraped-513**	tuned	8.5	40	cryPow	0.45887446
**hmm-17**	starting	7.5	60	aFilm	0.87123288
**hmm-17**	starting	7.5	40	aFilm	0.85829308
**hmm-17**	starting	6.5	40	aFilm	0.82608696
**hmm-17**	starting	7.5	60	cryPow	0.7172302
**hmm-17**	starting	6.5	60	cryPow	0.66624632
**hmm-17**	starting	5.5	60	cryPow	0.65663017
**hmm-17**	starting	7.5	40	cryPow	0.55349462
**hmm-17**	starting	6.5	40	cryPow	0.51107567
**hmm-17**	starting	5.5	40	cryPow	0.47003676
**hmm-17**	starting	8.5	40	cryPow	0.45887446
**hmm-17**	starting	4.5	40	cryPow	0.448
**hmm-17**	tuned	7.5	60	aFilm	0.86027397
**hmm-17**	tuned	7.5	40	aFilm	0.8115942
**hmm-17**	tuned	6.5	40	aFilm	0.74235105

**hmm-17**	tuned	4.5	40	cryPow	0.73348571
**hmm-17**	tuned	5.5	40	cryPow	0.70606618
**hmm-17**	tuned	6.5	40	cryPow	0.66764133
**hmm-17**	tuned	7.5	40	cryPow	0.60170251
**hmm-17**	tuned	7.5	60	cryPow	0.54955998
**hmm-17**	tuned	5.5	60	cryPow	0.52007299
**hmm-17**	tuned	6.5	60	cryPow	0.44976881
**hmm-17**	tuned	8.5	40	cryPow	0.44877345
**hmm-61**	starting	7.5	60	aFilm	0.76986301
**hmm-61**	starting	7.5	40	aFilm	0.74879227
**hmm-61**	starting	6.5	40	aFilm	0.74074074
**hmm-61**	starting	7.5	60	cryPow	0.58012969
**hmm-61**	starting	6.5	60	cryPow	0.57608239
**hmm-61**	starting	5.5	60	cryPow	0.54653285
**hmm-61**	starting	7.5	40	cryPow	0.51684588
**hmm-61**	starting	6.5	40	cryPow	0.50930356
**hmm-61**	starting	4.5	40	cryPow	0.50834286
**hmm-61**	starting	5.5	40	cryPow	0.47417279
**hmm-61**	starting	8.5	40	cryPow	0.43867244
**hmm-61**	tuned	7.5	40	aFilm	0.80676329
**hmm-61**	tuned	7.5	60	aFilm	0.76438356
**hmm-61**	tuned	6.5	40	aFilm	0.7294686
**hmm-61**	tuned	5.5	40	cryPow	0.69154412
**hmm-61**	tuned	4.5	40	cryPow	0.68
**hmm-61**	tuned	6.5	40	cryPow	0.65266702
**hmm-61**	tuned	7.5	40	cryPow	0.57840502
**hmm-61**	tuned	7.5	60	cryPow	0.56206577
**hmm-61**	tuned	5.5	60	cryPow	0.52524331
**hmm-61**	tuned	6.5	60	cryPow	0.50504414
**hmm-61**	tuned	8.5	40	cryPow	0.45743146

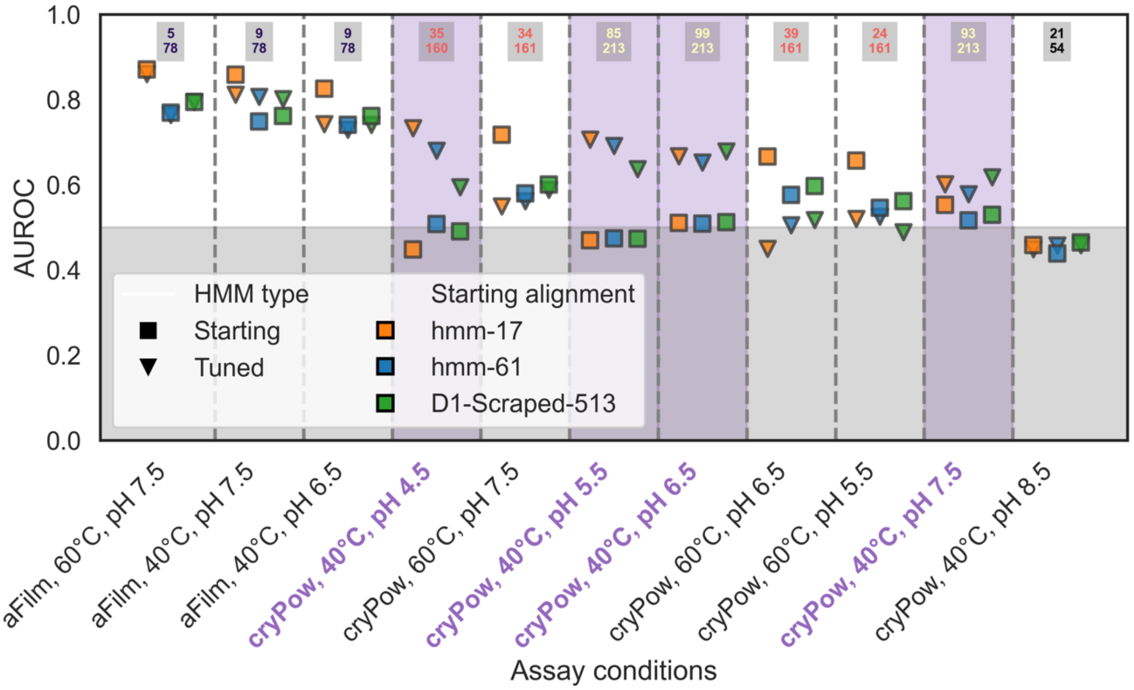

Figure S10: Ability of HMMs scraped from literature to differentiate active PETase homologs against various conditions with and without "tuning" using active PETases at that target condition, triangles and squares respectively. Scores computed in 5-fold cross validation. Total number tested and number of actives found given as numbers at the top of each column (bottom and top, respectively).

Table S7: Precision scores (hit rate) and AUROC of supervised models on the dataset in hindsight, 5-fold CV. Missing row/data indicate too few actives tested to determine scores.

Substrate	T[C]	pH	Count	Num active	Precision (hit rate)	AUROC
**cryPow**	40	5.5	213	85	0.75	0.79
**cryPow**	60	5.5	161	24	0.50	0.61
**aFilm**	40	6.5	78	9		0.68
**cryPow**	40	6.5	213	99	0.74	0.78
**cryPow**	60	6.5	161	39	0.69	0.68
**aFilm**	40	7.5	78	9		0.75
**cryPow**	40	7.5	213	93	0.69	0.75
**aFilm**	60	7.5	78	5		0.91
**cryPow**	60	7.5	161	34	0.77	0.78
**cryPow**	40	8.5	54	21	0.33	0.46
**cryPow**	40	4.5	160	35	0.79	0.77

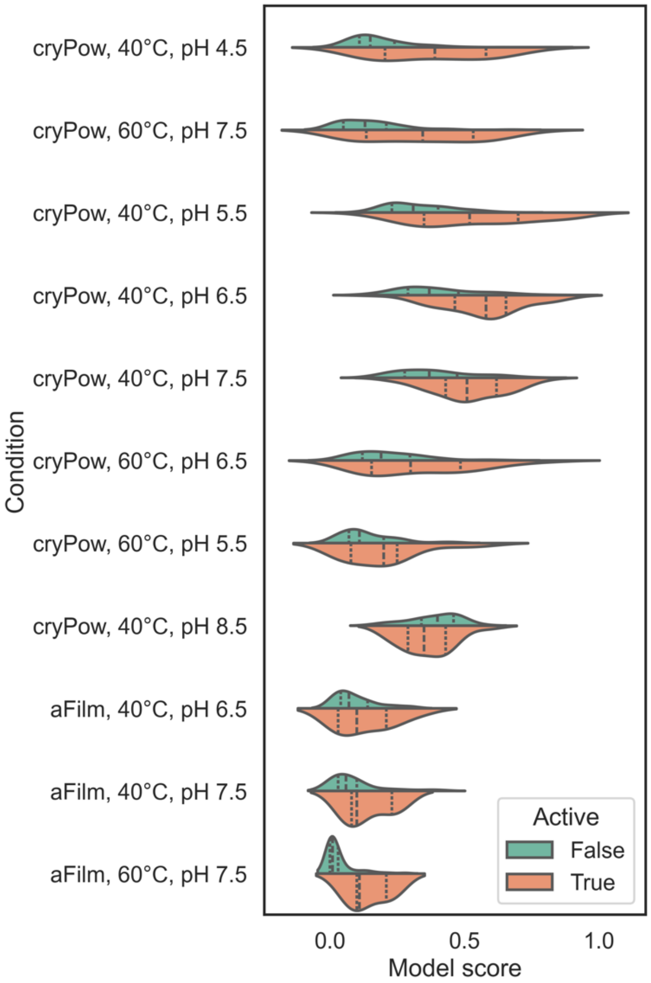

Figure S11: Parity in 5-fold CV for models trained on each condition set using our assay data. Only conditions for which we tested a sufficient quantity of data to estimate CV scores are shown. In most cases, the bulk of active enzymes receive a significantly higher score than inactive, and in some cases the model is effective and saturating a filtered set with active enzymes.

References

(1) Steinegger, M.; Söding, J. MMseqs2 Enables Sensitive Protein Sequence Searching for the Analysis of Massive Data Sets. *Nat. Biotechnol.* **2017**, *35* (11), 1026–1028.

(2) Katoh, K. MAFFT: A Novel Method for Rapid Multiple Sequence Alignment Based on Fast Fourier Transform. *Nucleic Acids Res.* **2002**, *30* (14), 3059–3066.

(3) Hopf, T. A.; Ingraham, J. B.; Poelwijk, F. J.; Schärfe, C. P. I.; Springer, M.; Sander, C.; Marks, D. S. Mutation Effects Predicted from Sequence Co-Variation. *Nat. Biotechnol.* **2017**, *35* (2), 128–135.

(4) Gado, J. E.; Knotts, M.; Shaw, A. Y.; Marks, D.; Gauthier, N. P.; Sander, C.; Beckham, G. T. Machine Learning Prediction of Enzyme Optimum pH. *Nat. Mach. Intell.* **2025**.

(5) Teufel, F.; Almagro Armenteros, J. J.; Johansen, A. R.; Gíslason, M. H.; Pihl, S. I.; Tsirigos, K. D.; Winther, O.; Brunak, S.; Von Heijne, G.; Nielsen, H. SignalP 6.0 Predicts All Five Types of Signal Peptides Using Protein Language Models. *Nat. Biotechnol.* **2022**, *40* (7), 1023–1025.

(6) Elnaggar, A.; Heinzinger, M.; Dallago, C.; Rehawi, G.; Wang, Y.; Jones, L.; Gibbs, T.; Feher, T.; Angerer, C.; Steinegger, M.; Bhowmik, D.; Rost, B. ProtTrans: Toward Understanding the Language of Life through Self-Supervised Learning. *IEEE Trans. Pattern Anal. Mach. Intell.* **2022**, *44* (10), 7112–7127.

(7) Lin, Z.; Akin, H.; Rao, R.; Hie, B.; Zhu, Z.; Lu, W.; Smetanin, N.; Verkuil, R.; Kabeli, O.; Shmueli, Y.; Fazel-Zarandi, M.; Sercu, T.; Candido, S.; Rives, A. Evolutionary-Scale Prediction of Atomic-Level Protein Structure with a Language Model. *Science* **2023**, No. 379, 1123–1130.

(8) Norton-Baker, B.; Denton, M. C. R.; Murphy, N. P.; Fram, B.; Lim, S.; Erickson, E.; Gauthier, N. P.; Beckham, G. T. Enabling High-Throughput Enzyme Discovery and Engineering with a Low-Cost, Robot-Assisted Pipeline. *Sci. Rep.* **2024**, *14* (1), 14449.

(9) Waterhouse, A. M.; Procter, J. B.; Martin, D. M. A.; Clamp, M.; Barton, G. J. Jalview Version 2—a Multiple Sequence Alignment Editor and Analysis Workbench. *Bioinformatics* **2009**, *25* (9), 1189–1191.

(10) Schweke, H.; Mucchielli, M.-H.; Chevrollier, N.; Gosset, S.; Lopes, A. SURFMAP: A Software for Mapping in Two Dimensions Protein Surface Features. *J. Chem. Inf. Model* **2022**, *62* (7), 1595–1601.

(11) Mirdita, M.; Schütze, K.; Moriwaki, Y.; Heo, L.; Ovchinnikov, S.; Steinegger, M. ColabFold: Making Protein Folding Accessible to All. *Nat. Methods* **2022**, *19* (6), 679–682.

(12) Dong, R.; Peng, Z.; Zhang, Y.; Yang, J. mTM-Align: An Algorithm for Fast and Accurate Multiple Protein Structure Alignment. *Bioinformatics* **2018**, *34* (10), 1719–1725.

(13) Jurrus, E.; Engel, D.; Star, K.; Monson, K.; Brandi, J.; Felberg, L. E.; Brookes, D. H.; Wilson, L.; Chen, J.; Liles, K.; Chun, M.; Li, P.; Gohara, D. W.; Dolinsky, T.; Konecny, R.; Koes, D. R.; Nielsen, J. E.; Head-Gordon, T.; Geng, W.; Krasny, R.; Wei, G.; Holst, M. J.; McCammon, J. A.; Baker, N. A. Improvements to the APBS Biomolecular Solvation Software Suite. *Protein Sci.* **2018**, *27* (1), 112–128.

(14) Olsson, M. H. M.; Søndergaard, C. R.; Rostkowski, M.; Jensen, J. H. PROPKA3: Consistent Treatment of Internal and Surface Residues in Empirical pK_a Predictions. *J. Chem. Theory Comput.* **2011**, *7* (2), 525–537.

(15) Rodella, C.; Lazaridi, S.; Lemmin, T. TemBERTure: Advancing Protein Thermostability Prediction with Deep Learning and Attention Mechanisms. *Bioinform. Adv.* **2024**, *4* (1), vbae103.

(16) Bell, E. L.; Smithson, R.; Kilbride, S.; Foster, J.; Hardy, F. J.; Ramachandran, S.; Tedstone, A. A.; Haigh, S. J.; Garforth, A. A.; Day, P. J. R.; Levy, C.; Shaver, M. P.; Green, A. P. Directed Evolution of an Efficient and Thermostable PET Depolymerase. *Nat. Catal.* **2022**, *5* (8), 673–681.

(17) Brott, S.; Pfaff, L.; Schuricht, J.; Schwarz, J.; Böttcher, D.; Badenhorst, C. P. S.; Wei, R.; Bornscheuer, U. T. Engineering and Evaluation of Thermostable *Is*PETase Variants for PET Degradation. *Eng. Life Sci.* **2022**, *22* (3–4), 192–203.

(18) Chen, C.-C.; Han, X.; Li, X.; Jiang, P.; Niu, D.; Ma, L.; Liu, W.; Li, S.; Qu, Y.; Hu, H.; Min, J.; Yang, Y.; Zhang, L.; Zeng, W.; Huang, J.-W.; Dai, L.; Guo, R.-T. General Features to Enhance Enzymatic Activity of Poly(Ethylene Terephthalate) Hydrolysis. *Nat. Catal.* **2021**, *4* (5), 425–430.

(19) Cui, Y.; Chen, Y.; Liu, X.; Dong, S.; Tian, Y.; Qiao, Y.; Mitra, R.; Han, J.; Li, C.; Han, X.; Liu, W.; Chen, Q.; Wei, W.; Wang, X.; Du, W.; Tang, S.; Xiang, H.; Liu, H.; Liang, Y.; Houk, K. N.; Wu, B. Computational Redesign of a PETase for Plastic Biodegradation under Ambient Condition by the GRAPE Strategy. *ACS Catal.* **2021**, *11* (3), 1340–1350.
(20) Erickson, E.; Gado, J. E.; Avilán, L.; Bratti, F.; Brizendine, R. K.; Cox, P. A.; Gill, R.; Graham, R.; Kim, D.-J.; König, G.; Michener, W. E.; Poudel, S.; Ramirez, K. J.; Shakespeare, T. J.; Zahn, M.; Boyd, E. S.; Payne, C. M.; DuBois, J. L.; Pickford, A. R.; Beckham, G. T.; McGeehan, J. E. Sourcing Thermotolerant Poly(Ethylene Terephthalate) Hydrolase Scaffolds from Natural Diversity. *Nat. Comm.* **2022**, *13* (1), 7850.
(21) Furukawa, M.; Kawakami, N.; Tomizawa, A.; Miyamoto, K. Efficient Degradation of Poly(Ethylene Terephthalate) with Thermobifida Fusca Cutinase Exhibiting Improved Catalytic Activity Generated Using Mutagenesis and Additive-Based Approaches. *Sci. Rep.* **2019**, *9* (1), 16038.
(22) Guo, B.; Vanga, S. R.; Lopez-Lorenzo, X.; Saenz-Mendez, P.; Ericsson, S. R.; Fang, Y.; Ye, X.; Schriever, K.; Bäckström, E.; Biundo, A.; Zubarev, R. A.; Furó, I.; Hakkarainen, M.; Syrén, P.-O. Conformational Selection in Biocatalytic Plastic Degradation by PETase. *ACS Catal.* **2022**, *12* (6), 3397–3409.
(23) Han, X.; Liu, W.; Huang, J.-W.; Ma, J.; Zheng, Y.; Ko, T.-P.; Xu, L.; Cheng, Y.-S.; Chen, C.-C.; Guo, R.-T. Structural Insight into Catalytic Mechanism of PET Hydrolase. *Nat. Comm.* **2017**, *8* (1), 2106.
(24) Joo, S.; Cho, I. J.; Seo, H.; Son, H. F.; Sagong, H.-Y.; Shin, T. J.; Choi, S. Y.; Lee, S. Y.; Kim, K.-J. Structural Insight into Molecular Mechanism of Poly(Ethylene Terephthalate) Degradation. *Nat. Comm.* **2018**, *9* (1), 382.
(25) Li, Q.; Zheng, Y.; Su, T.; Wang, Q.; Liang, Q.; Zhang, Z.; Qi, Q.; Tian, J. Computational Design of a Cutinase for Plastic Biodegradation by Mining Molecular Dynamics Simulations Trajectories. *Comput. Struct. Biotechnol. J.* **2022**, *20*, 459–470.
(26) Li, Z.; Zhao, Y.; Wu, P.; Wang, H.; Li, Q.; Gao, J.; Qin, H.-M.; Wei, H.; Bornscheuer, U. T.; Han, X.; Wei, R.; Liu, W. Structural Insight and Engineering of a Plastic Degrading Hydrolase Ple629. *Biochem. Biophys. Res. Commun.* **2022**, *626*, 100–106.
(27) Liu, B.; He, L.; Wang, L.; Li, T.; Li, C.; Liu, H.; Luo, Y.; Bao, R. Protein Crystallography and Site-Direct Mutagenesis Analysis of the Poly(Ethylene Terephthalate) Hydrolase PETase from *Ideonella Sakaiensis*. *ChemBioChem* **2018**, *19* (14), 1471–1475.
(28) Lu, H.; Diaz, D. J.; Czarnecki, N. J.; Zhu, C.; Kim, W.; Shroff, R.; Acosta, D. J.; Alexander, B. R.; Cole, H. O.; Zhang, Y.; Lynd, N. A.; Ellington, A. D.; Alper, H. S. Machine Learning-Aided Engineering of Hydrolases for PET Depolymerization. *Nature* **2022**, *604* (7907), 662–667.
(29) Ma, Y.; Yao, M.; Li, B.; Ding, M.; He, B.; Chen, S.; Zhou, X.; Yuan, Y. Enhanced Poly(Ethylene Terephthalate) Hydrolase Activity by Protein Engineering. *Engineering* **2018**, *4* (6), 888–893.
(30) Nakamura, A.; Kobayashi, N.; Koga, N.; Iino, R. Positive Charge Introduction on the Surface of Thermostabilized PET Hydrolase Facilitates PET Binding and Degradation. *ACS Catal.* **2021**, *11* (14), 8550–8564.
(31) Pfaff, L.; Gao, J.; Li, Z.; Jäckering, A.; Weber, G.; Mican, J.; Chen, Y.; Dong, W.; Han, X.; Feiler, C. G.; Ao, Y.-F.; Badenhorst, C. P. S.; Bednar, D.; Palm, G. J.; Lammers, M.; Damborsky, J.; Strodel, B.; Liu, W.; Bornscheuer, U. T.; Wei, R. Multiple Substrate Binding Mode-Guided Engineering of a Thermophilic PET Hydrolase. *ACS Catal.* **2022**, *12* (15), 9790–9800.
(32) Sagong, H.-Y.; Kim, S.; Lee, D.; Hong, H.; Lee, S. H.; Seo, H.; Kim, K.-J. Structural and Functional Characterization of an Auxiliary Domain-Containing PET Hydrolase from Burkholderiales Bacterium. *J. Hazard. Mater.* **2022**, *429*, 128267.
(33) Son, H. F.; Cho, I. J.; Joo, S.; Seo, H.; Sagong, H.-Y.; Choi, S. Y.; Lee, S. Y.; Kim, K.-J. Rational Protein Engineering of Thermo-Stable PETase from *Ideonella Sakaiensis* for Highly Efficient PET Degradation. *ACS Catal.* **2019**, *9* (4), 3519–3526.
(34) Sonnendecker, C.; Oeser, J.; Richter, P. K.; Hille, P.; Zhao, Z.; Fischer, C.; Lippold, H.; Blázquez-Sánchez, P.; Engelberger, F.; Ramírez-Sarmiento, C. A.; Oeser, T.; Lihanova, Y.; Frank, R.; Jahnke, H.; Billig, S.; Abel, B.; Sträter, N.; Matysik, J.; Zimmermann, W. Low Carbon Footprint Recycling of Post-consumer PET Plastic with a Metagenomic Polyester Hydrolase. *ChemSusChem* **2022**, *15* (9), e202101062.

(35) Then, J.; Wei, R.; Oeser, T.; Gerdts, A.; Schmidt, J.; Barth, M.; Zimmermann, W. A Disulfide Bridge in the Calcium Binding Site of a Polyester Hydrolase Increases Its Thermal Stability and Activity against Polyethylene Terephthalate. *FEBS Open Bio* **2016**, *6* (5), 425–432.
(36) Tournier, V.; Topham, C. M.; Gilles, A.; David, B.; Folgoas, C.; Moya-Leclair, E.; Kamionka, E.; Desrousseaux, M.-L.; Texier, H.; Gavalda, S.; Cot, M.; Guémard, E.; Dalibey, M.; Nomme, J.; Cioci, G.; Barbe, S.; Chateau, M.; André, I.; Duquesne, S.; Marty, A. An Engineered PET Depolymerase to Break down and Recycle Plastic Bottles. *Nature* **2020**, *580* (7802), 216–219.
(37) Wang, X.; Song, C.; Qi, Q.; Zhang, Y.; Li, R.; Huo, L. Biochemical Characterization of a Polyethylene Terephthalate Hydrolase and Design of High-Throughput Screening for Its Directed Evolution. *Eng. Microbiol.* **2022**, *2* (2), 100020.
(38) Wei, R.; Oeser, T.; Schmidt, J.; Meier, R.; Barth, M.; Then, J.; Zimmermann, W. Engineered Bacterial Polyester Hydrolases Efficiently Degrade Polyethylene Terephthalate Due to Relieved Product Inhibition. *Biotechnol. Bioeng.* **2016**, *113* (8), 1658–1665.
(39) Xi, X.; Ni, K.; Hao, H.; Shang, Y.; Zhao, B.; Qian, Z. Secretory Expression in *Bacillus Subtilis* and Biochemical Characterization of a Highly Thermostable Polyethylene Terephthalate Hydrolase from Bacterium HR29. *Enzyme Microb. Technol.* **2021**, *143*, 109715.
(40) Zeng, W.; Li, X.; Yang, Y.; Min, J.; Huang, J.-W.; Liu, W.; Niu, D.; Yang, X.; Han, X.; Zhang, L.; Dai, L.; Chen, C.-C.; Guo, R.-T. Substrate-Binding Mode of a Thermophilic Pet Hydrolase and Engineering the Enzyme to Enhance the Hydrolytic Efficacy. *ACS Catal.* **2022**, *12* (5), 3033–3040.
(41) Zhang, H.; Perez-Garcia, P.; Dierkes, R. F.; Applegate, V.; Schumacher, J.; Chibani, C. M.; Sternagel, S.; Preuss, L.; Weigert, S.; Schmeisser, C.; Danso, D.; Pleiss, J.; Almeida, A.; Höcker, B.; Hallam, S. J.; Schmitz, R. A.; Smits, S. H. J.; Chow, J.; Streit, W. R. The Bacteroidetes *Aequorivita* Sp. and *Kaistella Jeonii* Produce Promiscuous Esterases With PET-Hydrolyzing Activity. *Front. Microbiol.* **2022**, *12*, 803896.
(42) Jarzab, A.; Kurzawa, N.; Hopf, T.; Moerch, M.; Zecha, J.; Leijten, N.; Bian, Y.; Musiol, E.; Maschberger, M.; Stoehr, G.; Becher, I.; Daly, C.; Samaras, P.; Mergner, J.; Spanier, B.; Angelov, A.; Werner, T.; Bantscheff, M.; Wilhelm, M.; Klingenspor, M.; Lemeer, S.; Liebl, W.; Hahne, H.; Savitski, M. M.; Kuster, B. Meltome Atlas—Thermal Proteome Stability across the Tree of Life. *Nat. Methods* **2020**, *17* (5), 495–503.
(43) Verkuil, R.; Kabeli, O.; Du, Y.; Wicky, B. I. M.; Milles, L. F.; Dauparas, J.; Baker, D.; Ovchinnikov, S.; Sercu, T.; Rives, A. Language Models Generalize beyond Natural Proteins. *bioRxiv*, December 22, 2022. DOI: 10.1101/2022.12.21.521521
(44) Yang, K. K.; Fusi, N.; Lu, A. X. Convolutions Are Competitive with Transformers for Protein Sequence Pretraining. *Cell Systems* **2024**, *15* (3), 286-294.e2.
(45) Notin, P.; Dias, M.; Frazer, J.; Li, S. S.; Green, T.; Benaim, S.; Jumper, J.; Hassabis, D.; Marks, D. S.; Fowler, D. M. Tranception: Protein Fitness Prediction with Autoregressive Transformers and Inference-Time Retrieval. In *International Conference on Machine Learning*; *Proceedings of Machine Learning Research*, Vol. 162; PMLR: Baltimore, MD, 2022; pp 16990–17017.
(46) Alley, E. C.; Khimulya, G.; Biswas, S.; AlQuraishi, M.; Church, G. M. Unified Rational Protein Engineering with Sequence-Based Deep Representation Learning. *Nat. Methods* **2019**, *16* (12), 1315–1322.
(47) Nijkamp, E.; Ruffolo, J. A.; Weinstein, E. N.; Naik, N.; Madani, A. ProGen2: Exploring the Boundaries of Protein Language Models. *Cell Systems* **2023**, *14* (11), 968-978.e3.
(48) Seo, H.; Hong, H.; Park, J.; Lee, S. H.; Ki, D.; Ryu, A.; Sagong, H.-Y.; Kim, K.-J. Landscape Profiling of PET Depolymerases Using a Natural Sequence Cluster Framework. *Science* **2025**, *387* (6729), eadp5637.
(49) Danso, D.; Schmeisser, C.; Chow, J.; Zimmermann, W.; Wei, R.; Leggewie, C.; Li, X.; Hazen, T.; Streit, W. R. New Insights into the Function and Global Distribution of Polyethylene Terephthalate (PET)-Degrading Bacteria and Enzymes in Marine and Terrestrial Metagenomes. *Appl. Environ. Microbiol.* **2018**, *84* (8), e02773-17.
