# Supplementary figures and images for: Machine Learning-Guided Identification of PET Hydrolases from Natural Diversity
Source: ACS Catal. 2025 Sep 3;15(18):16070–83. doi: 10.1021/acscatal.5c03460 (PMC12455559; doi:10.1021/acscatal.5c03460)

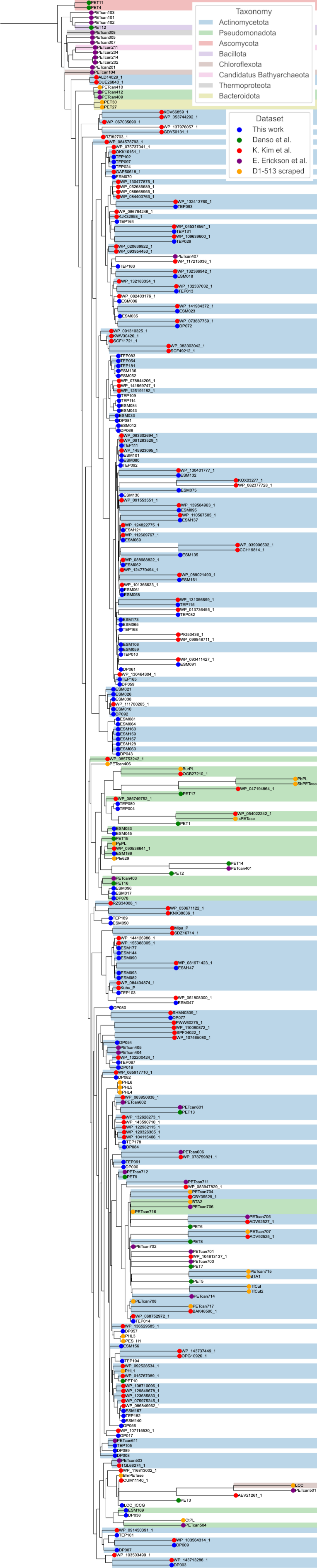

Supplement: Supplementary file 3 [file cs5c03460_si_003.pdf]
