# Supplementary material for: Machine Learning-Guided Identification of PET Hydrolases from Natural Diversity
Source: ACS Catal. 2025 Sep 3;15(18):16070–83. doi: 10.1021/acscatal.5c03460 (PMC12455559; doi:10.1021/acscatal.5c03460)
Supplement: Supplementary file 4 [file cs5c03460_si_004.pdf]

Appendix B

Observed density distributions of predicted pKa values of amino acids where a statistically significant difference was observed for low pH activity over active sequence alignment, column in alignment.

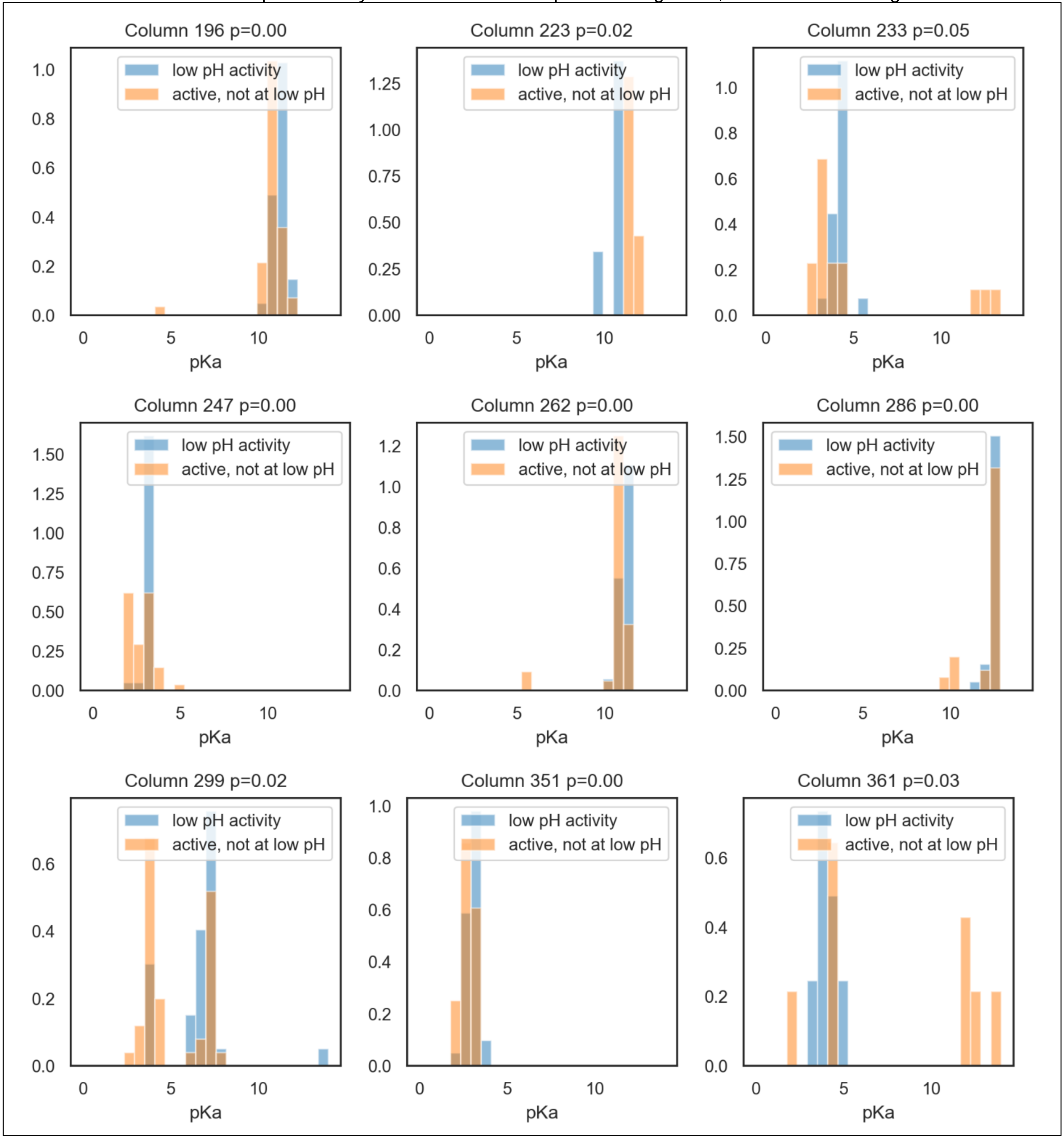

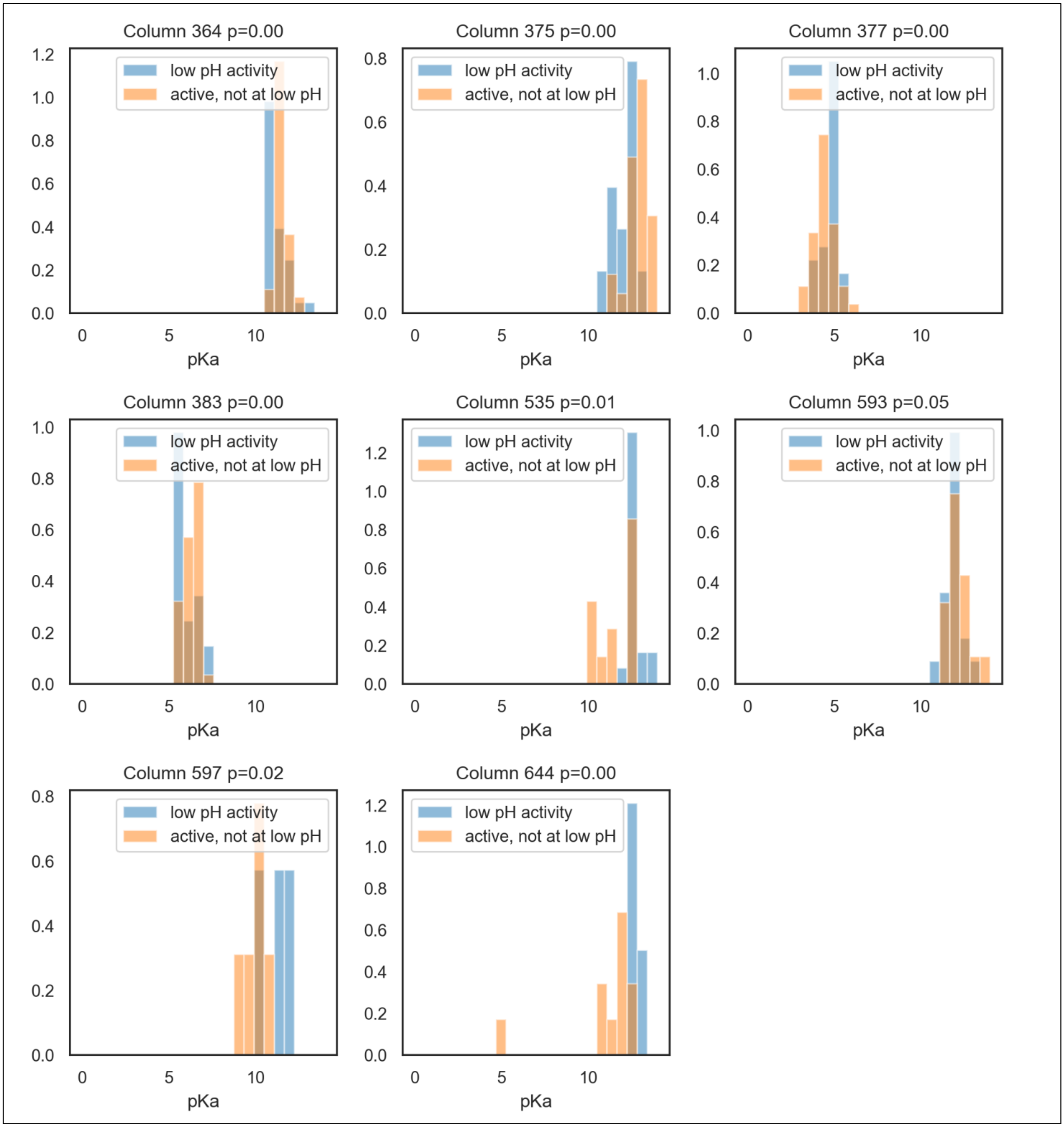
Column 364 p=0.00
Column 375 p=0.00
Column 377 p=0.00
Column 383 p=0.00
Column 535 p=0.01
Column 593 p=0.05
Column 597 p=0.02
Column 644 p=0.00
low pH activity
active, not at low pH
pKa

Observed distributions of amino acids where statistically significant difference in conservation properties was observed for low pH activity over active sequence alignment.

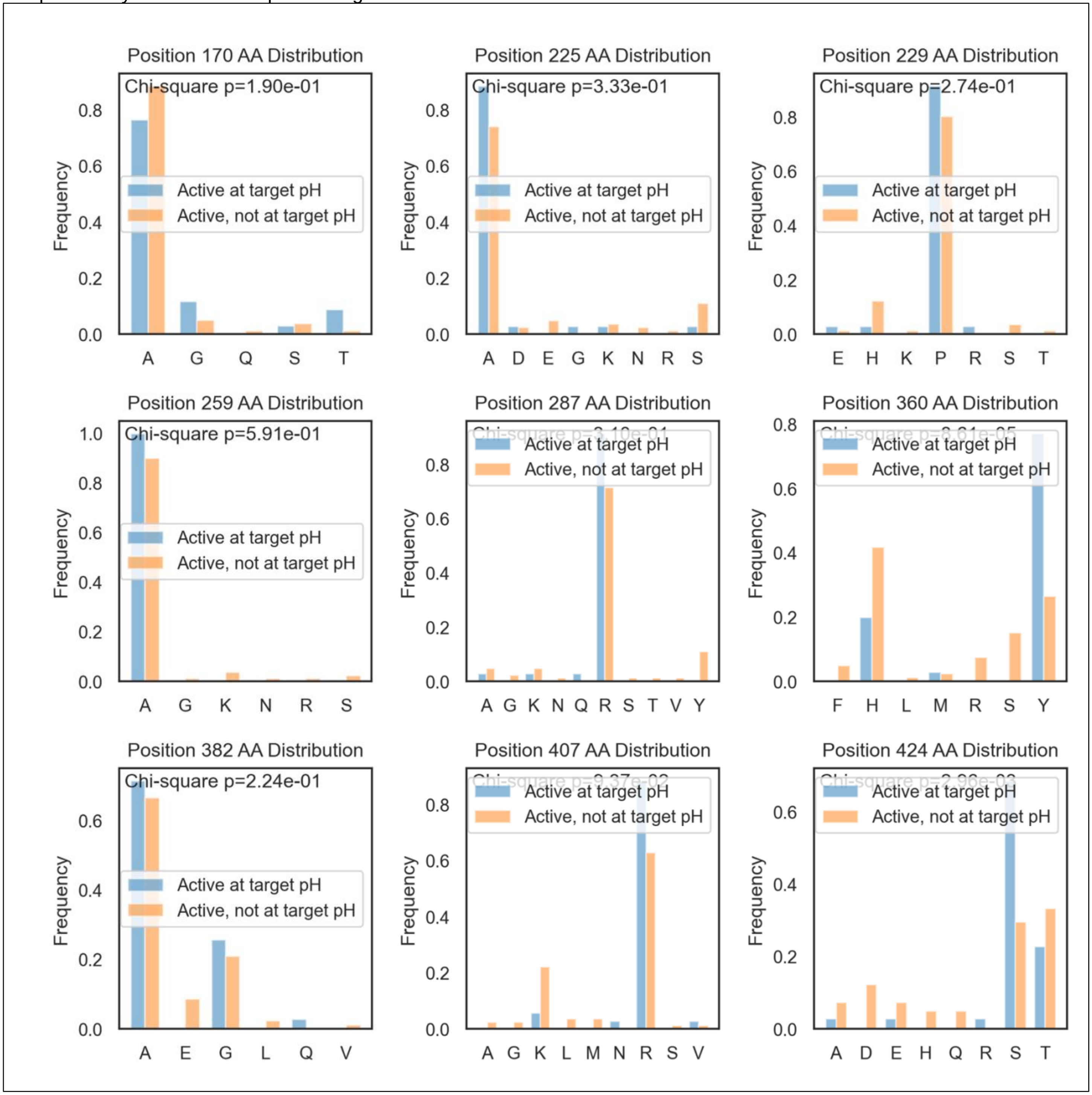

Observed distributions of surface properties where they were found to have statistically significant differences, mapped back to nearest surface residue on LCC-ICCG.

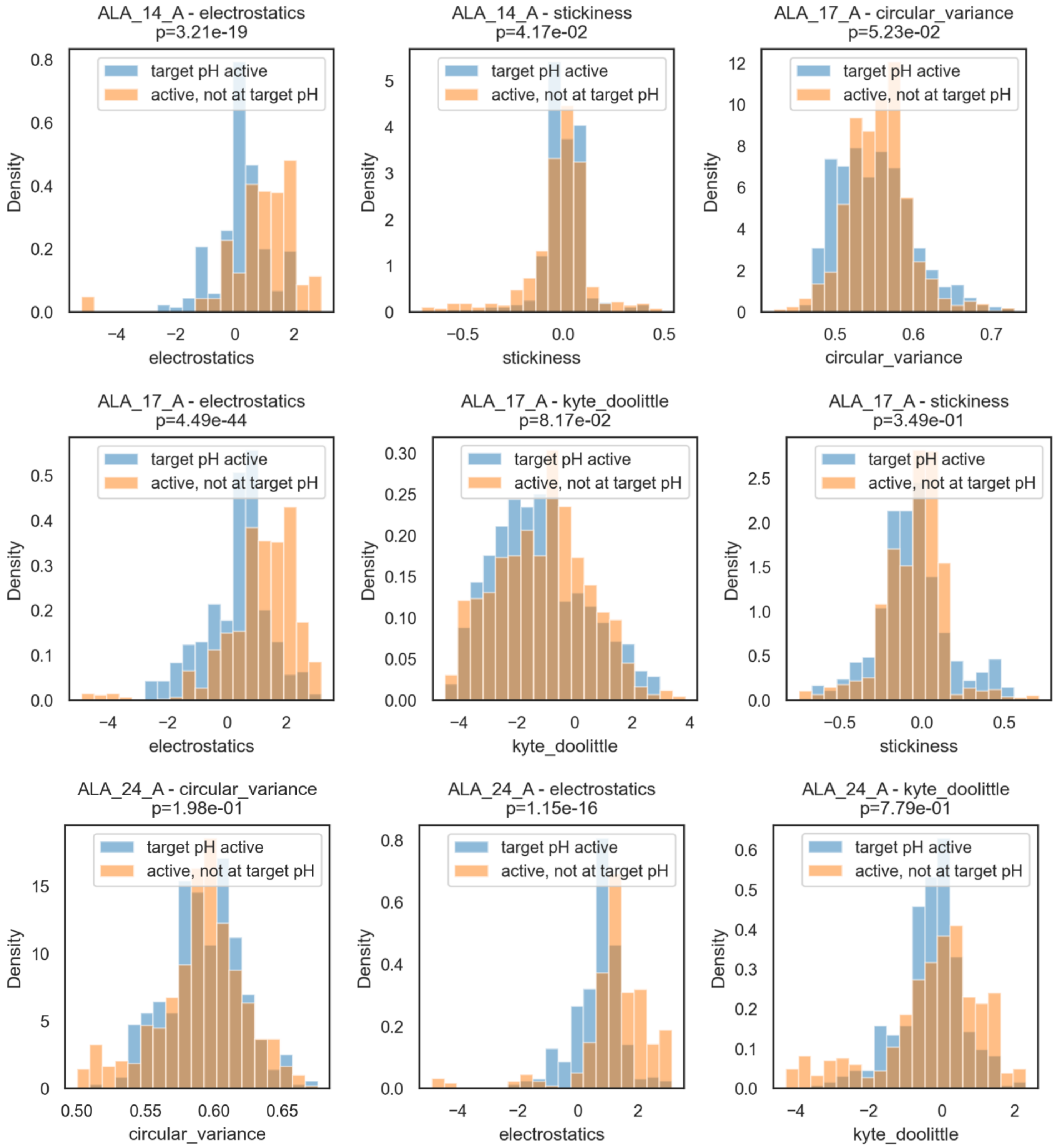

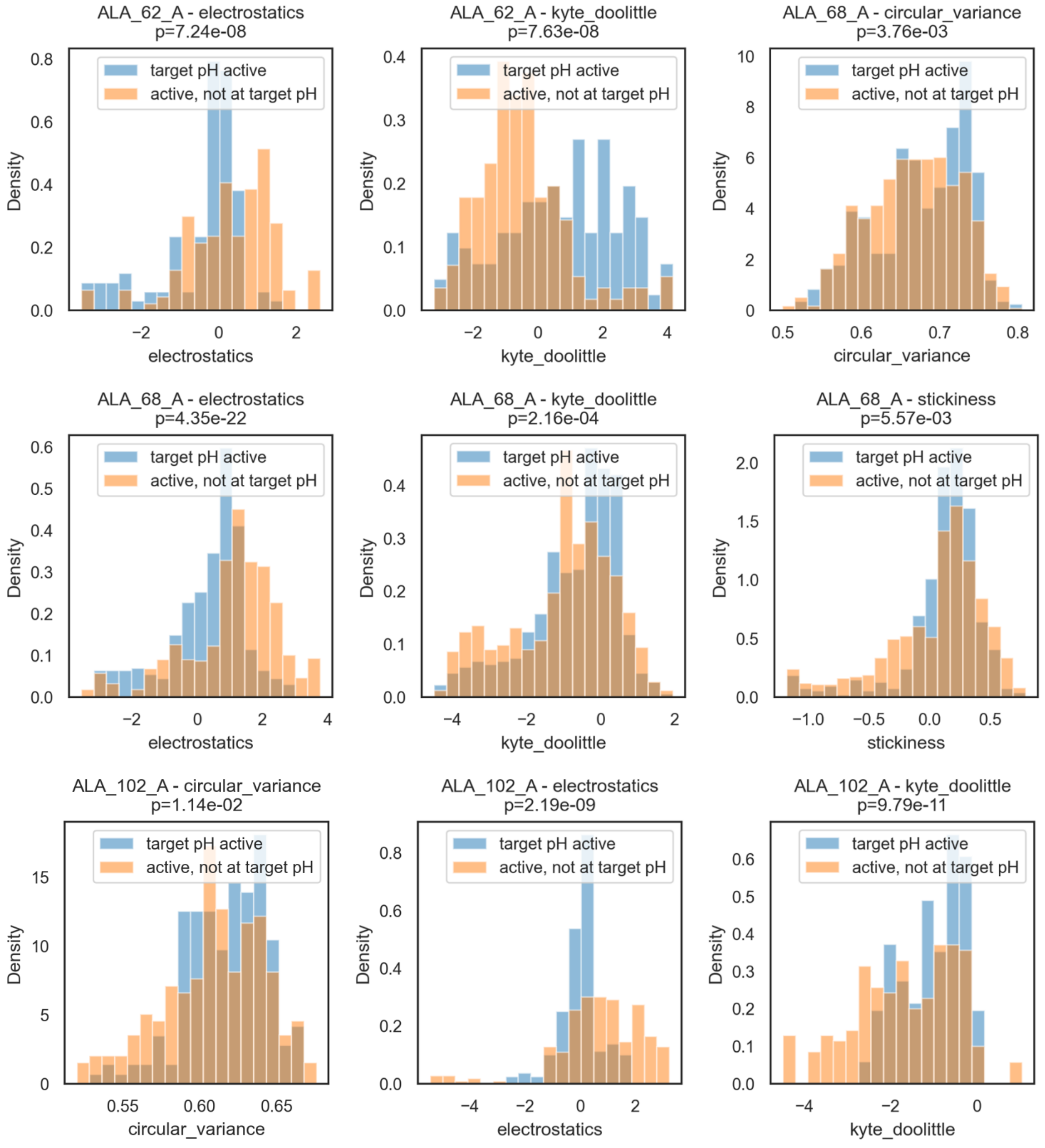
ALA_62_A - electrostatics
p=7.24e-08
target pH active
active, not at target pH
Density
electrostatics
ALA_62_A - kyte_doolittle
p=7.63e-08
target pH active
active, not at target pH
Density
kyte_doolittle
ALA_68_A - circular_variance
p=3.76e-03
target pH active
active, not at target pH
Density
circular_variance
ALA_68_A - electrostatics
p=4.35e-22
target pH active
active, not at target pH
Density
electrostatics
ALA_68_A - kyte_doolittle
p=2.16e-04
target pH active
active, not at target pH
Density
kyte_doolittle
ALA_68_A - stickiness
p=5.57e-03
target pH active
active, not at target pH
Density
stickiness
ALA_102_A - circular_variance
p=1.14e-02
target pH active
active, not at target pH
Density
circular_variance
ALA_102_A - electrostatics
p=2.19e-09
target pH active
active, not at target pH
Density
electrostatics
ALA_102_A - kyte_doolittle
p=9.79e-11
target pH active
active, not at target pH
Density
kyte_doolittle

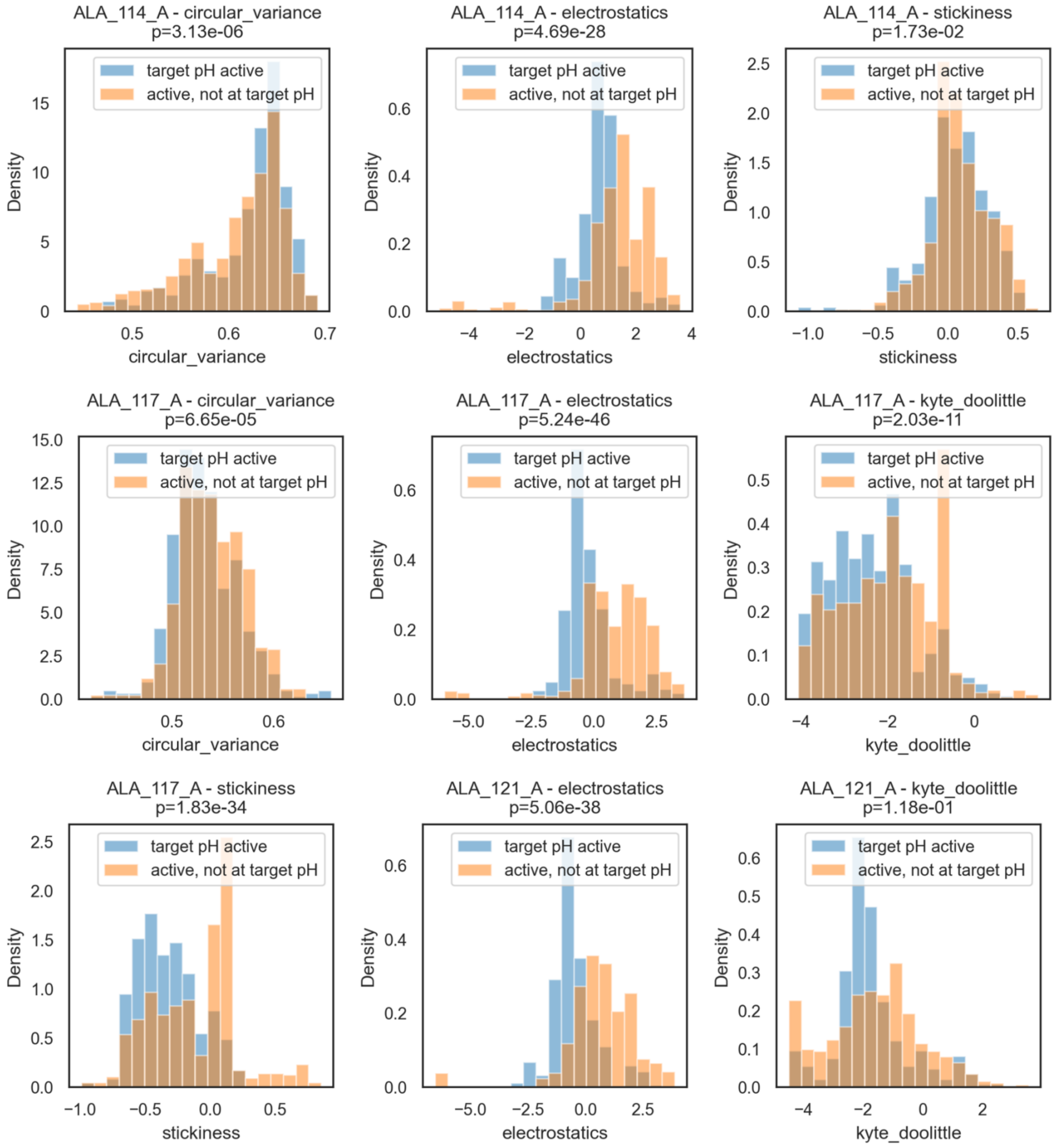
ALA_114_A - circular_variance
p=3.13e-06
target pH active
active, not at target pH
Density
circular_variance
ALA_114_A - electrostatics
p=4.69e-28
target pH active
active, not at target pH
Density
electrostatics
ALA_114_A - stickiness
p=1.73e-02
target pH active
active, not at target pH
Density
stickiness
ALA_117_A - circular_variance
p=6.65e-05
target pH active
active, not at target pH
Density
circular_variance
ALA_117_A - electrostatics
p=5.24e-46
target pH active
active, not at target pH
Density
electrostatics
ALA_117_A - kyte_doolittle
p=2.03e-11
target pH active
active, not at target pH
Density
kyte_doolittle
ALA_117_A - stickiness
p=1.83e-34
target pH active
active, not at target pH
Density
stickiness
ALA_121_A - electrostatics
p=5.06e-38
target pH active
active, not at target pH
Density
electrostatics
ALA_121_A - kyte_doolittle
p=1.18e-01
target pH active
active, not at target pH
Density
kyte_doolittle

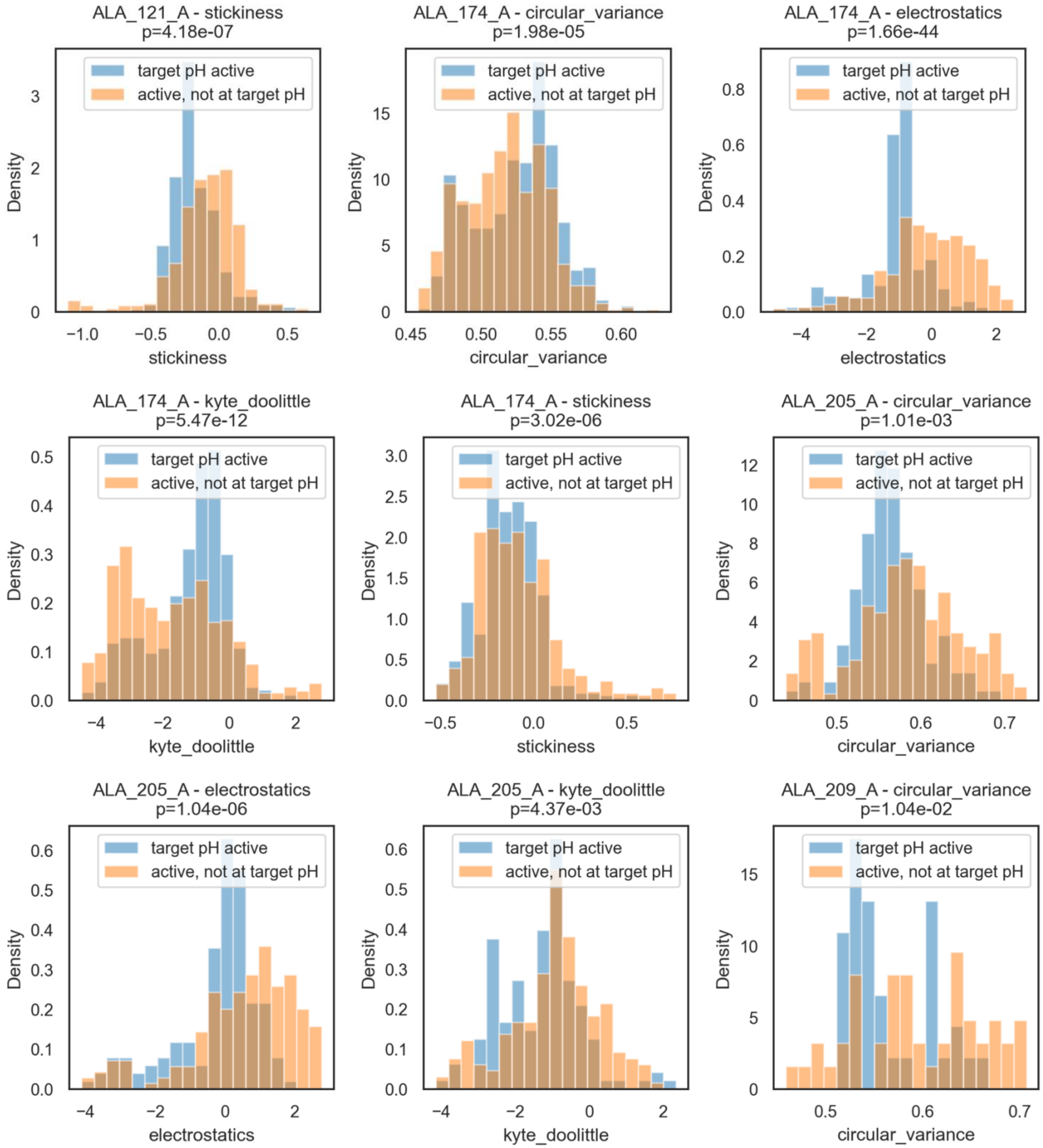
ALA_121_A - stickiness
p=4.18e-07
ALA_174_A - circular_variance
p=1.98e-05
ALA_174_A - electrostatics
p=1.66e-44
ALA_174_A - kyte_doolittle
p=5.47e-12
ALA_174_A - stickiness
p=3.02e-06
ALA_205_A - circular_variance
p=1.01e-03
ALA_205_A - electrostatics
p=1.04e-06
ALA_205_A - kyte_doolittle
p=4.37e-03
ALA_209_A - circular_variance
p=1.04e-02
target pH active
active, not at target pH
Density
stickiness
circular_variance
electrostatics
kyte_doolittle

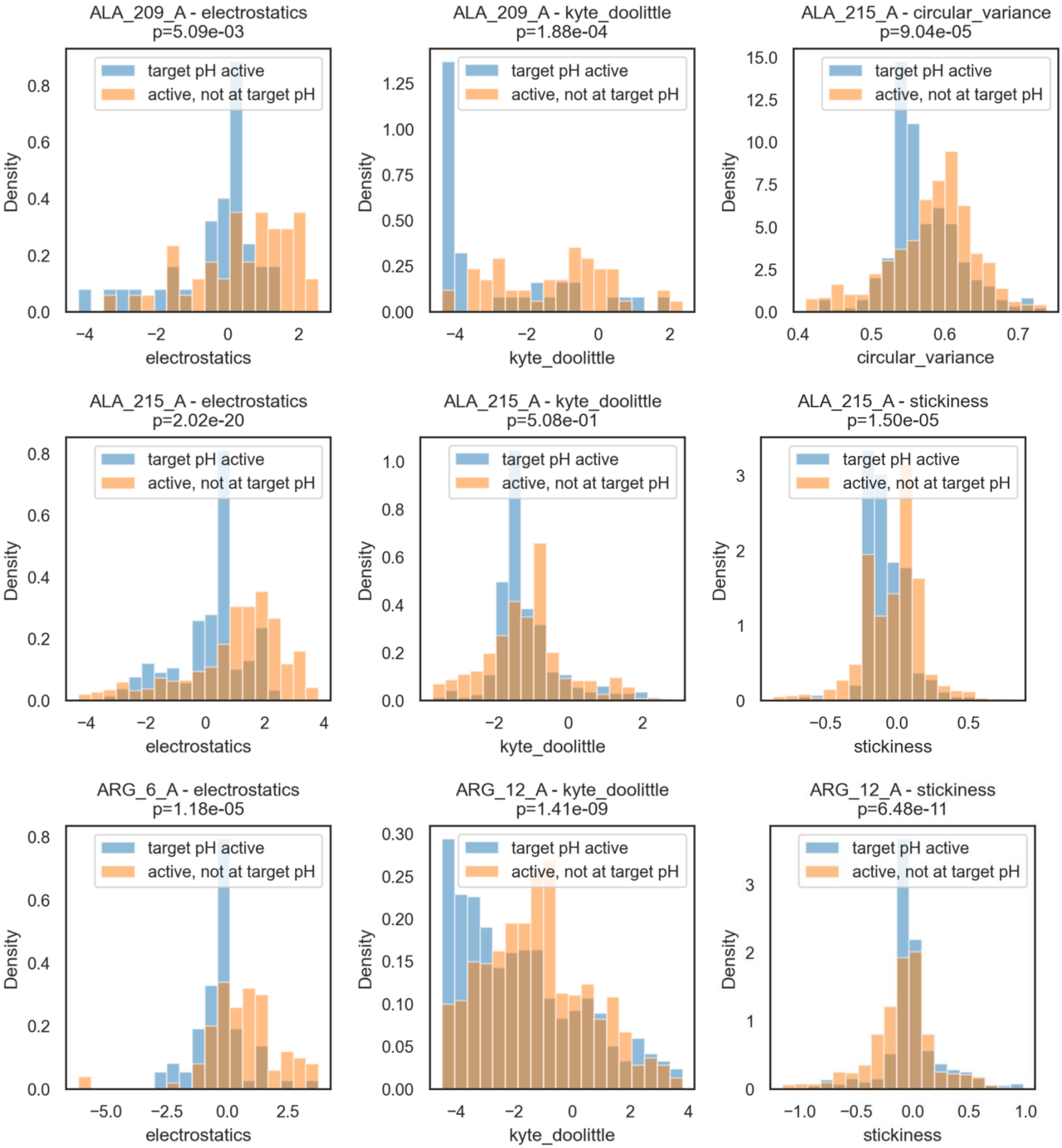
ALA_209_A - electrostatics
p=5.09e-03
target pH active
active, not at target pH
Density
electrostatics
ALA_209_A - kyte_doolittle
p=1.88e-04
target pH active
active, not at target pH
Density
kyte_doolittle
ALA_215_A - circular_variance
p=9.04e-05
target pH active
active, not at target pH
Density
circular_variance
ALA_215_A - electrostatics
p=2.02e-20
target pH active
active, not at target pH
Density
electrostatics
ALA_215_A - kyte_doolittle
p=5.08e-01
target pH active
active, not at target pH
Density
kyte_doolittle
ALA_215_A - stickiness
p=1.50e-05
target pH active
active, not at target pH
Density
stickiness
ARG_6_A - electrostatics
p=1.18e-05
target pH active
active, not at target pH
Density
electrostatics
ARG_12_A - kyte_doolittle
p=1.41e-09
target pH active
active, not at target pH
Density
kyte_doolittle
ARG_12_A - stickiness
p=6.48e-11
target pH active
active, not at target pH
Density
stickiness

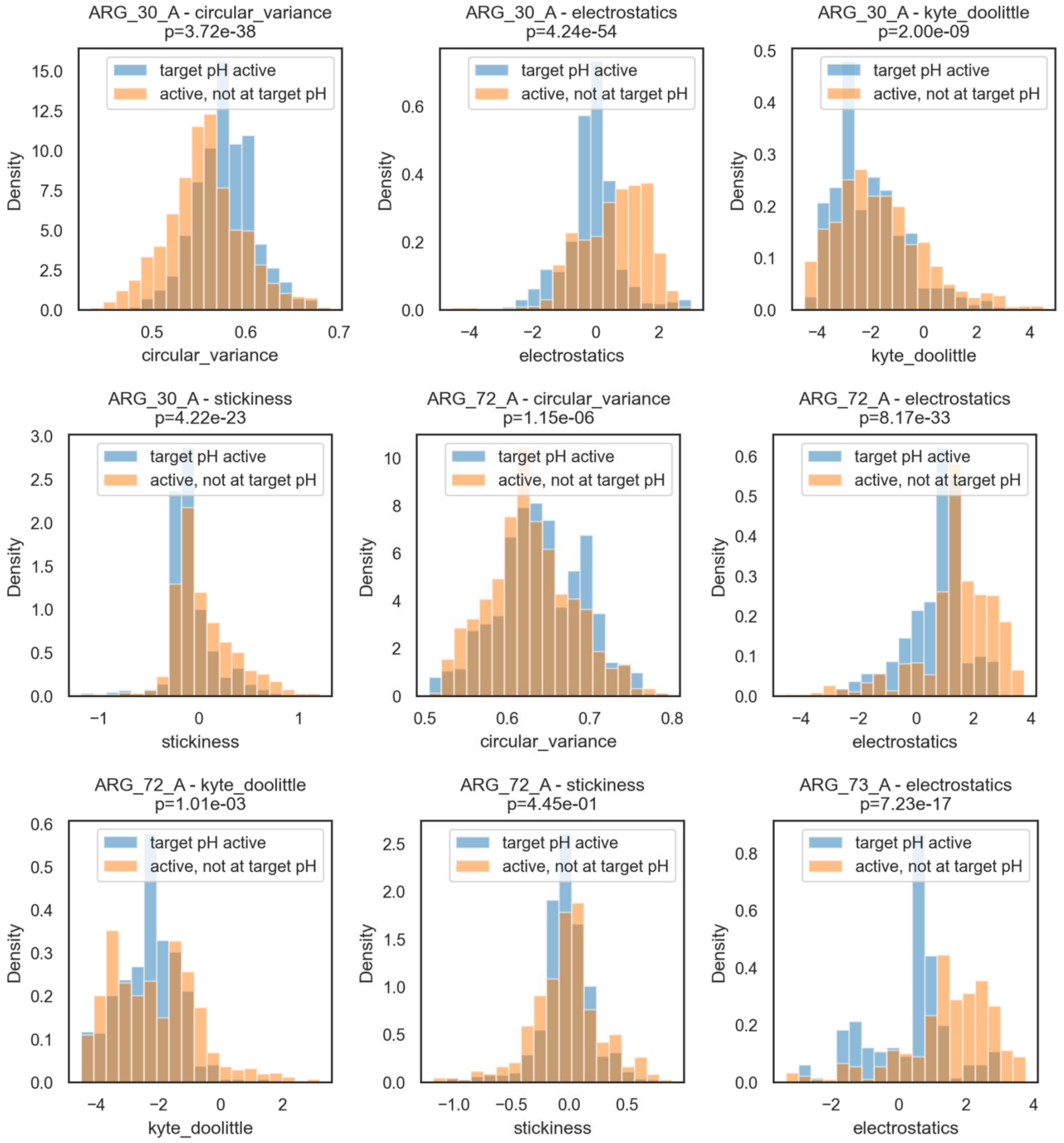

ARG_30_A - circular_variance
p=3.72e-38
target pH active
active, not at target pH
Density
circular_variance
ARG_30_A - electrostatics
p=4.24e-54
target pH active
active, not at target pH
Density
electrostatics
ARG_30_A - kyte_doolittle
p=2.00e-09
target pH active
active, not at target pH
Density
kyte_doolittle
ARG_30_A - stickiness
p=4.22e-23
target pH active
active, not at target pH
Density
stickiness
ARG_72_A - circular_variance
p=1.15e-06
target pH active
active, not at target pH
Density
circular_variance
ARG_72_A - electrostatics
p=8.17e-33
target pH active
active, not at target pH
Density
electrostatics
ARG_72_A - kyte_doolittle
p=1.01e-03
target pH active
active, not at target pH
Density
kyte_doolittle
ARG_72_A - stickiness
p=4.45e-01
target pH active
active, not at target pH
Density
stickiness
ARG_73_A - electrostatics
p=7.23e-17
target pH active
active, not at target pH
Density
electrostatics

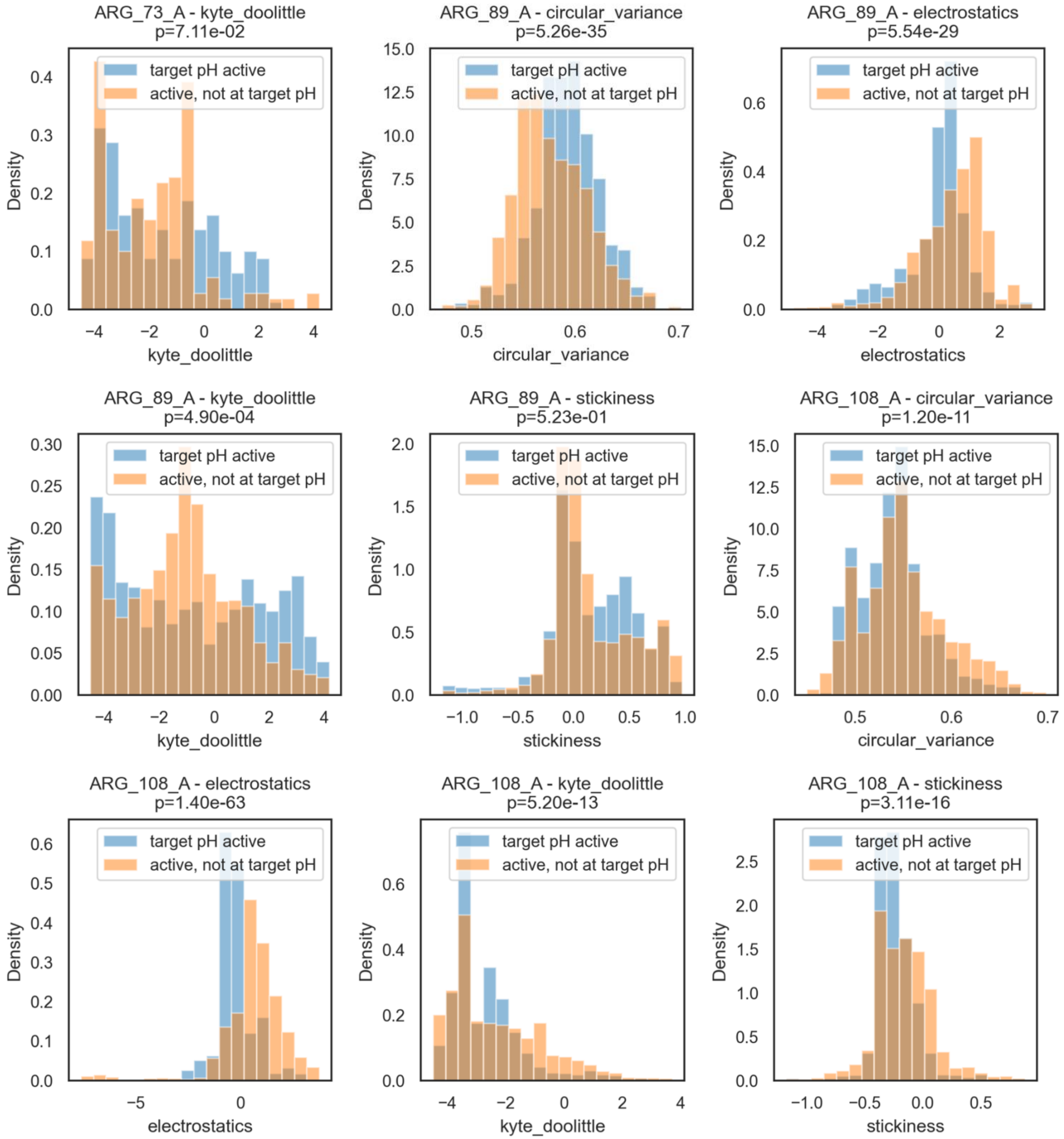
ARG_73_A - kyte_doolittle
p=7.11e-02
target pH active
active, not at target pH
Density
kyte_doolittle
ARG_89_A - circular_variance
p=5.26e-35
target pH active
active, not at target pH
Density
circular_variance
ARG_89_A - electrostatics
p=5.54e-29
target pH active
active, not at target pH
Density
electrostatics
ARG_89_A - kyte_doolittle
p=4.90e-04
target pH active
active, not at target pH
Density
kyte_doolittle
ARG_89_A - stickiness
p=5.23e-01
target pH active
active, not at target pH
Density
stickiness
ARG_108_A - circular_variance
p=1.20e-11
target pH active
active, not at target pH
Density
circular_variance
ARG_108_A - electrostatics
p=1.40e-63
target pH active
active, not at target pH
Density
electrostatics
ARG_108_A - kyte_doolittle
p=5.20e-13
target pH active
active, not at target pH
Density
kyte_doolittle
ARG_108_A - stickiness
p=3.11e-16
target pH active
active, not at target pH
Density
stickiness

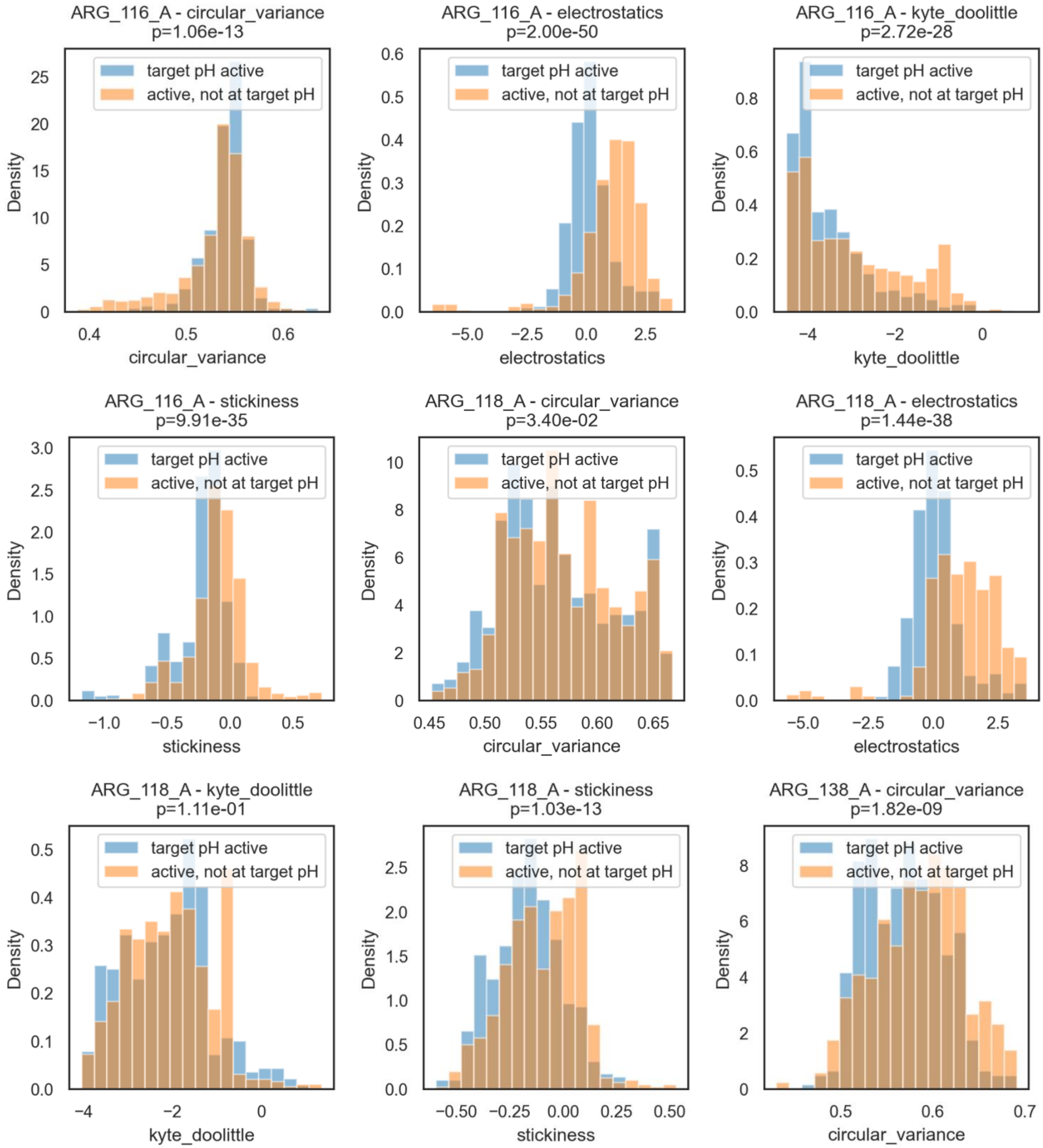
ARG_116_A - circular_variance
p=1.06e-13
target pH active
active, not at target pH
Density
circular_variance
ARG_116_A - electrostatics
p=2.00e-50
electrostatics
ARG_116_A - kyte_doolittle
p=2.72e-28
kyte_doolittle
ARG_116_A - stickiness
p=9.91e-35
stickiness
ARG_118_A - circular_variance
p=3.40e-02
ARG_118_A - electrostatics
p=1.44e-38
ARG_118_A - kyte_doolittle
p=1.11e-01
ARG_118_A - stickiness
p=1.03e-13
ARG_138_A - circular_variance
p=1.82e-09

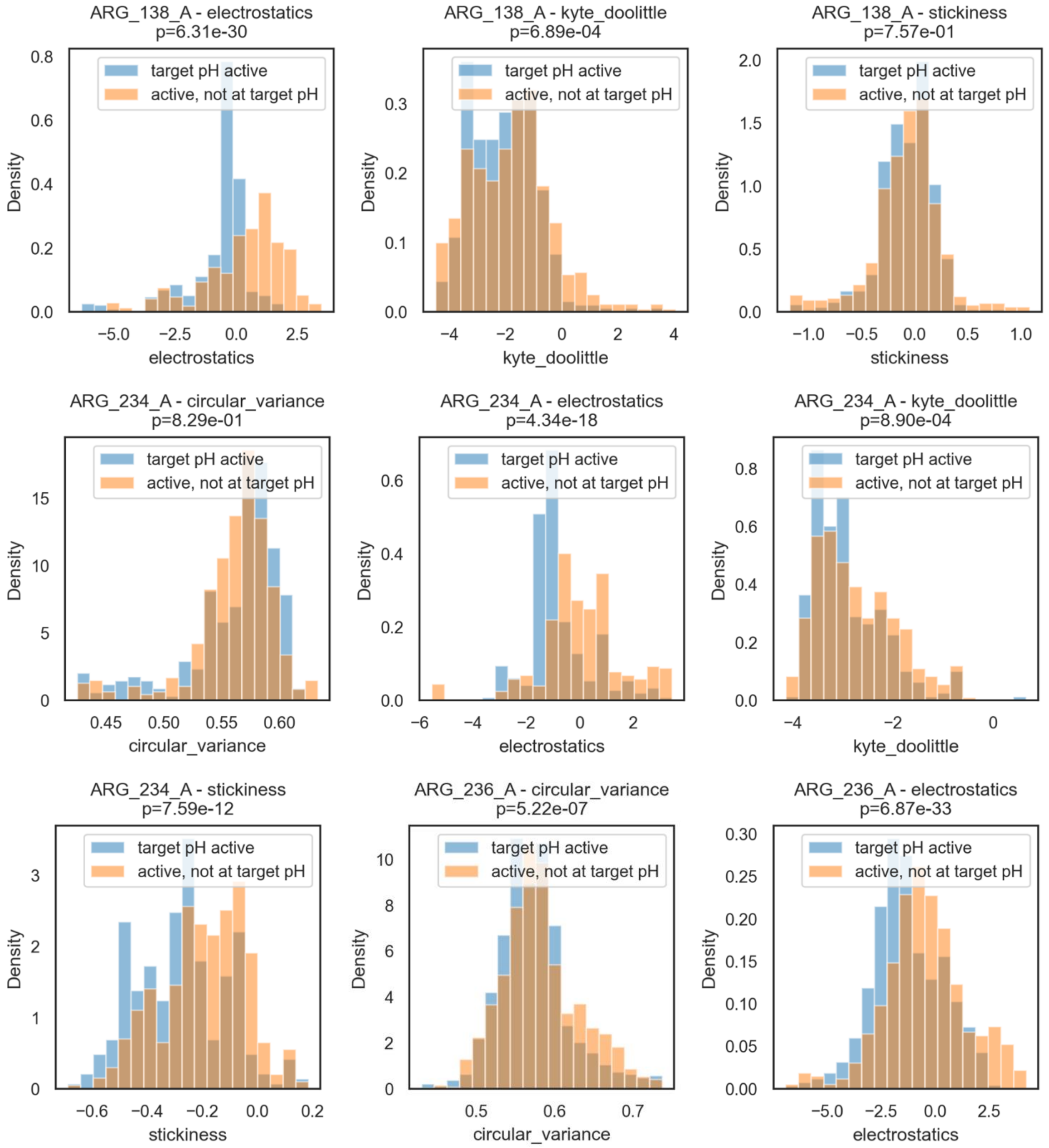
ARG_138_A - electrostatics
p=6.31e-30
target pH active
active, not at target pH
Density
electrostatics
ARG_138_A - kyte_doolittle
p=6.89e-04
target pH active
active, not at target pH
Density
kyte_doolittle
ARG_138_A - stickiness
p=7.57e-01
target pH active
active, not at target pH
Density
stickiness
ARG_234_A - circular_variance
p=8.29e-01
target pH active
active, not at target pH
Density
circular_variance
ARG_234_A - electrostatics
p=4.34e-18
target pH active
active, not at target pH
Density
electrostatics
ARG_234_A - kyte_doolittle
p=8.90e-04
target pH active
active, not at target pH
Density
kyte_doolittle
ARG_234_A - stickiness
p=7.59e-12
target pH active
active, not at target pH
Density
stickiness
ARG_236_A - circular_variance
p=5.22e-07
target pH active
active, not at target pH
Density
circular_variance
ARG_236_A - electrostatics
p=6.87e-33
target pH active
active, not at target pH
Density
electrostatics

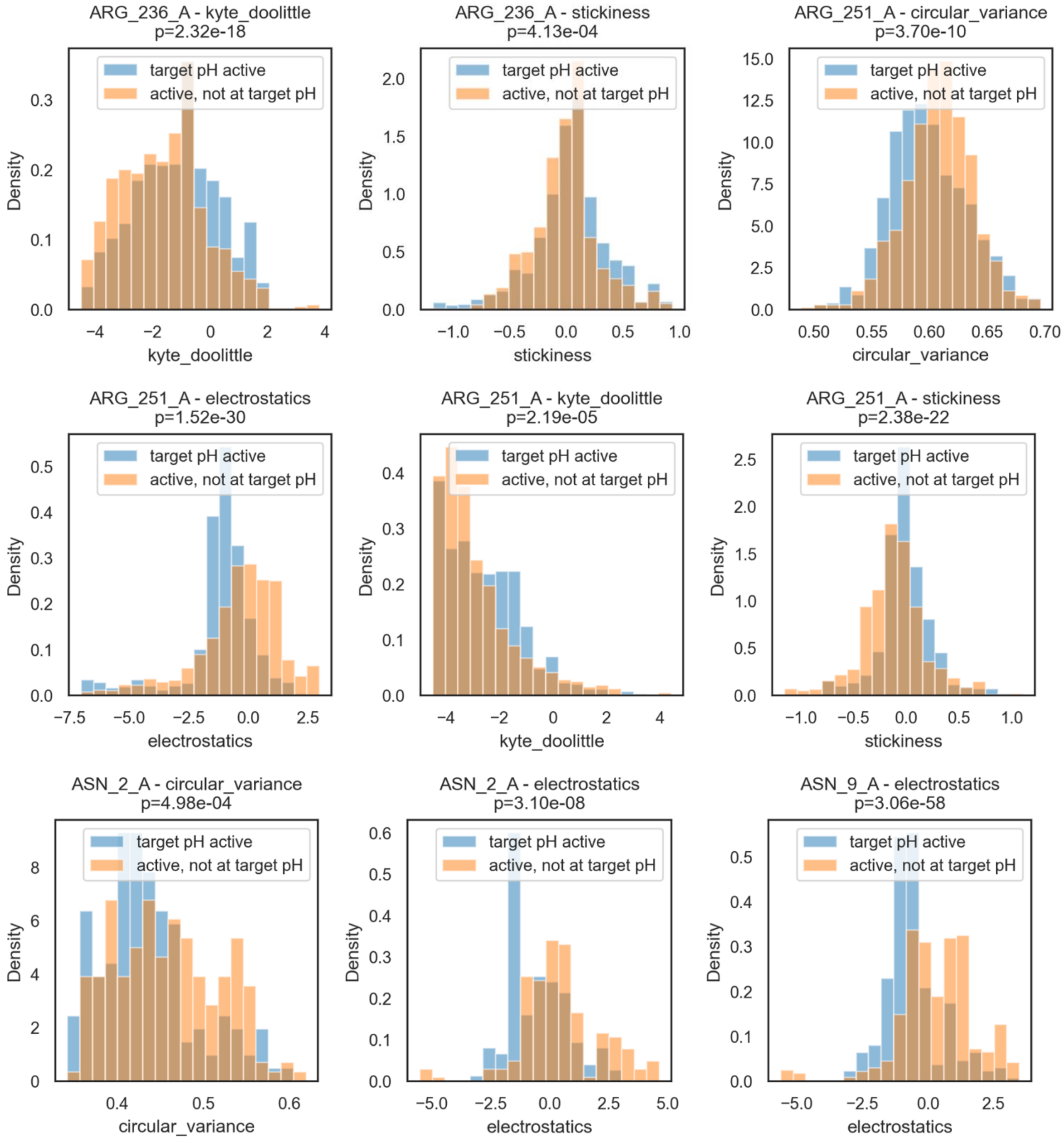
ARG_236_A - kyte_doolittle
p=2.32e-18
target pH active
active, not at target pH
Density
kyte_doolittle
ARG_236_A - stickiness
p=4.13e-04
target pH active
active, not at target pH
Density
stickiness
ARG_251_A - circular_variance
p=3.70e-10
target pH active
active, not at target pH
Density
circular_variance
ARG_251_A - electrostatics
p=1.52e-30
target pH active
active, not at target pH
Density
electrostatics
ARG_251_A - kyte_doolittle
p=2.19e-05
target pH active
active, not at target pH
Density
kyte_doolittle
ARG_251_A - stickiness
p=2.38e-22
target pH active
active, not at target pH
Density
stickiness
ASN_2_A - circular_variance
p=4.98e-04
target pH active
active, not at target pH
Density
circular_variance
ASN_2_A - electrostatics
p=3.10e-08
target pH active
active, not at target pH
Density
electrostatics
ASN_9_A - electrostatics
p=3.06e-58
target pH active
active, not at target pH
Density
electrostatics

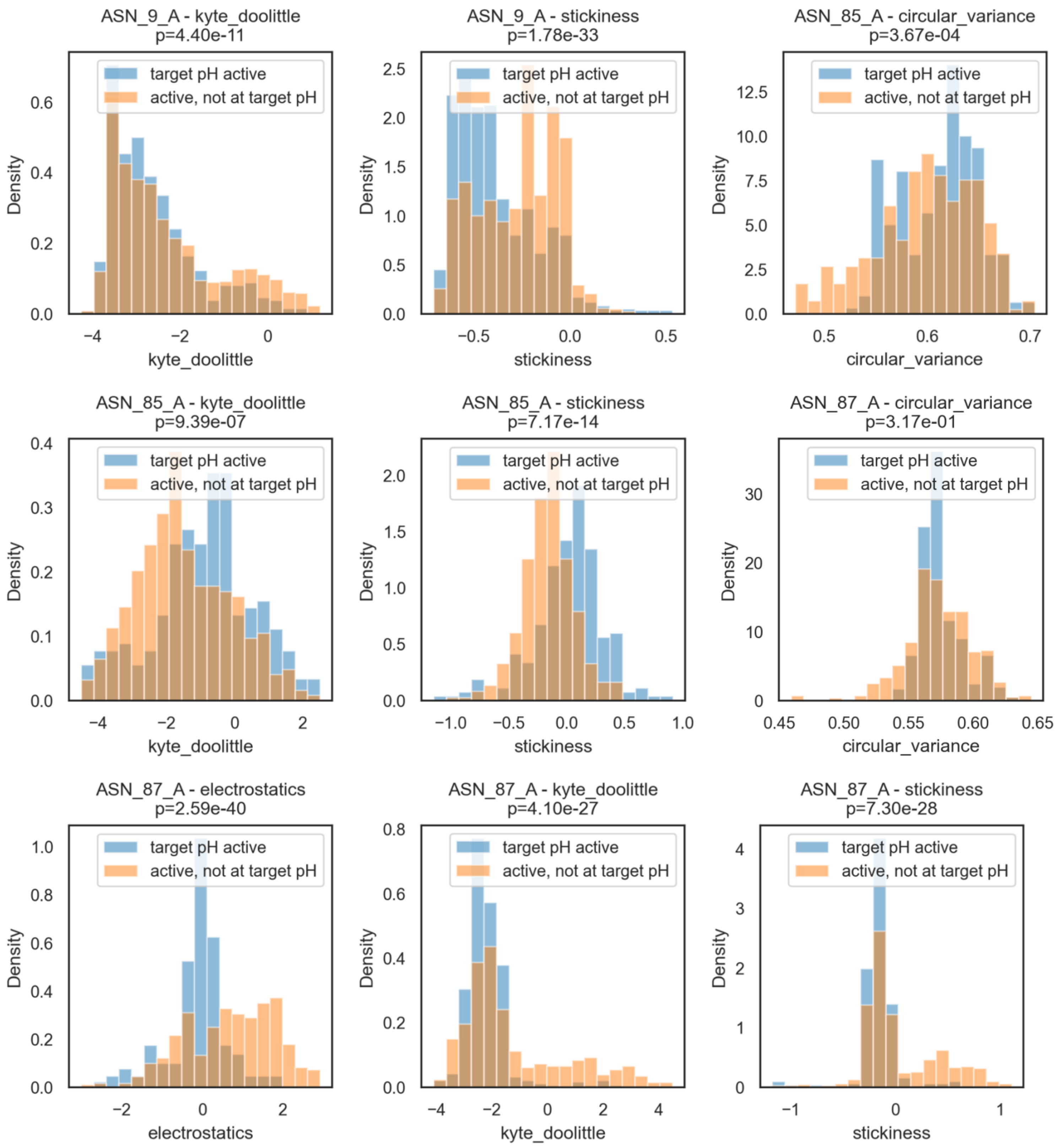
ASN_9_A - kyte_doolittle
p=4.40e-11
target pH active
active, not at target pH
Density
kyte_doolittle
ASN_9_A - stickiness
p=1.78e-33
target pH active
active, not at target pH
Density
stickiness
ASN_85_A - circular_variance
p=3.67e-04
target pH active
active, not at target pH
Density
circular_variance
ASN_85_A - kyte_doolittle
p=9.39e-07
target pH active
active, not at target pH
Density
kyte_doolittle
ASN_85_A - stickiness
p=7.17e-14
target pH active
active, not at target pH
Density
stickiness
ASN_87_A - circular_variance
p=3.17e-01
target pH active
active, not at target pH
Density
circular_variance
ASN_87_A - electrostatics
p=2.59e-40
target pH active
active, not at target pH
Density
electrostatics
ASN_87_A - kyte_doolittle
p=4.10e-27
target pH active
active, not at target pH
Density
kyte_doolittle
ASN_87_A - stickiness
p=7.30e-28
target pH active
active, not at target pH
Density
stickiness

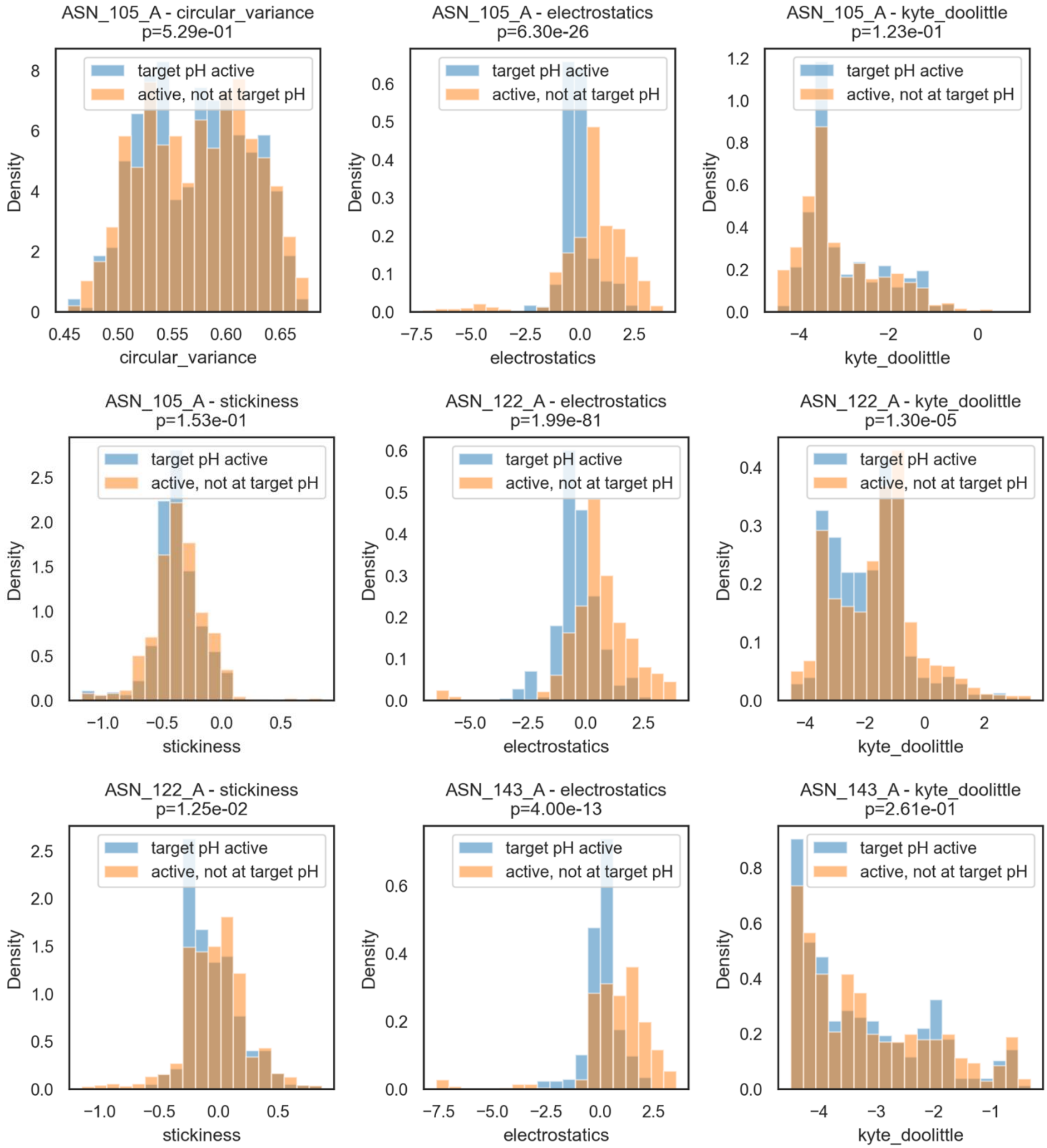
ASN_105_A - circular_variance
p=5.29e-01
target pH active
active, not at target pH
Density
circular_variance
ASN_105_A - electrostatics
p=6.30e-26
target pH active
active, not at target pH
Density
electrostatics
ASN_105_A - kyte_doolittle
p=1.23e-01
target pH active
active, not at target pH
Density
kyte_doolittle
ASN_105_A - stickiness
p=1.53e-01
target pH active
active, not at target pH
Density
stickiness
ASN_122_A - electrostatics
p=1.99e-81
target pH active
active, not at target pH
Density
electrostatics
ASN_122_A - kyte_doolittle
p=1.30e-05
target pH active
active, not at target pH
Density
kyte_doolittle
ASN_122_A - stickiness
p=1.25e-02
target pH active
active, not at target pH
Density
stickiness
ASN_143_A - electrostatics
p=4.00e-13
target pH active
active, not at target pH
Density
electrostatics
ASN_143_A - kyte_doolittle
p=2.61e-01
target pH active
active, not at target pH
Density
kyte_doolittle

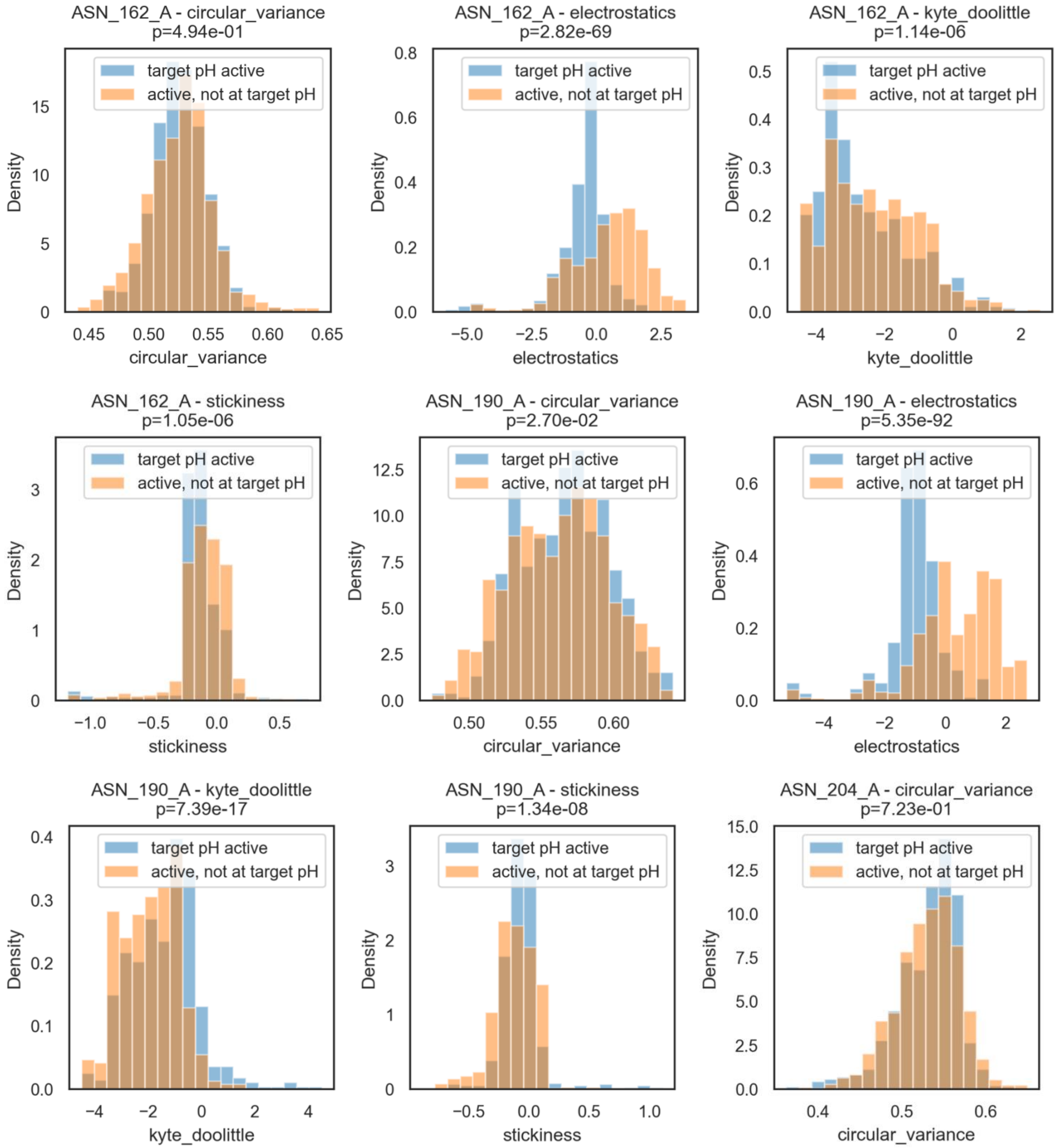
ASN_162_A - circular_variance
p=4.94e-01
target pH active
active, not at target pH
Density
circular_variance
ASN_162_A - electrostatics
p=2.82e-69
target pH active
active, not at target pH
Density
electrostatics
ASN_162_A - kyte_doolittle
p=1.14e-06
target pH active
active, not at target pH
Density
kyte_doolittle
ASN_162_A - stickiness
p=1.05e-06
target pH active
active, not at target pH
Density
stickiness
ASN_190_A - circular_variance
p=2.70e-02
target pH active
active, not at target pH
Density
circular_variance
ASN_190_A - electrostatics
p=5.35e-92
target pH active
active, not at target pH
Density
electrostatics
ASN_190_A - kyte_doolittle
p=7.39e-17
target pH active
active, not at target pH
Density
kyte_doolittle
ASN_190_A - stickiness
p=1.34e-08
target pH active
active, not at target pH
Density
stickiness
ASN_204_A - circular_variance
p=7.23e-01
target pH active
active, not at target pH
Density
circular_variance

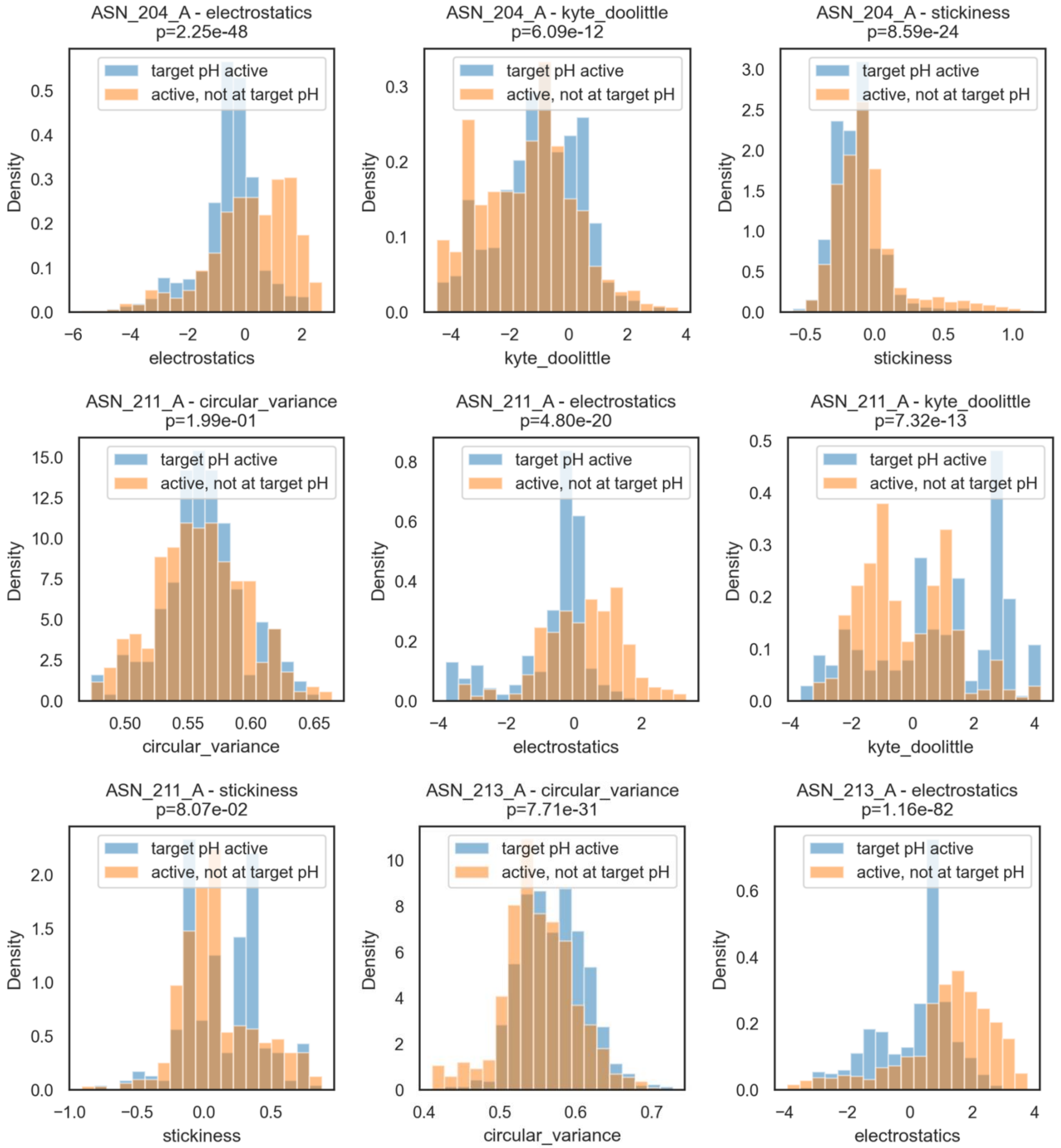
ASN_204_A - electrostatics
p=2.25e-48
ASN_204_A - kyte_doolittle
p=6.09e-12
ASN_204_A - stickiness
p=8.59e-24
ASN_211_A - circular_variance
p=1.99e-01
ASN_211_A - electrostatics
p=4.80e-20
ASN_211_A - kyte_doolittle
p=7.32e-13
ASN_211_A - stickiness
p=8.07e-02
ASN_213_A - circular_variance
p=7.71e-31
ASN_213_A - electrostatics
p=1.16e-82
target pH active
active, not at target pH
Density
electrostatics
kyte_doolittle
stickiness
circular_variance

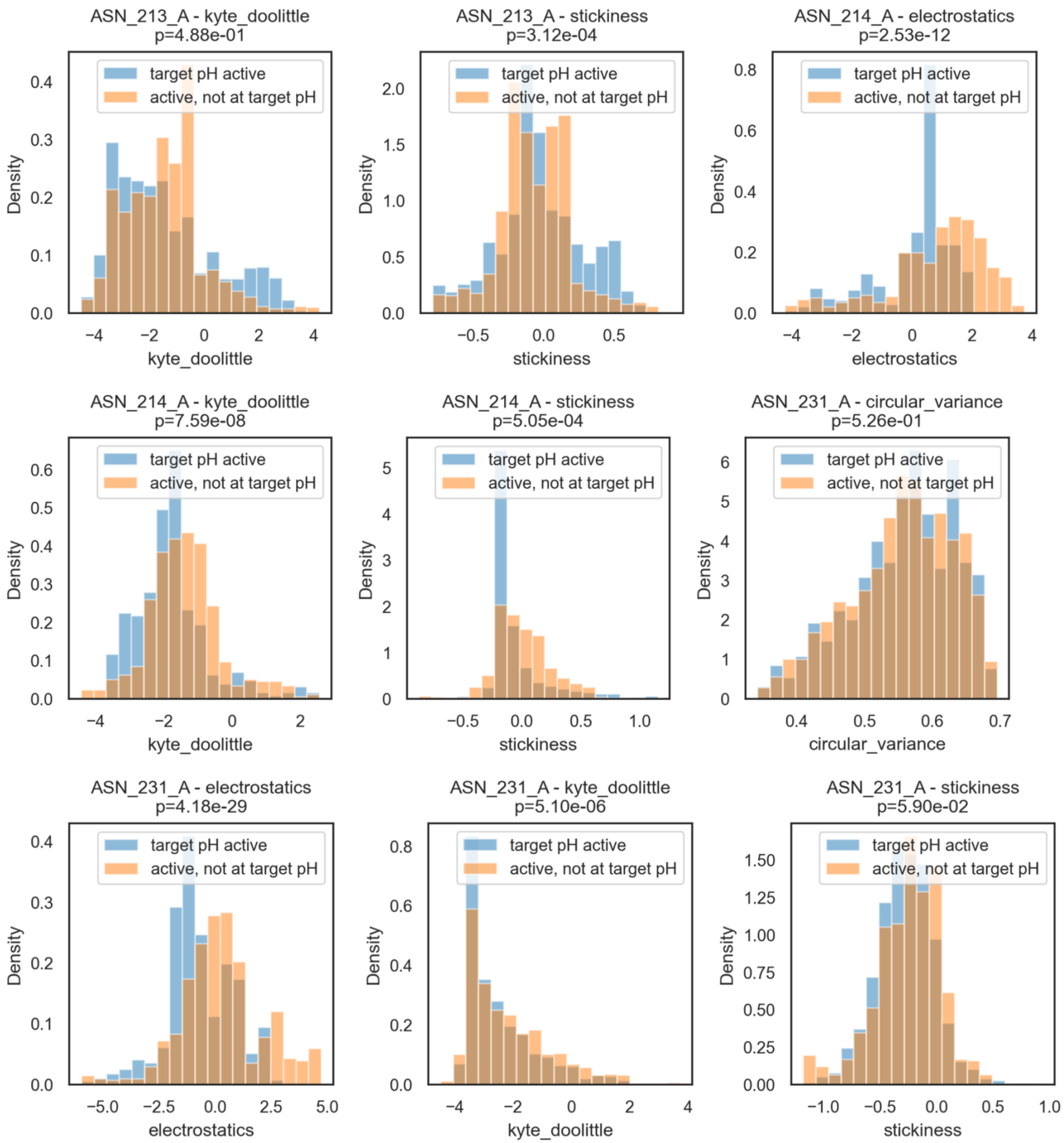
ASN_213_A - kyte_doolittle
p=4.88e-01
target pH active
active, not at target pH
Density
kyte_doolittle
ASN_213_A - stickiness
p=3.12e-04
target pH active
active, not at target pH
Density
stickiness
ASN_214_A - electrostatics
p=2.53e-12
target pH active
active, not at target pH
Density
electrostatics
ASN_214_A - kyte_doolittle
p=7.59e-08
target pH active
active, not at target pH
Density
kyte_doolittle
ASN_214_A - stickiness
p=5.05e-04
target pH active
active, not at target pH
Density
stickiness
ASN_231_A - circular_variance
p=5.26e-01
target pH active
active, not at target pH
Density
circular_variance
ASN_231_A - electrostatics
p=4.18e-29
target pH active
active, not at target pH
Density
electrostatics
ASN_231_A - kyte_doolittle
p=5.10e-06
target pH active
active, not at target pH
Density
kyte_doolittle
ASN_231_A - stickiness
p=5.90e-02
target pH active
active, not at target pH
Density
stickiness

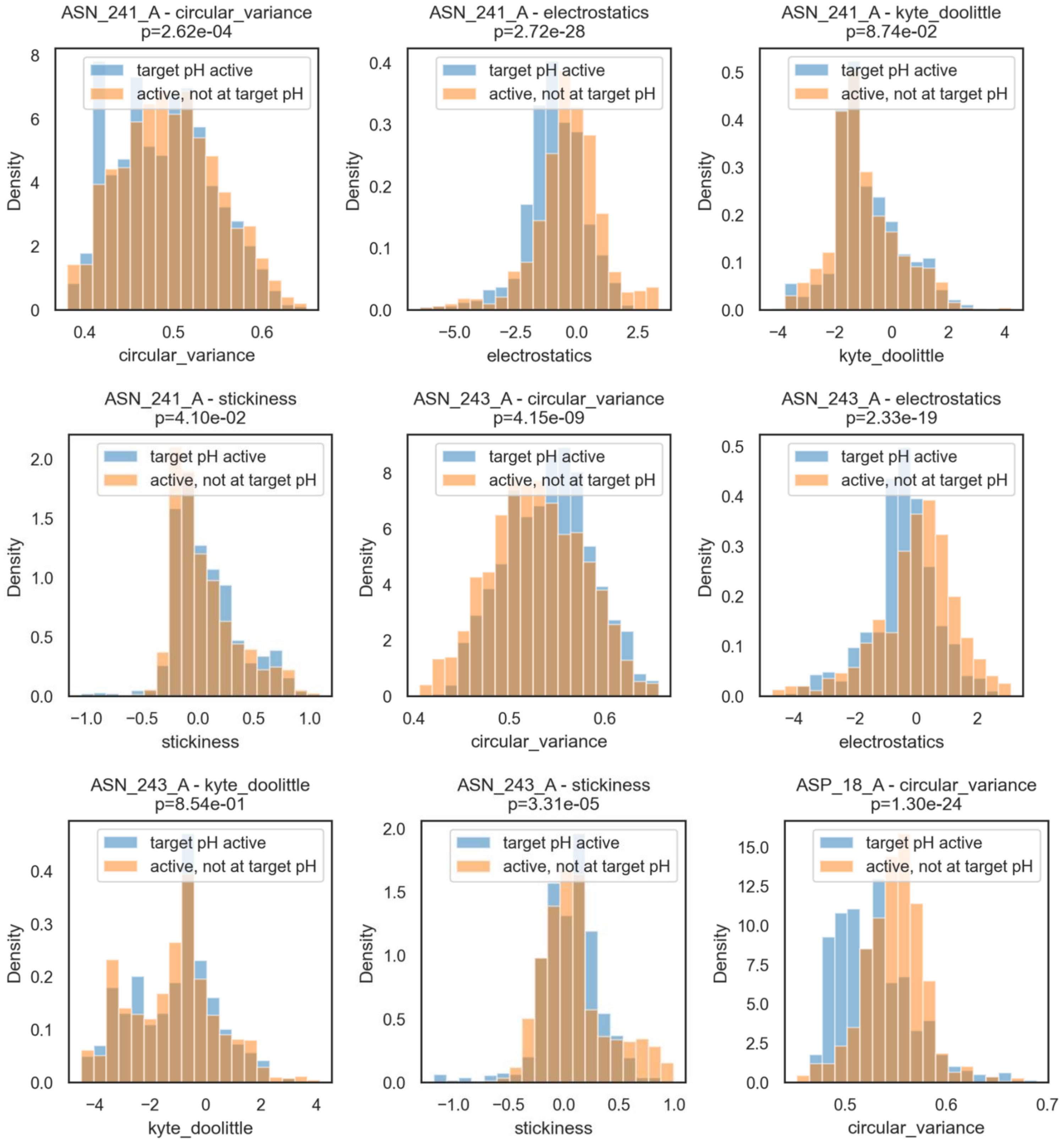
ASN_241_A - circular_variance
p=2.62e-04
target pH active
active, not at target pH
Density
circular_variance
ASN_241_A - electrostatics
p=2.72e-28
target pH active
active, not at target pH
Density
electrostatics
ASN_241_A - kyte_doolittle
p=8.74e-02
target pH active
active, not at target pH
Density
kyte_doolittle
ASN_241_A - stickiness
p=4.10e-02
target pH active
active, not at target pH
Density
stickiness
ASN_243_A - circular_variance
p=4.15e-09
target pH active
active, not at target pH
Density
circular_variance
ASN_243_A - electrostatics
p=2.33e-19
target pH active
active, not at target pH
Density
electrostatics
ASN_243_A - kyte_doolittle
p=8.54e-01
target pH active
active, not at target pH
Density
kyte_doolittle
ASN_243_A - stickiness
p=3.31e-05
target pH active
active, not at target pH
Density
stickiness
ASP_18_A - circular_variance
p=1.30e-24
target pH active
active, not at target pH
Density
circular_variance

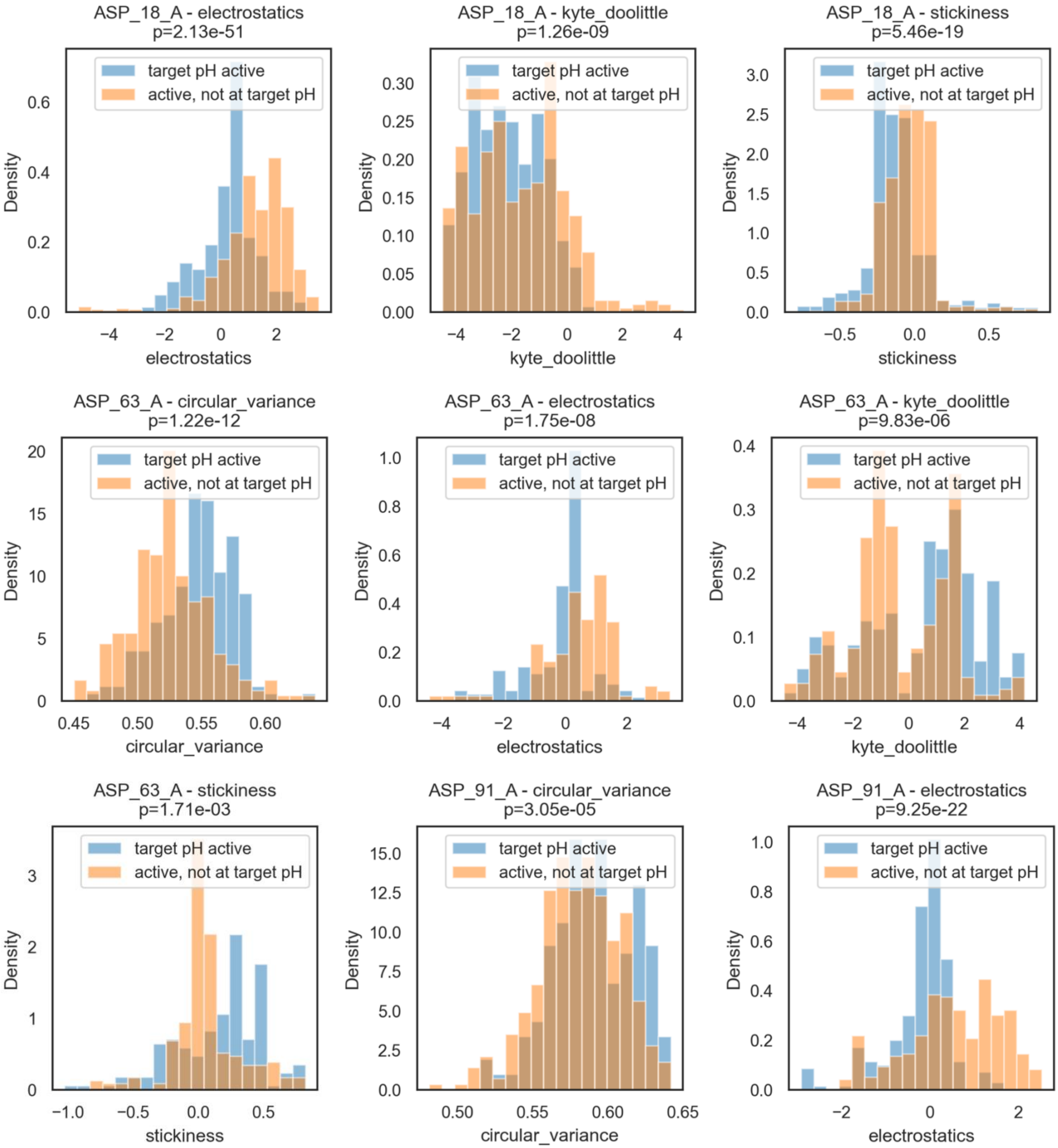
ASP_18_A - electrostatics
p=2.13e-51
ASP_18_A - kyte_doolittle
p=1.26e-09
ASP_18_A - stickiness
p=5.46e-19
ASP_63_A - circular_variance
p=1.22e-12
ASP_63_A - electrostatics
p=1.75e-08
ASP_63_A - kyte_doolittle
p=9.83e-06
ASP_63_A - stickiness
p=1.71e-03
ASP_91_A - circular_variance
p=3.05e-05
ASP_91_A - electrostatics
p=9.25e-22
target pH active
active, not at target pH
Density
electrostatics
kyte_doolittle
stickiness
circular_variance

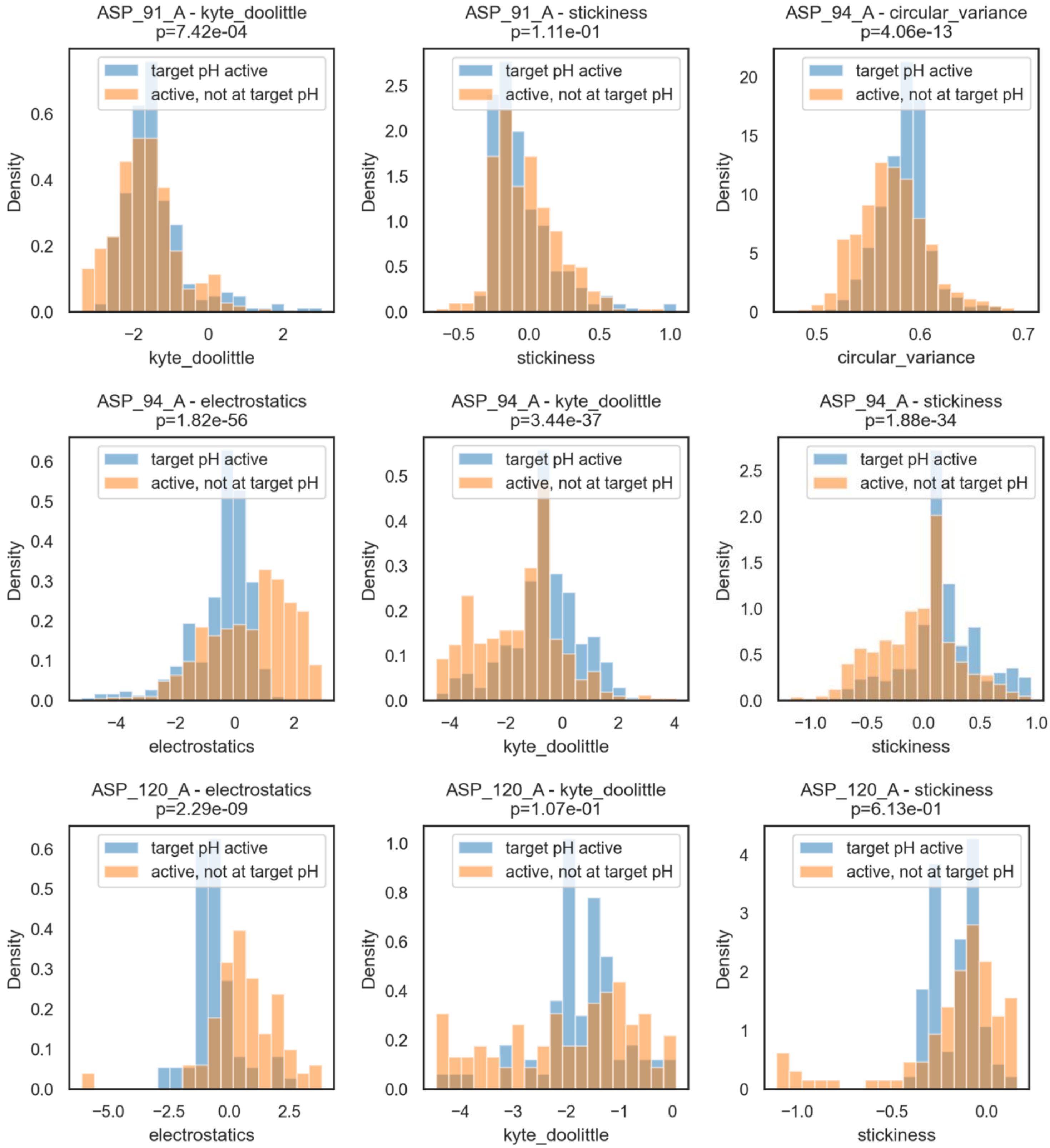
ASP_91_A - kyte_doolittle
p=7.42e-04
target pH active
active, not at target pH
Density
kyte_doolittle
ASP_91_A - stickiness
p=1.11e-01
target pH active
active, not at target pH
Density
stickiness
ASP_94_A - circular_variance
p=4.06e-13
target pH active
active, not at target pH
Density
circular_variance
ASP_94_A - electrostatics
p=1.82e-56
target pH active
active, not at target pH
Density
electrostatics
ASP_94_A - kyte_doolittle
p=3.44e-37
target pH active
active, not at target pH
Density
kyte_doolittle
ASP_94_A - stickiness
p=1.88e-34
target pH active
active, not at target pH
Density
stickiness
ASP_120_A - electrostatics
p=2.29e-09
target pH active
active, not at target pH
Density
electrostatics
ASP_120_A - kyte_doolittle
p=1.07e-01
target pH active
active, not at target pH
Density
kyte_doolittle
ASP_120_A - stickiness
p=6.13e-01
target pH active
active, not at target pH
Density
stickiness

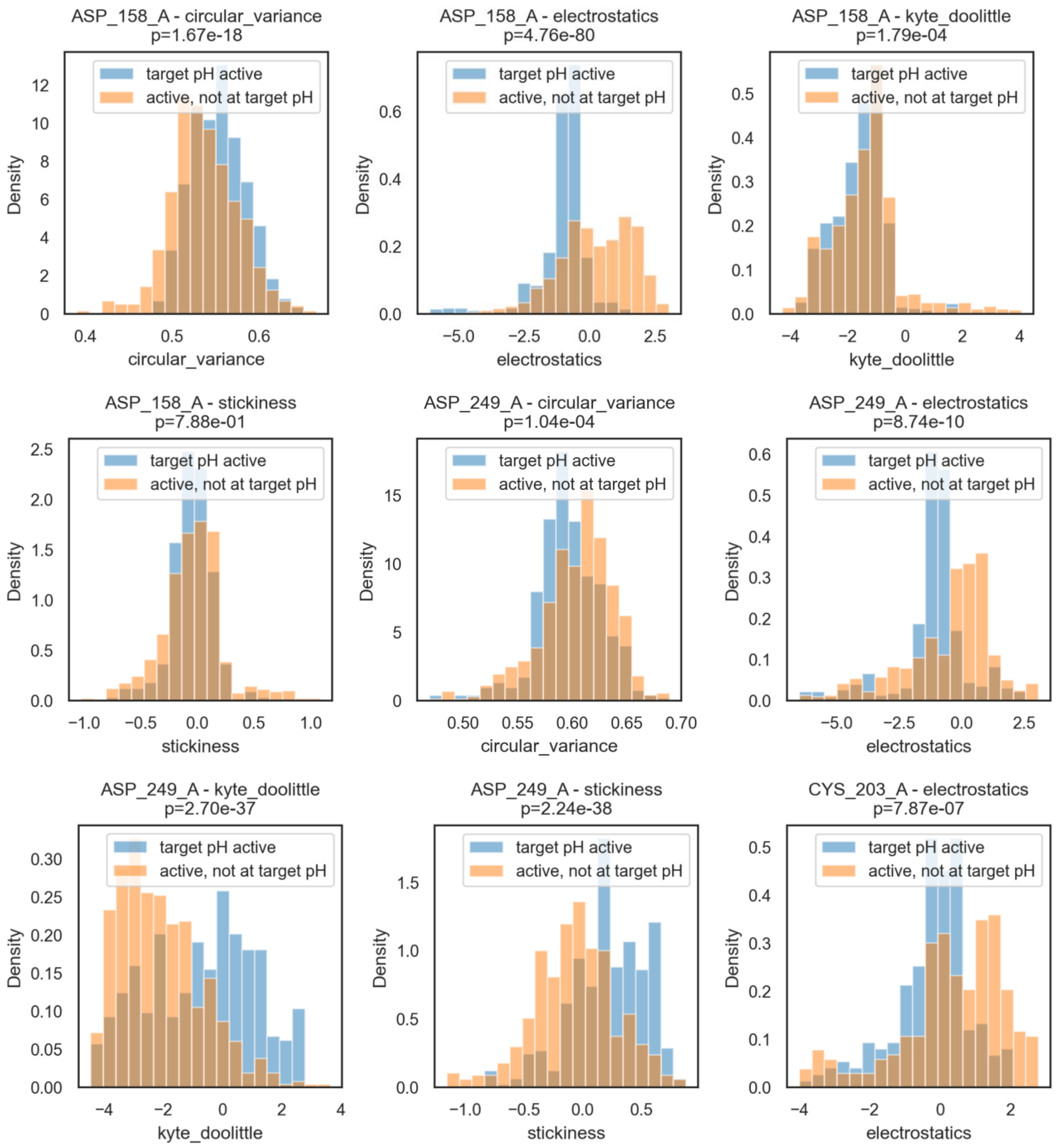
ASP_158_A - circular_variance
p=1.67e-18
target pH active
active, not at target pH
Density
circular_variance
ASP_158_A - electrostatics
p=4.76e-80
target pH active
active, not at target pH
Density
electrostatics
ASP_158_A - kyte_doolittle
p=1.79e-04
target pH active
active, not at target pH
Density
kyte_doolittle
ASP_158_A - stickiness
p=7.88e-01
target pH active
active, not at target pH
Density
stickiness
ASP_249_A - circular_variance
p=1.04e-04
target pH active
active, not at target pH
Density
circular_variance
ASP_249_A - electrostatics
p=8.74e-10
target pH active
active, not at target pH
Density
electrostatics
ASP_249_A - kyte_doolittle
p=2.70e-37
target pH active
active, not at target pH
Density
kyte_doolittle
ASP_249_A - stickiness
p=2.24e-38
target pH active
active, not at target pH
Density
stickiness
CYS_203_A - electrostatics
p=7.87e-07
target pH active
active, not at target pH
Density
electrostatics

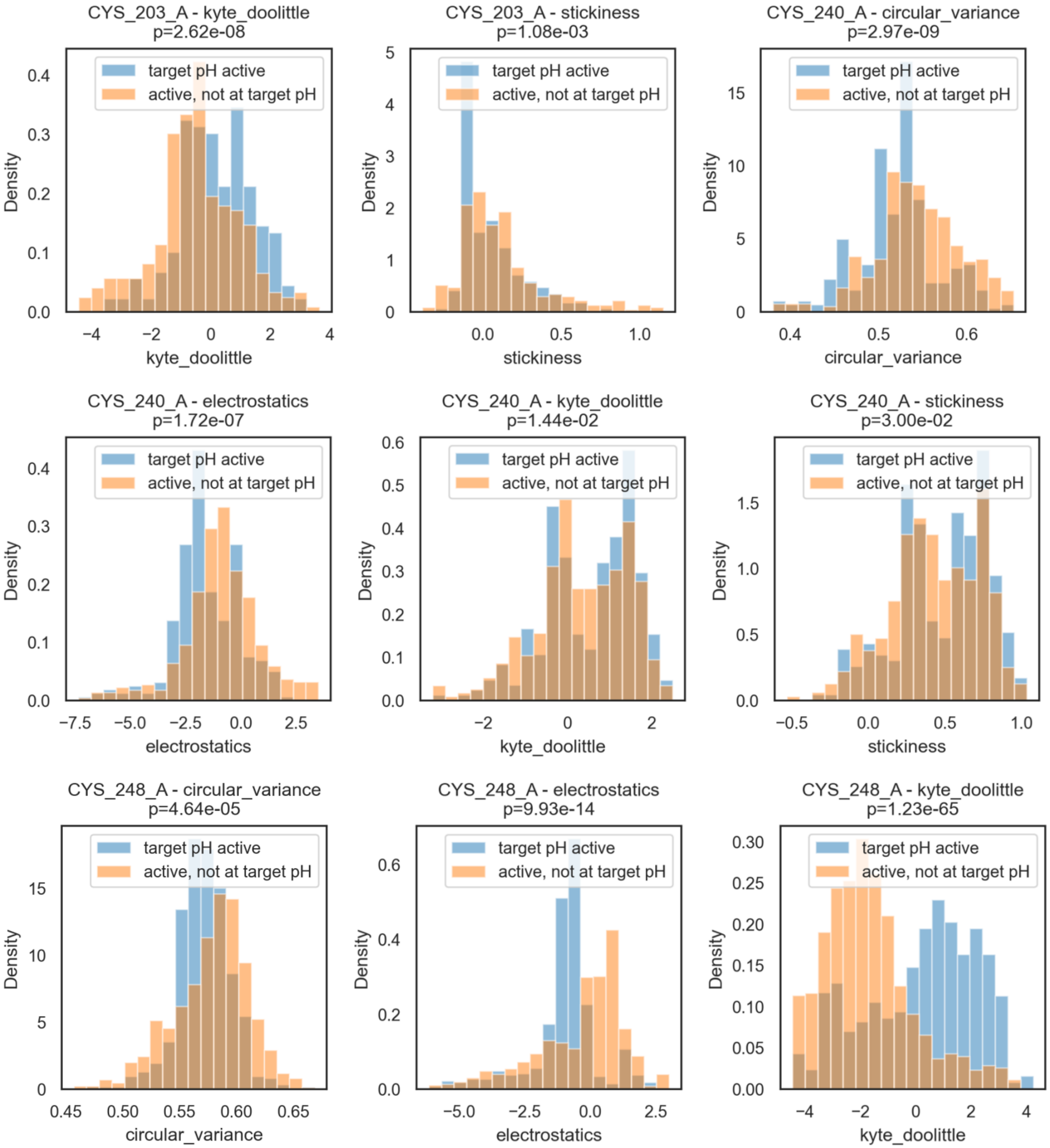
CYS_203_A - kyte_doolittle
p=2.62e-08
CYS_203_A - stickiness
p=1.08e-03
CYS_240_A - circular_variance
p=2.97e-09
CYS_240_A - electrostatics
p=1.72e-07
CYS_240_A - kyte_doolittle
p=1.44e-02
CYS_240_A - stickiness
p=3.00e-02
CYS_248_A - circular_variance
p=4.64e-05
CYS_248_A - electrostatics
p=9.93e-14
CYS_248_A - kyte_doolittle
p=1.23e-65
target pH active
active, not at target pH
Density
kyte_doolittle
stickiness
circular_variance
electrostatics

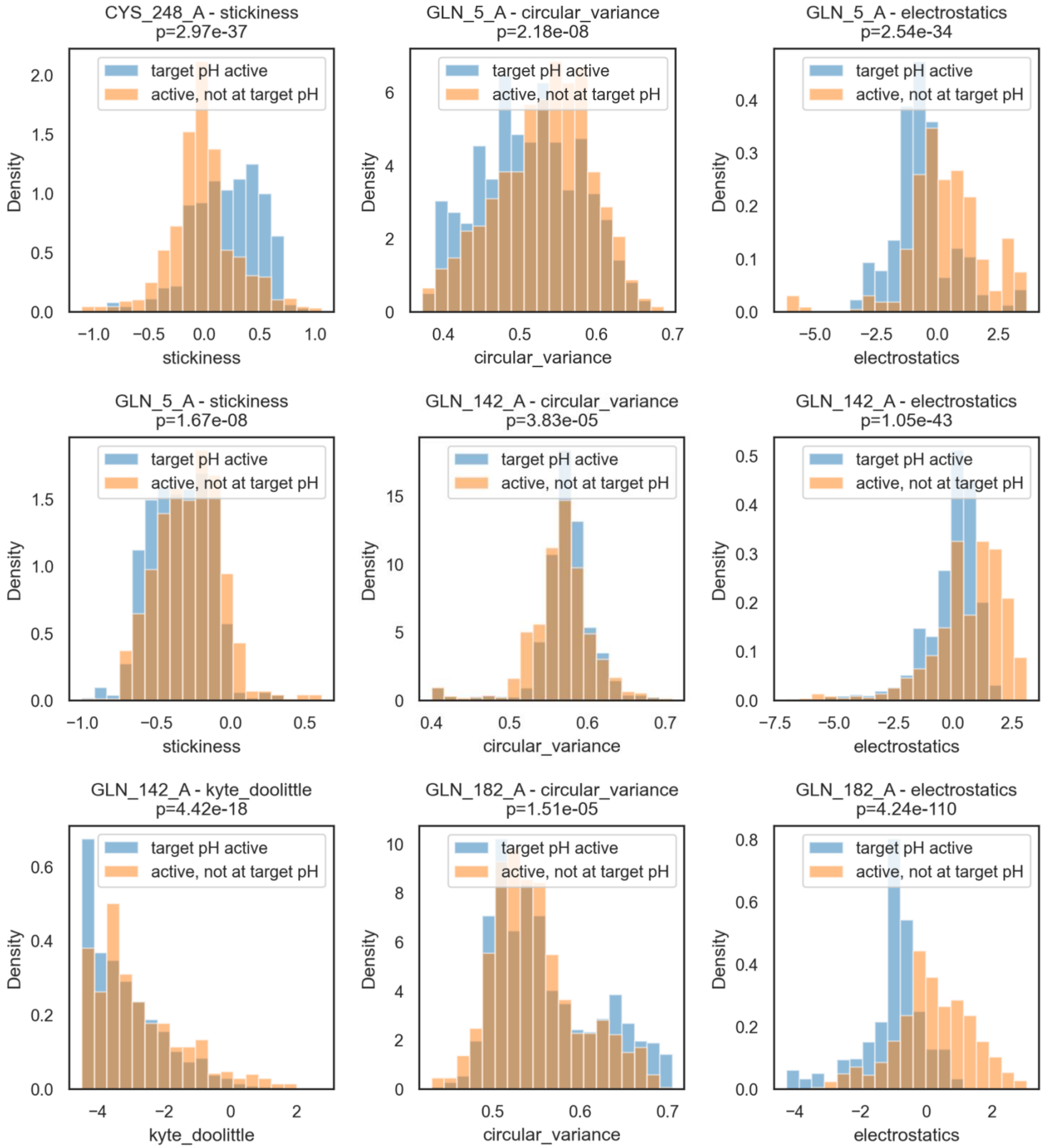
CYS_248_A - stickiness
p=2.97e-37
GLN_5_A - circular_variance
p=2.18e-08
GLN_5_A - electrostatics
p=2.54e-34
GLN_5_A - stickiness
p=1.67e-08
GLN_142_A - circular_variance
p=3.83e-05
GLN_142_A - electrostatics
p=1.05e-43
GLN_142_A - kyte_doolittle
p=4.42e-18
GLN_182_A - circular_variance
p=1.51e-05
GLN_182_A - electrostatics
p=4.24e-110
target pH active
active, not at target pH
Density
stickiness
circular_variance
electrostatics
kyte_doolittle

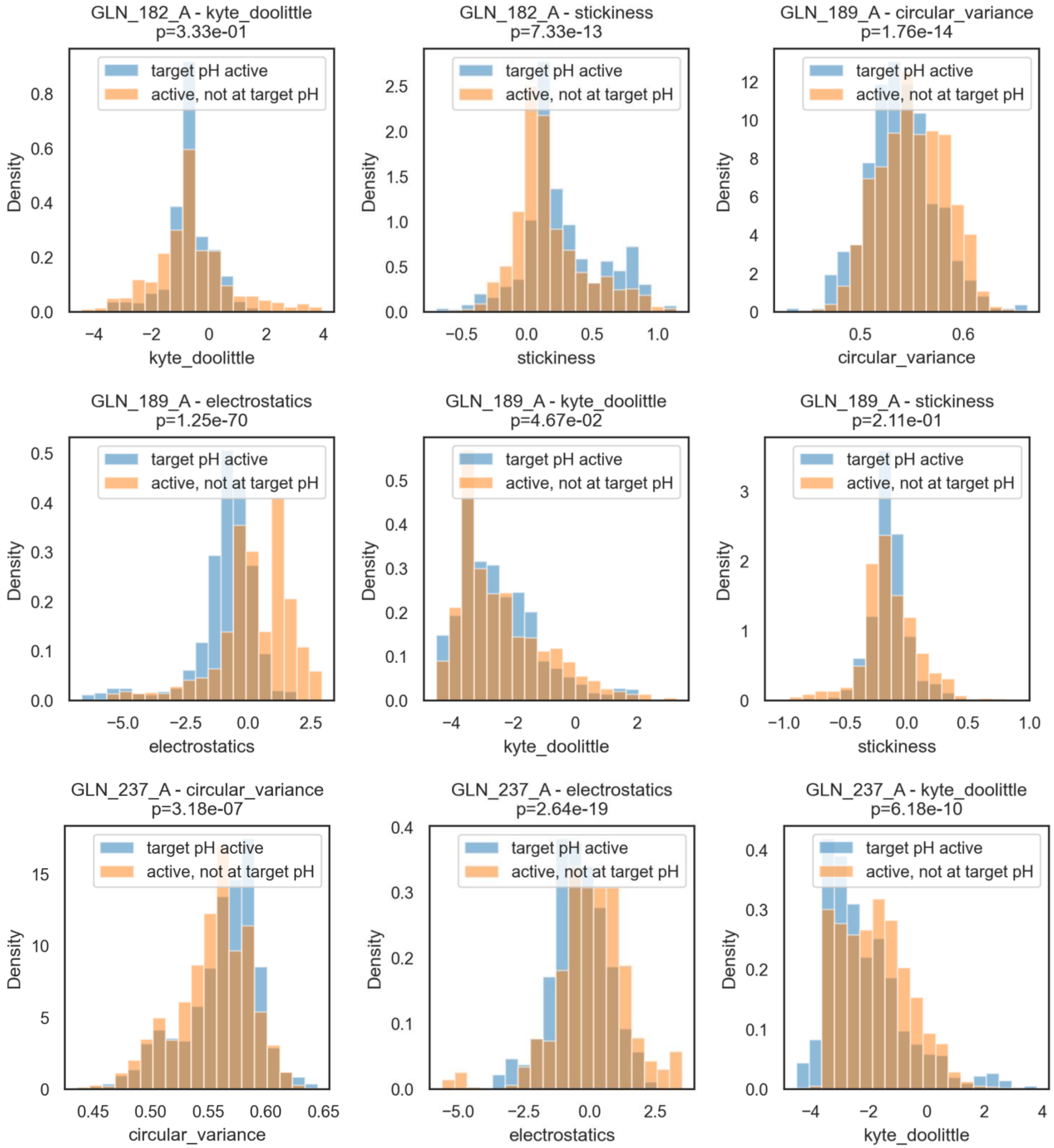
GLN_182_A - kyte_doolittle
p=3.33e-01
GLN_182_A - stickiness
p=7.33e-13
GLN_189_A - circular_variance
p=1.76e-14
GLN_189_A - electrostatics
p=1.25e-70
GLN_189_A - kyte_doolittle
p=4.67e-02
GLN_189_A - stickiness
p=2.11e-01
GLN_237_A - circular_variance
p=3.18e-07
GLN_237_A - electrostatics
p=2.64e-19
GLN_237_A - kyte_doolittle
p=6.18e-10
target pH active
active, not at target pH
Density
kyte_doolittle
stickiness
circular_variance
electrostatics

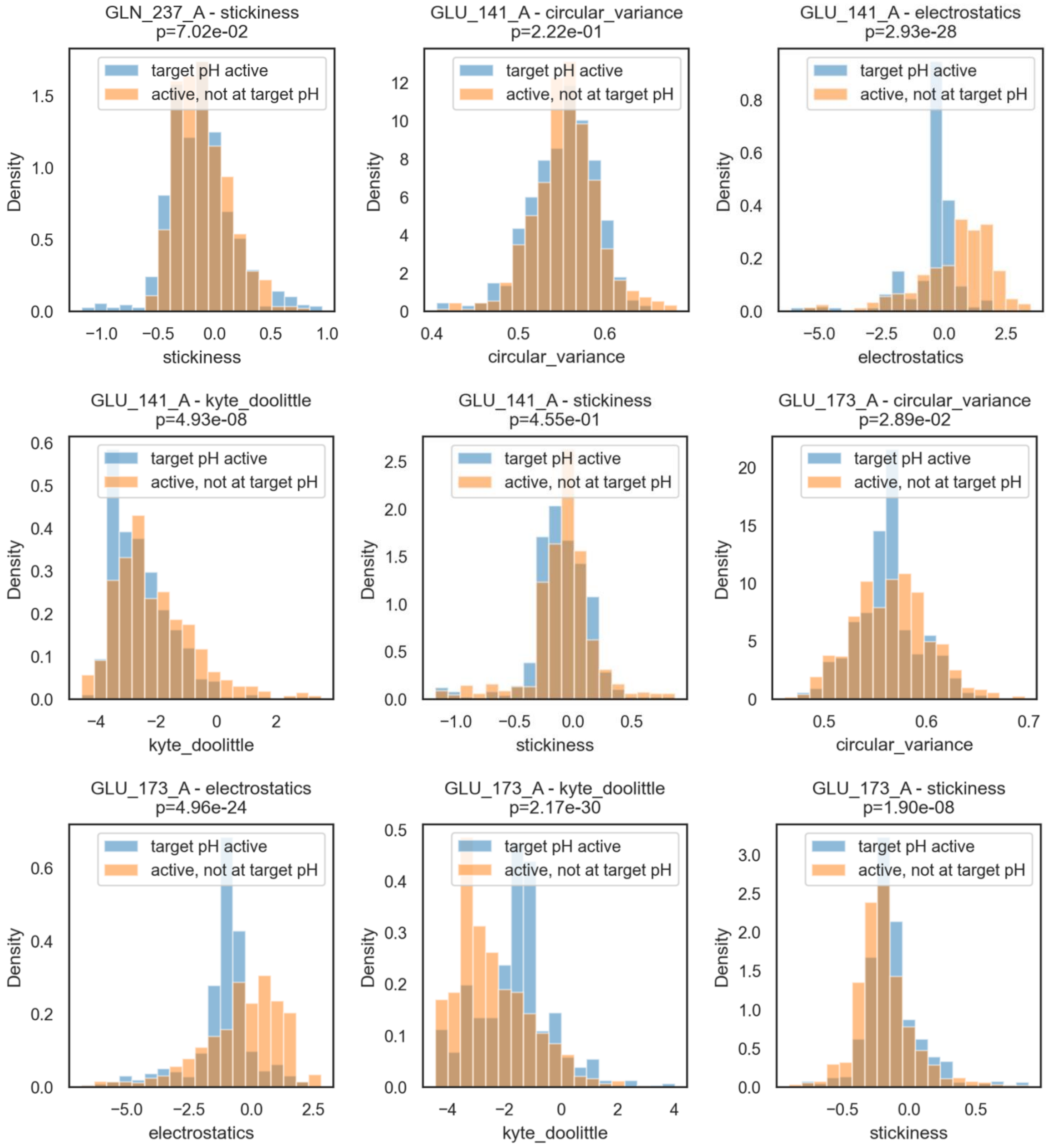
GLN_237_A - stickiness
p=7.02e-02
target pH active
active, not at target pH
Density
stickiness
GLU_141_A - circular_variance
p=2.22e-01
target pH active
active, not at target pH
Density
circular_variance
GLU_141_A - electrostatics
p=2.93e-28
target pH active
active, not at target pH
Density
electrostatics
GLU_141_A - kyte_doolittle
p=4.93e-08
target pH active
active, not at target pH
Density
kyte_doolittle
GLU_141_A - stickiness
p=4.55e-01
target pH active
active, not at target pH
Density
stickiness
GLU_173_A - circular_variance
p=2.89e-02
target pH active
active, not at target pH
Density
circular_variance
GLU_173_A - electrostatics
p=4.96e-24
target pH active
active, not at target pH
Density
electrostatics
GLU_173_A - kyte_doolittle
p=2.17e-30
target pH active
active, not at target pH
Density
kyte_doolittle
GLU_173_A - stickiness
p=1.90e-08
target pH active
active, not at target pH
Density
stickiness

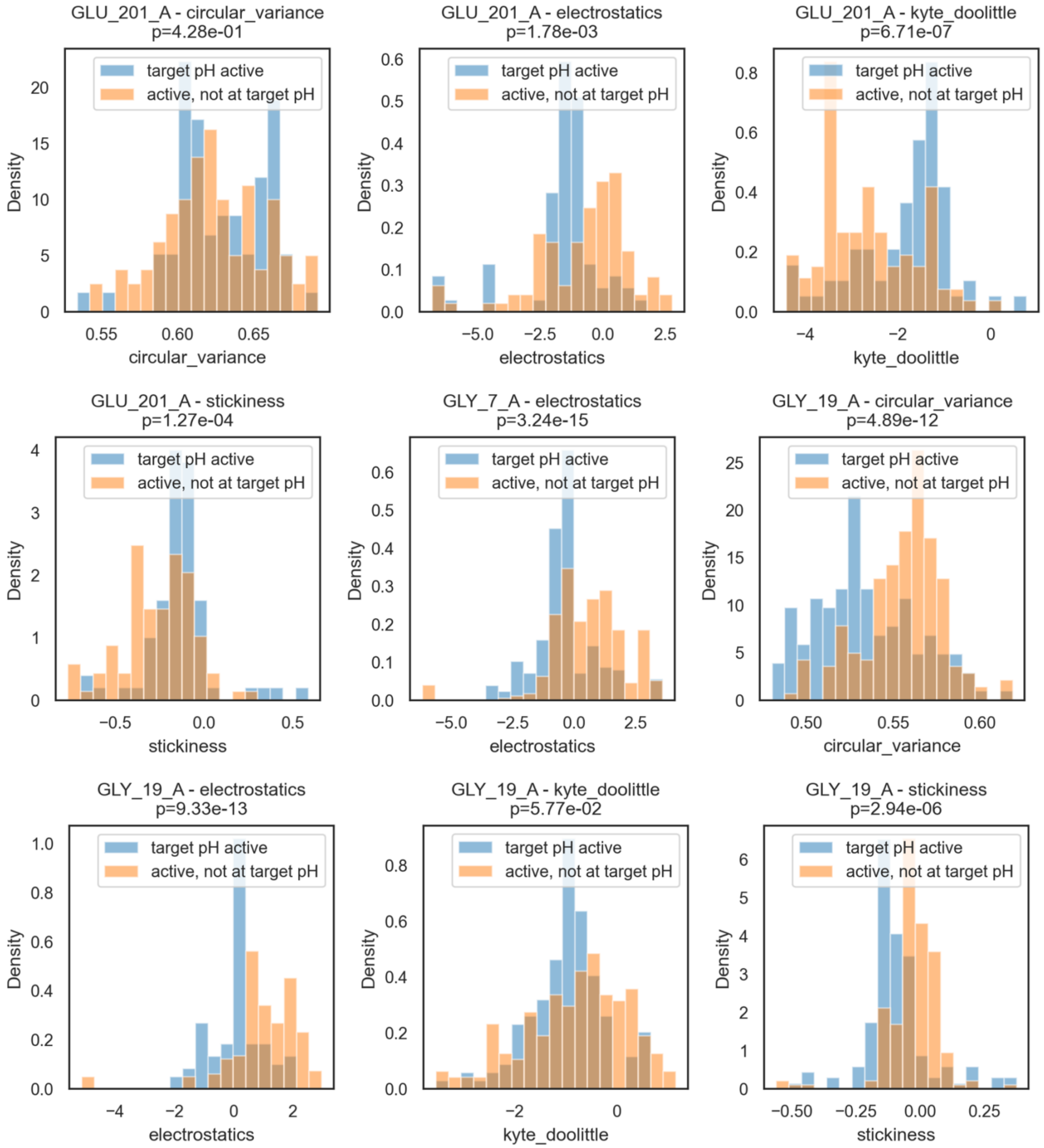
GLU_201_A - circular_variance
p=4.28e-01
target pH active
active, not at target pH
Density
circular_variance
GLU_201_A - electrostatics
p=1.78e-03
target pH active
active, not at target pH
Density
electrostatics
GLU_201_A - kyte_doolittle
p=6.71e-07
target pH active
active, not at target pH
Density
kyte_doolittle
GLU_201_A - stickiness
p=1.27e-04
target pH active
active, not at target pH
Density
stickiness
GLY_7_A - electrostatics
p=3.24e-15
target pH active
active, not at target pH
Density
electrostatics
GLY_19_A - circular_variance
p=4.89e-12
target pH active
active, not at target pH
Density
circular_variance
GLY_19_A - electrostatics
p=9.33e-13
target pH active
active, not at target pH
Density
electrostatics
GLY_19_A - kyte_doolittle
p=5.77e-02
target pH active
active, not at target pH
Density
kyte_doolittle
GLY_19_A - stickiness
p=2.94e-06
target pH active
active, not at target pH
Density
stickiness

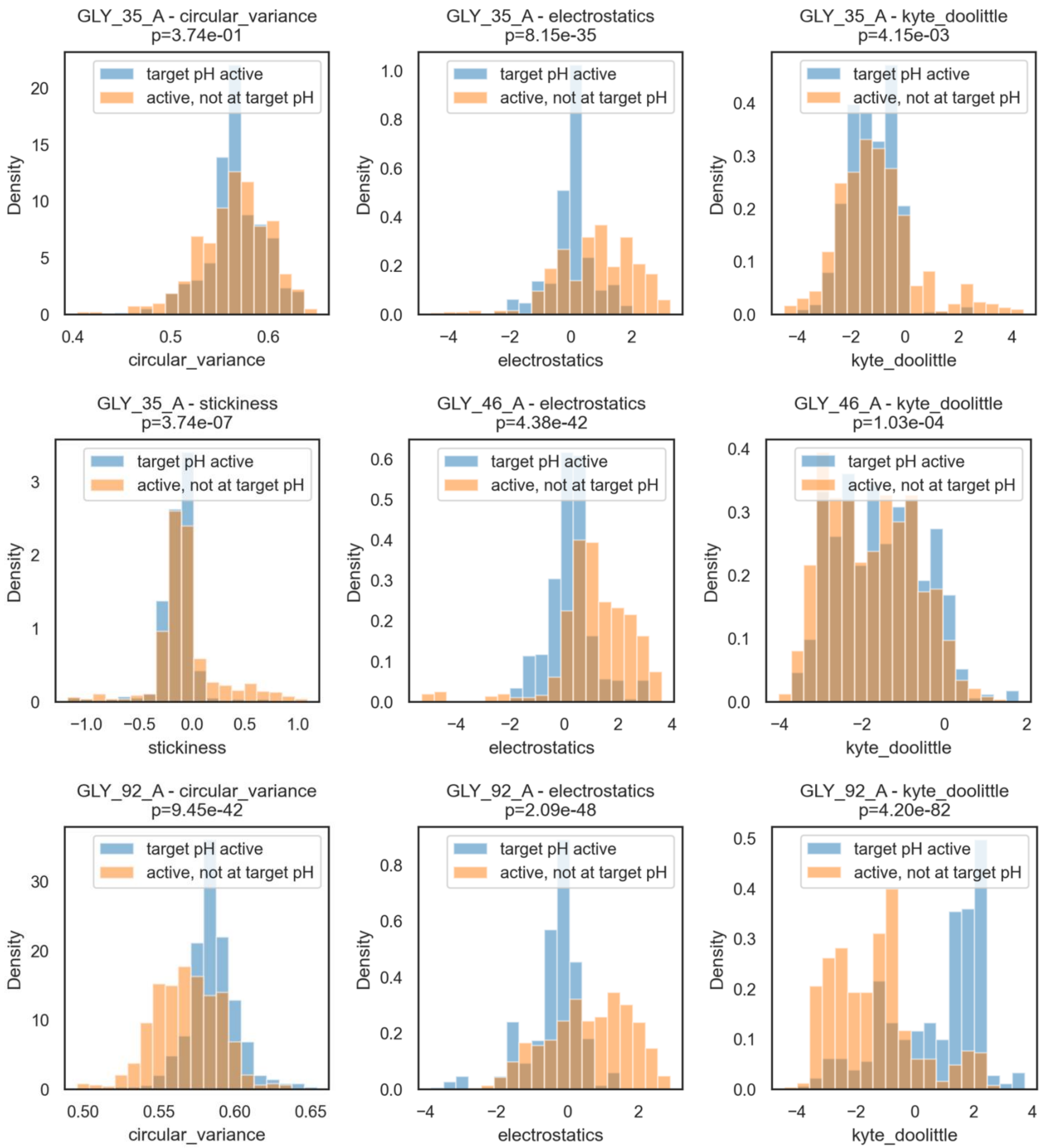
GLY_35_A - circular_variance
p=3.74e-01
GLY_35_A - electrostatics
p=8.15e-35
GLY_35_A - kyte_doolittle
p=4.15e-03
GLY_35_A - stickiness
p=3.74e-07
GLY_46_A - electrostatics
p=4.38e-42
GLY_46_A - kyte_doolittle
p=1.03e-04
GLY_92_A - circular_variance
p=9.45e-42
GLY_92_A - electrostatics
p=2.09e-48
GLY_92_A - kyte_doolittle
p=4.20e-82
target pH active
active, not at target pH
Density
circular_variance
electrostatics
kyte_doolittle
stickiness

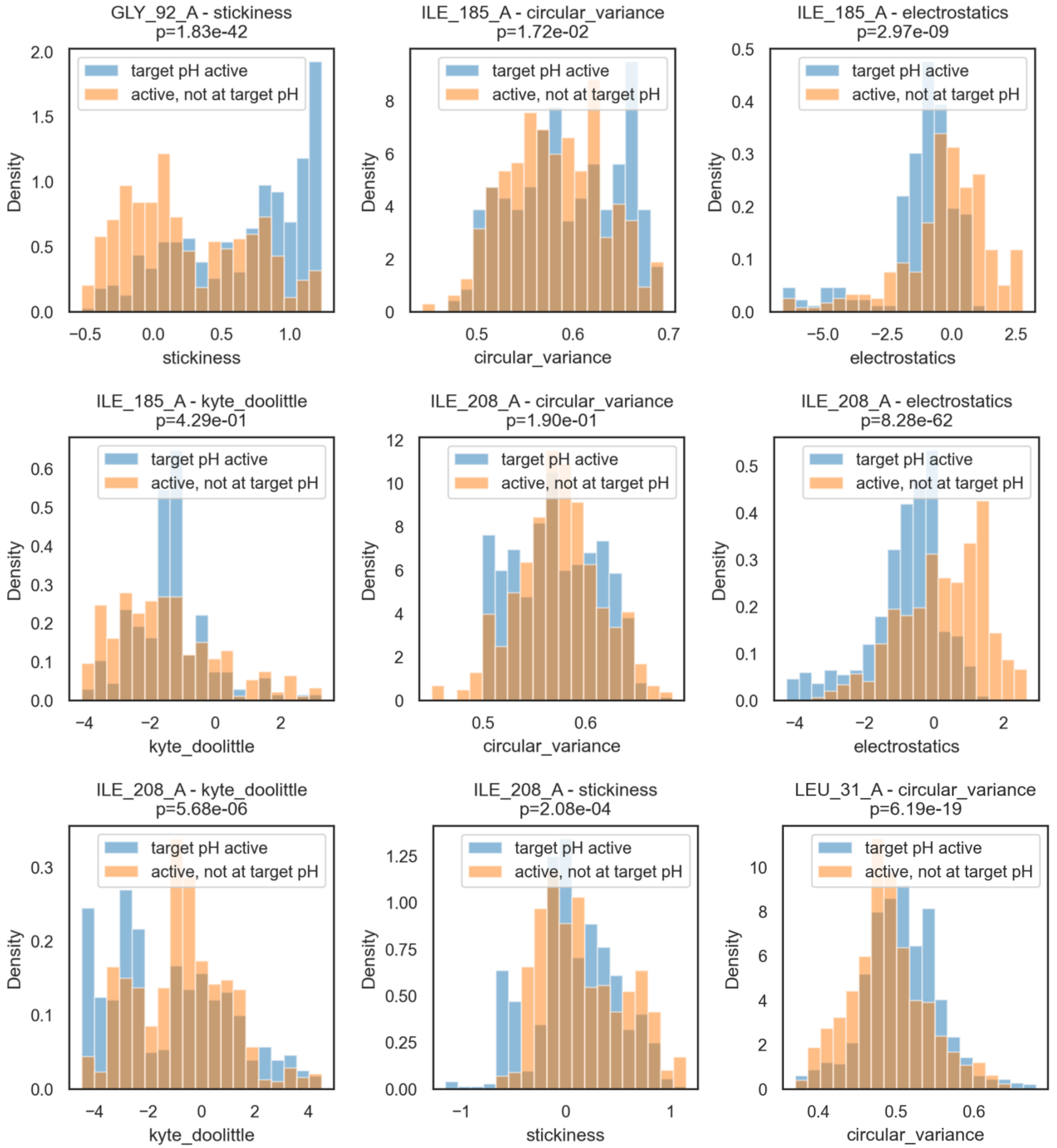
GLY_92_A - stickiness
p=1.83e-42
ILE_185_A - circular_variance
p=1.72e-02
ILE_185_A - electrostatics
p=2.97e-09
ILE_185_A - kyte_doolittle
p=4.29e-01
ILE_208_A - circular_variance
p=1.90e-01
ILE_208_A - electrostatics
p=8.28e-62
ILE_208_A - kyte_doolittle
p=5.68e-06
ILE_208_A - stickiness
p=2.08e-04
LEU_31_A - circular_variance
p=6.19e-19
target pH active
active, not at target pH
Density
stickiness
circular_variance
electrostatics
kyte_doolittle

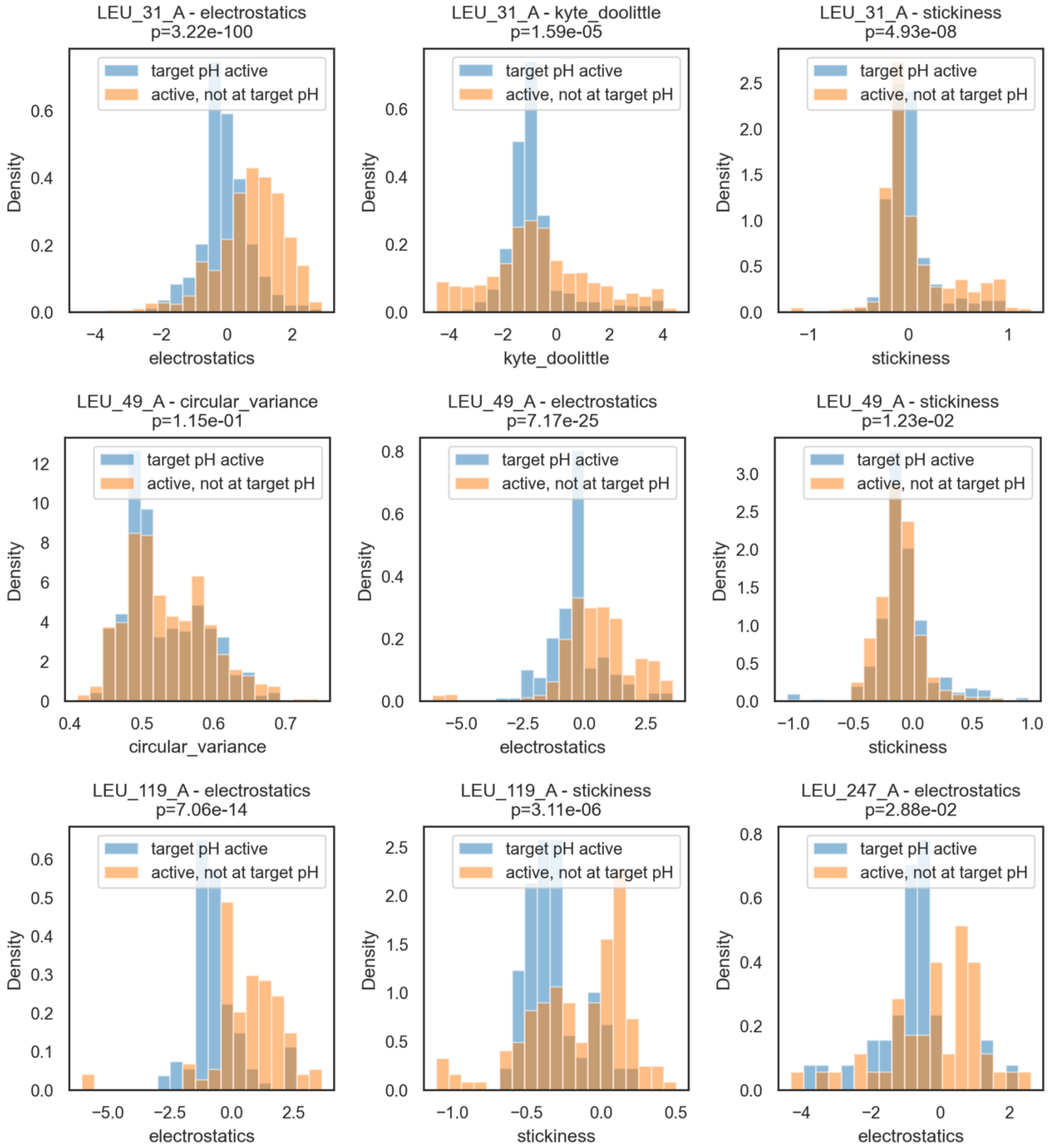
LEU_31_A - electrostatics
p=3.22e-100
target pH active
active, not at target pH
Density
electrostatics
LEU_31_A - kyte_doolittle
p=1.59e-05
target pH active
active, not at target pH
Density
kyte_doolittle
LEU_31_A - stickiness
p=4.93e-08
target pH active
active, not at target pH
Density
stickiness
LEU_49_A - circular_variance
p=1.15e-01
target pH active
active, not at target pH
Density
circular_variance
LEU_49_A - electrostatics
p=7.17e-25
target pH active
active, not at target pH
Density
electrostatics
LEU_49_A - stickiness
p=1.23e-02
target pH active
active, not at target pH
Density
stickiness
LEU_119_A - electrostatics
p=7.06e-14
target pH active
active, not at target pH
Density
electrostatics
LEU_119_A - stickiness
p=3.11e-06
target pH active
active, not at target pH
Density
stickiness
LEU_247_A - electrostatics
p=2.88e-02
target pH active
active, not at target pH
Density
electrostatics

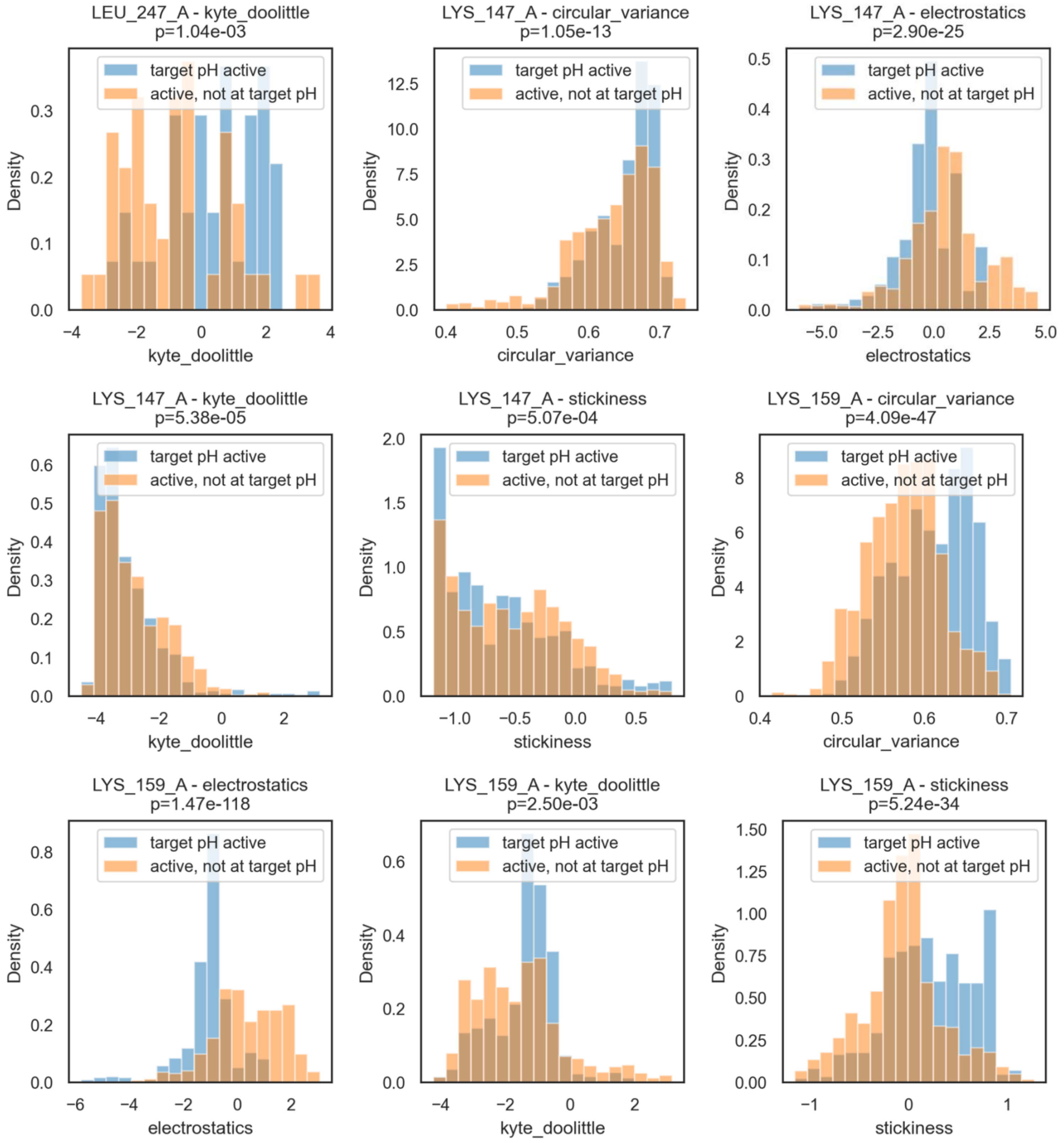
LEU_247_A - kyte_doolittle
p=1.04e-03
LYS_147_A - circular_variance
p=1.05e-13
LYS_147_A - electrostatics
p=2.90e-25
LYS_147_A - kyte_doolittle
p=5.38e-05
LYS_147_A - stickiness
p=5.07e-04
LYS_159_A - circular_variance
p=4.09e-47
LYS_159_A - electrostatics
p=1.47e-118
LYS_159_A - kyte_doolittle
p=2.50e-03
LYS_159_A - stickiness
p=5.24e-34
target pH active
active, not at target pH
Density
kyte_doolittle
circular_variance
electrostatics
stickiness

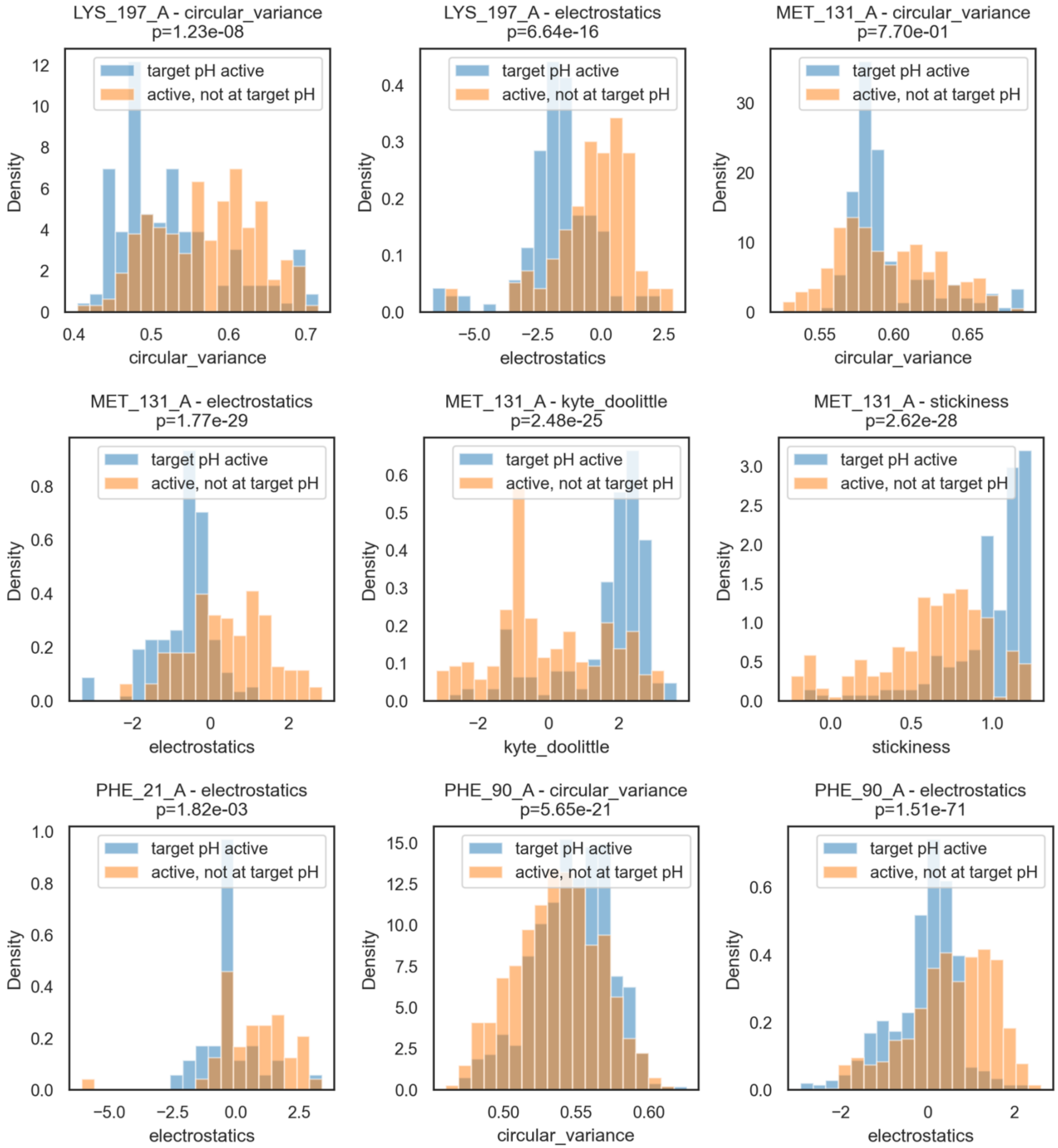
LYS_197_A - circular_variance
p=1.23e-08
target pH active
active, not at target pH
Density
0
2
4
6
8
10
12
0.4
0.5
0.6
0.7
circular_variance
LYS_197_A - electrostatics
p=6.64e-16
target pH active
active, not at target pH
Density
0.0
0.1
0.2
0.3
0.4
−5.0
−2.5
0.0
2.5
electrostatics
MET_131_A - circular_variance
p=7.70e-01
target pH active
active, not at target pH
Density
0
10
20
30
0.55
0.60
0.65
circular_variance
MET_131_A - electrostatics
p=1.77e-29
target pH active
active, not at target pH
Density
0.0
0.2
0.4
0.6
0.8
−2
0
2
electrostatics
MET_131_A - kyte_doolittle
p=2.48e-25
target pH active
active, not at target pH
Density
0.0
0.1
0.2
0.3
0.4
0.5
0.6
−2
0
2
kyte_doolittle
MET_131_A - stickiness
p=2.62e-28
target pH active
active, not at target pH
Density
0.0
0.5
1.0
1.5
2.0
2.5
3.0
0.0
0.5
1.0
stickiness
PHE_21_A - electrostatics
p=1.82e-03
target pH active
active, not at target pH
Density
0.0
0.2
0.4
0.6
0.8
1.0
−5.0
−2.5
0.0
2.5
electrostatics
PHE_90_A - circular_variance
p=5.65e-21
target pH active
active, not at target pH
Density
0.0
2.5
5.0
7.5
10.0
12.5
15.0
0.50
0.55
0.60
circular_variance
PHE_90_A - electrostatics
p=1.51e-71
target pH active
active, not at target pH
Density
0.0
0.2
0.4
0.6
−2
0
2
electrostatics

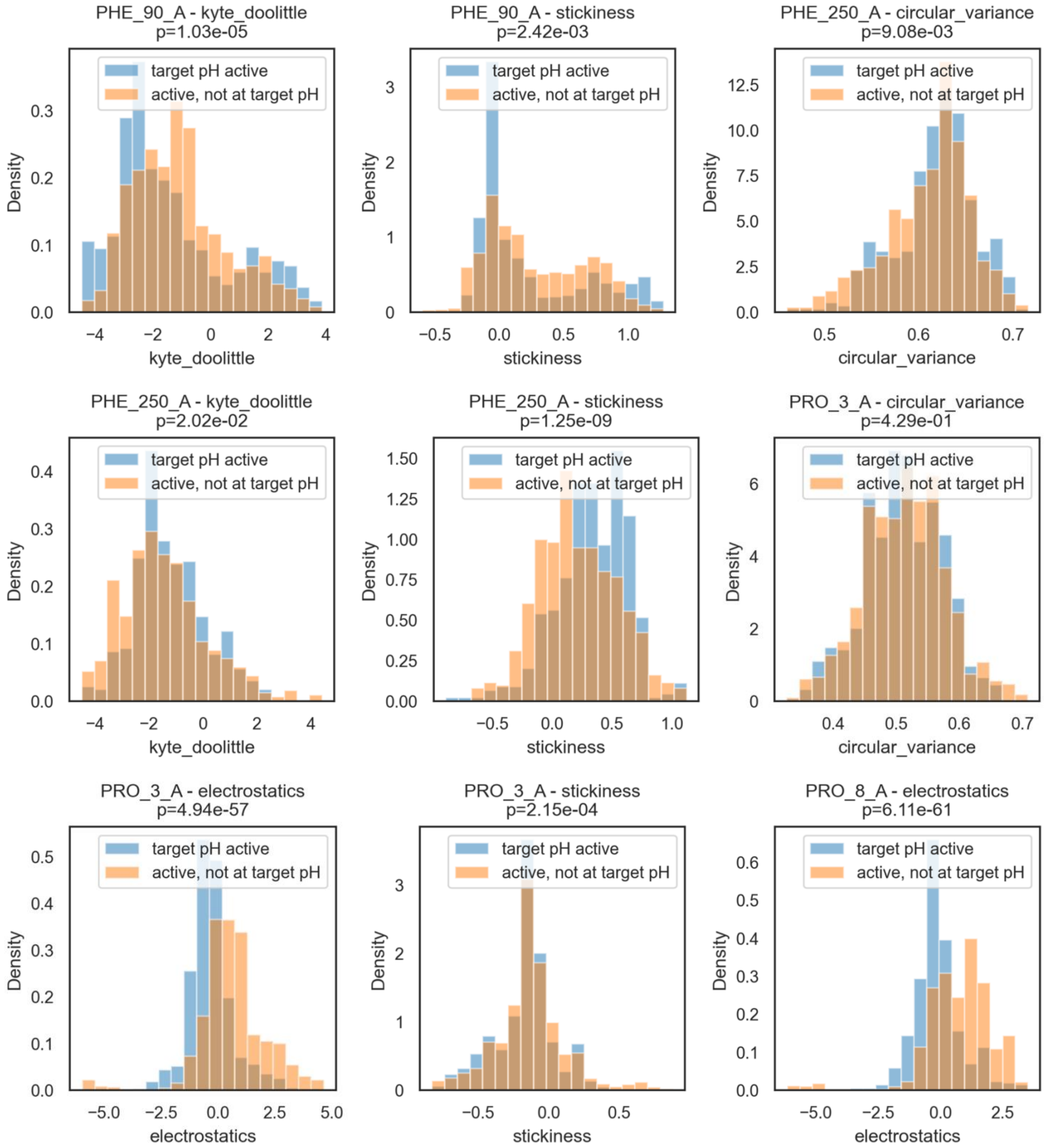
PHE_90_A - kyte_doolittle
p=1.03e-05
target pH active
active, not at target pH
Density
kyte_doolittle
PHE_90_A - stickiness
p=2.42e-03
target pH active
active, not at target pH
Density
stickiness
PHE_250_A - circular_variance
p=9.08e-03
target pH active
active, not at target pH
Density
circular_variance
PHE_250_A - kyte_doolittle
p=2.02e-02
target pH active
active, not at target pH
Density
kyte_doolittle
PHE_250_A - stickiness
p=1.25e-09
target pH active
active, not at target pH
Density
stickiness
PRO_3_A - circular_variance
p=4.29e-01
target pH active
active, not at target pH
Density
circular_variance
PRO_3_A - electrostatics
p=4.94e-57
target pH active
active, not at target pH
Density
electrostatics
PRO_3_A - stickiness
p=2.15e-04
target pH active
active, not at target pH
Density
stickiness
PRO_8_A - electrostatics
p=6.11e-61
target pH active
active, not at target pH
Density
electrostatics

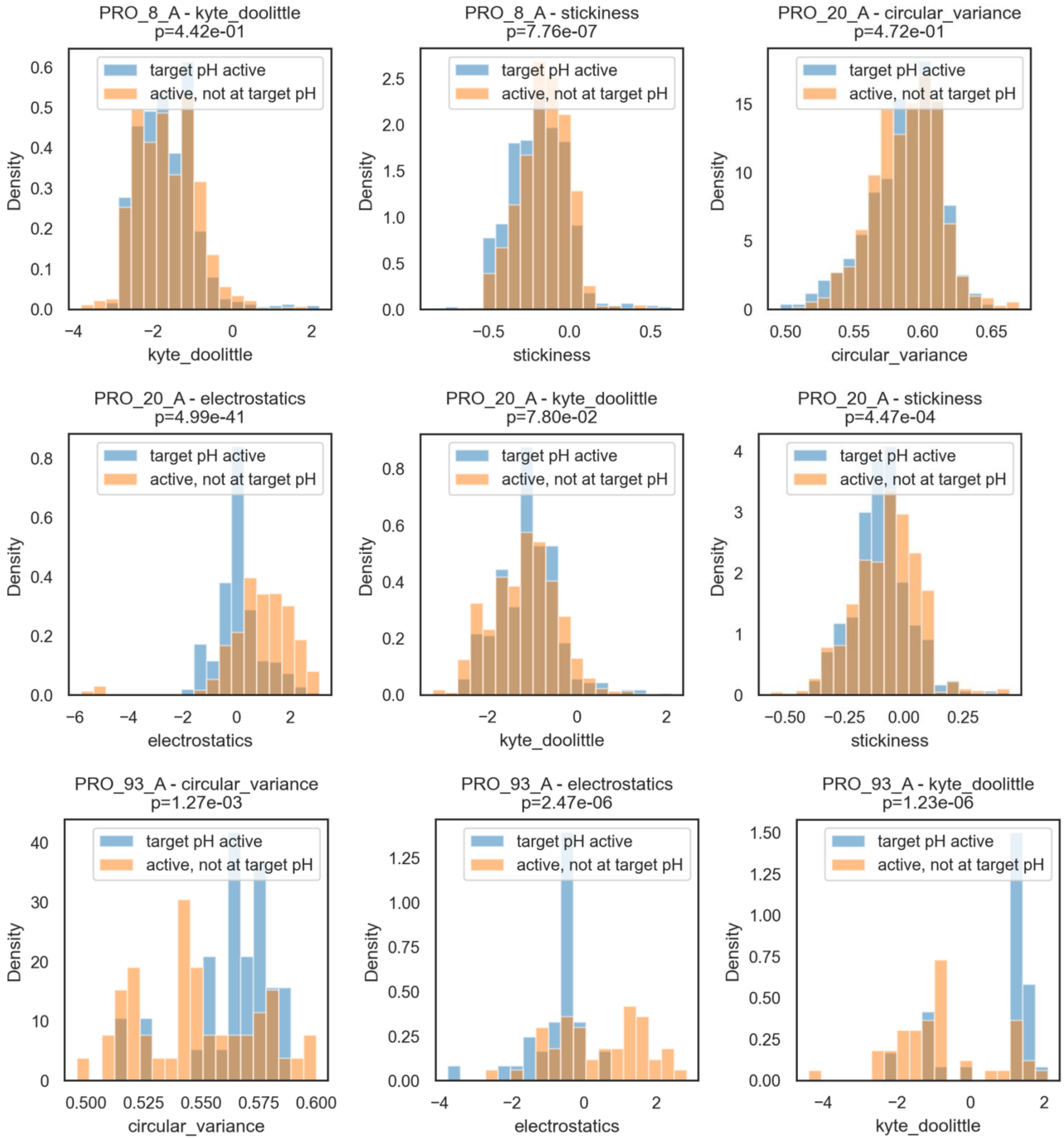
PRO_8_A - kyte_doolittle
p=4.42e-01
target pH active
active, not at target pH
Density
kyte_doolittle
PRO_8_A - stickiness
p=7.76e-07
target pH active
active, not at target pH
Density
stickiness
PRO_20_A - circular_variance
p=4.72e-01
target pH active
active, not at target pH
Density
circular_variance
PRO_20_A - electrostatics
p=4.99e-41
target pH active
active, not at target pH
Density
electrostatics
PRO_20_A - kyte_doolittle
p=7.80e-02
target pH active
active, not at target pH
Density
kyte_doolittle
PRO_20_A - stickiness
p=4.47e-04
target pH active
active, not at target pH
Density
stickiness
PRO_93_A - circular_variance
p=1.27e-03
target pH active
active, not at target pH
Density
circular_variance
PRO_93_A - electrostatics
p=2.47e-06
target pH active
active, not at target pH
Density
electrostatics
PRO_93_A - kyte_doolittle
p=1.23e-06
target pH active
active, not at target pH
Density
kyte_doolittle

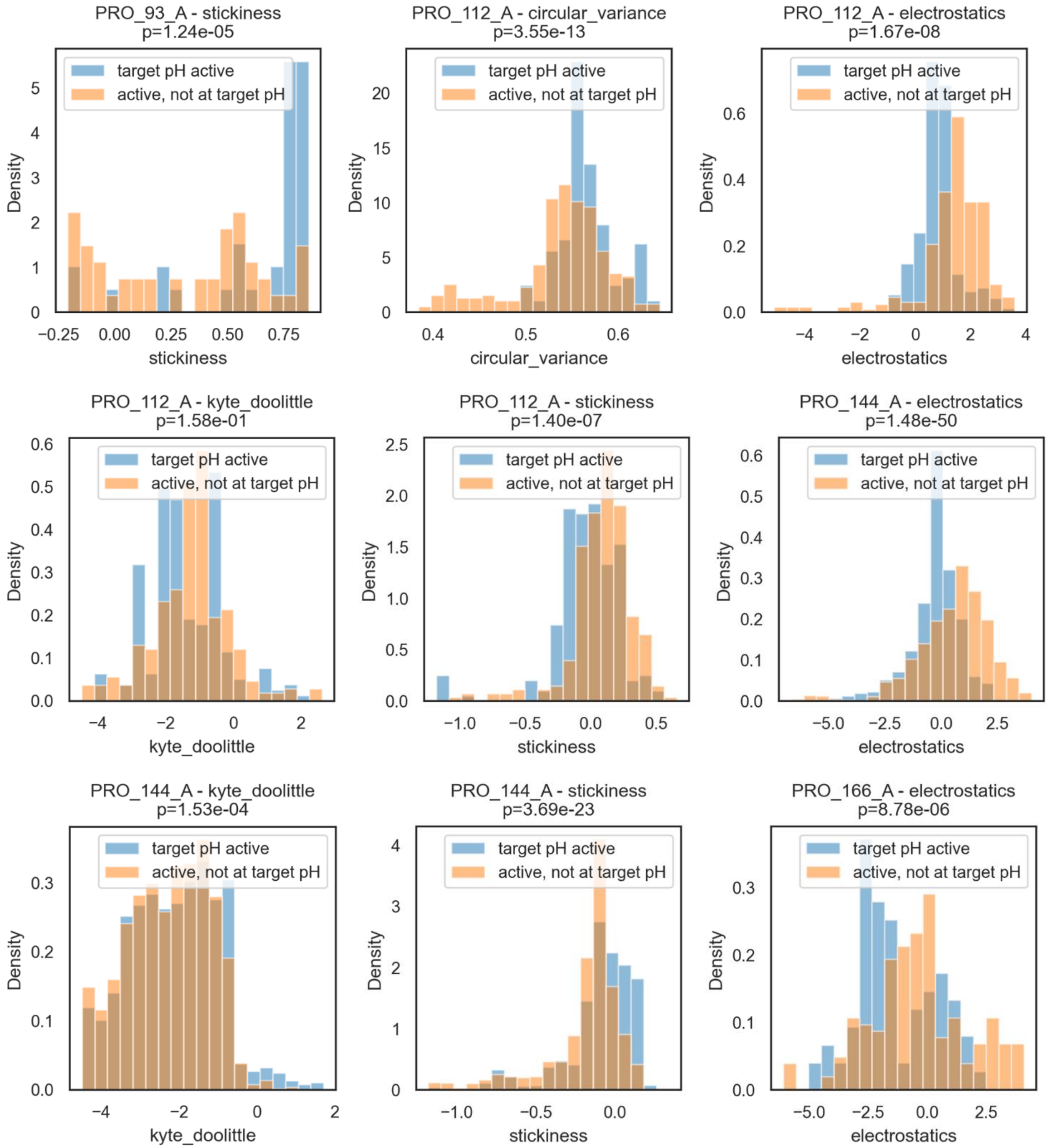
PRO_93_A - stickiness
p=1.24e-05
target pH active
active, not at target pH
Density
stickiness
PRO_112_A - circular_variance
p=3.55e-13
circular_variance
PRO_112_A - electrostatics
p=1.67e-08
electrostatics
PRO_112_A - kyte_doolittle
p=1.58e-01
kyte_doolittle
PRO_112_A - stickiness
p=1.40e-07
stickiness
PRO_144_A - electrostatics
p=1.48e-50
electrostatics
PRO_144_A - kyte_doolittle
p=1.53e-04
kyte_doolittle
PRO_144_A - stickiness
p=3.69e-23
stickiness
PRO_166_A - electrostatics
p=8.78e-06
electrostatics

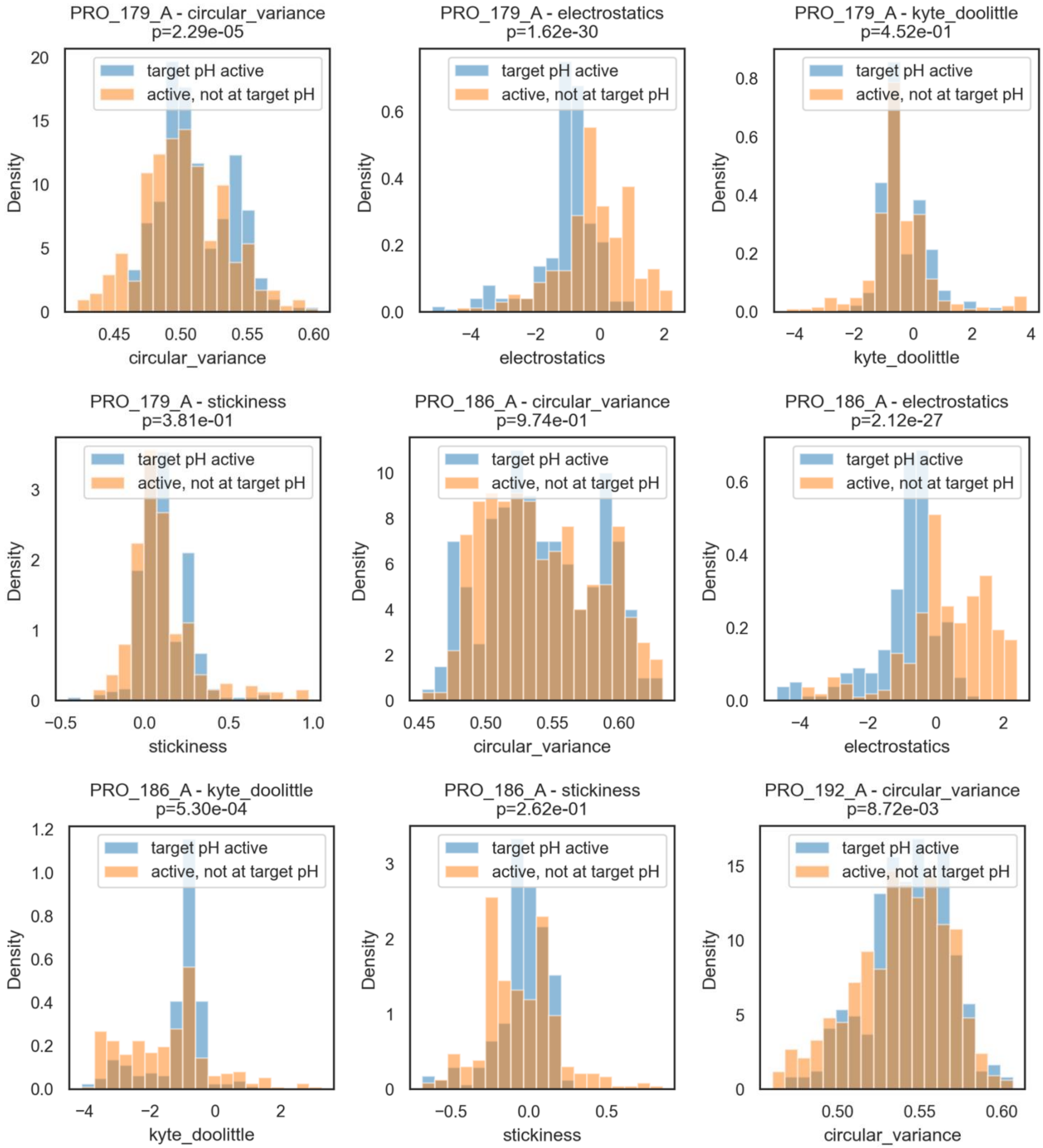
PRO_179_A - circular_variance
p=2.29e-05
Density
circular_variance
target pH active
active, not at target pH
PRO_179_A - electrostatics
p=1.62e-30
Density
electrostatics
target pH active
active, not at target pH
PRO_179_A - kyte_doolittle
p=4.52e-01
Density
kyte_doolittle
target pH active
active, not at target pH
PRO_179_A - stickiness
p=3.81e-01
Density
stickiness
target pH active
active, not at target pH
PRO_186_A - circular_variance
p=9.74e-01
Density
circular_variance
target pH active
active, not at target pH
PRO_186_A - electrostatics
p=2.12e-27
Density
electrostatics
target pH active
active, not at target pH
PRO_186_A - kyte_doolittle
p=5.30e-04
Density
kyte_doolittle
target pH active
active, not at target pH
PRO_186_A - stickiness
p=2.62e-01
Density
stickiness
target pH active
active, not at target pH
PRO_192_A - circular_variance
p=8.72e-03
Density
circular_variance
target pH active
active, not at target pH

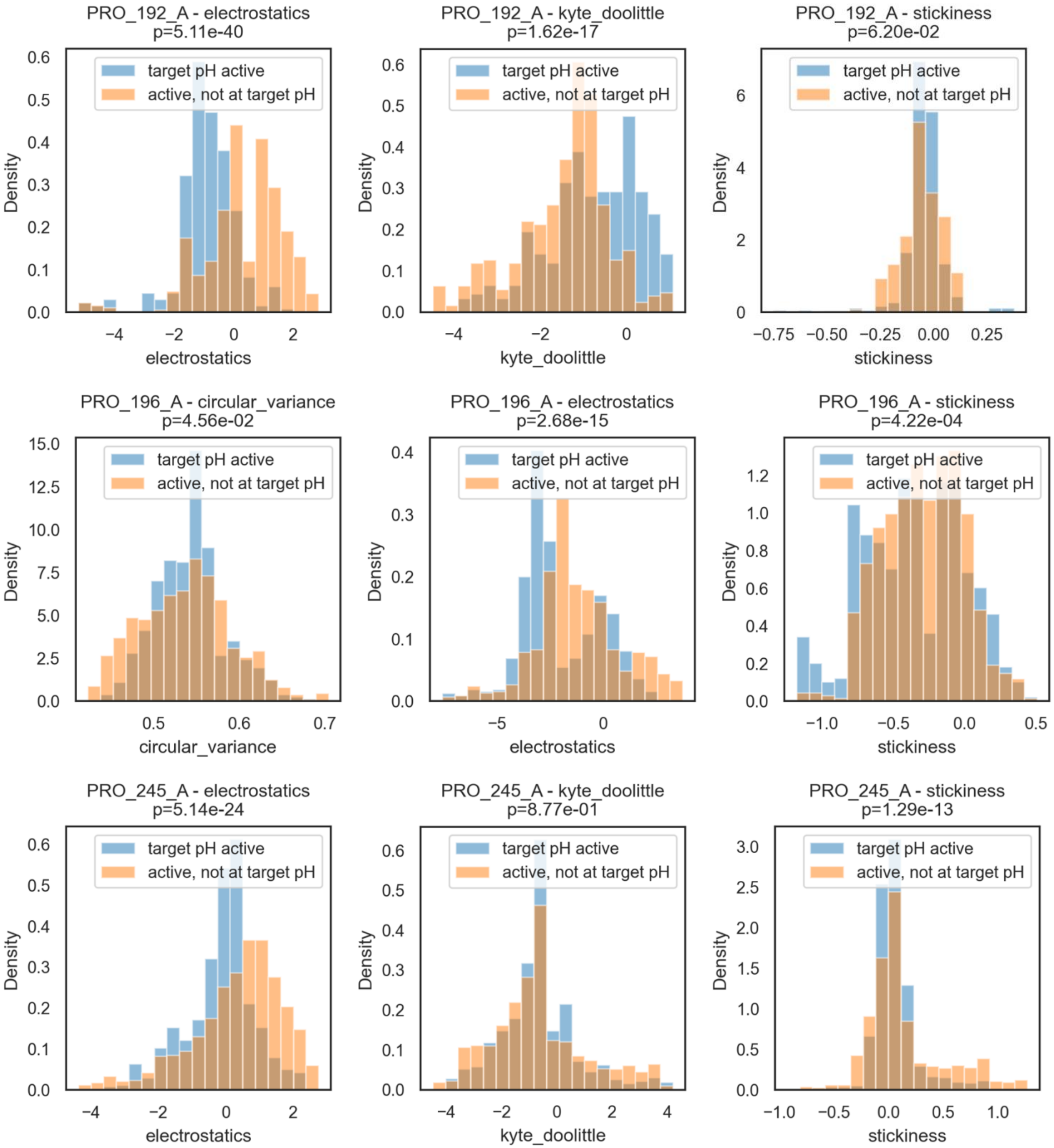
PRO_192_A - electrostatics
p=5.11e-40
PRO_192_A - kyte_doolittle
p=1.62e-17
PRO_192_A - stickiness
p=6.20e-02
PRO_196_A - circular_variance
p=4.56e-02
PRO_196_A - electrostatics
p=2.68e-15
PRO_196_A - stickiness
p=4.22e-04
PRO_245_A - electrostatics
p=5.14e-24
PRO_245_A - kyte_doolittle
p=8.77e-01
PRO_245_A - stickiness
p=1.29e-13
target pH active
active, not at target pH
Density
electrostatics
kyte_doolittle
stickiness
circular_variance

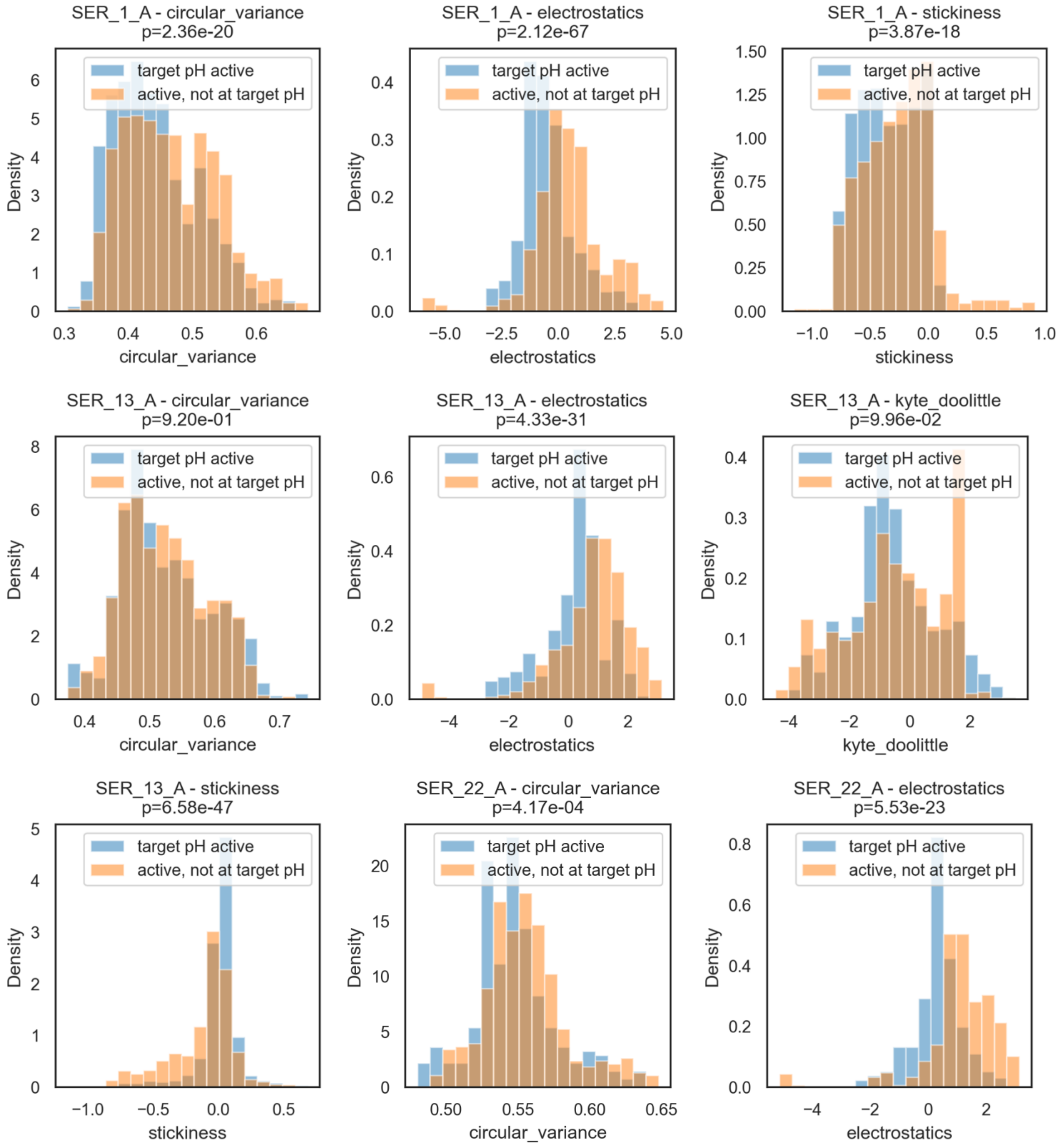
SER_1_A - circular_variance
p=2.36e-20
target pH active
active, not at target pH
Density
circular_variance
SER_1_A - electrostatics
p=2.12e-67
electrostatics
SER_1_A - stickiness
p=3.87e-18
stickiness
SER_13_A - circular_variance
p=9.20e-01
SER_13_A - electrostatics
p=4.33e-31
SER_13_A - kyte_doolittle
p=9.96e-02
kyte_doolittle
SER_13_A - stickiness
p=6.58e-47
SER_22_A - circular_variance
p=4.17e-04
SER_22_A - electrostatics
p=5.53e-23

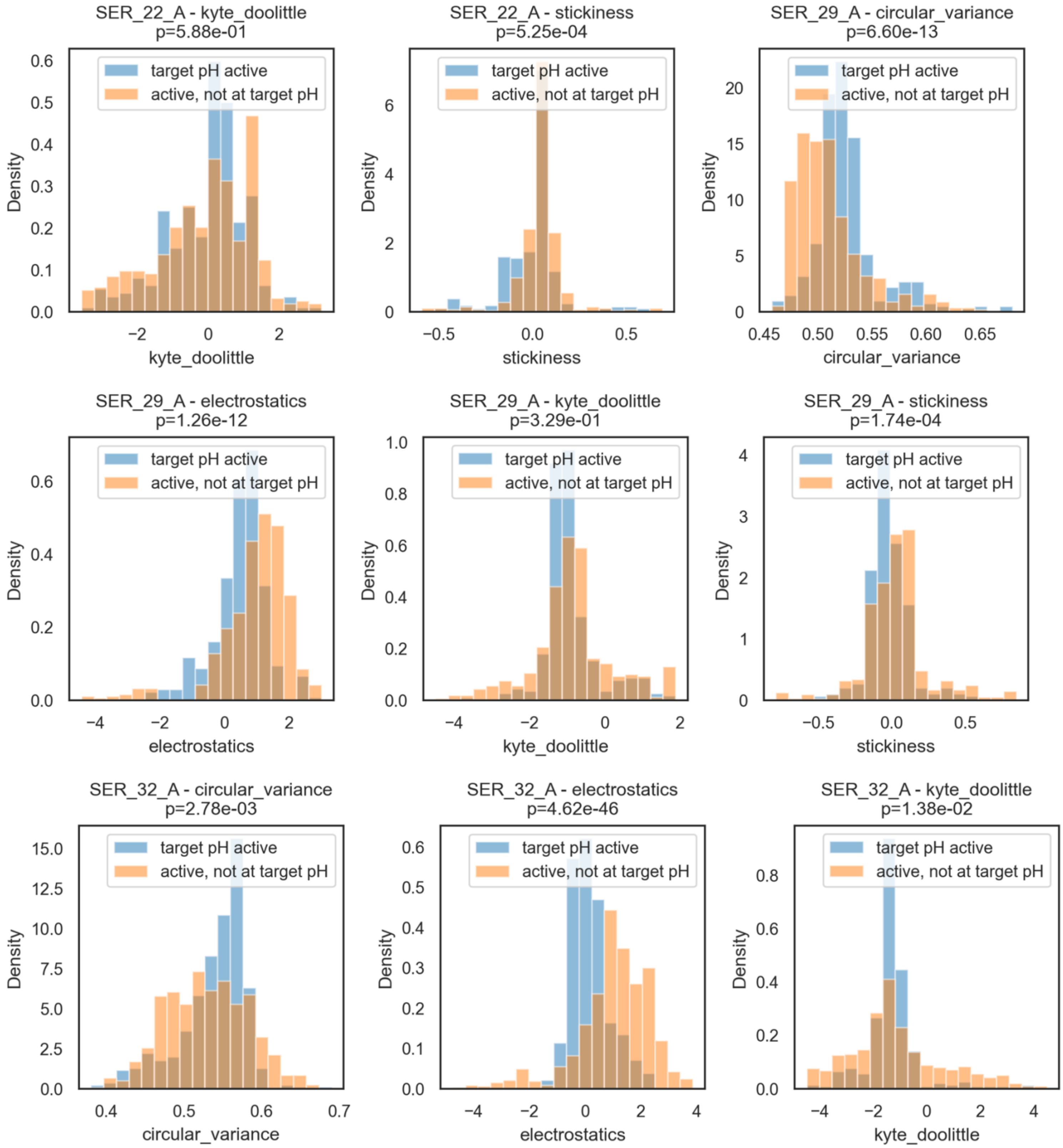
SER_22_A - kyte_doolittle
p=5.88e-01
SER_22_A - stickiness
p=5.25e-04
SER_29_A - circular_variance
p=6.60e-13
SER_29_A - electrostatics
p=1.26e-12
SER_29_A - kyte_doolittle
p=3.29e-01
SER_29_A - stickiness
p=1.74e-04
SER_32_A - circular_variance
p=2.78e-03
SER_32_A - electrostatics
p=4.62e-46
SER_32_A - kyte_doolittle
p=1.38e-02
target pH active
active, not at target pH
Density
kyte_doolittle
stickiness
circular_variance
electrostatics

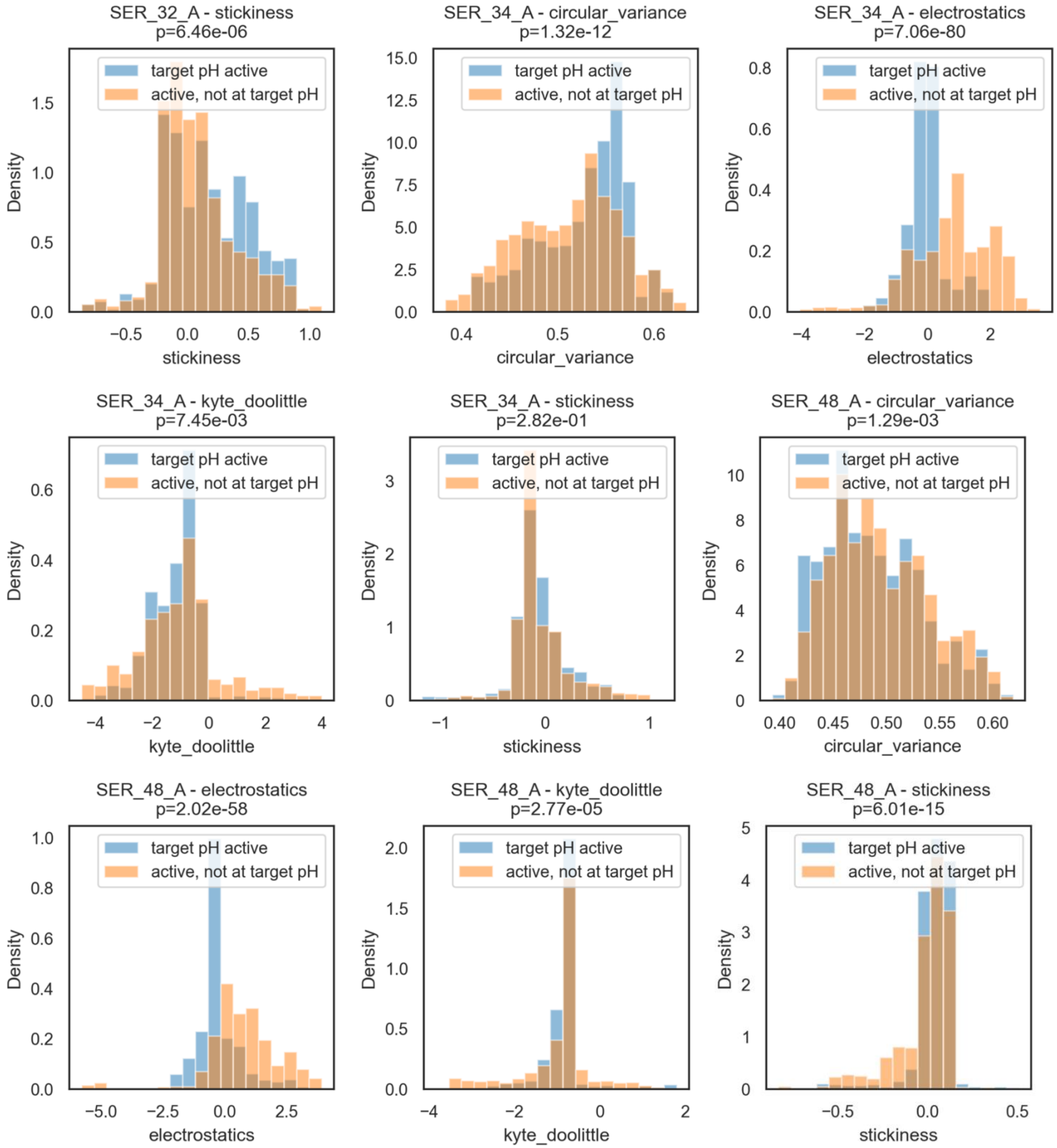
SER_32_A - stickiness
p=6.46e-06
target pH active
active, not at target pH
Density
stickiness
SER_34_A - circular_variance
p=1.32e-12
target pH active
active, not at target pH
Density
circular_variance
SER_34_A - electrostatics
p=7.06e-80
target pH active
active, not at target pH
Density
electrostatics
SER_34_A - kyte_doolittle
p=7.45e-03
target pH active
active, not at target pH
Density
kyte_doolittle
SER_34_A - stickiness
p=2.82e-01
target pH active
active, not at target pH
Density
stickiness
SER_48_A - circular_variance
p=1.29e-03
target pH active
active, not at target pH
Density
circular_variance
SER_48_A - electrostatics
p=2.02e-58
target pH active
active, not at target pH
Density
electrostatics
SER_48_A - kyte_doolittle
p=2.77e-05
target pH active
active, not at target pH
Density
kyte_doolittle
SER_48_A - stickiness
p=6.01e-15
target pH active
active, not at target pH
Density
stickiness

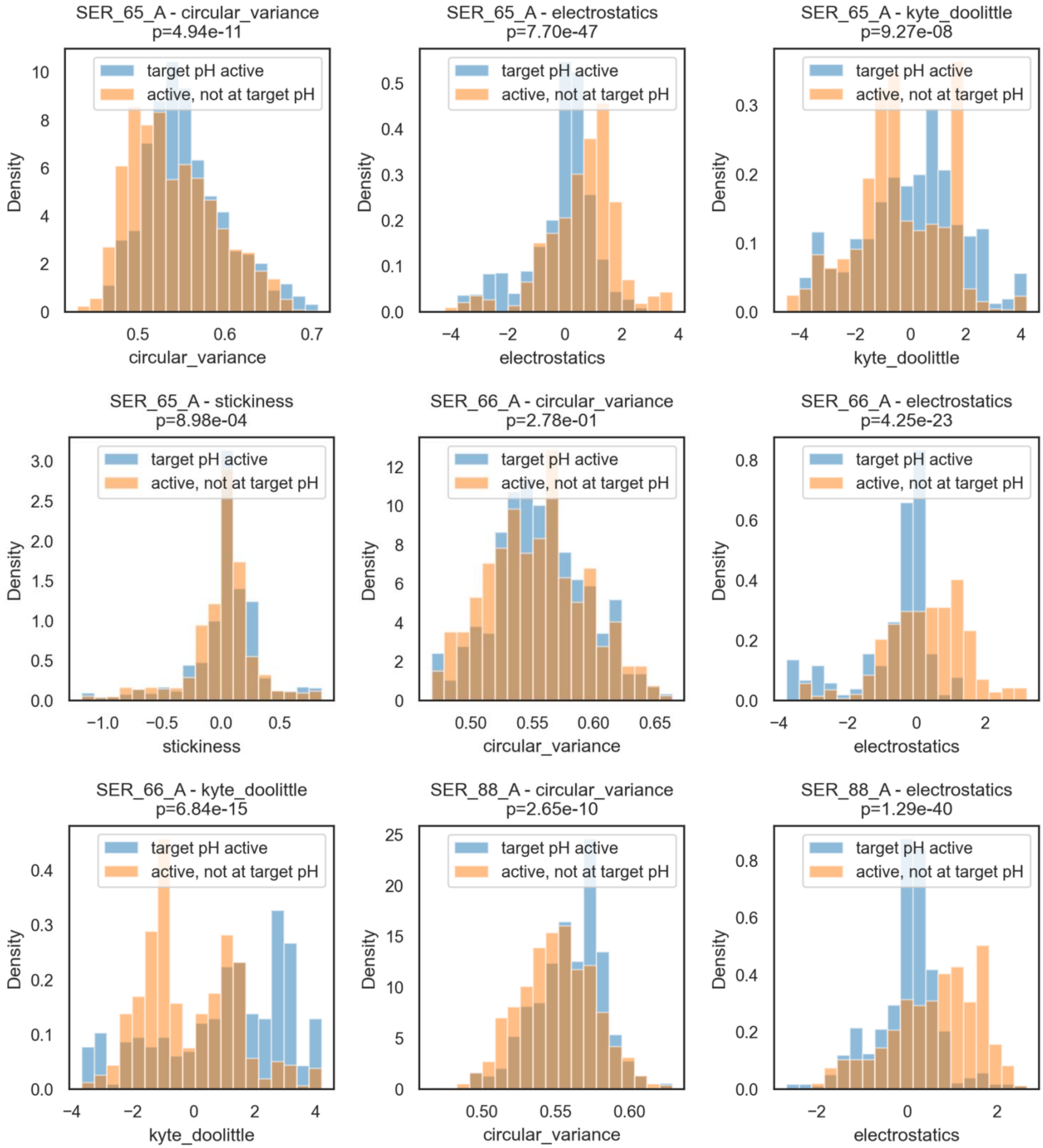
SER_65_A - circular_variance
p=4.94e-11
SER_65_A - electrostatics
p=7.70e-47
SER_65_A - kyte_doolittle
p=9.27e-08
SER_65_A - stickiness
p=8.98e-04
SER_66_A - circular_variance
p=2.78e-01
SER_66_A - electrostatics
p=4.25e-23
SER_66_A - kyte_doolittle
p=6.84e-15
SER_88_A - circular_variance
p=2.65e-10
SER_88_A - electrostatics
p=1.29e-40
target pH active
active, not at target pH
Density
circular_variance
electrostatics
kyte_doolittle
stickiness

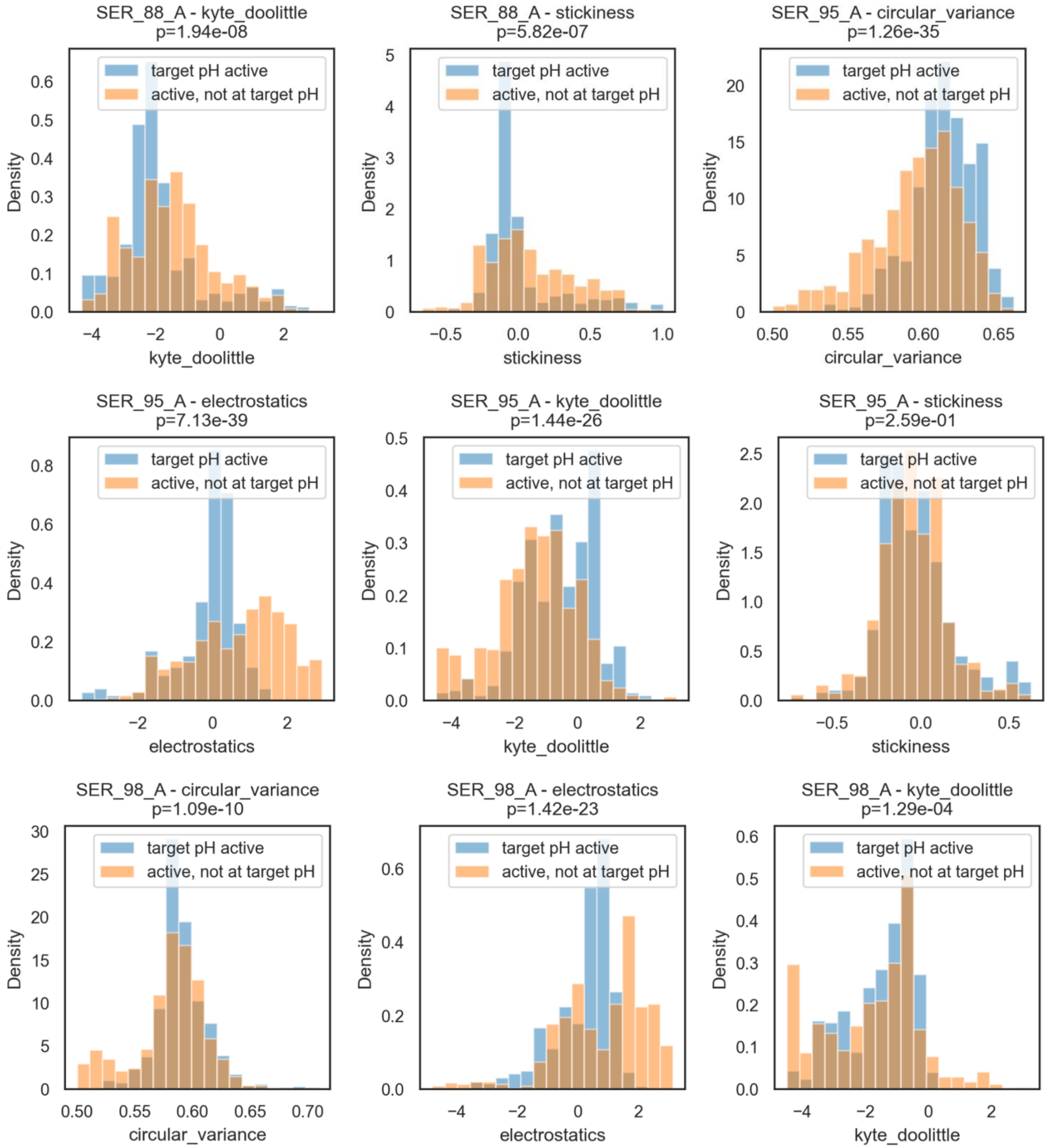
SER_88_A - kyte_doolittle
p=1.94e-08
target pH active
active, not at target pH
Density
kyte_doolittle
SER_88_A - stickiness
p=5.82e-07
target pH active
active, not at target pH
Density
stickiness
SER_95_A - circular_variance
p=1.26e-35
target pH active
active, not at target pH
Density
circular_variance
SER_95_A - electrostatics
p=7.13e-39
target pH active
active, not at target pH
Density
electrostatics
SER_95_A - kyte_doolittle
p=1.44e-26
target pH active
active, not at target pH
Density
kyte_doolittle
SER_95_A - stickiness
p=2.59e-01
target pH active
active, not at target pH
Density
stickiness
SER_98_A - circular_variance
p=1.09e-10
target pH active
active, not at target pH
Density
circular_variance
SER_98_A - electrostatics
p=1.42e-23
target pH active
active, not at target pH
Density
electrostatics
SER_98_A - kyte_doolittle
p=1.29e-04
target pH active
active, not at target pH
Density
kyte_doolittle

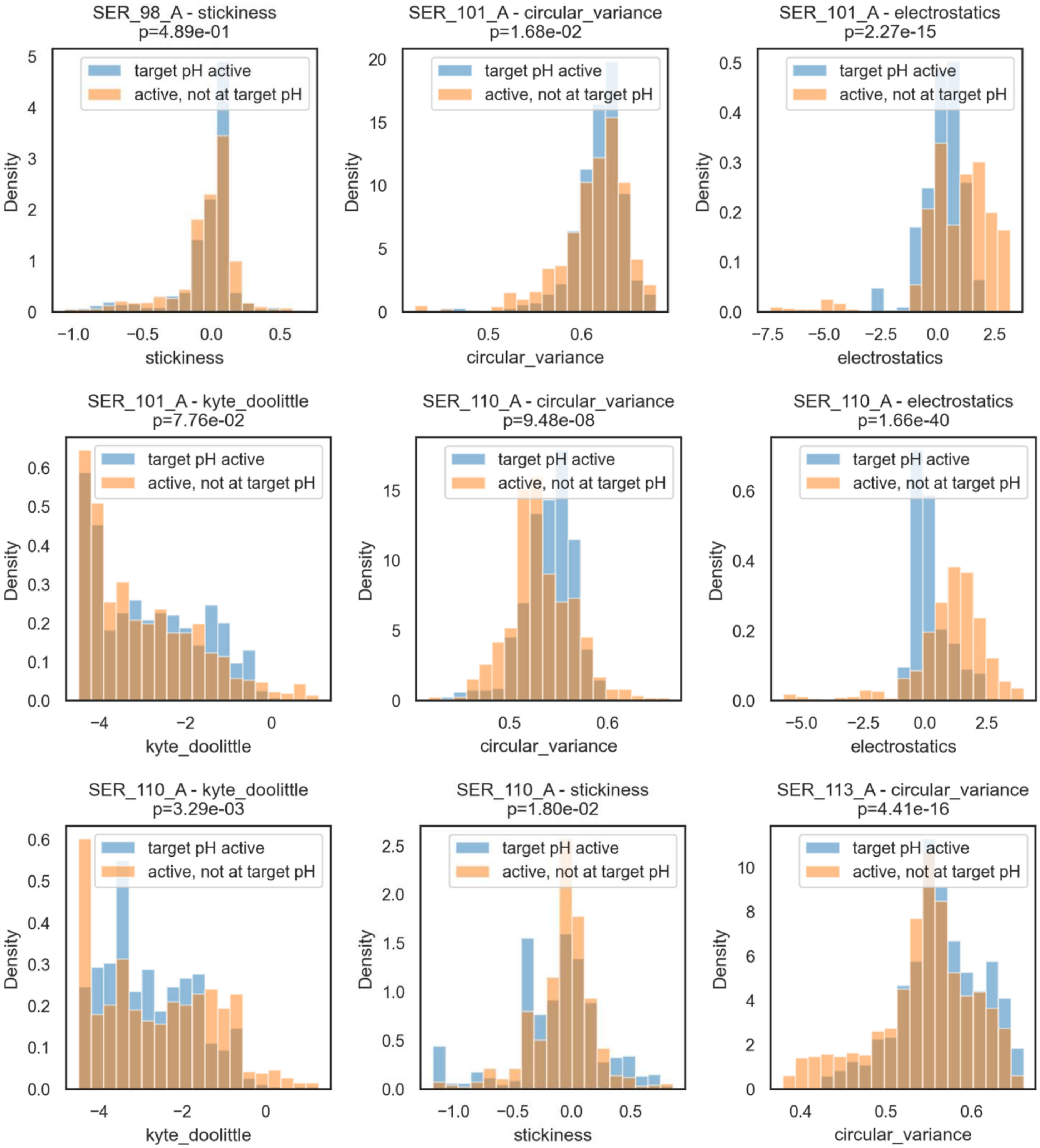
SER_98_A - stickiness
p=4.89e-01
SER_101_A - circular_variance
p=1.68e-02
SER_101_A - electrostatics
p=2.27e-15
SER_101_A - kyte_doolittle
p=7.76e-02
SER_110_A - circular_variance
p=9.48e-08
SER_110_A - electrostatics
p=1.66e-40
SER_110_A - kyte_doolittle
p=3.29e-03
SER_110_A - stickiness
p=1.80e-02
SER_113_A - circular_variance
p=4.41e-16
target pH active
active, not at target pH
Density
stickiness
circular_variance
electrostatics
kyte_doolittle

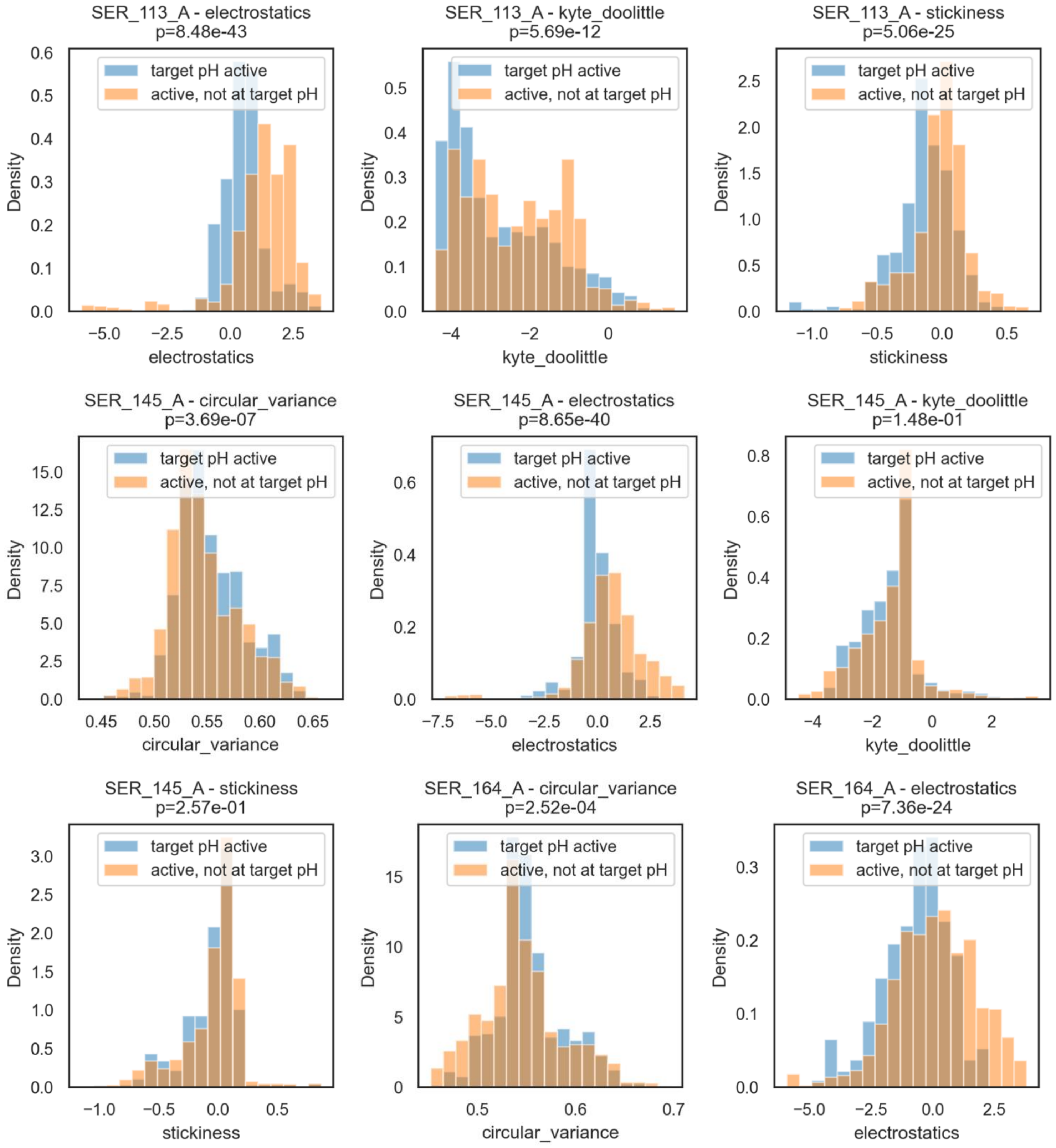
SER_113_A - electrostatics
p=8.48e-43
target pH active
active, not at target pH
Density
electrostatics
SER_113_A - kyte_doolittle
p=5.69e-12
target pH active
active, not at target pH
Density
kyte_doolittle
SER_113_A - stickiness
p=5.06e-25
target pH active
active, not at target pH
Density
stickiness
SER_145_A - circular_variance
p=3.69e-07
target pH active
active, not at target pH
Density
circular_variance
SER_145_A - electrostatics
p=8.65e-40
target pH active
active, not at target pH
Density
electrostatics
SER_145_A - kyte_doolittle
p=1.48e-01
target pH active
active, not at target pH
Density
kyte_doolittle
SER_145_A - stickiness
p=2.57e-01
target pH active
active, not at target pH
Density
stickiness
SER_164_A - circular_variance
p=2.52e-04
target pH active
active, not at target pH
Density
circular_variance
SER_164_A - electrostatics
p=7.36e-24
target pH active
active, not at target pH
Density
electrostatics

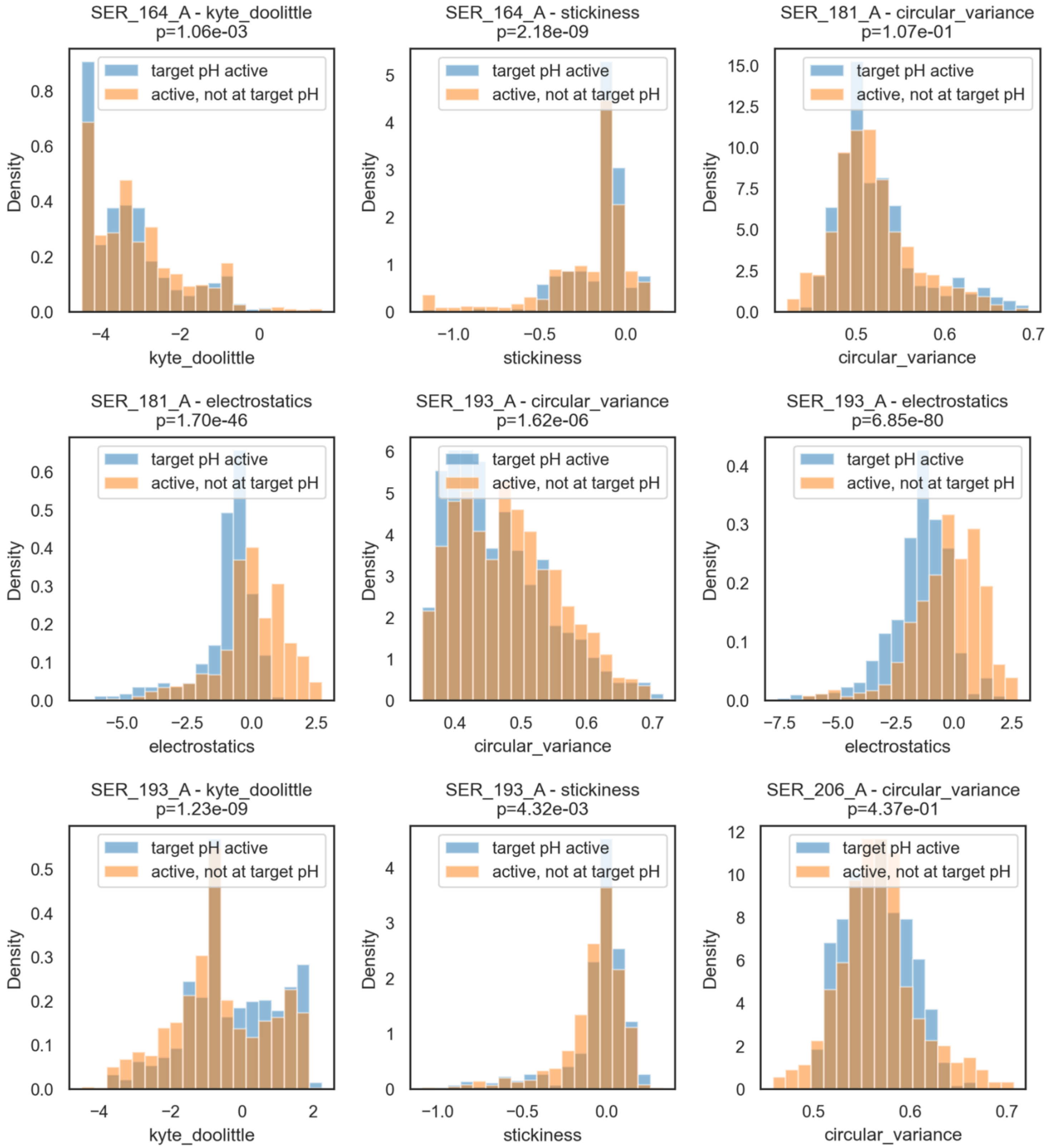
SER_164_A - kyte_doolittle
p=1.06e-03
target pH active
active, not at target pH
Density
kyte_doolittle
SER_164_A - stickiness
p=2.18e-09
target pH active
active, not at target pH
Density
stickiness
SER_181_A - circular_variance
p=1.07e-01
target pH active
active, not at target pH
Density
circular_variance
SER_181_A - electrostatics
p=1.70e-46
target pH active
active, not at target pH
Density
electrostatics
SER_193_A - circular_variance
p=1.62e-06
target pH active
active, not at target pH
Density
circular_variance
SER_193_A - electrostatics
p=6.85e-80
target pH active
active, not at target pH
Density
electrostatics
SER_193_A - kyte_doolittle
p=1.23e-09
target pH active
active, not at target pH
Density
kyte_doolittle
SER_193_A - stickiness
p=4.32e-03
target pH active
active, not at target pH
Density
stickiness
SER_206_A - circular_variance
p=4.37e-01
target pH active
active, not at target pH
Density
circular_variance

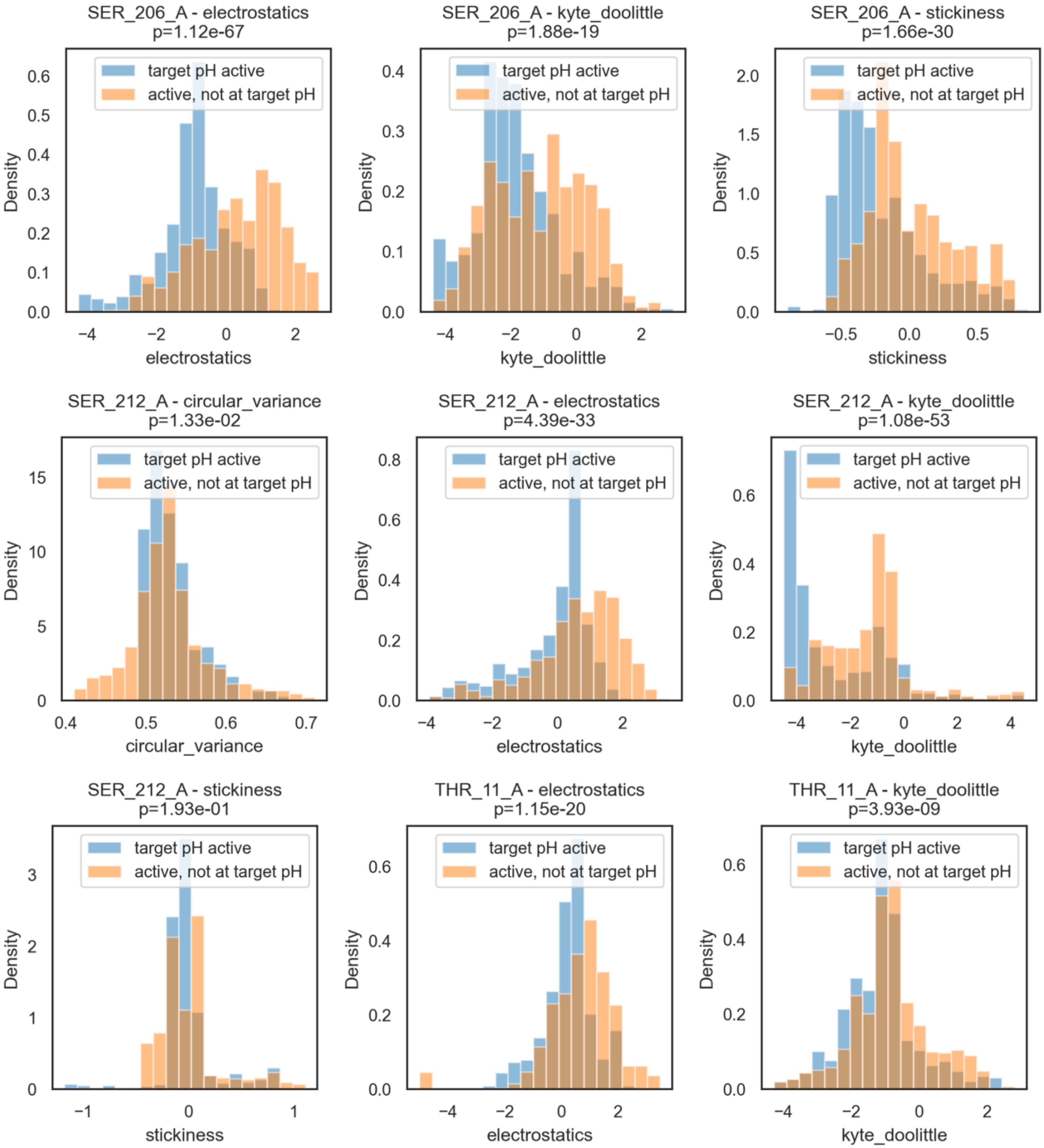
SER_206_A - electrostatics
p=1.12e-67
SER_206_A - kyte_doolittle
p=1.88e-19
SER_206_A - stickiness
p=1.66e-30
SER_212_A - circular_variance
p=1.33e-02
SER_212_A - electrostatics
p=4.39e-33
SER_212_A - kyte_doolittle
p=1.08e-53
SER_212_A - stickiness
p=1.93e-01
THR_11_A - electrostatics
p=1.15e-20
THR_11_A - kyte_doolittle
p=3.93e-09
target pH active
active, not at target pH
Density
electrostatics
kyte_doolittle
stickiness
circular_variance

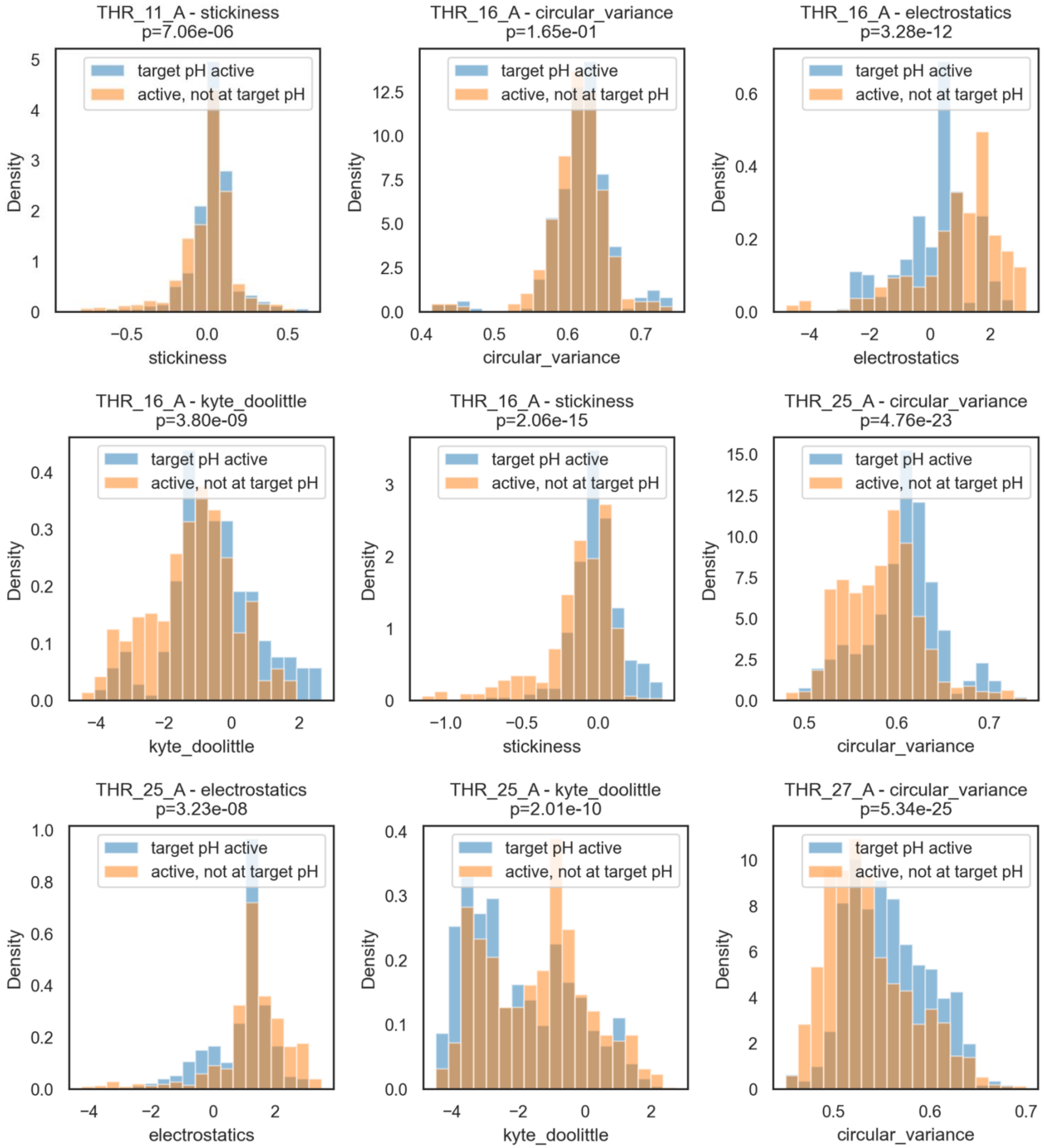
THR_11_A - stickiness
p=7.06e-06
target pH active
active, not at target pH
Density
stickiness
THR_16_A - circular_variance
p=1.65e-01
target pH active
active, not at target pH
Density
circular_variance
THR_16_A - electrostatics
p=3.28e-12
target pH active
active, not at target pH
Density
electrostatics
THR_16_A - kyte_doolittle
p=3.80e-09
target pH active
active, not at target pH
Density
kyte_doolittle
THR_16_A - stickiness
p=2.06e-15
target pH active
active, not at target pH
Density
stickiness
THR_25_A - circular_variance
p=4.76e-23
target pH active
active, not at target pH
Density
circular_variance
THR_25_A - electrostatics
p=3.23e-08
target pH active
active, not at target pH
Density
electrostatics
THR_25_A - kyte_doolittle
p=2.01e-10
target pH active
active, not at target pH
Density
kyte_doolittle
THR_27_A - circular_variance
p=5.34e-25
target pH active
active, not at target pH
Density
circular_variance

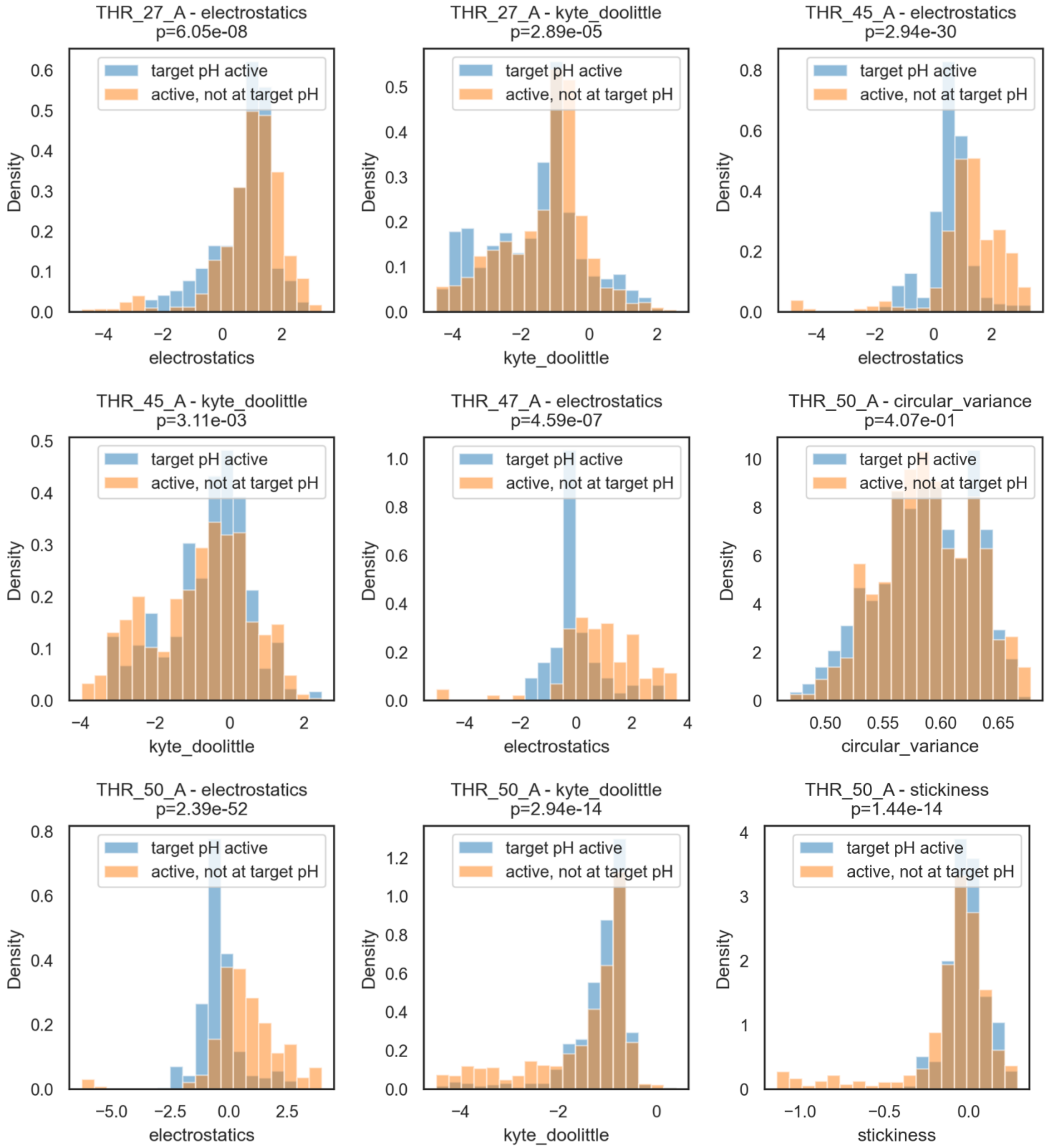
THR_27_A - electrostatics
p=6.05e-08
THR_27_A - kyte_doolittle
p=2.89e-05
THR_45_A - electrostatics
p=2.94e-30
THR_45_A - kyte_doolittle
p=3.11e-03
THR_47_A - electrostatics
p=4.59e-07
THR_50_A - circular_variance
p=4.07e-01
THR_50_A - electrostatics
p=2.39e-52
THR_50_A - kyte_doolittle
p=2.94e-14
THR_50_A - stickiness
p=1.44e-14
target pH active
active, not at target pH
Density
electrostatics
kyte_doolittle
circular_variance
stickiness

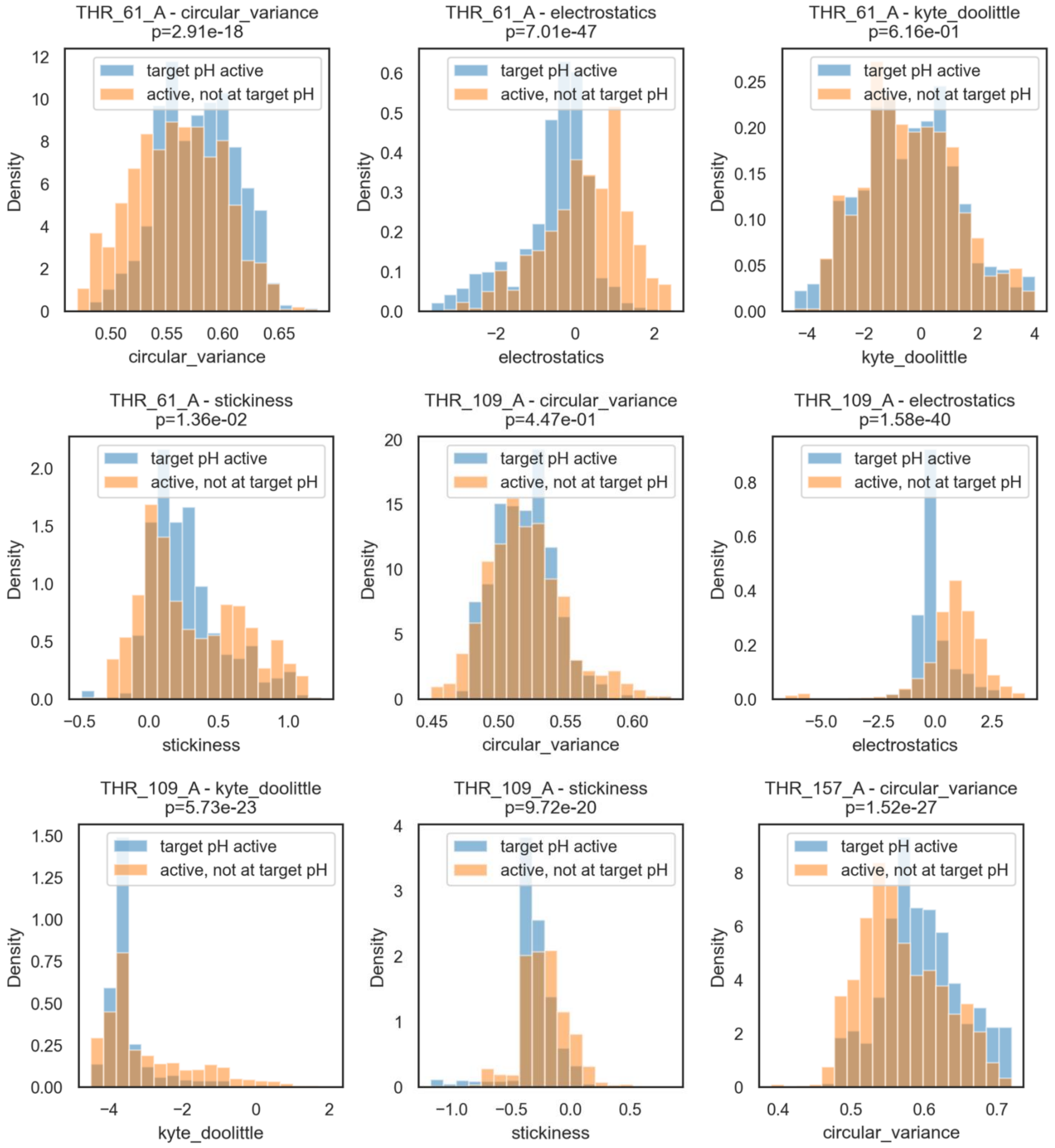
THR_61_A - circular_variance
p=2.91e-18
THR_61_A - electrostatics
p=7.01e-47
THR_61_A - kyte_doolittle
p=6.16e-01
THR_61_A - stickiness
p=1.36e-02
THR_109_A - circular_variance
p=4.47e-01
THR_109_A - electrostatics
p=1.58e-40
THR_109_A - kyte_doolittle
p=5.73e-23
THR_109_A - stickiness
p=9.72e-20
THR_157_A - circular_variance
p=1.52e-27
target pH active
active, not at target pH
Density
circular_variance
electrostatics
kyte_doolittle
stickiness

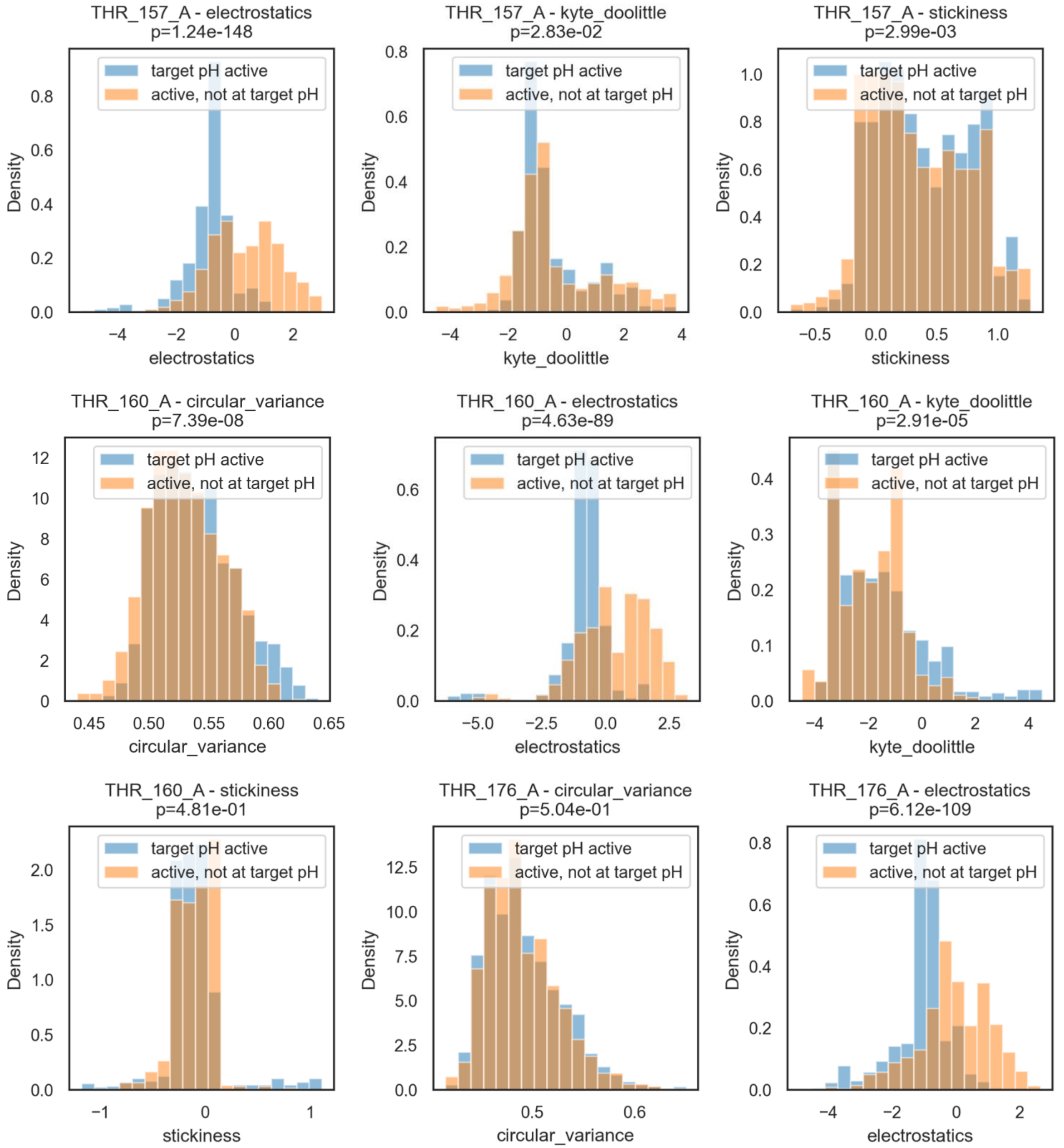
THR_157_A - electrostatics
p=1.24e-148
target pH active
active, not at target pH
Density
electrostatics
THR_157_A - kyte_doolittle
p=2.83e-02
target pH active
active, not at target pH
Density
kyte_doolittle
THR_157_A - stickiness
p=2.99e-03
target pH active
active, not at target pH
Density
stickiness
THR_160_A - circular_variance
p=7.39e-08
target pH active
active, not at target pH
Density
circular_variance
THR_160_A - electrostatics
p=4.63e-89
target pH active
active, not at target pH
Density
electrostatics
THR_160_A - kyte_doolittle
p=2.91e-05
target pH active
active, not at target pH
Density
kyte_doolittle
THR_160_A - stickiness
p=4.81e-01
target pH active
active, not at target pH
Density
stickiness
THR_176_A - circular_variance
p=5.04e-01
target pH active
active, not at target pH
Density
circular_variance
THR_176_A - electrostatics
p=6.12e-109
target pH active
active, not at target pH
Density
electrostatics

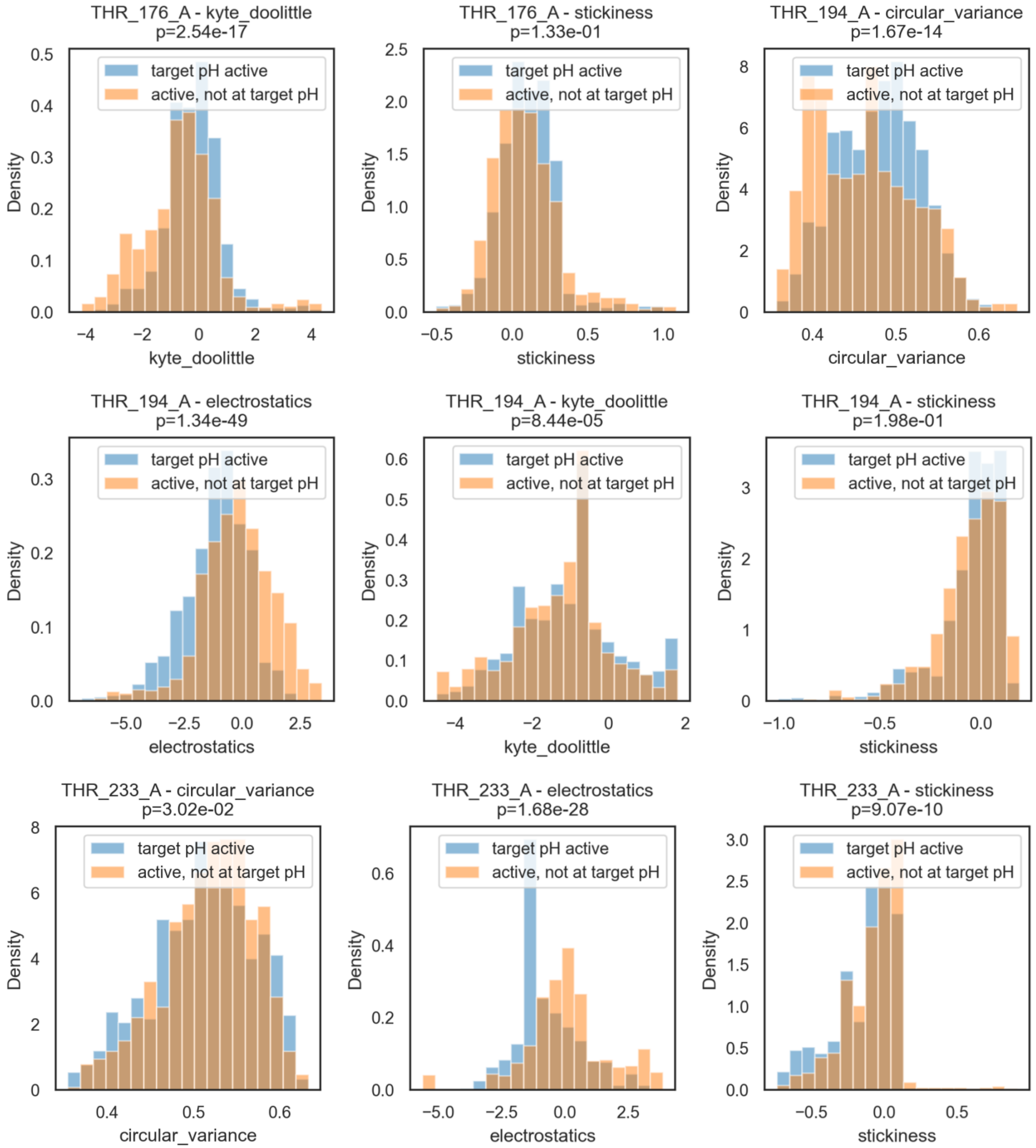
THR_176_A - kyte_doolittle
p=2.54e-17
THR_176_A - stickiness
p=1.33e-01
THR_194_A - circular_variance
p=1.67e-14
THR_194_A - electrostatics
p=1.34e-49
THR_194_A - kyte_doolittle
p=8.44e-05
THR_194_A - stickiness
p=1.98e-01
THR_233_A - circular_variance
p=3.02e-02
THR_233_A - electrostatics
p=1.68e-28
THR_233_A - stickiness
p=9.07e-10
target pH active
active, not at target pH
Density
kyte_doolittle
stickiness
circular_variance
electrostatics

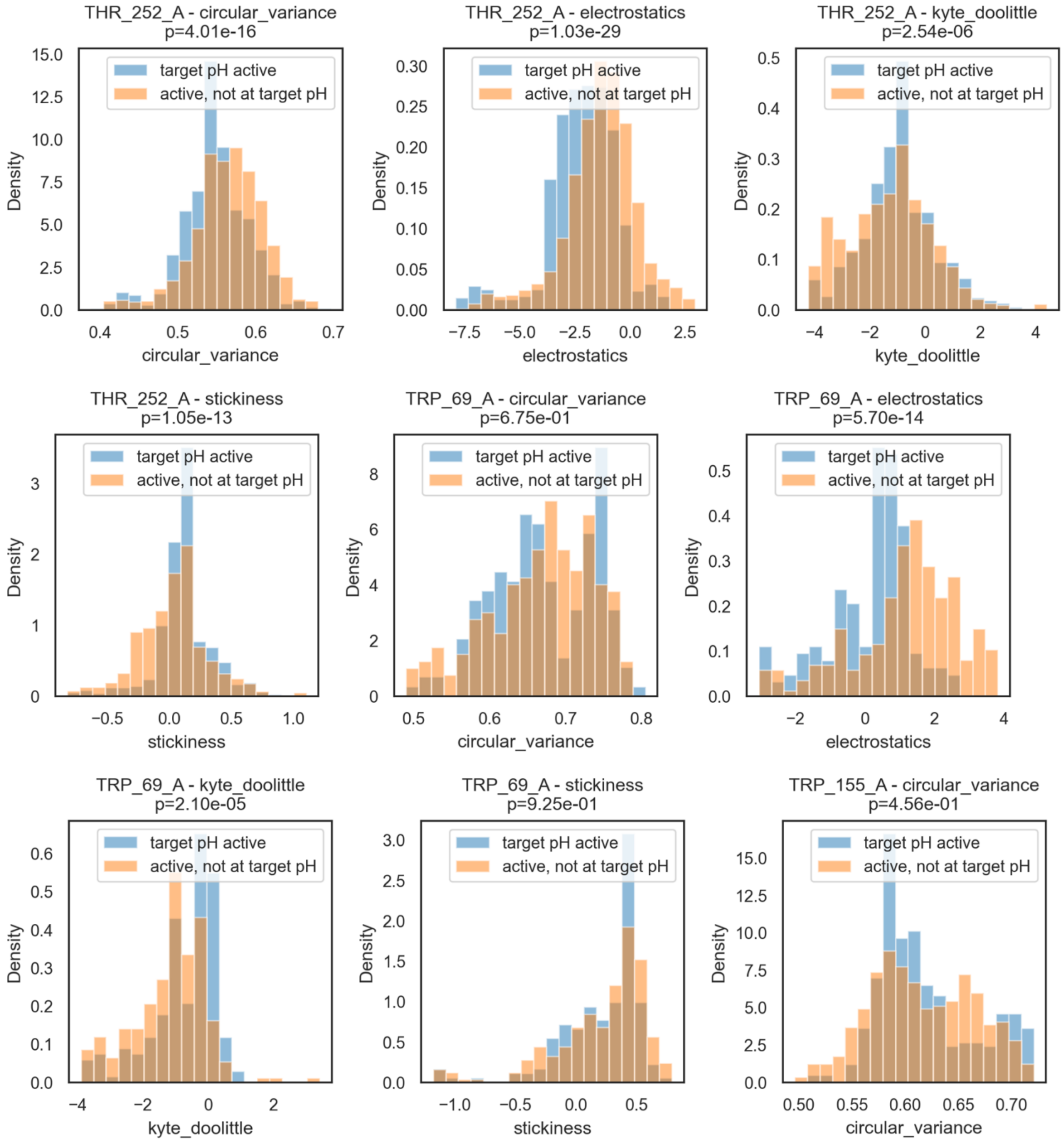
THR_252_A - circular_variance
p=4.01e-16
target pH active
active, not at target pH
Density
circular_variance
THR_252_A - electrostatics
p=1.03e-29
target pH active
active, not at target pH
Density
electrostatics
THR_252_A - kyte_doolittle
p=2.54e-06
target pH active
active, not at target pH
Density
kyte_doolittle
THR_252_A - stickiness
p=1.05e-13
target pH active
active, not at target pH
Density
stickiness
TRP_69_A - circular_variance
p=6.75e-01
target pH active
active, not at target pH
Density
circular_variance
TRP_69_A - electrostatics
p=5.70e-14
target pH active
active, not at target pH
Density
electrostatics
TRP_69_A - kyte_doolittle
p=2.10e-05
target pH active
active, not at target pH
Density
kyte_doolittle
TRP_69_A - stickiness
p=9.25e-01
target pH active
active, not at target pH
Density
stickiness
TRP_155_A - circular_variance
p=4.56e-01
target pH active
active, not at target pH
Density
circular_variance

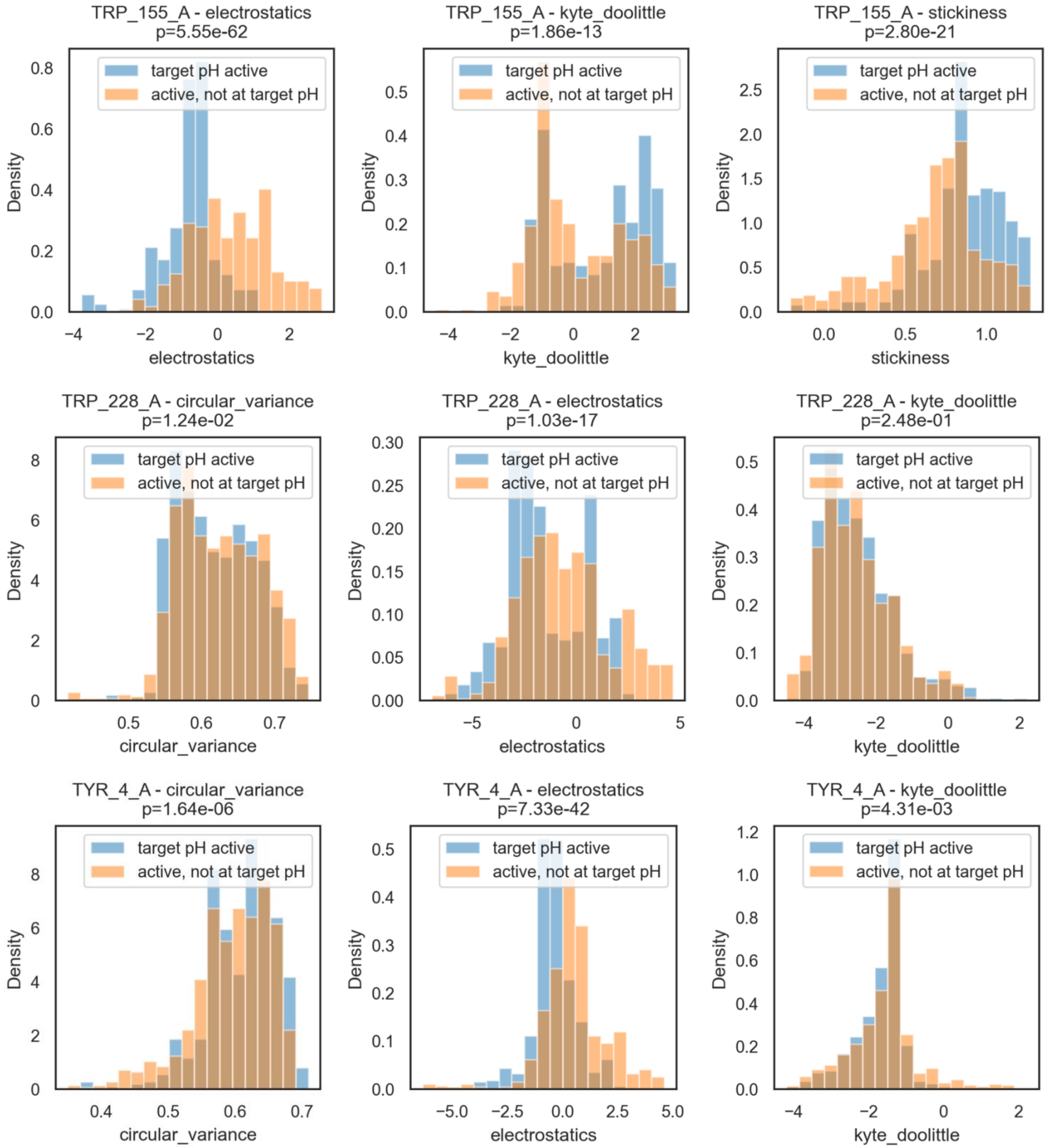
TRP_155_A - electrostatics
p=5.55e-62
target pH active
active, not at target pH
Density
electrostatics
TRP_155_A - kyte_doolittle
p=1.86e-13
kyte_doolittle
TRP_155_A - stickiness
p=2.80e-21
stickiness
TRP_228_A - circular_variance
p=1.24e-02
circular_variance
TRP_228_A - electrostatics
p=1.03e-17
TRP_228_A - kyte_doolittle
p=2.48e-01
TYR_4_A - circular_variance
p=1.64e-06
TYR_4_A - electrostatics
p=7.33e-42
TYR_4_A - kyte_doolittle
p=4.31e-03

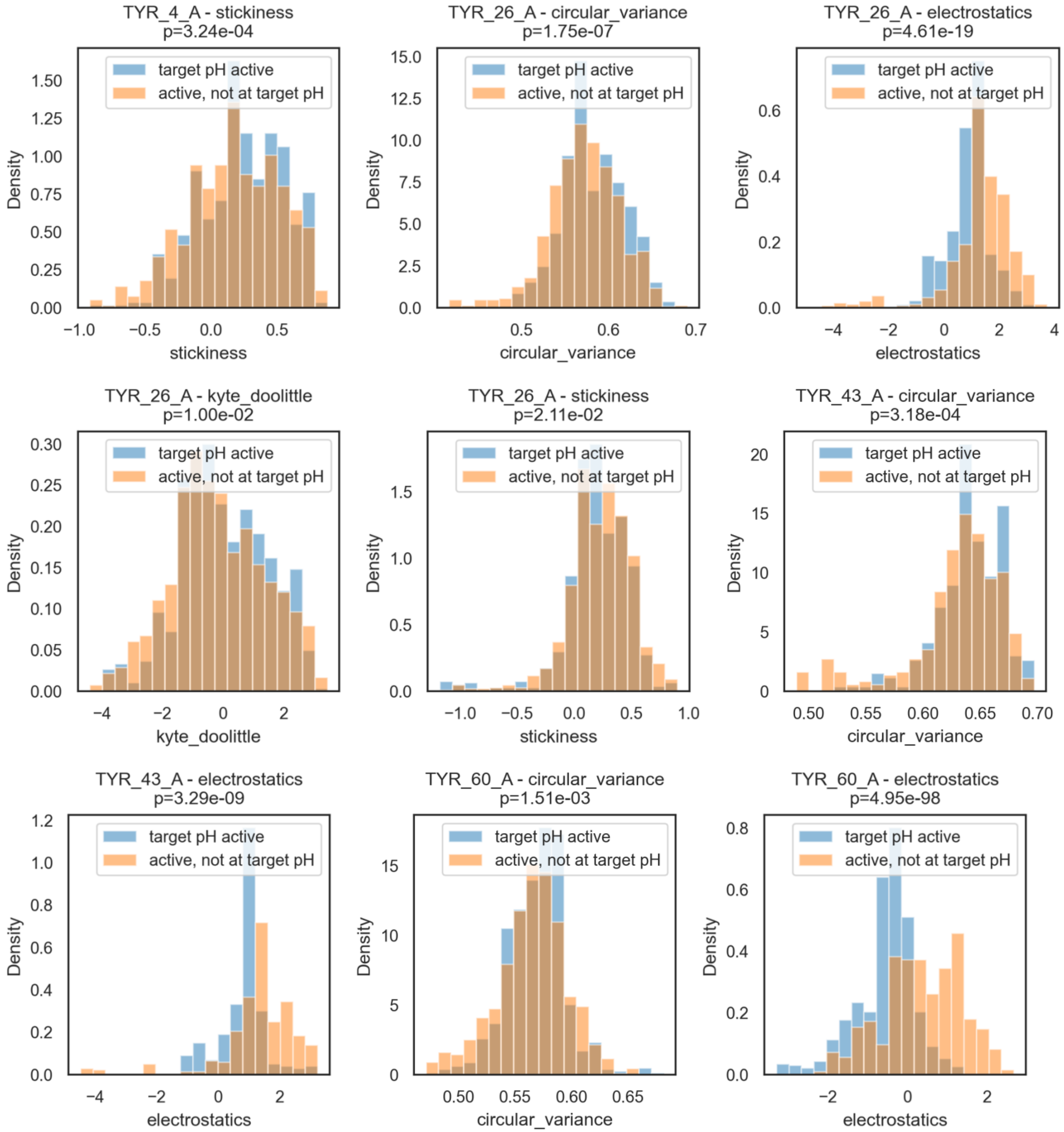
TYR_4_A - stickiness
p=3.24e-04
target pH active
active, not at target pH
Density
stickiness
TYR_26_A - circular_variance
p=1.75e-07
target pH active
active, not at target pH
Density
circular_variance
TYR_26_A - electrostatics
p=4.61e-19
target pH active
active, not at target pH
Density
electrostatics
TYR_26_A - kyte_doolittle
p=1.00e-02
target pH active
active, not at target pH
Density
kyte_doolittle
TYR_26_A - stickiness
p=2.11e-02
target pH active
active, not at target pH
Density
stickiness
TYR_43_A - circular_variance
p=3.18e-04
target pH active
active, not at target pH
Density
circular_variance
TYR_43_A - electrostatics
p=3.29e-09
target pH active
active, not at target pH
Density
electrostatics
TYR_60_A - circular_variance
p=1.51e-03
target pH active
active, not at target pH
Density
circular_variance
TYR_60_A - electrostatics
p=4.95e-98
target pH active
active, not at target pH
Density
electrostatics

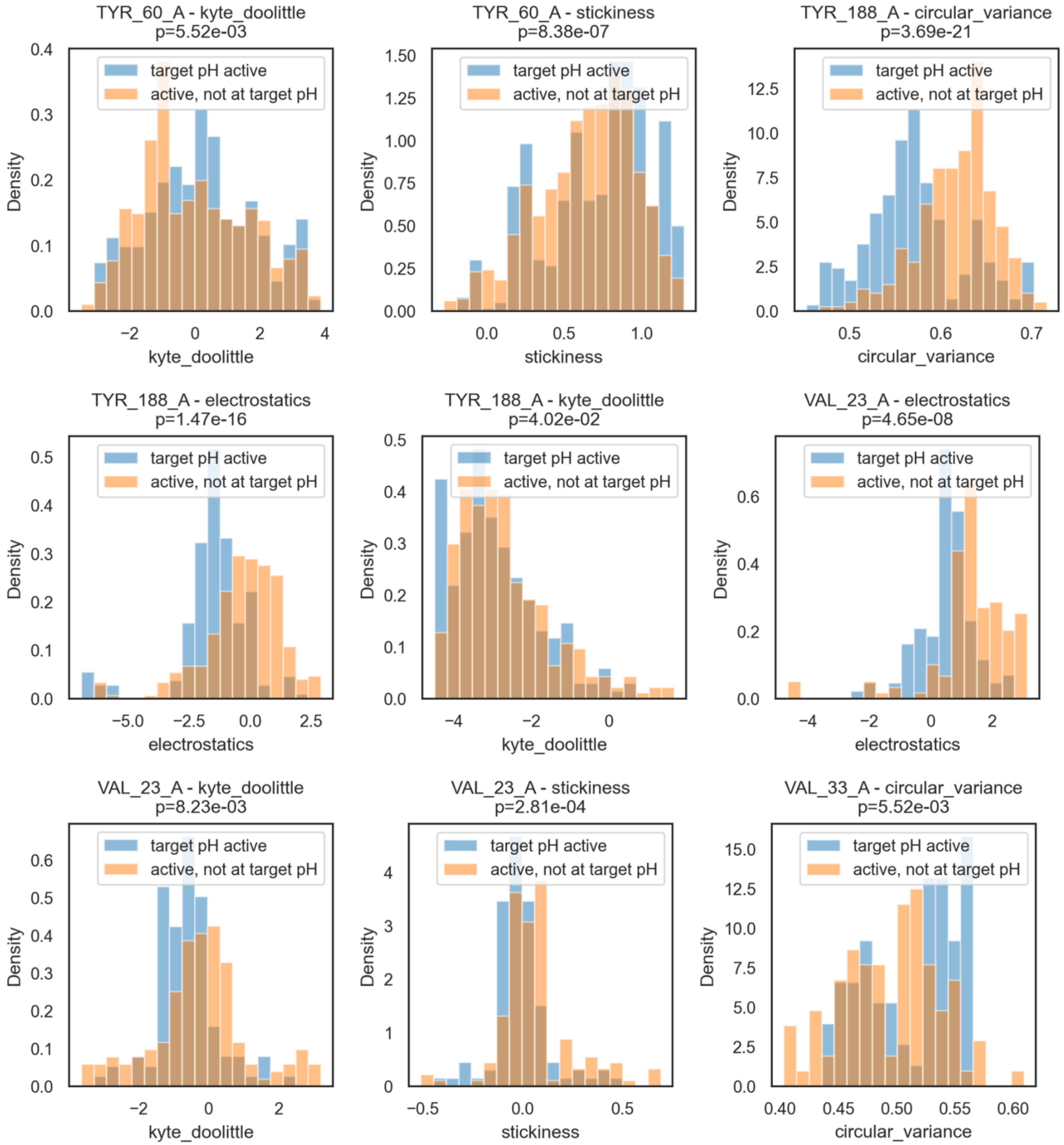
TYR_60_A - kyte_doolittle
p=5.52e-03
TYR_60_A - stickiness
p=8.38e-07
TYR_188_A - circular_variance
p=3.69e-21
TYR_188_A - electrostatics
p=1.47e-16
TYR_188_A - kyte_doolittle
p=4.02e-02
VAL_23_A - electrostatics
p=4.65e-08
VAL_23_A - kyte_doolittle
p=8.23e-03
VAL_23_A - stickiness
p=2.81e-04
VAL_33_A - circular_variance
p=5.52e-03
target pH active
active, not at target pH
Density
kyte_doolittle
stickiness
circular_variance
electrostatics

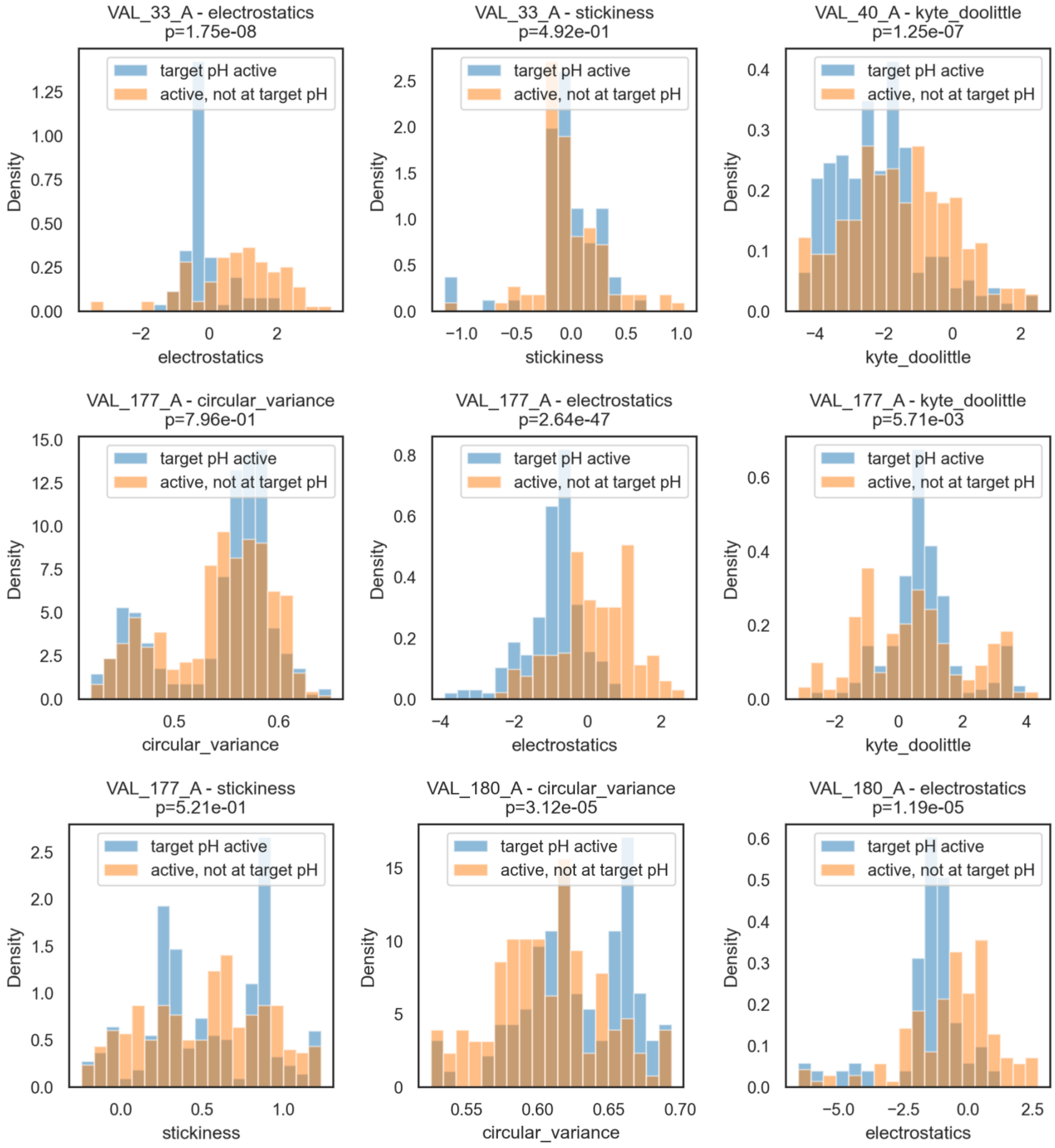
VAL_33_A - electrostatics
p=1.75e-08
target pH active
active, not at target pH
Density
electrostatics
VAL_33_A - stickiness
p=4.92e-01
target pH active
active, not at target pH
Density
stickiness
VAL_40_A - kyte_doolittle
p=1.25e-07
target pH active
active, not at target pH
Density
kyte_doolittle
VAL_177_A - circular_variance
p=7.96e-01
target pH active
active, not at target pH
Density
circular_variance
VAL_177_A - electrostatics
p=2.64e-47
target pH active
active, not at target pH
Density
electrostatics
VAL_177_A - kyte_doolittle
p=5.71e-03
target pH active
active, not at target pH
Density
kyte_doolittle
VAL_177_A - stickiness
p=5.21e-01
target pH active
active, not at target pH
Density
stickiness
VAL_180_A - circular_variance
p=3.12e-05
target pH active
active, not at target pH
Density
circular_variance
VAL_180_A - electrostatics
p=1.19e-05
target pH active
active, not at target pH
Density
electrostatics
