# Supplementary material for: Machine Learning-Guided Identification of PET Hydrolases from Natural Diversity
Source: ACS Catal. 2025 Sep 3;15(18):16070–83. doi: 10.1021/acscatal.5c03460 (PMC12455559; doi:10.1021/acscatal.5c03460)
Supplement: Supplementary file 5 [file cs5c03460_si_005.pdf]

Difference, acid tolerant (positive) mean, and neutral active (negative) mean for significant factors, mapped to LCC structure

Created on: 2025-04-15 16:11:38

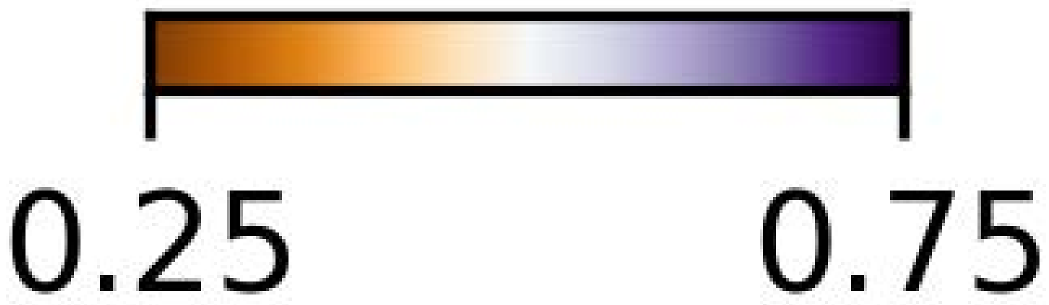

ph_circular_variance_0_legend.png

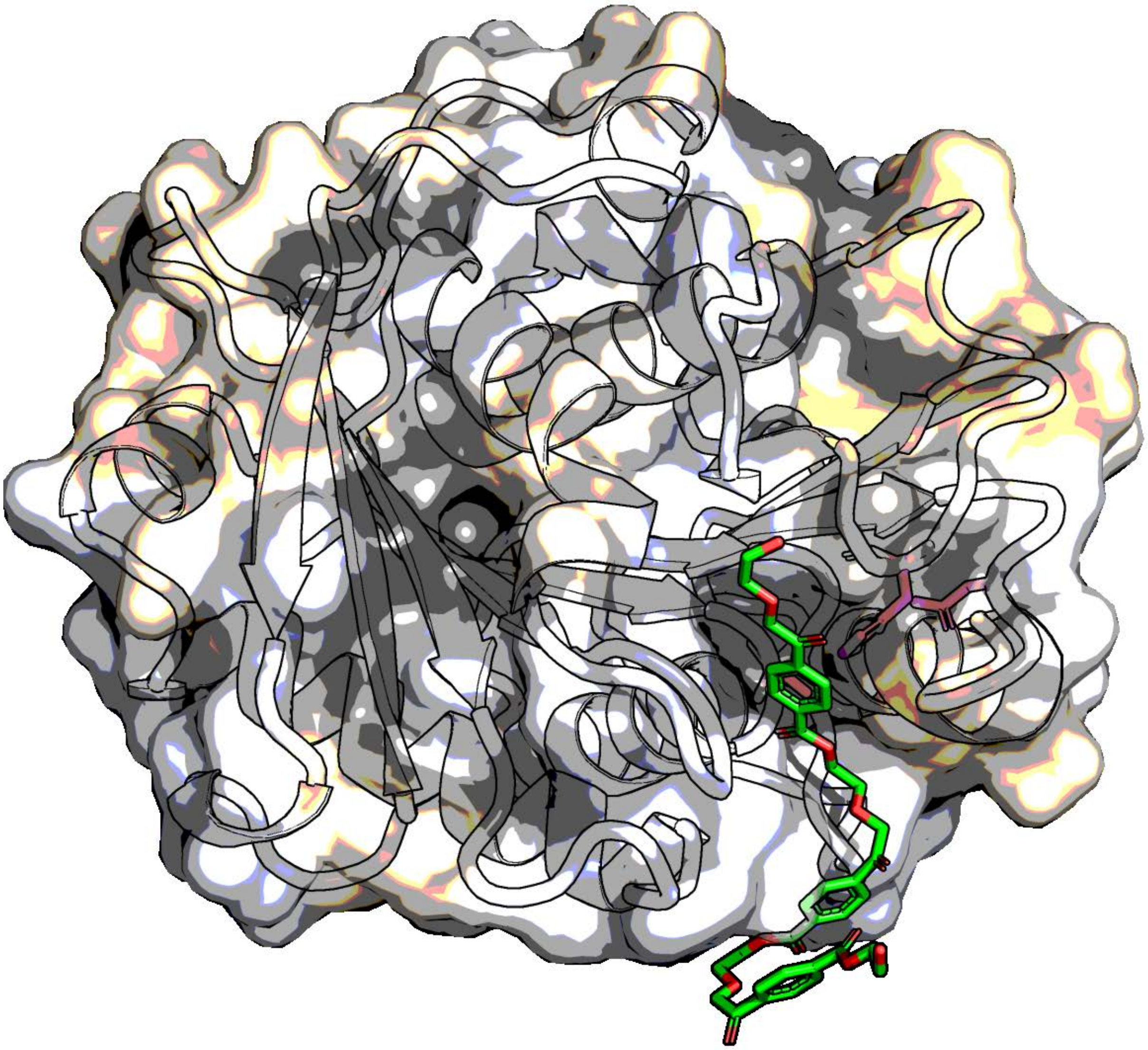

ph_circular_variance_difference_LCC-ICCG.png

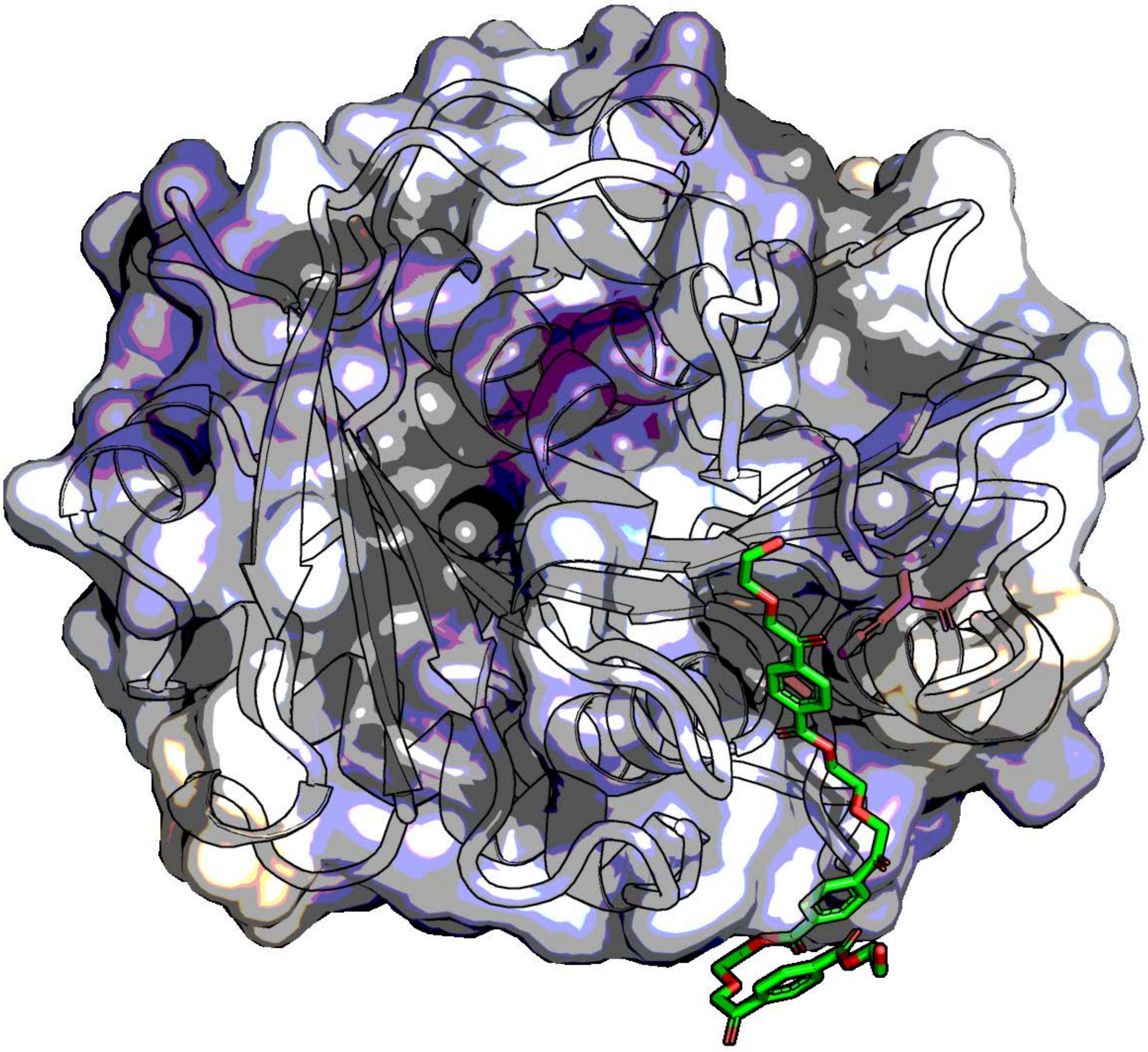

ph_circular_variance_negative_mean_LCC-ICCG.png

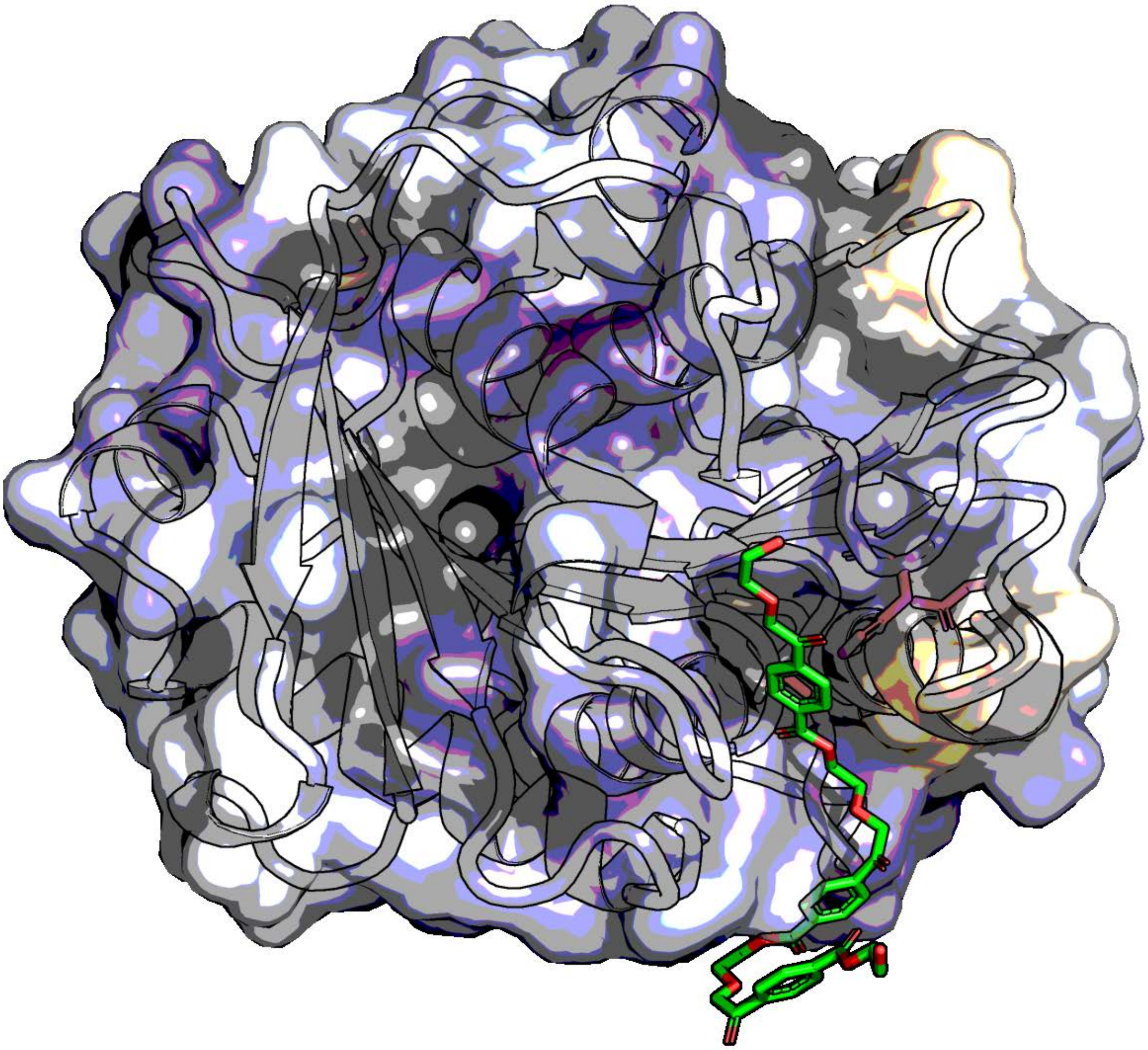

ph_circular_variance_positive_mean_LCC-ICCG.png

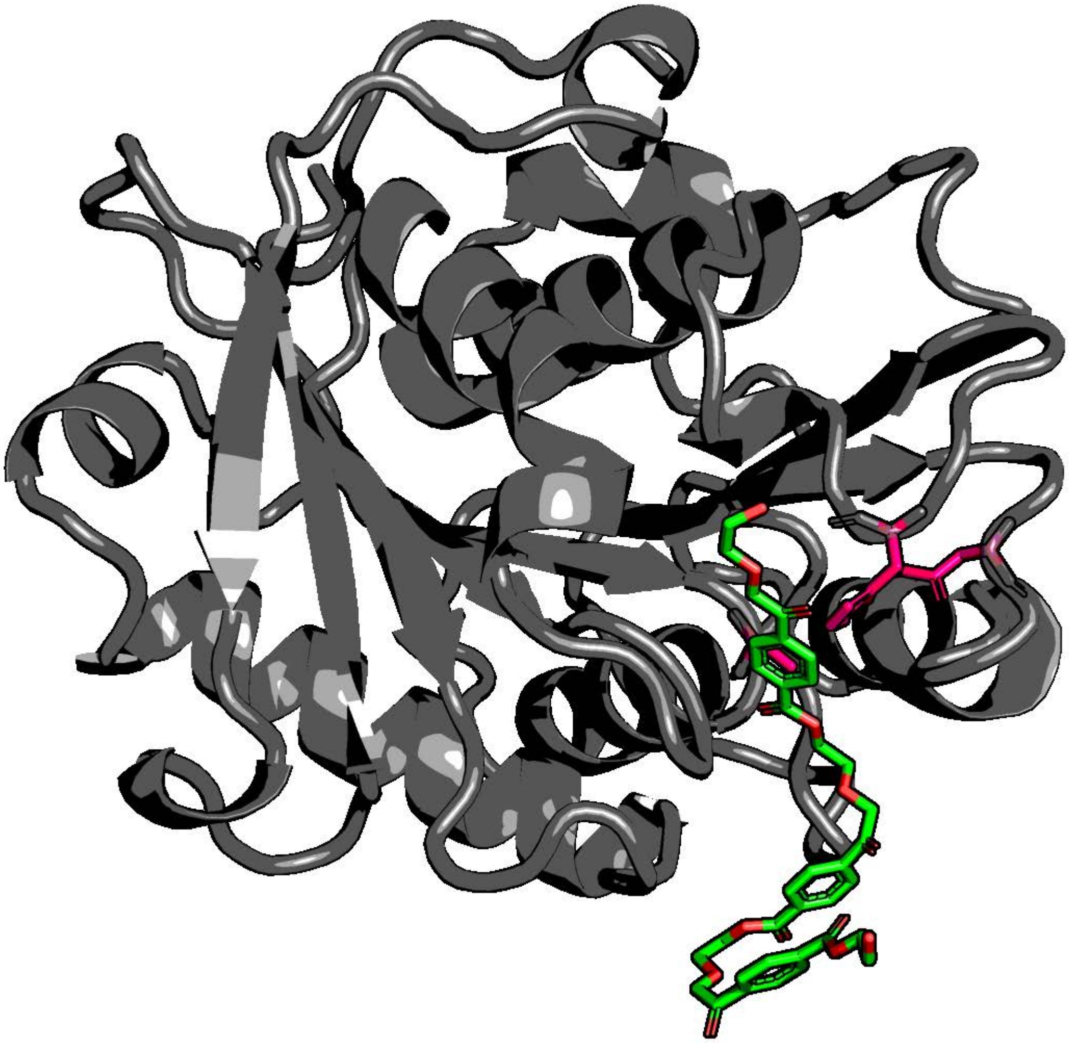

ph_conservation_negative_mean_LCC-ICCG.png

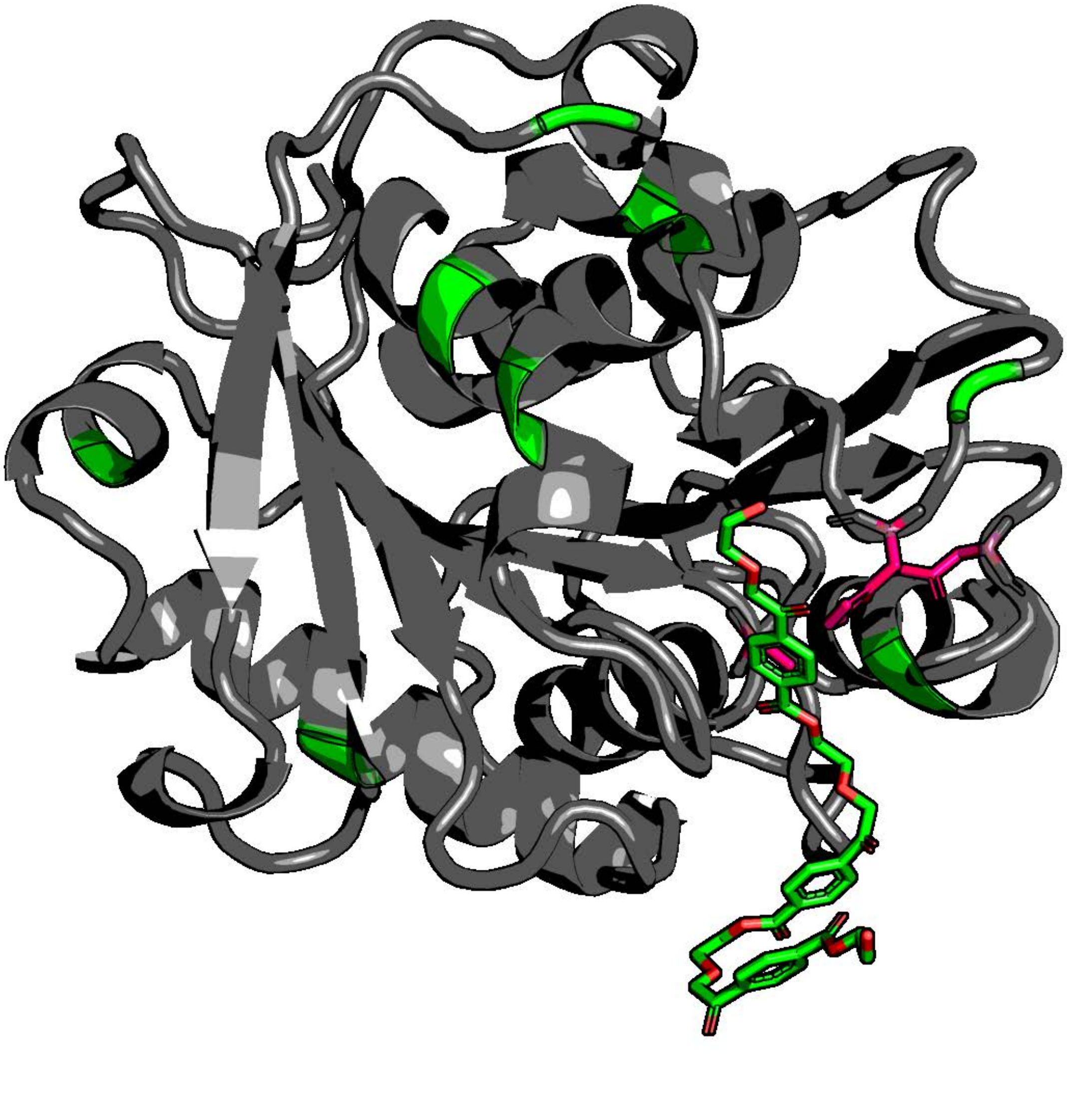

ph_conservation_positive_mean_LCC-ICCG.png

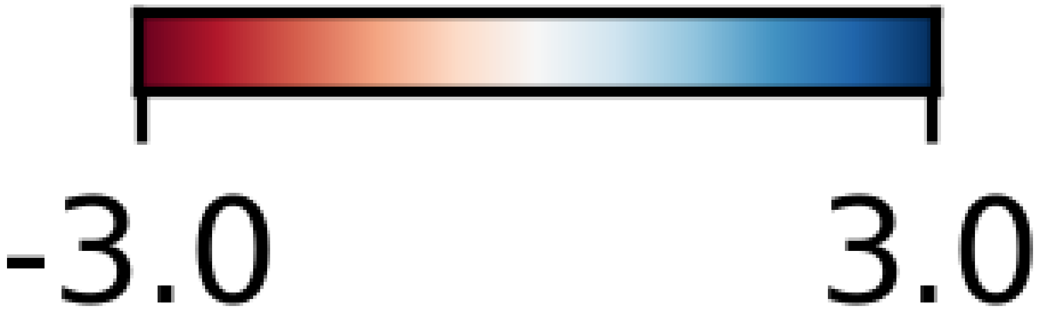

ph_electrostatics_0_legend.png

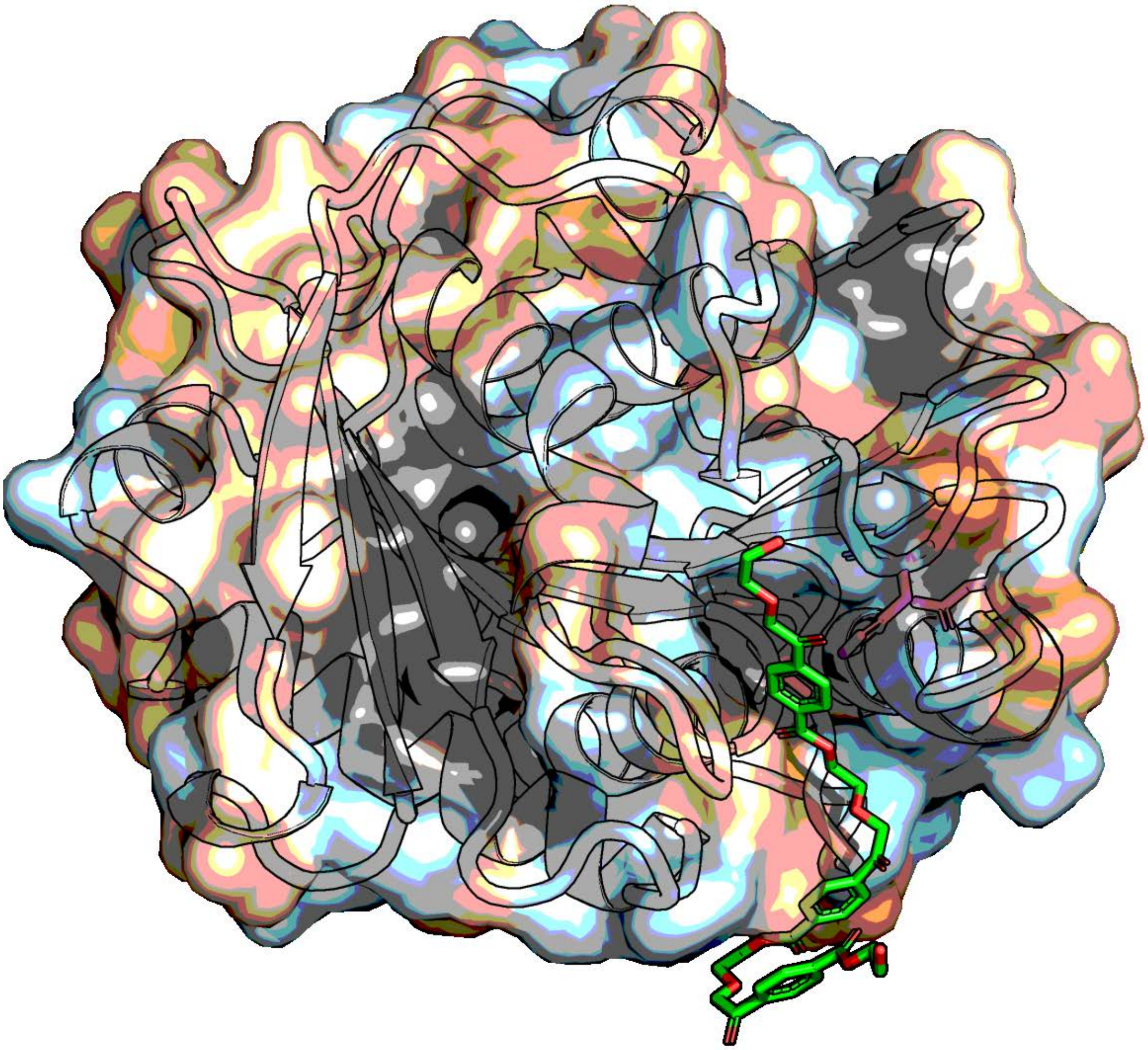

ph_electrostatics_difference_LCC-ICCG.png

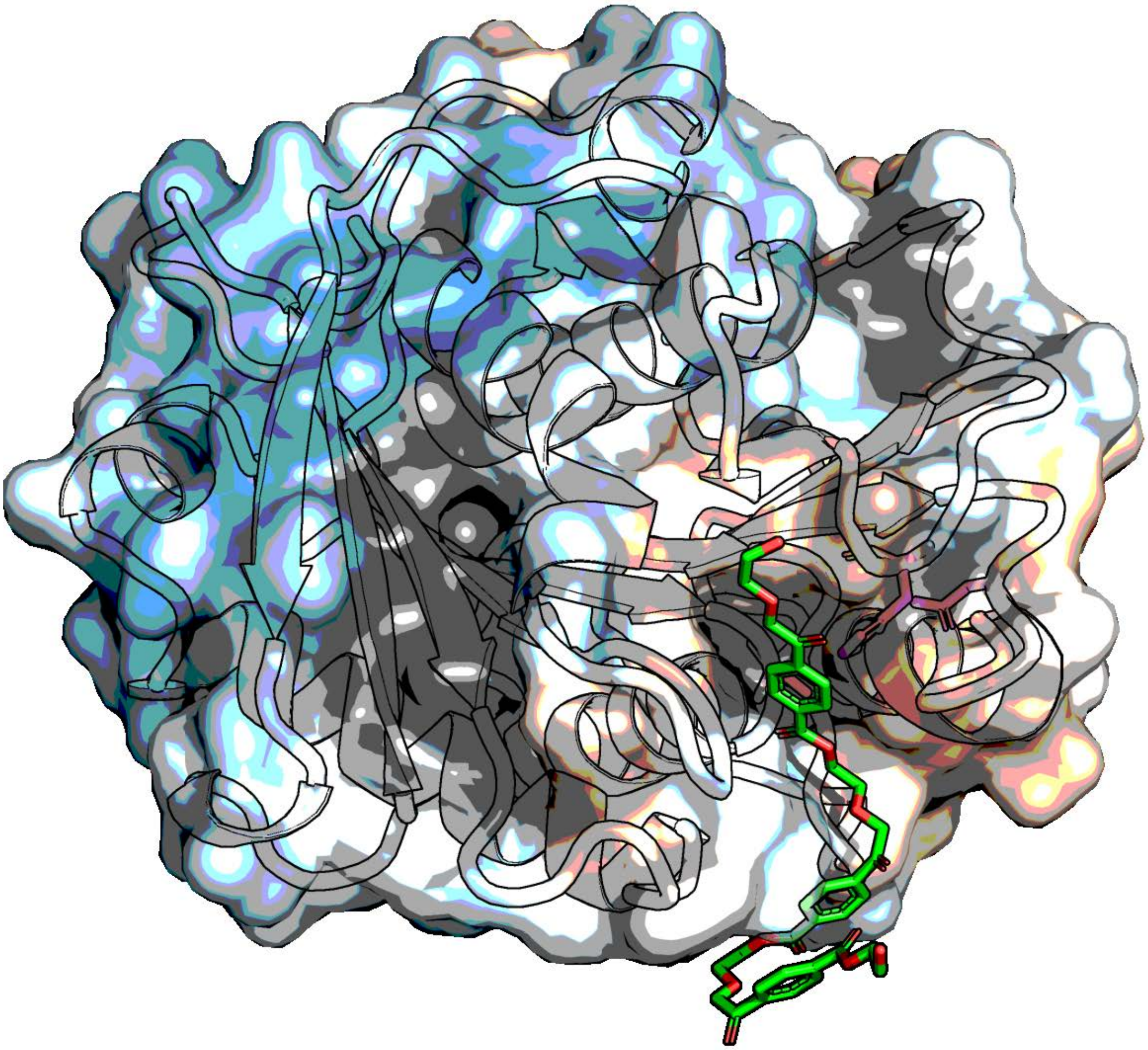

ph_electrostatics_negative_mean_LCC-ICCG.png

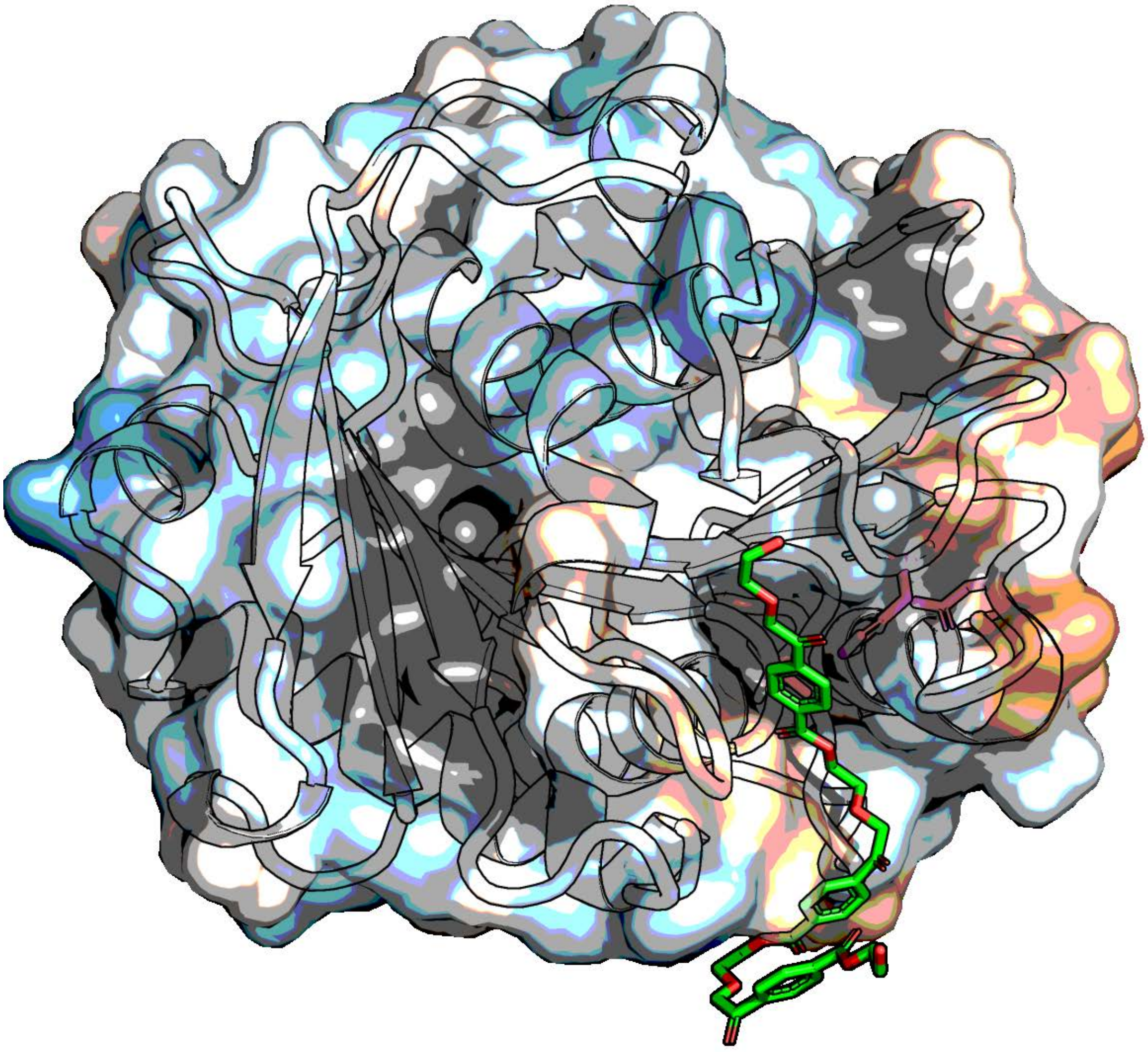

ph_electrostatics_positive_mean_LCC-ICCG.png

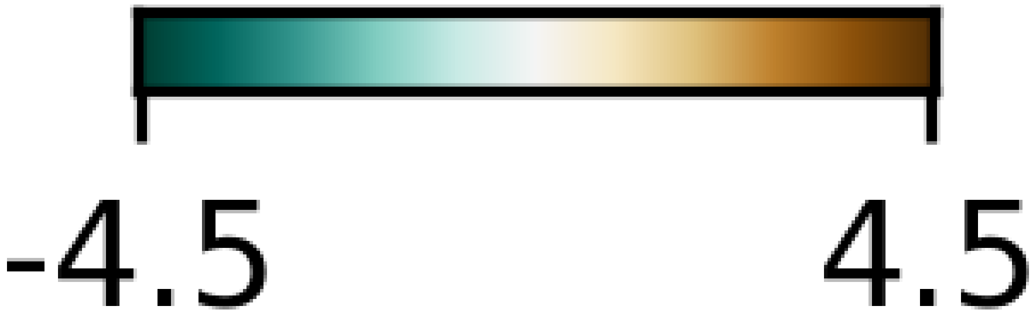

ph_kyte_doolittle_0_legend.png

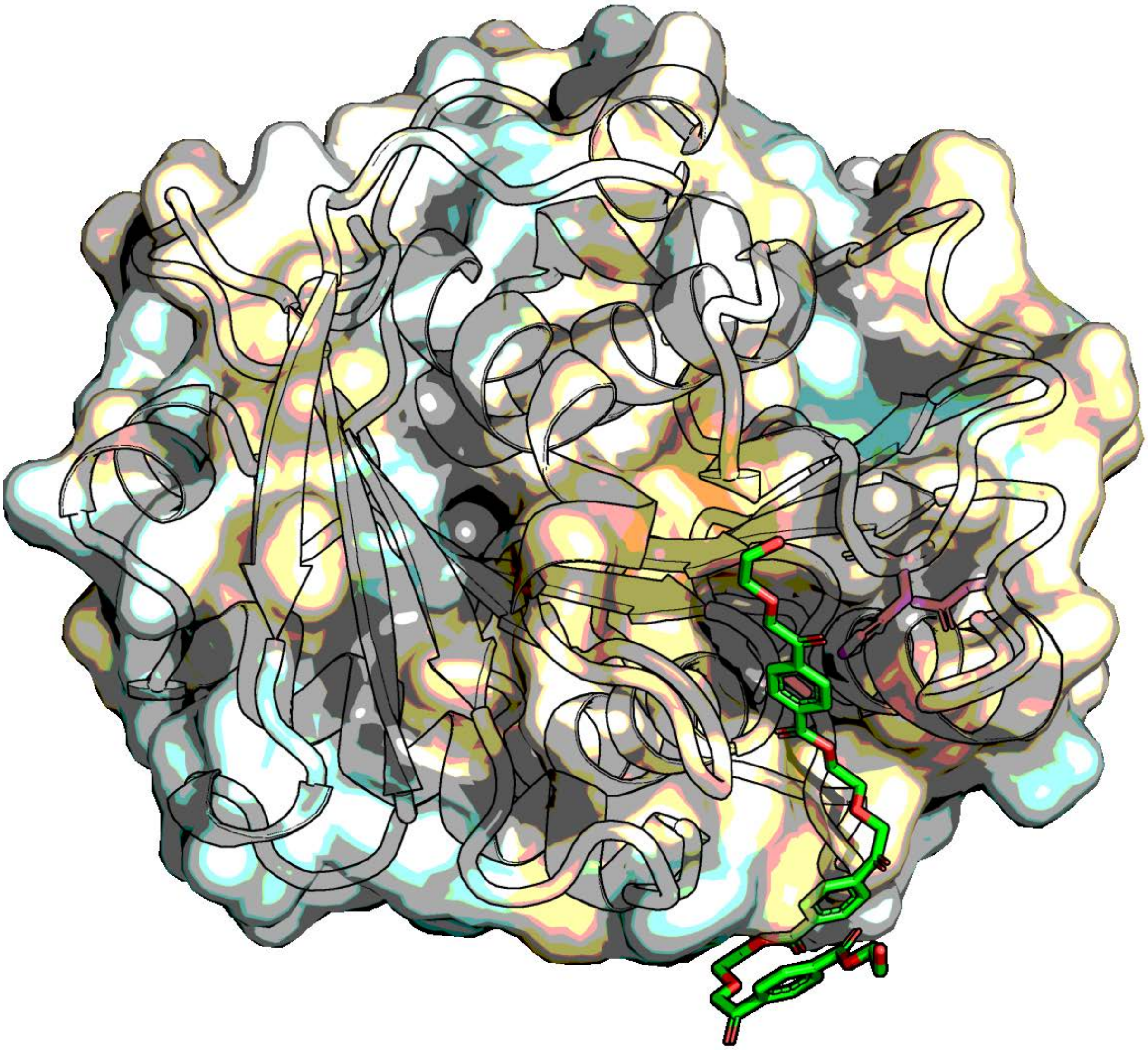

ph_kyte_doolittle_difference_LCC-ICCG.png

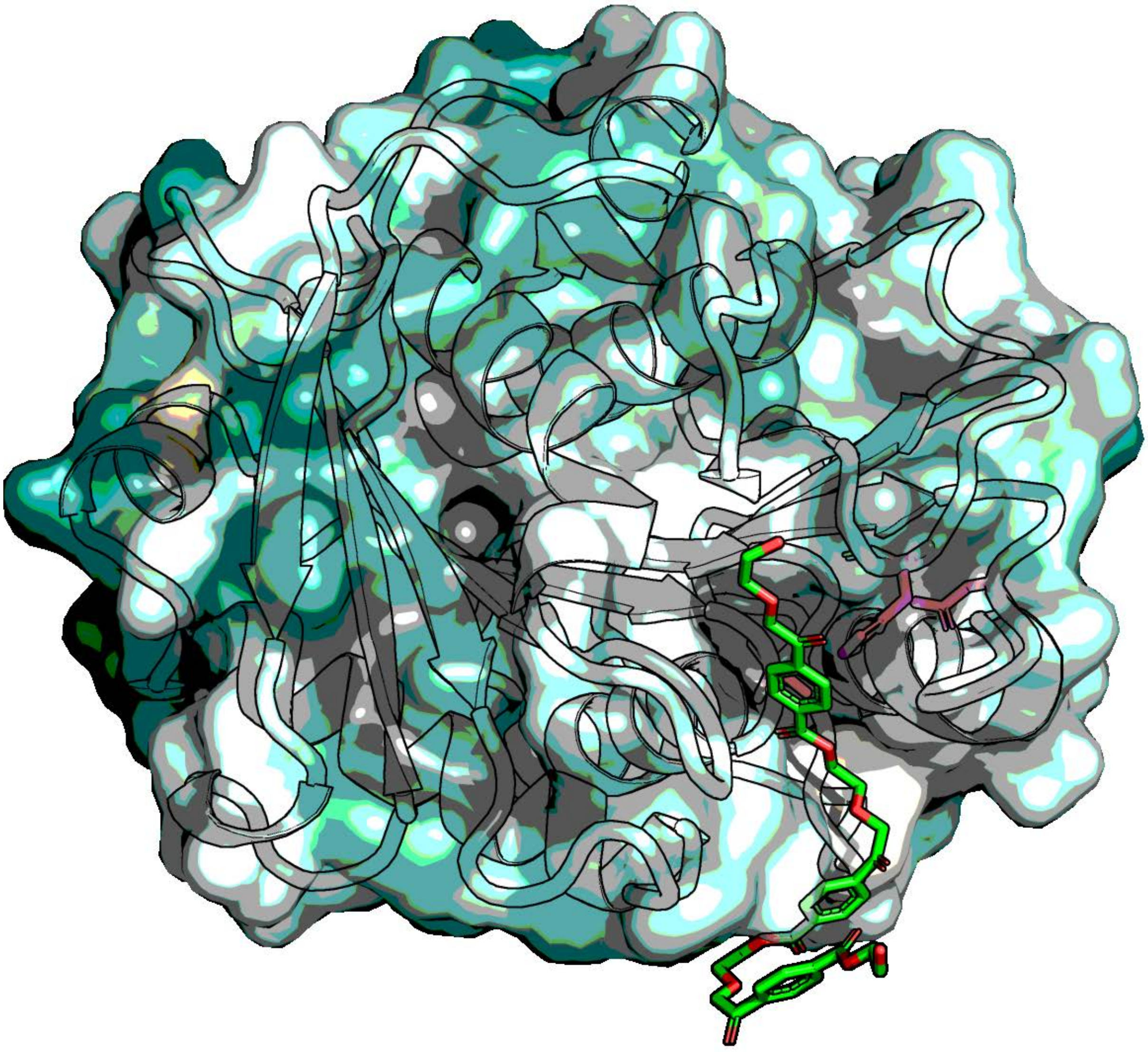

ph_kyte_doolittle_negative_mean_LCC-ICCG.png

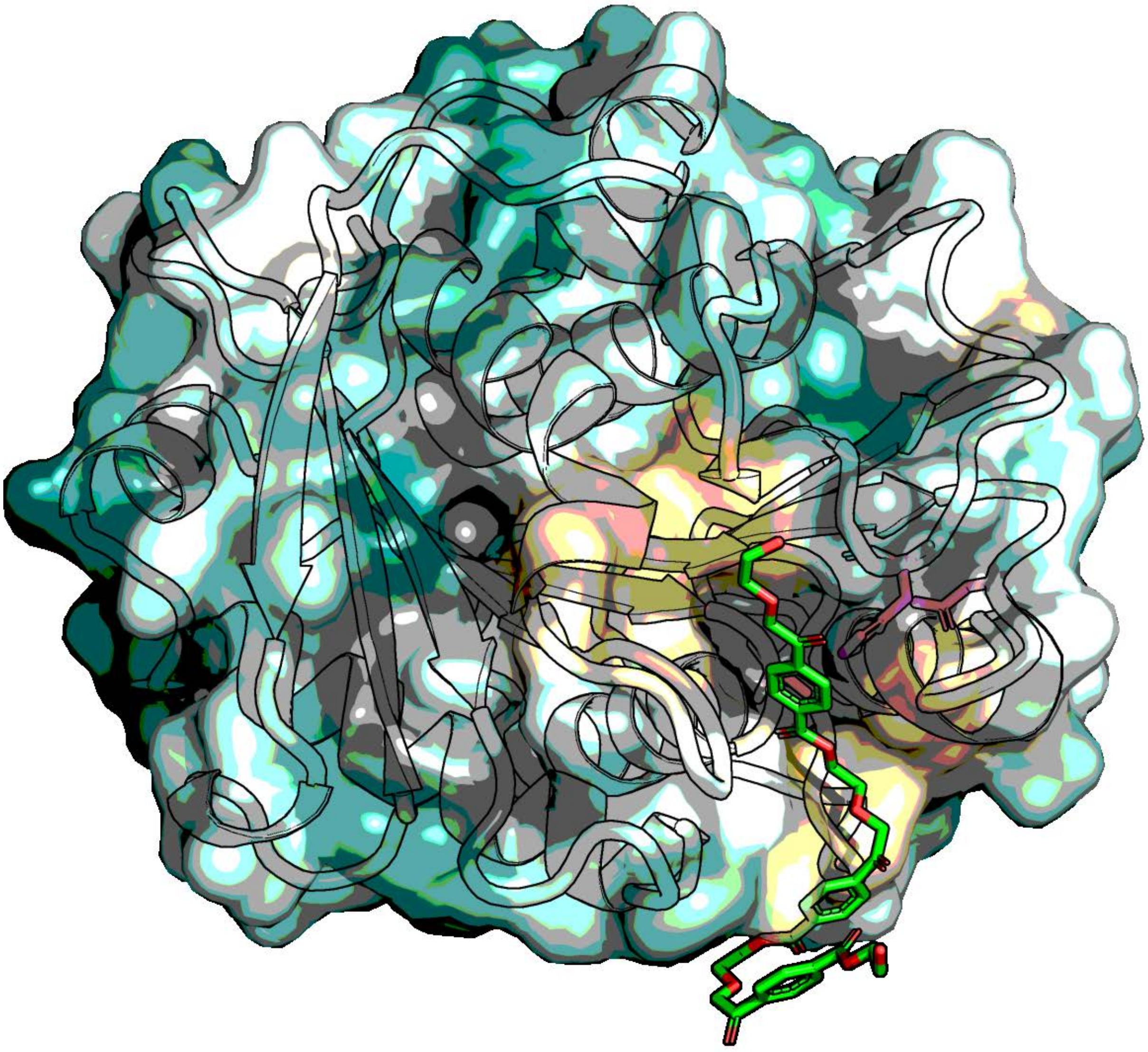

ph_kyte_doolittle_positive_mean_LCC-ICCG.png

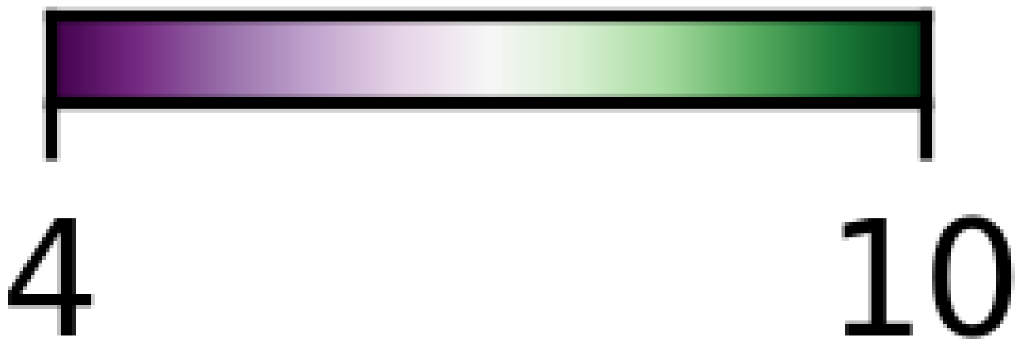

ph_pka_0_legend.png

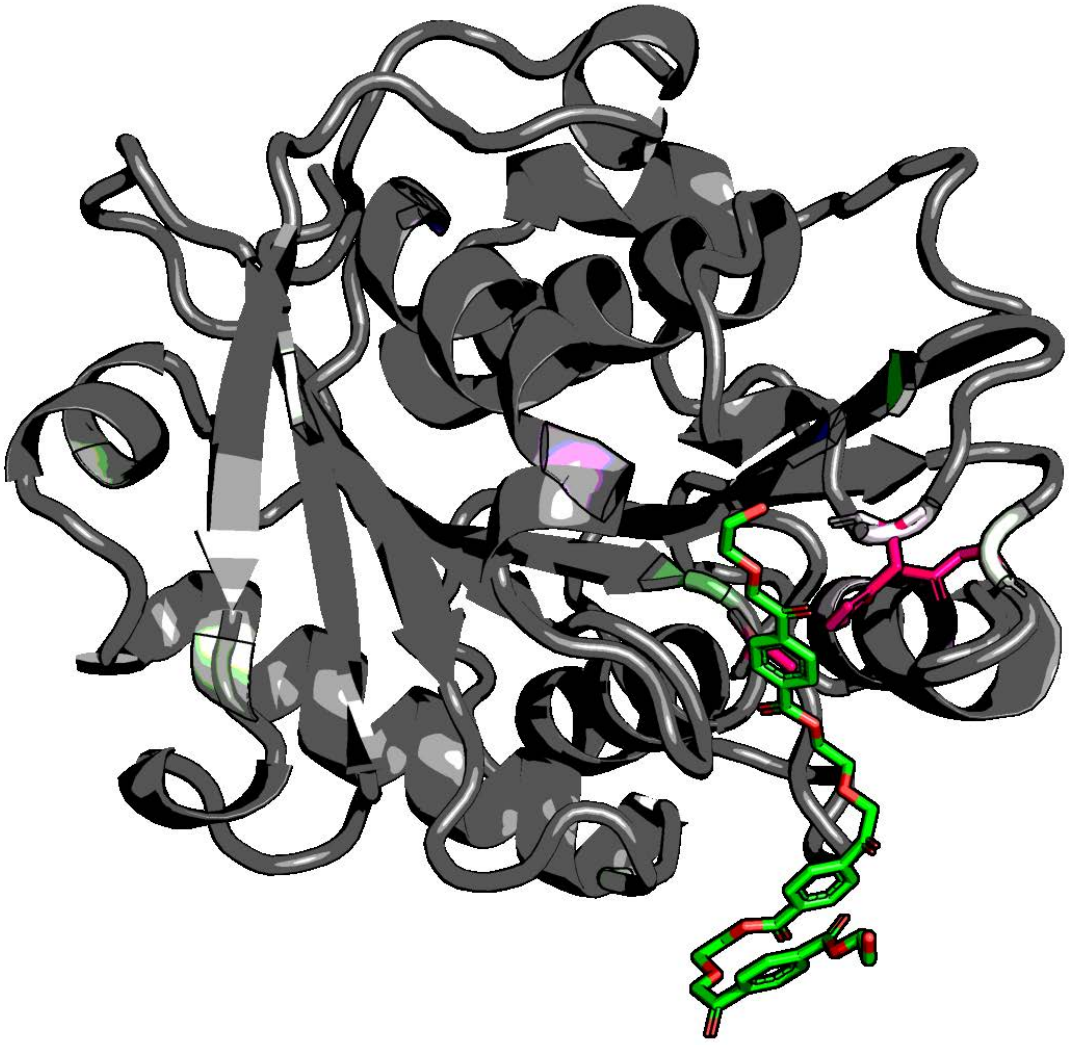

ph_pka_difference_LCC-ICCG.png

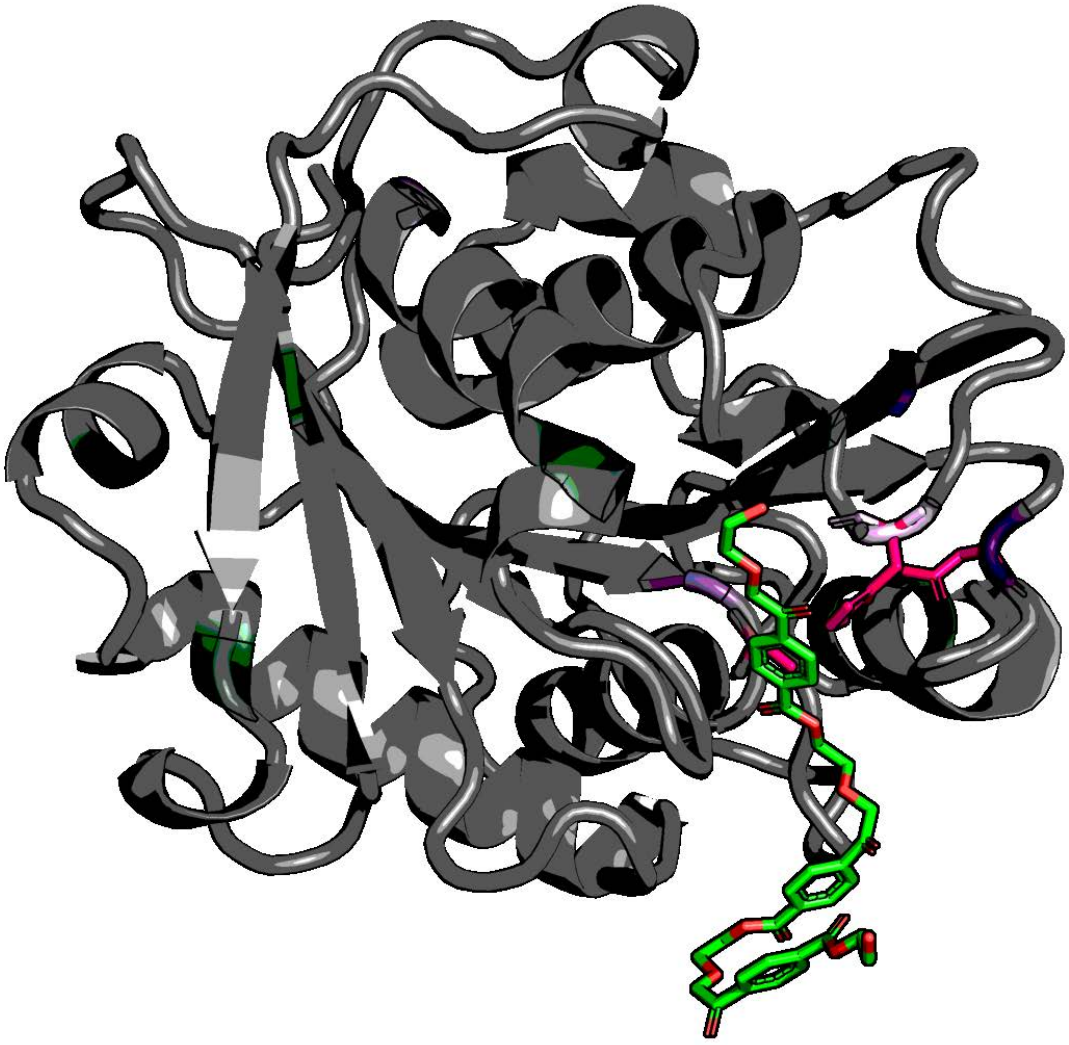

ph_pka_negative_mean_LCC-ICCG.png

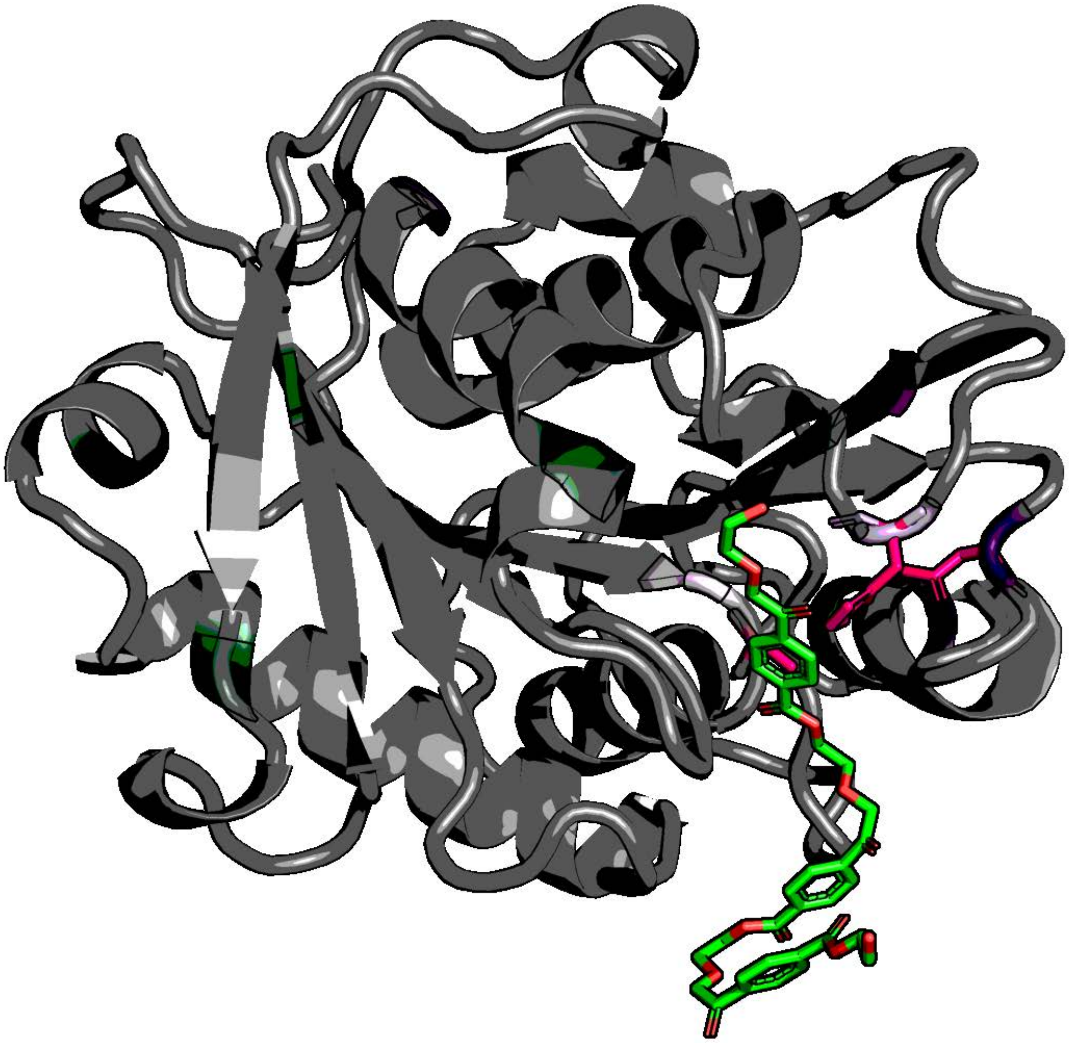

ph_pka_positive_mean_LCC-ICCG.png

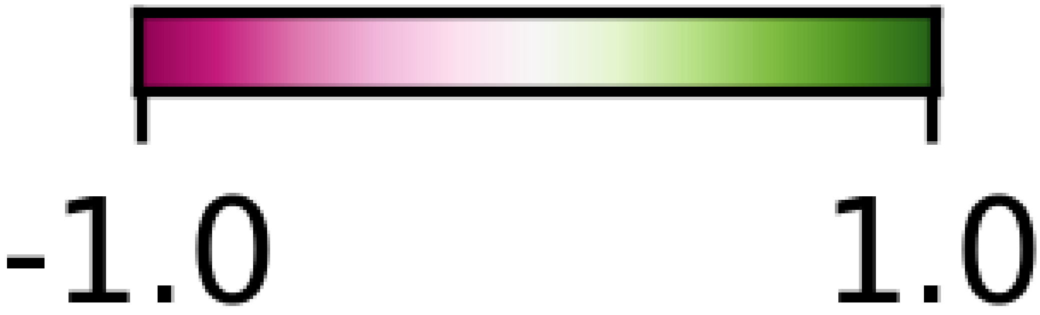

ph_stickiness_0_legend.png

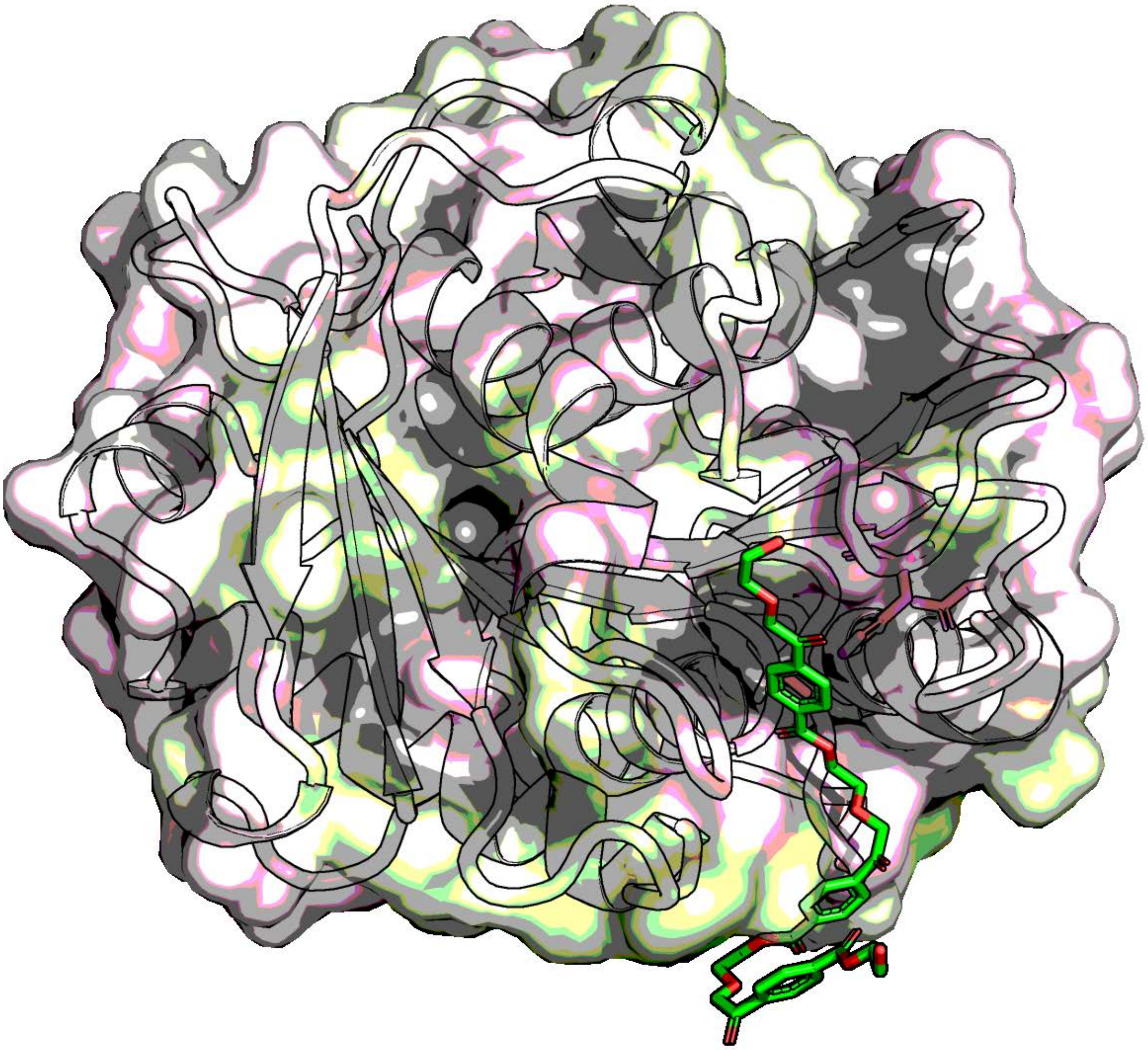

ph_stickiness_difference_LCC-ICCG.png

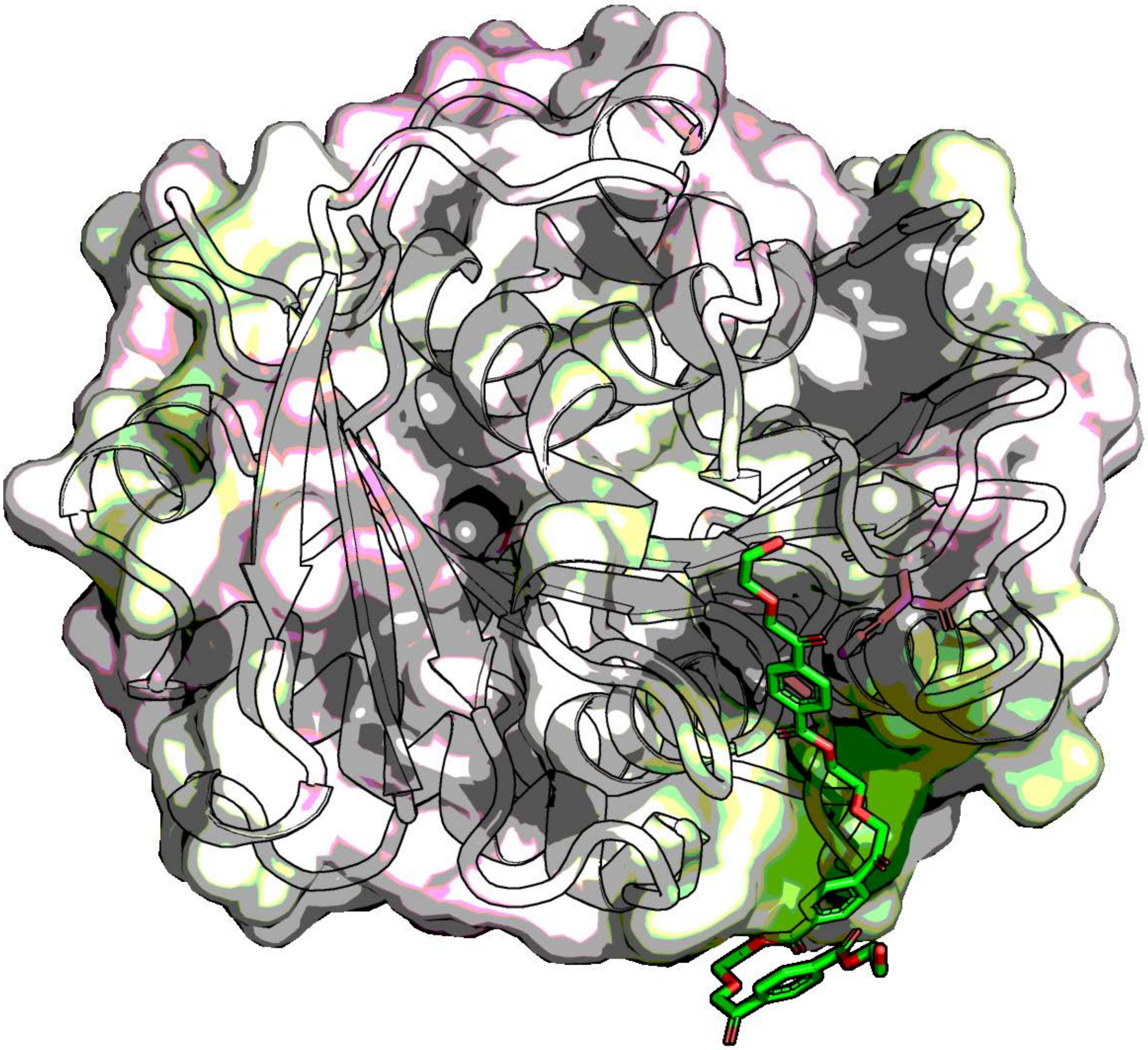

ph_stickiness_negative_mean_LCC-ICCG.png

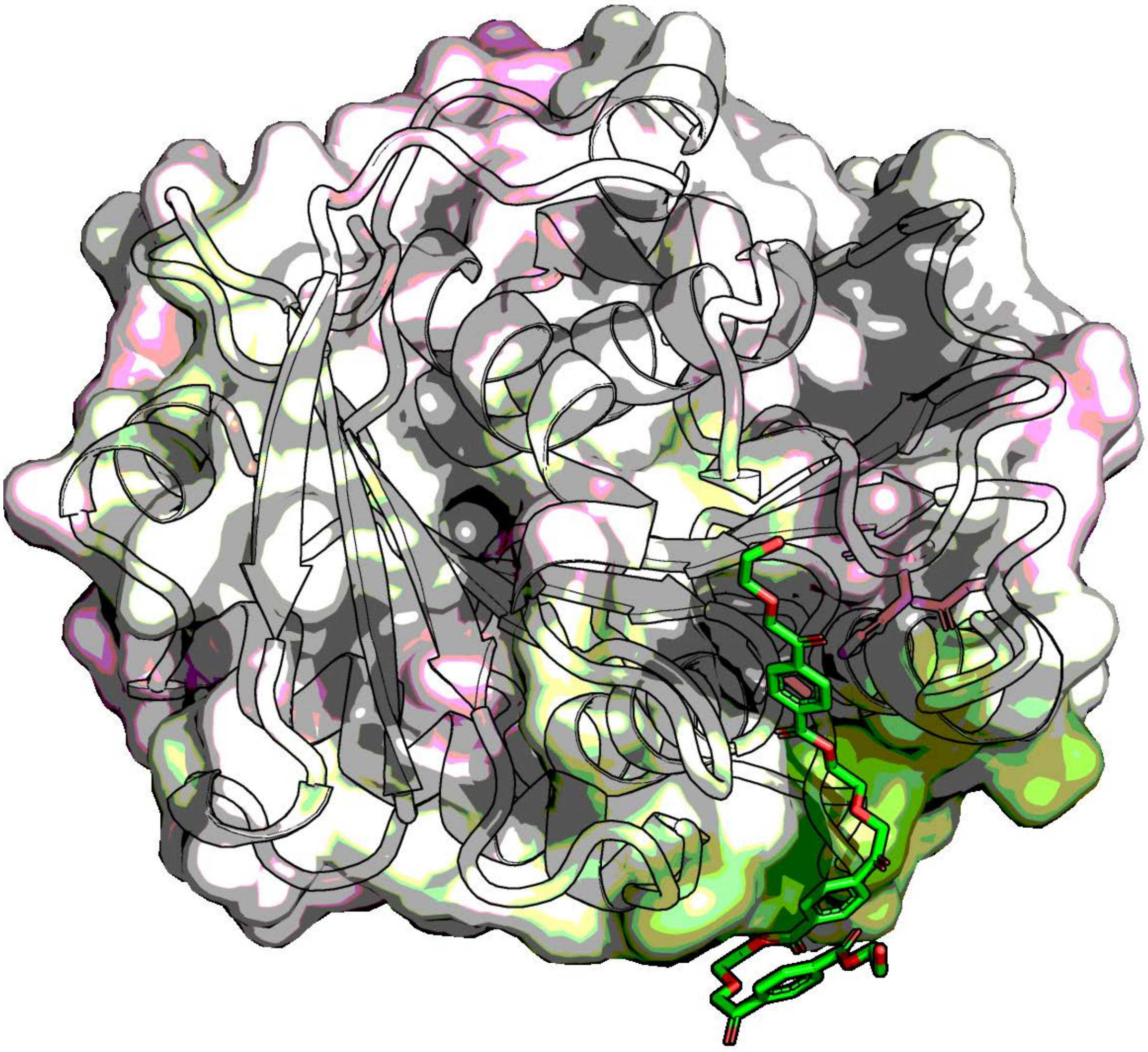

ph_stickiness_positive_mean_LCC-ICCG.png

Observed electrostatics

Created on: 2025-04-15 16:02:05

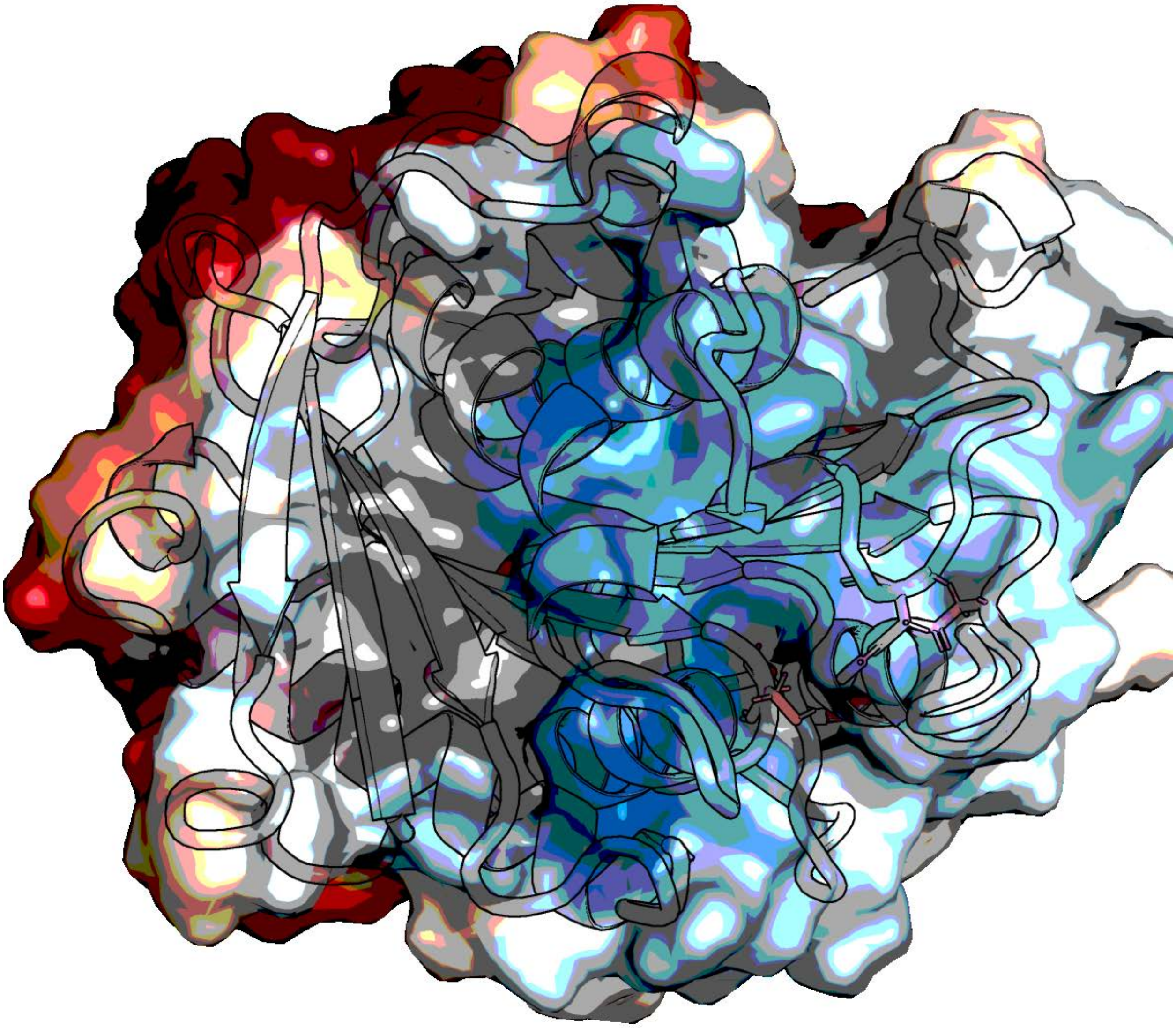

DP003_highlights.png

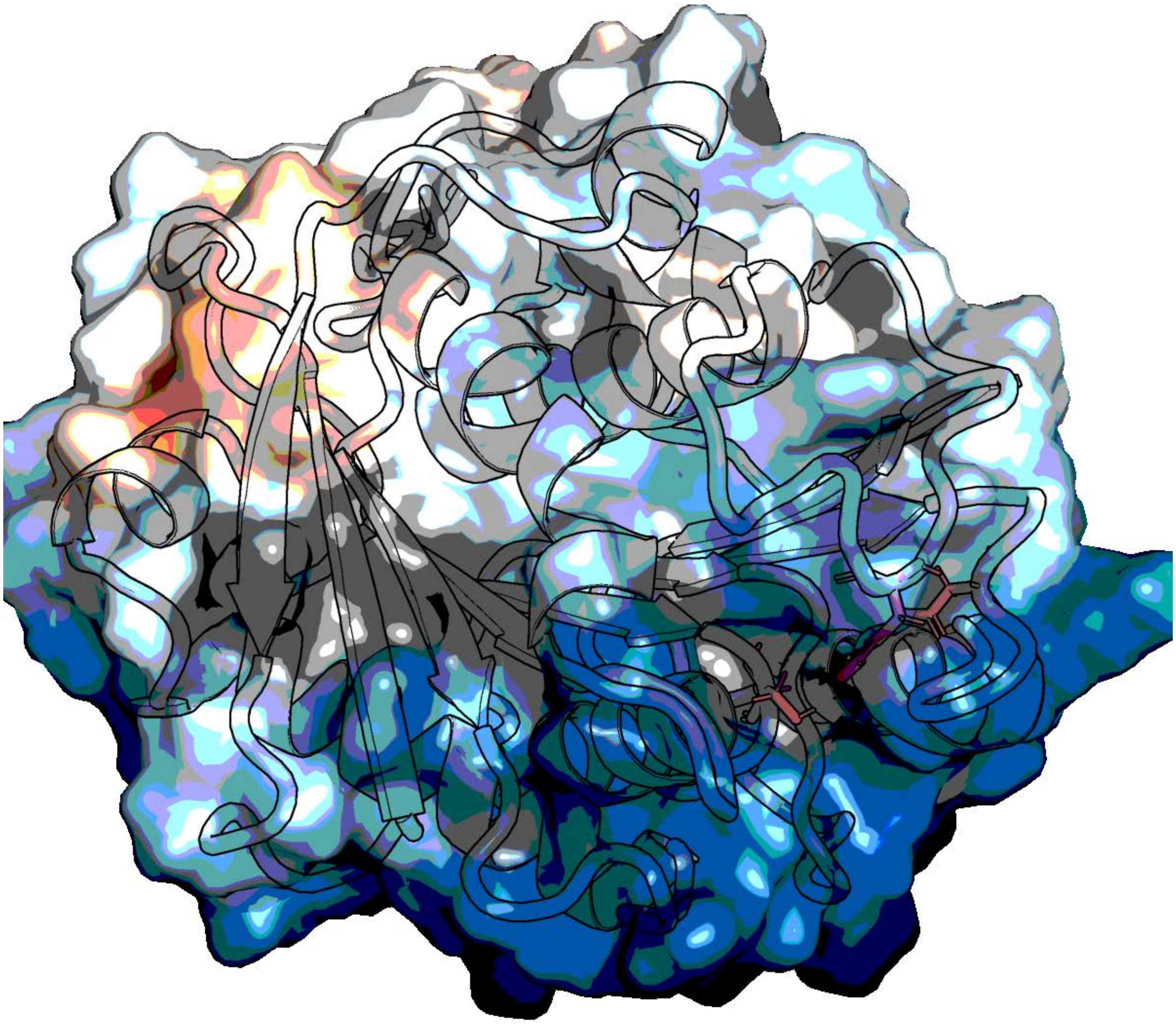

DP007_highlights.png

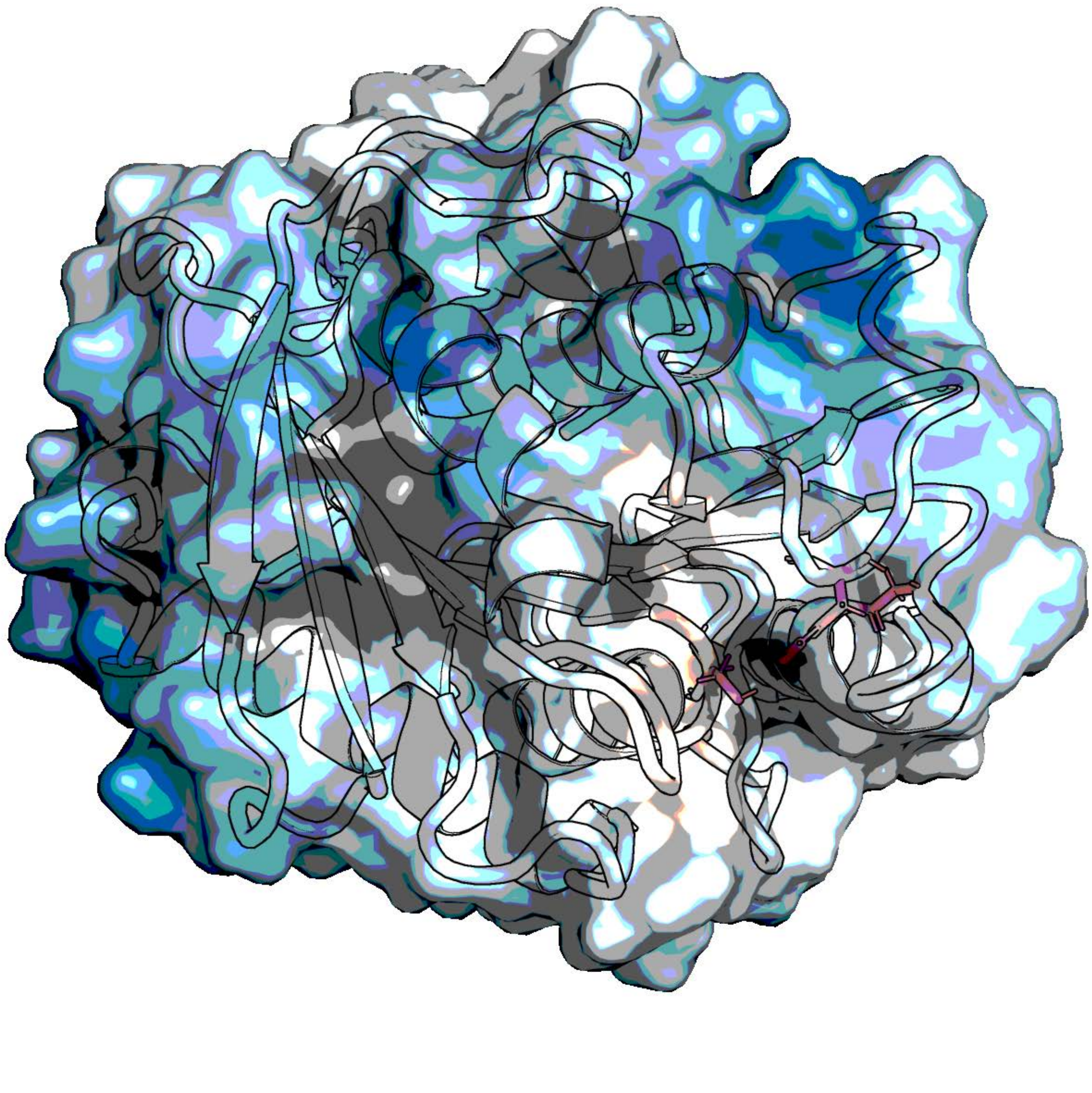

DP008_highlights.png

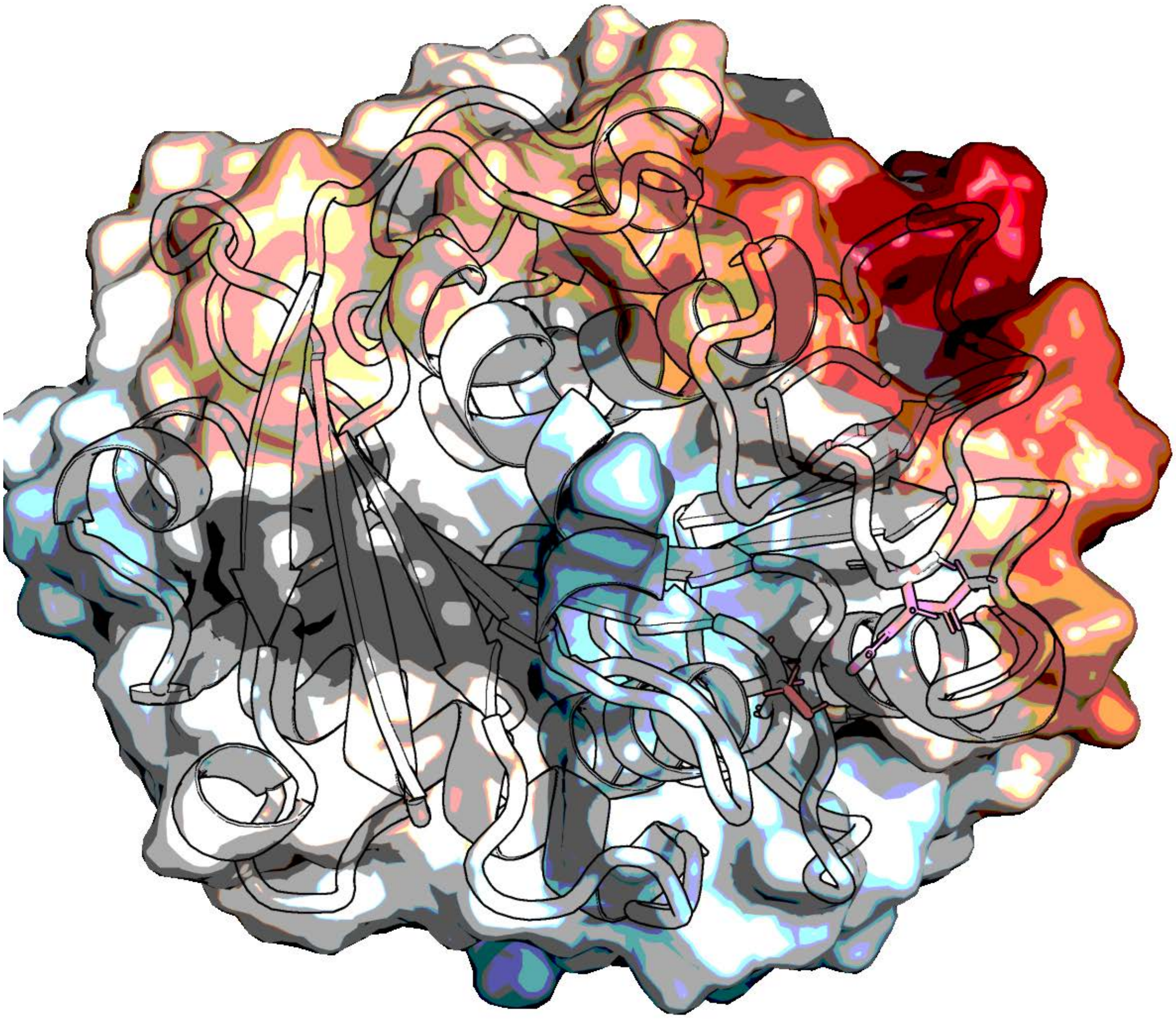

DP009_highlights.png

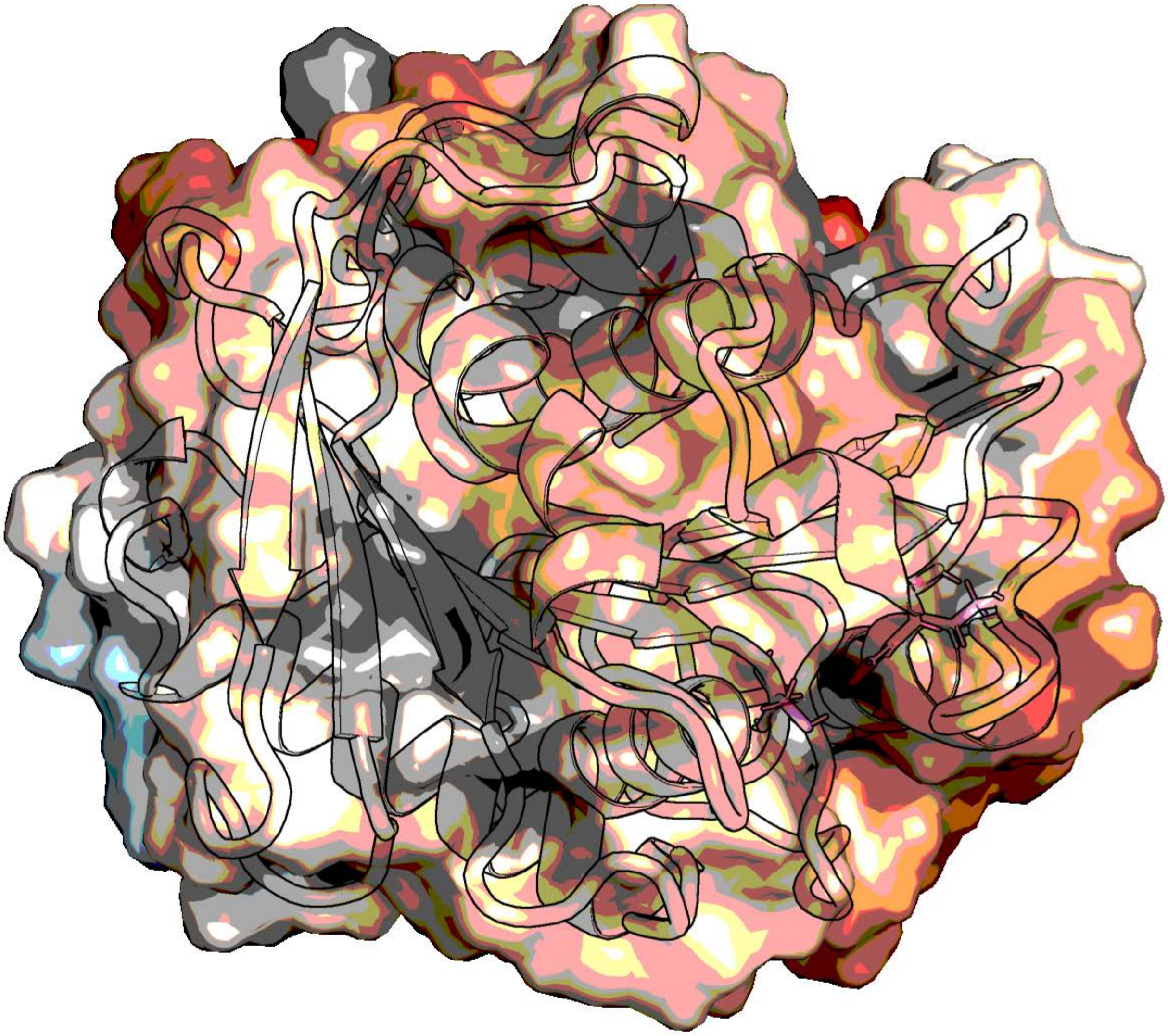

DP016_highlights.png

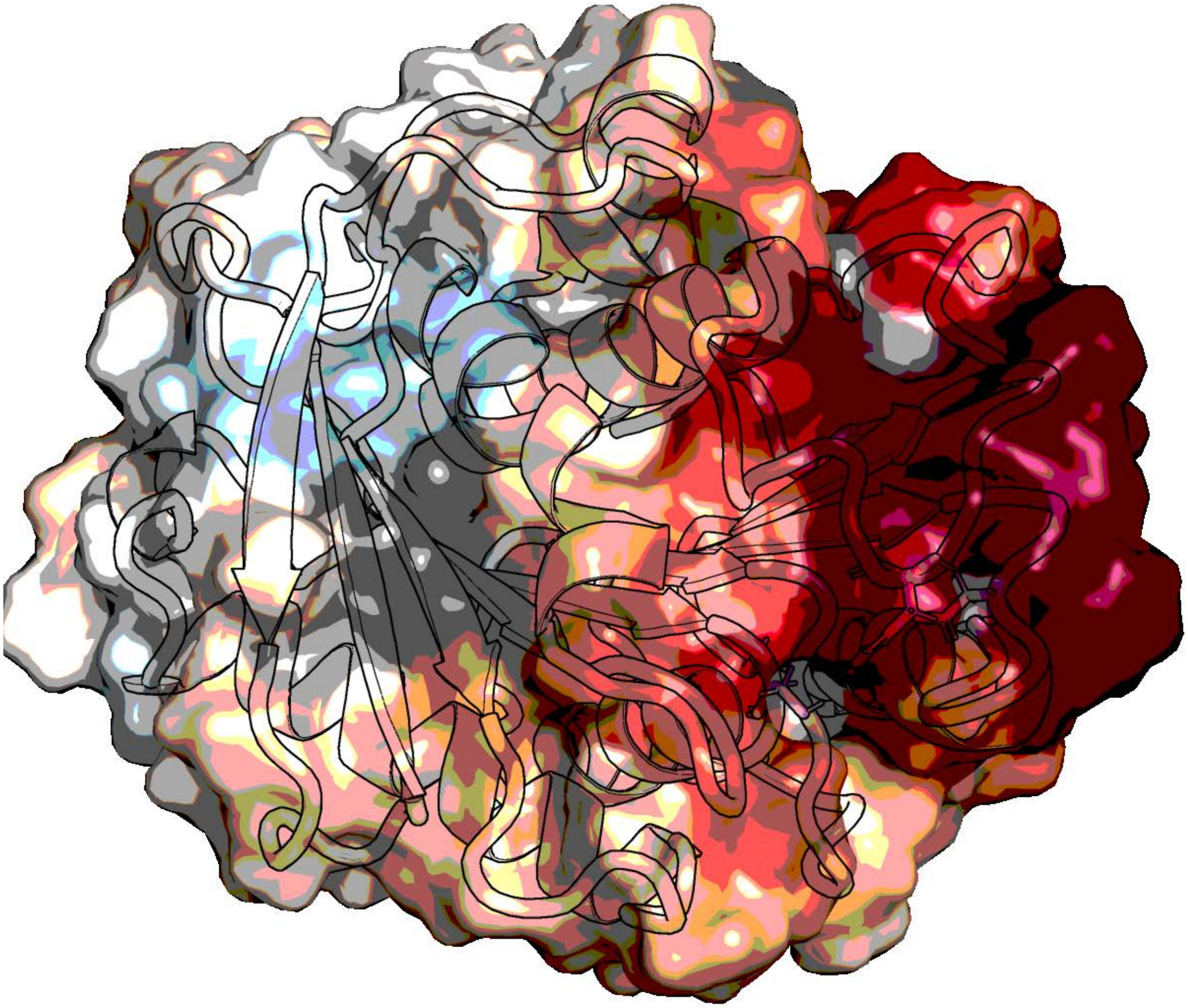

DP017_highlights.png

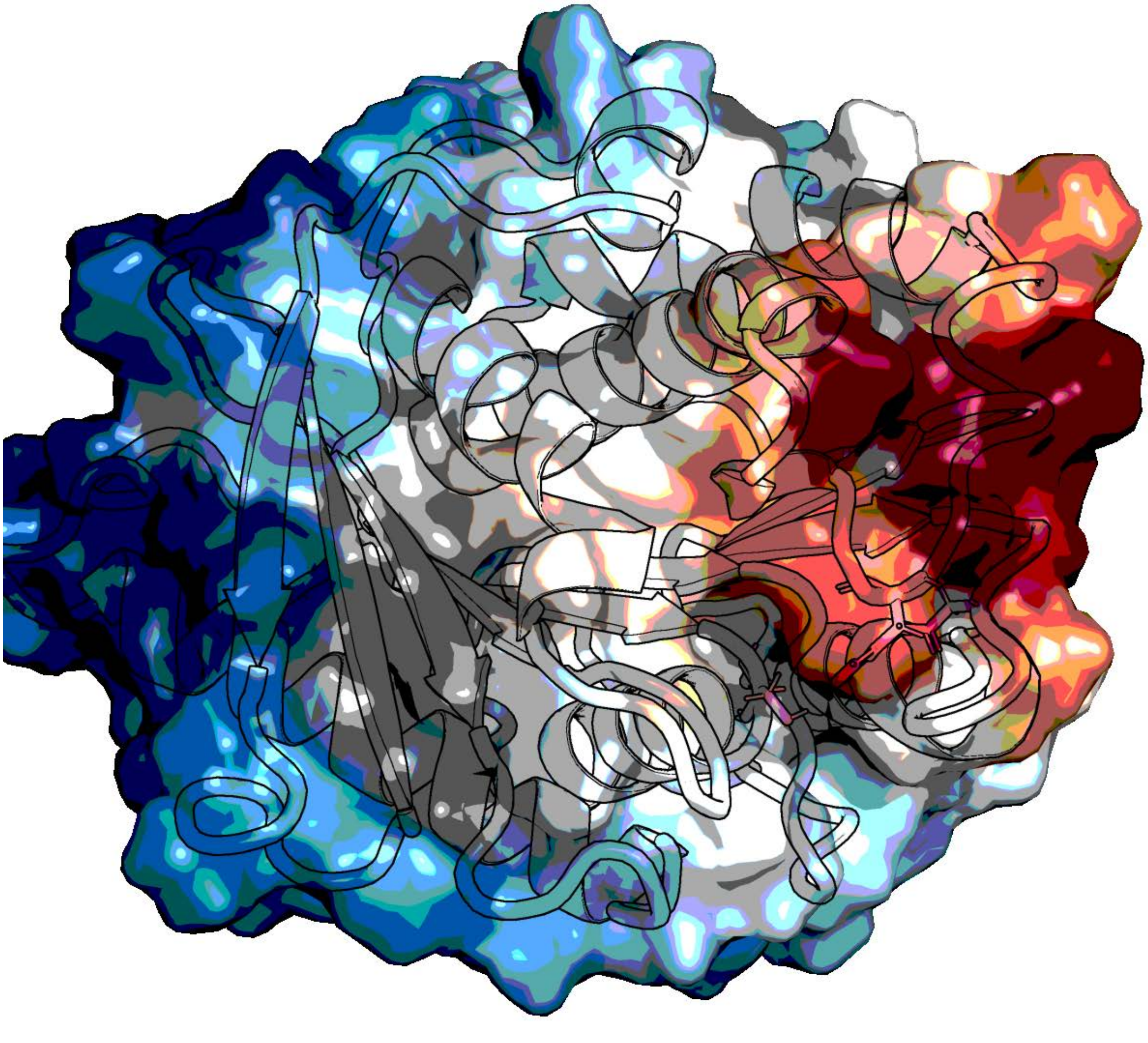

DP038_highlights.png

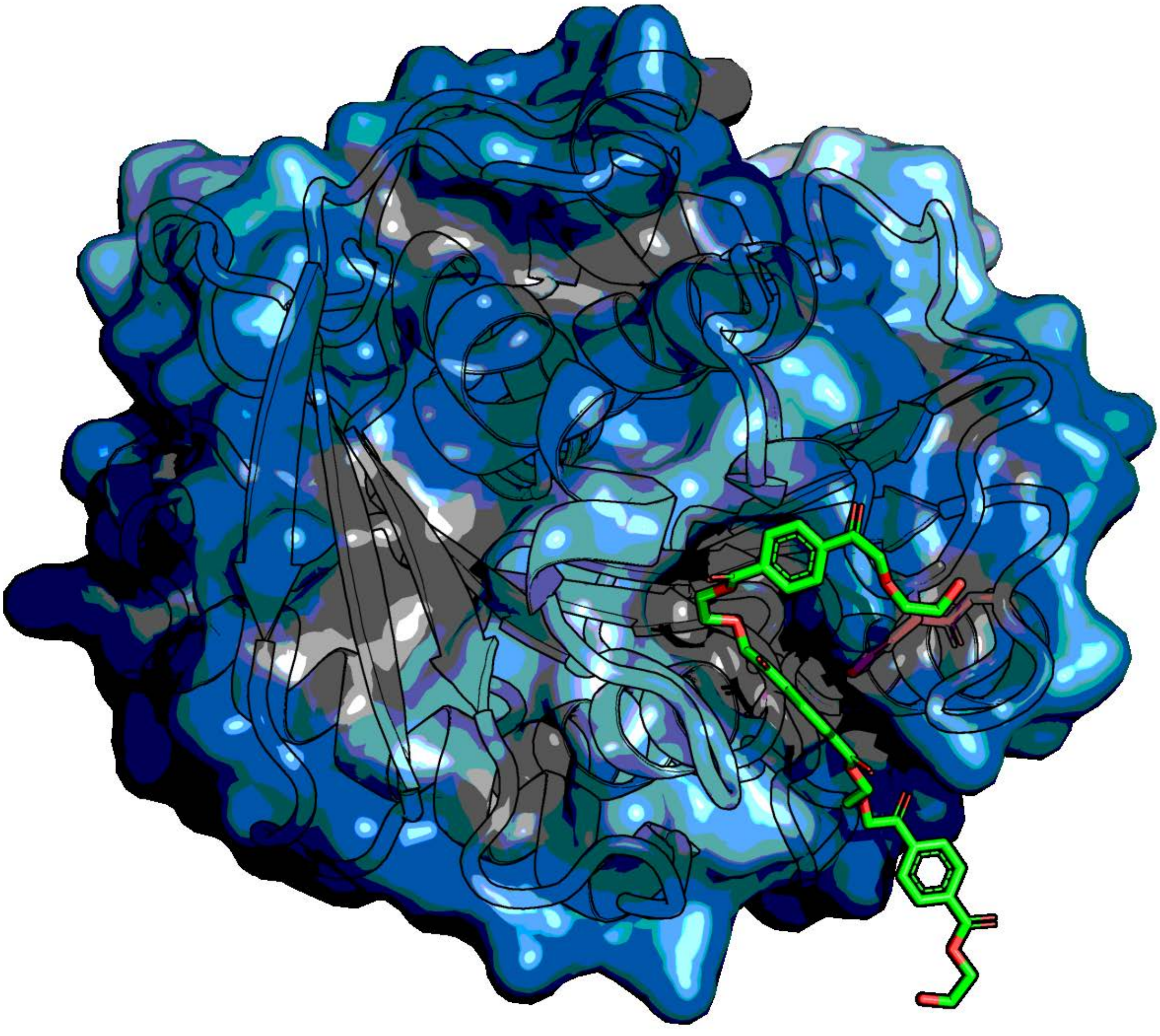

DP043_highlights.png

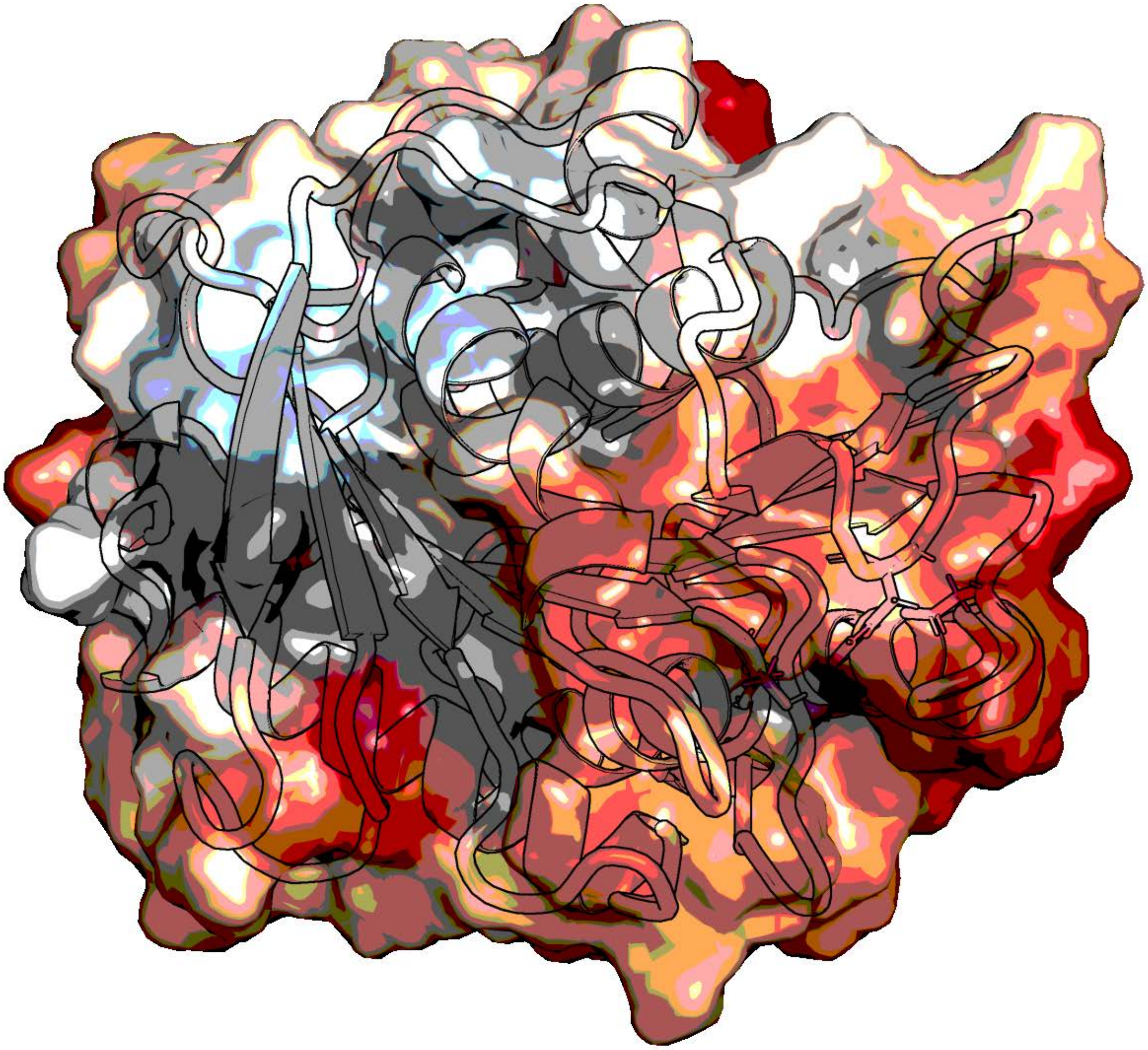

DP054_highlights.png

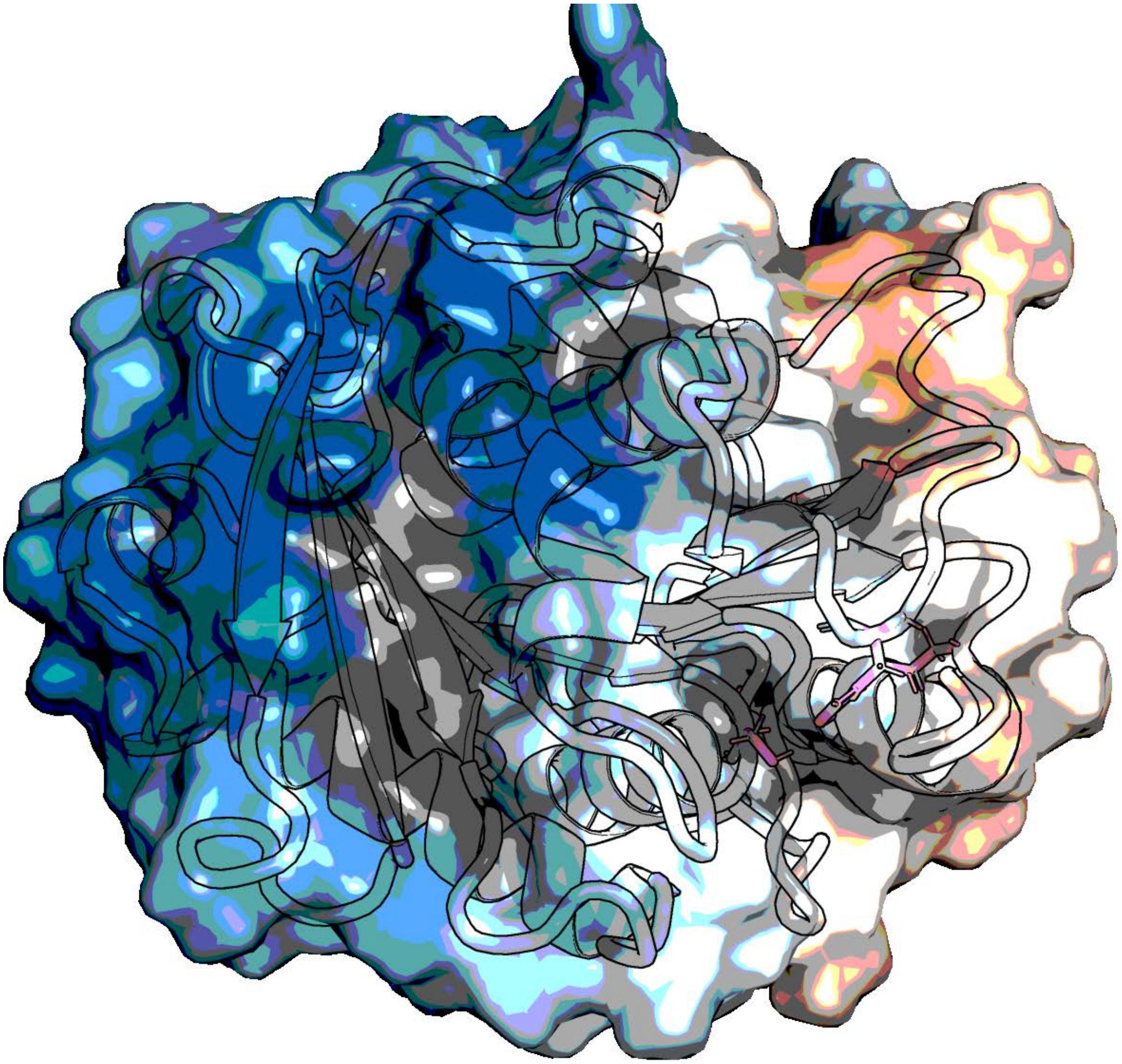

DP056_highlights.png

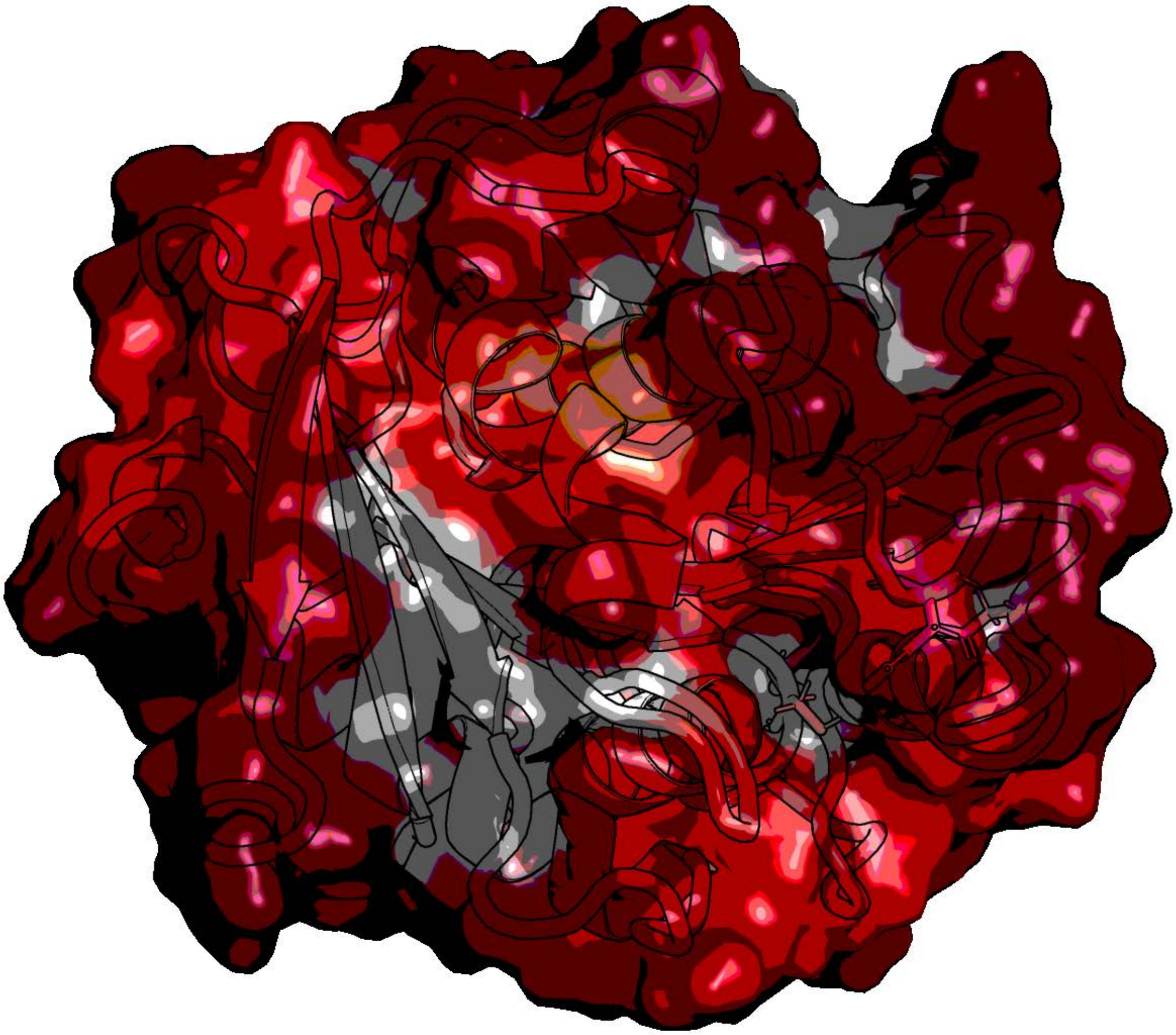

DP057_highlights.png

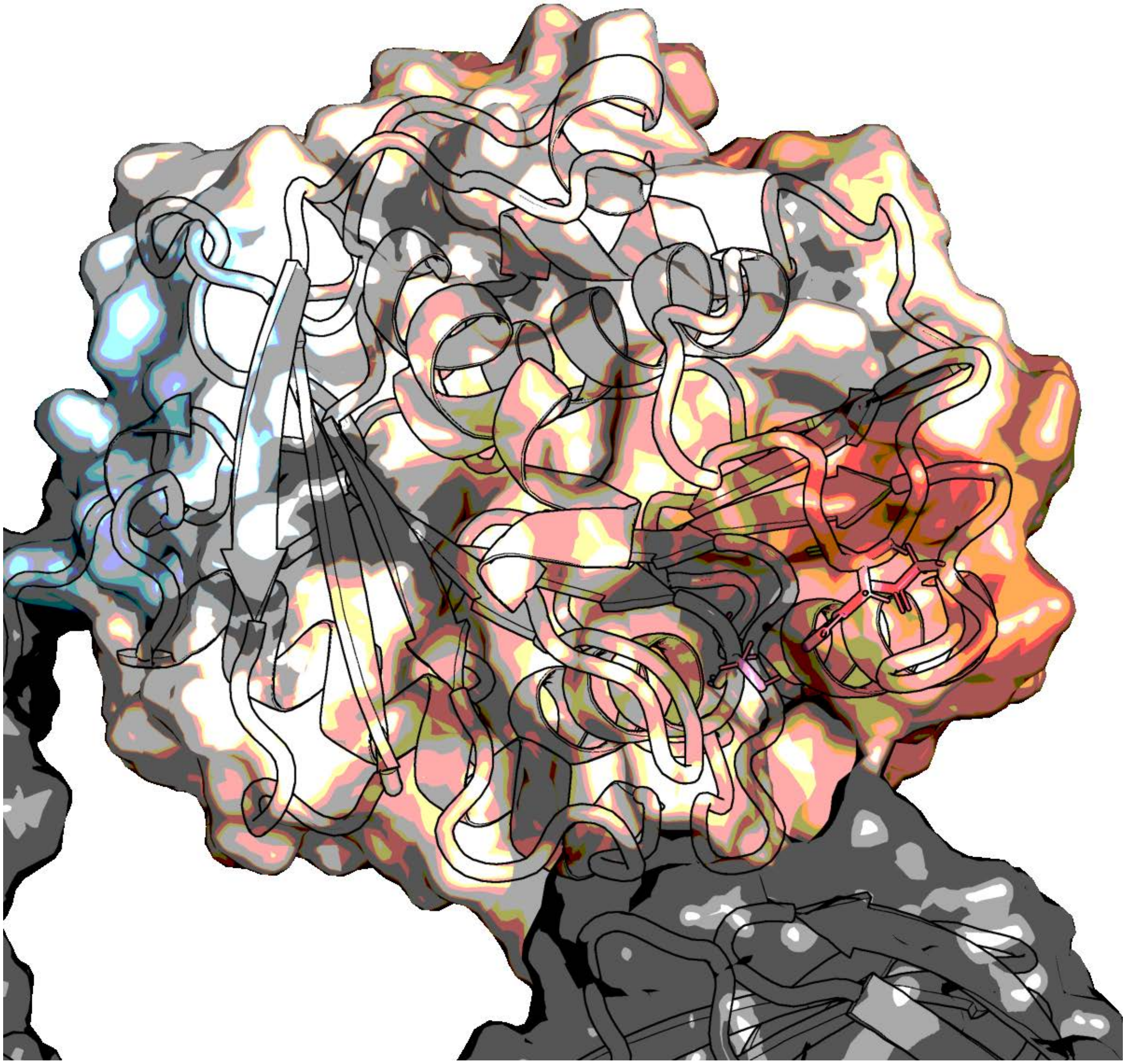

DP059_highlights.png

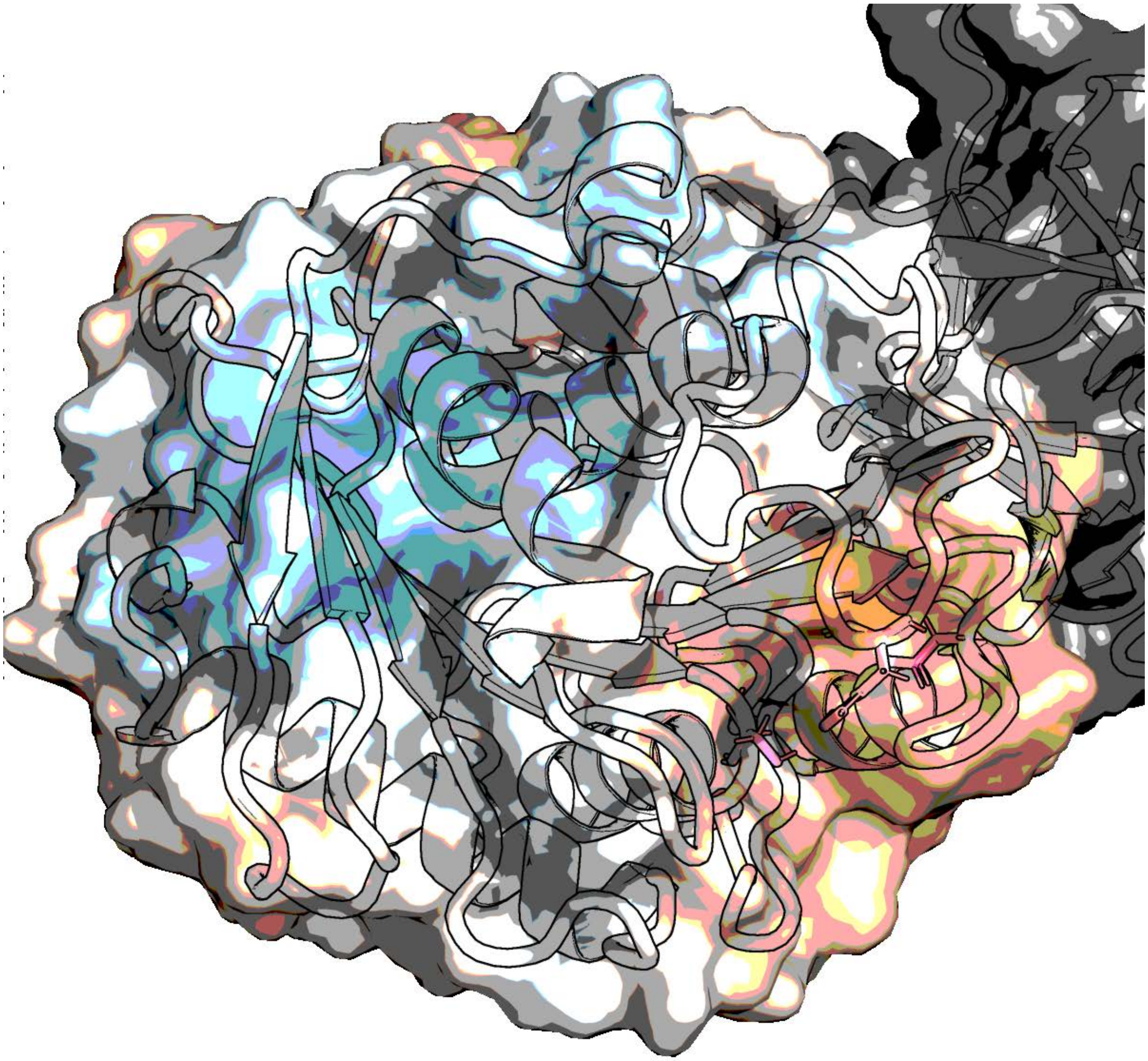

DP061_highlights.png

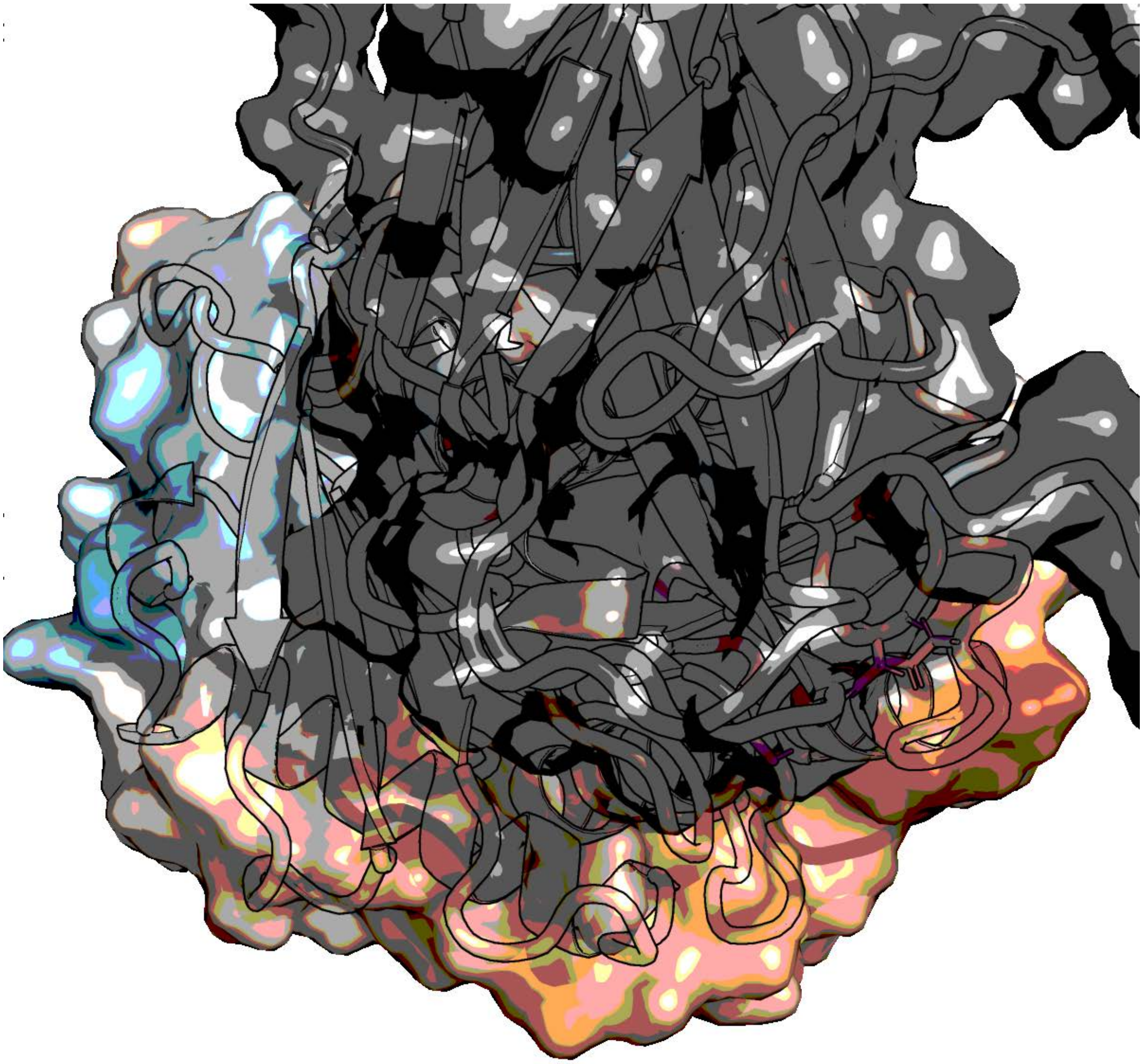

DP068_highlights.png

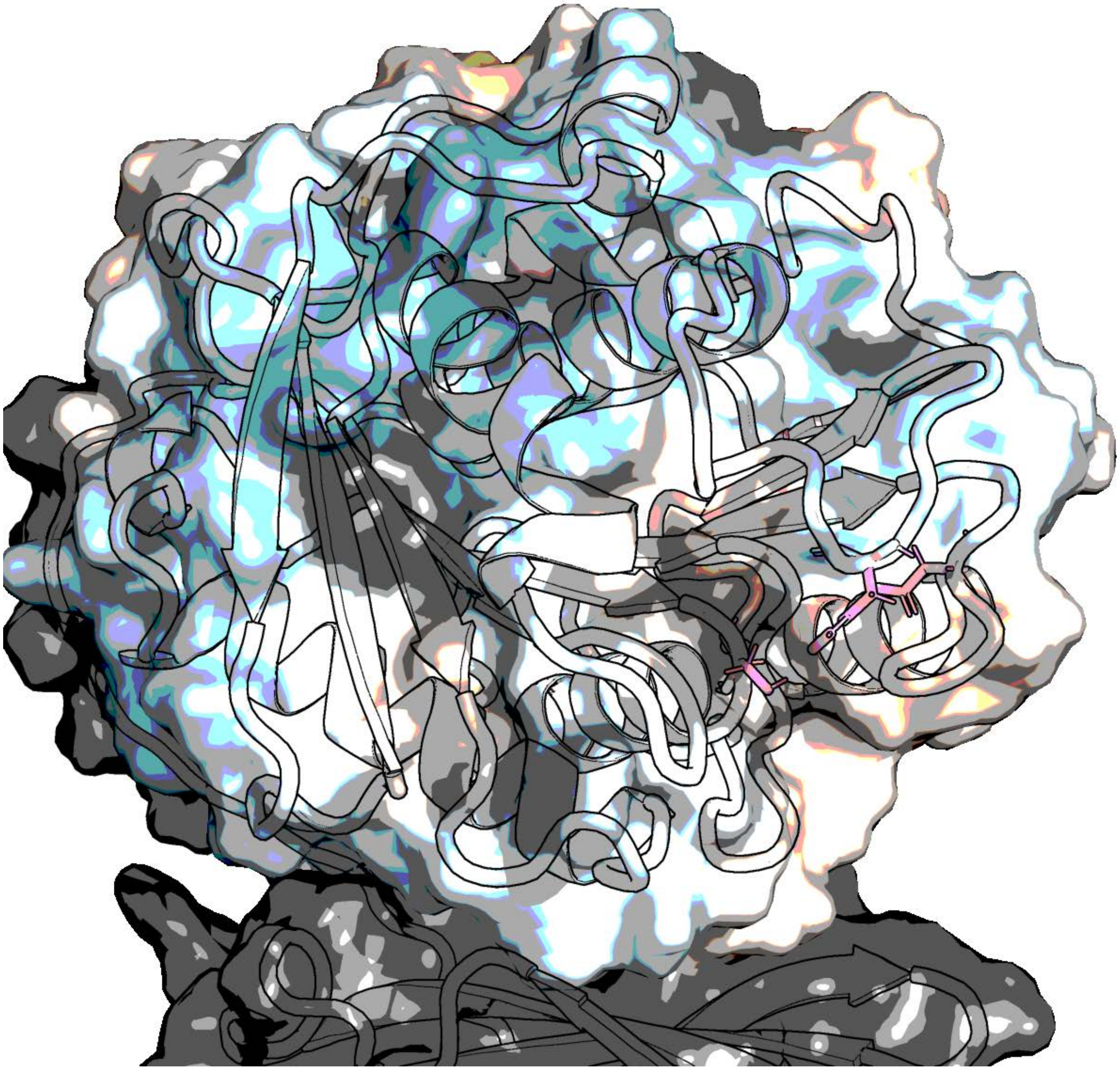

DP072_highlights.png

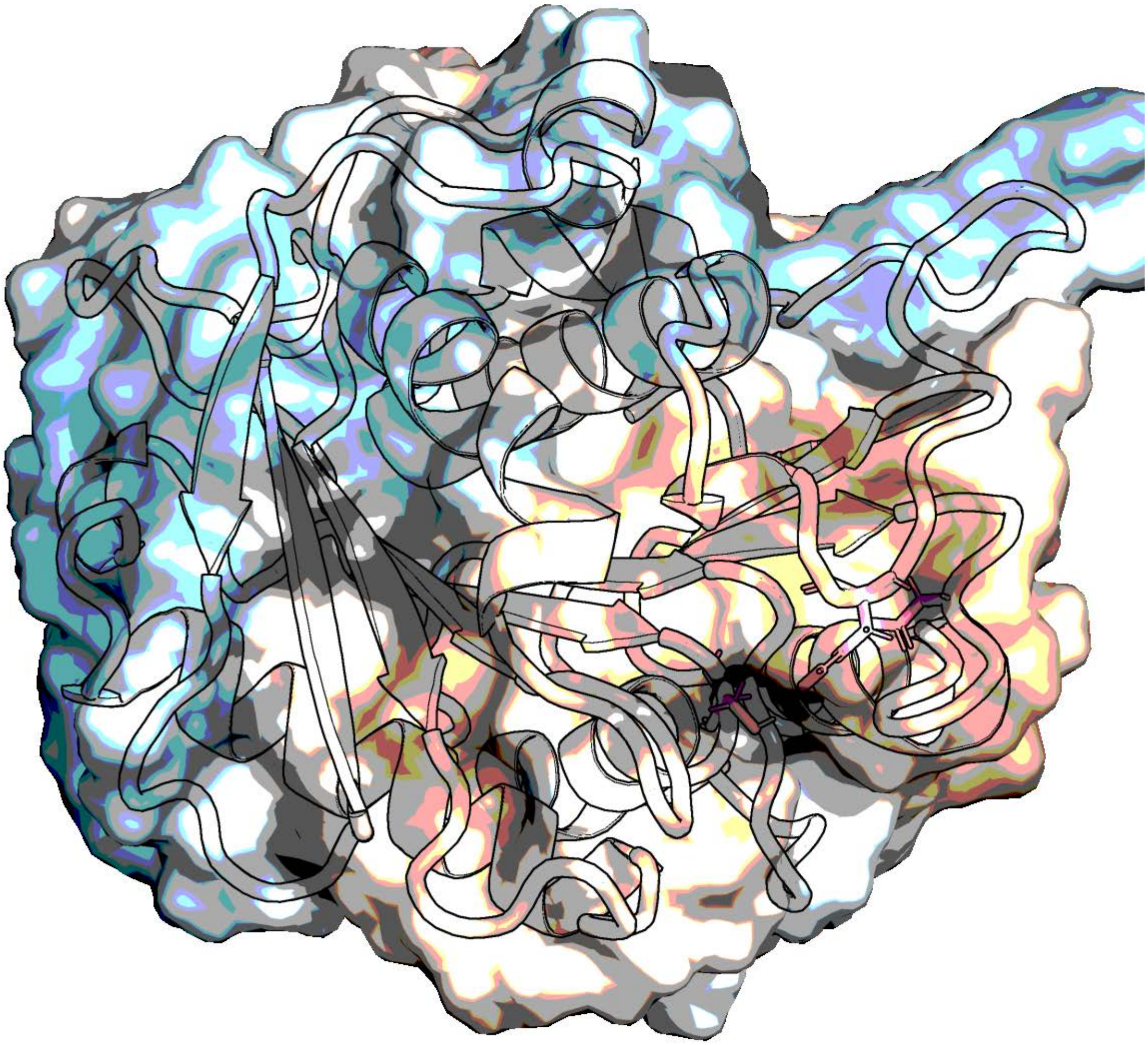

DP077_highlights.png

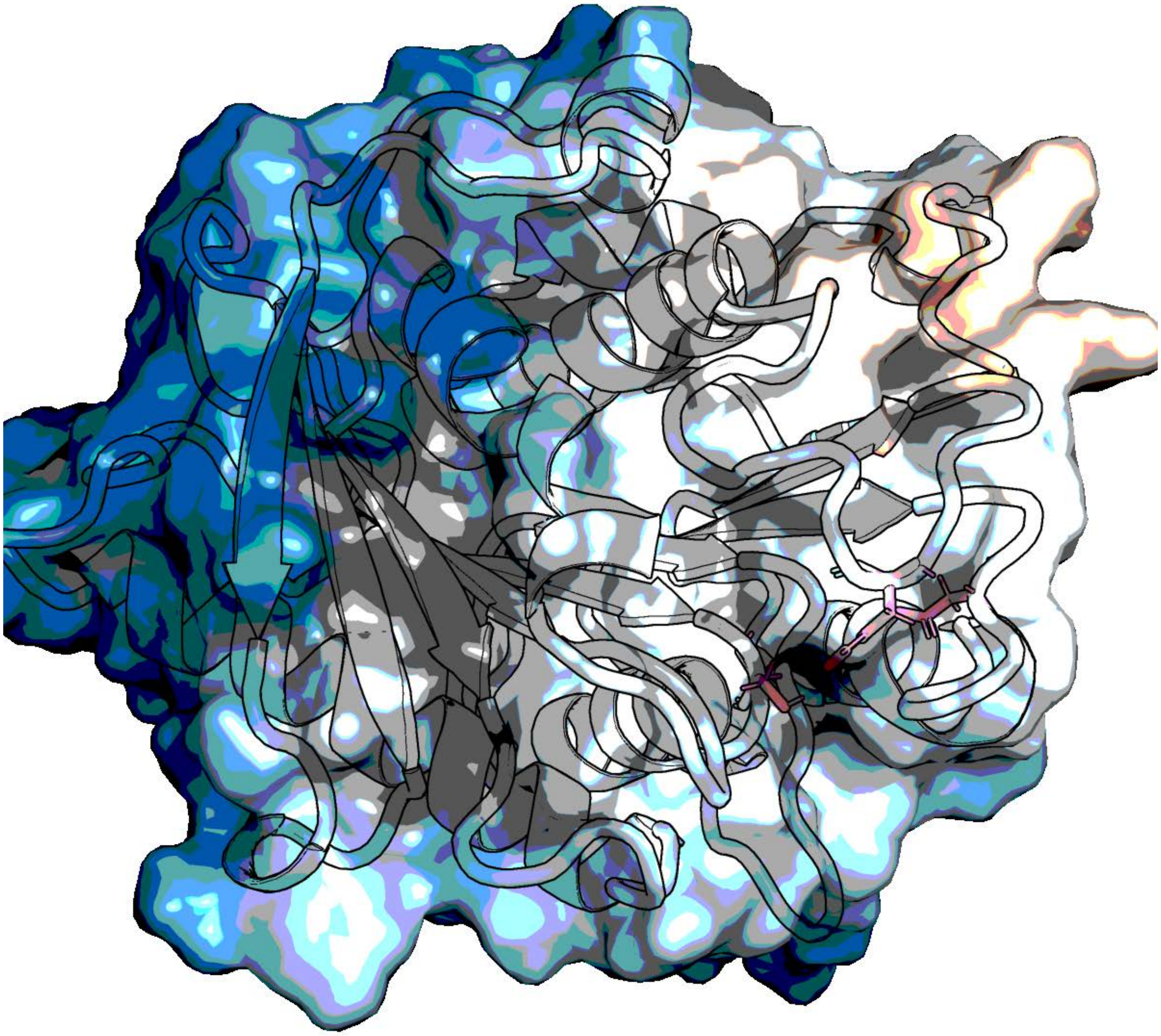

DP078_highlights.png

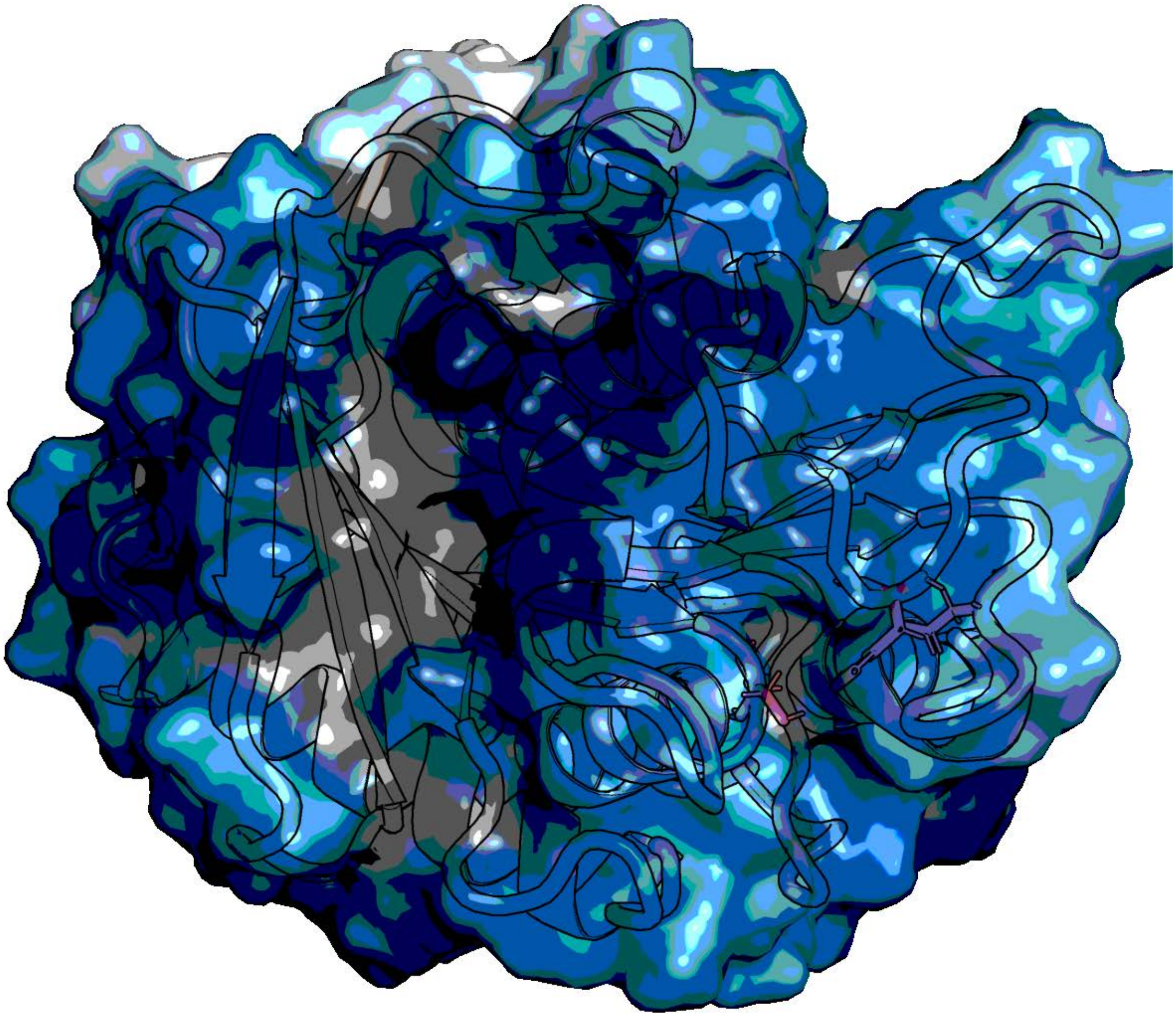

DP080_highlights.png

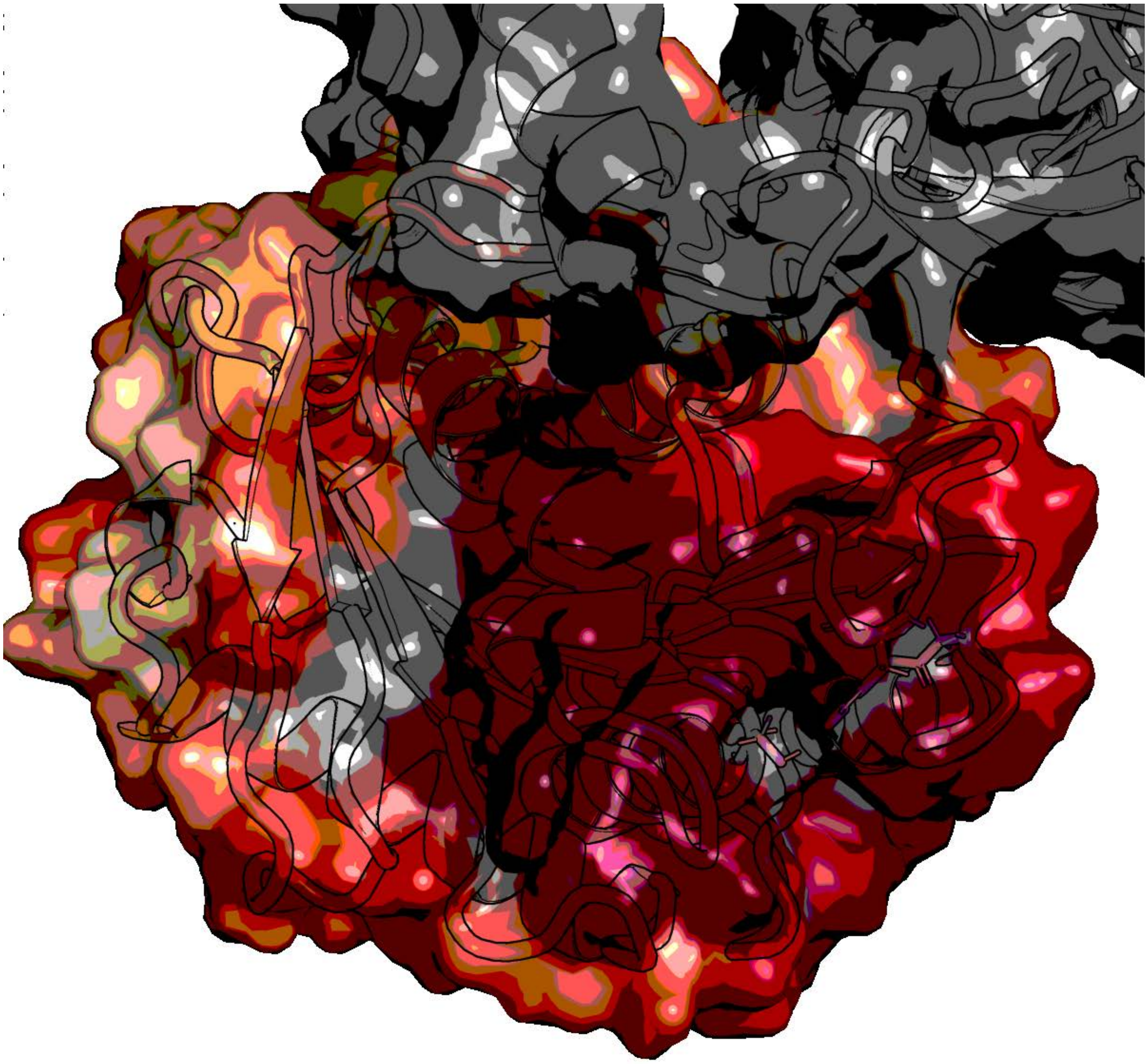

DP081_highlights.png

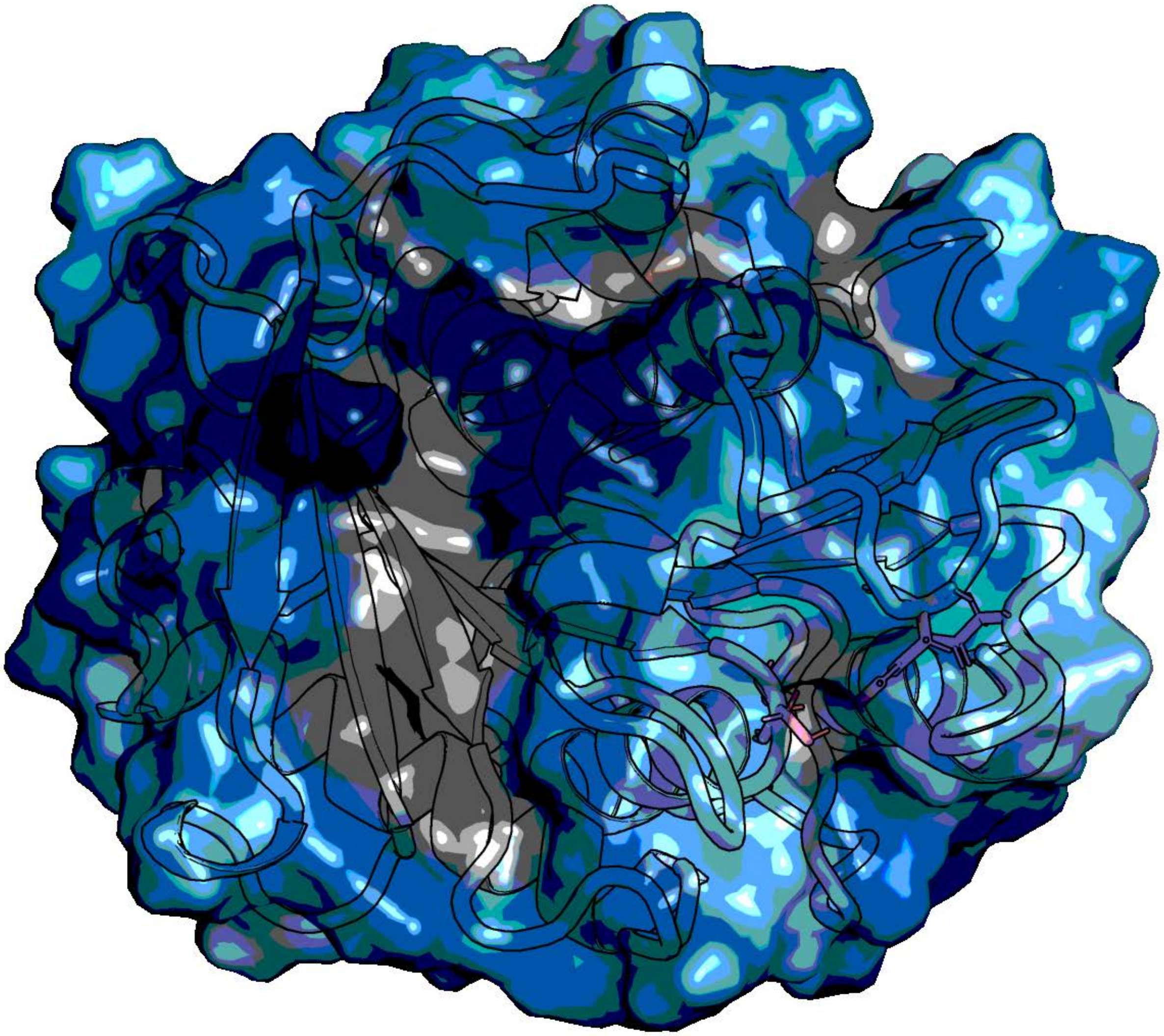

DP082_highlights.png

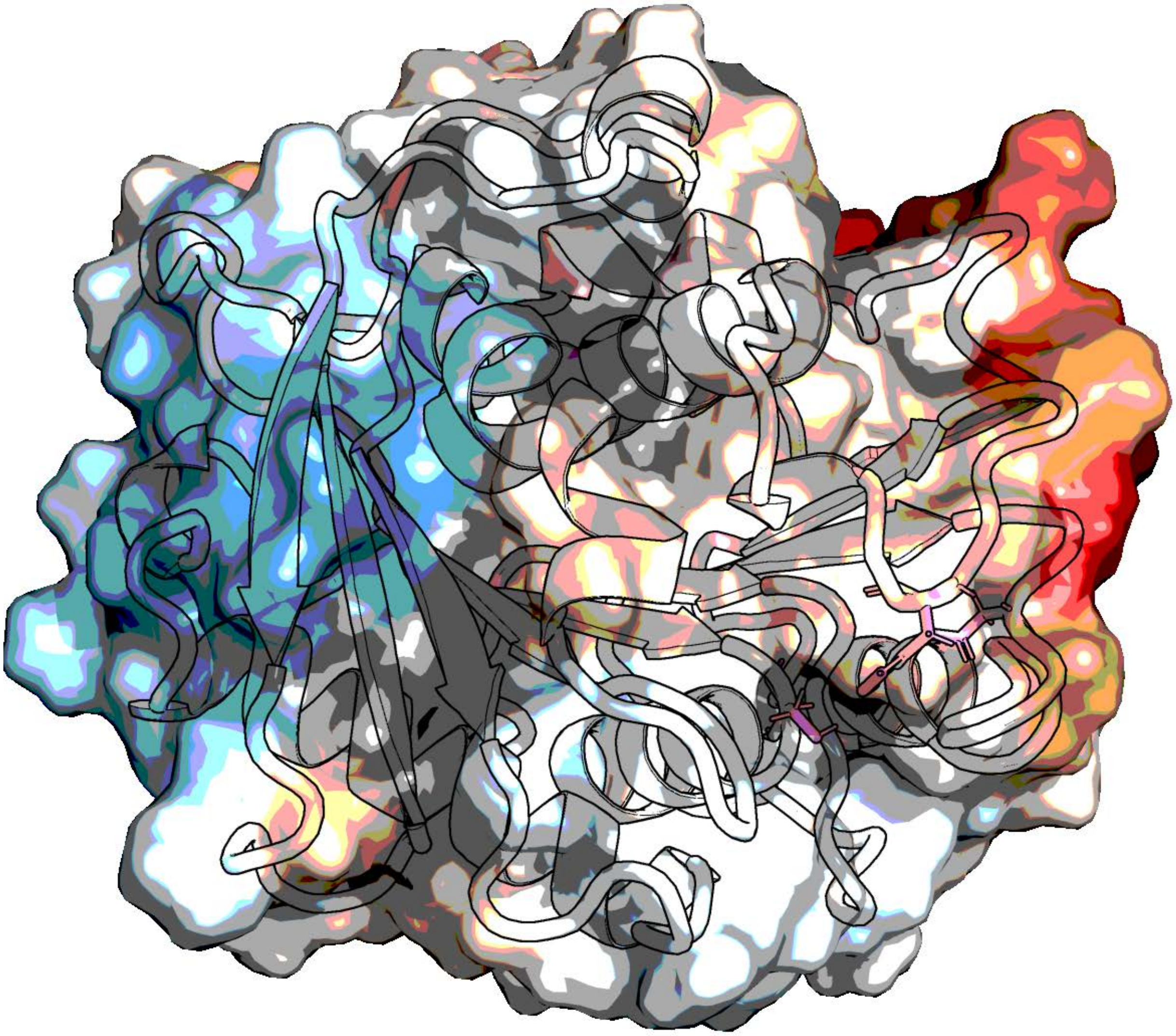

DP084_highlights.png

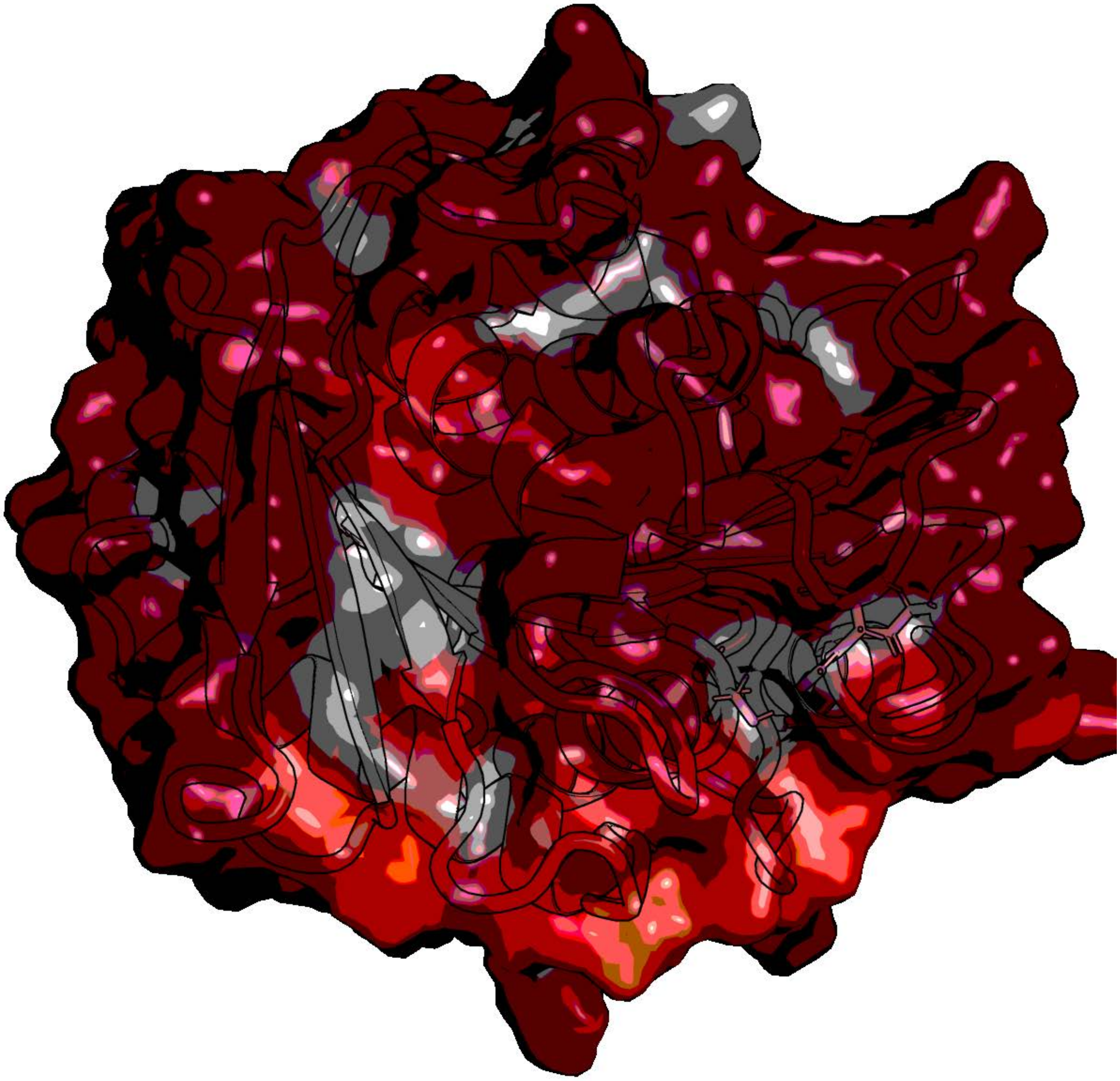

DP089_highlights.png

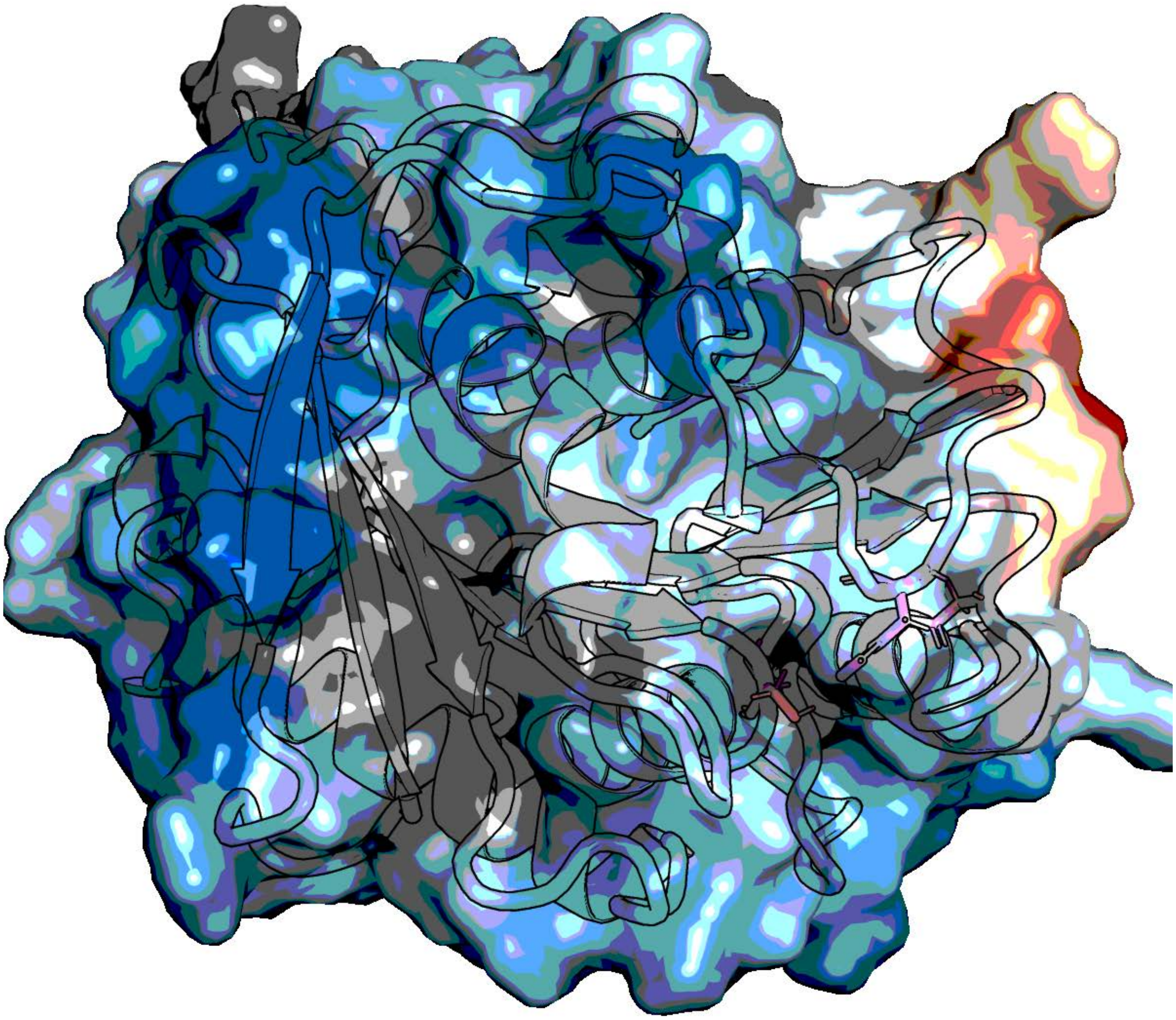

DP090_highlights.png

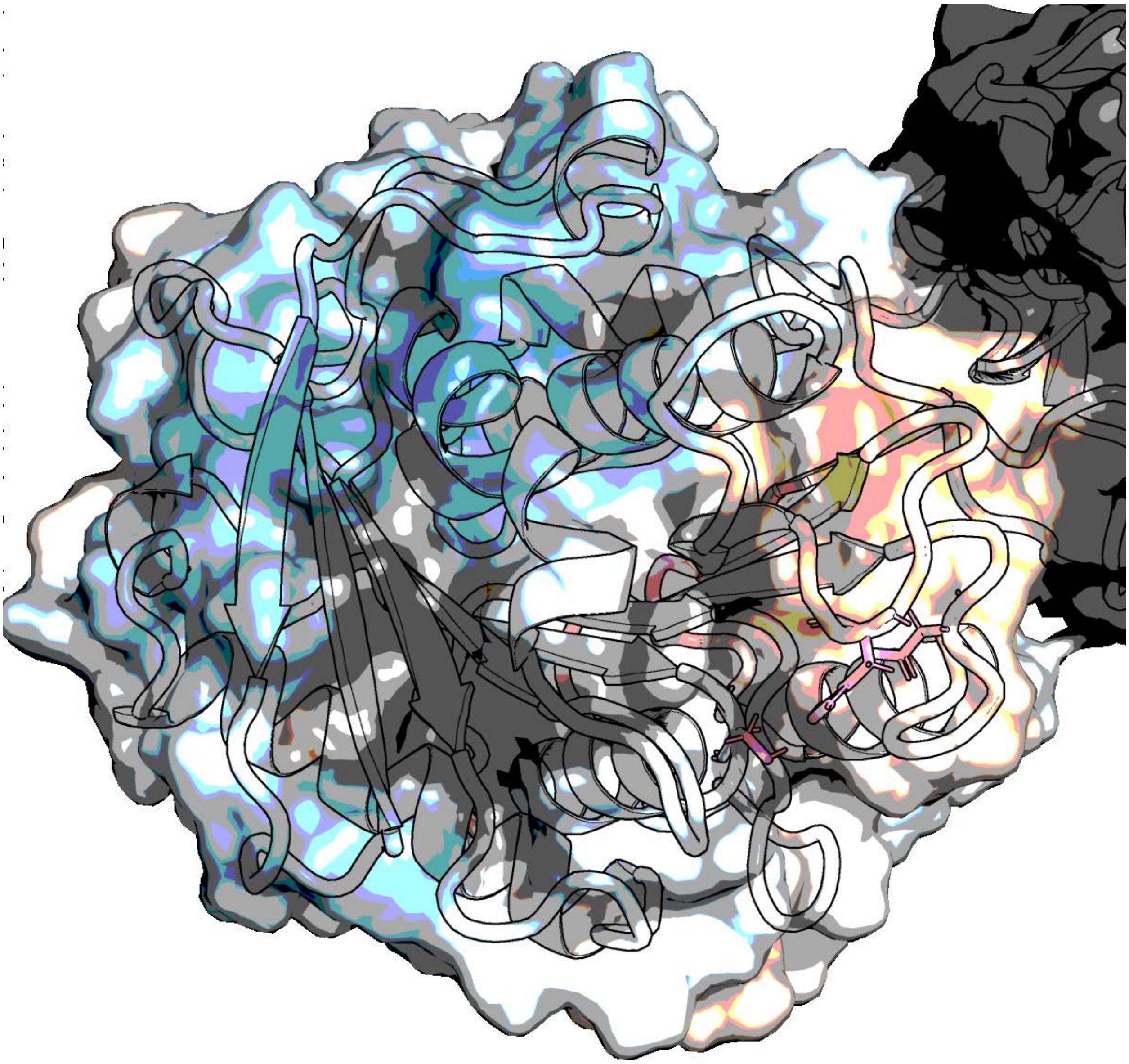

DP092_highlights.png

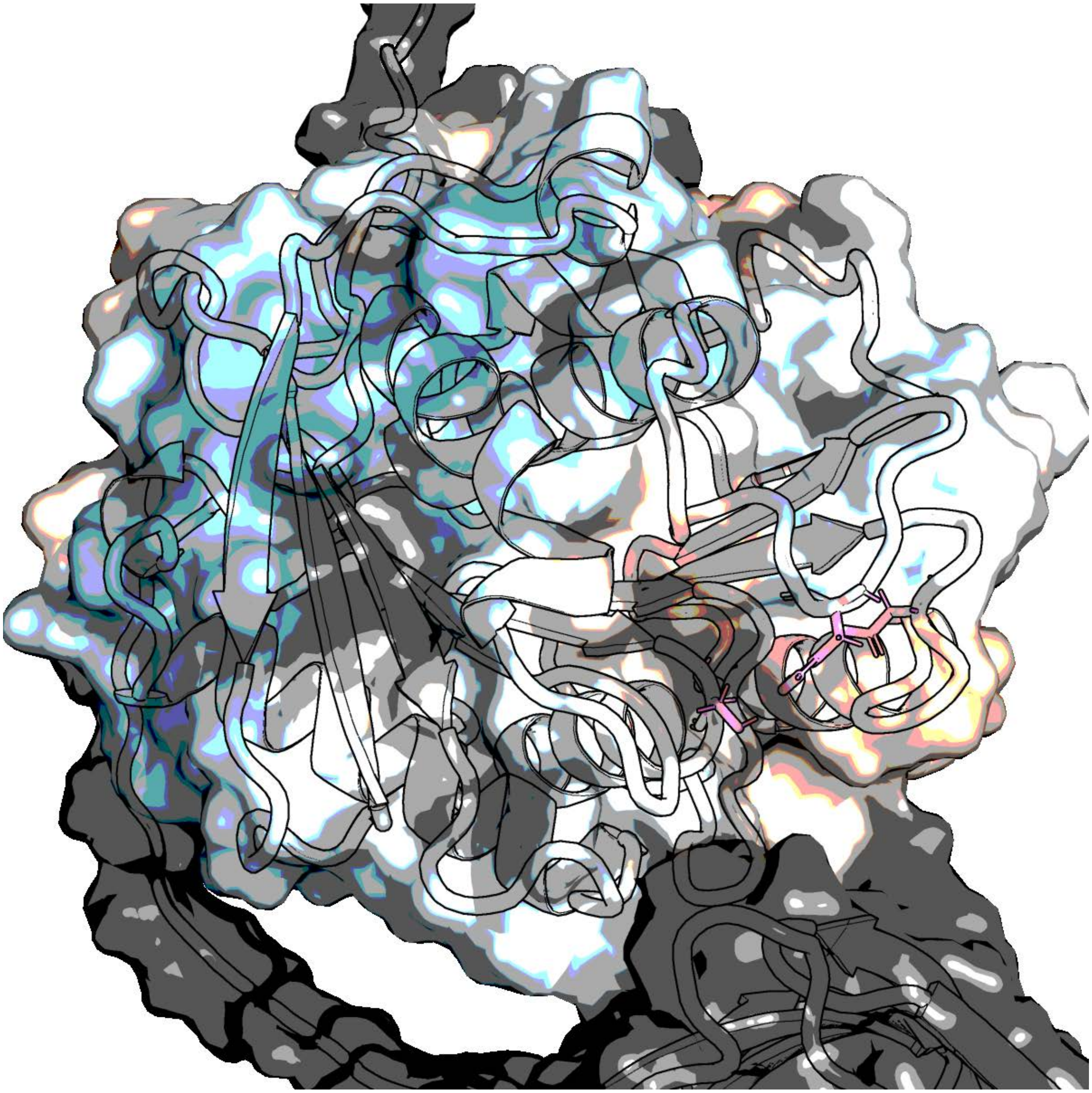

ESM006_highlights.png

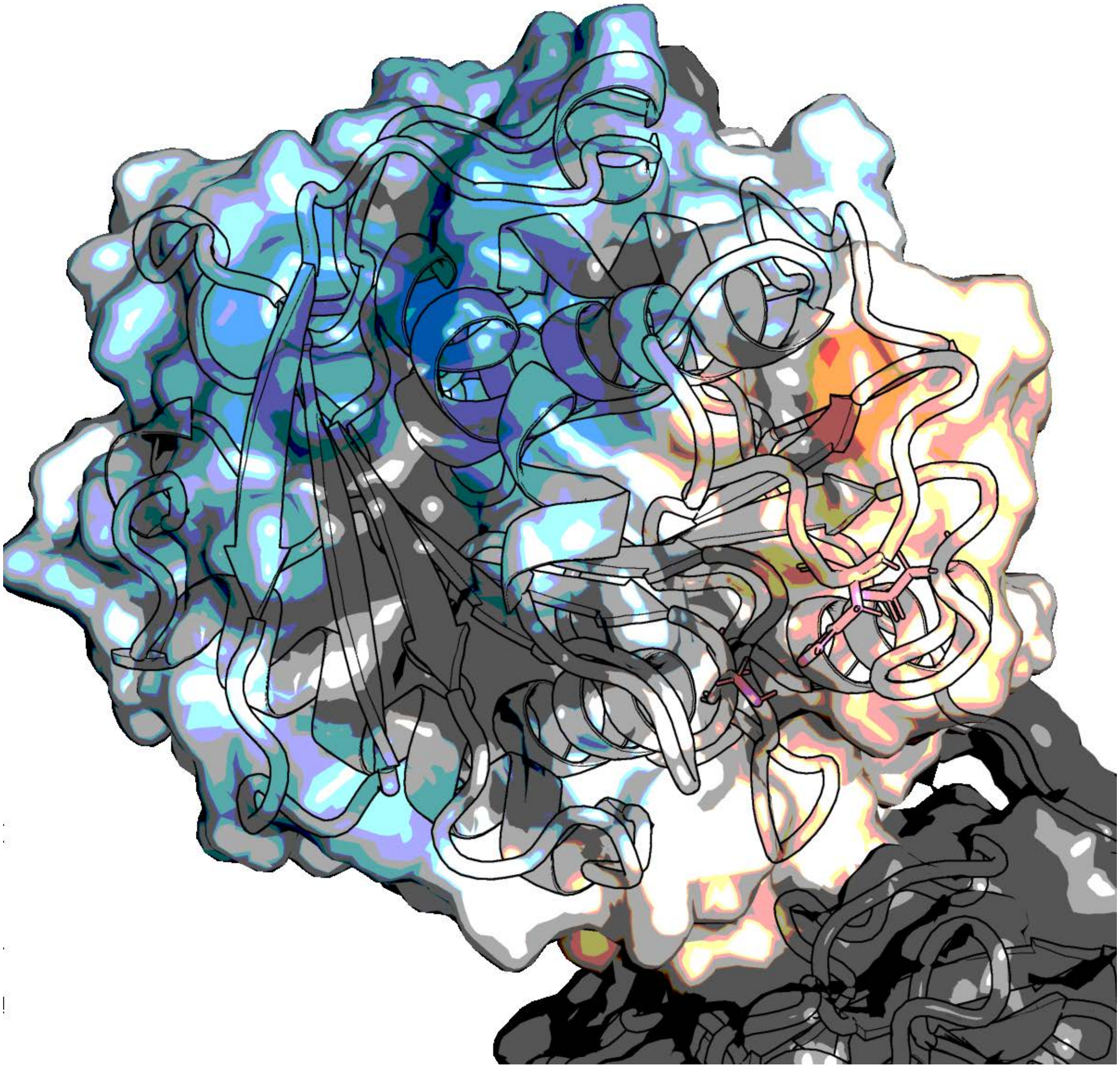

ESM010_highlights.png

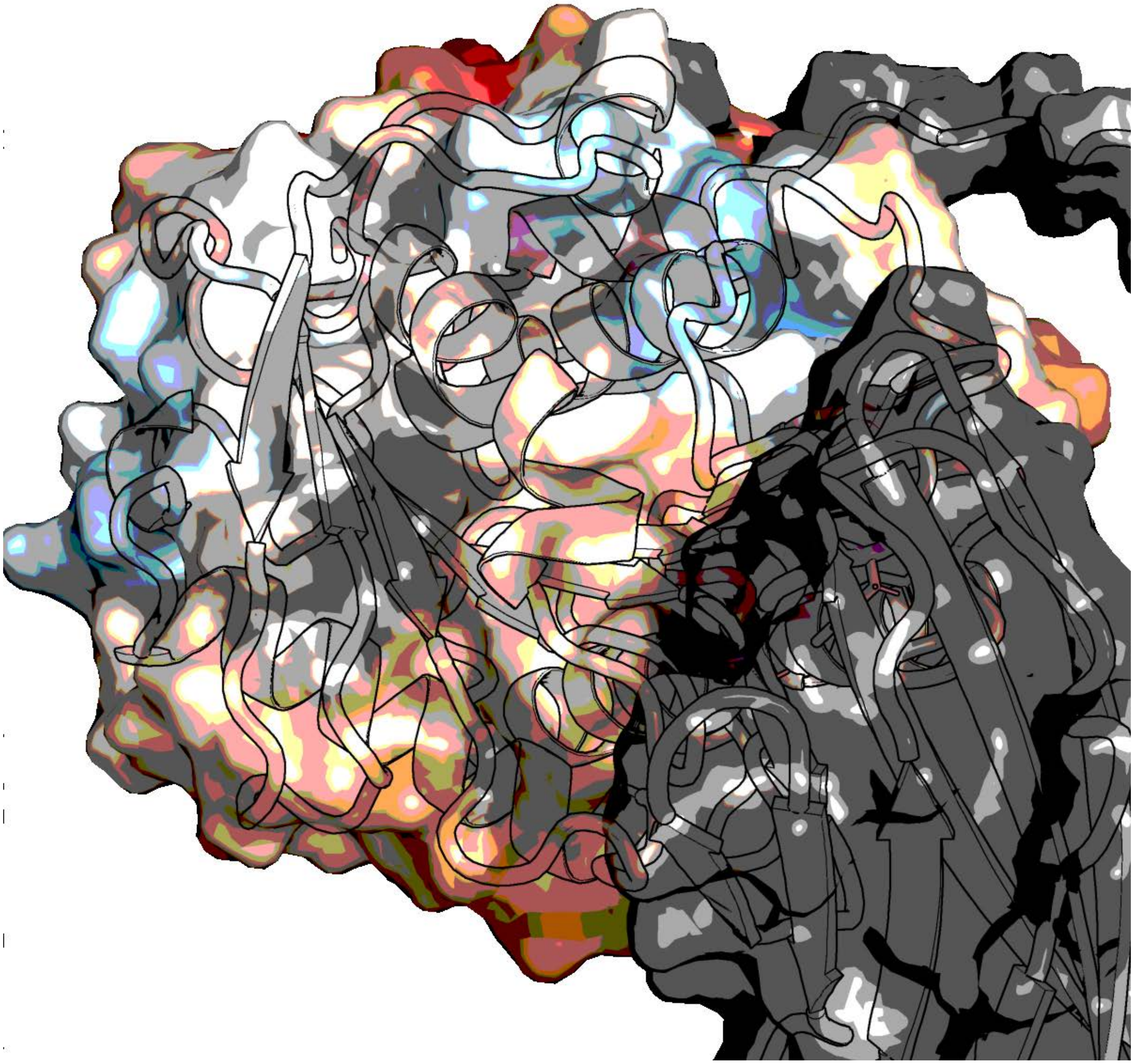

ESM012_highlights.png

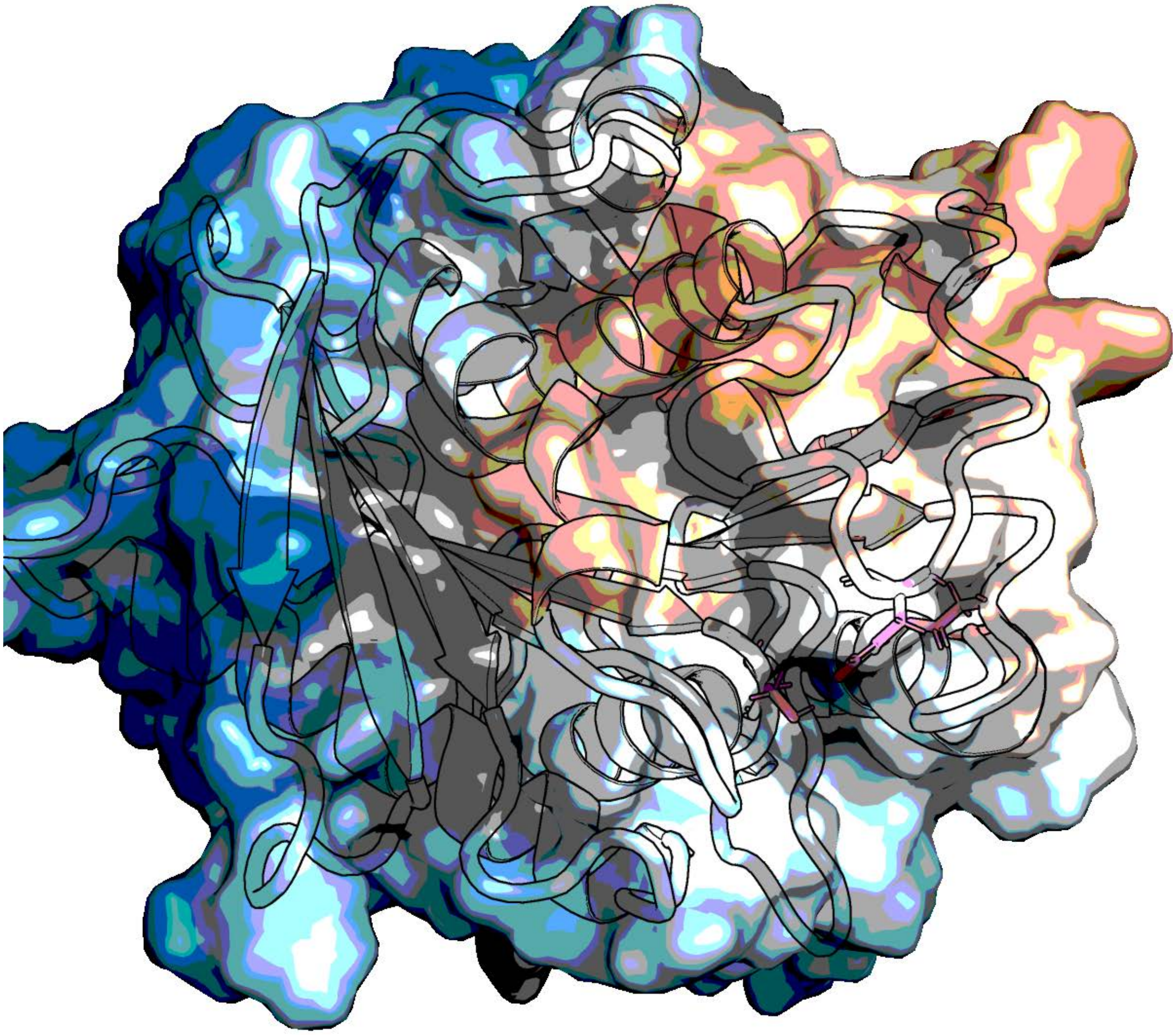

ESM017_highlights.png

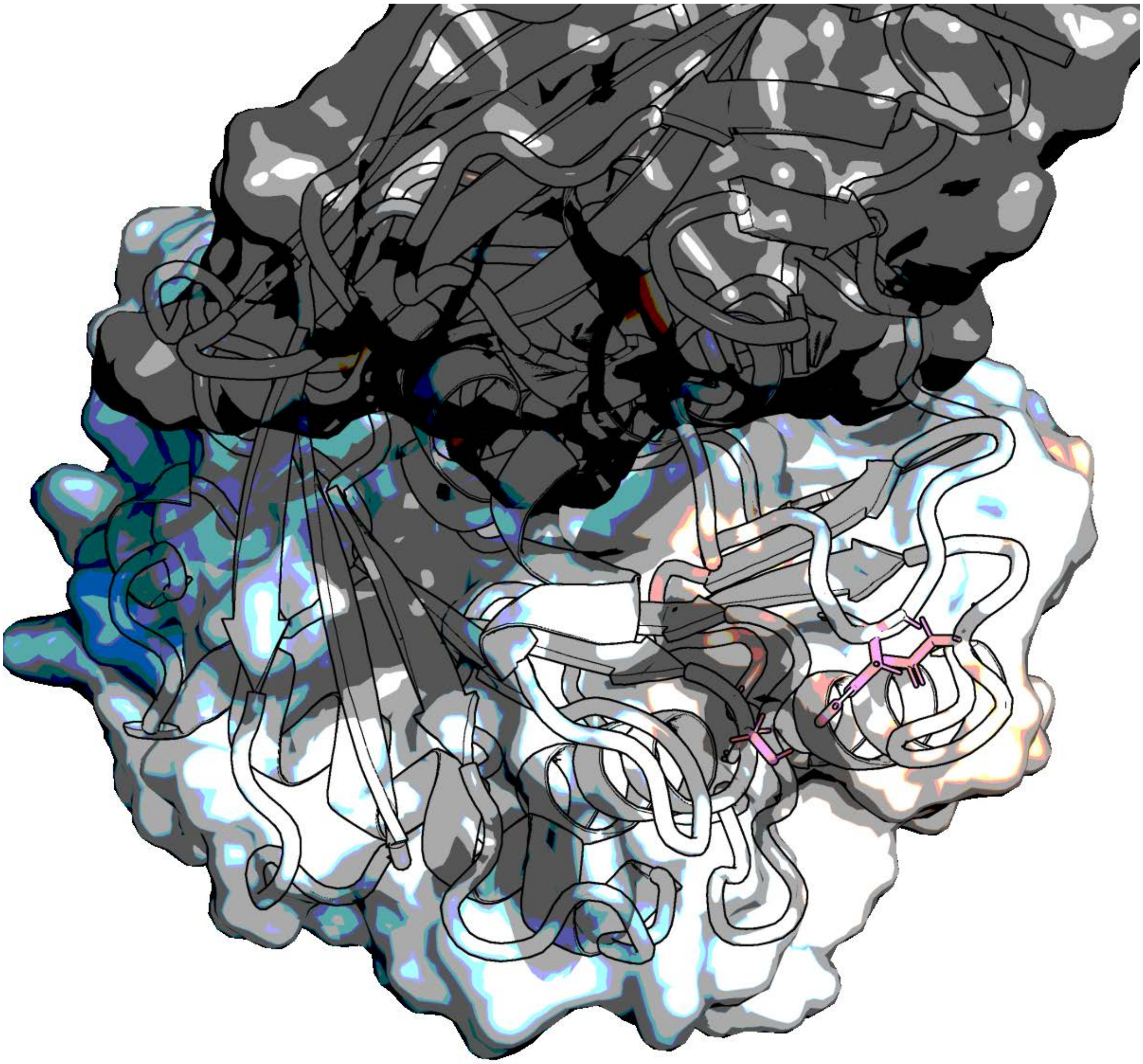

ESM018_highlights.png

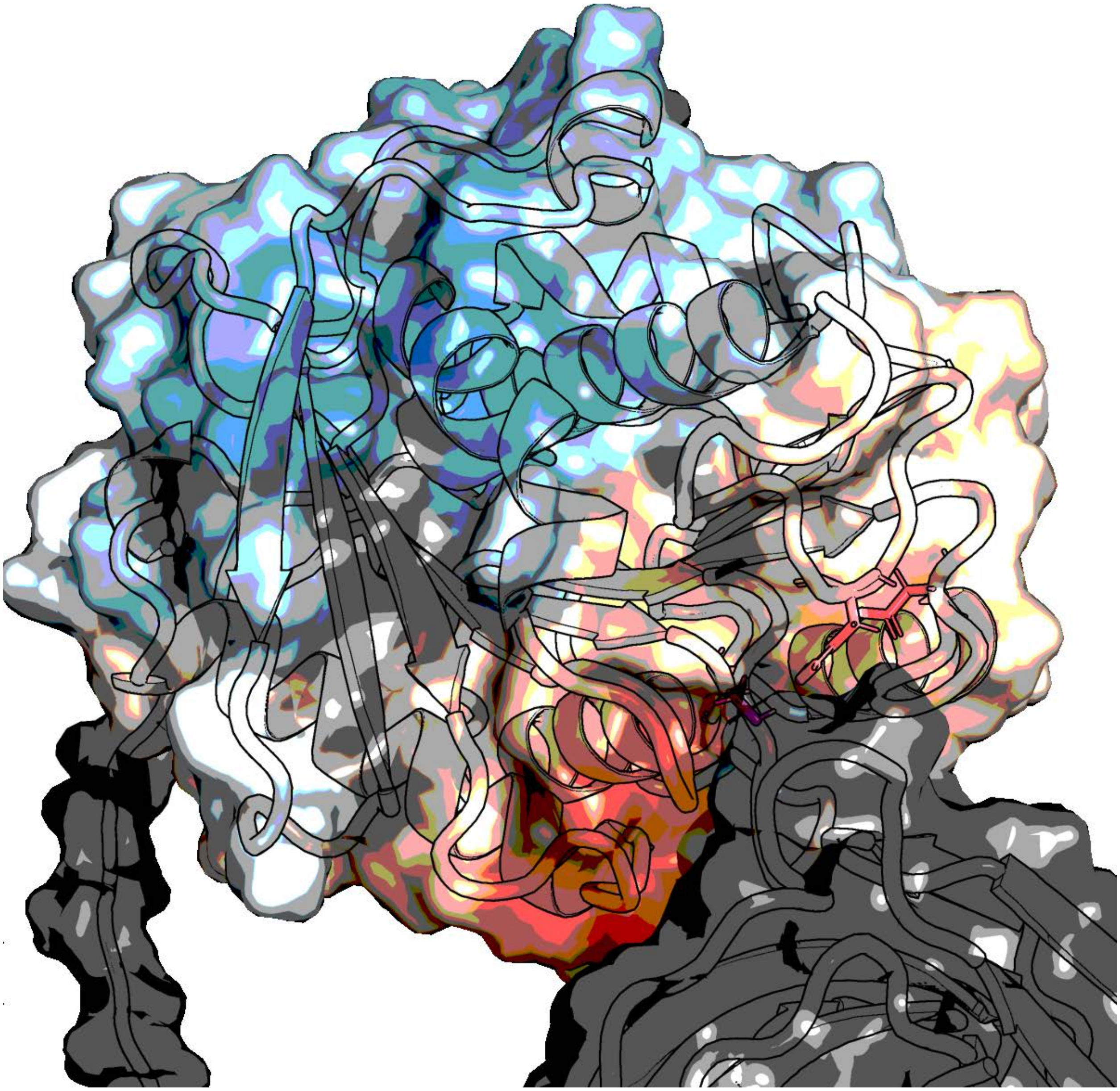

ESM021_highlights.png

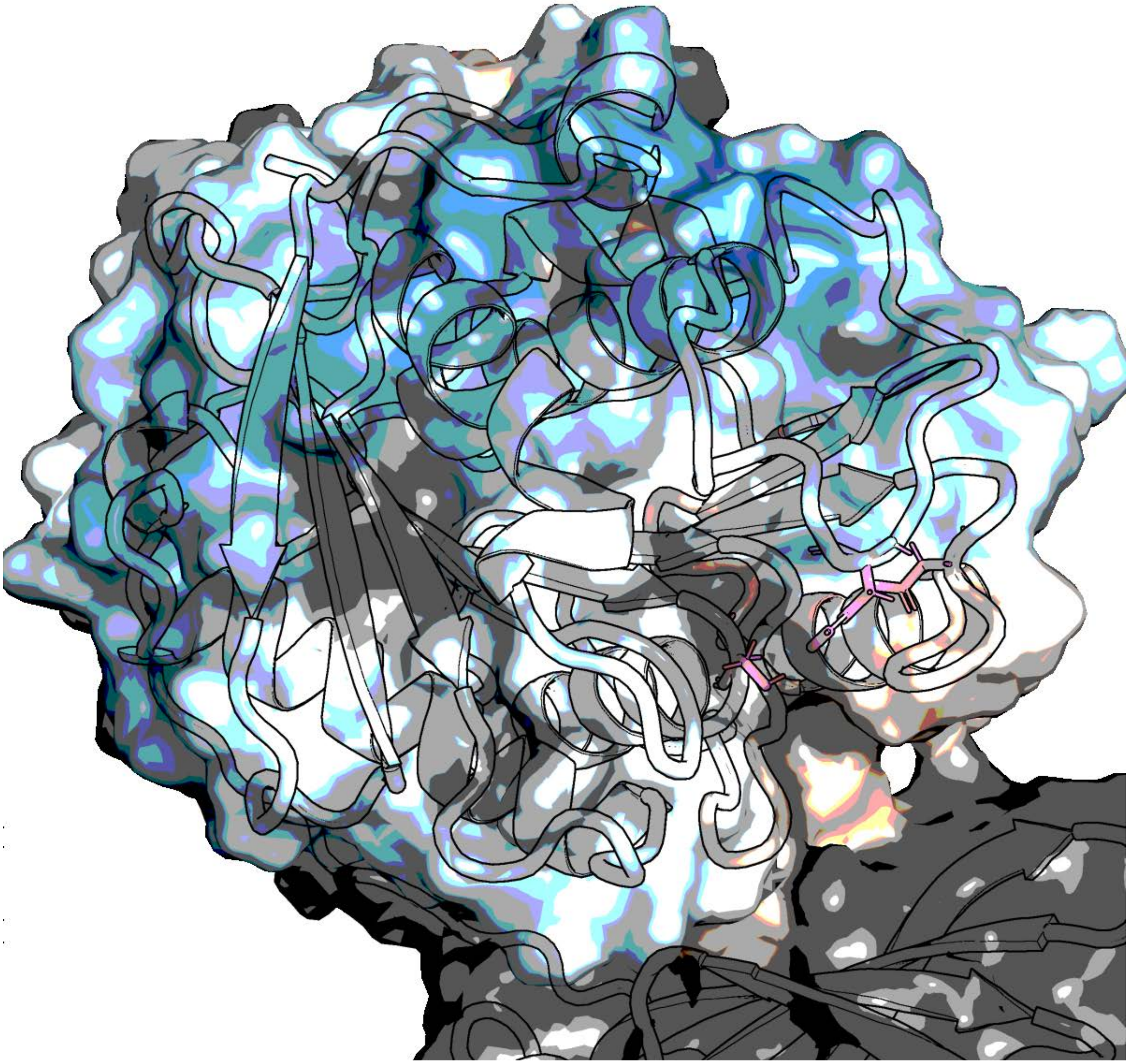

ESM023_highlights.png

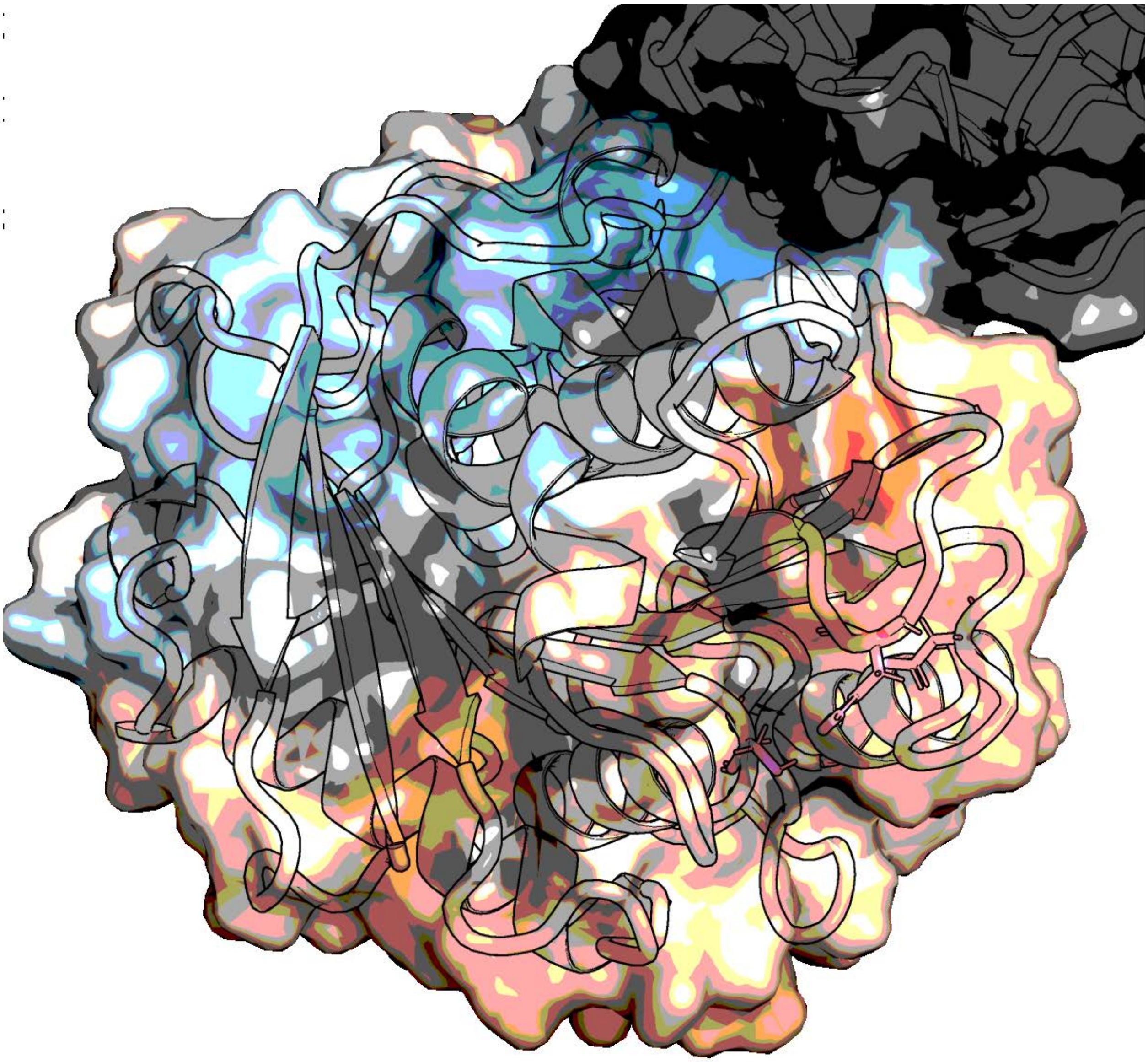

ESM026_highlights.png

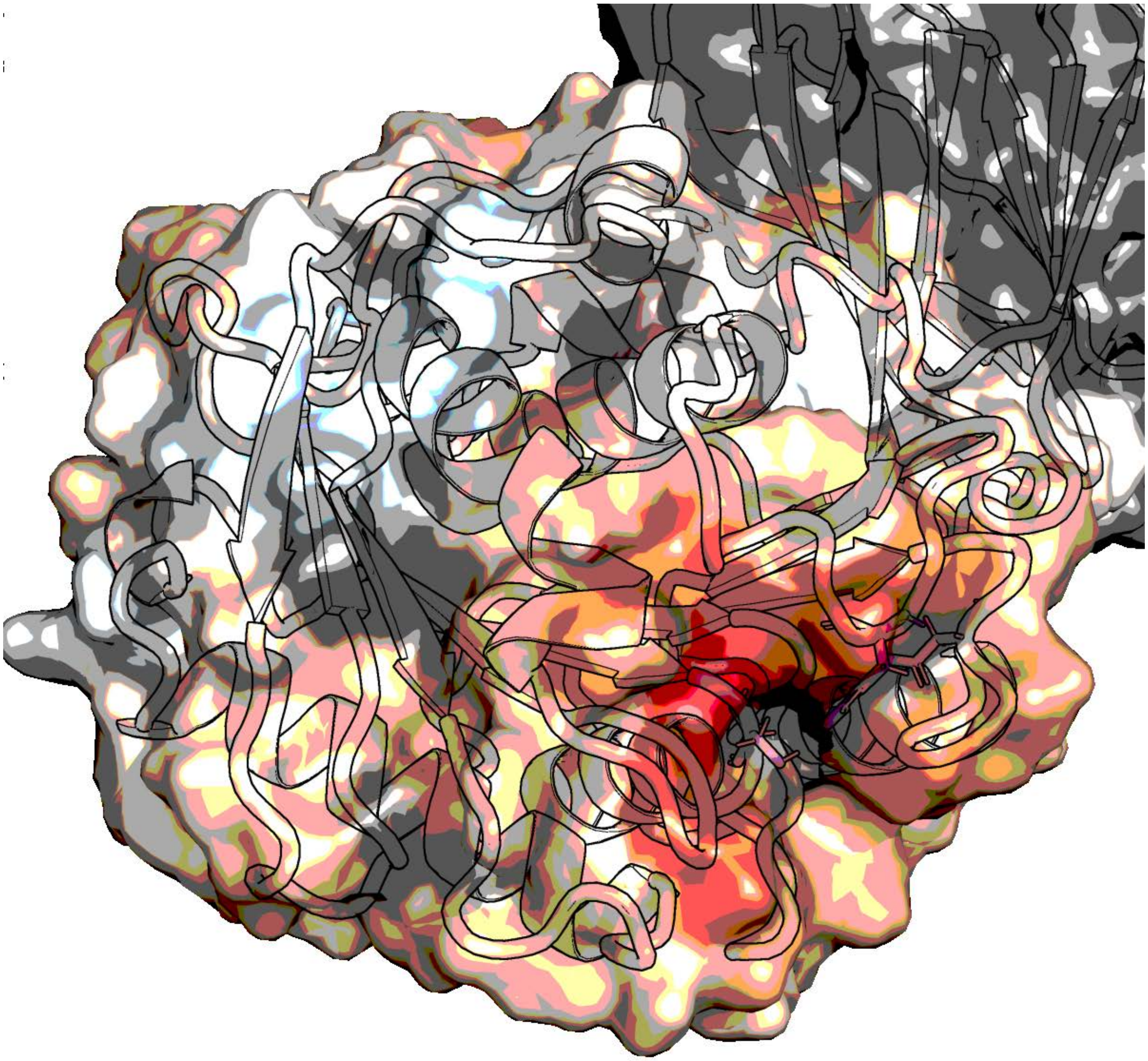

ESM033_highlights.png

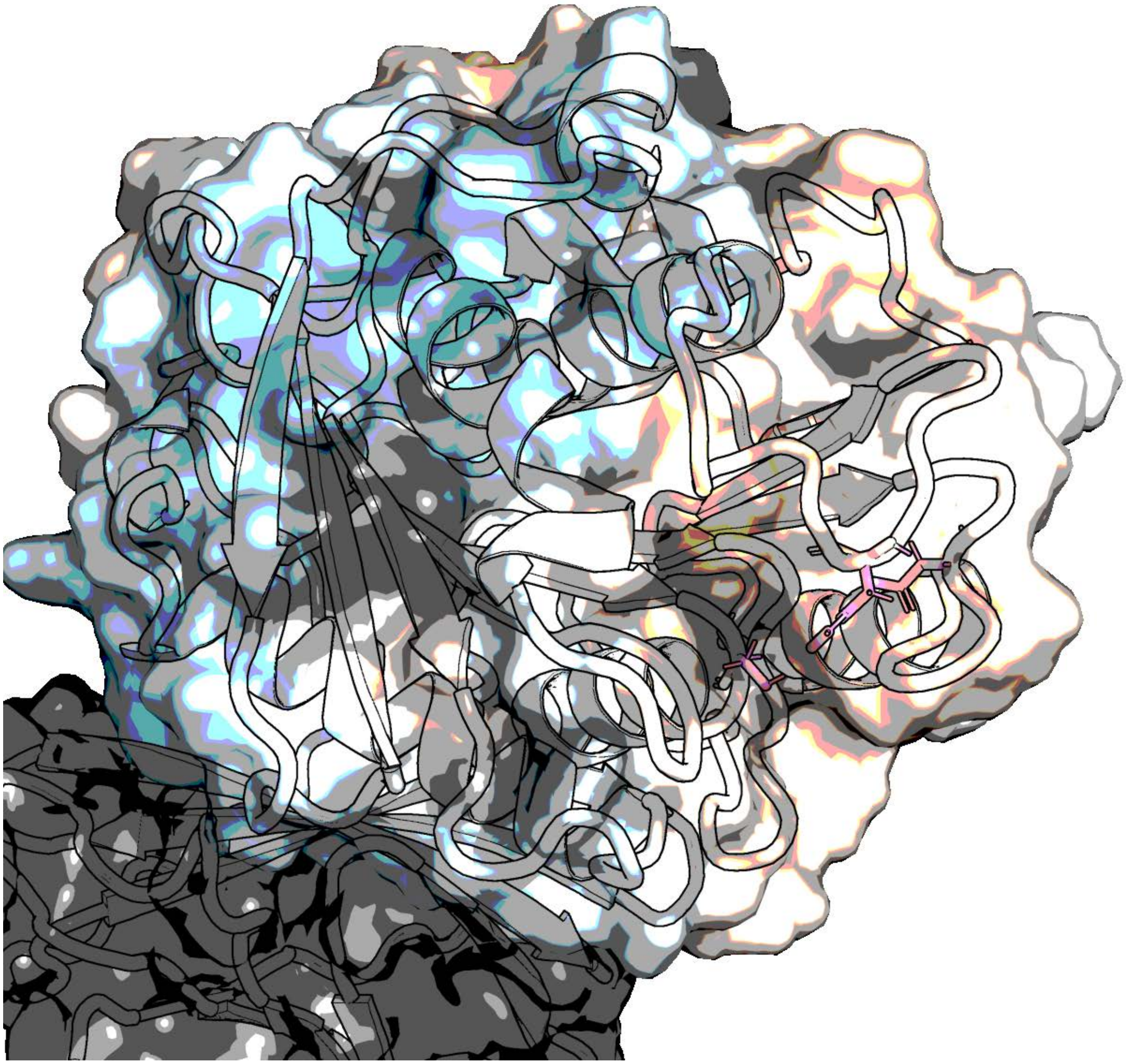

ESM035_highlights.png

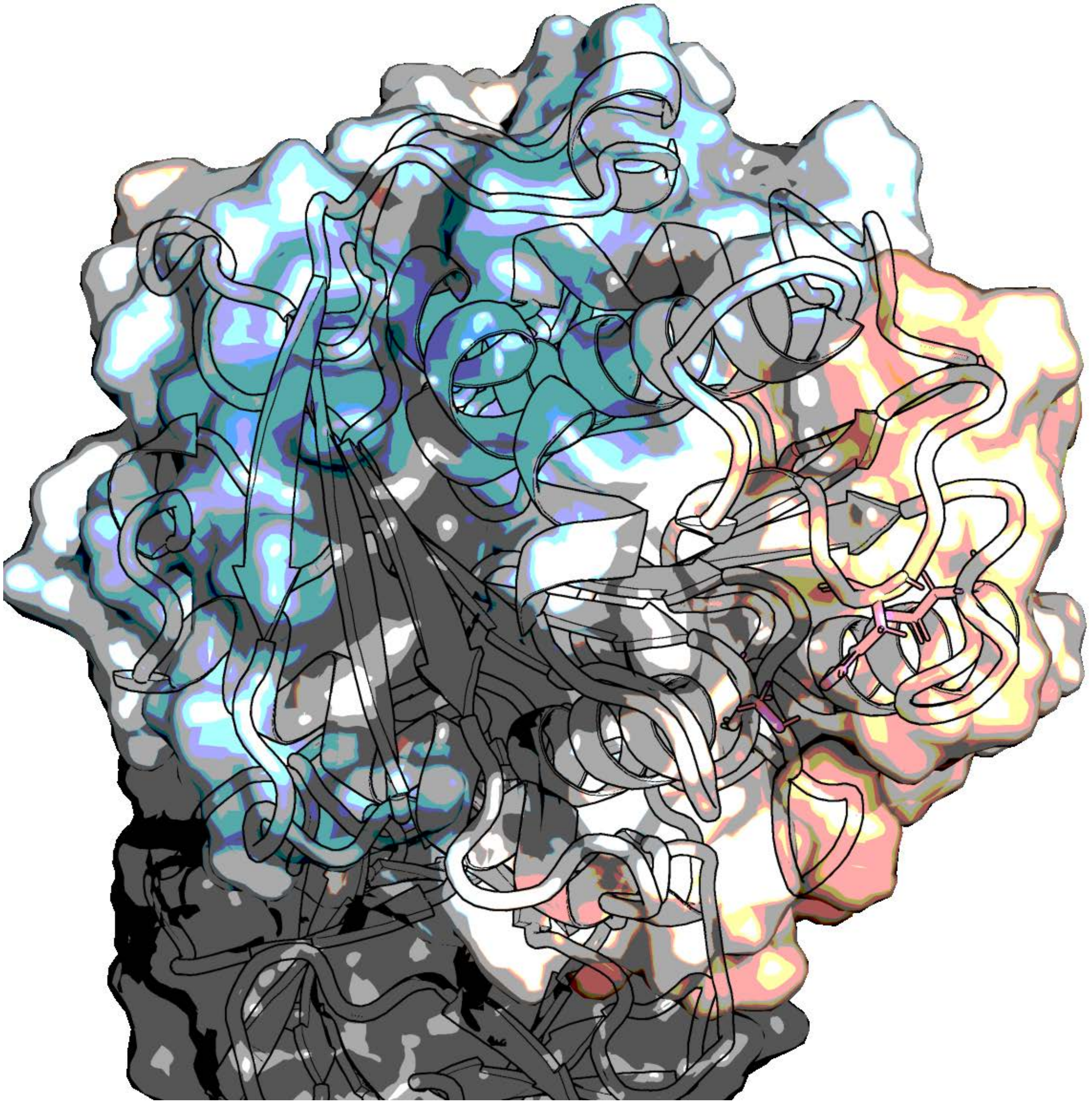

ESM038_highlights.png

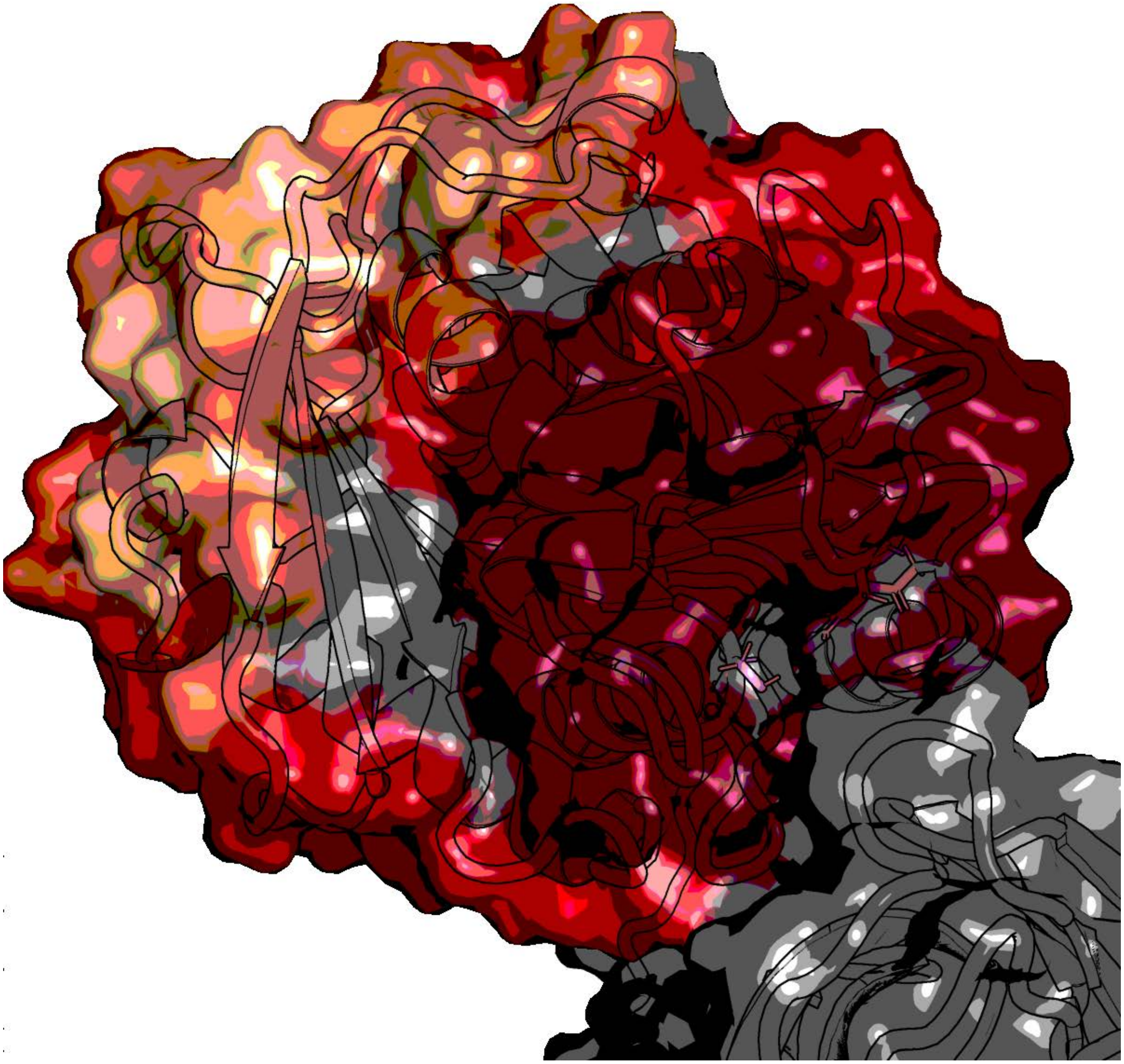

ESM043_highlights.png

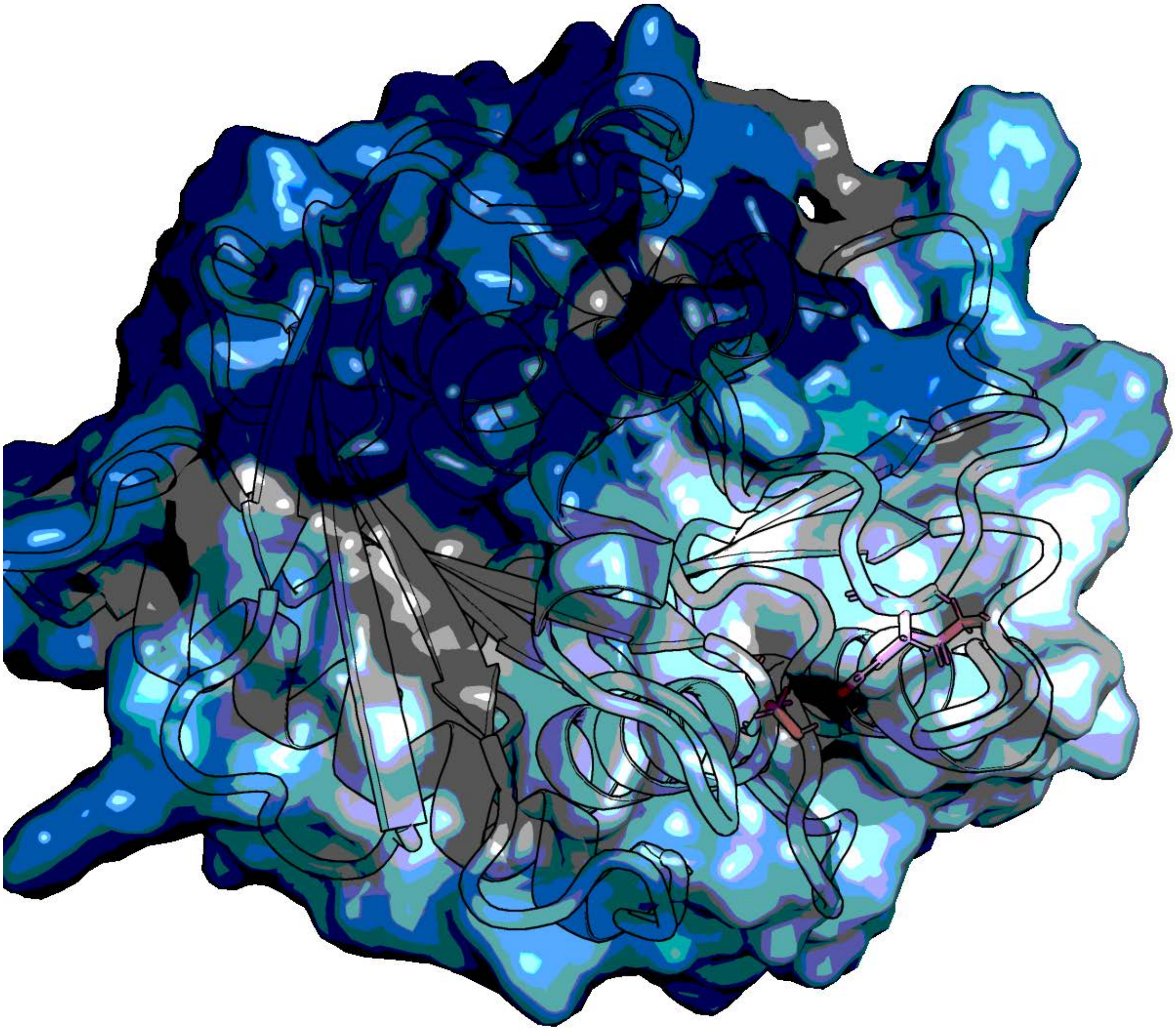

ESM045_highlights.png

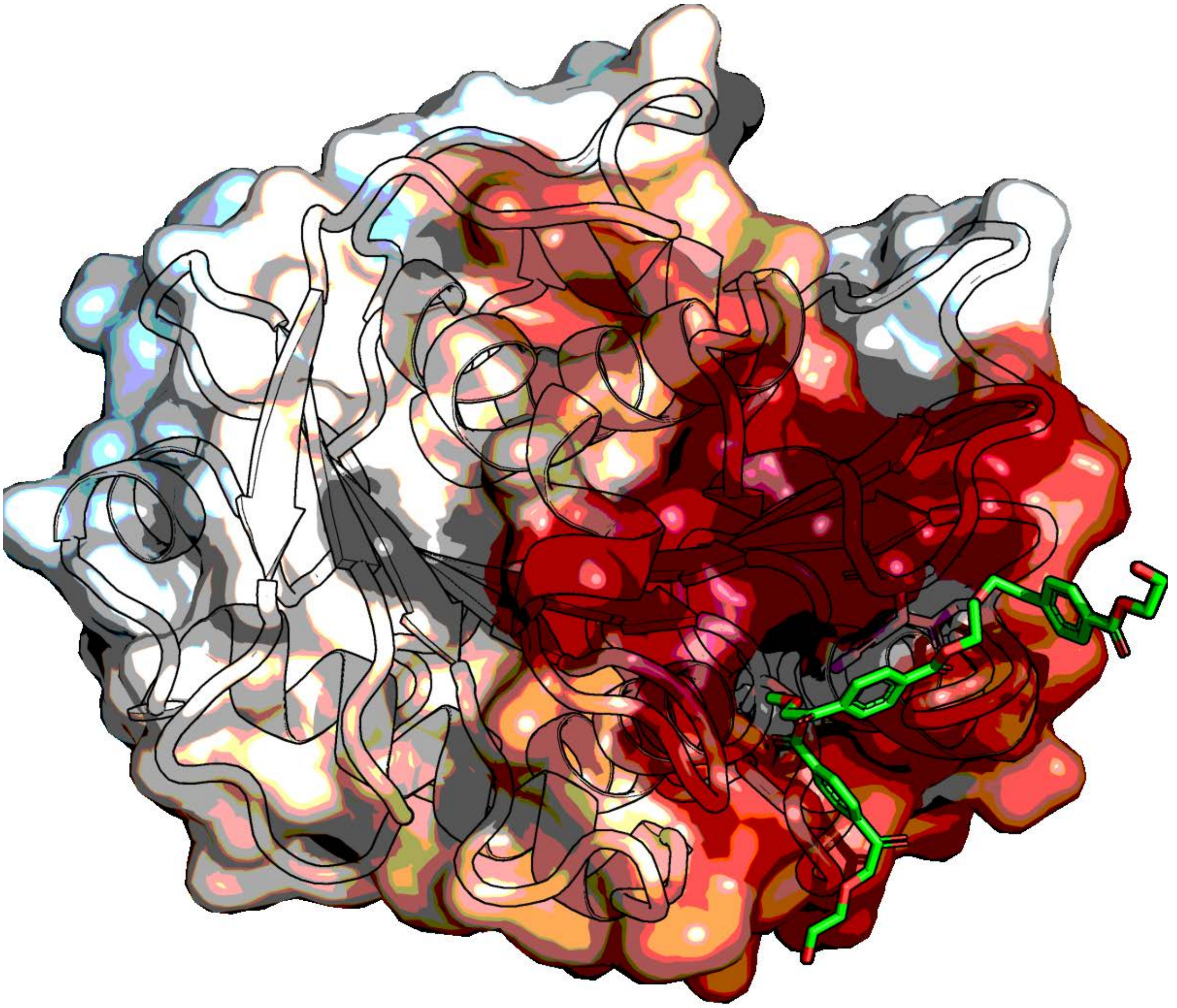

ESM047_highlights.png

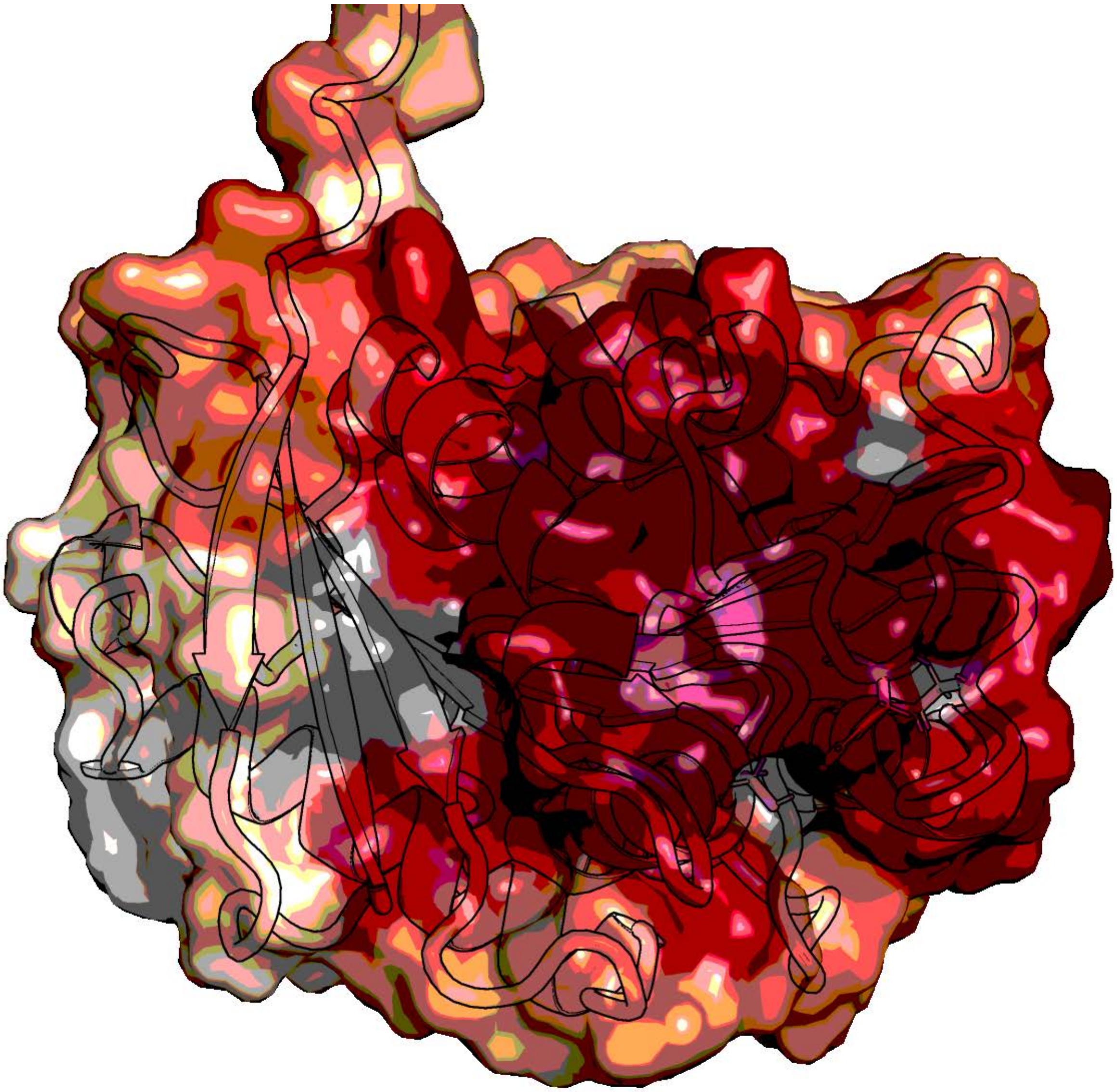

ESM050_highlights.png

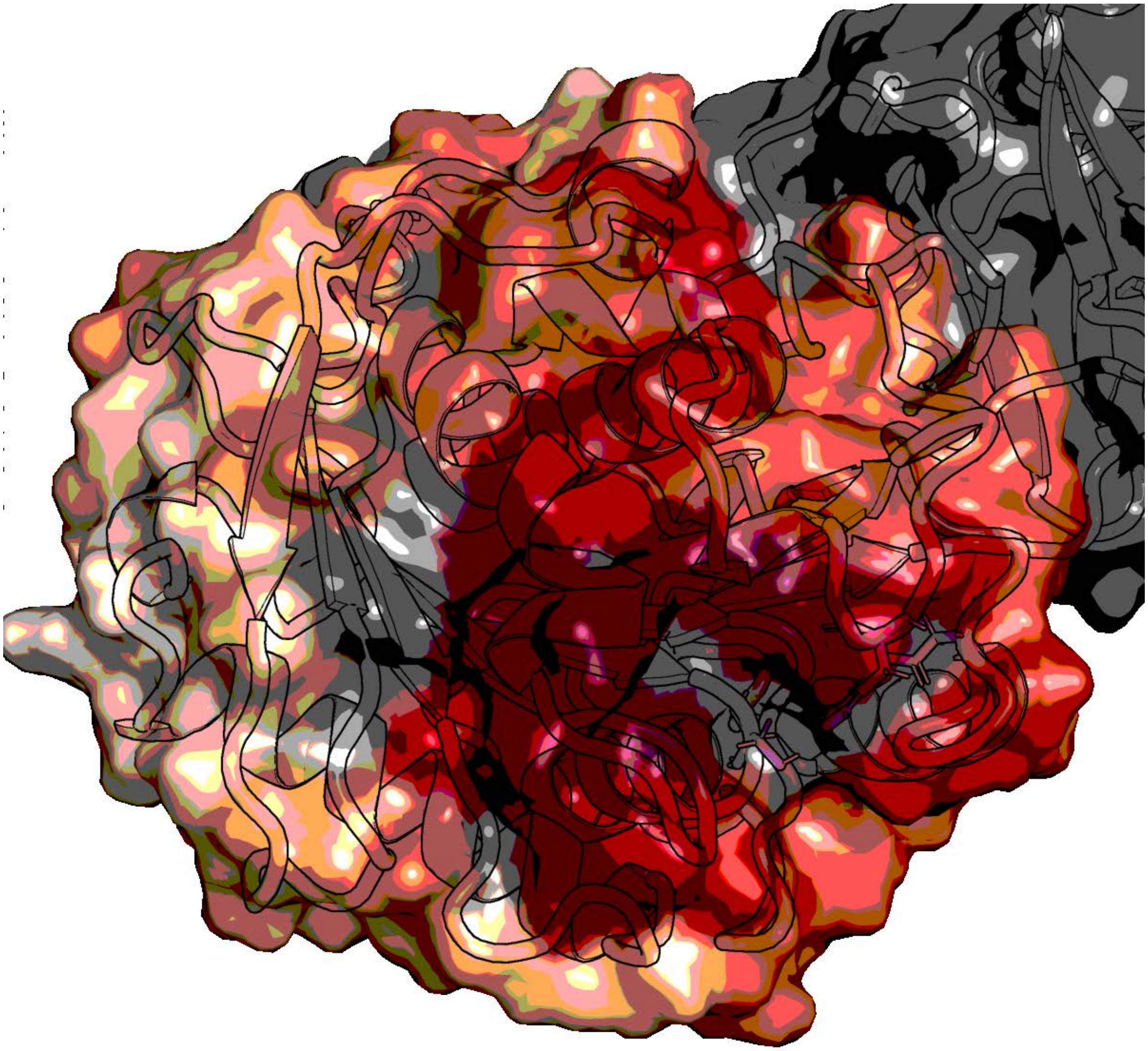

ESM052_highlights.png

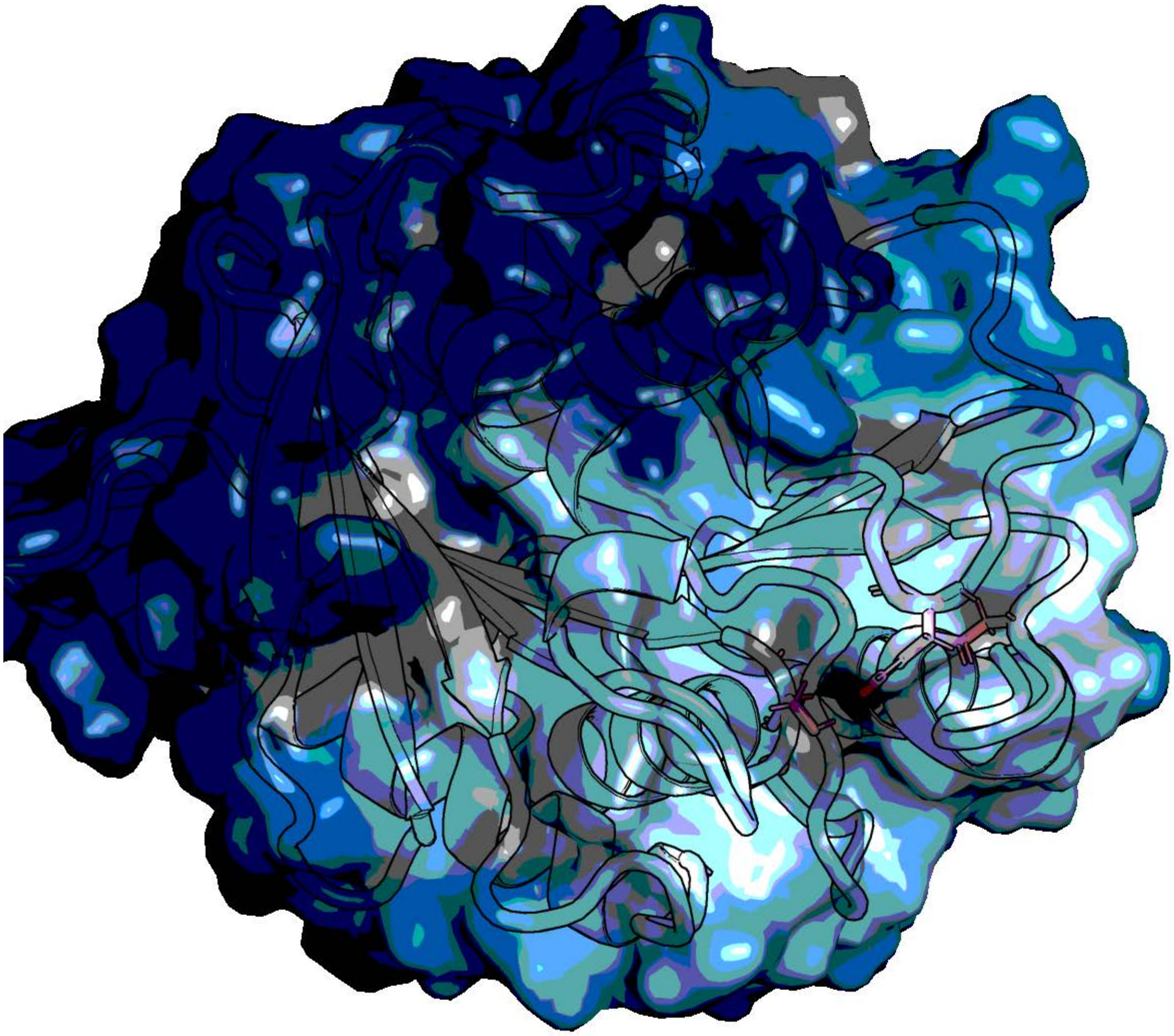

ESM053_highlights.png

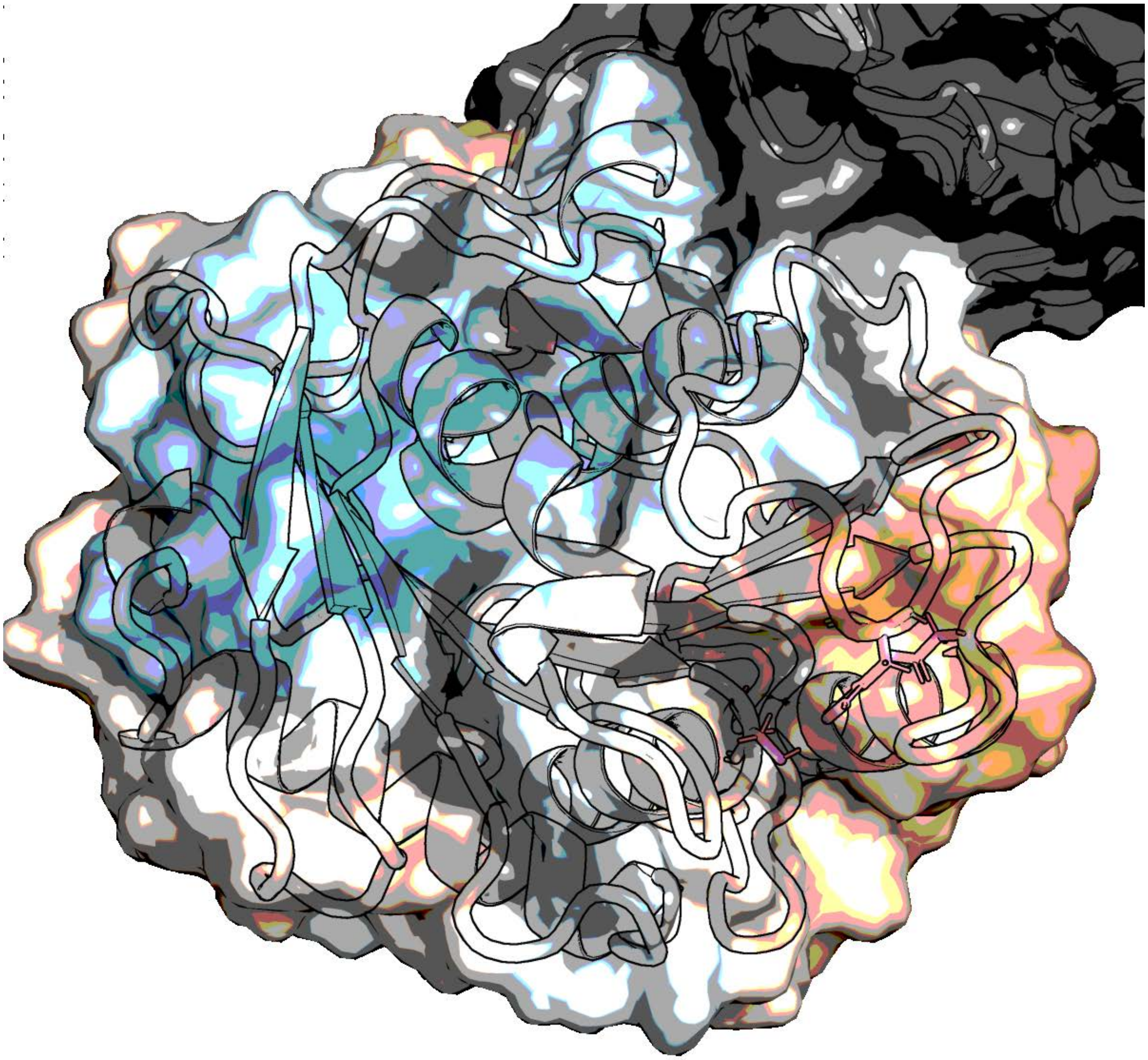

ESM058_highlights.png

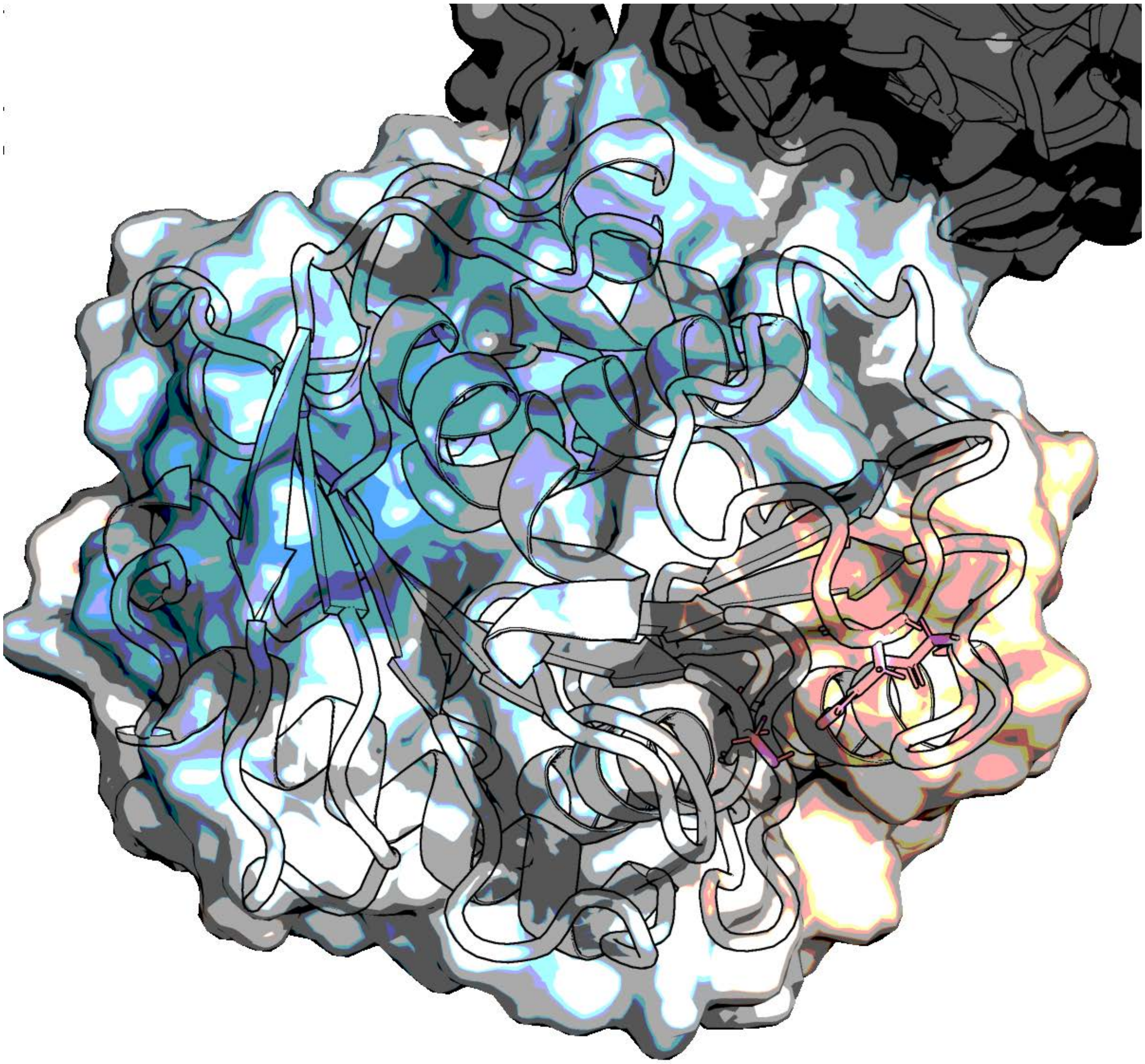

ESM059_highlights.png

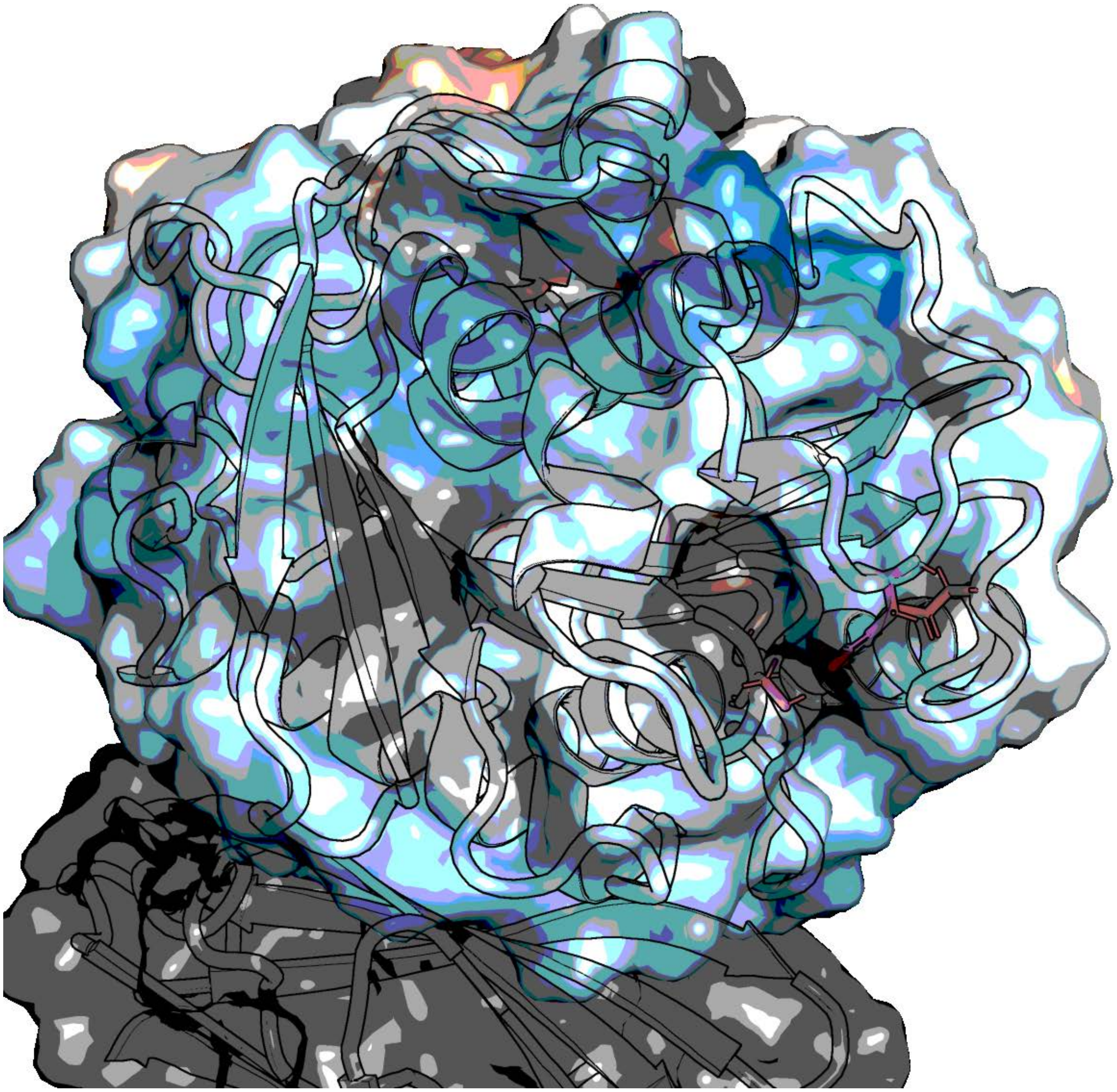

ESM060_highlights.png

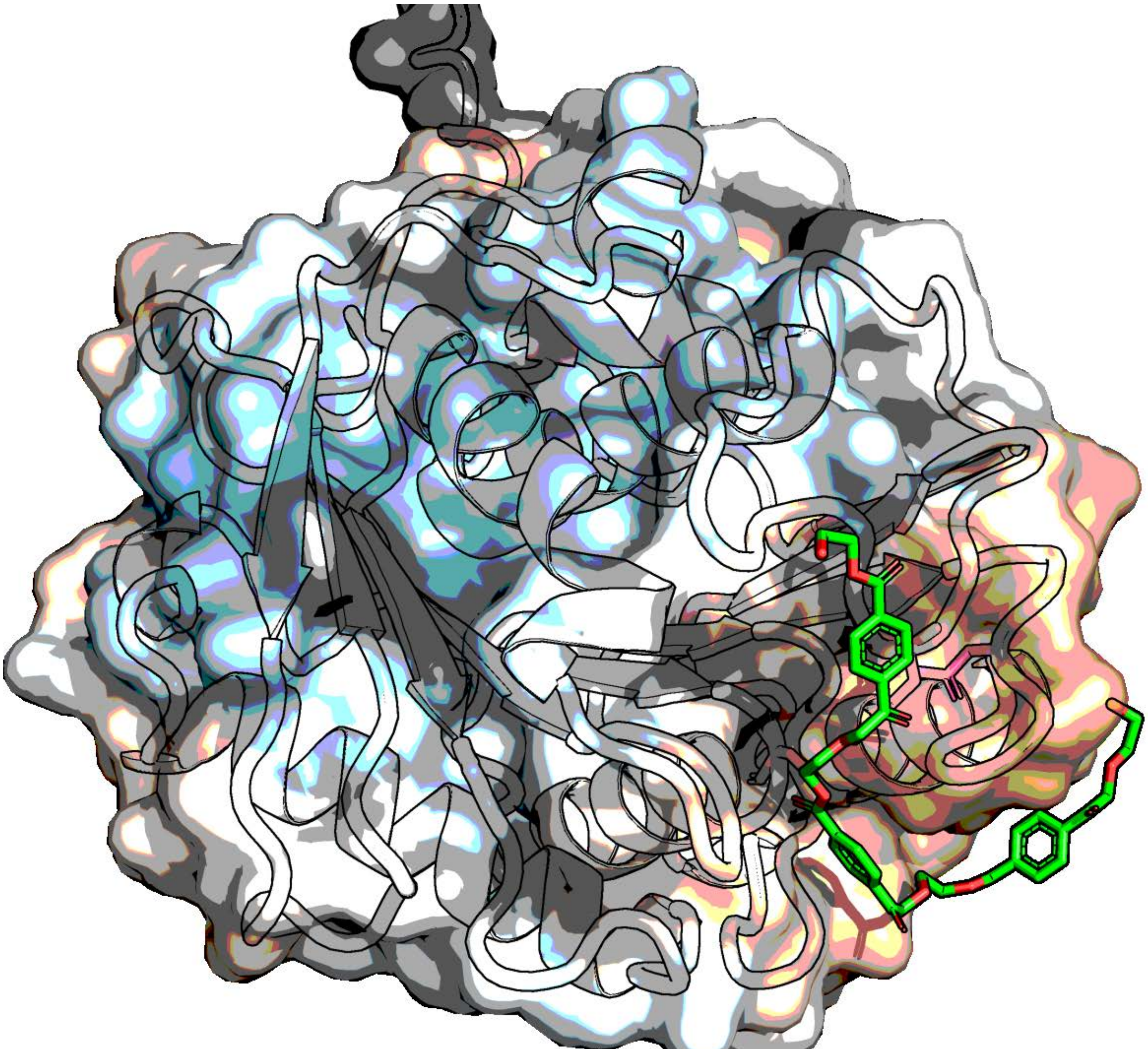

ESM061_highlights.png

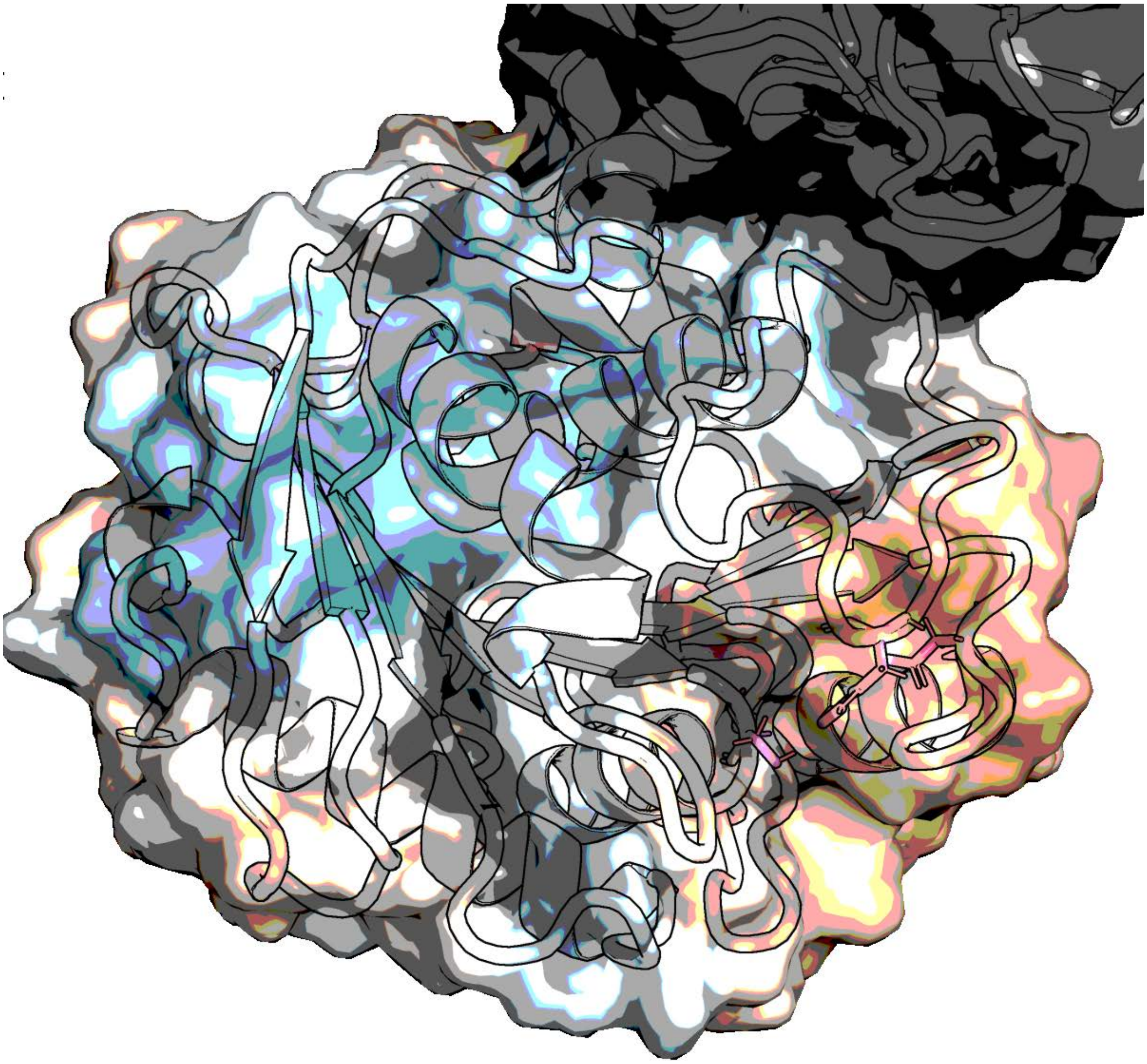

ESM062_highlights.png

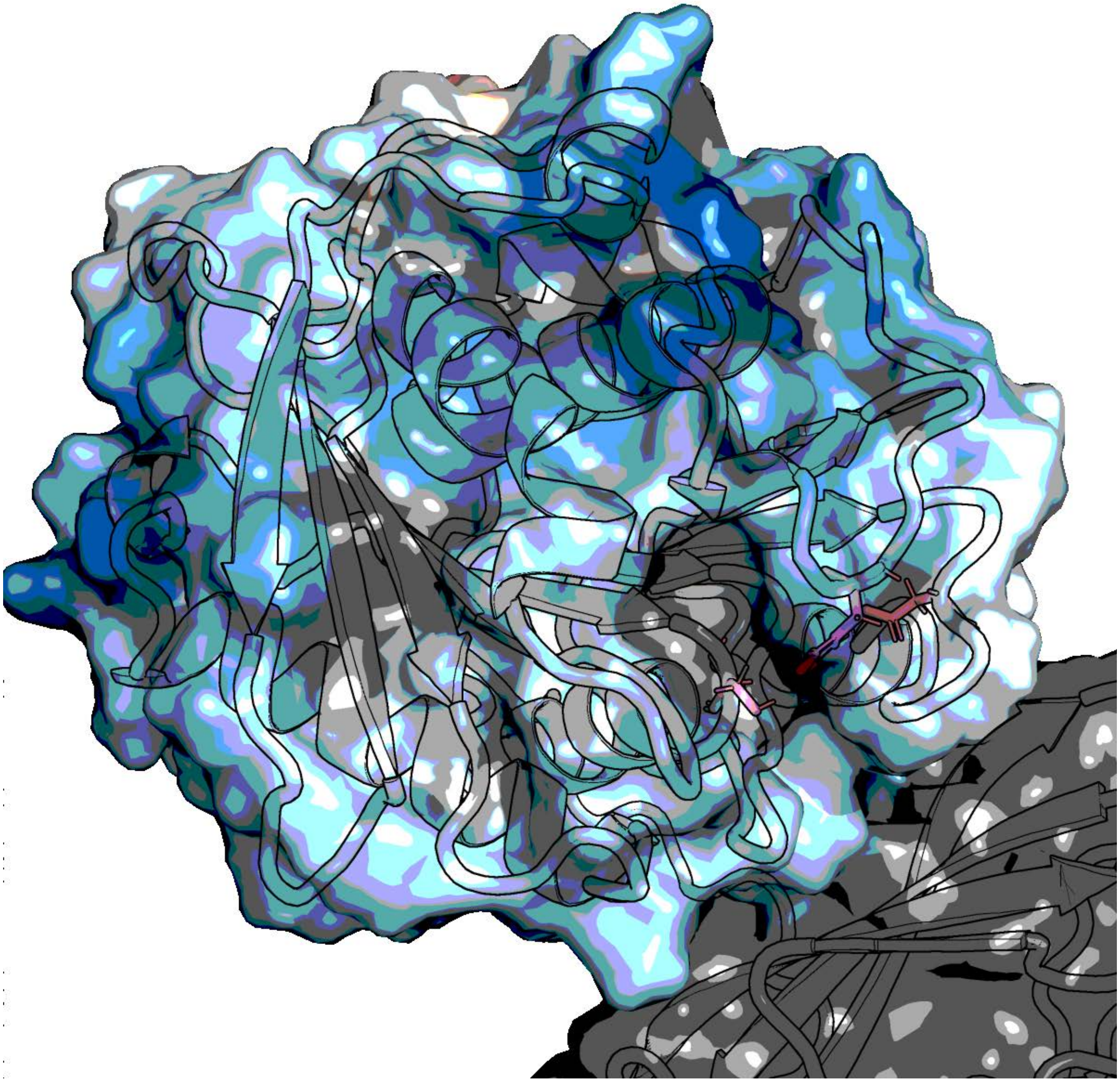

ESM064_highlights.png

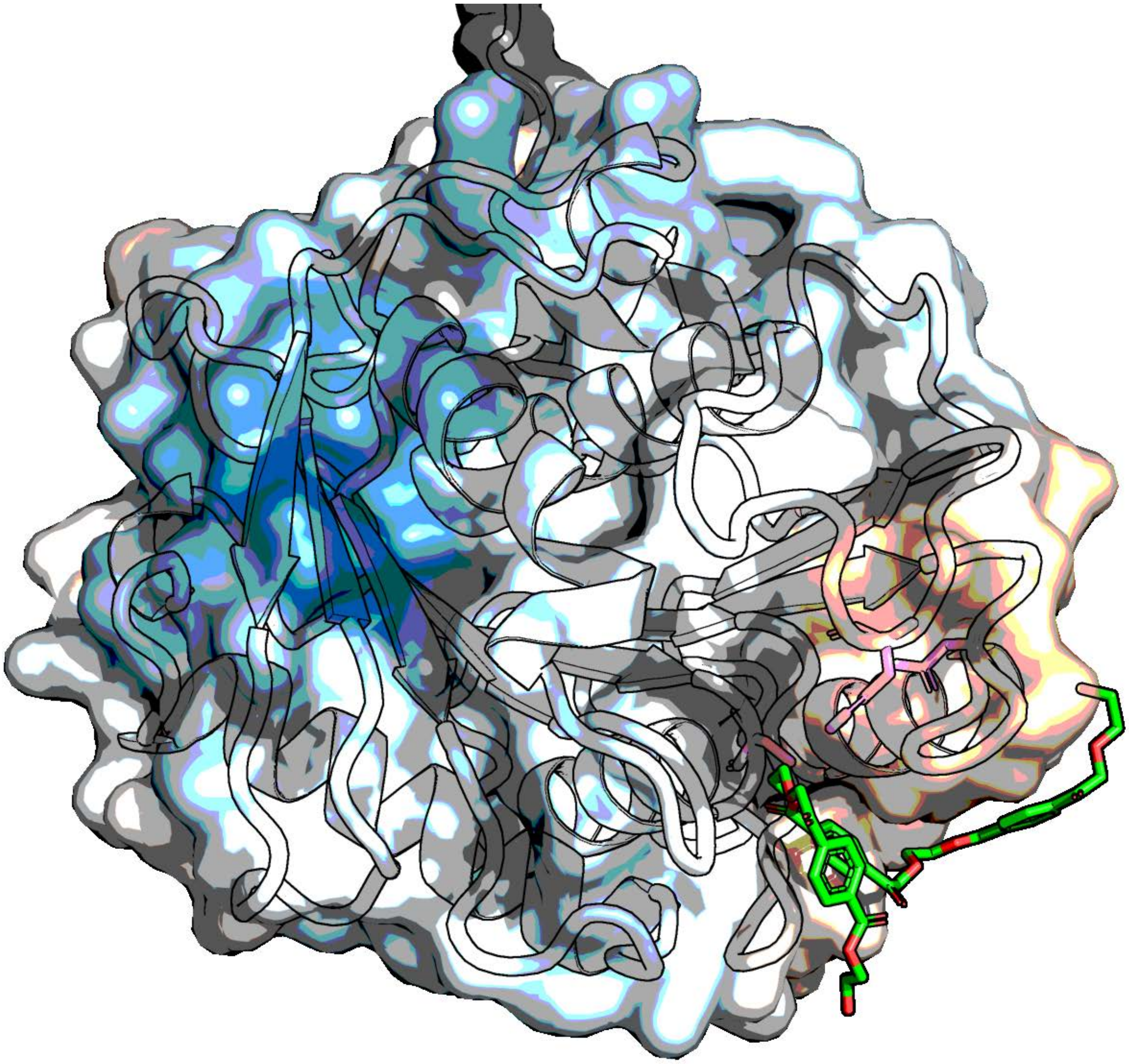

ESM065_highlights.png

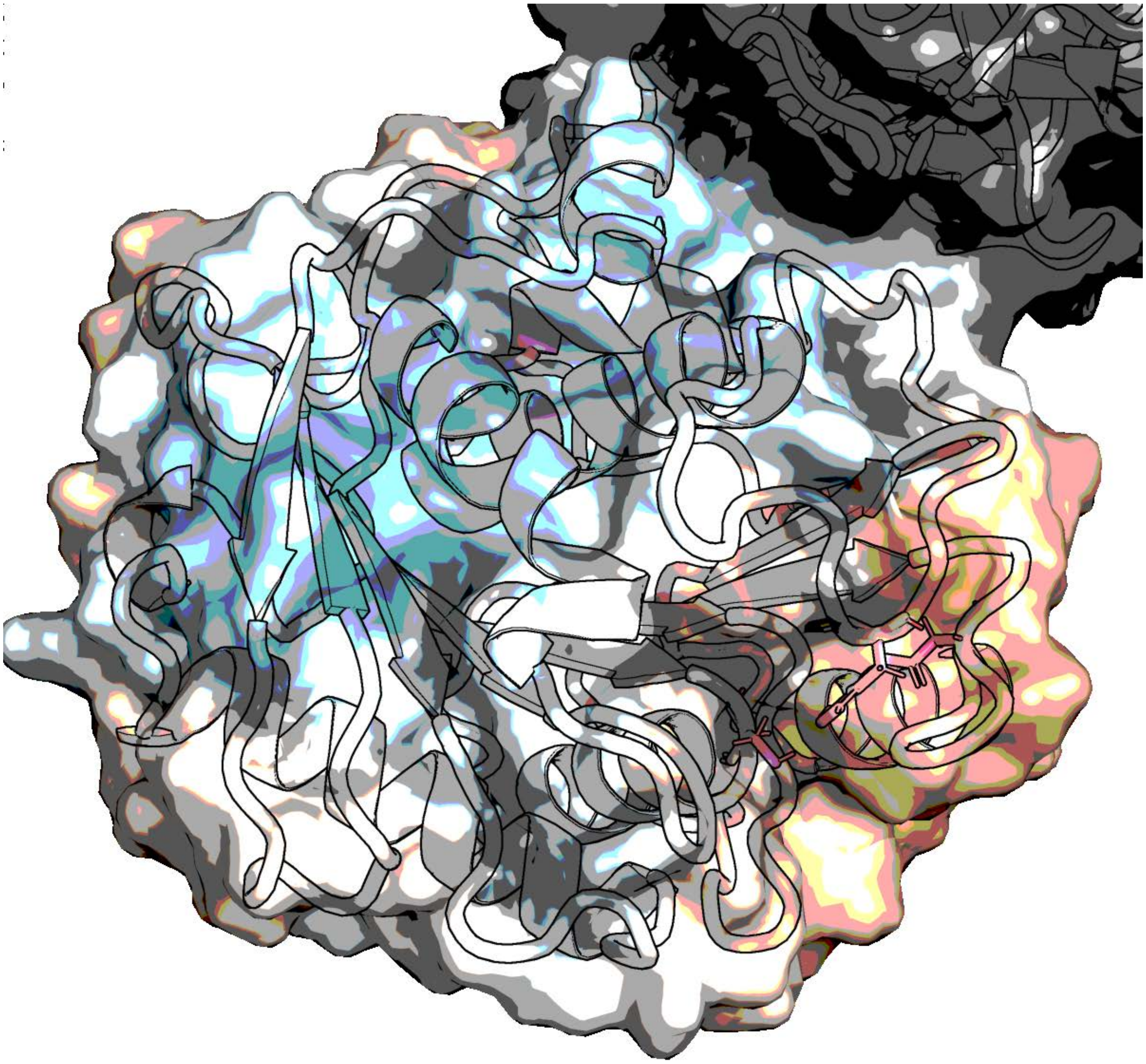

ESM069_highlights.png

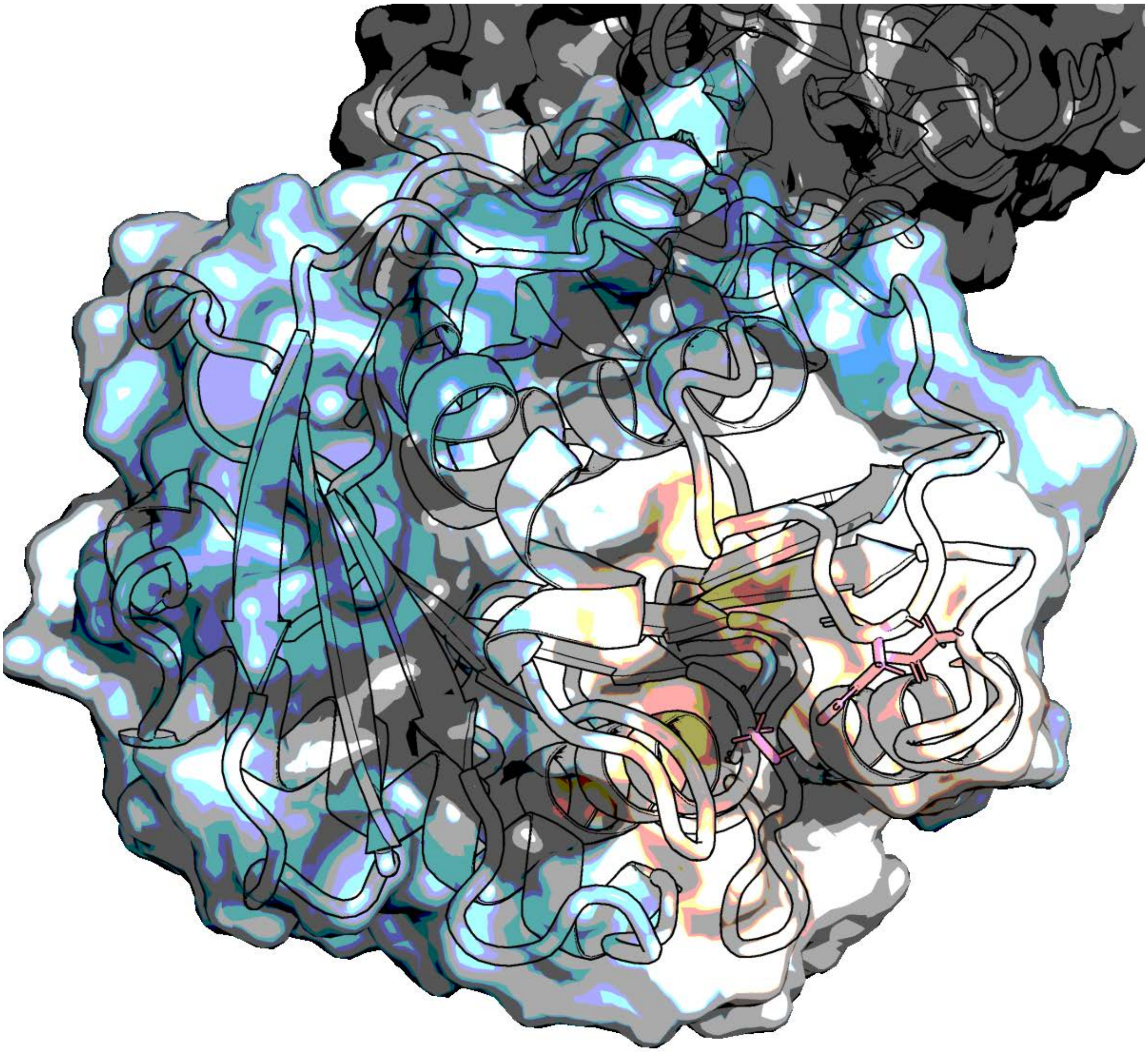

ESM070_highlights.png

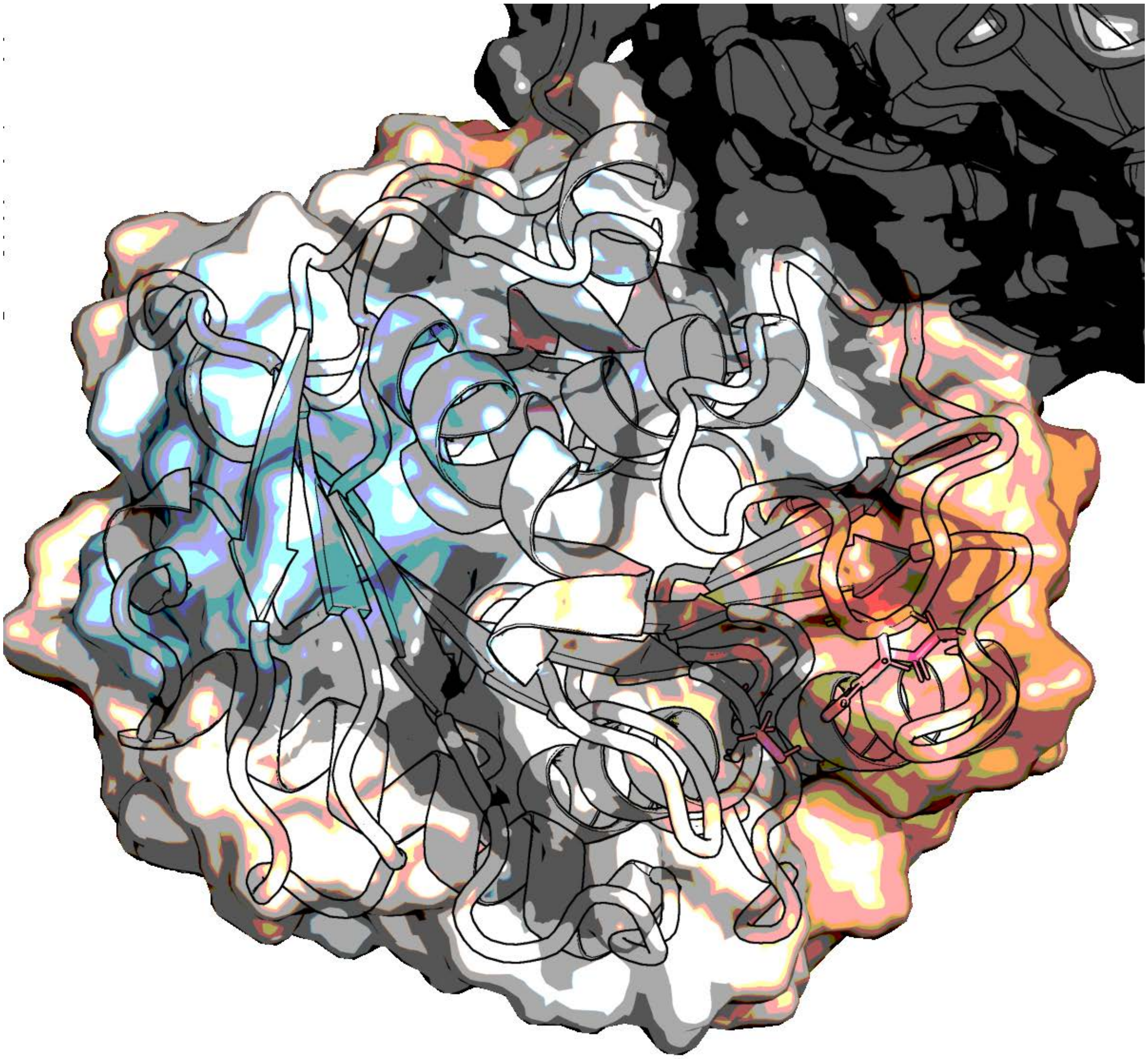

ESM075_highlights.png

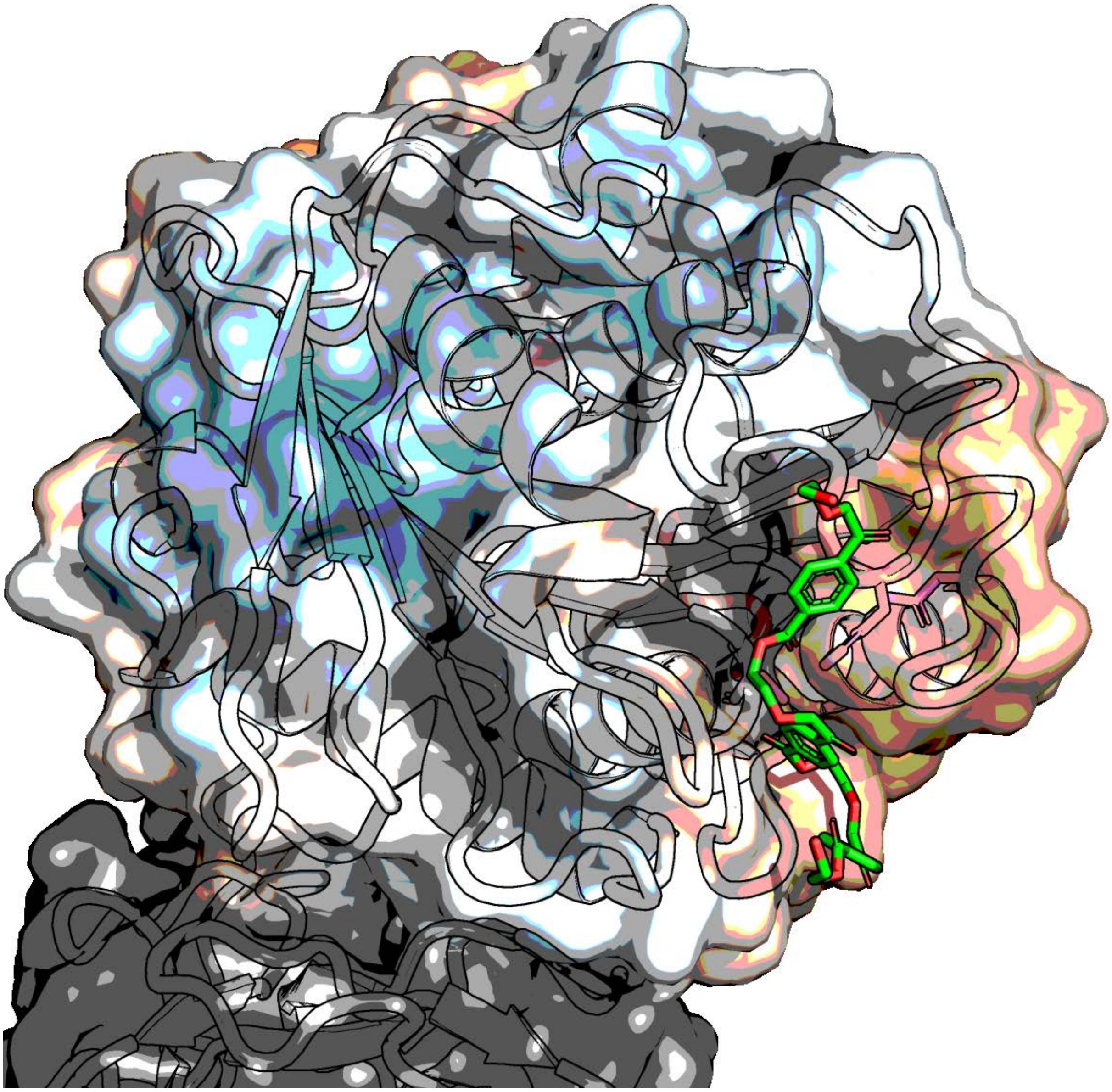

ESM080_highlights.png

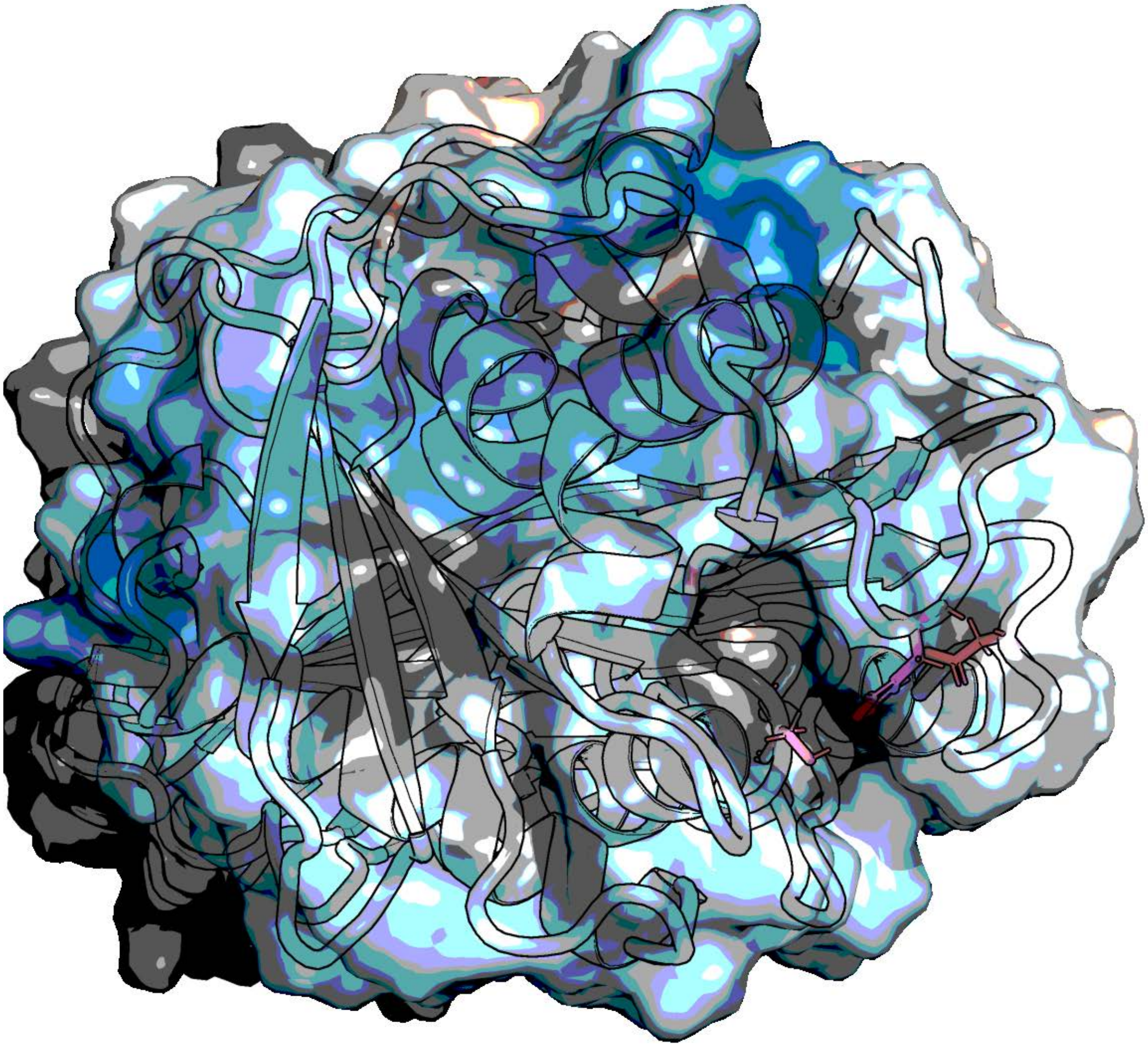

ESM081_highlights.png

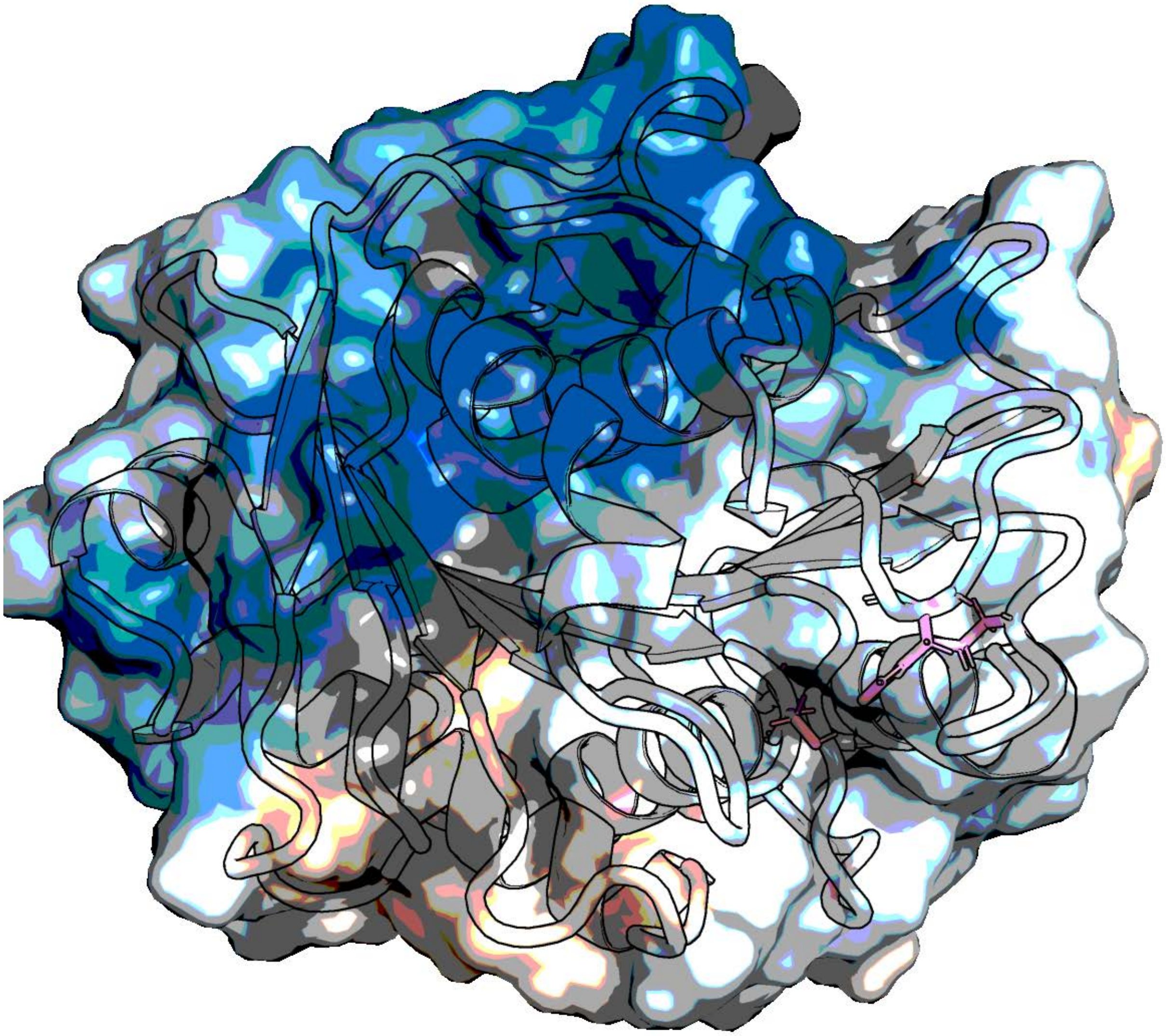

ESM082_highlights.png

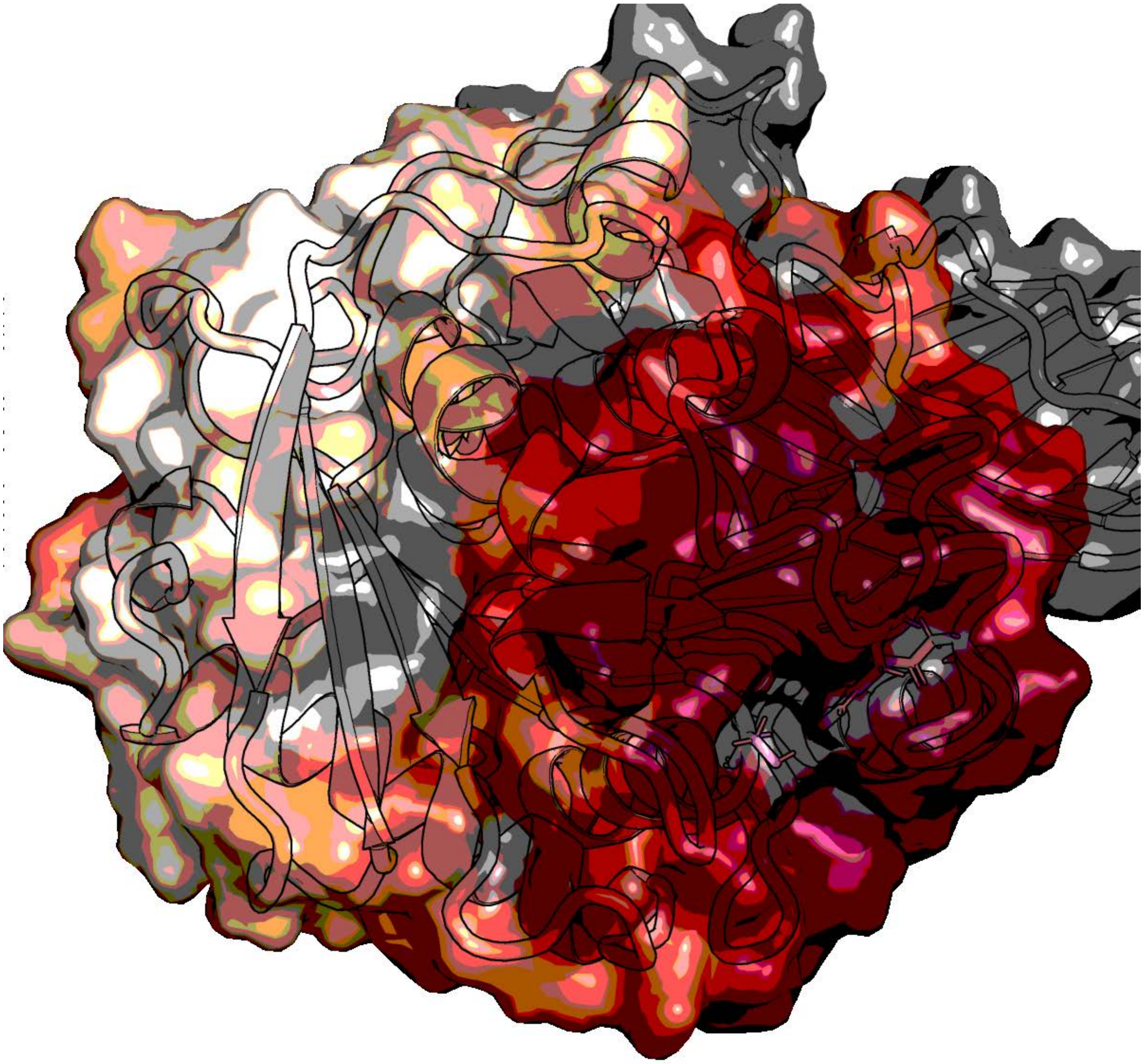

ESM084_highlights.png

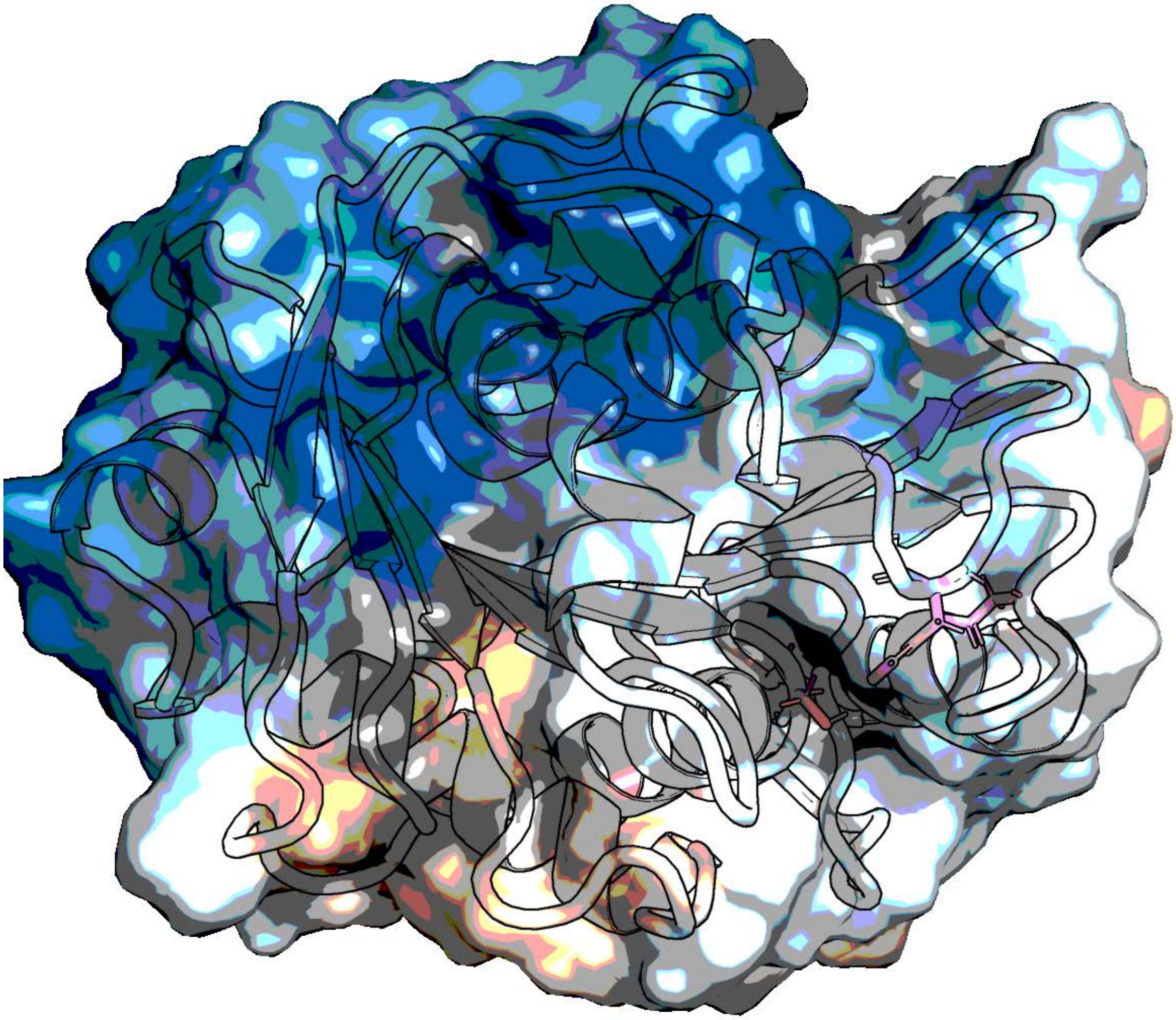

ESM090_highlights.png

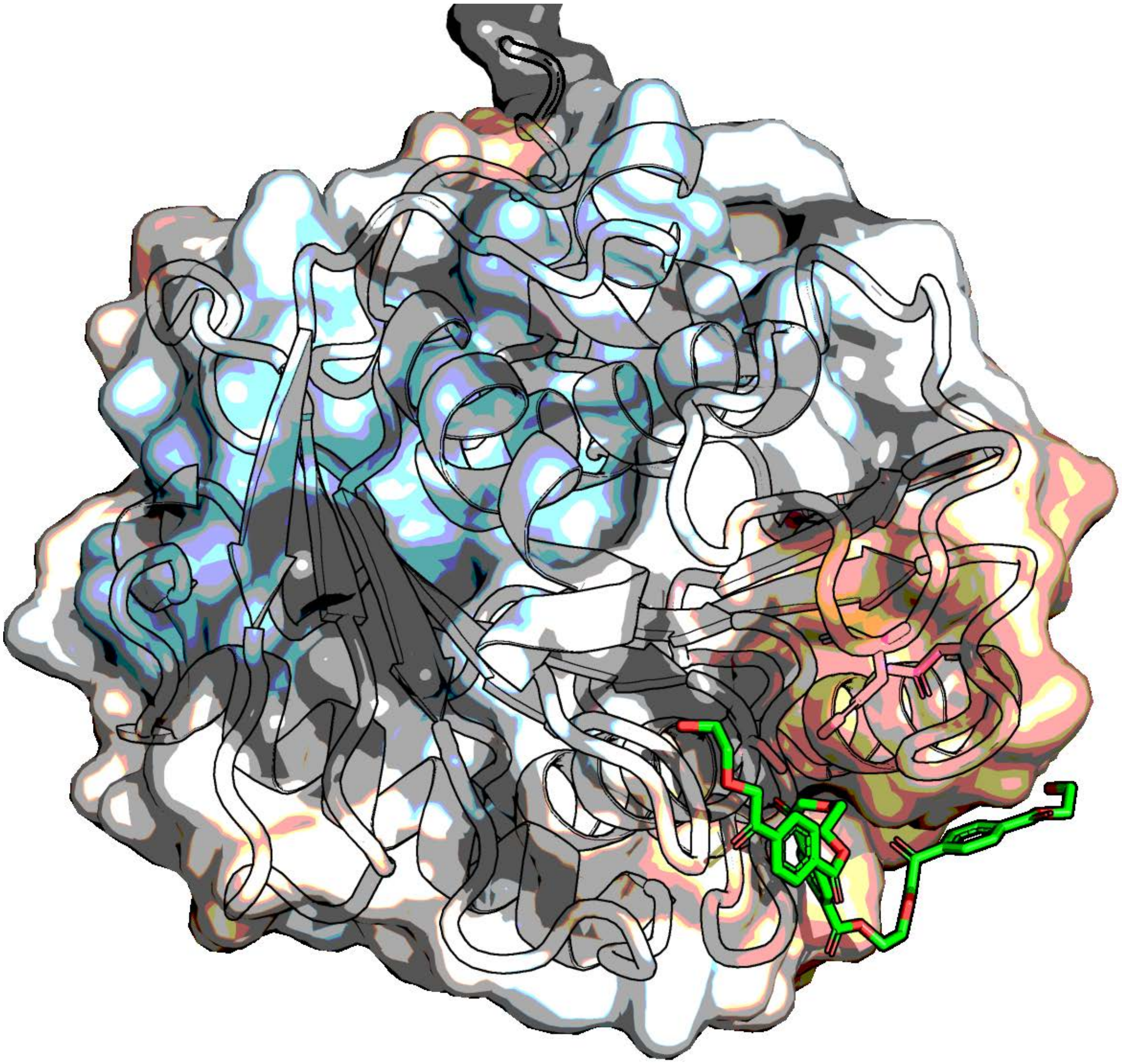

ESM091_highlights.png

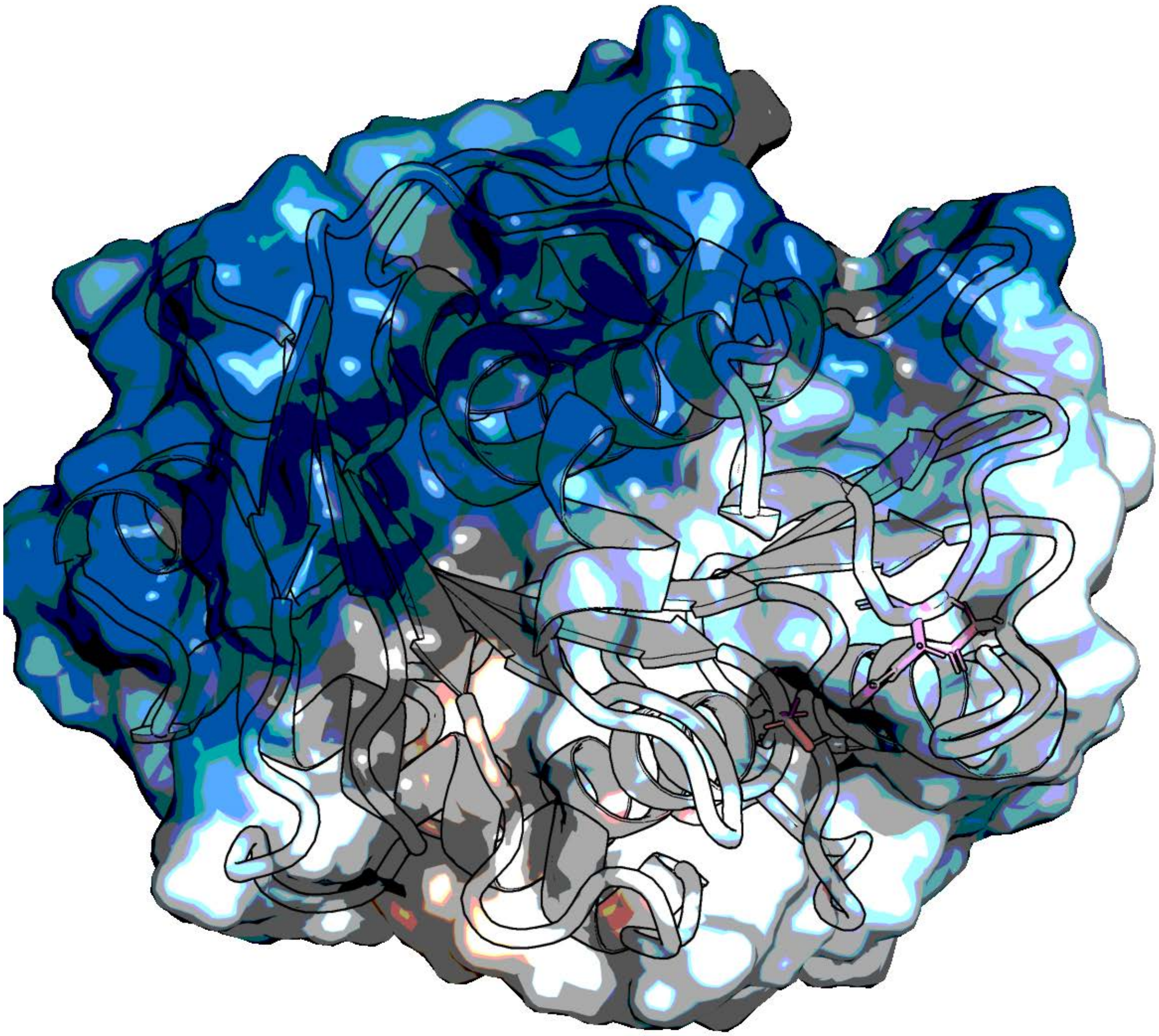

ESM093_highlights.png

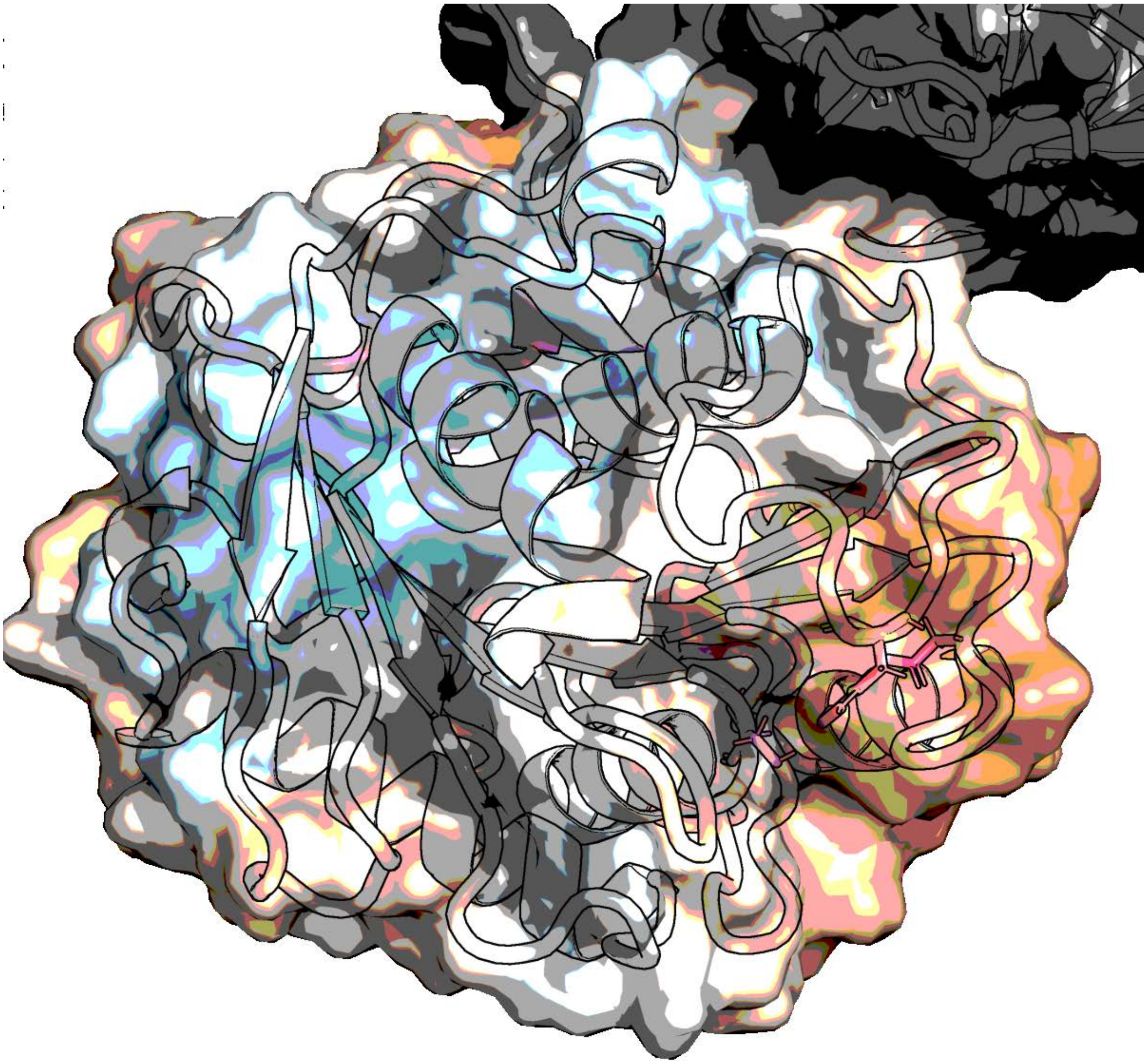

ESM095_highlights.png

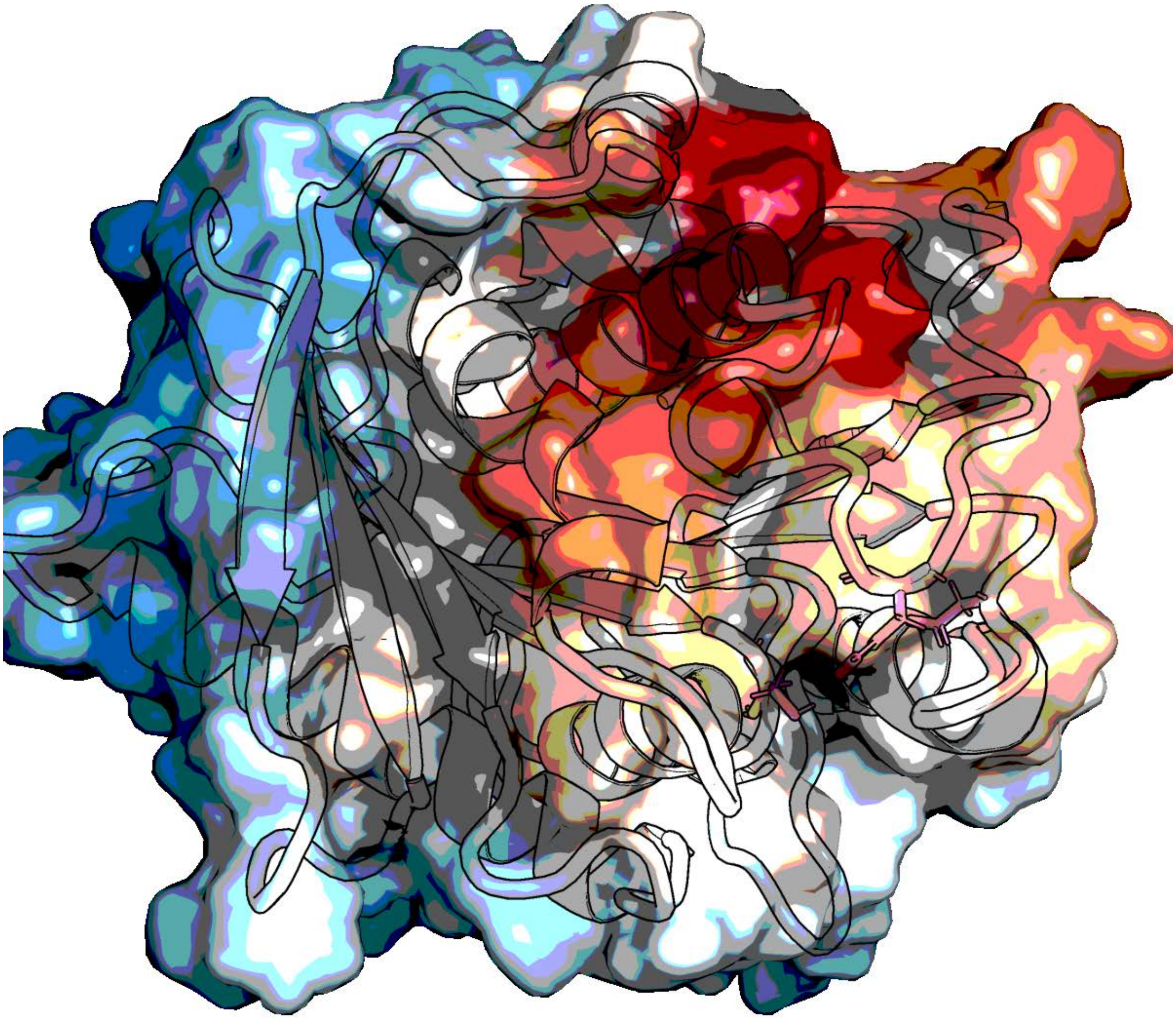

ESM096_highlights.png

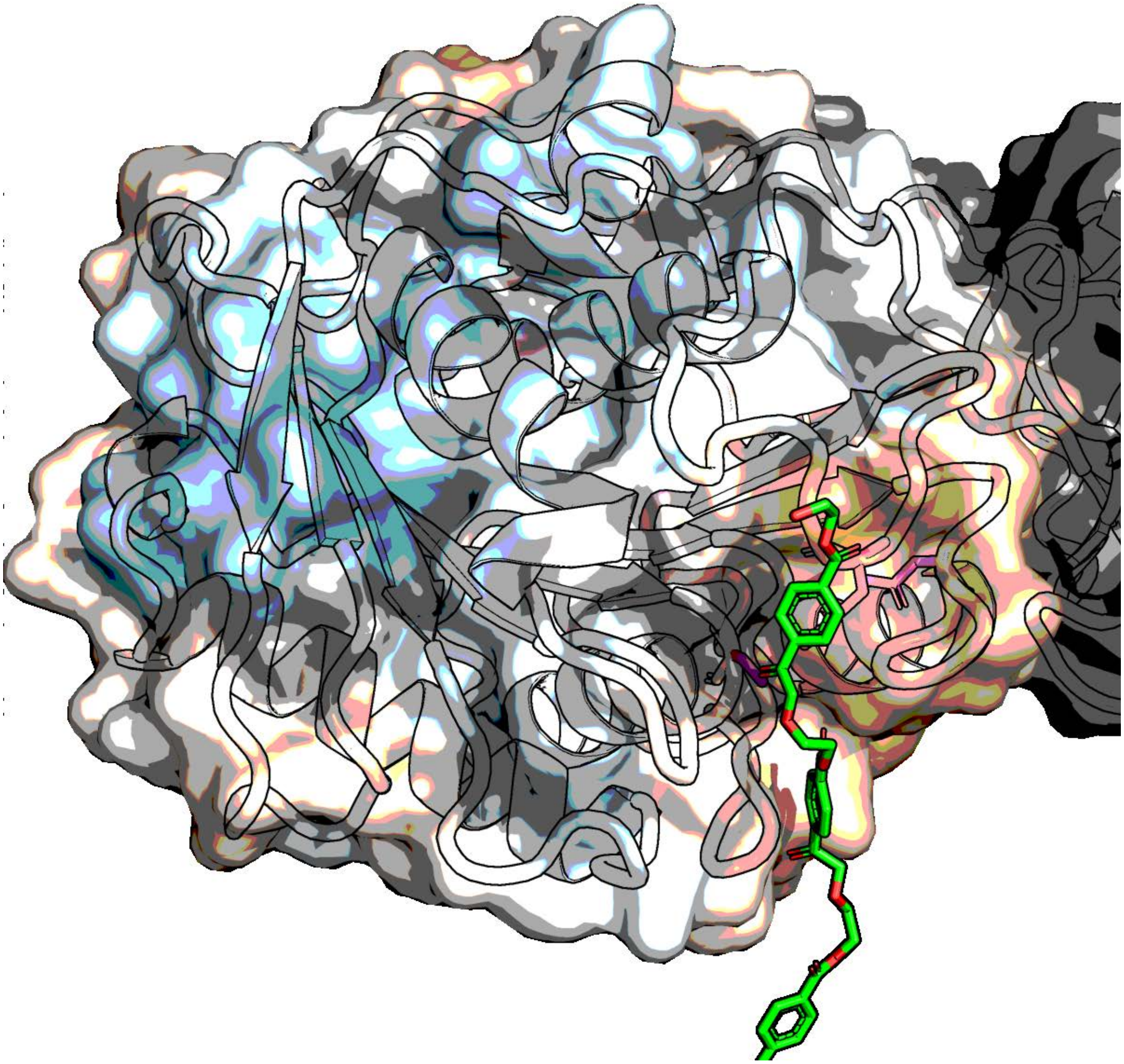

ESM101_highlights.png

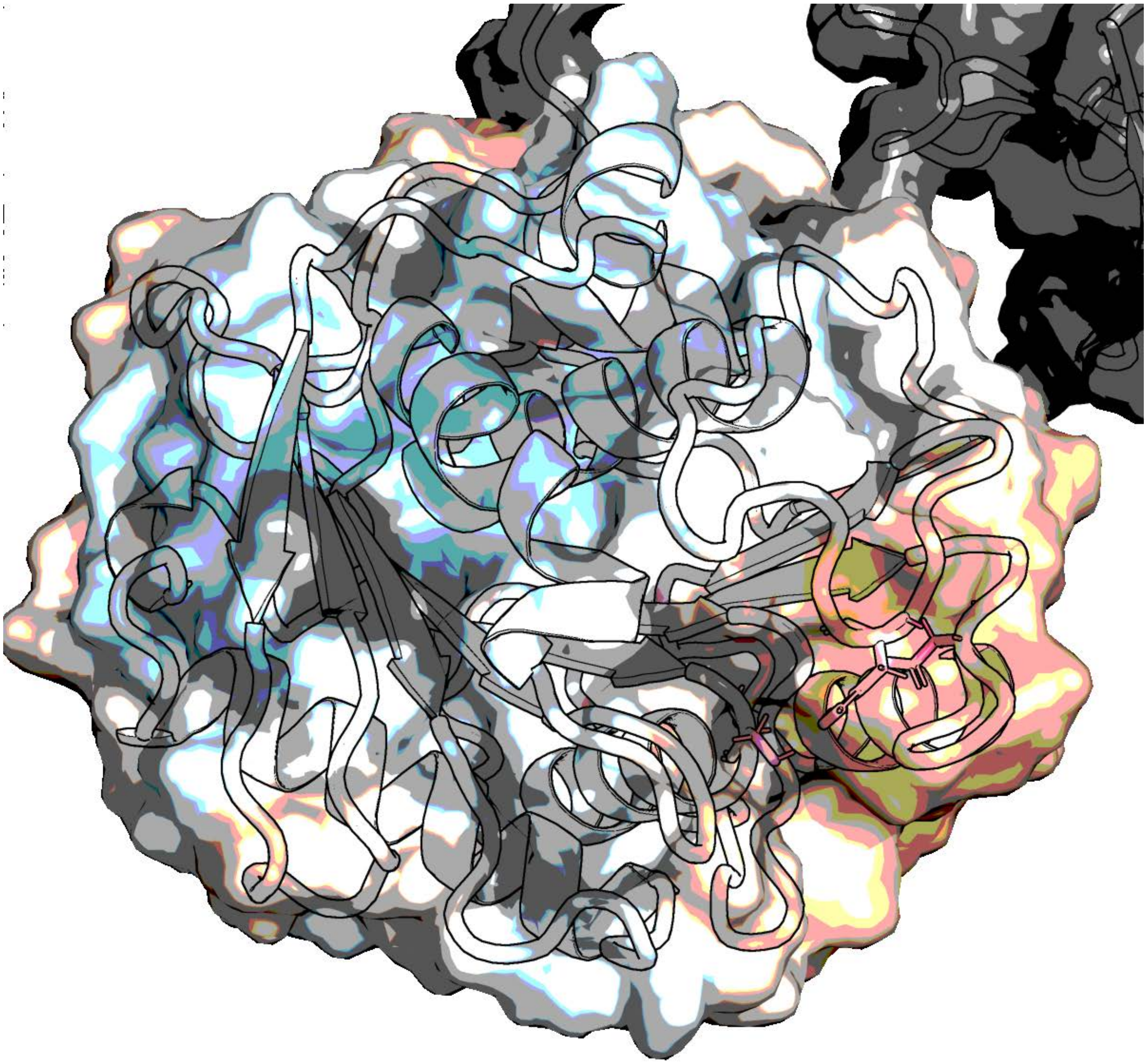

ESM106_highlights.png

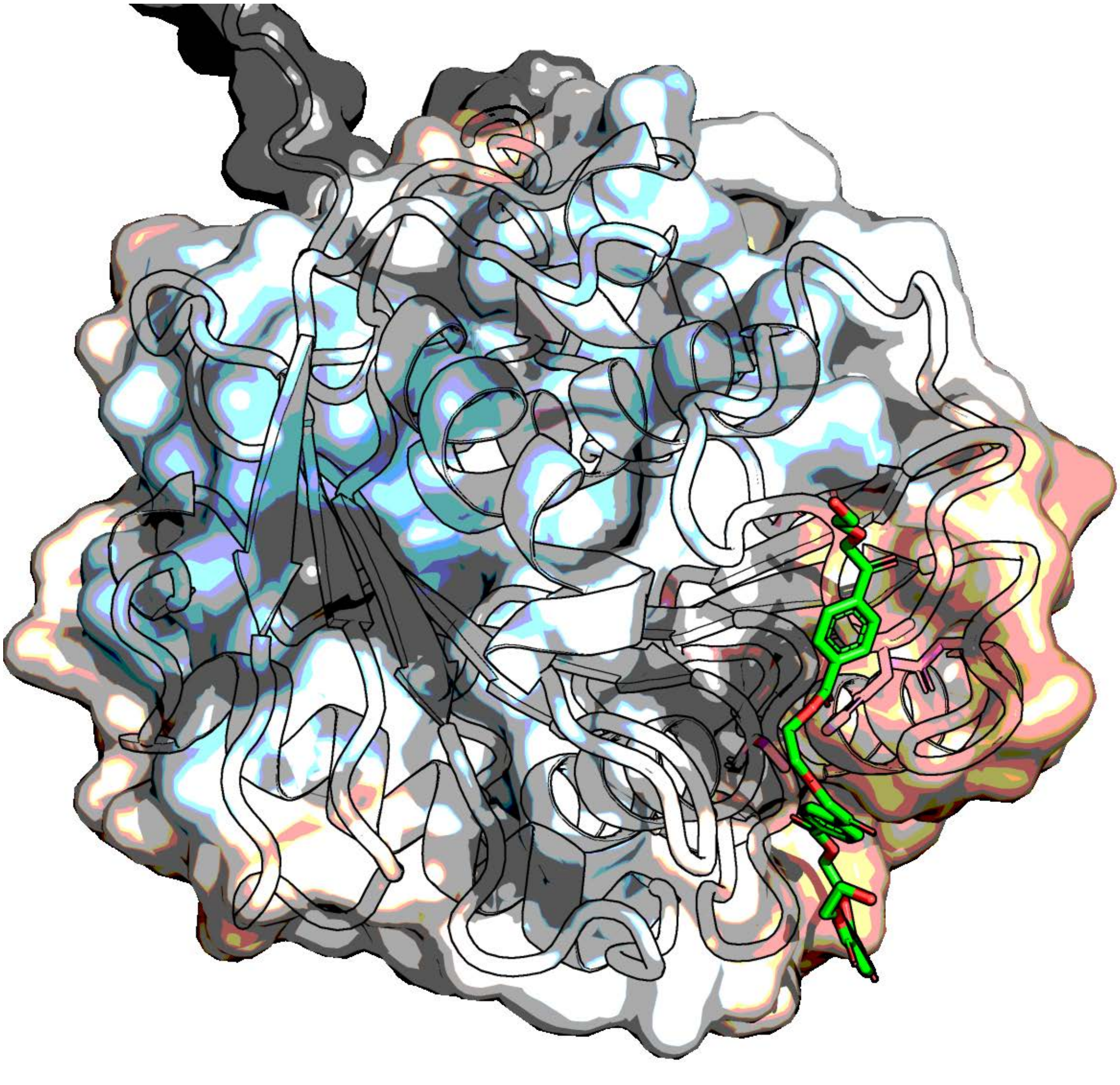

ESM121_highlights.png

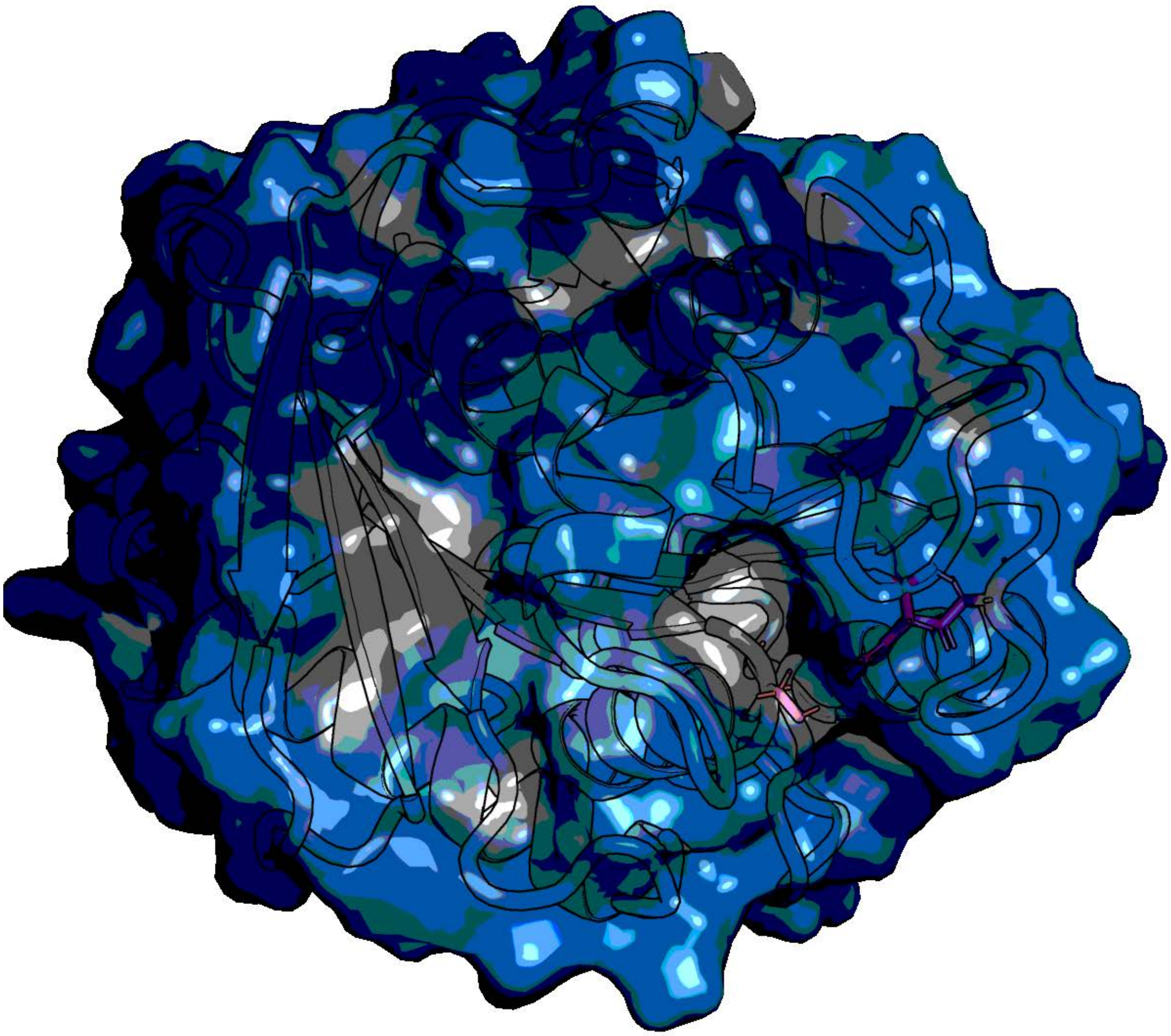

ESM128_highlights.png

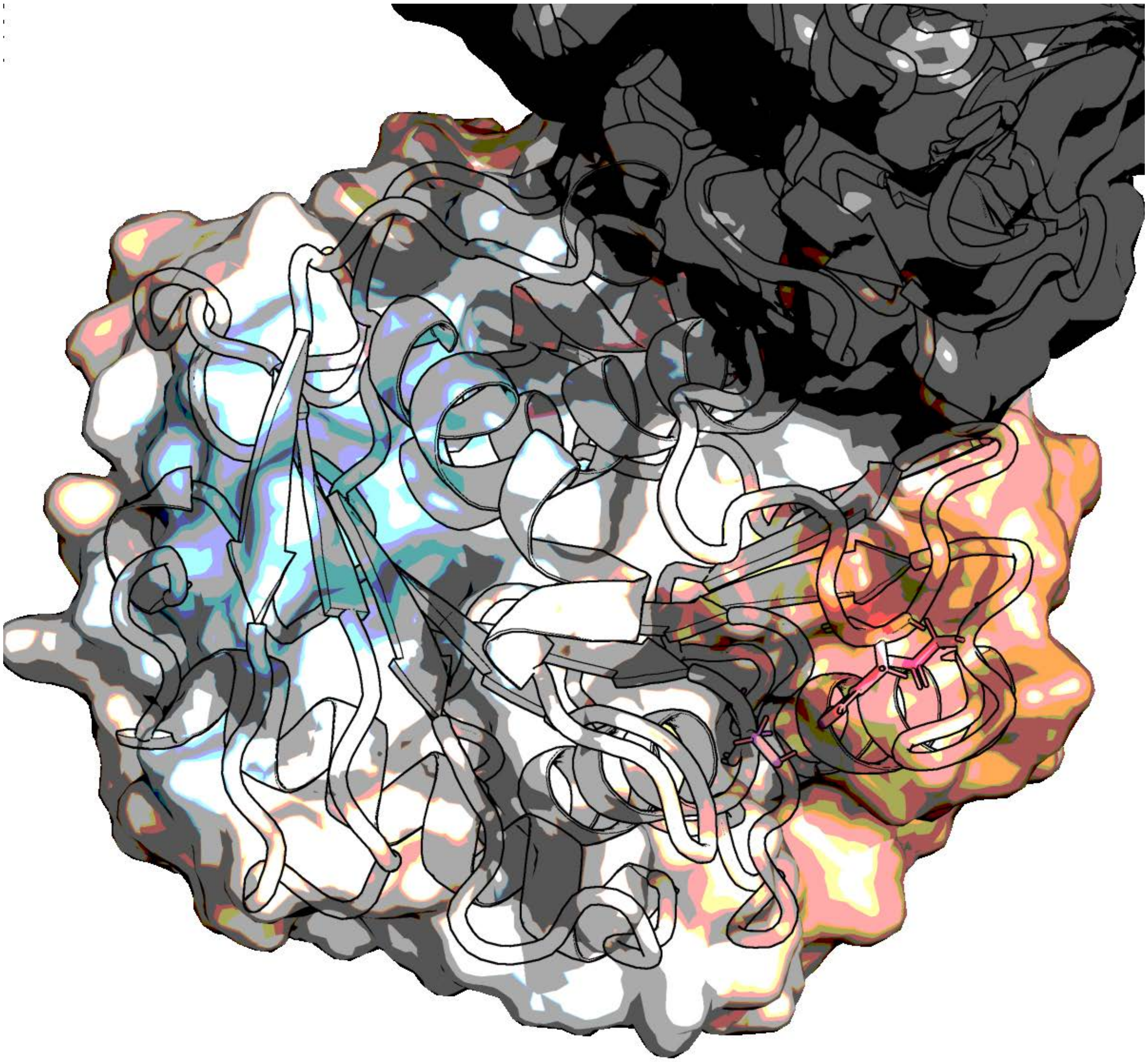

ESM130_highlights.png

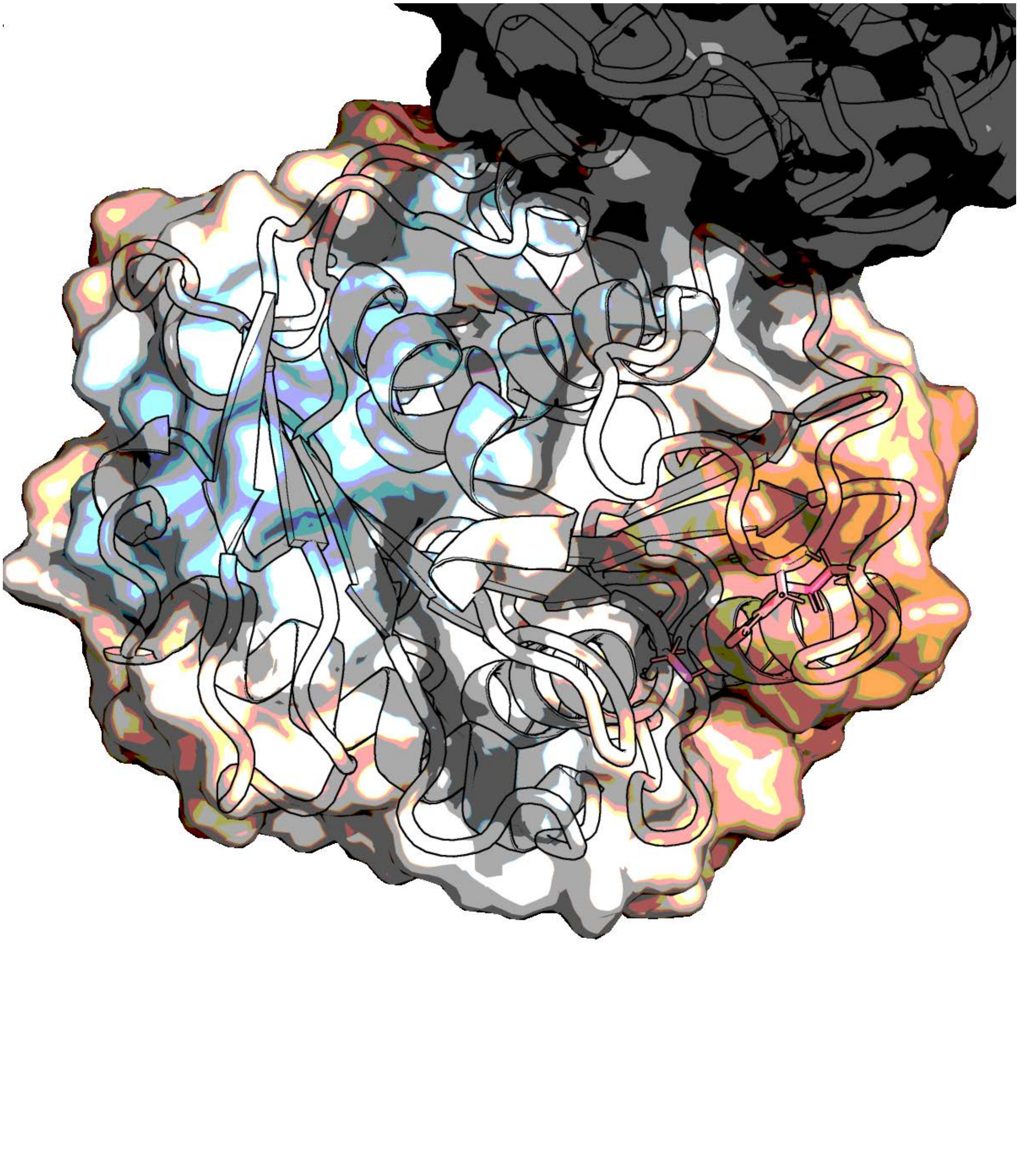

ESM132_highlights.png

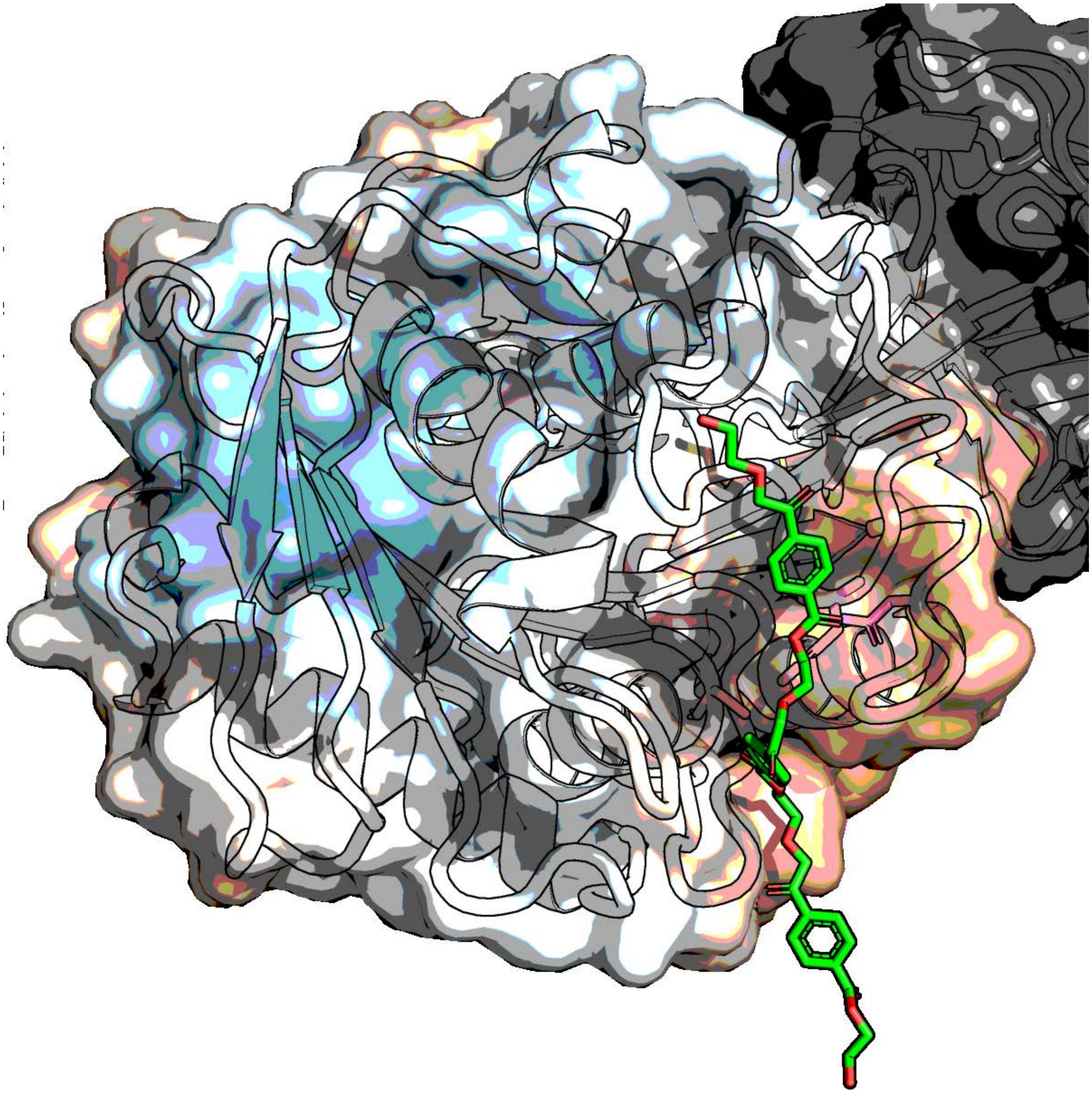

ESM135_highlights.png

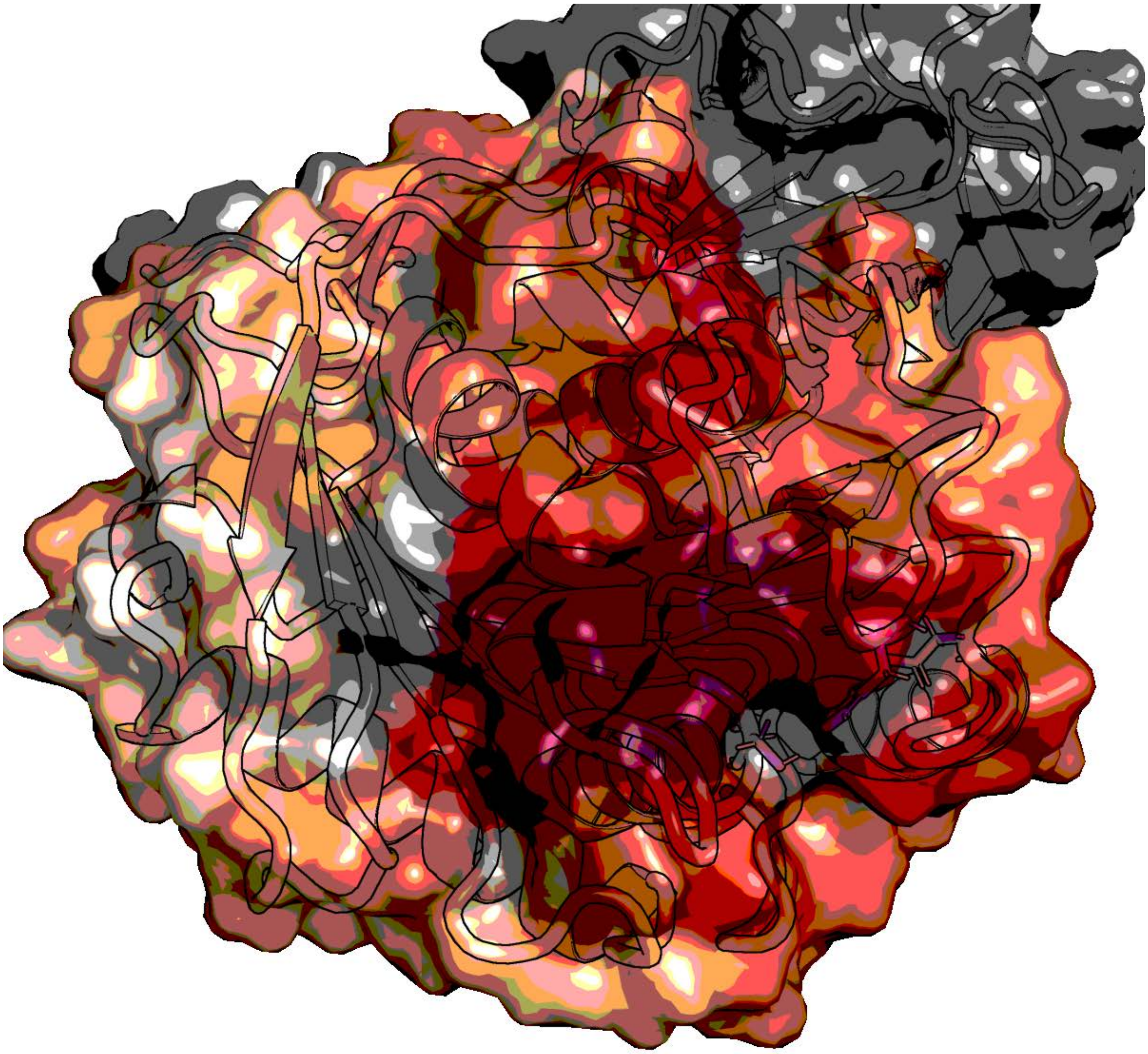

ESM136_highlights.png

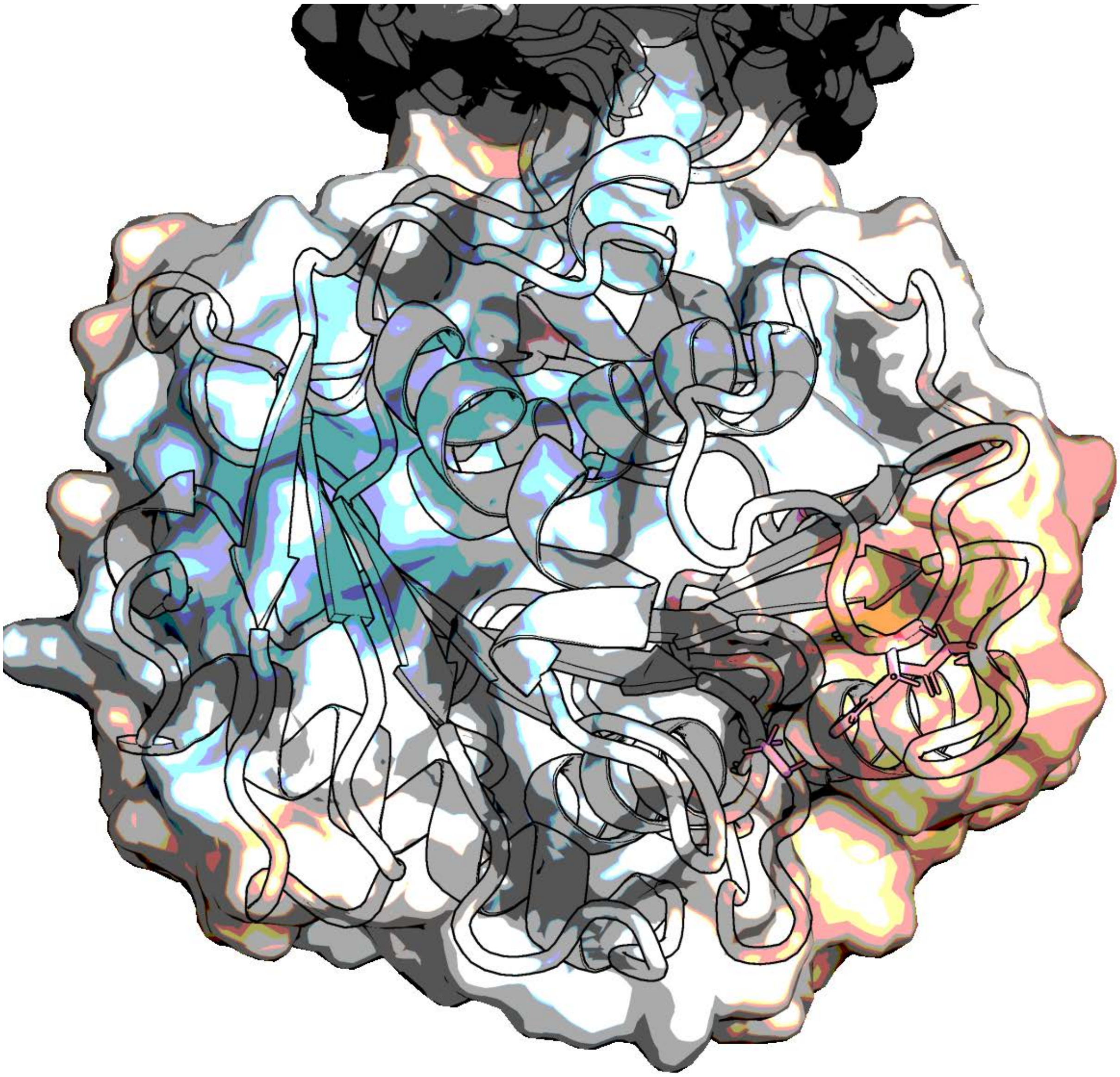

ESM137_highlights.png

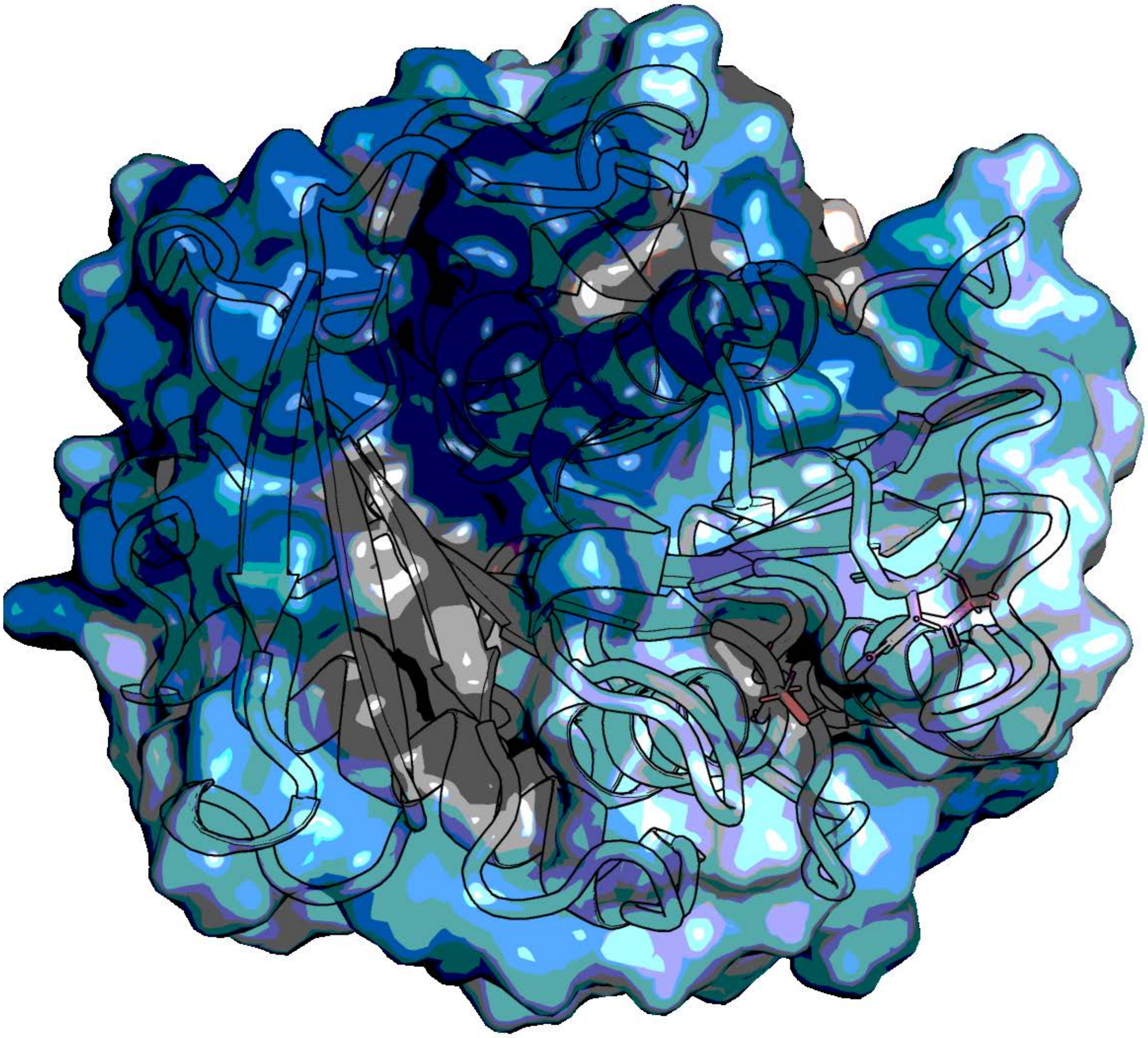

ESM140_highlights.png

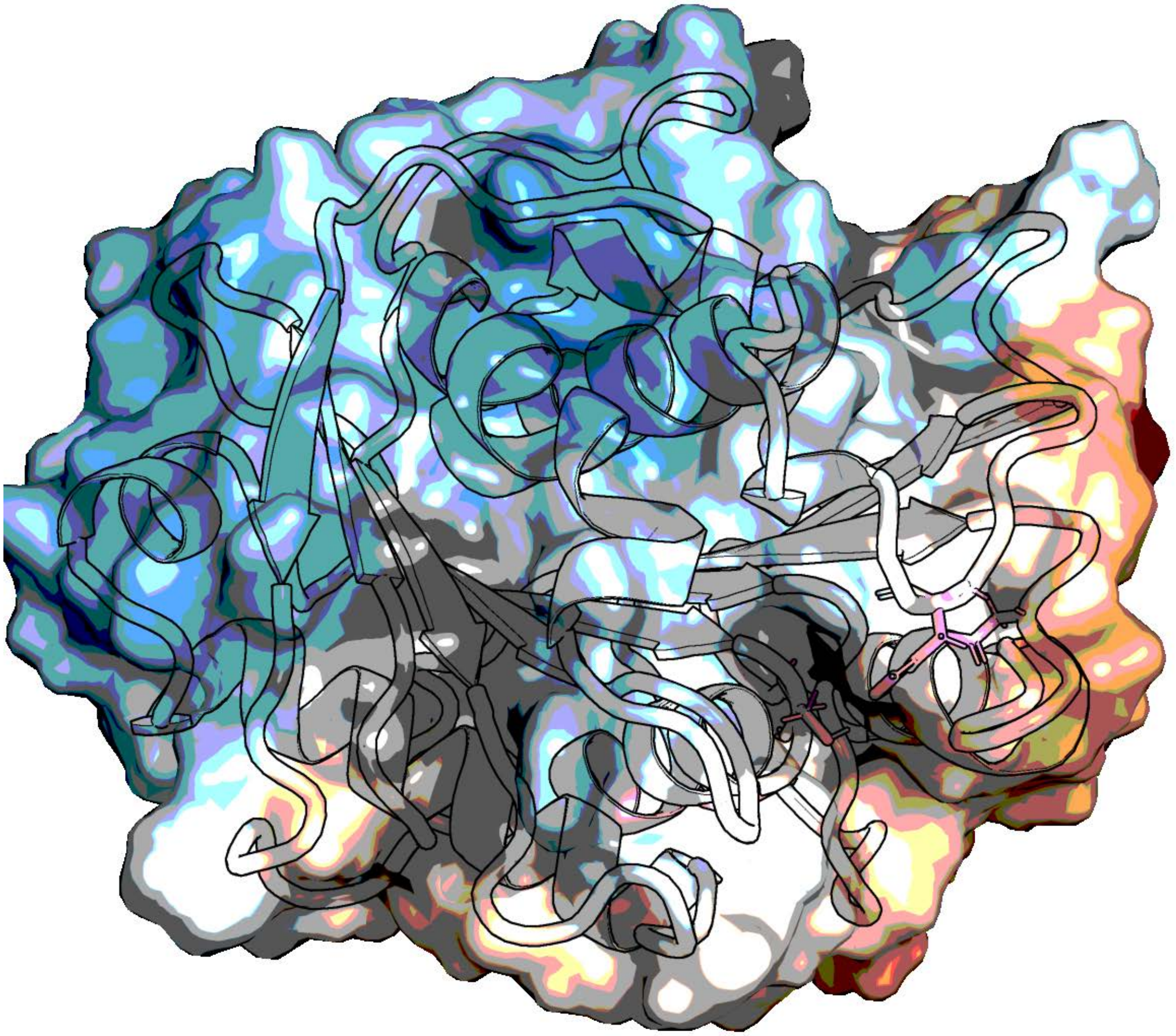

ESM144_highlights.png

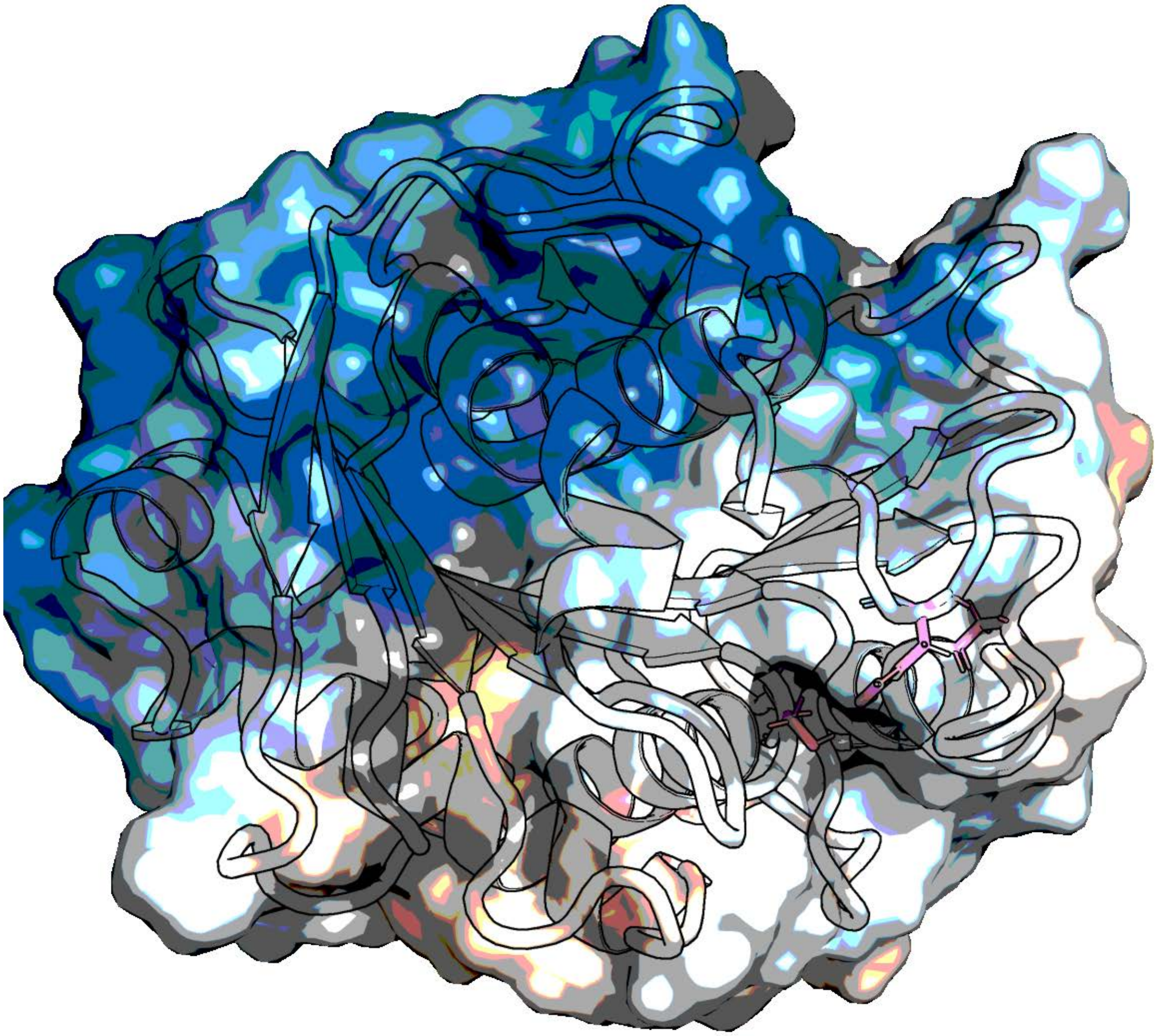

ESM147_highlights.png

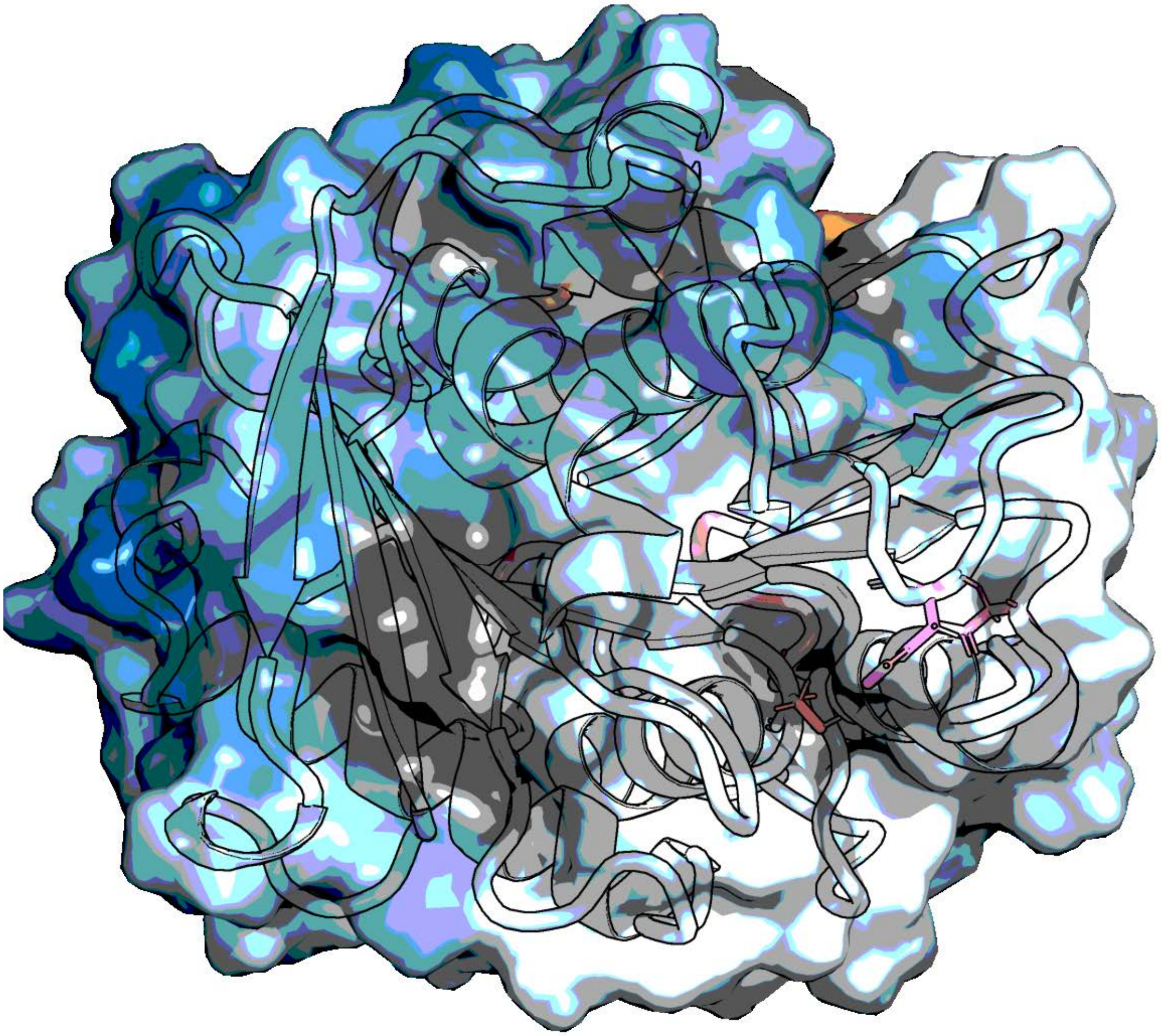

ESM156_highlights.png

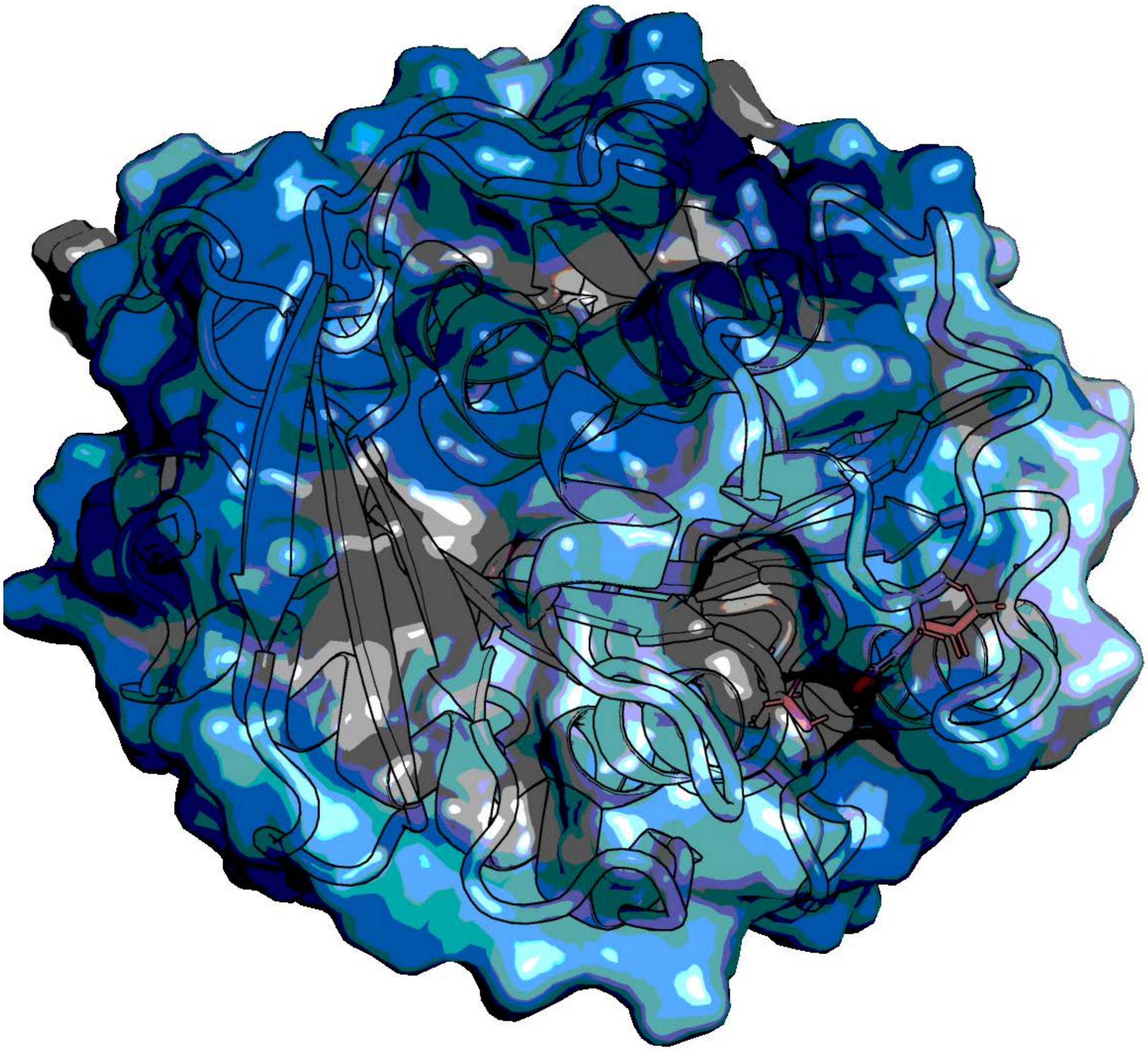

ESM157_highlights.png

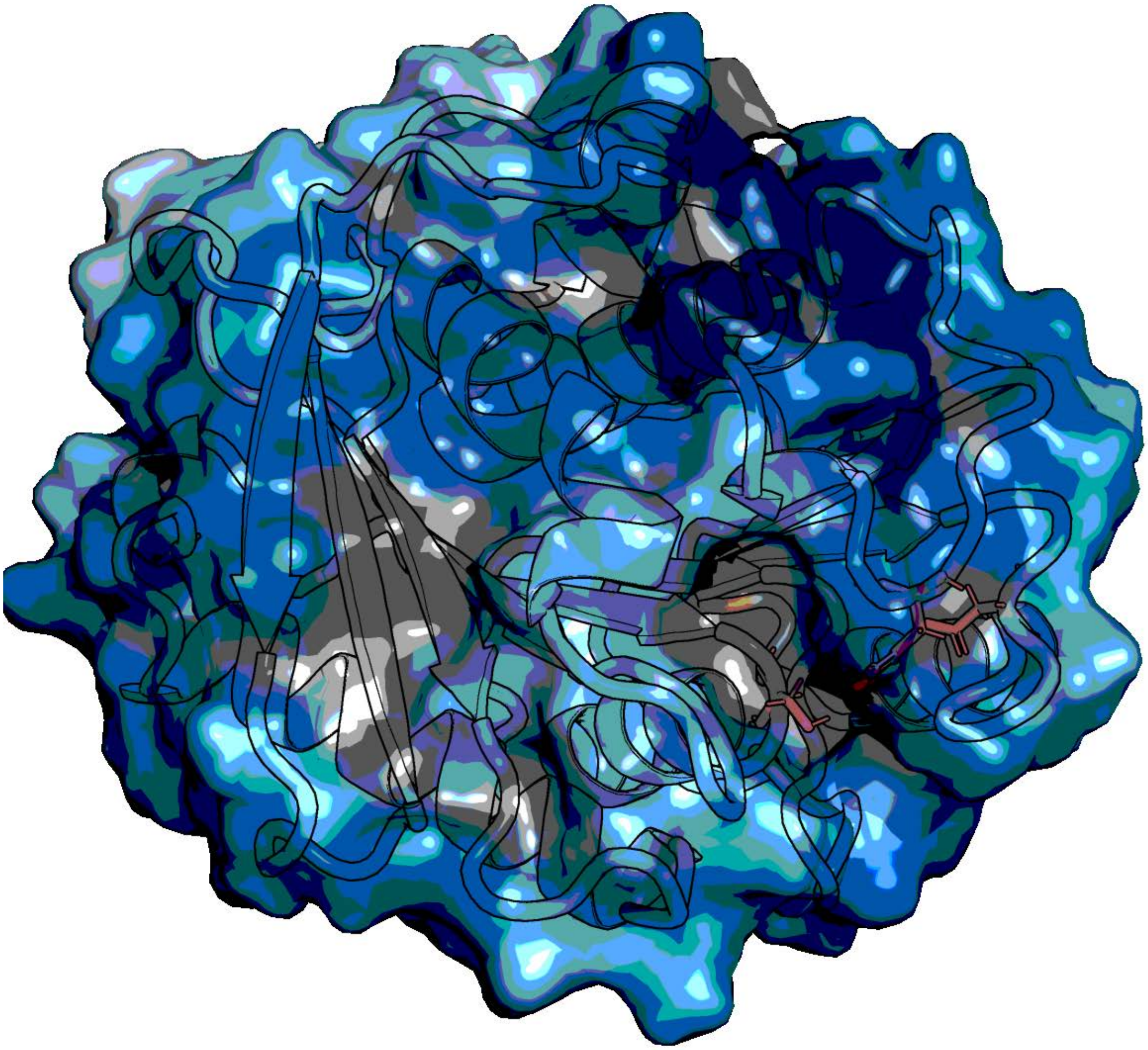

ESM159_highlights.png

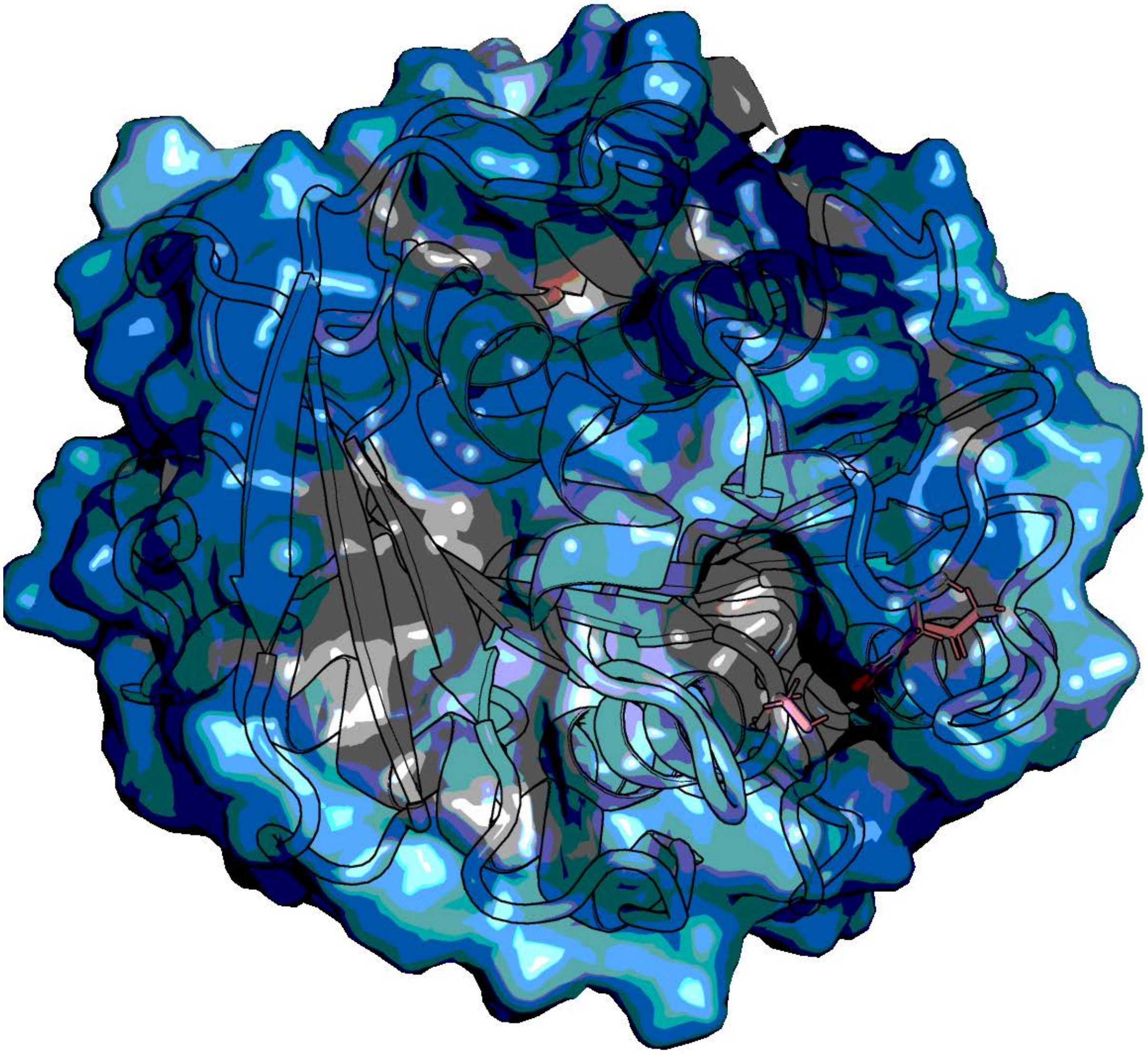

ESM160_highlights.png

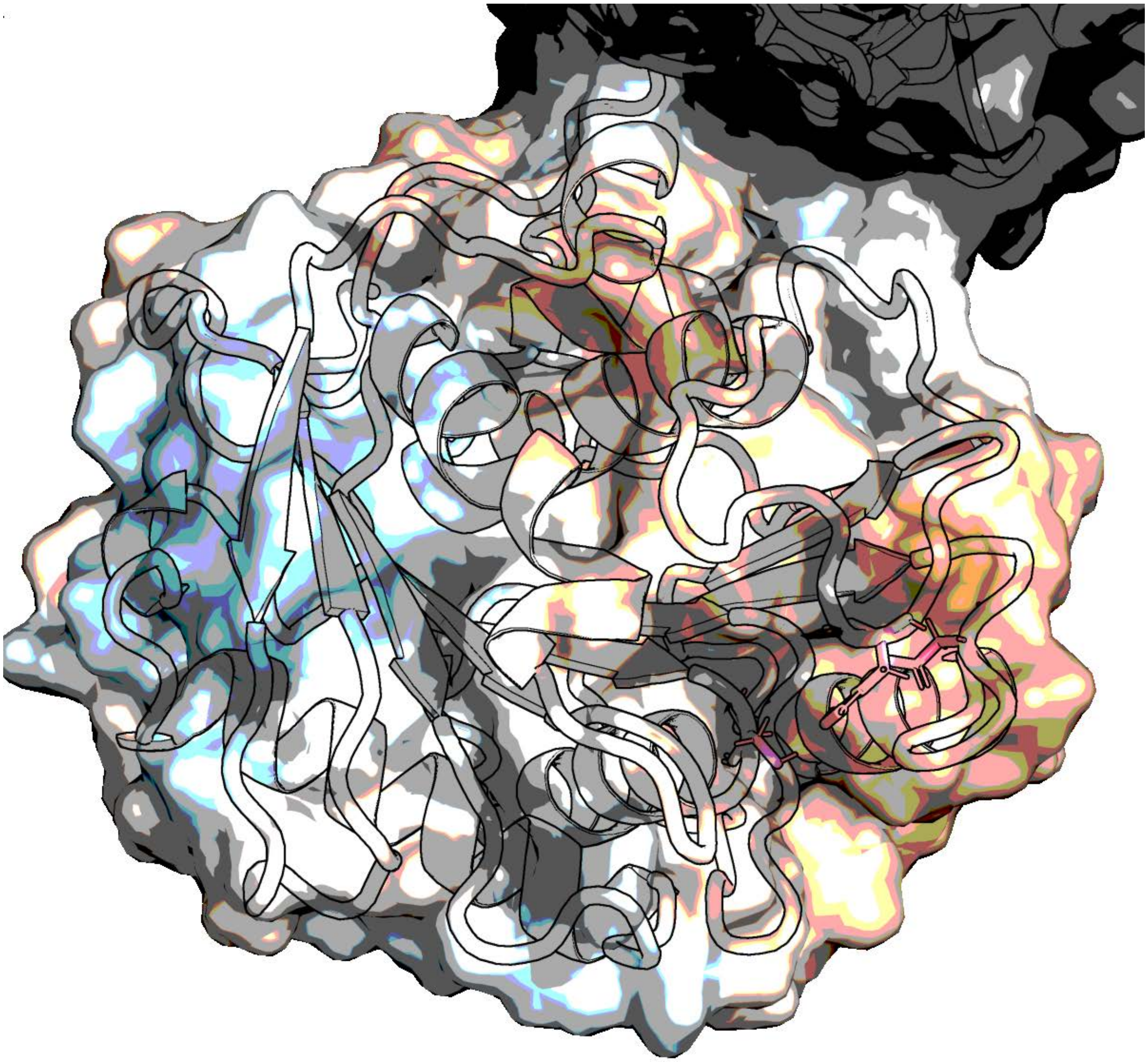

ESM161_highlights.png

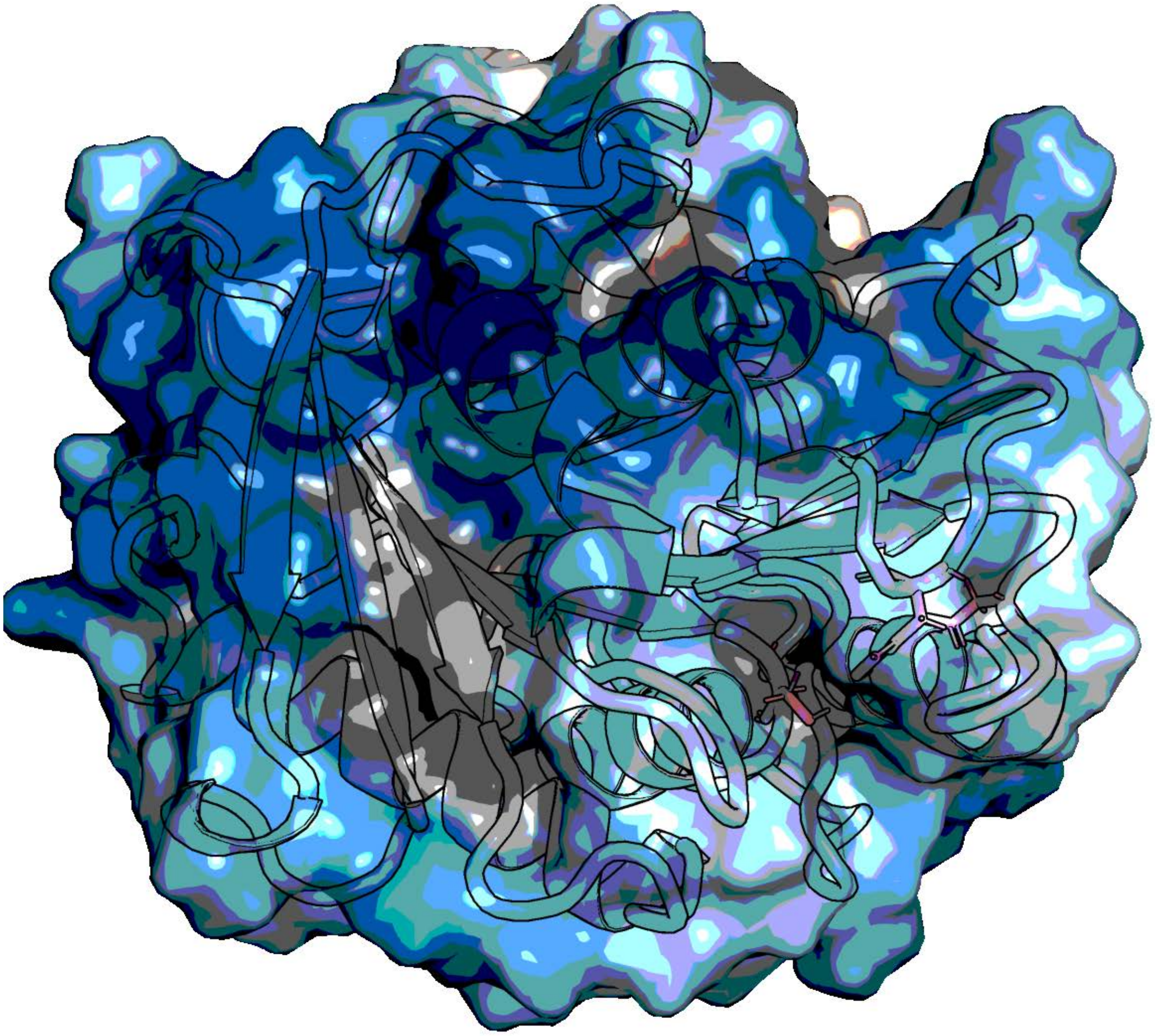

ESM167_highlights.png

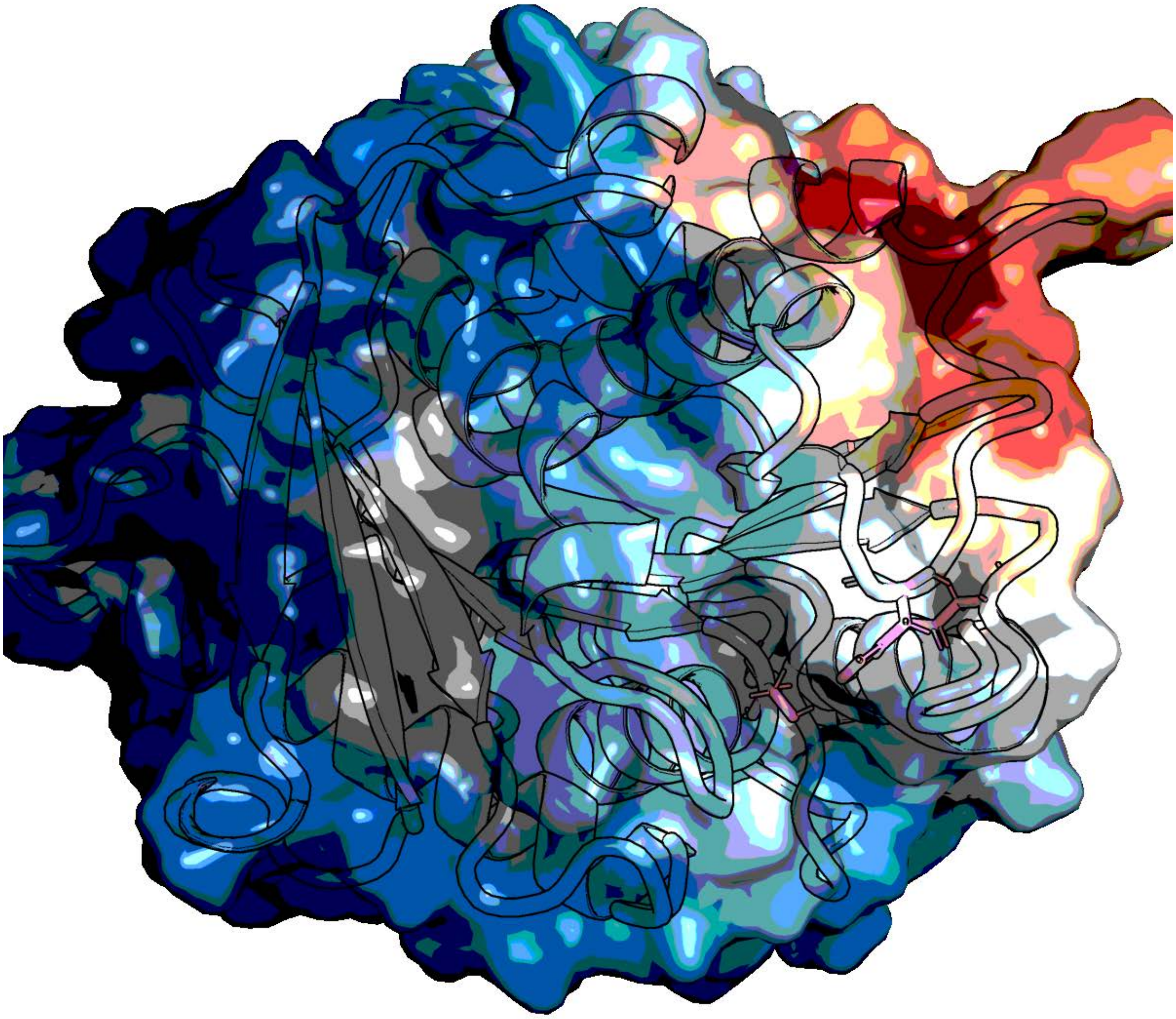

ESM169_highlights.png

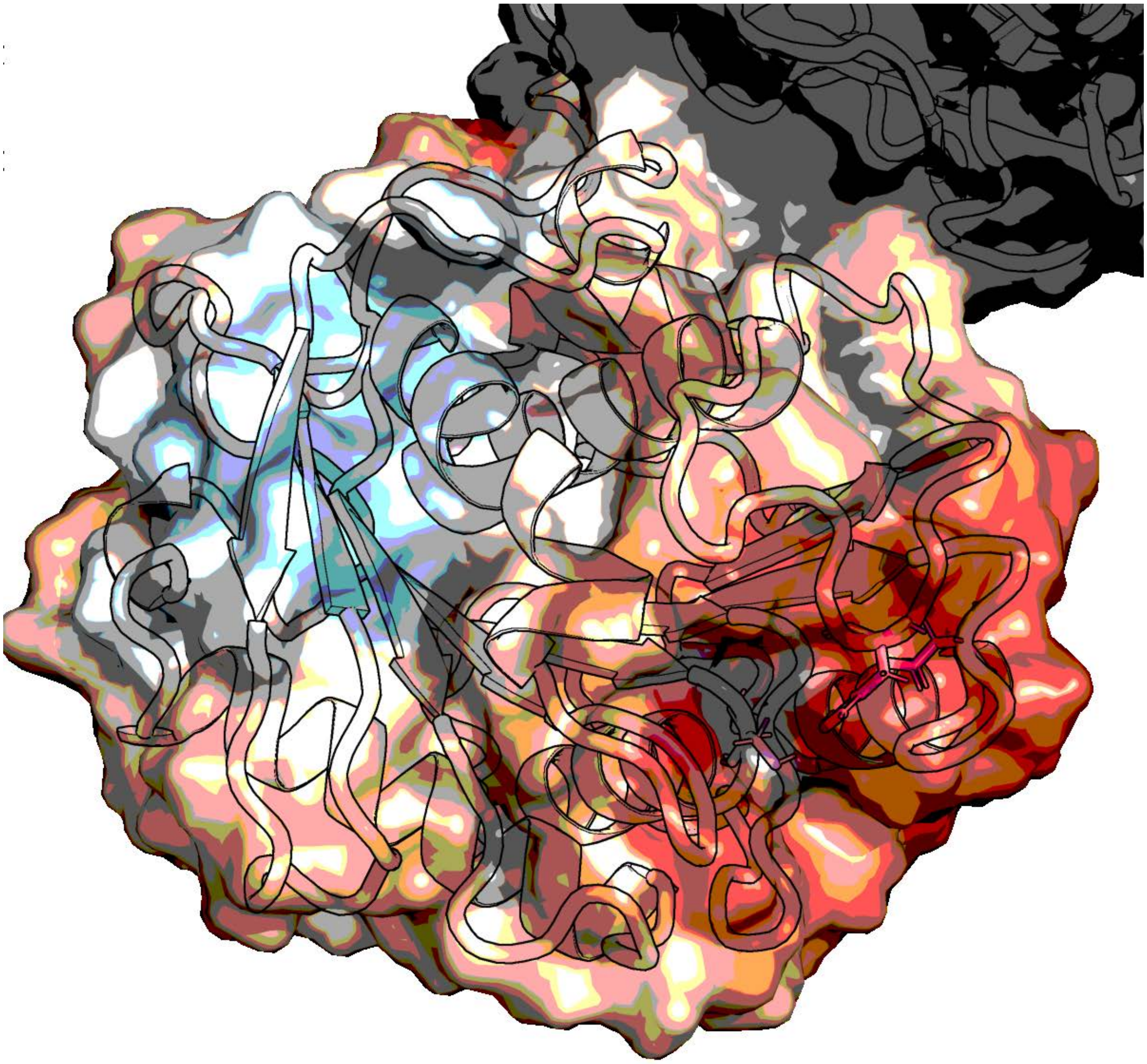

ESM173_highlights.png

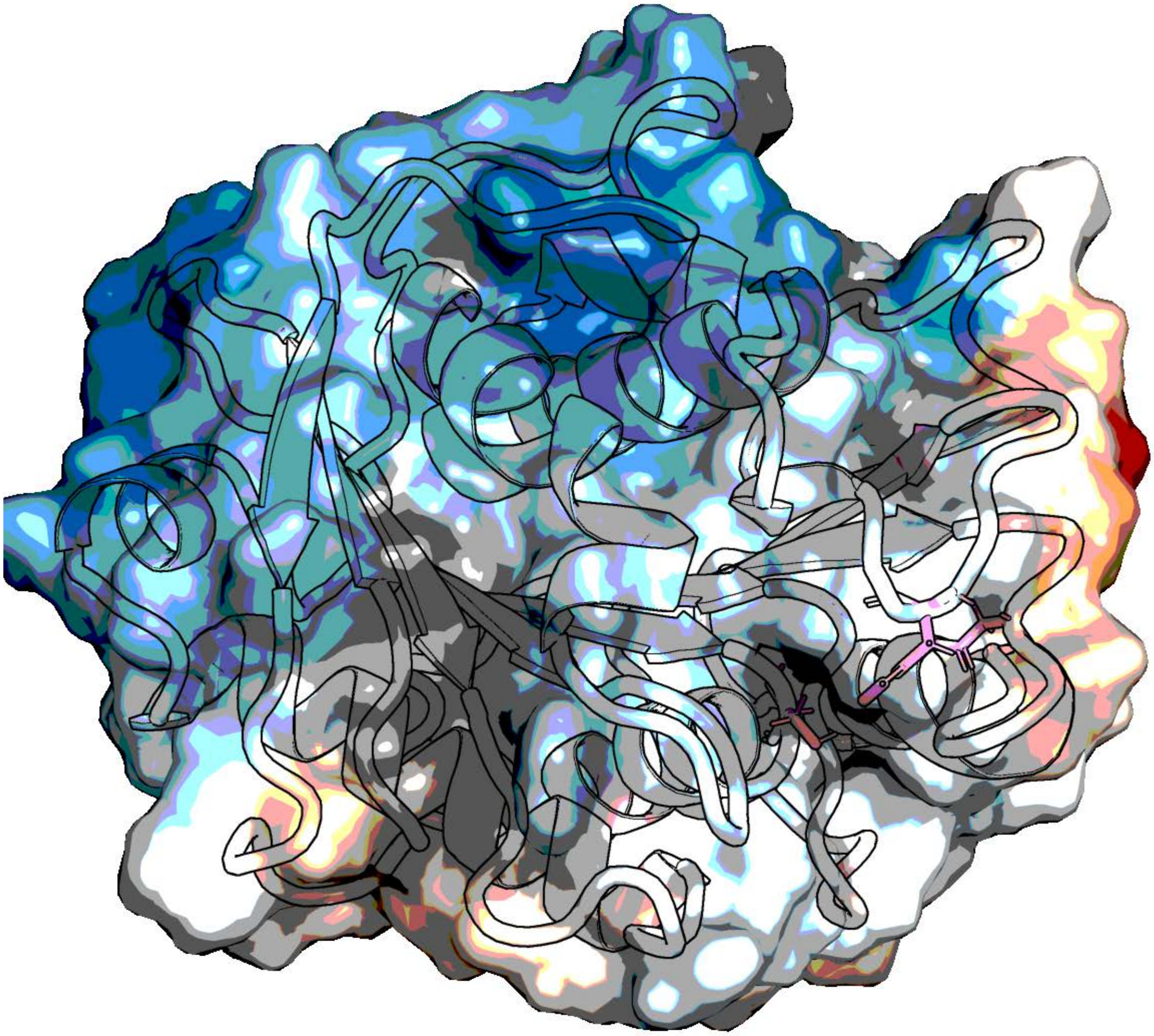

ESM177_highlights.png

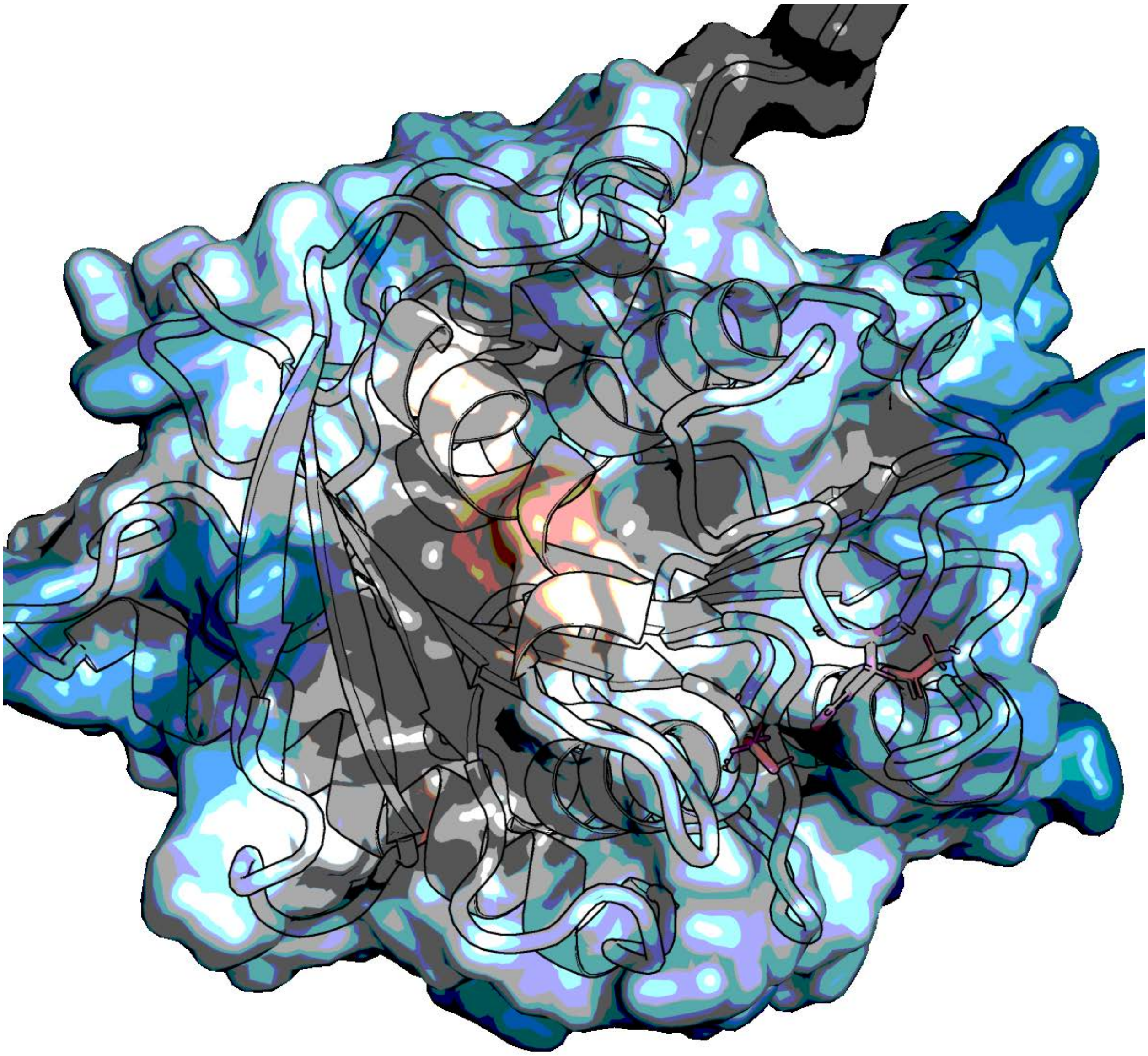

ESM186_highlights.png

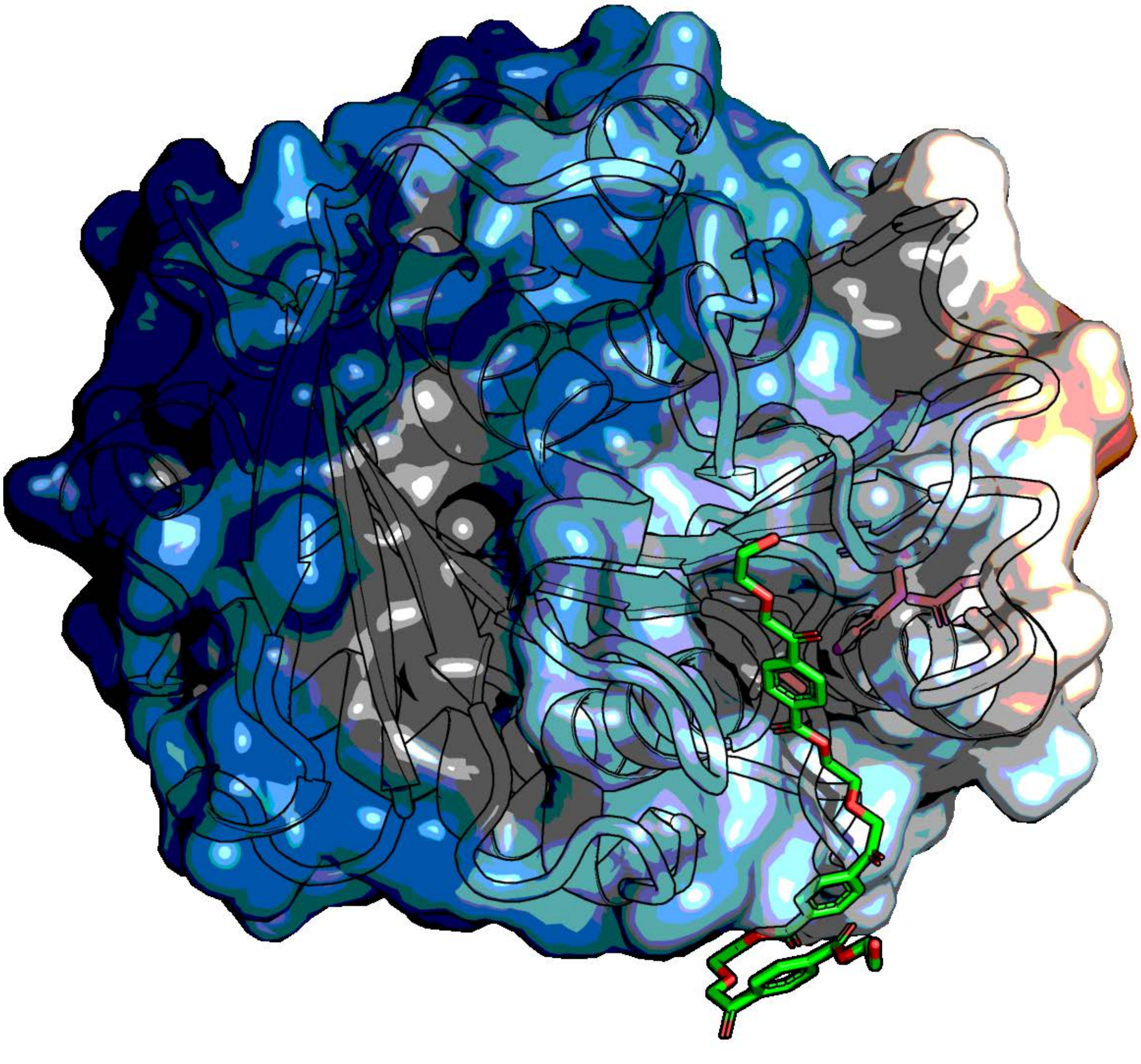

LCC-ICCG_highlights.png

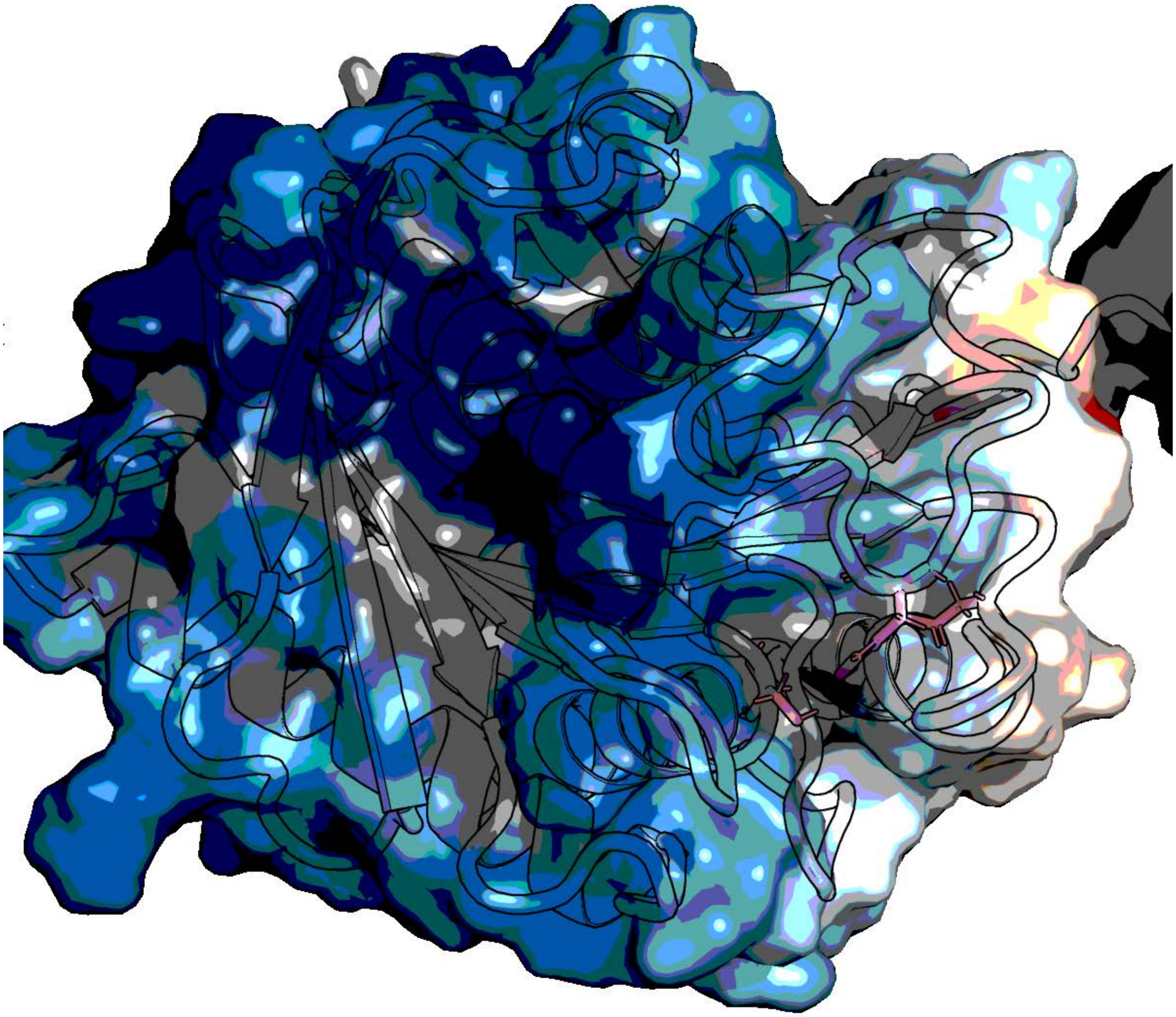

TEP004_highlights.png

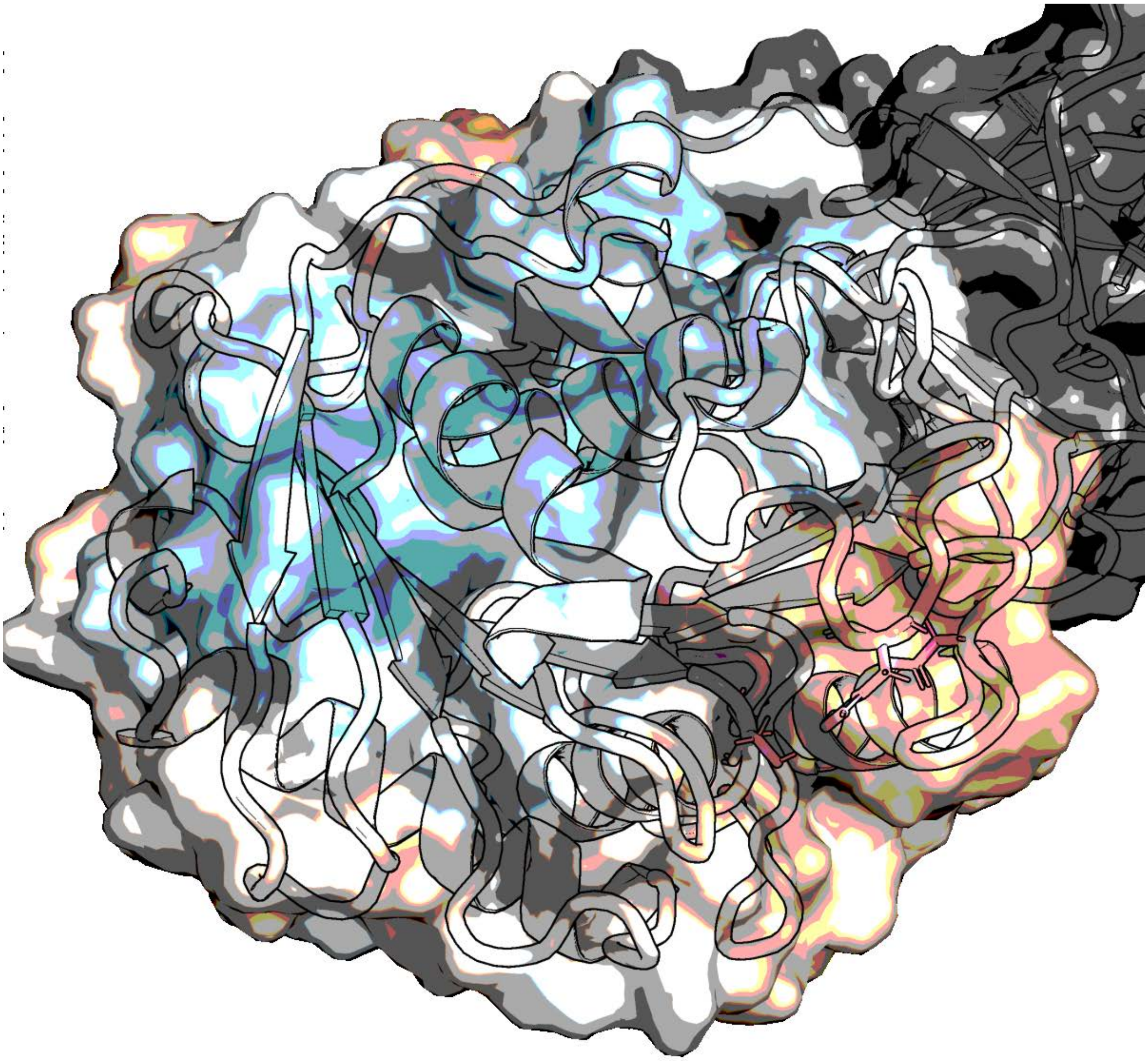

TEP010_highlights.png

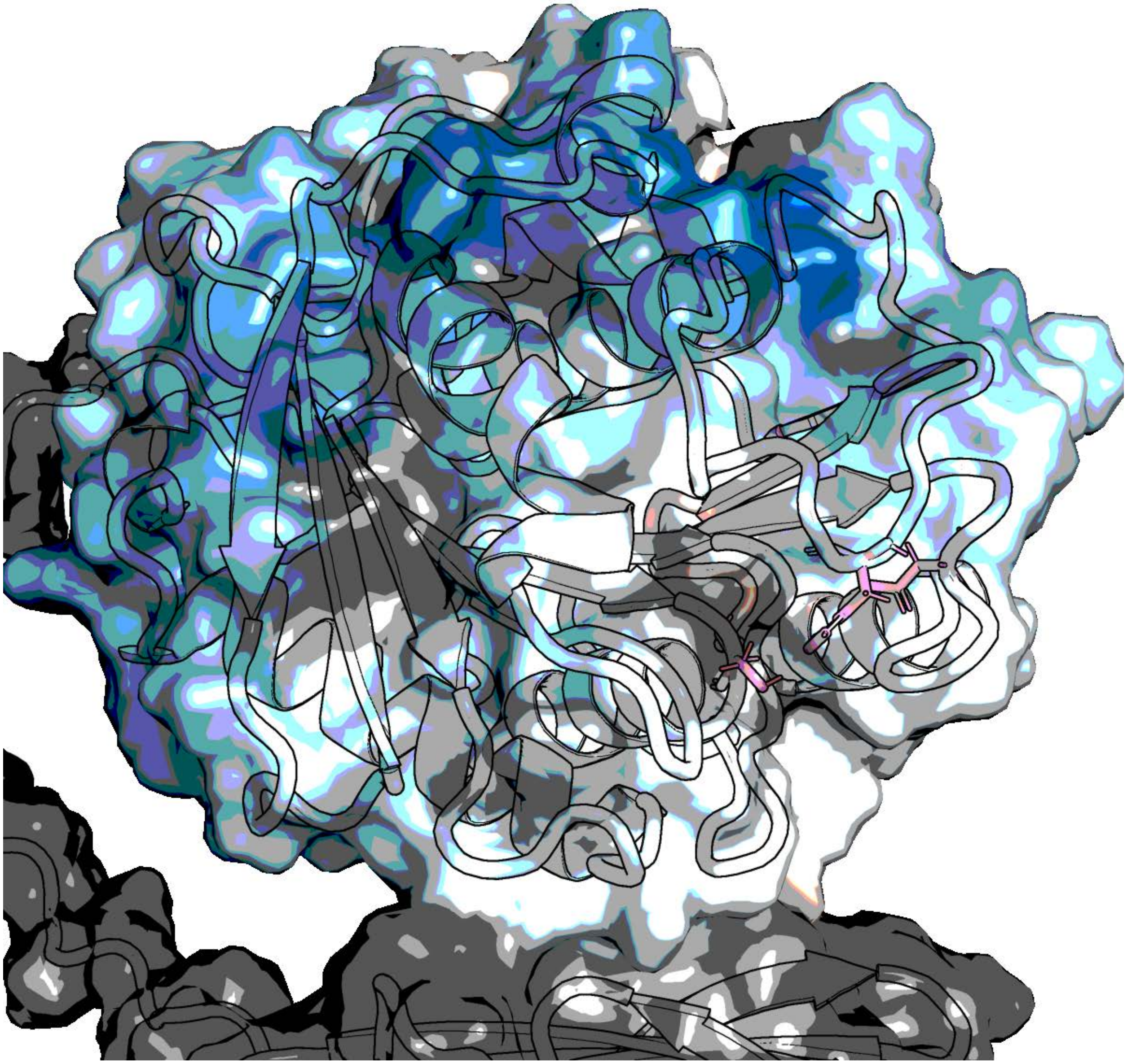

TEP013_highlights.png

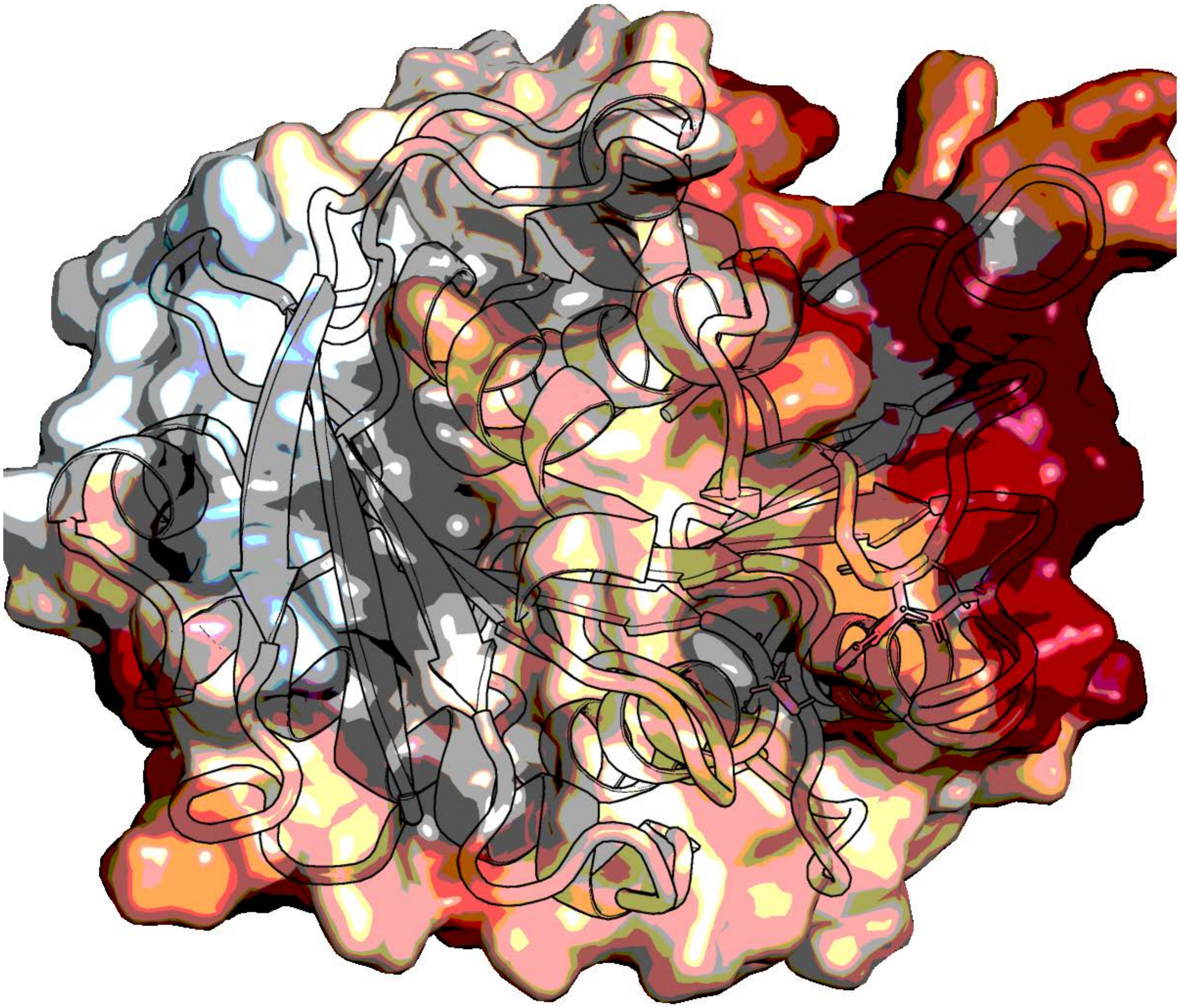

TEP014_highlights.png

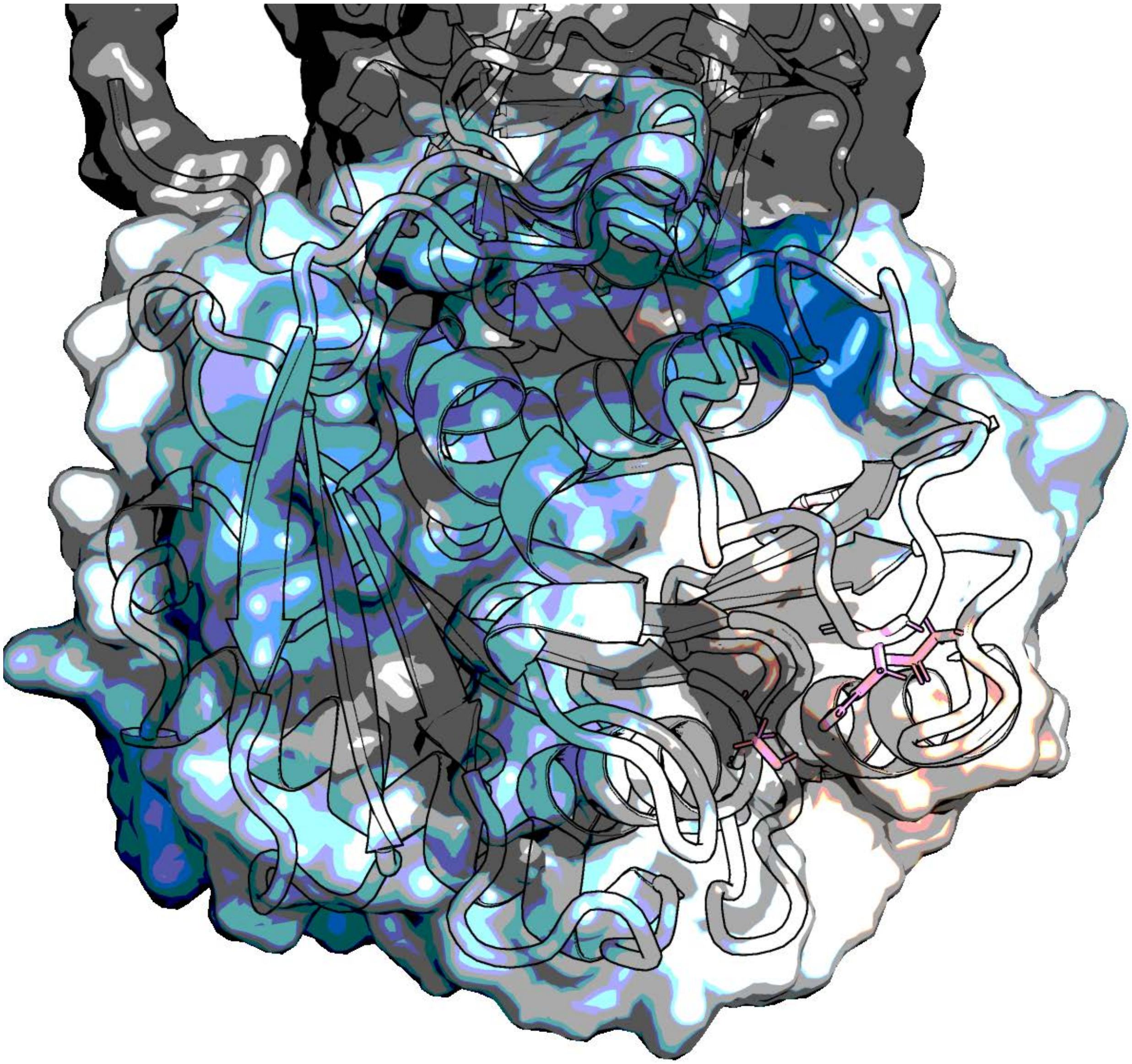

TEP024_highlights.png

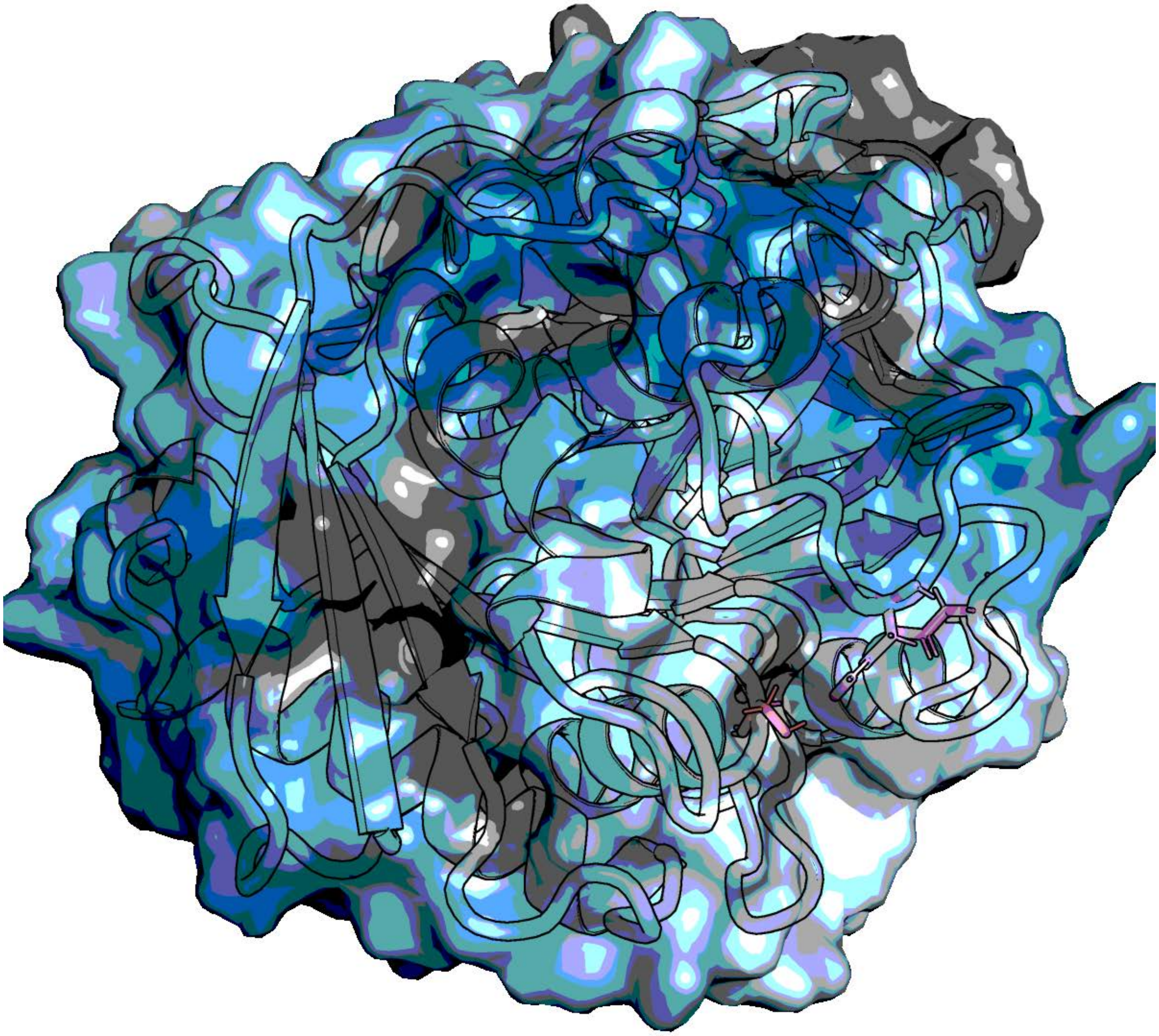

TEP029_highlights.png

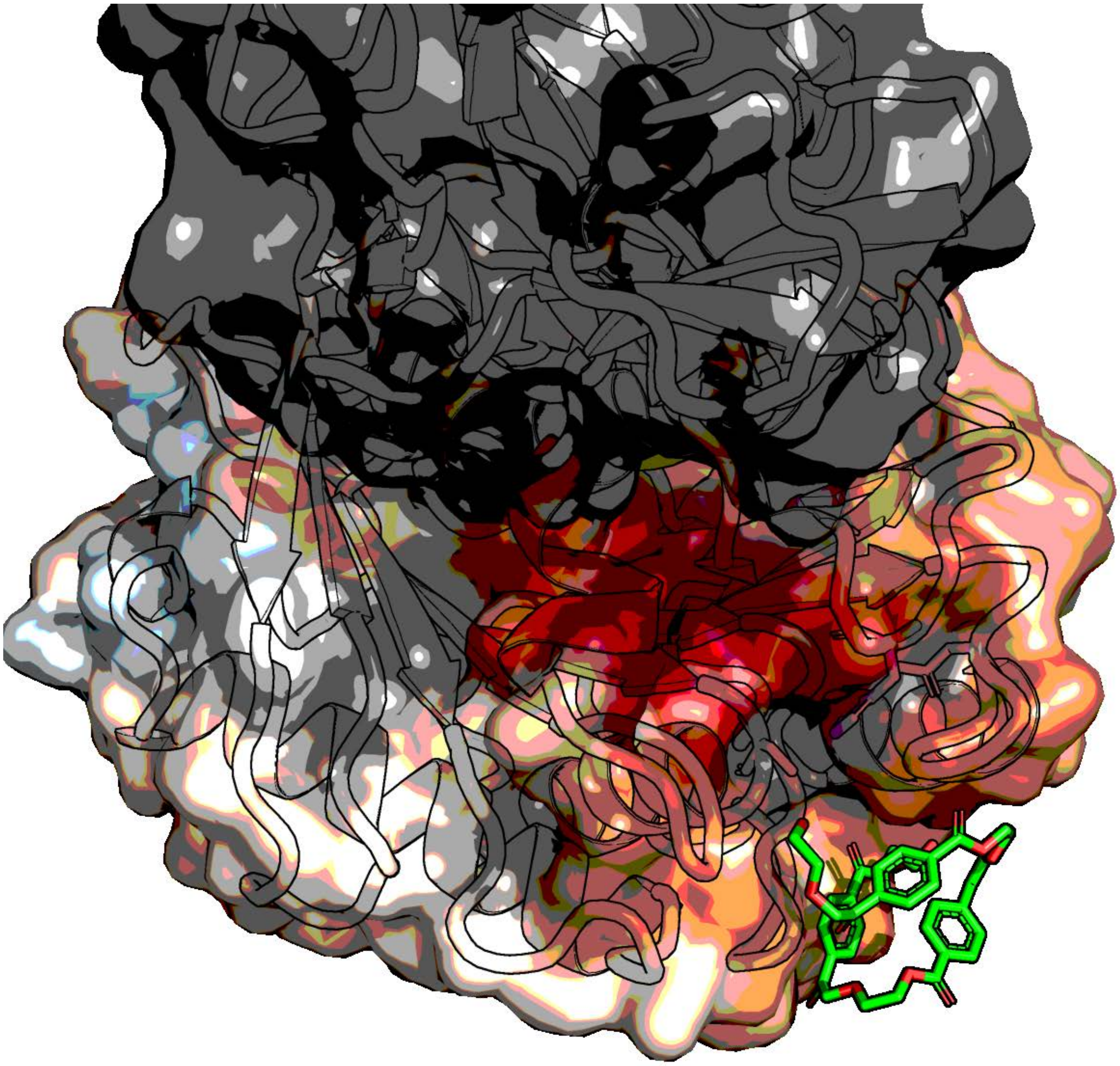

TEP054_highlights.png

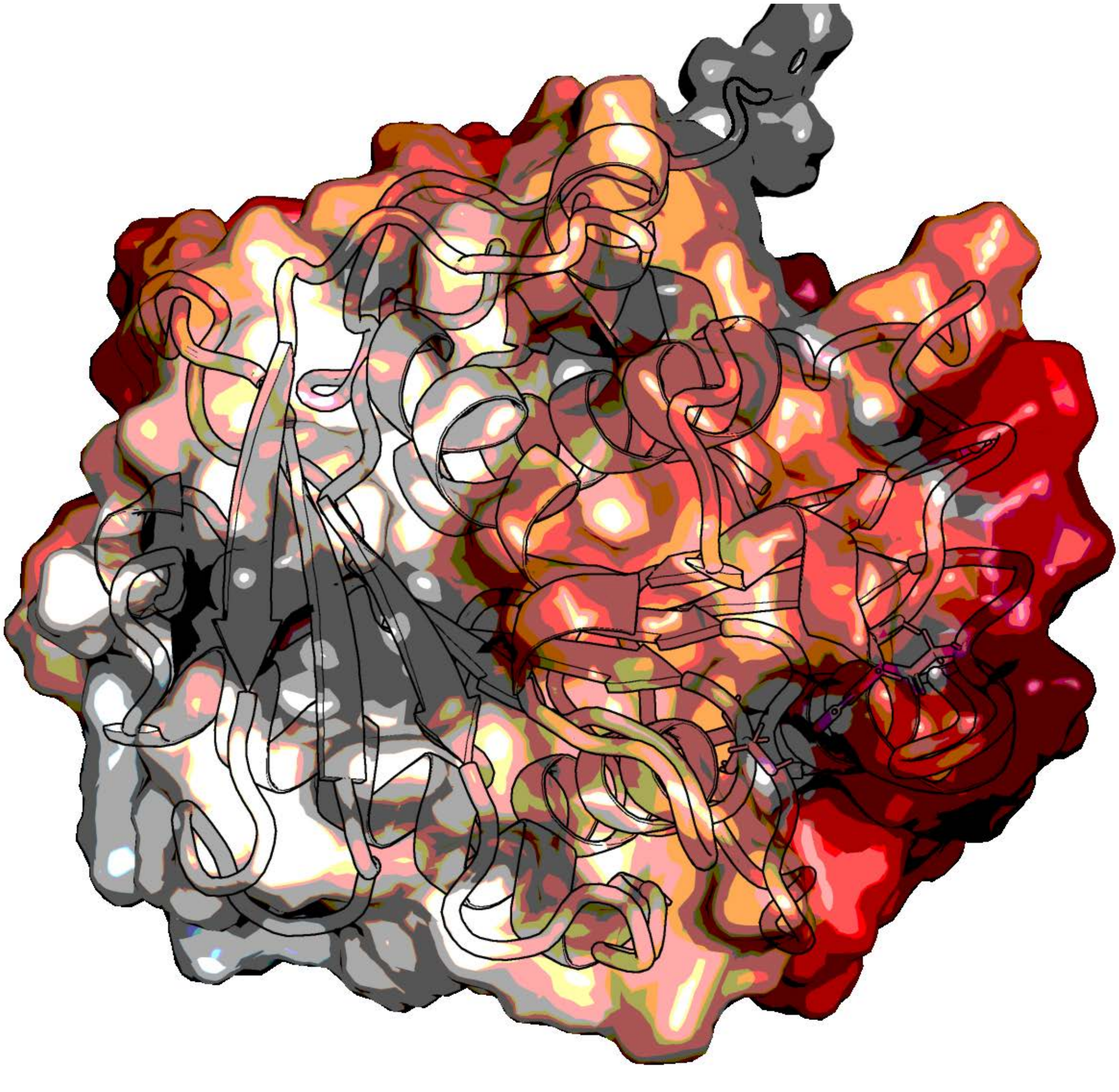

TEP067_highlights.png

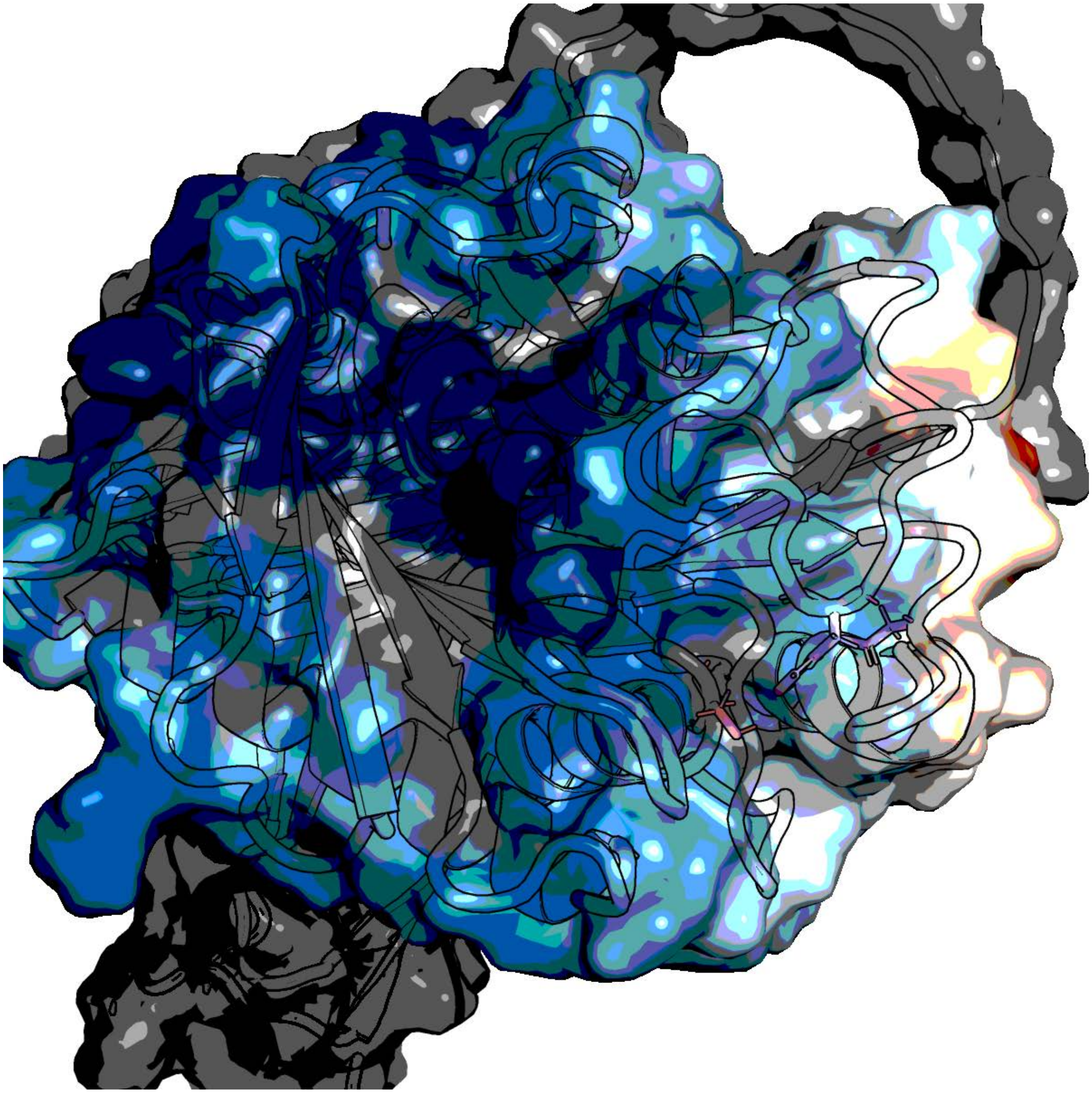

TEP080_highlights.png

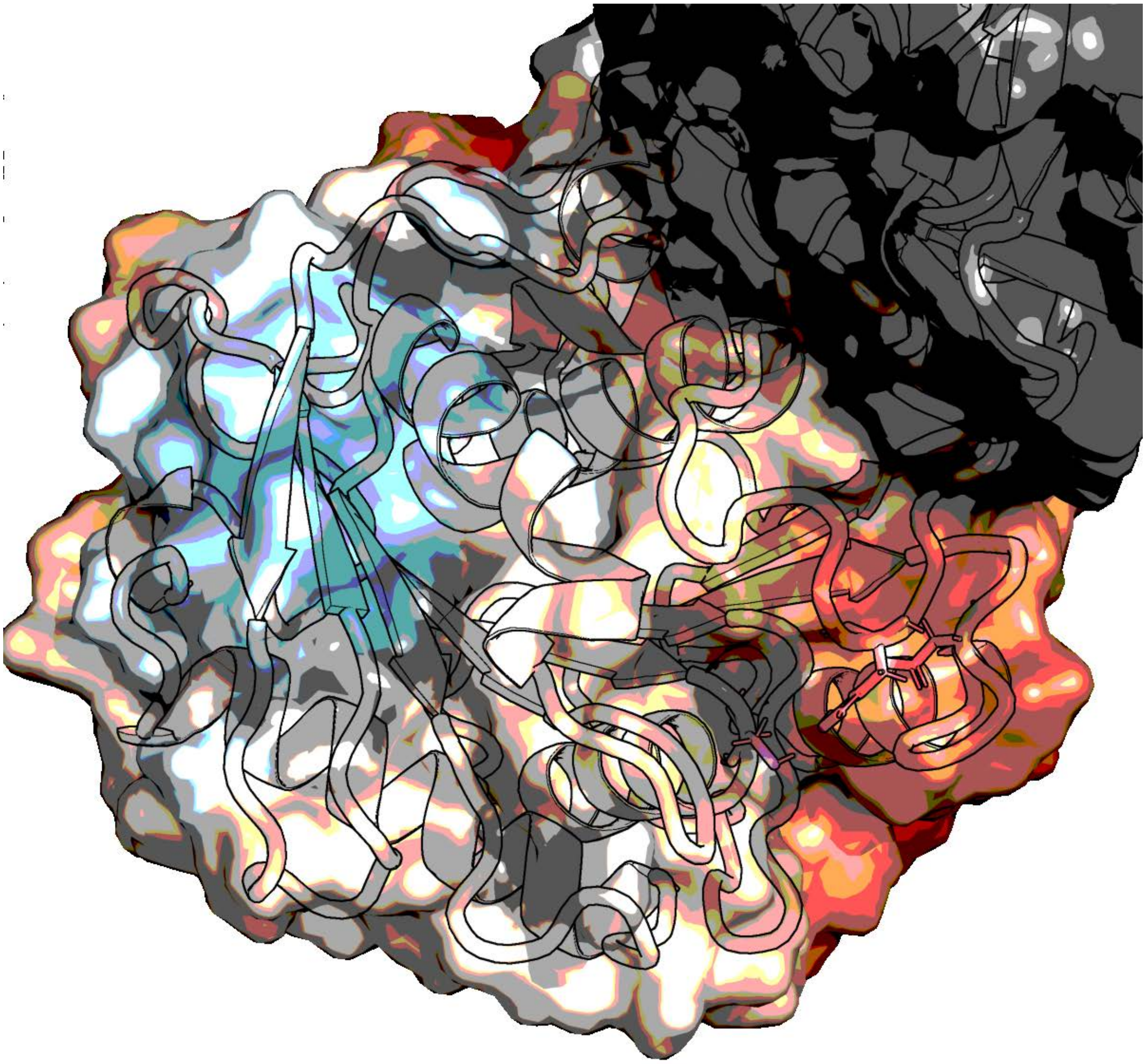

TEP082_highlights.png

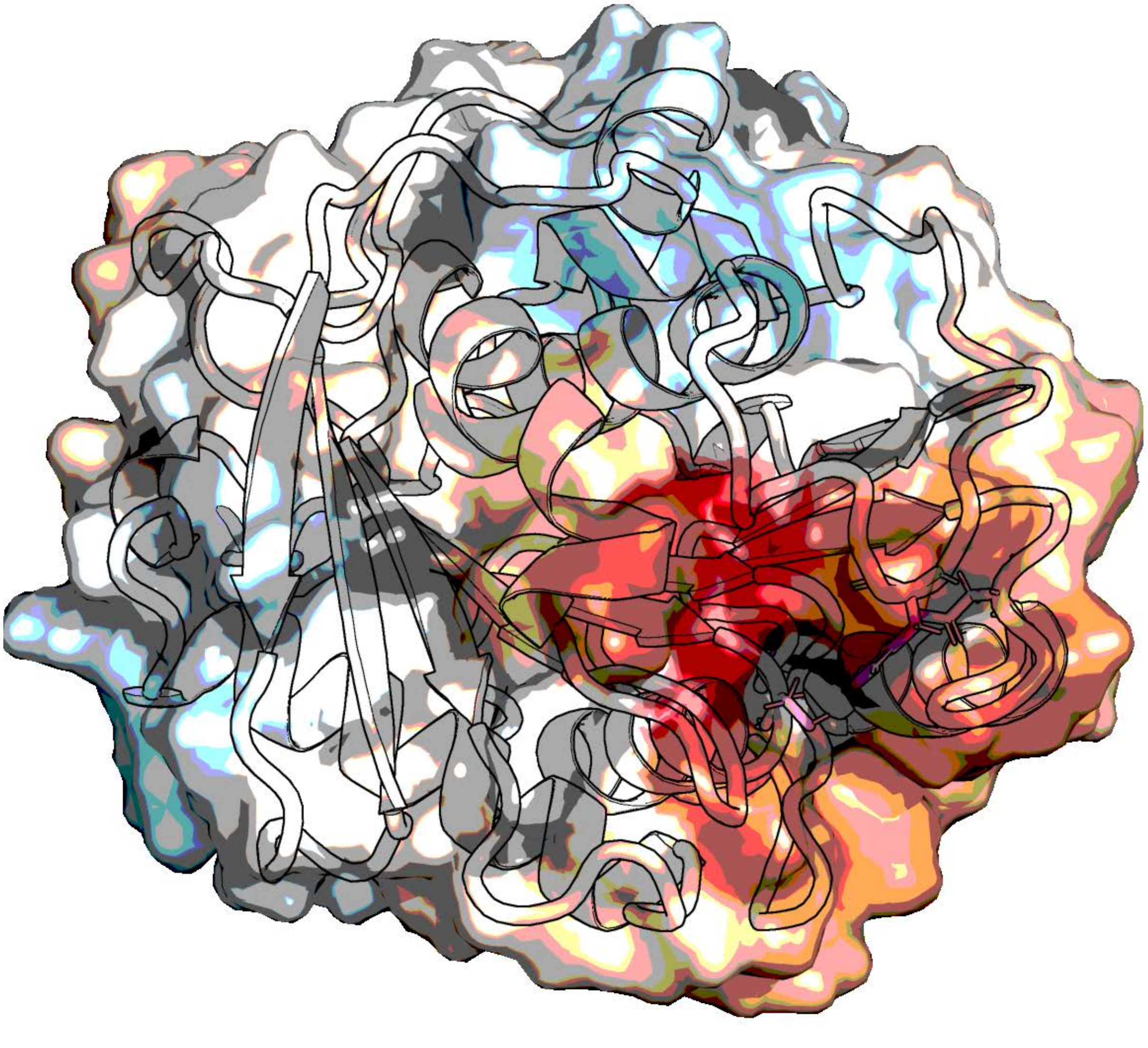

TEP083_highlights.png

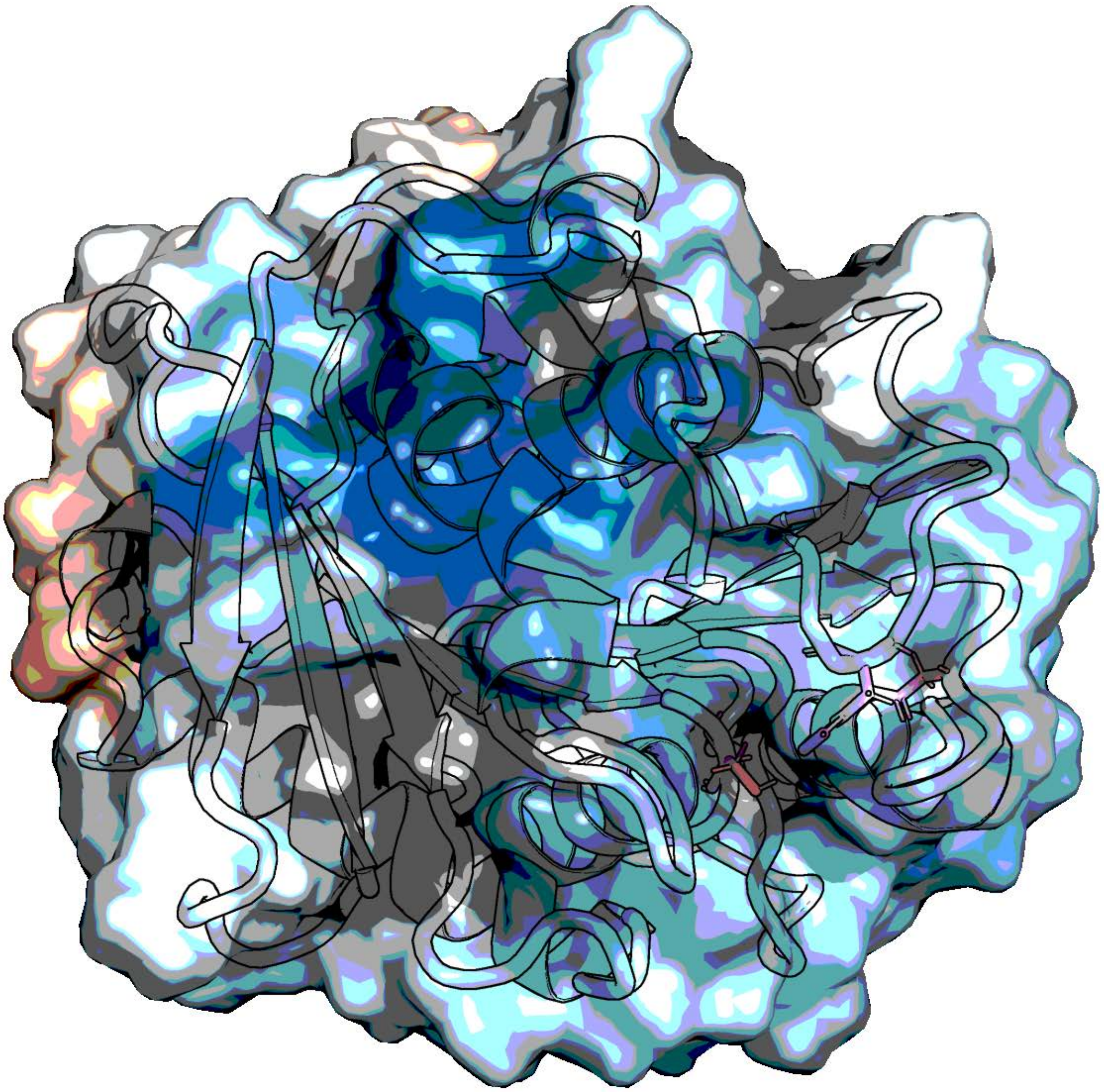

TEP091_highlights.png

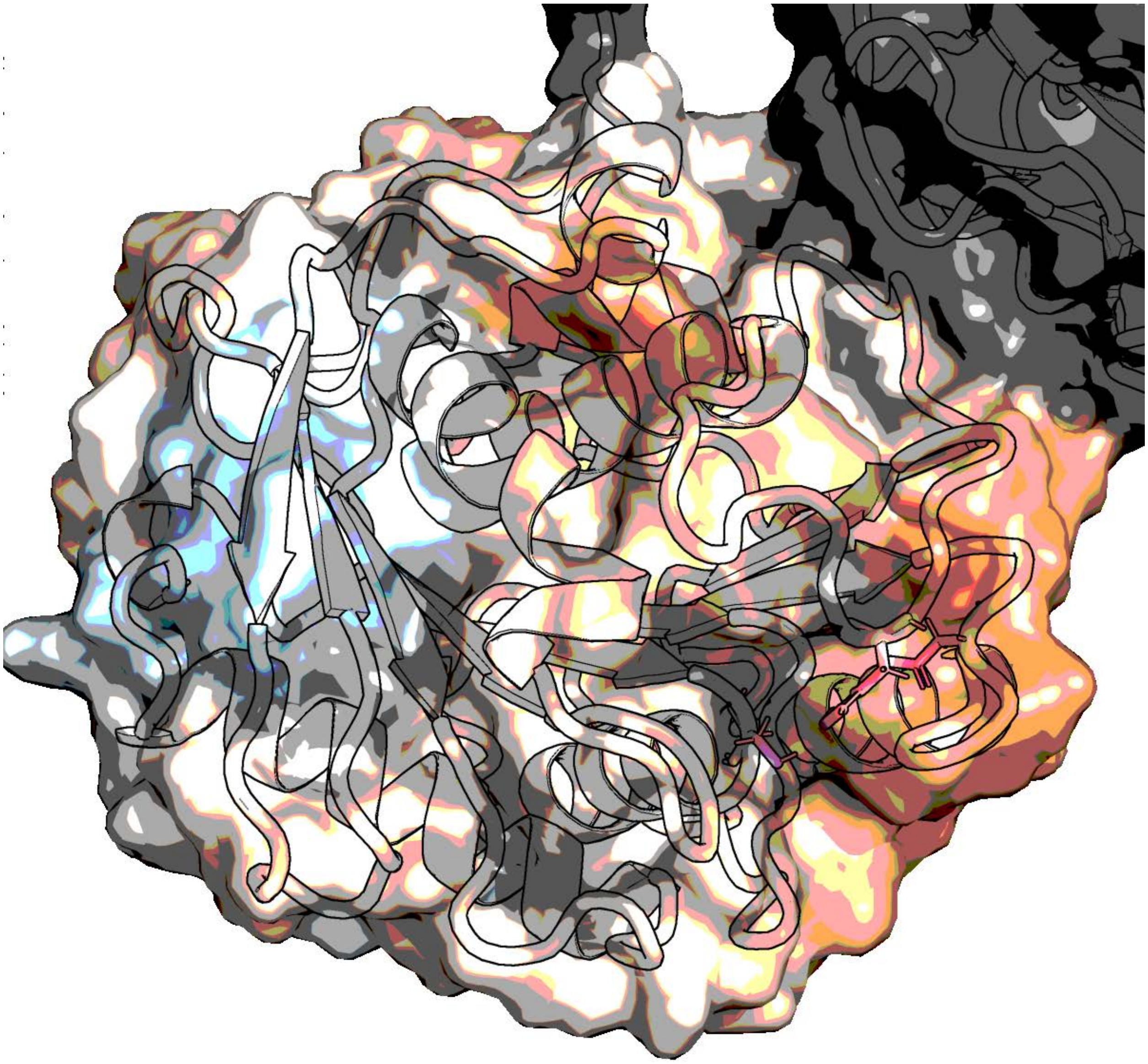

TEP092_highlights.png

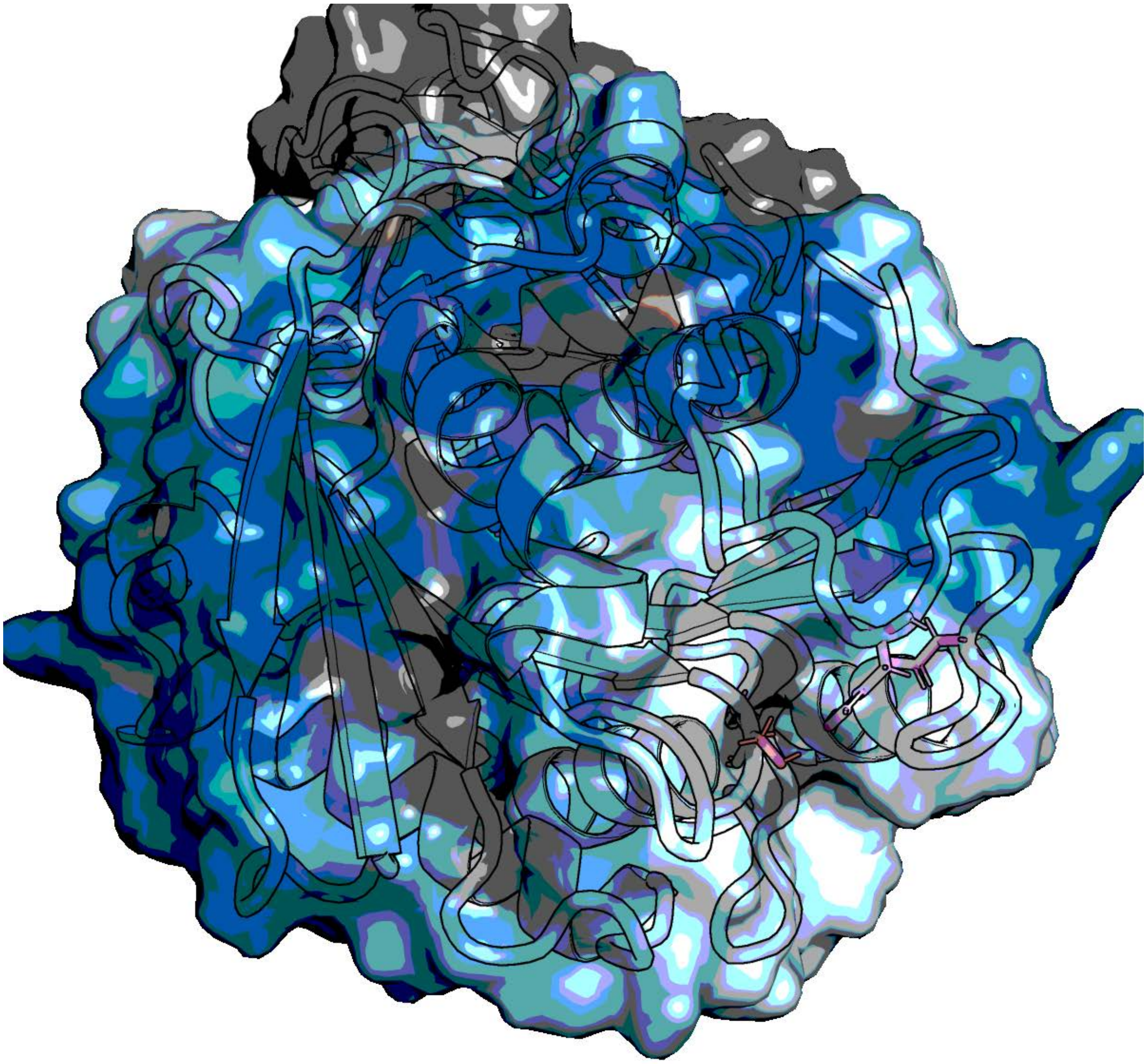

TEP093_highlights.png

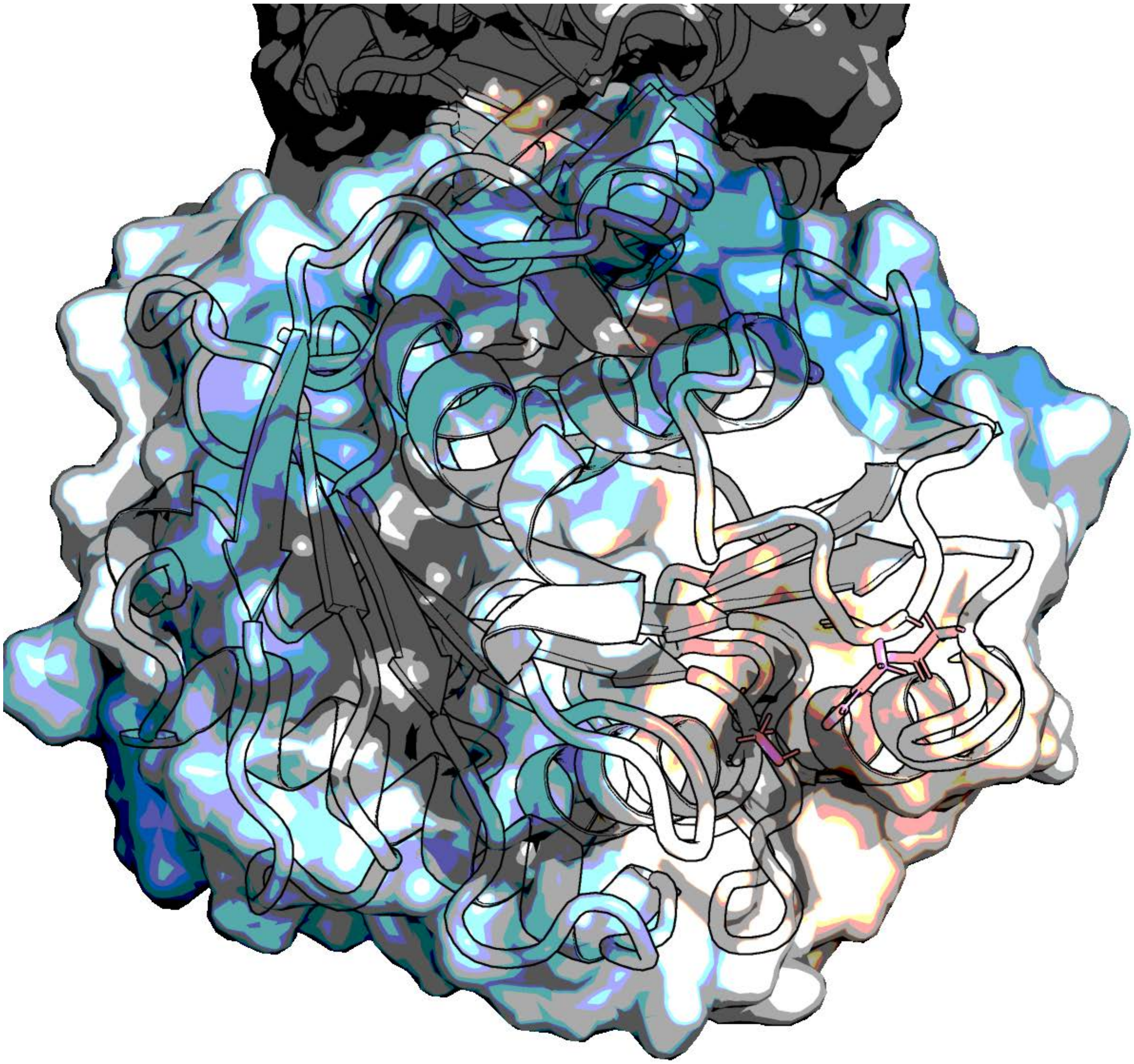

TEP097_highlights.png

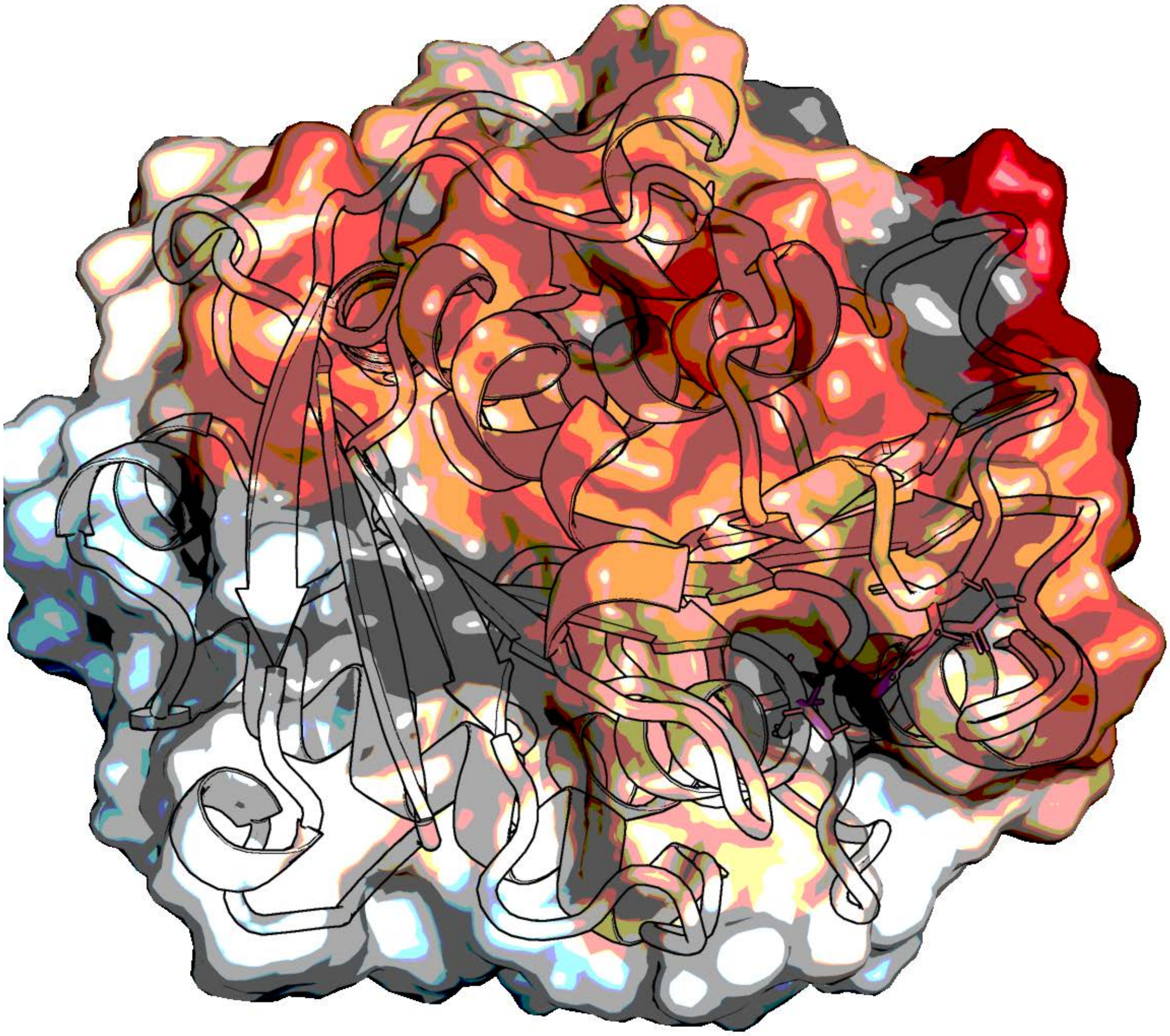

TEP101_highlights.png

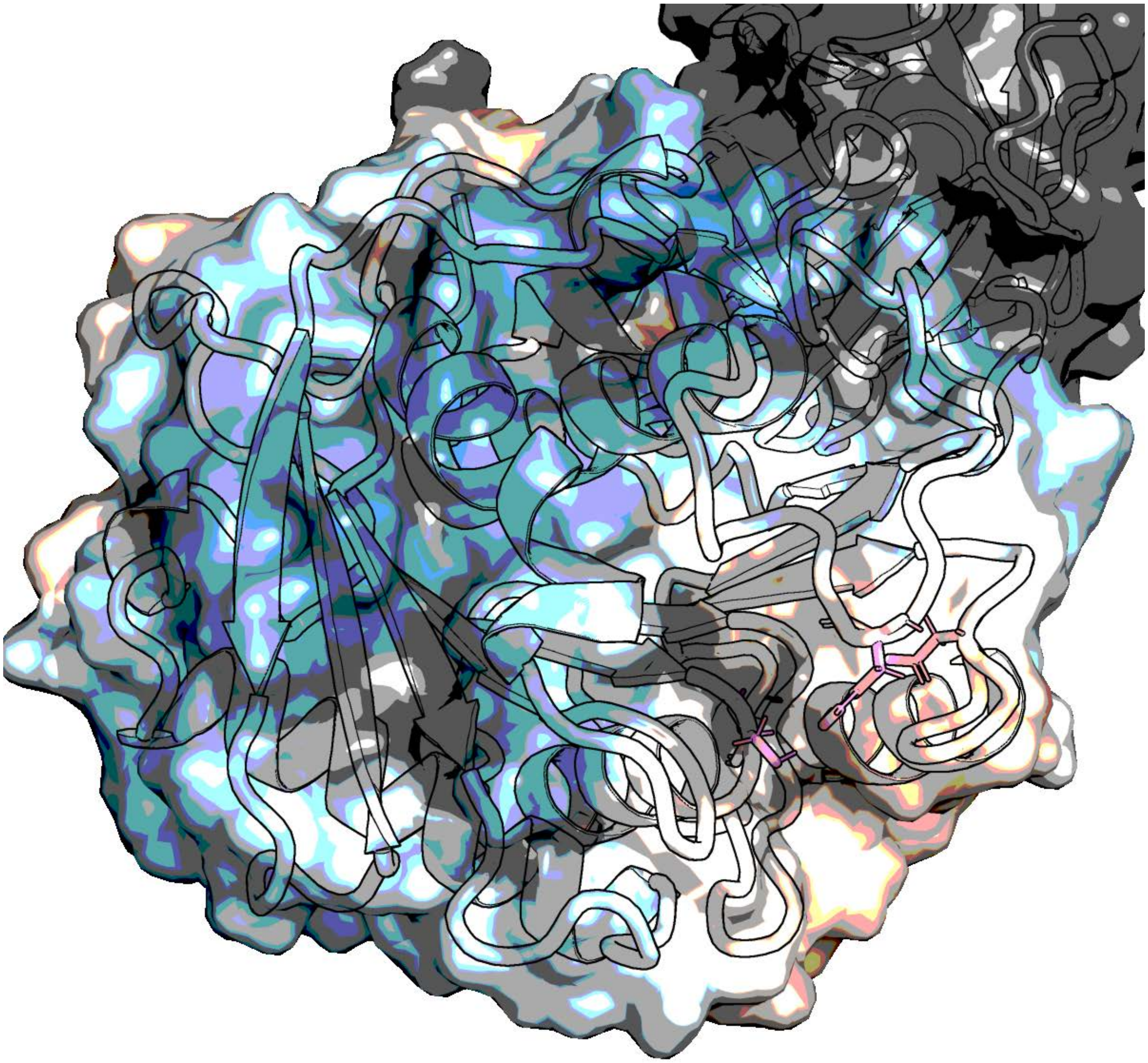

TEP102_highlights.png

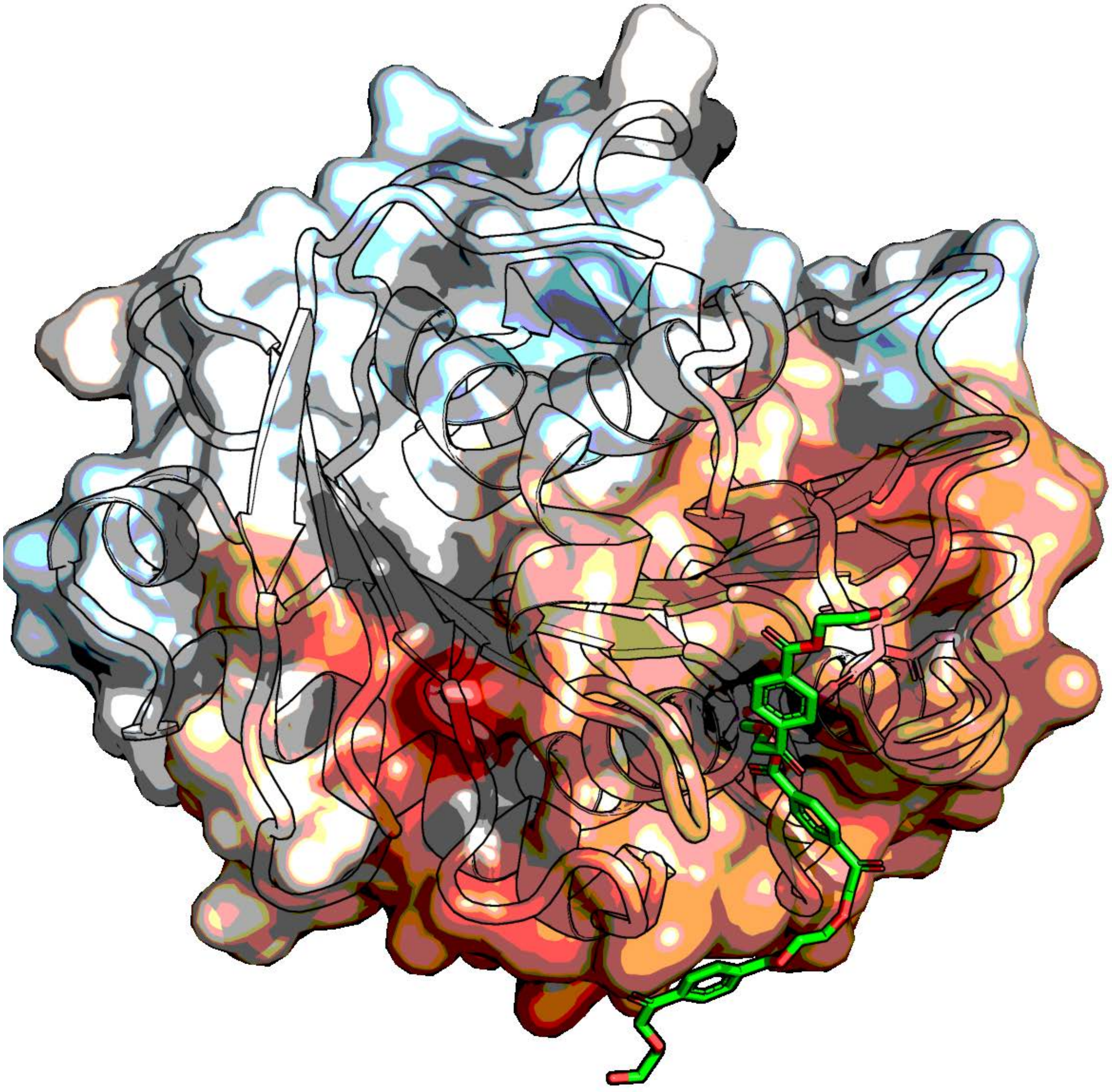

TEP103_highlights.png

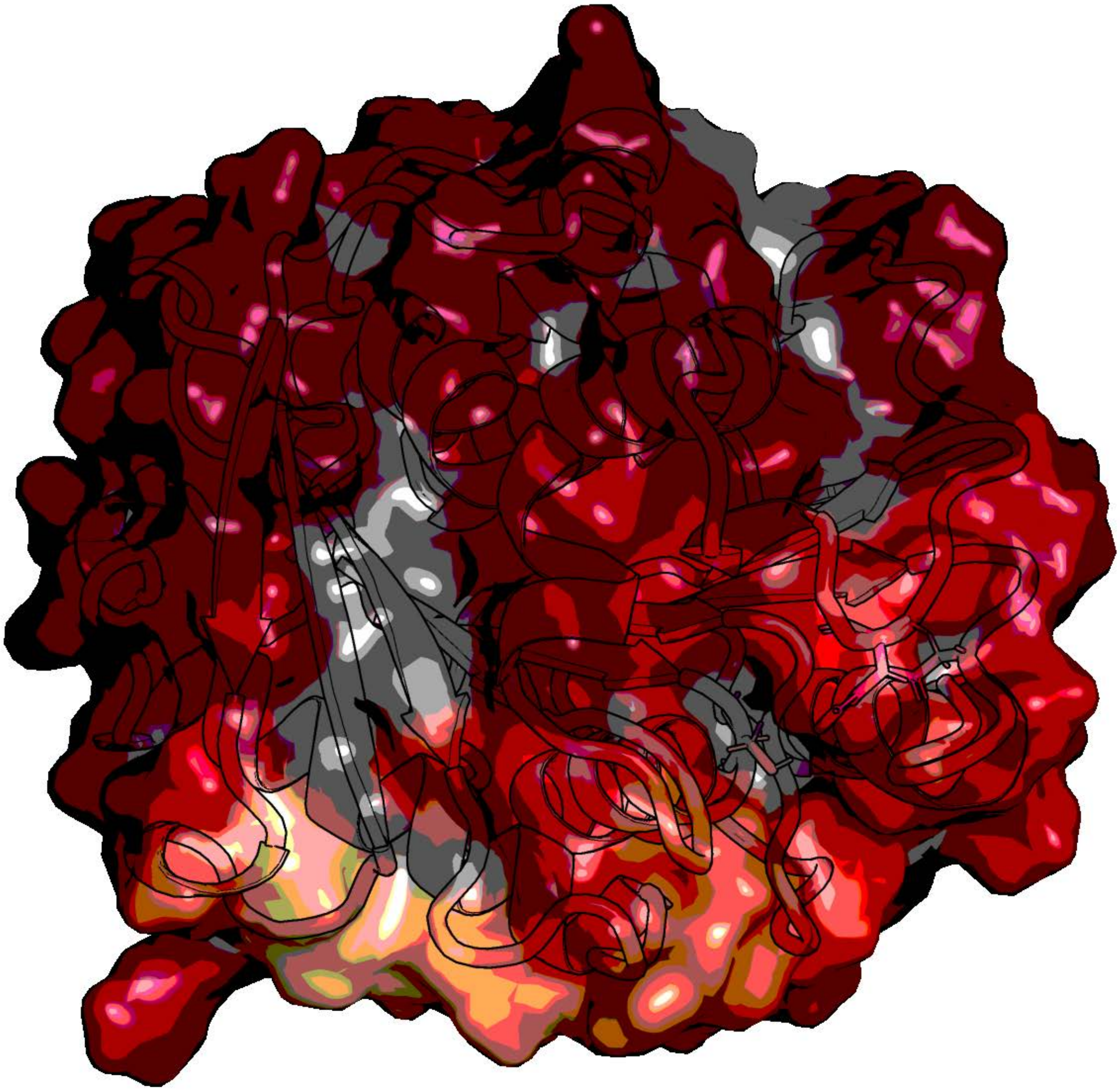

TEP105_highlights.png

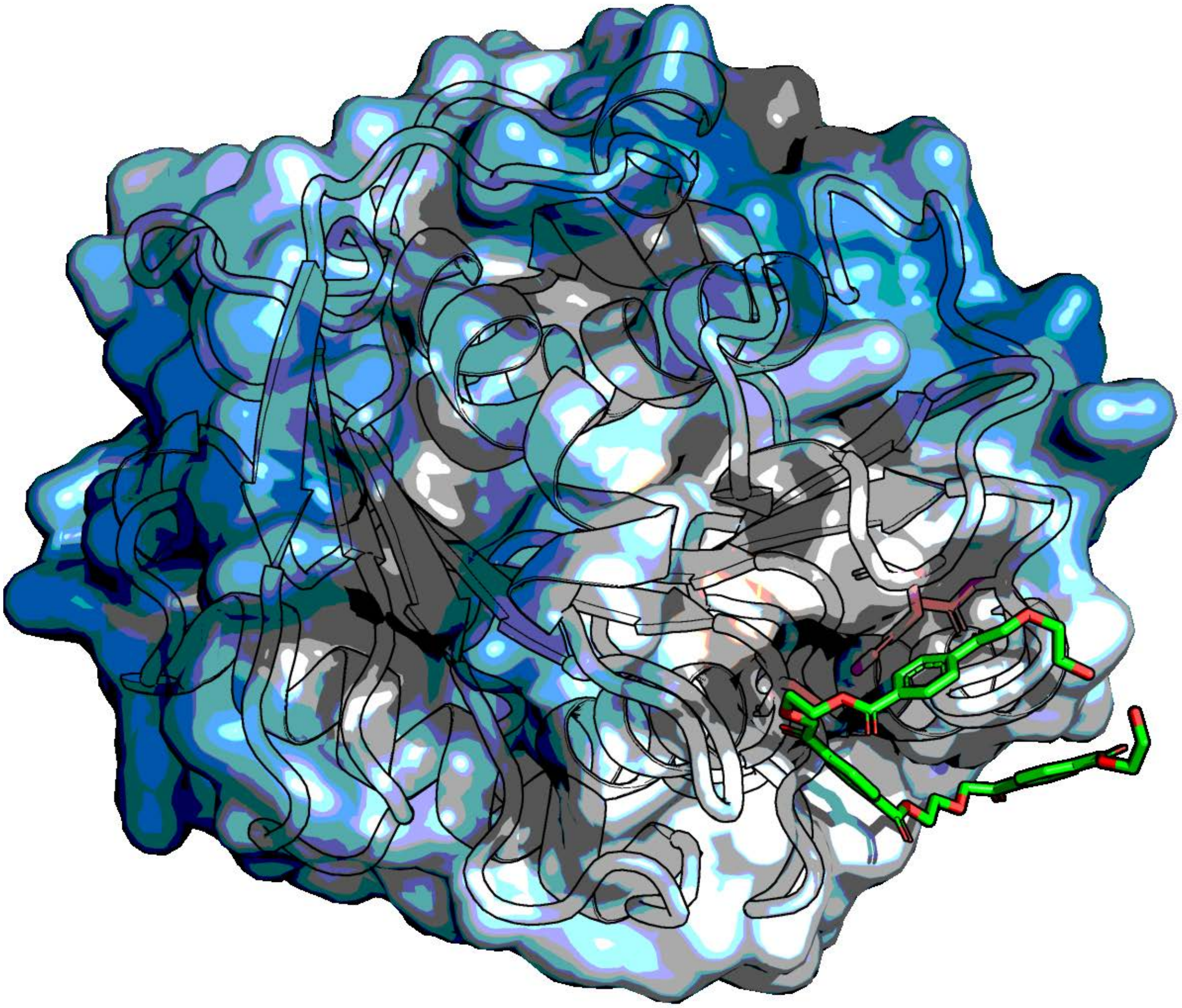

TEP109_highlights.png

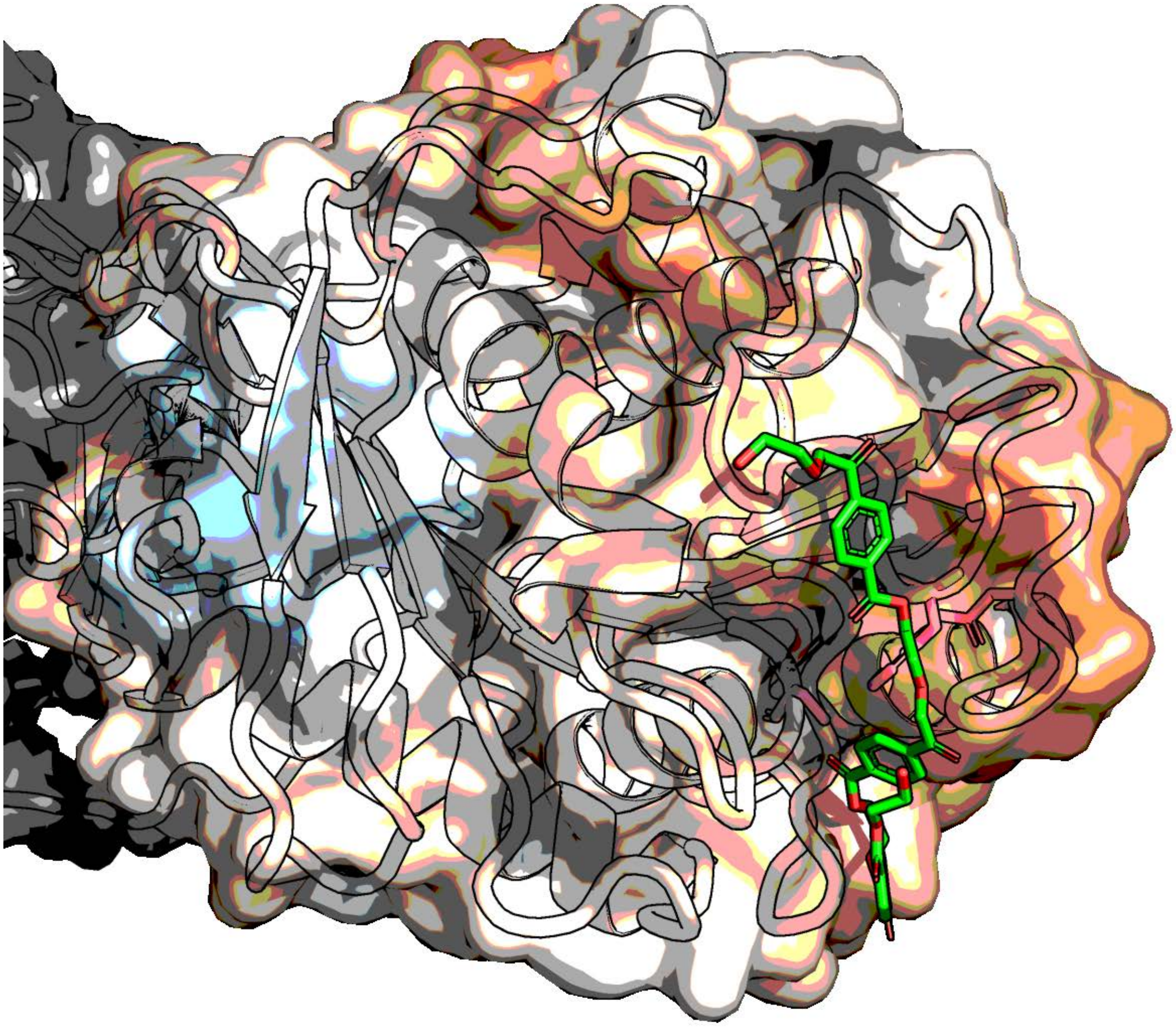

TEP111_highlights.png

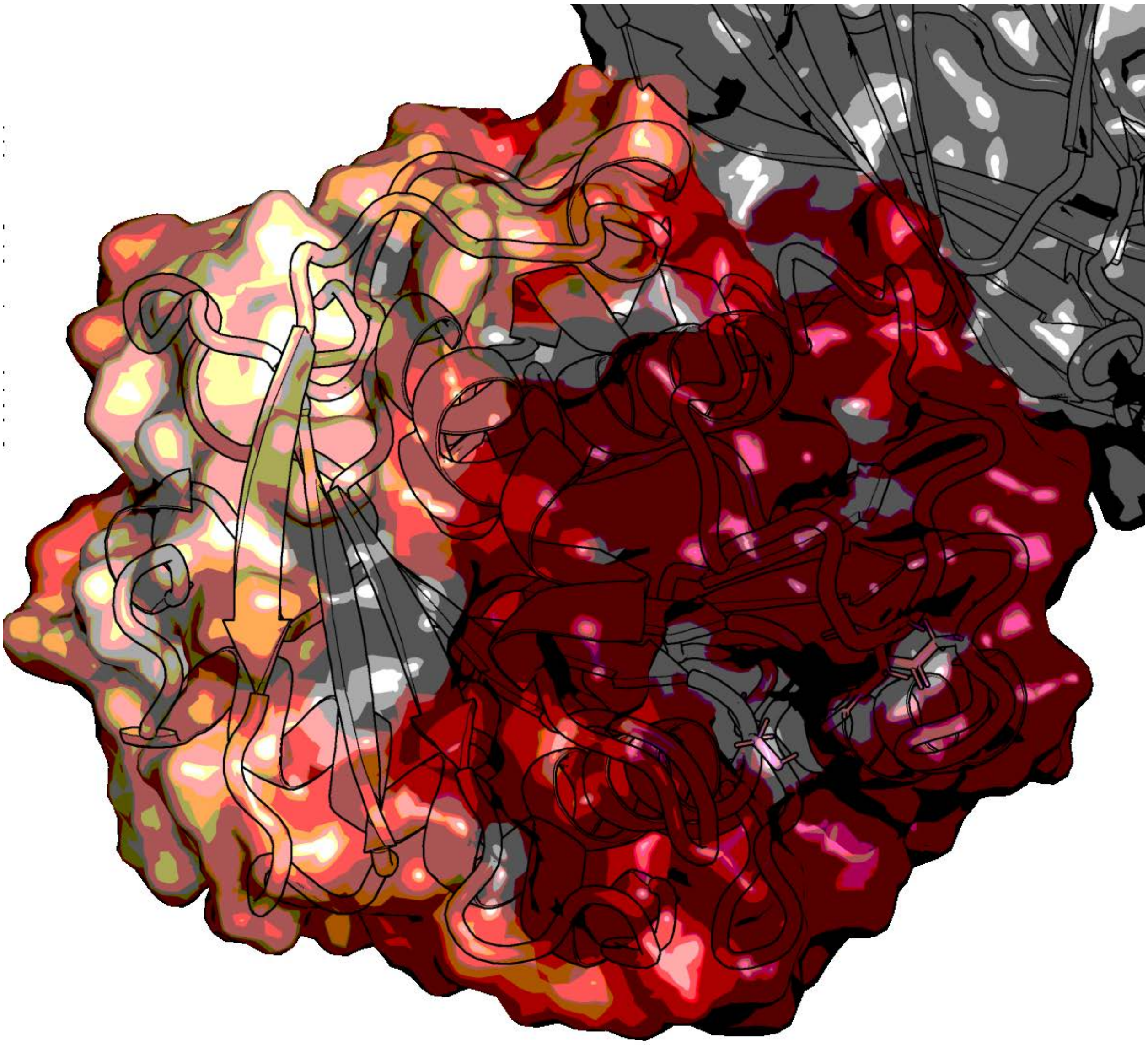

TEP114_highlights.png

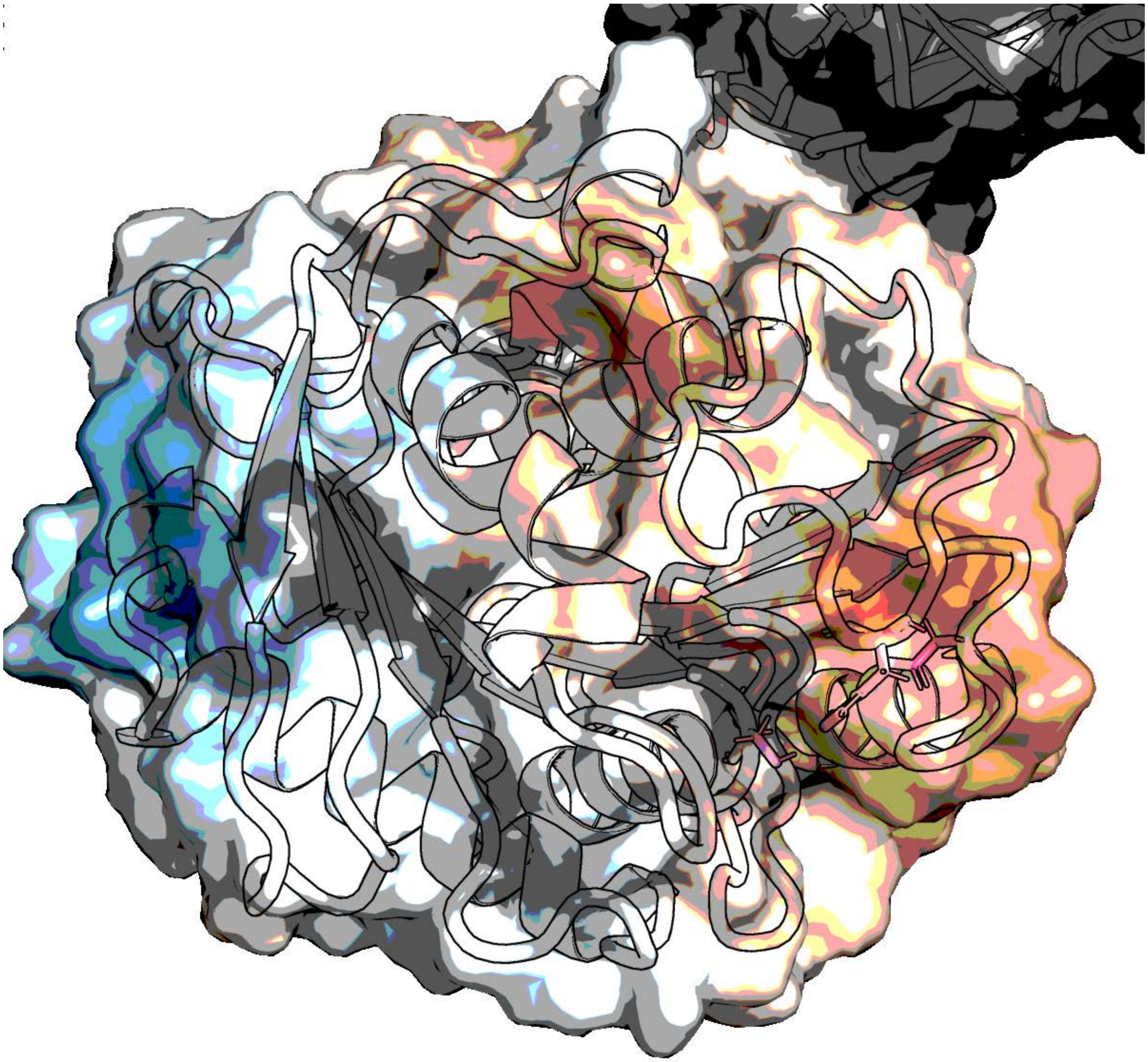

TEP115_highlights.png

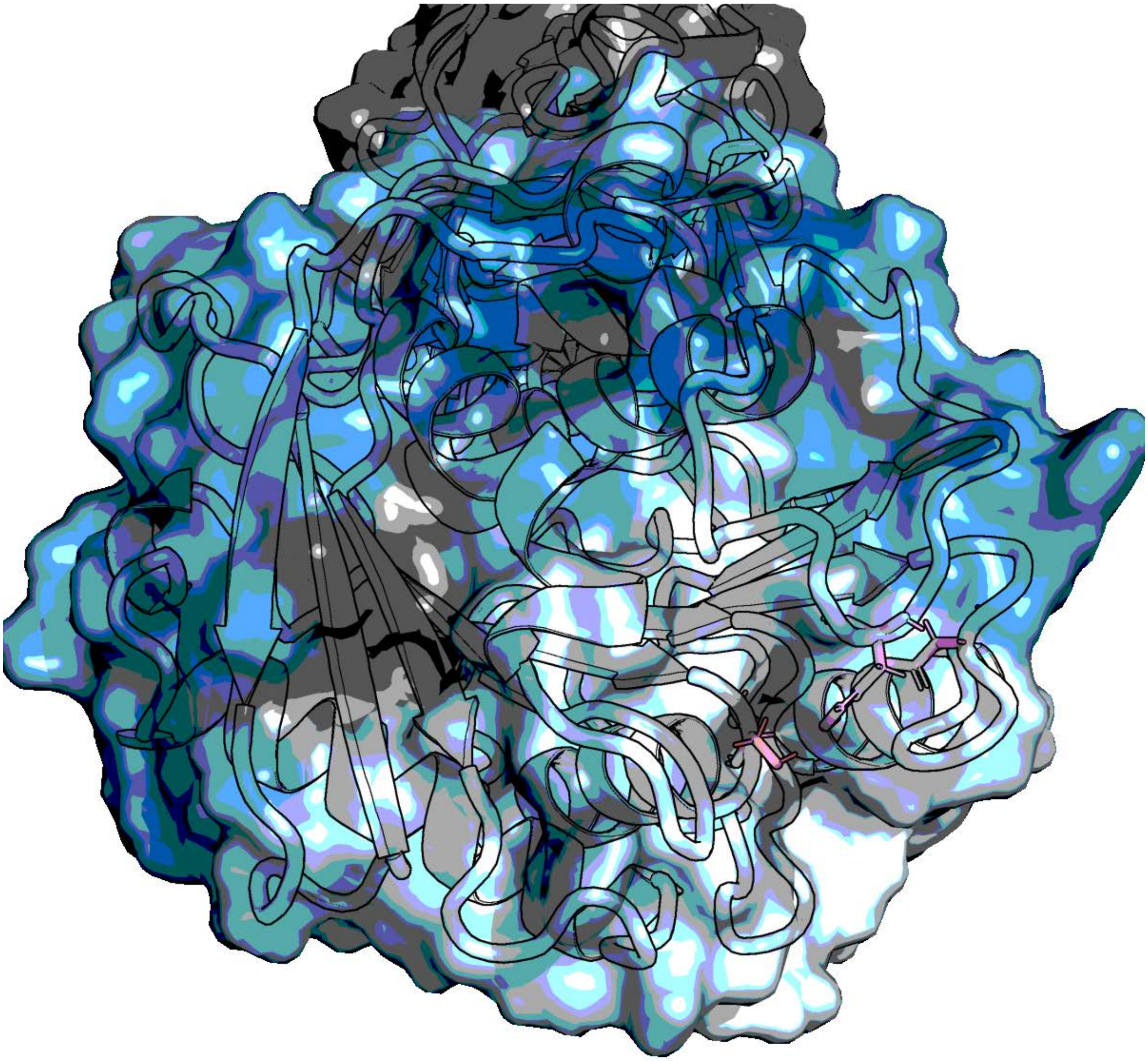

TEP131_highlights.png

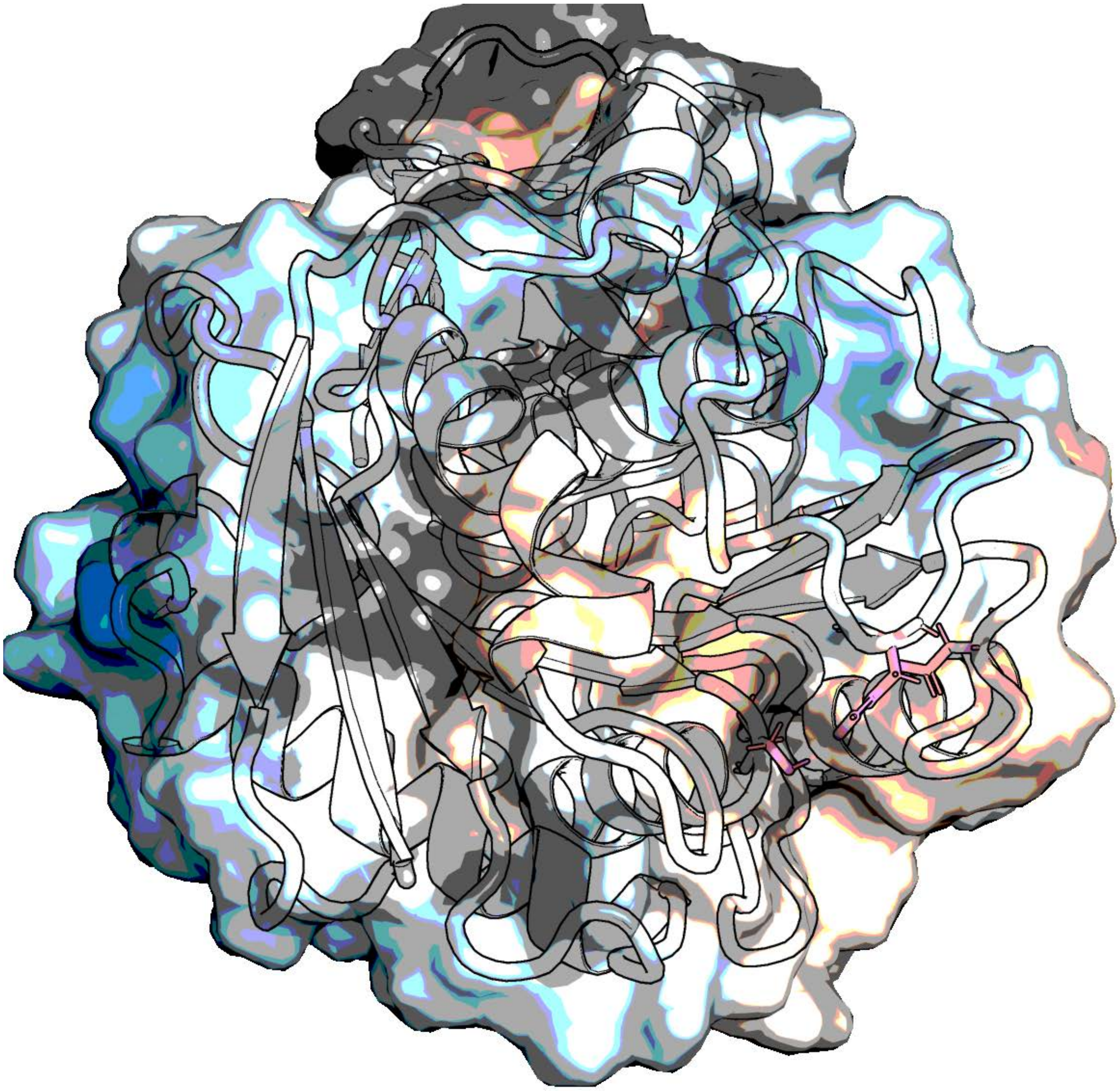

TEP163_highlights.png

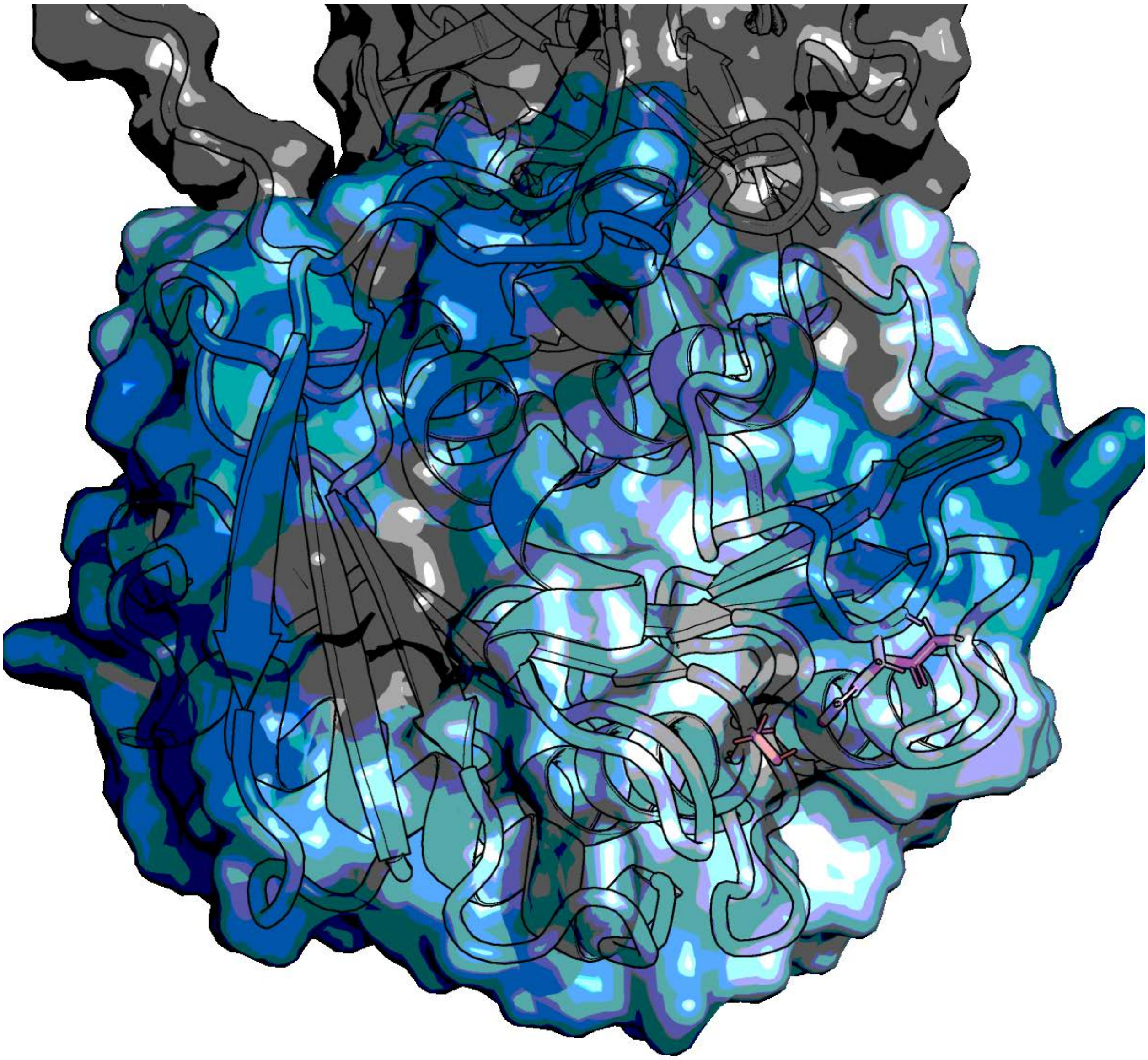

TEP164_highlights.png

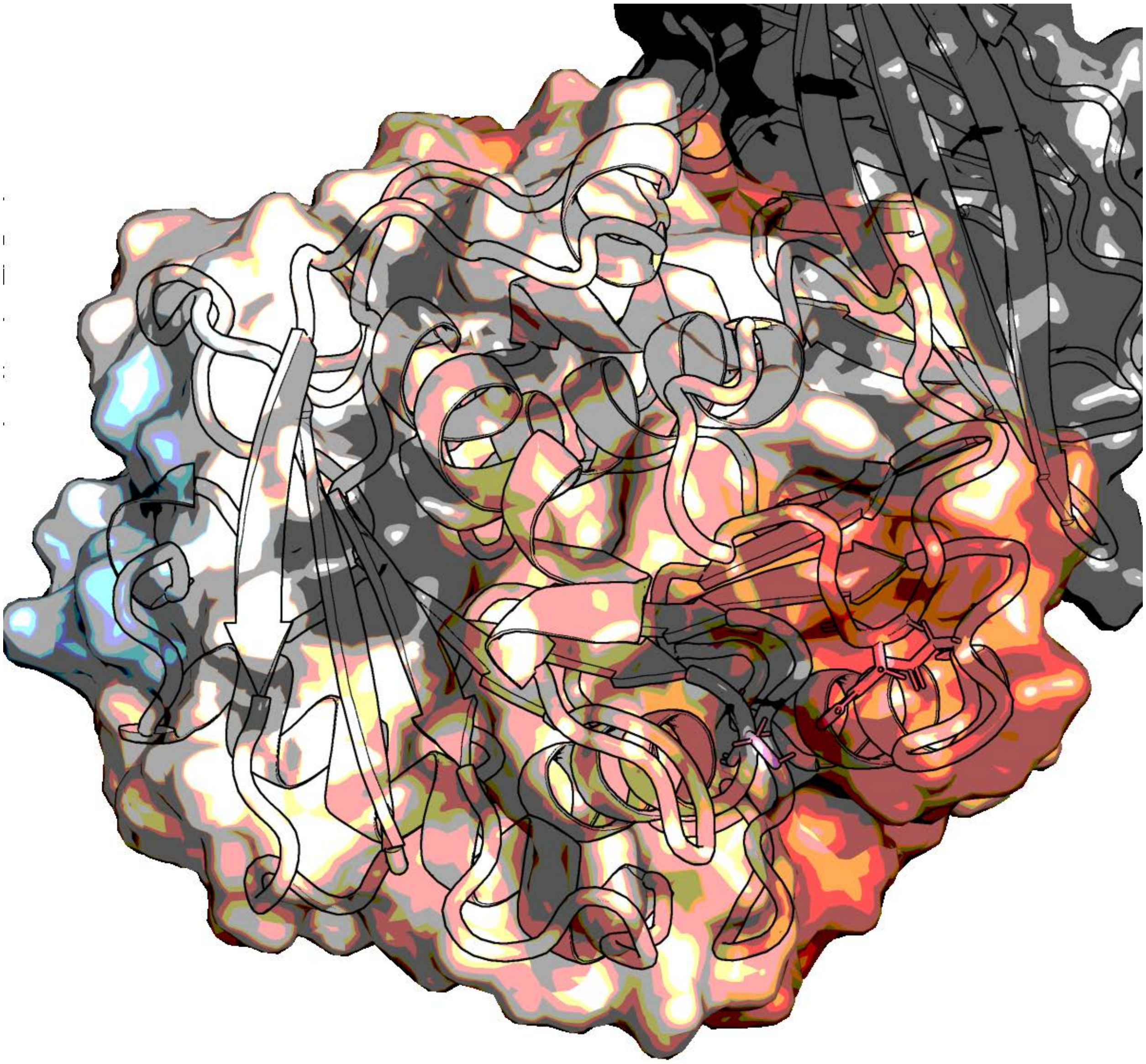

TEP165_highlights.png

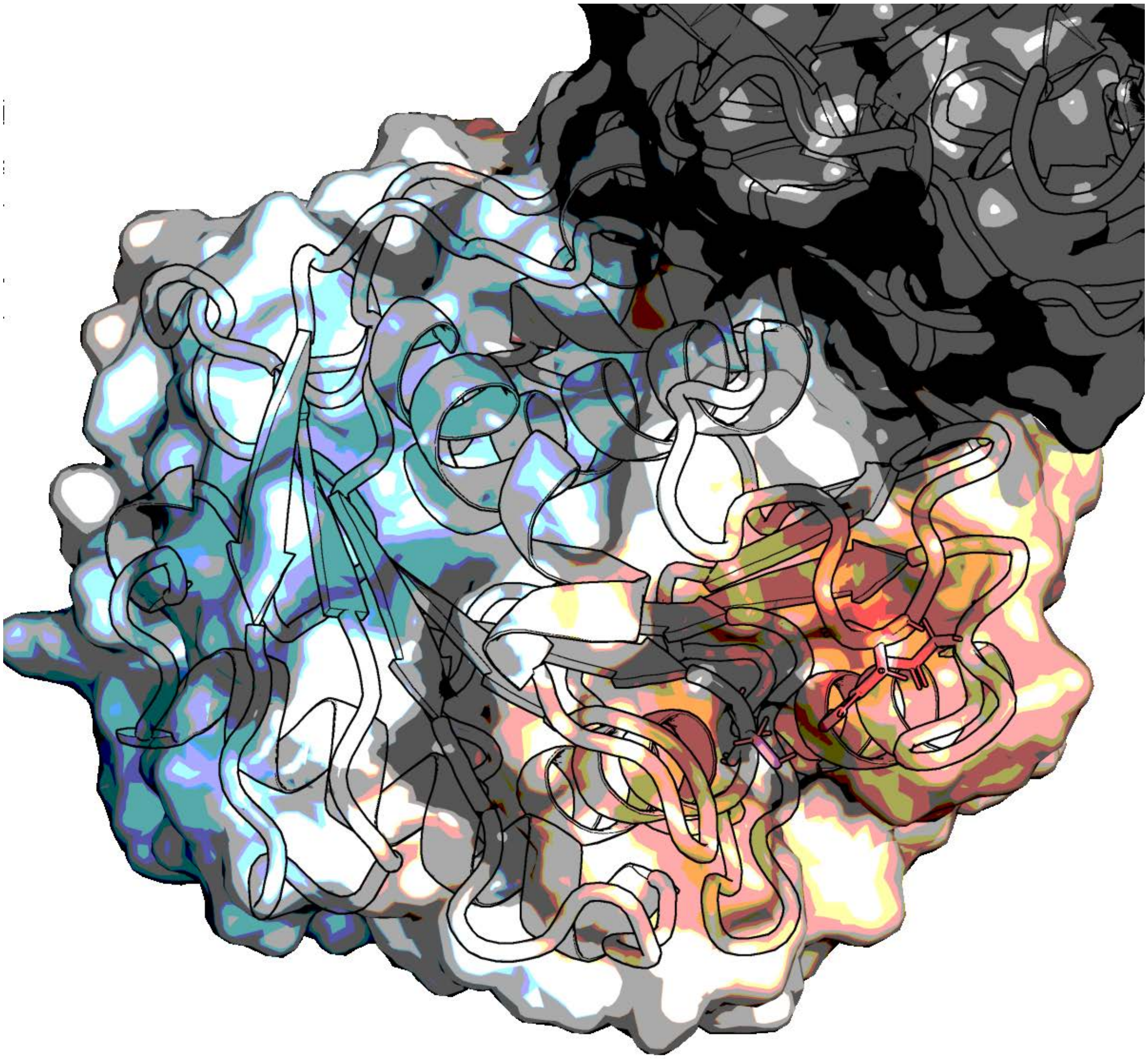

TEP168_highlights.png

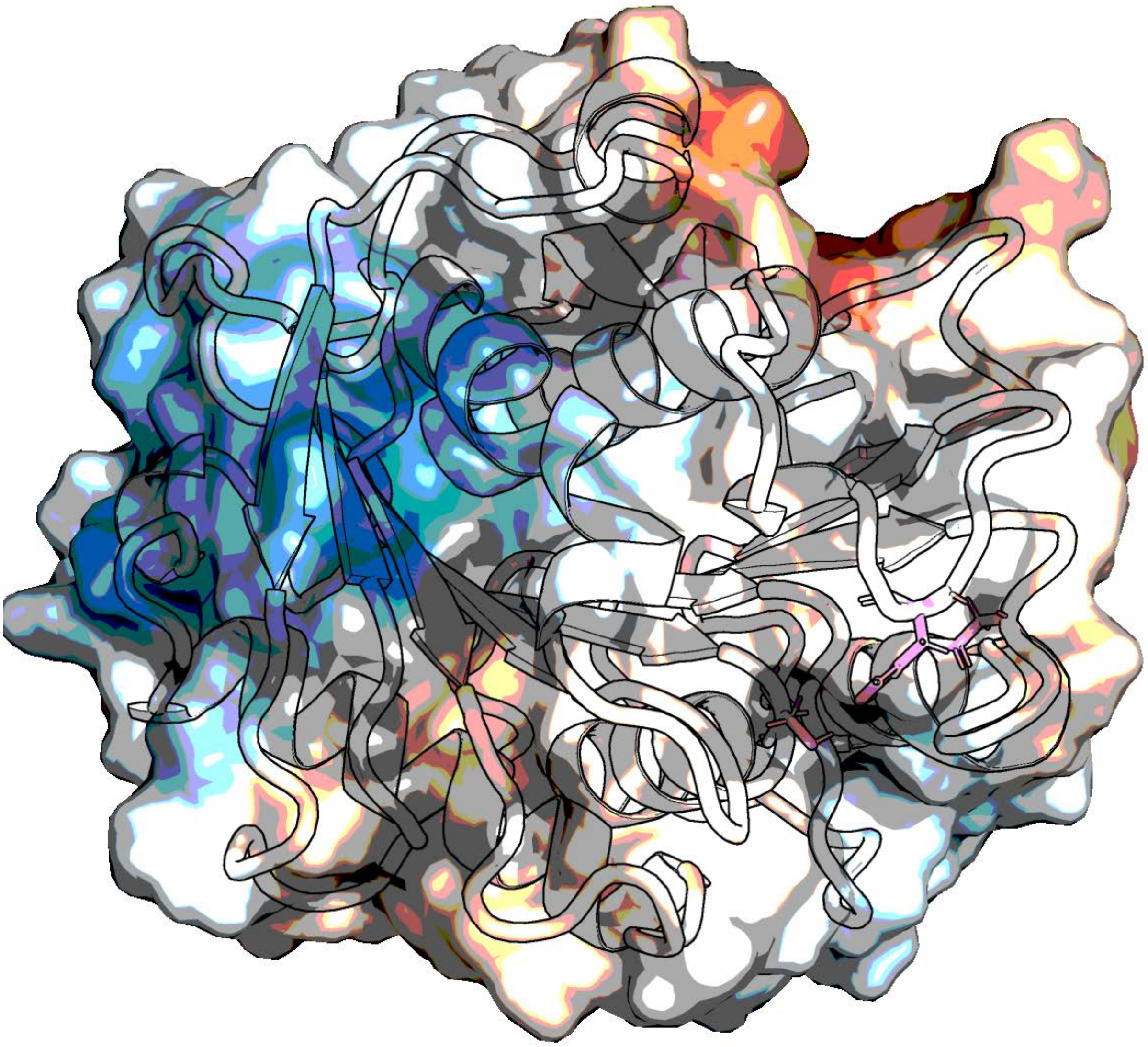

TEP178_highlights.png

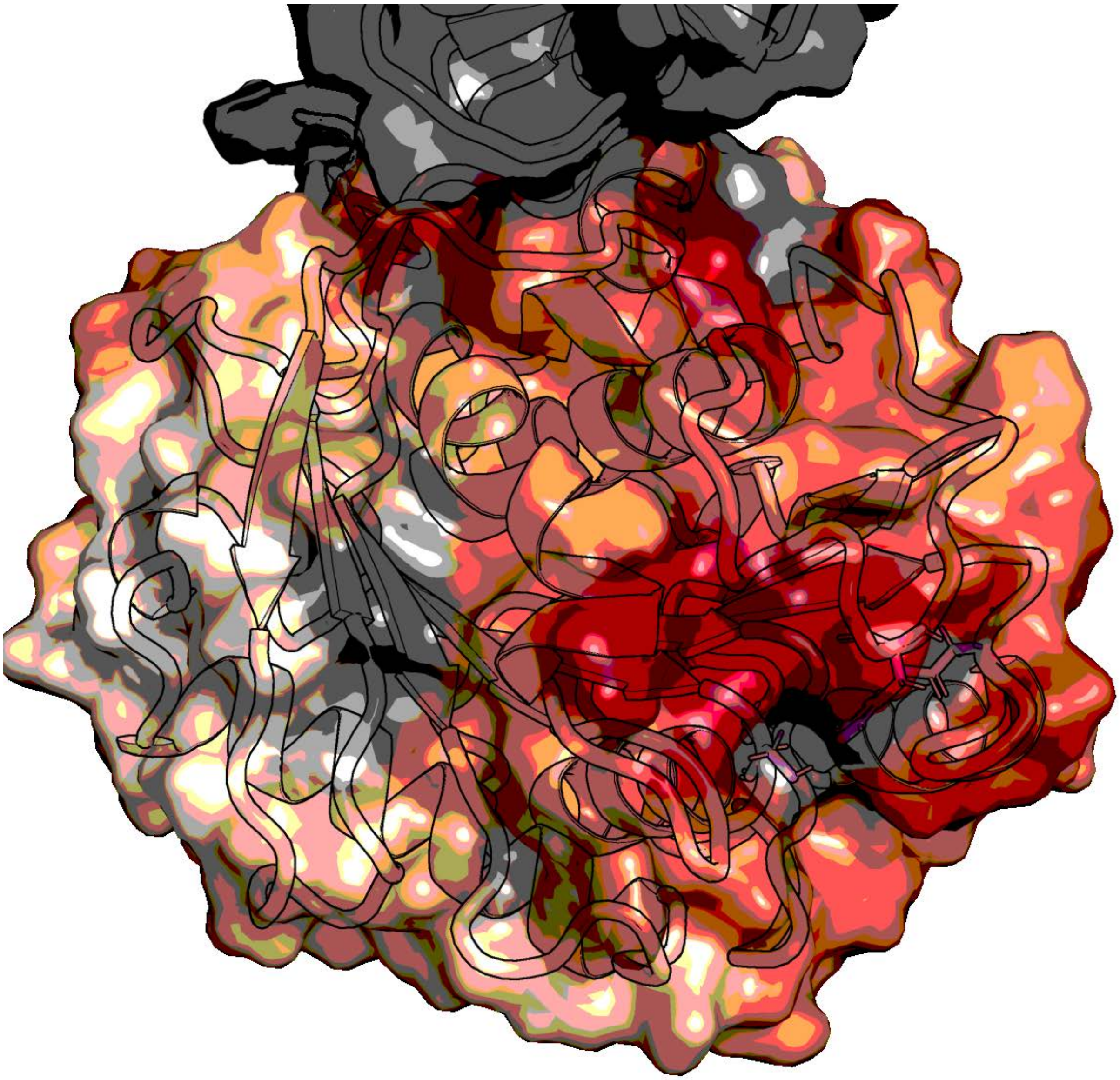

TEP181_highlights.png

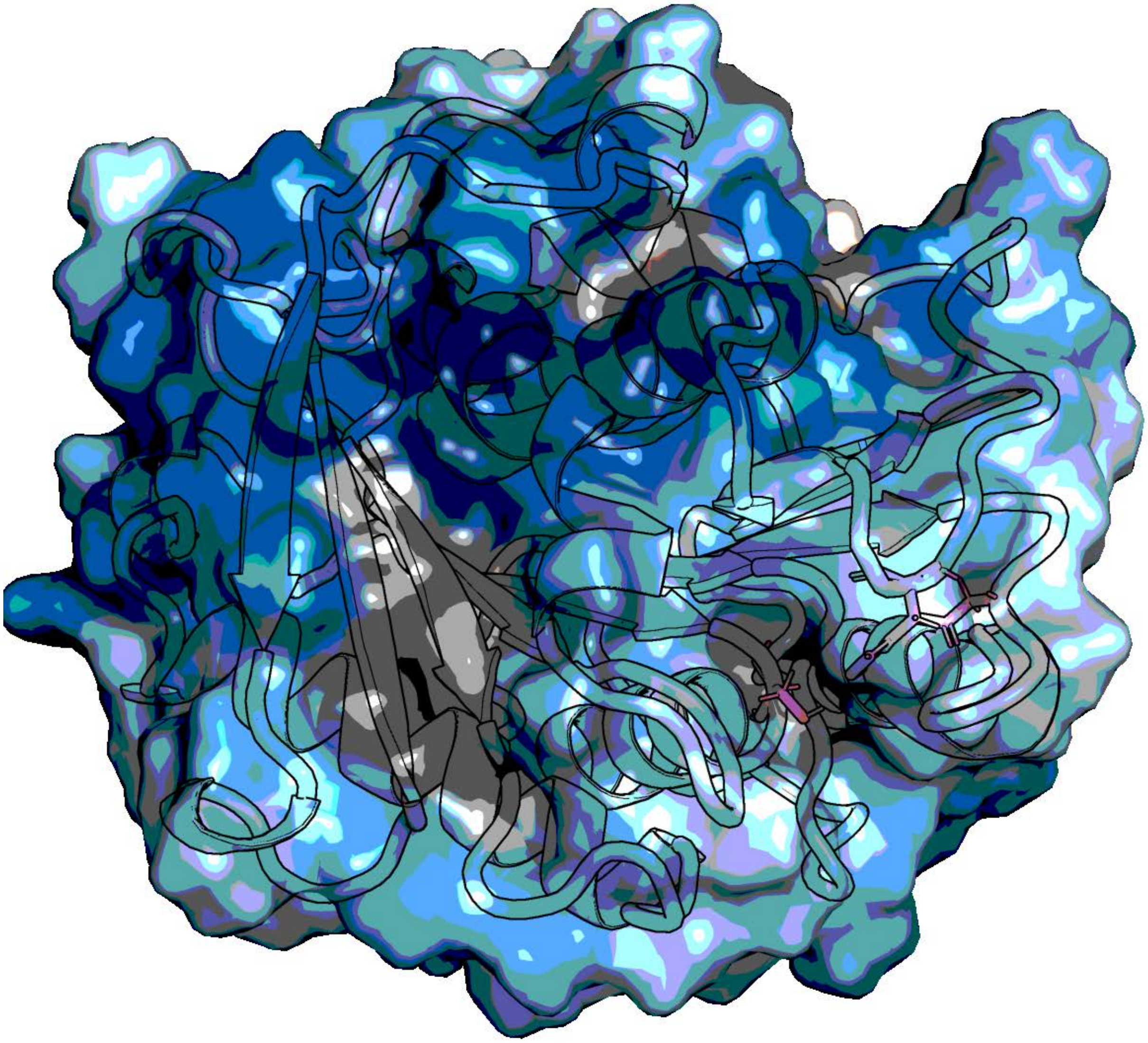

TEP182_highlights.png

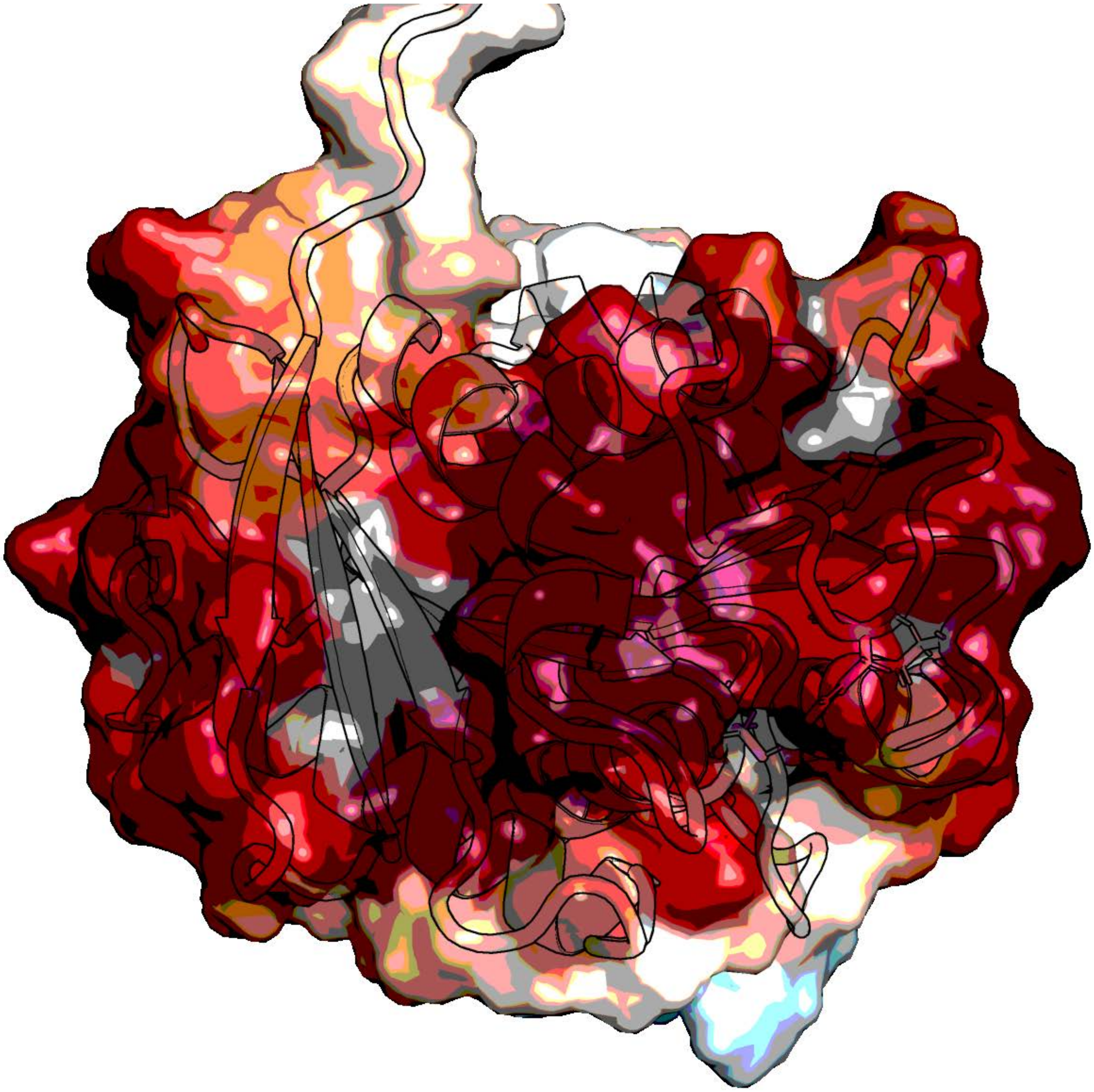

TEP189_highlights.png

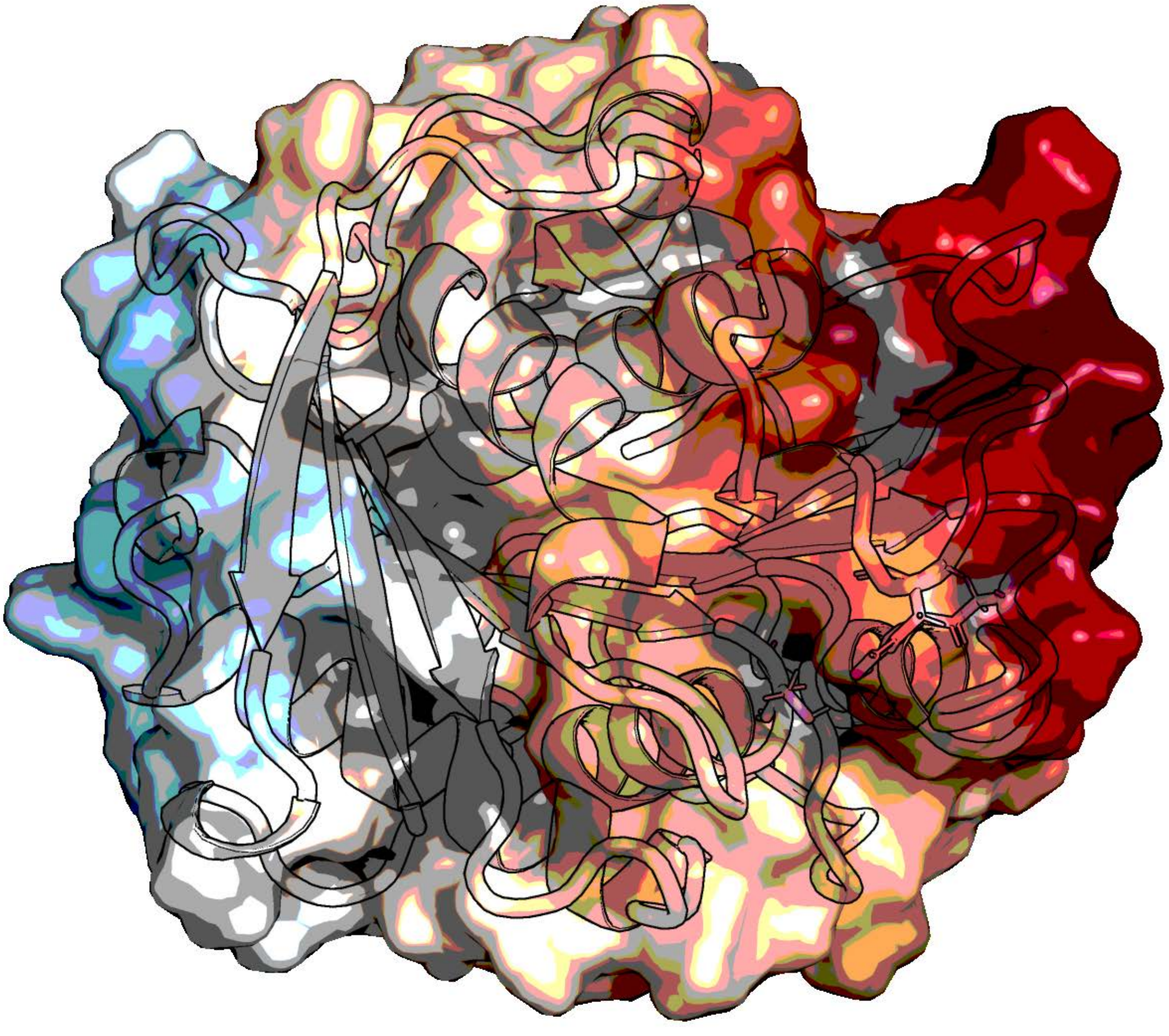

TEP194_highlights.png

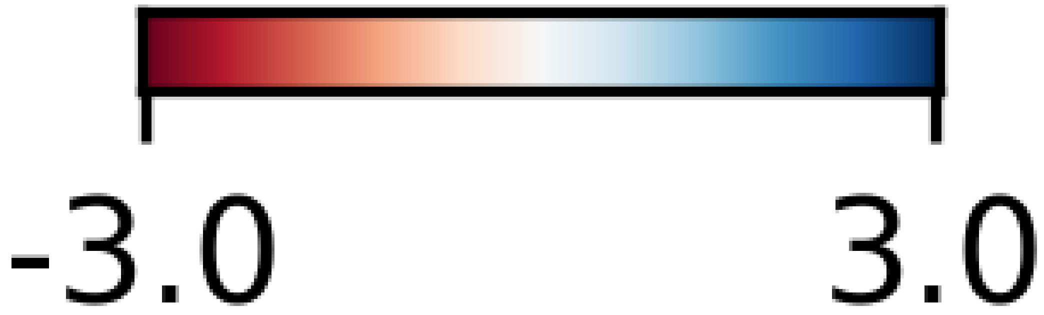

_LEGEND.png

Observed Hydrophobicity

Created on: 2025-04-15 16:04:53

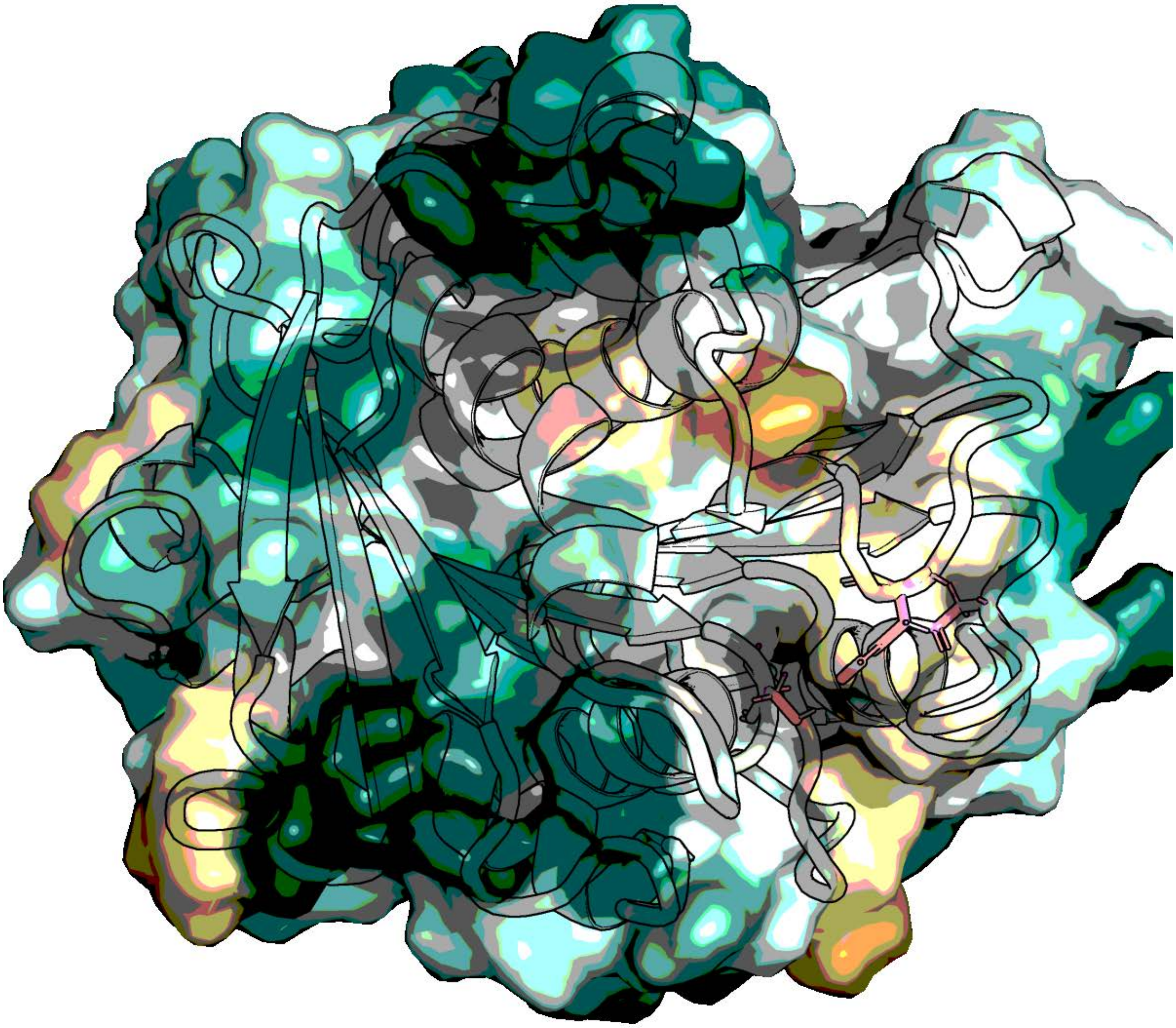

DP003_highlights.png

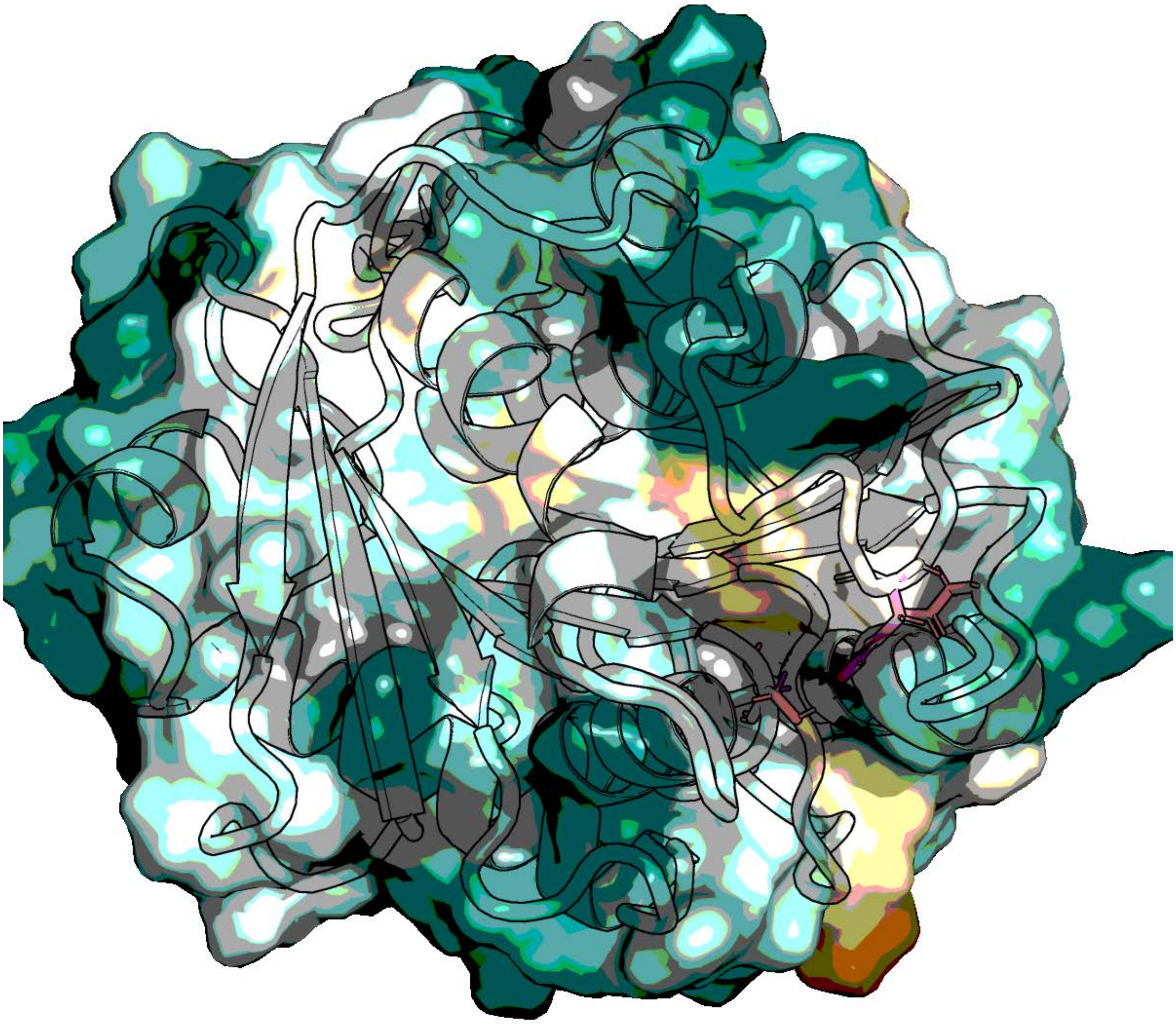

DP007_highlights.png

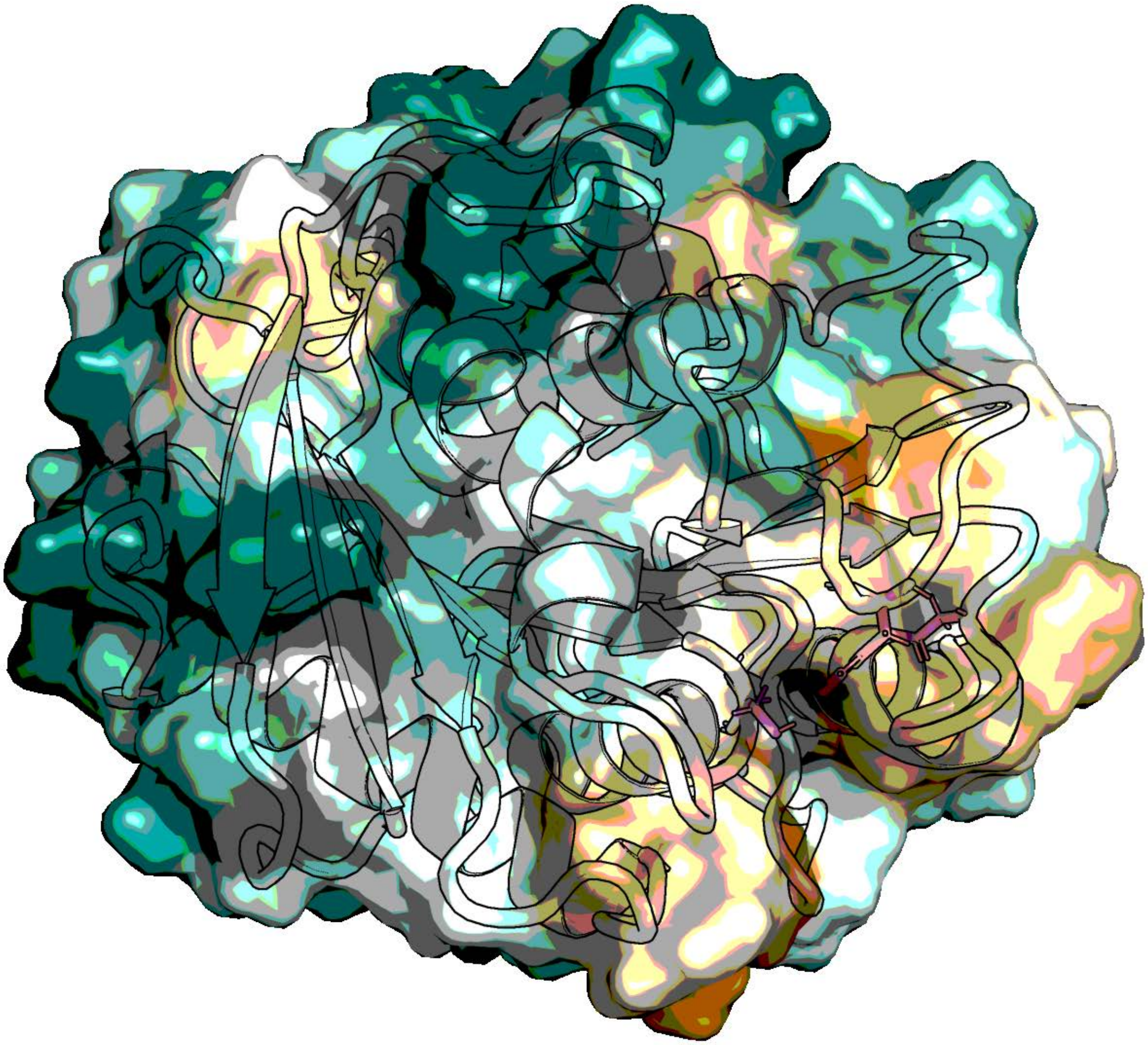

DP008_highlights.png

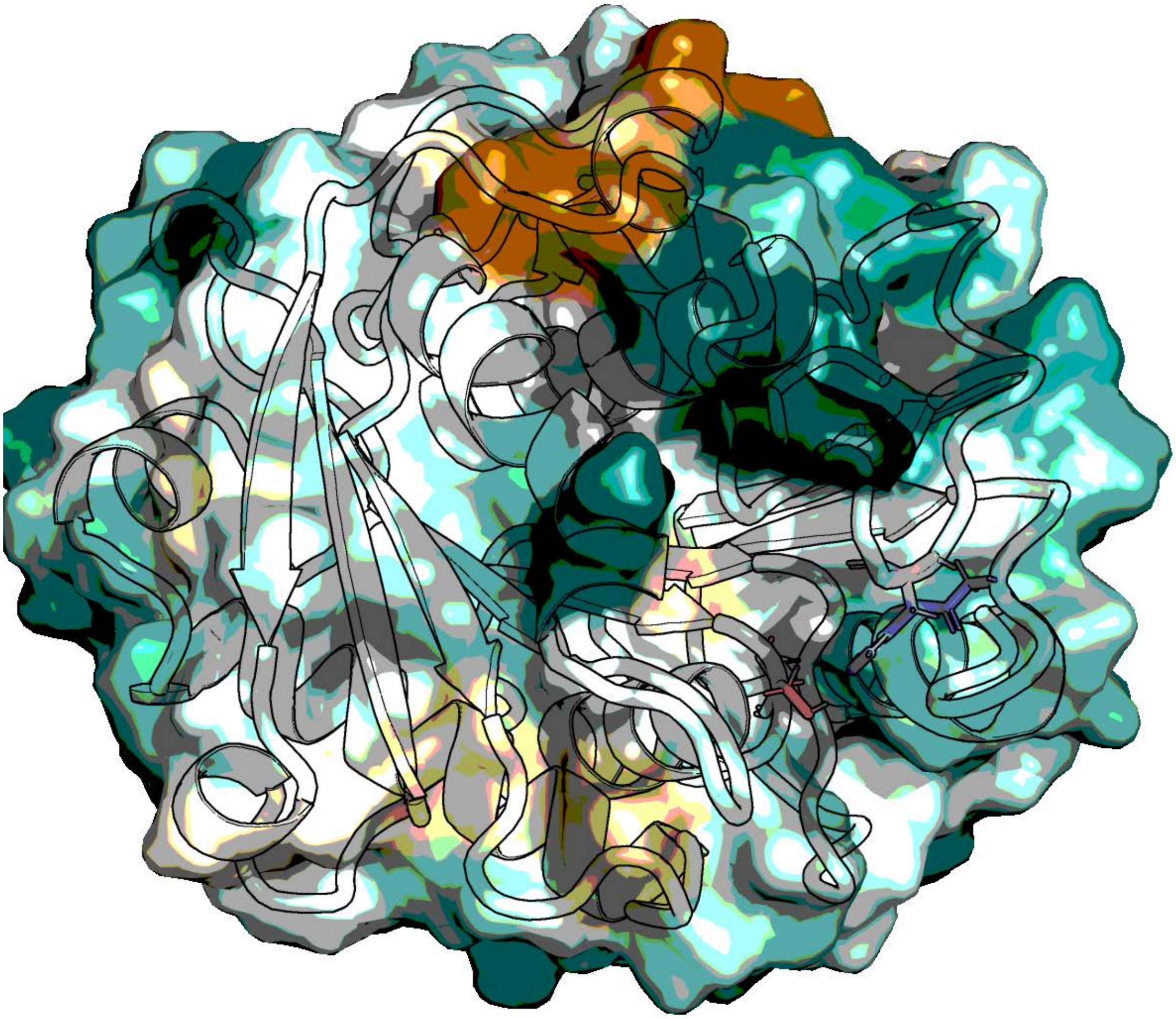

DP009_highlights.png

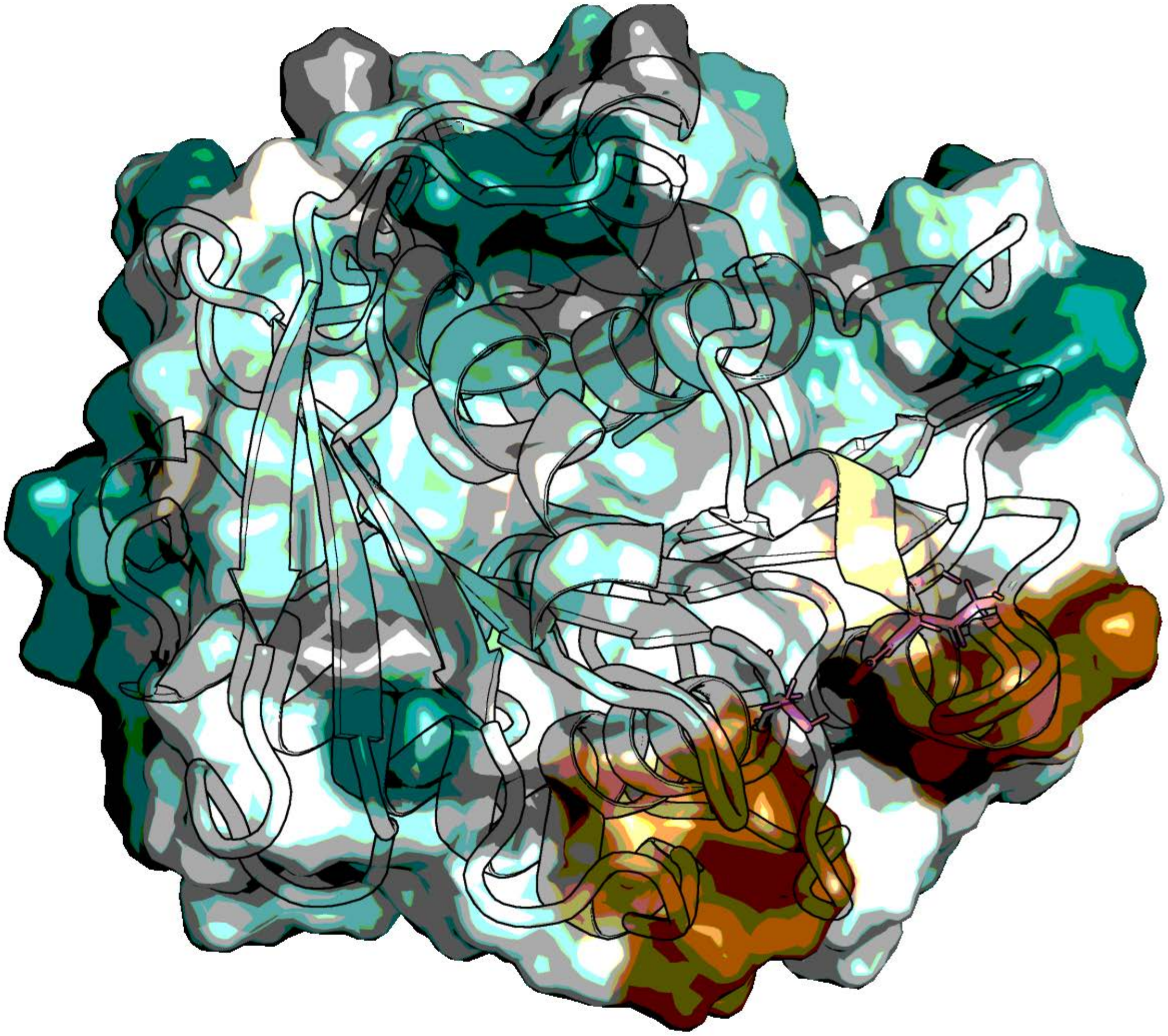

DP016_highlights.png

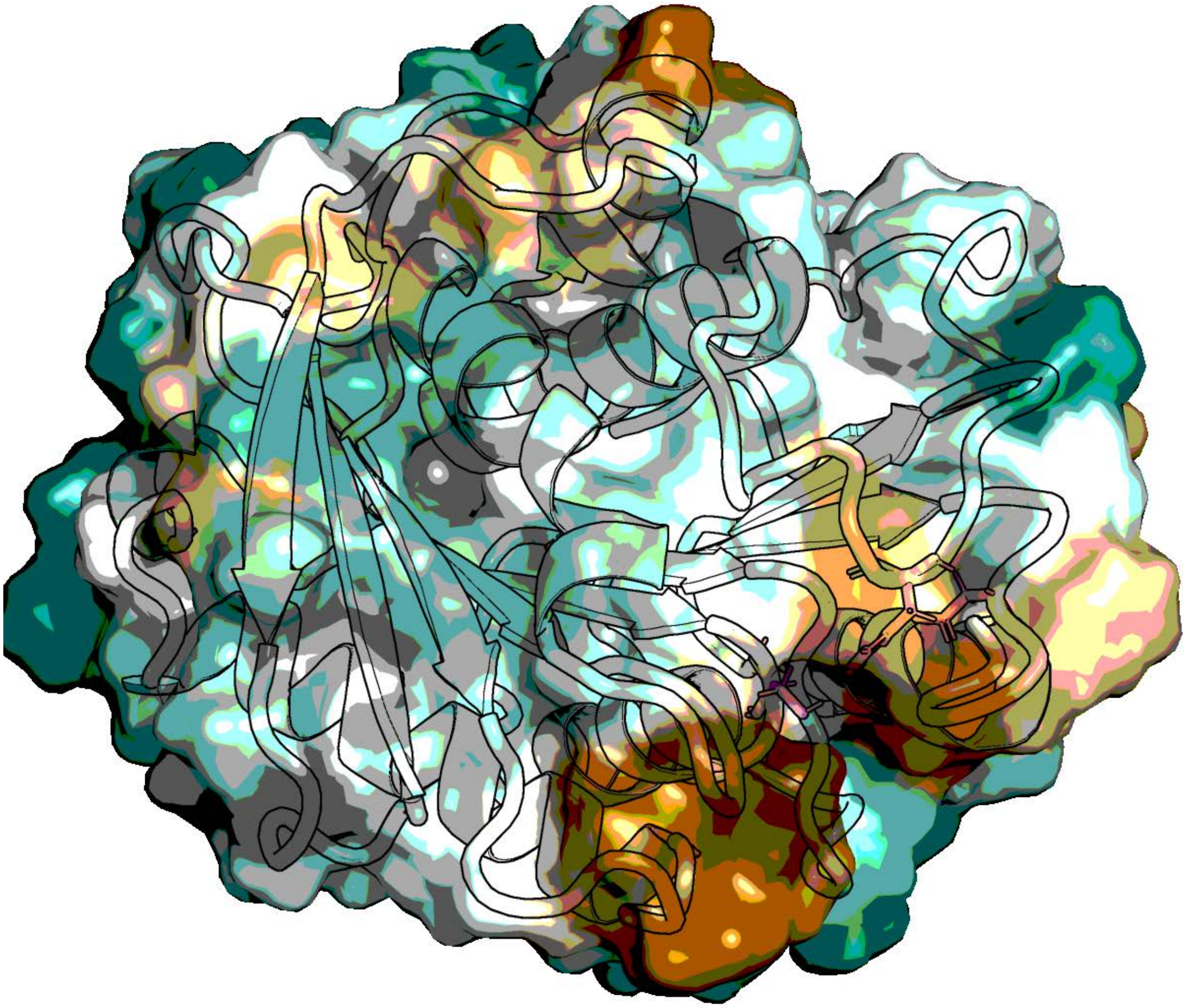

DP017_highlights.png

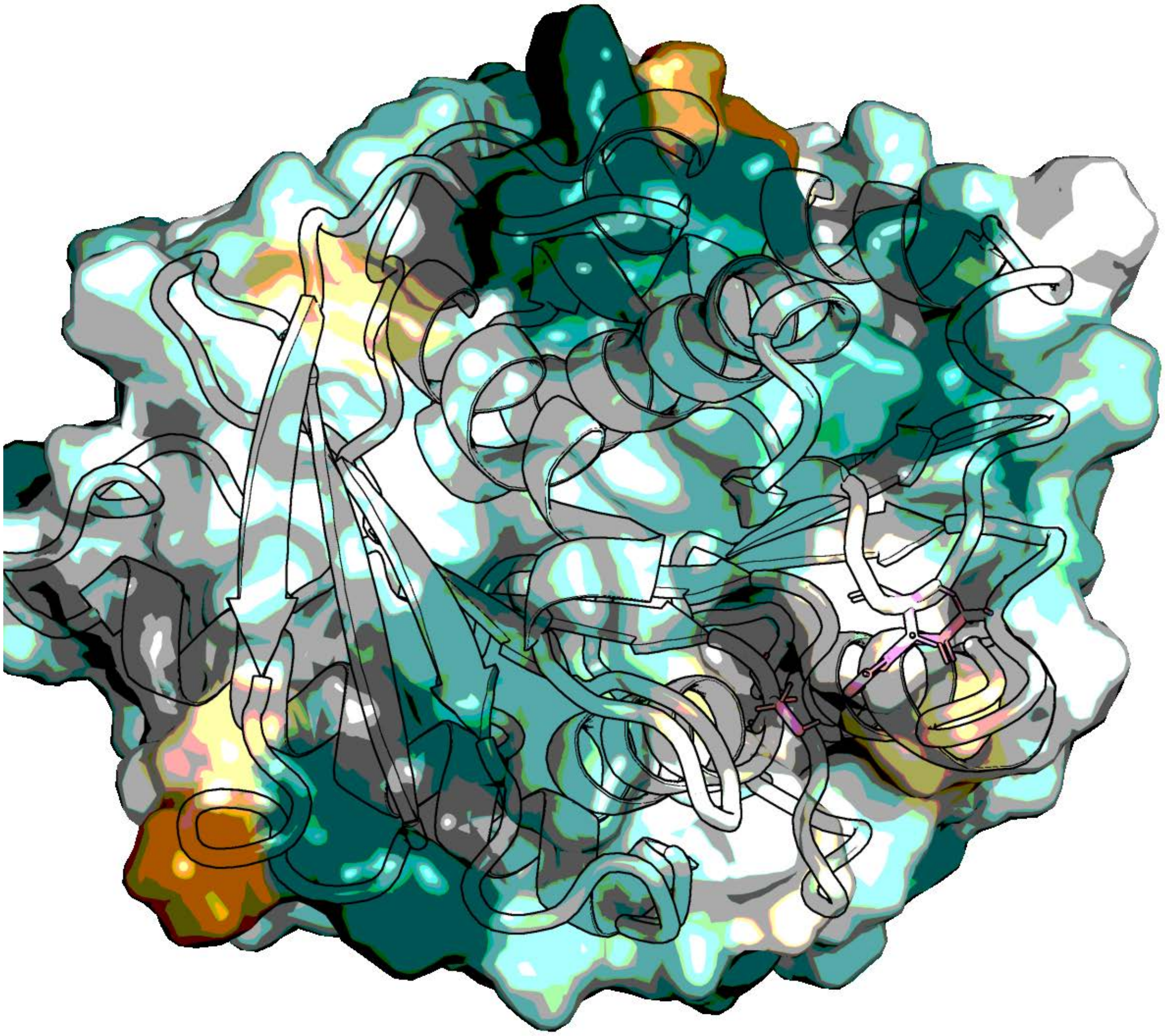

DP038_highlights.png

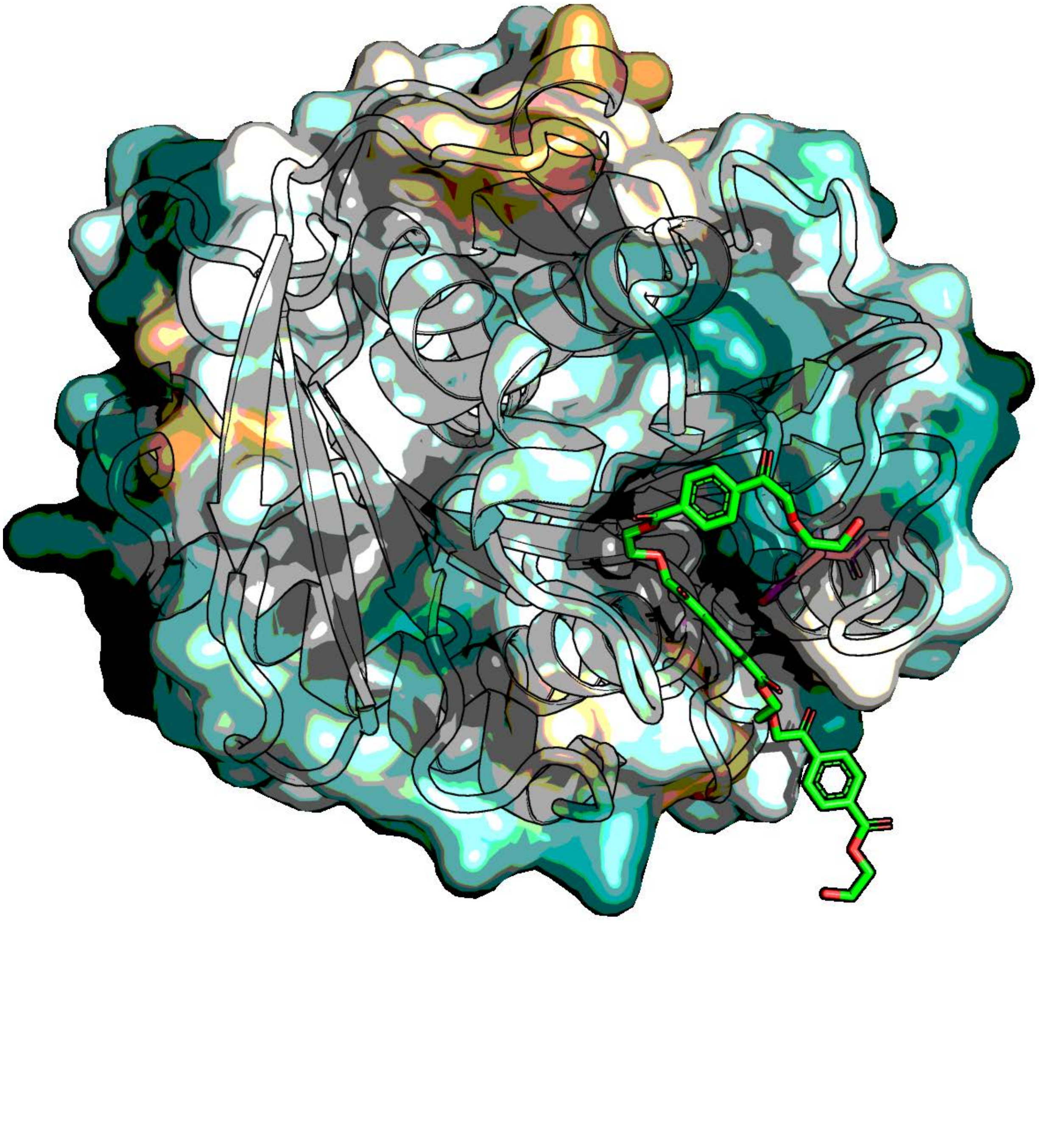

DP043_highlights.png

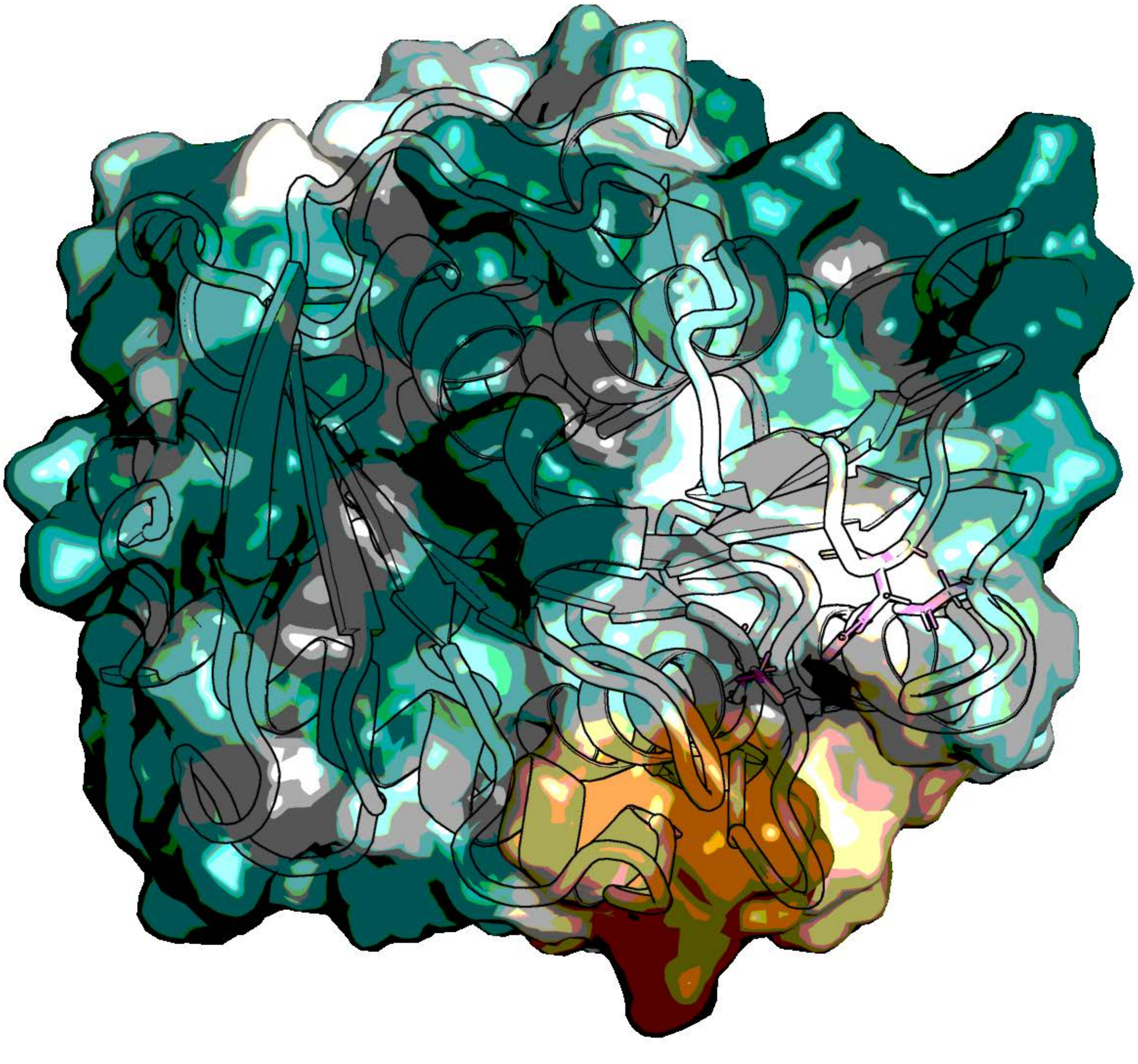

DP054_highlights.png

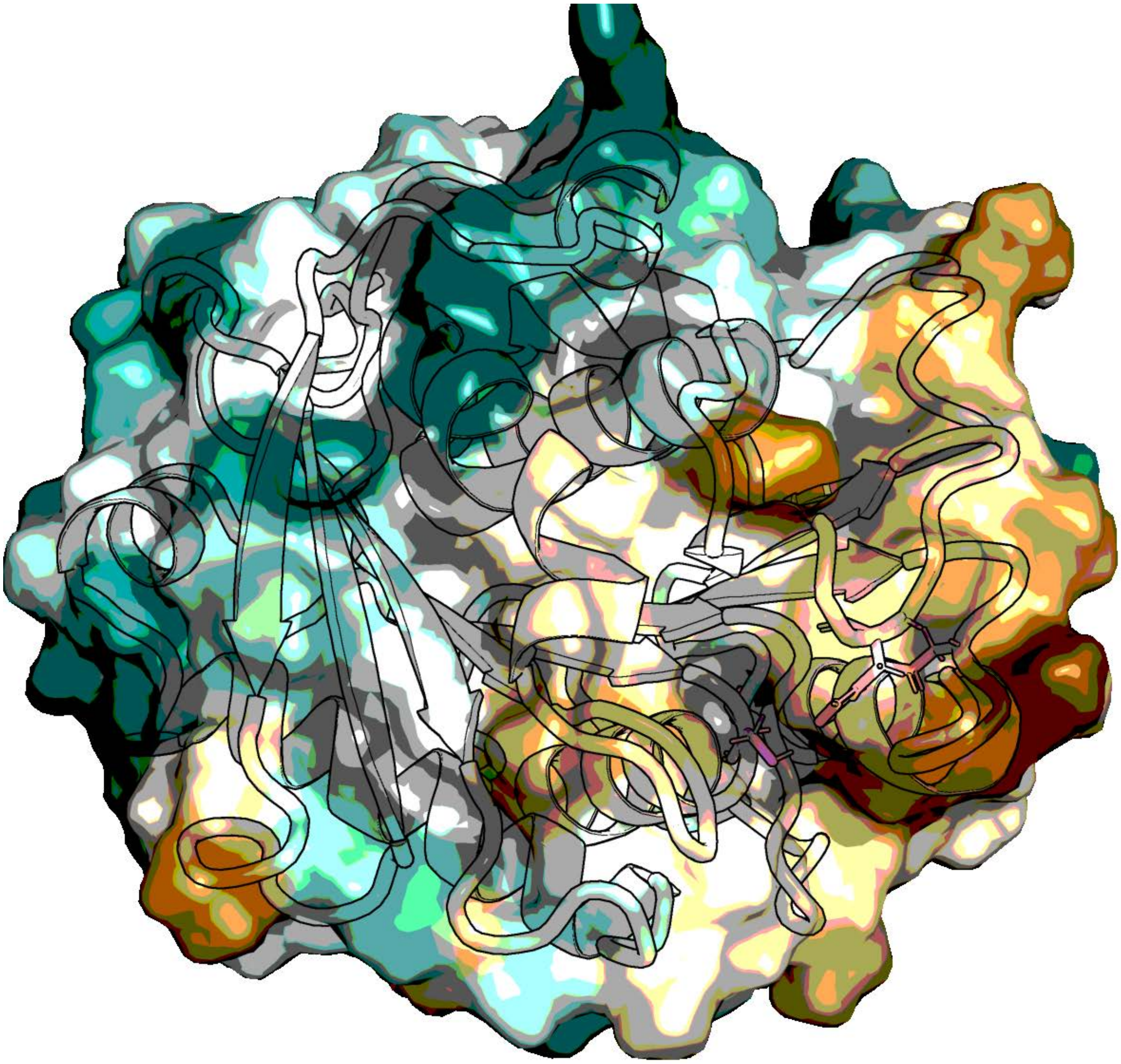

DP056_highlights.png

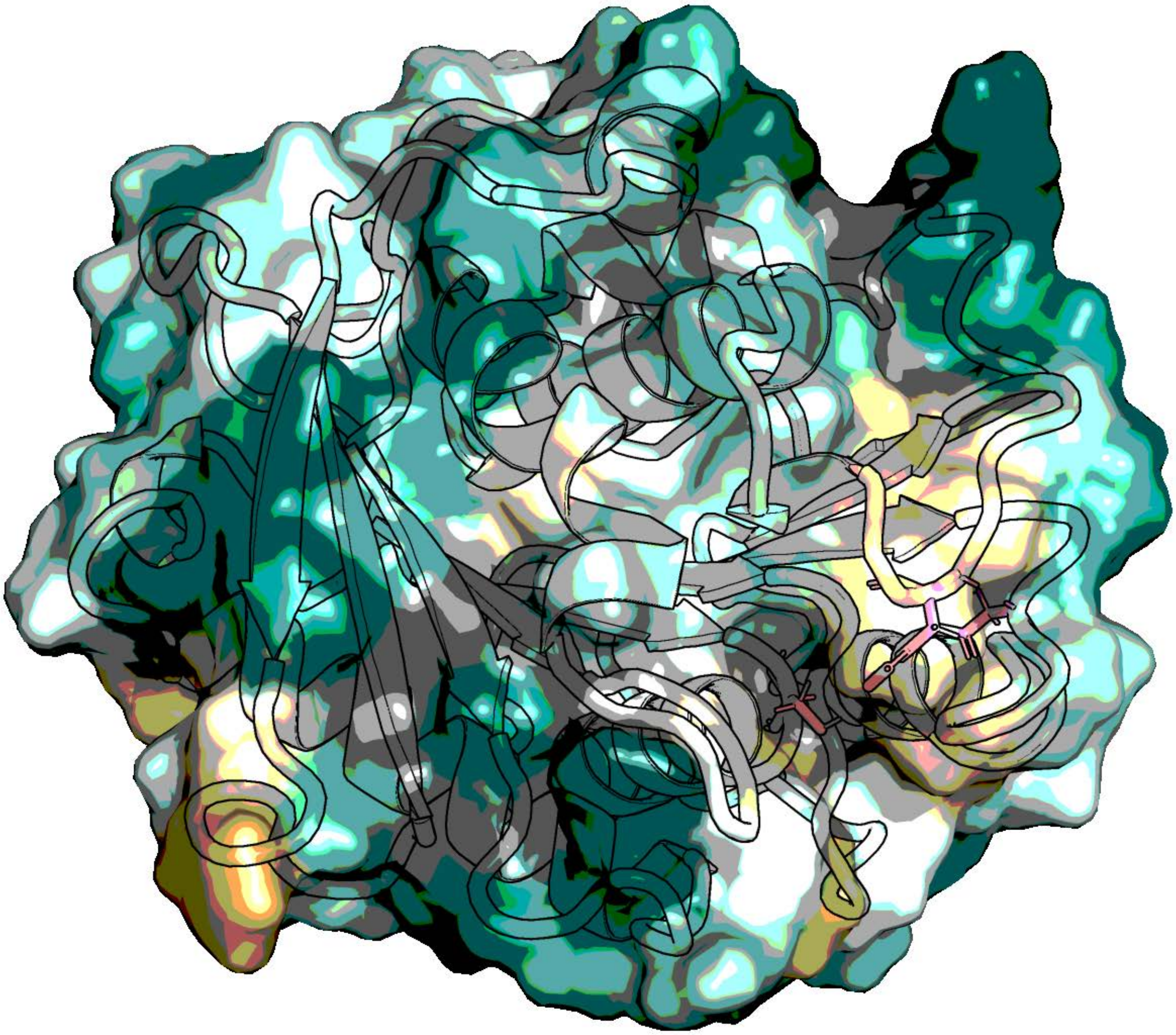

DP057_highlights.png

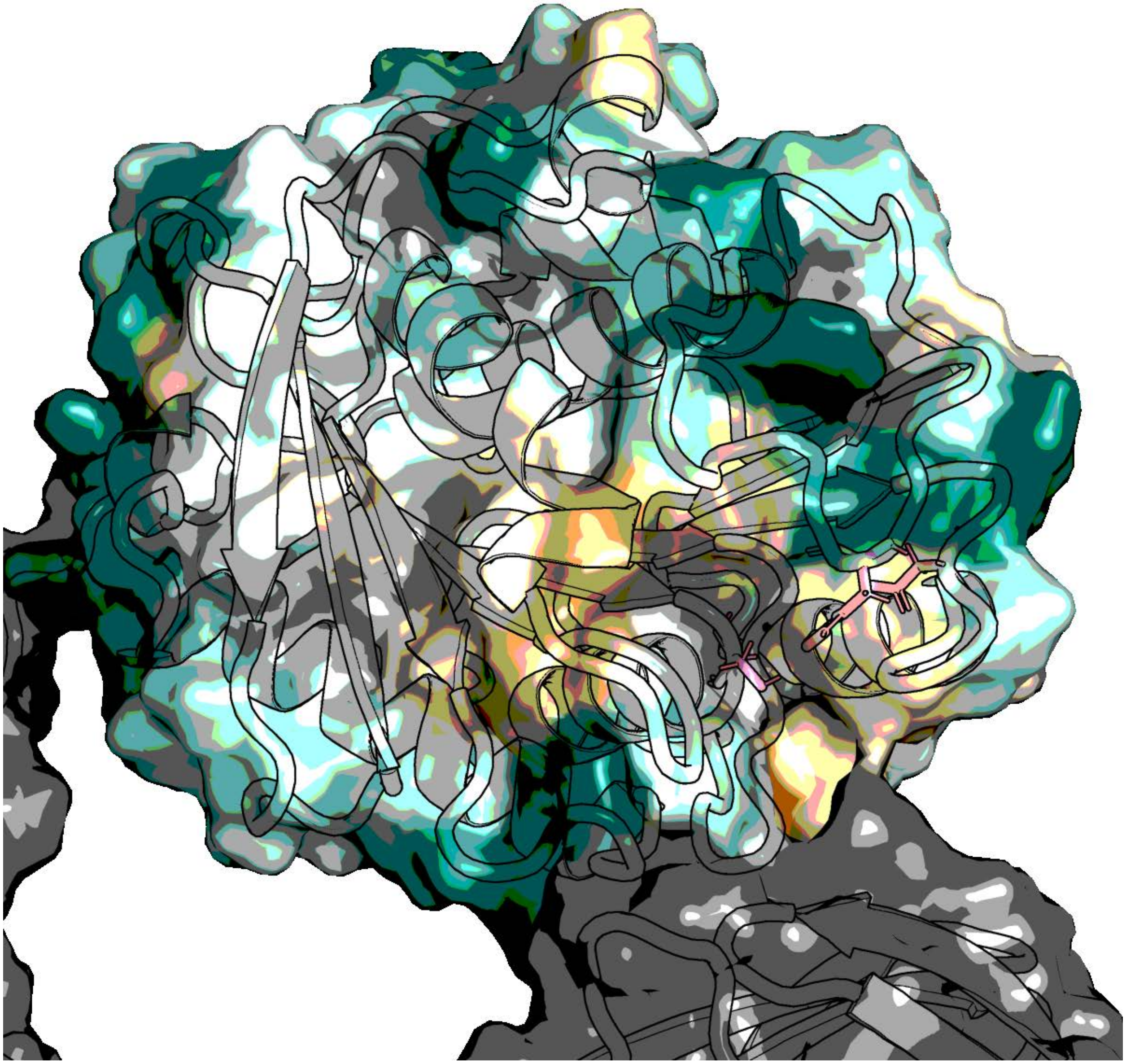

DP059_highlights.png

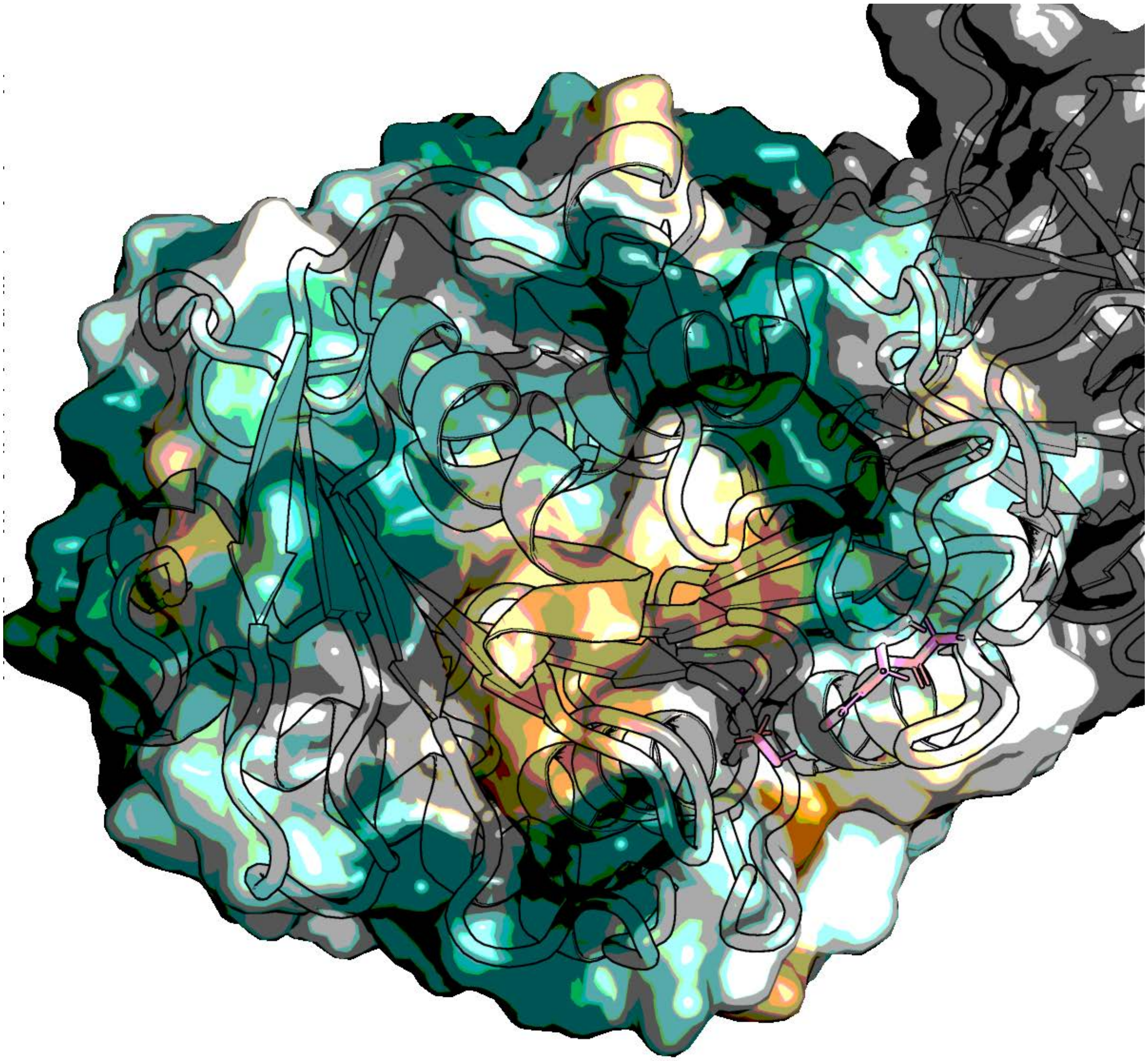

DP061_highlights.png

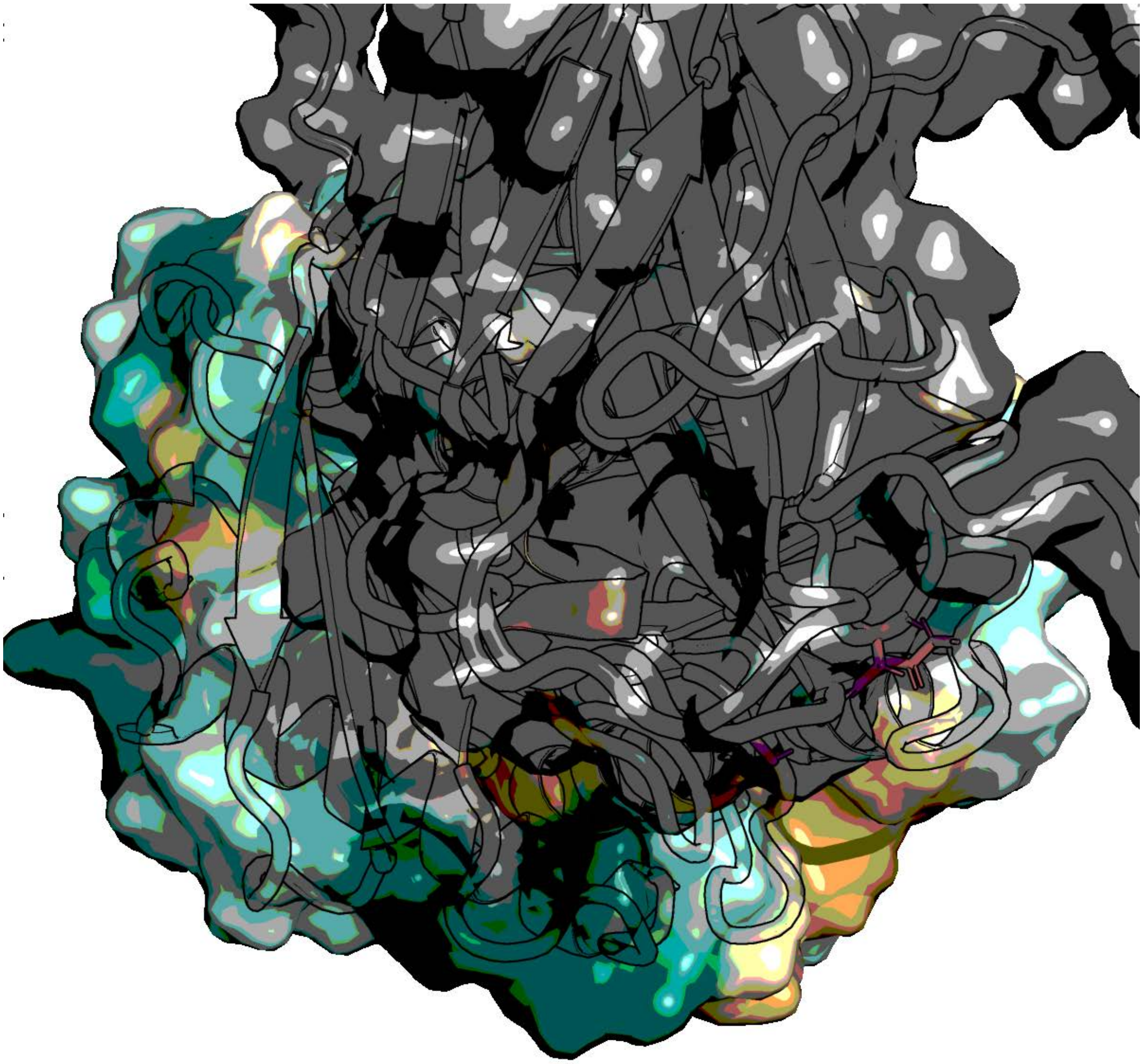

DP068_highlights.png

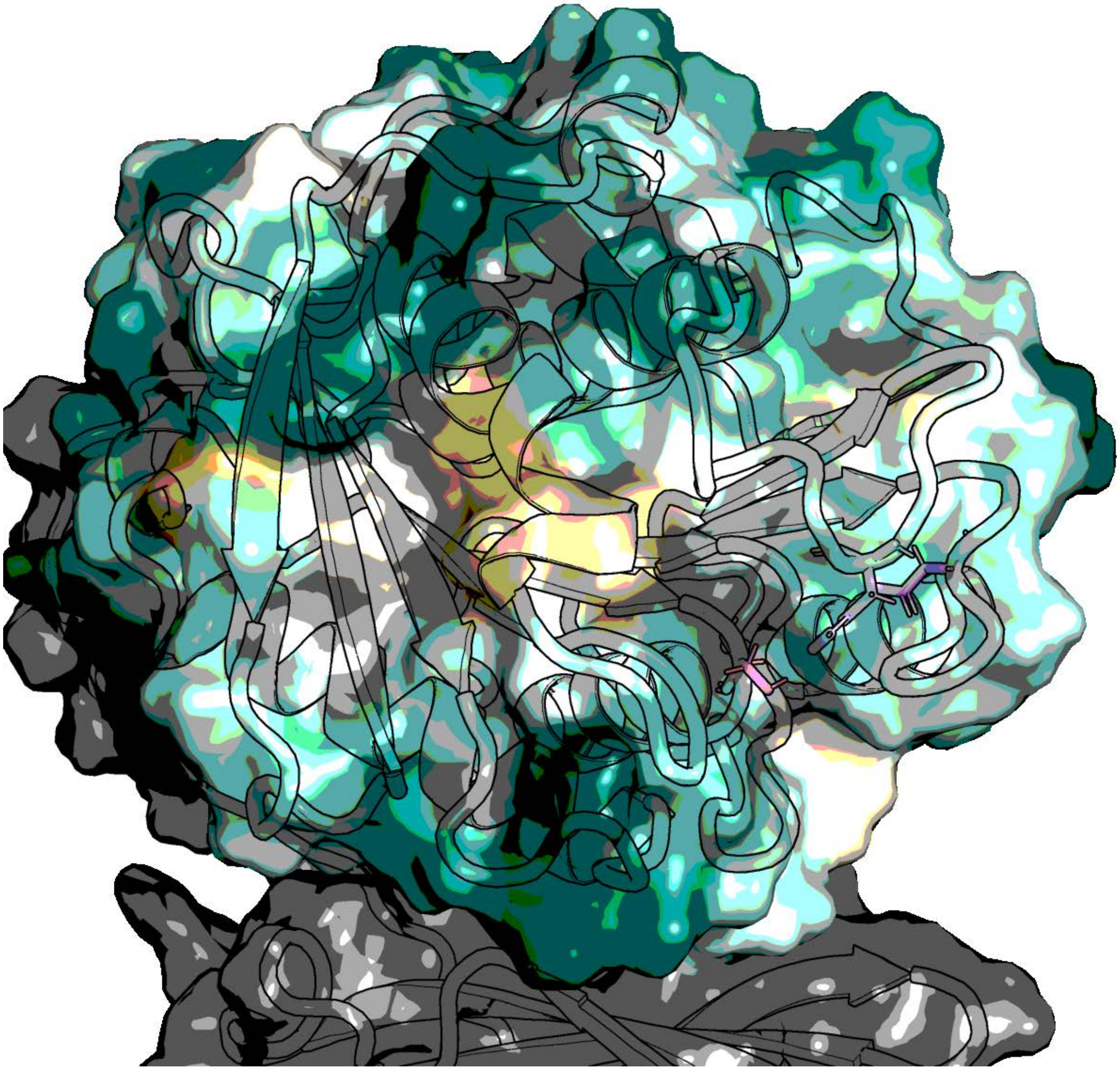

DP072_highlights.png

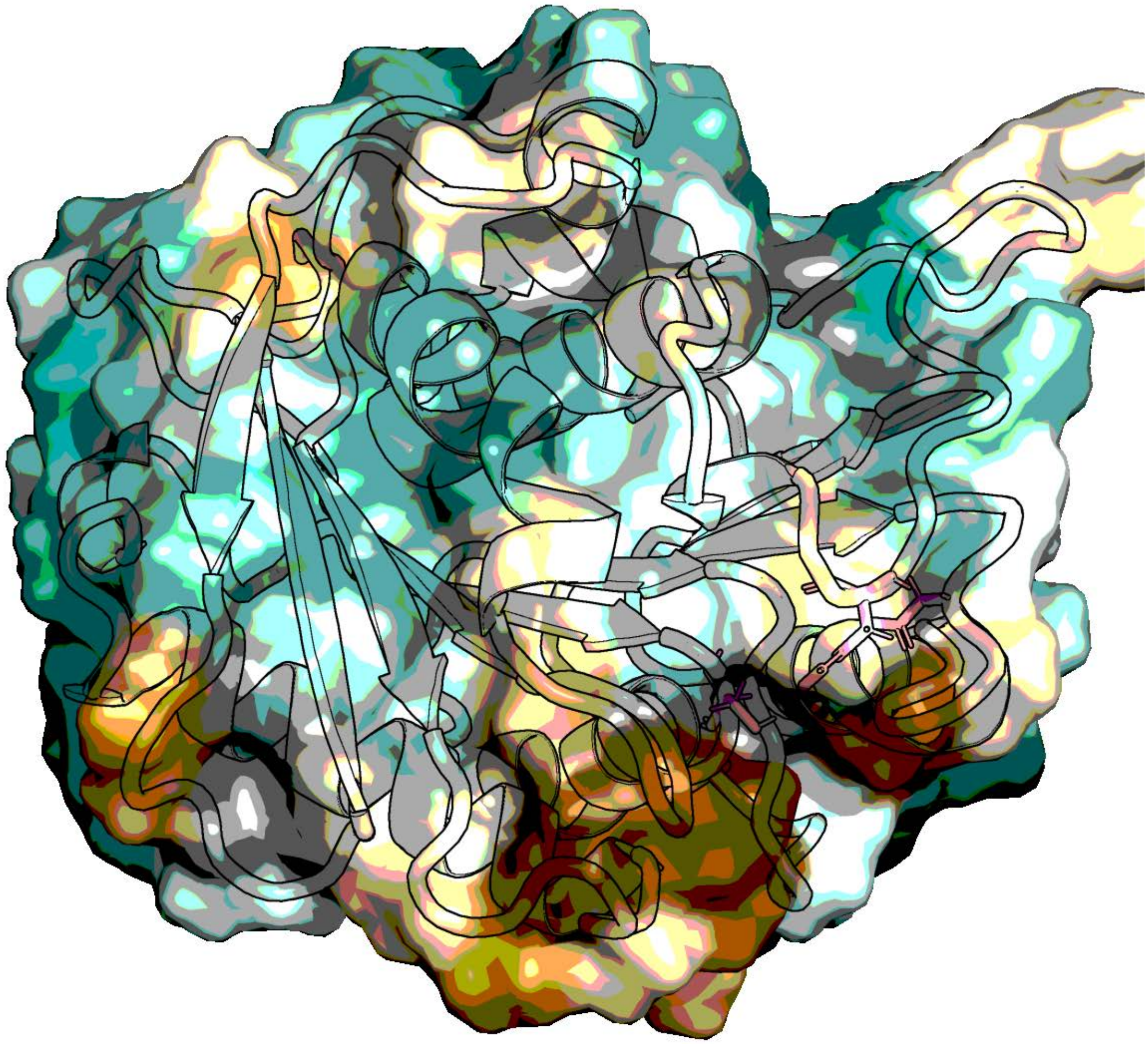

DP077_highlights.png

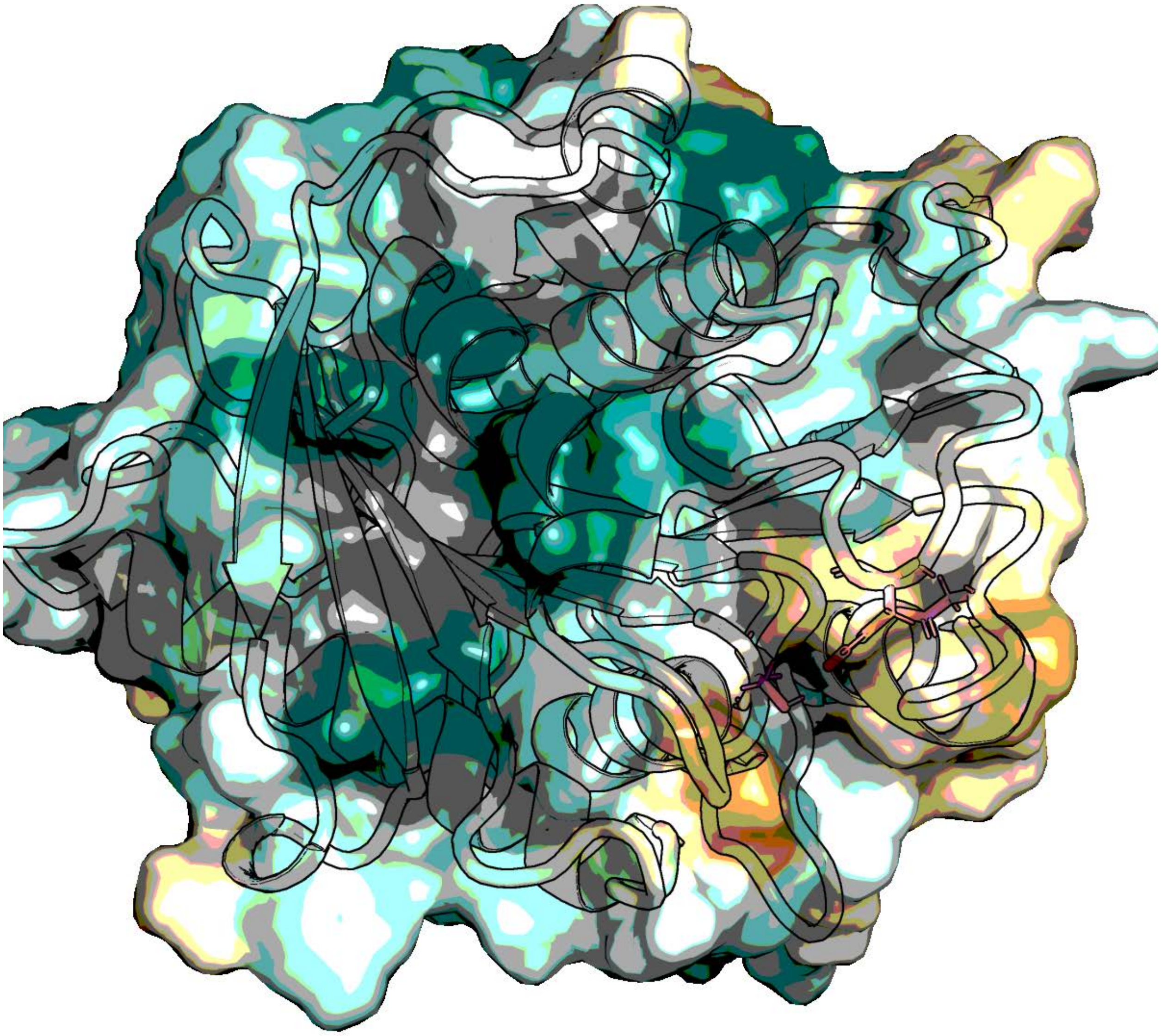

DP078_highlights.png

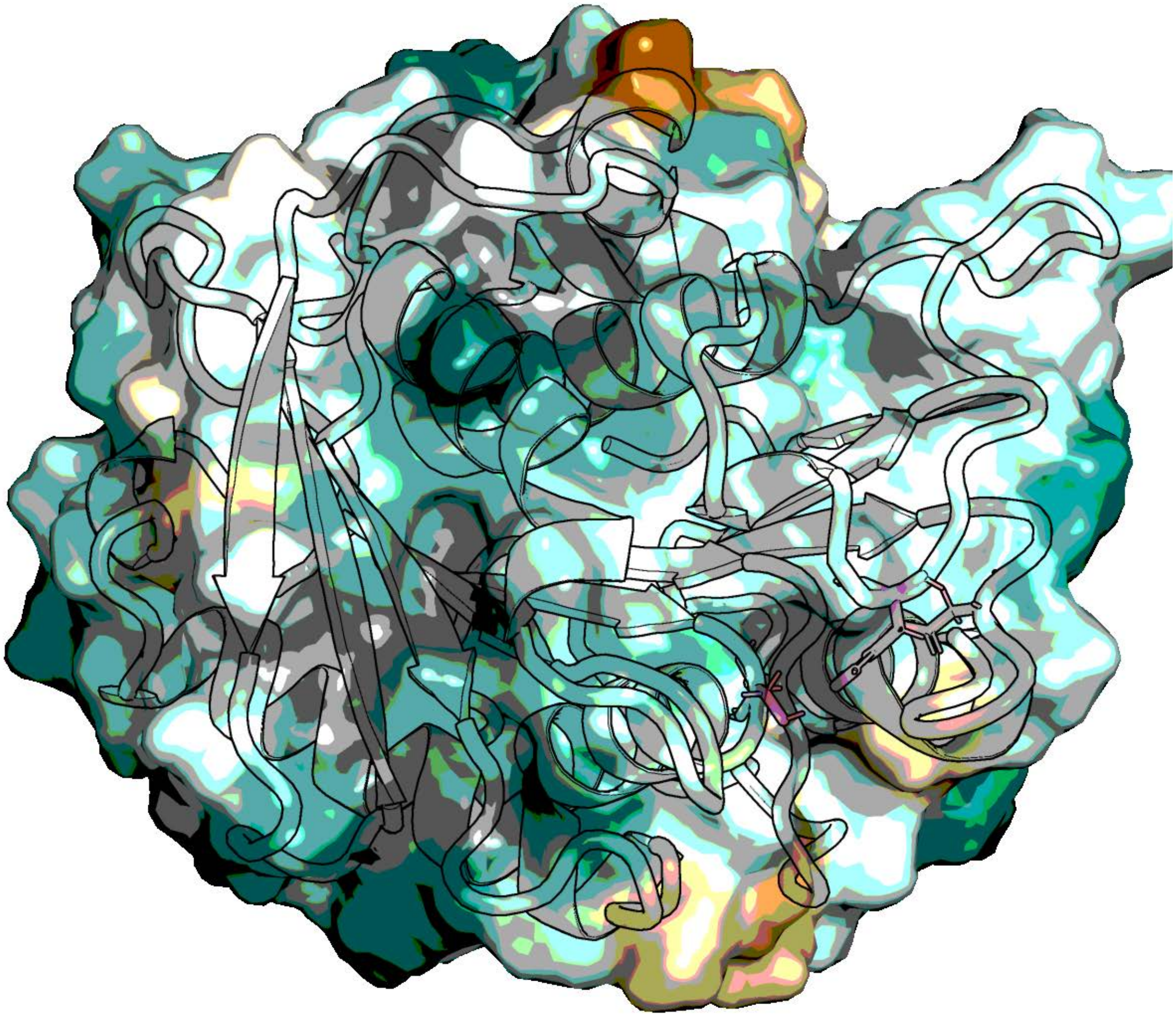

DP080_highlights.png

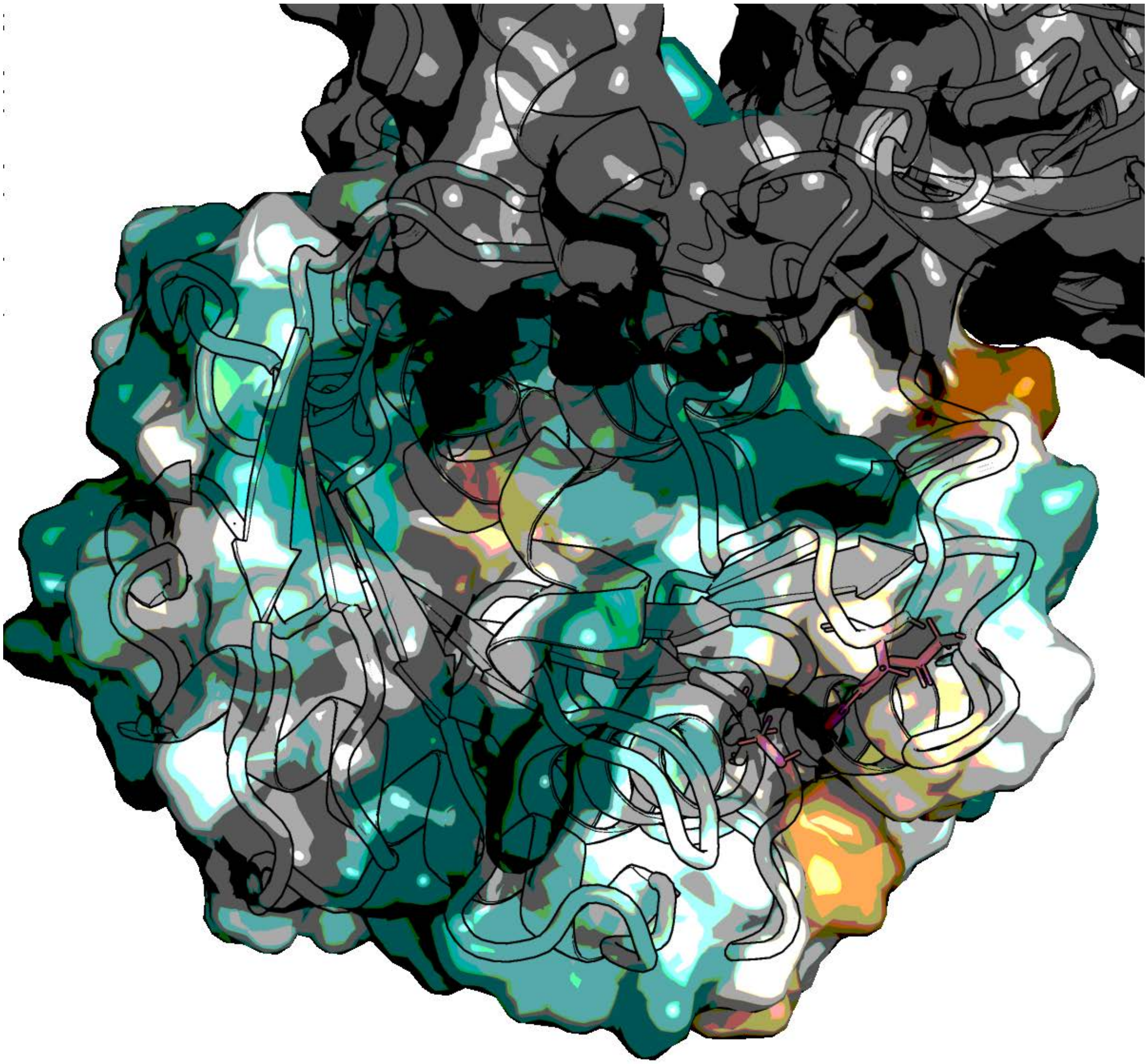

DP081_highlights.png

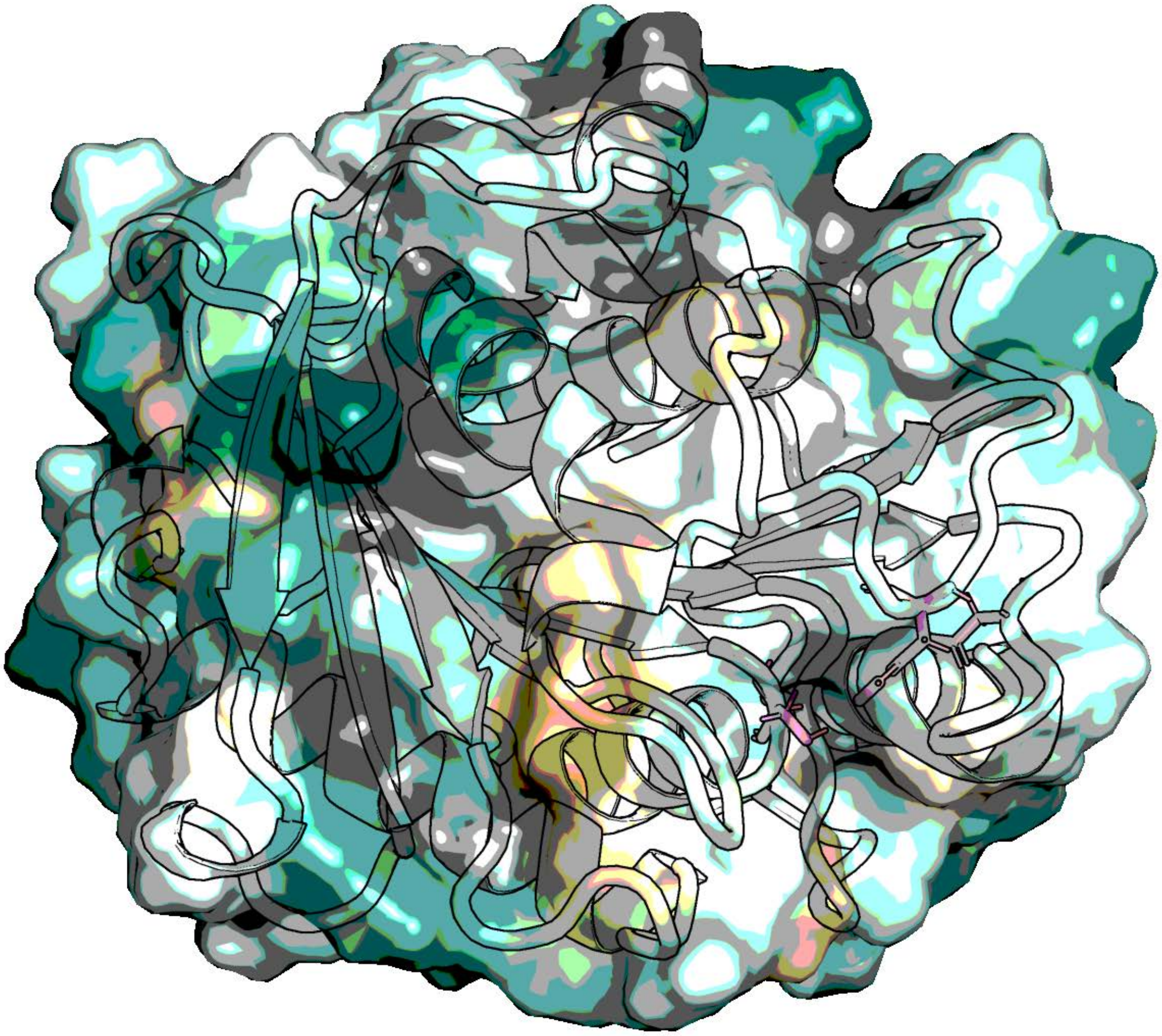

DP082_highlights.png

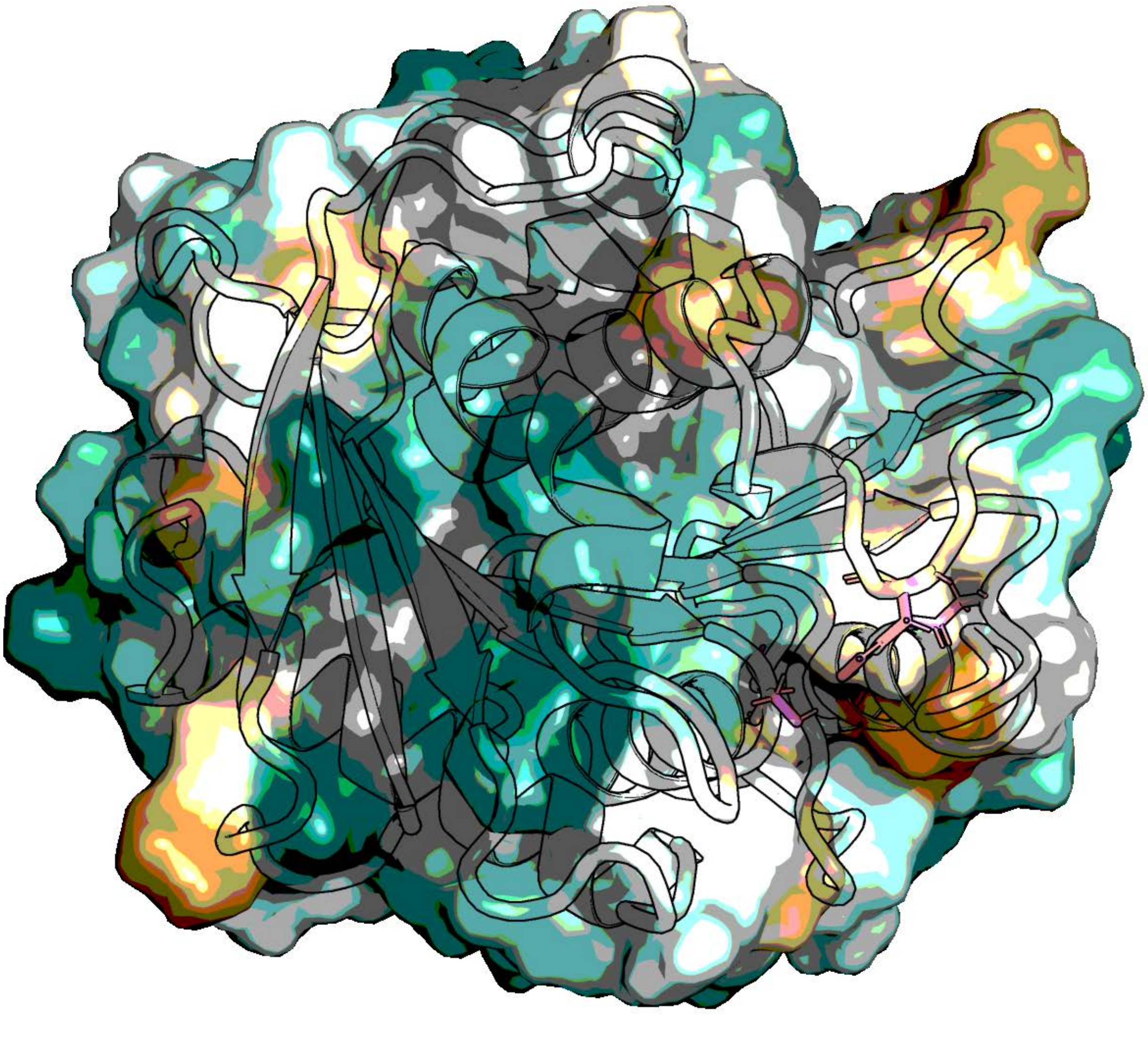

DP084_highlights.png

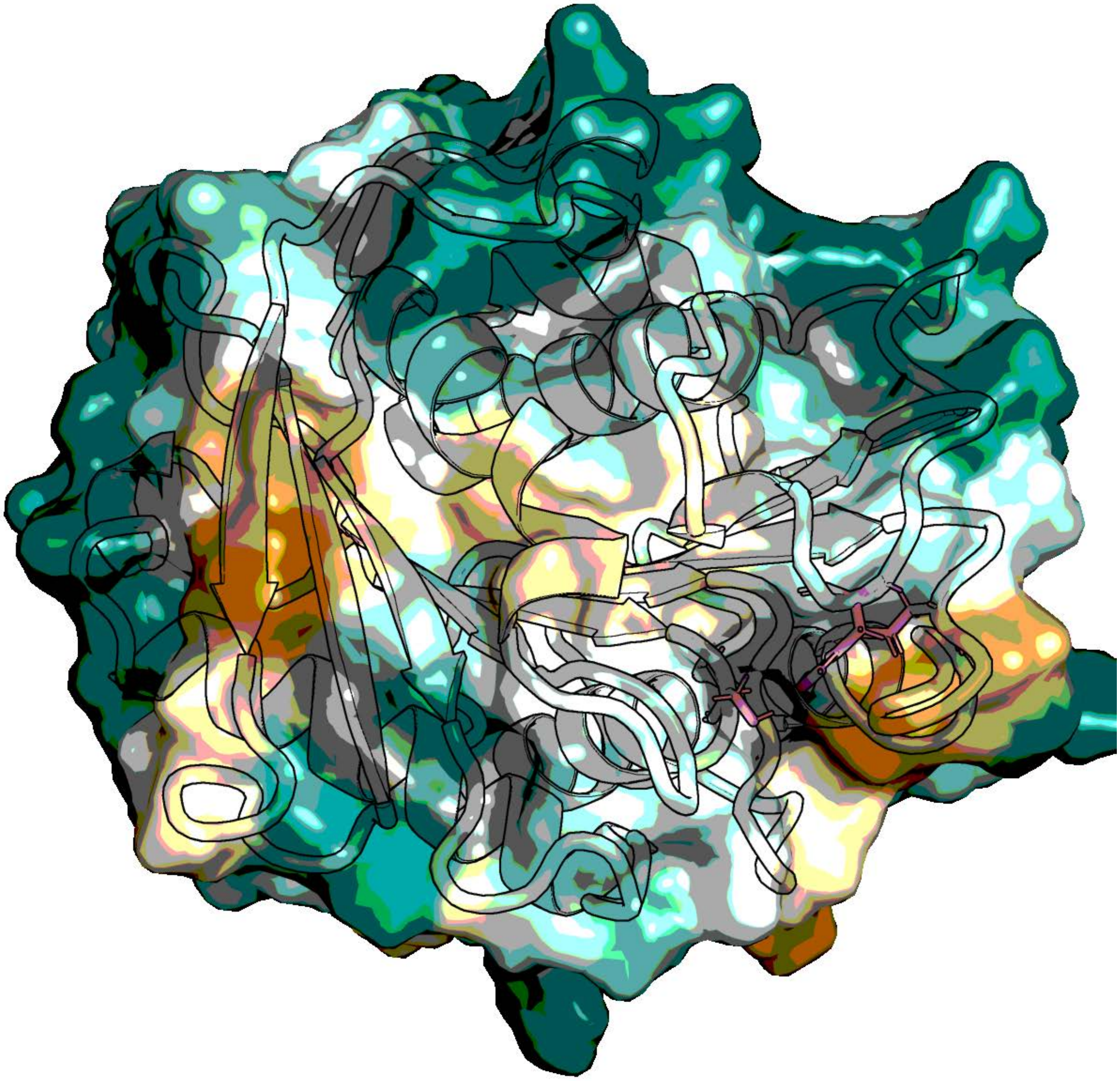

DP089_highlights.png

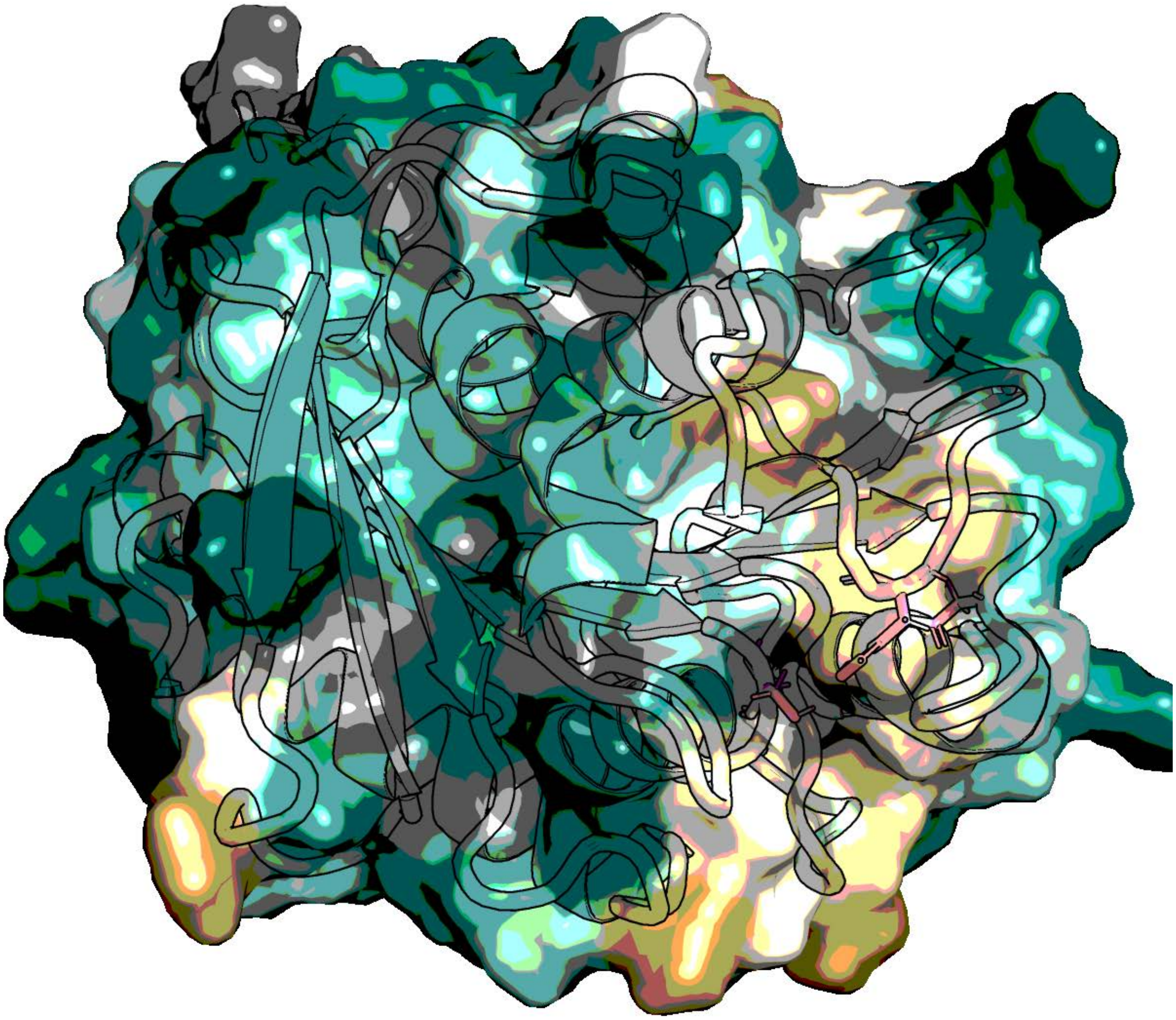

DP090_highlights.png

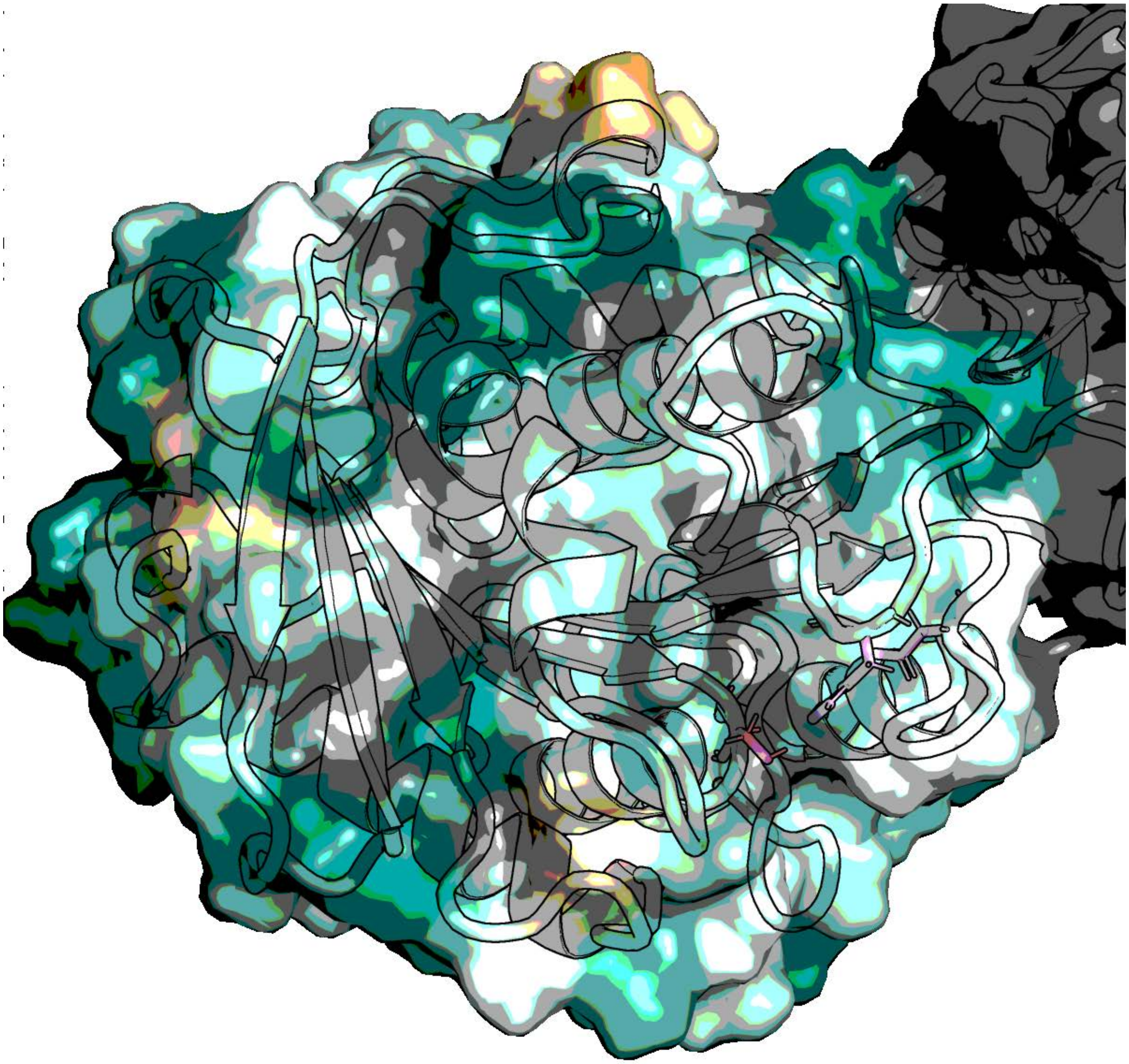

DP092_highlights.png

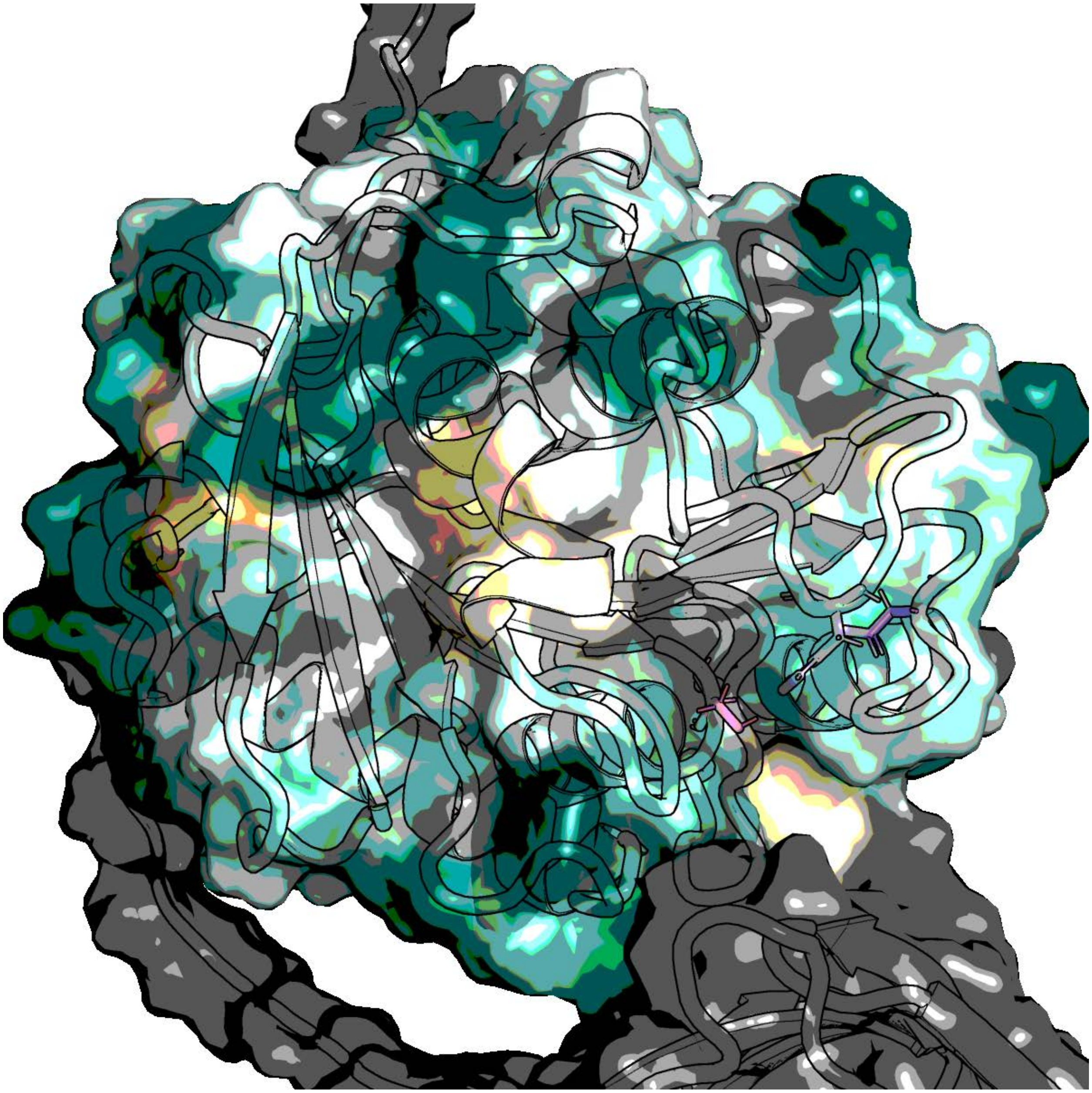

ESM006_highlights.png

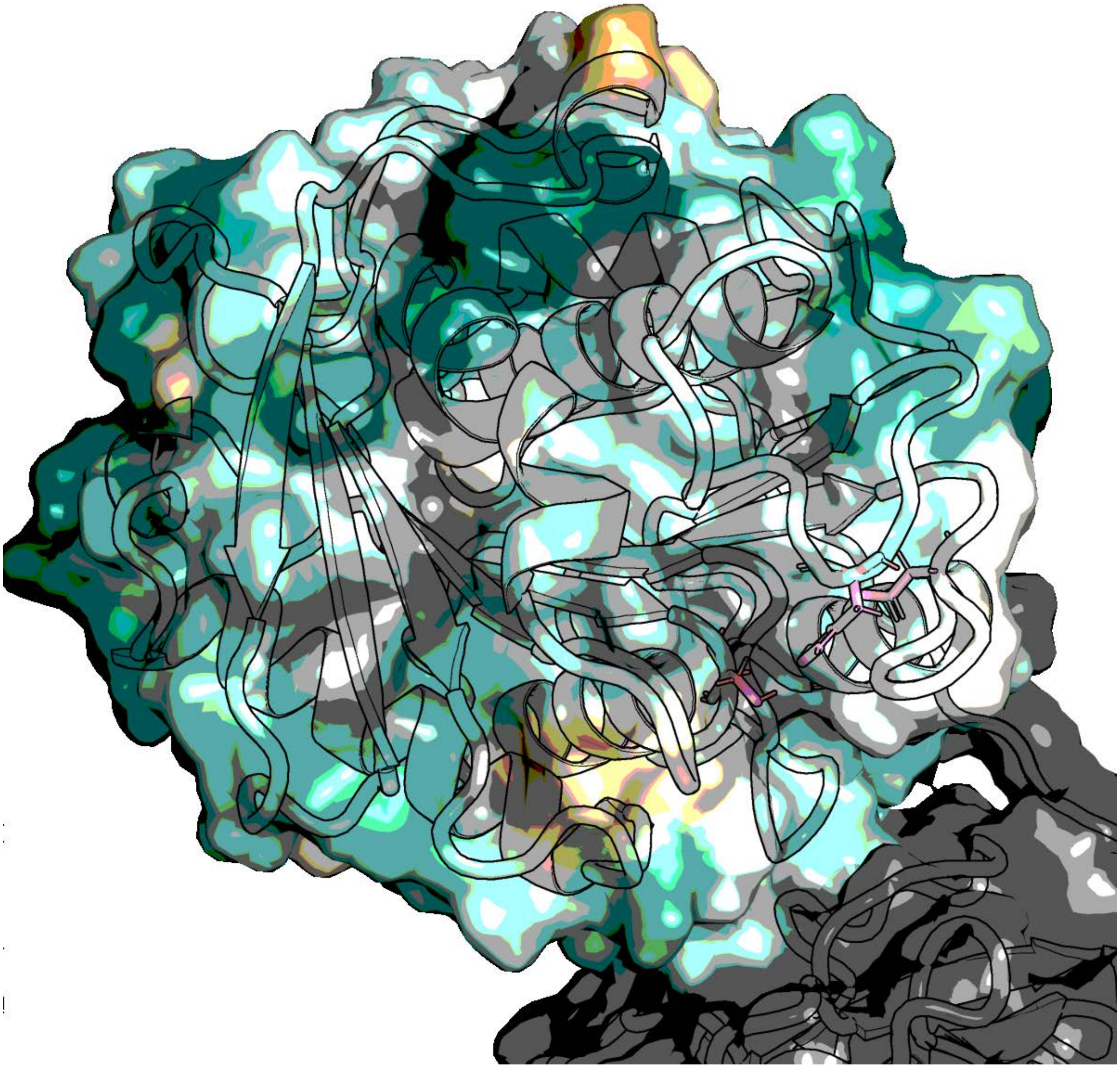

ESM010_highlights.png

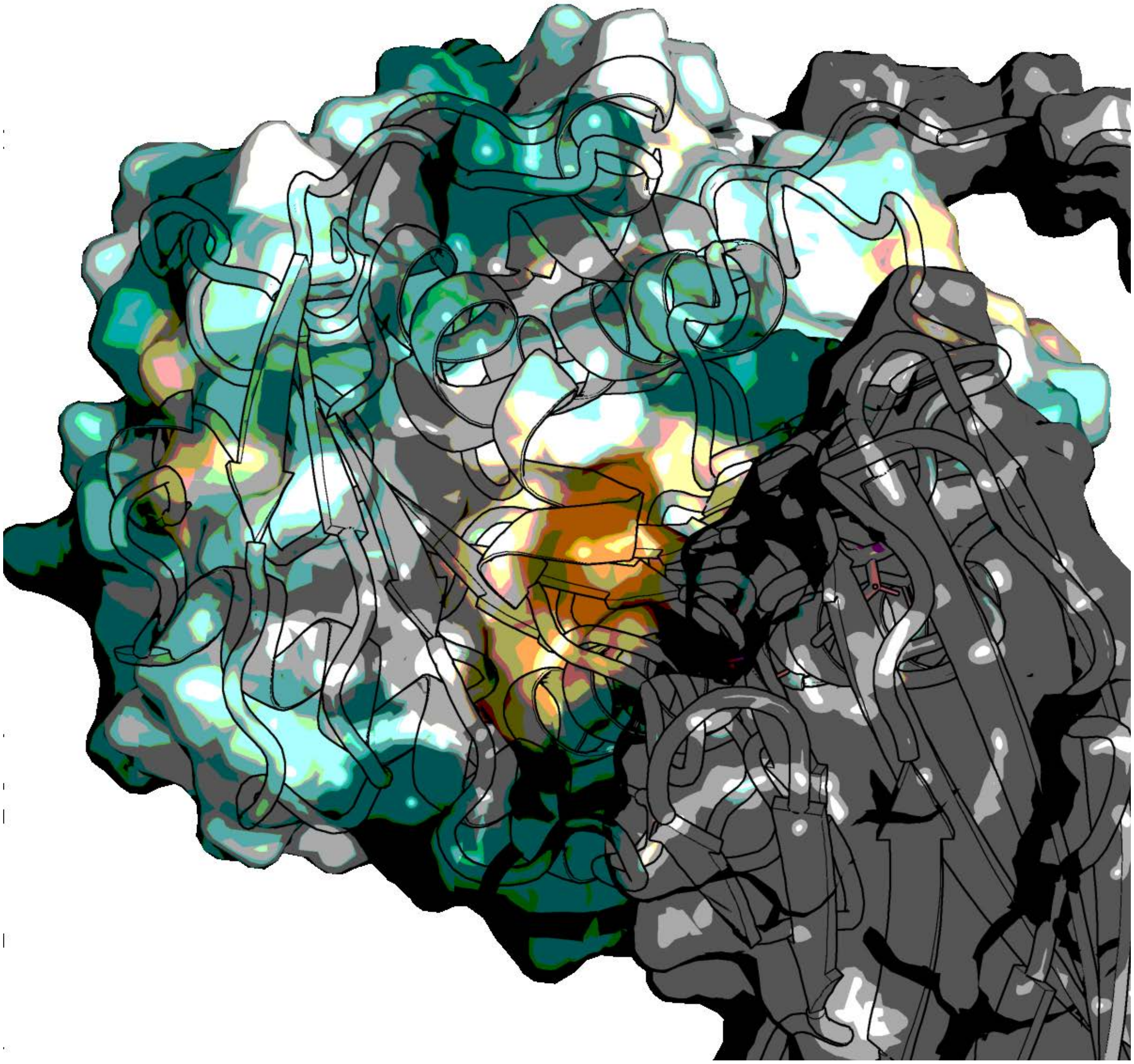

ESM012_highlights.png

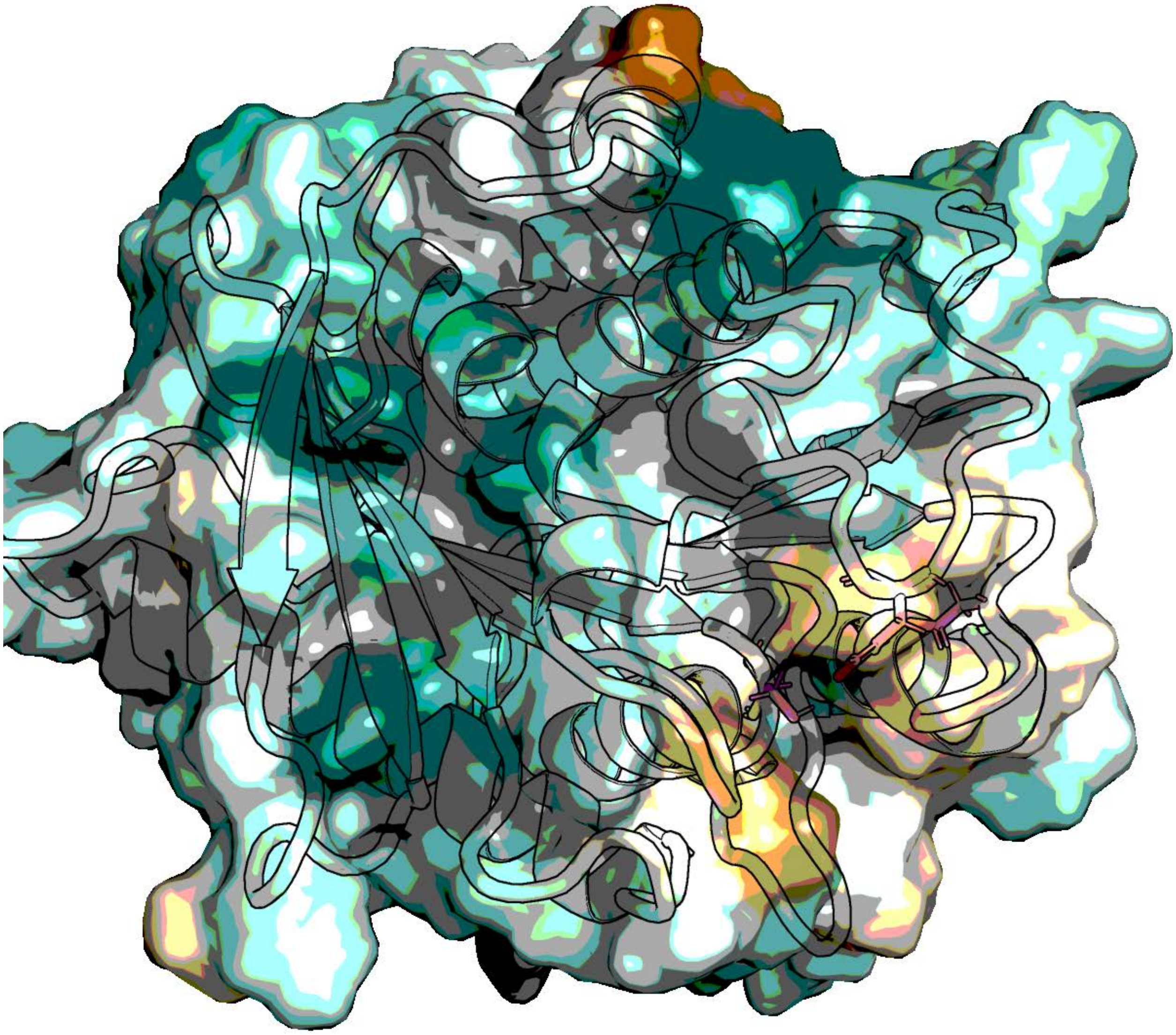

ESM017_highlights.png

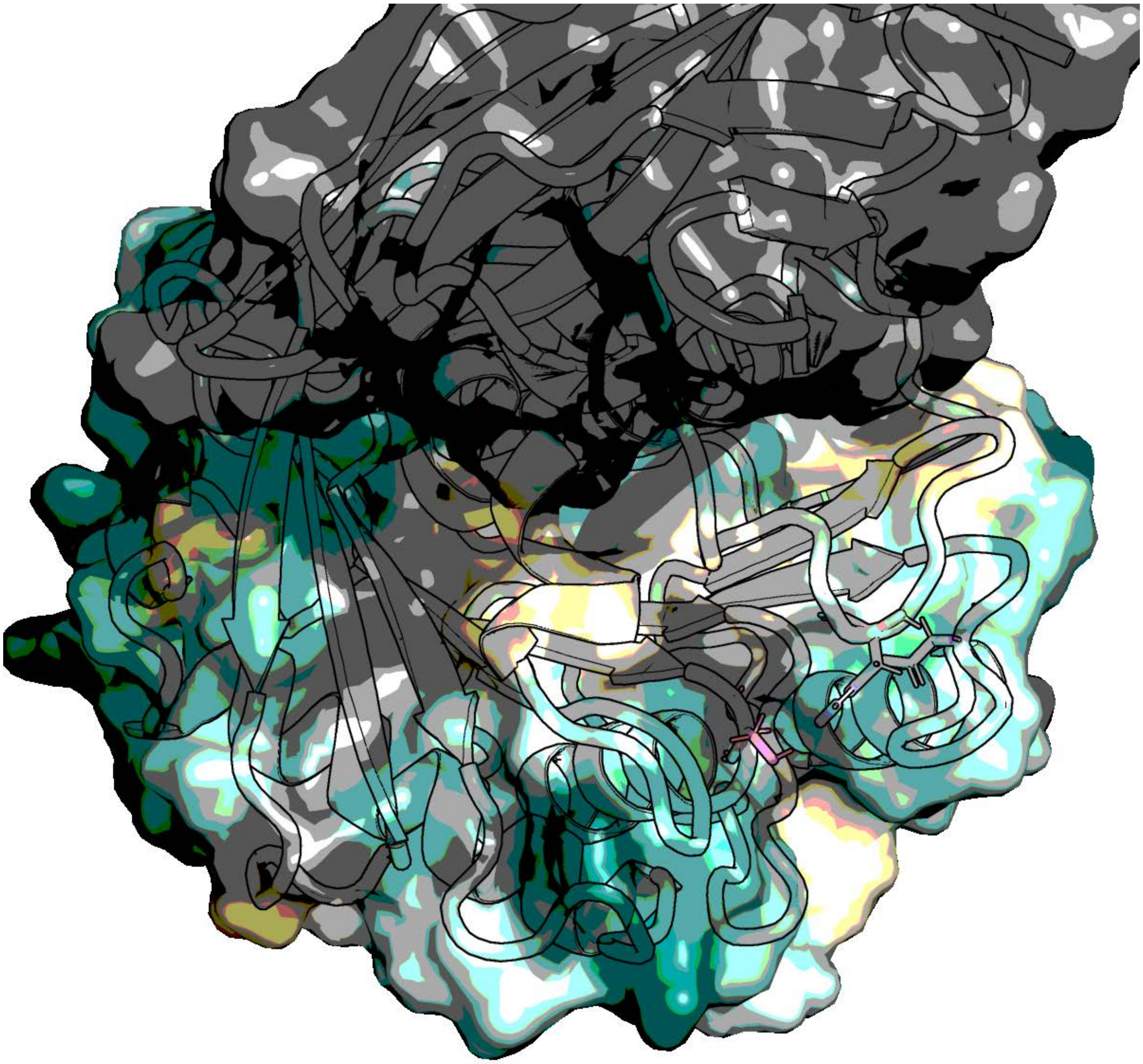

ESM018_highlights.png

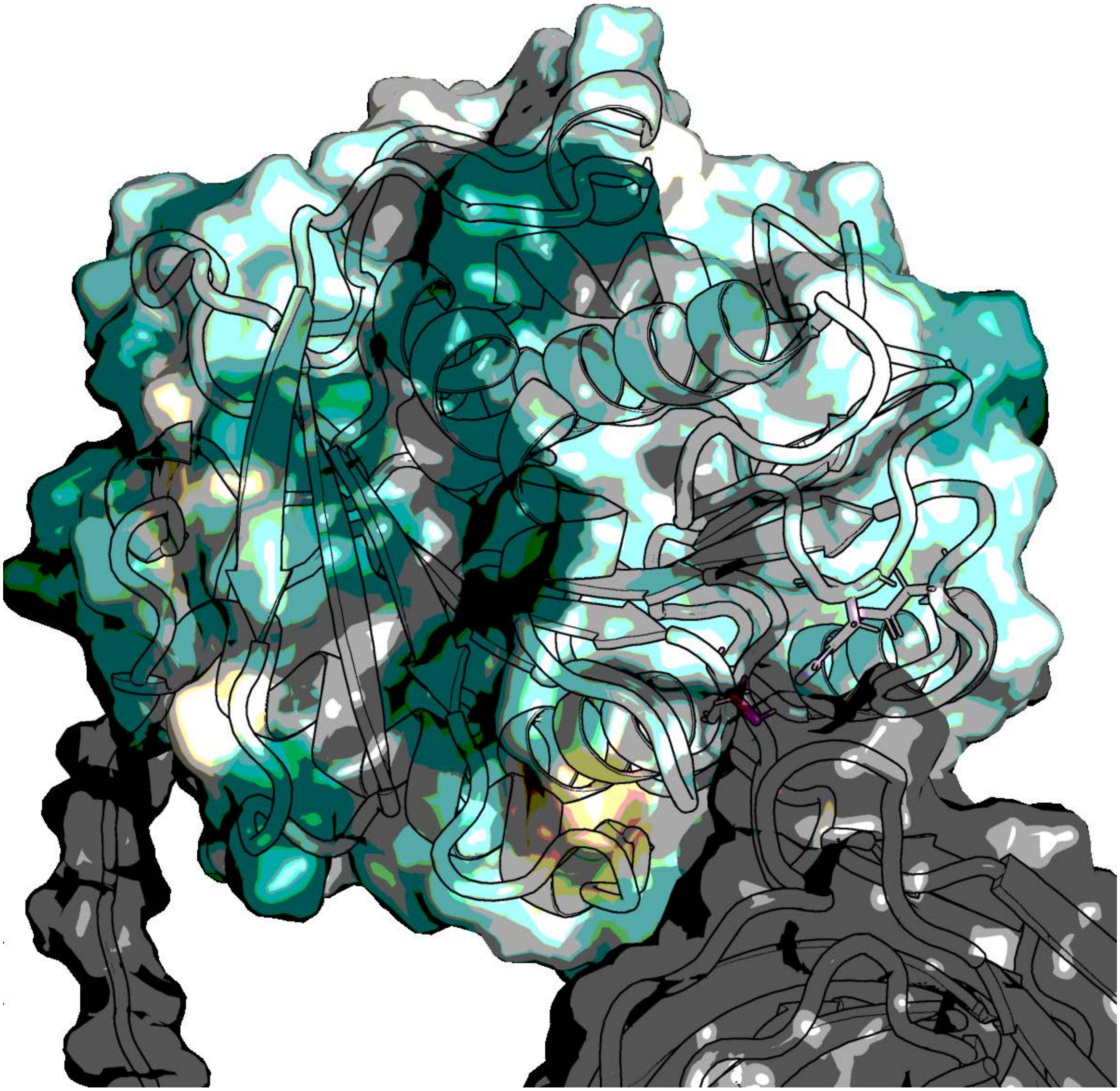

ESM021_highlights.png

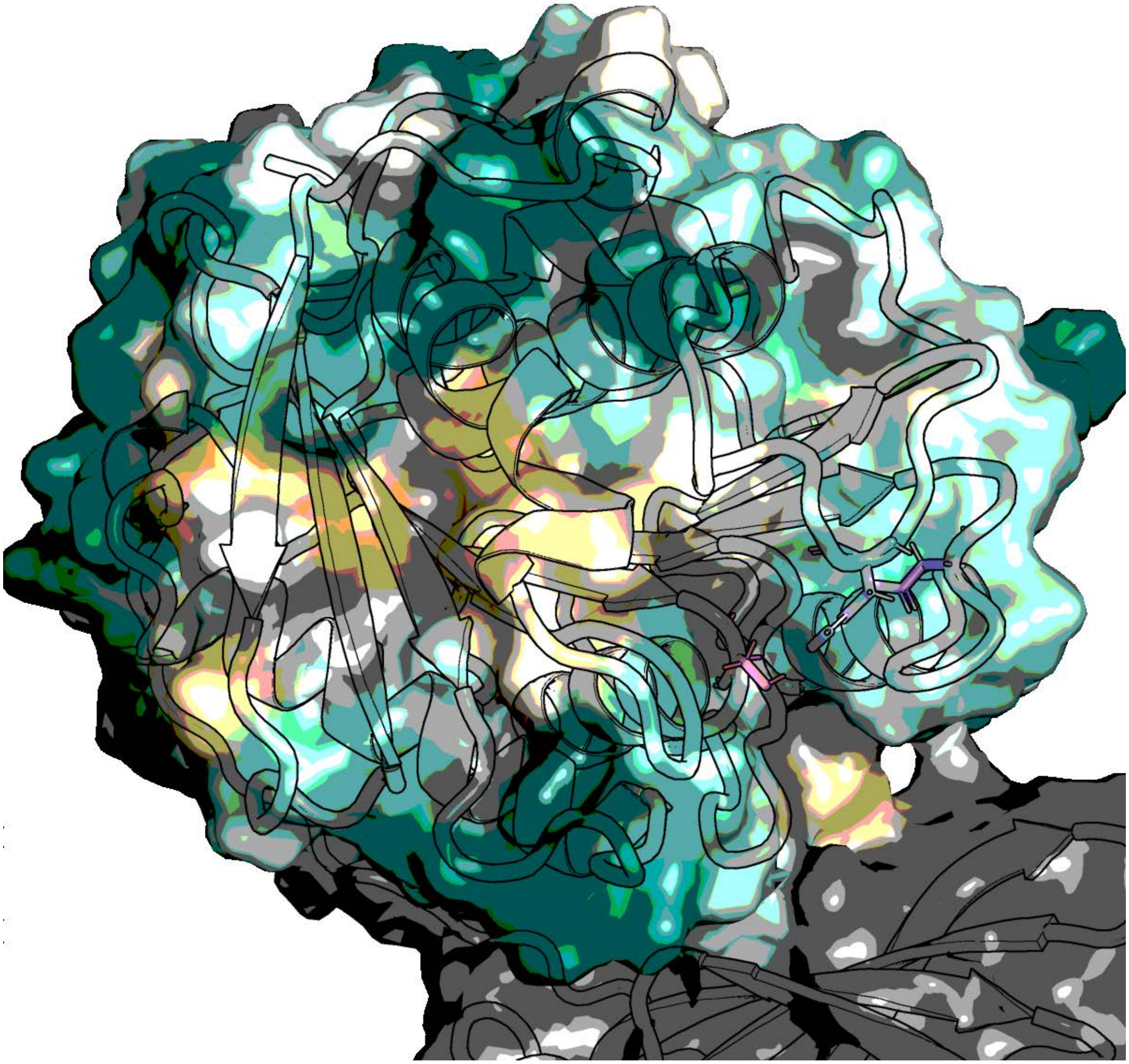

ESM023_highlights.png

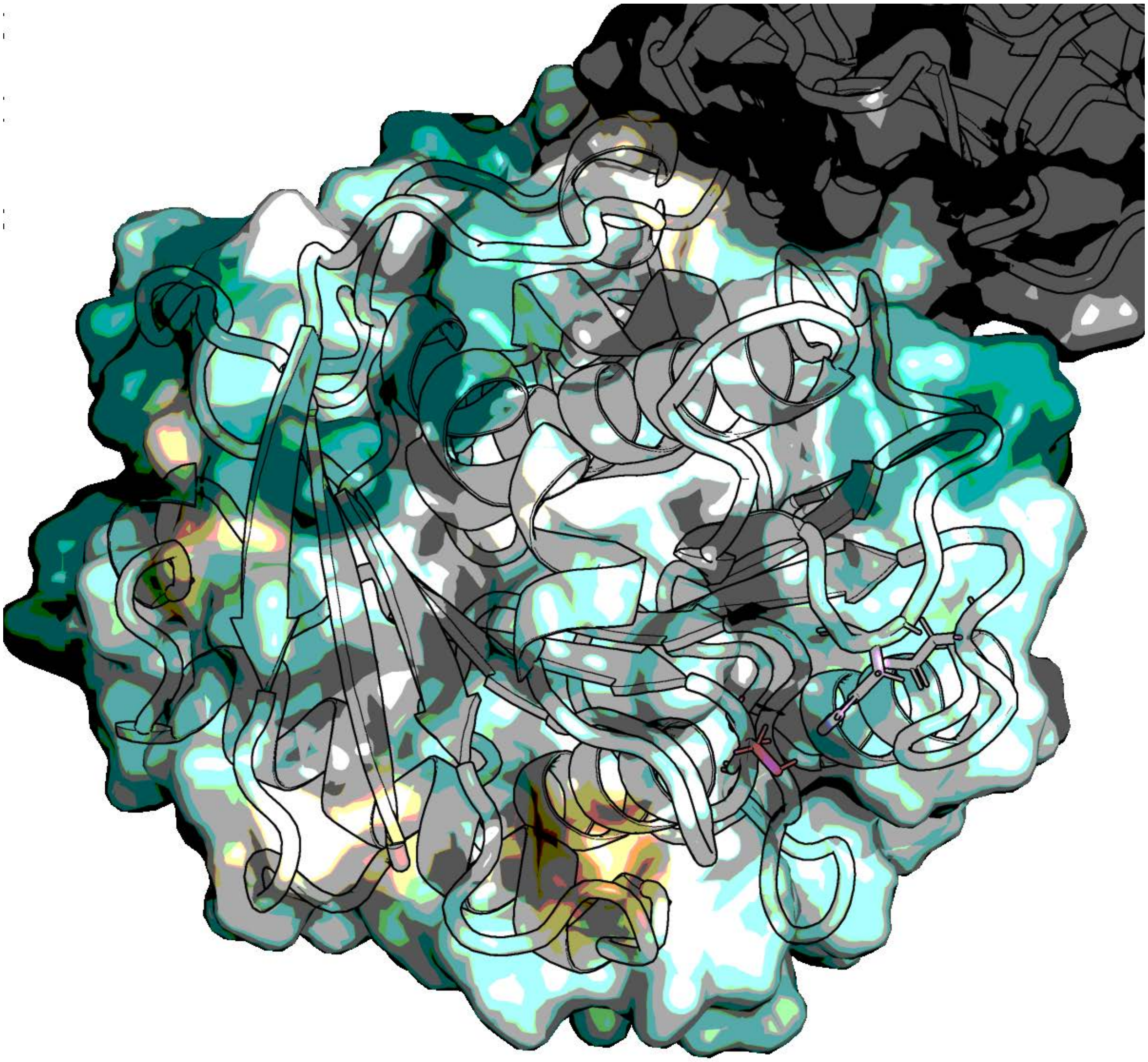

ESM026_highlights.png

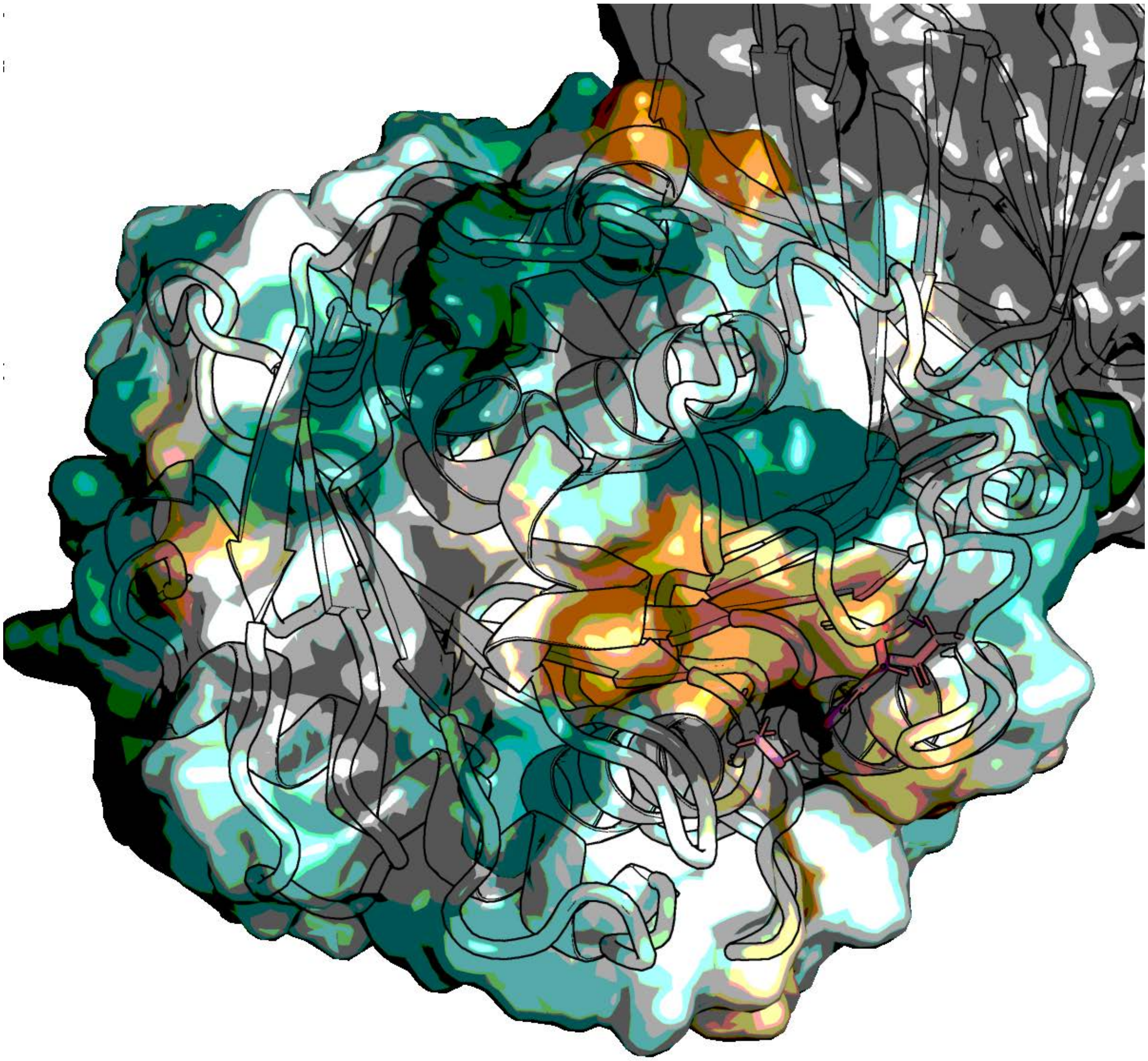

ESM033_highlights.png

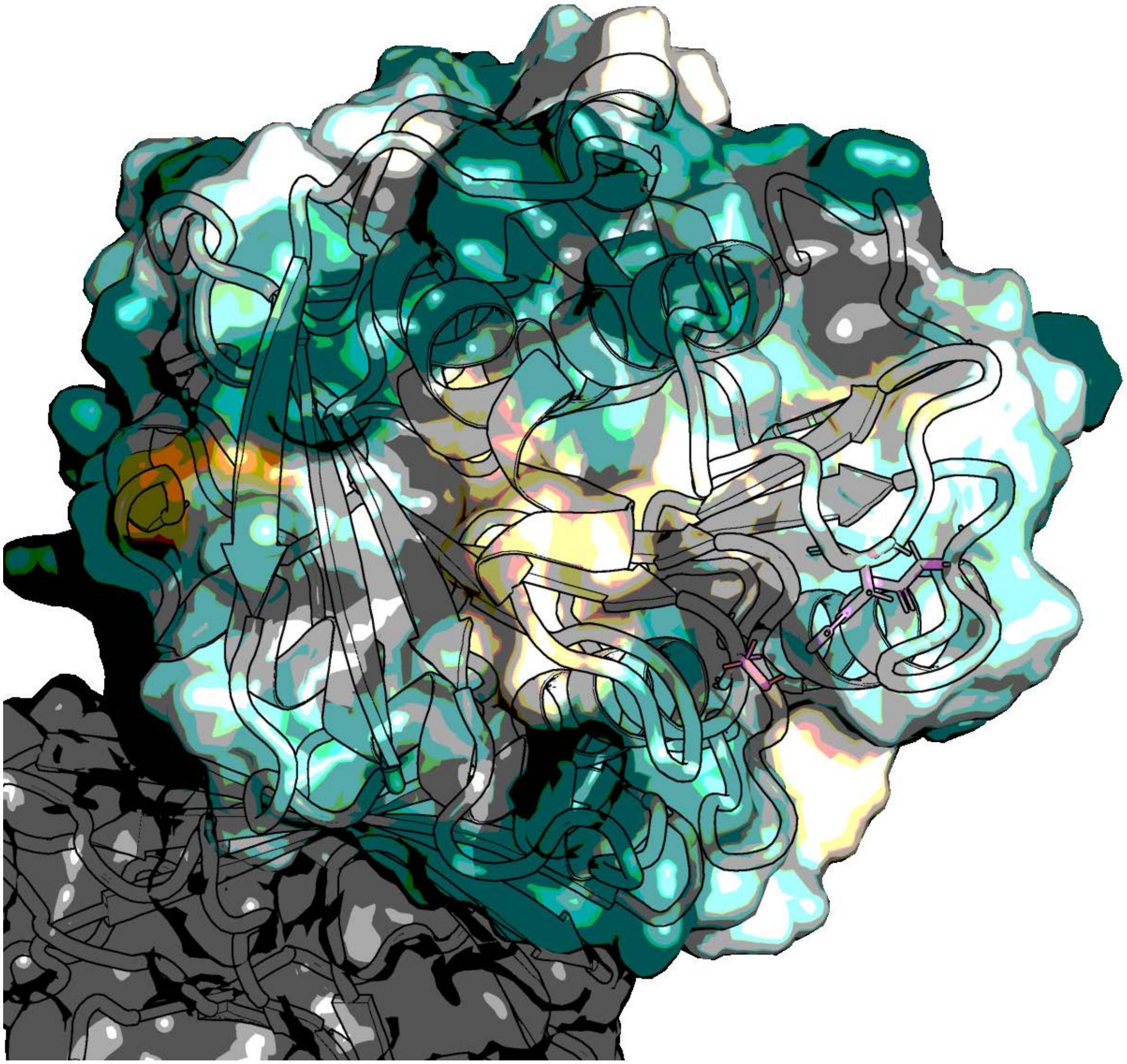

ESM035_highlights.png

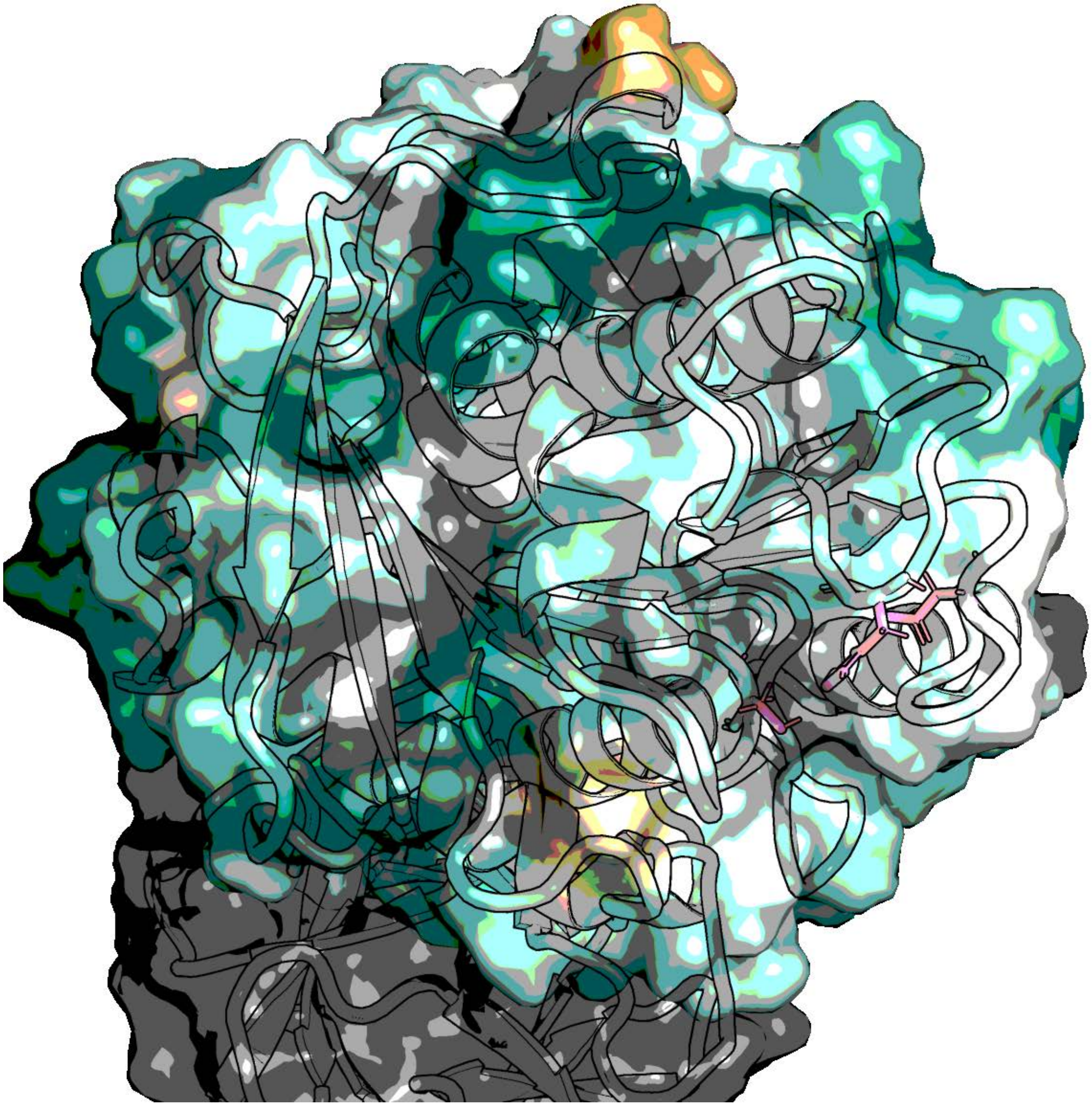

ESM038_highlights.png

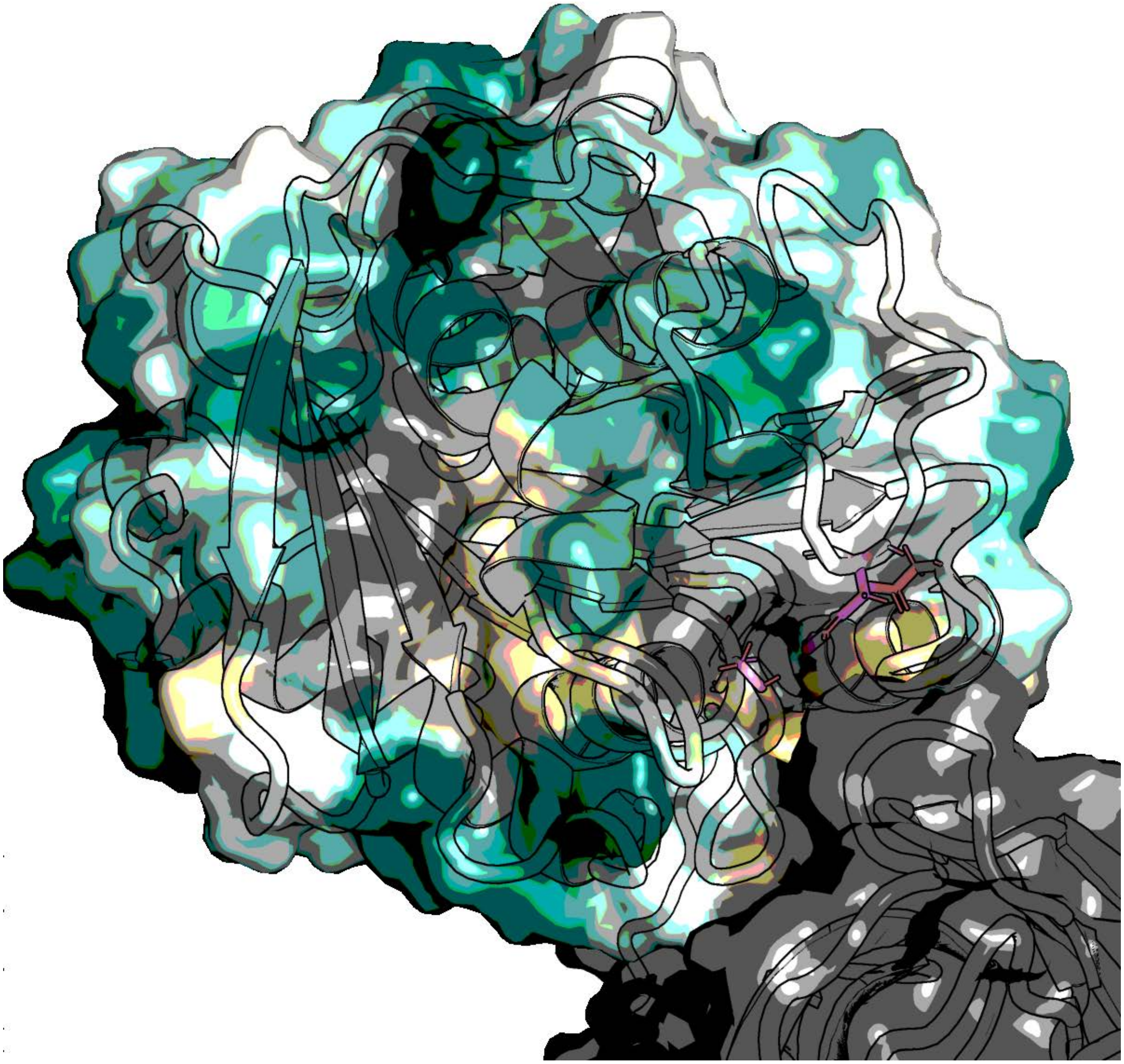

ESM043_highlights.png

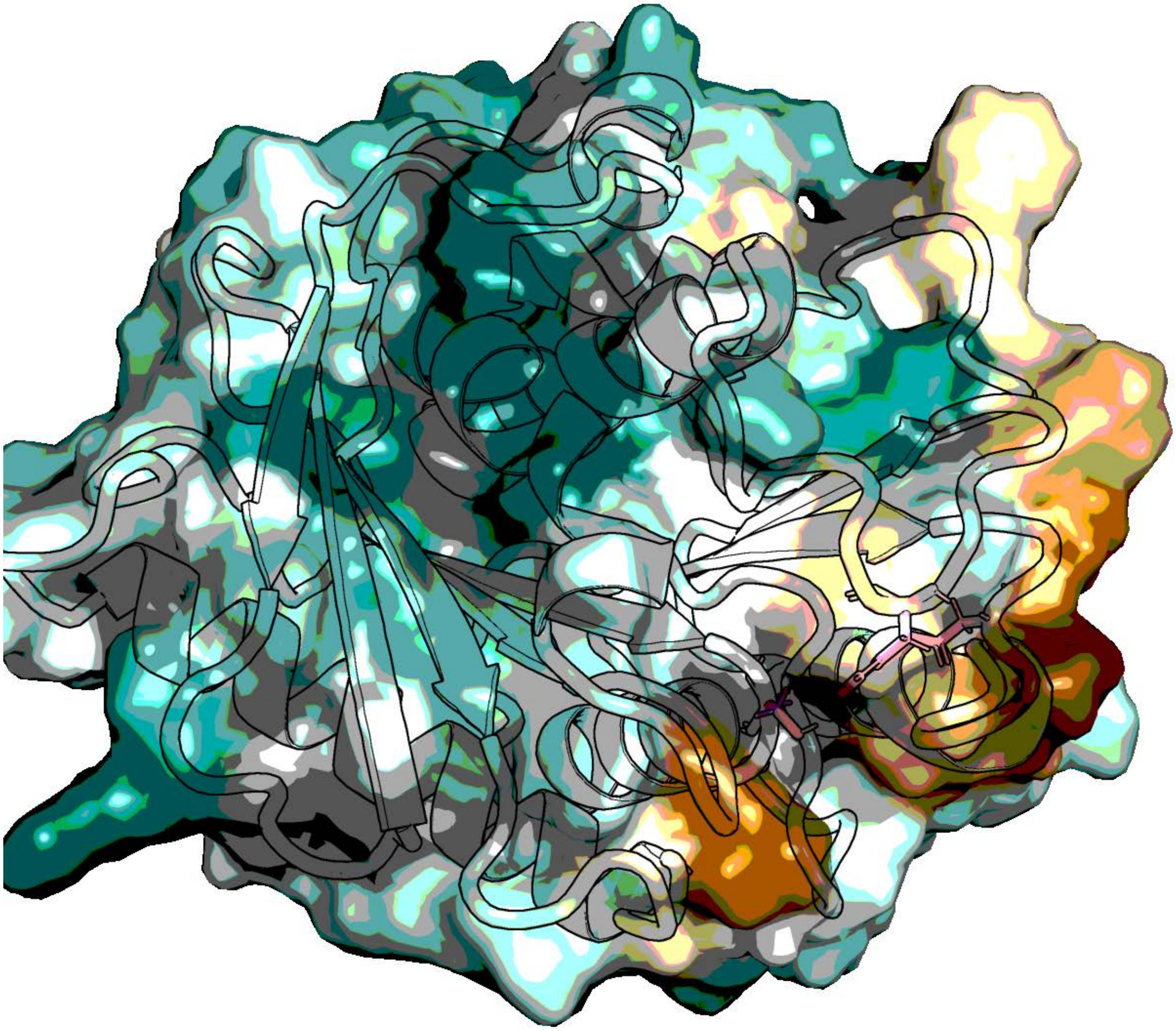

ESM045_highlights.png

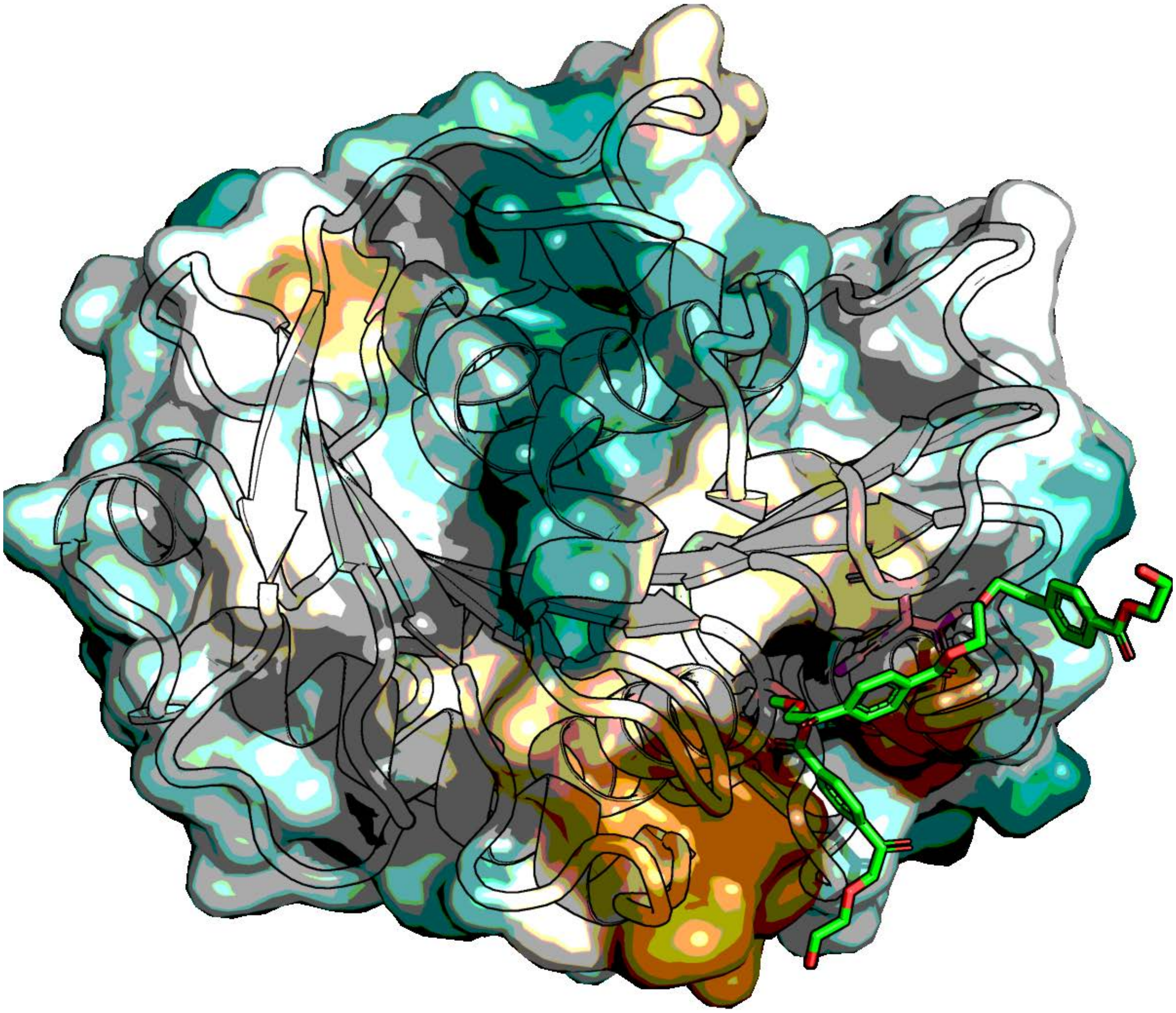

ESM047_highlights.png

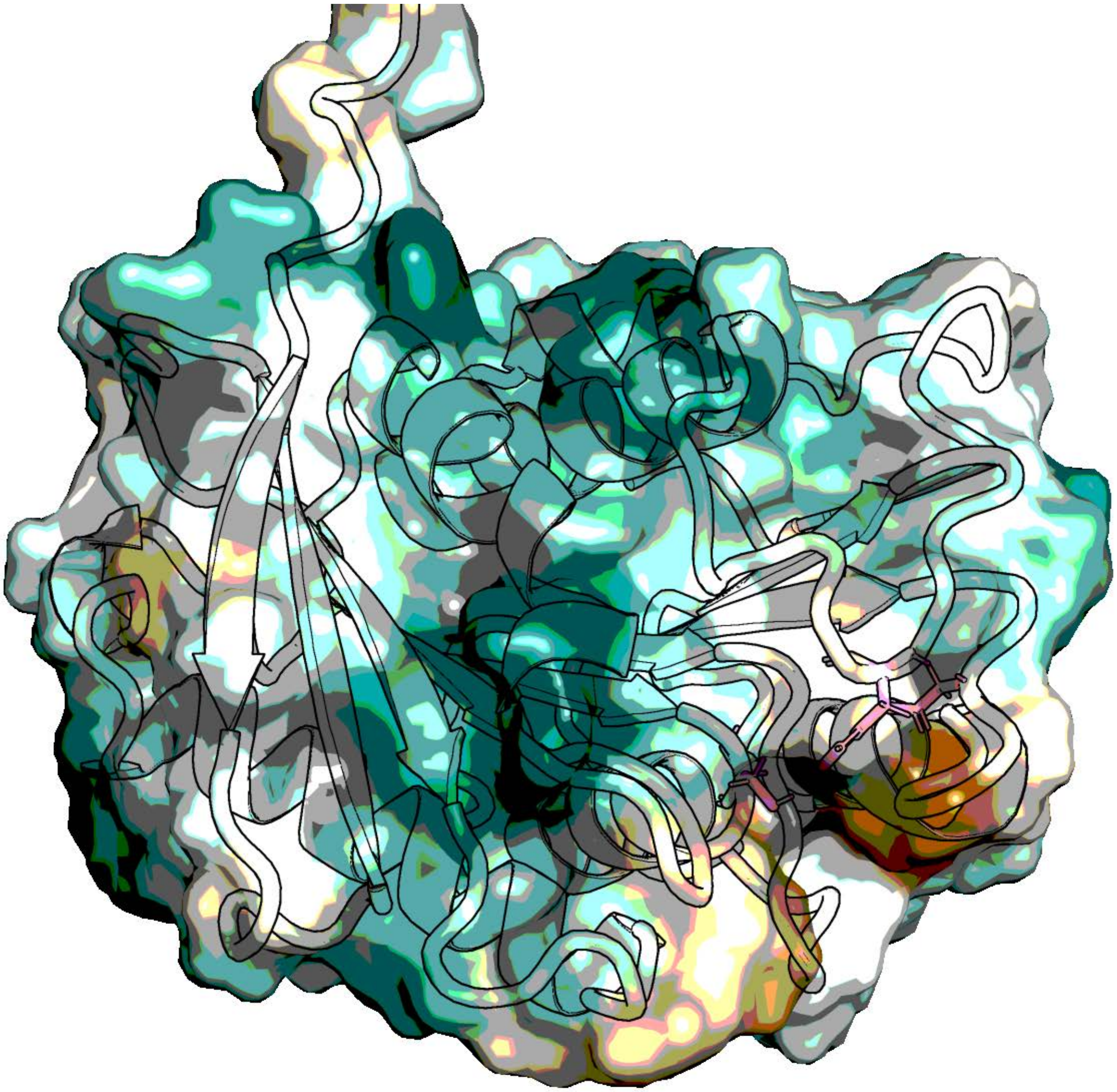

ESM050_highlights.png

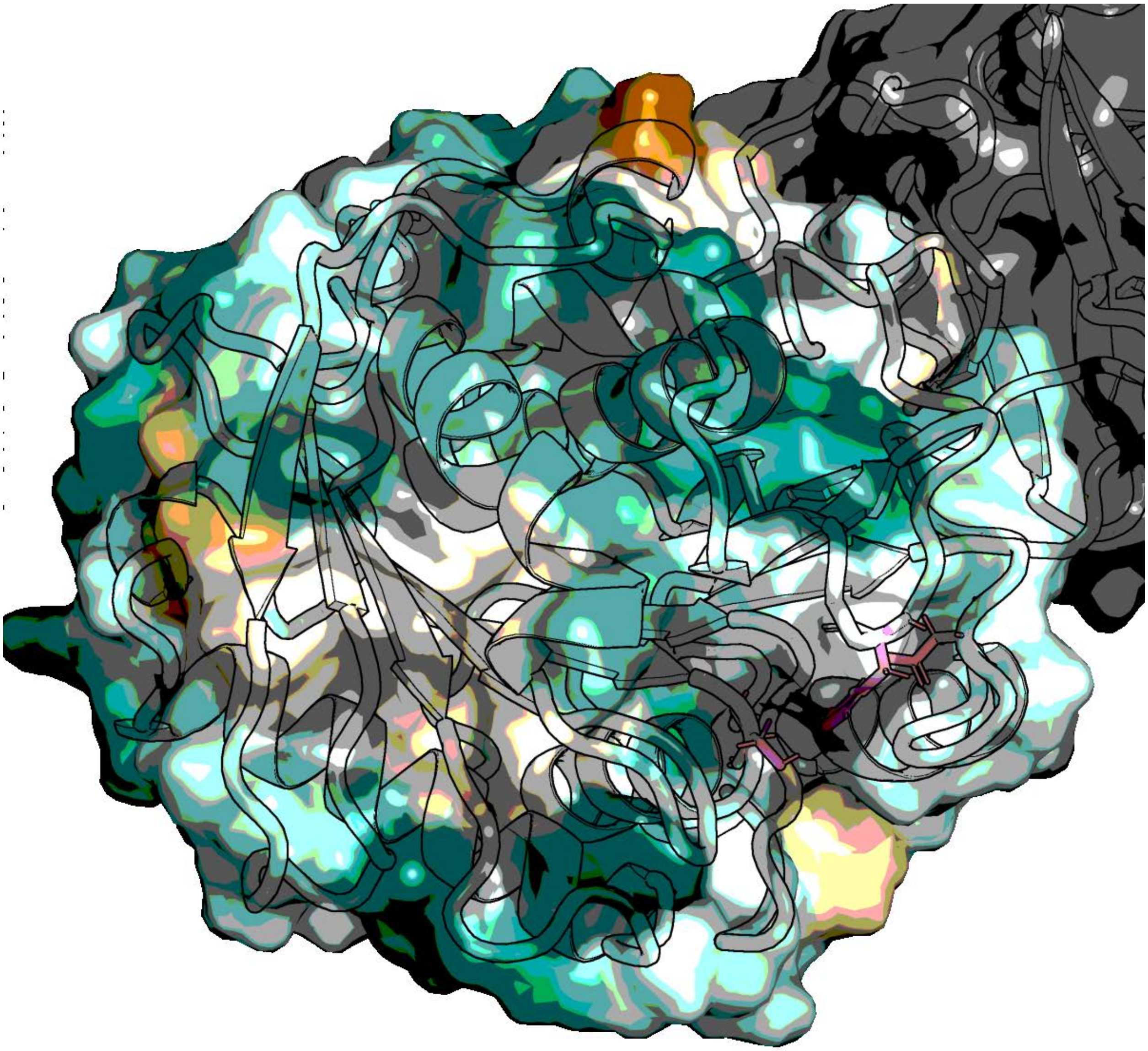

ESM052_highlights.png

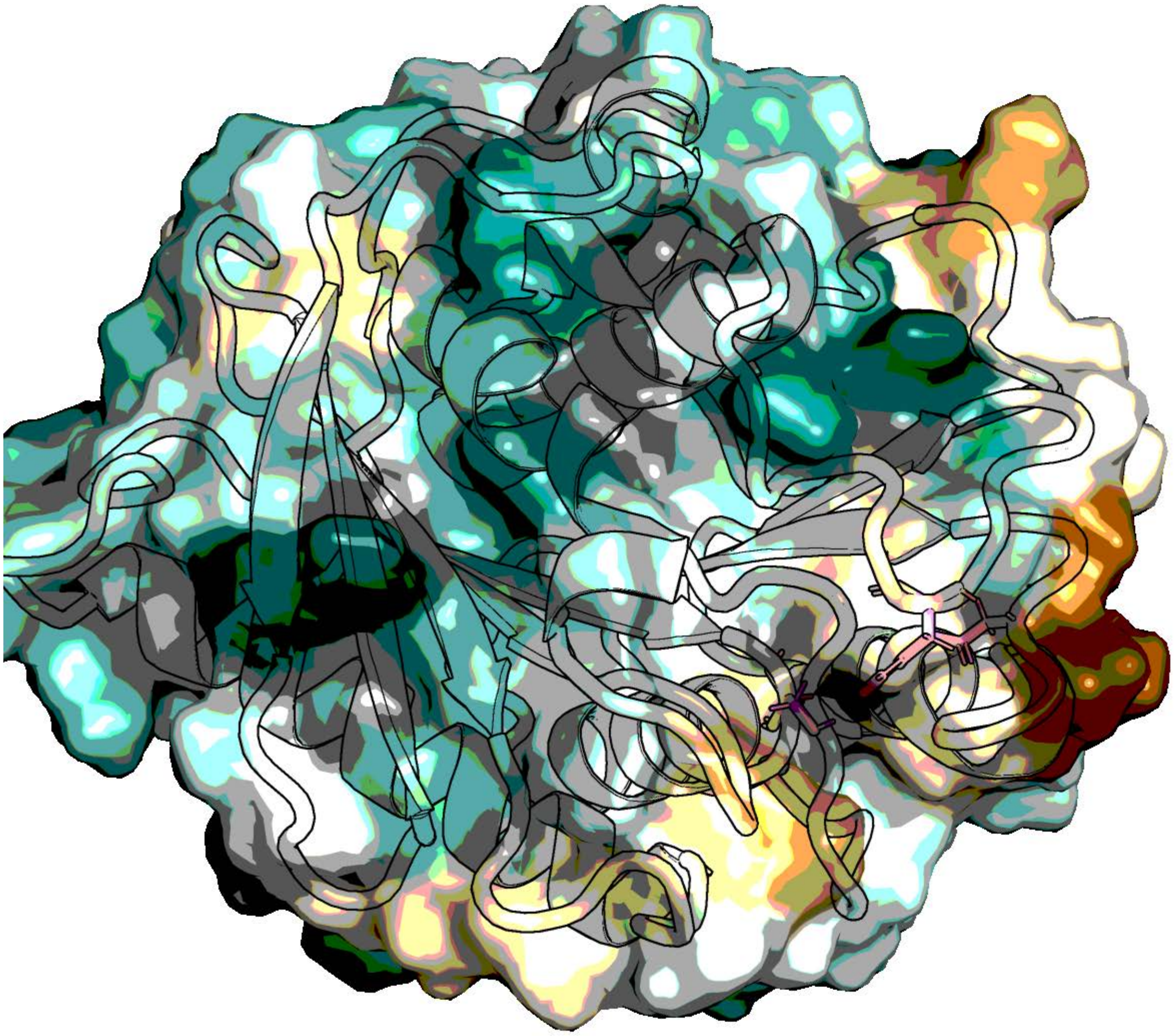

ESM053_highlights.png

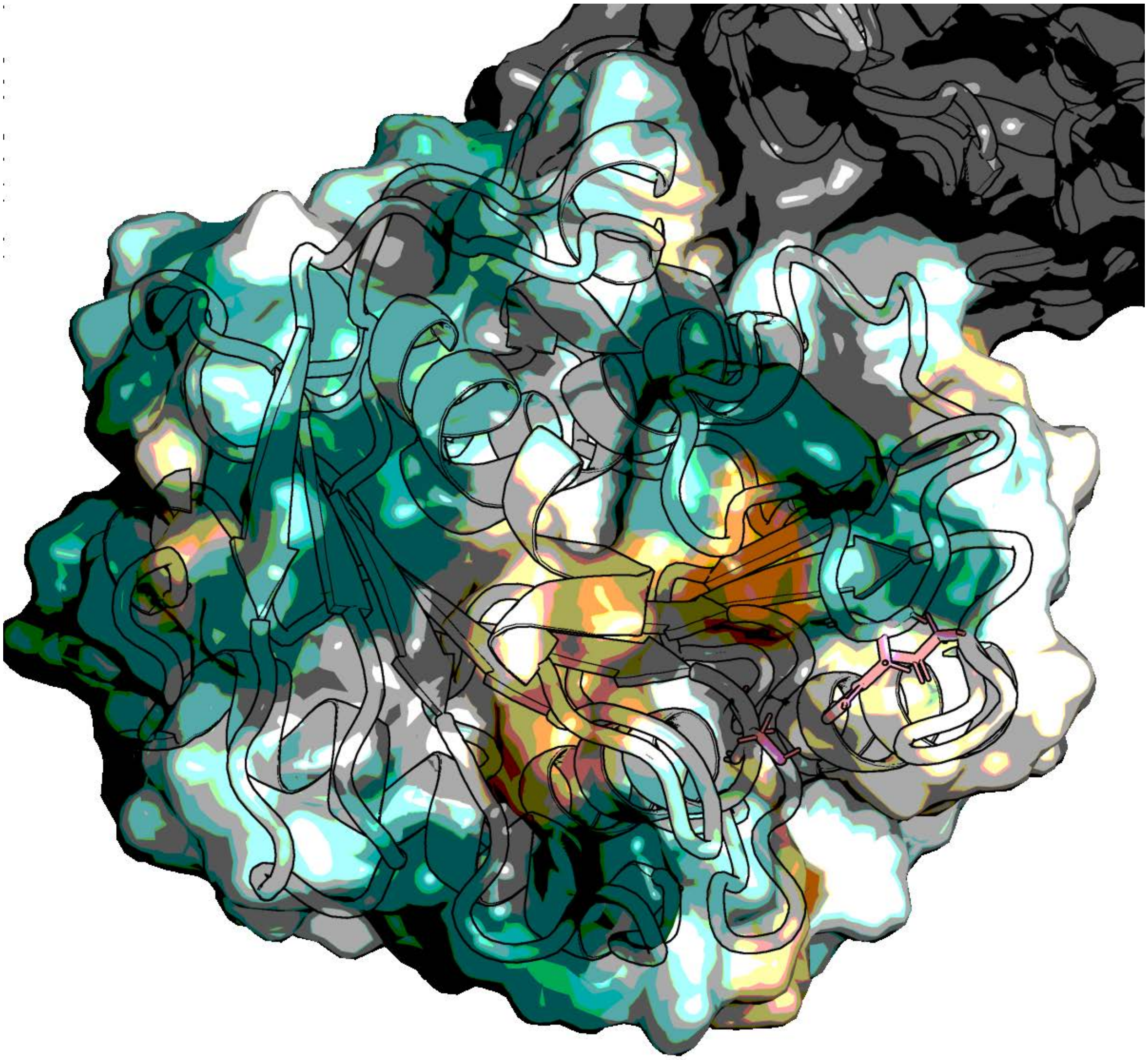

ESM058_highlights.png

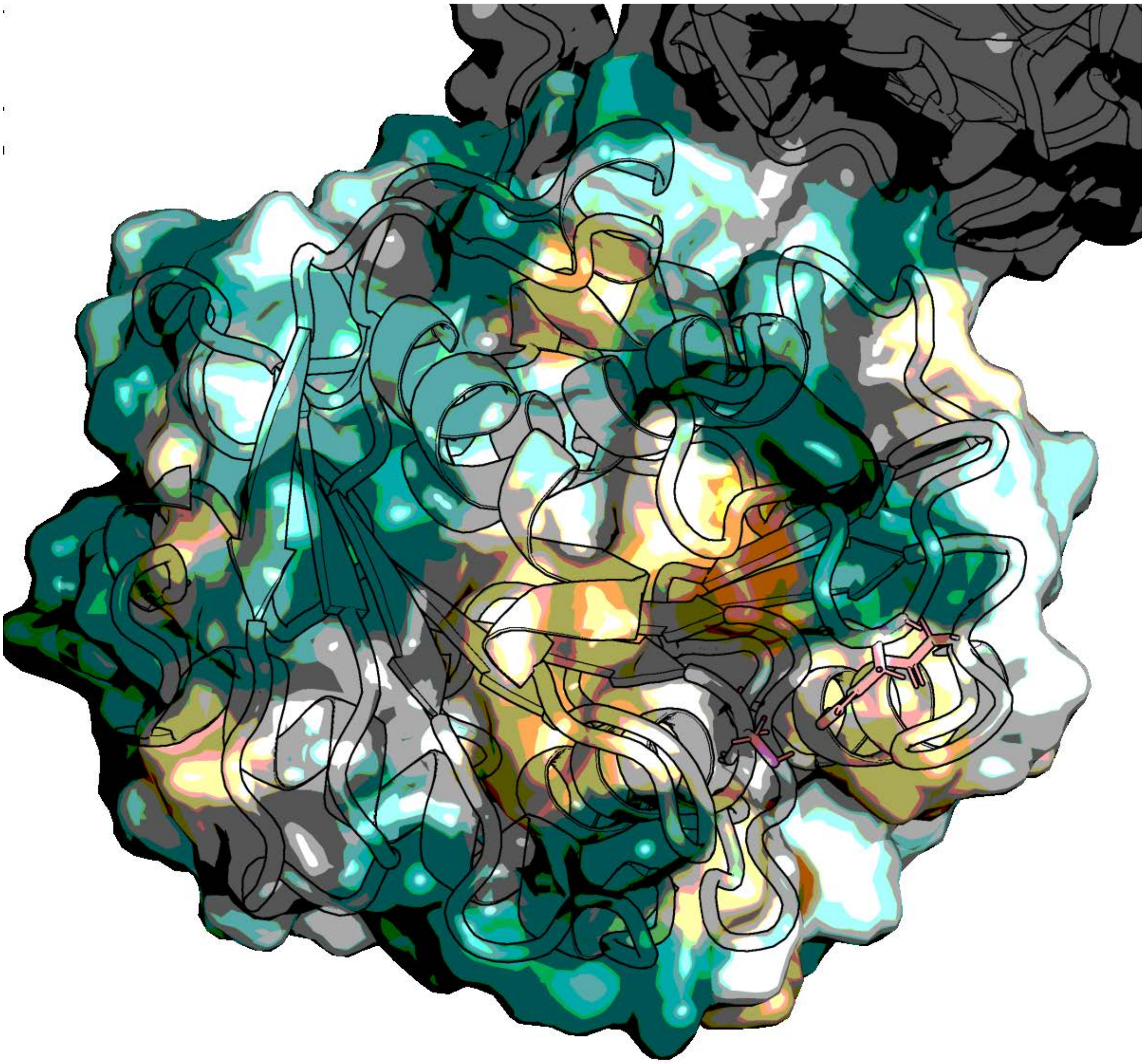

ESM059_highlights.png

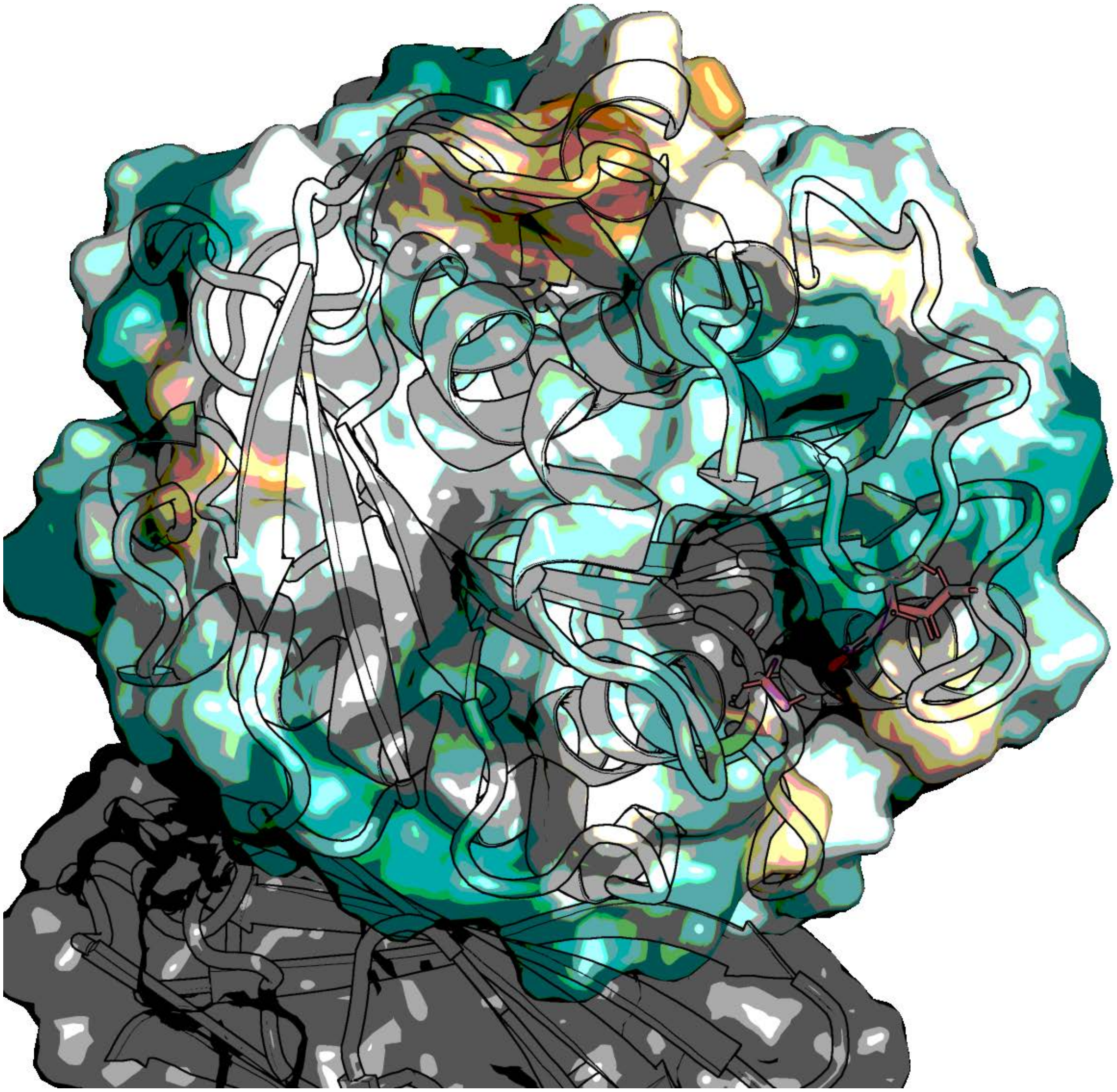

ESM060_highlights.png

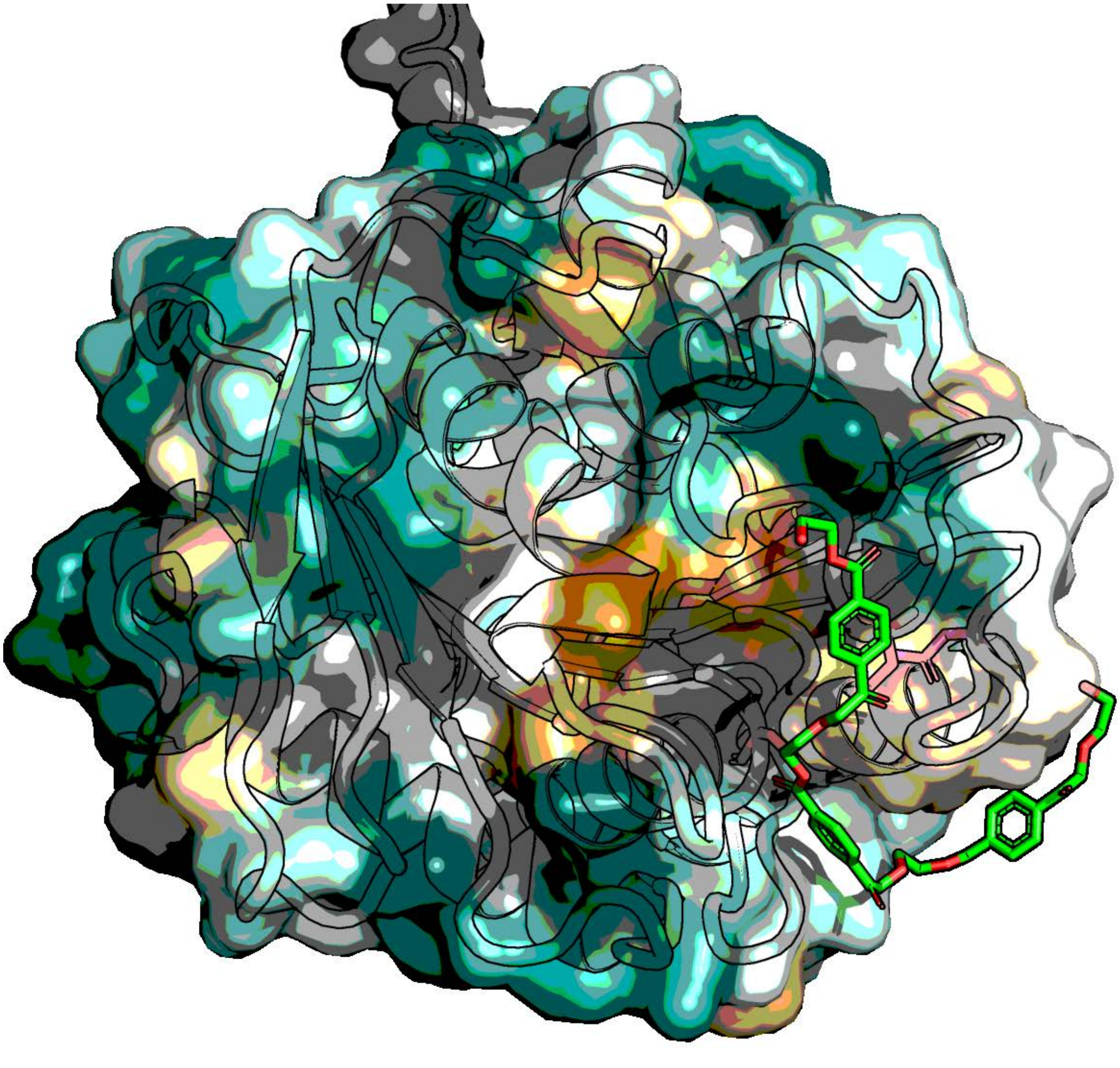

ESM061_highlights.png

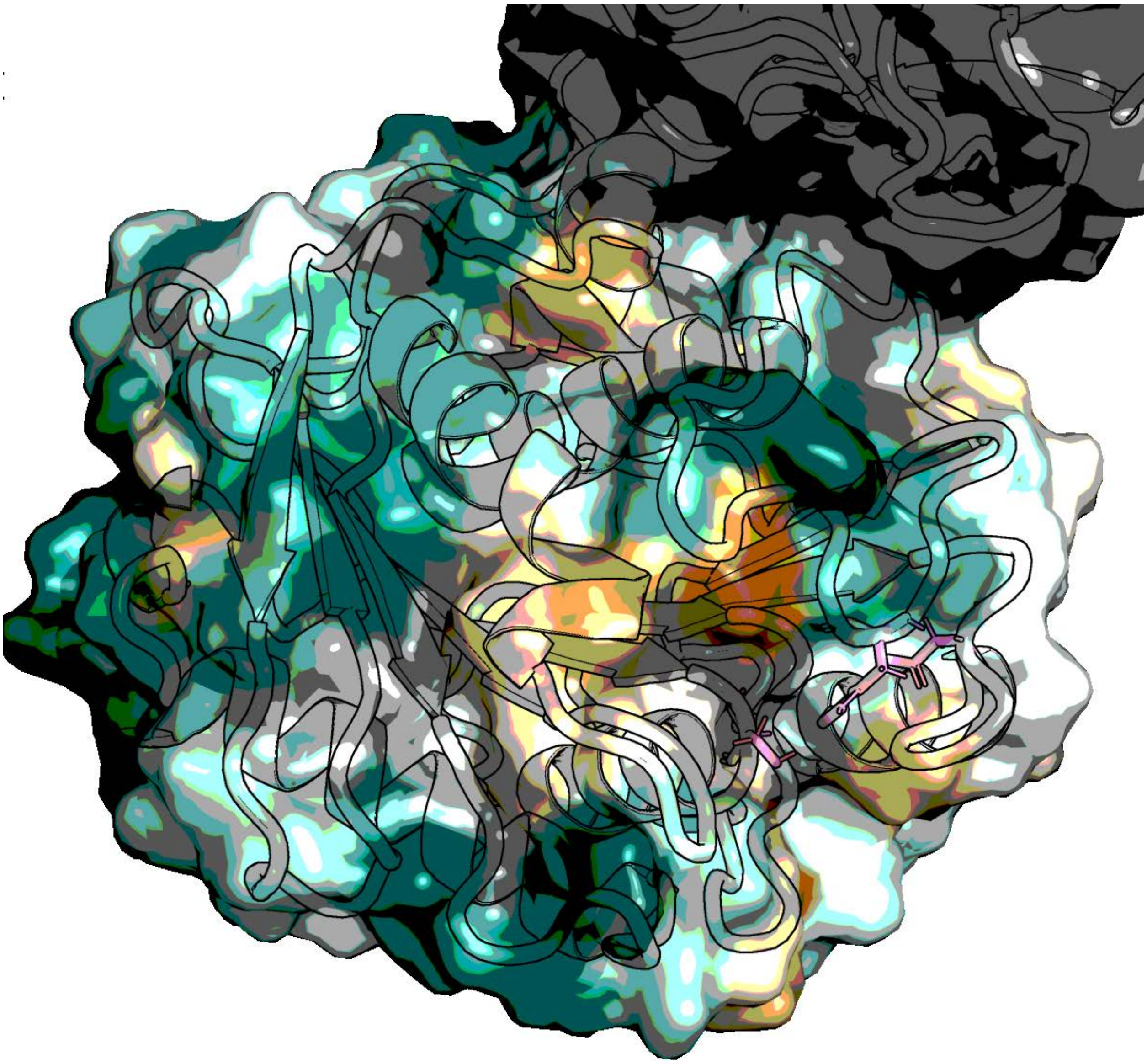

ESM062_highlights.png

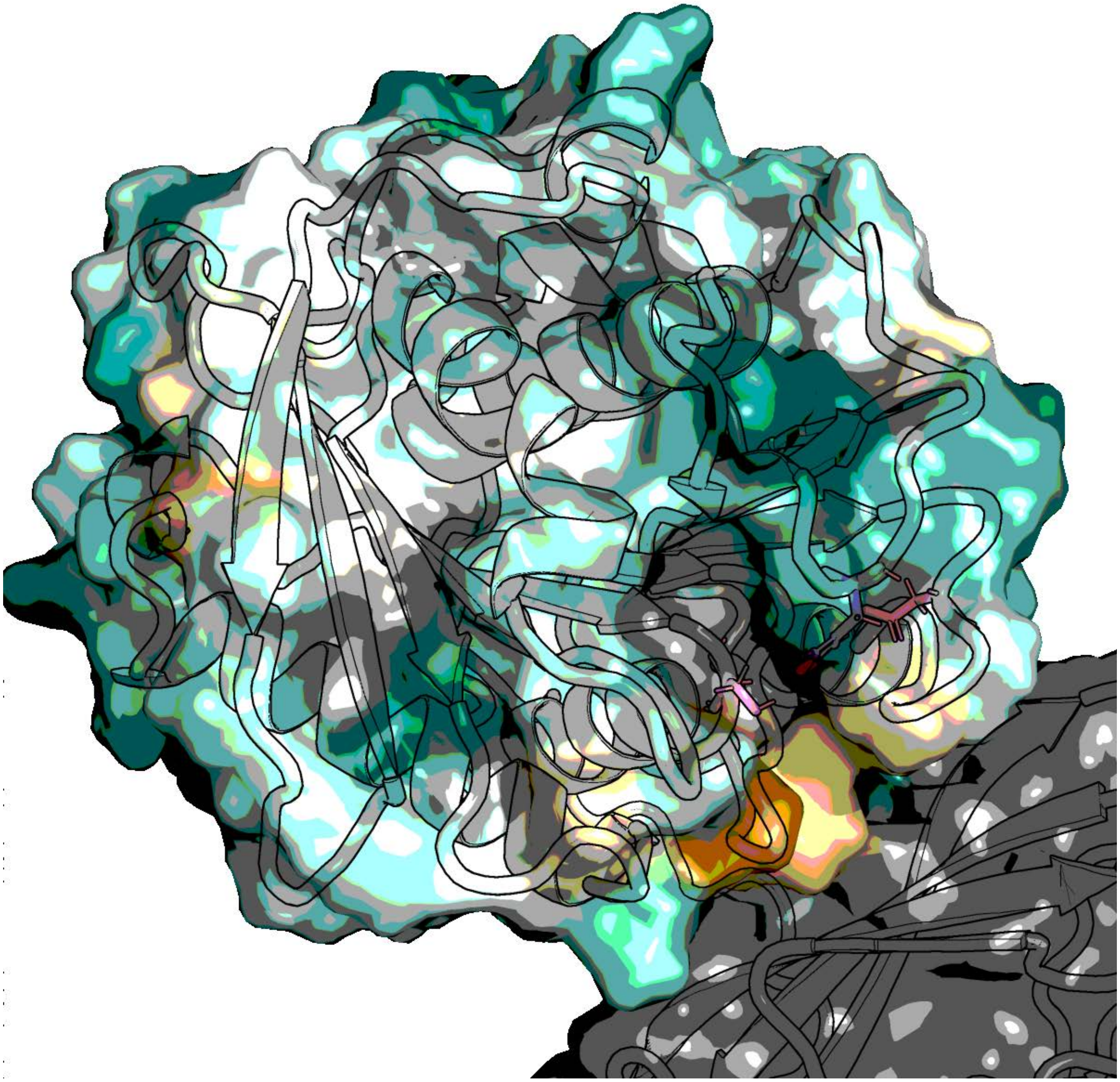

ESM064_highlights.png

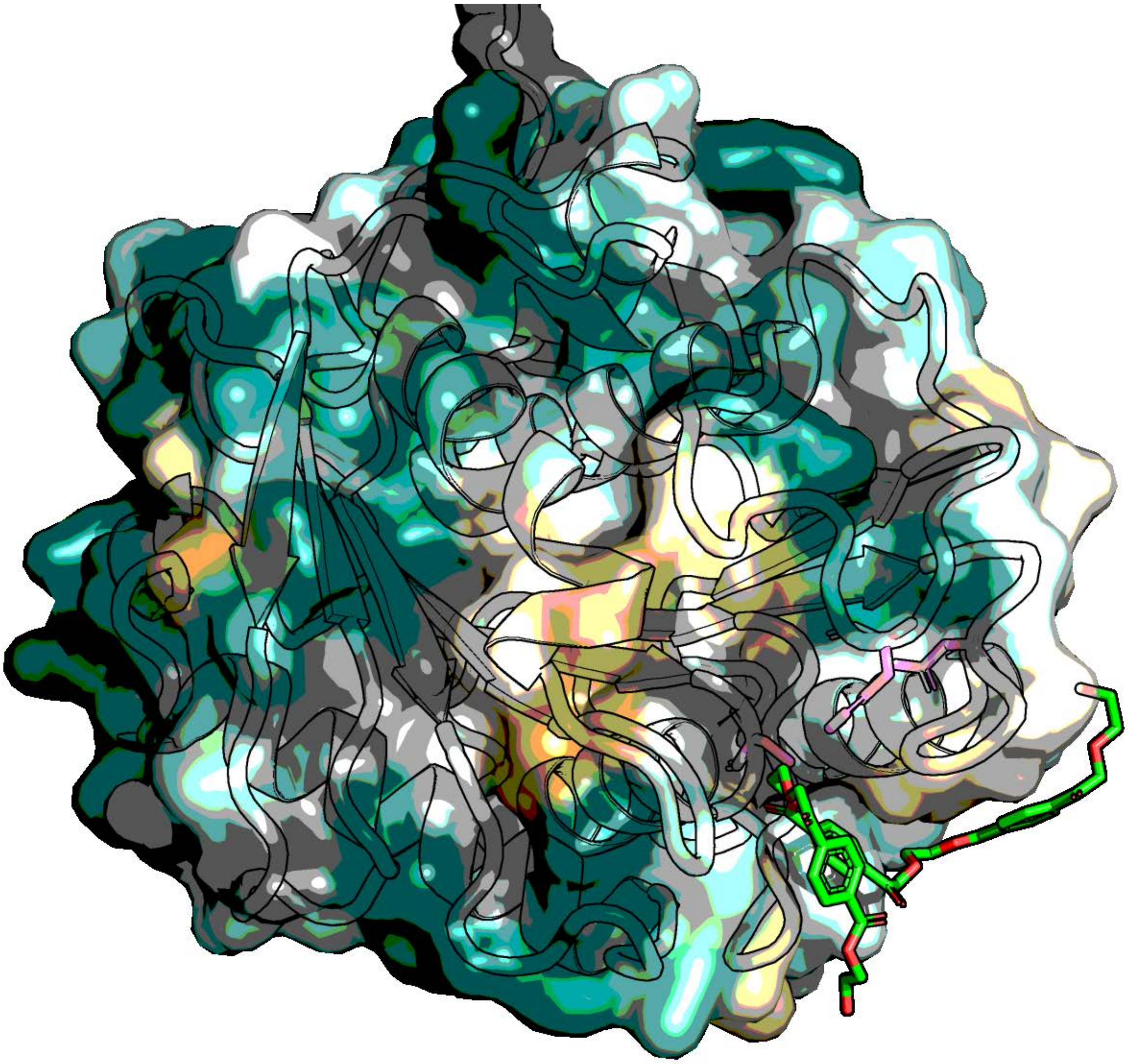

ESM065_highlights.png

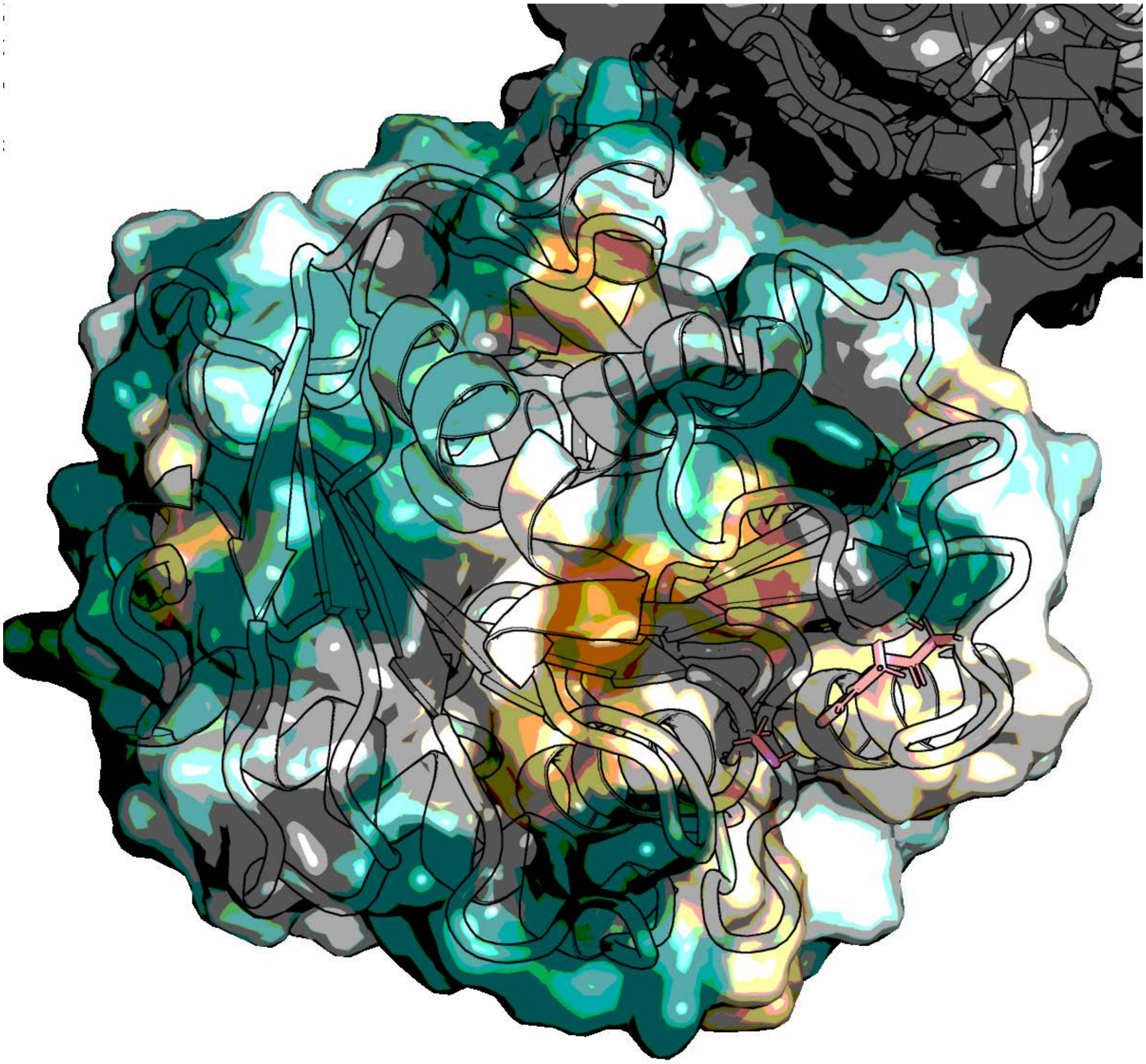

ESM069_highlights.png

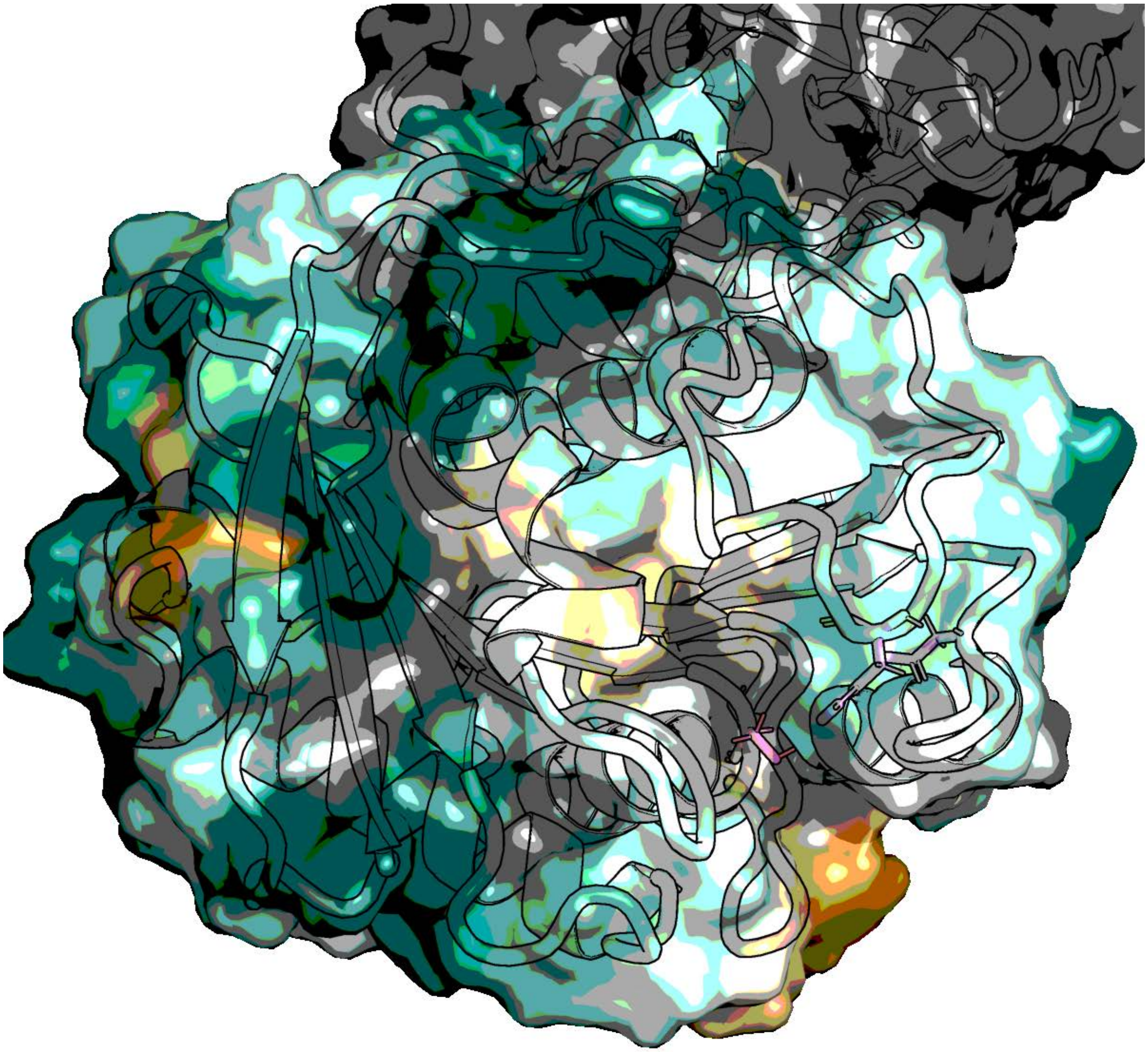

ESM070_highlights.png

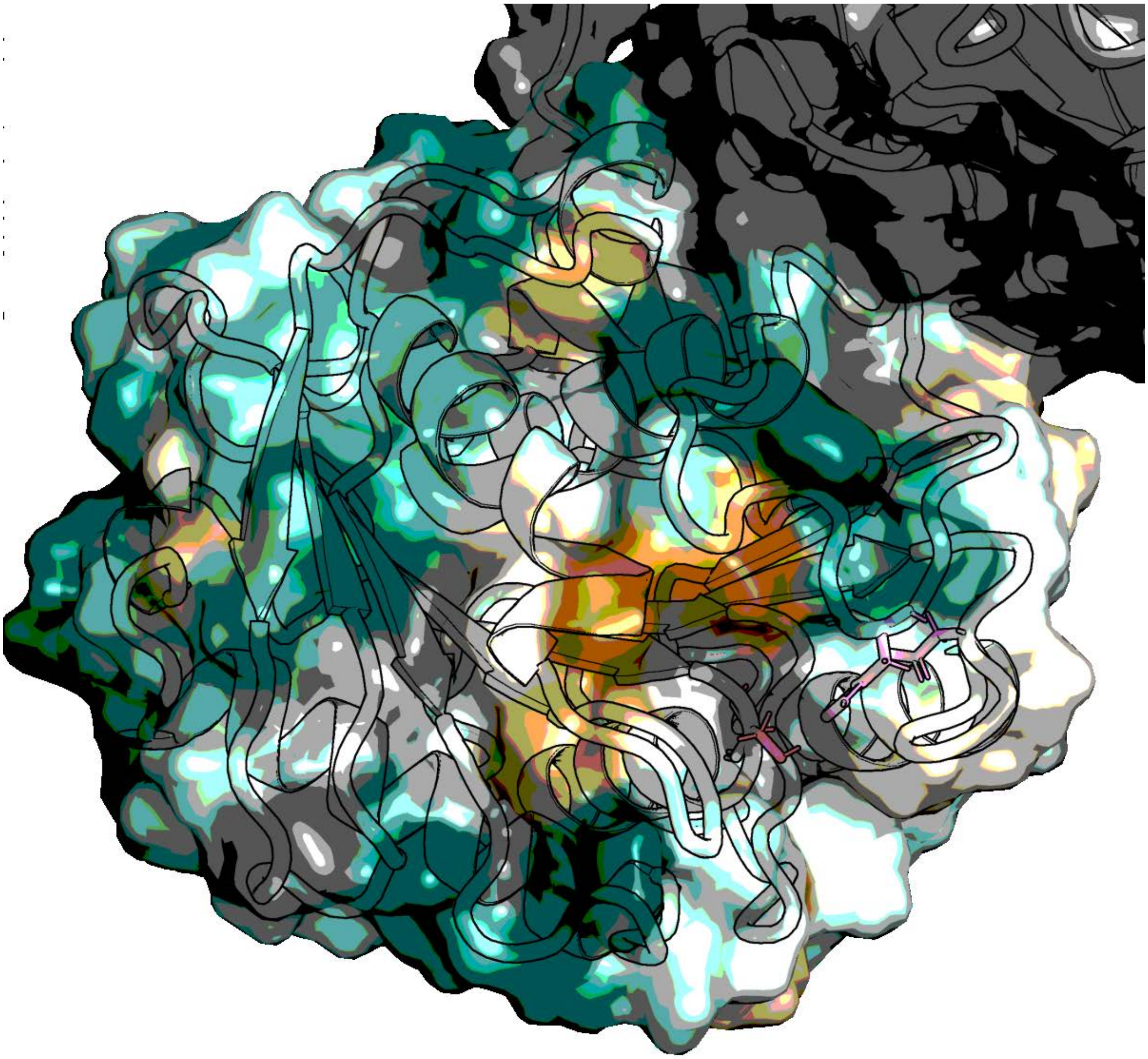

ESM075_highlights.png

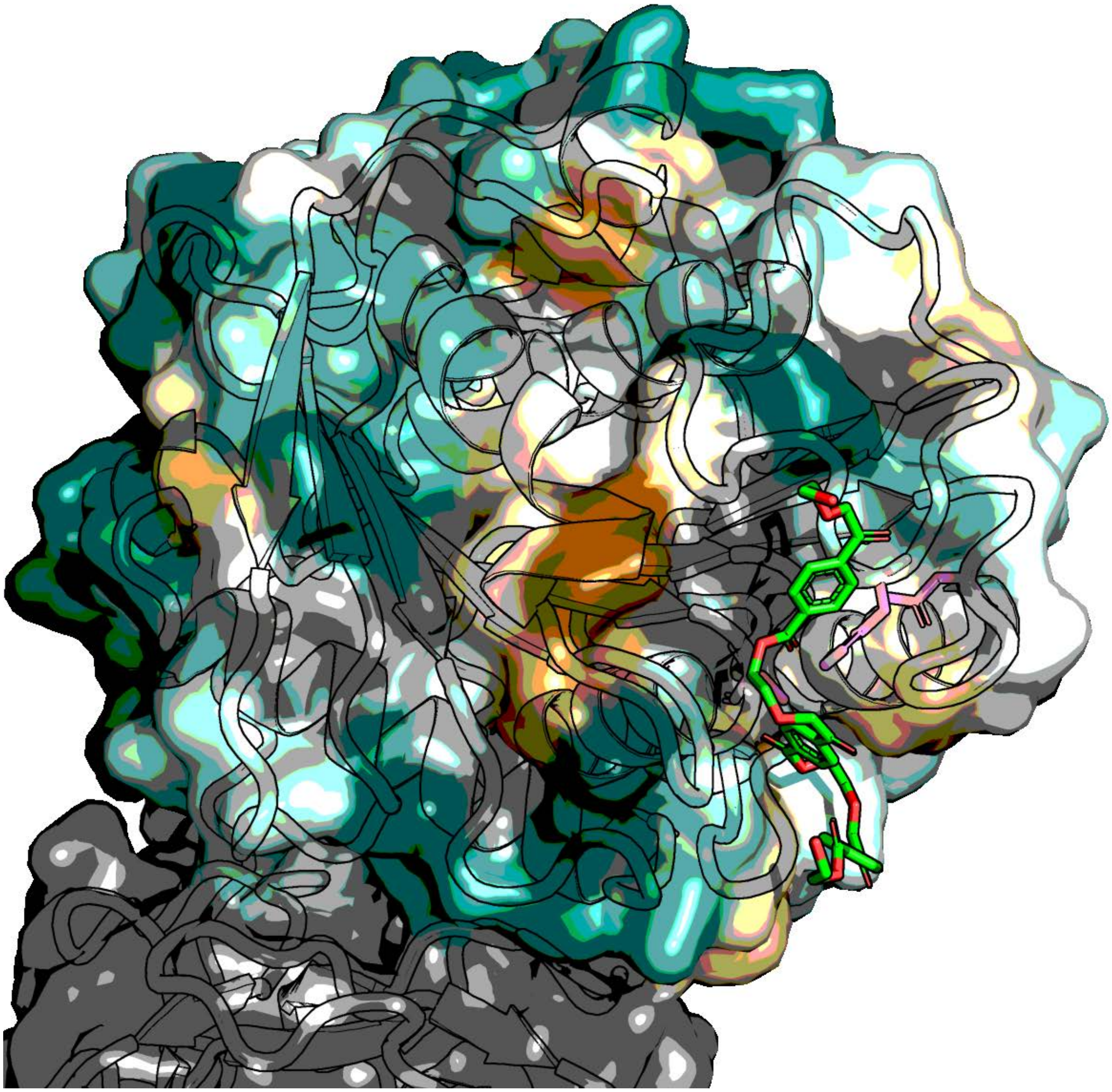

ESM080_highlights.png

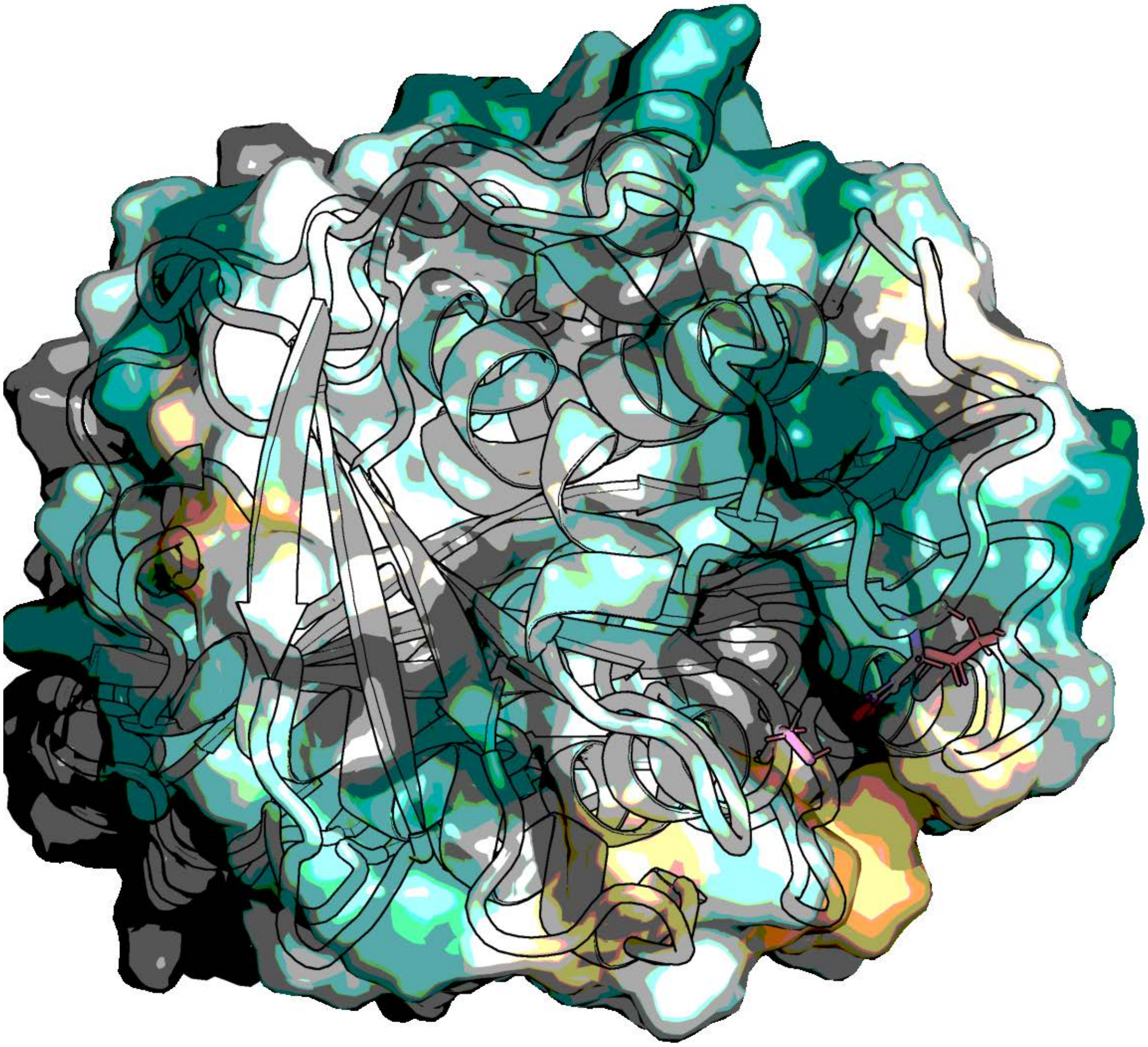

ESM081_highlights.png

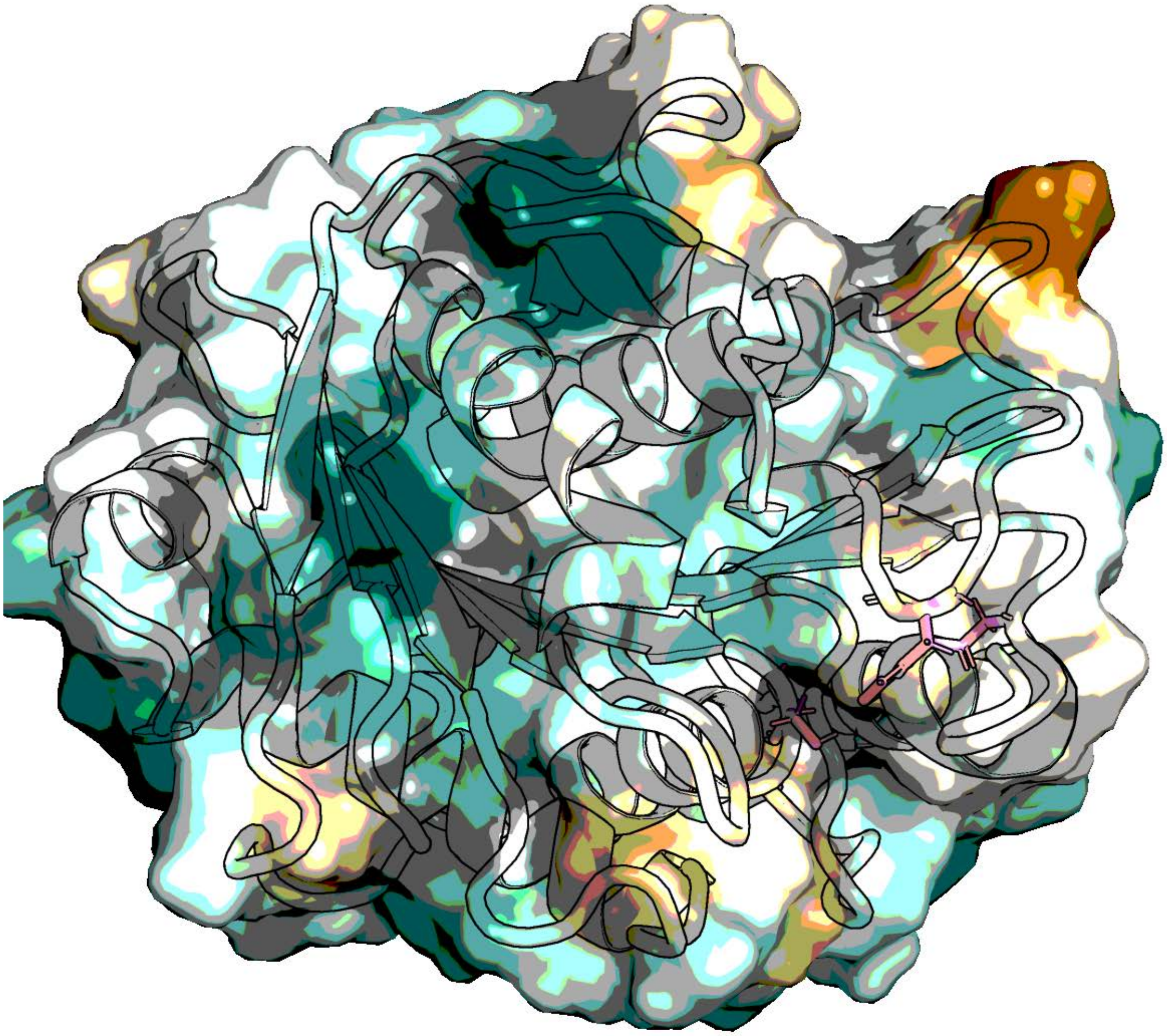

ESM082_highlights.png

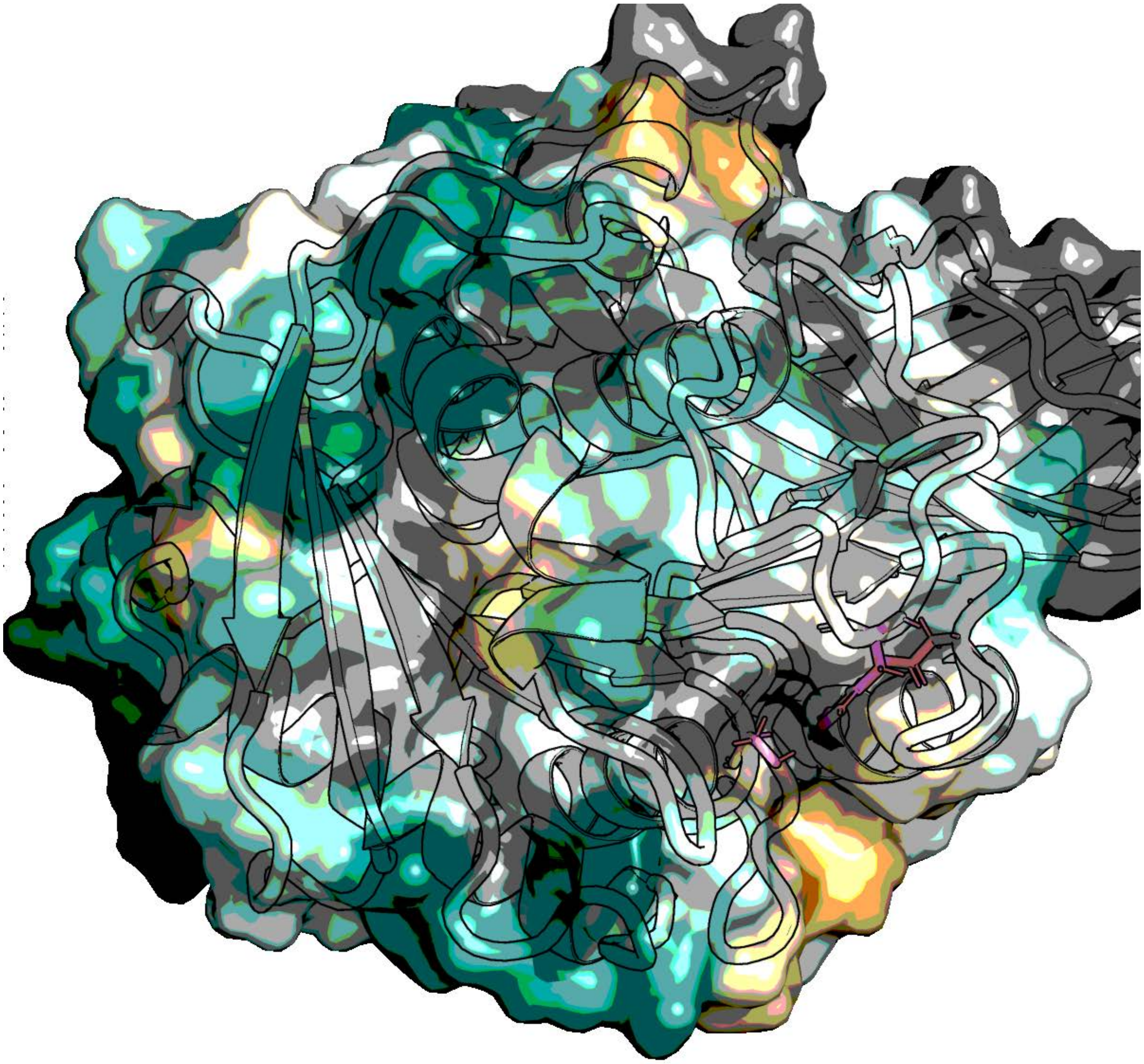

ESM084_highlights.png

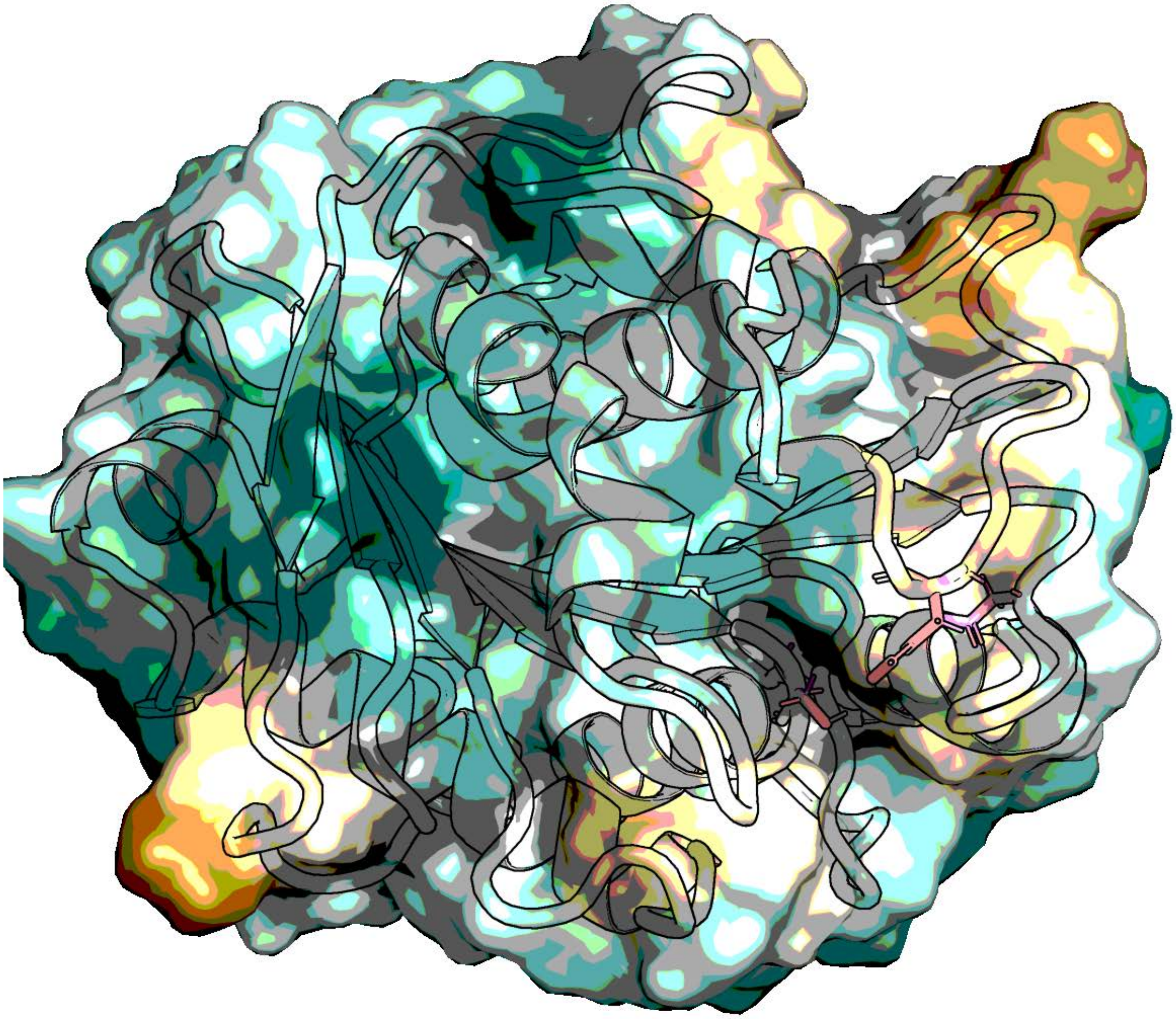

ESM090_highlights.png

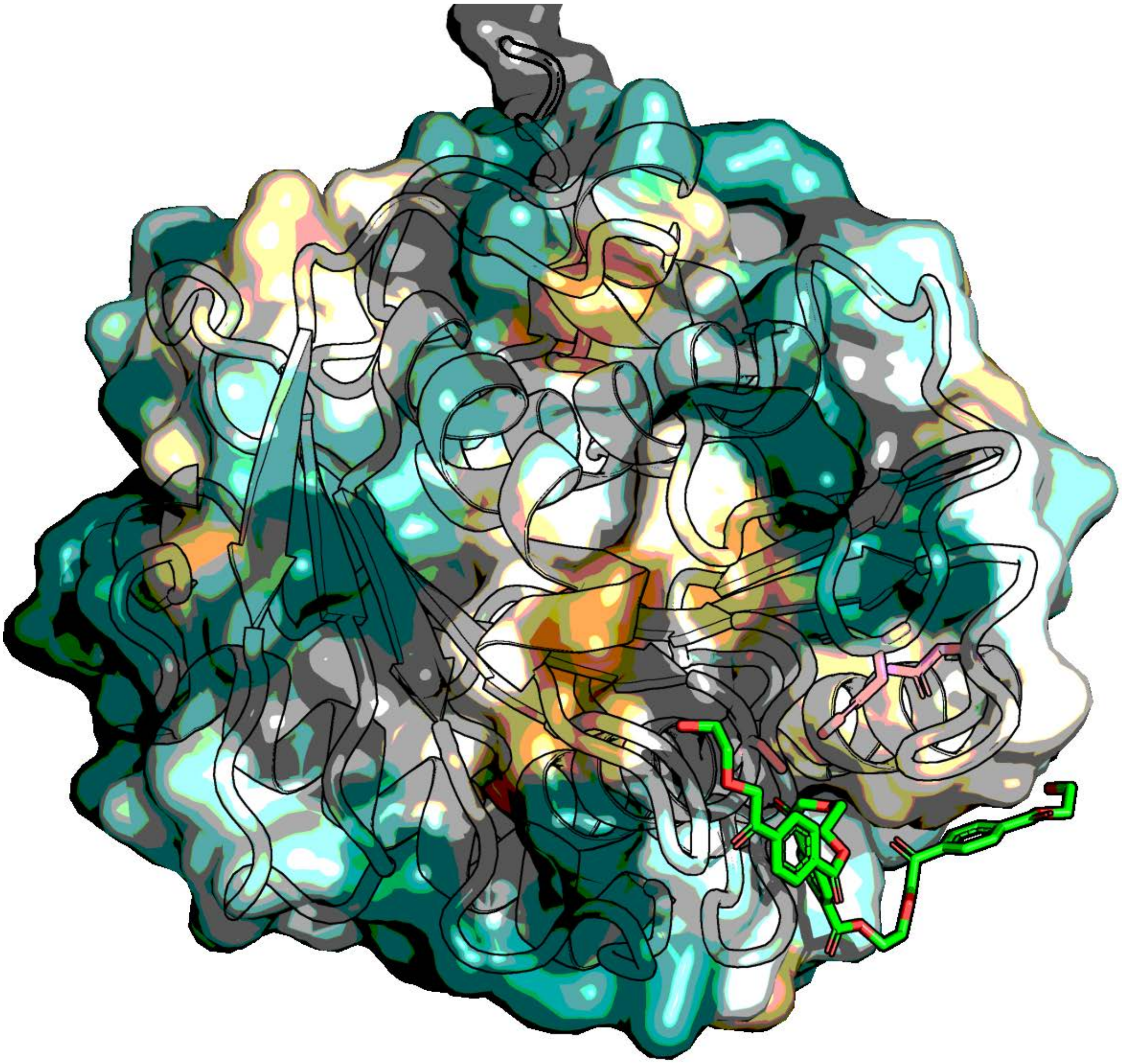

ESM091_highlights.png

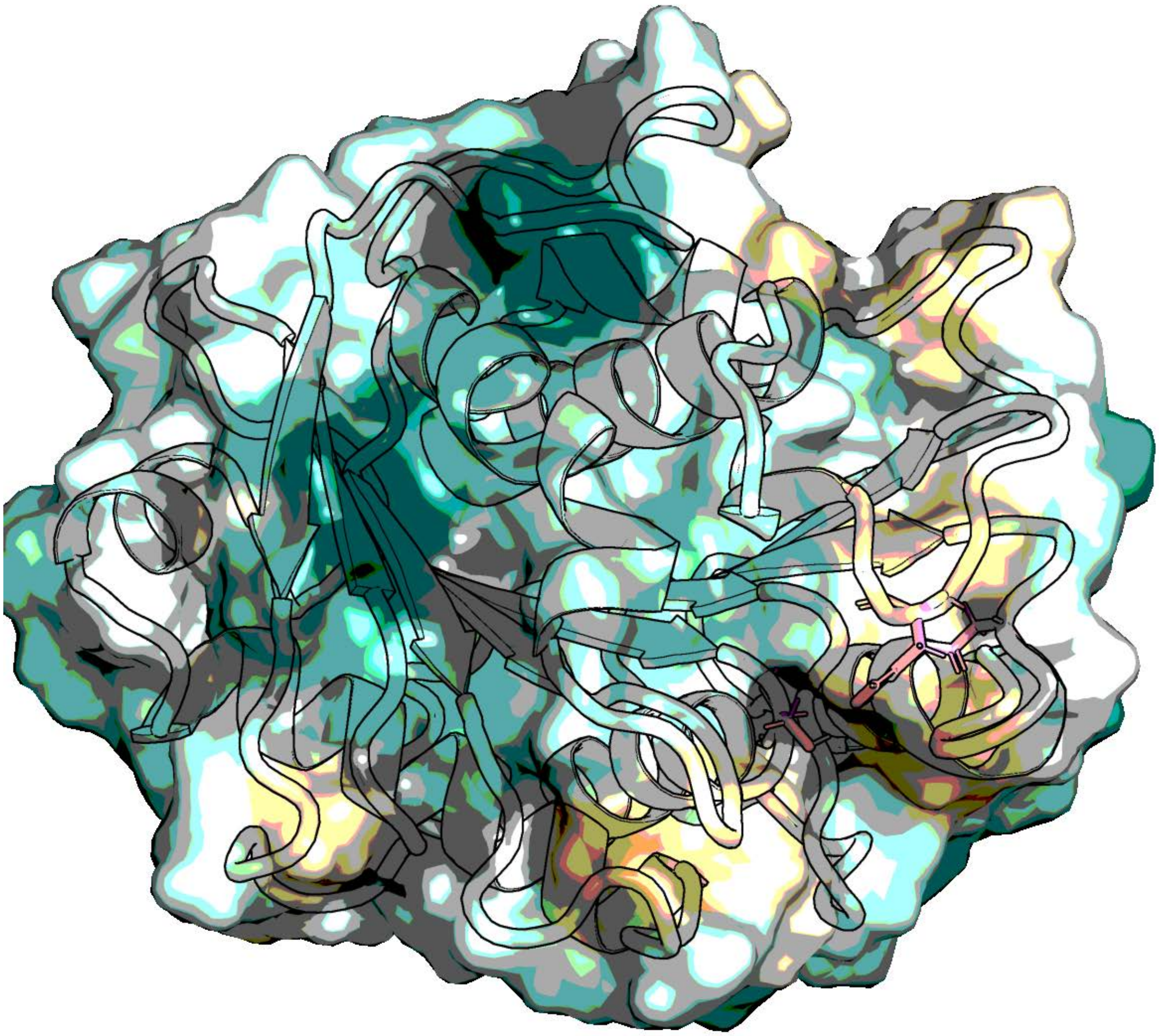

ESM093_highlights.png

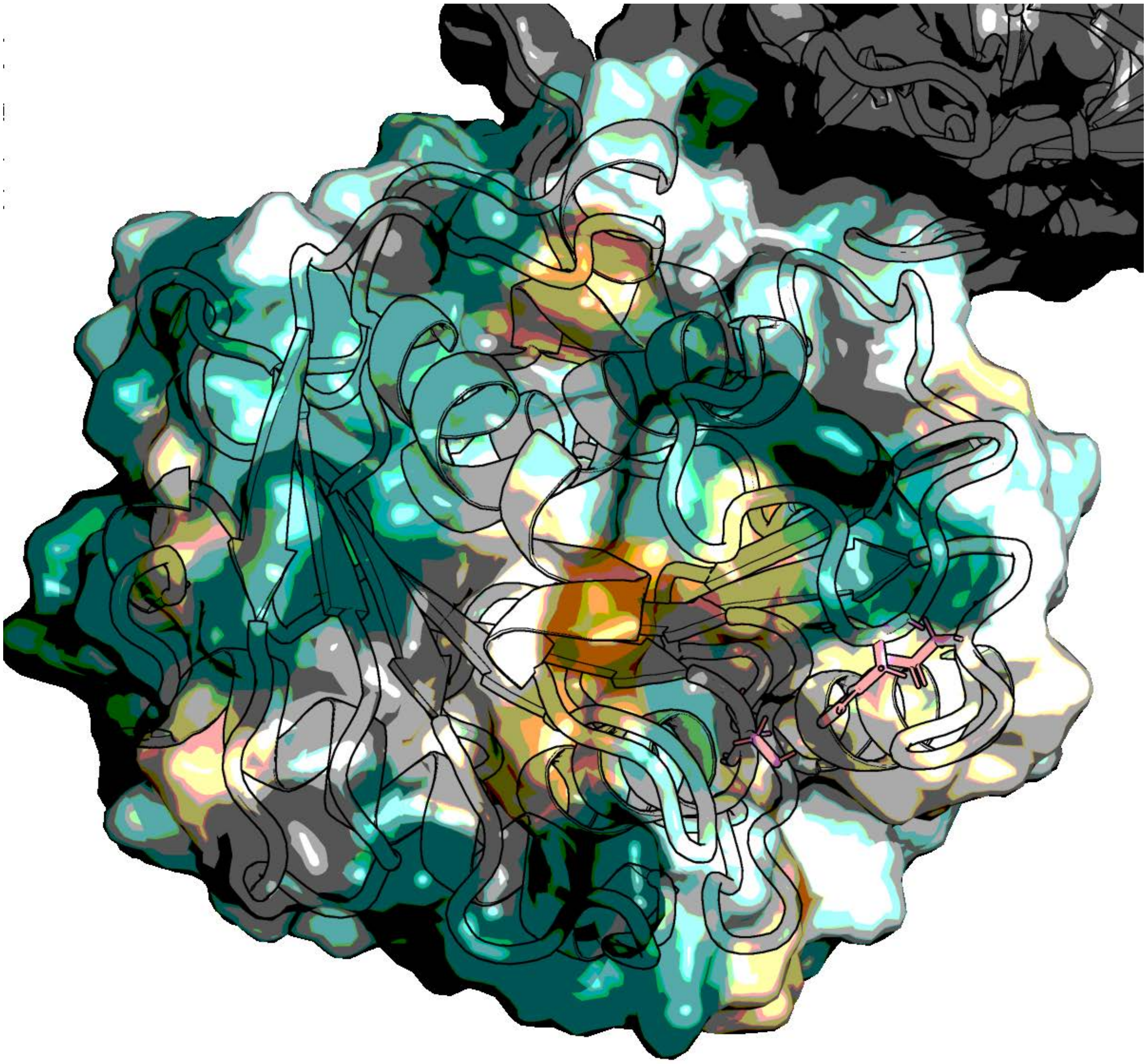

ESM095_highlights.png

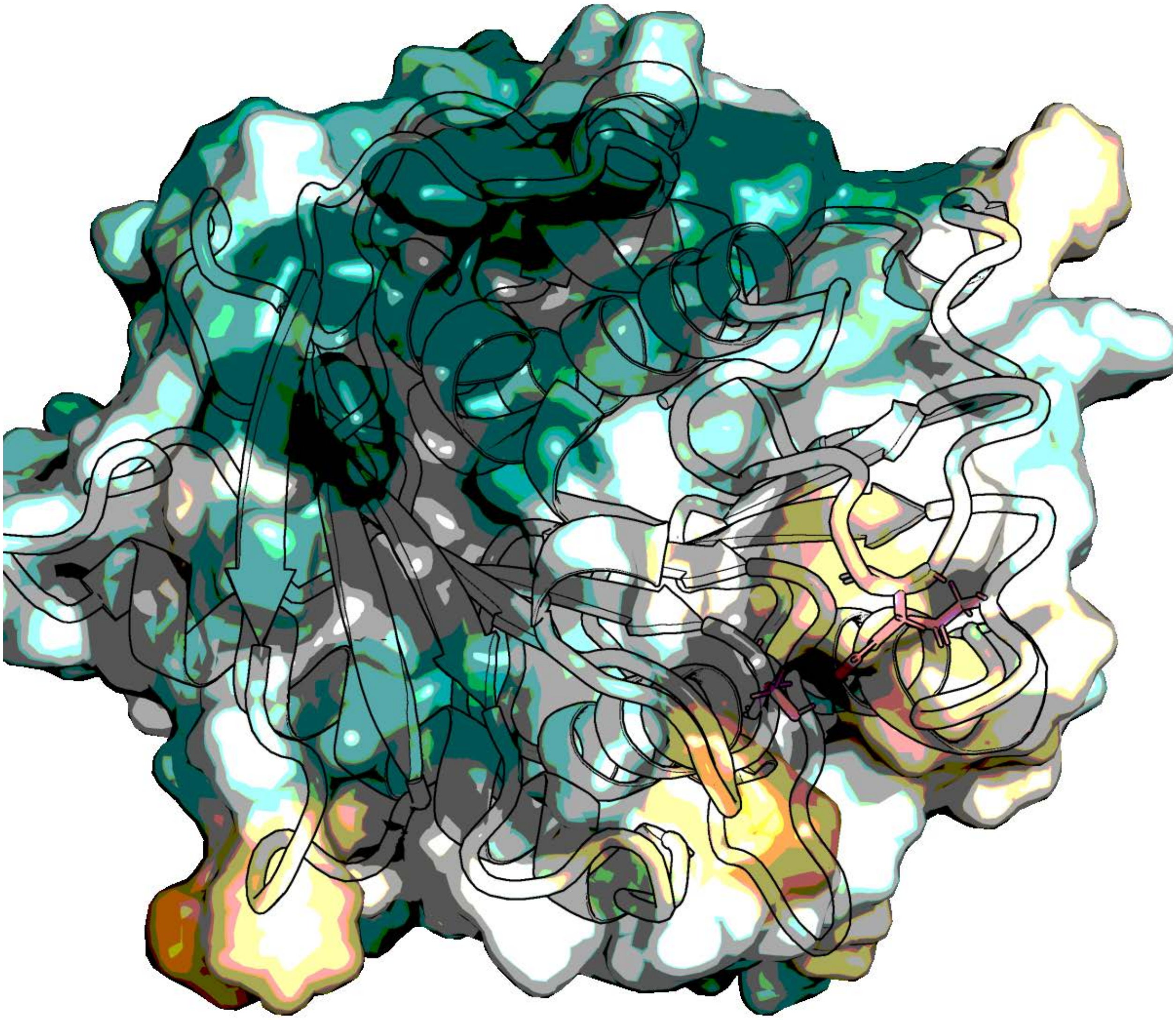

ESM096_highlights.png

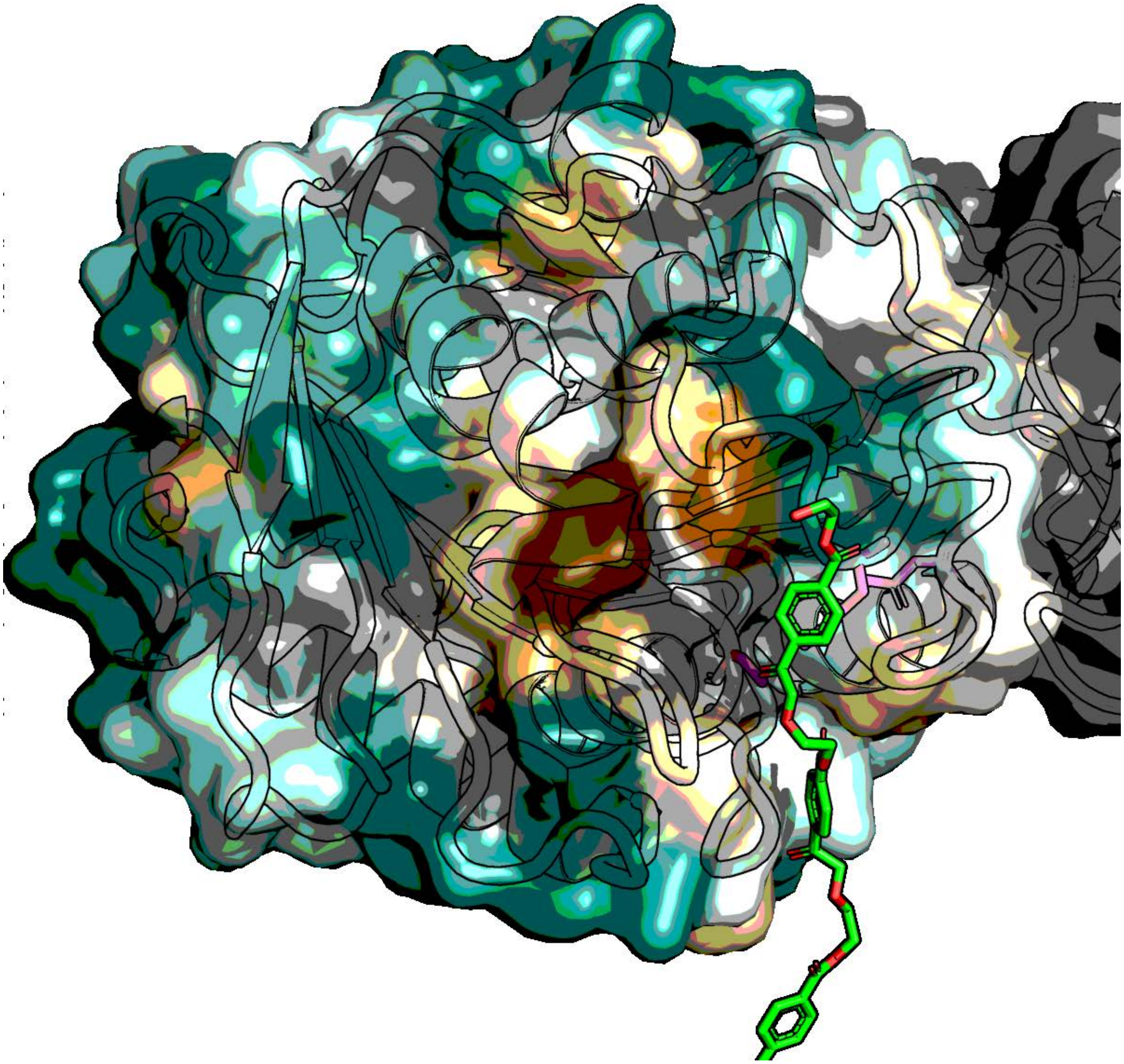

ESM101_highlights.png

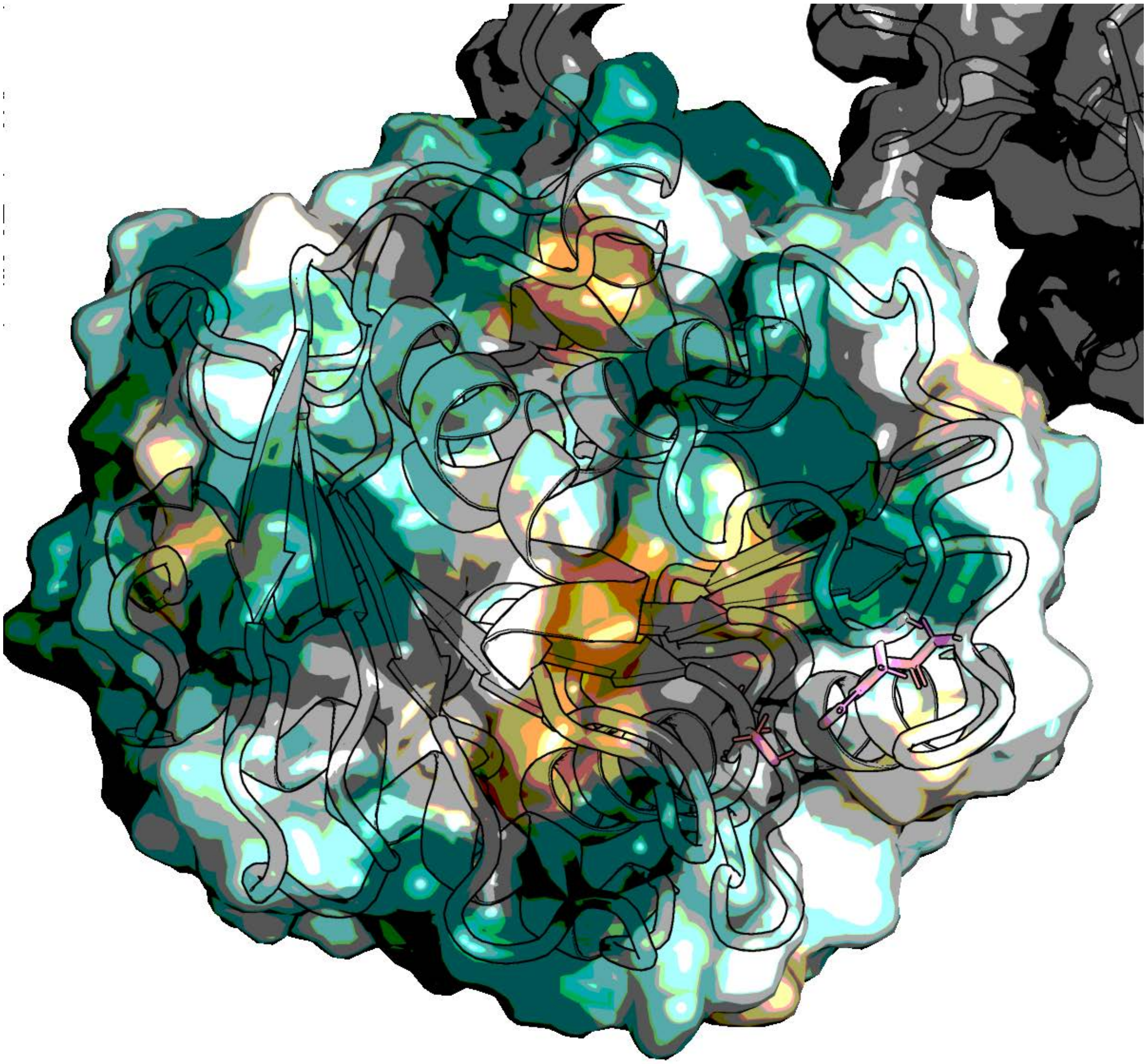

ESM106_highlights.png

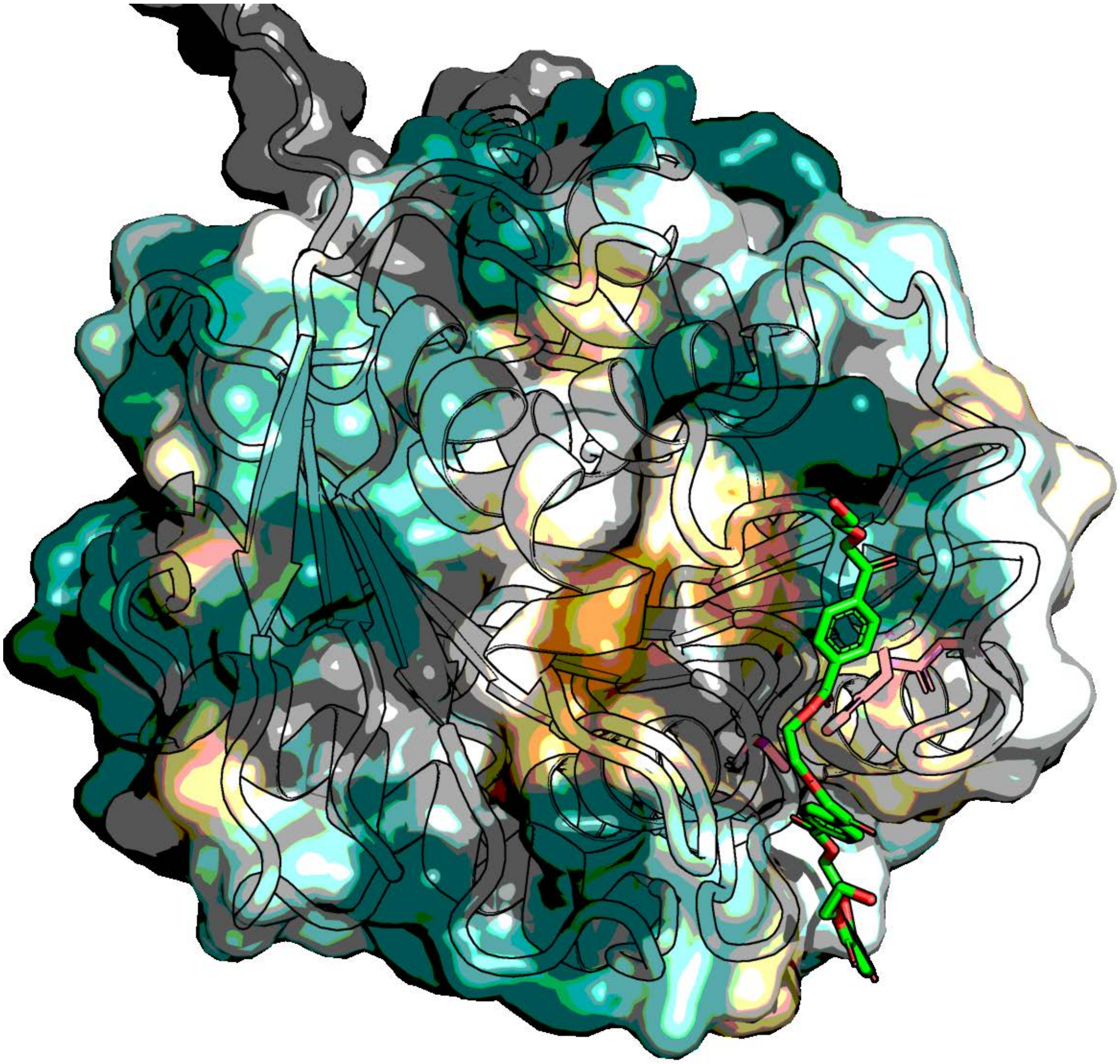

ESM121_highlights.png

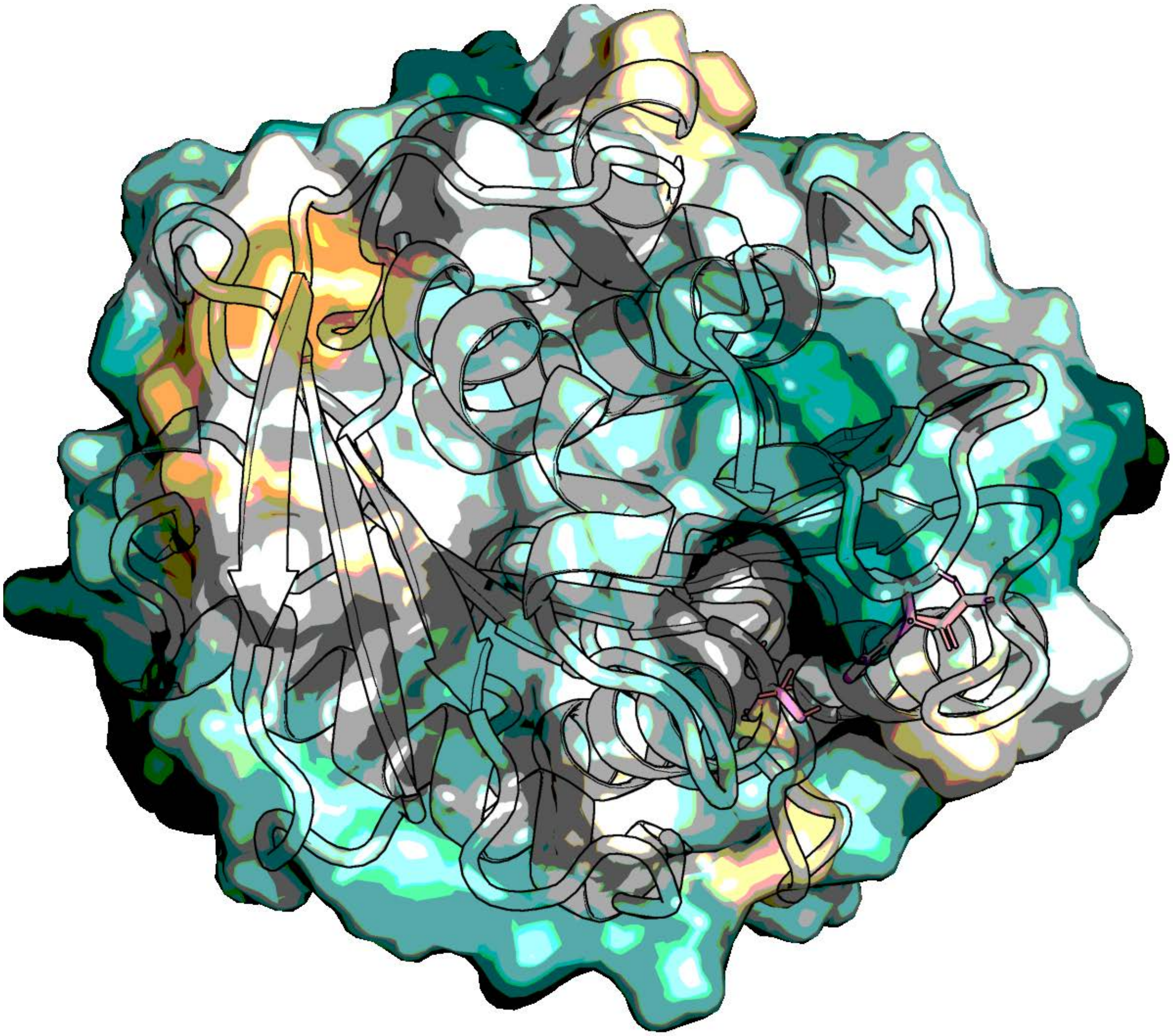

ESM128_highlights.png

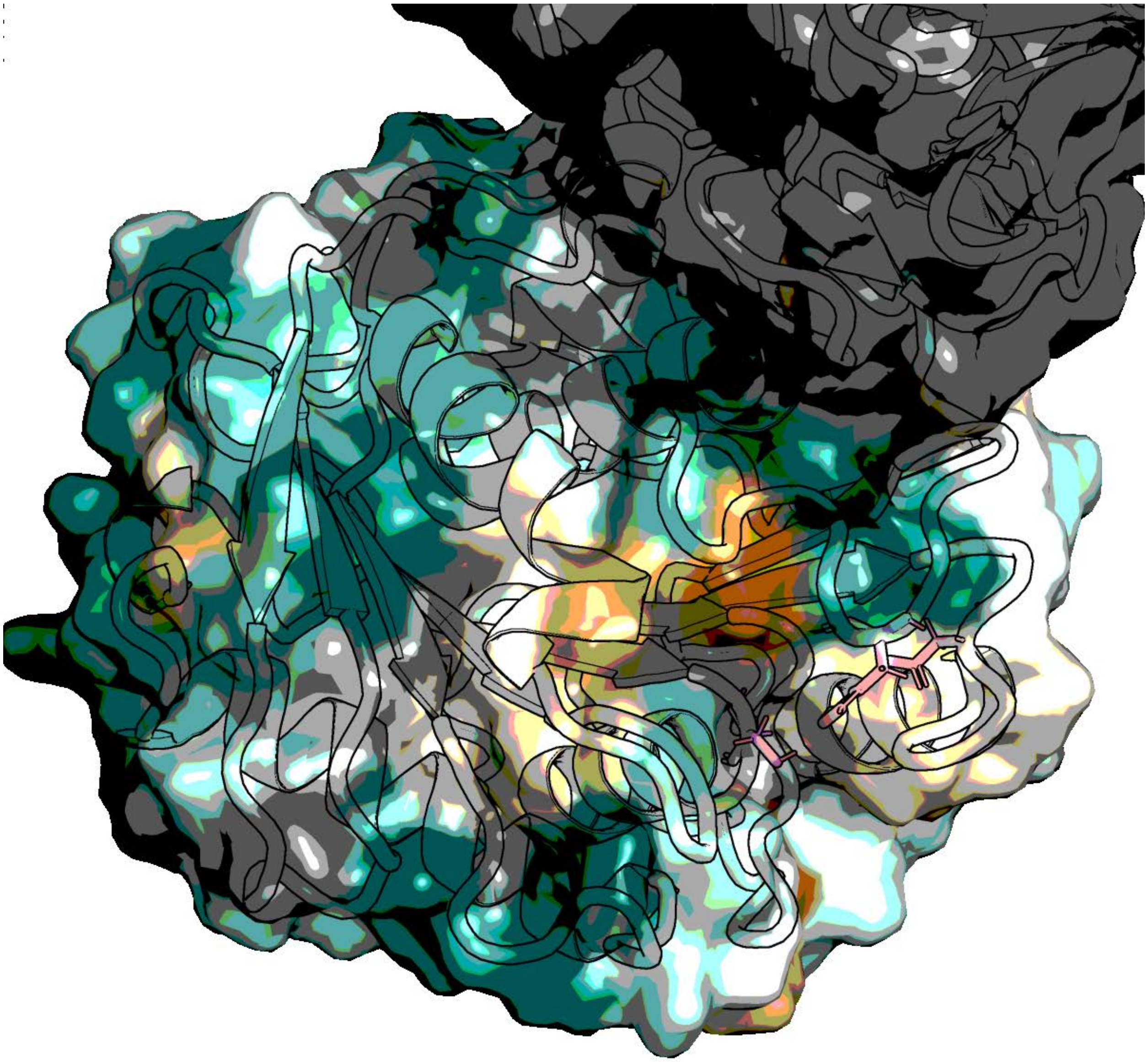

ESM130_highlights.png

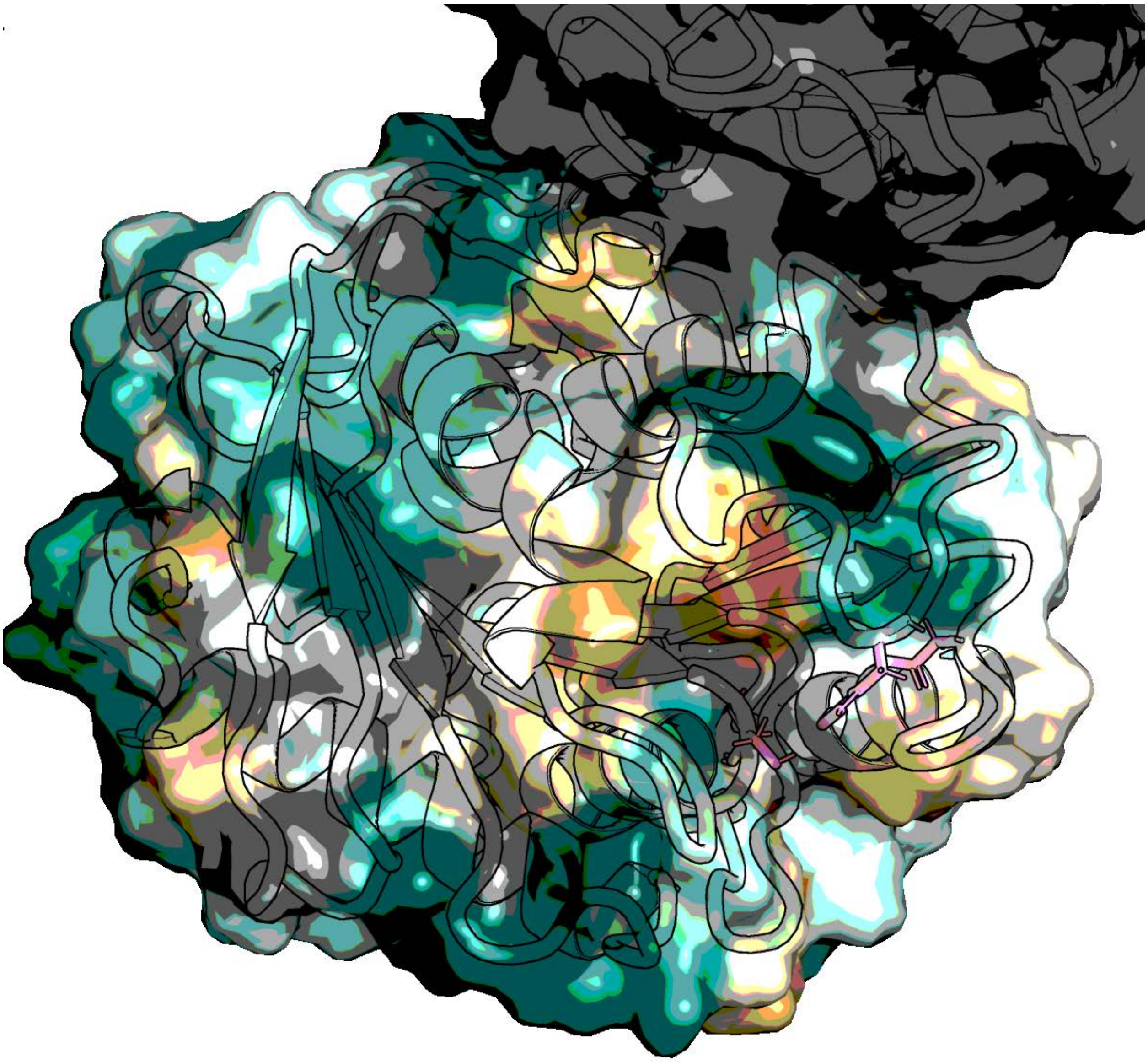

ESM132_highlights.png

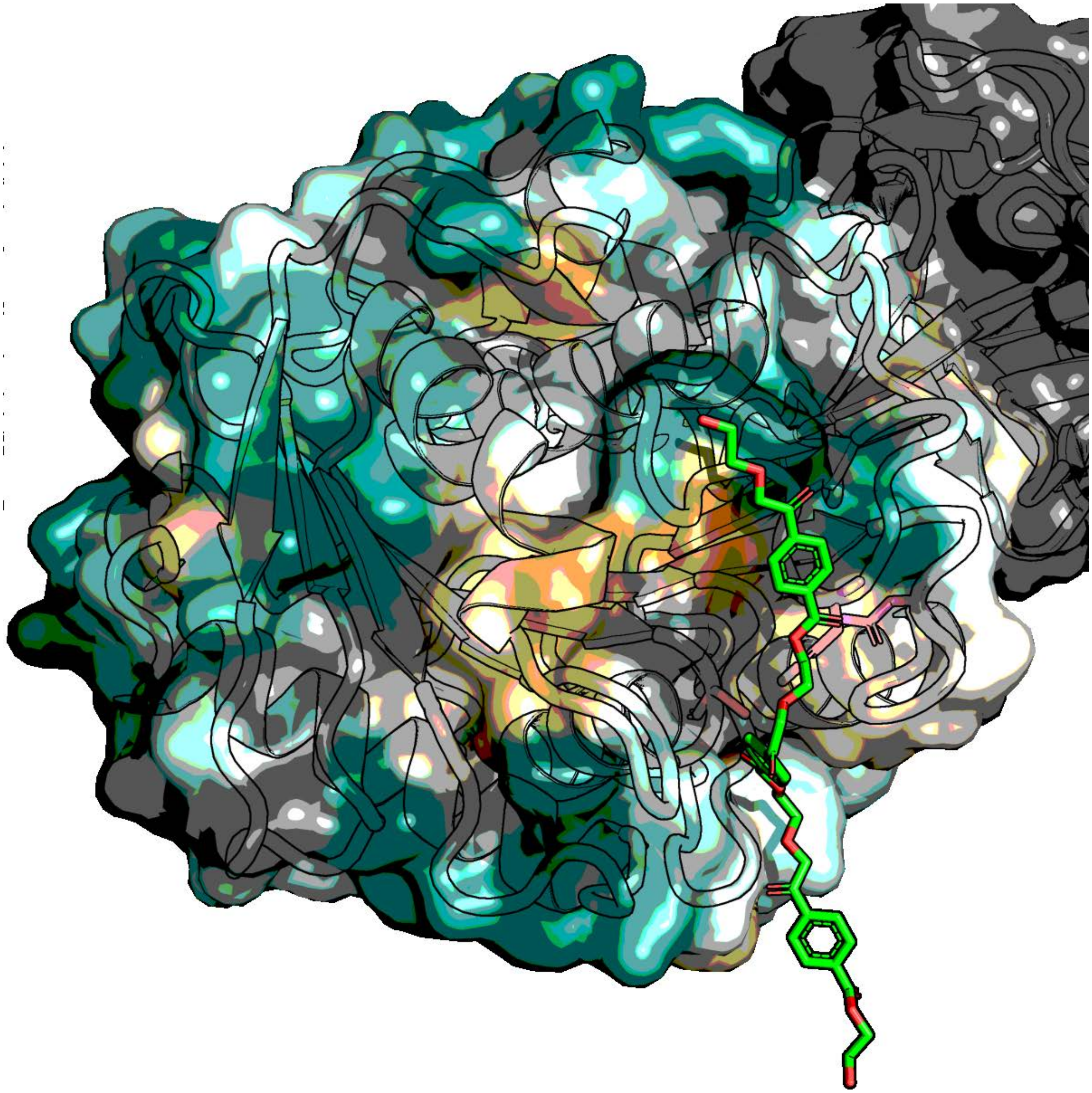

ESM135_highlights.png

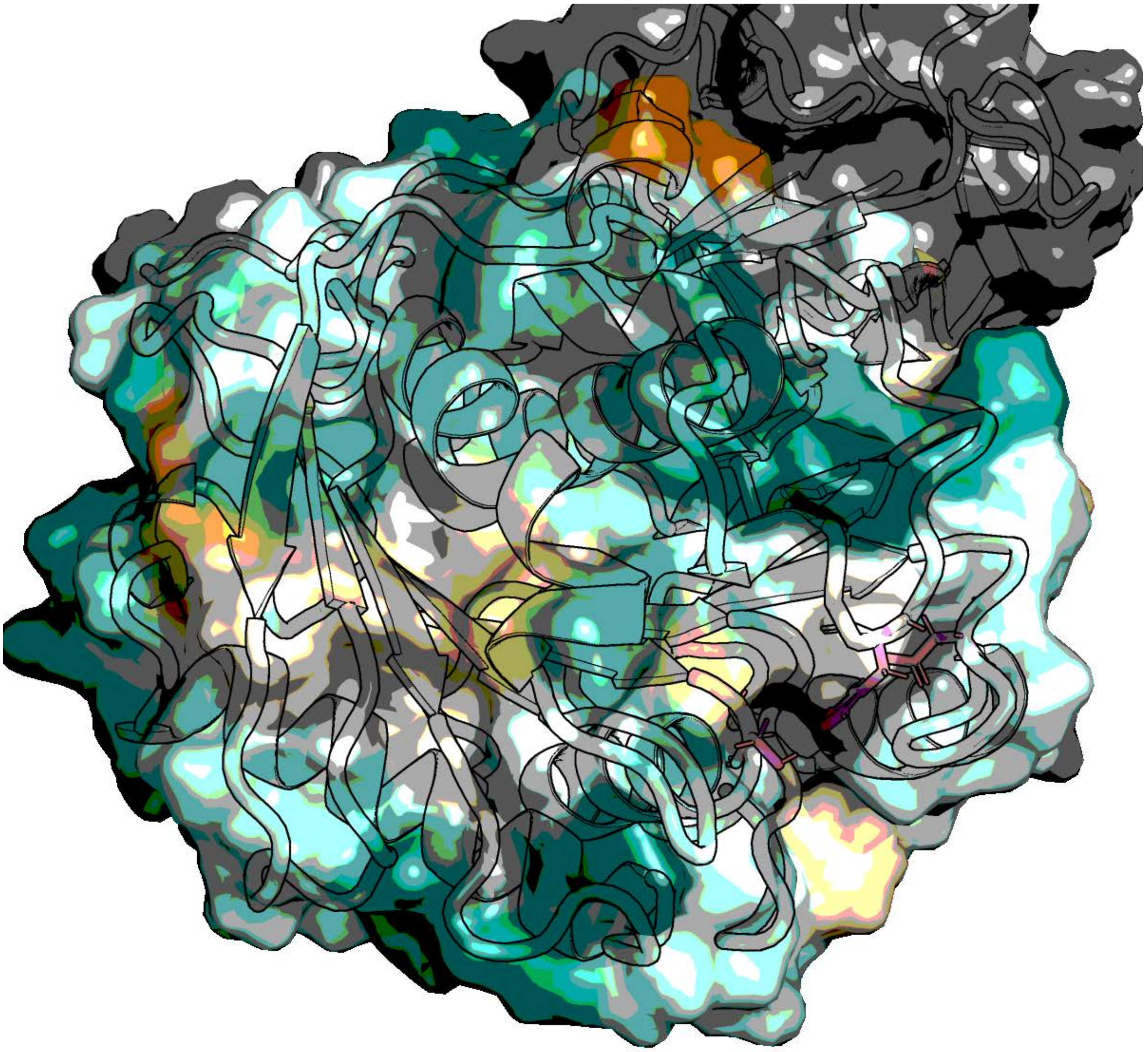

ESM136_highlights.png

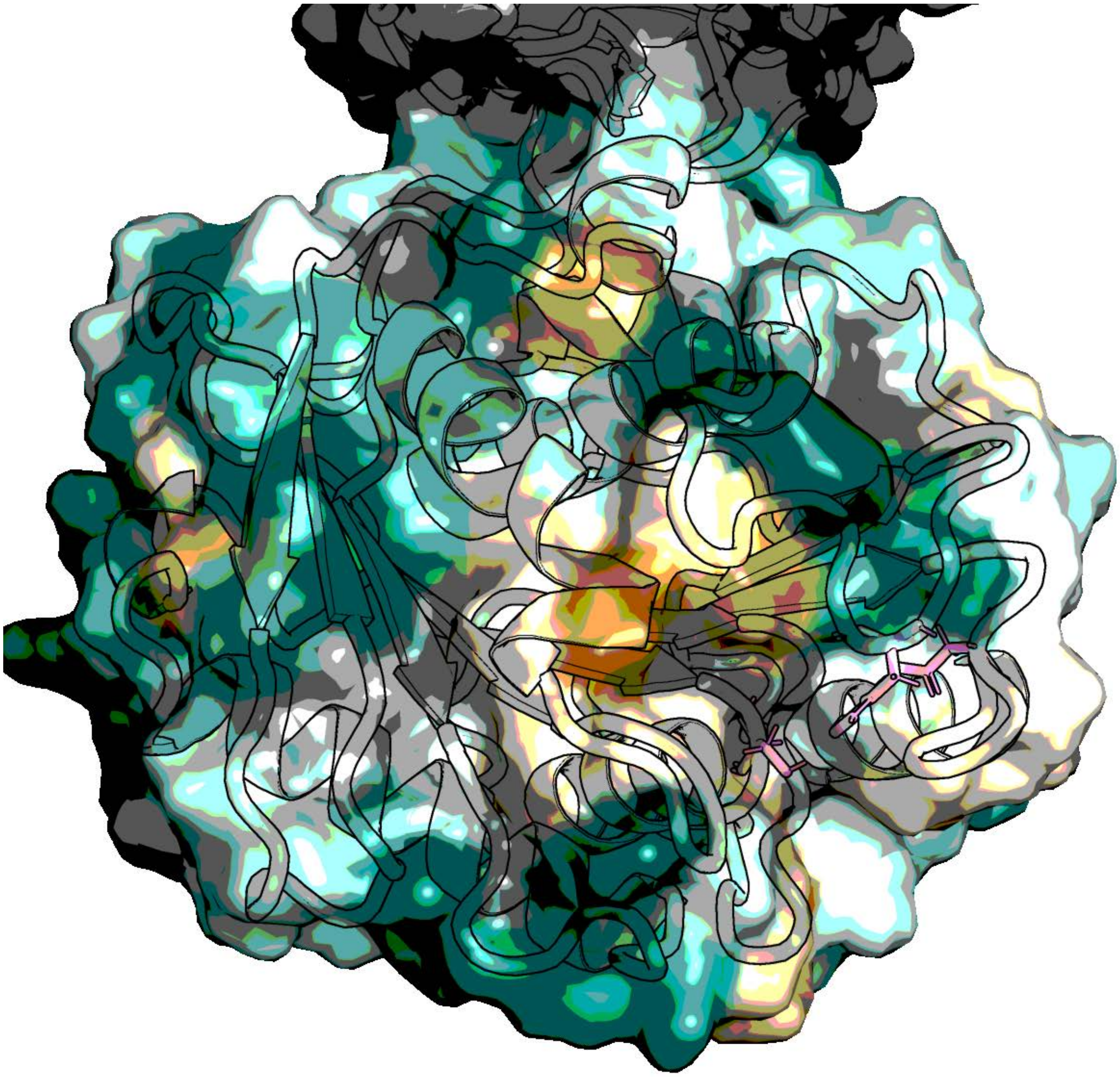

ESM137_highlights.png

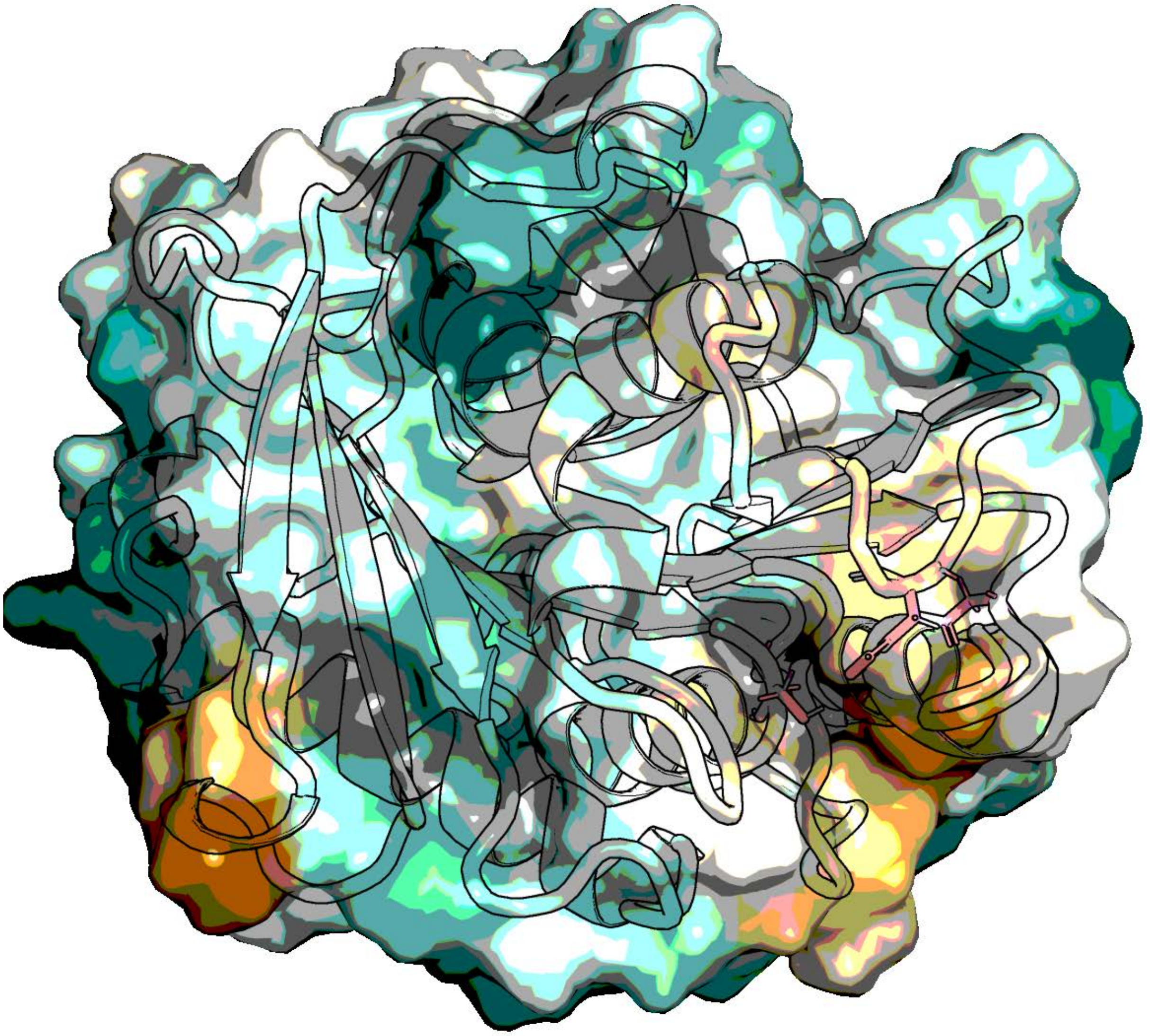

ESM140_highlights.png

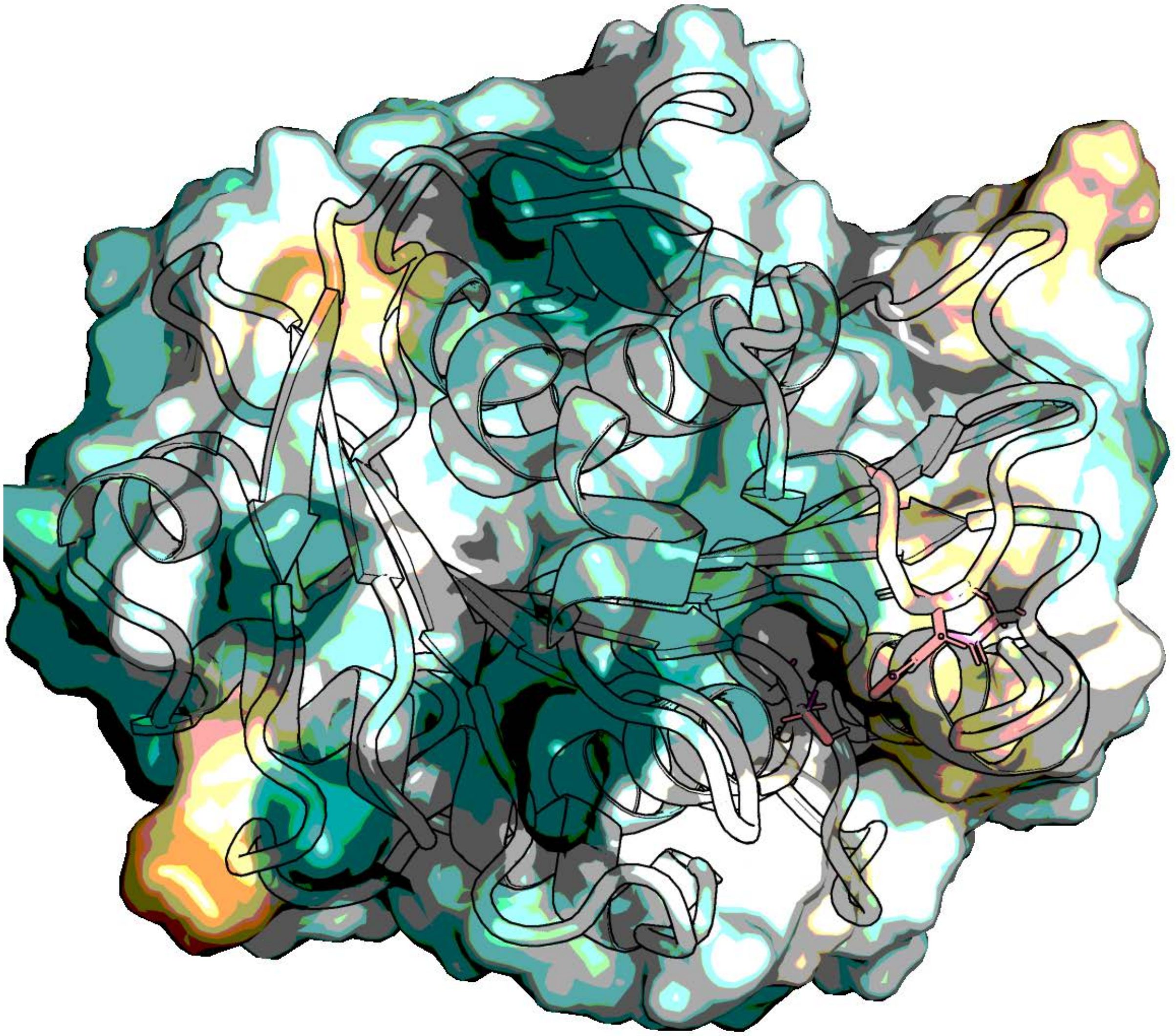

ESM144_highlights.png

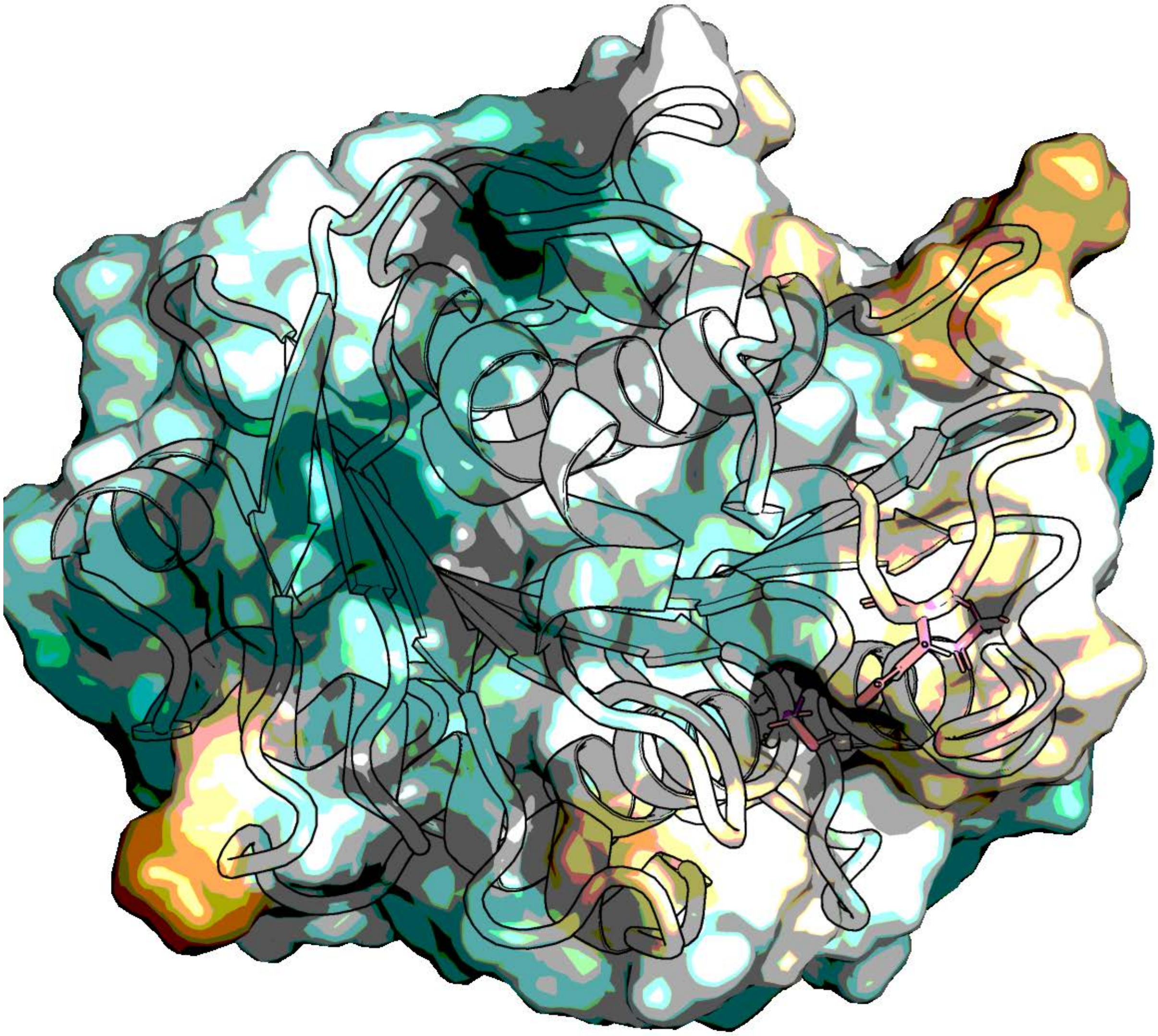

ESM147_highlights.png

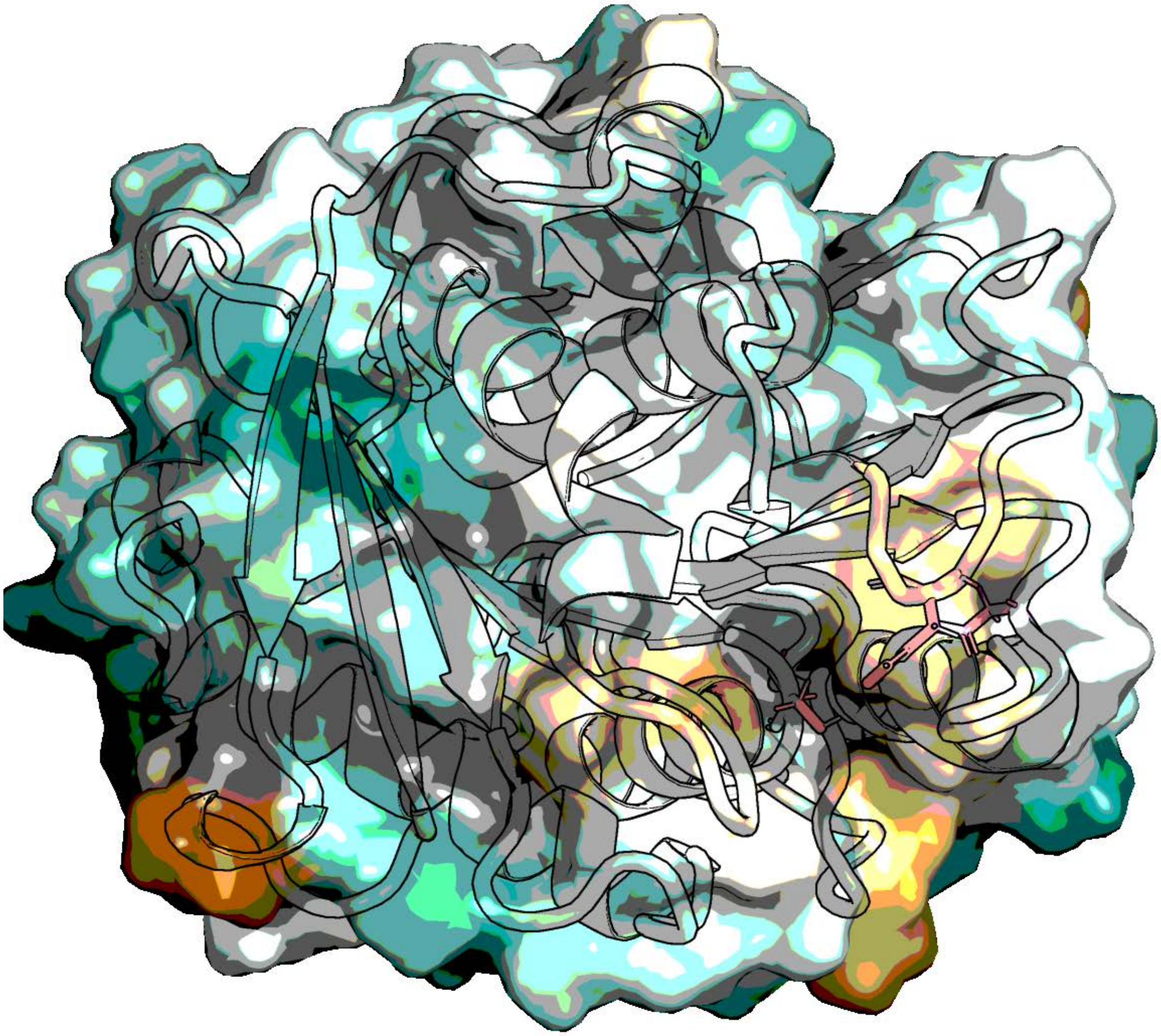

ESM156_highlights.png

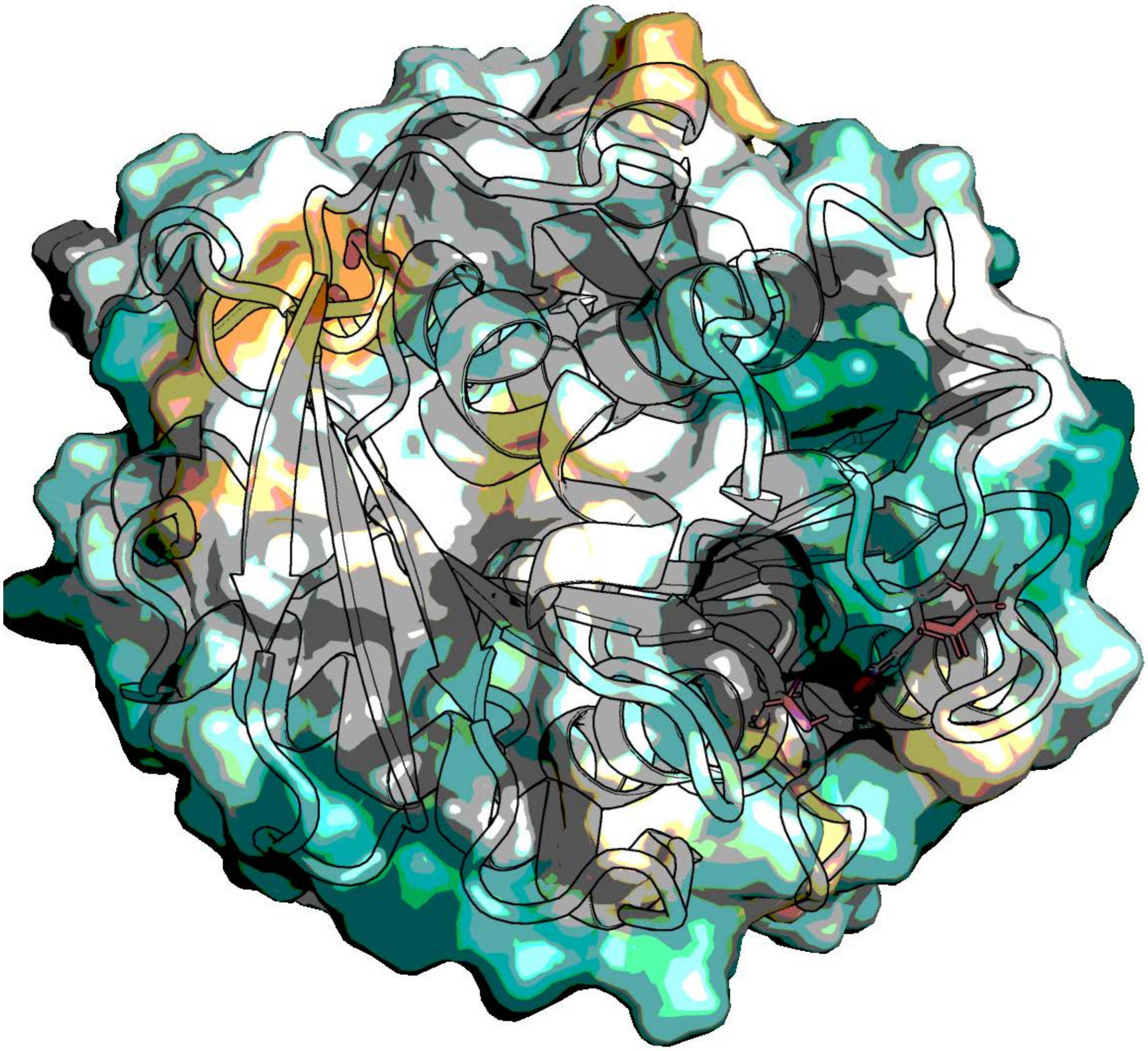

ESM157_highlights.png

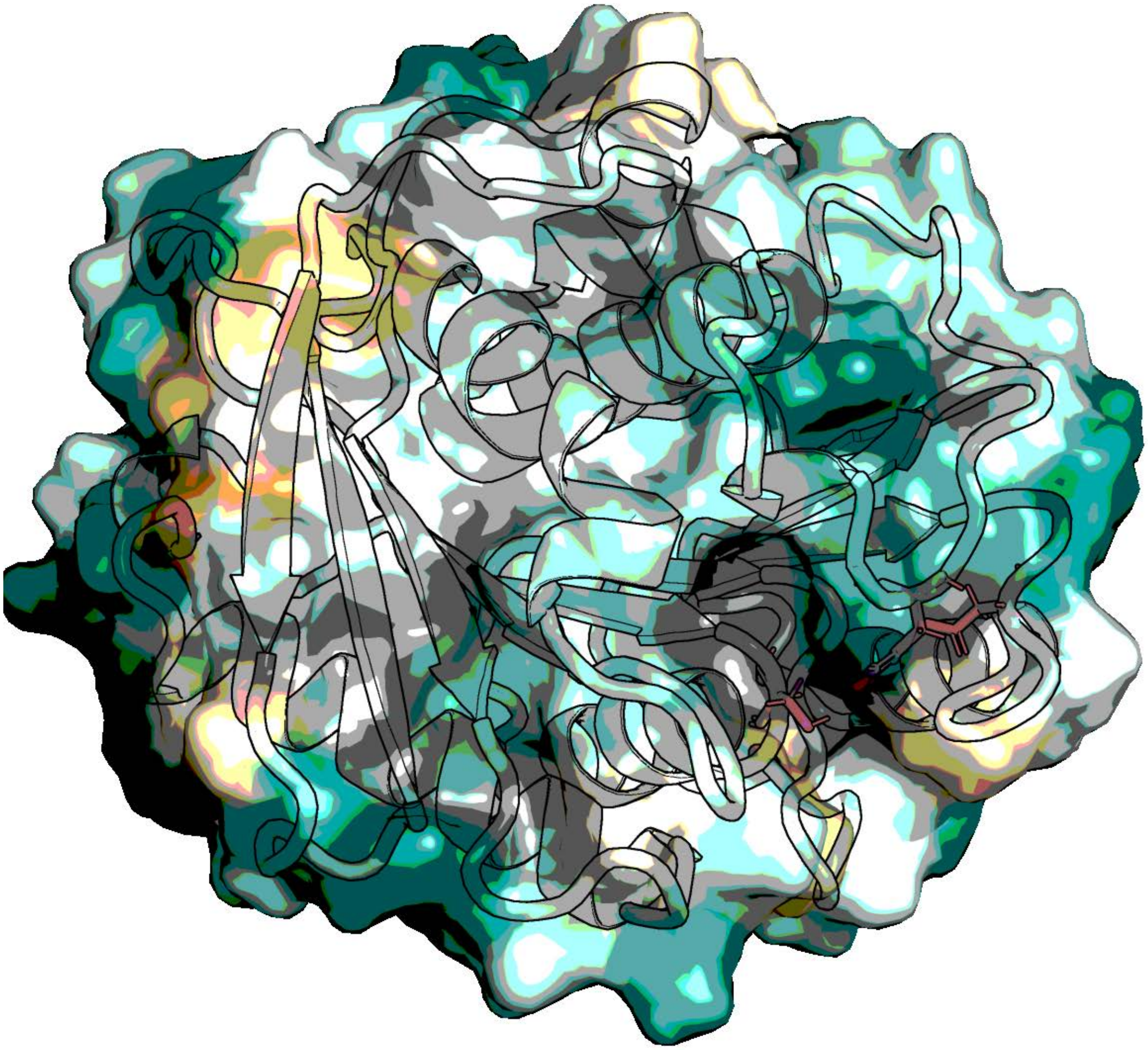

ESM159_highlights.png

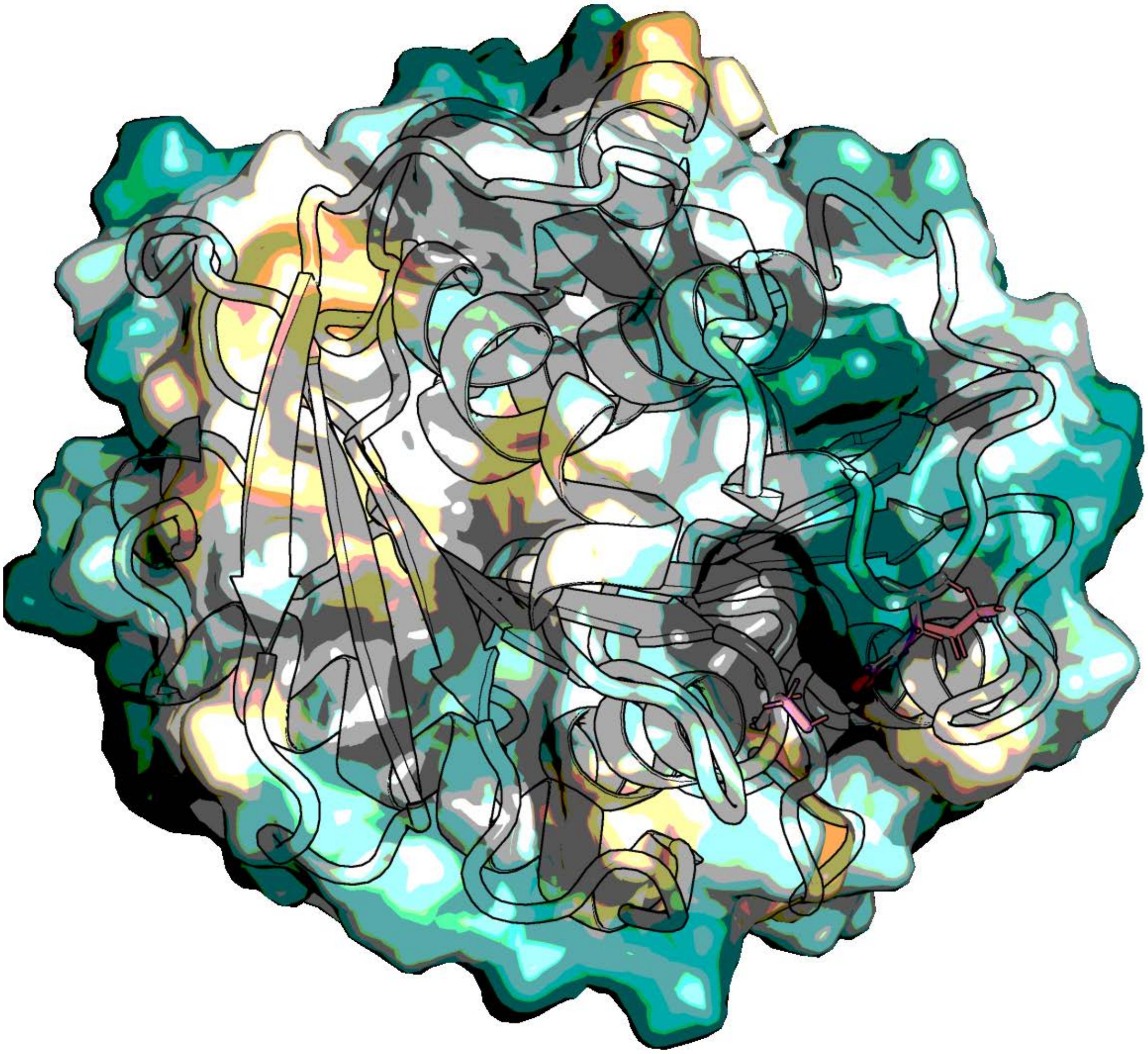

ESM160_highlights.png

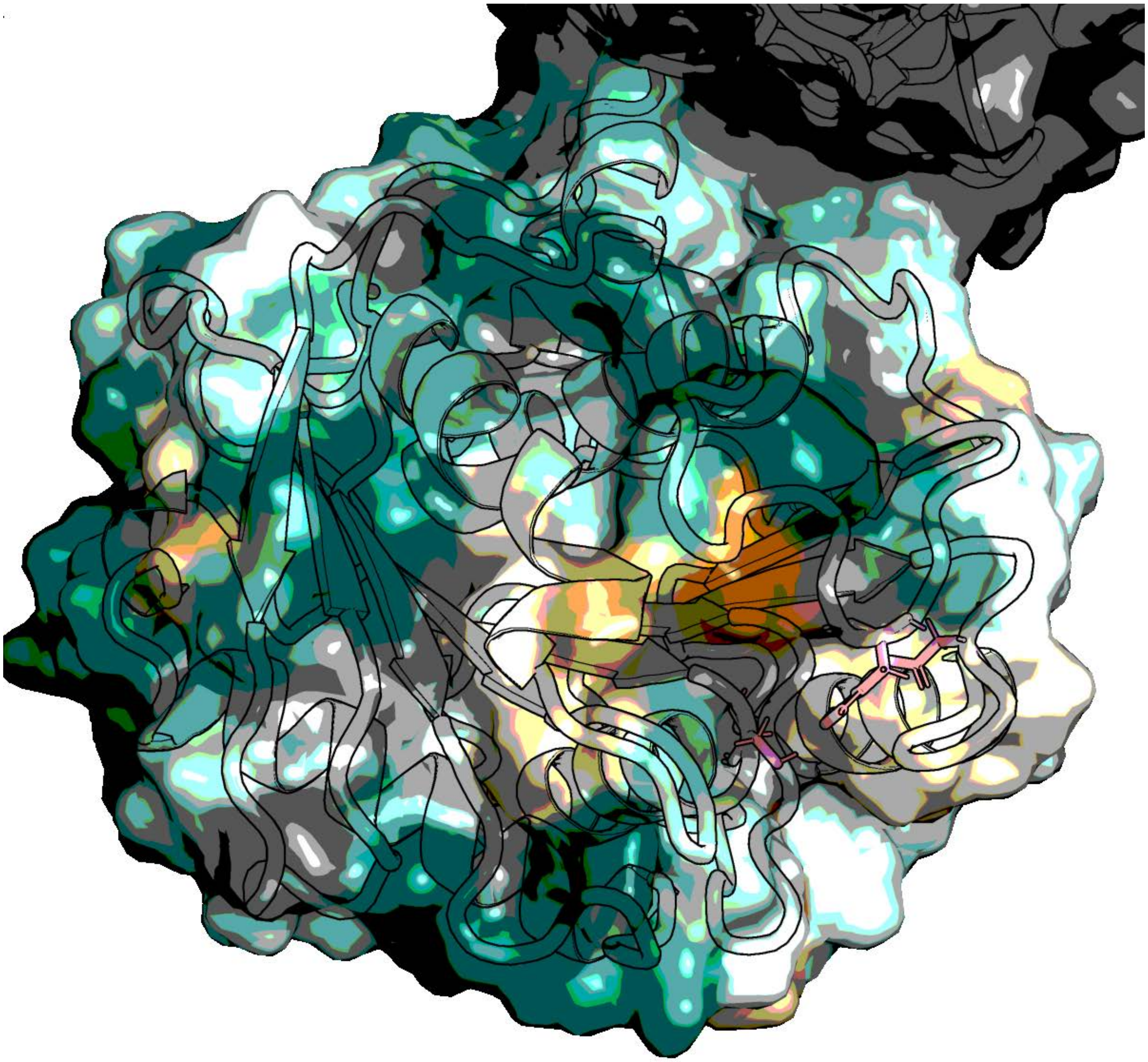

ESM161_highlights.png

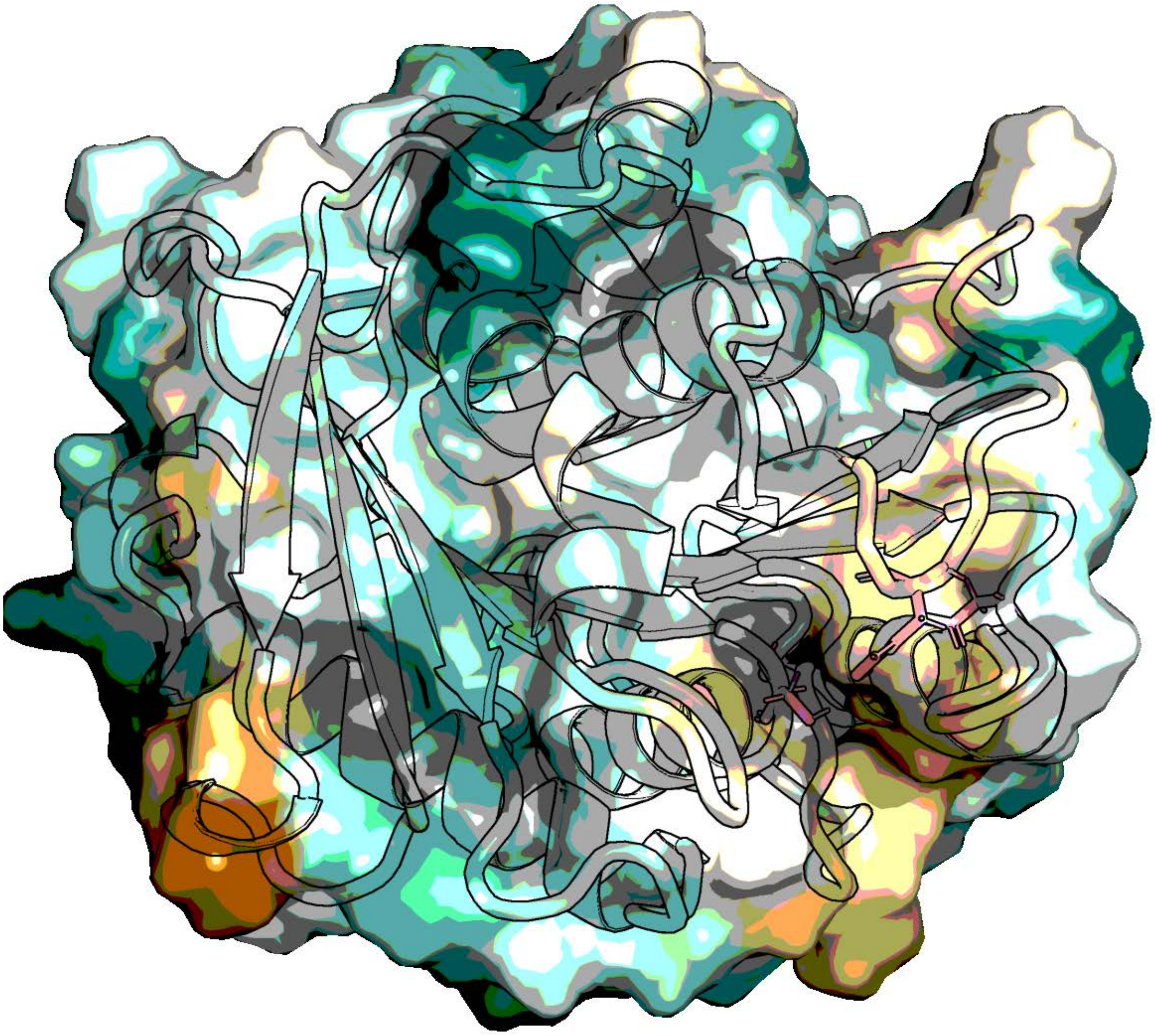

ESM167_highlights.png

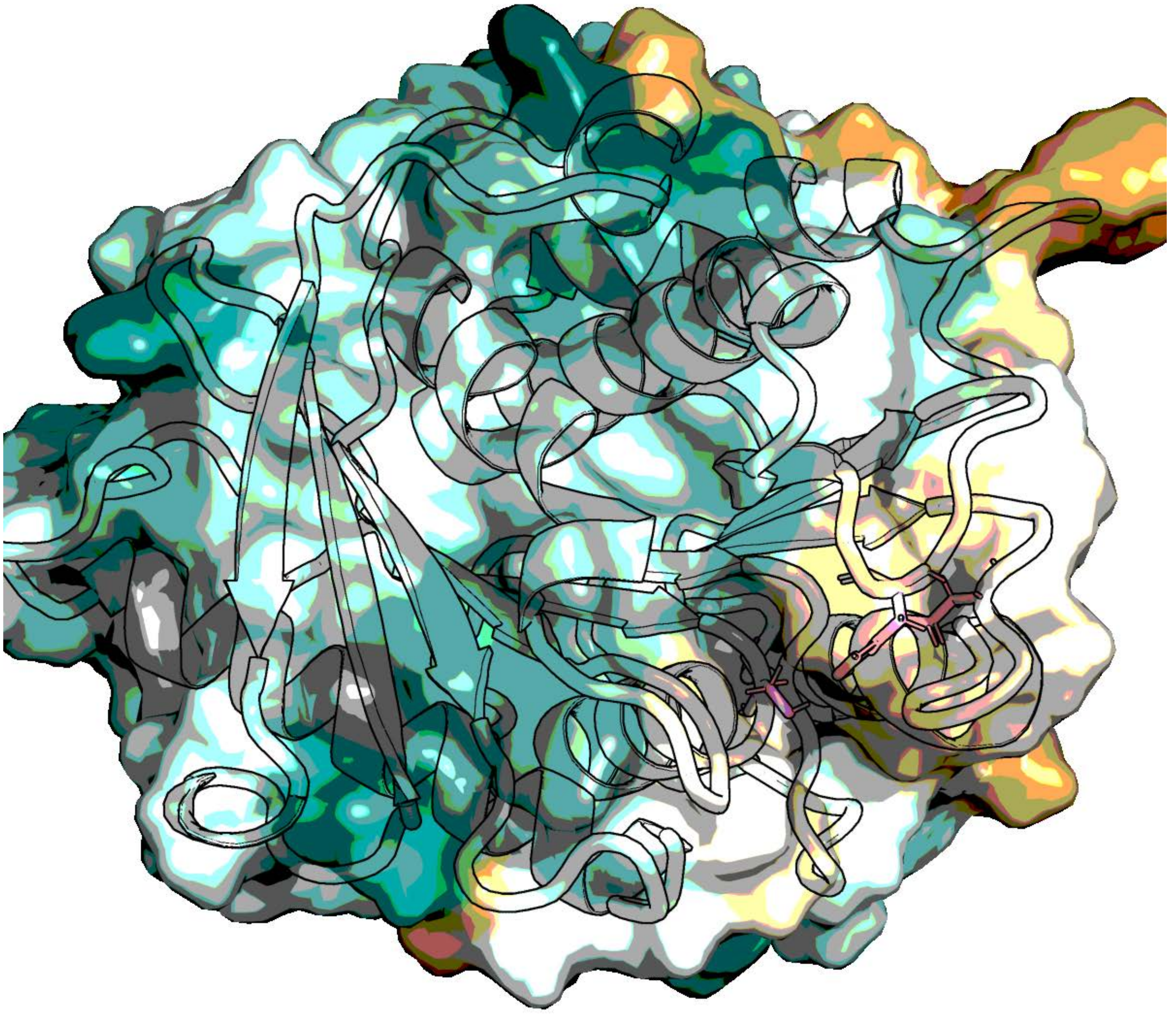

ESM169_highlights.png

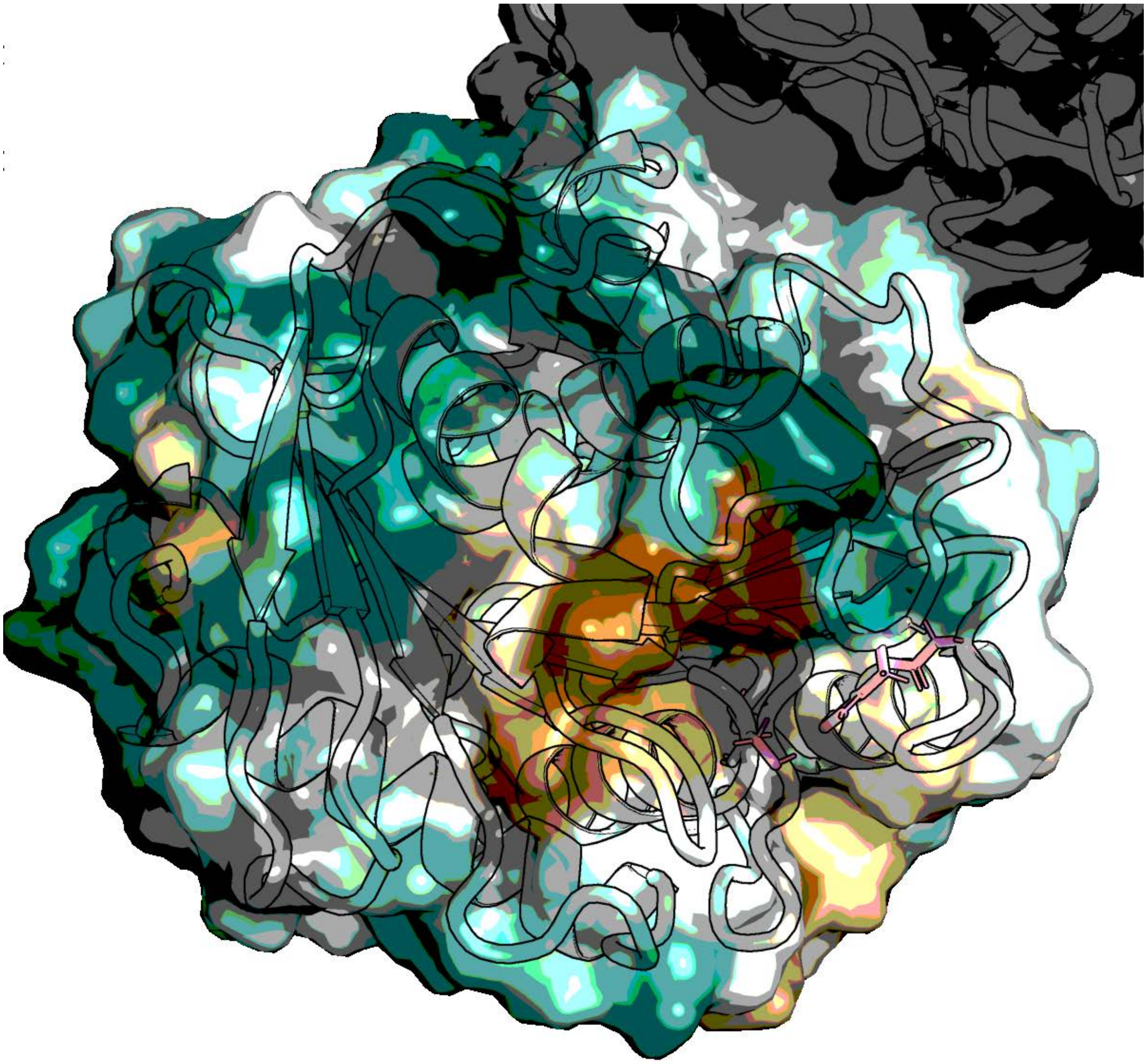

ESM173_highlights.png

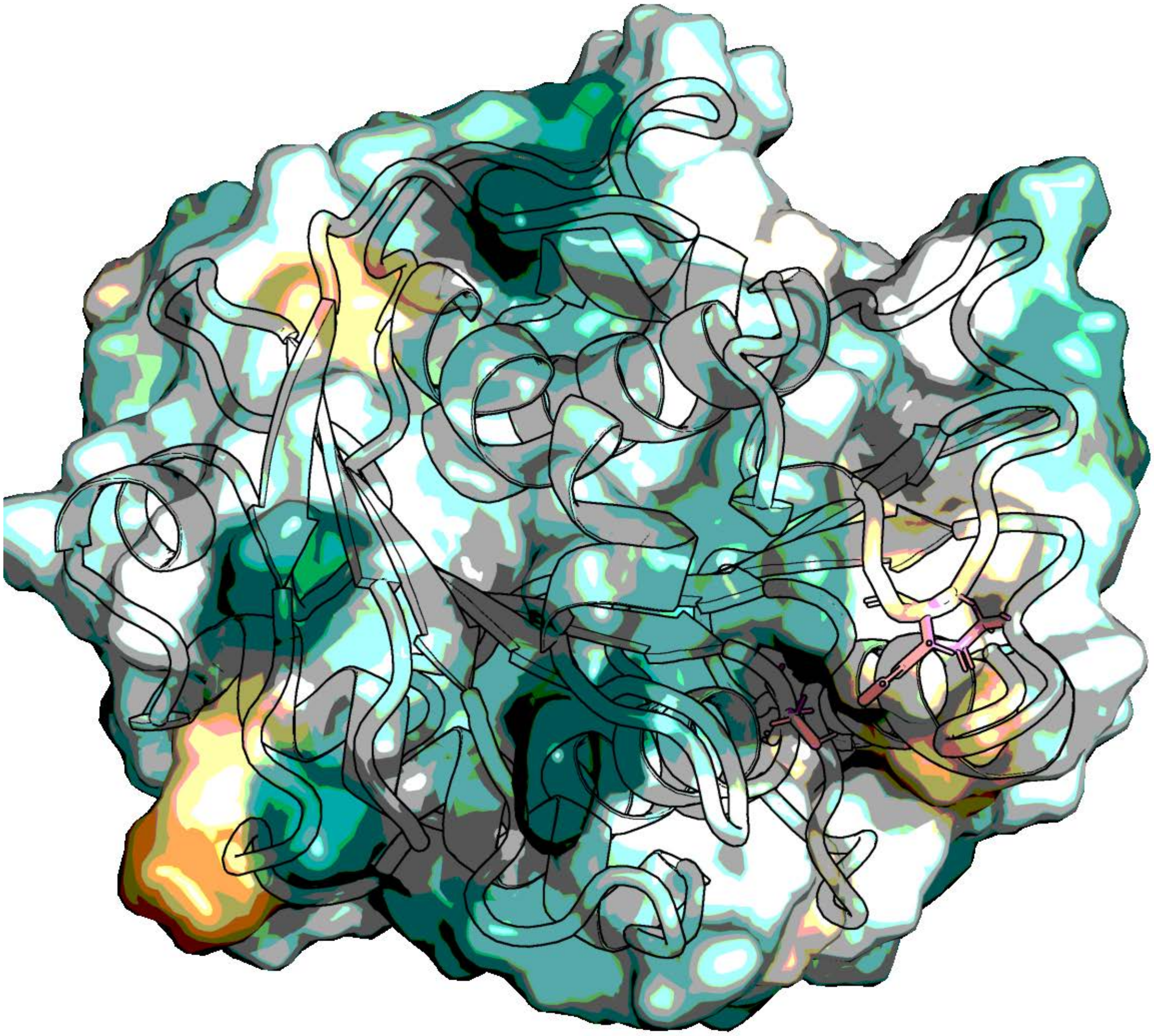

ESM177_highlights.png

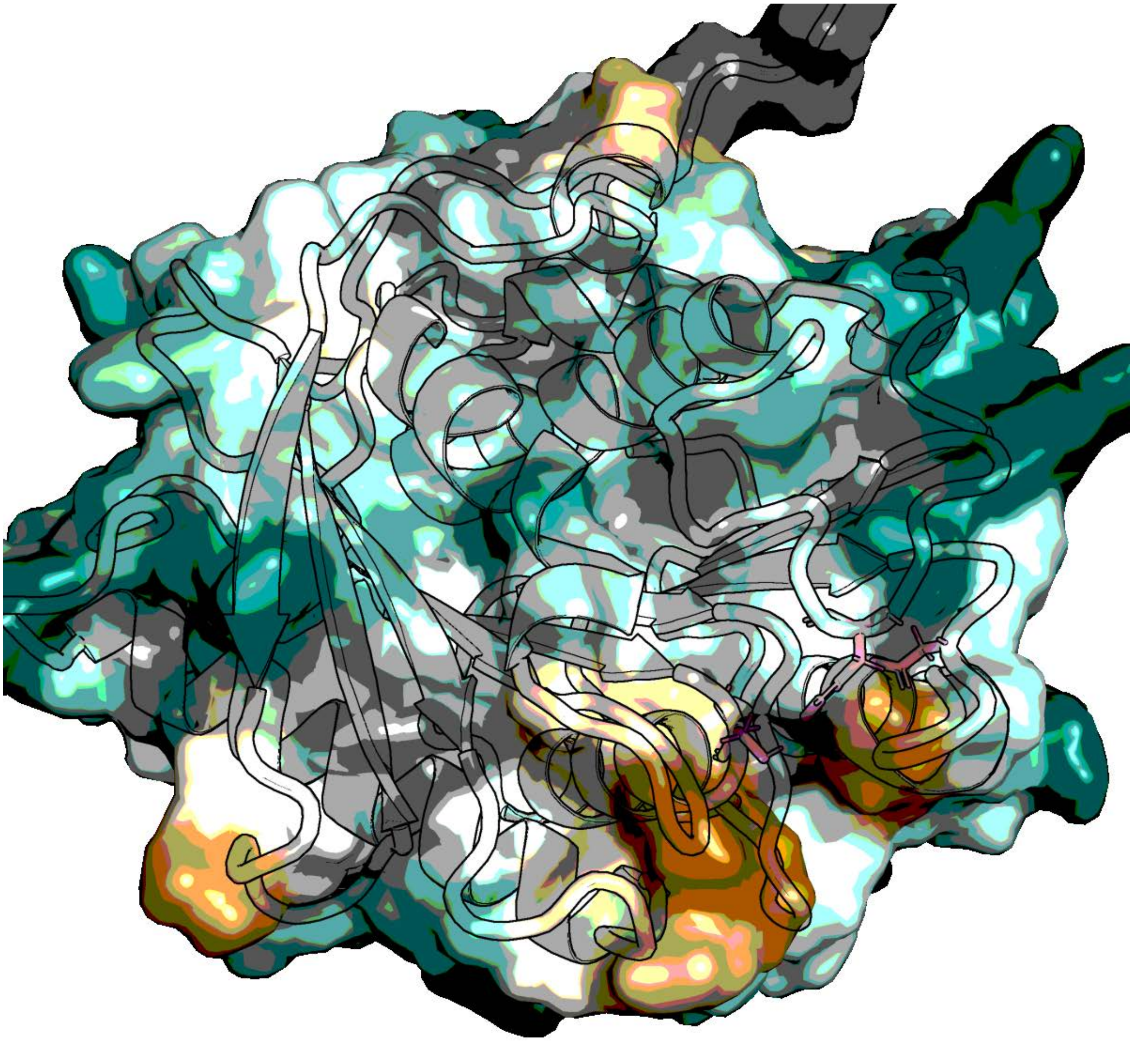

ESM186_highlights.png

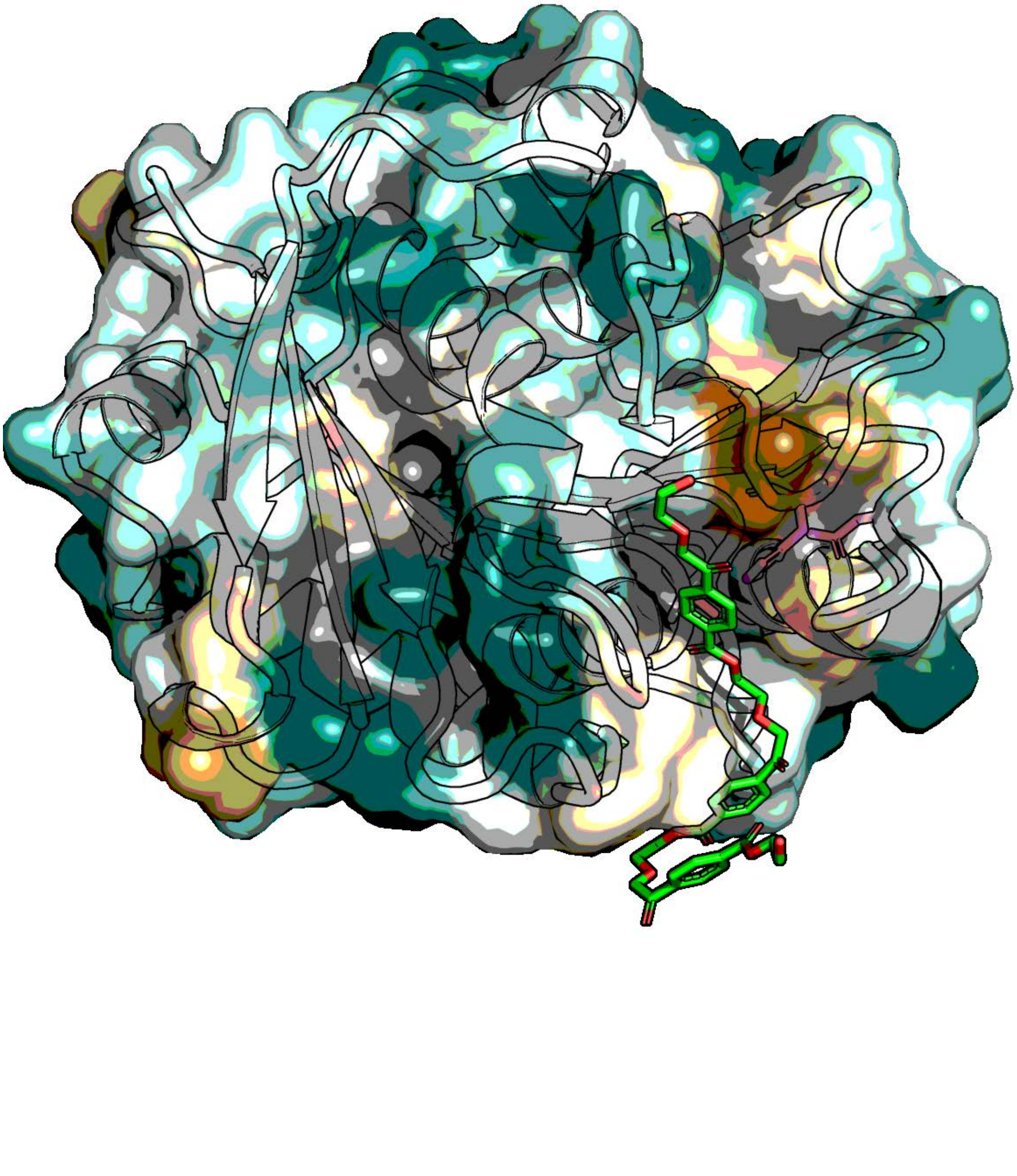

LCC-ICCG_highlights.png

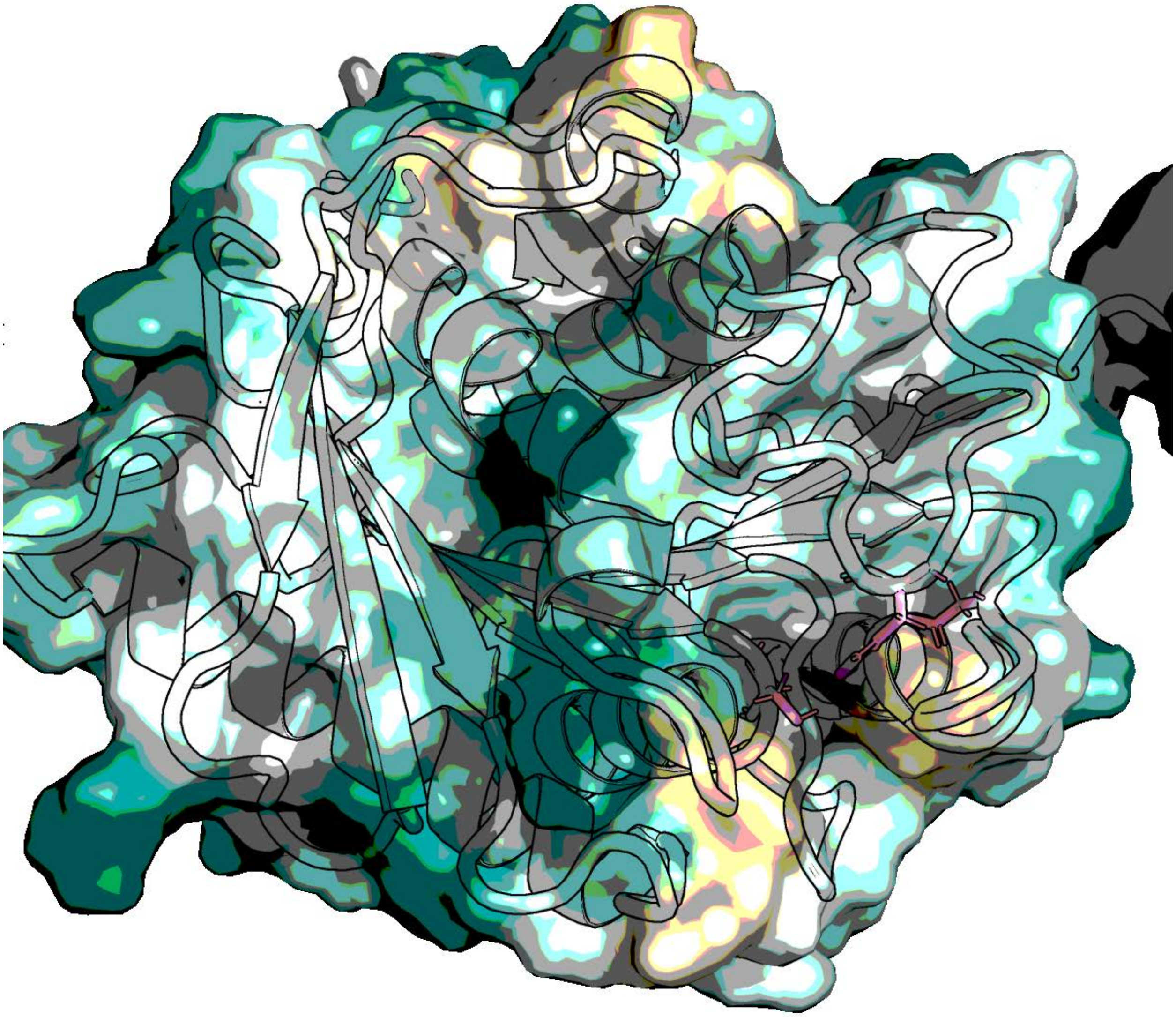

TEP004_highlights.png

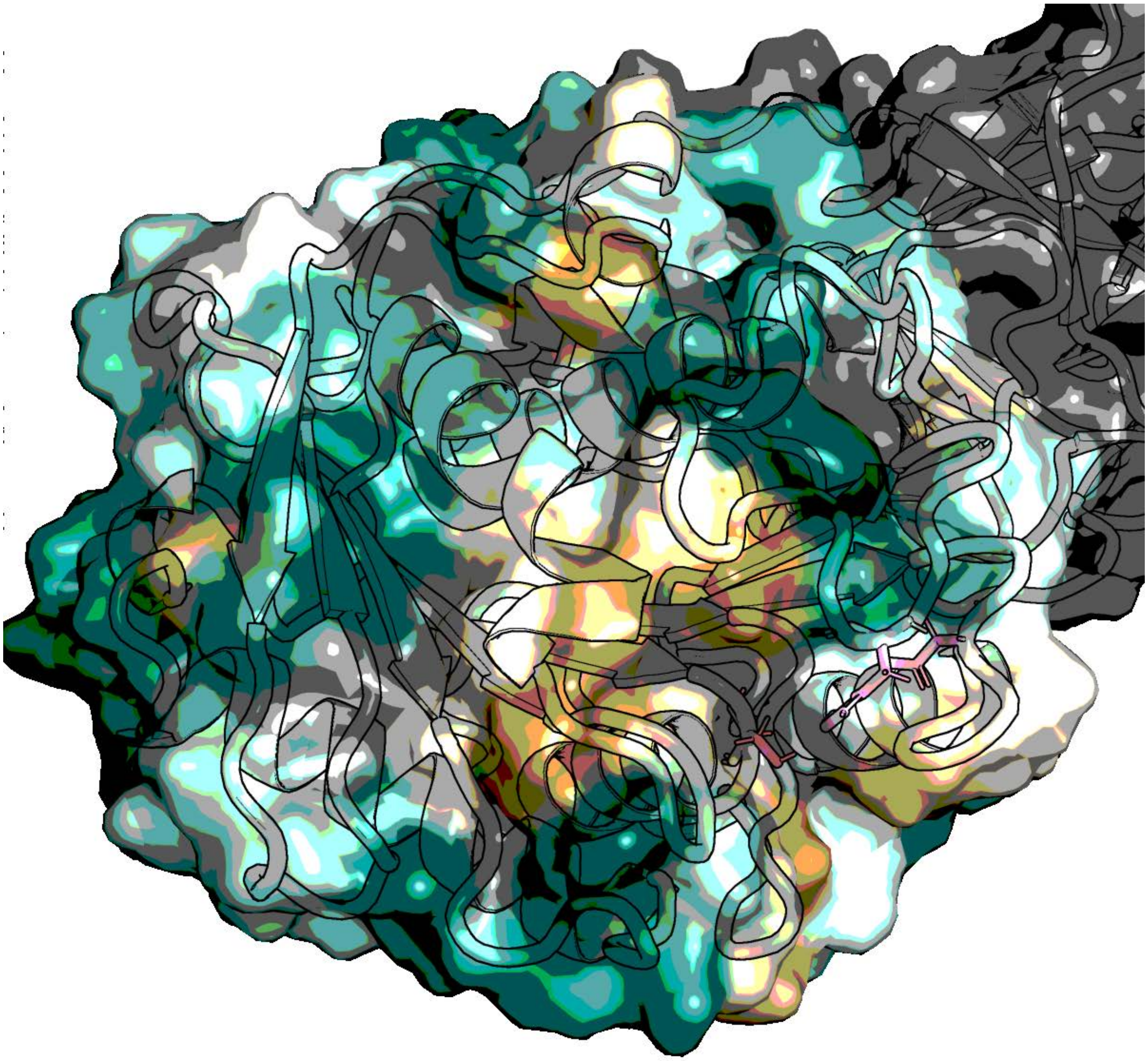

TEP010_highlights.png

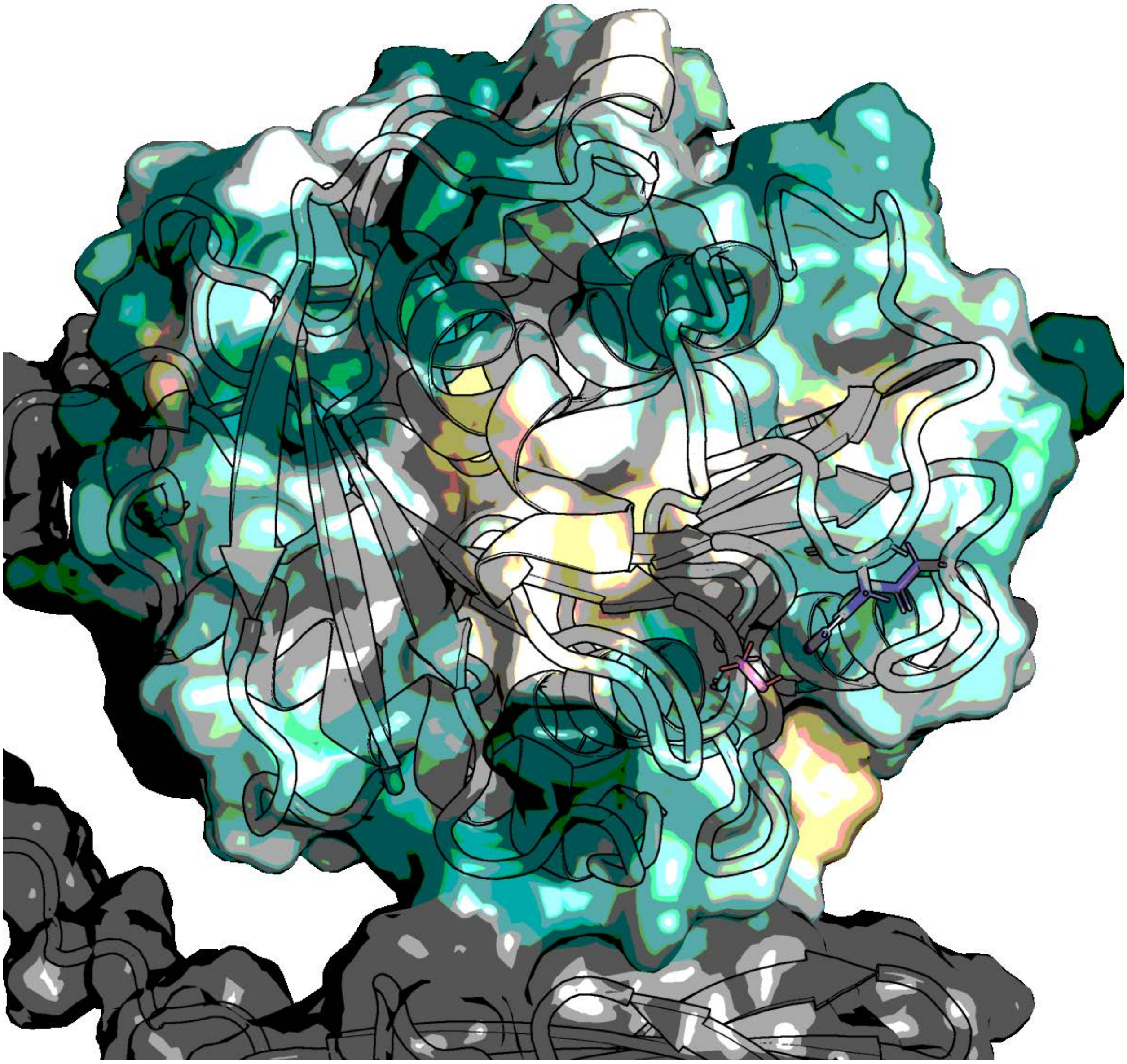

TEP013_highlights.png

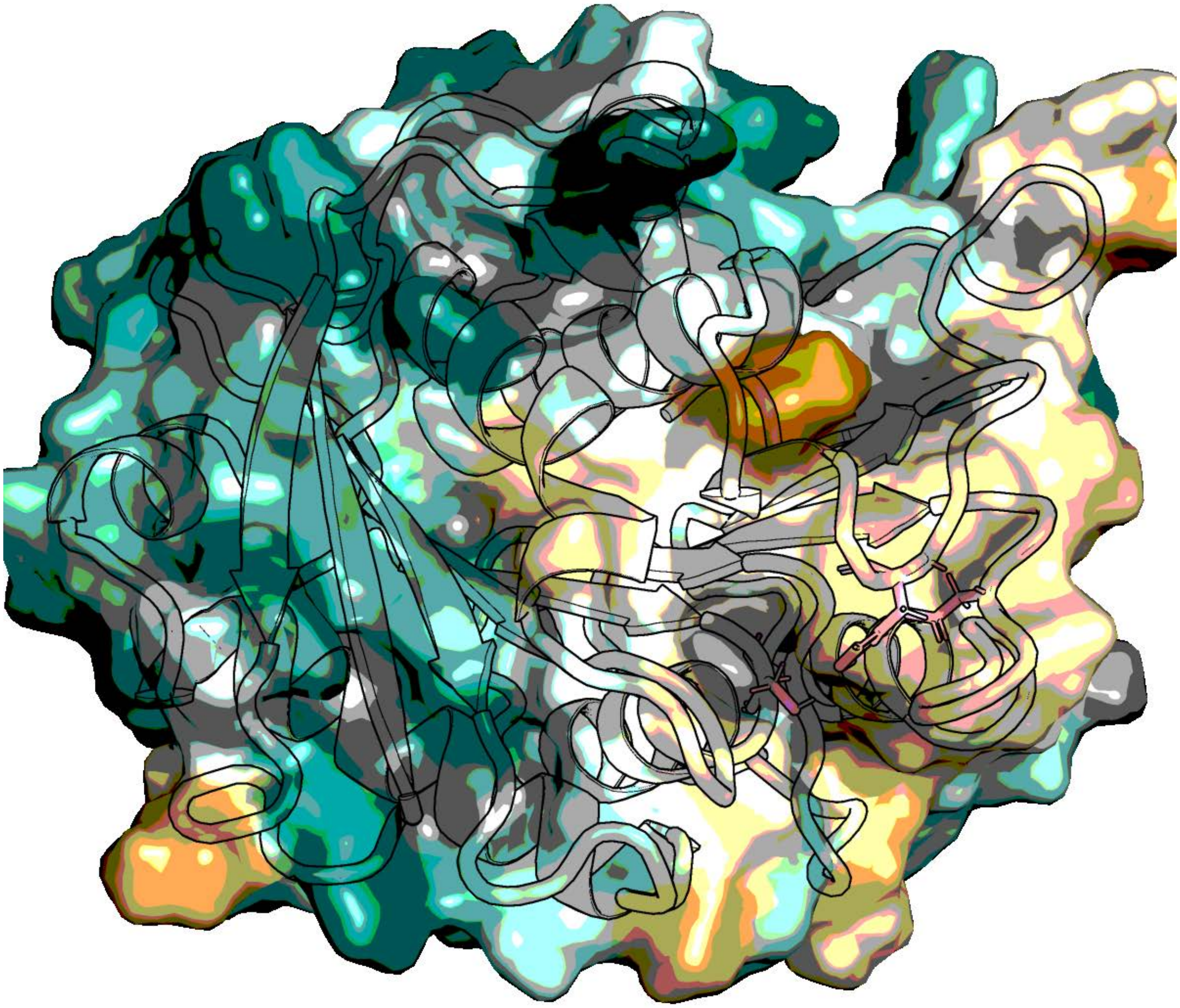

TEP014_highlights.png

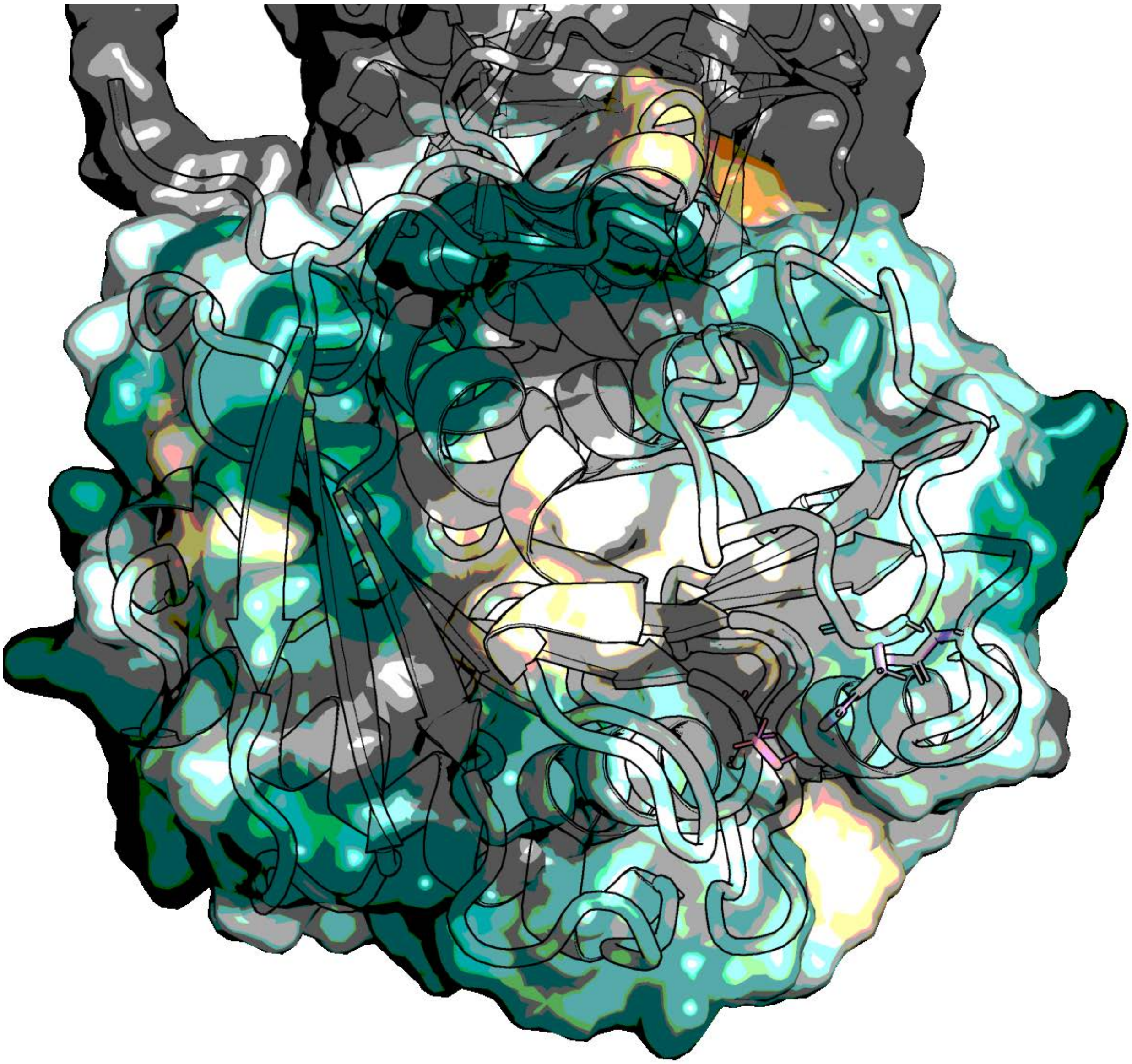

TEP024_highlights.png

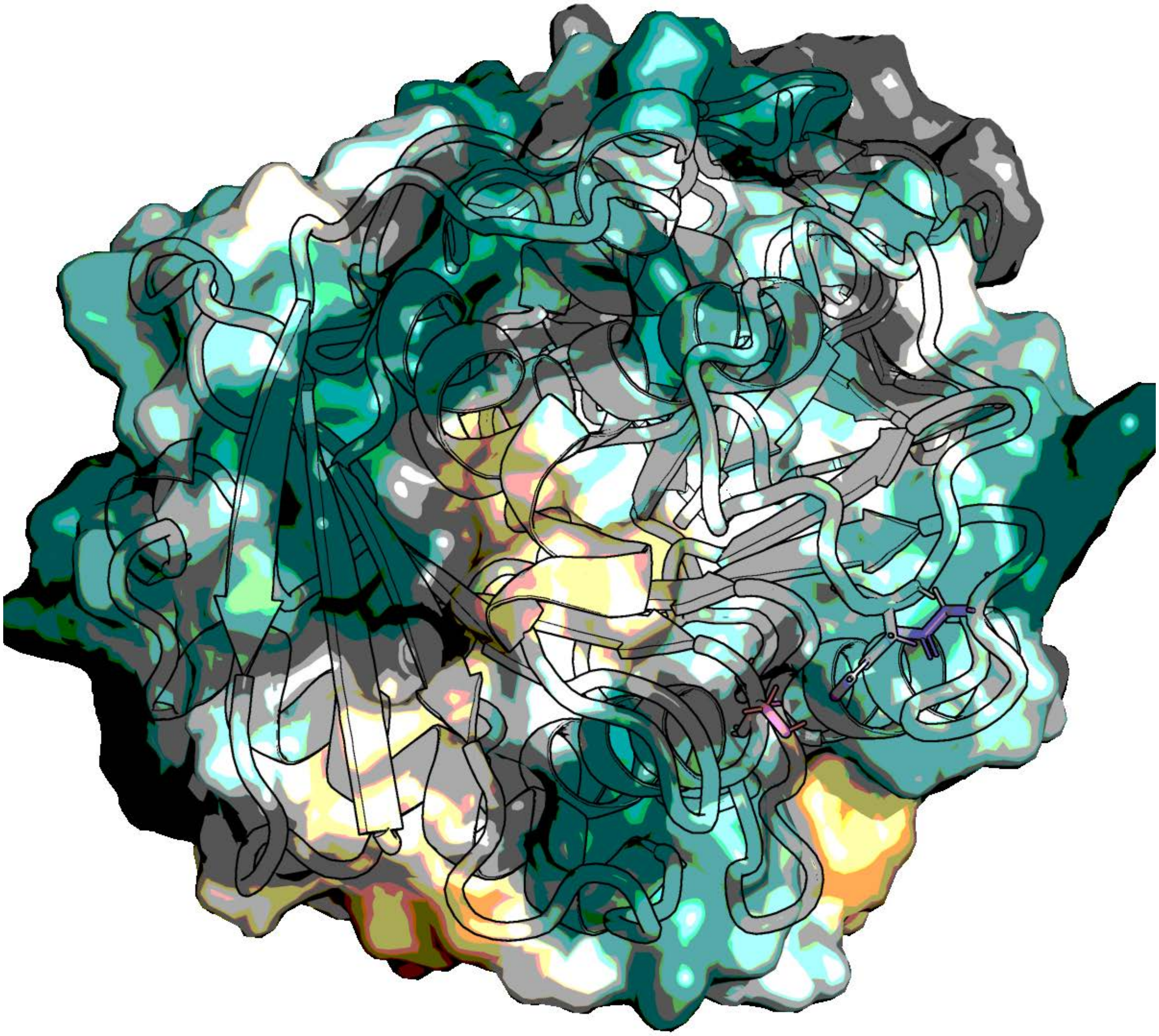

TEP029_highlights.png

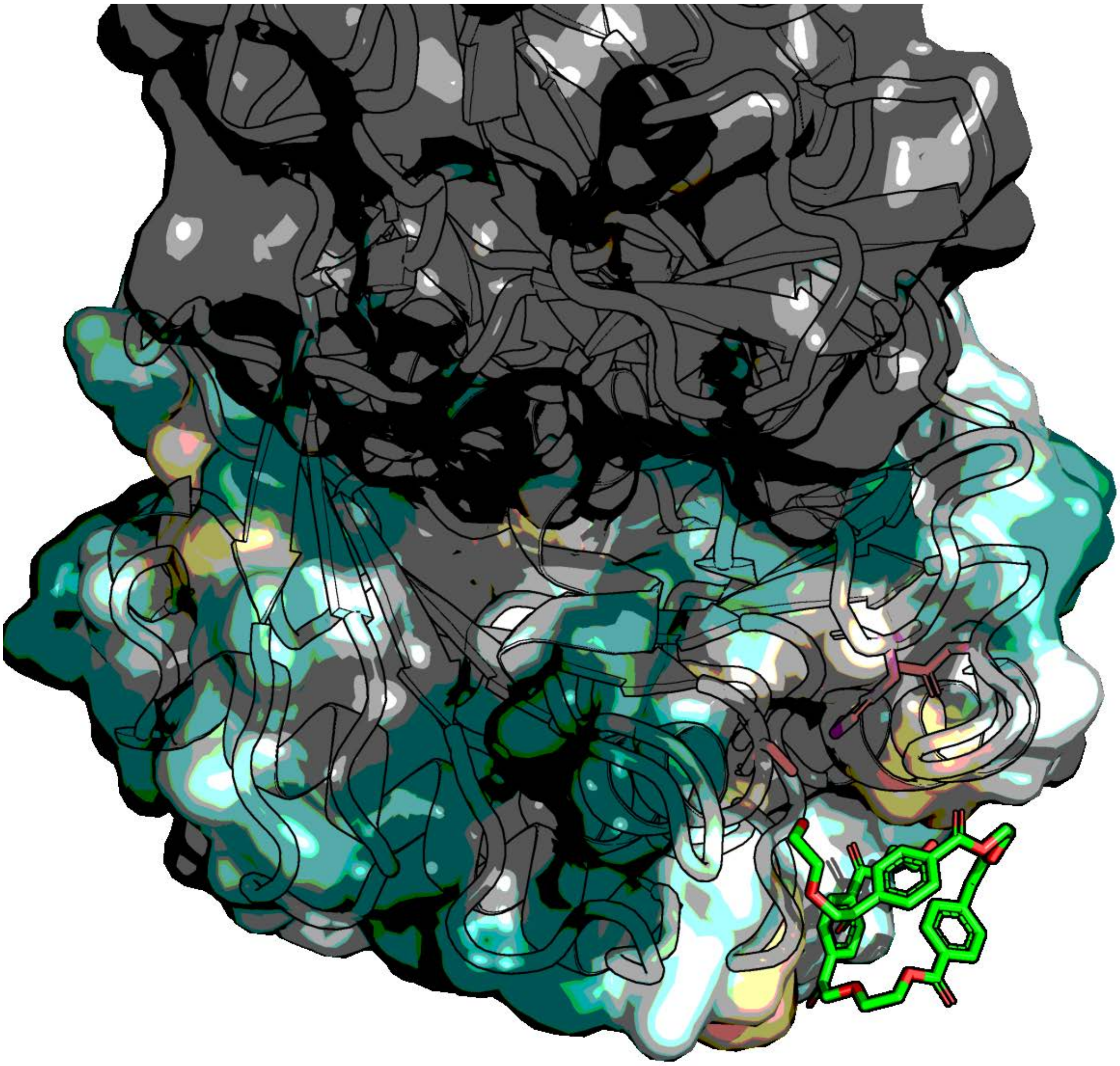

TEP054_highlights.png

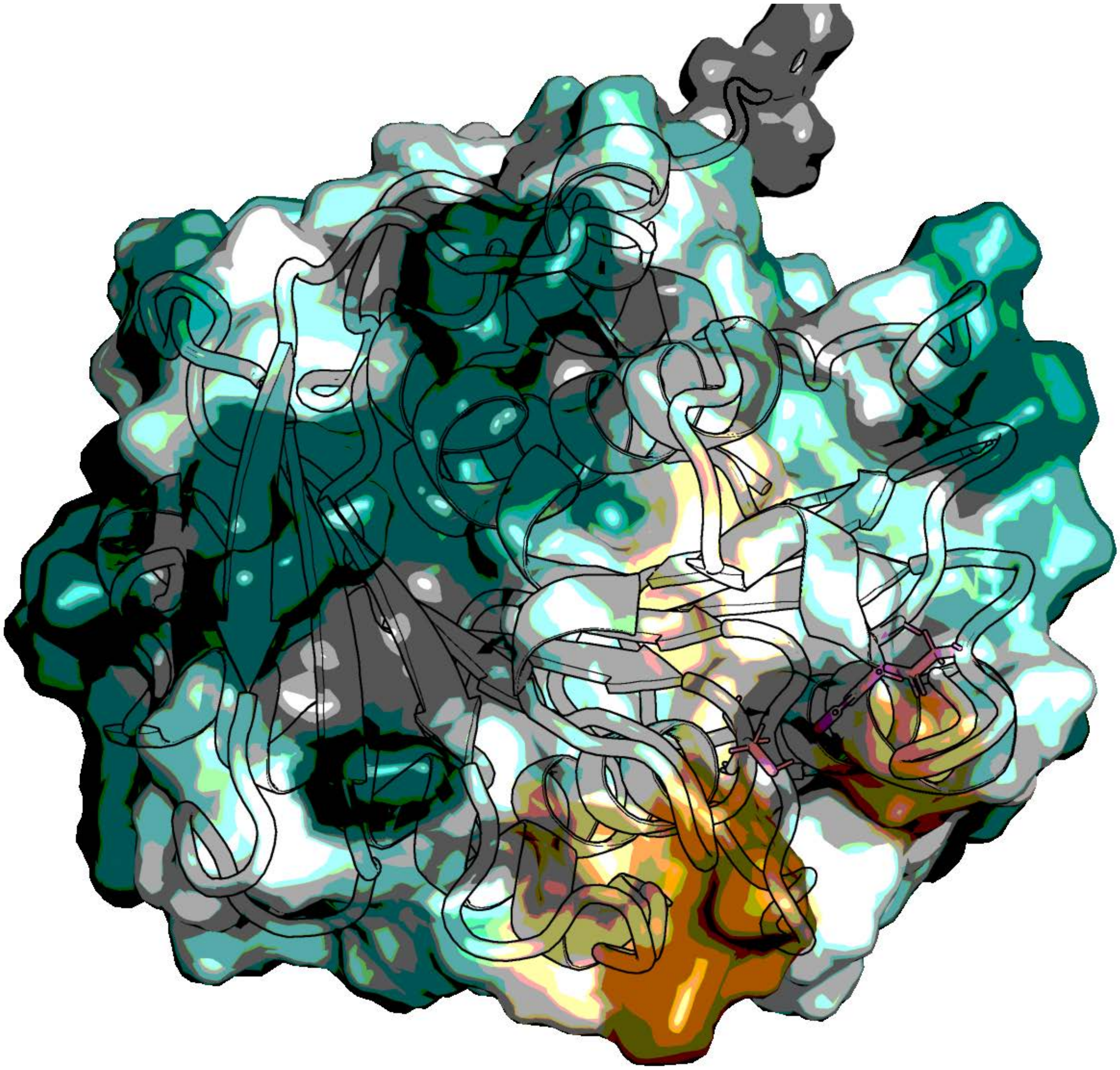

TEP067_highlights.png

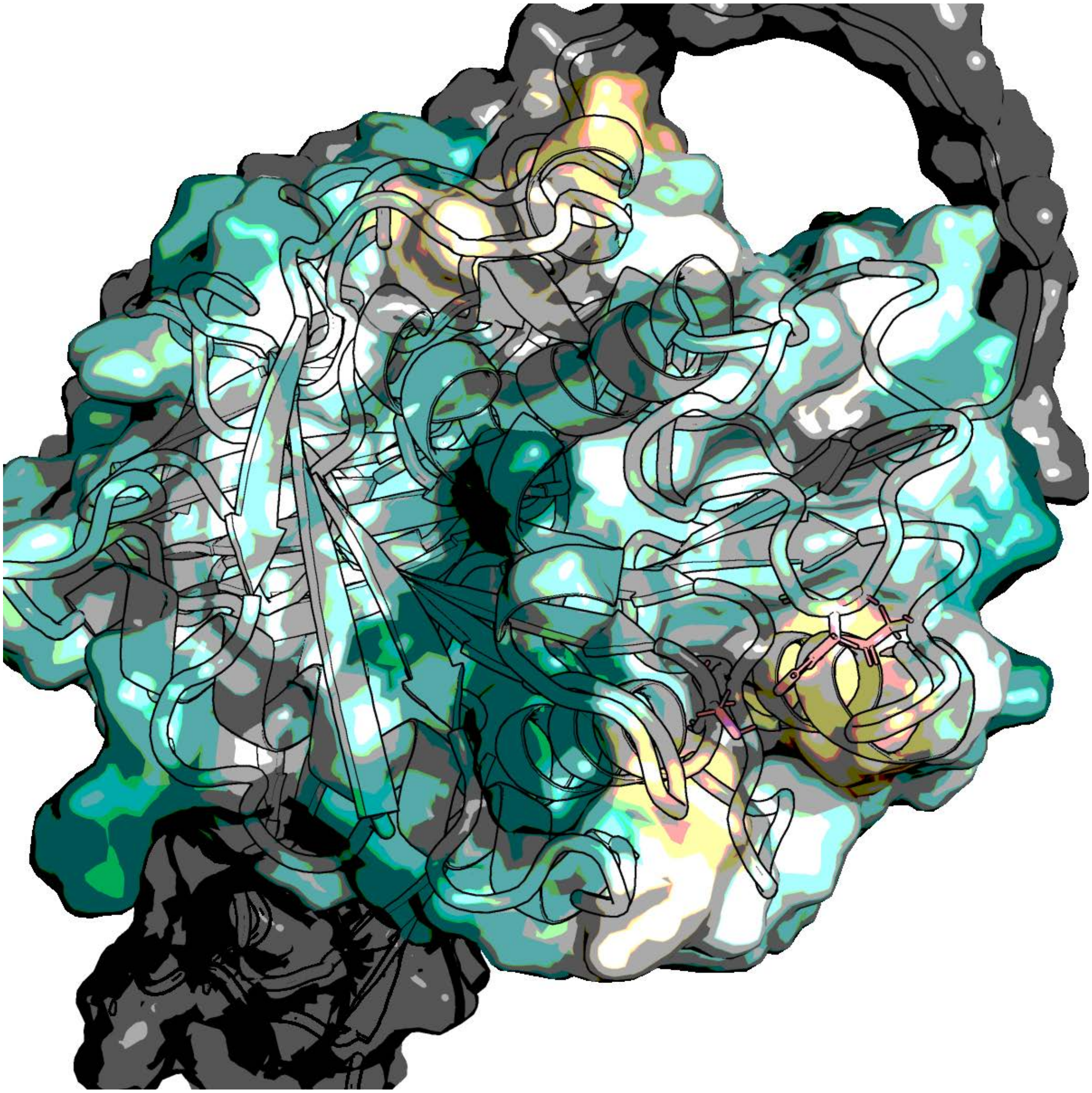

TEP080_highlights.png

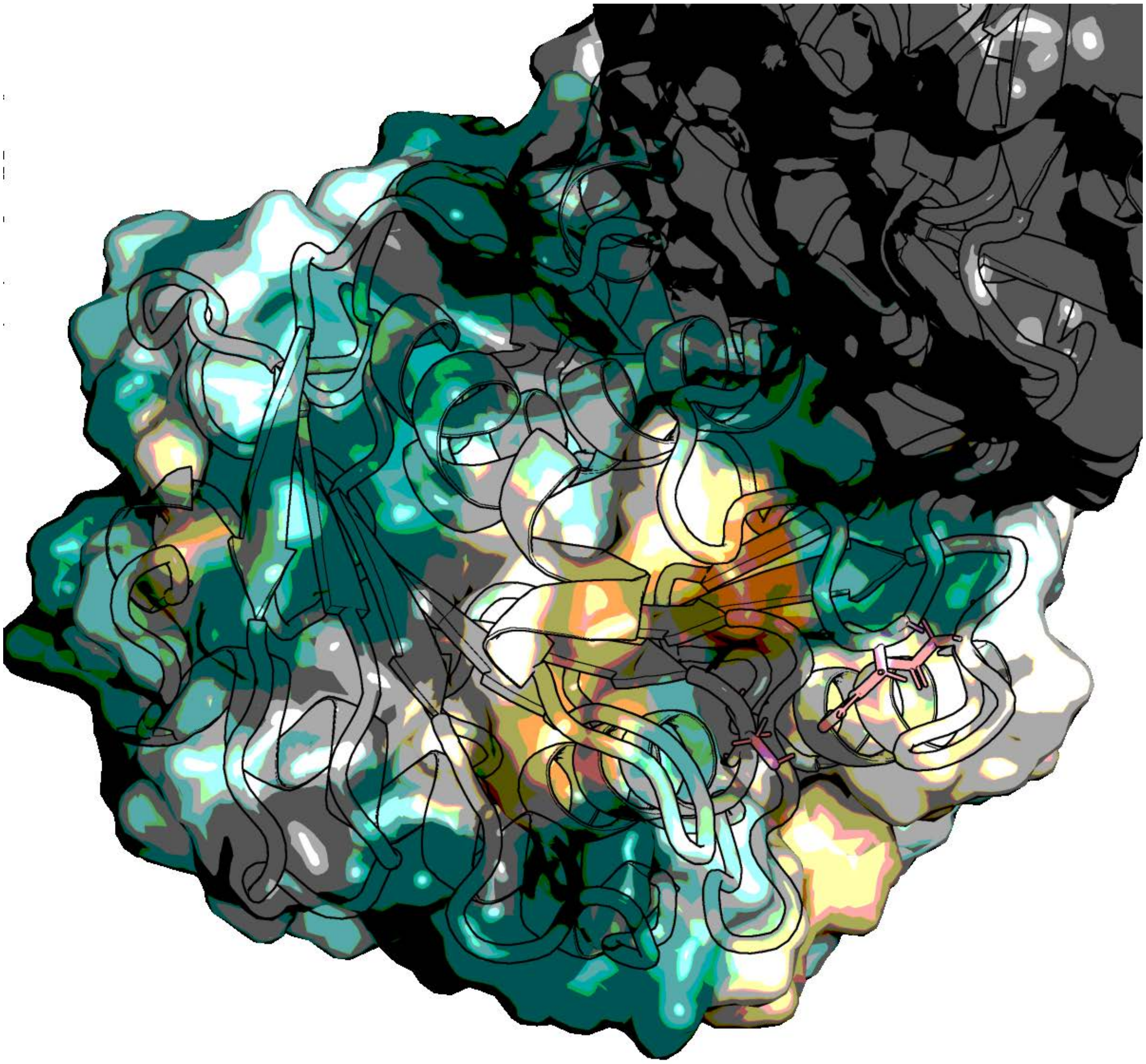

TEP082_highlights.png

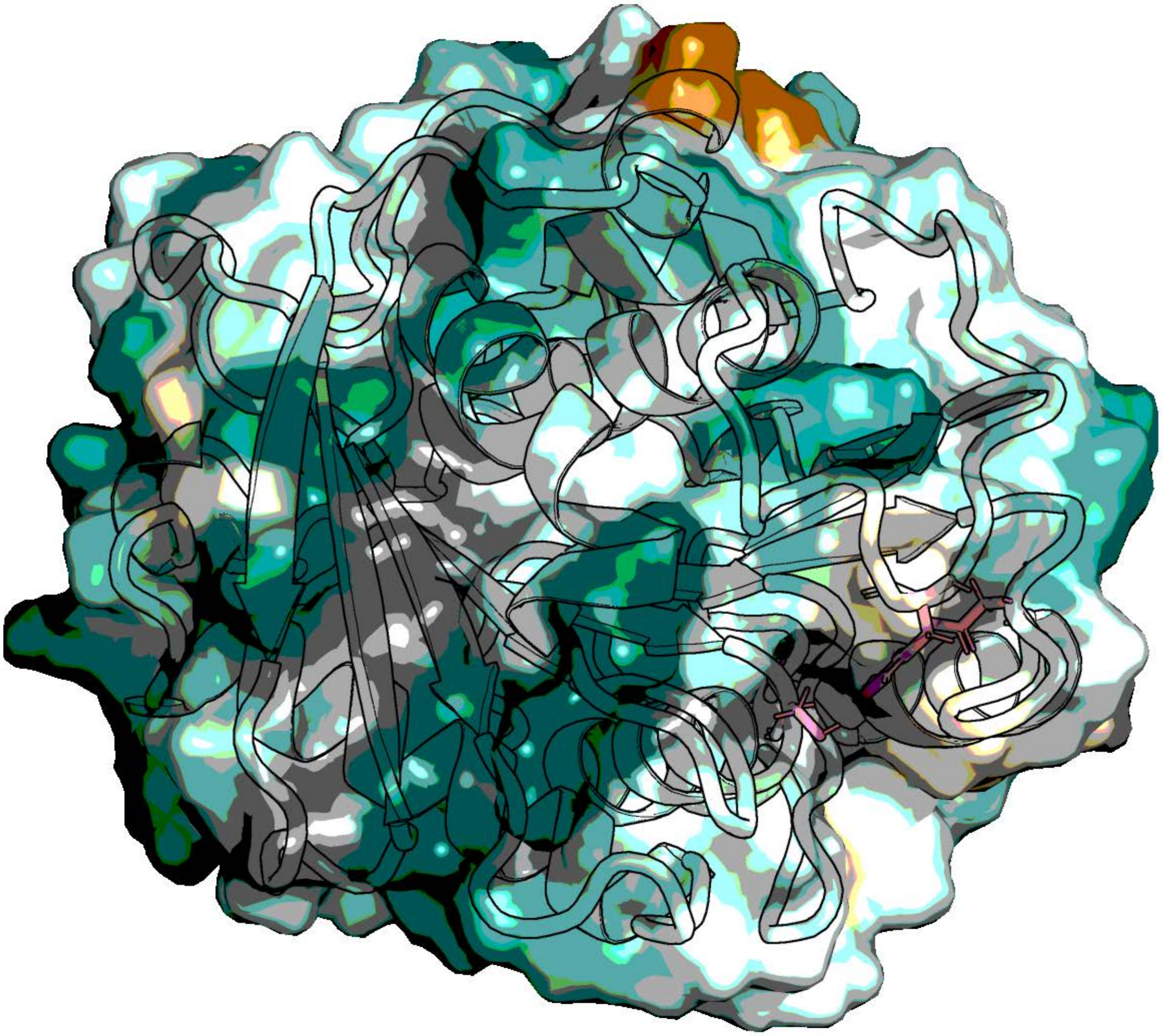

TEP083_highlights.png

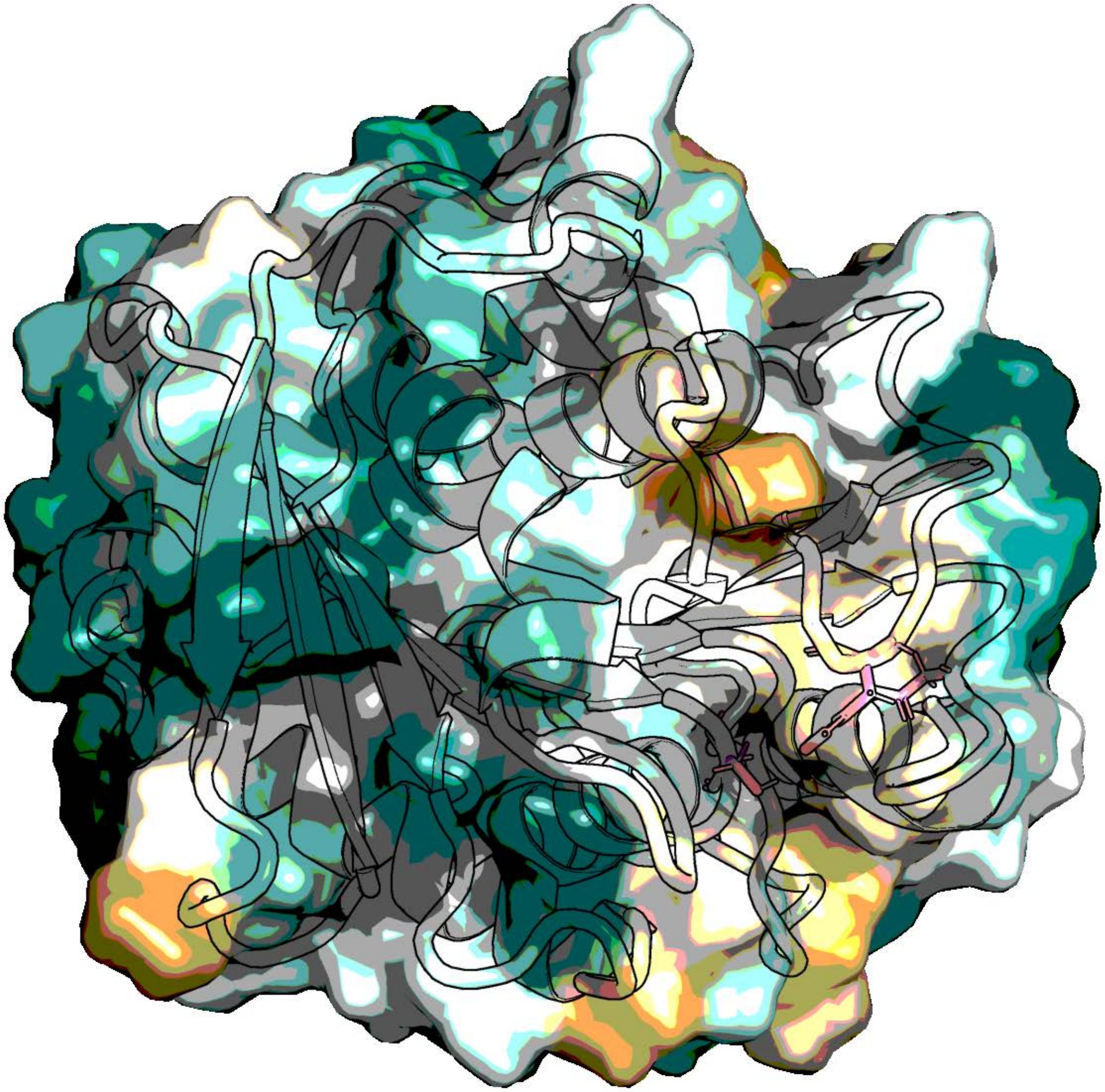

TEP091_highlights.png

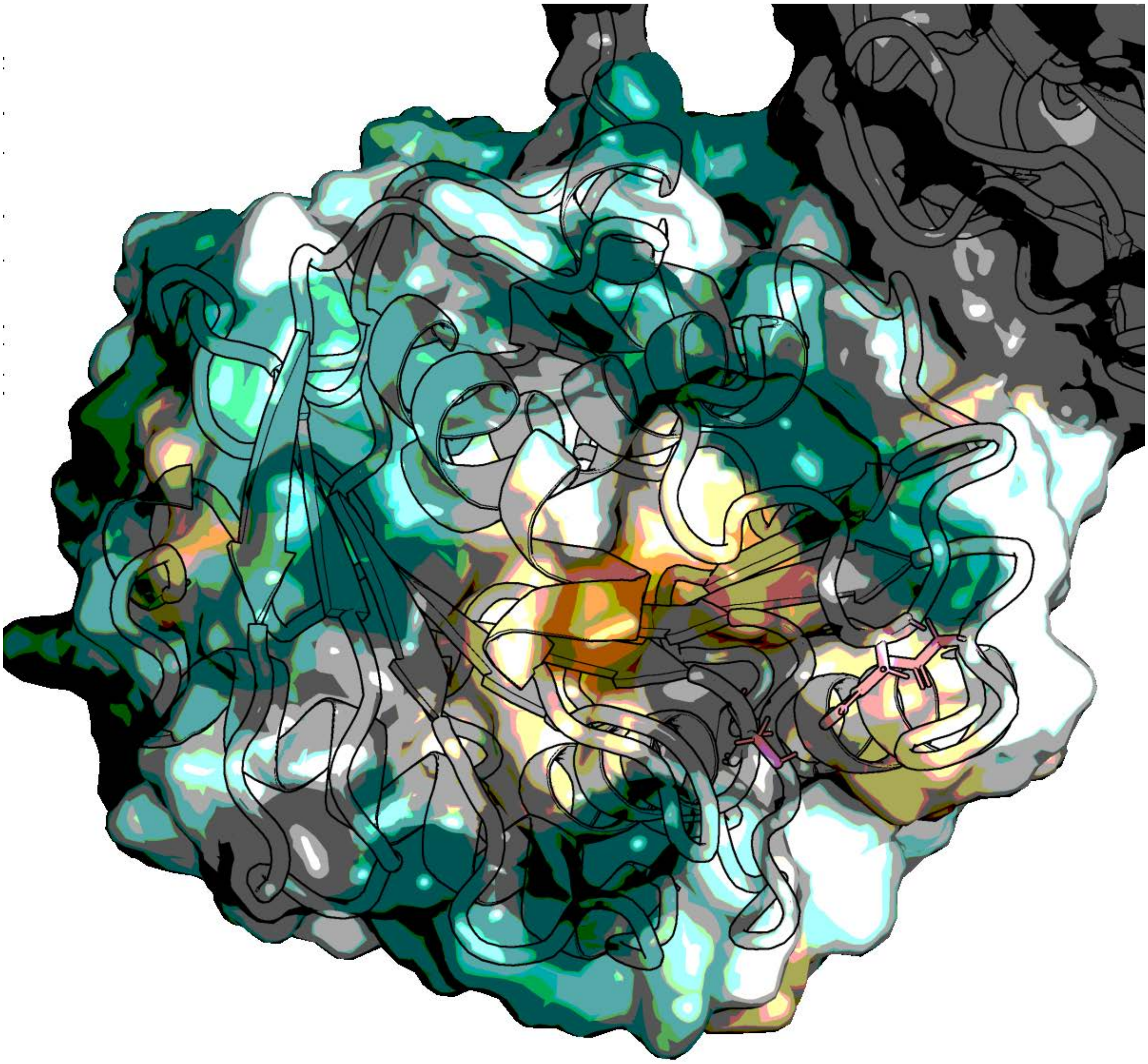

TEP092_highlights.png

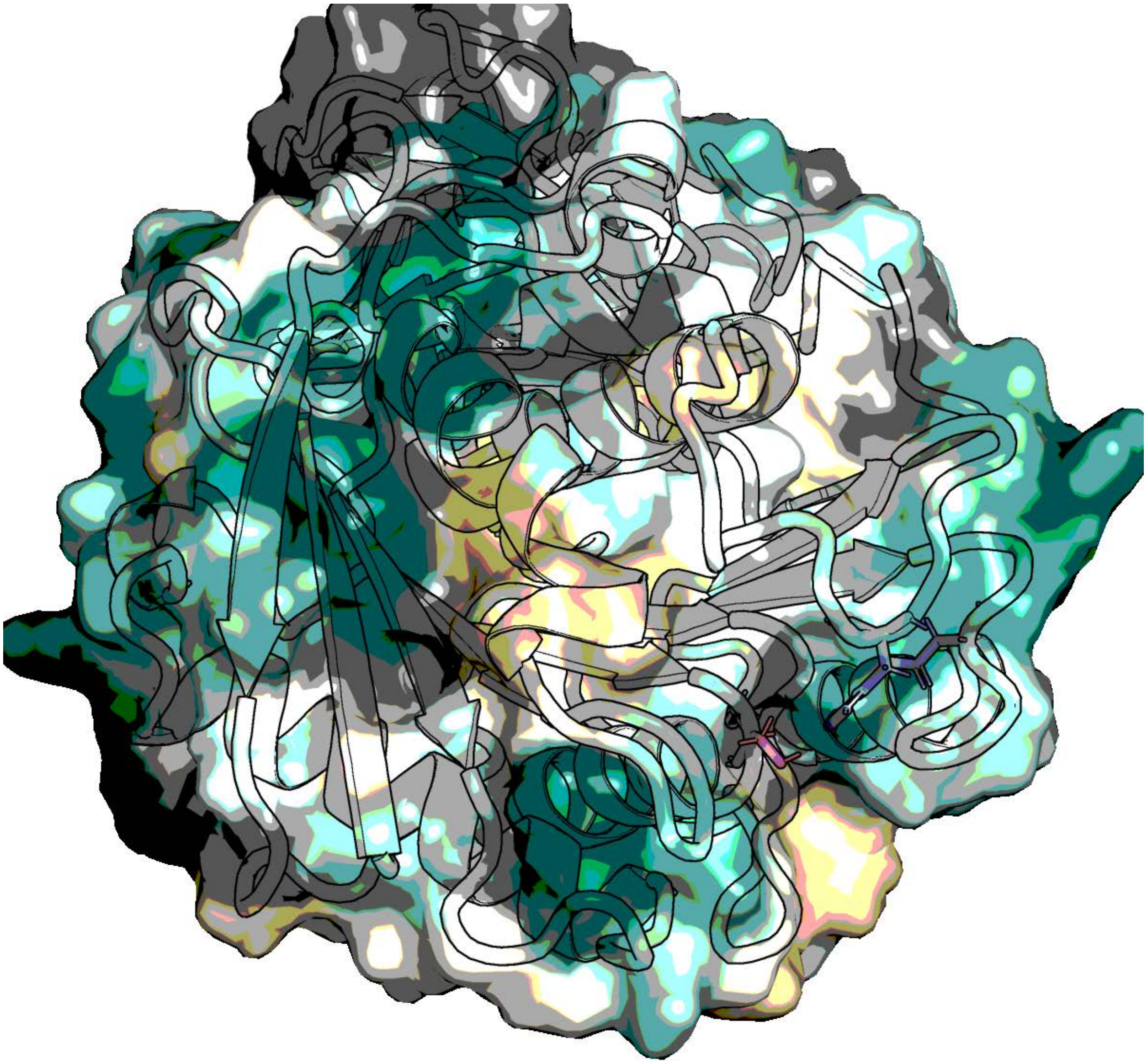

TEP093_highlights.png

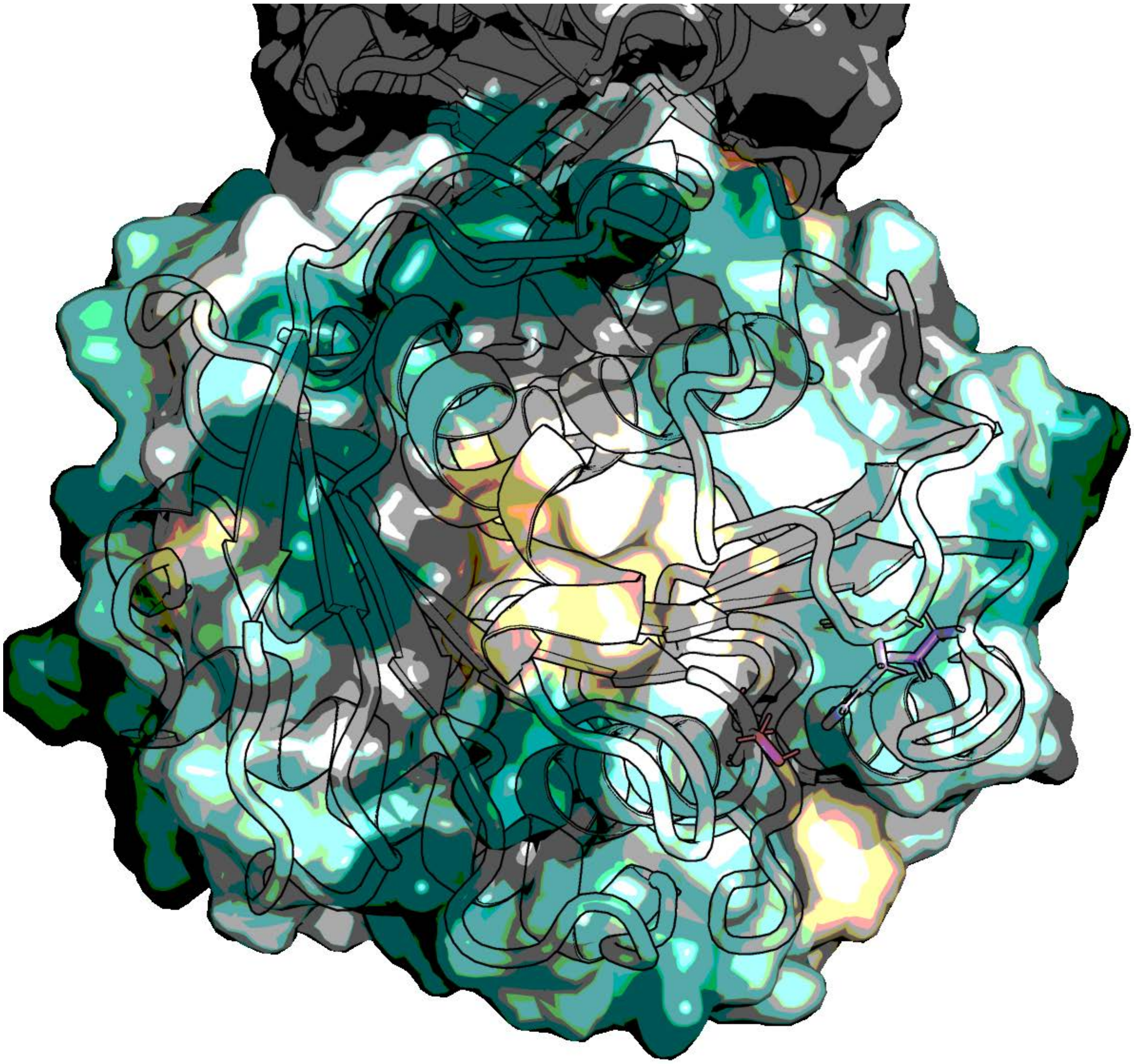

TEP097_highlights.png

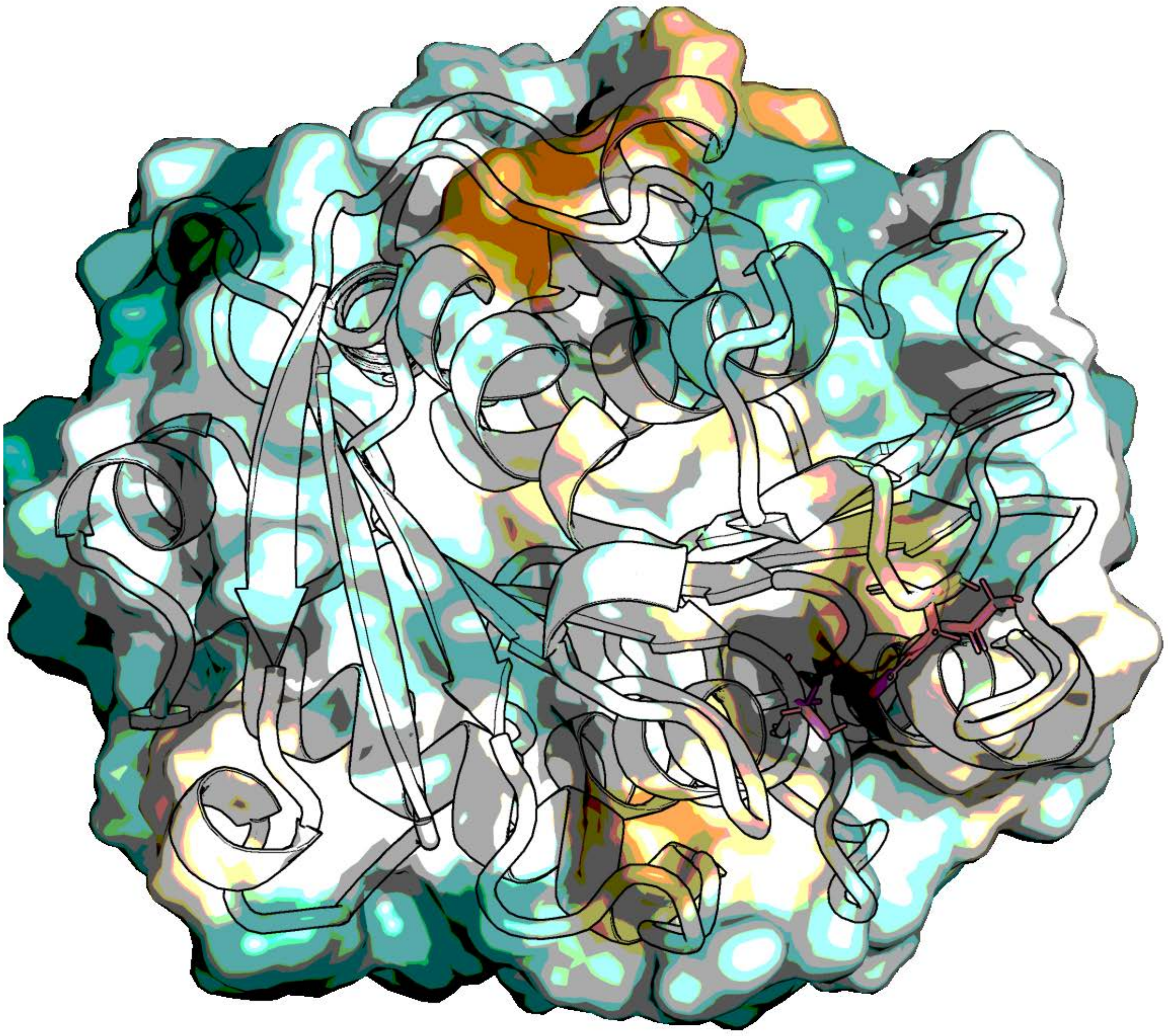

TEP101_highlights.png

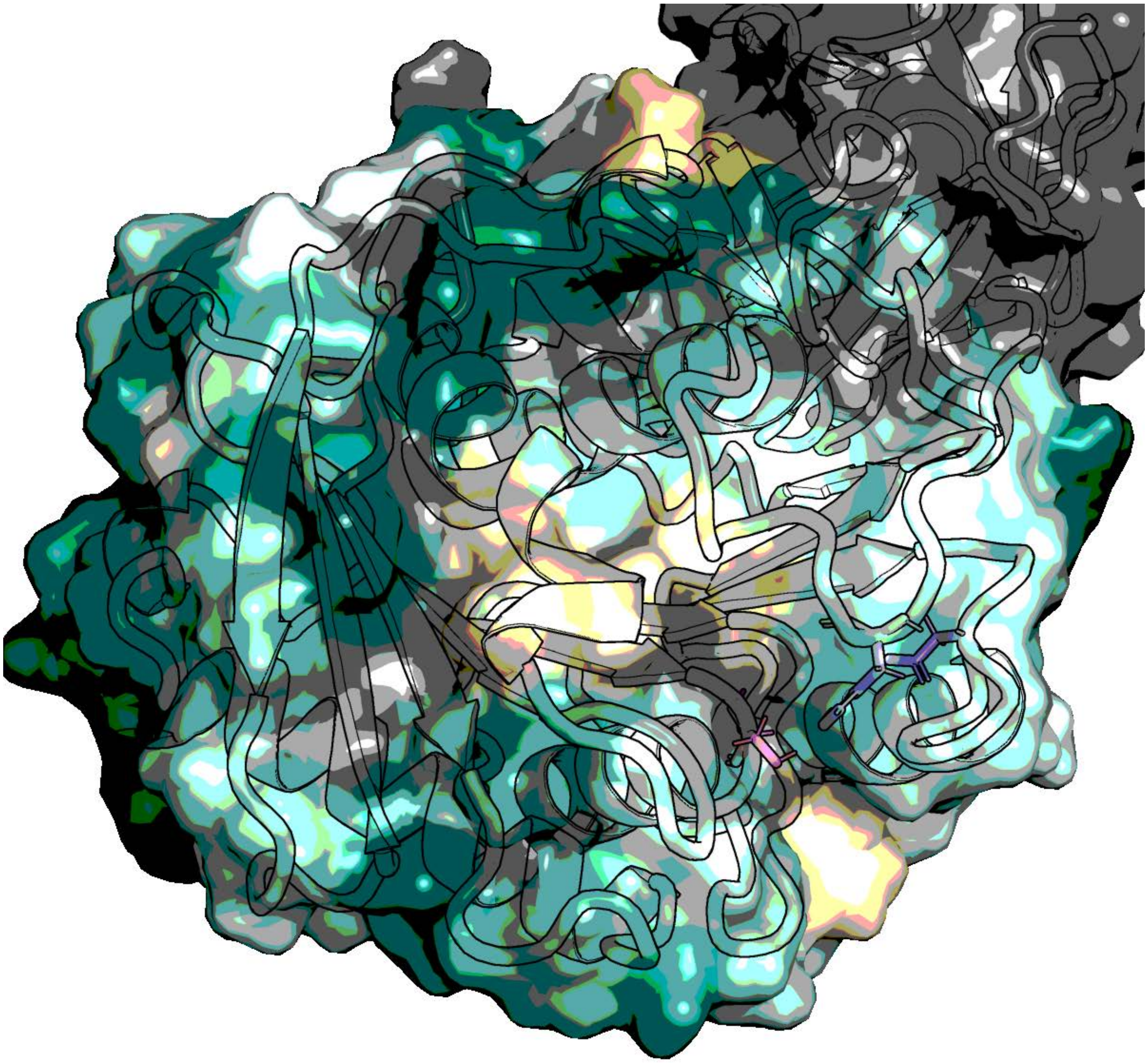

TEP102_highlights.png

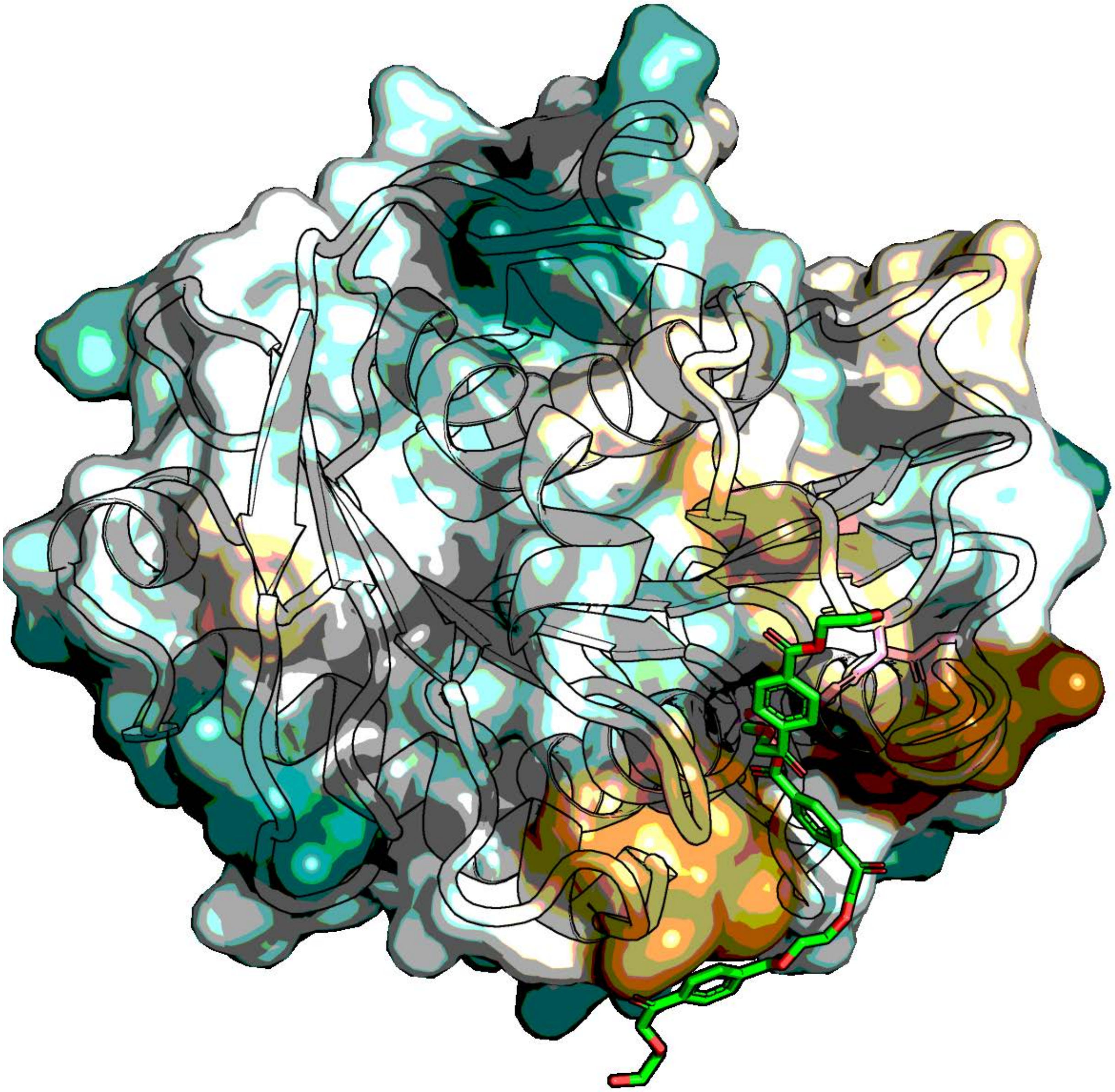

TEP103_highlights.png

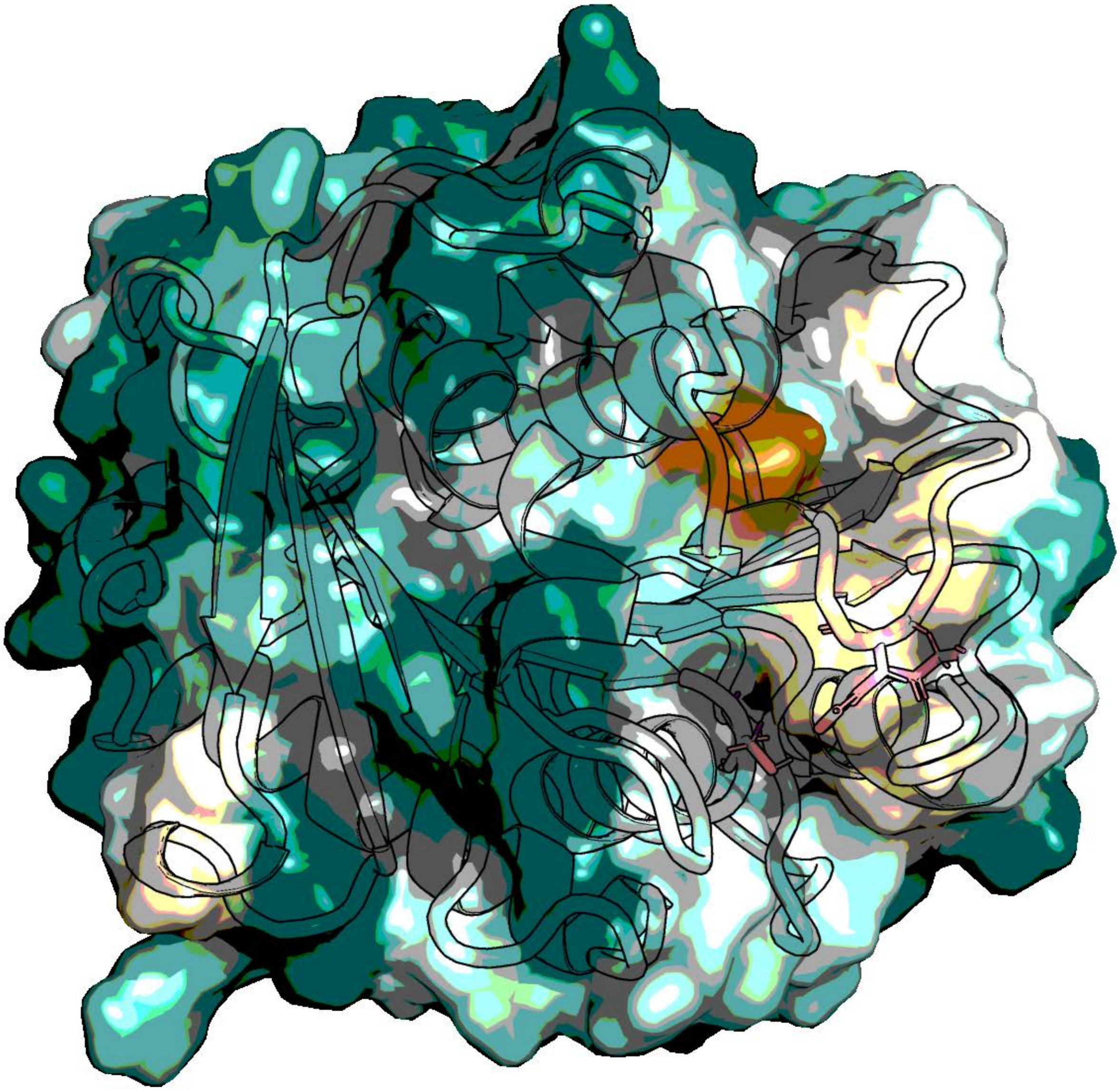

TEP105_highlights.png

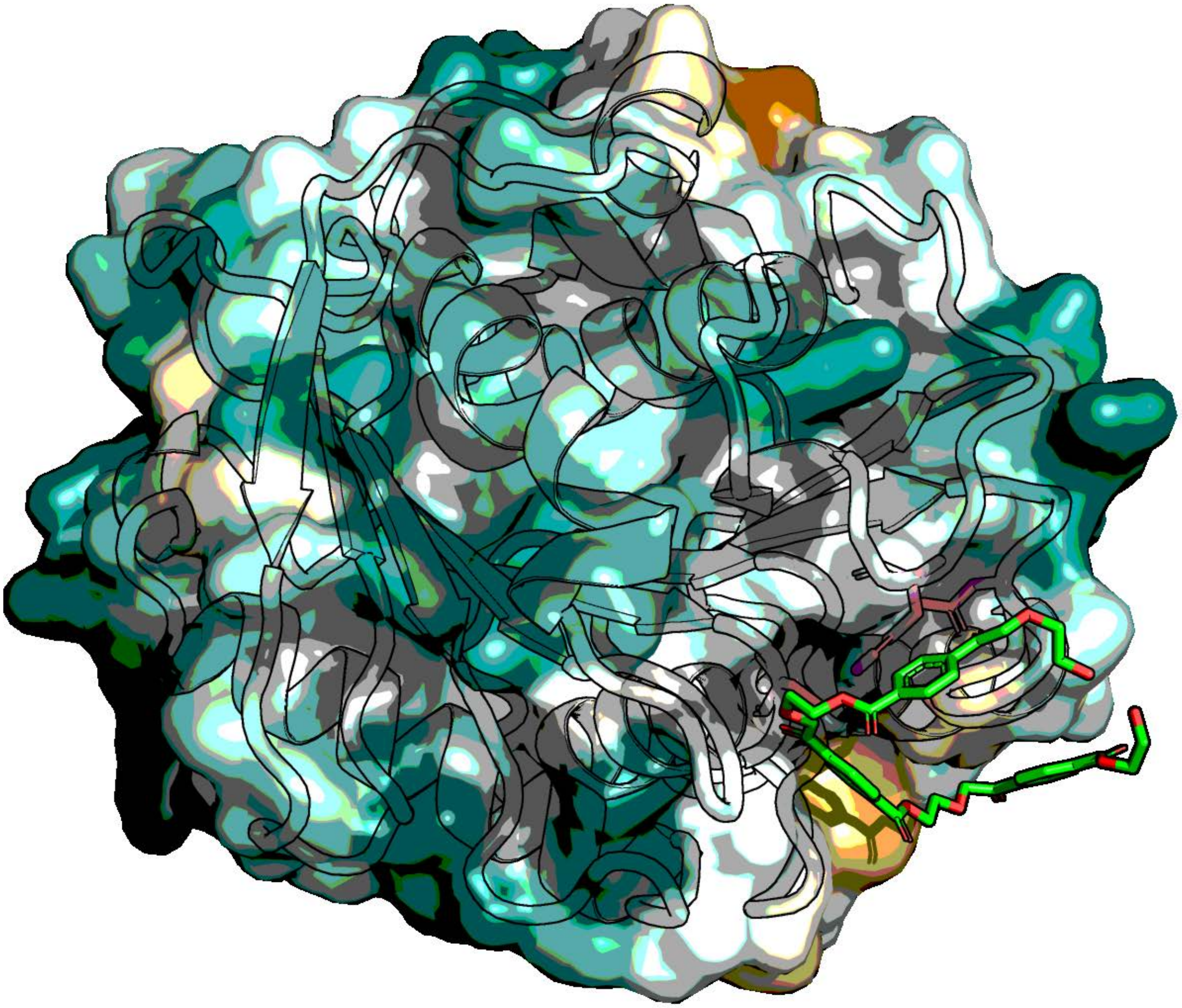

TEP109_highlights.png

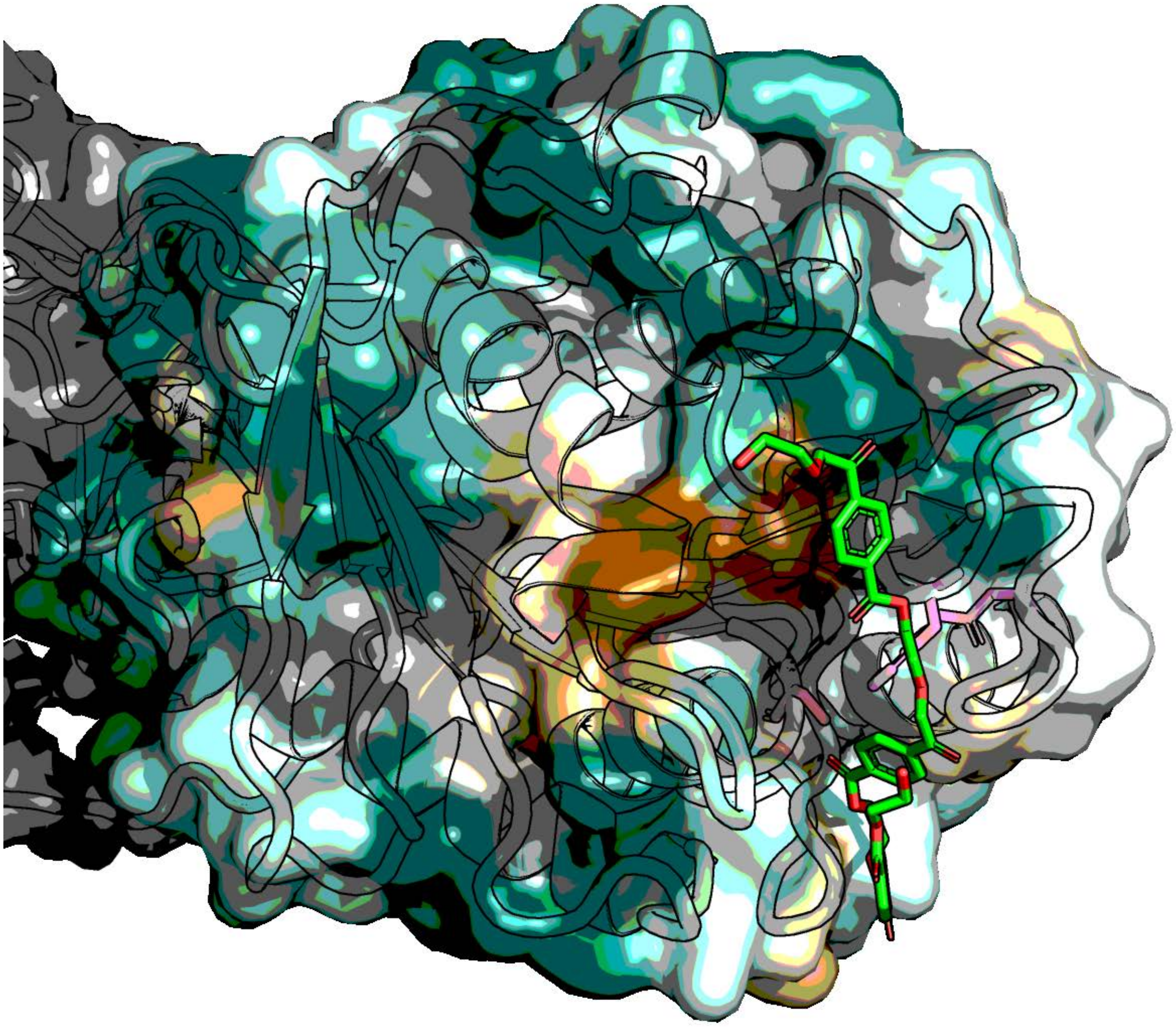

TEP111_highlights.png

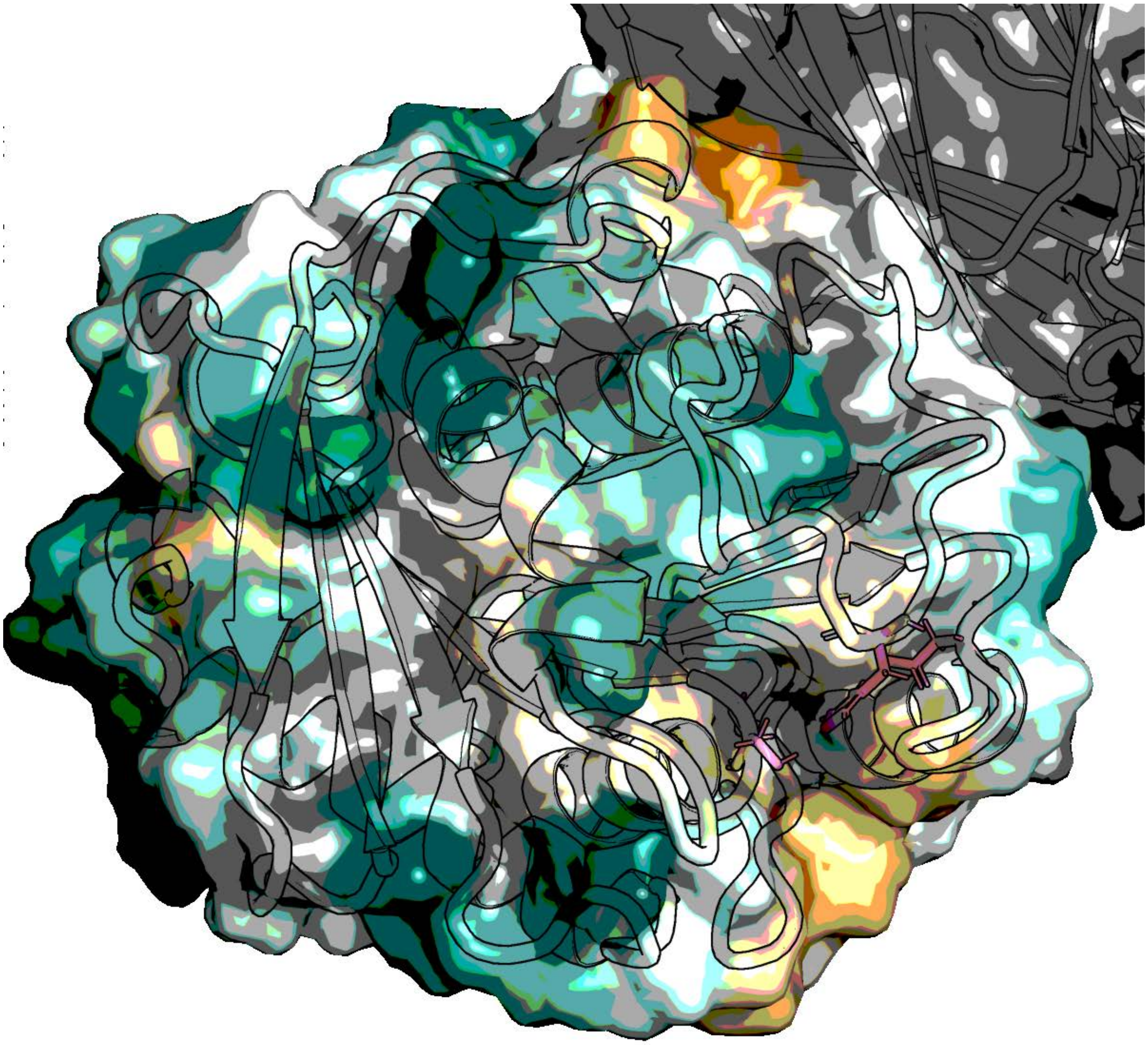

TEP114_highlights.png

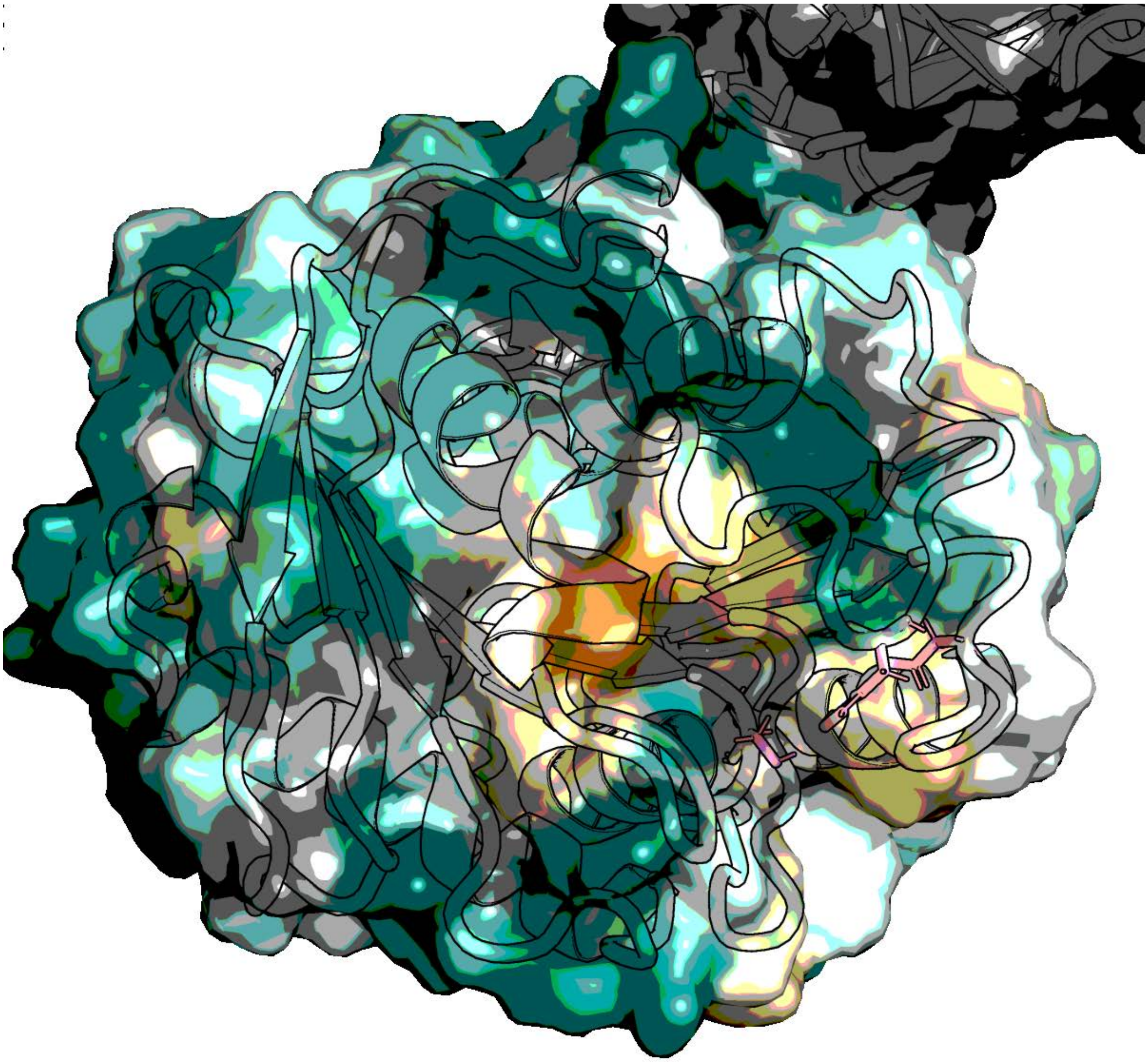

TEP115_highlights.png

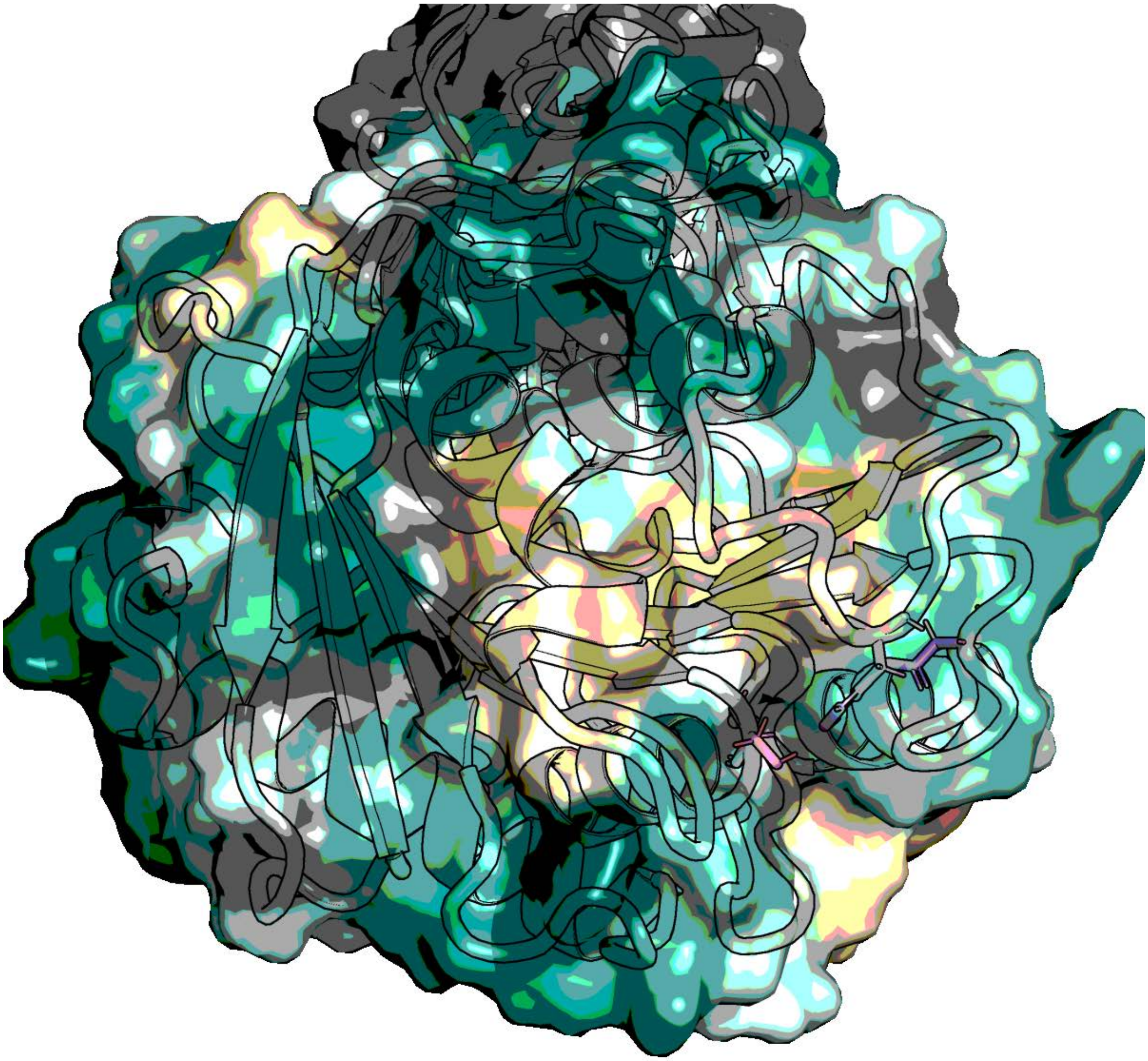

TEP131_highlights.png

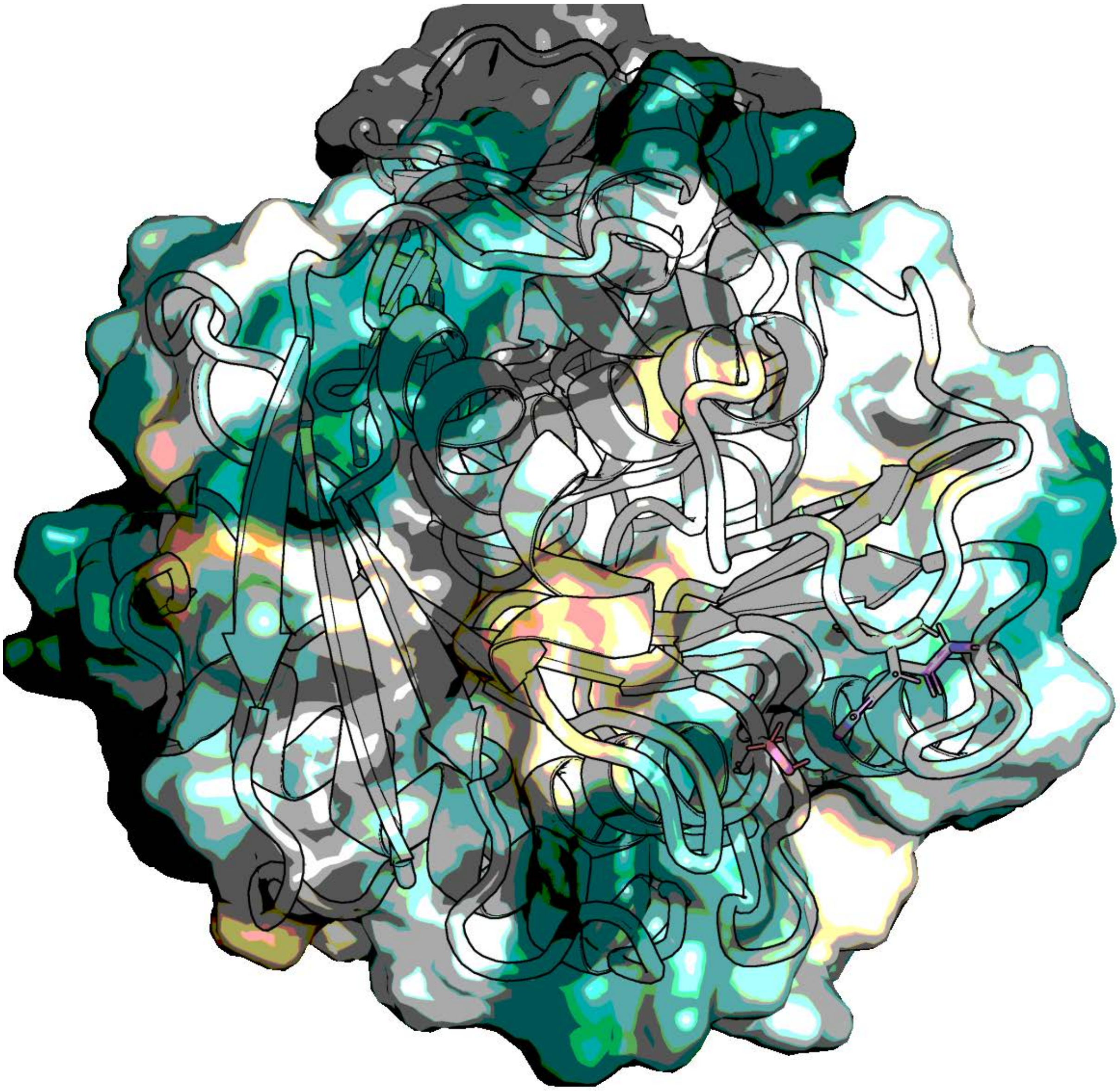

TEP163_highlights.png

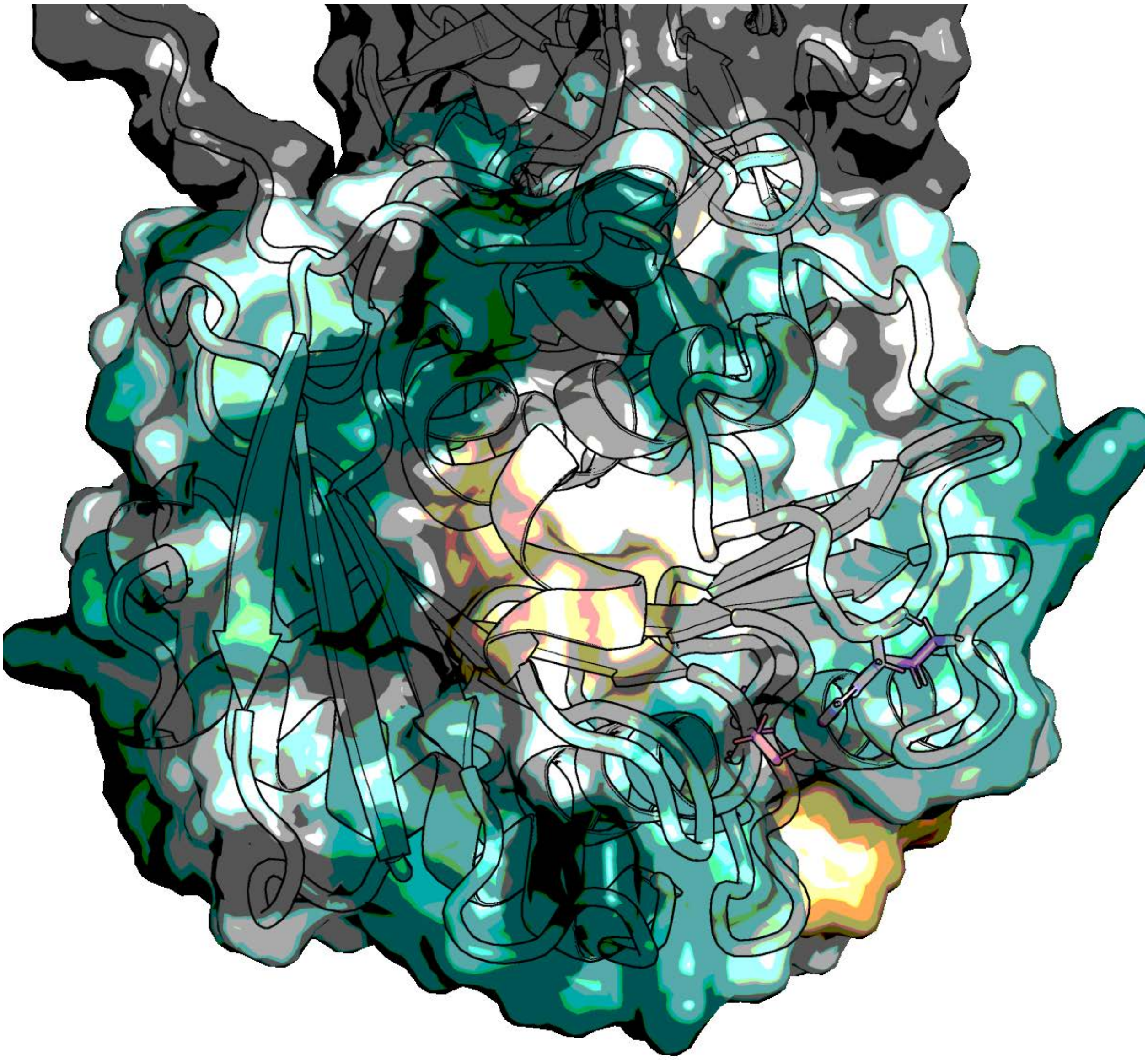

TEP164_highlights.png

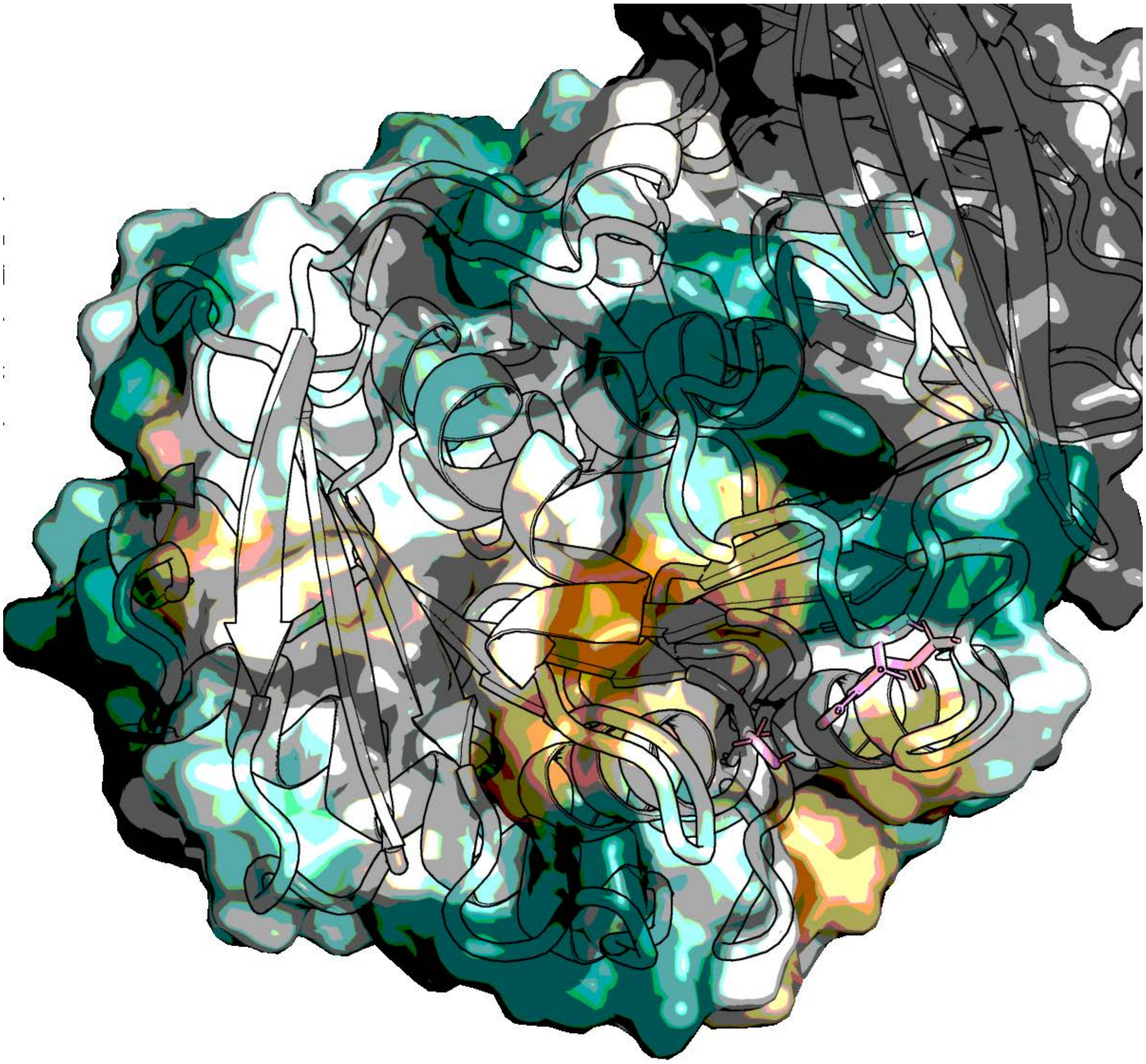

TEP165_highlights.png

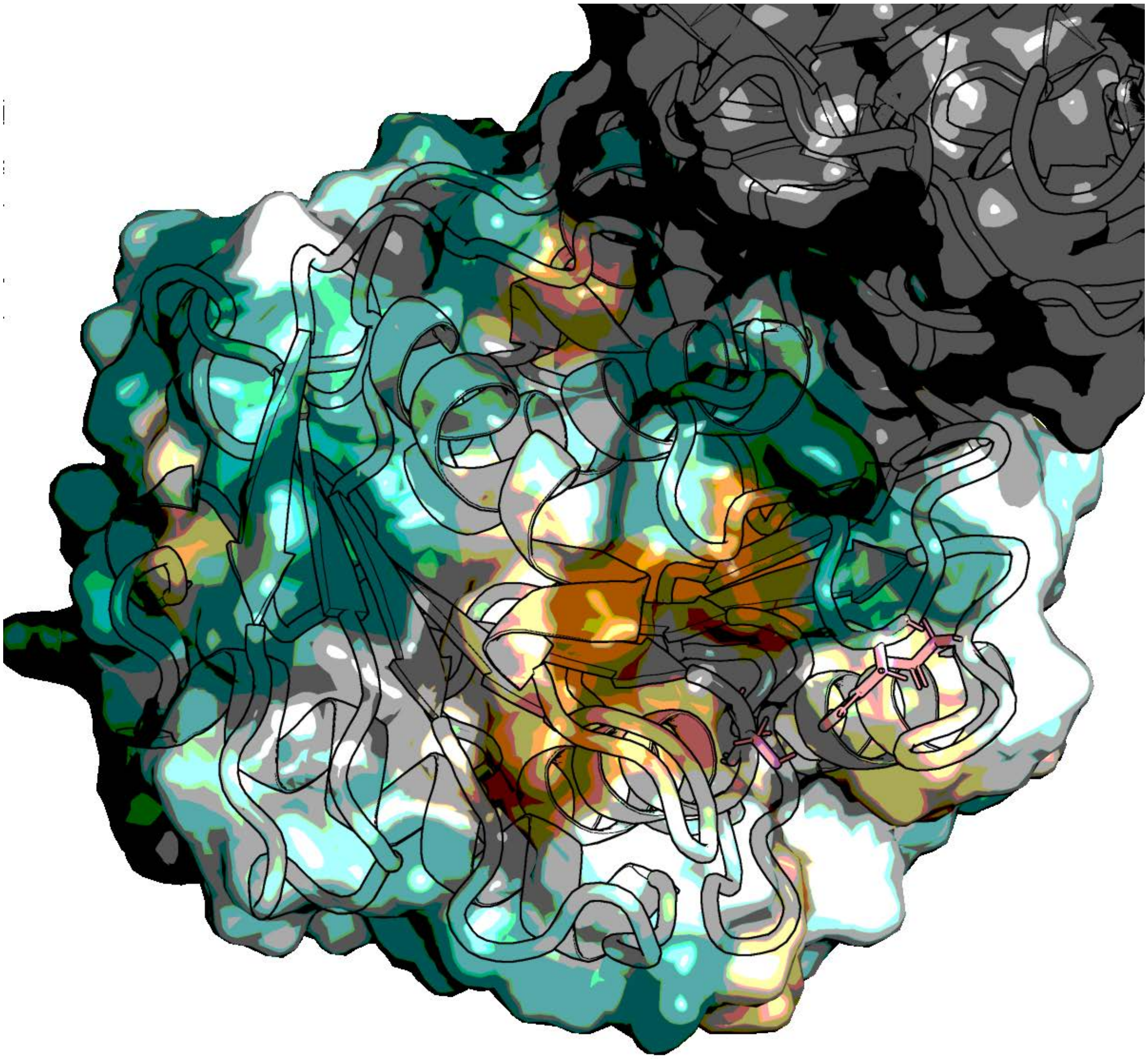

TEP168_highlights.png

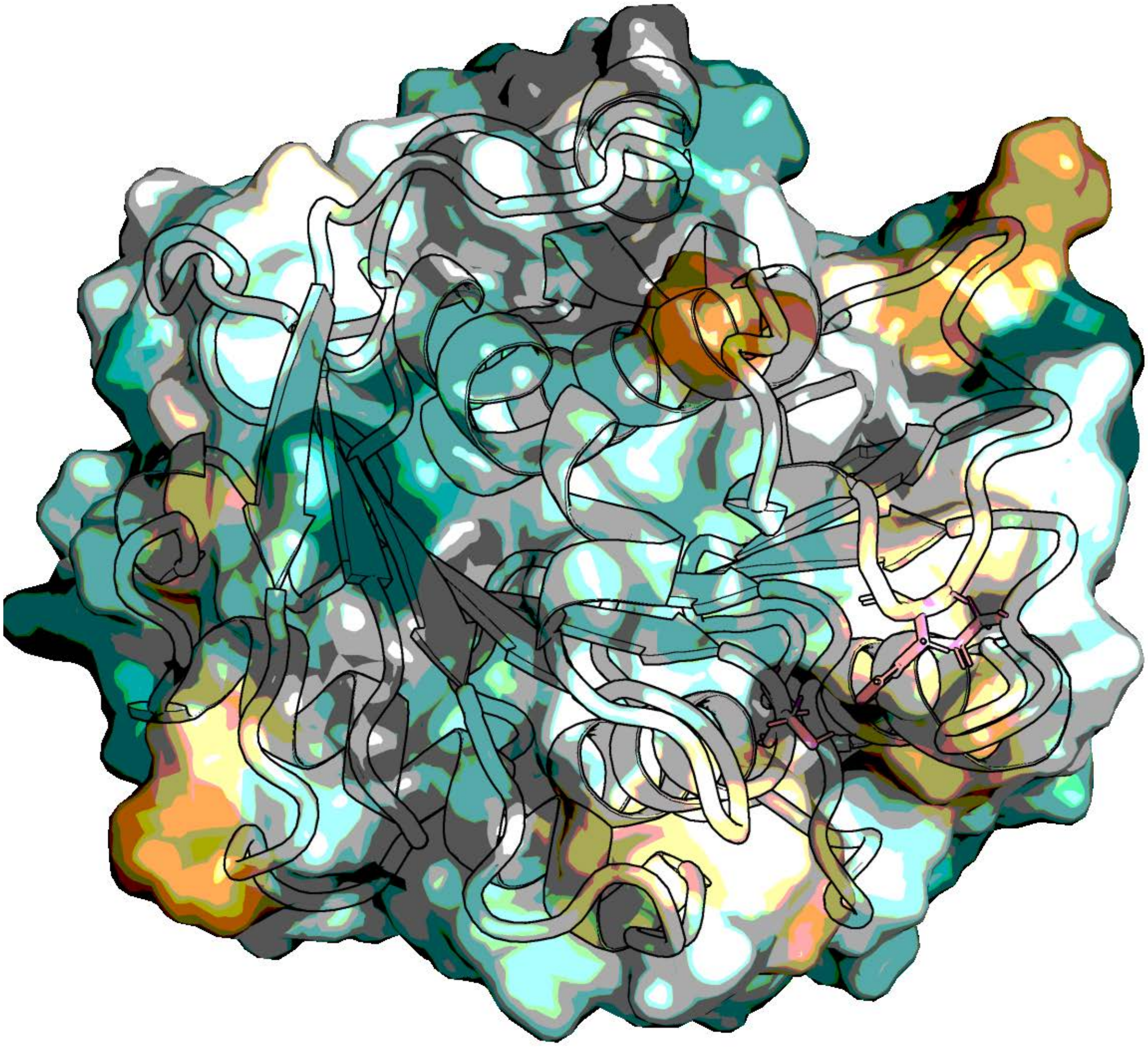

TEP178_highlights.png

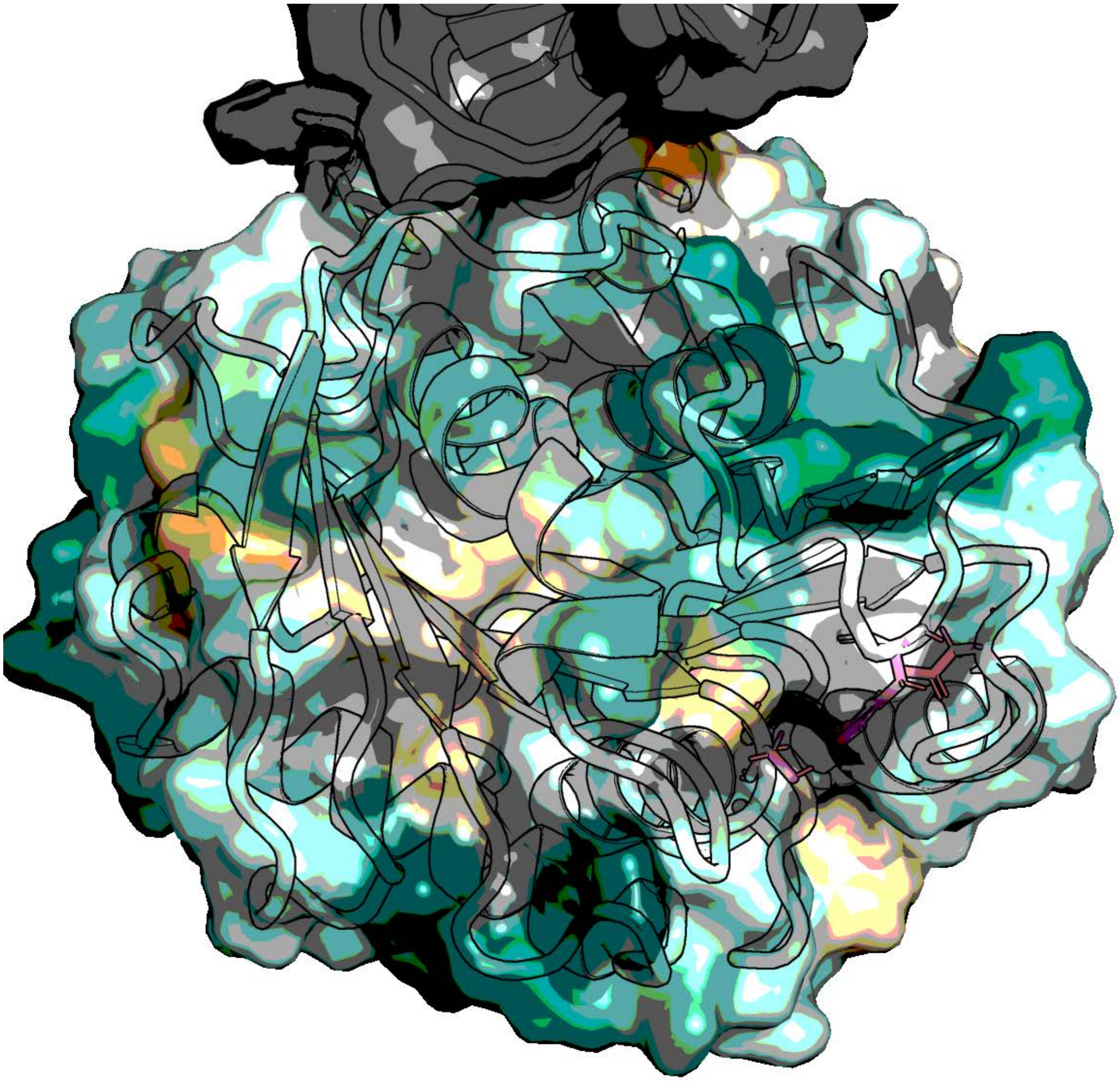

TEP181_highlights.png

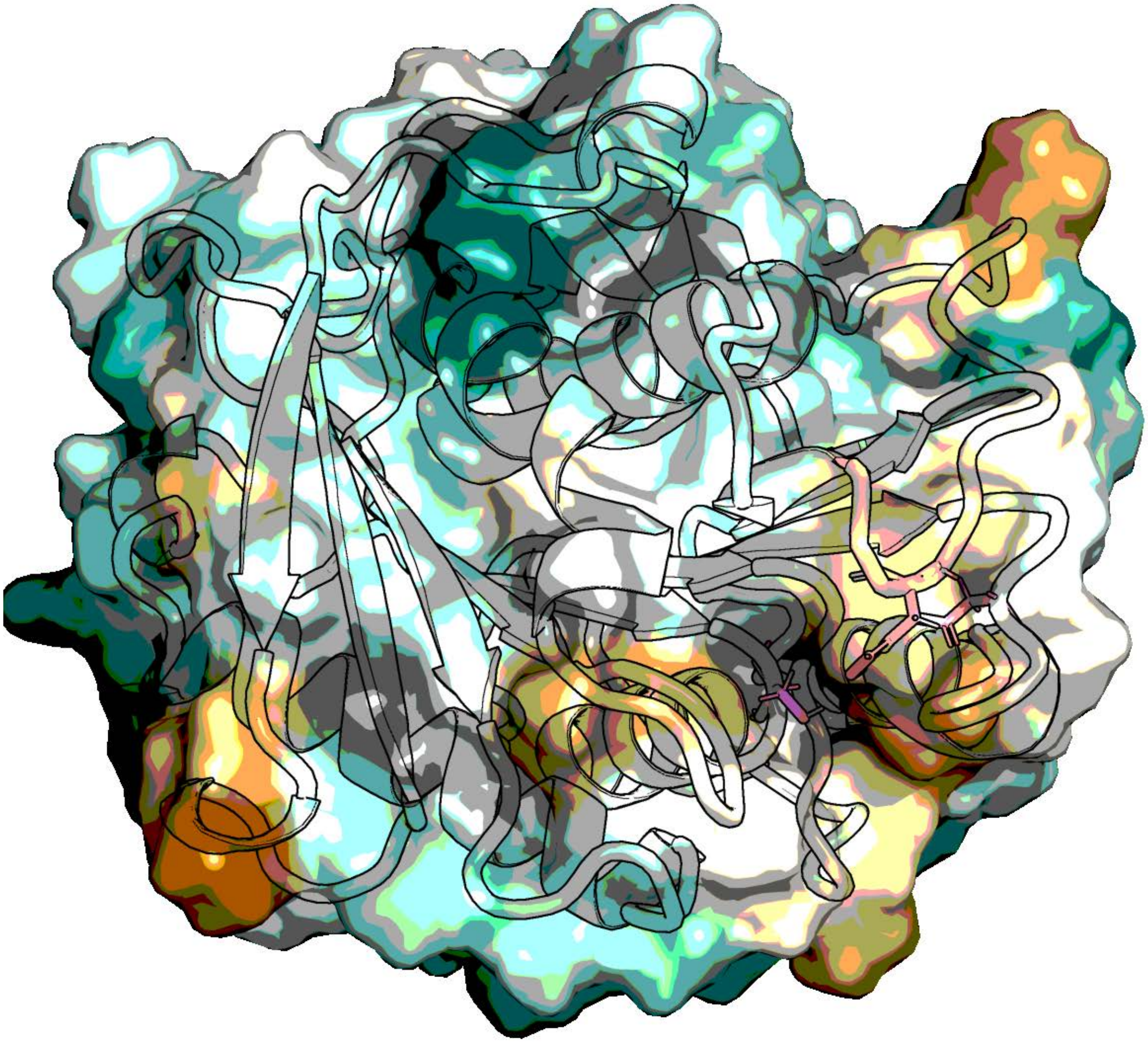

TEP182_highlights.png

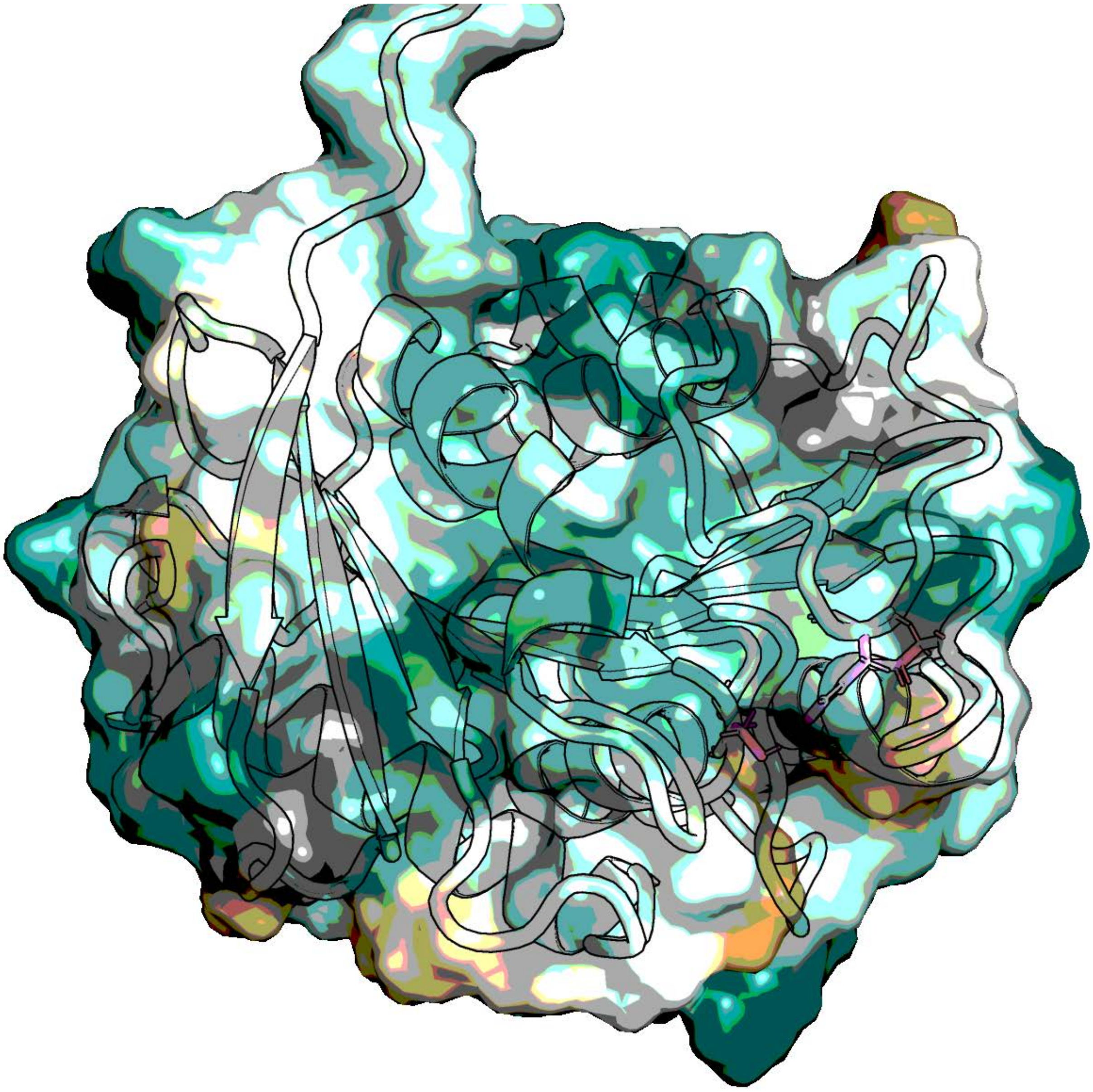

TEP189_highlights.png

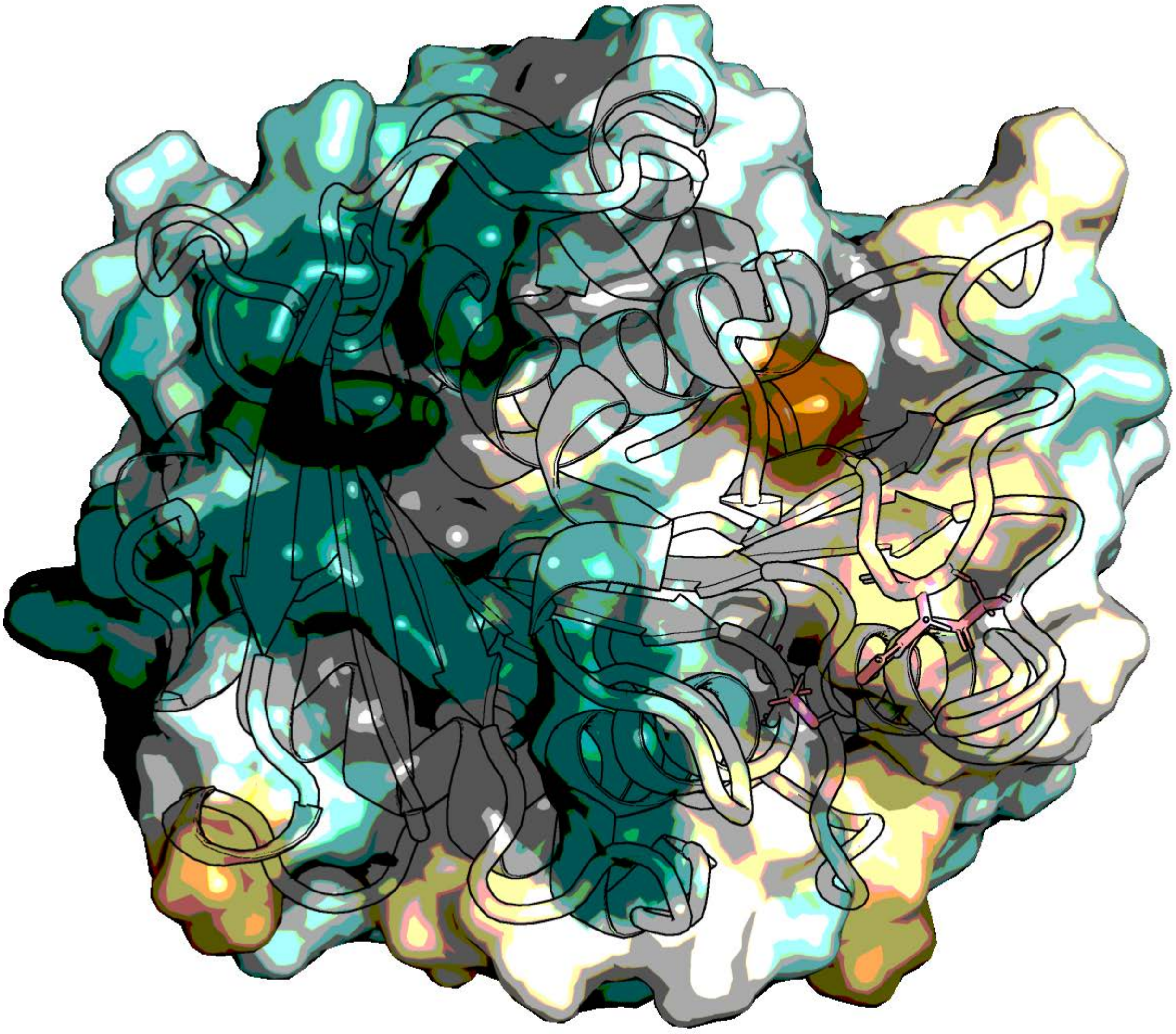

TEP194_highlights.png

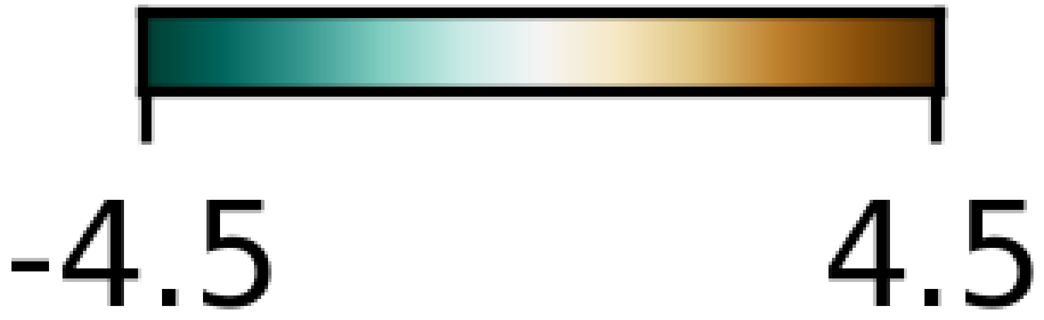

_LEGEND.png

Observed stickiness

Created on: 2025-04-15 16:03:37

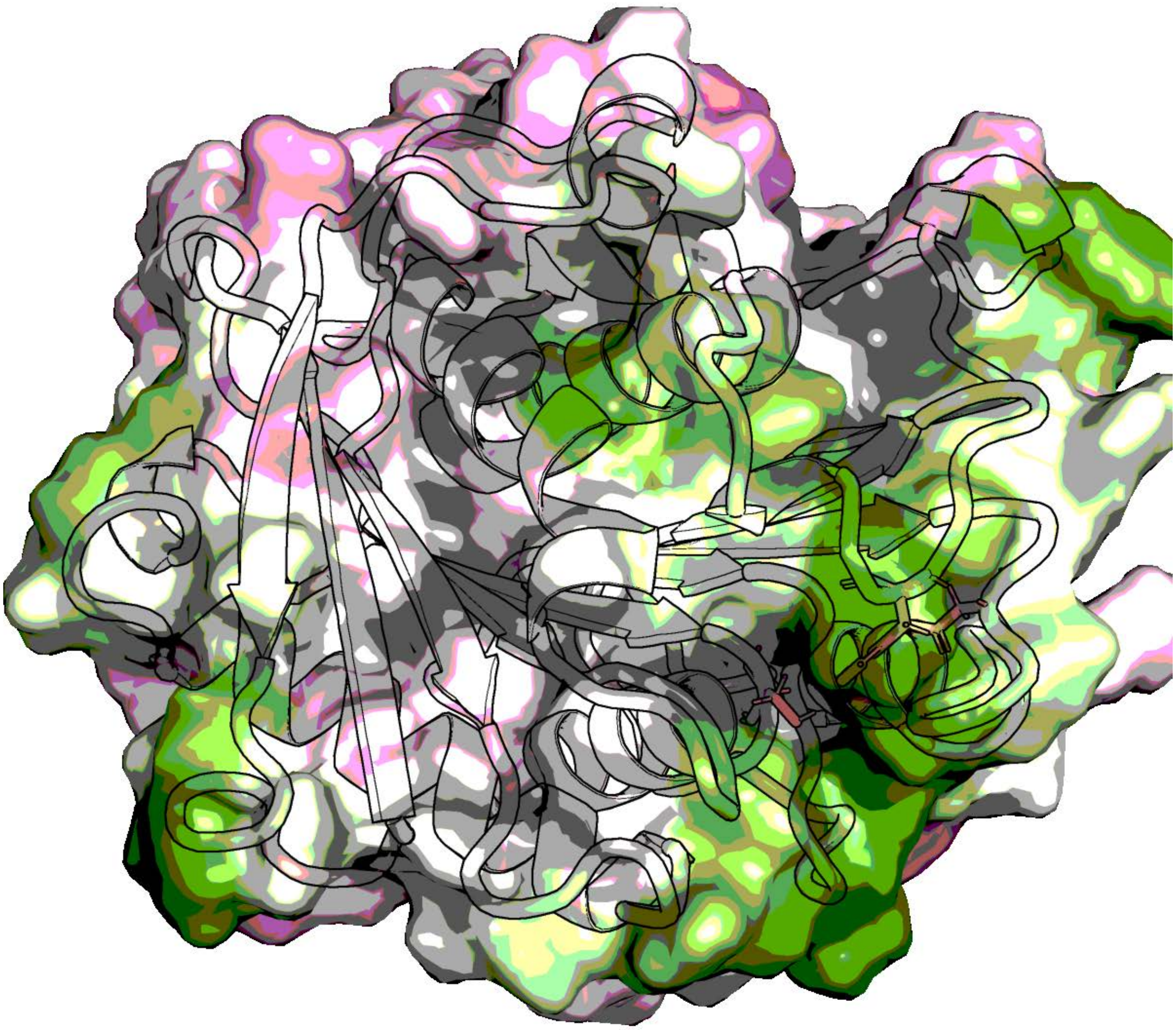

DP003_highlights.png

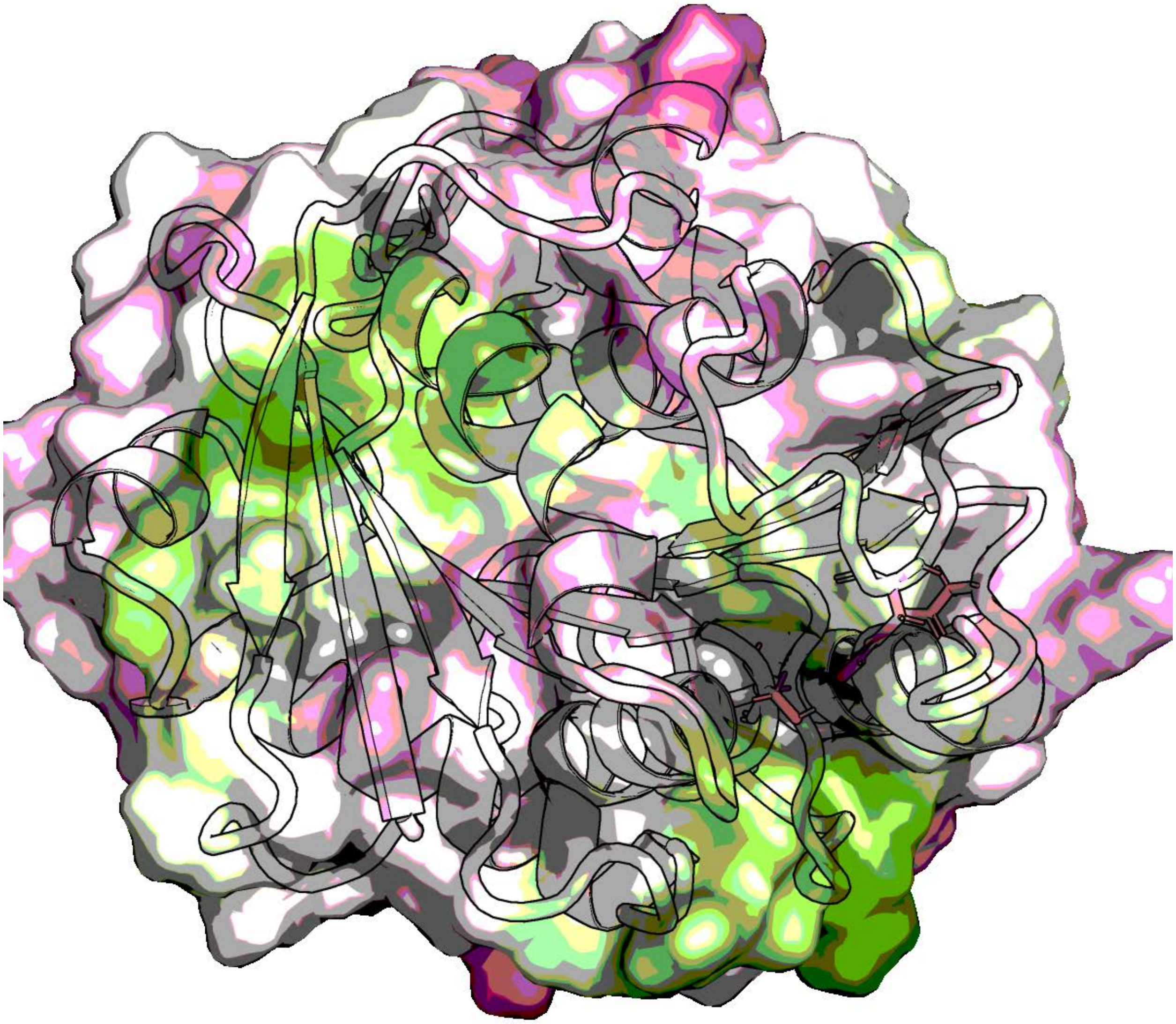

DP007_highlights.png

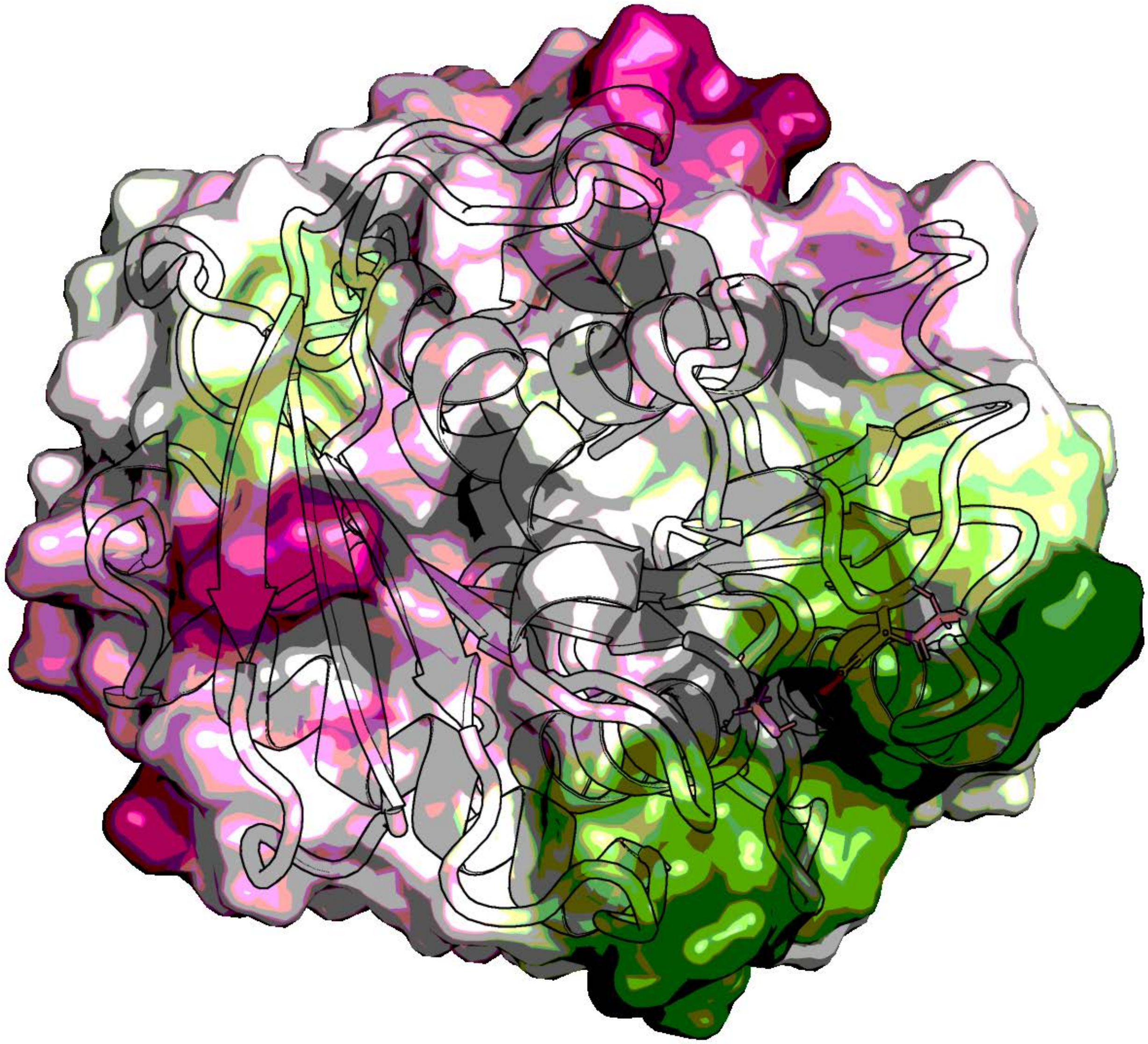

DP008_highlights.png

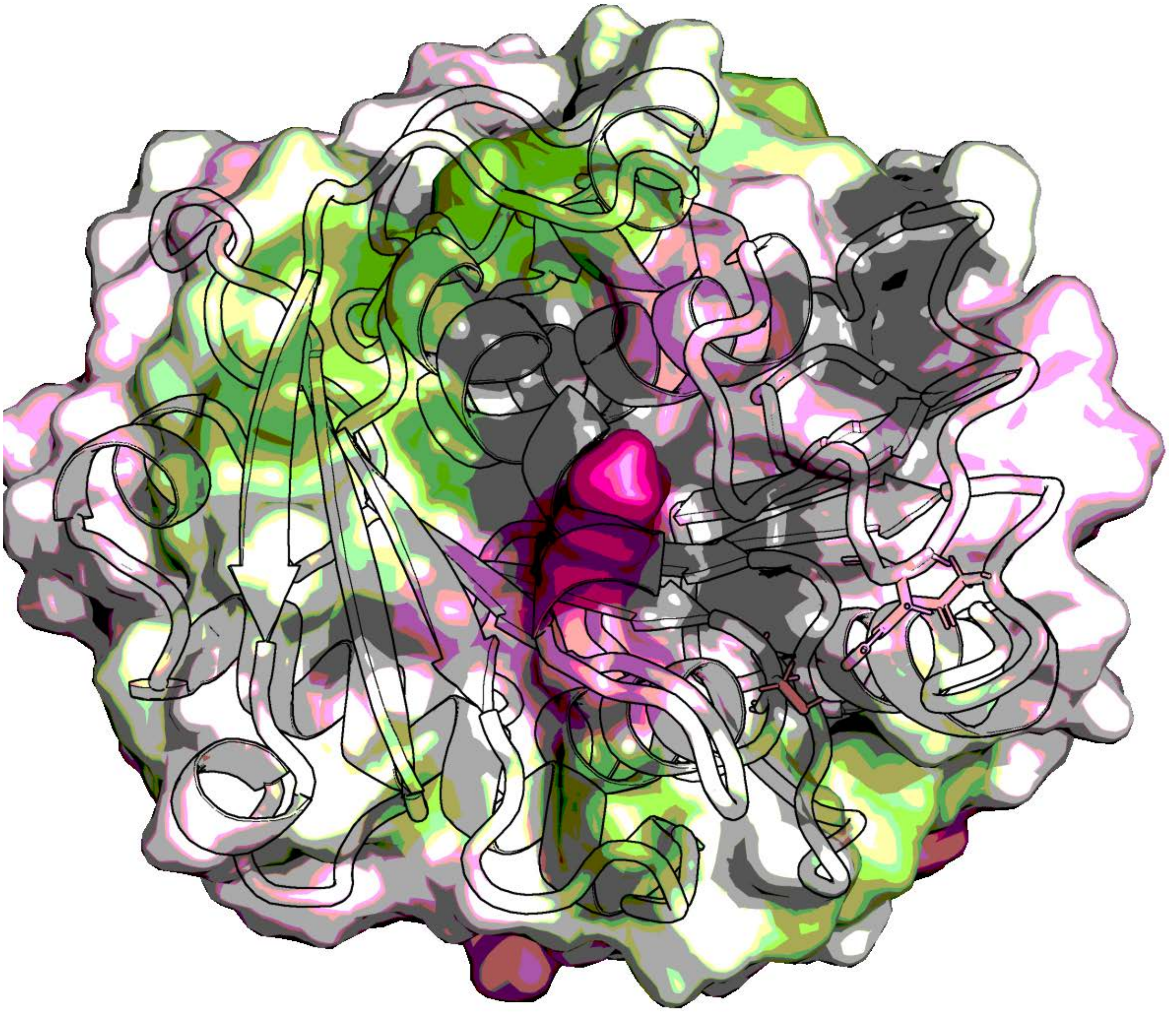

DP009_highlights.png

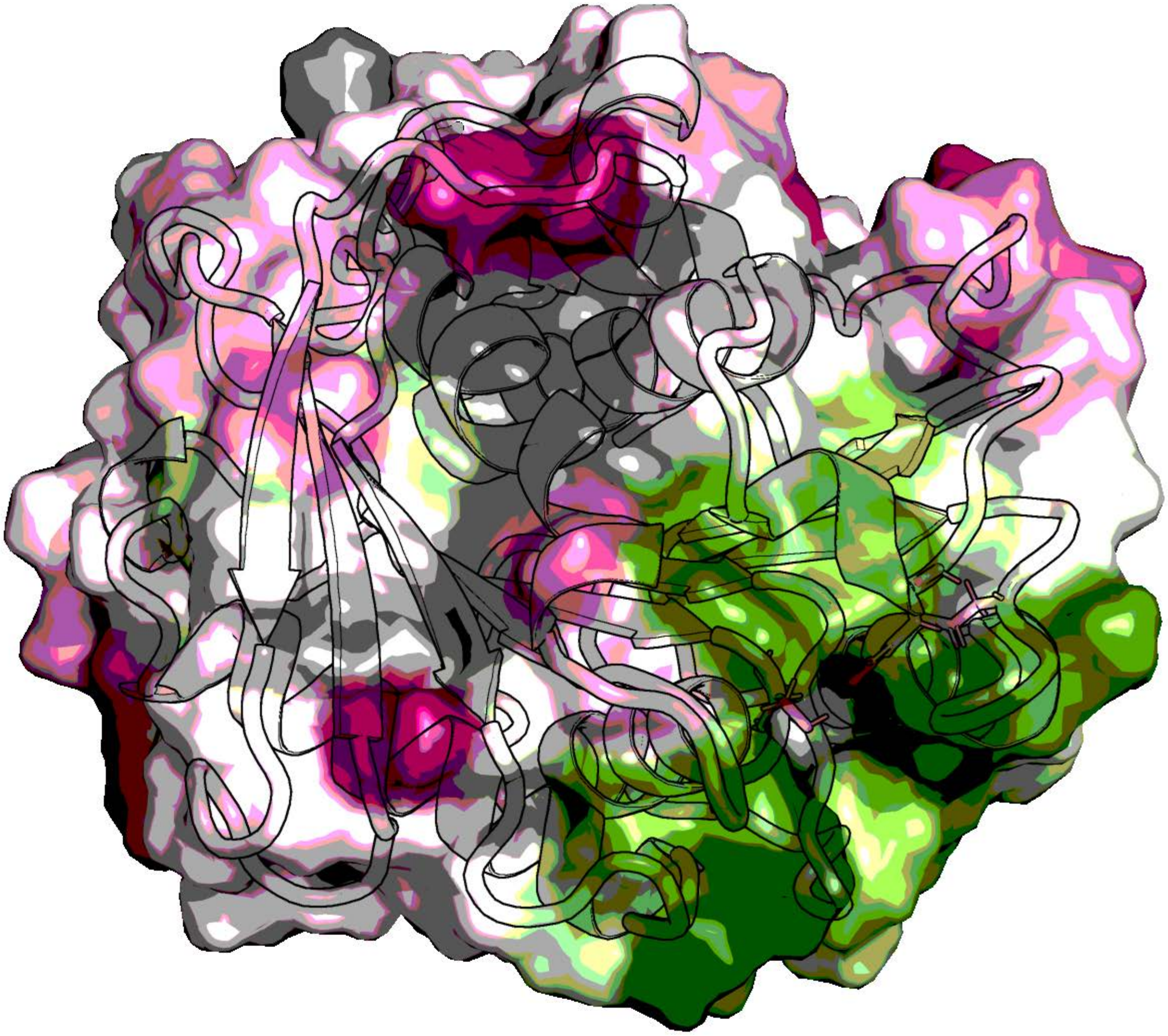

DP016_highlights.png

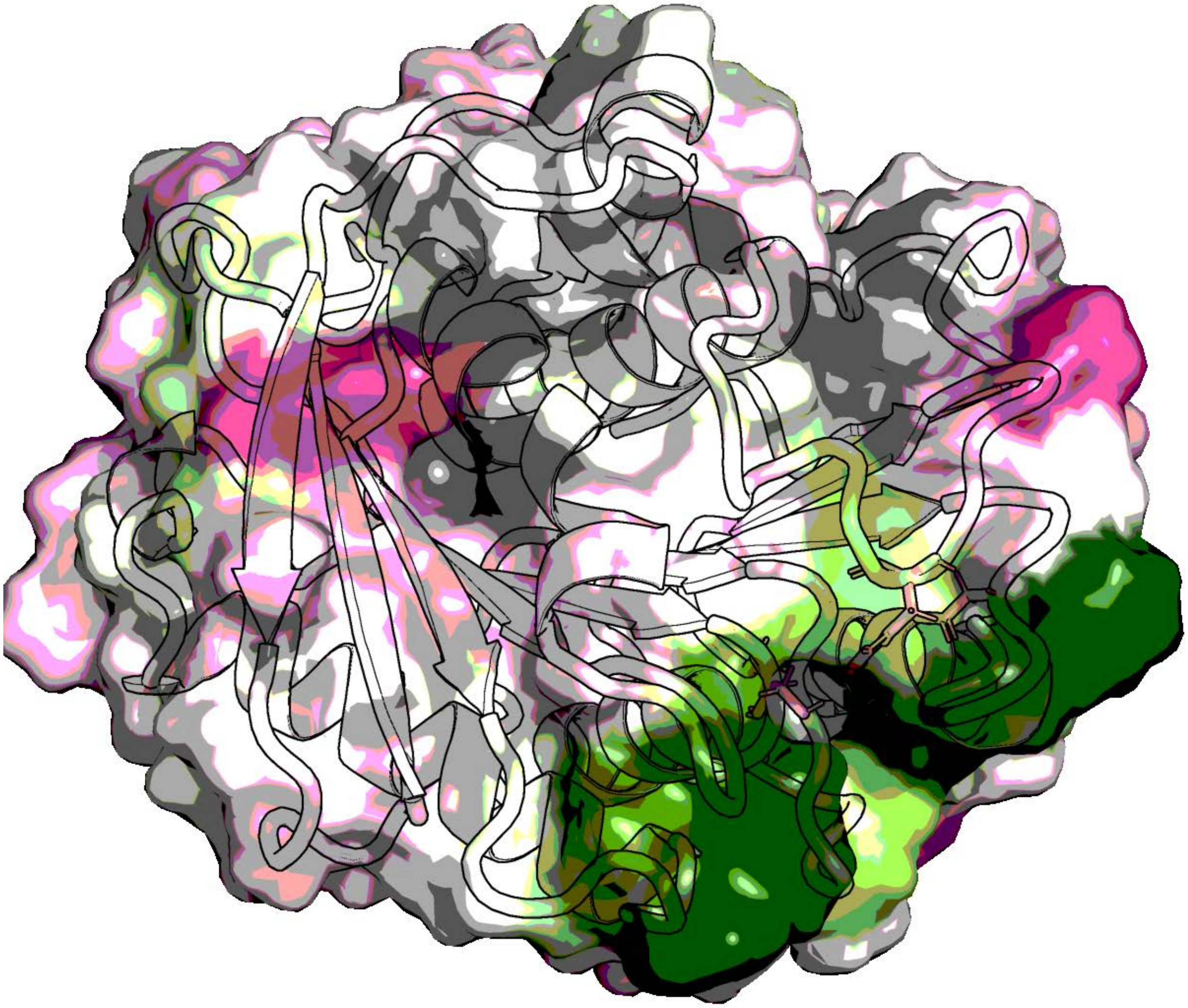

DP017_highlights.png

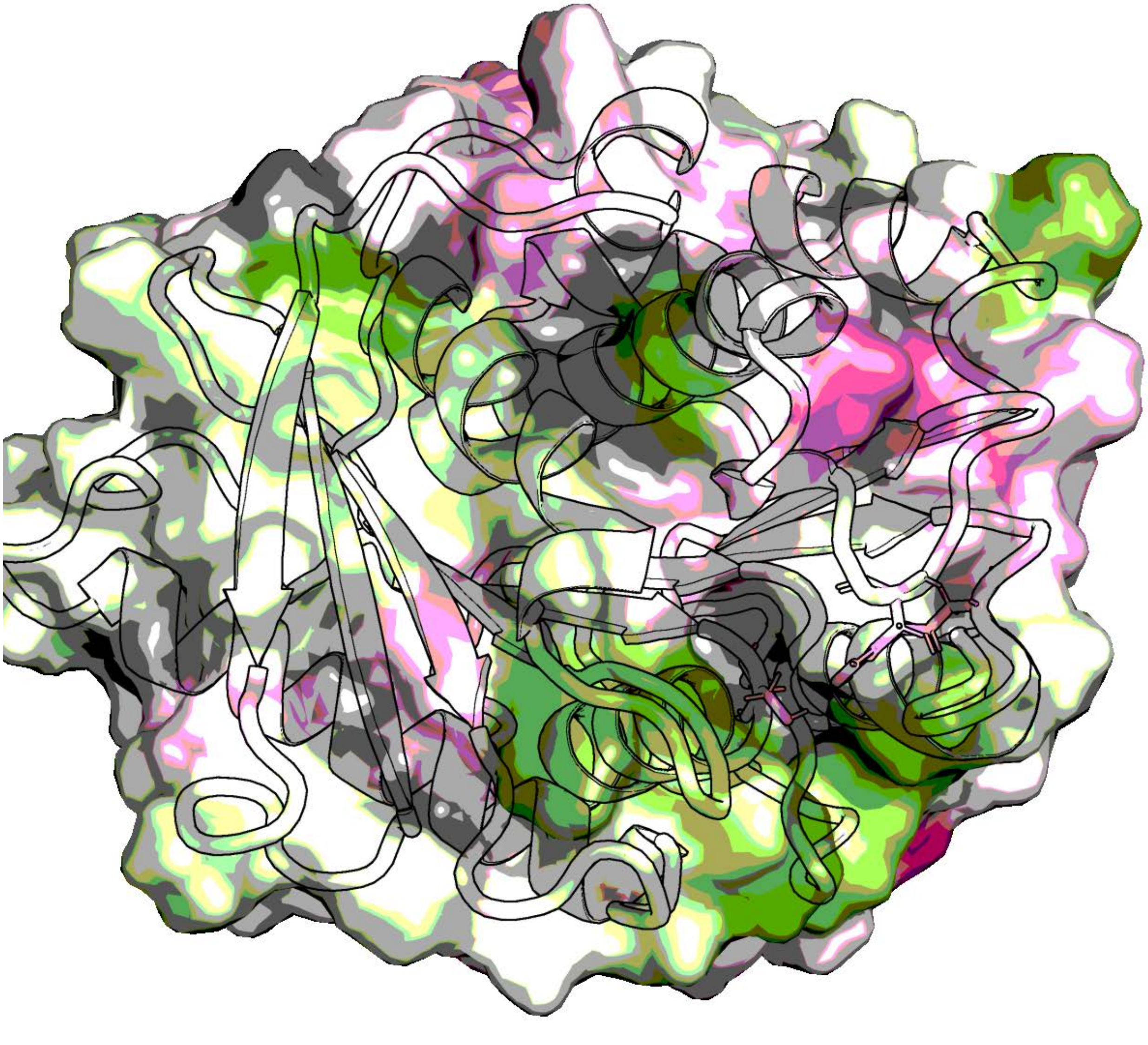

DP038_highlights.png

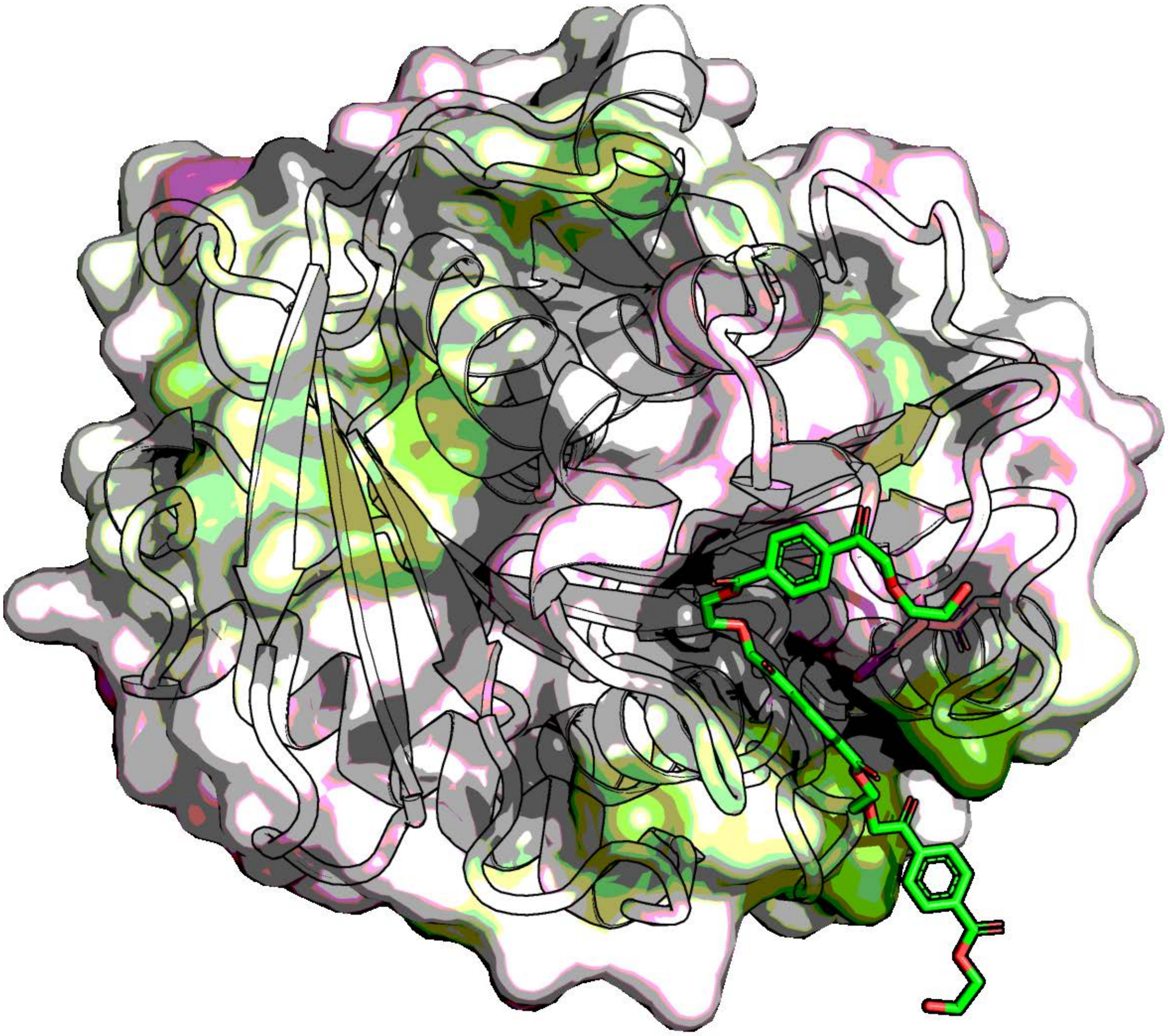

DP043_highlights.png

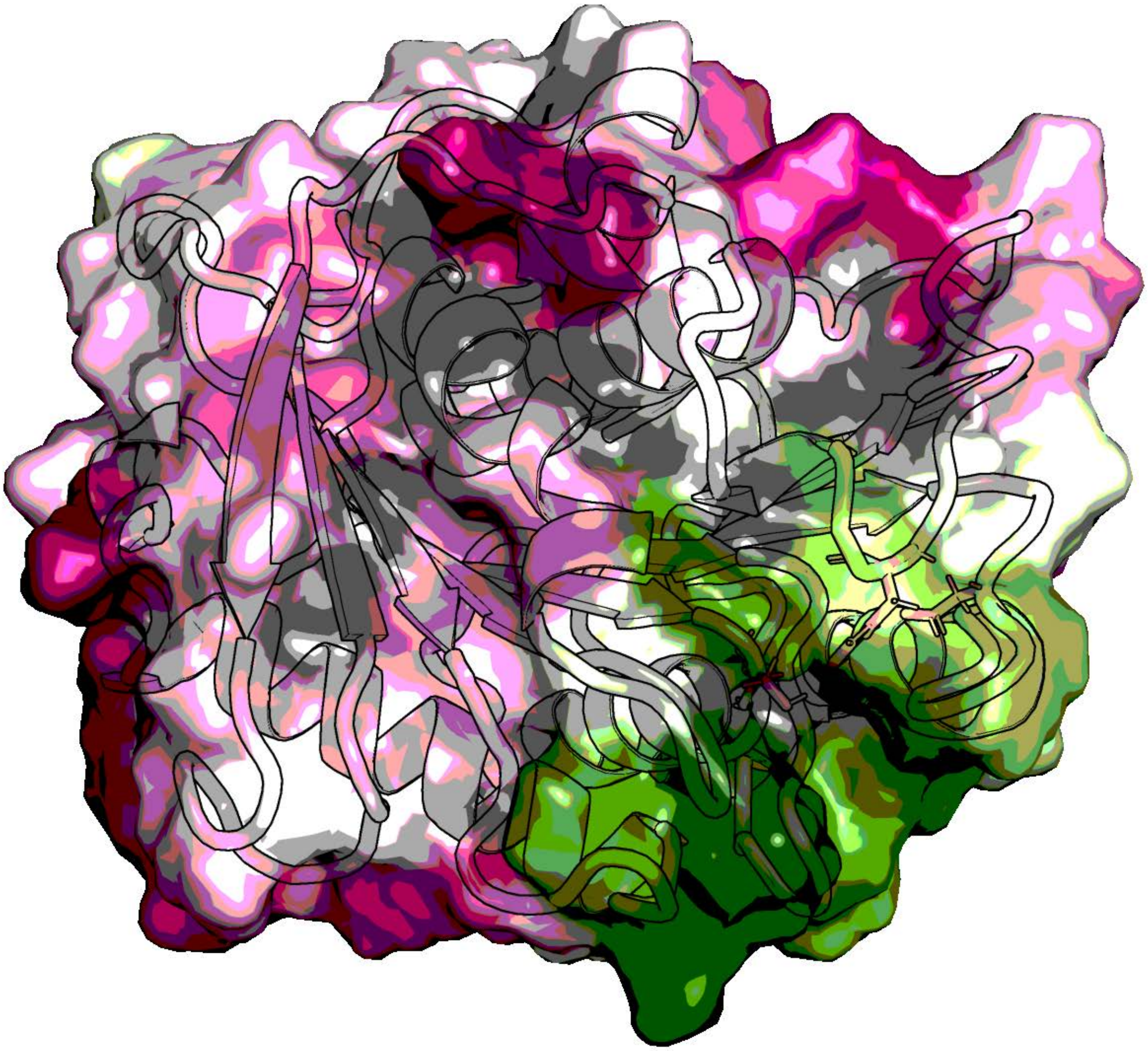

DP054_highlights.png

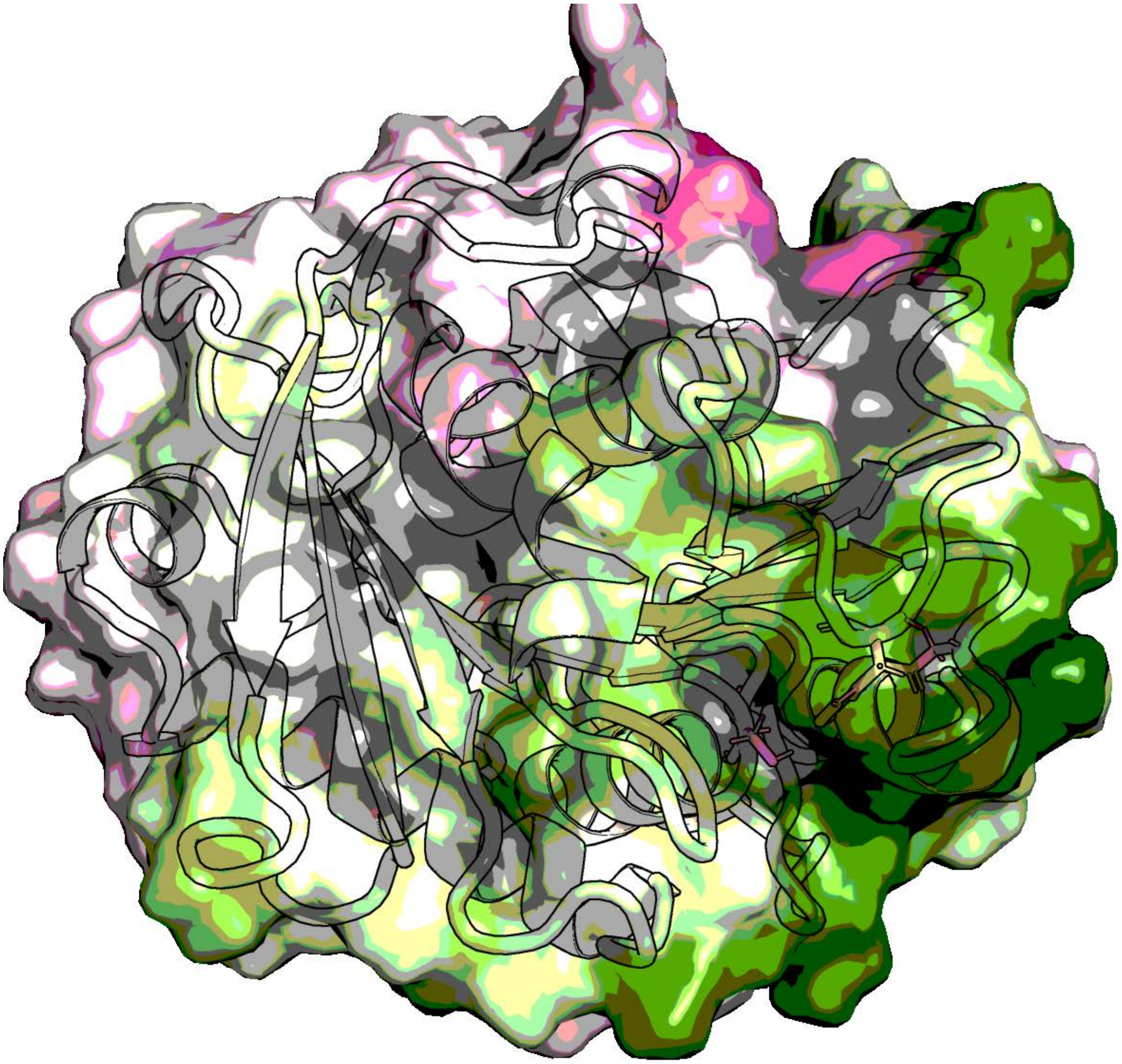

DP056_highlights.png

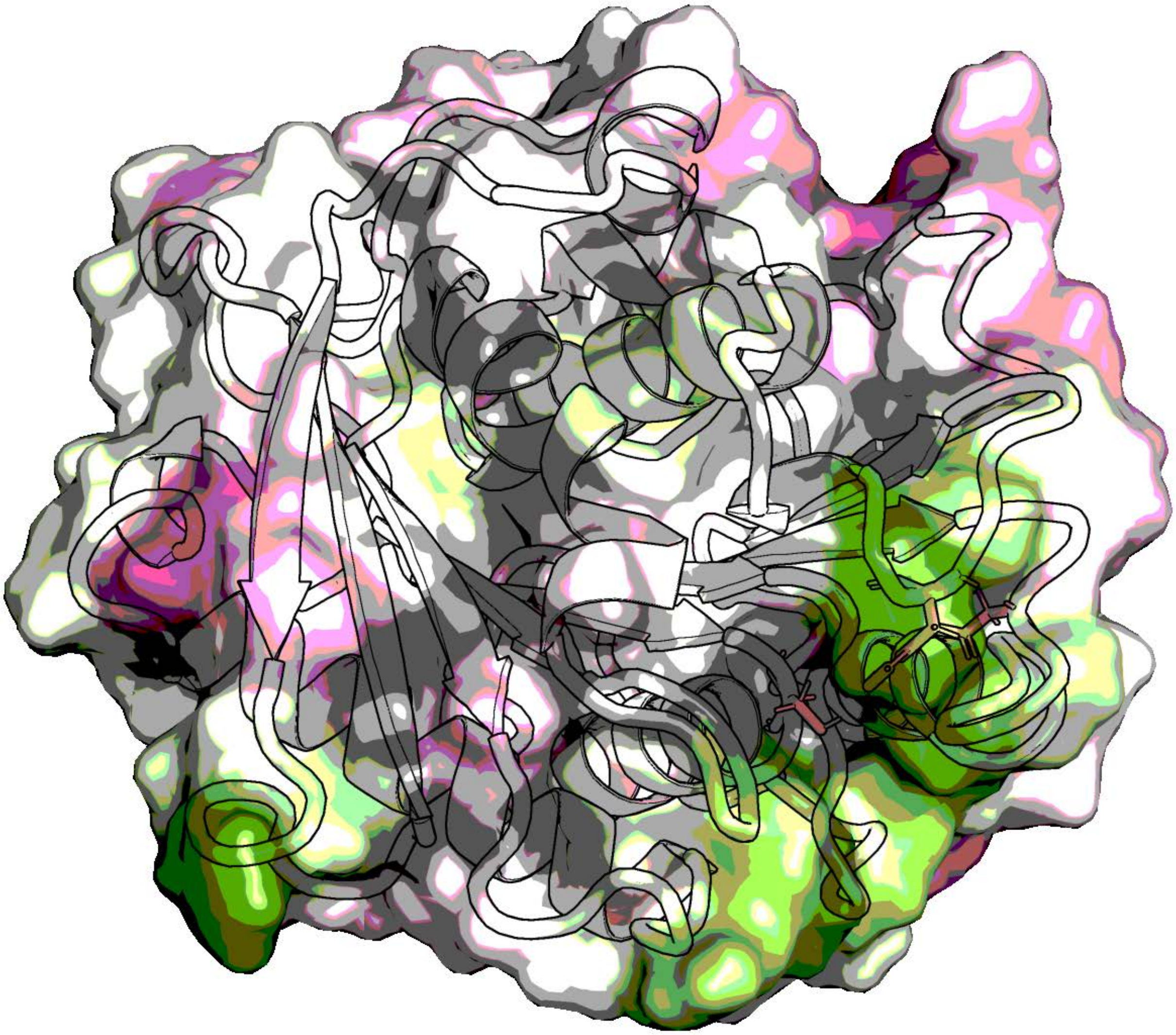

DP057_highlights.png

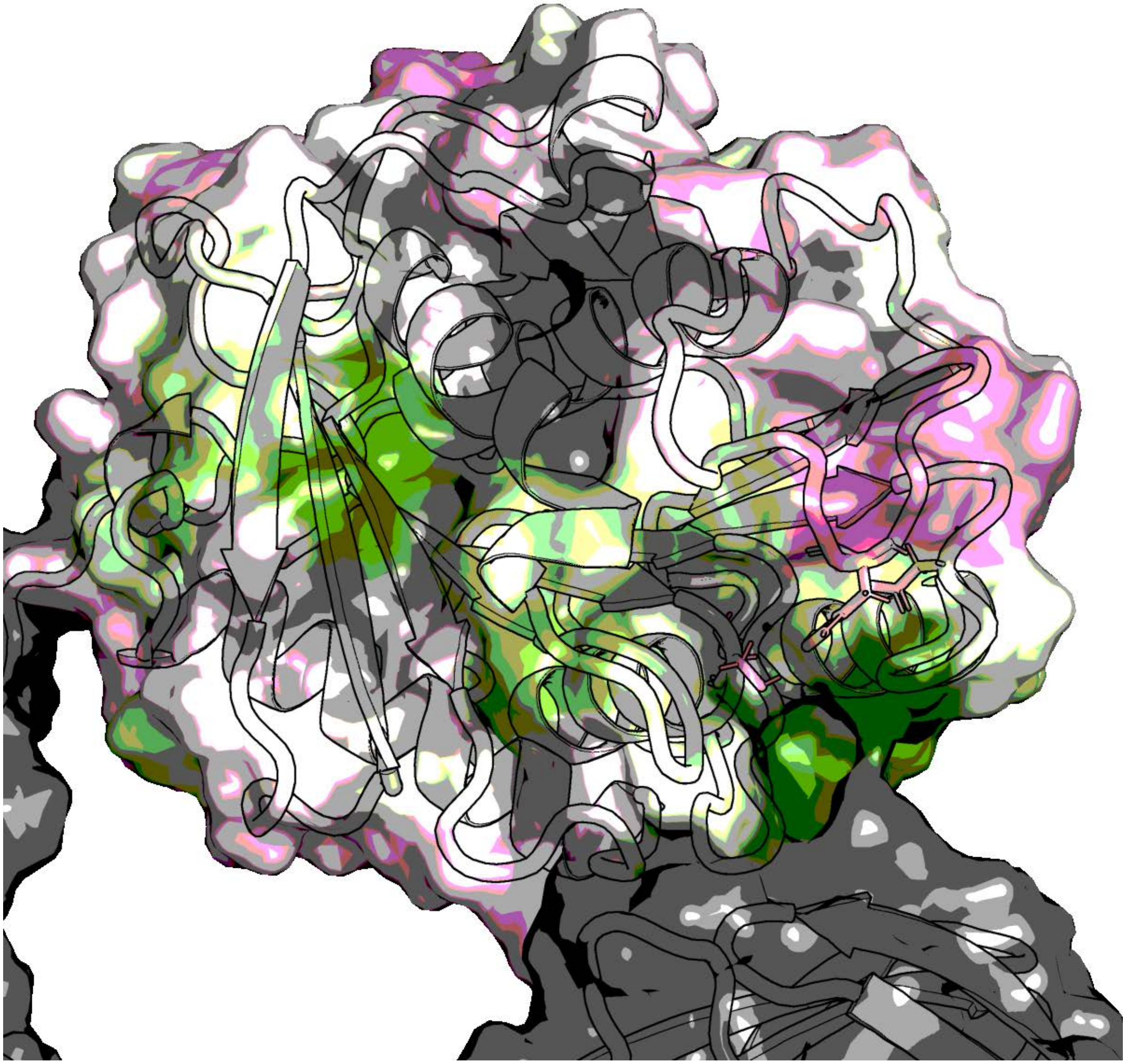

DP059_highlights.png

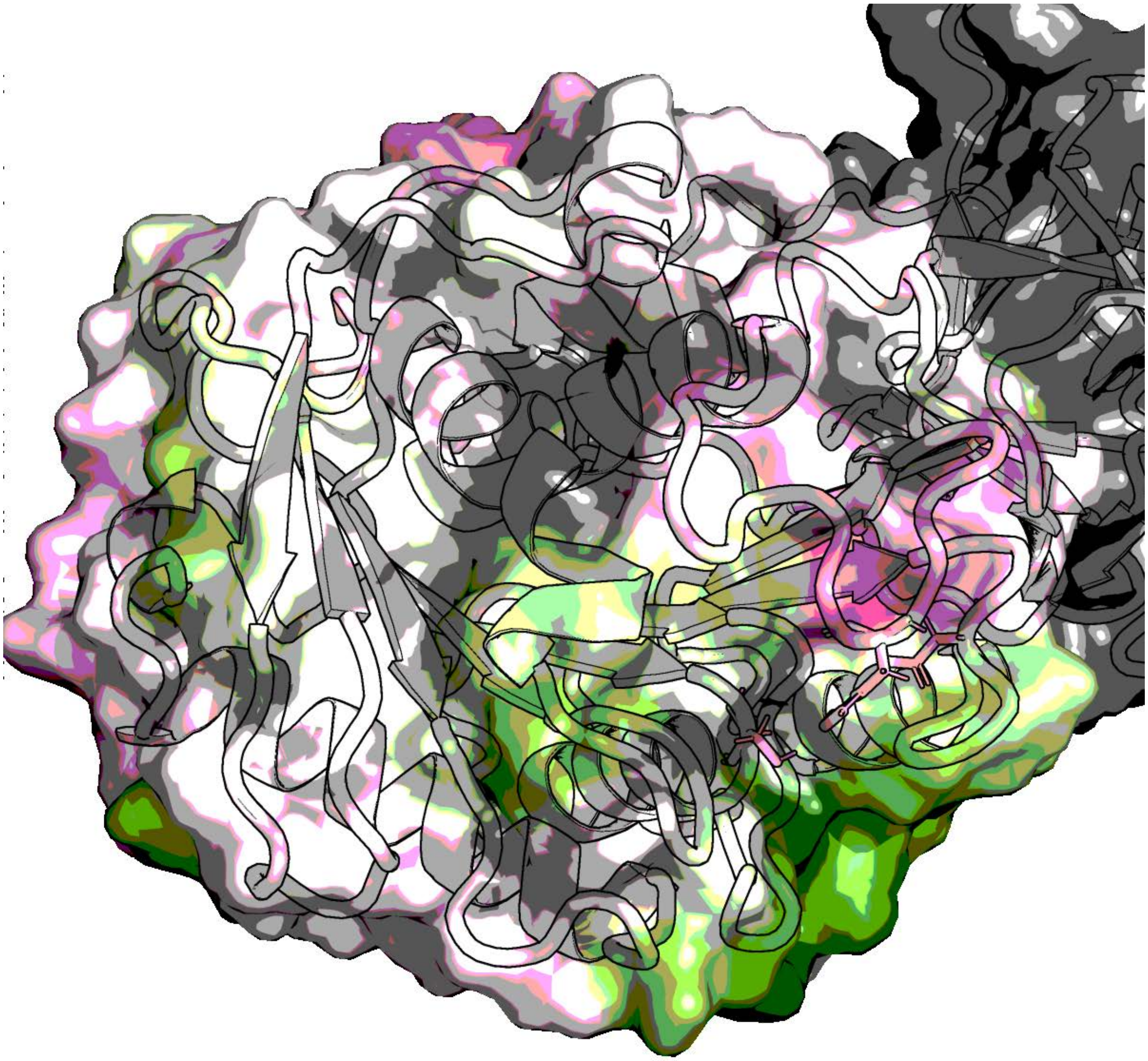

DP061_highlights.png

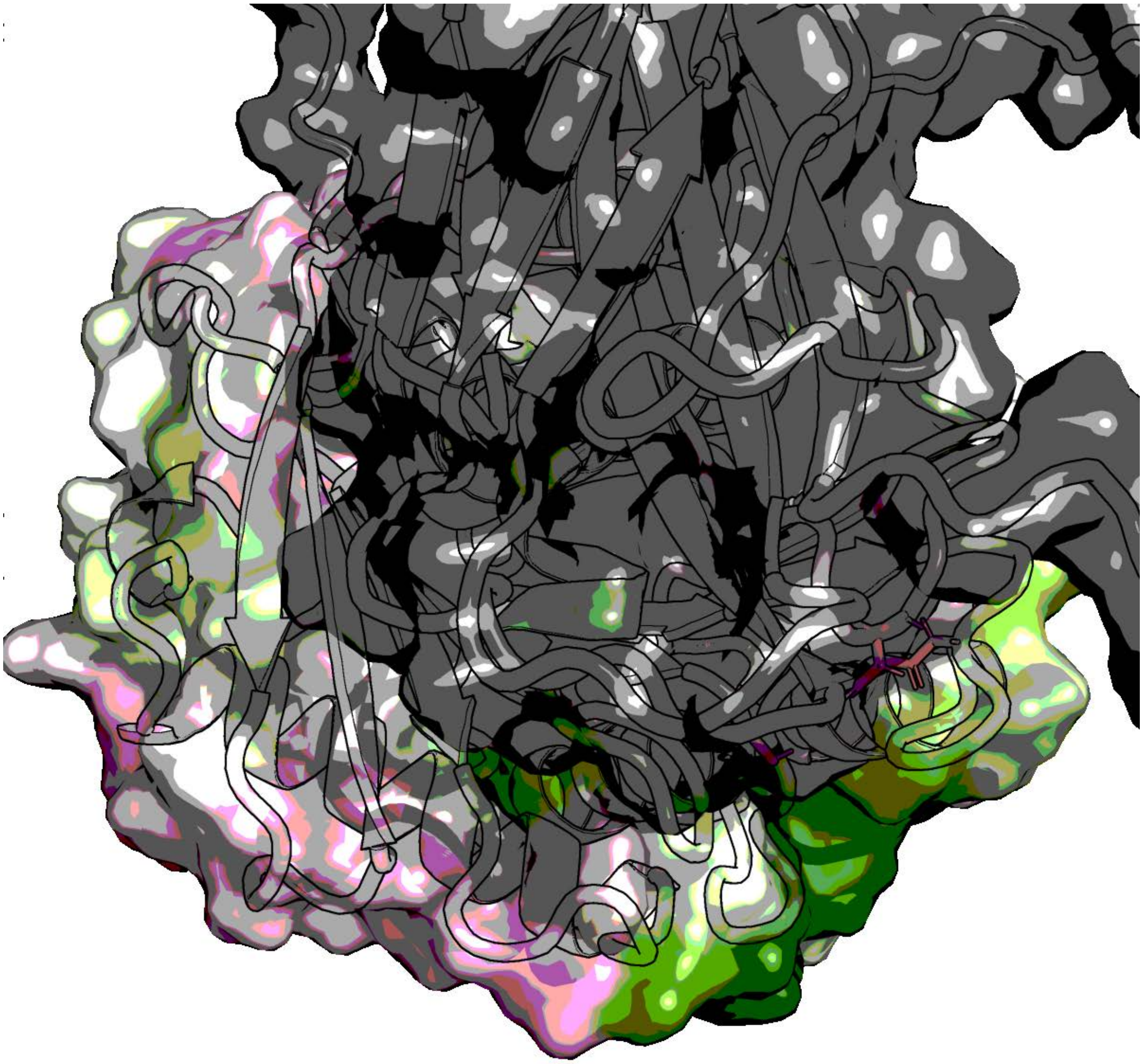

DP068_highlights.png

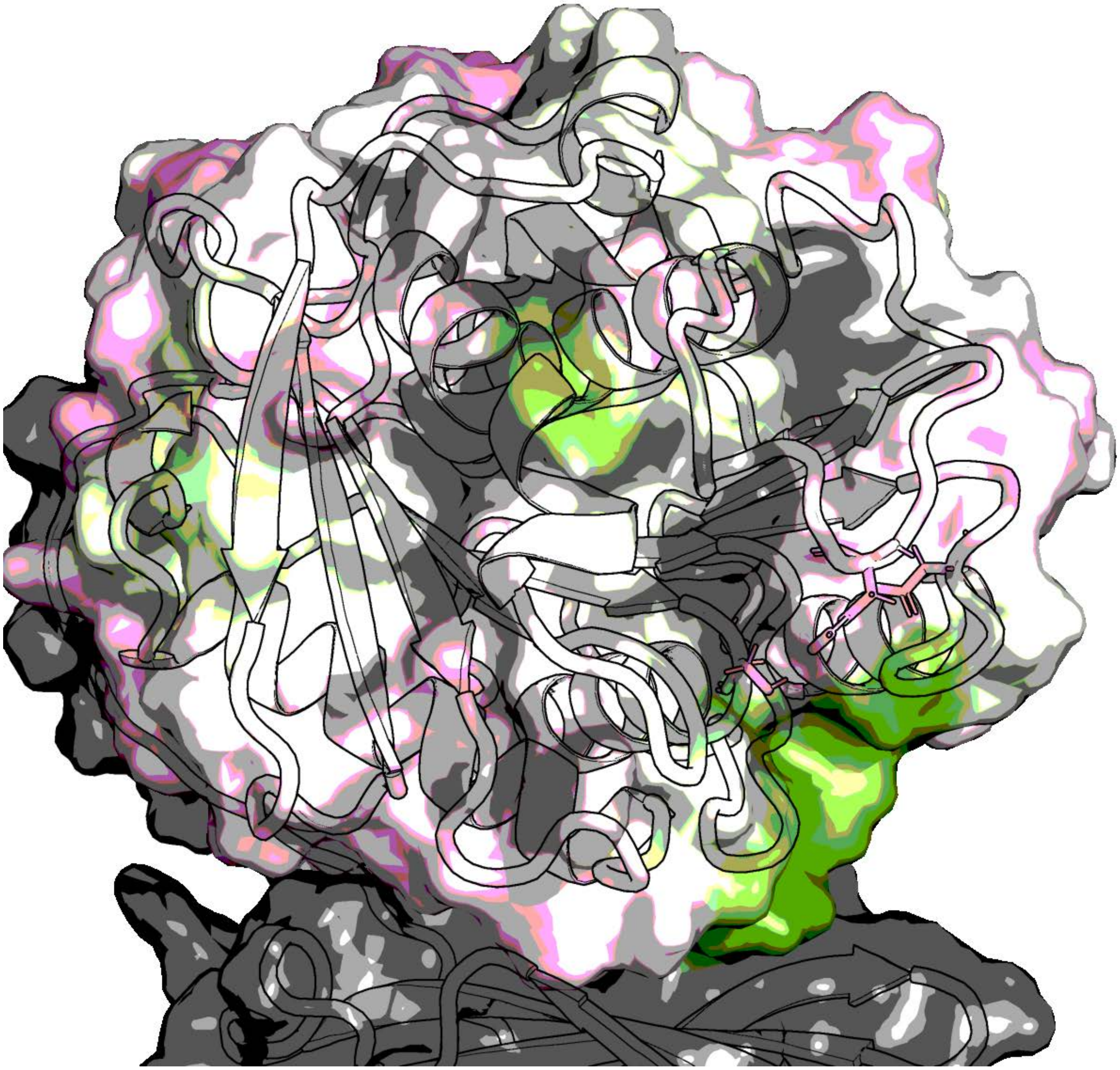

DP072_highlights.png

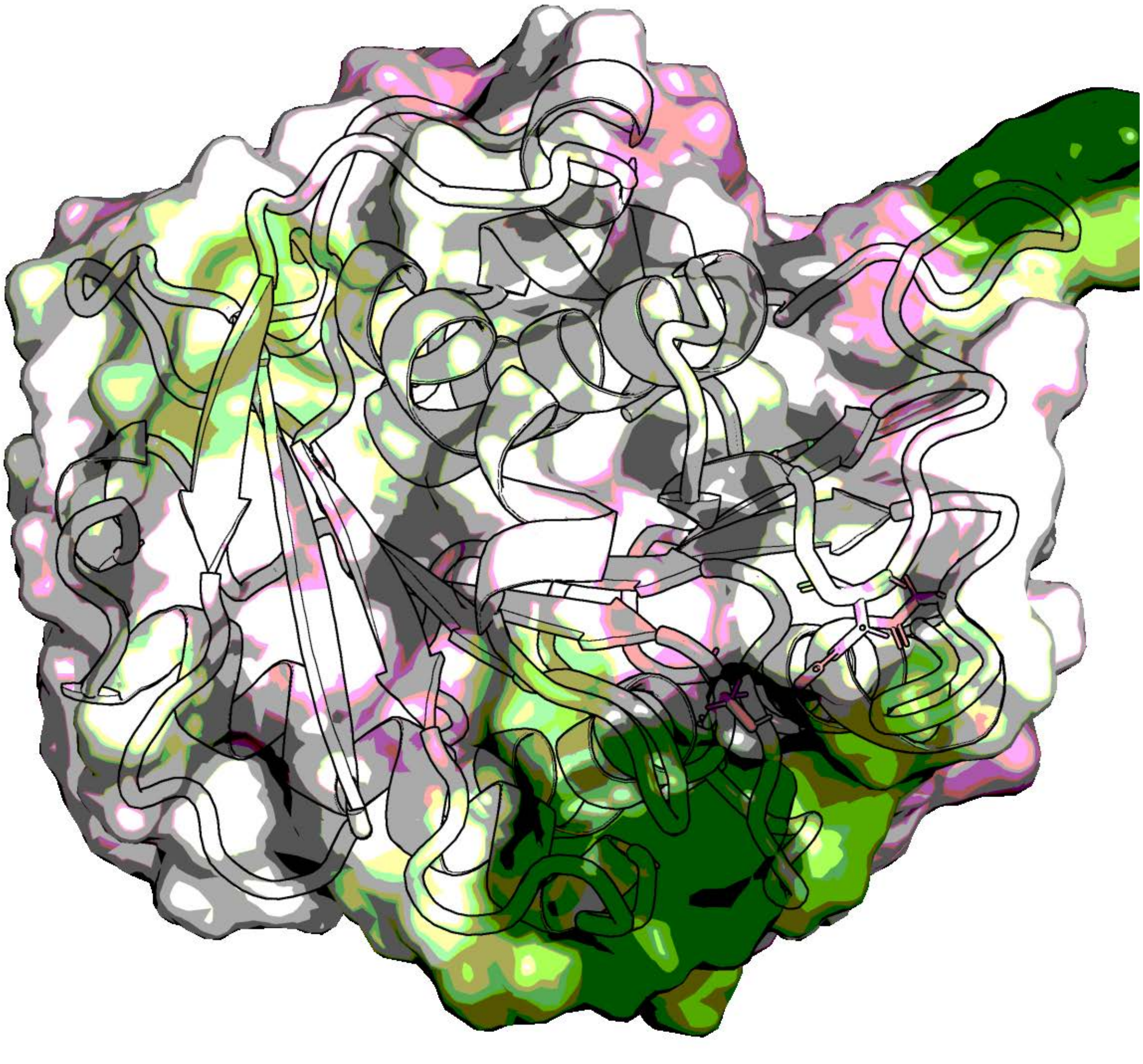

DP077_highlights.png

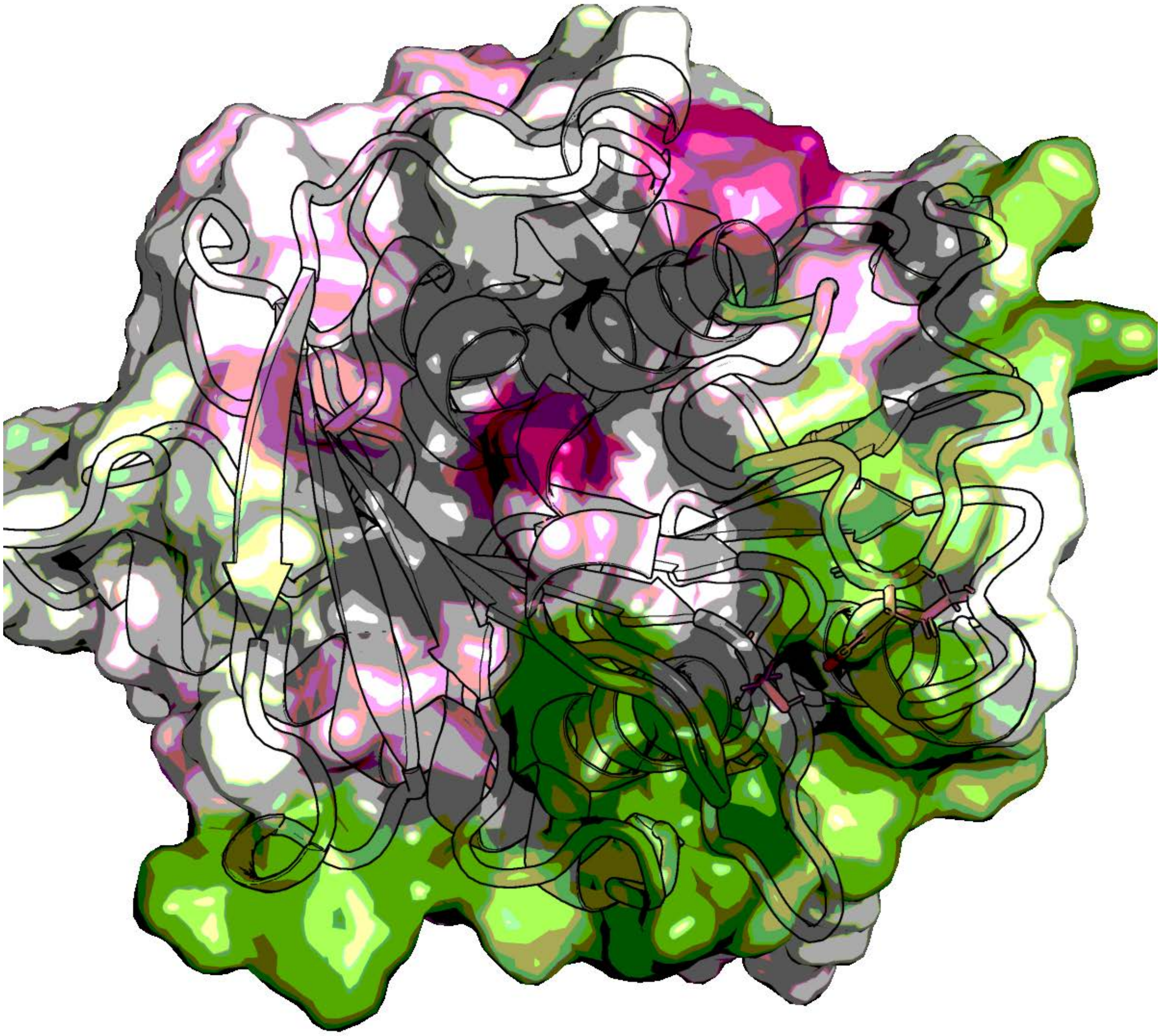

DP078_highlights.png

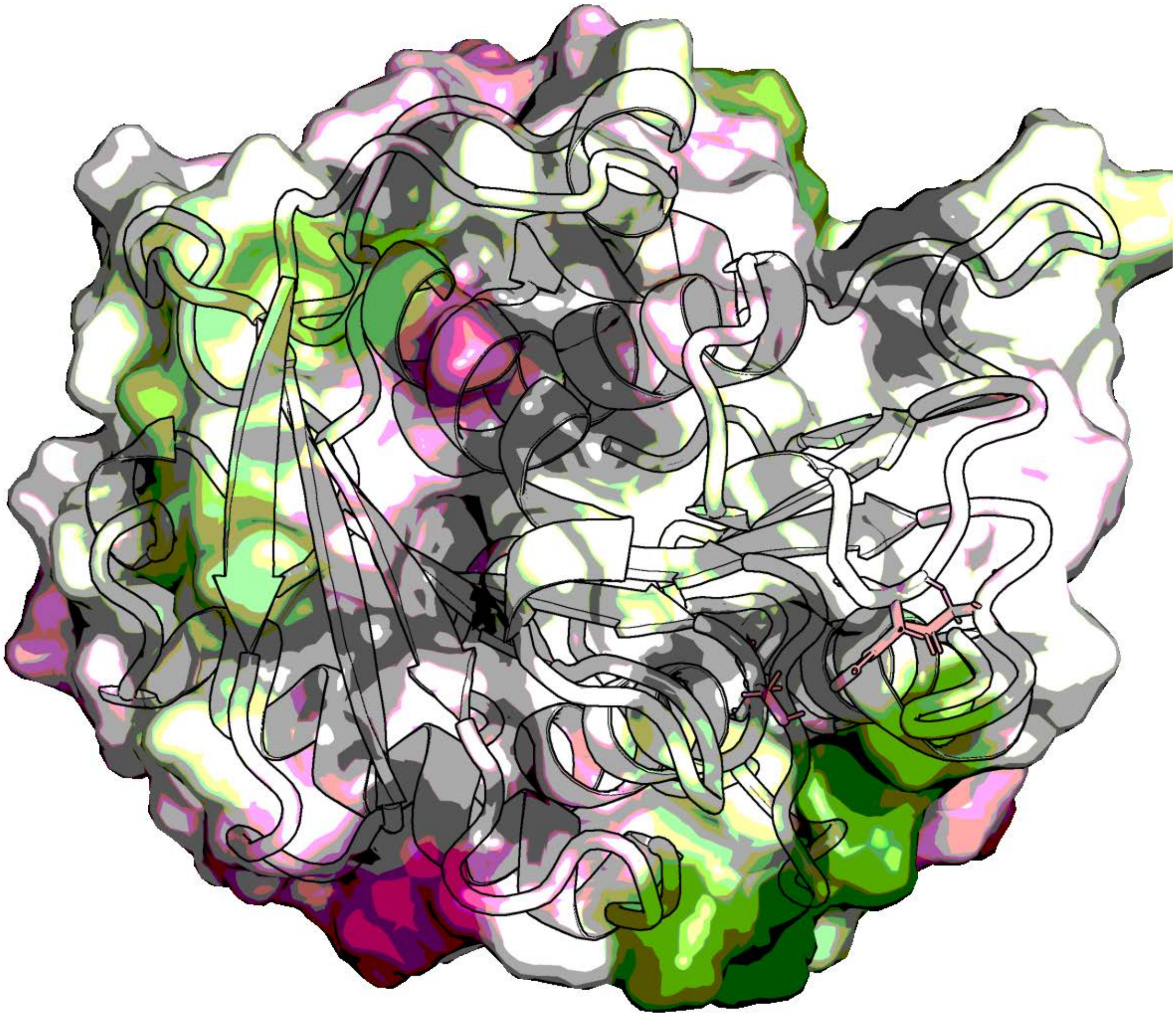

DP080_highlights.png

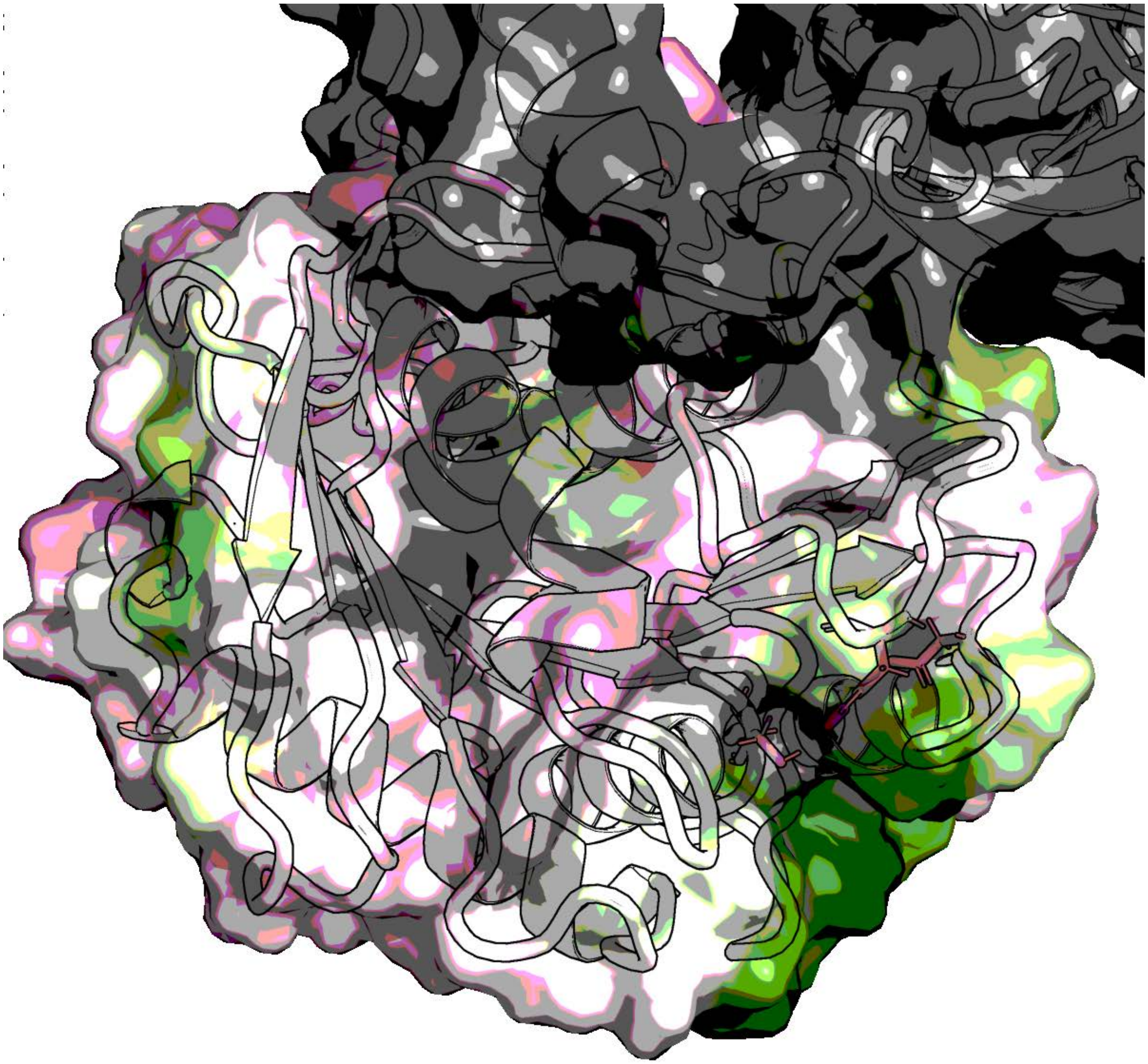

DP081_highlights.png

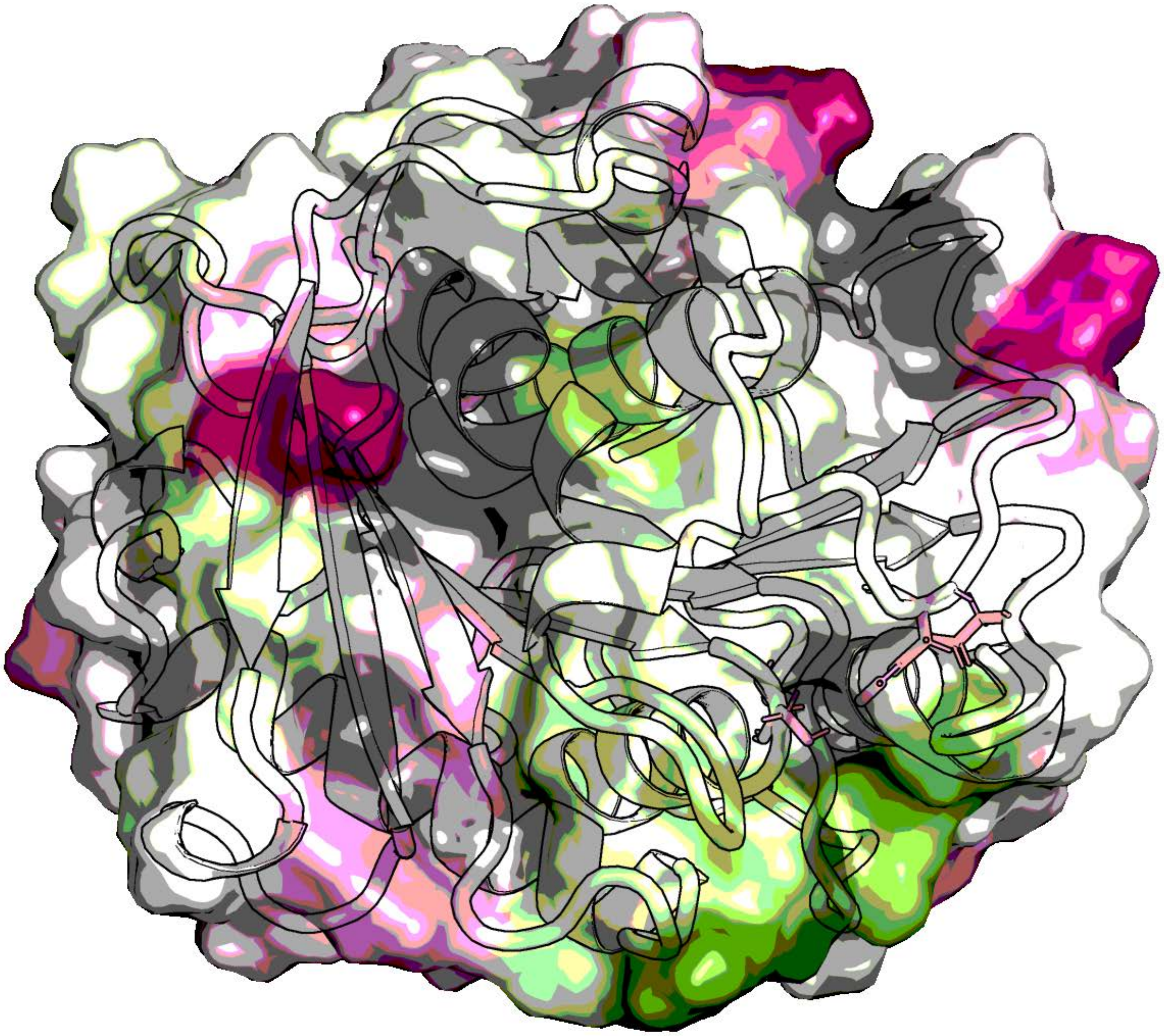

DP082_highlights.png

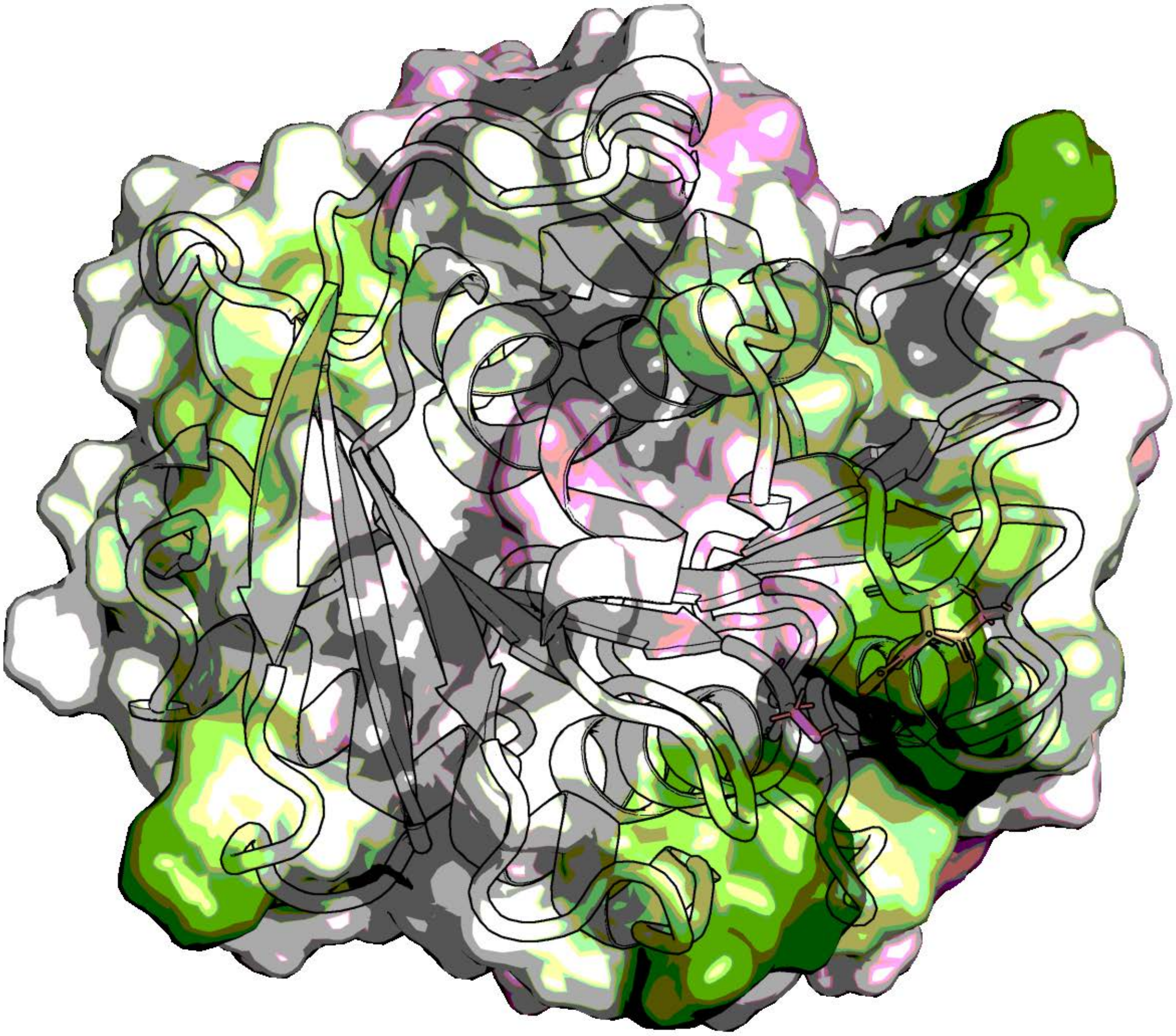

DP084_highlights.png

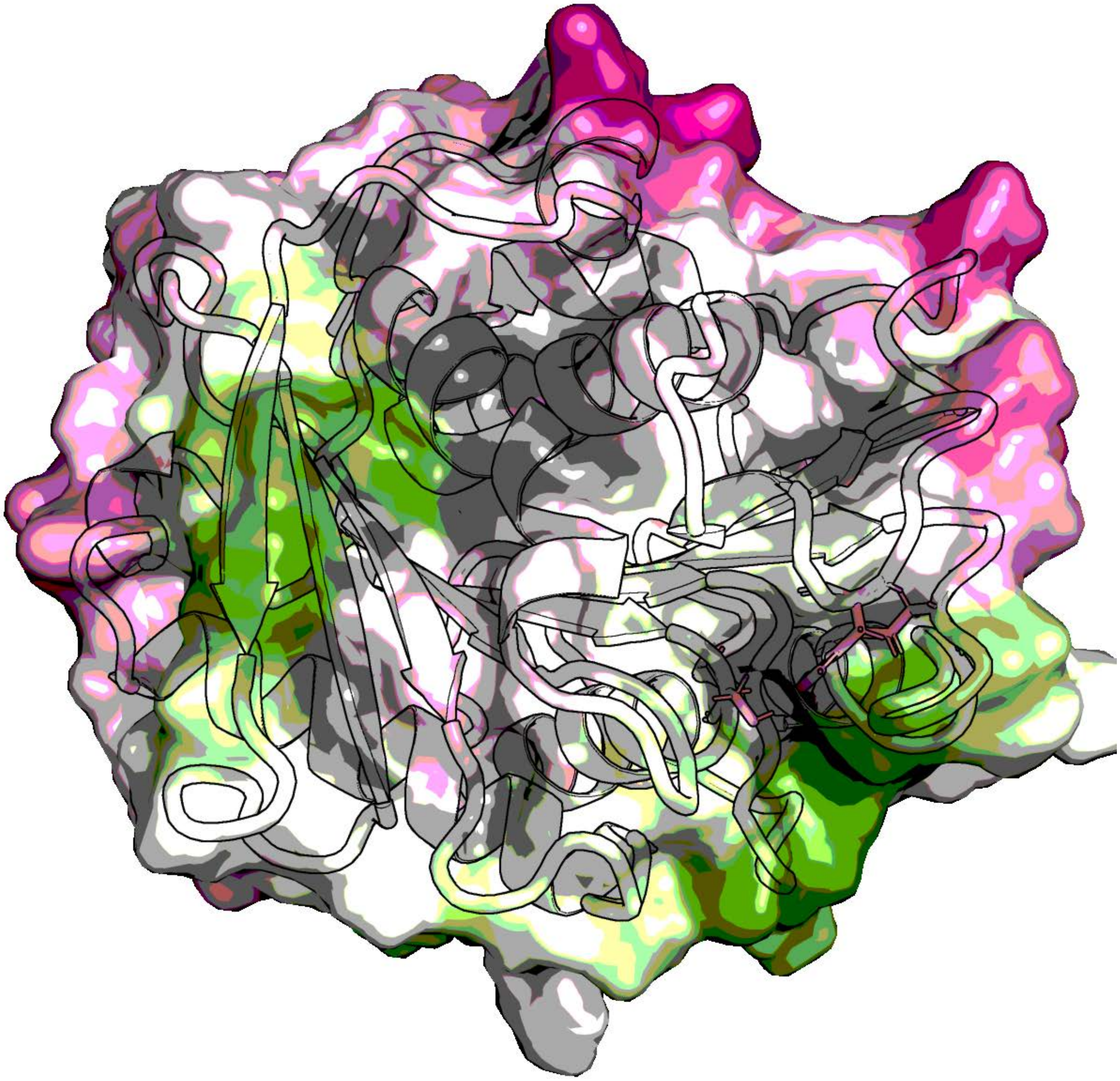

DP089_highlights.png

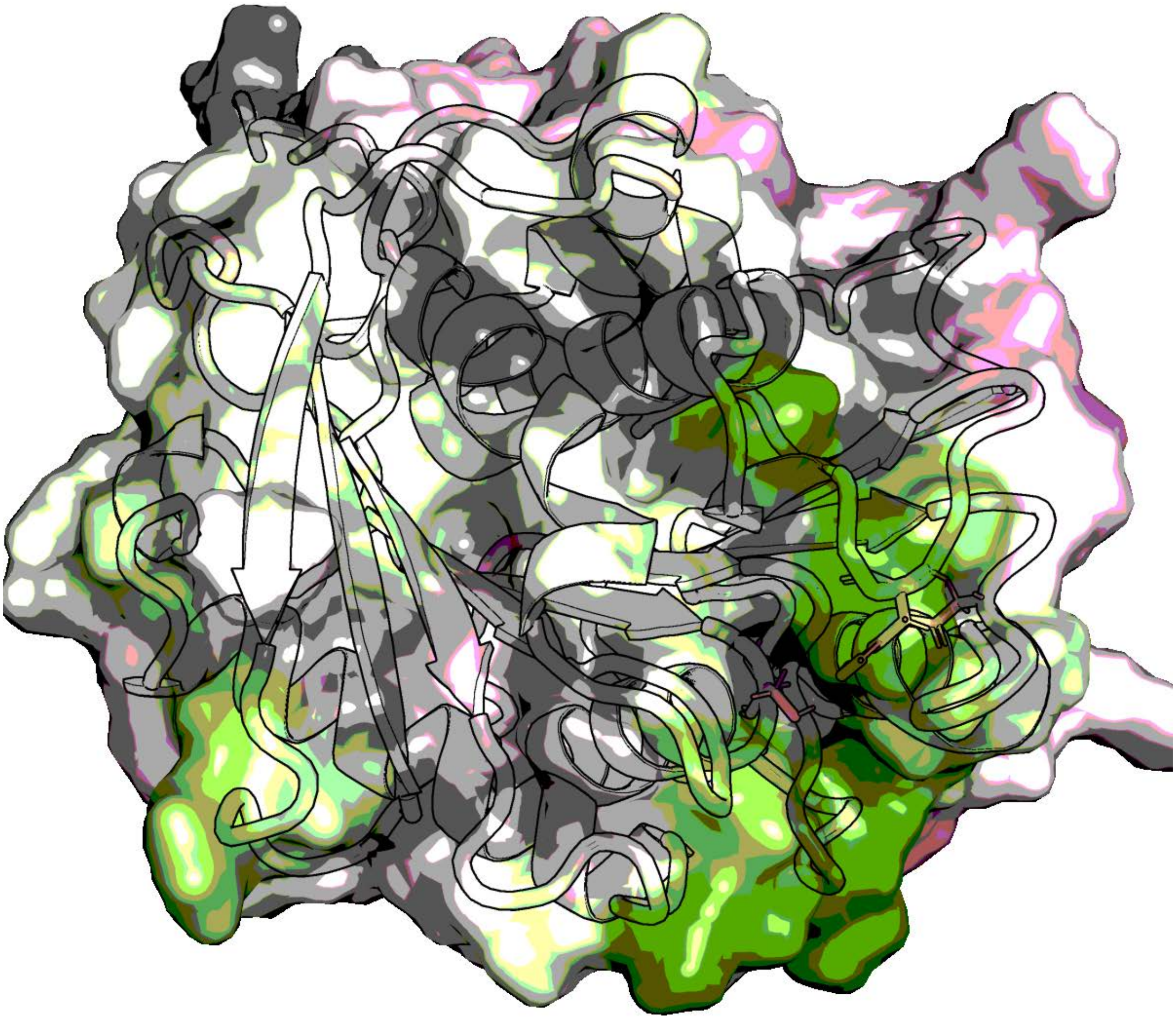

DP090_highlights.png

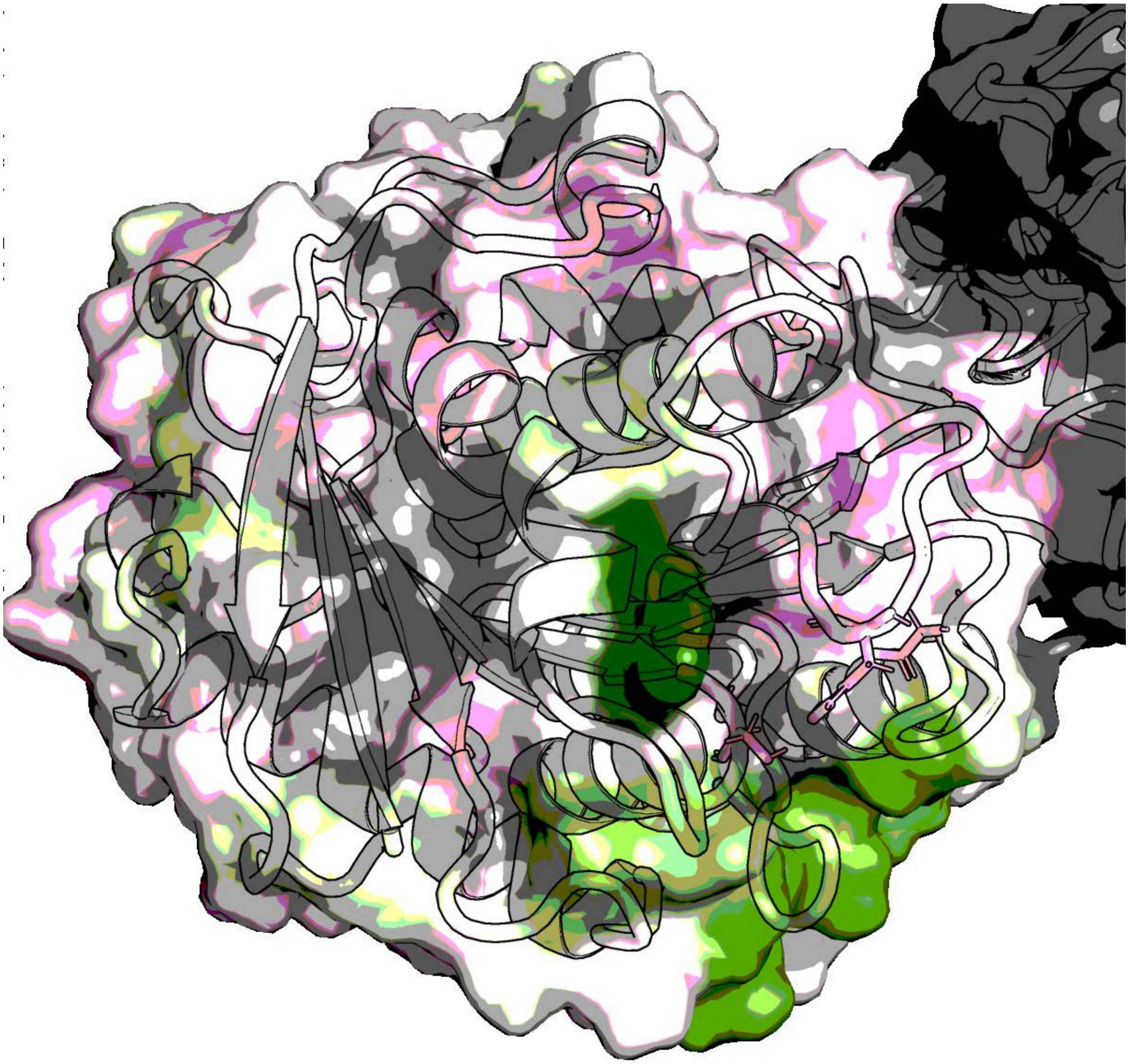

DP092_highlights.png

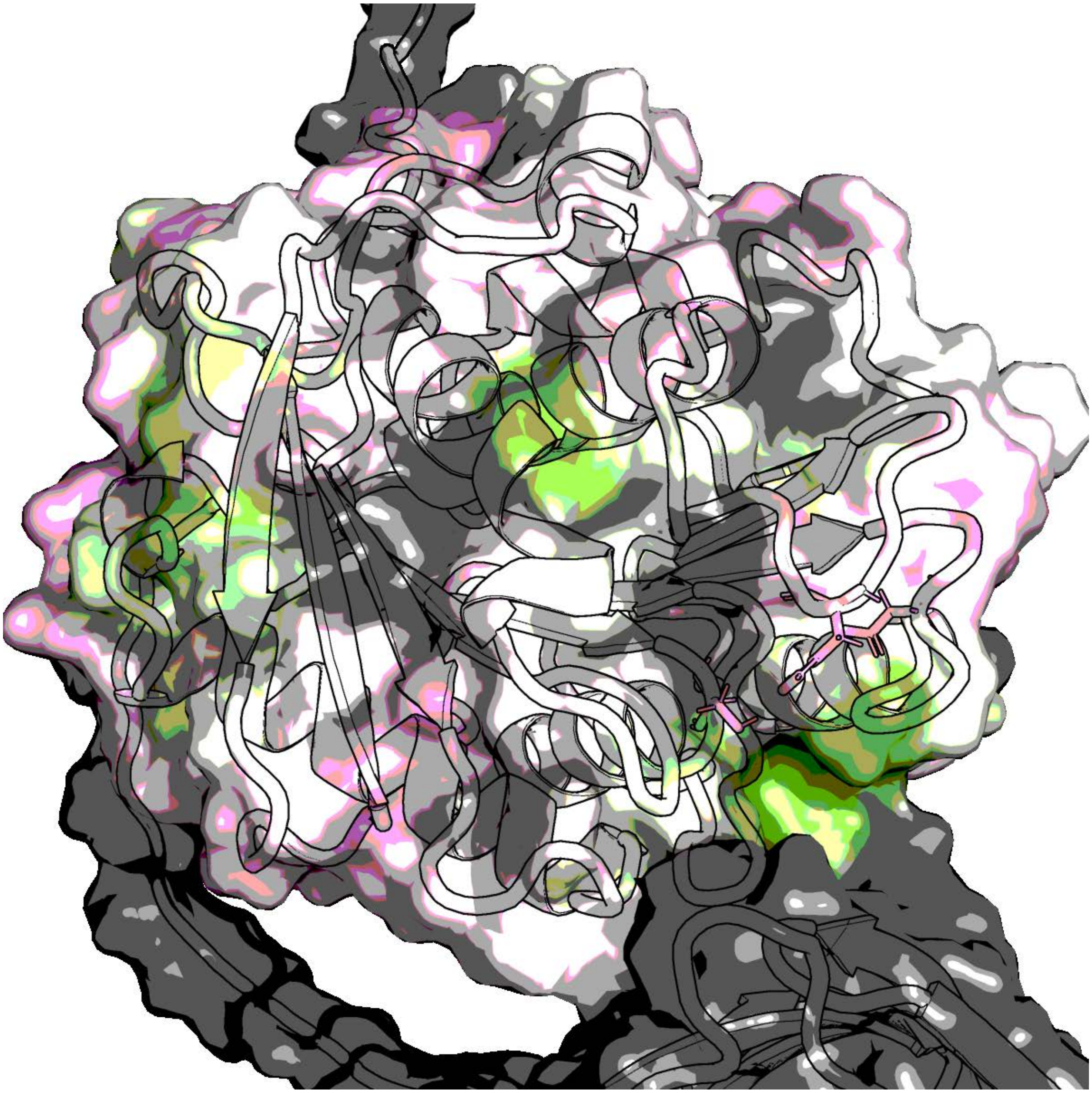

ESM006_highlights.png

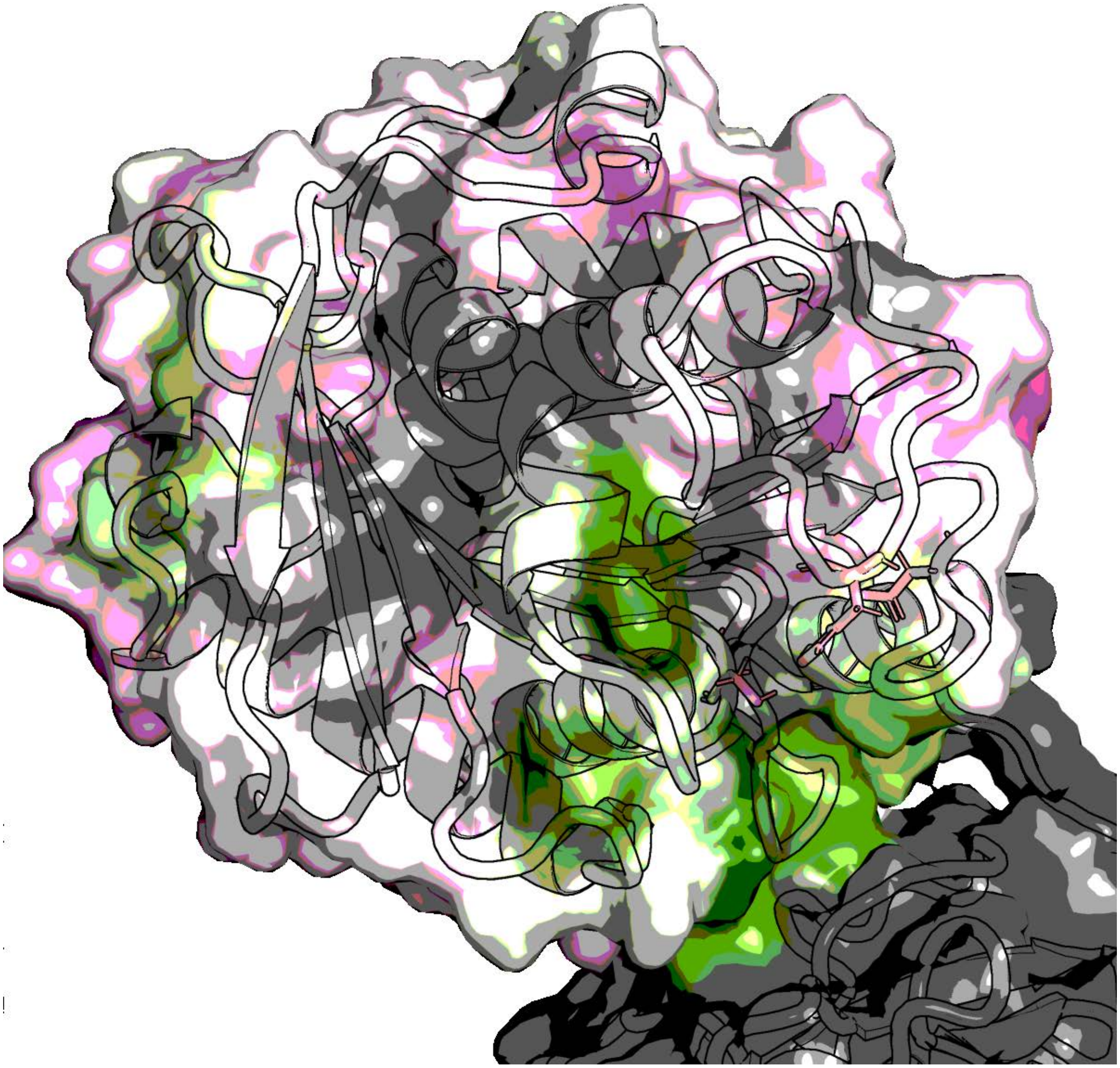

ESM010_highlights.png

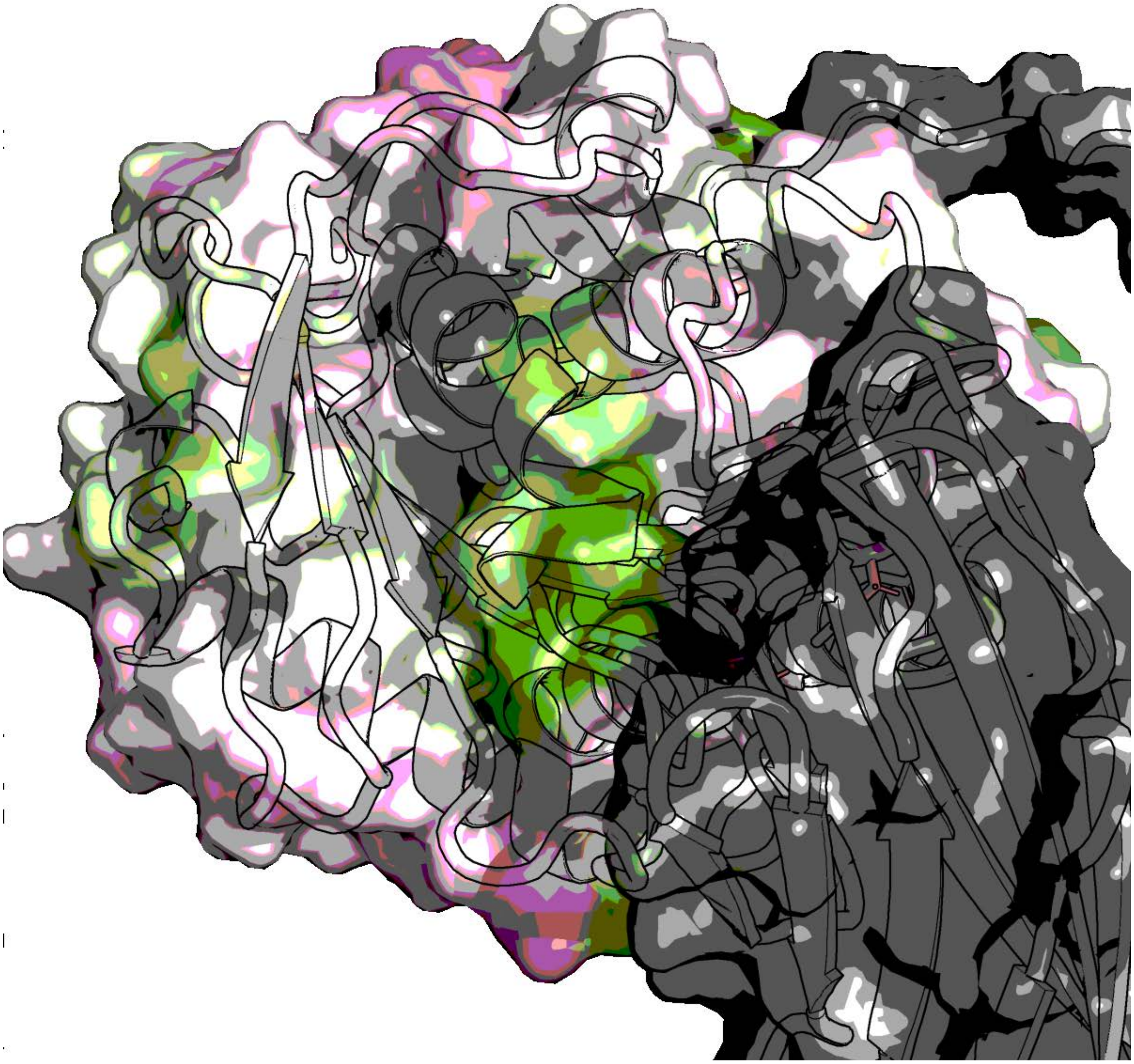

ESM012_highlights.png

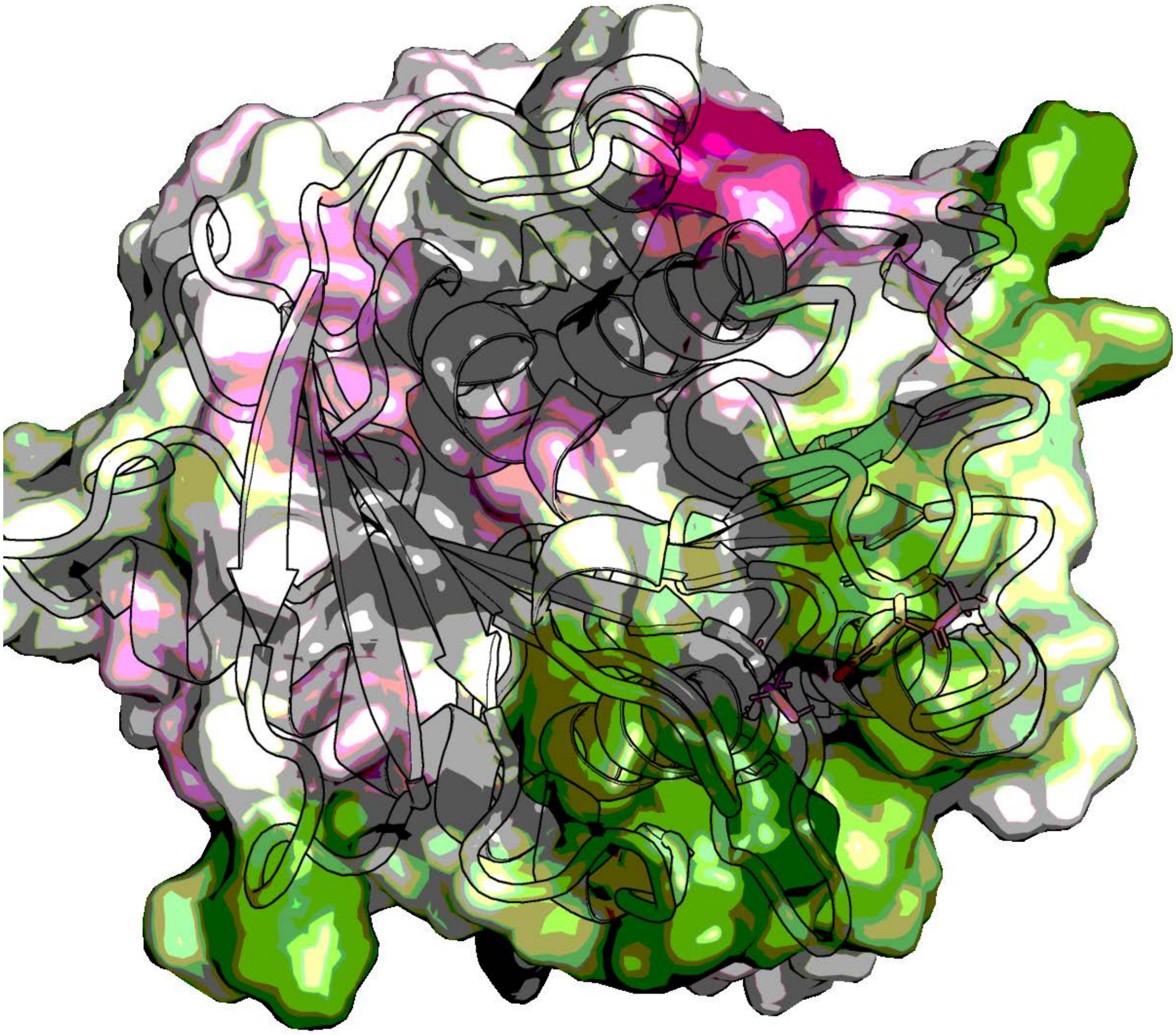

ESM017_highlights.png

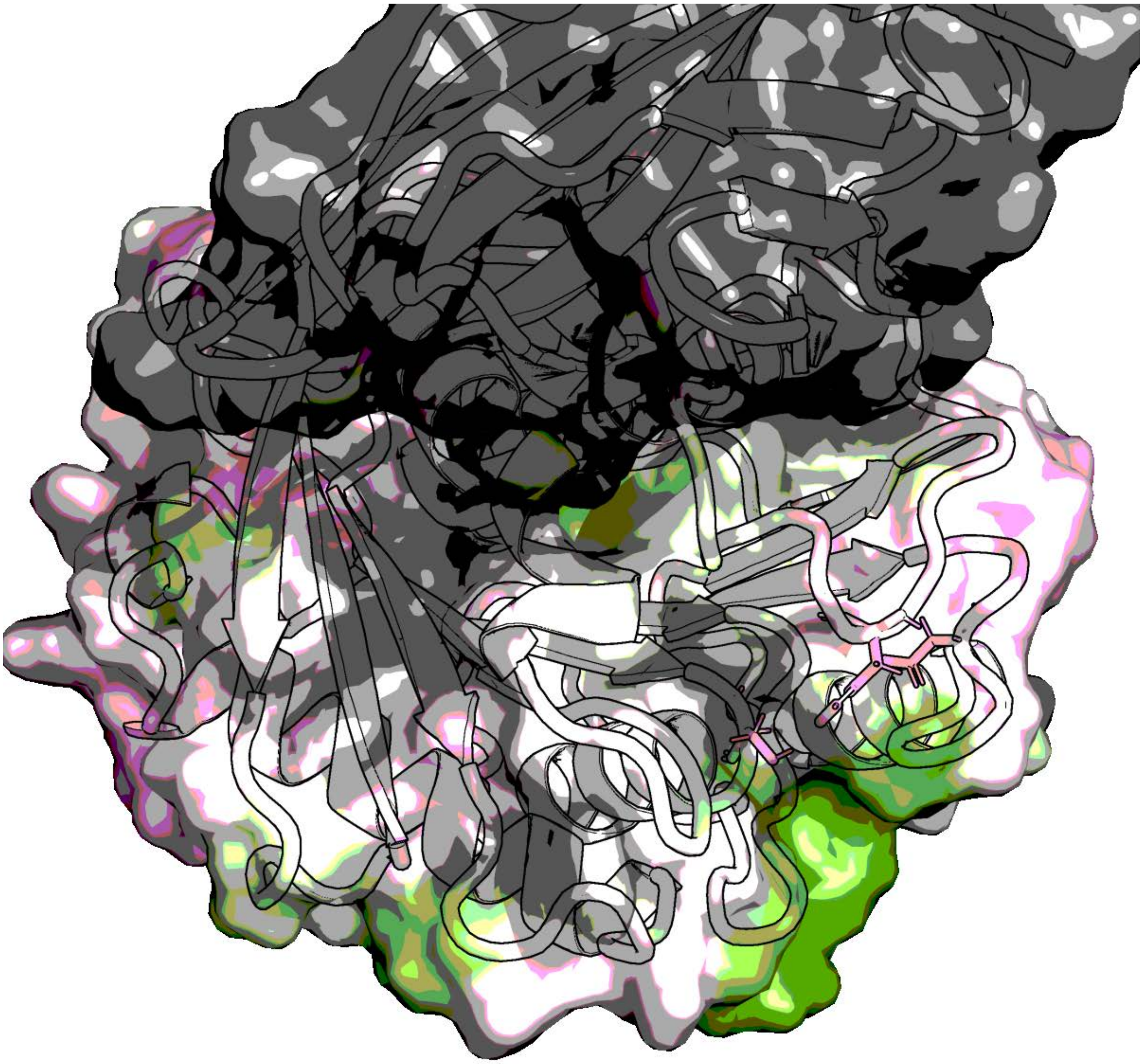

ESM018_highlights.png

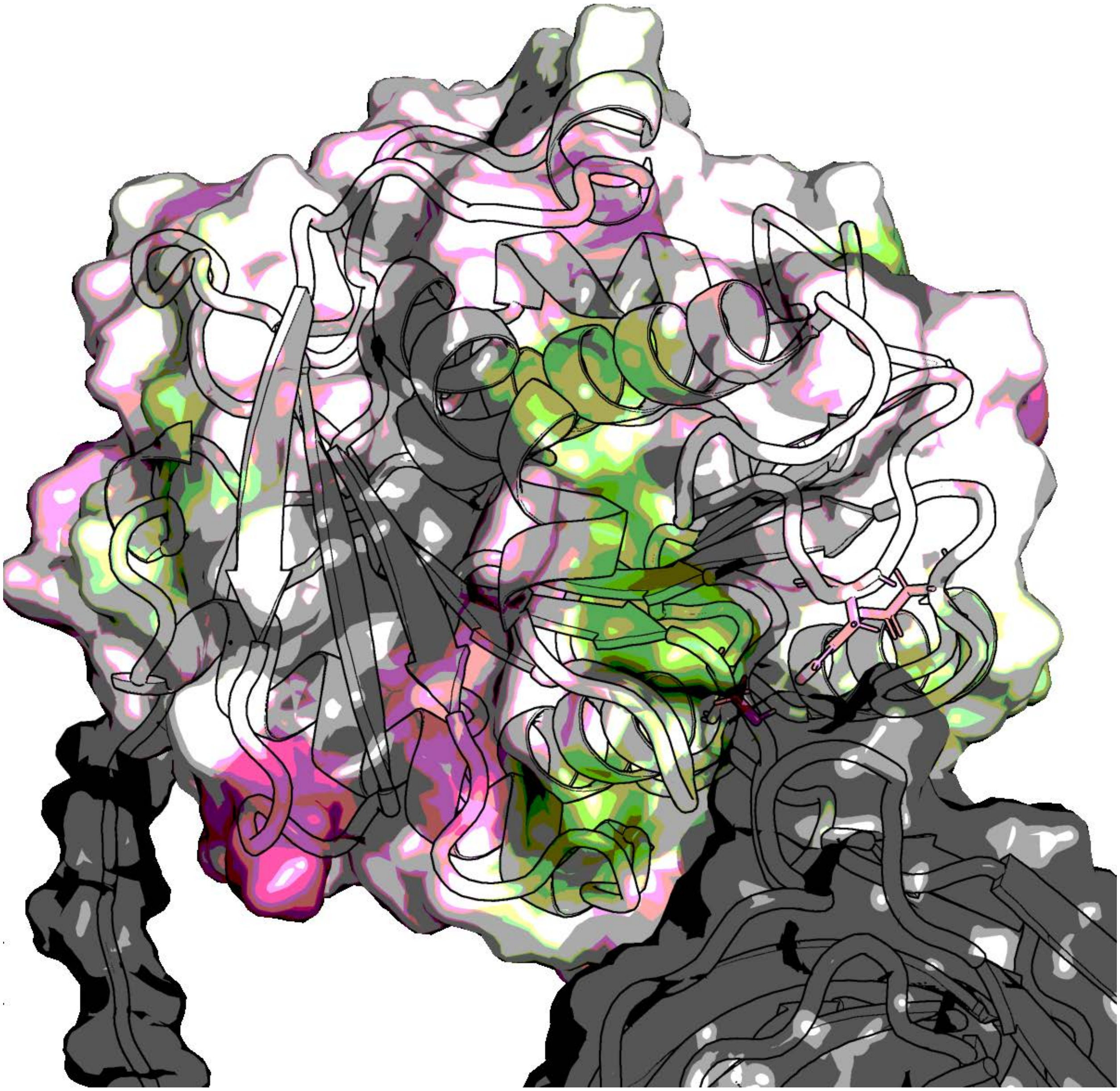

ESM021_highlights.png

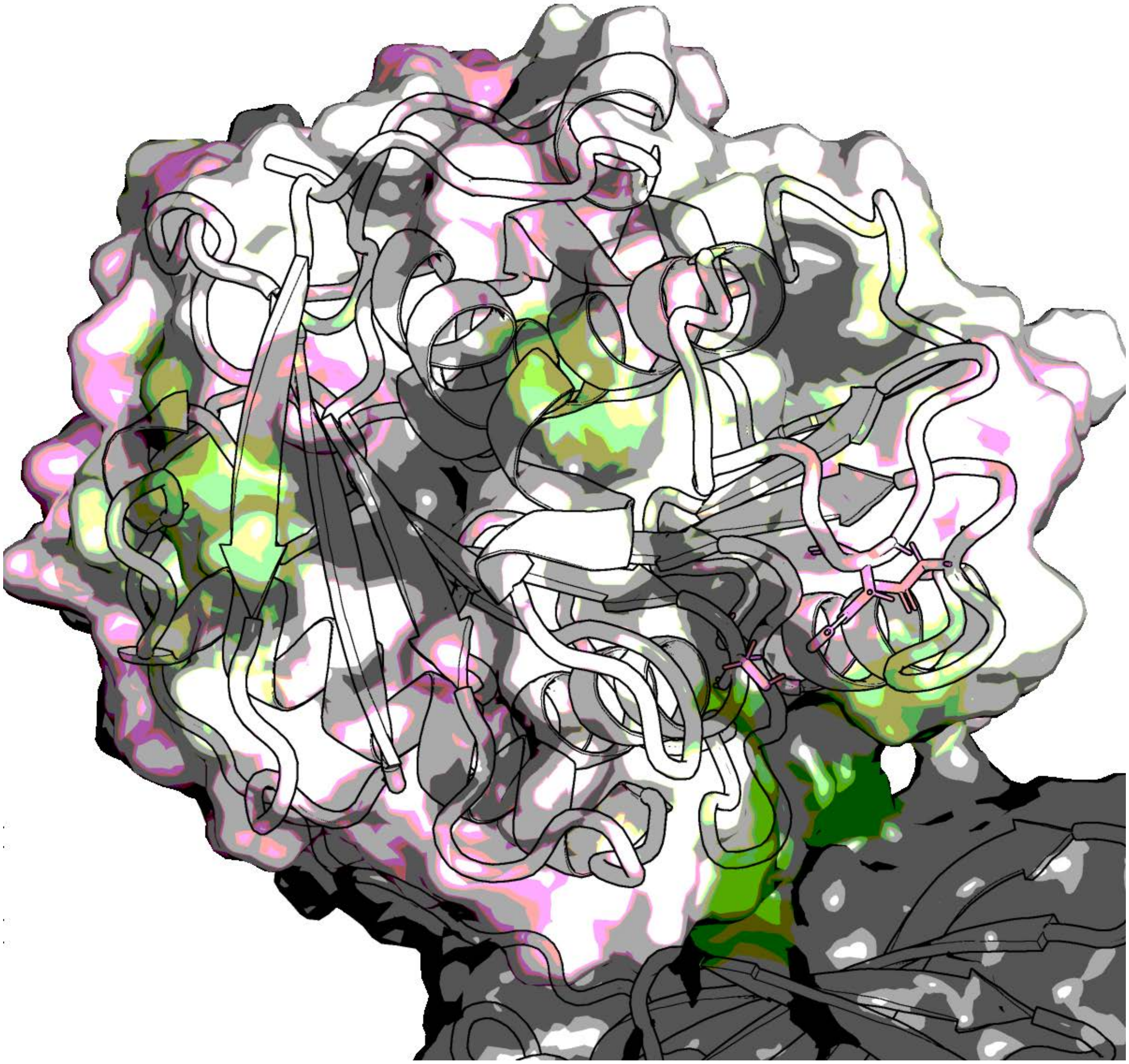

ESM023_highlights.png

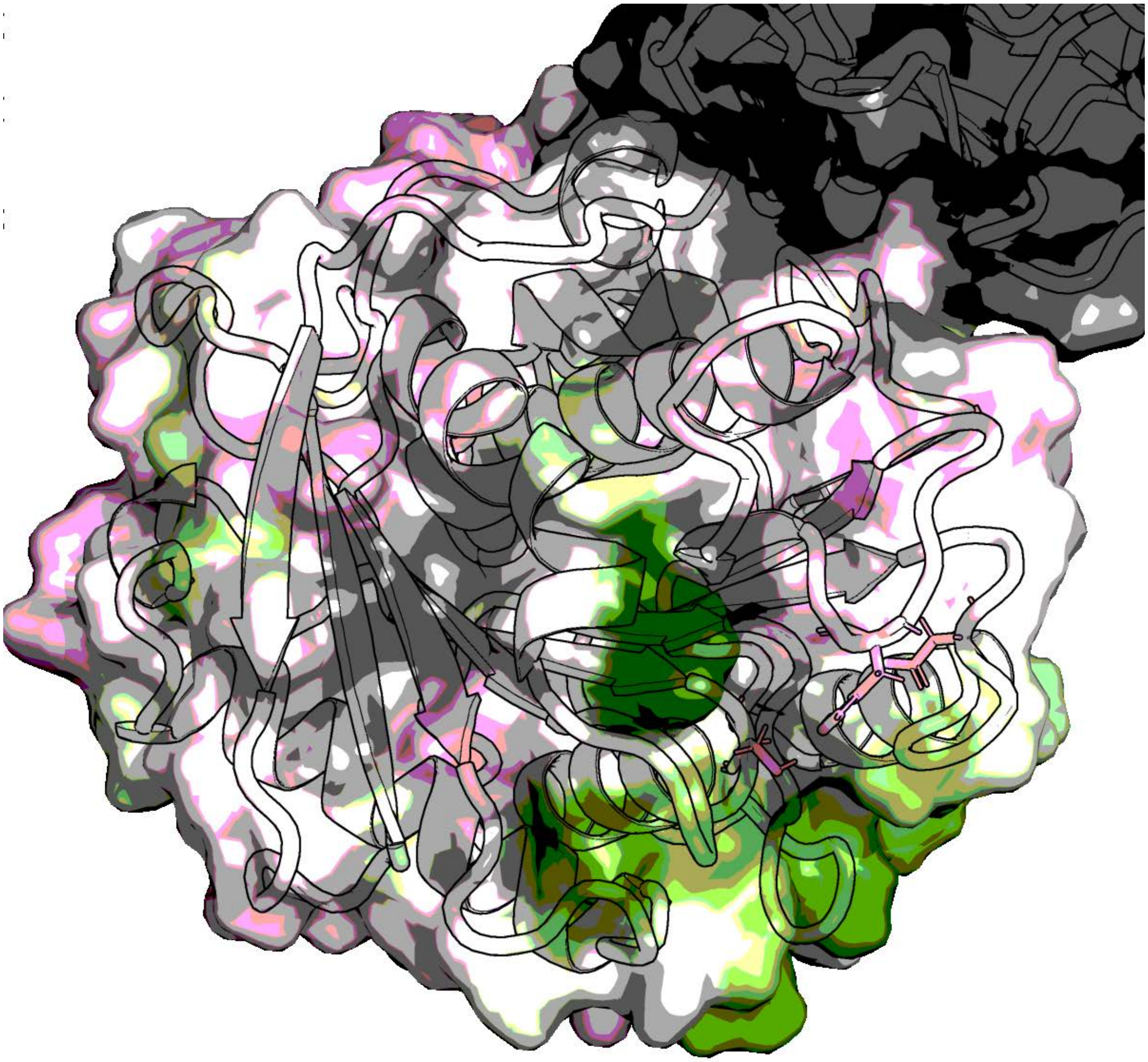

ESM026_highlights.png

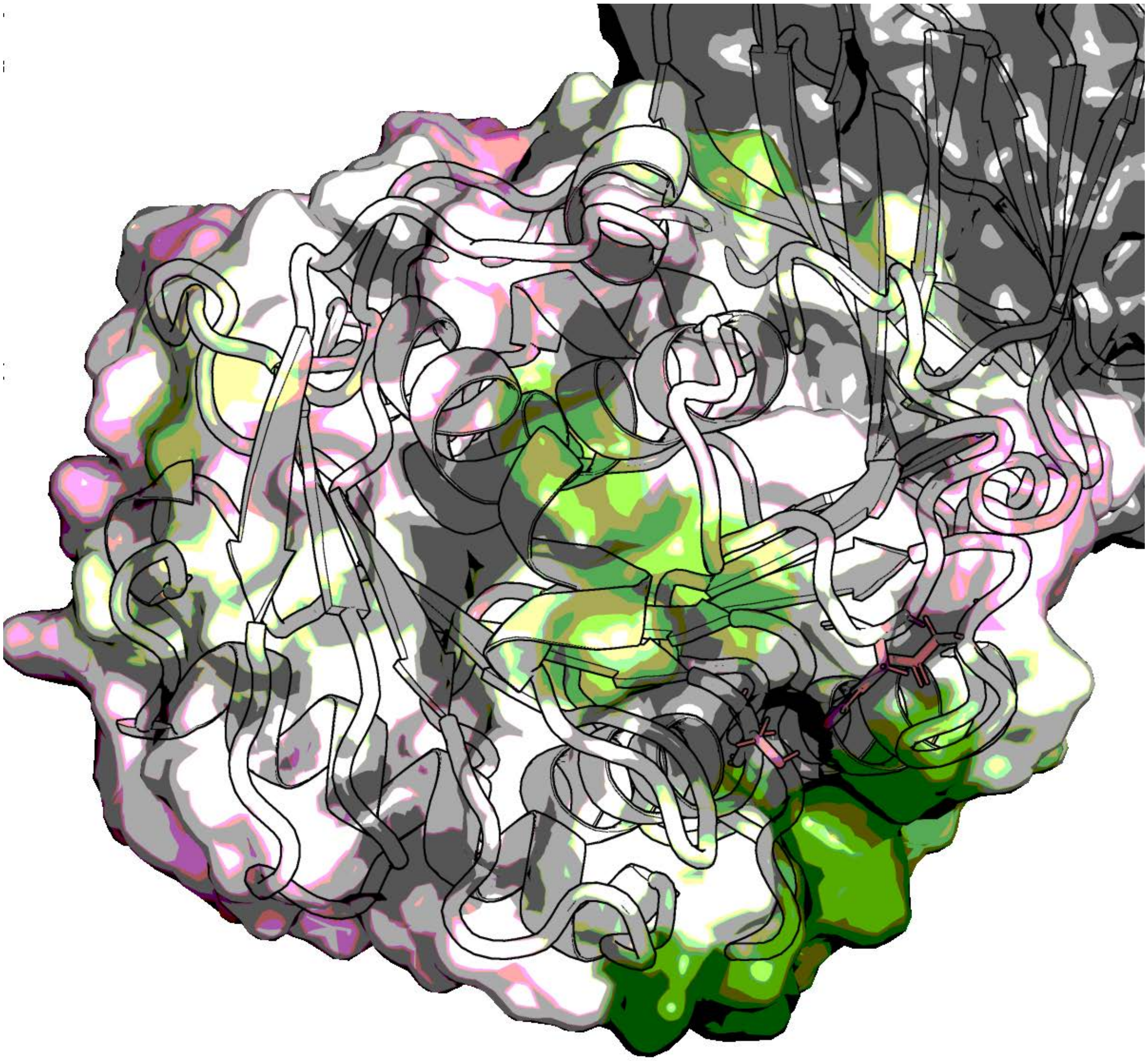

ESM033_highlights.png

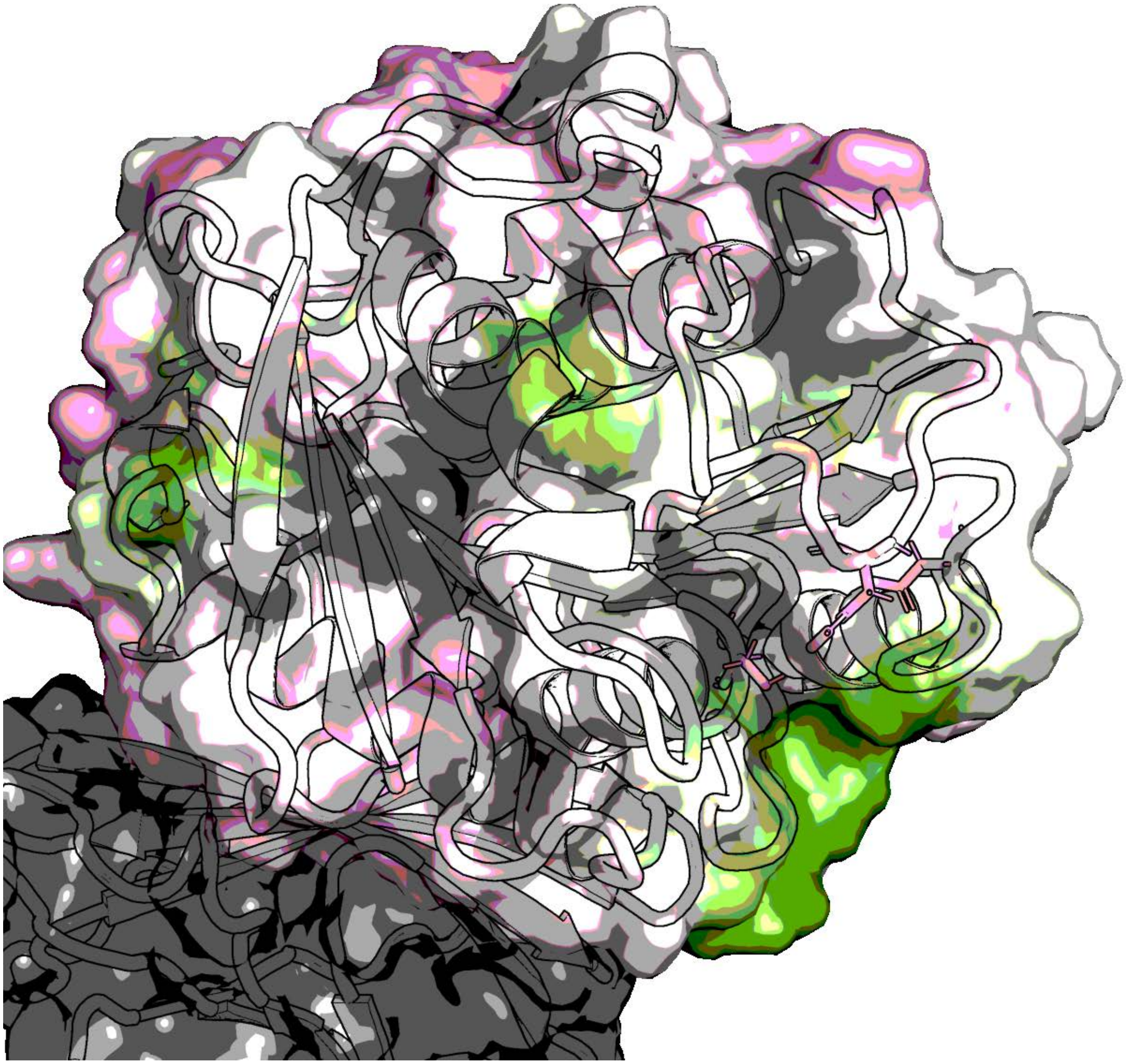

ESM035_highlights.png

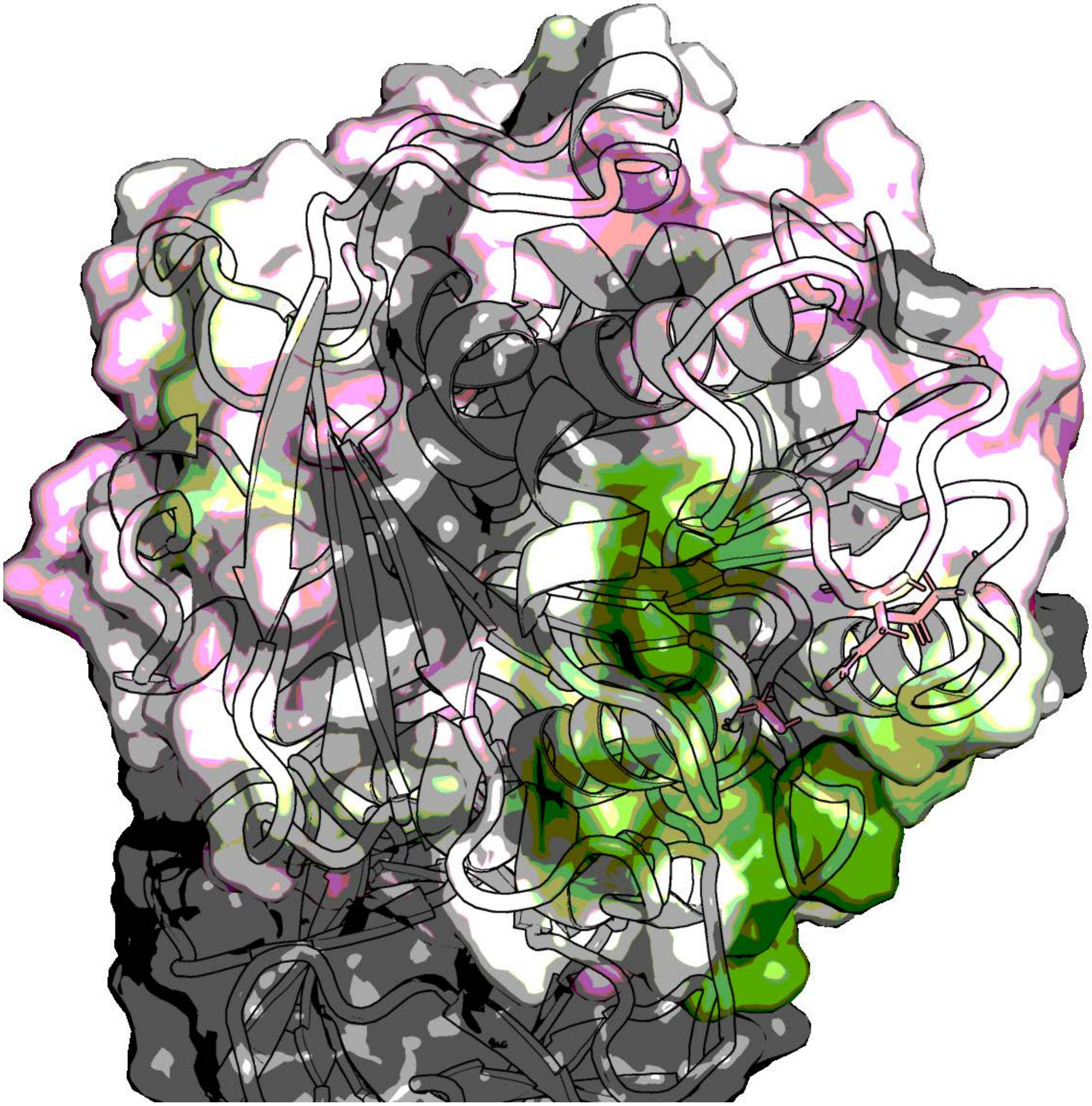

ESM038_highlights.png

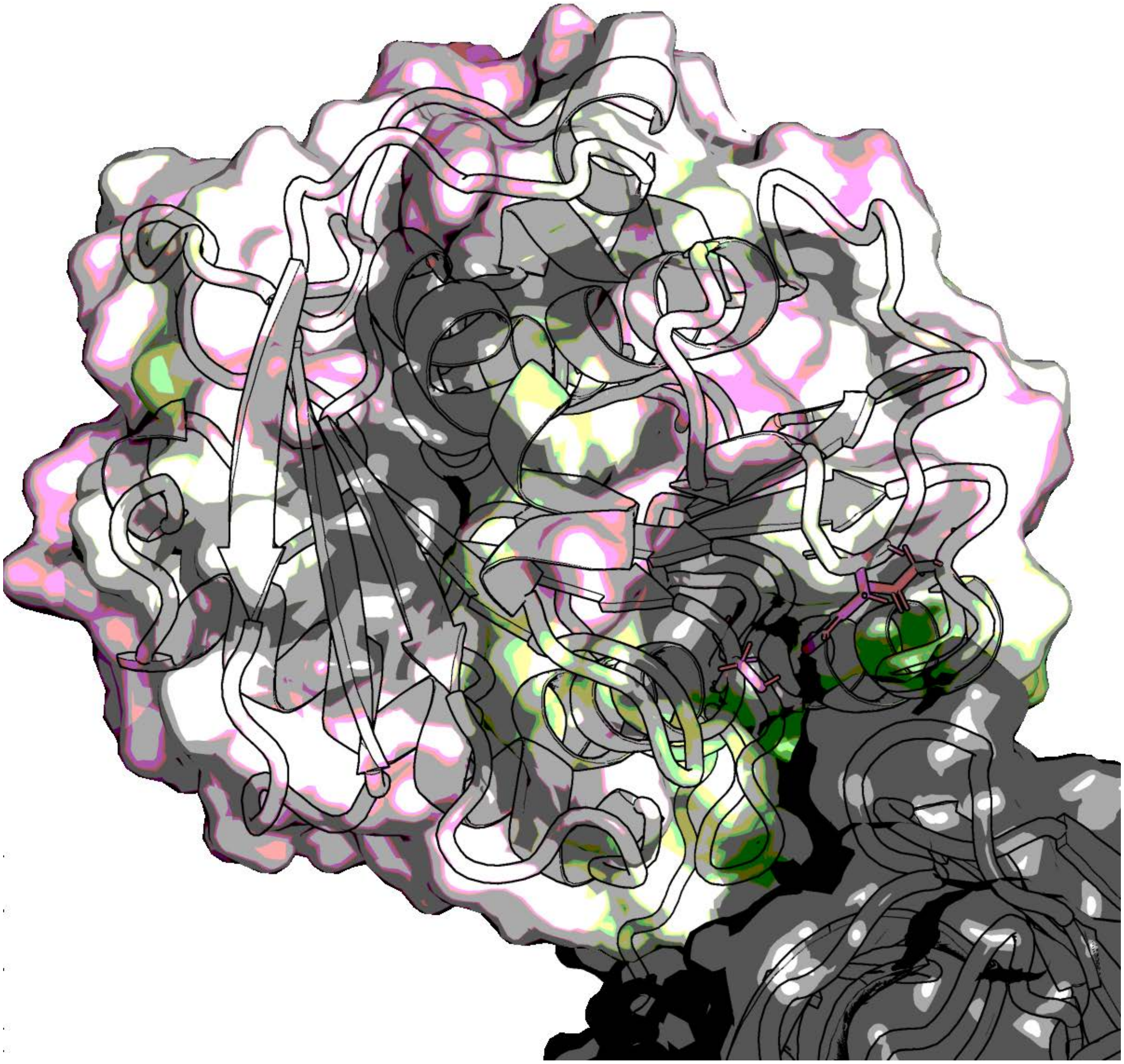

ESM043_highlights.png

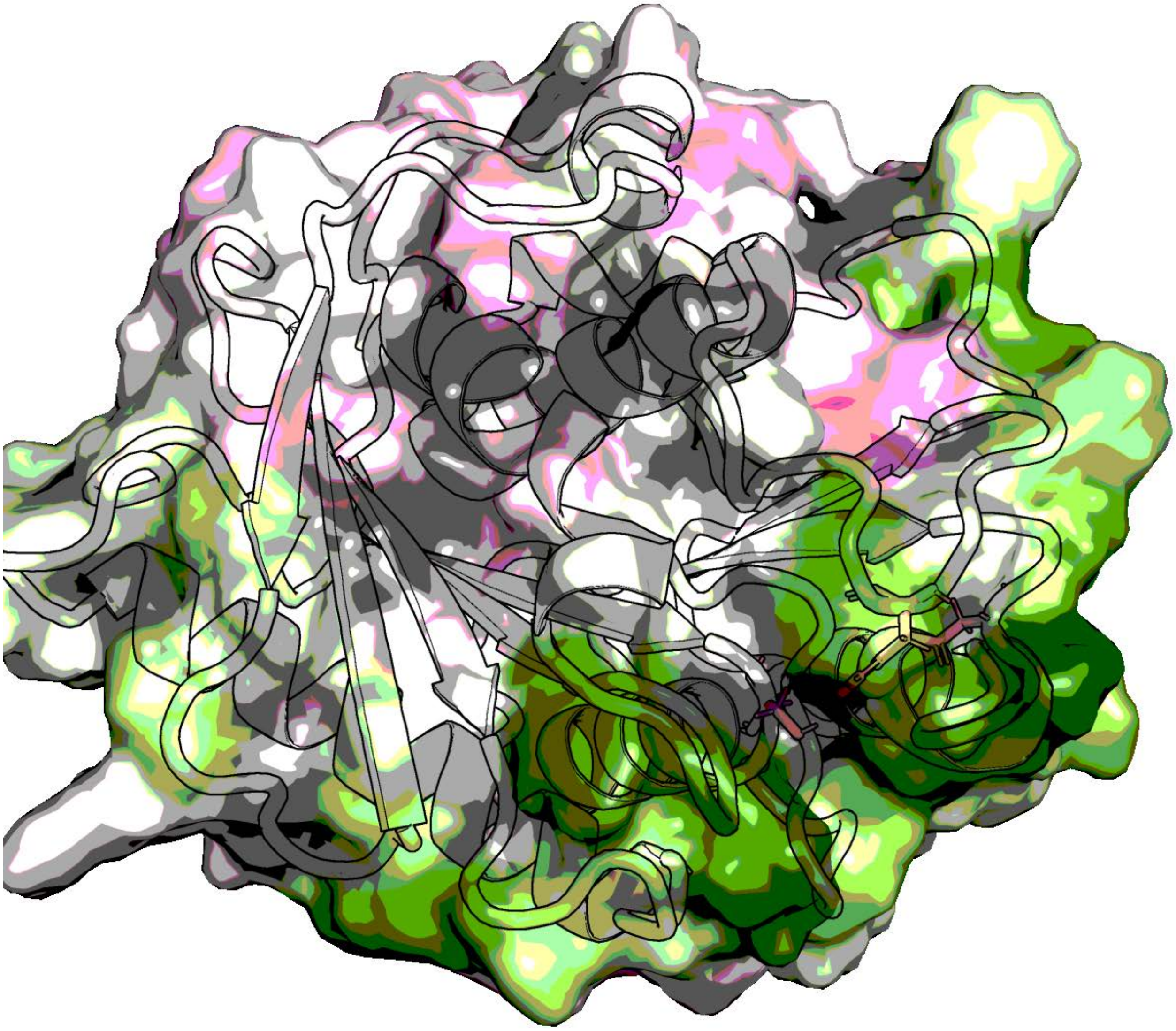

ESM045_highlights.png

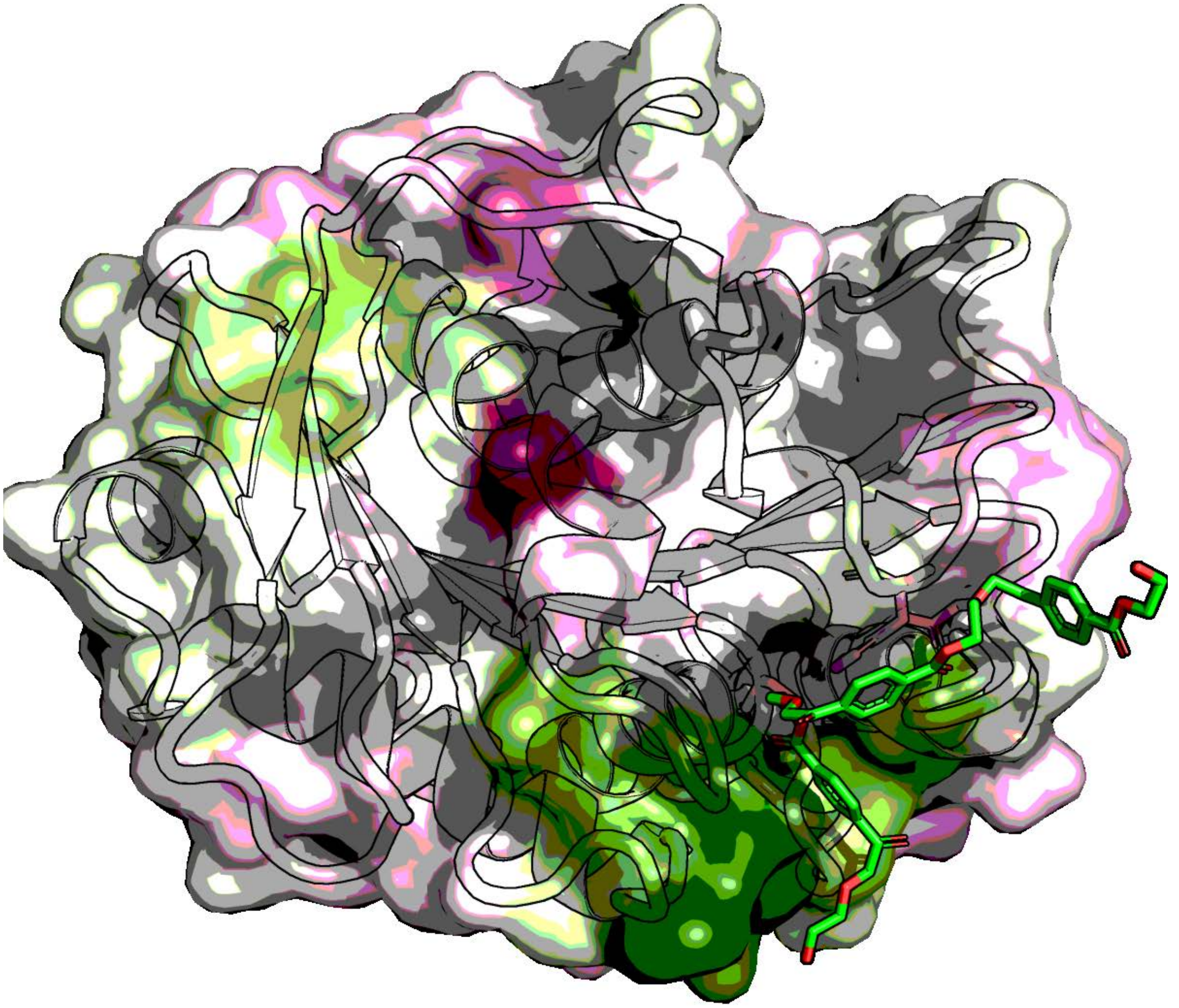

ESM047_highlights.png

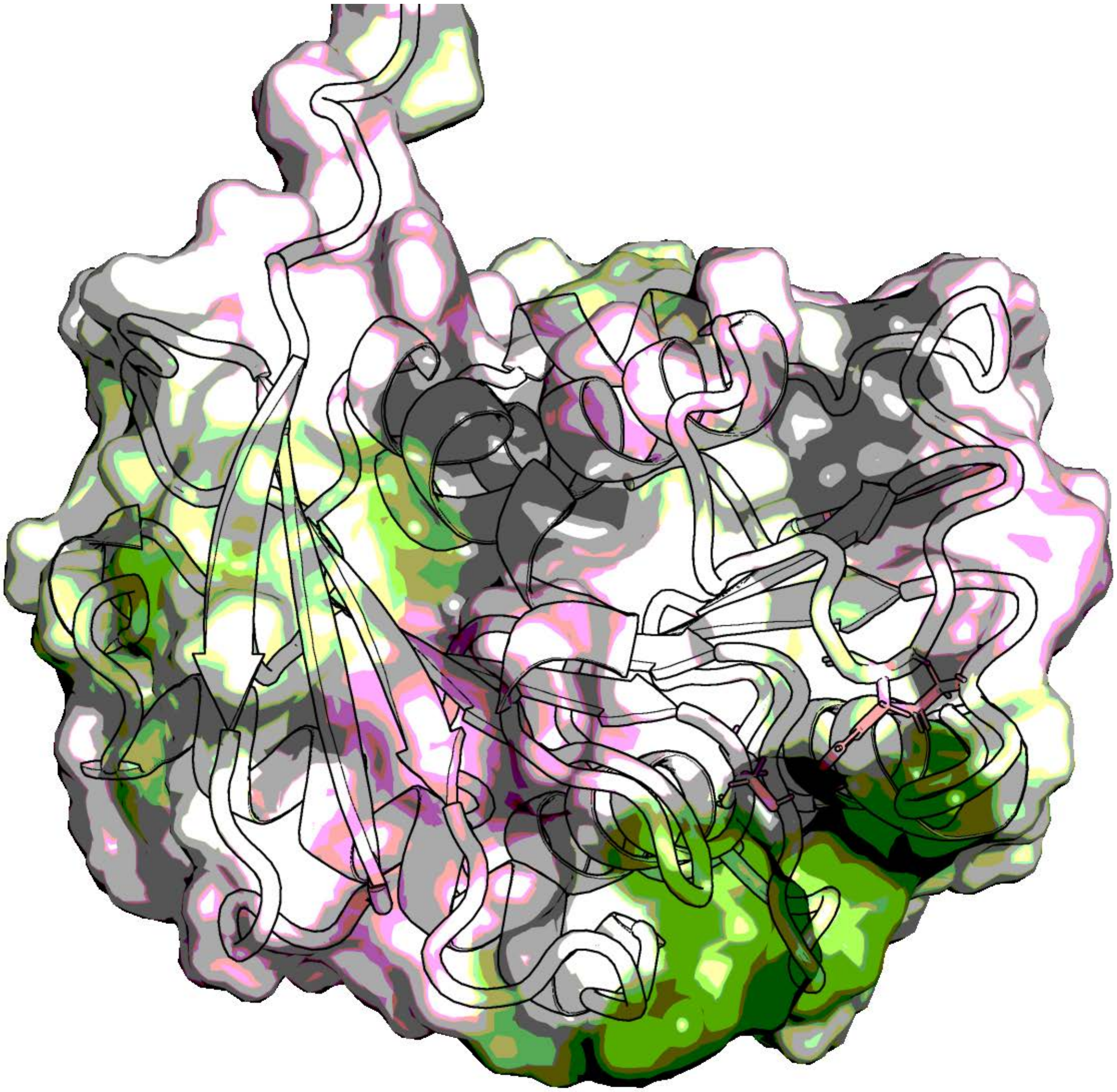

ESM050_highlights.png

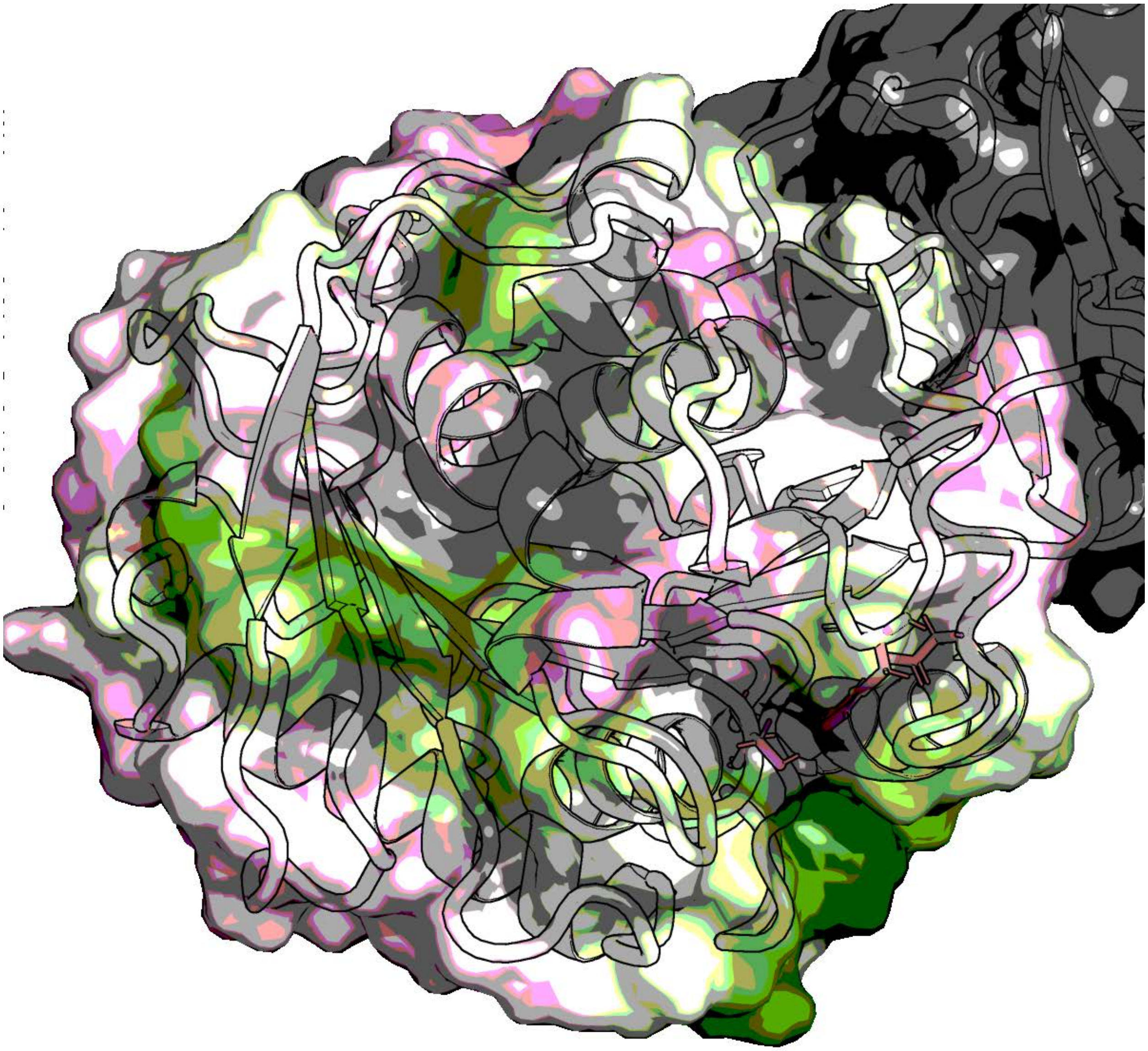

ESM052_highlights.png

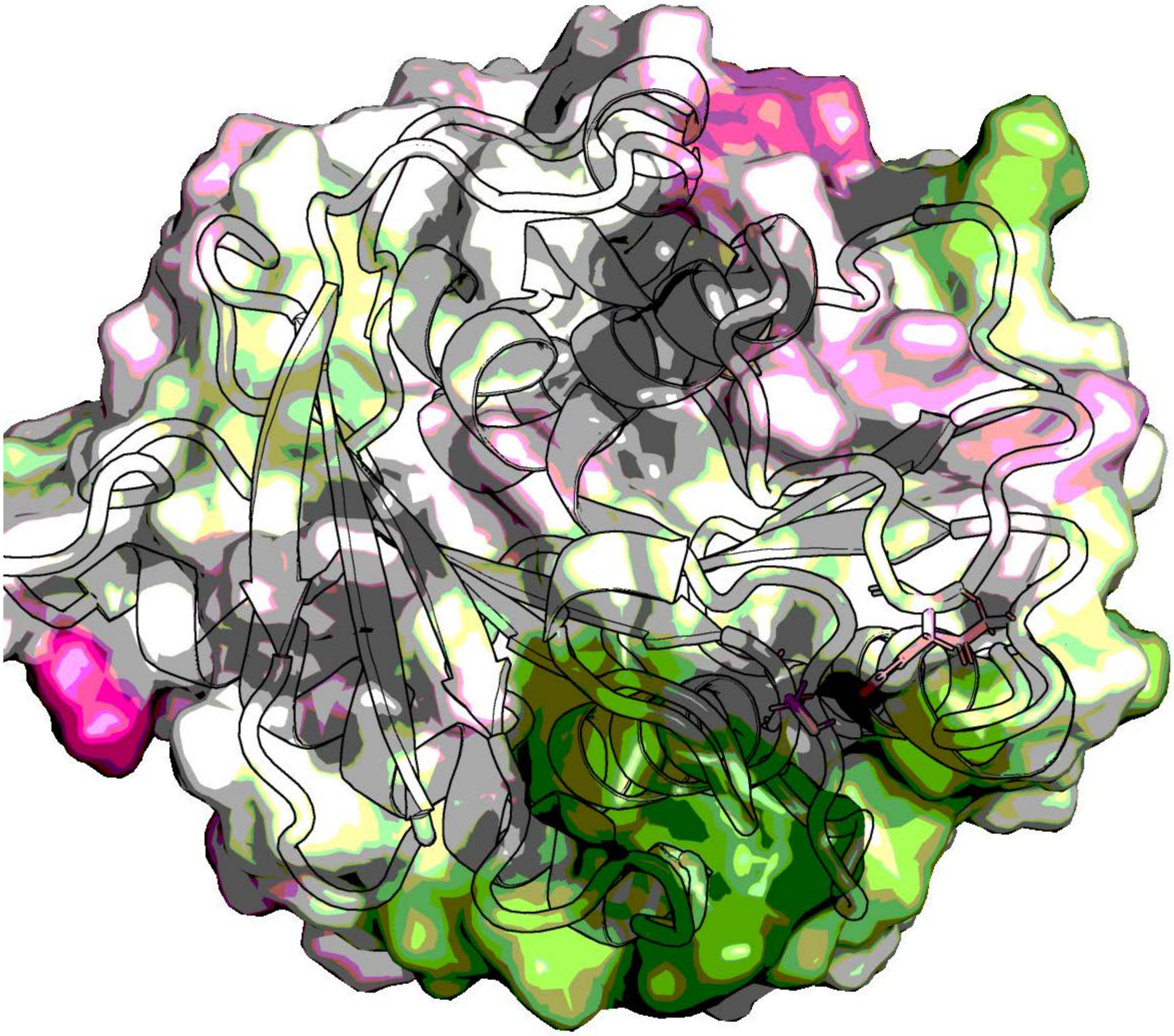

ESM053_highlights.png

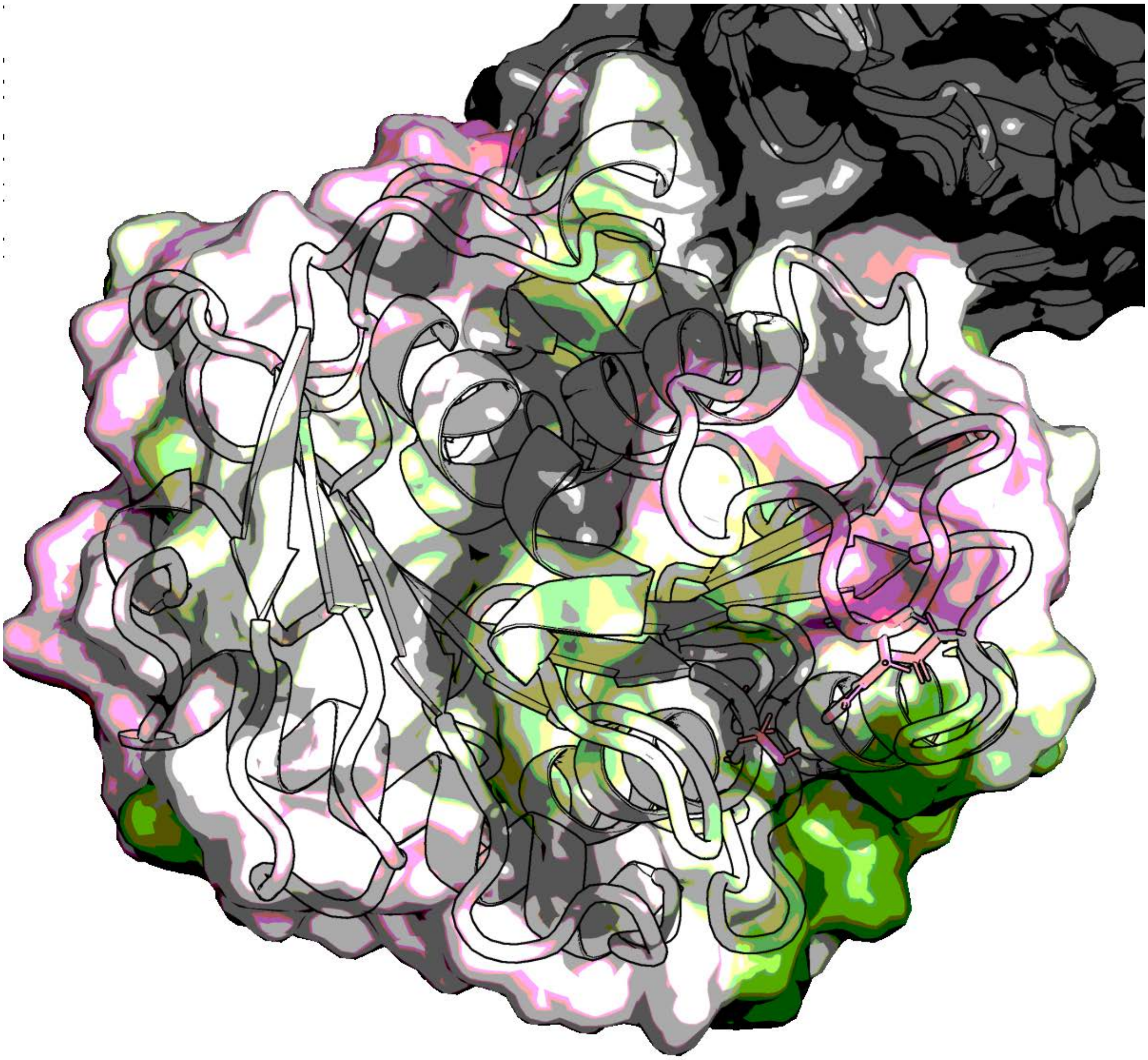

ESM058_highlights.png

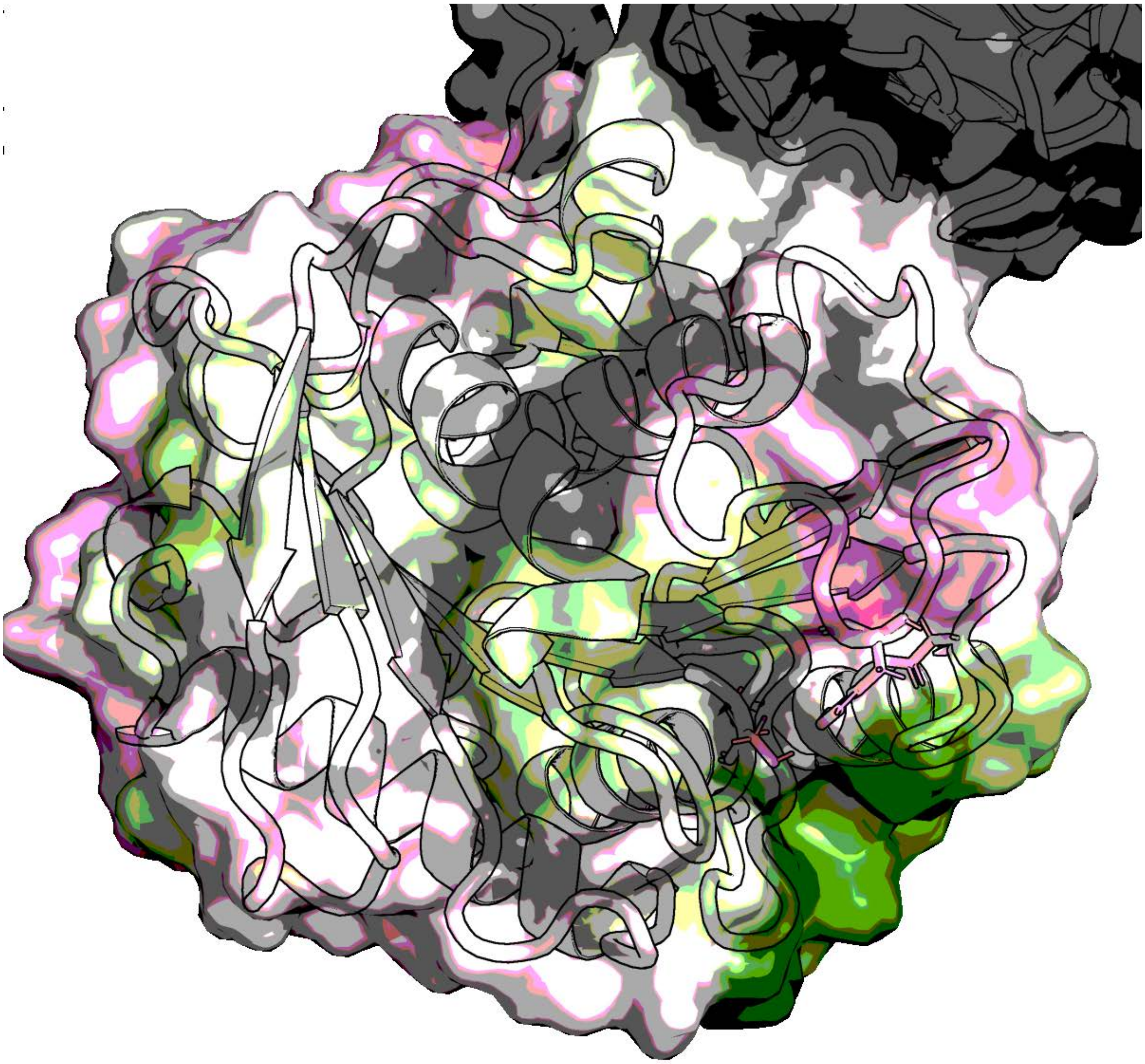

ESM059_highlights.png

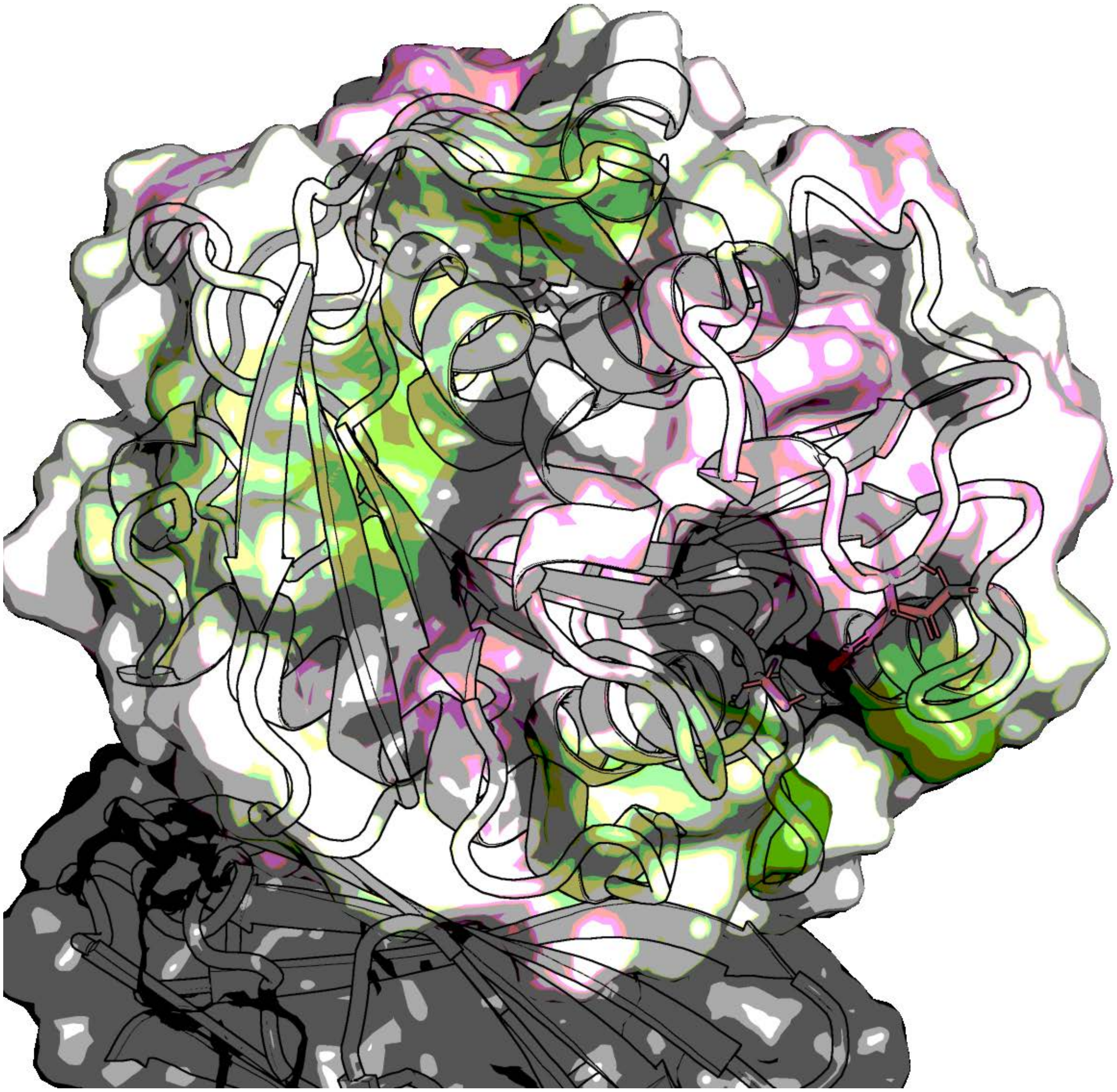

ESM060_highlights.png

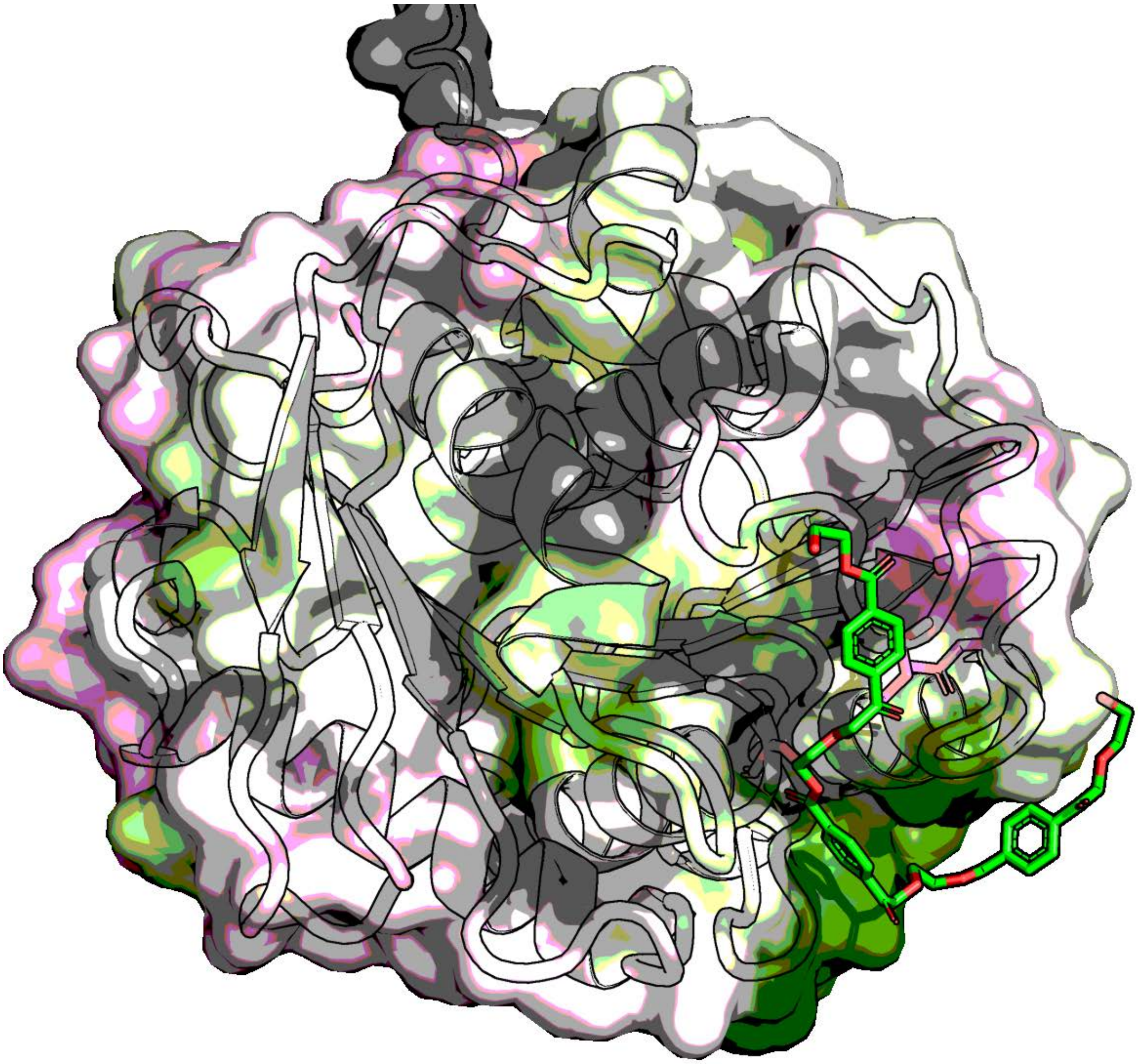

ESM061_highlights.png

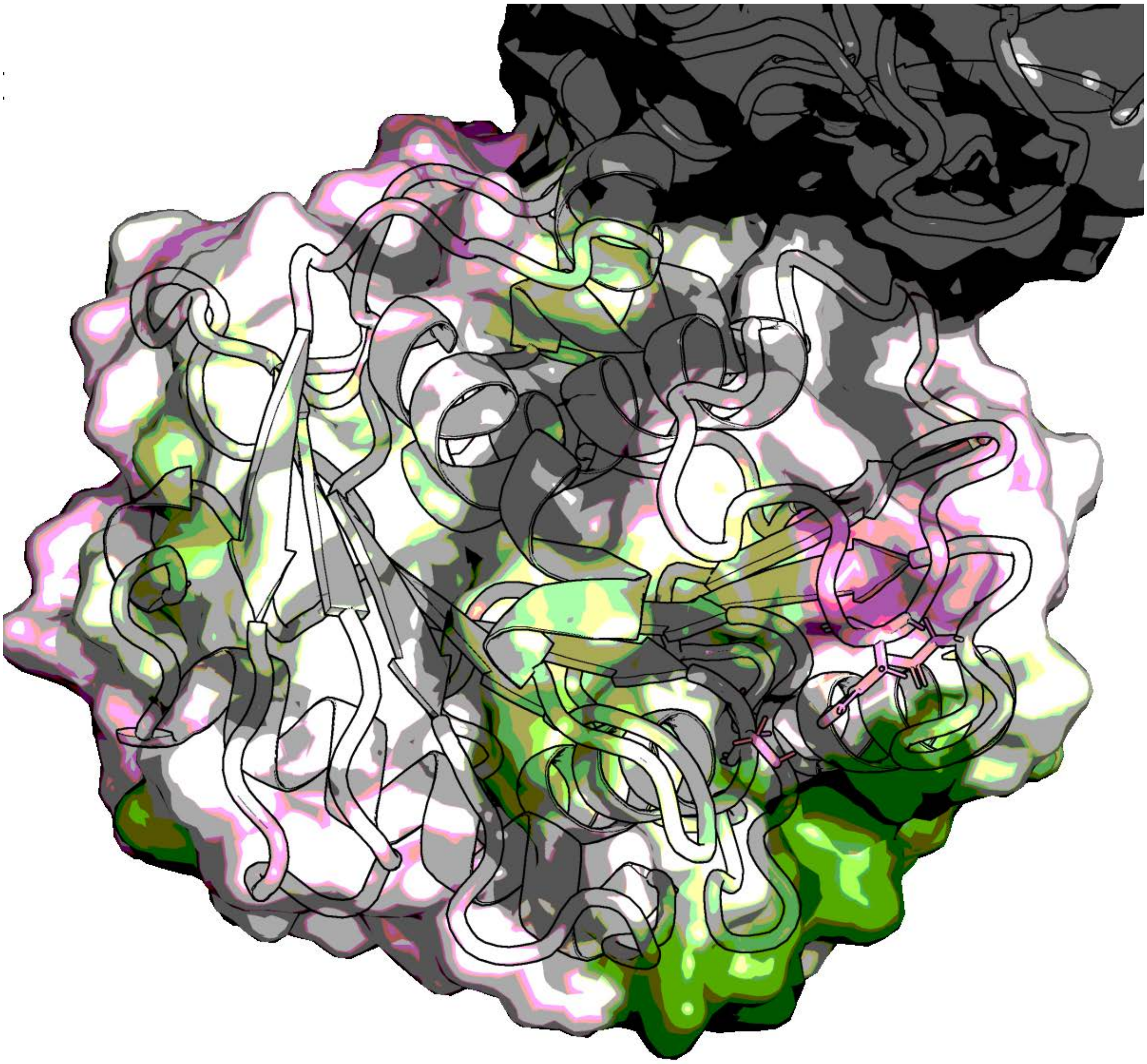

ESM062_highlights.png

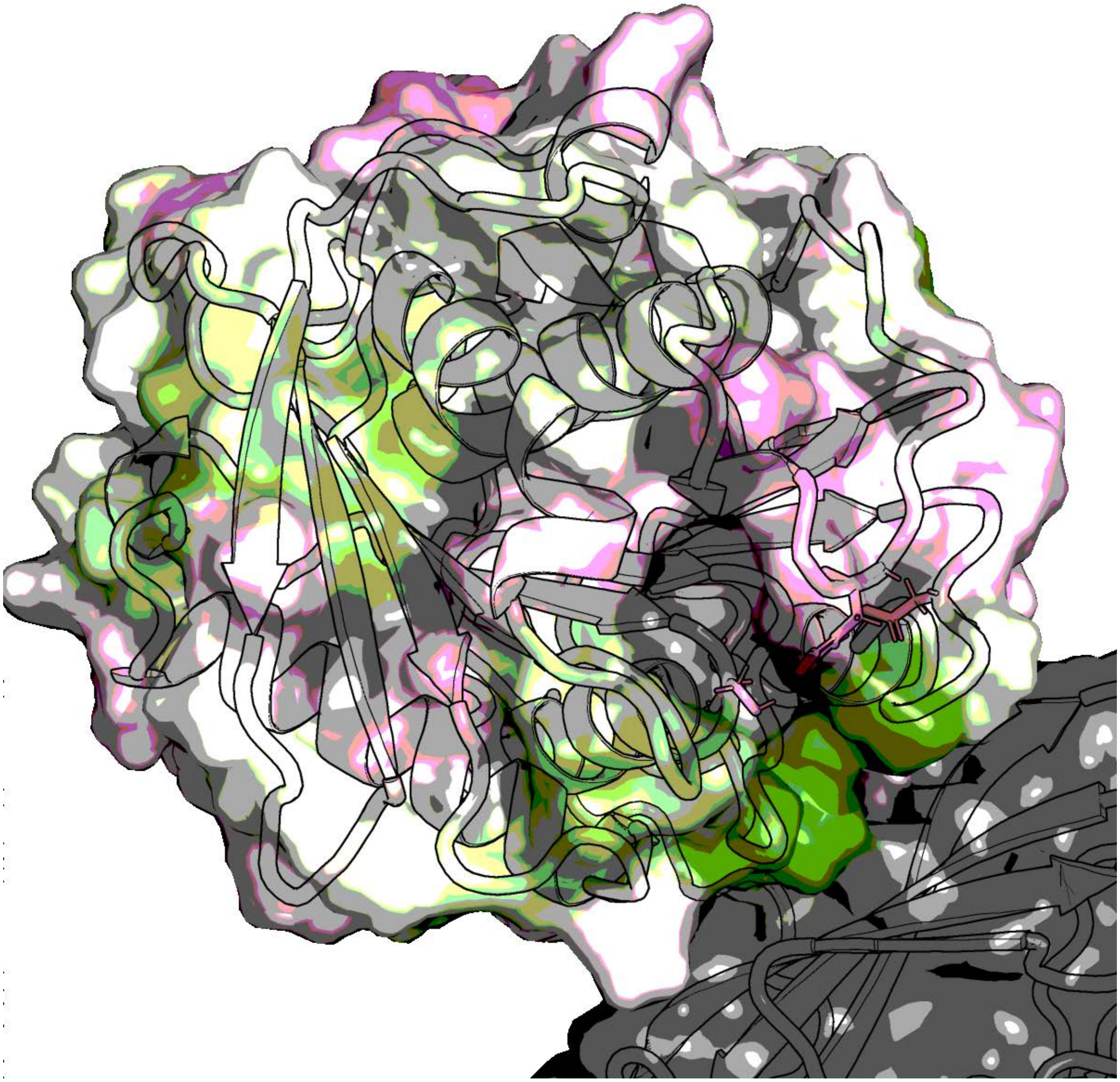

ESM064_highlights.png

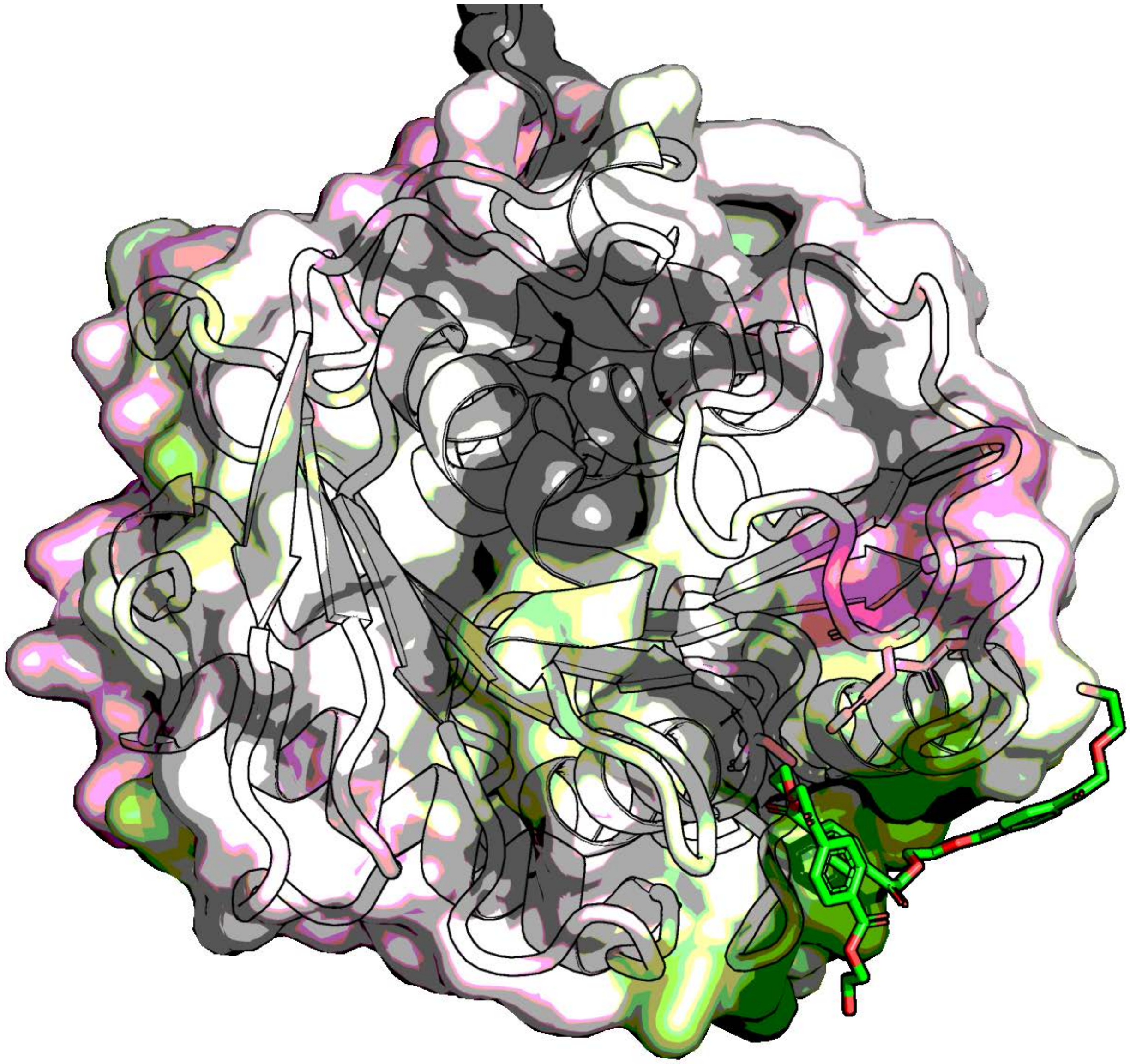

ESM065_highlights.png

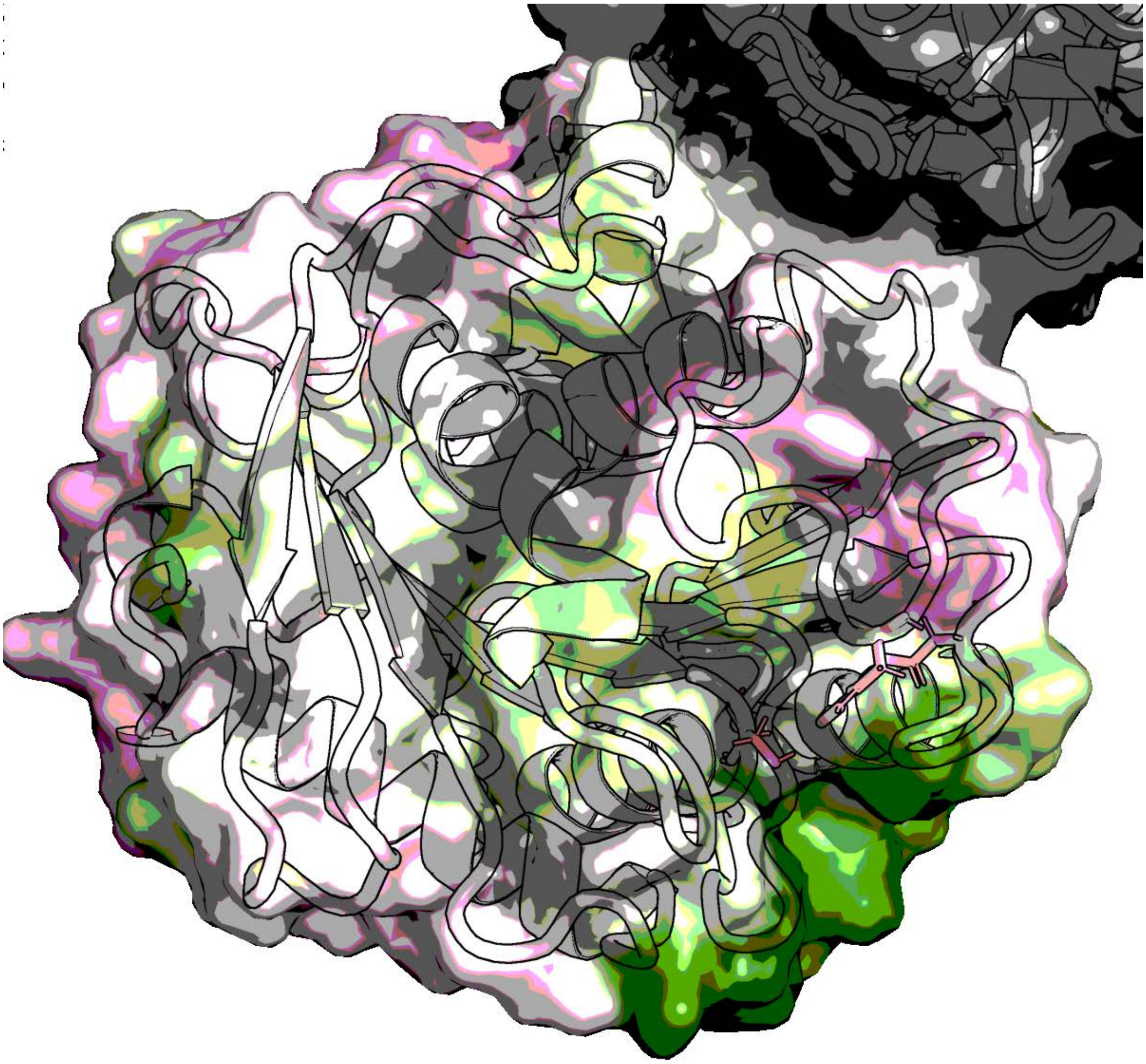

ESM069_highlights.png

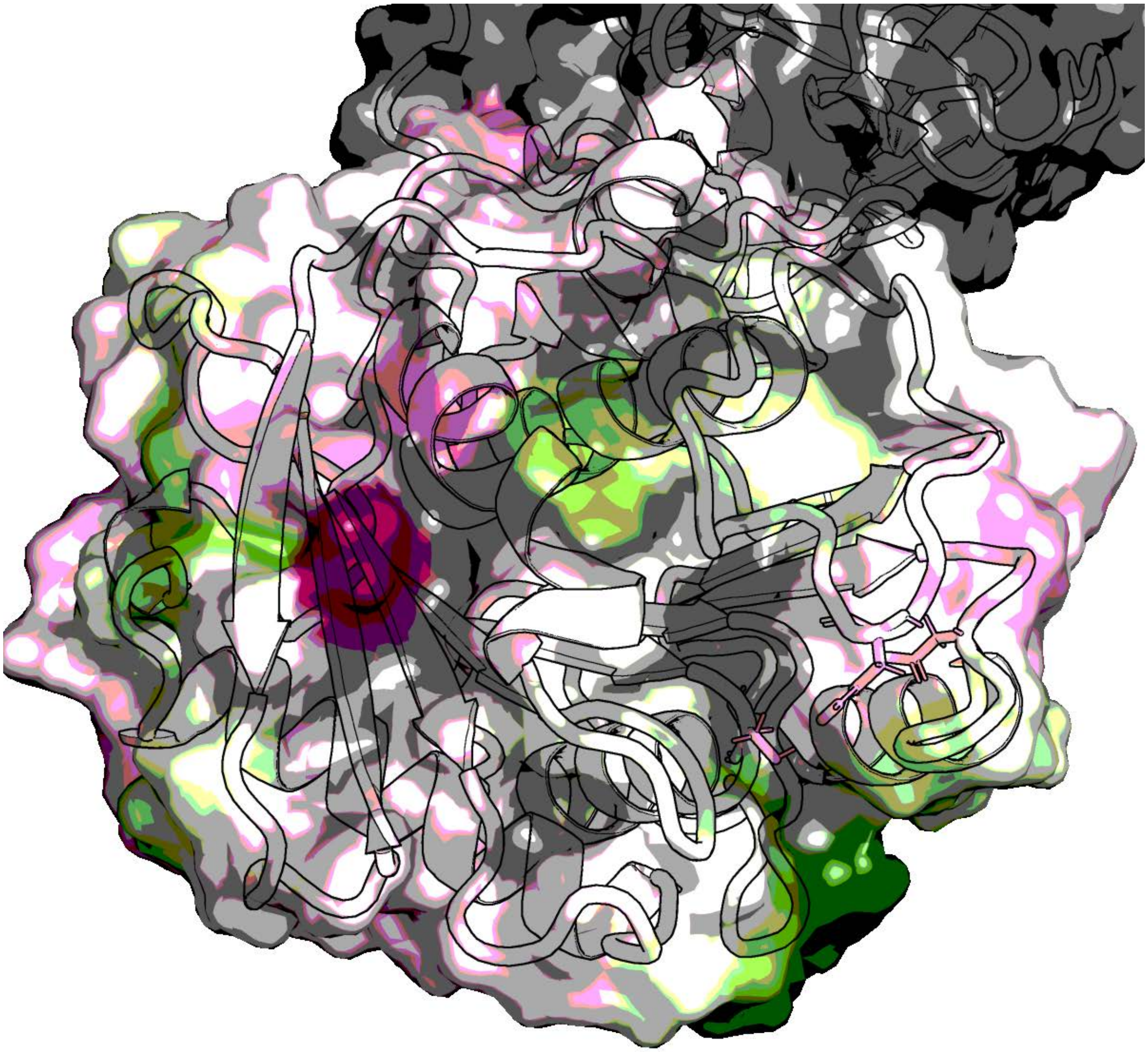

ESM070_highlights.png

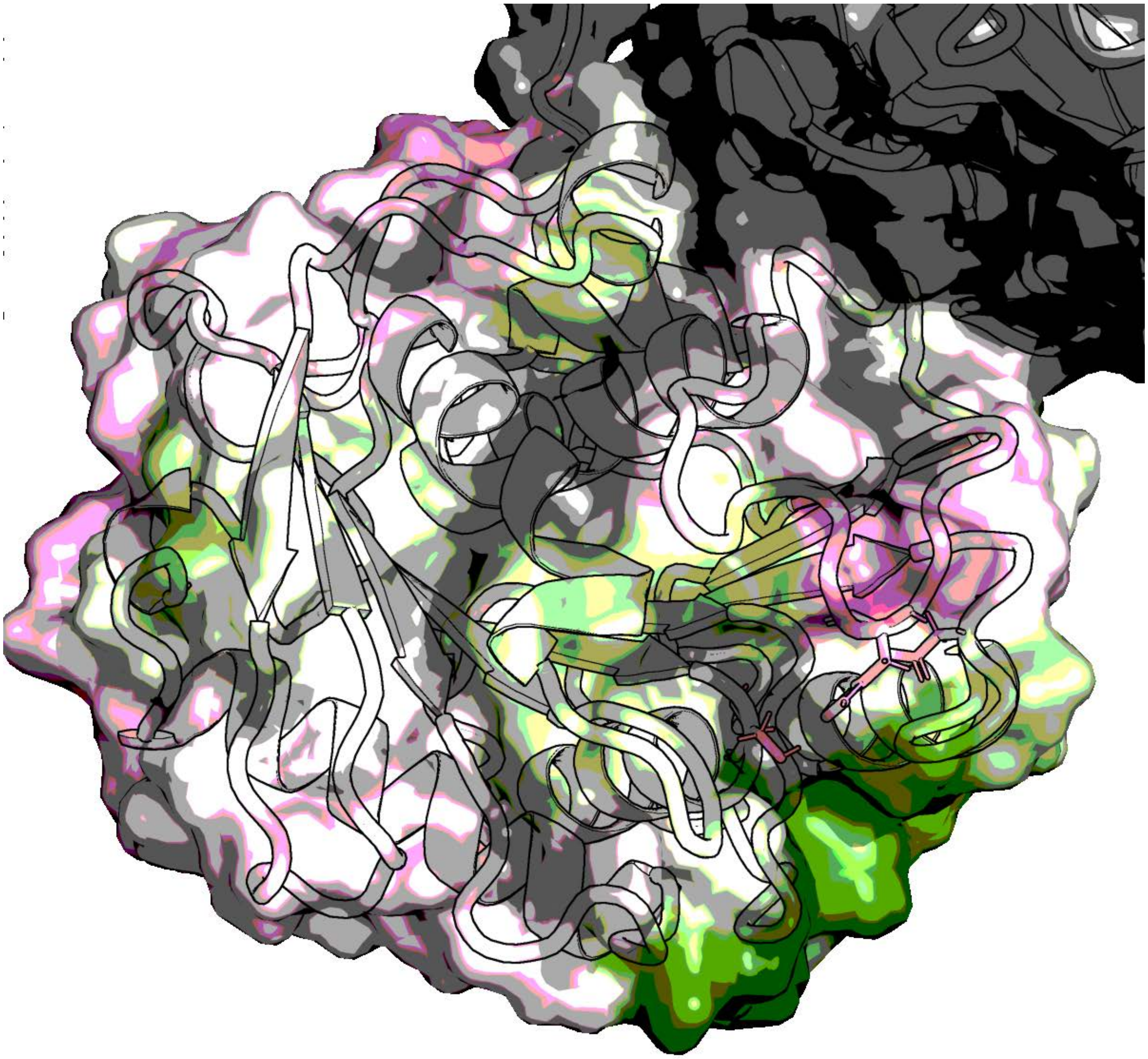

ESM075_highlights.png

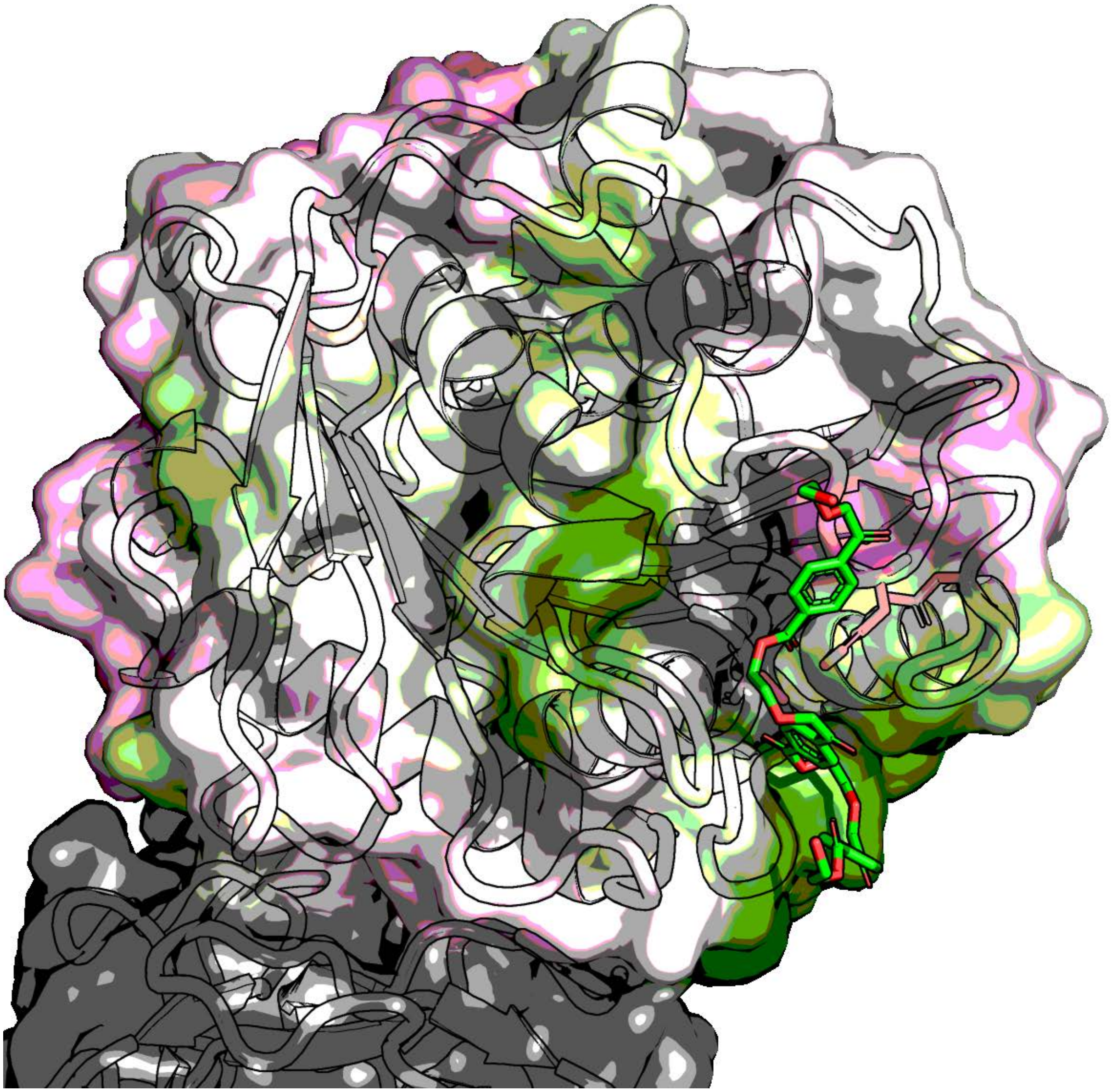

ESM080_highlights.png

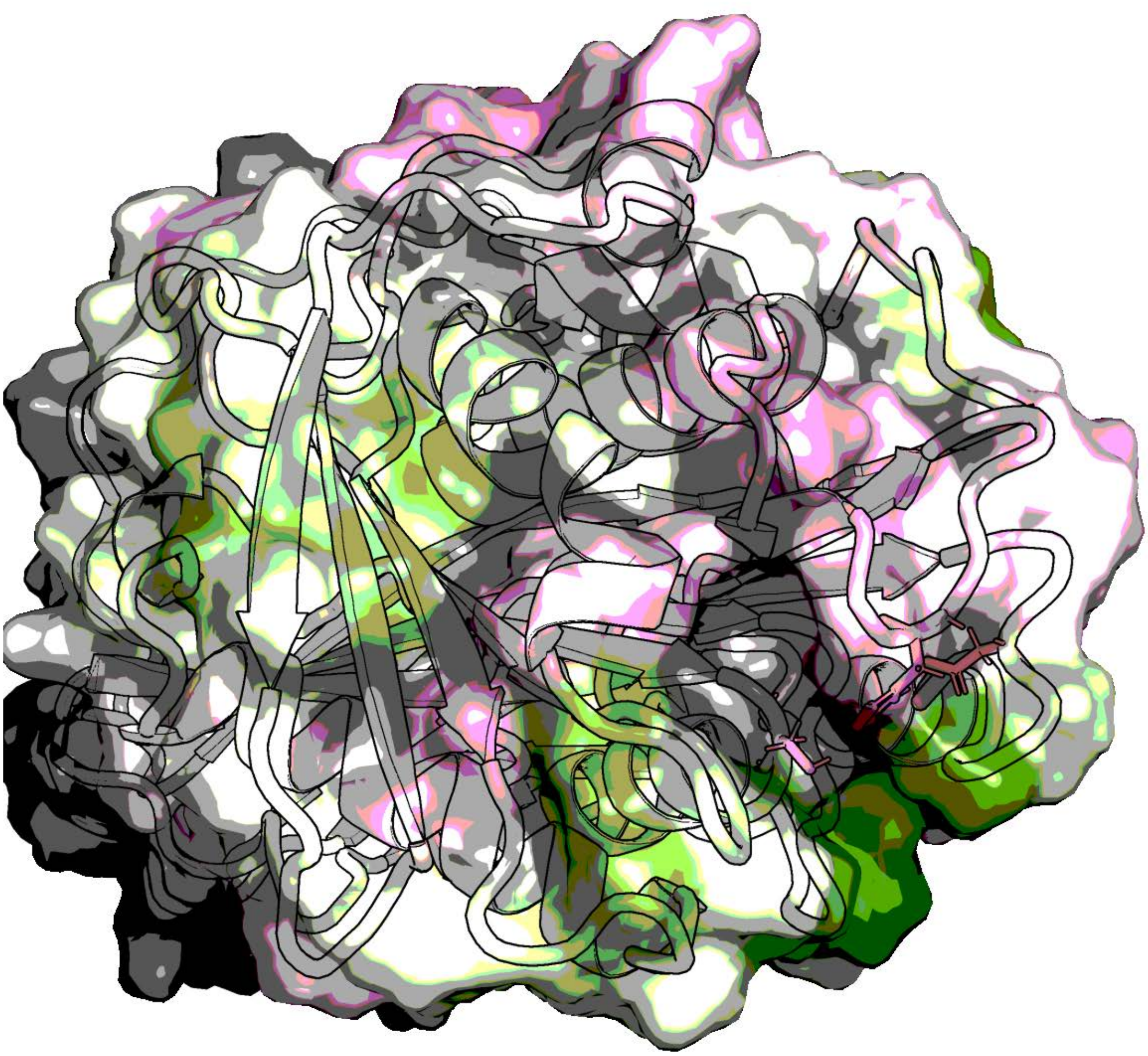

ESM081_highlights.png

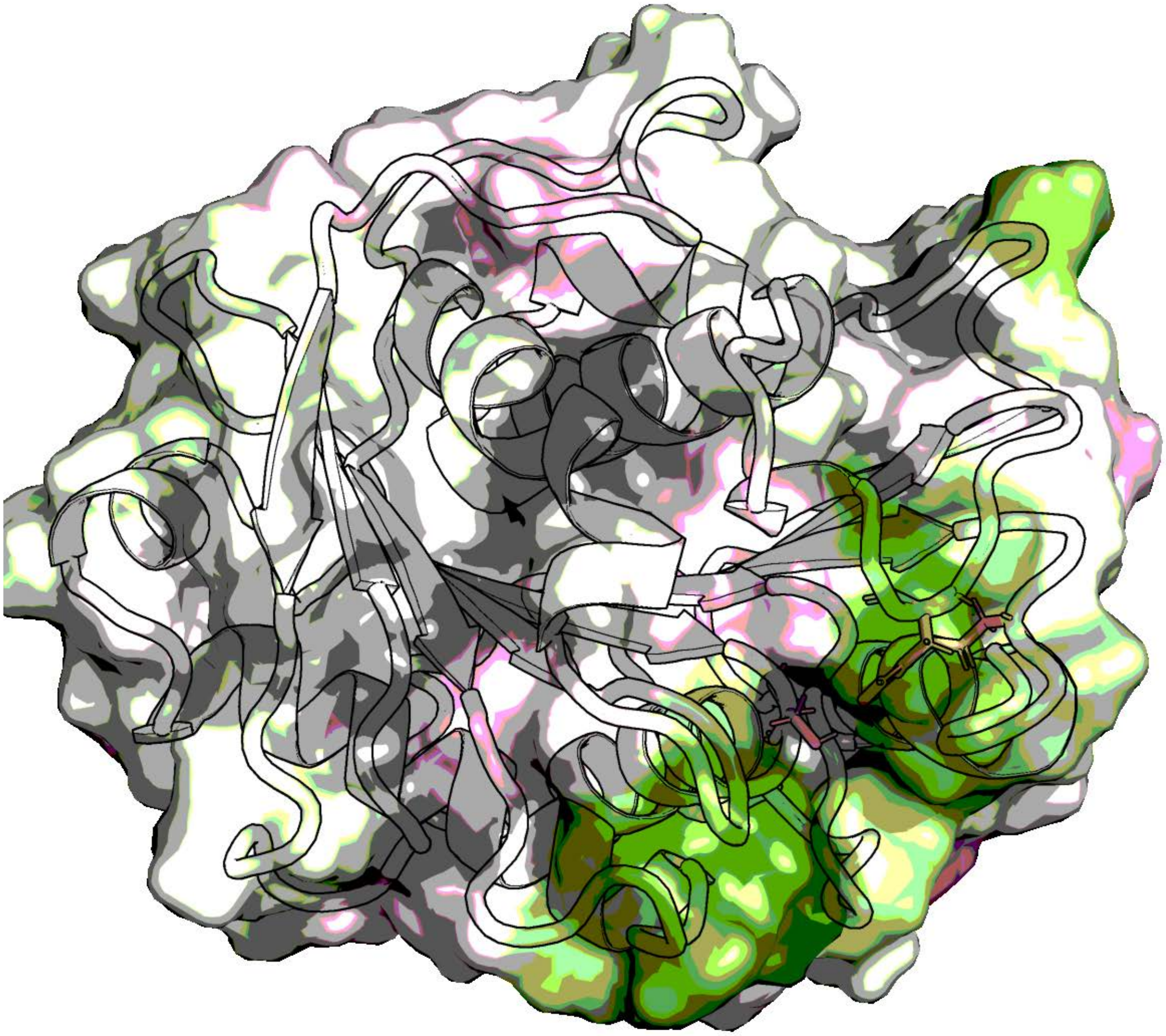

ESM082_highlights.png

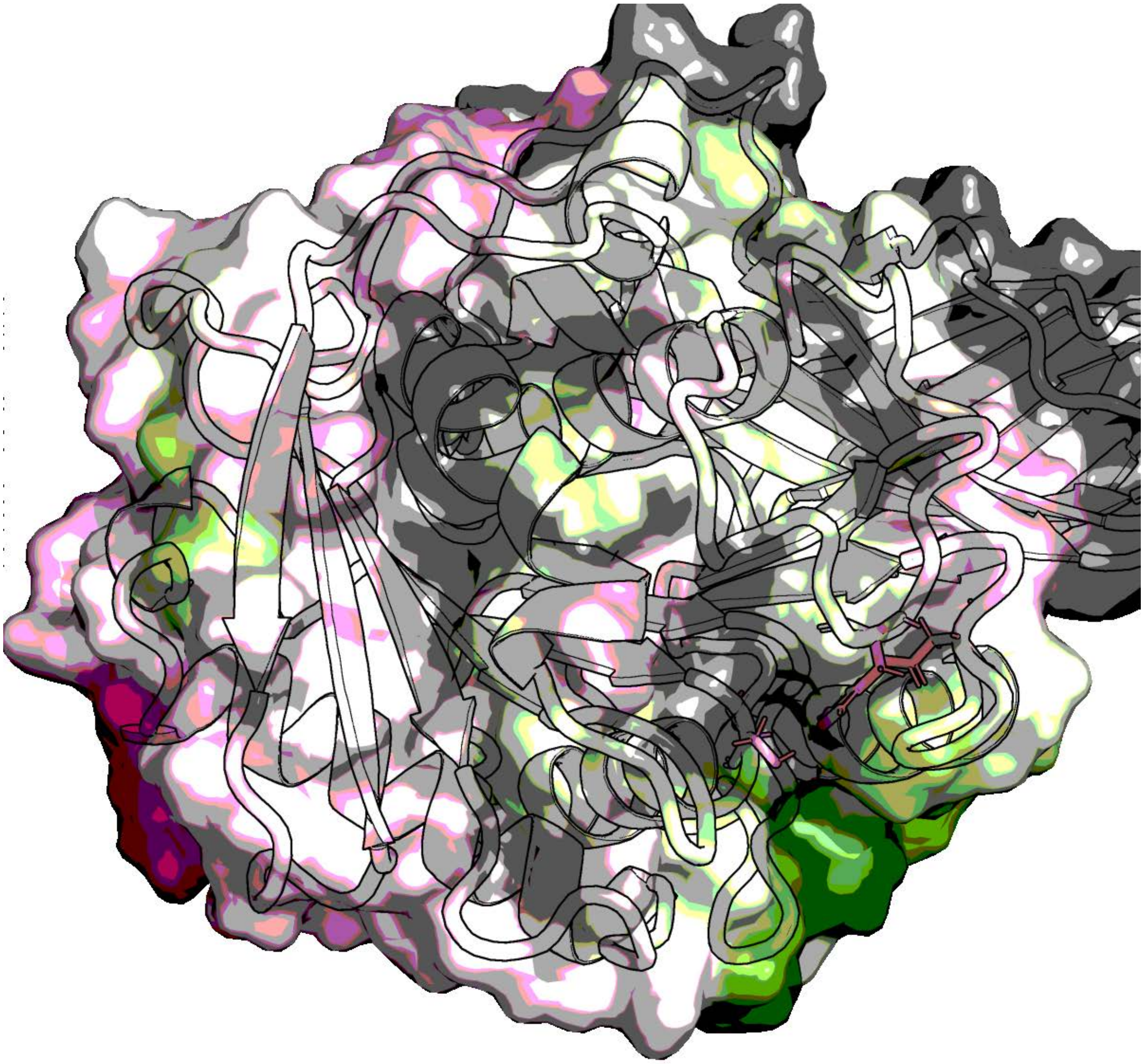

ESM084_highlights.png

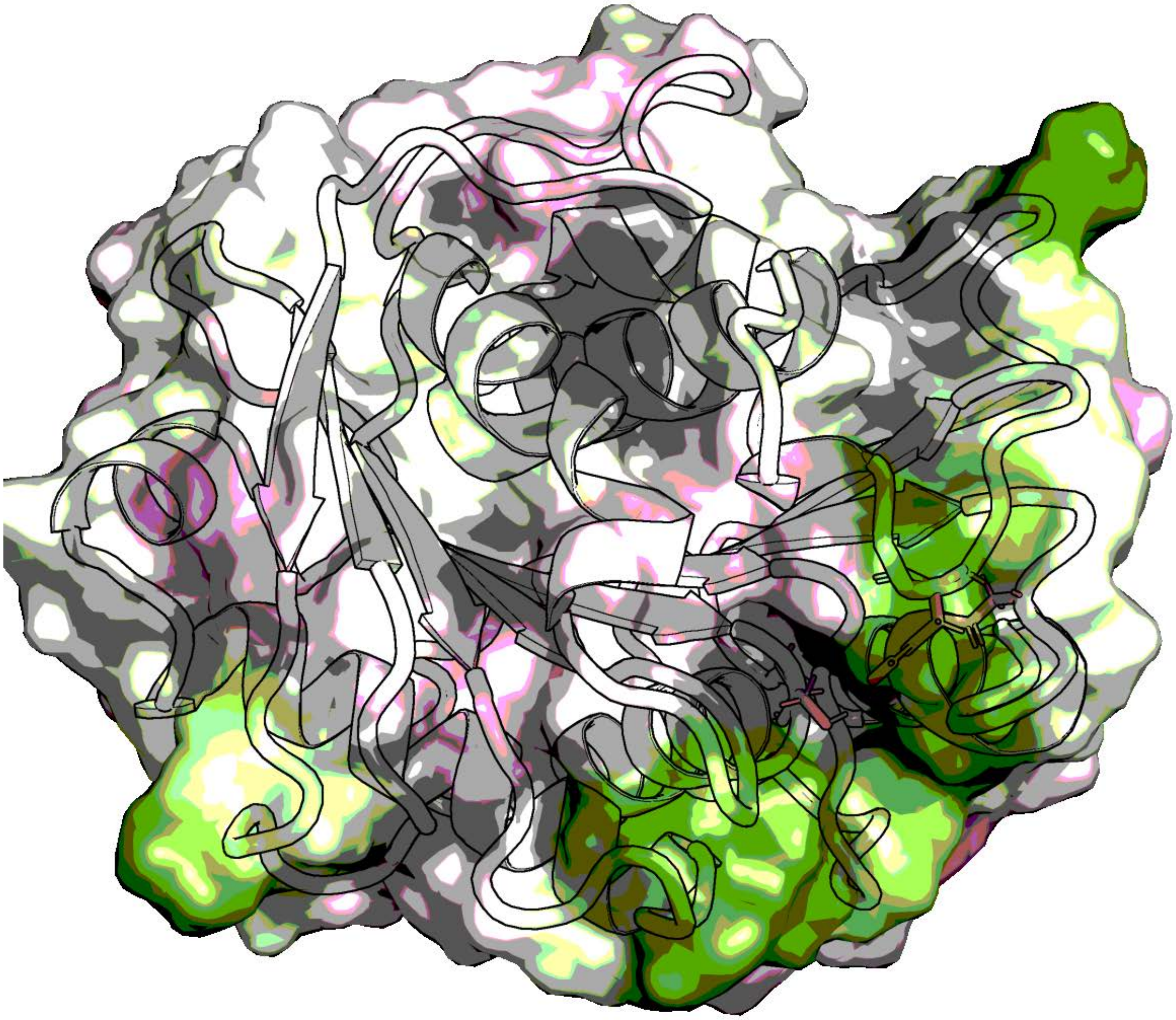

ESM090_highlights.png

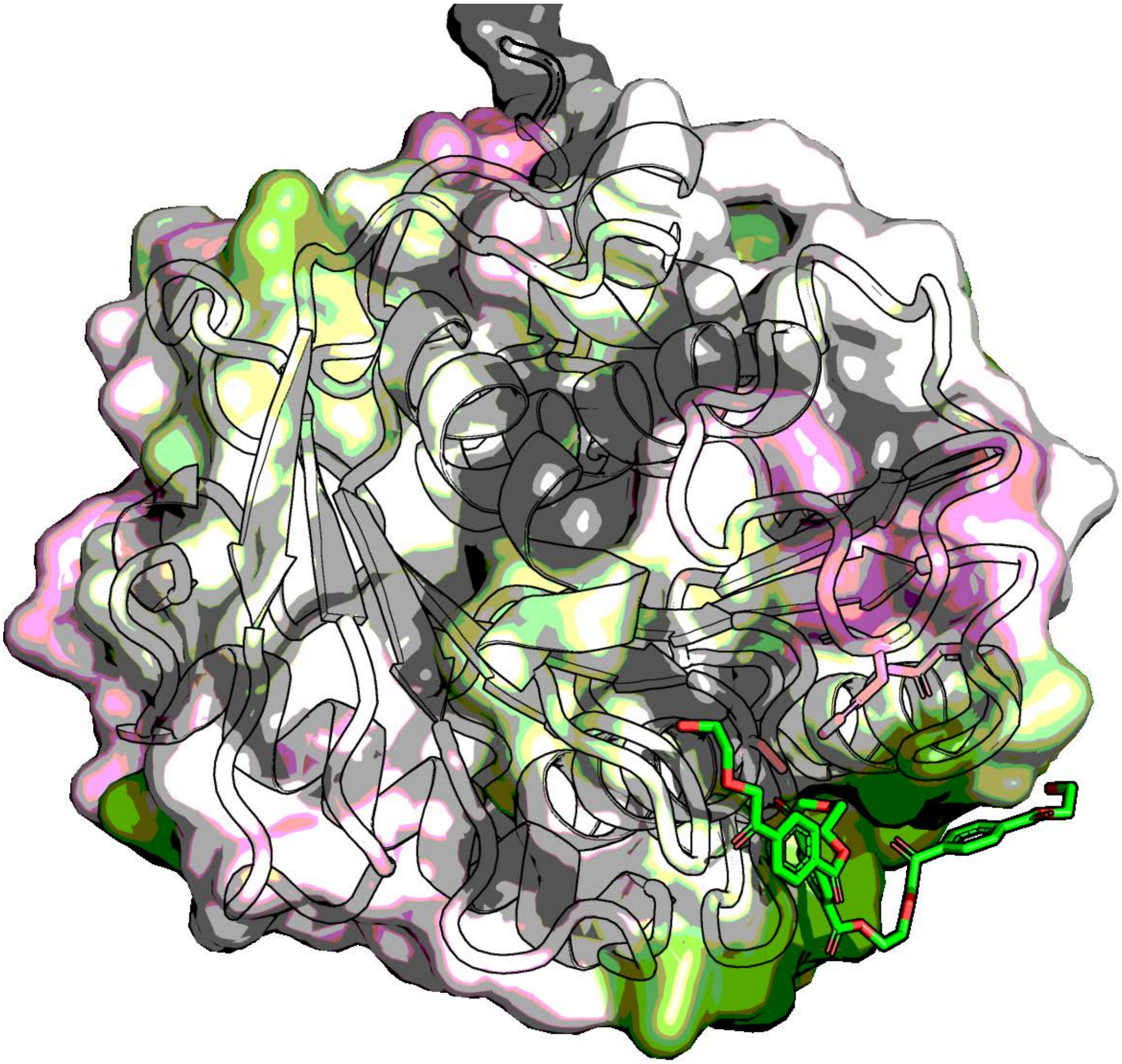

ESM091_highlights.png

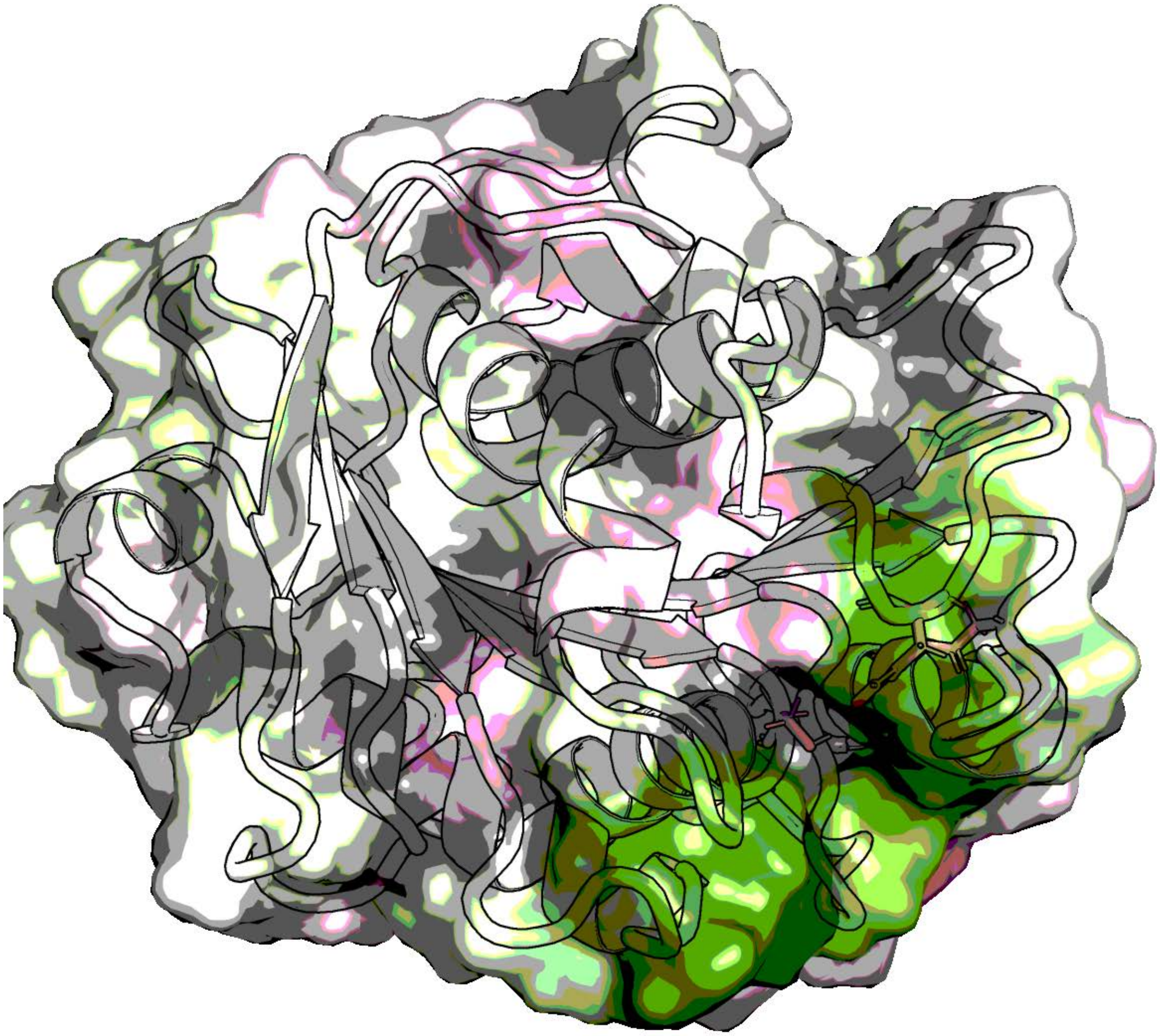

ESM093_highlights.png

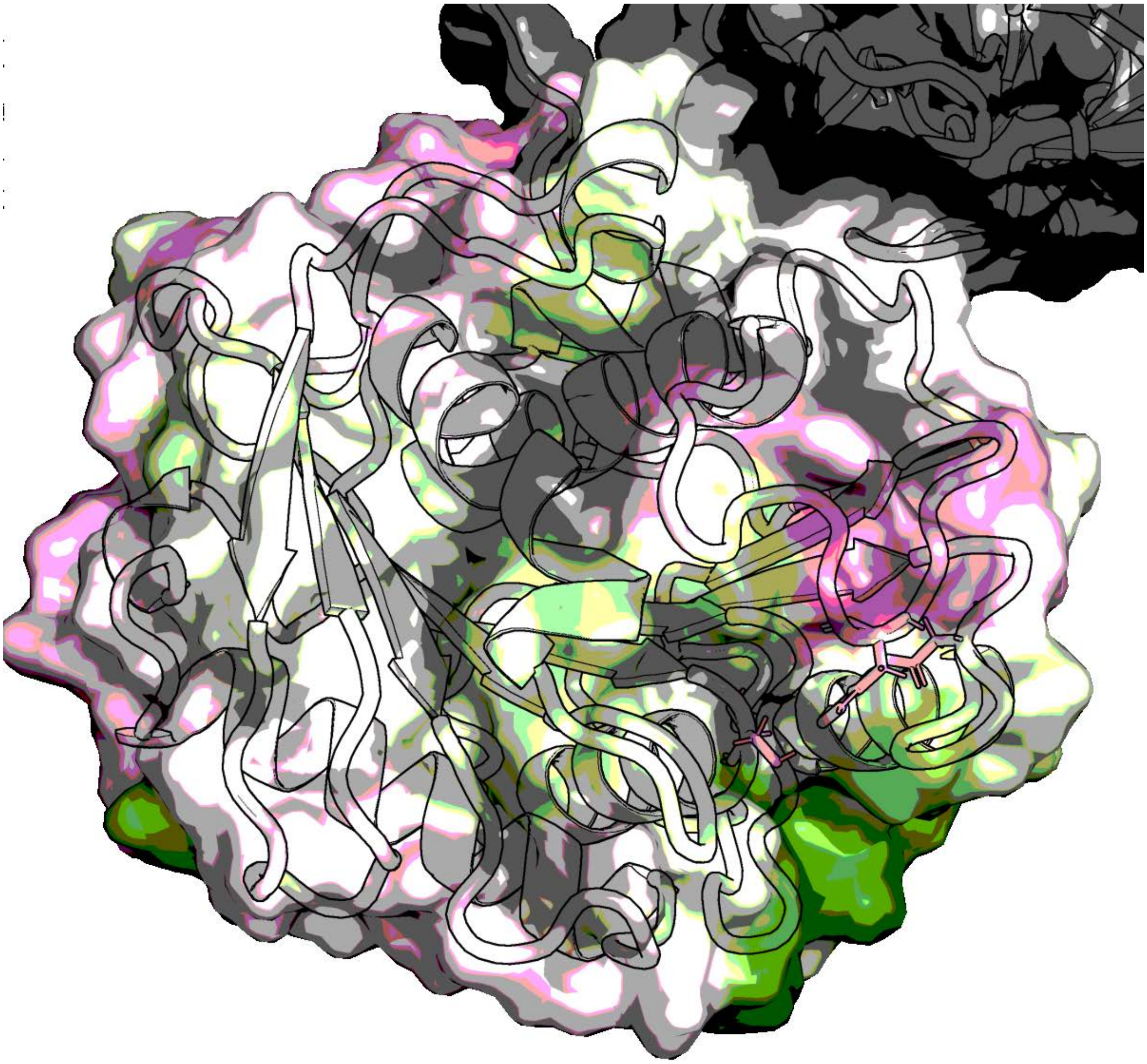

ESM095_highlights.png

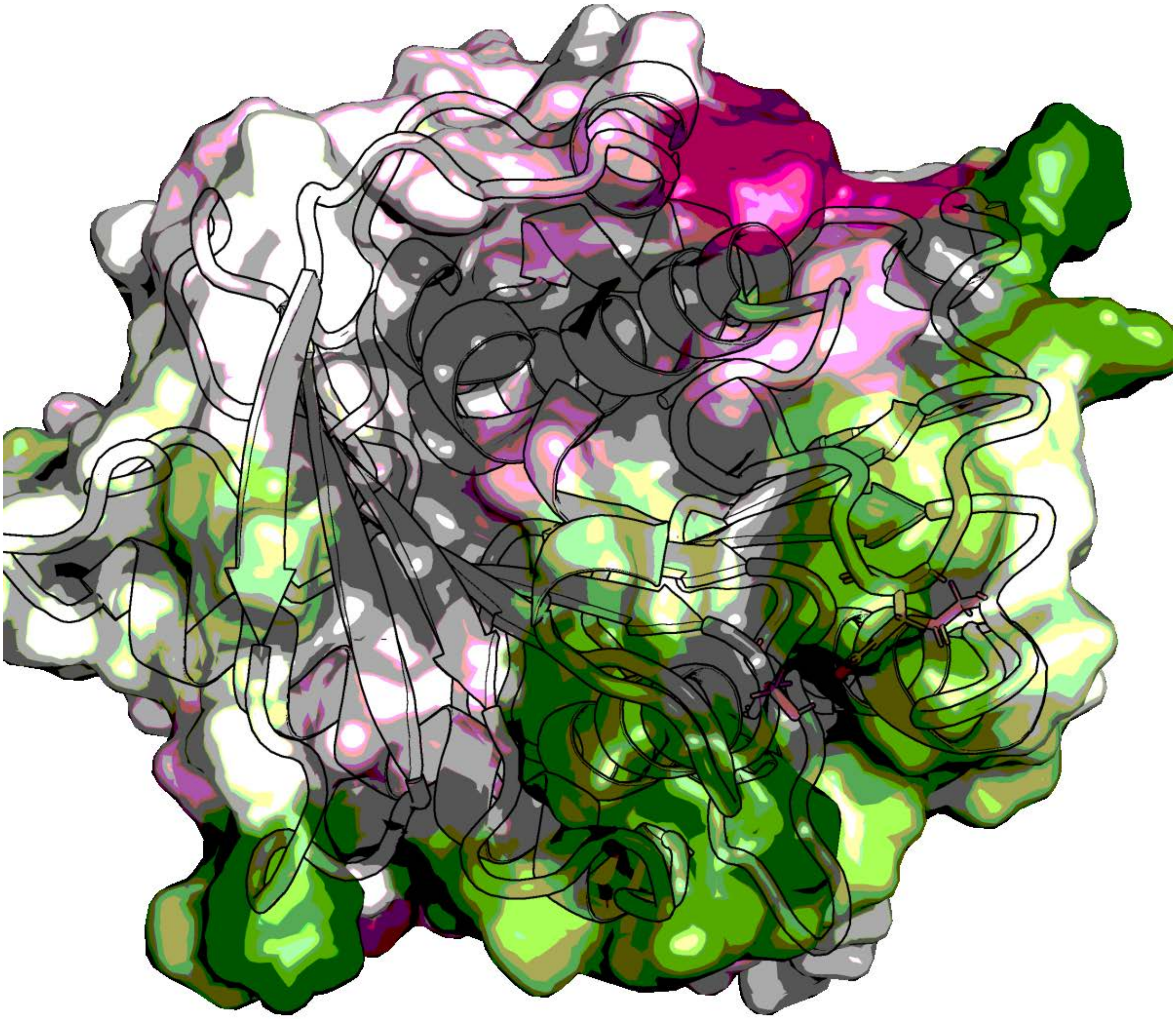

ESM096_highlights.png

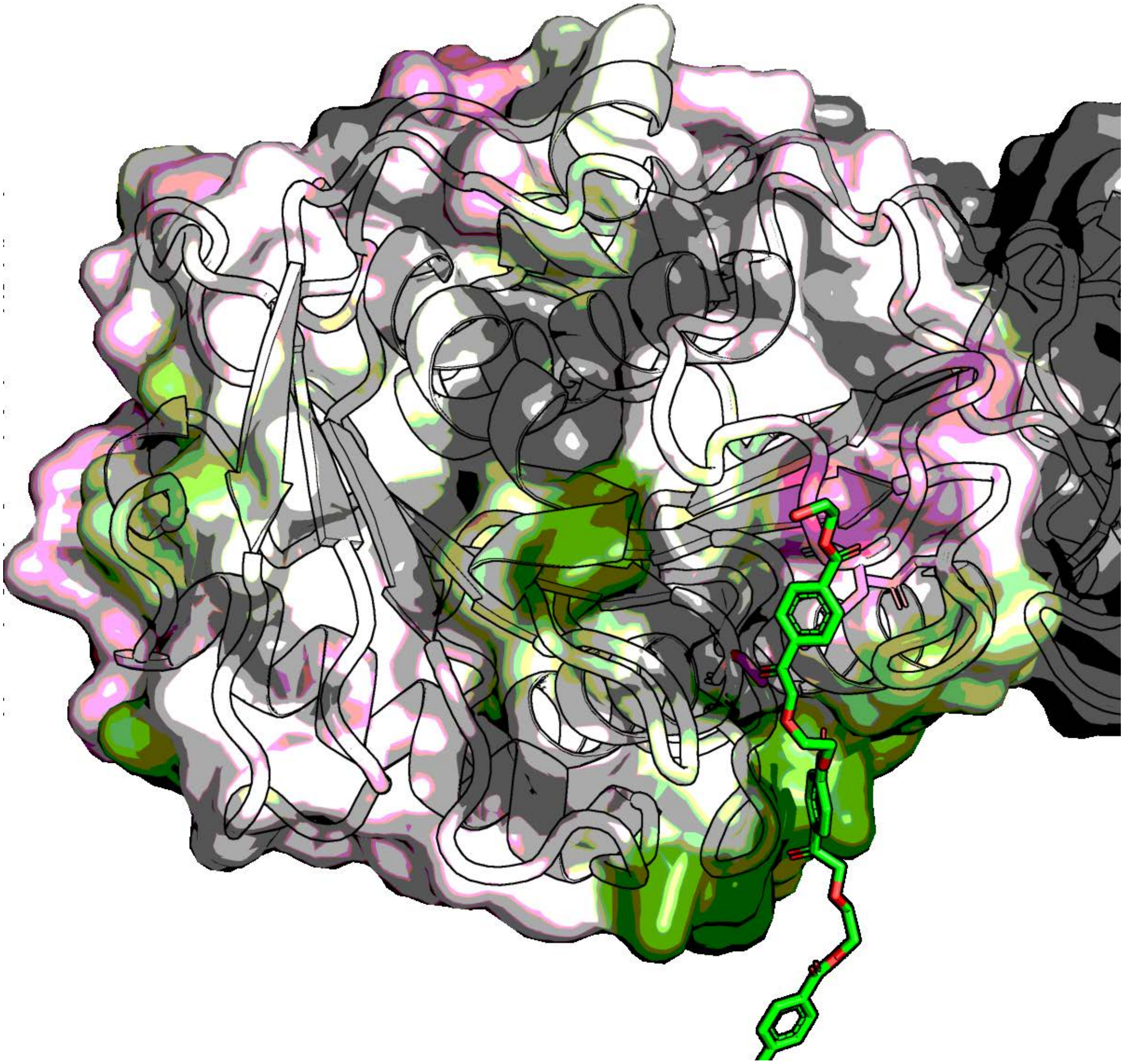

ESM101_highlights.png

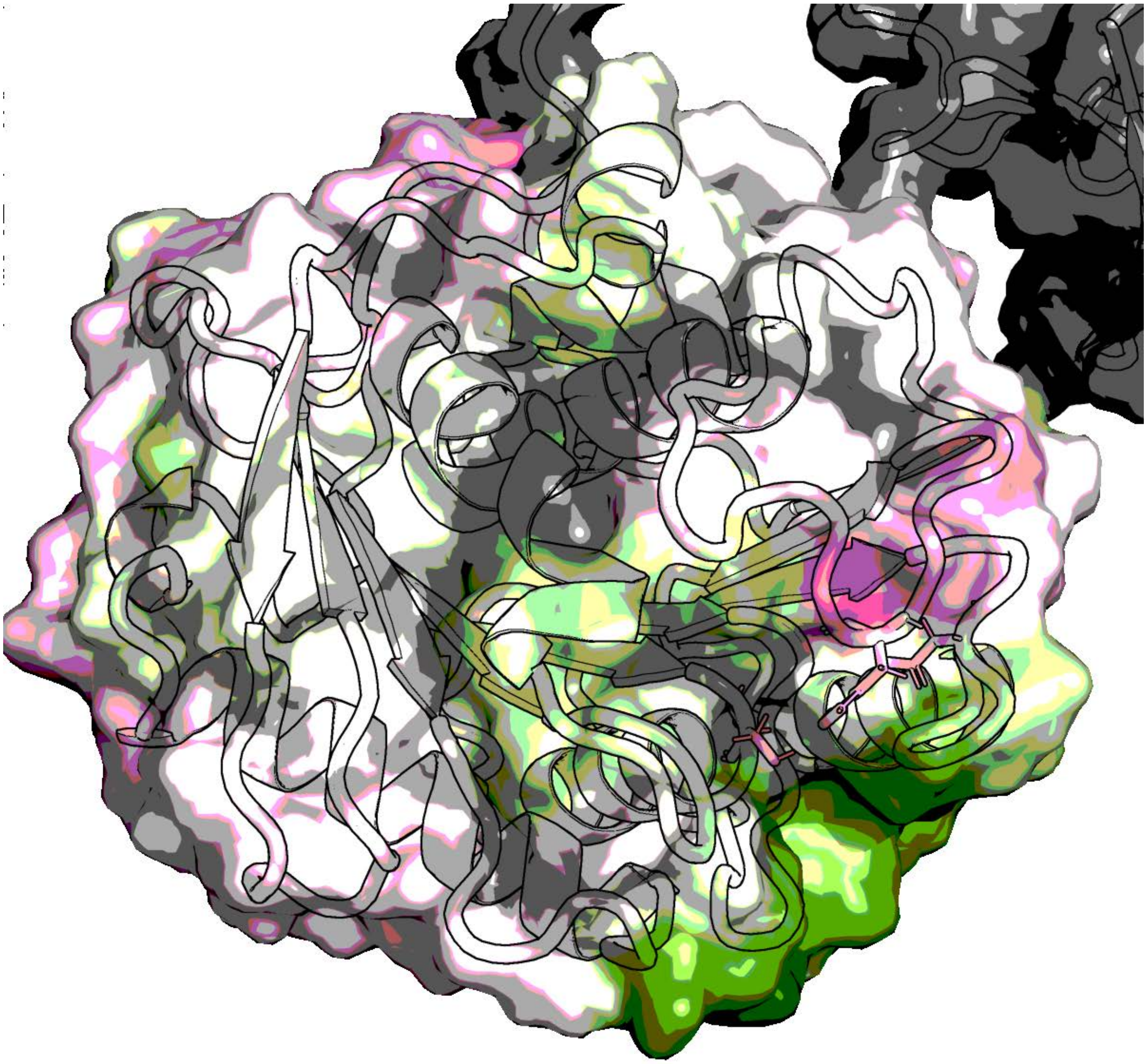

ESM106_highlights.png

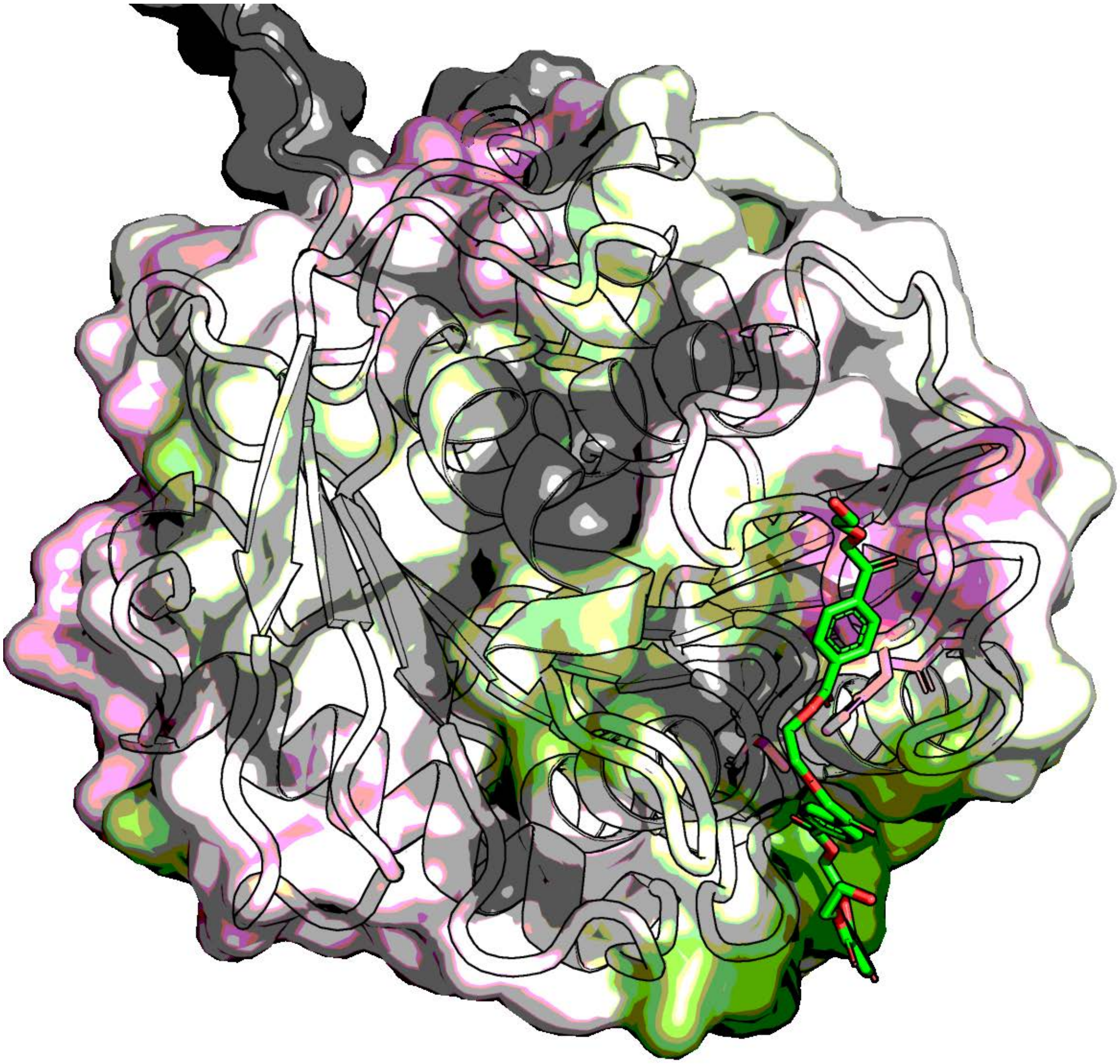

ESM121_highlights.png

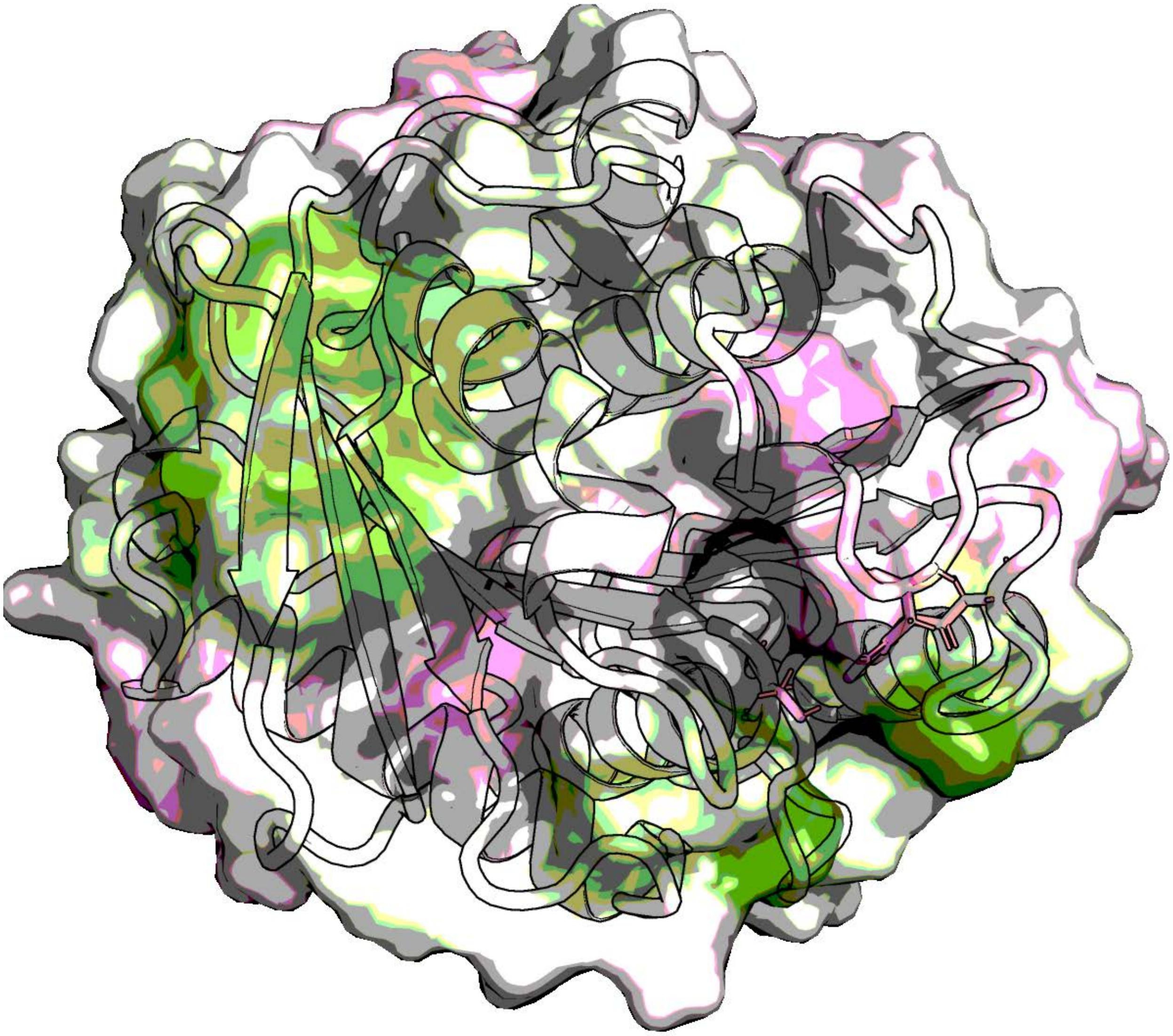

ESM128_highlights.png

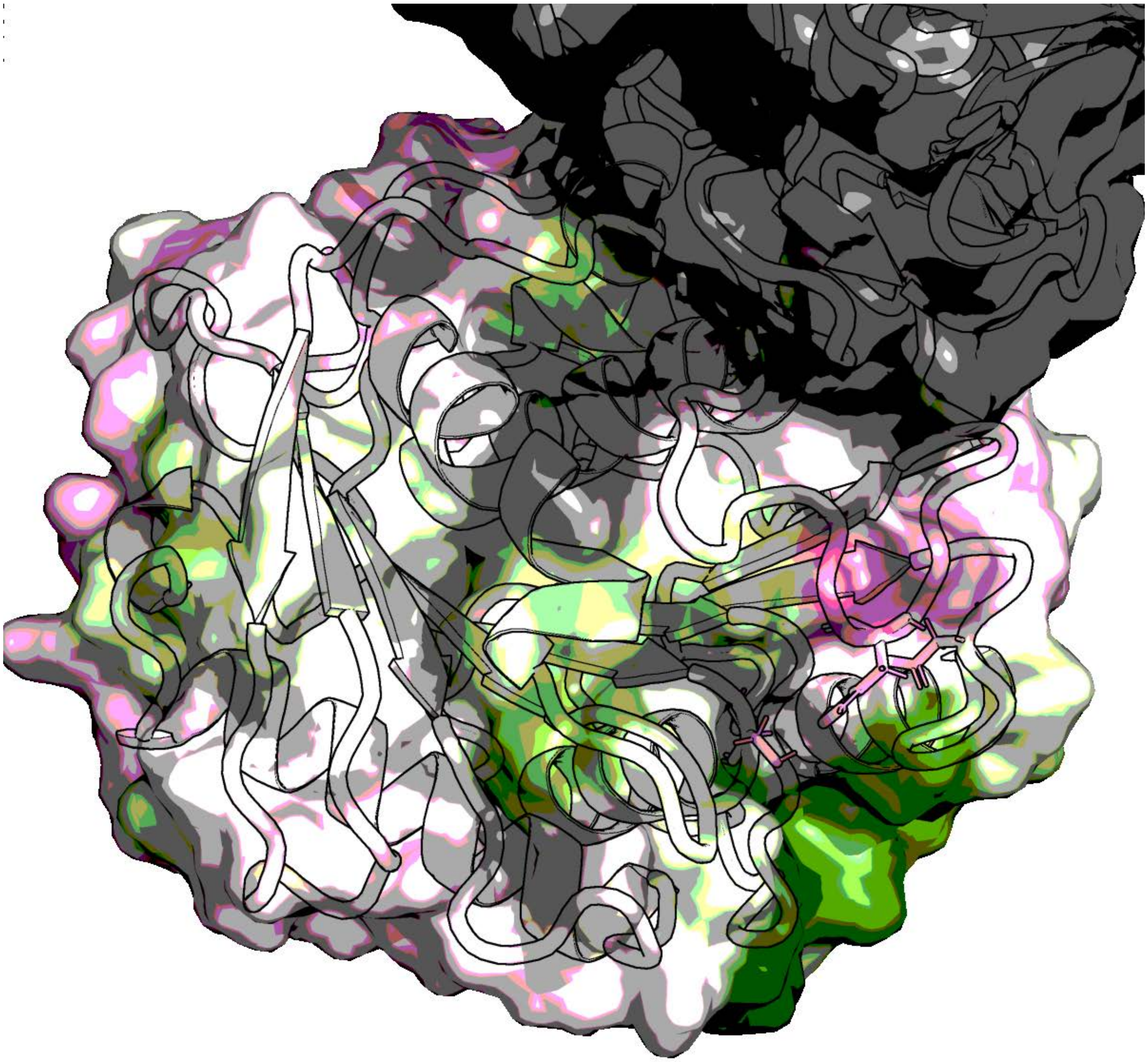

ESM130_highlights.png

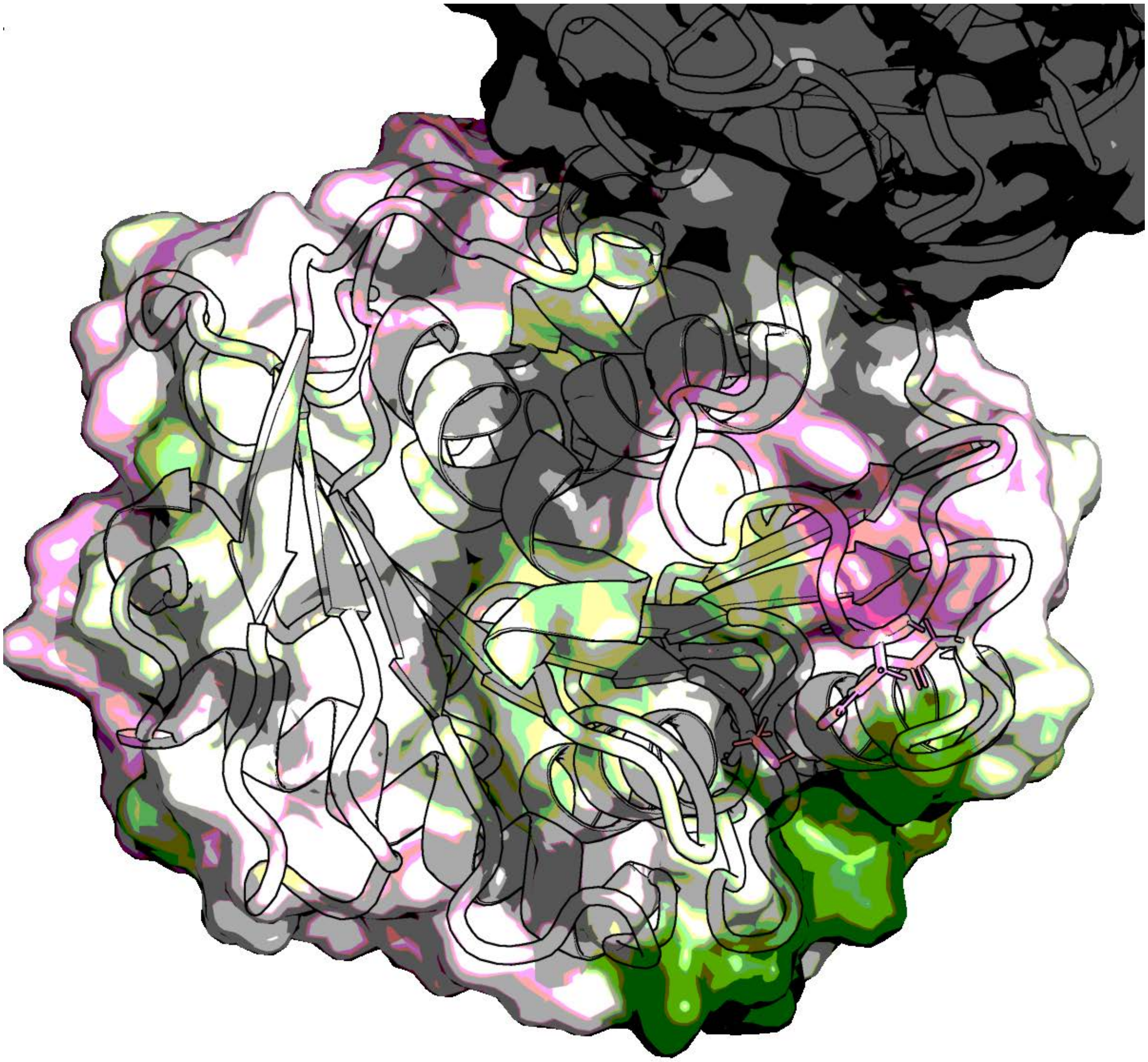

ESM132_highlights.png

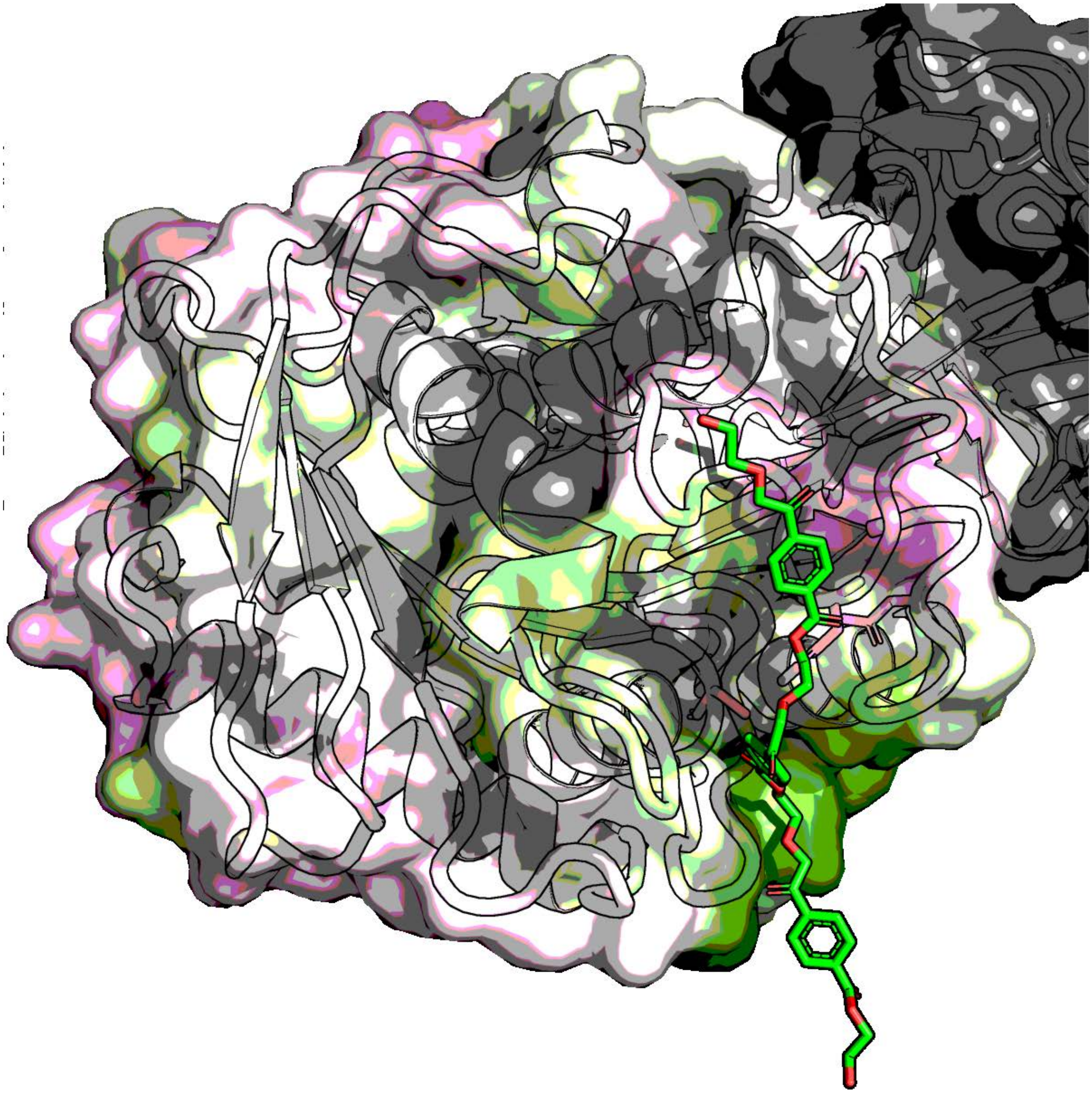

ESM135_highlights.png

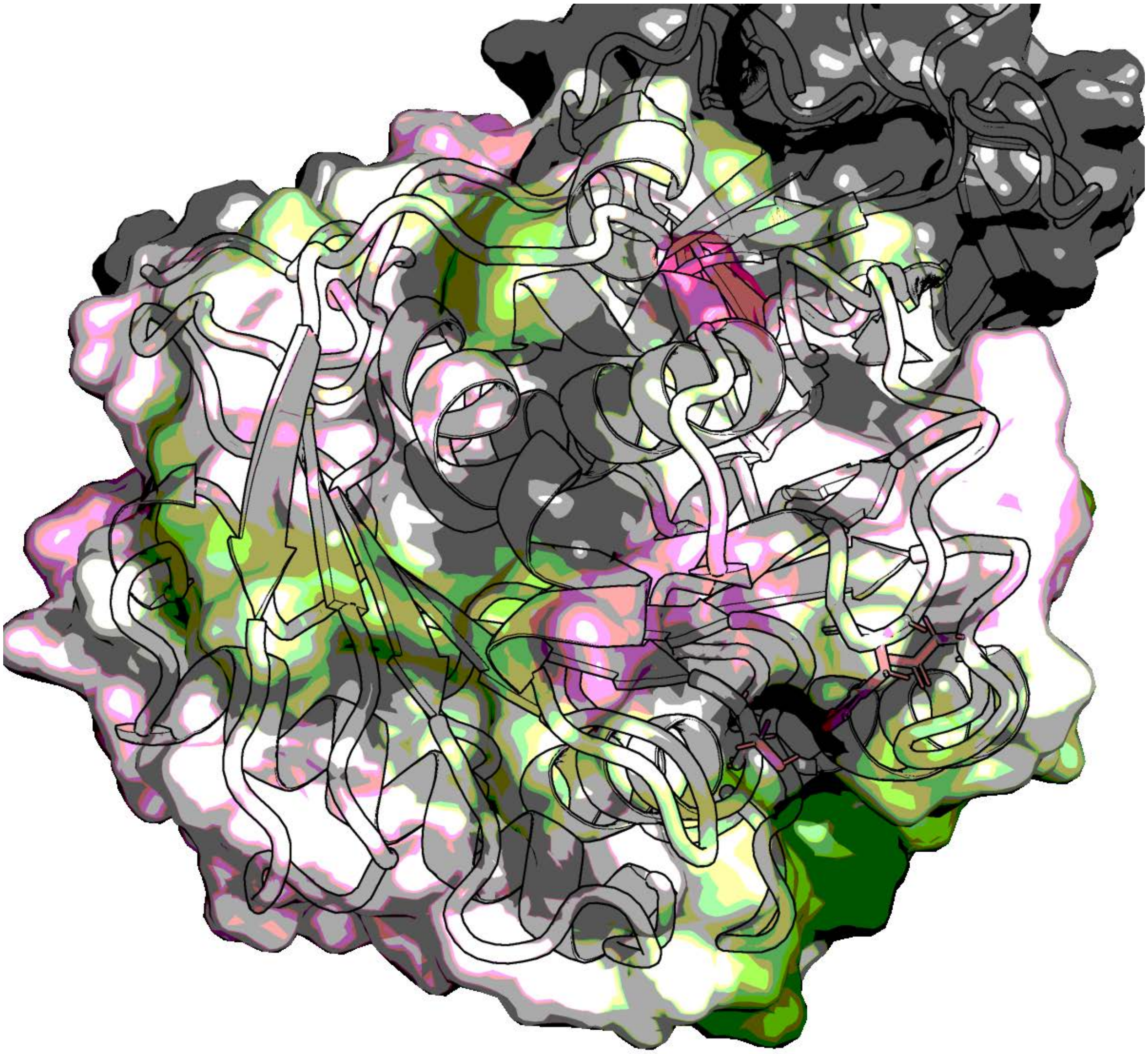

ESM136_highlights.png

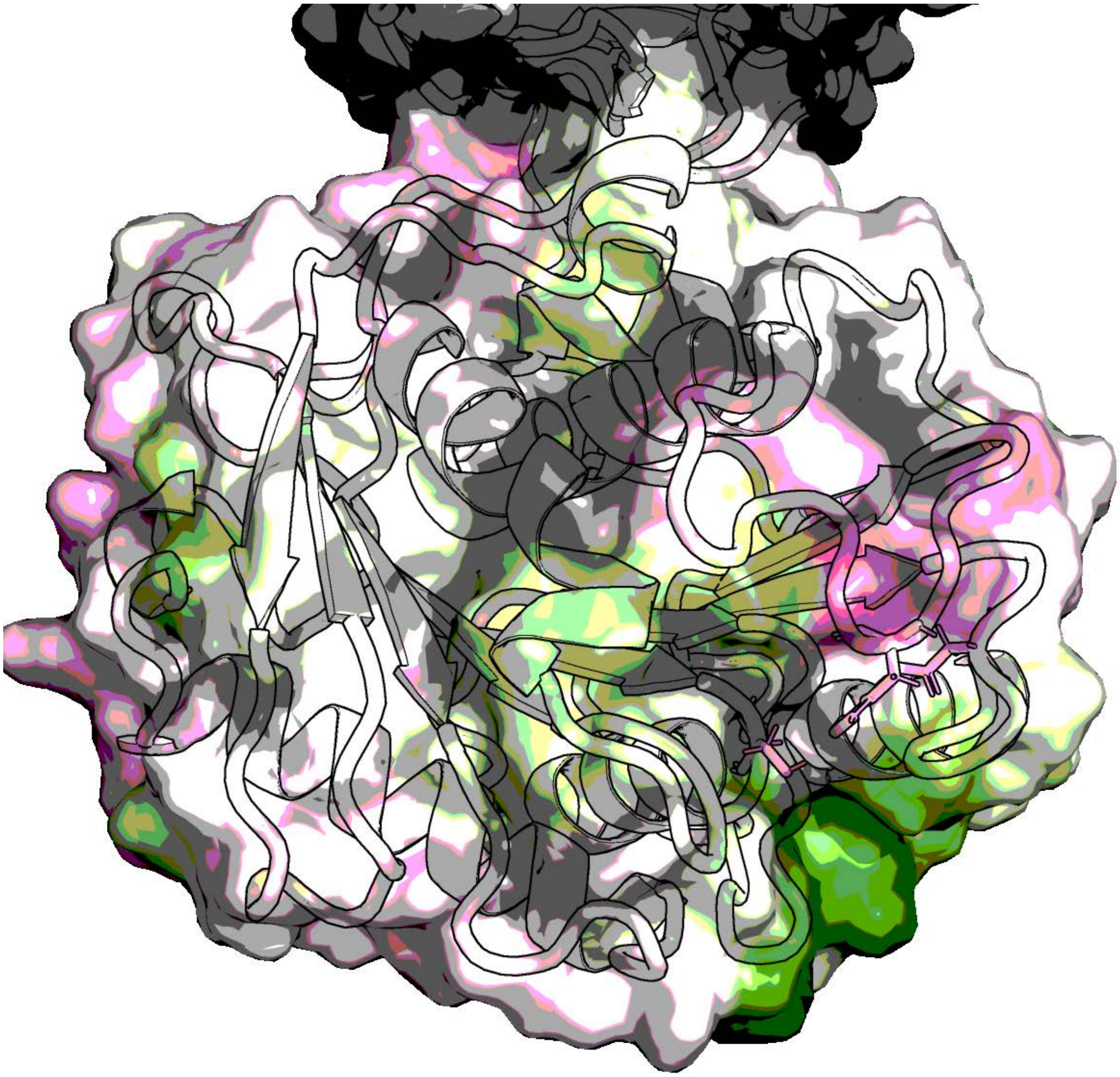

ESM137_highlights.png

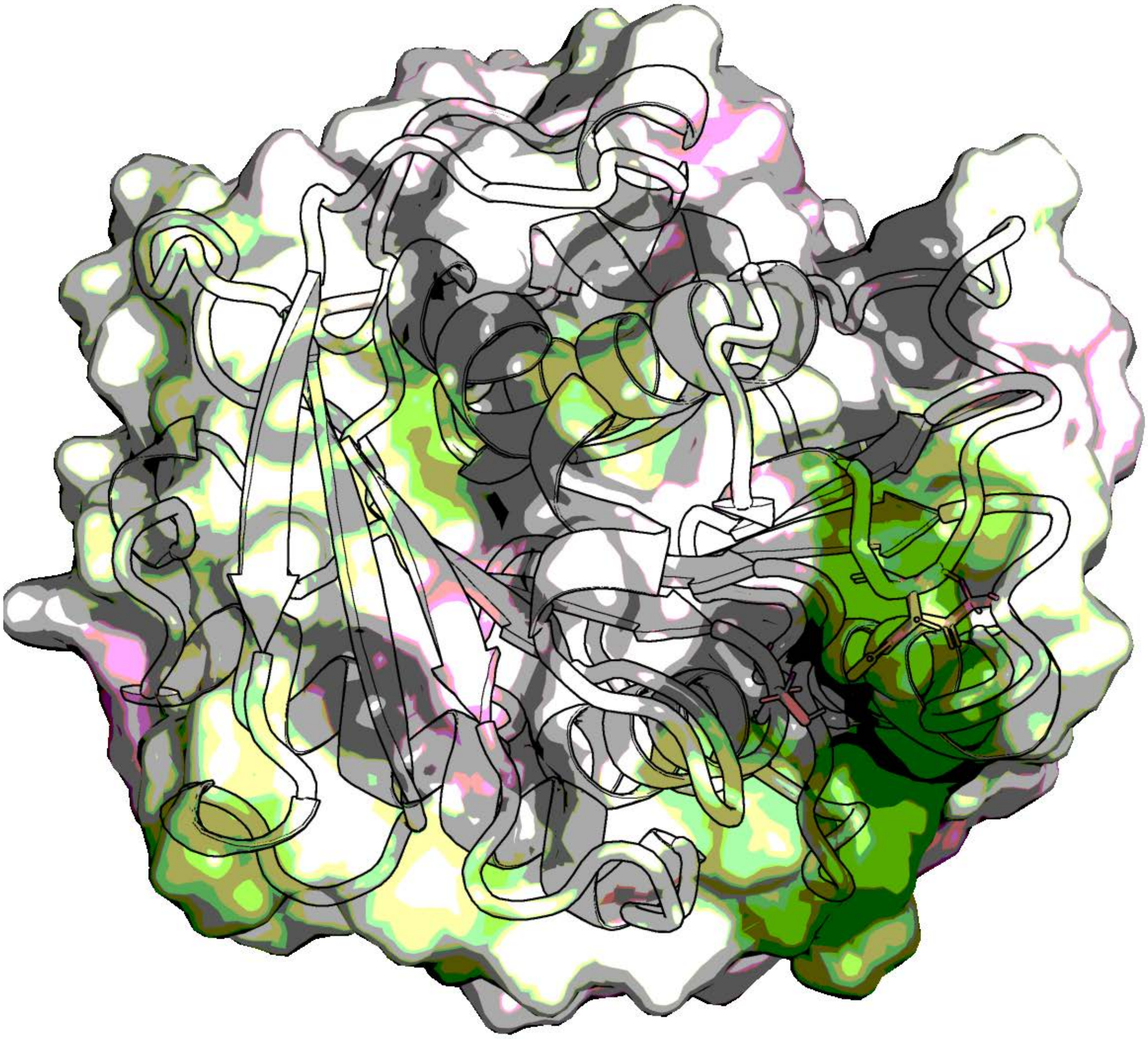

ESM140_highlights.png

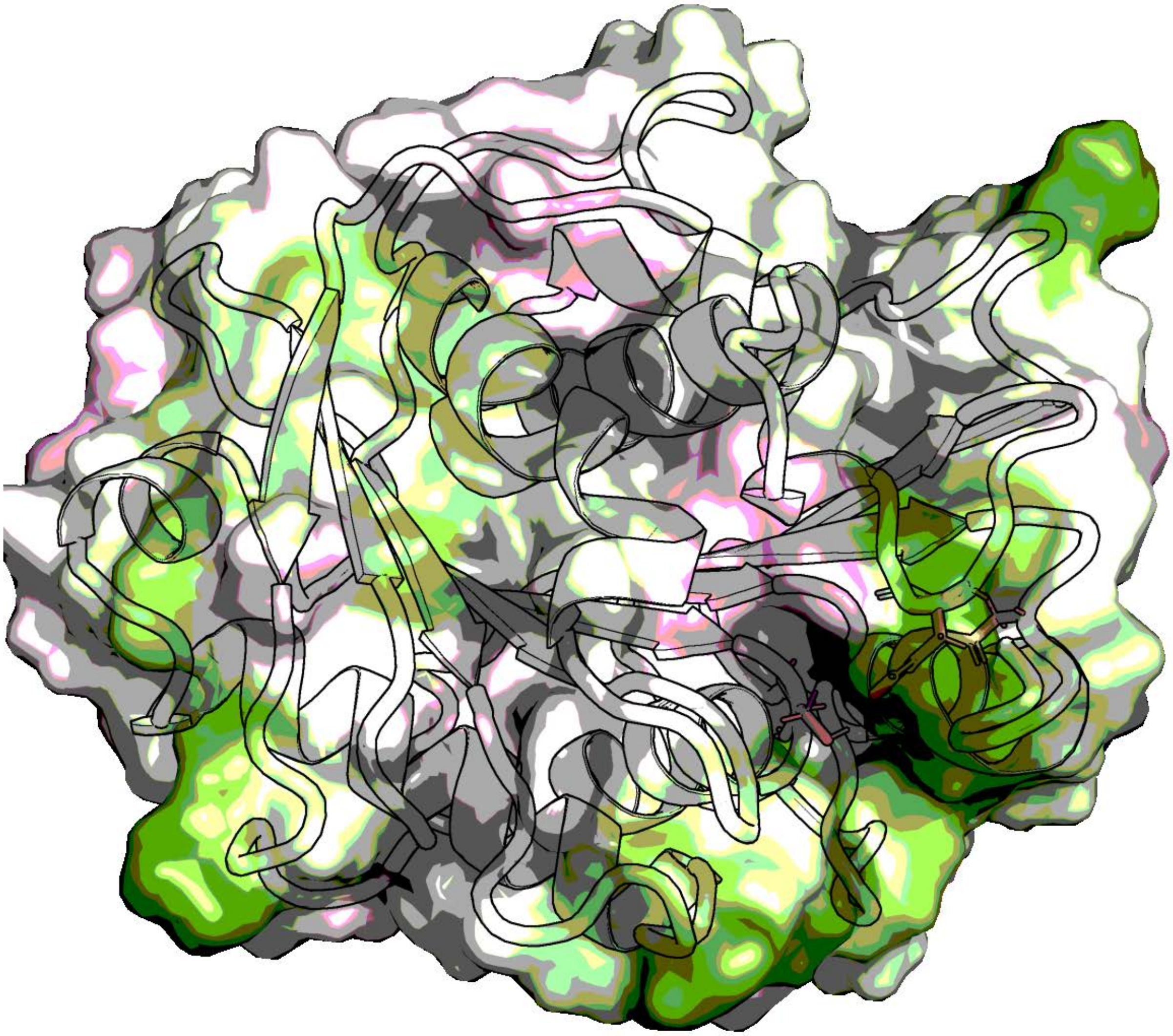

ESM144_highlights.png

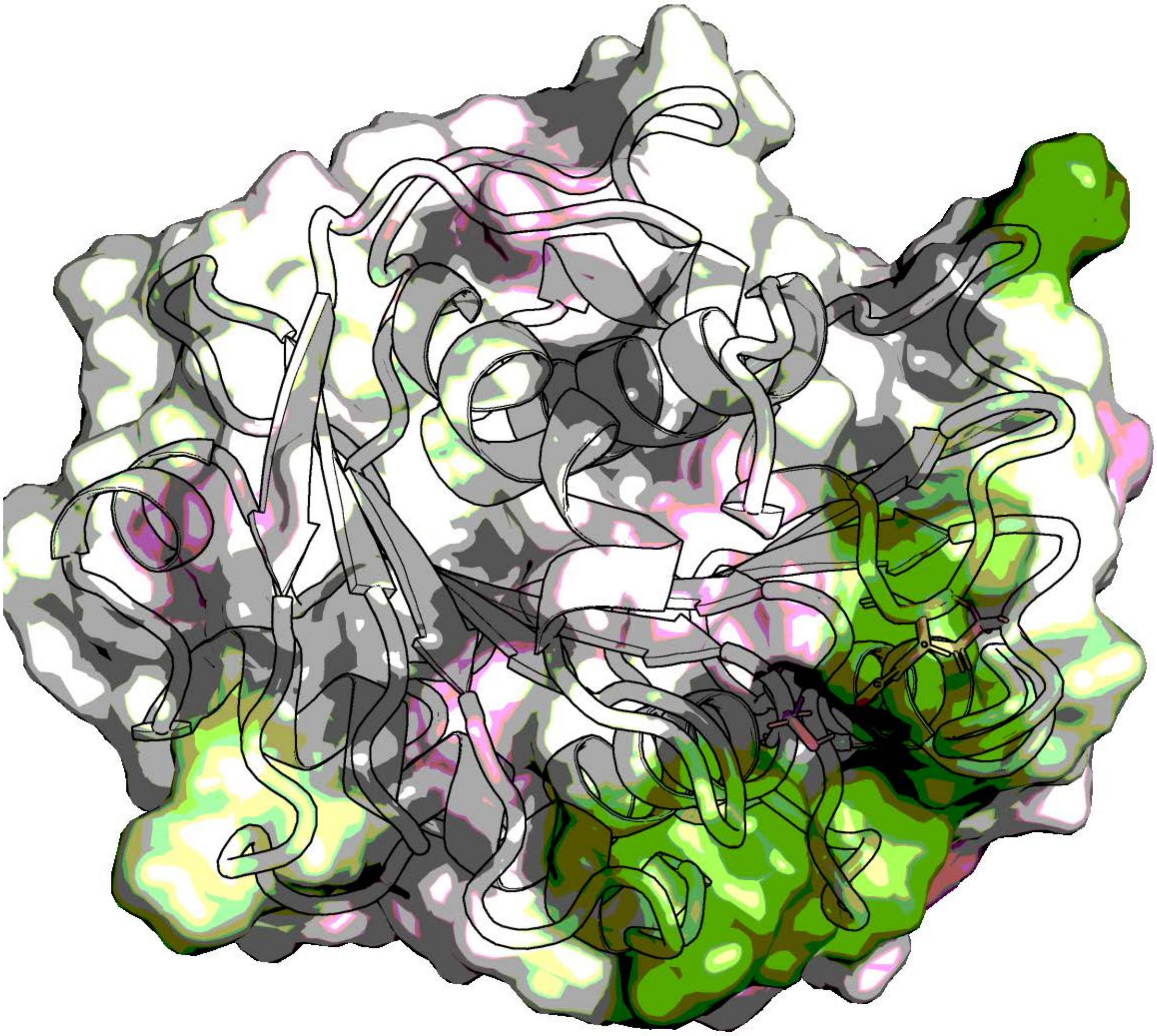

ESM147_highlights.png

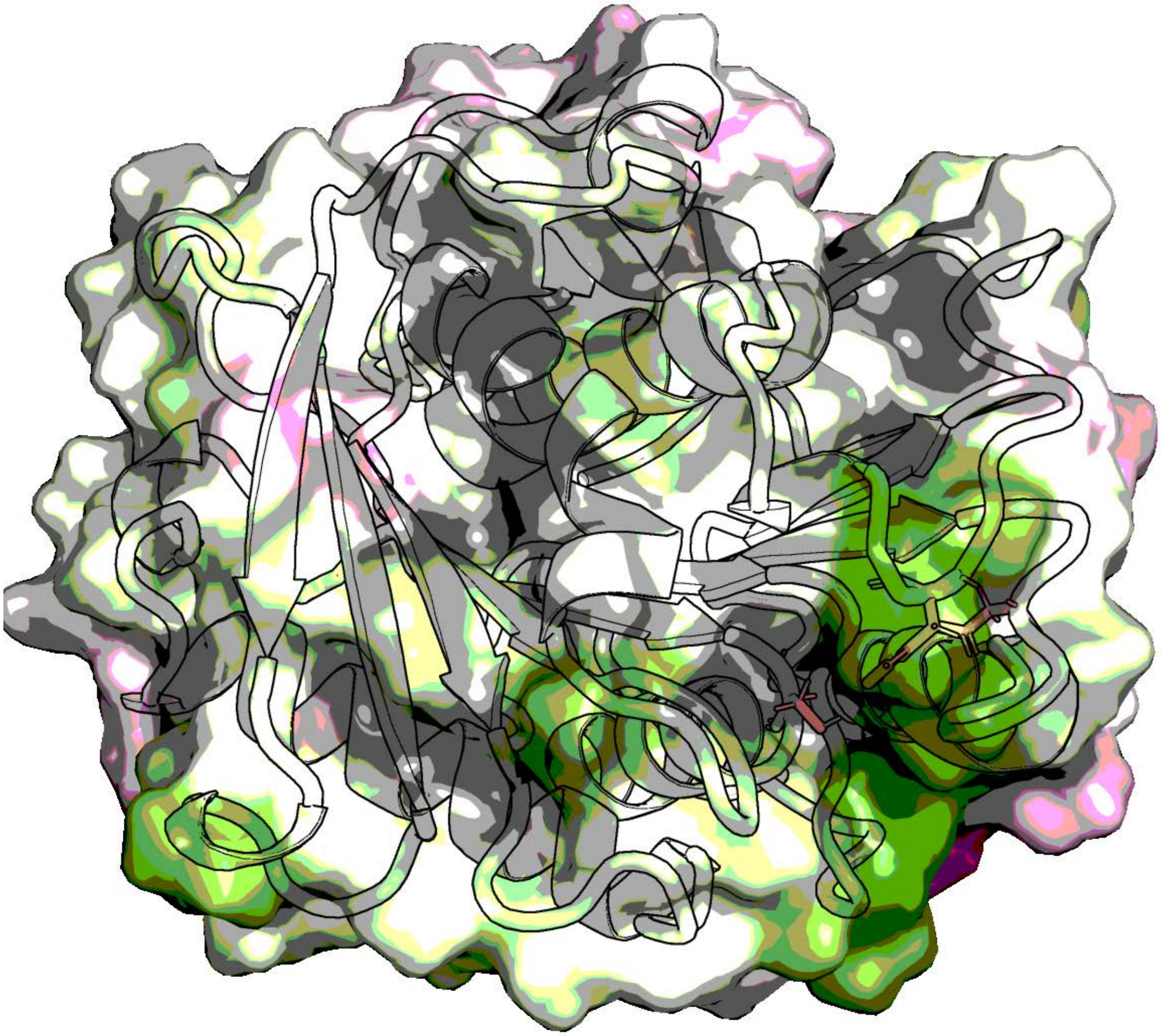

ESM156_highlights.png

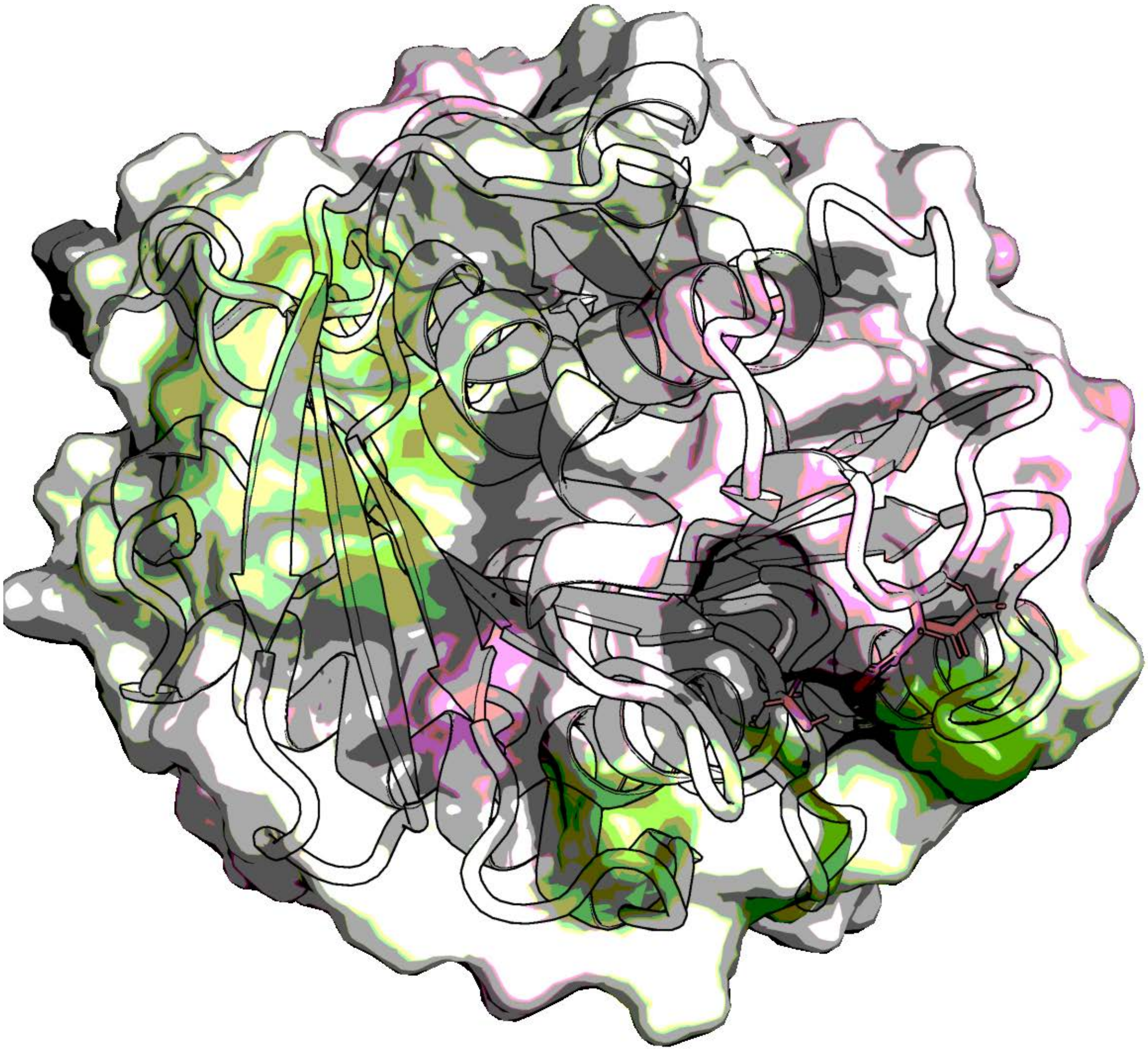

ESM157_highlights.png

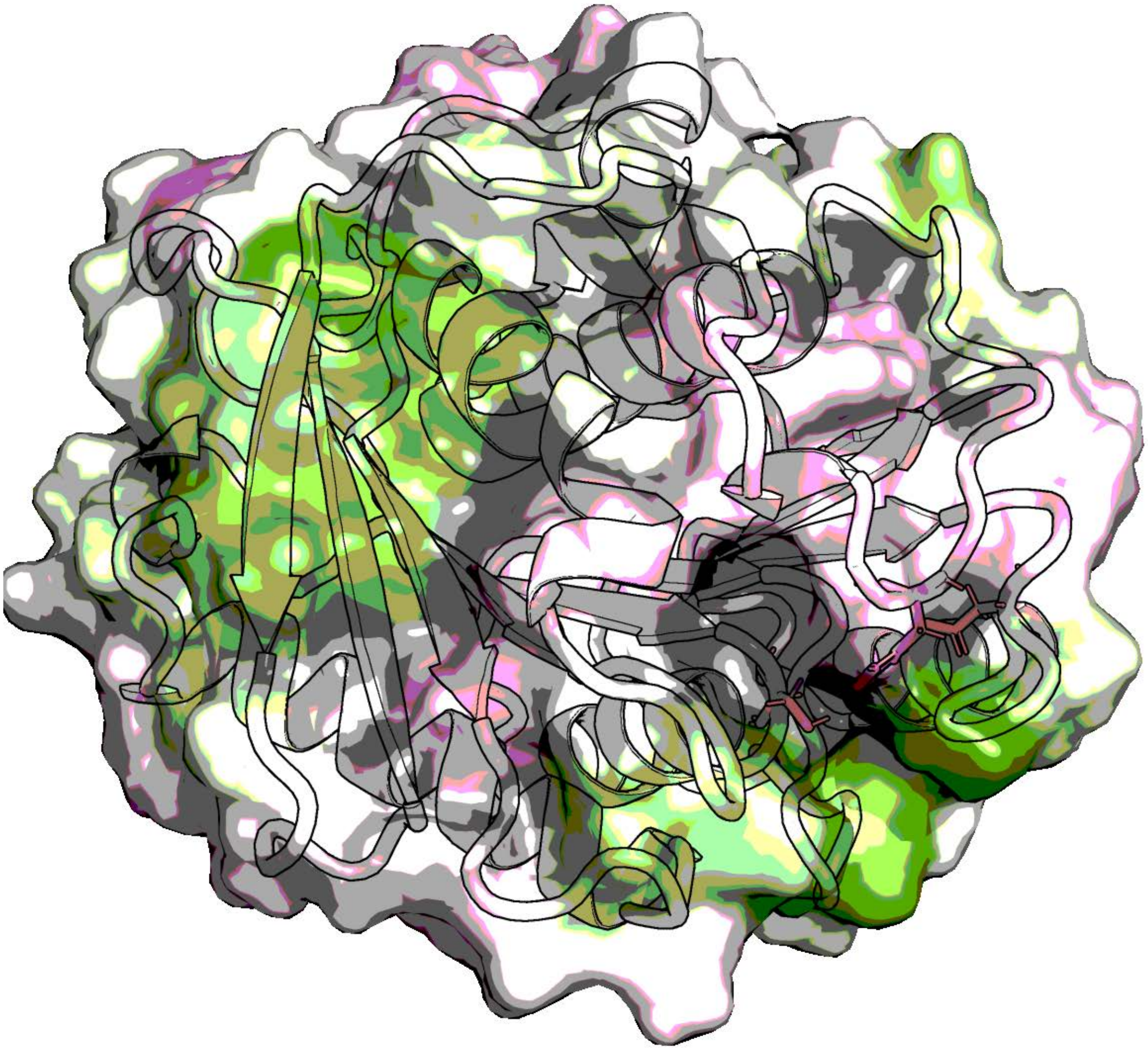

ESM159_highlights.png

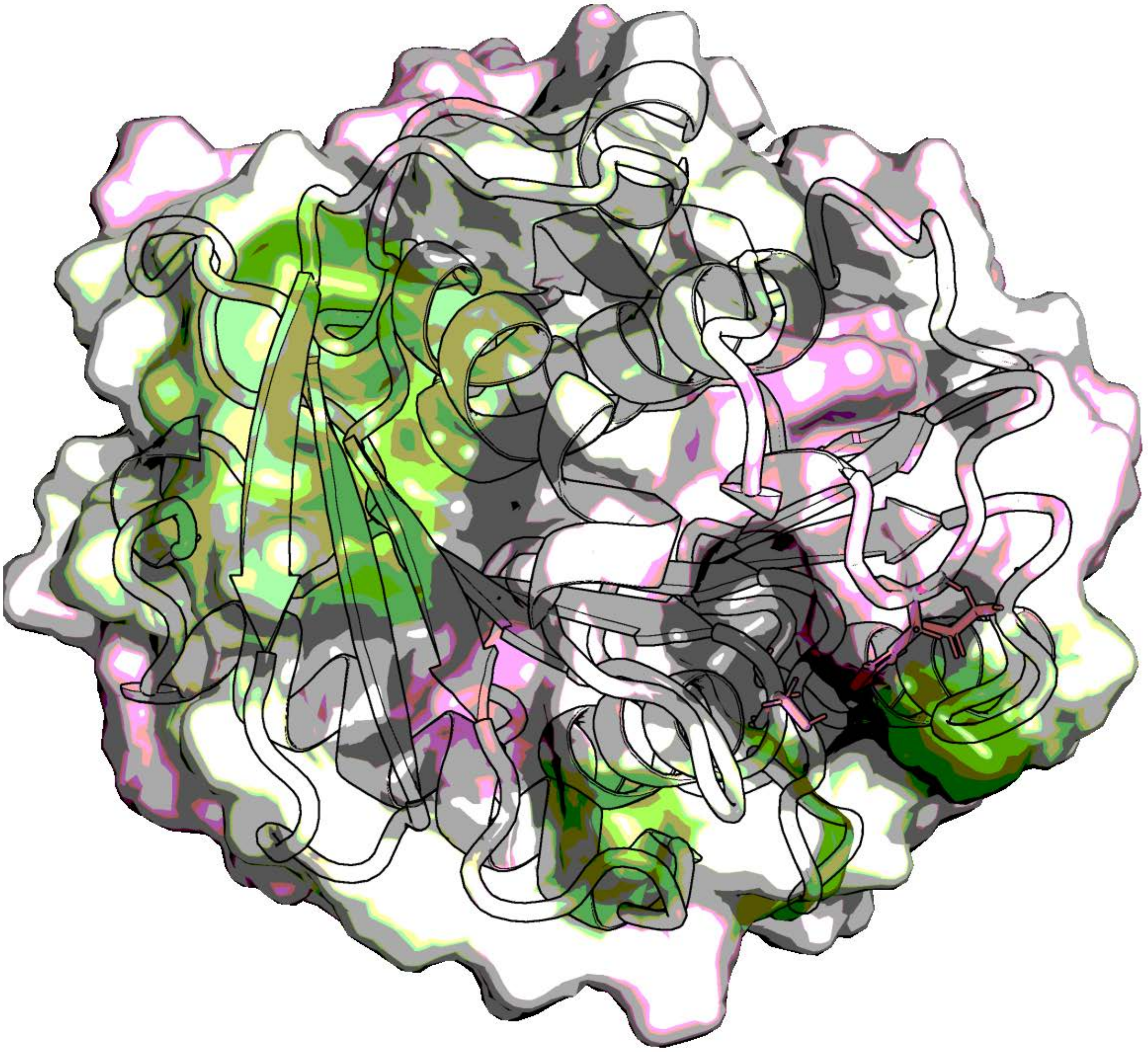

ESM160_highlights.png

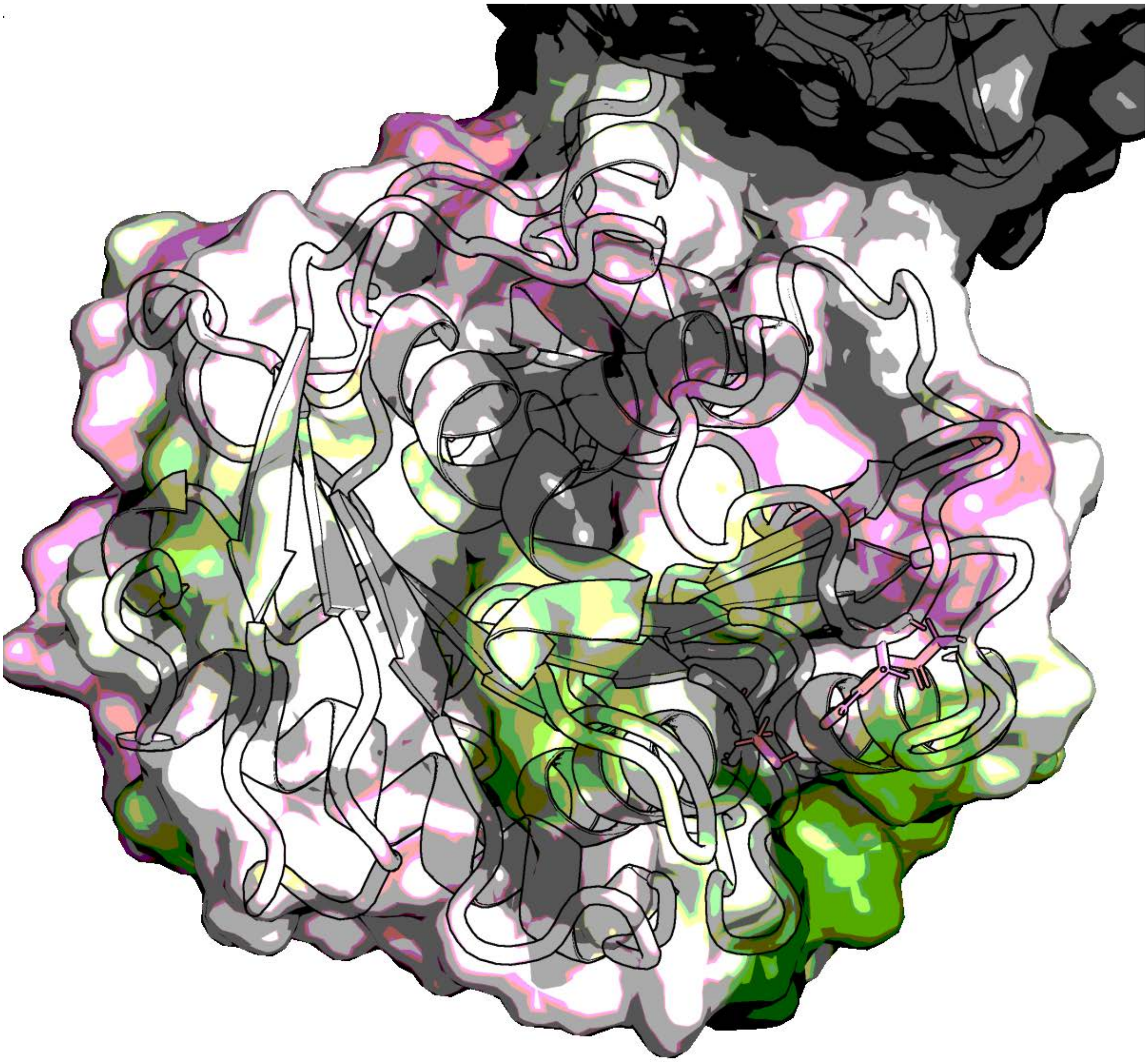

ESM161_highlights.png

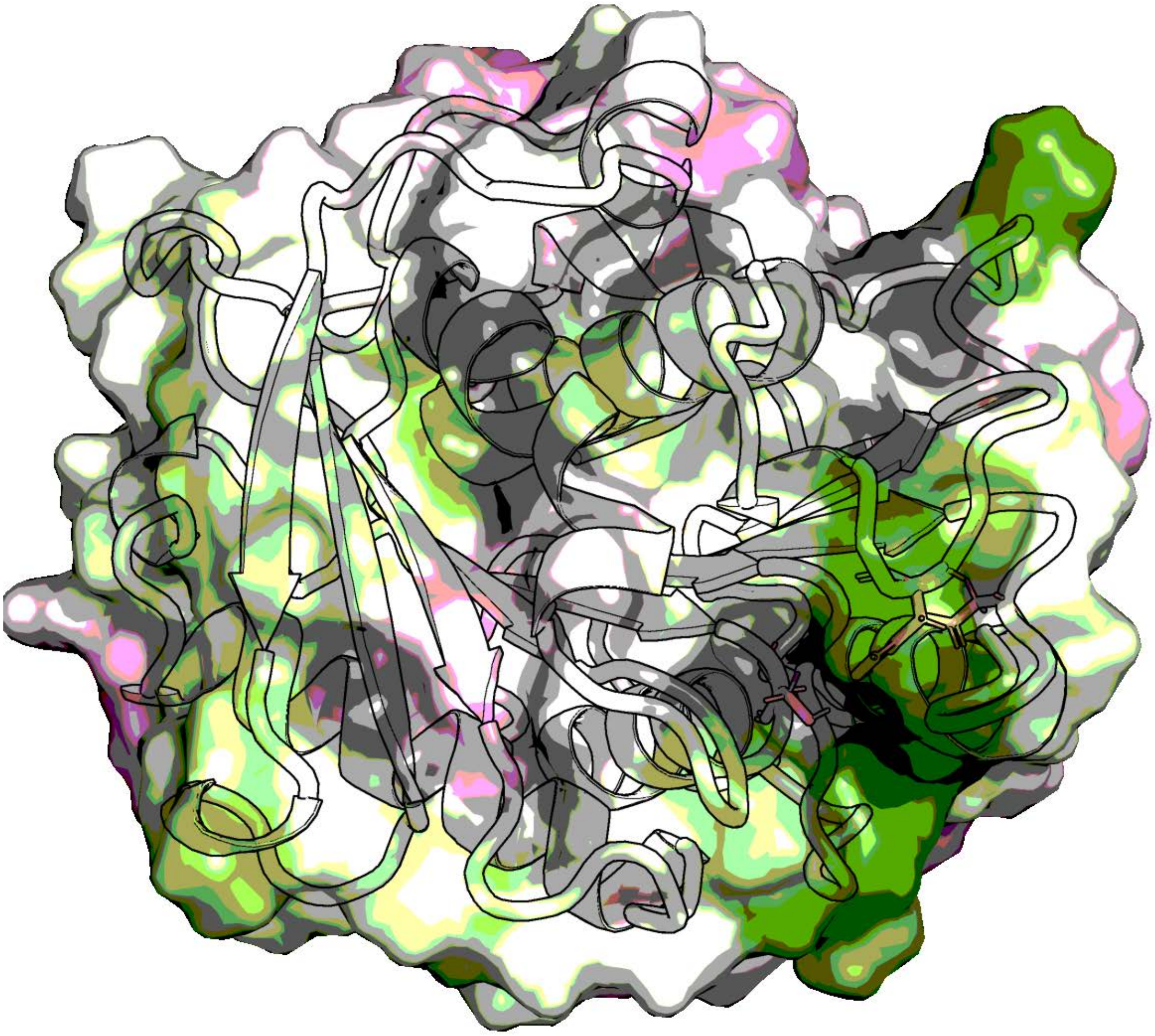

ESM167_highlights.png

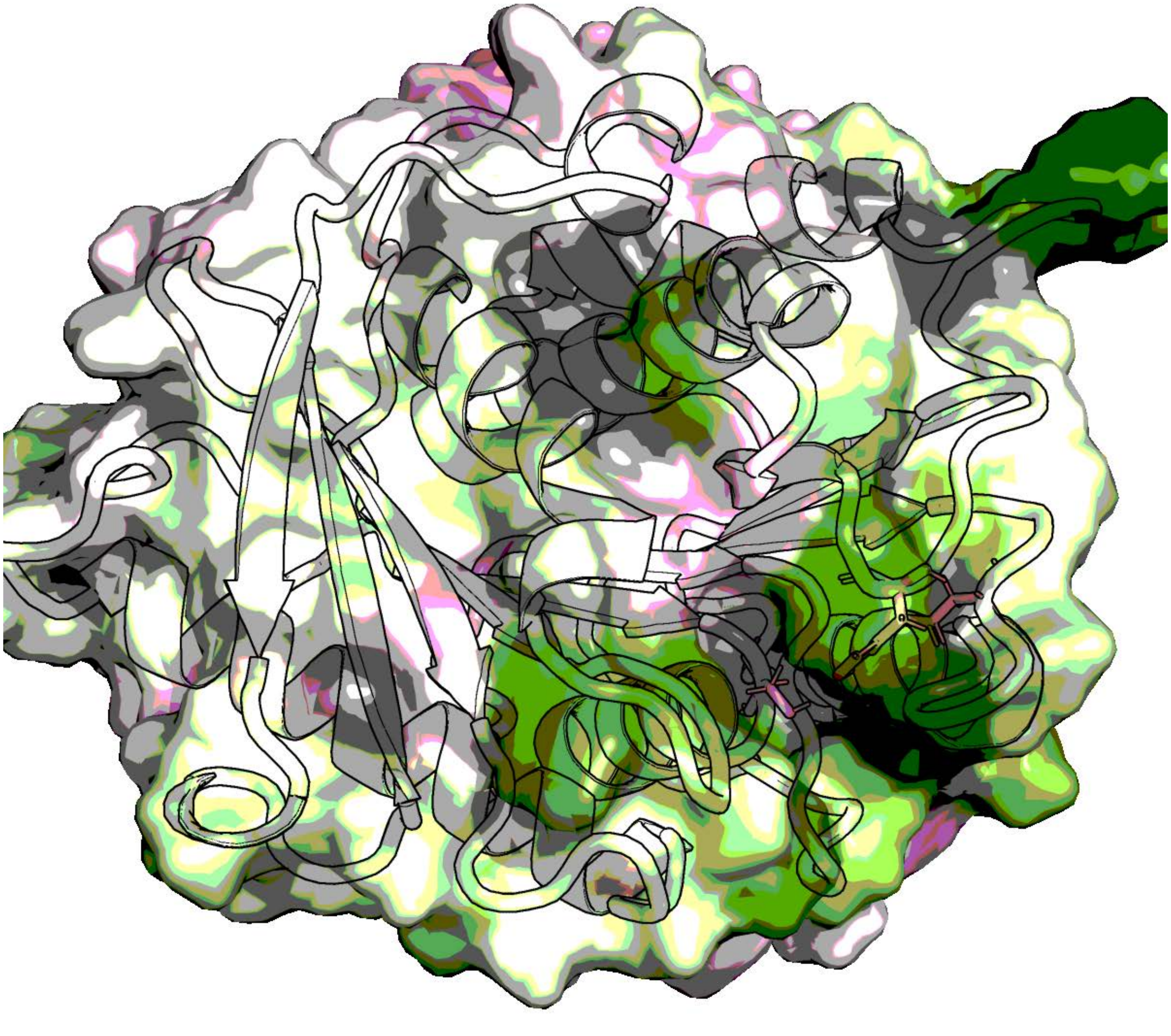

ESM169_highlights.png

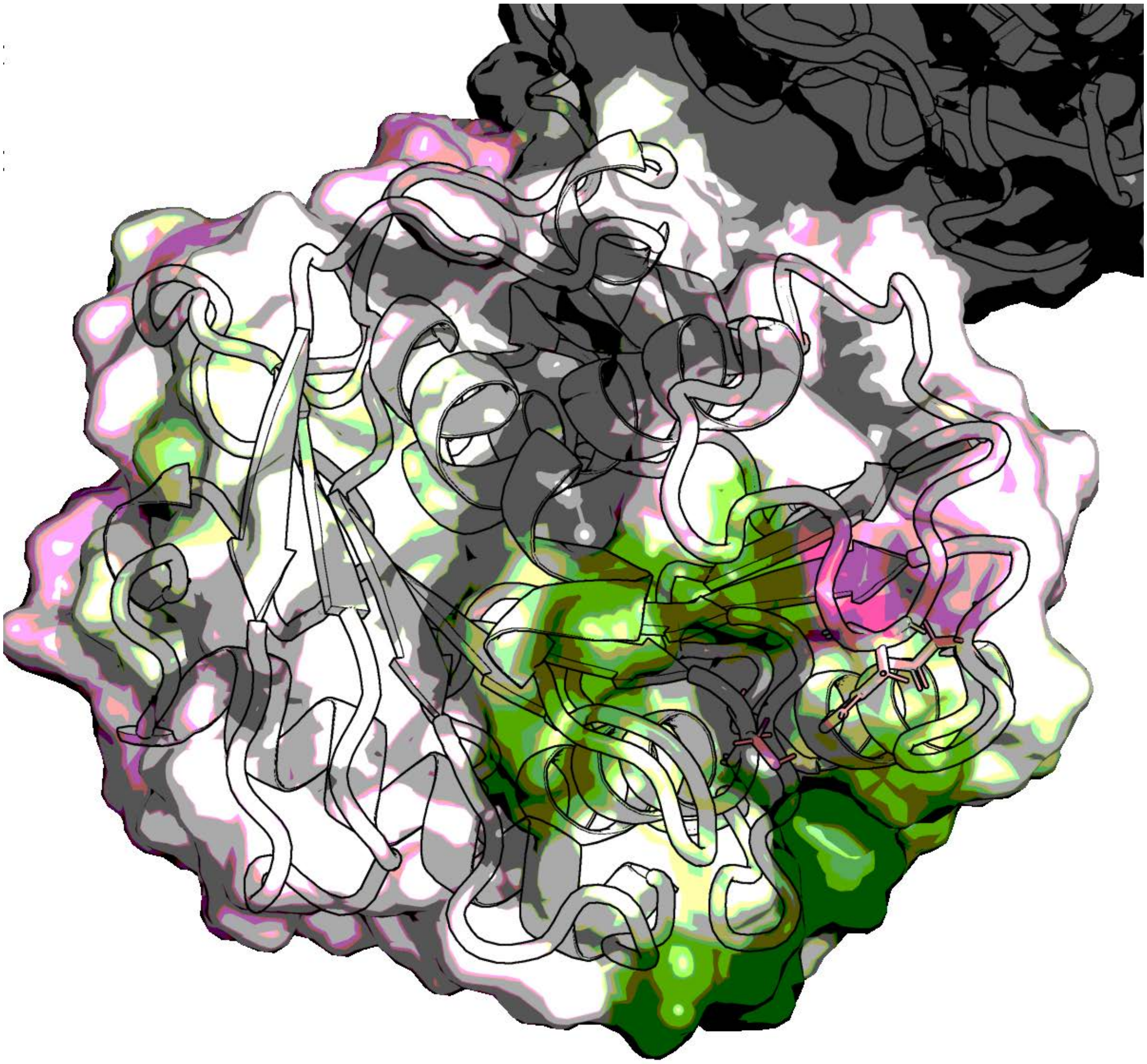

ESM173_highlights.png

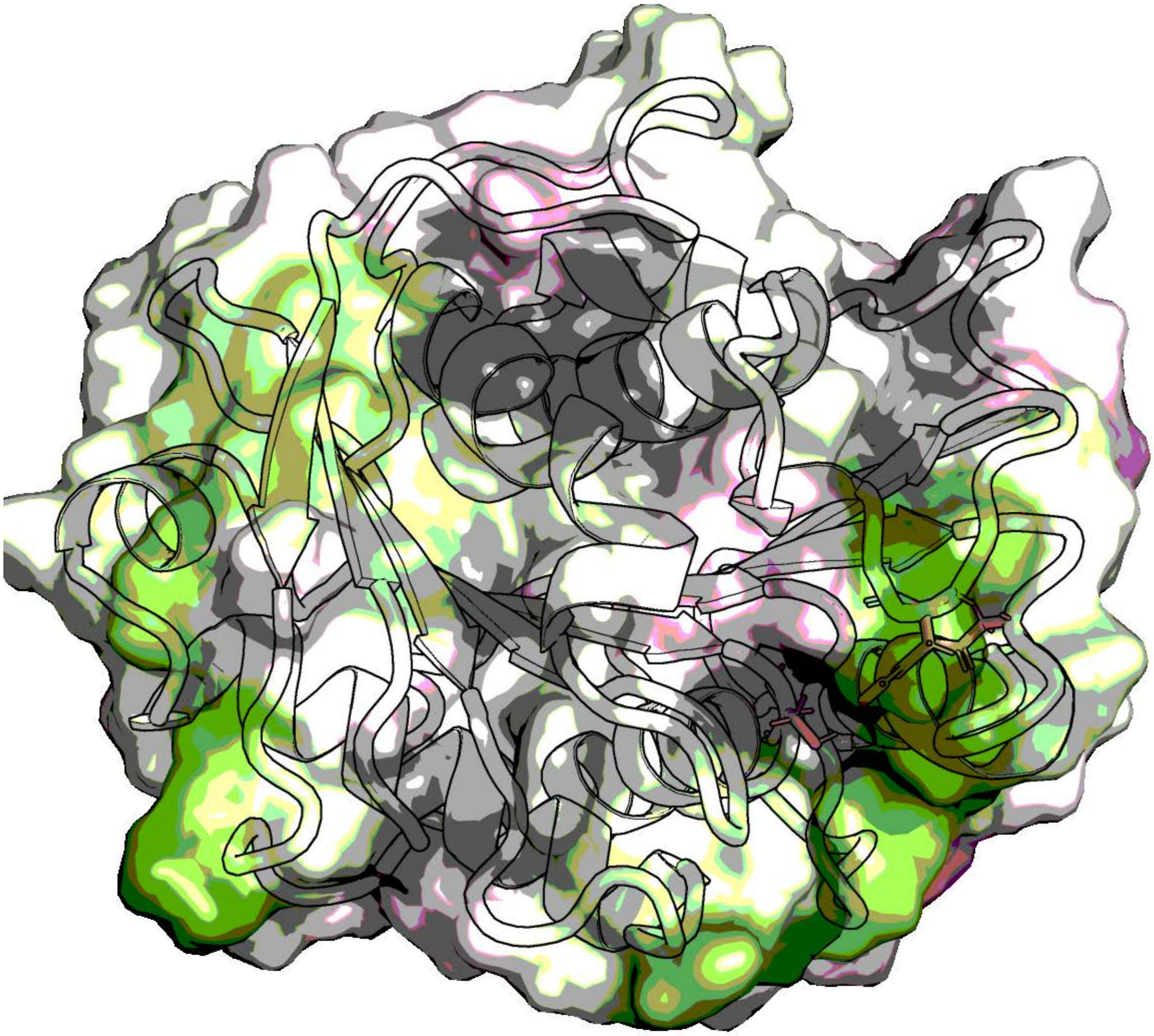

ESM177_highlights.png

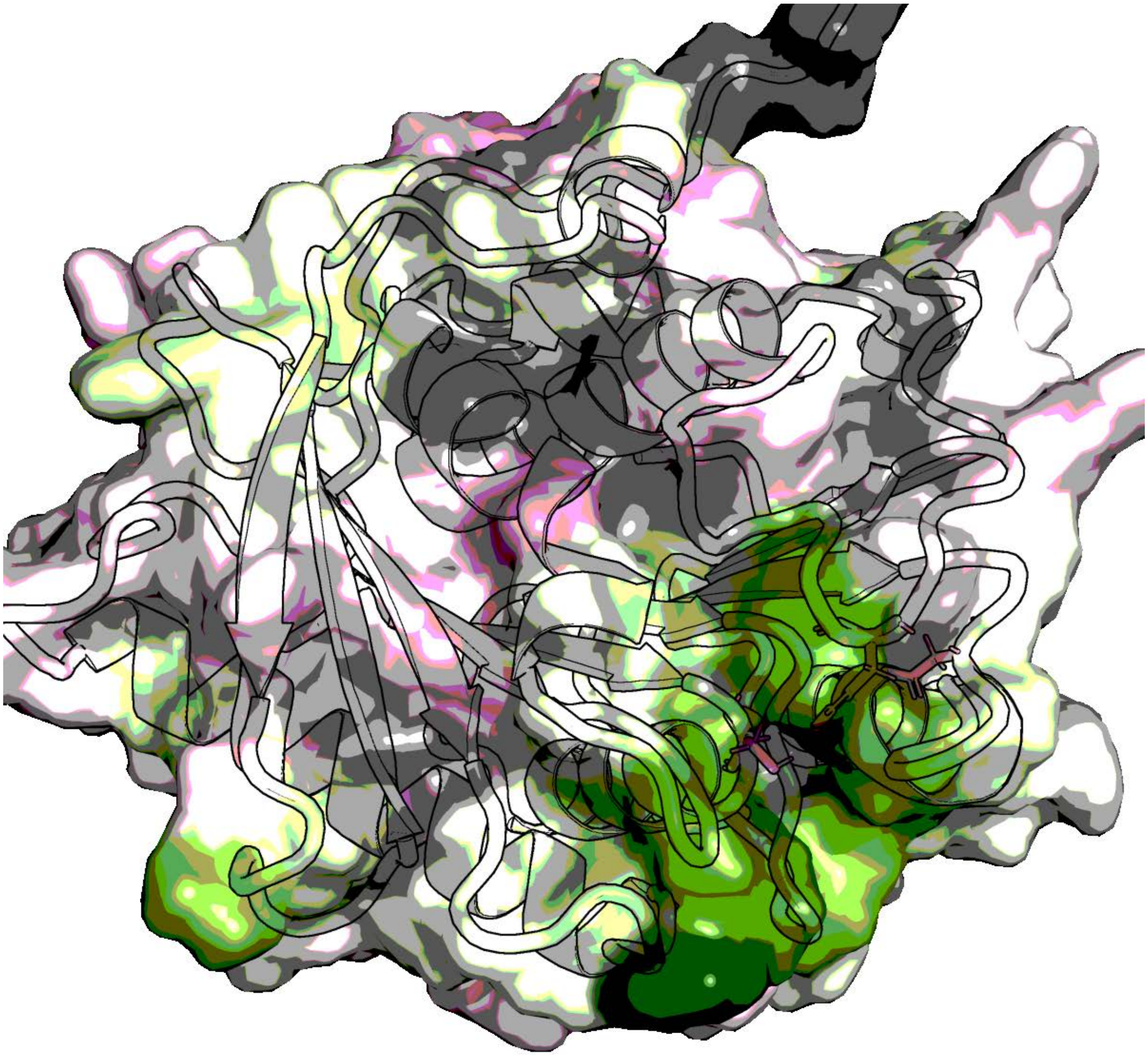

ESM186_highlights.png

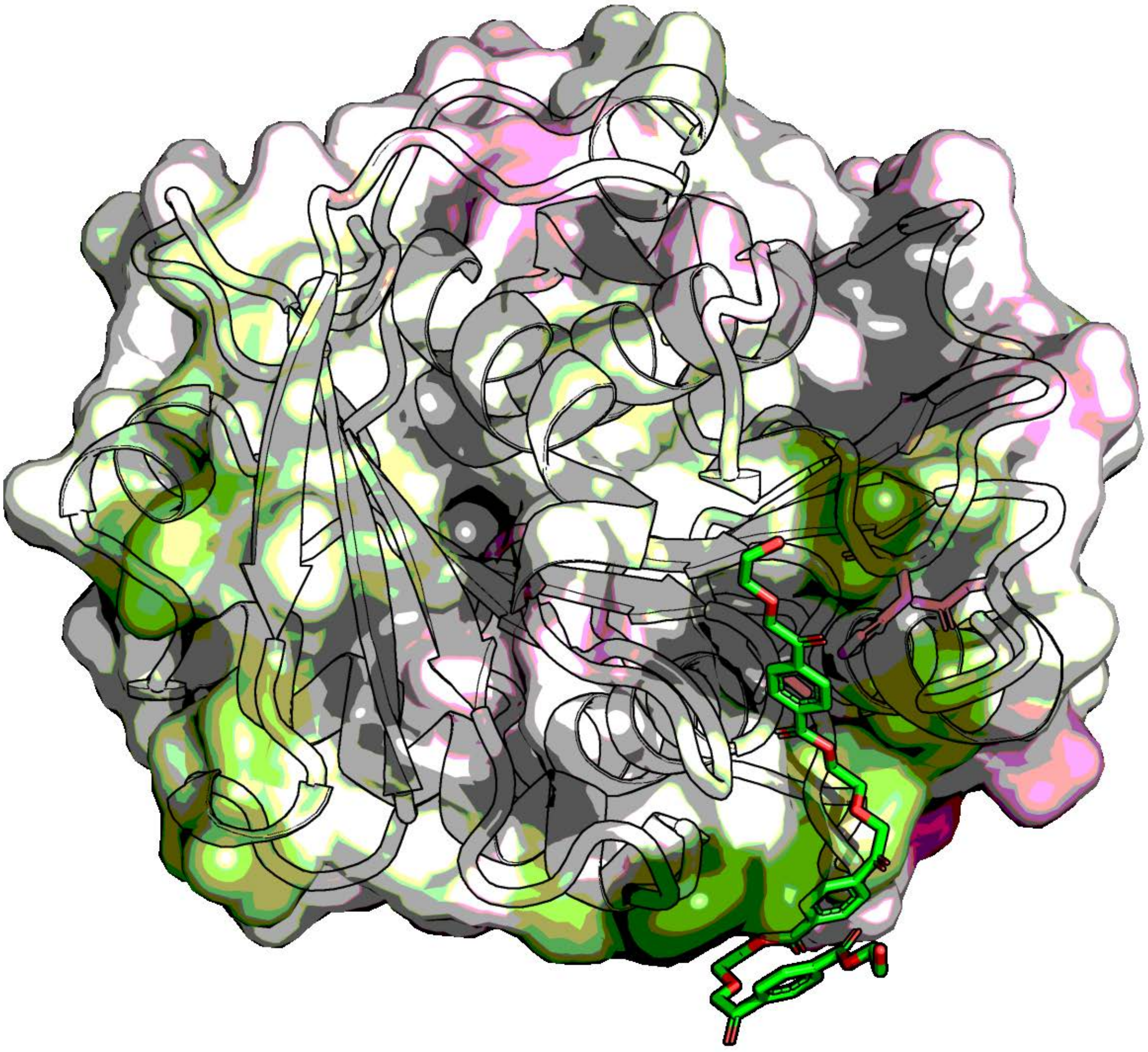

LCC-ICCG_highlights.png

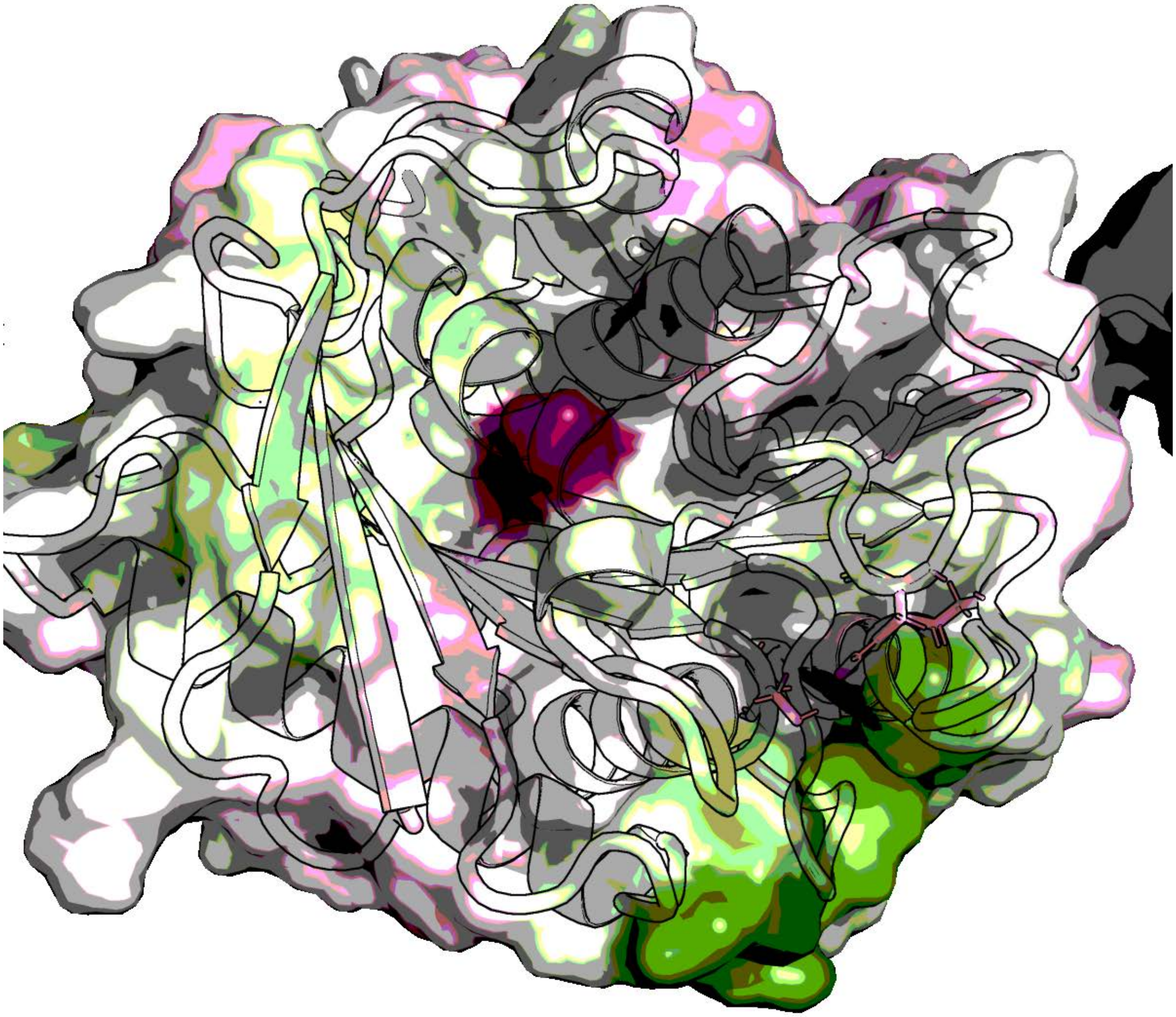

TEP004_highlights.png

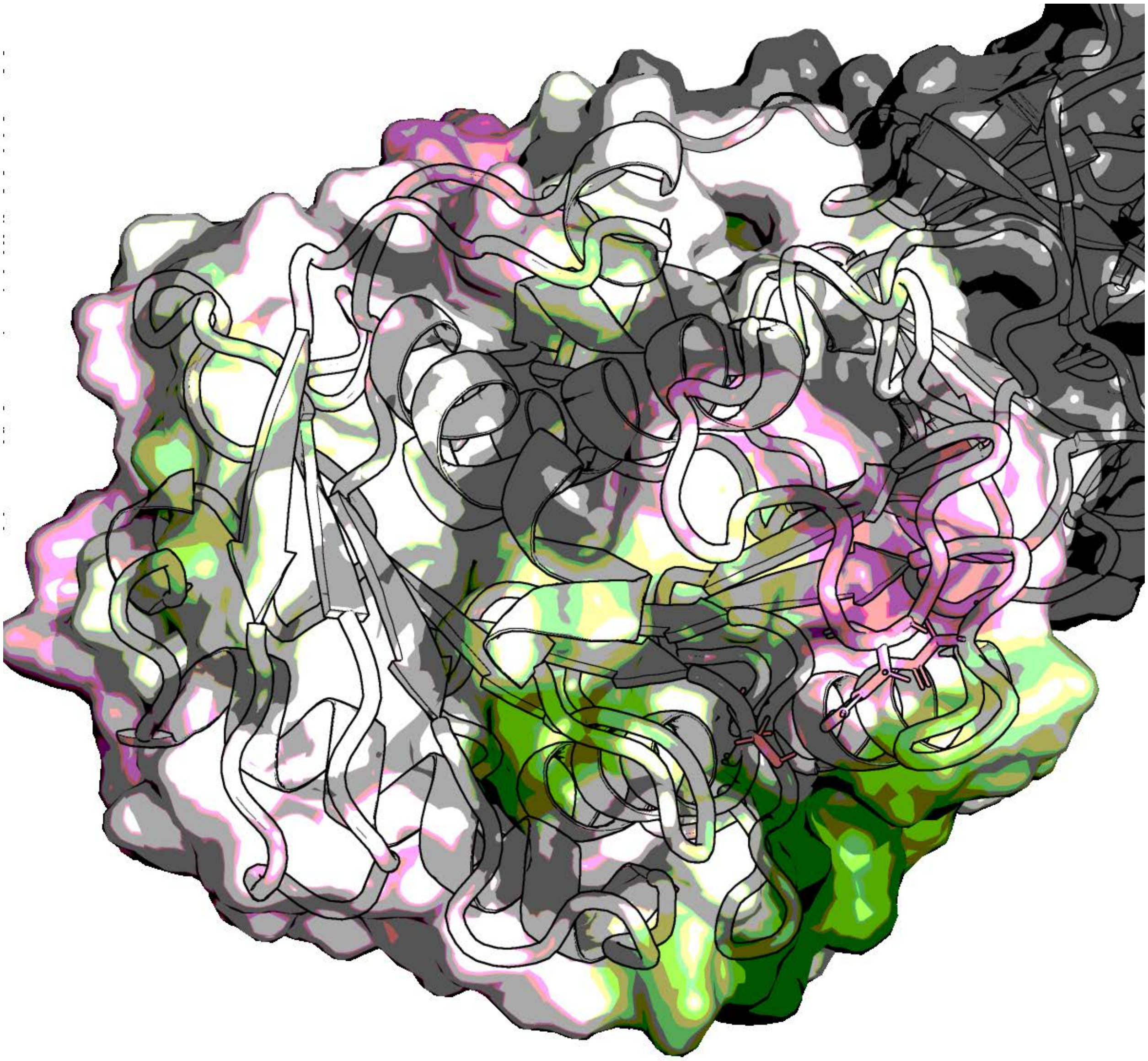

TEP010_highlights.png

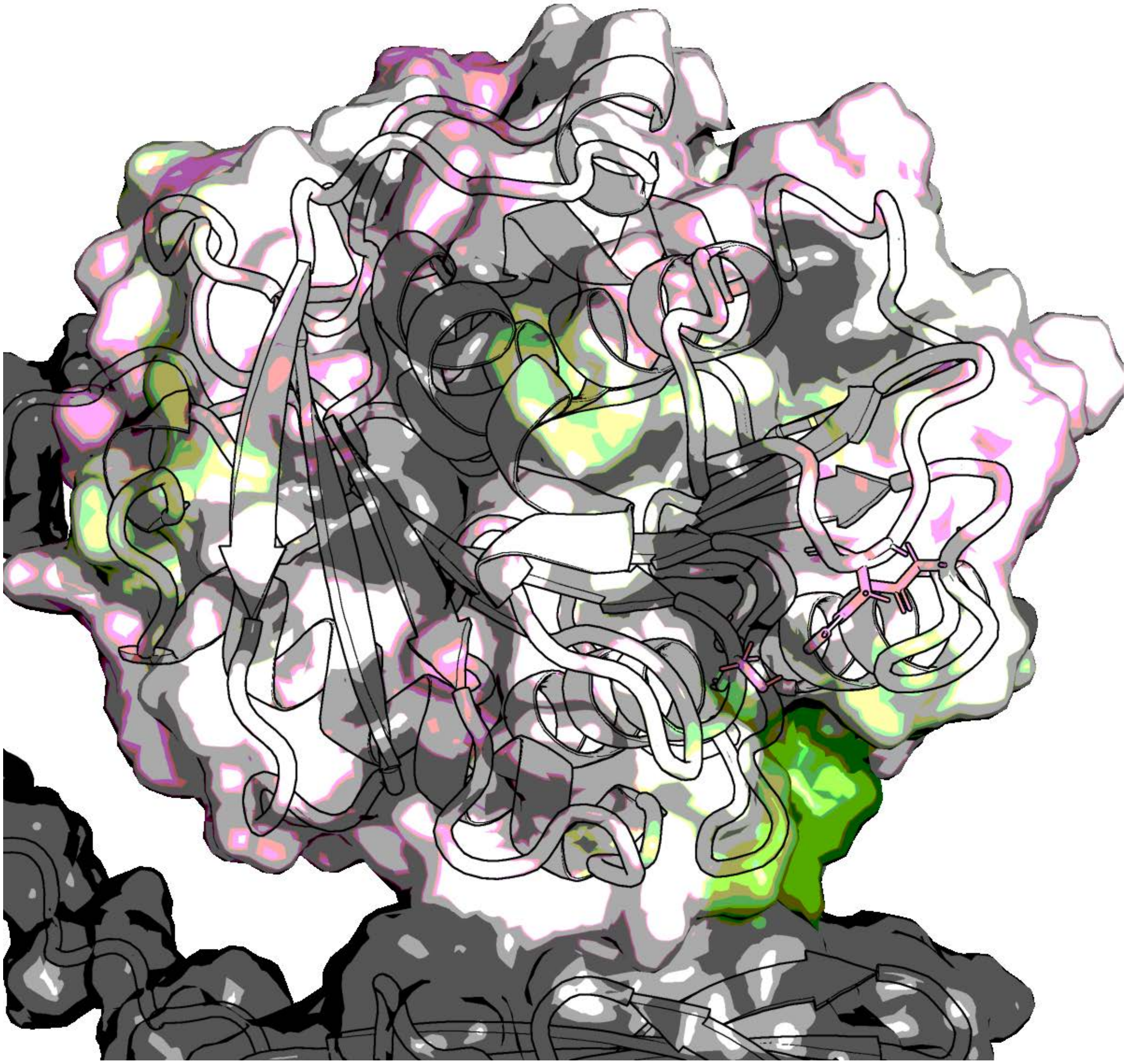

TEP013_highlights.png

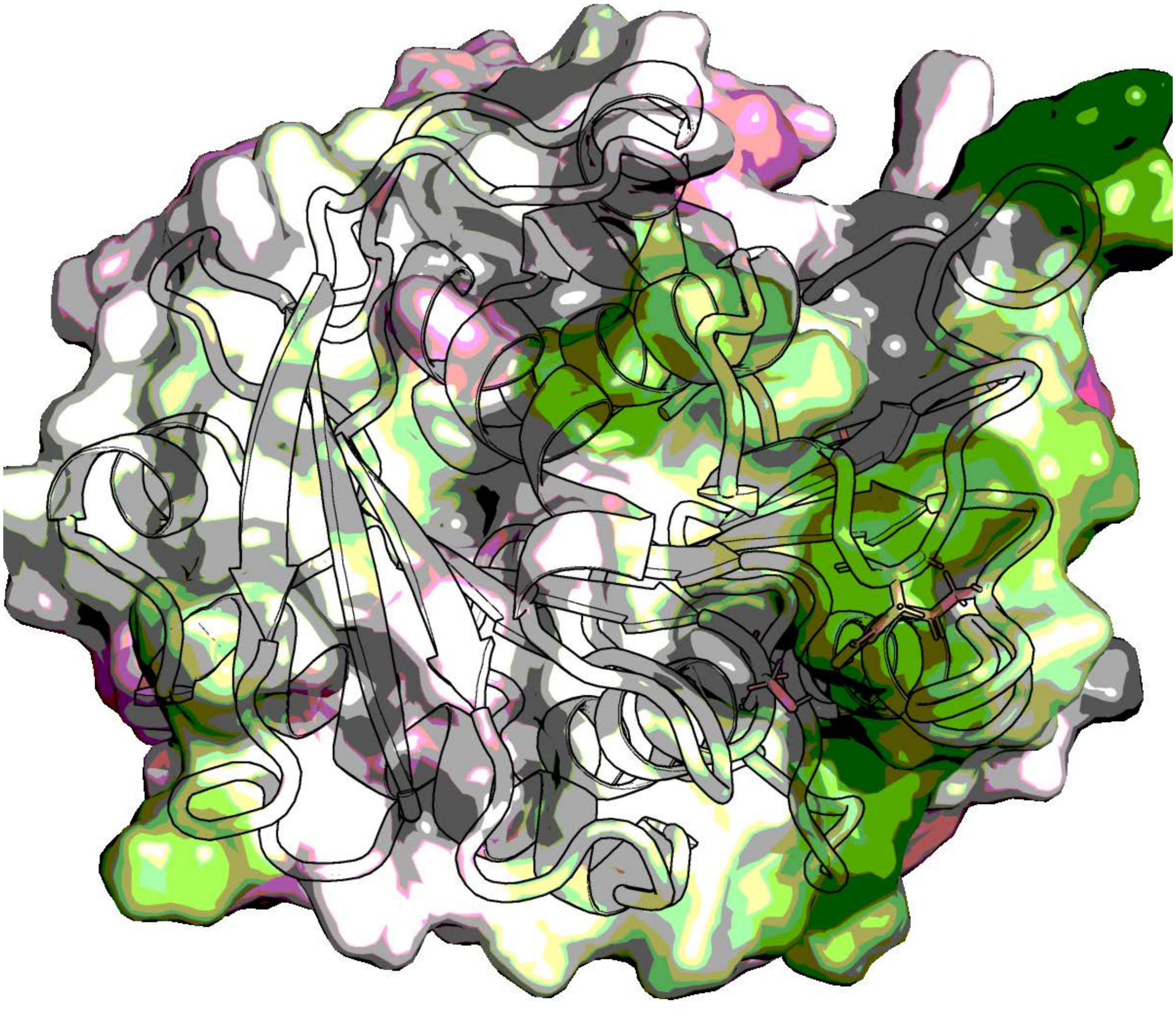

TEP014_highlights.png

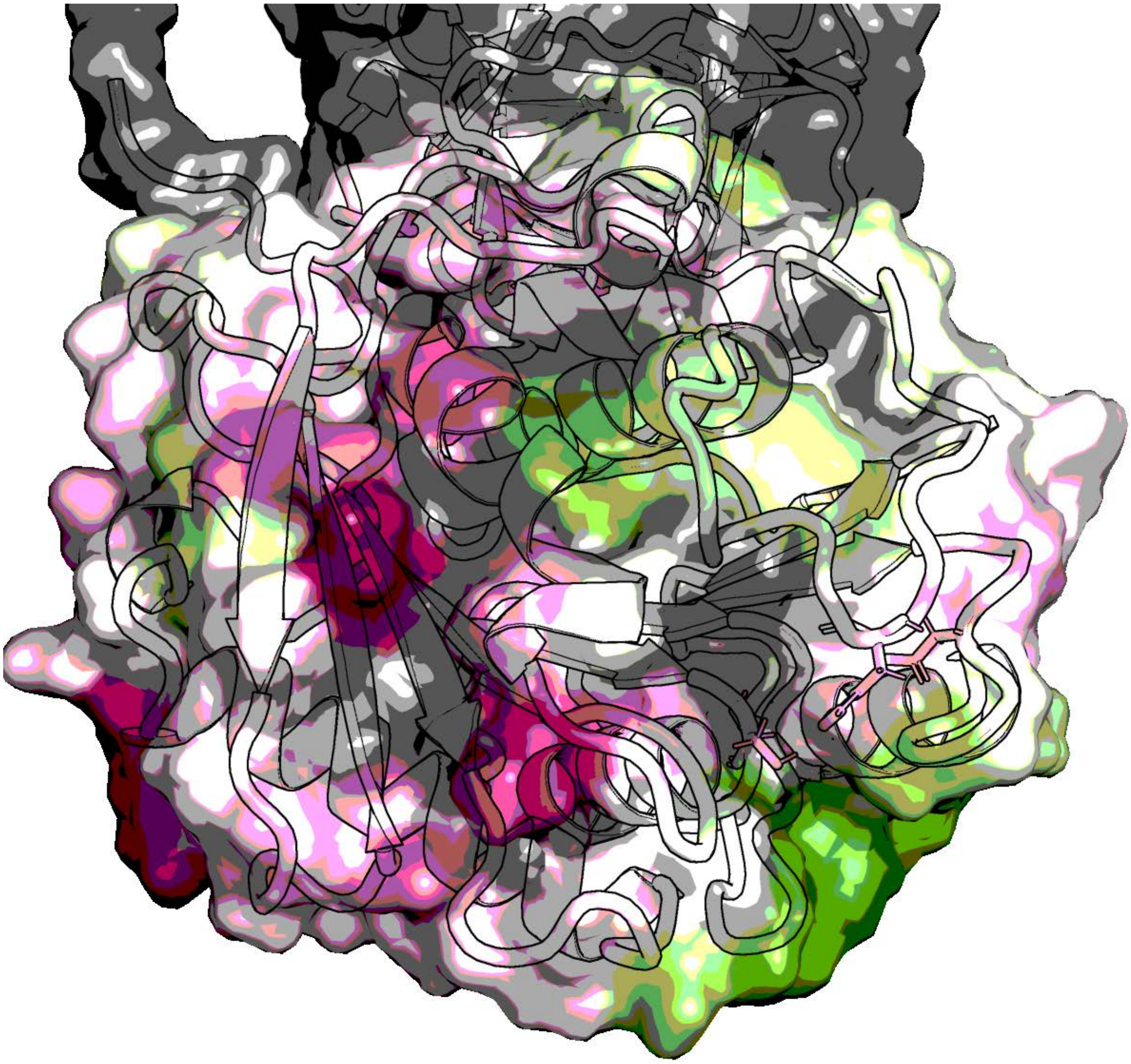

TEP024_highlights.png

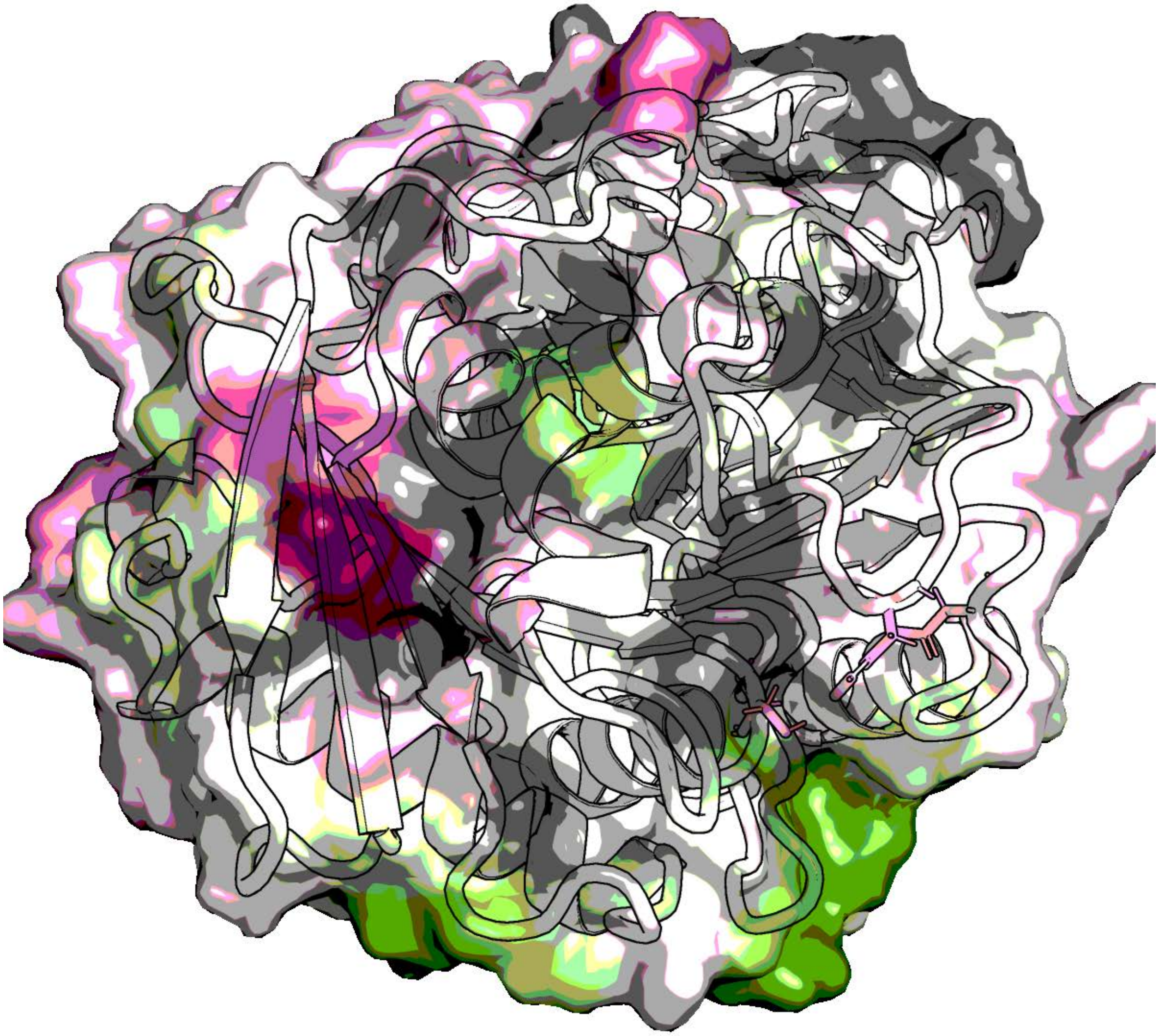

TEP029_highlights.png

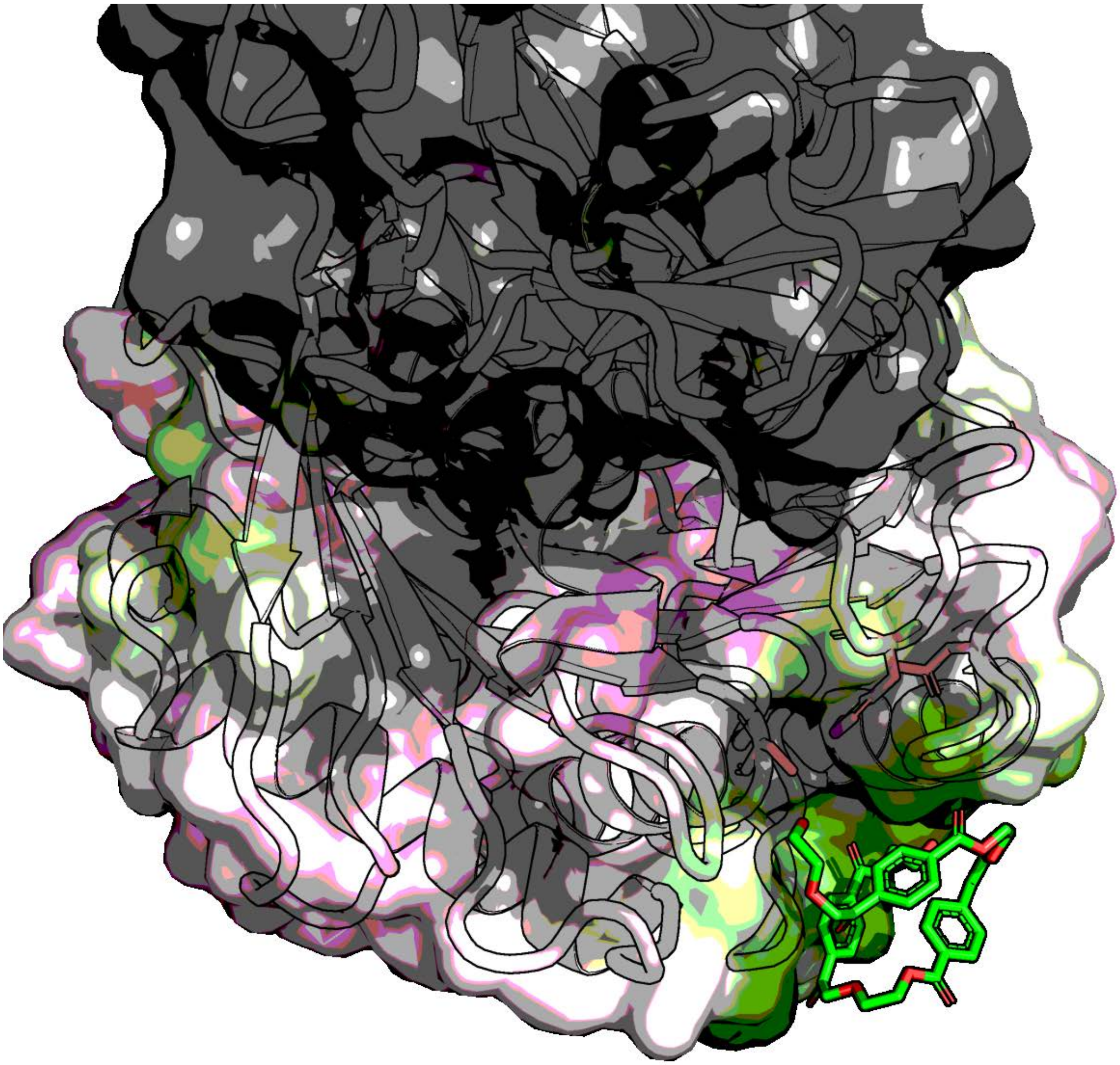

TEP054_highlights.png

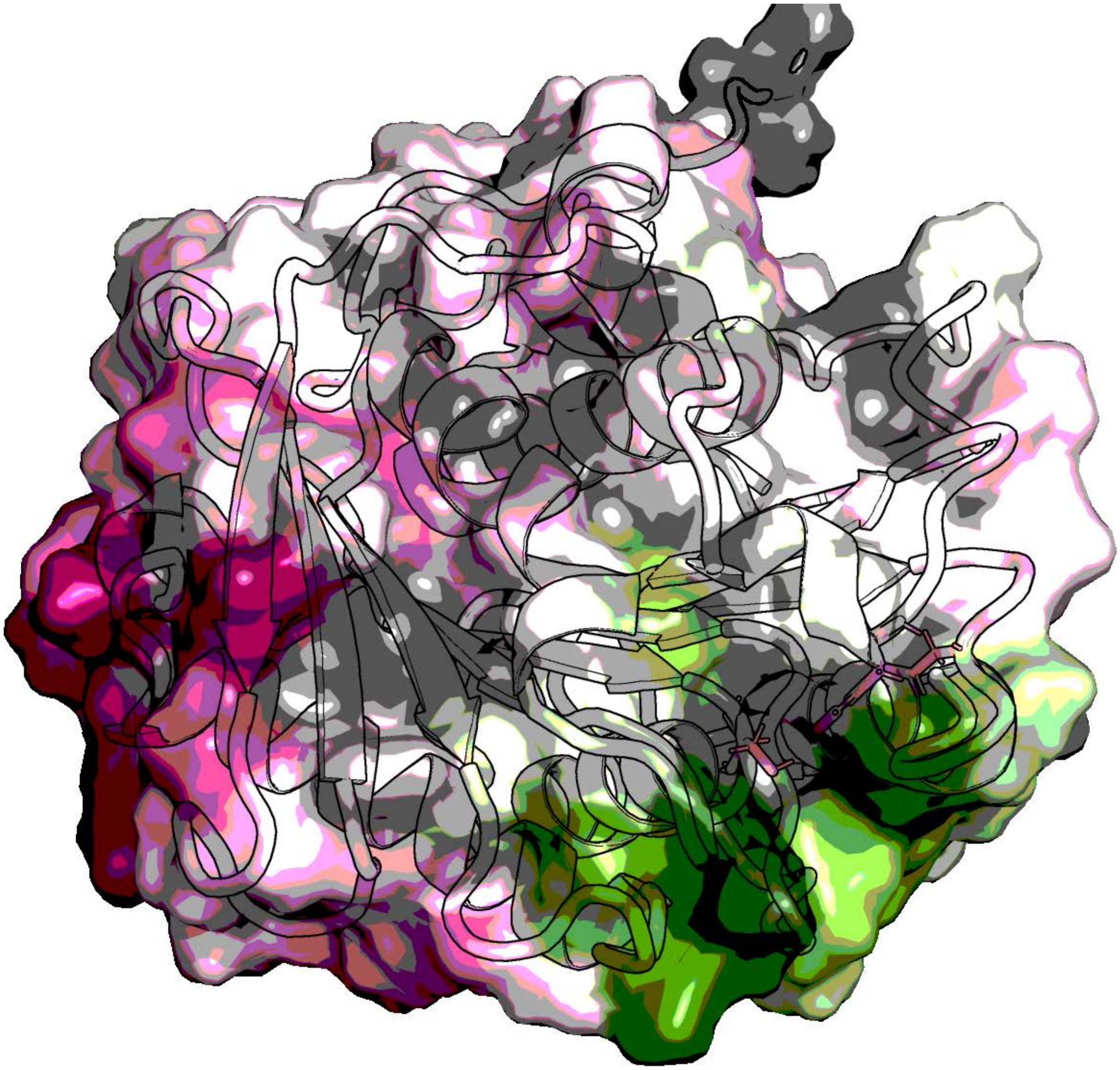

TEP067_highlights.png

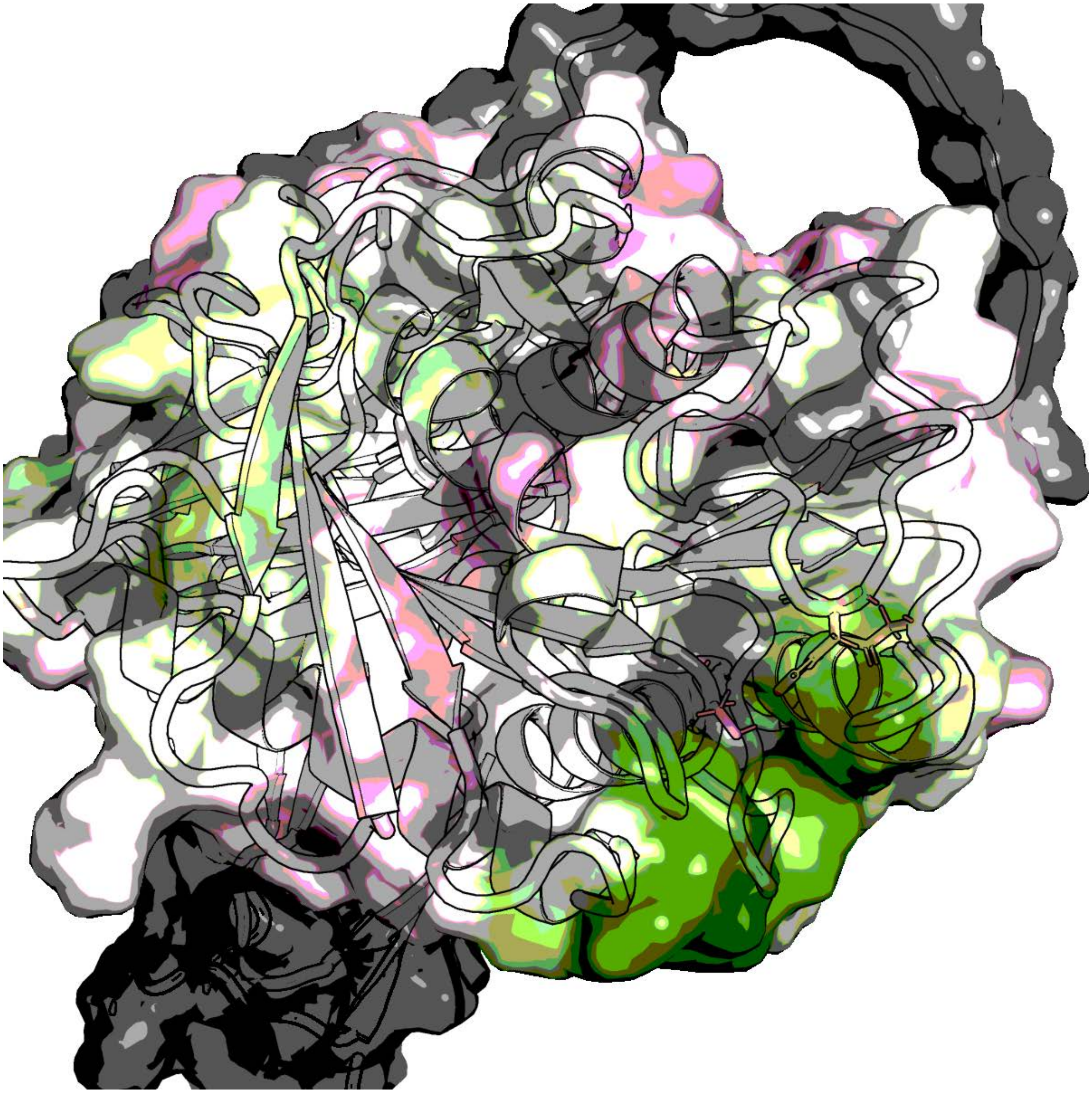

TEP080_highlights.png

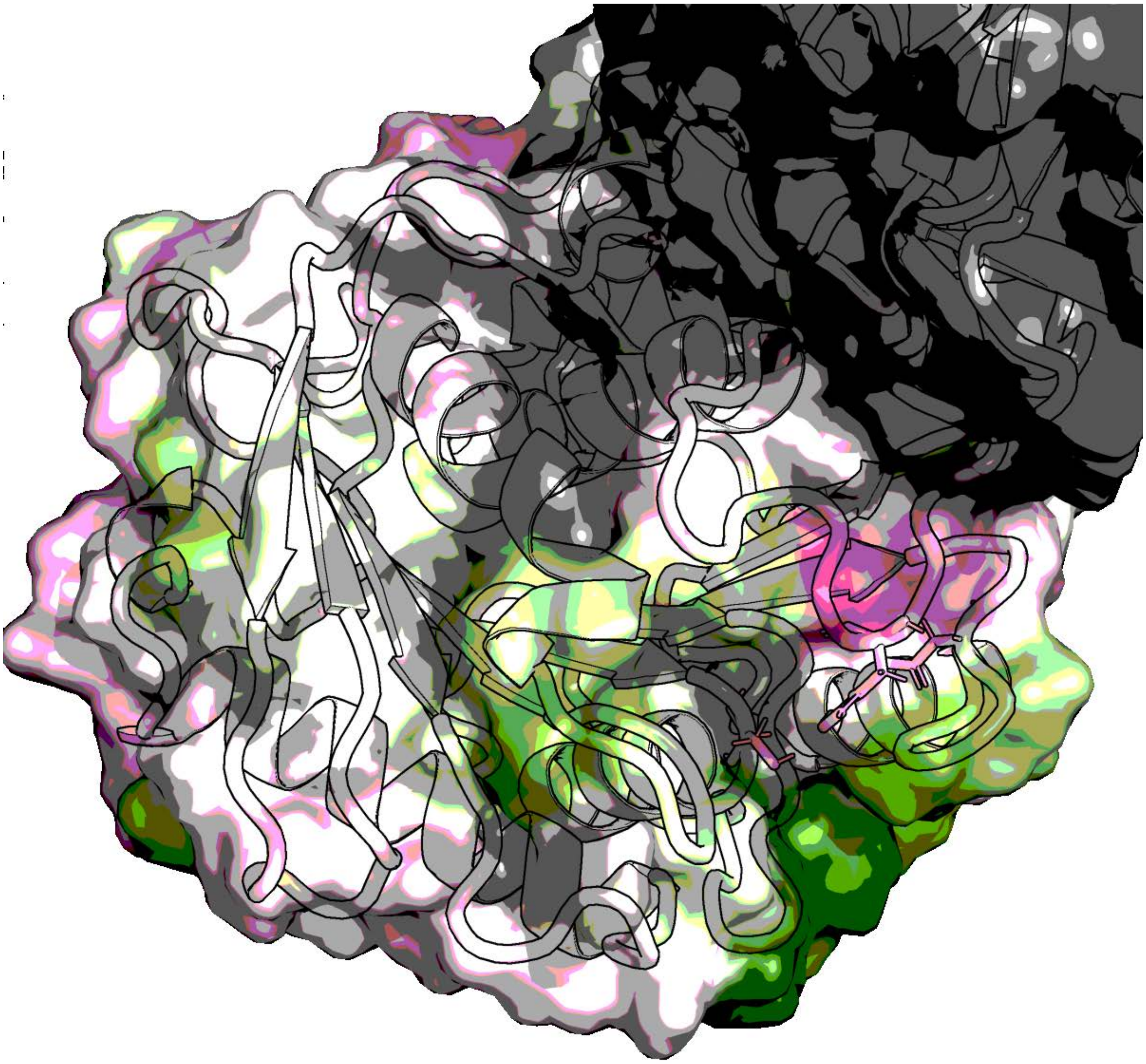

TEP082_highlights.png

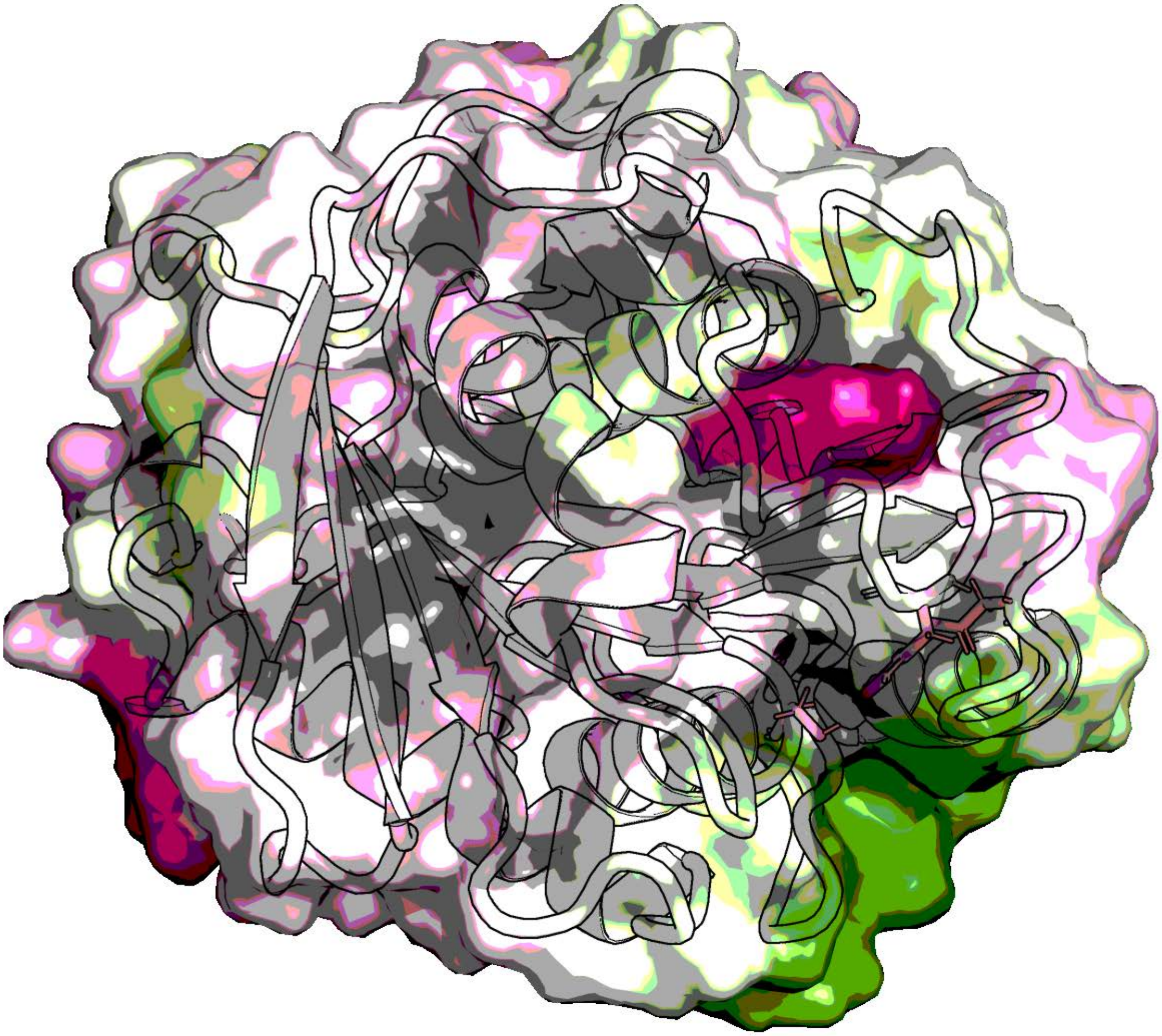

TEP083_highlights.png

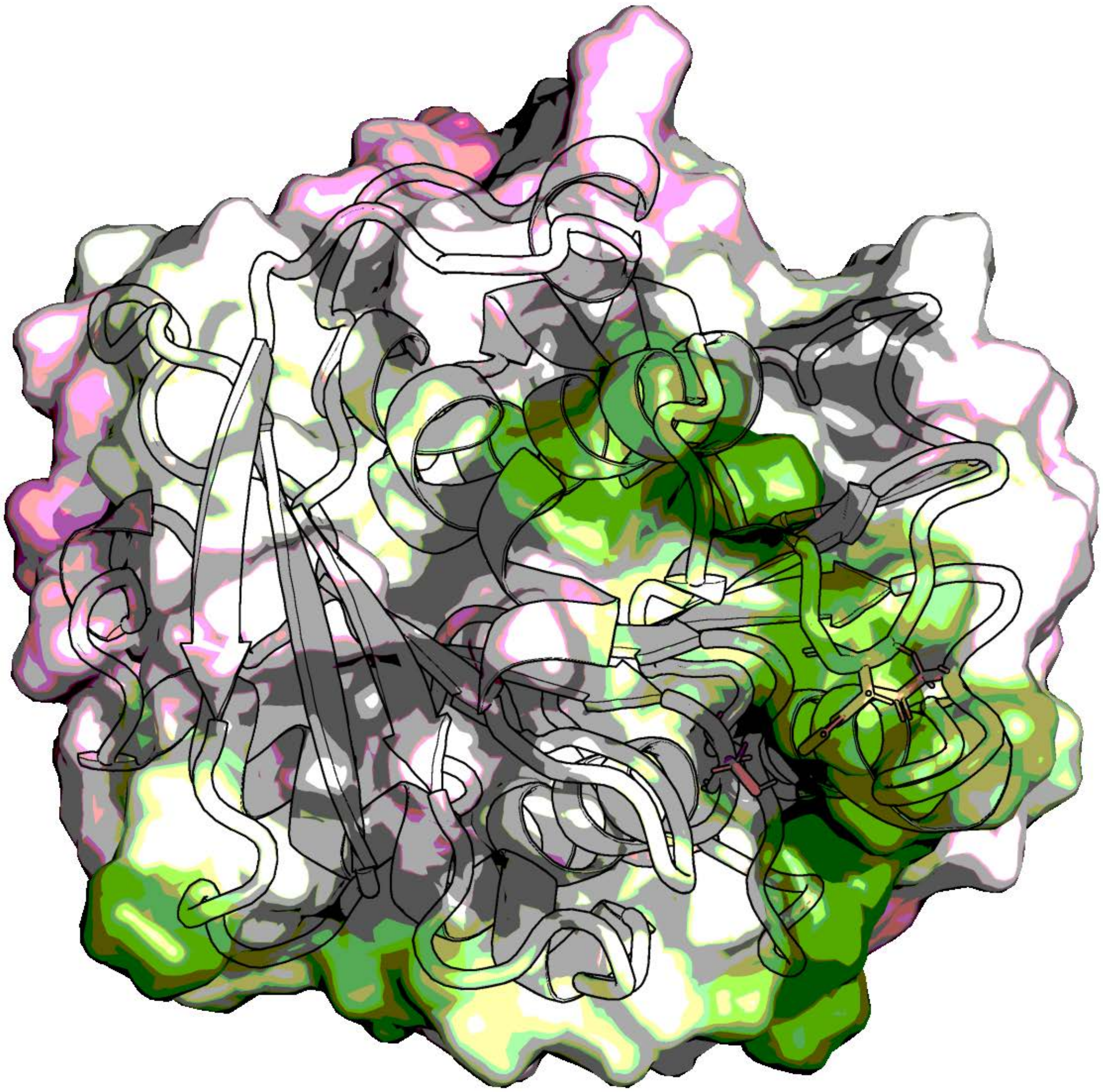

TEP091_highlights.png

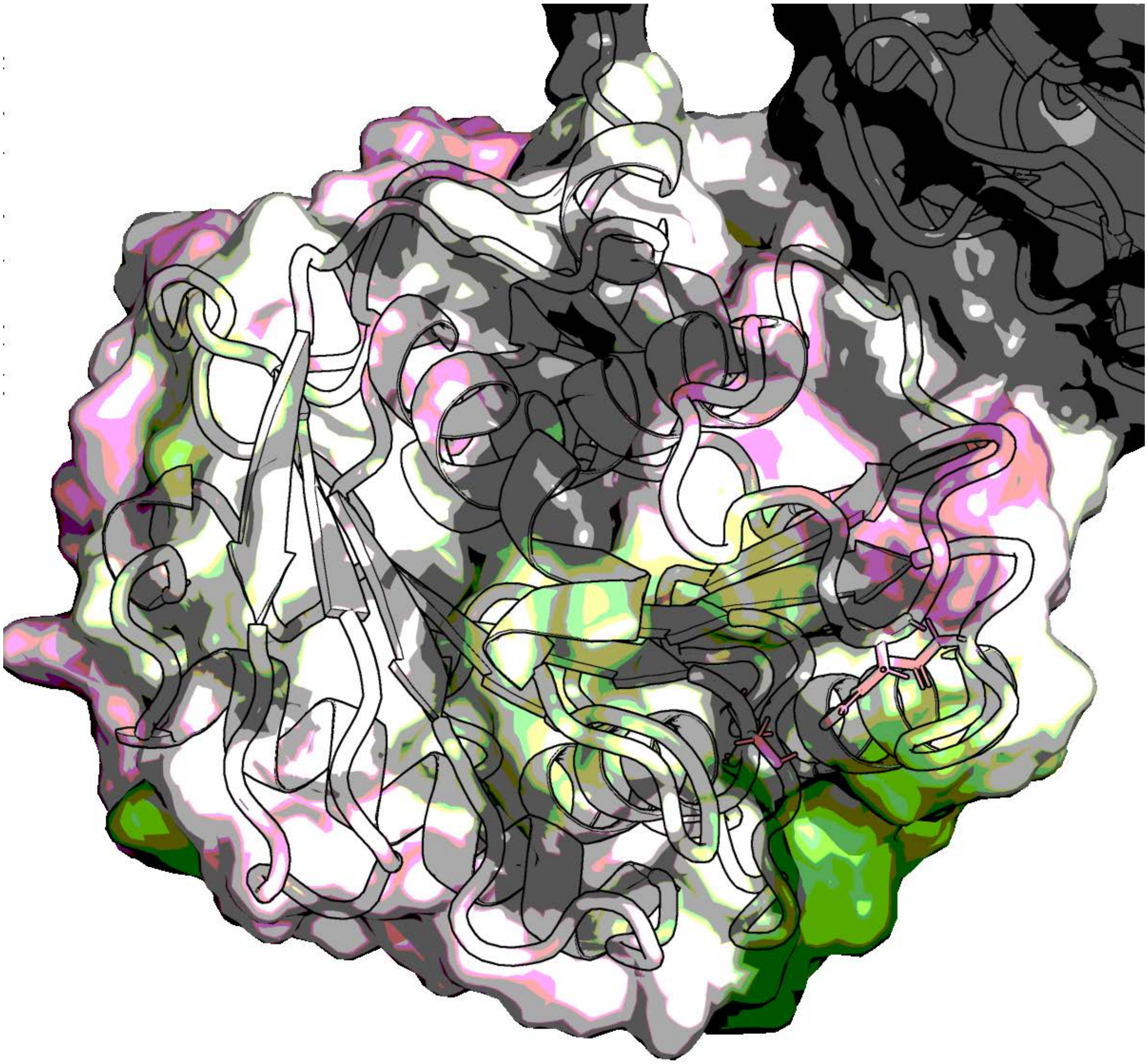

TEP092_highlights.png

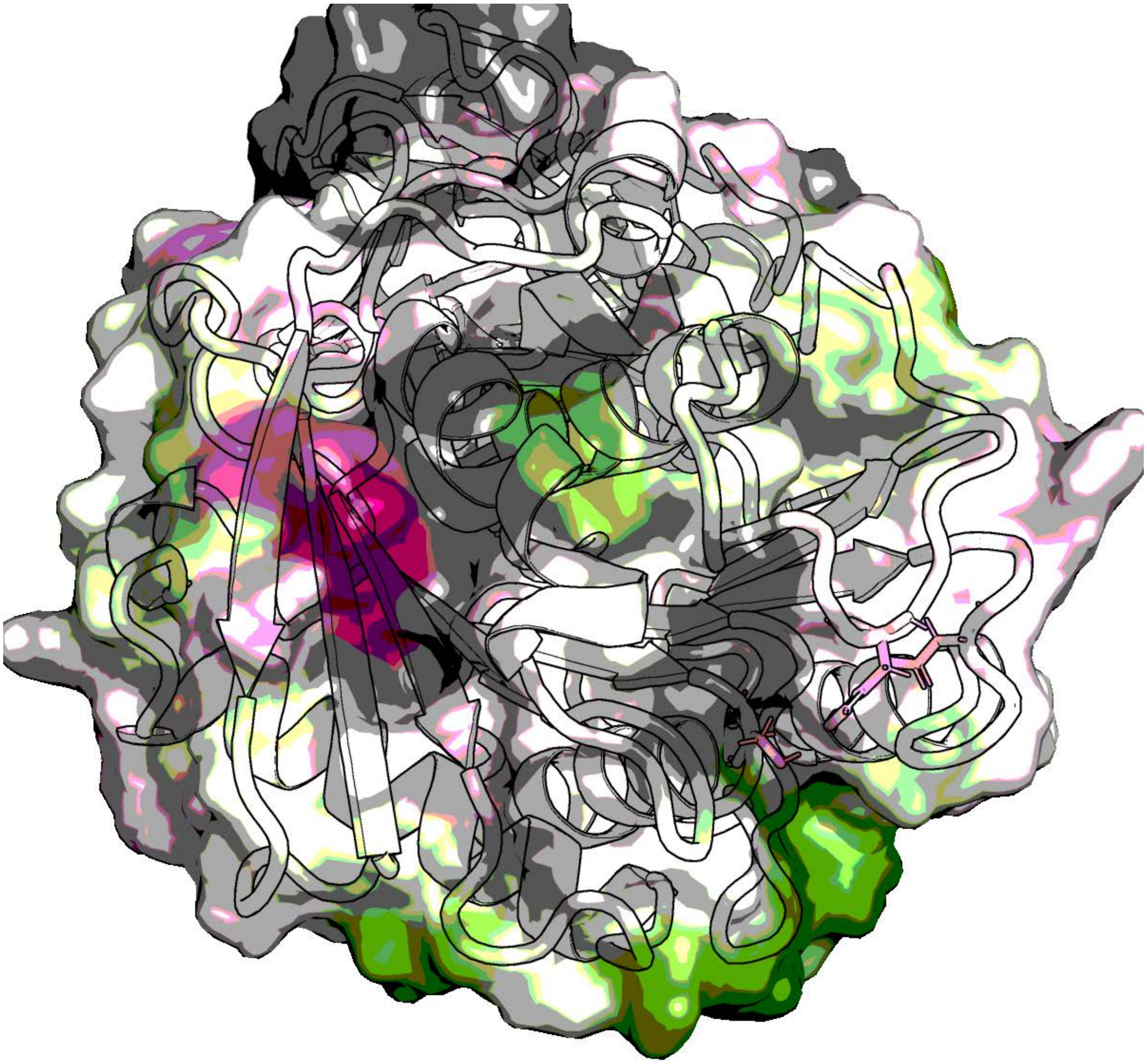

TEP093_highlights.png

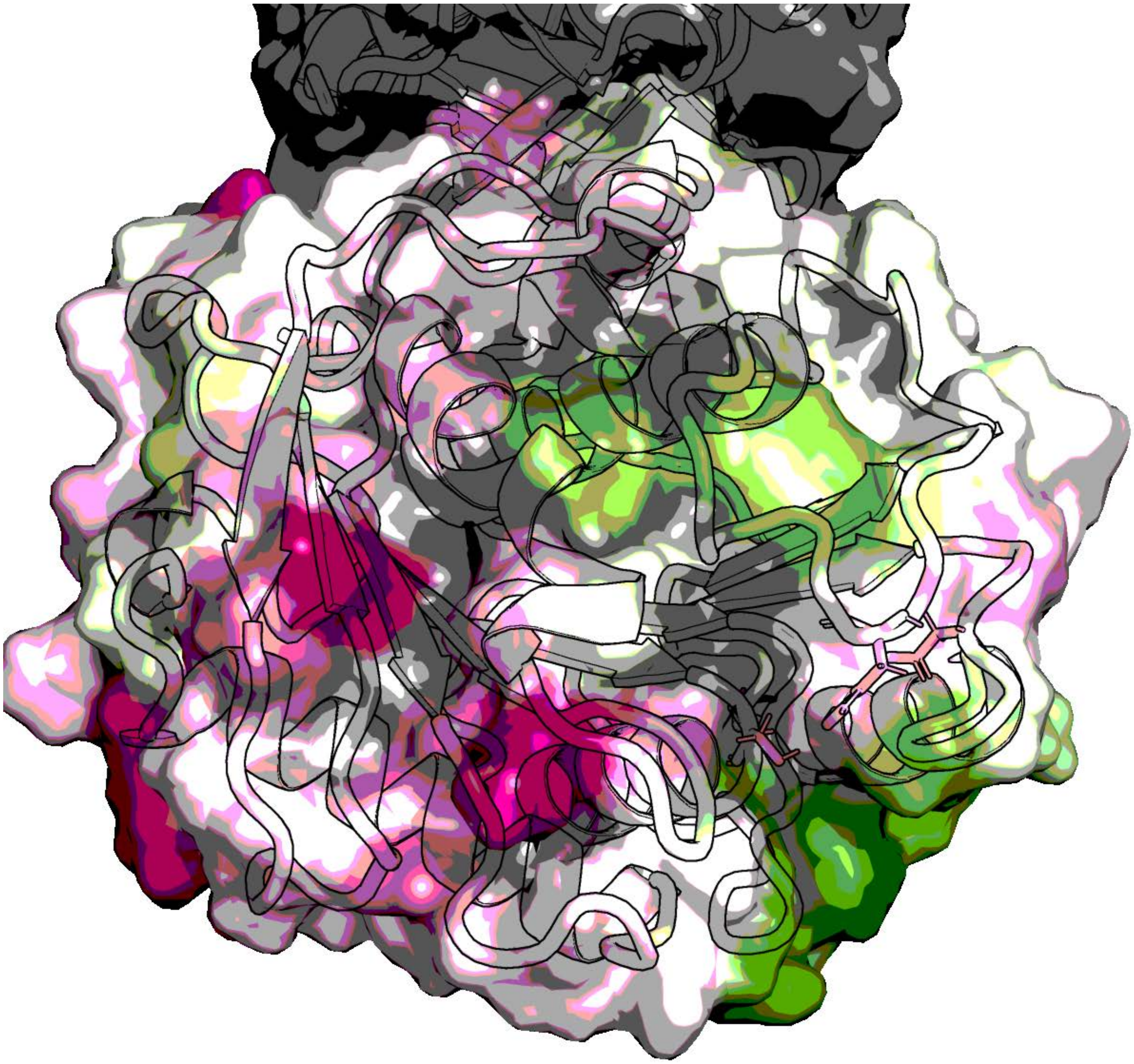

TEP097_highlights.png

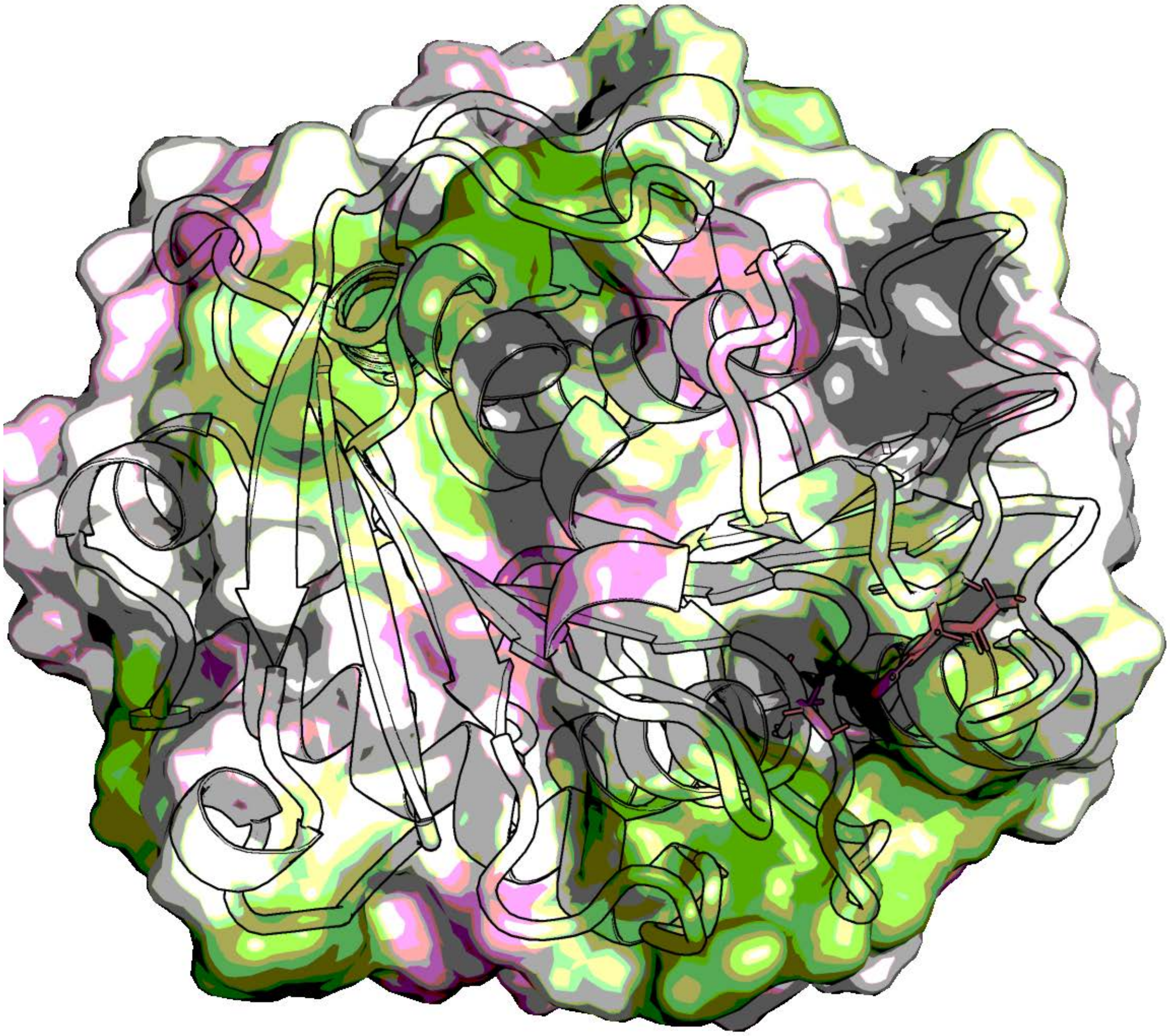

TEP101_highlights.png

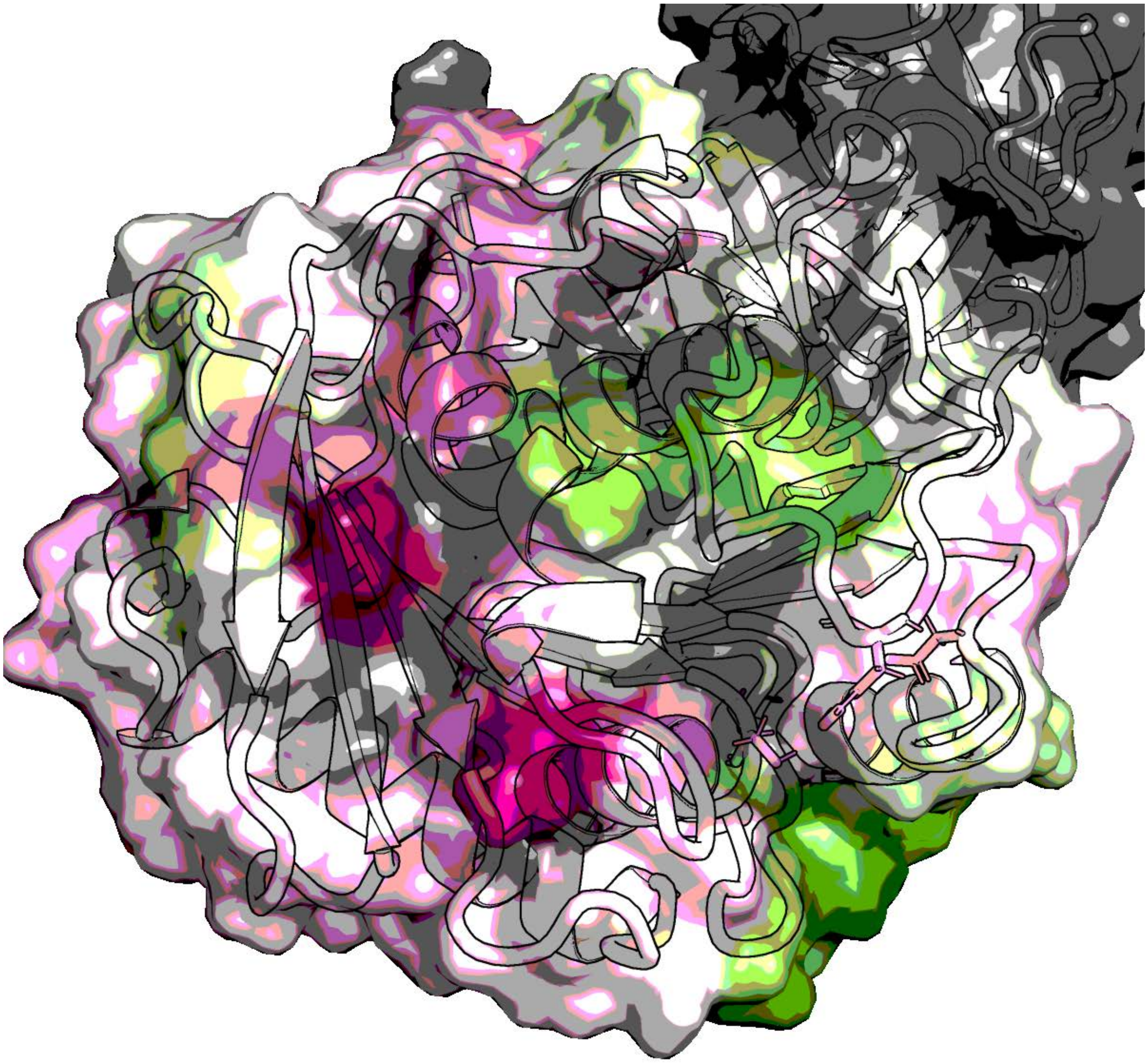

TEP102_highlights.png

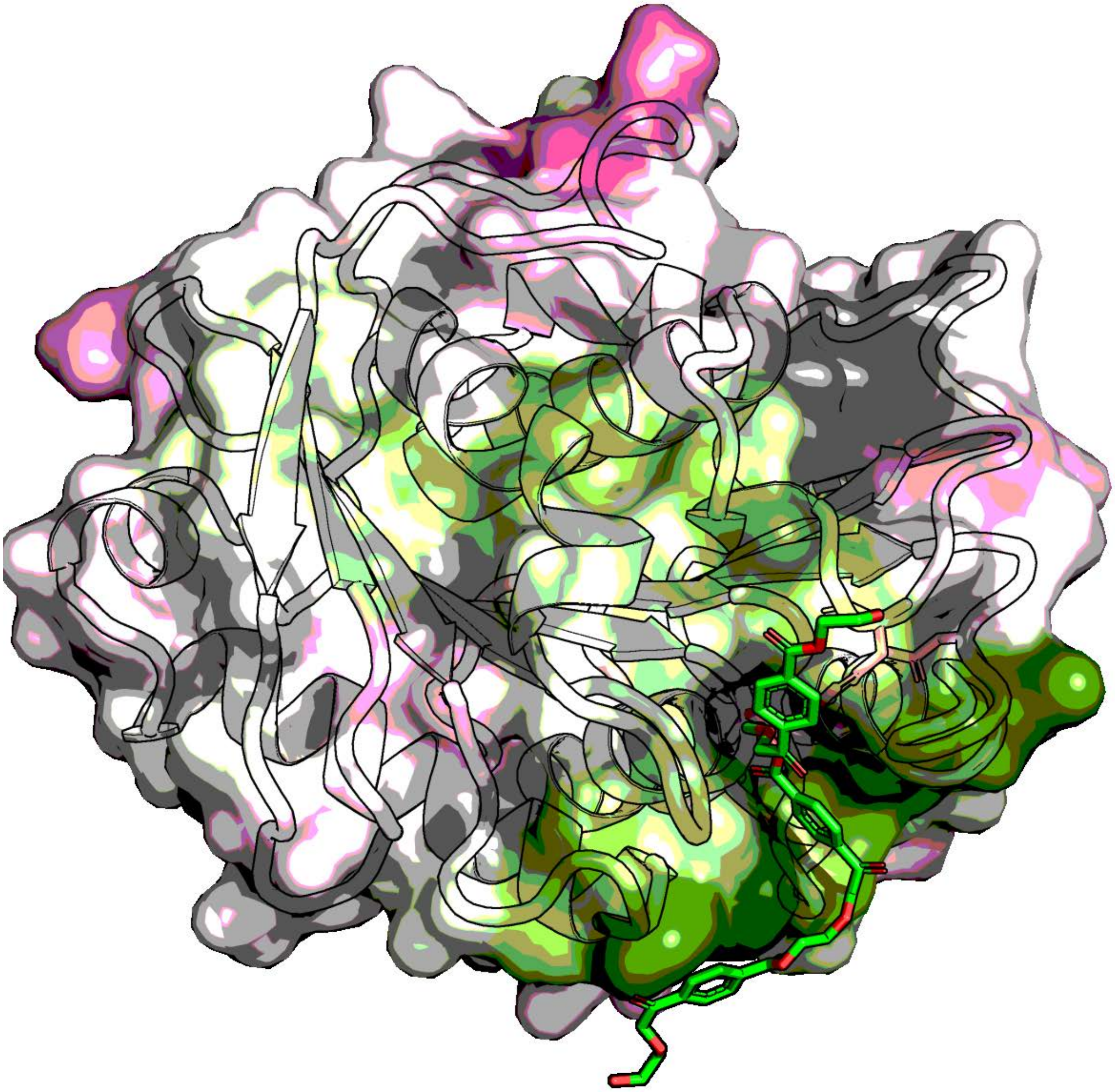

TEP103_highlights.png

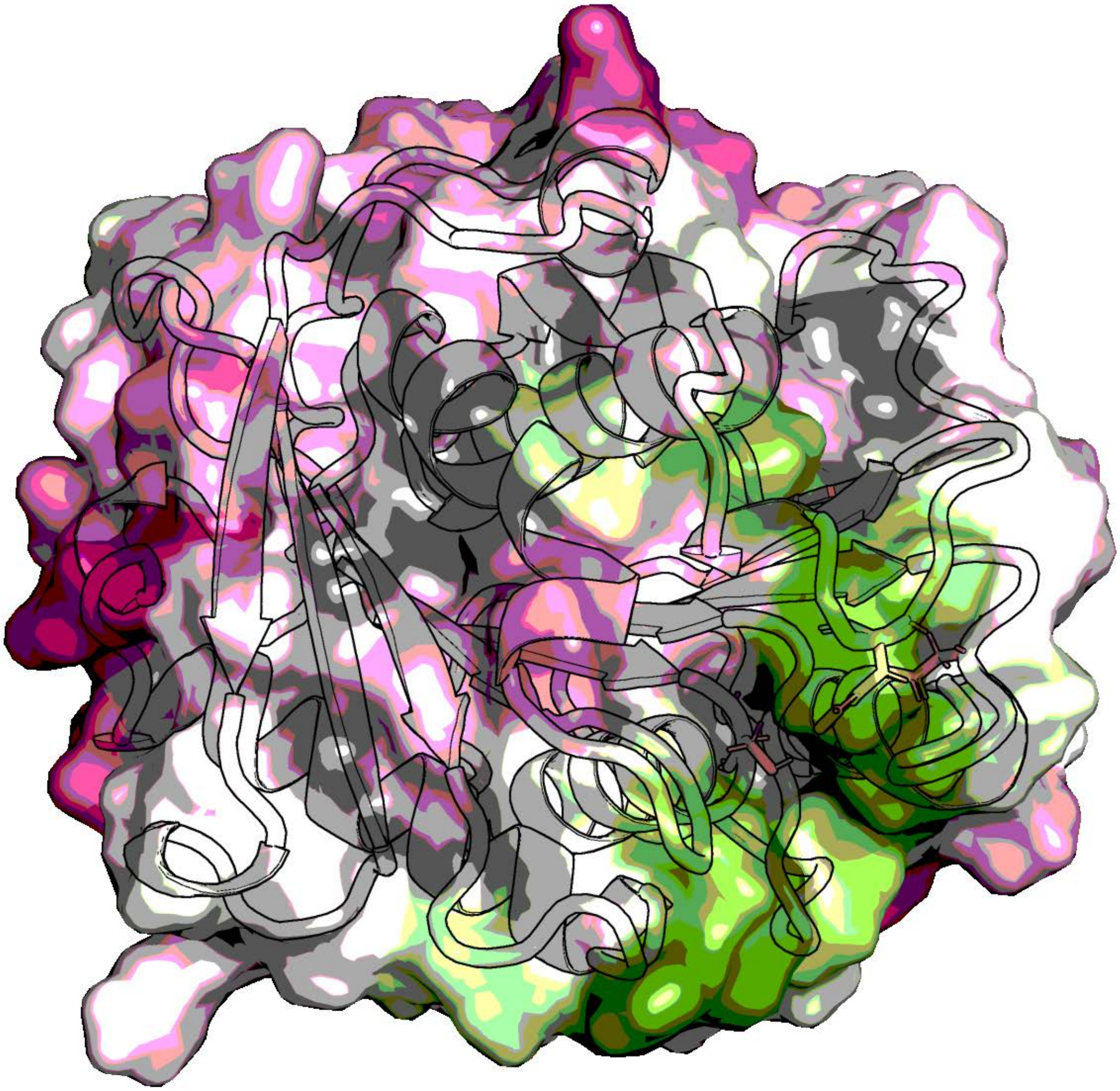

TEP105_highlights.png

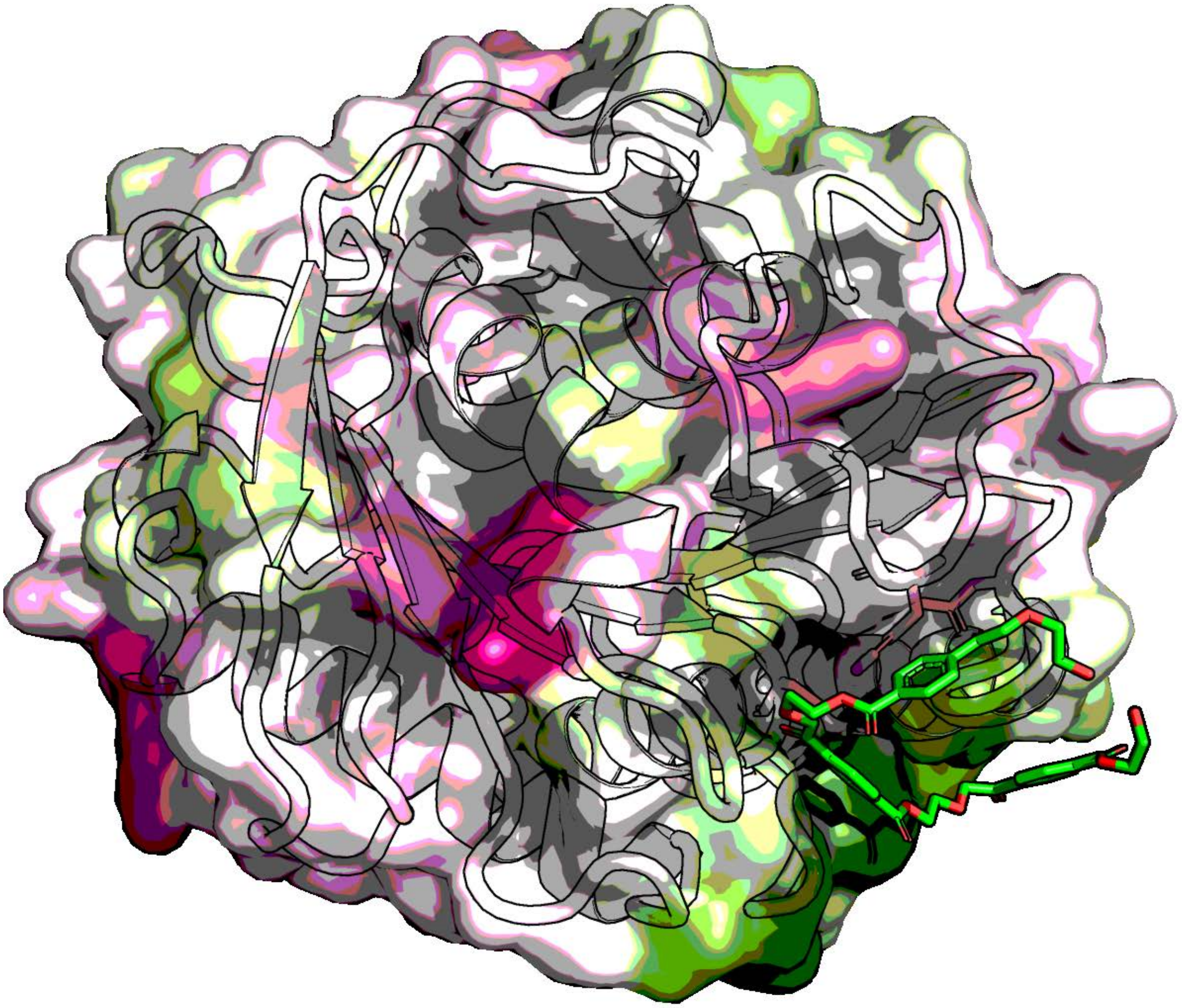

TEP109_highlights.png

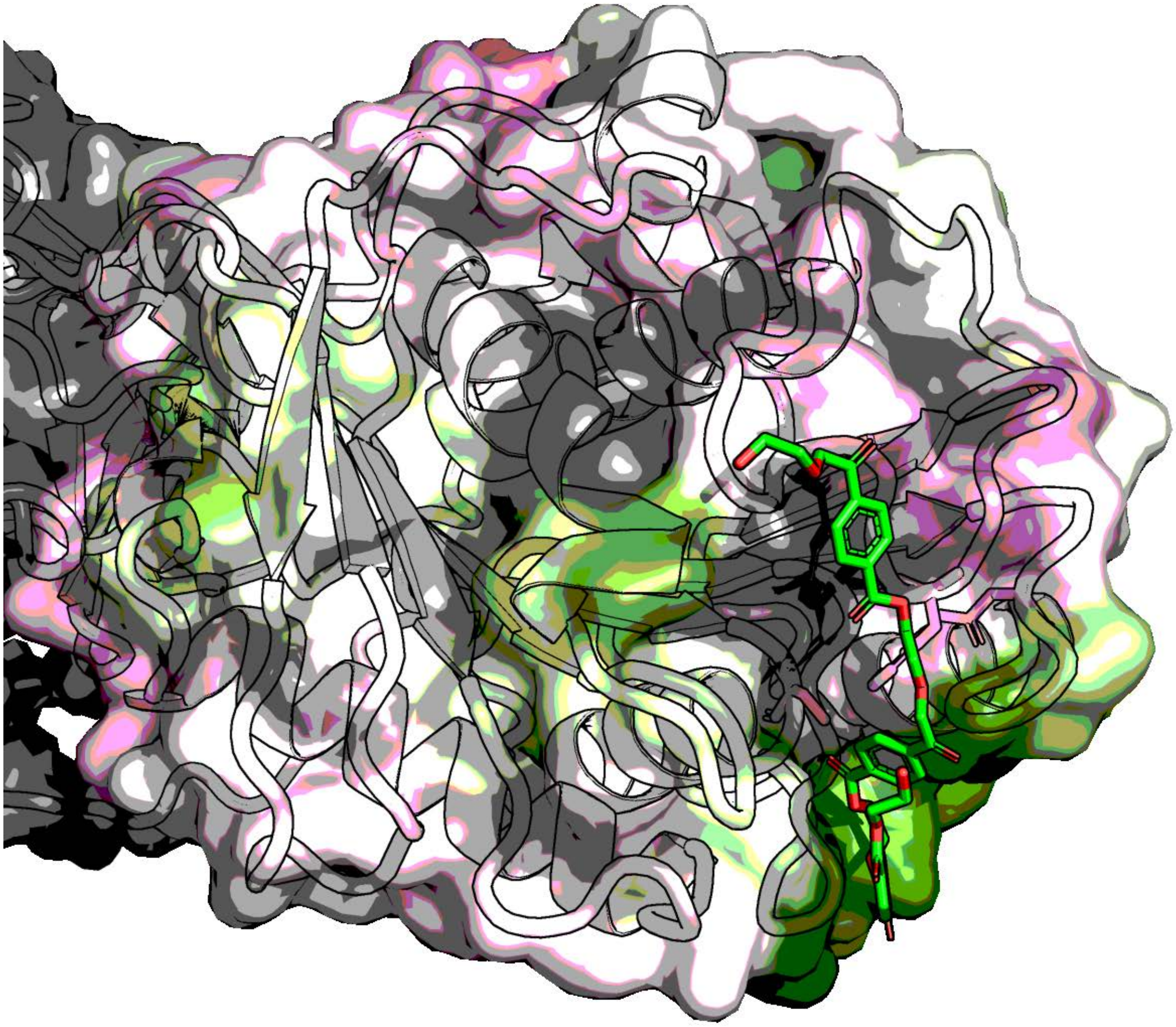

TEP111_highlights.png

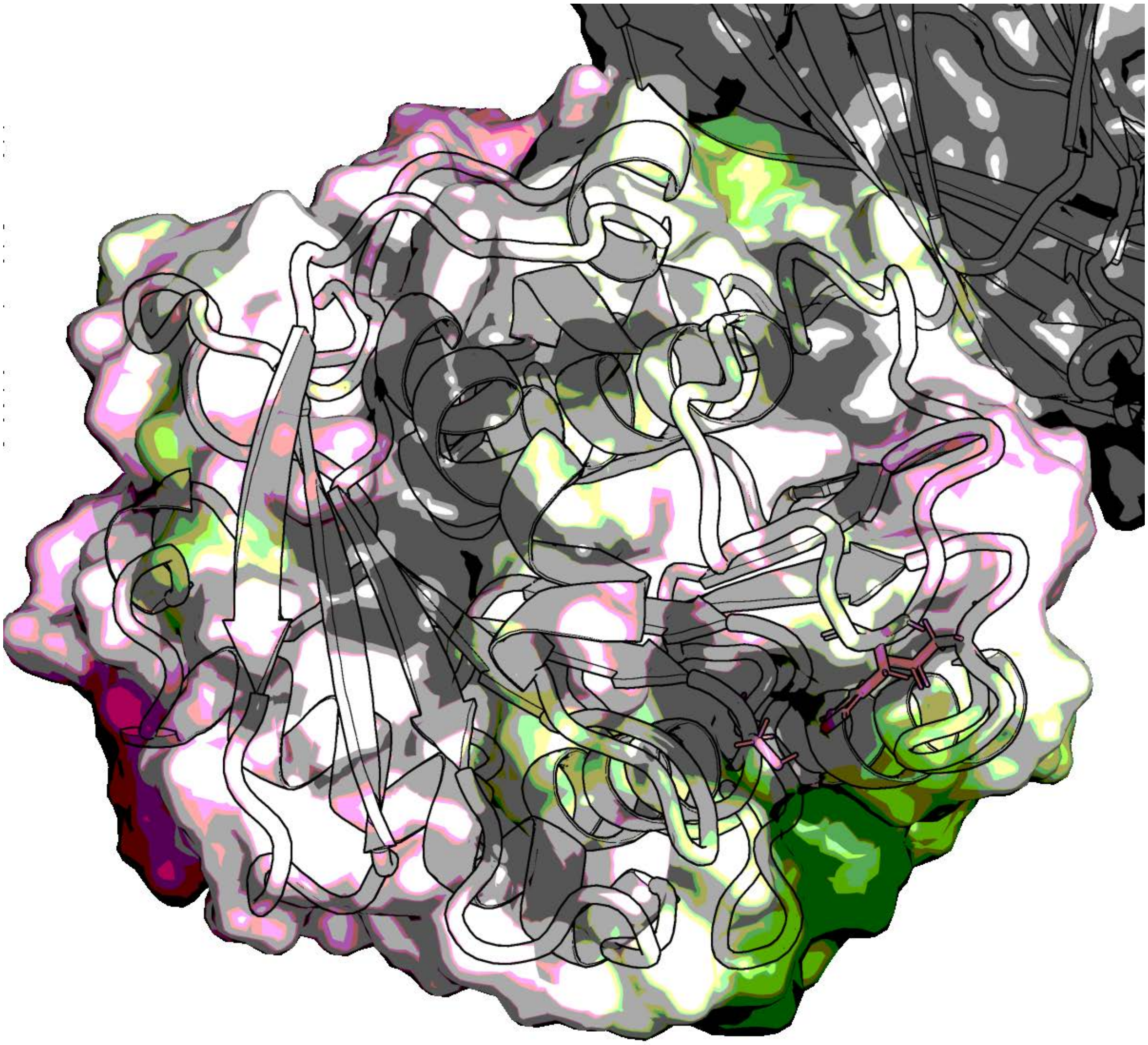

TEP114_highlights.png

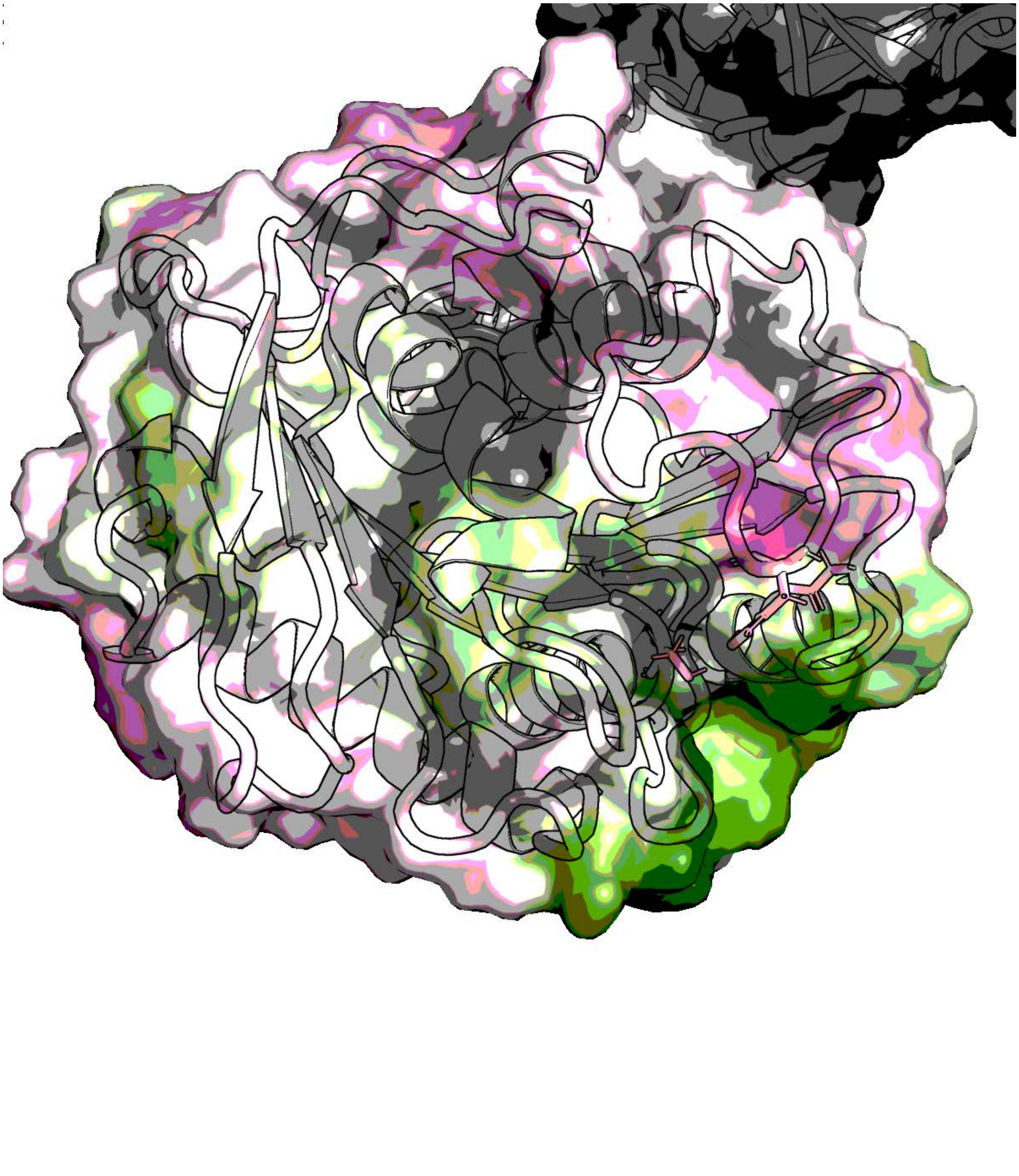

TEP115_highlights.png

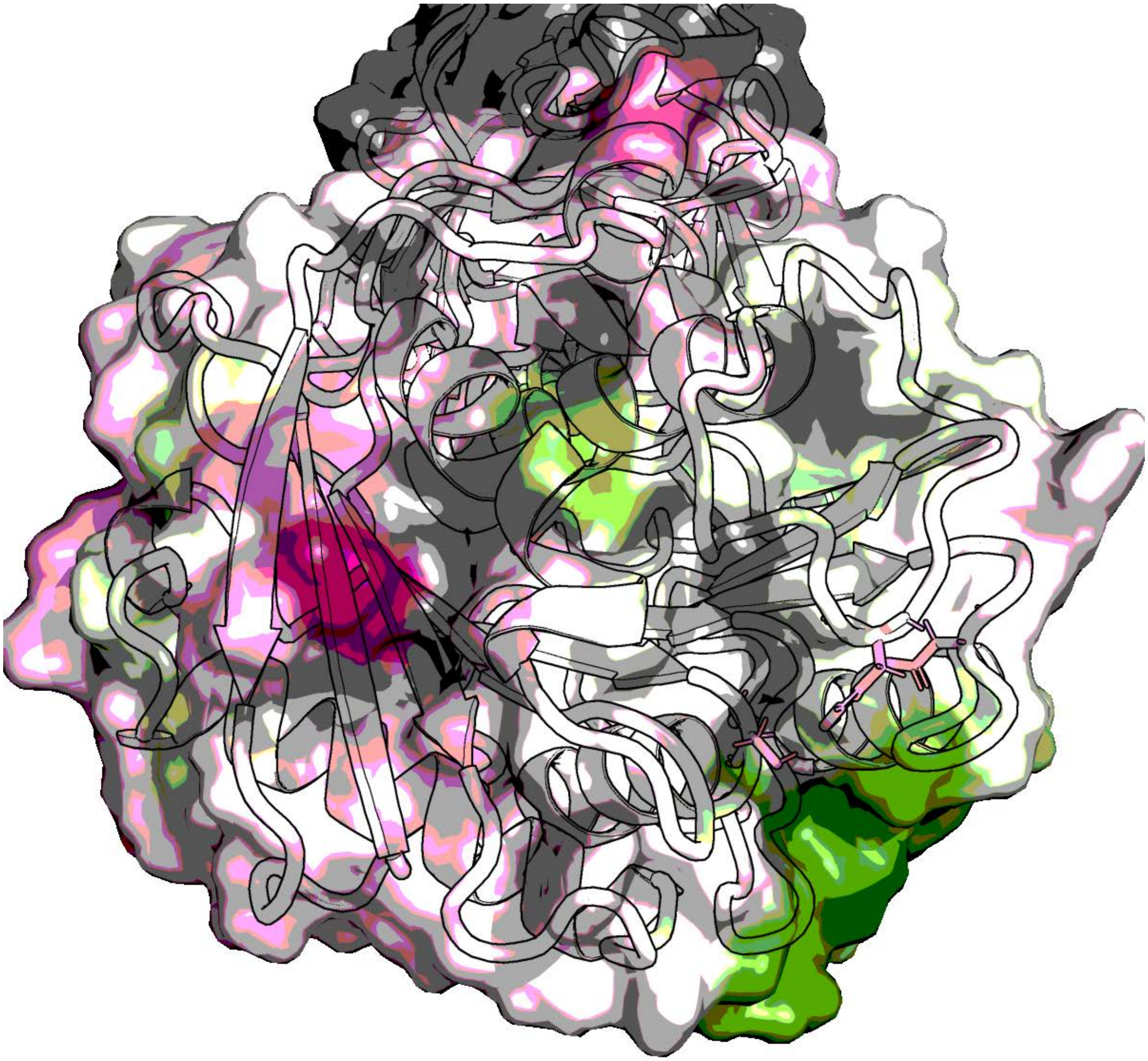

TEP131_highlights.png

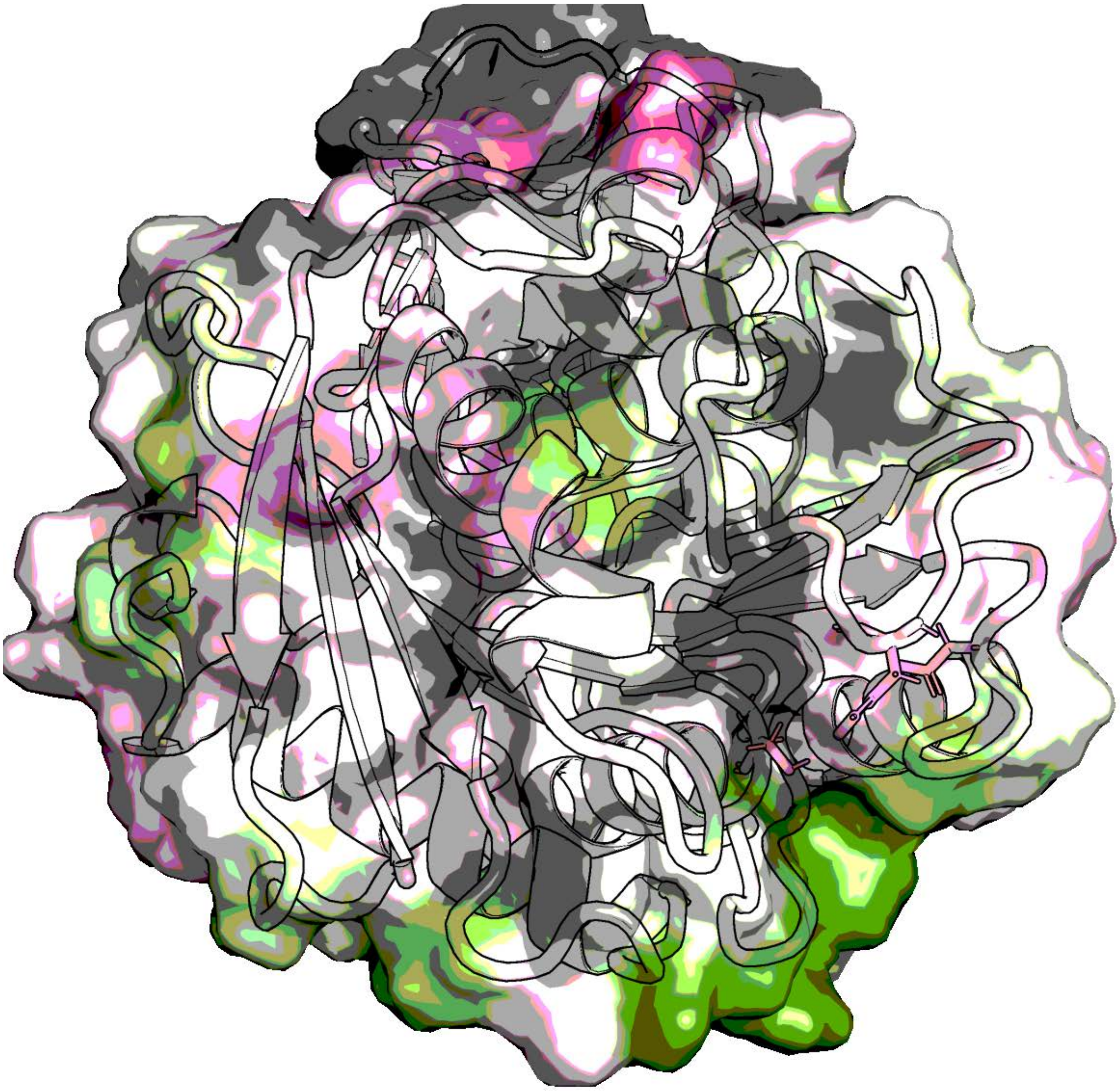

TEP163_highlights.png

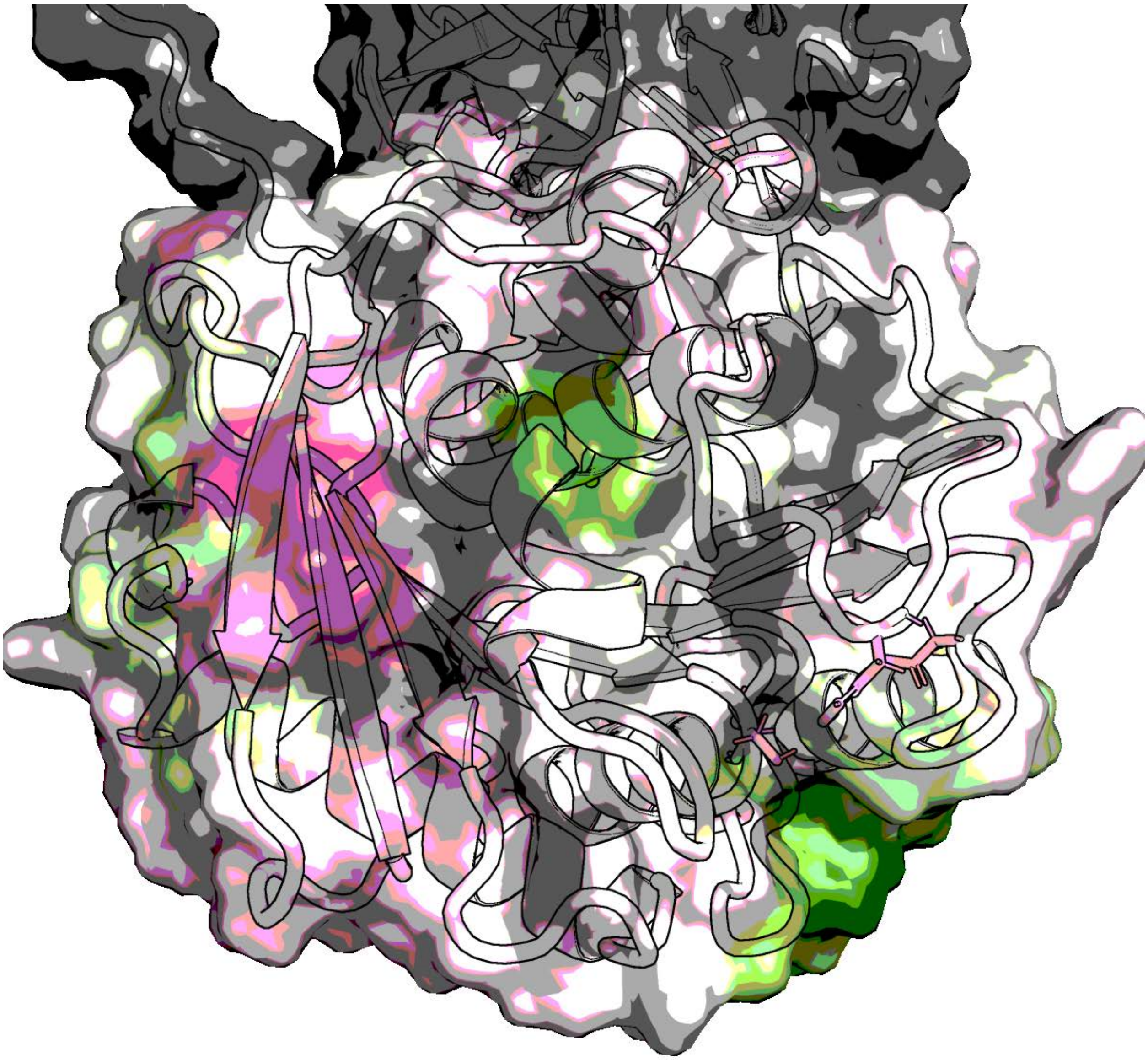

TEP164_highlights.png

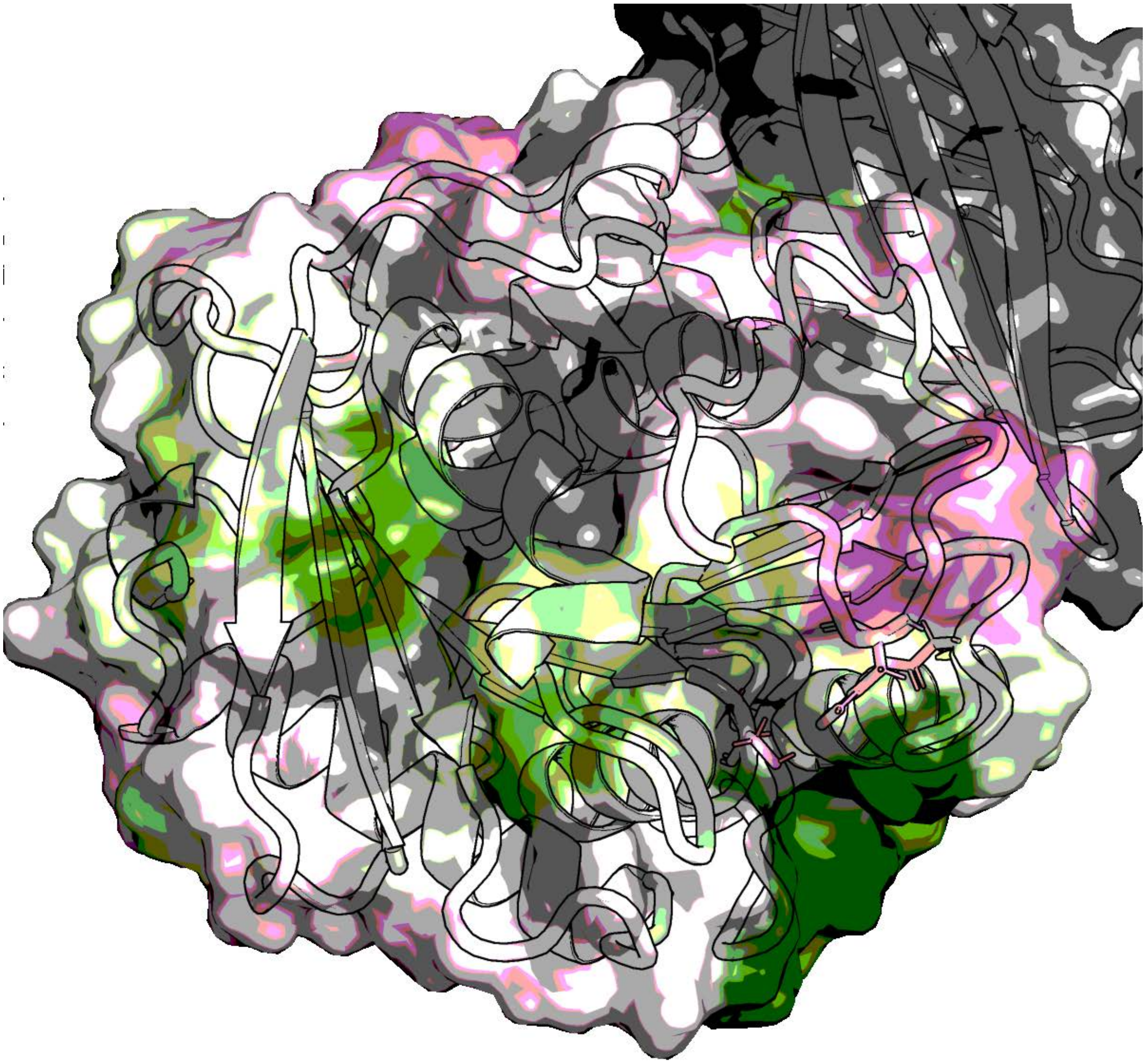

TEP165_highlights.png

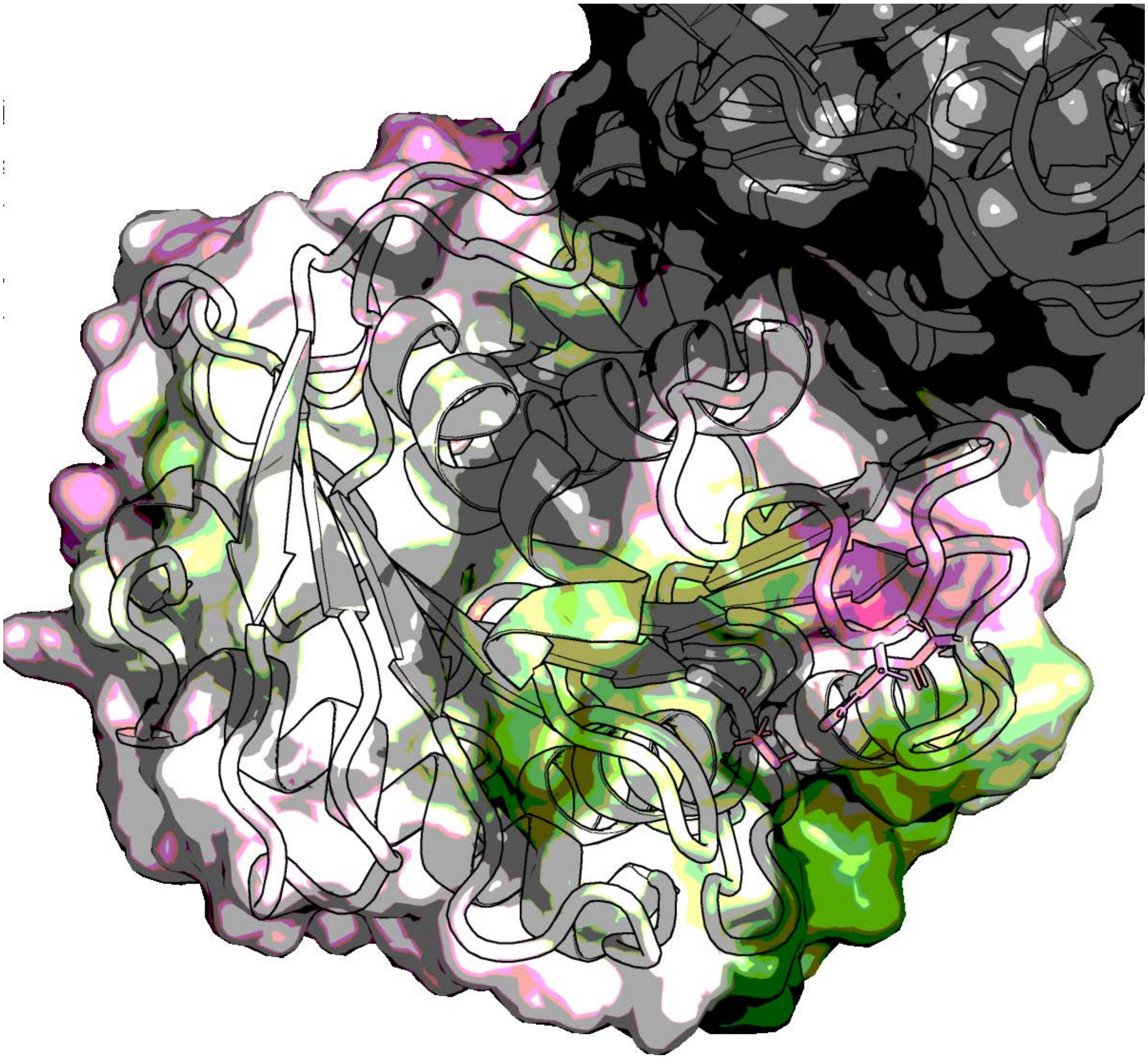

TEP168_highlights.png

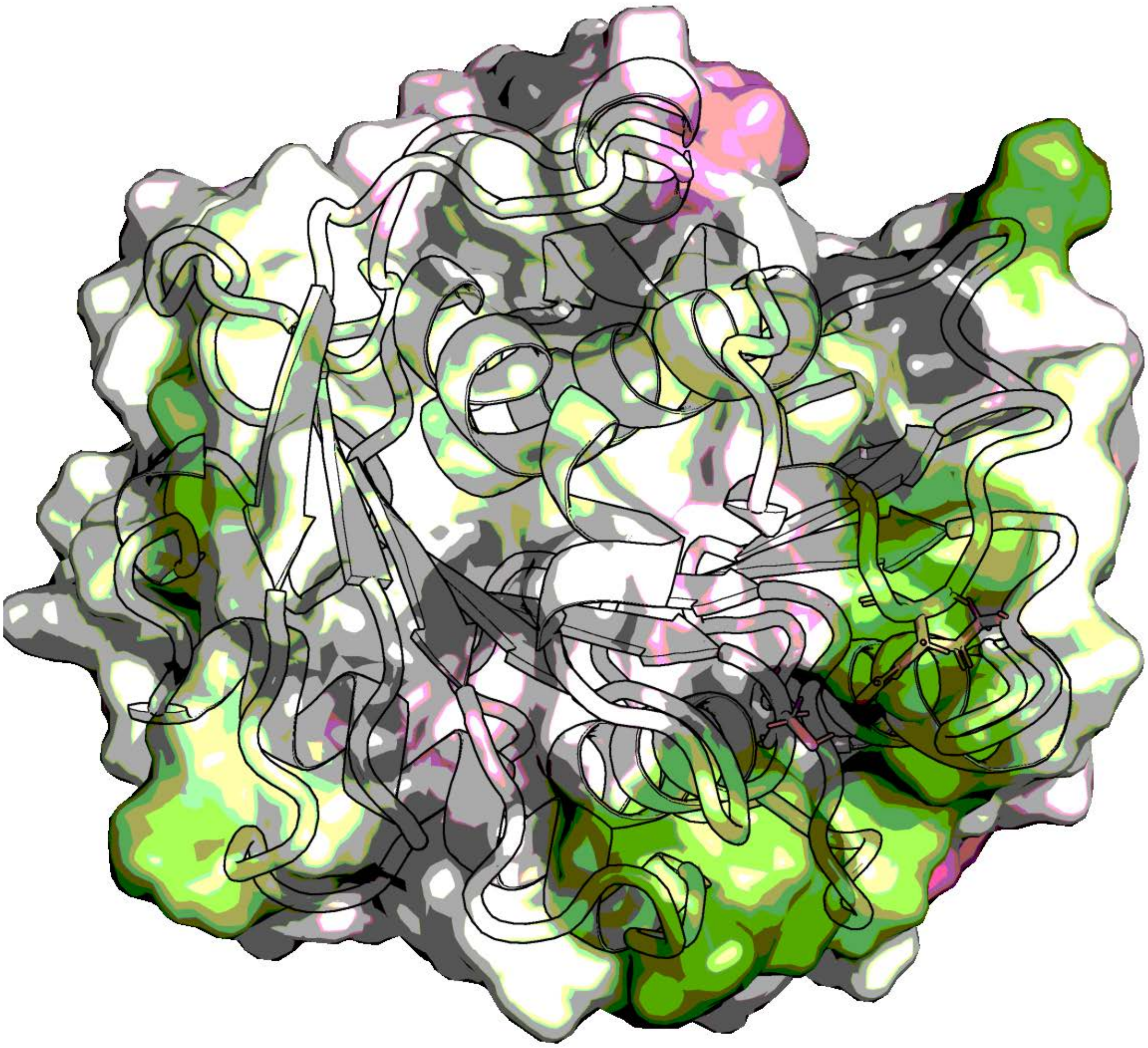

TEP178_highlights.png

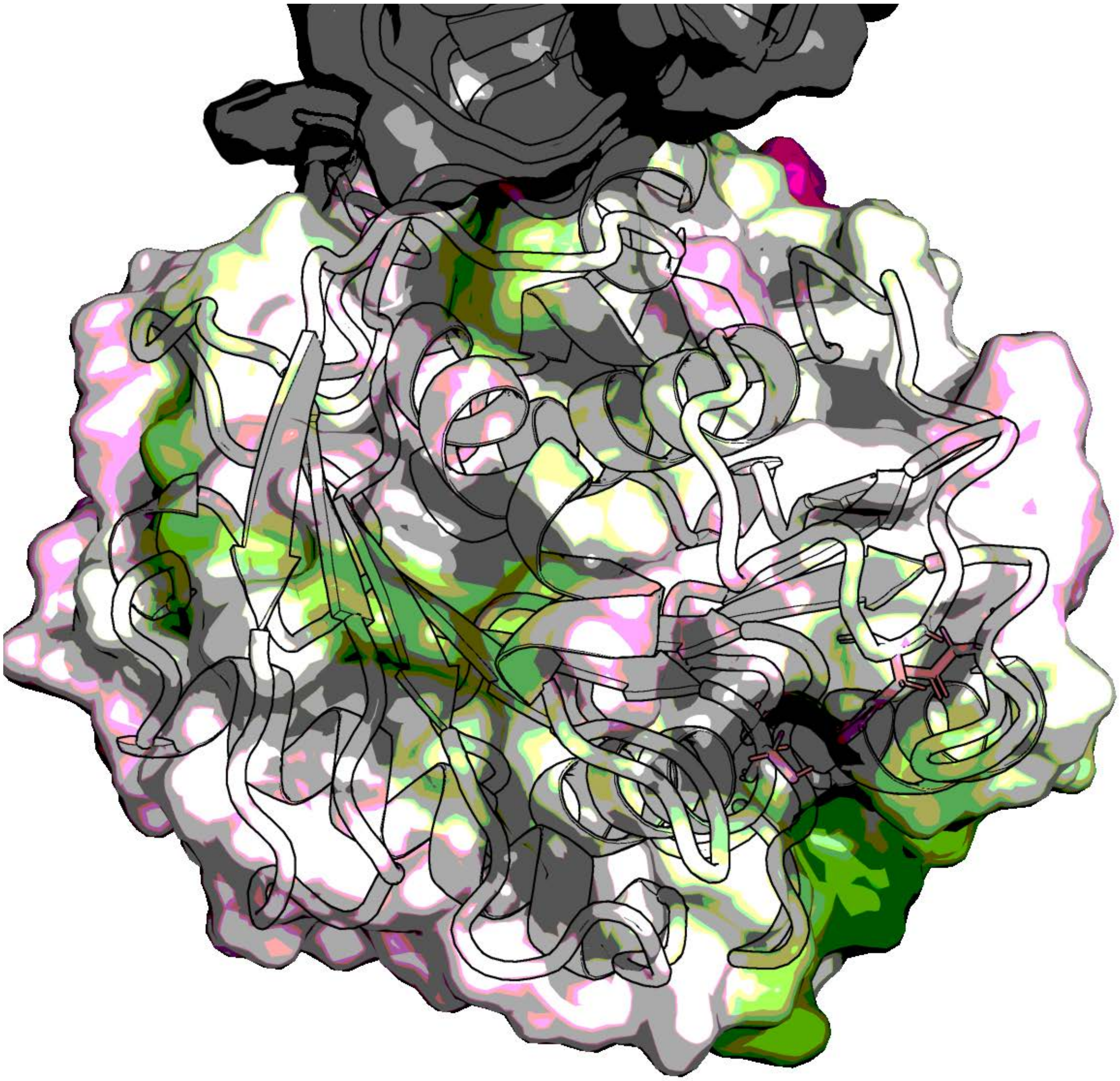

TEP181_highlights.png

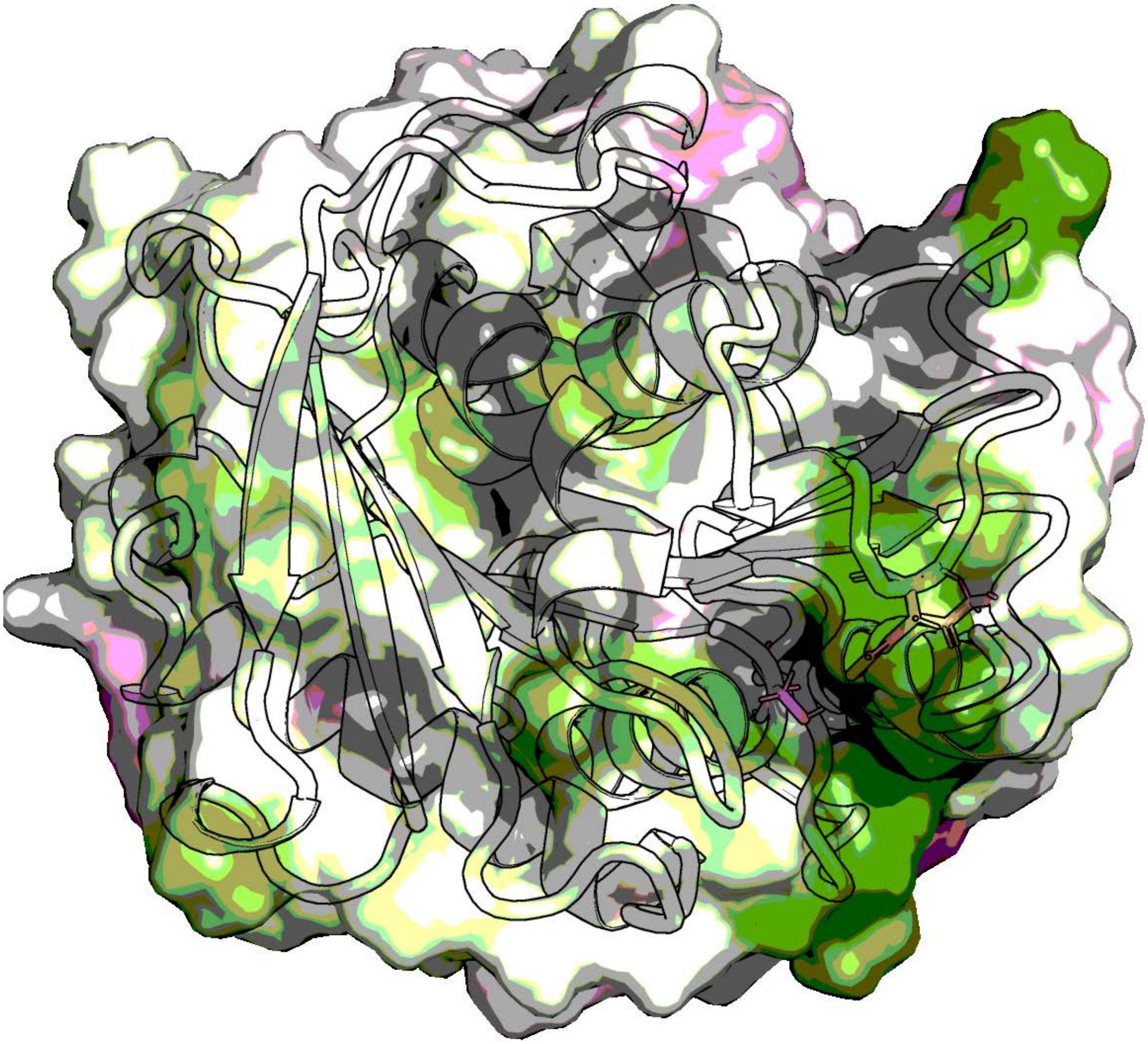

TEP182_highlights.png

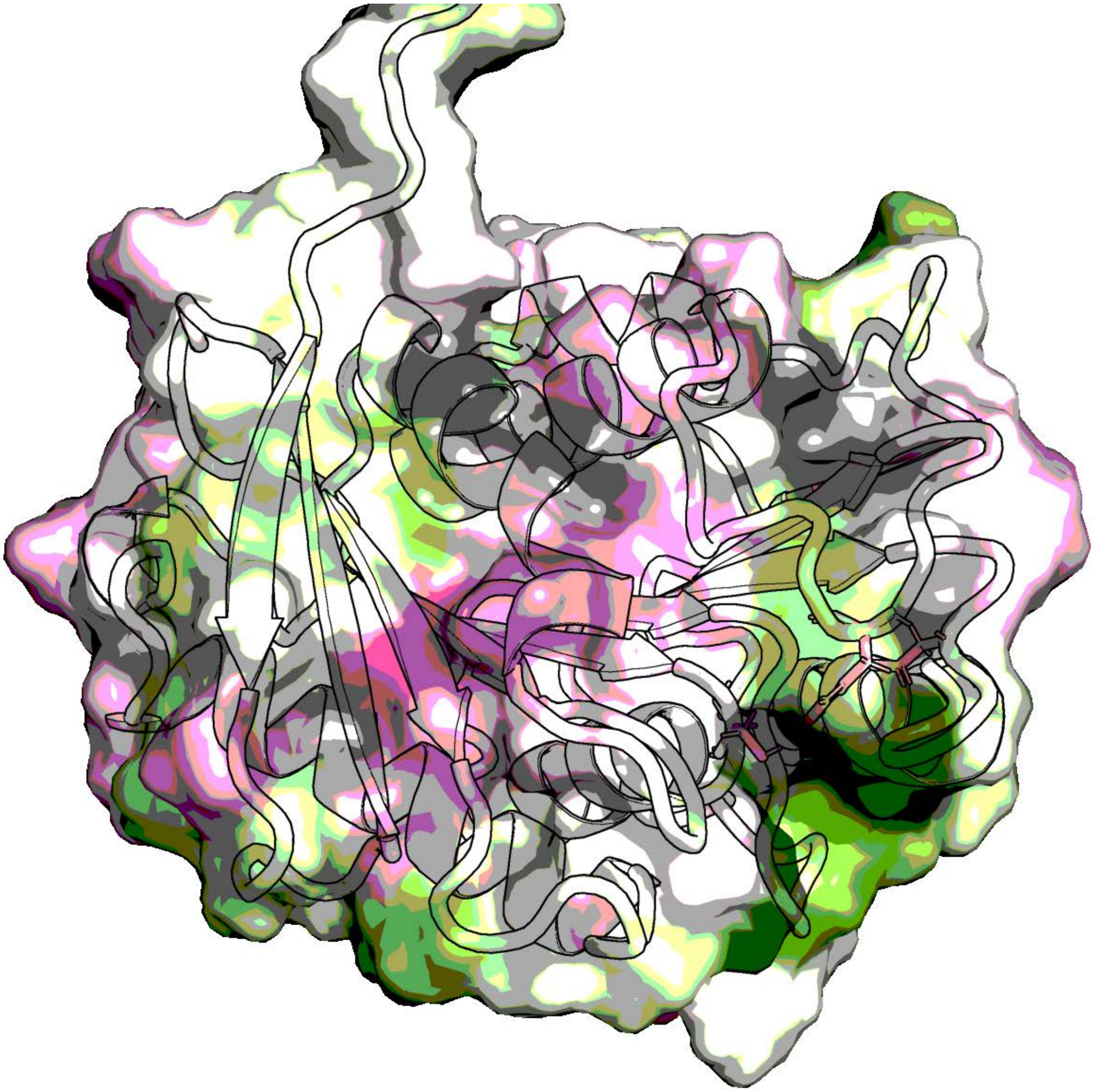

TEP189_highlights.png

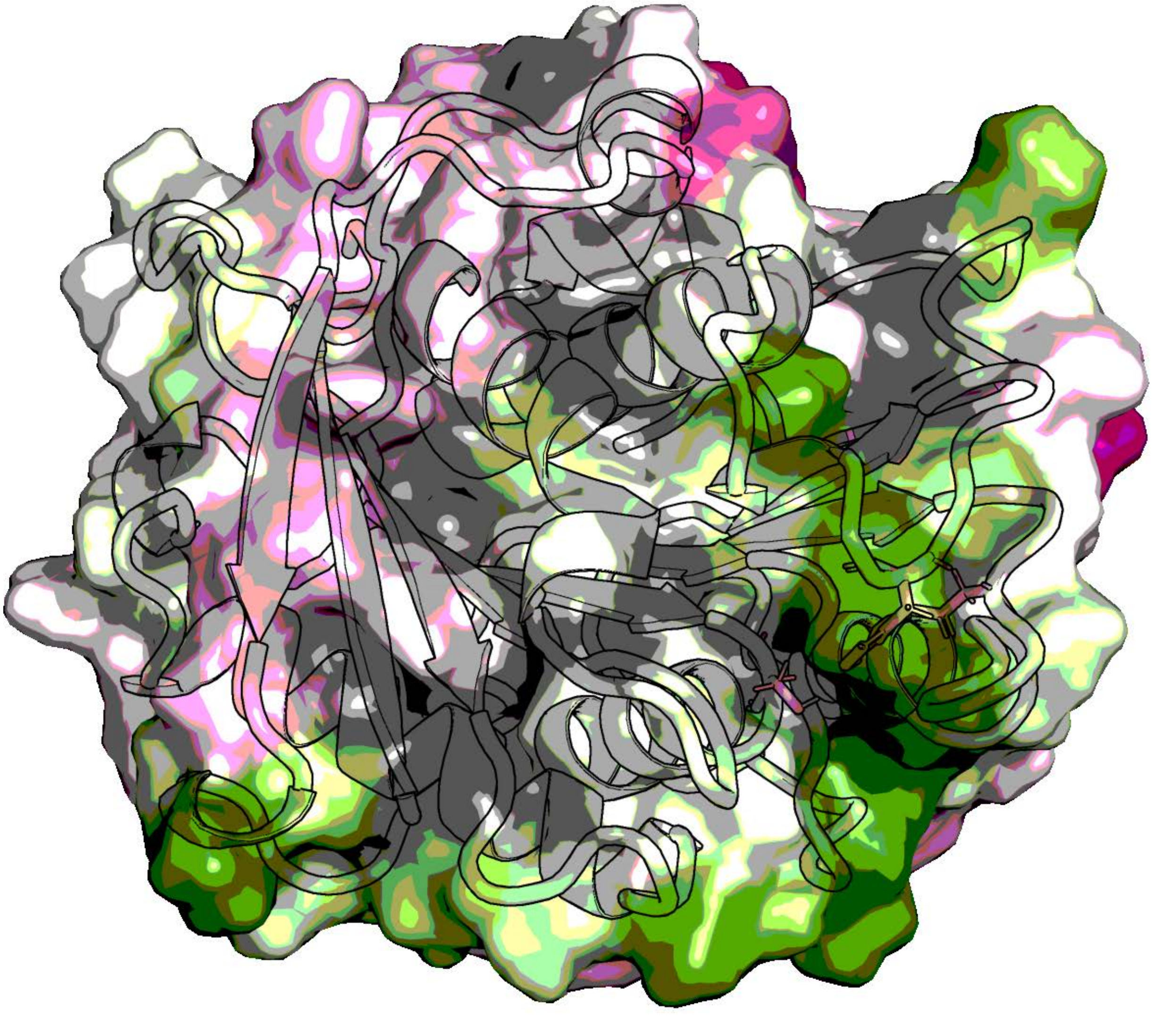

TEP194_highlights.png

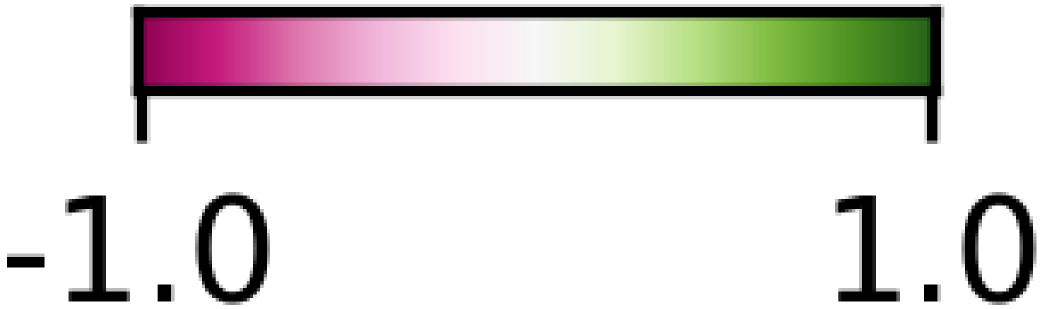

_LEGEND.png

Observed conserved residue features

Created on: 2025-04-15 16:07:37

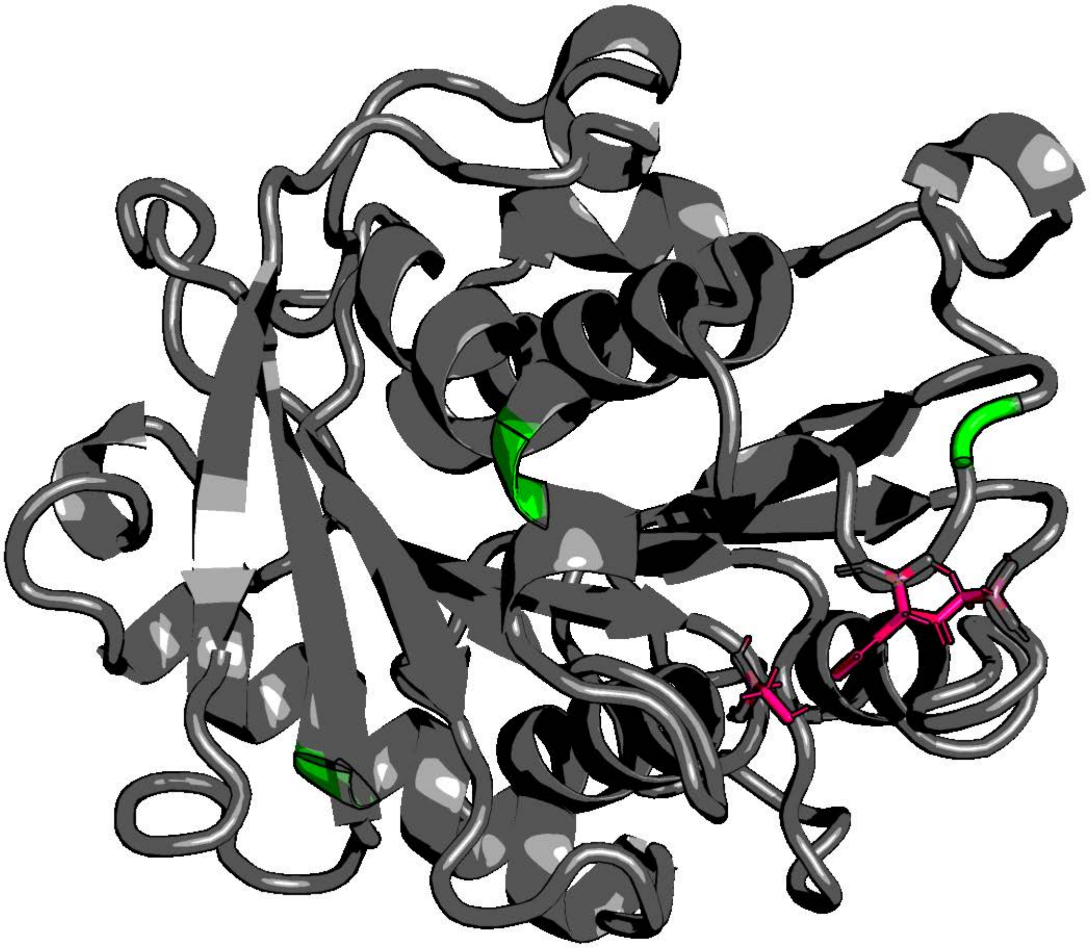

DP003_highlights.png

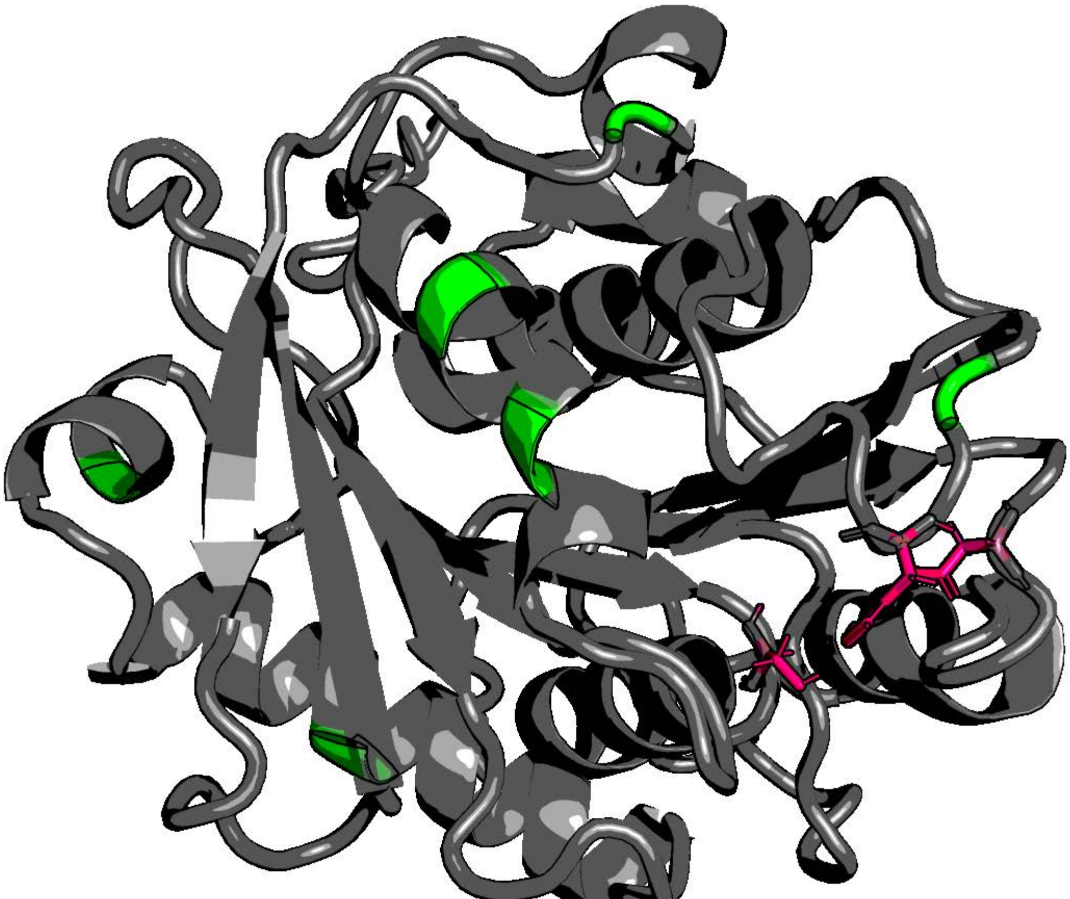

DP007_highlights.png

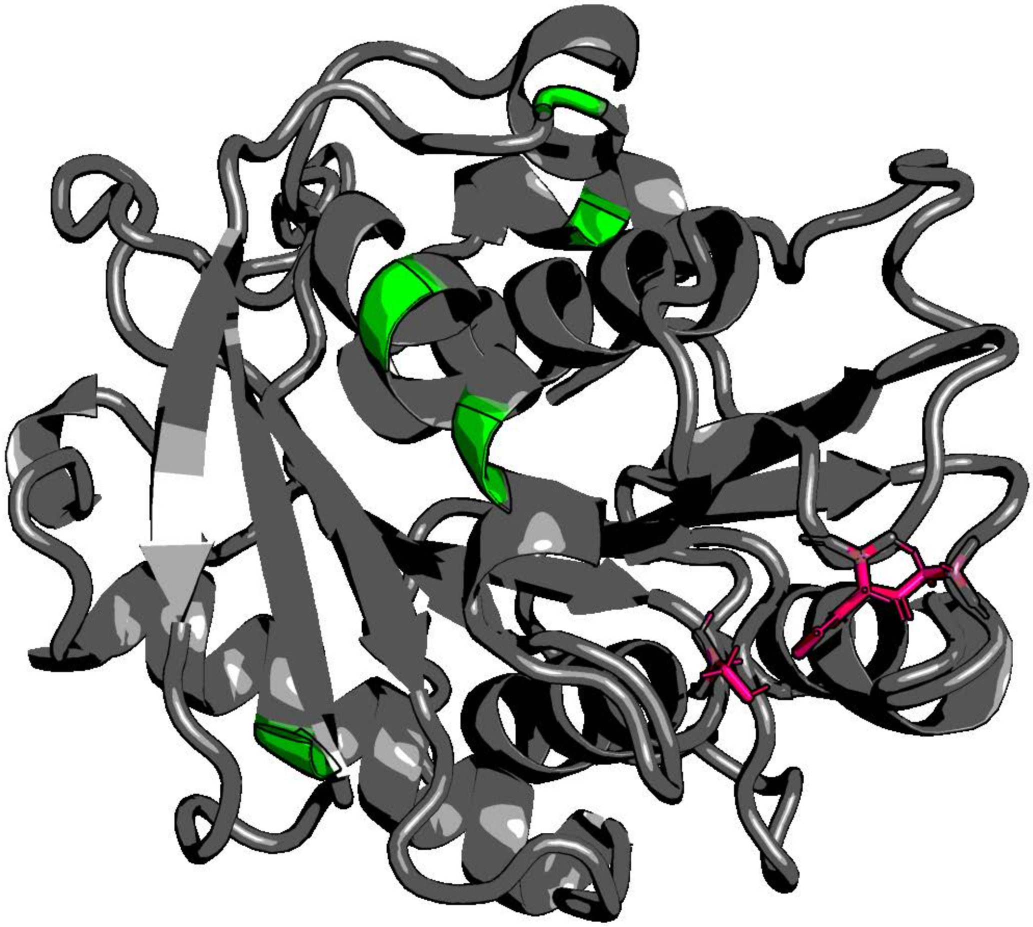

DP008_highlights.png

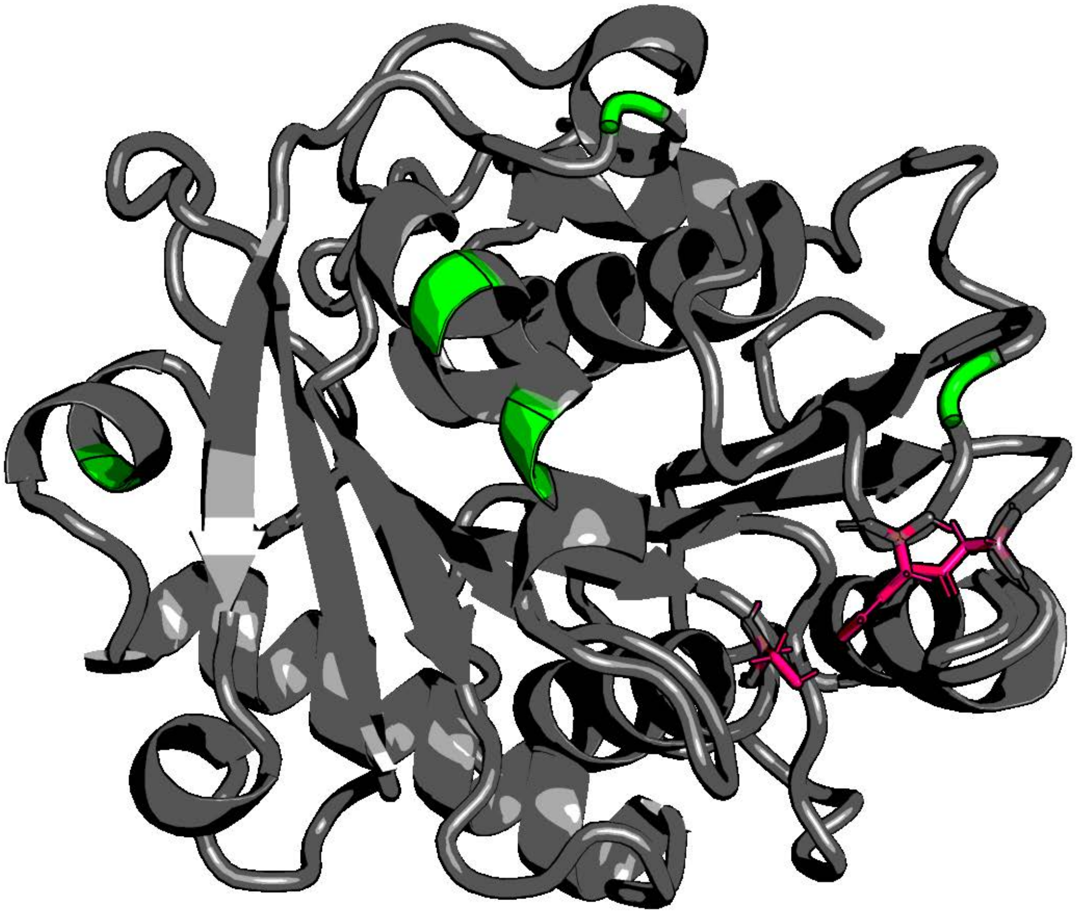

DP009_highlights.png

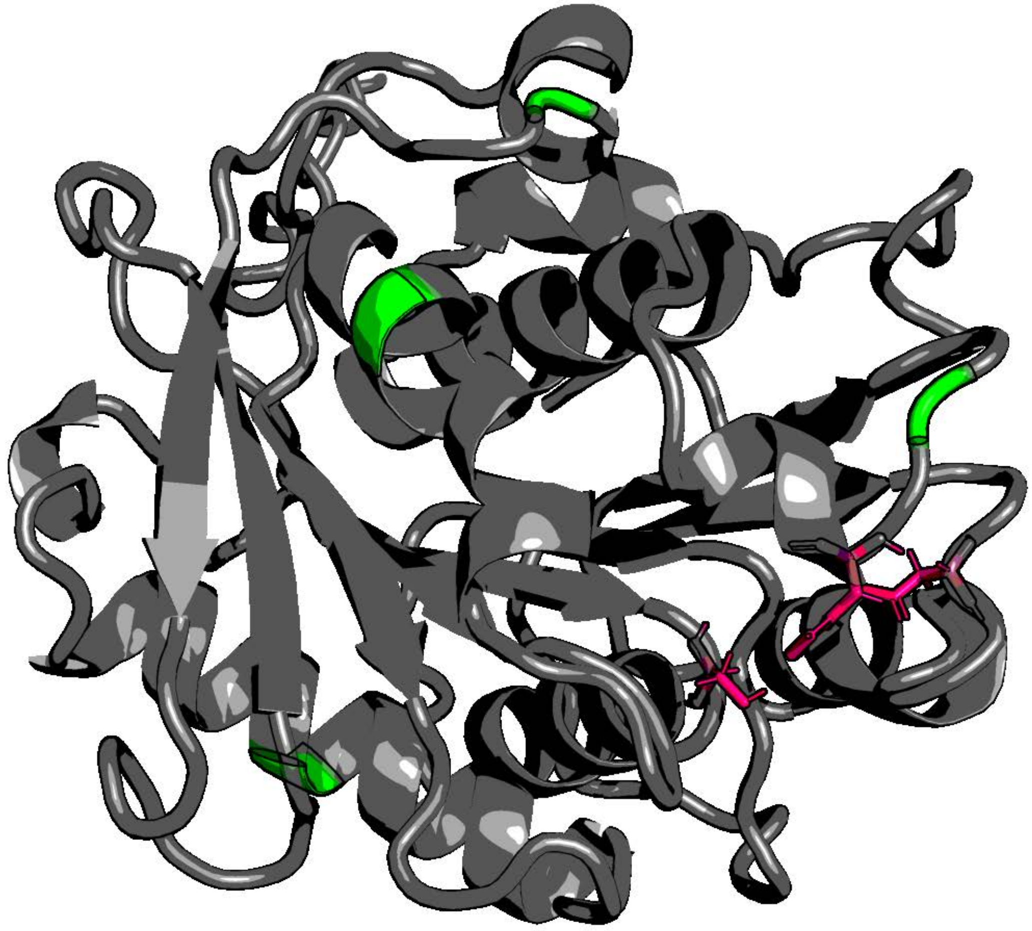

DP016_highlights.png

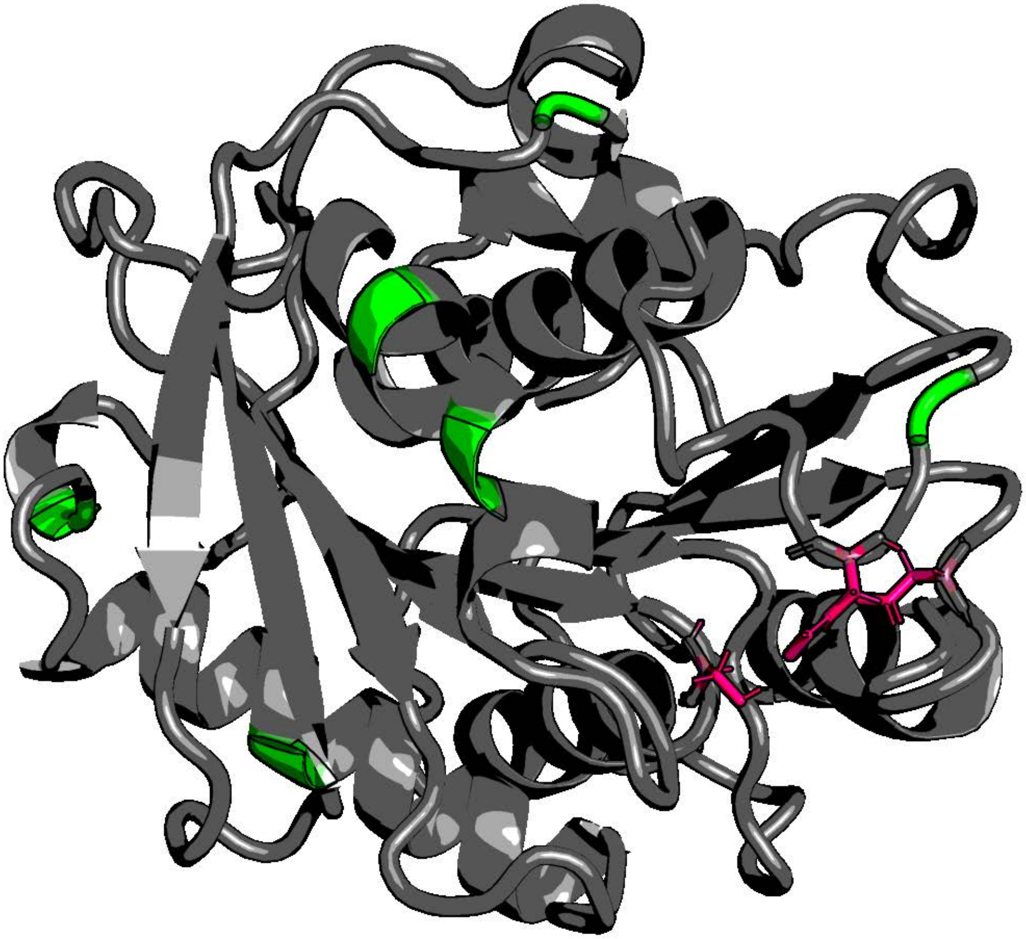

DP017_highlights.png

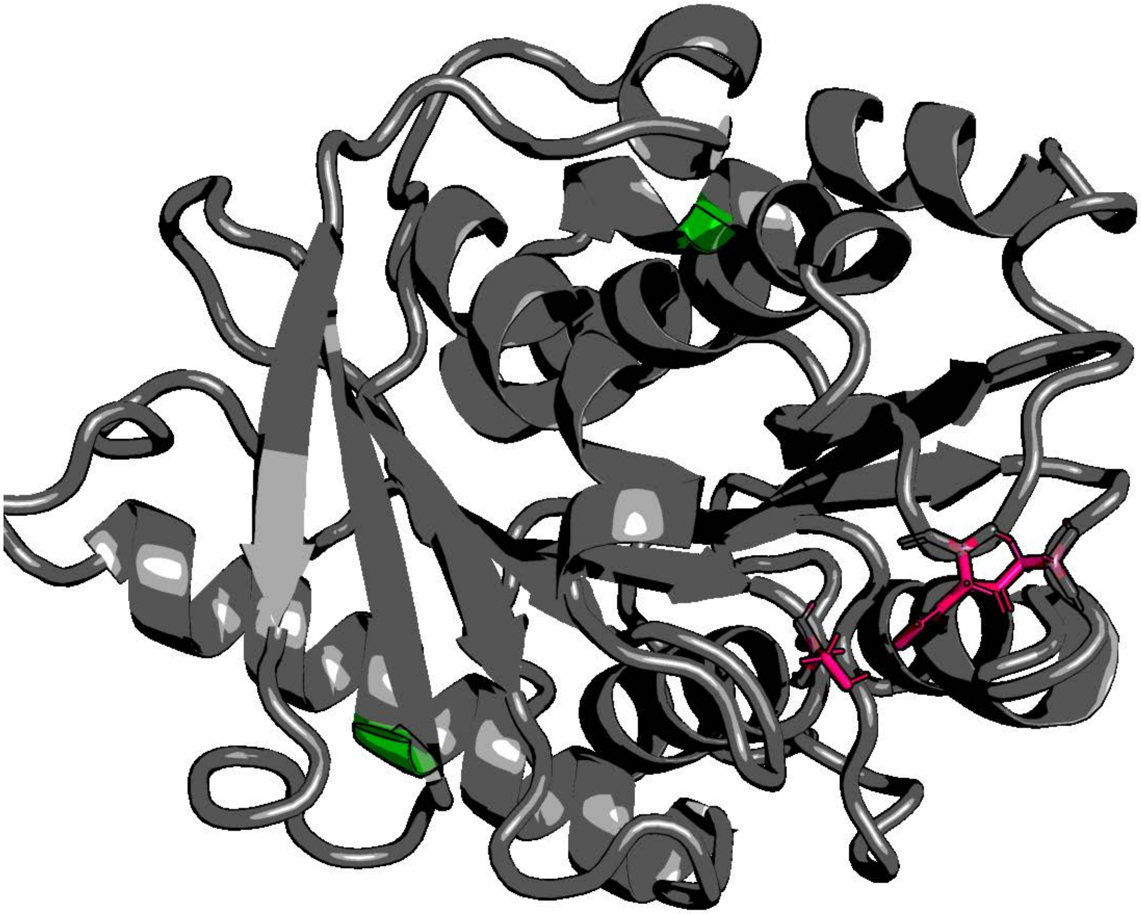

DP038_highlights.png

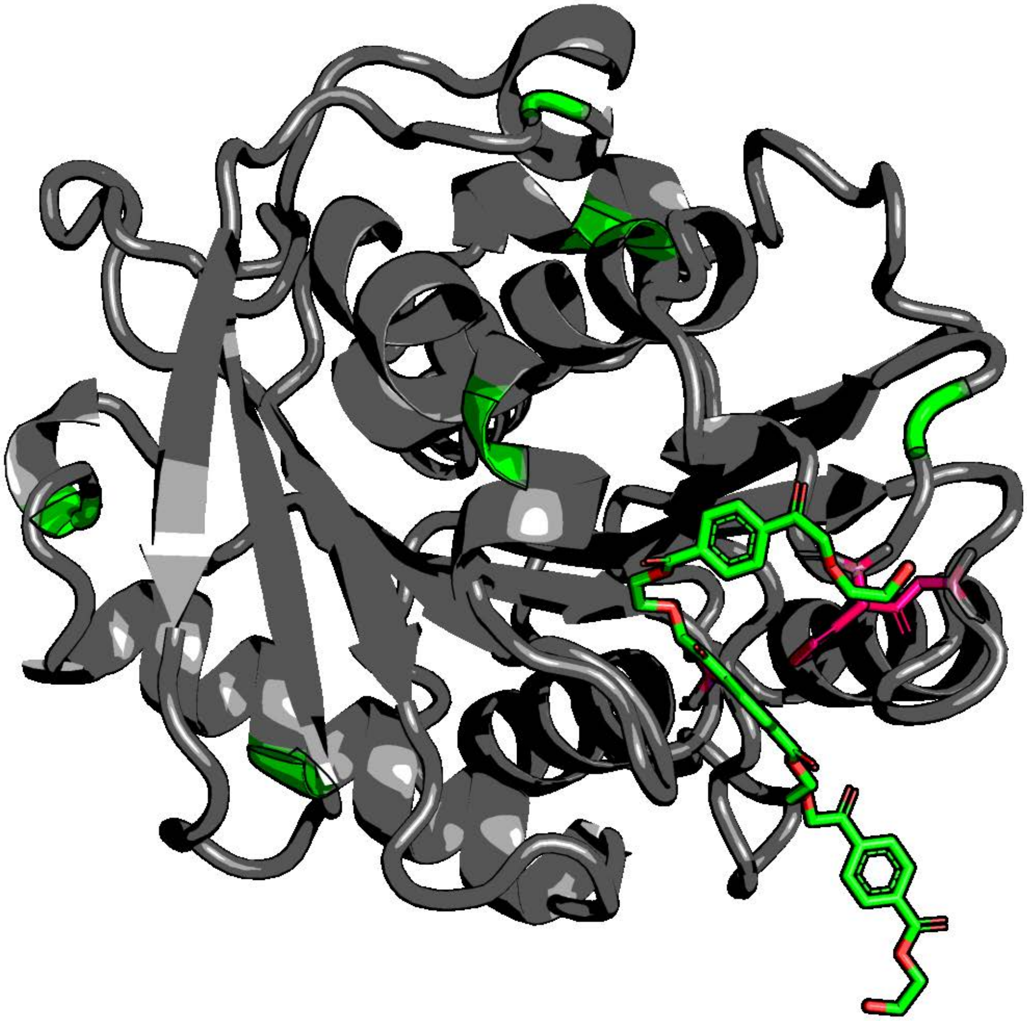

DP043_highlights.png

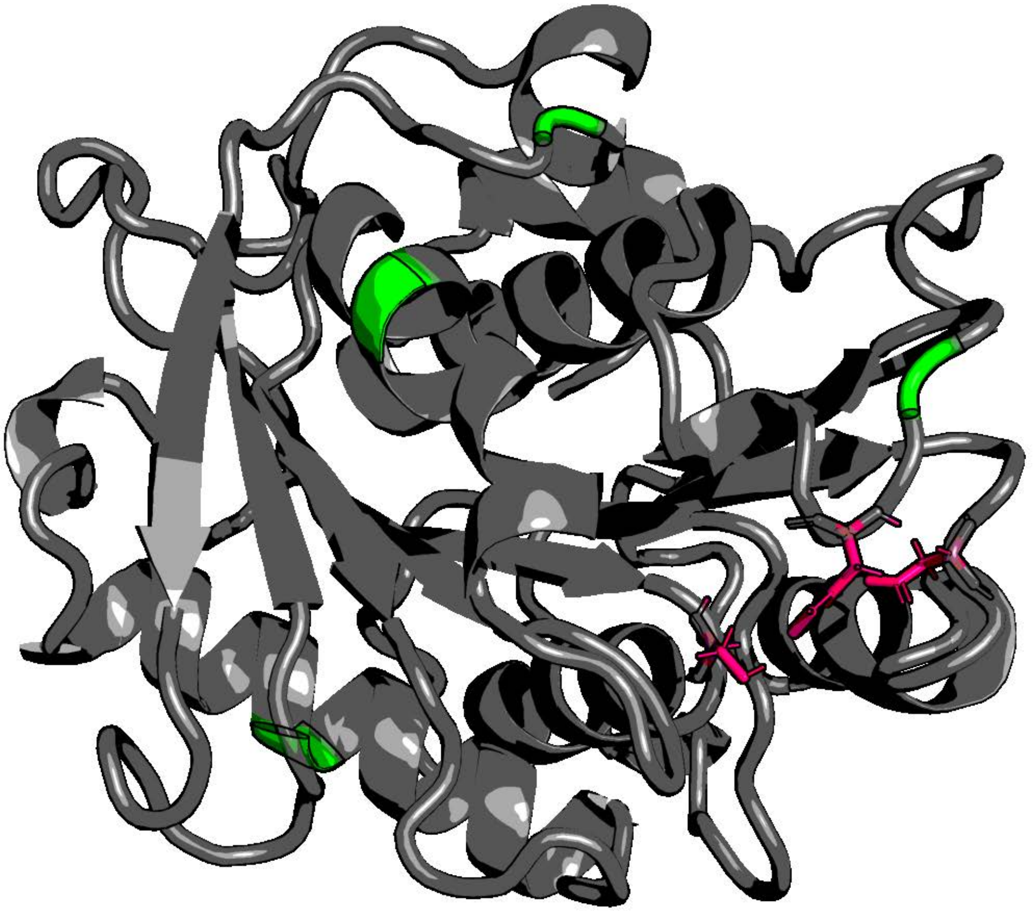

DP054_highlights.png

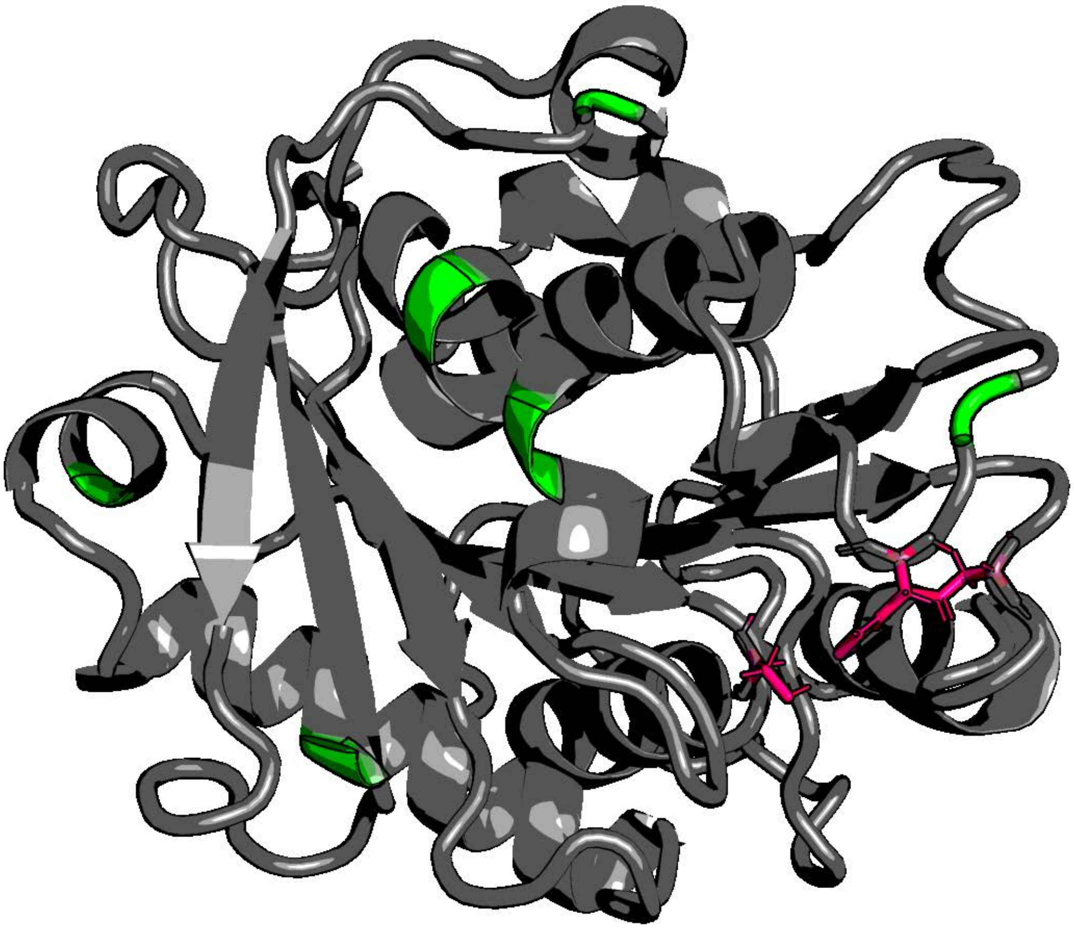

DP056_highlights.png

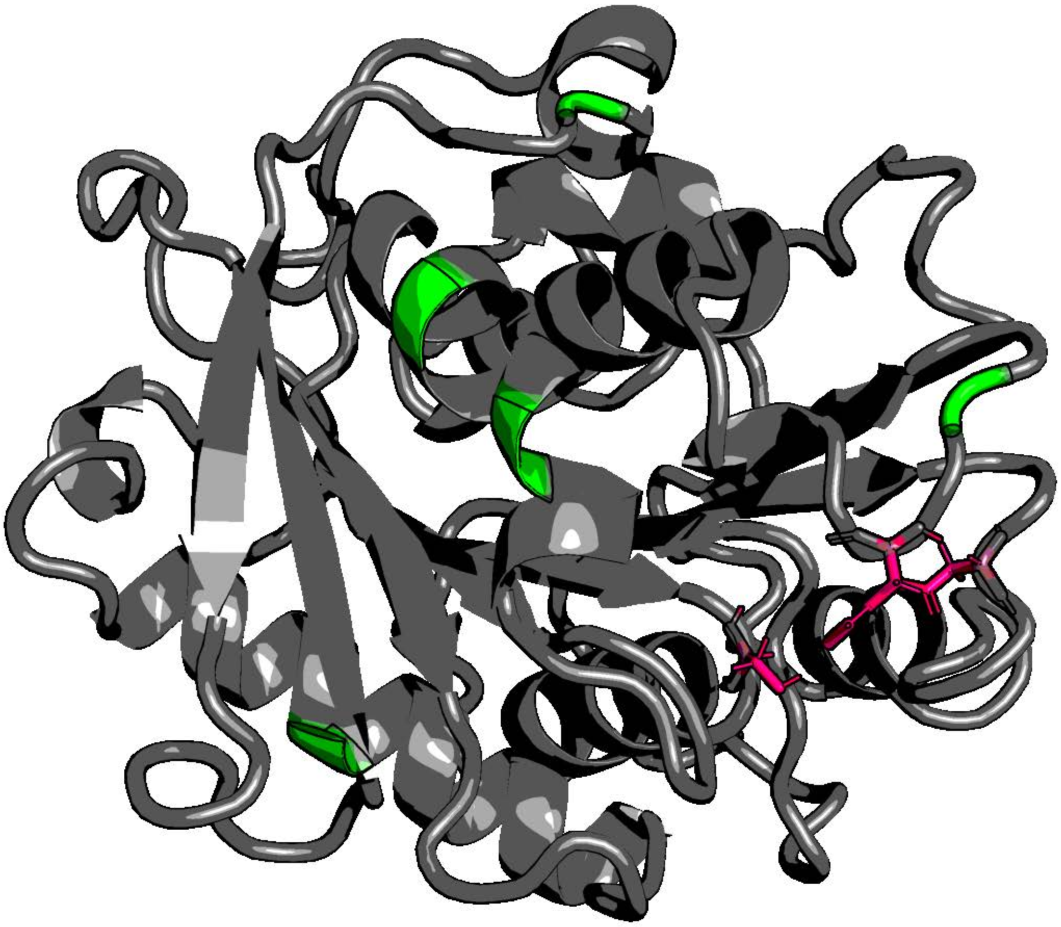

DP057_highlights.png

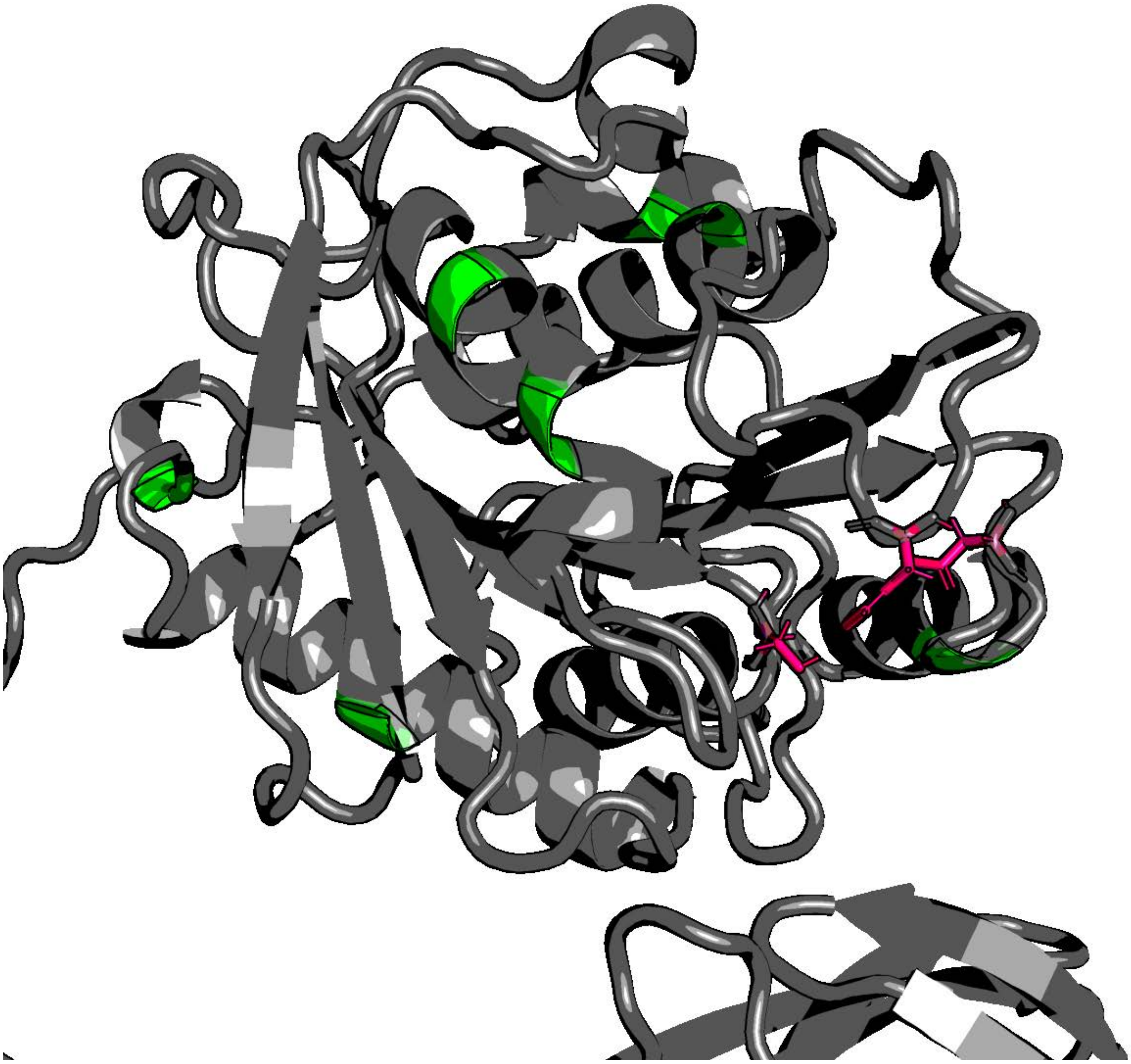

DP059_highlights.png

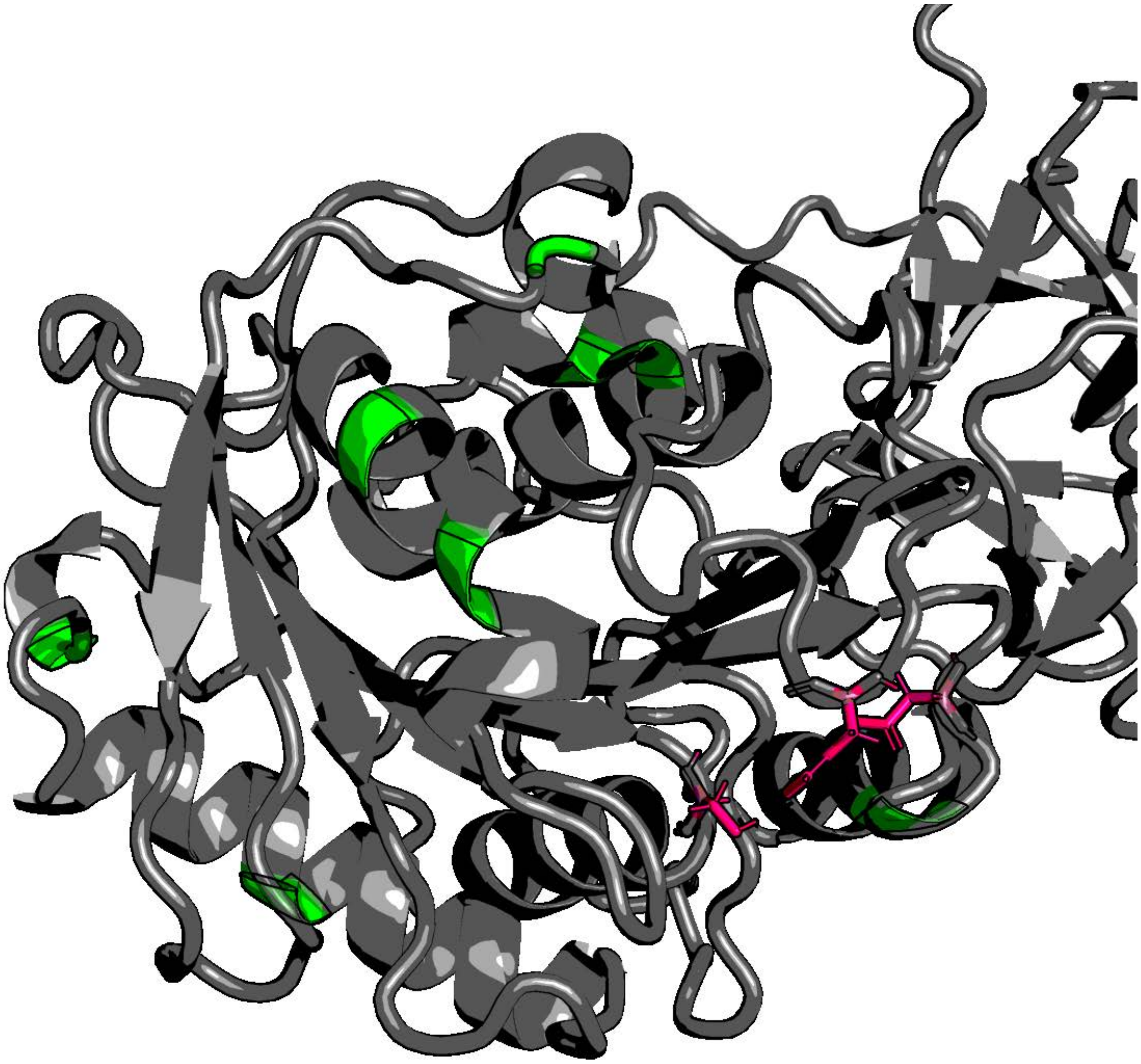

DP061_highlights.png

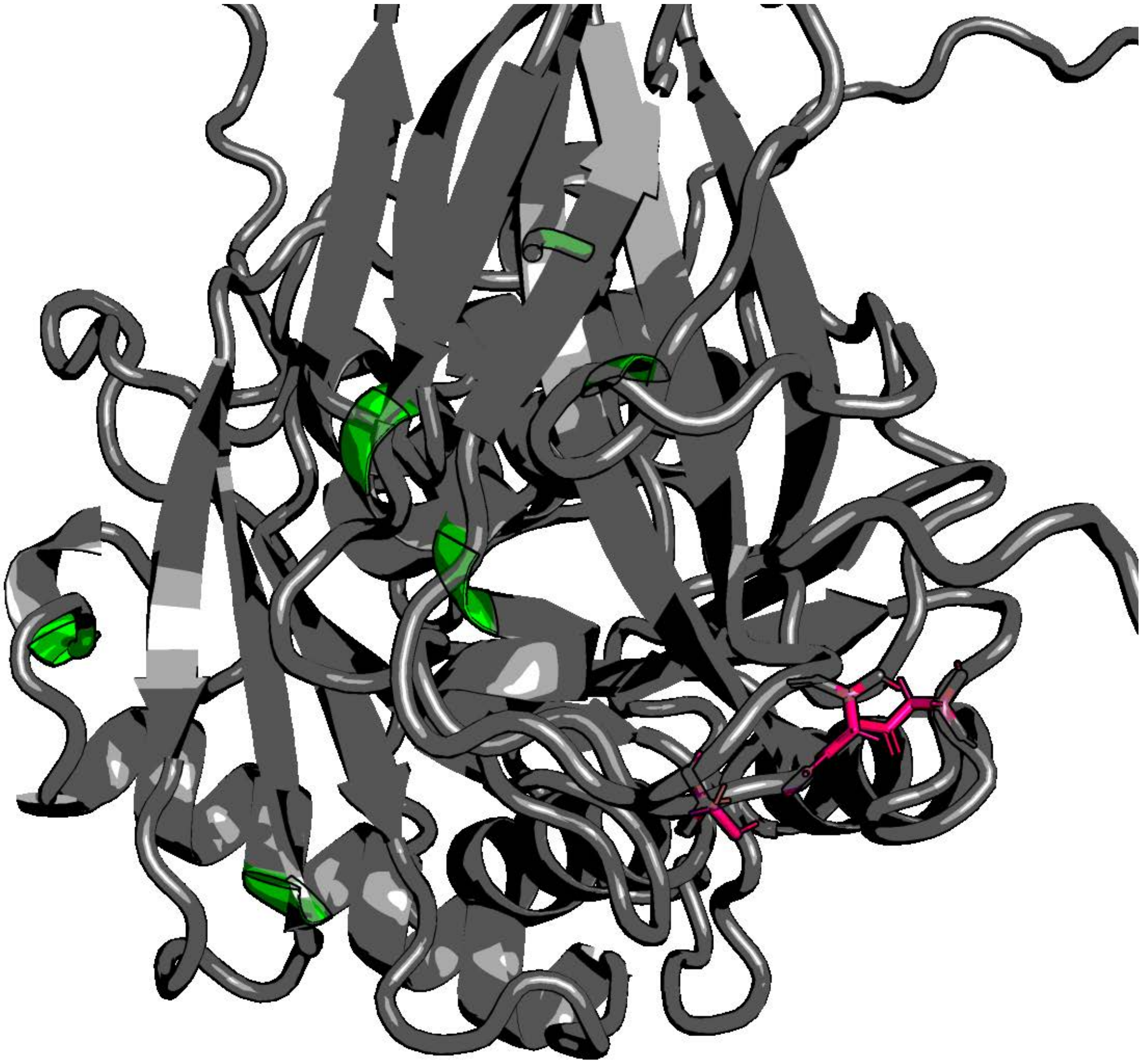

DP068_highlights.png

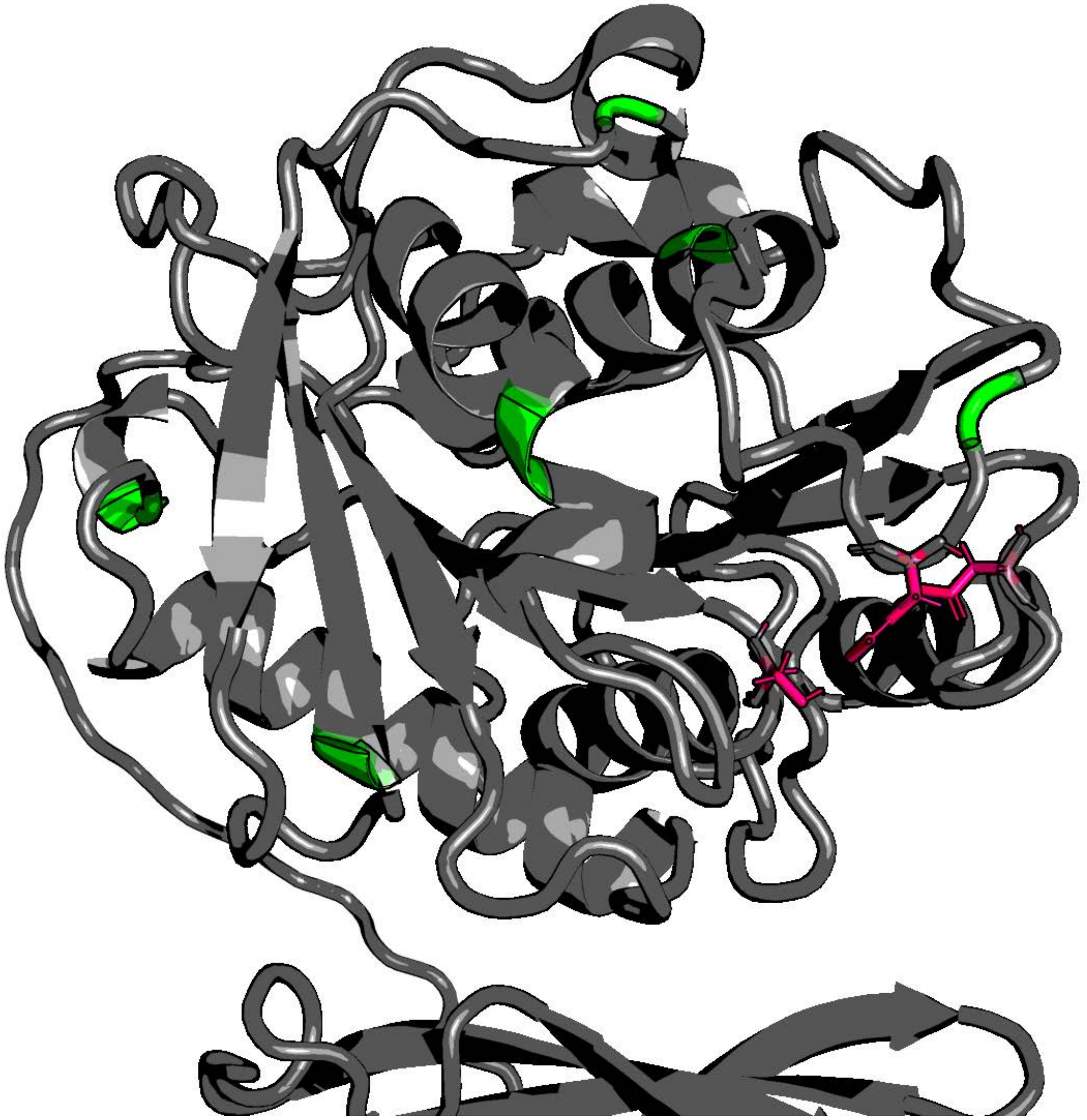

DP072_highlights.png

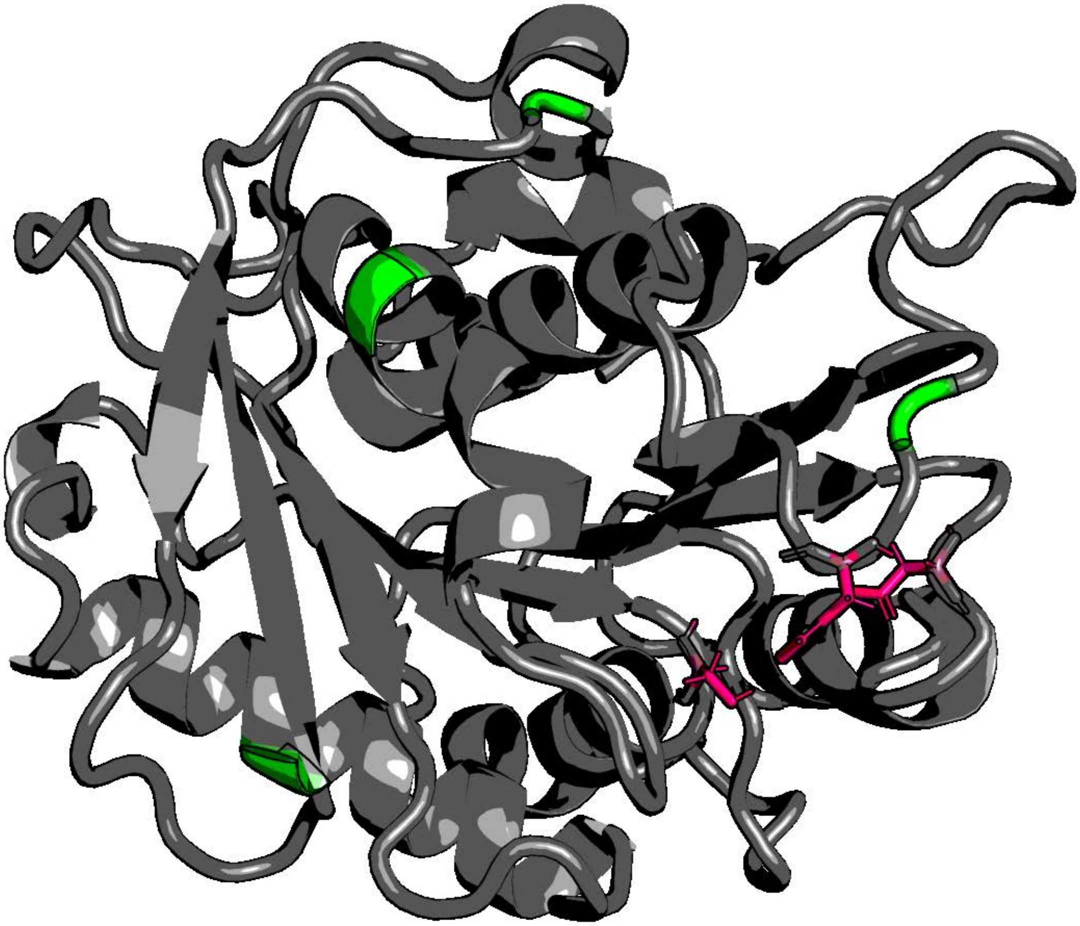

DP077_highlights.png

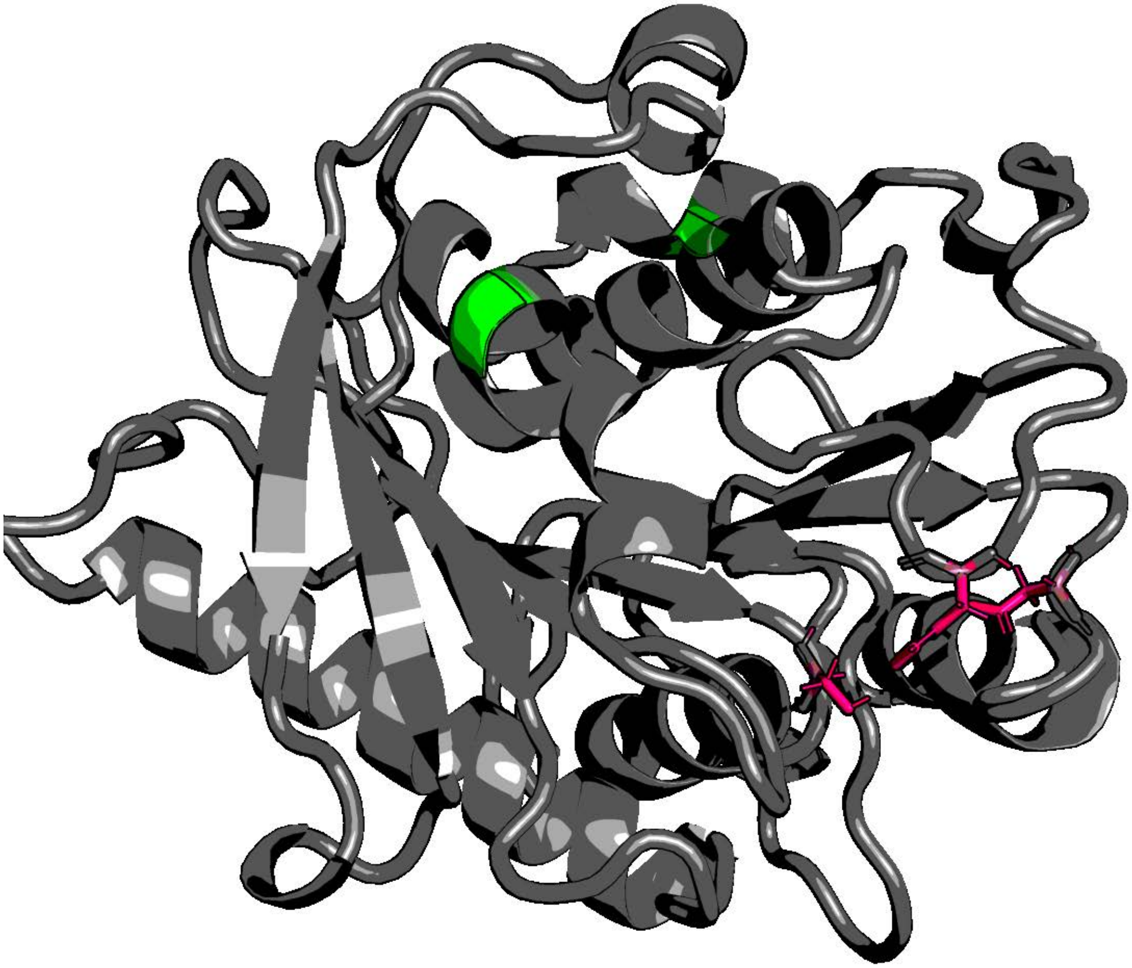

DP078_highlights.png

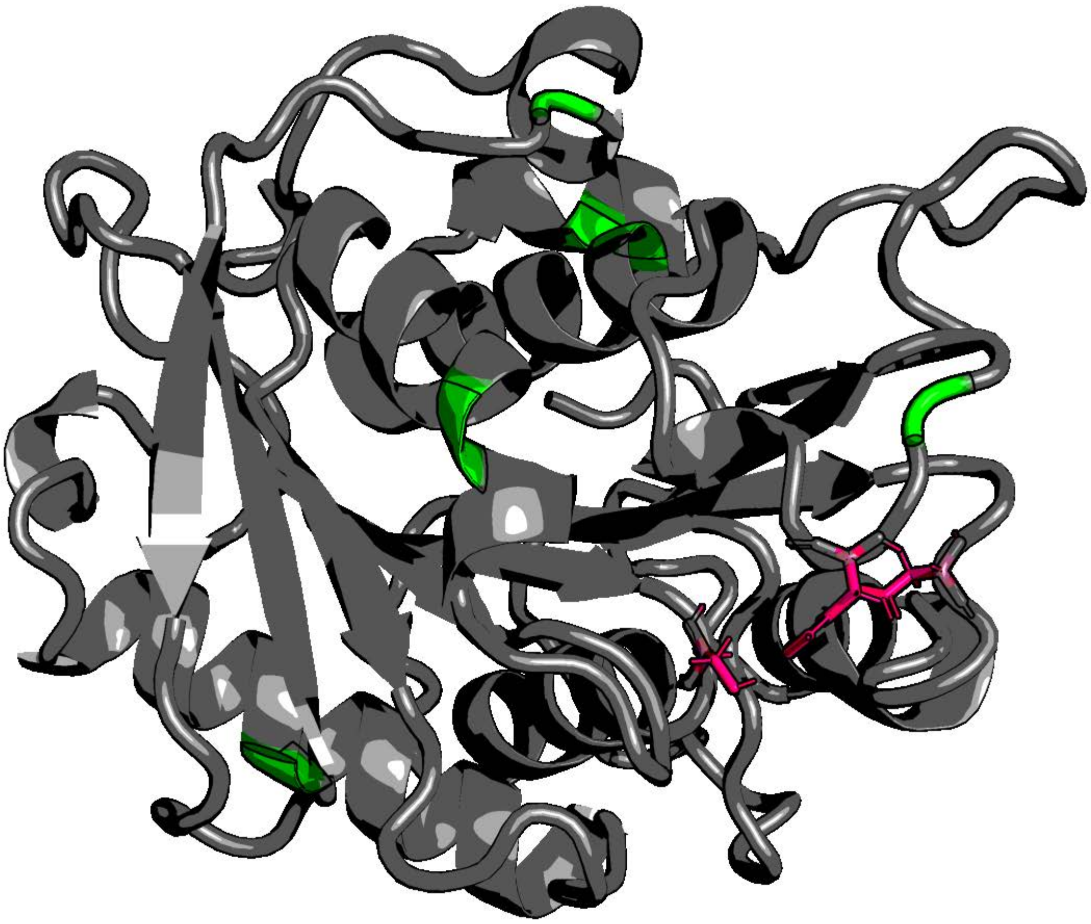

DP080_highlights.png

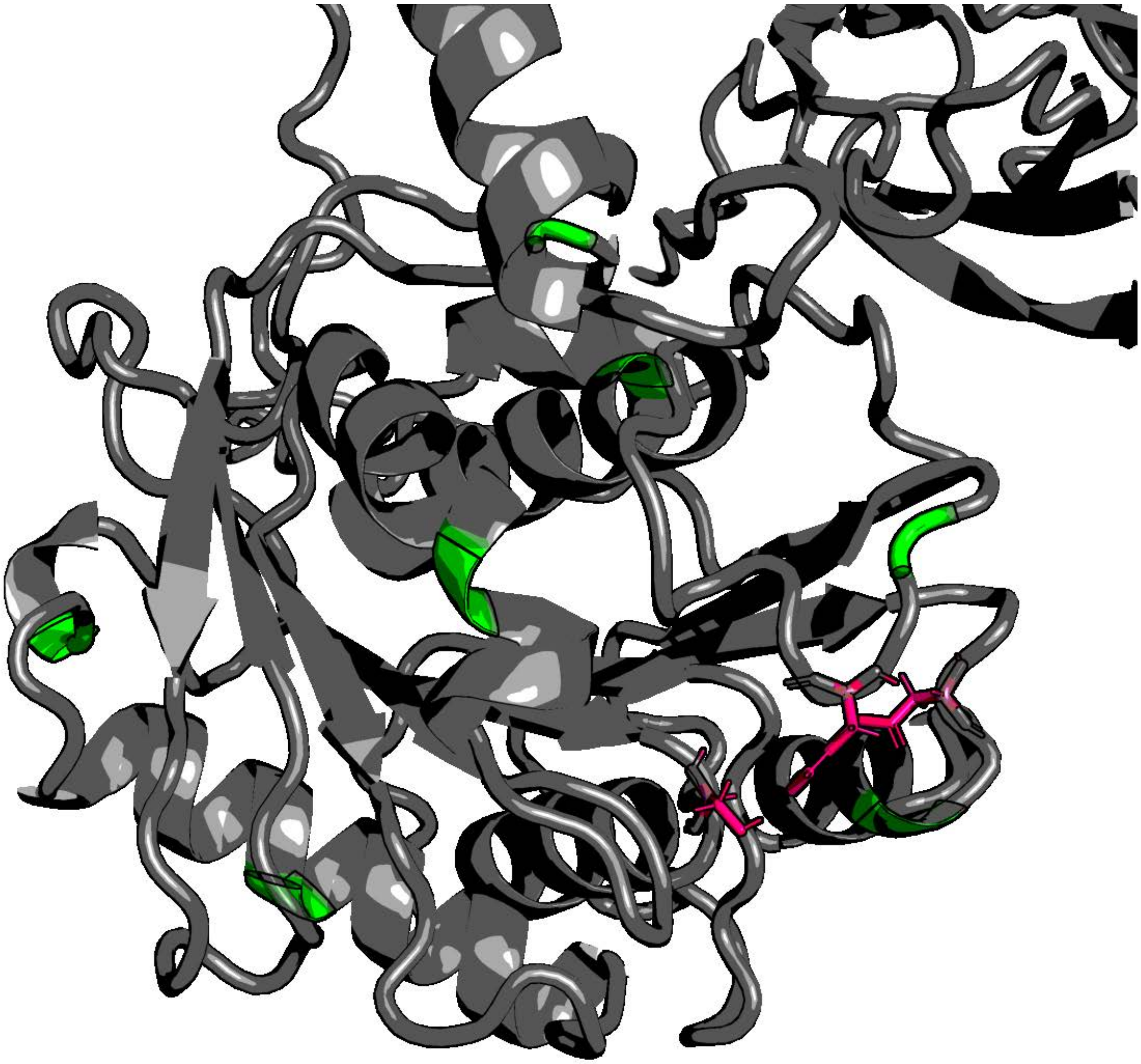

DP081_highlights.png

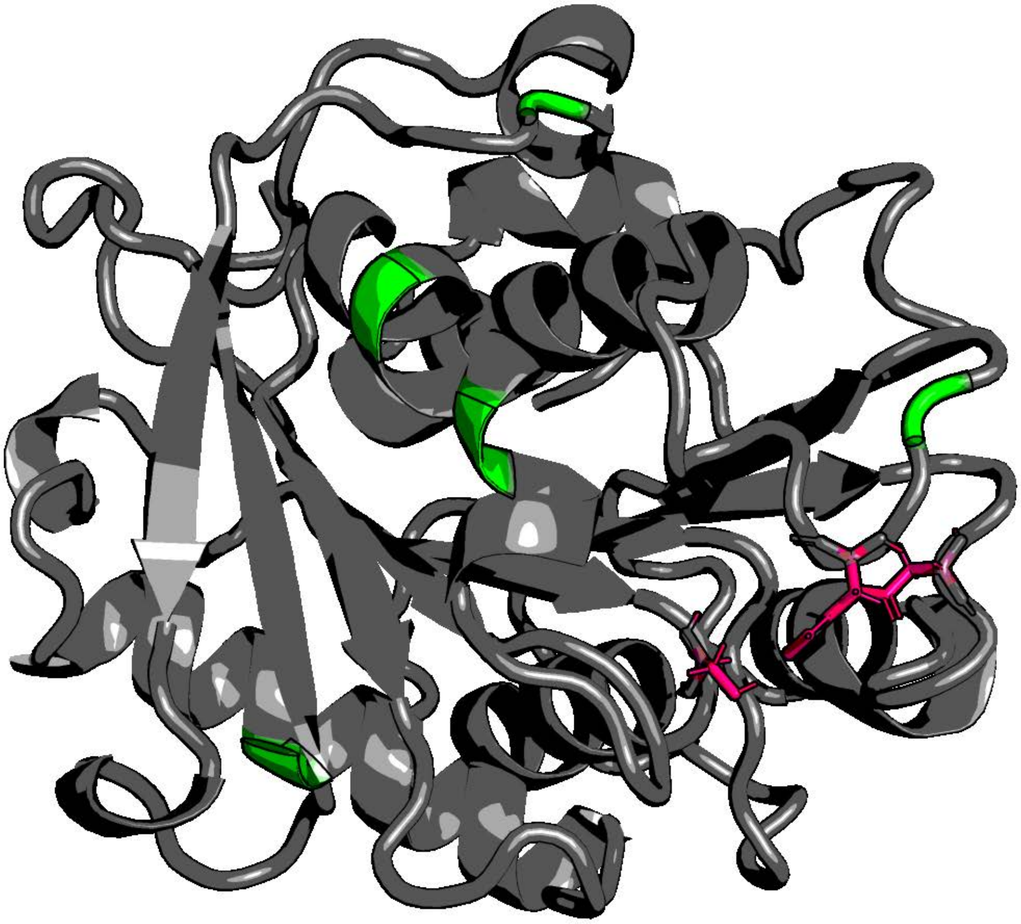

DP082_highlights.png

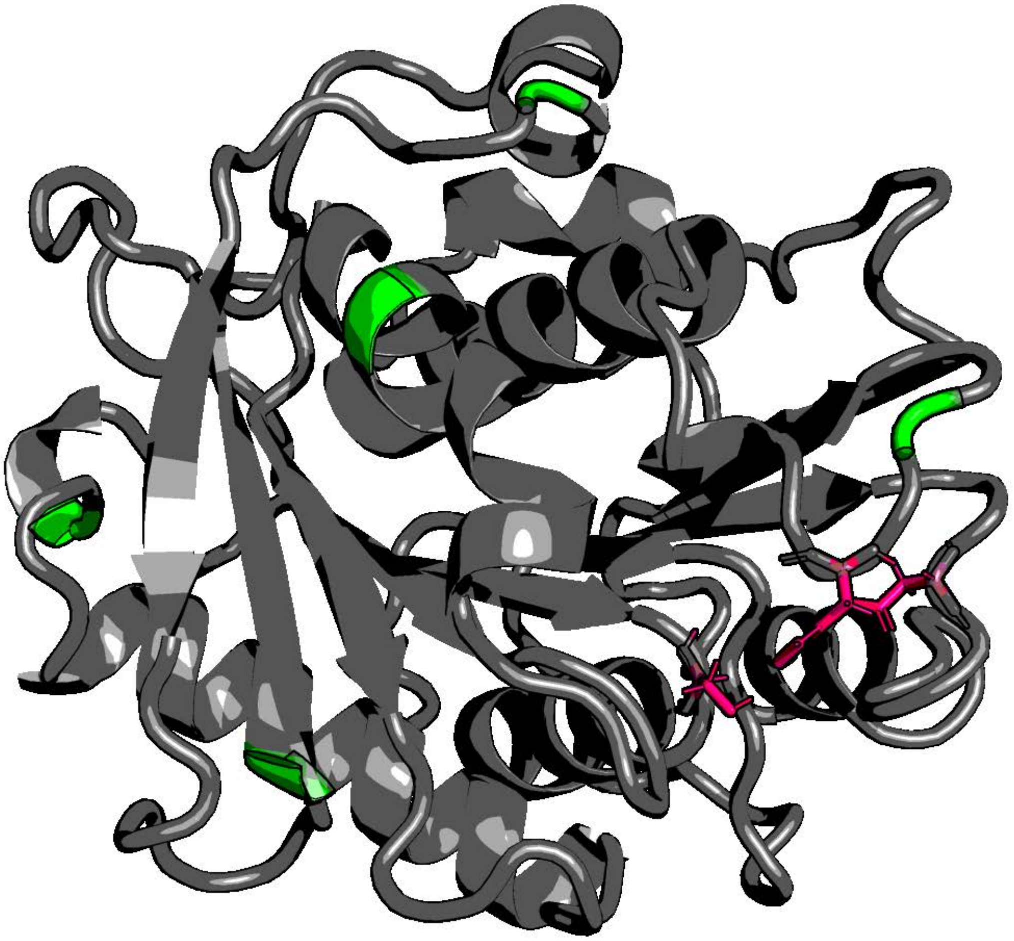

DP084_highlights.png

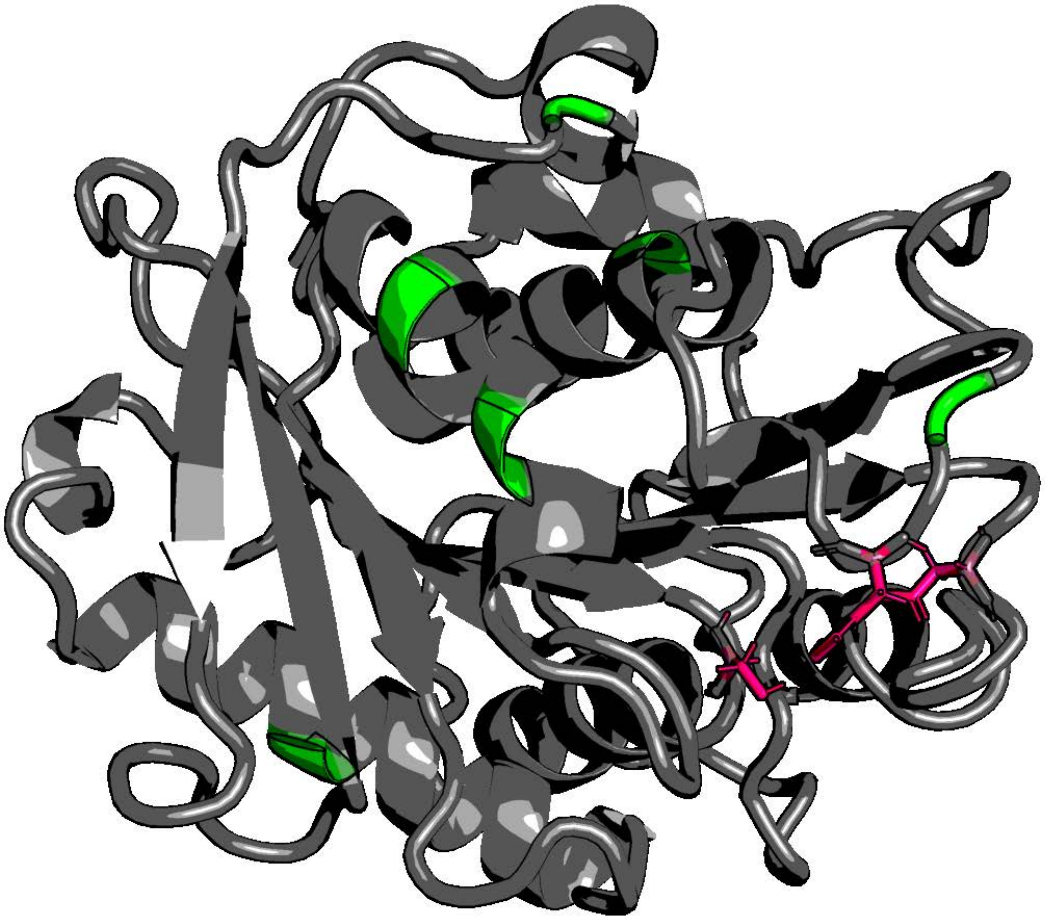

DP089_highlights.png

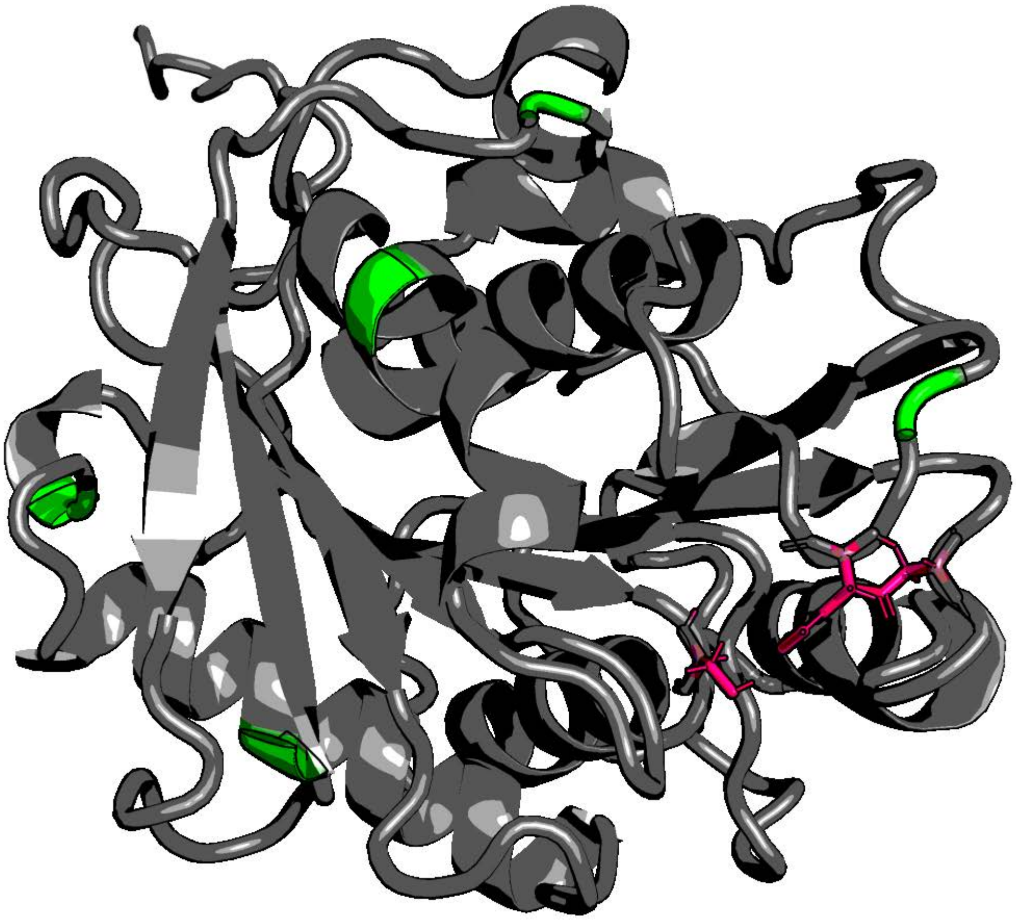

DP090_highlights.png

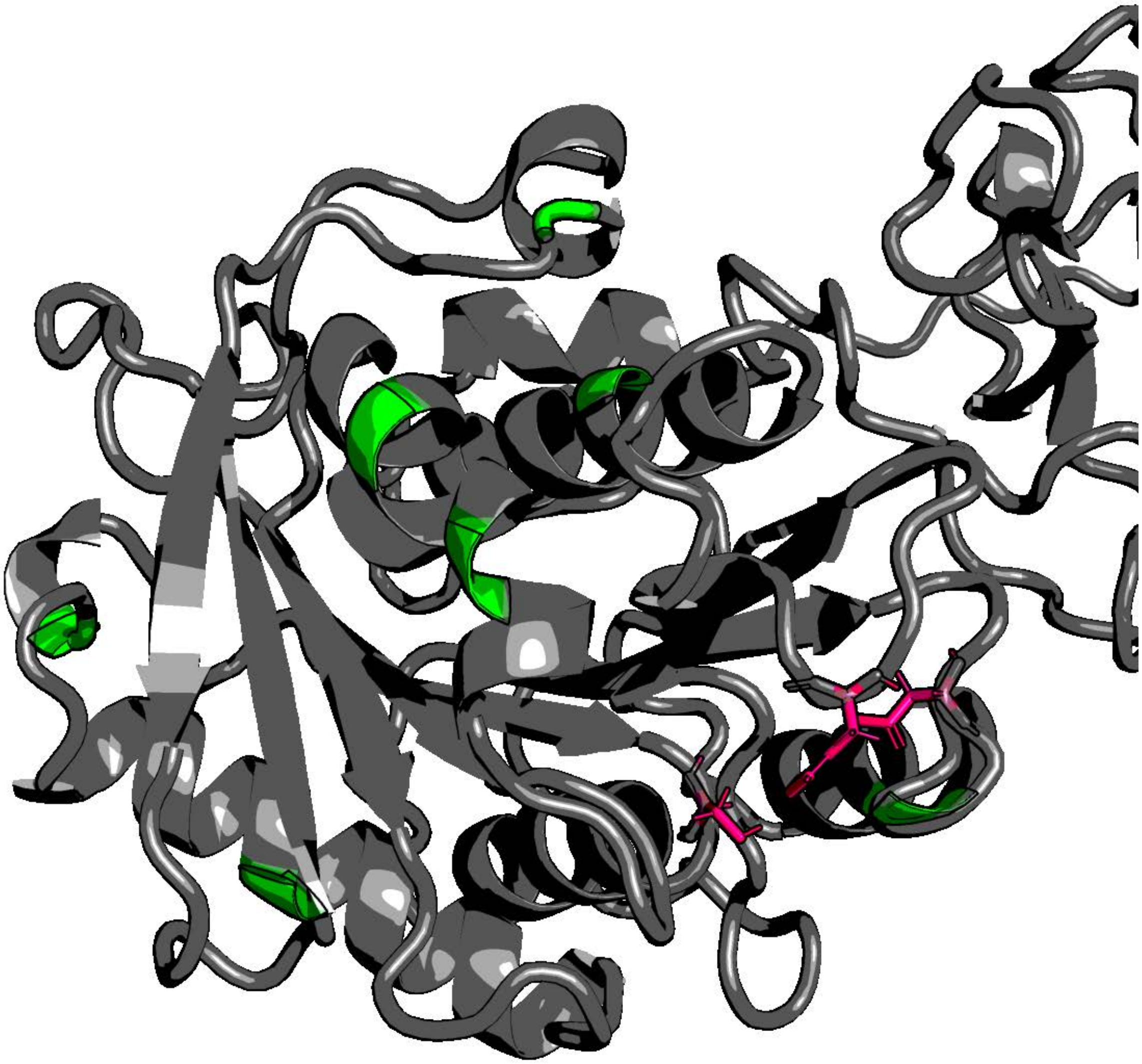

DP092_highlights.png

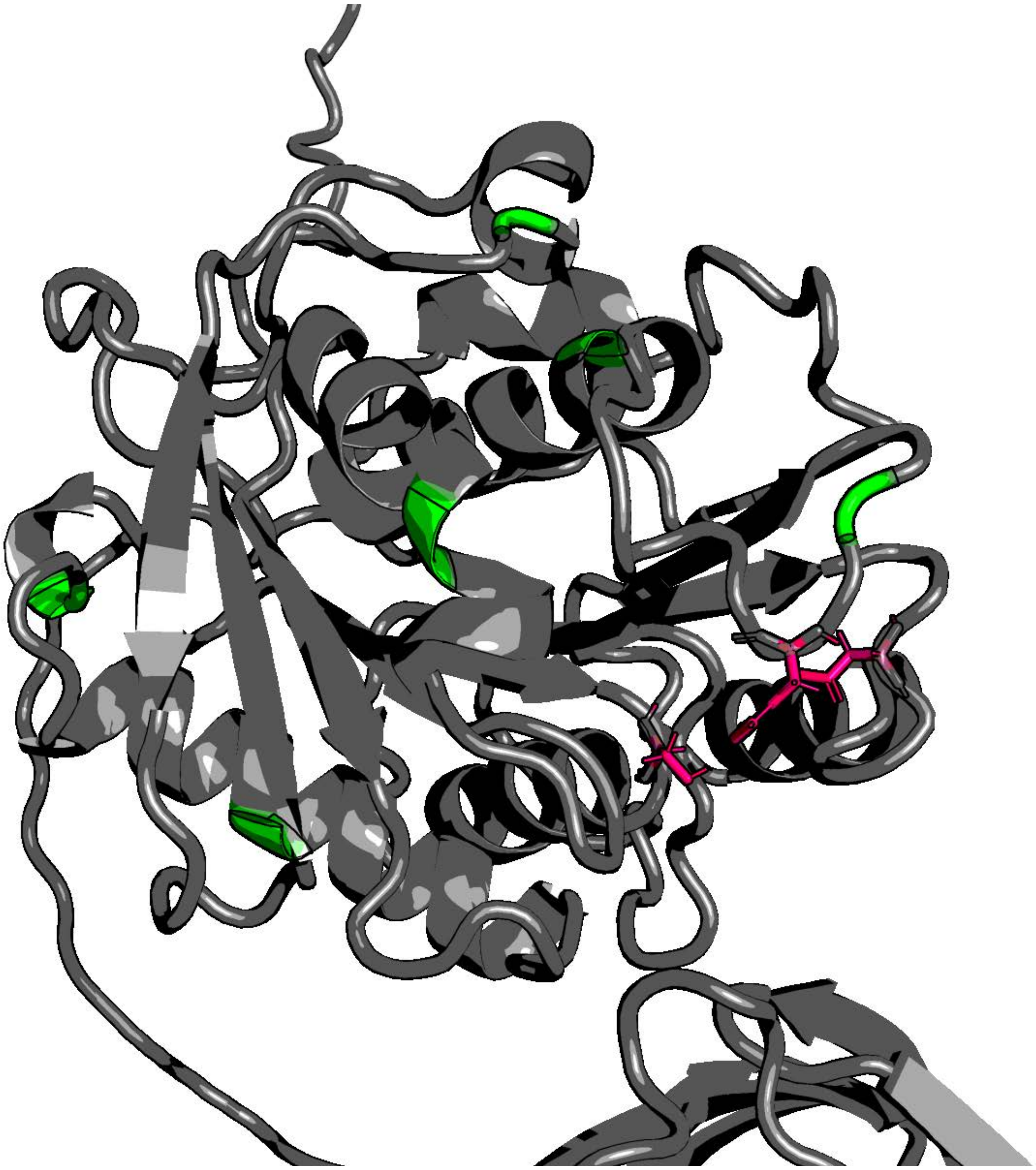

ESM006_highlights.png

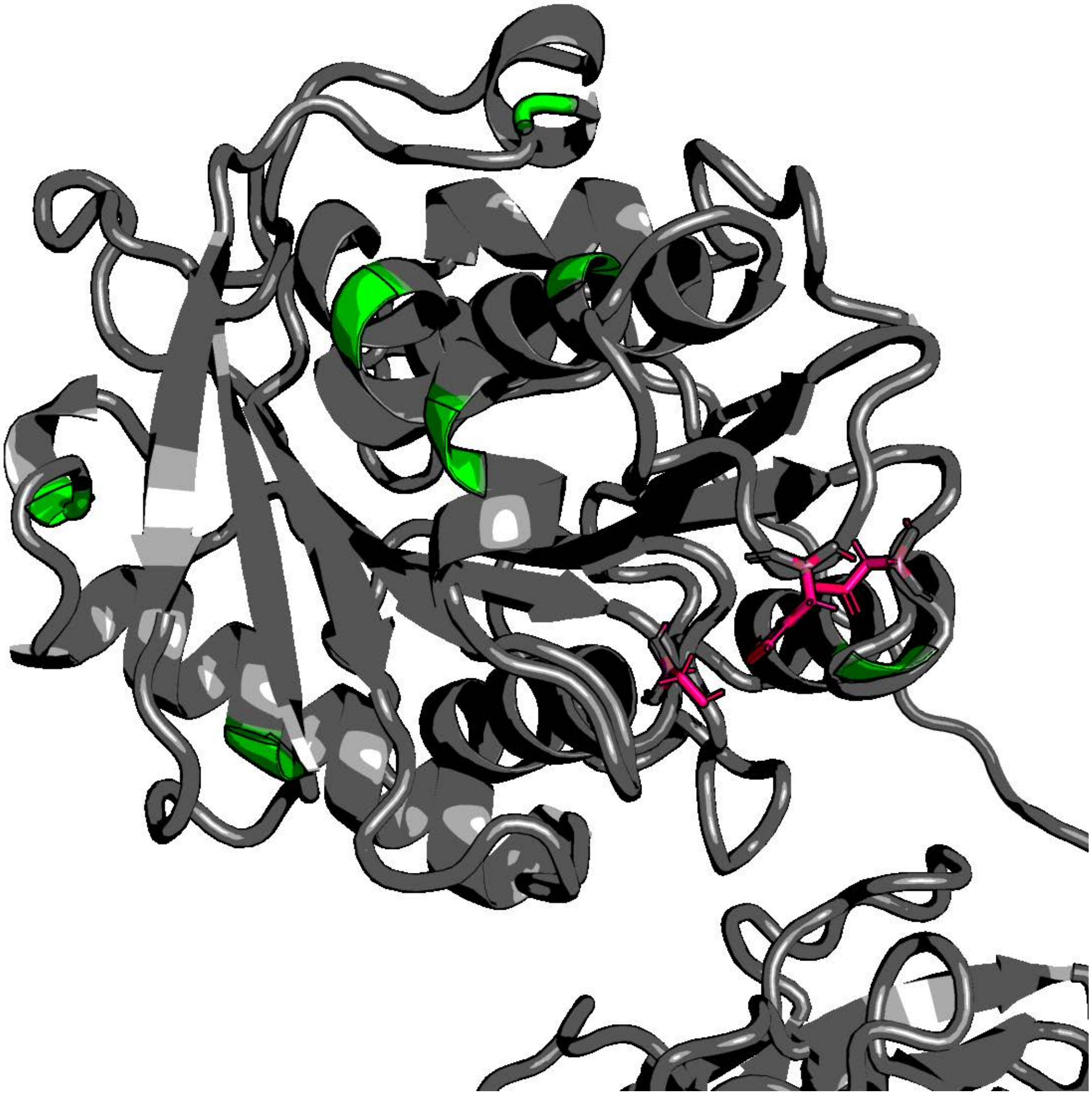

ESM010_highlights.png

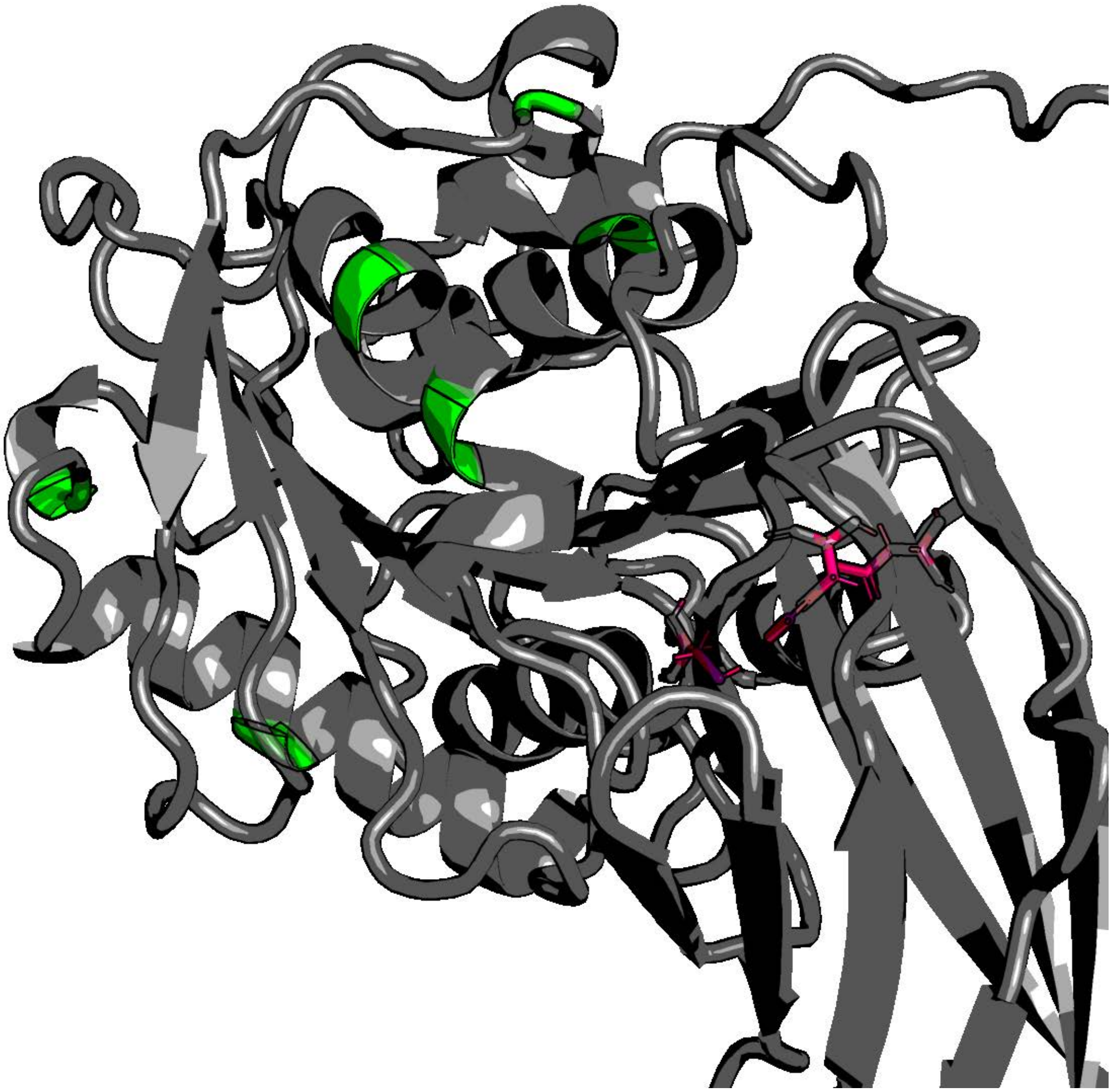

ESM012_highlights.png

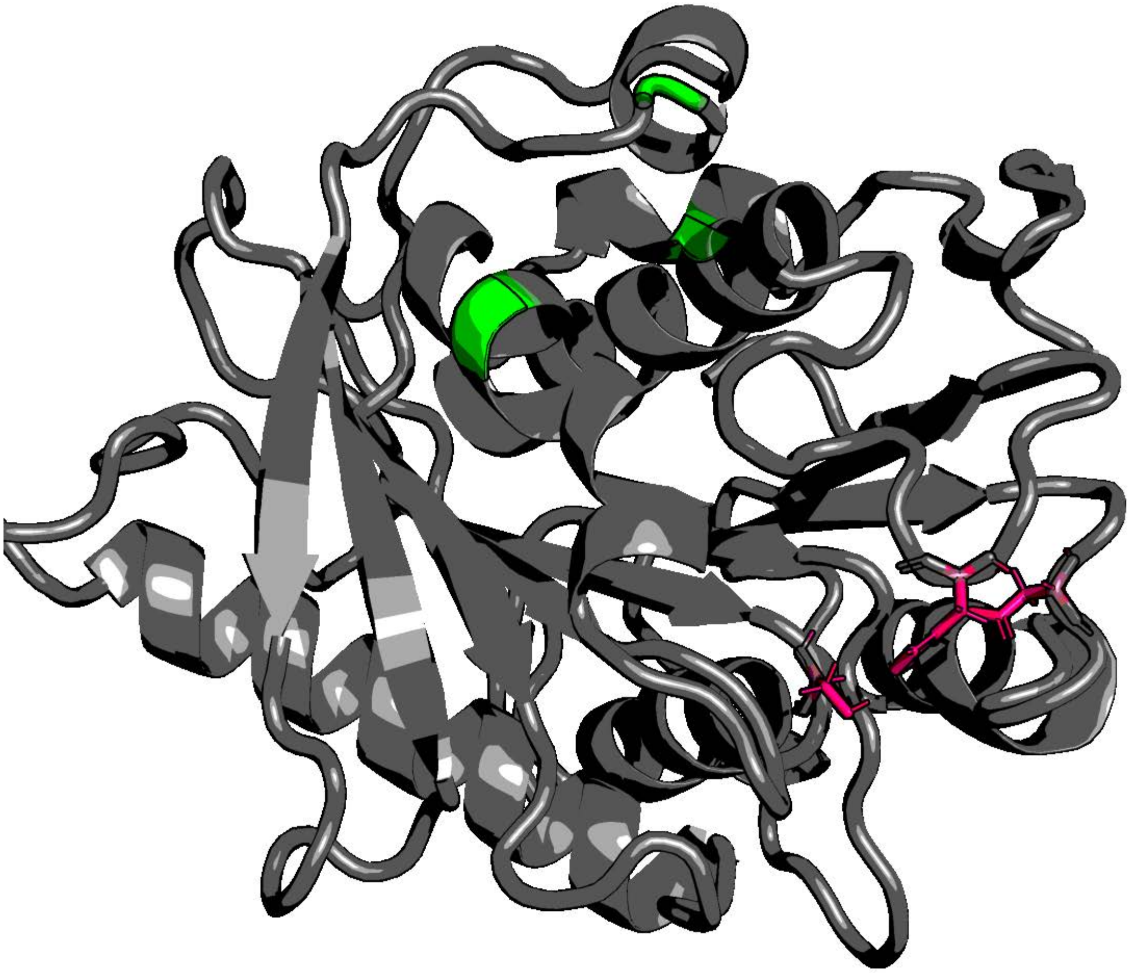

ESM017_highlights.png

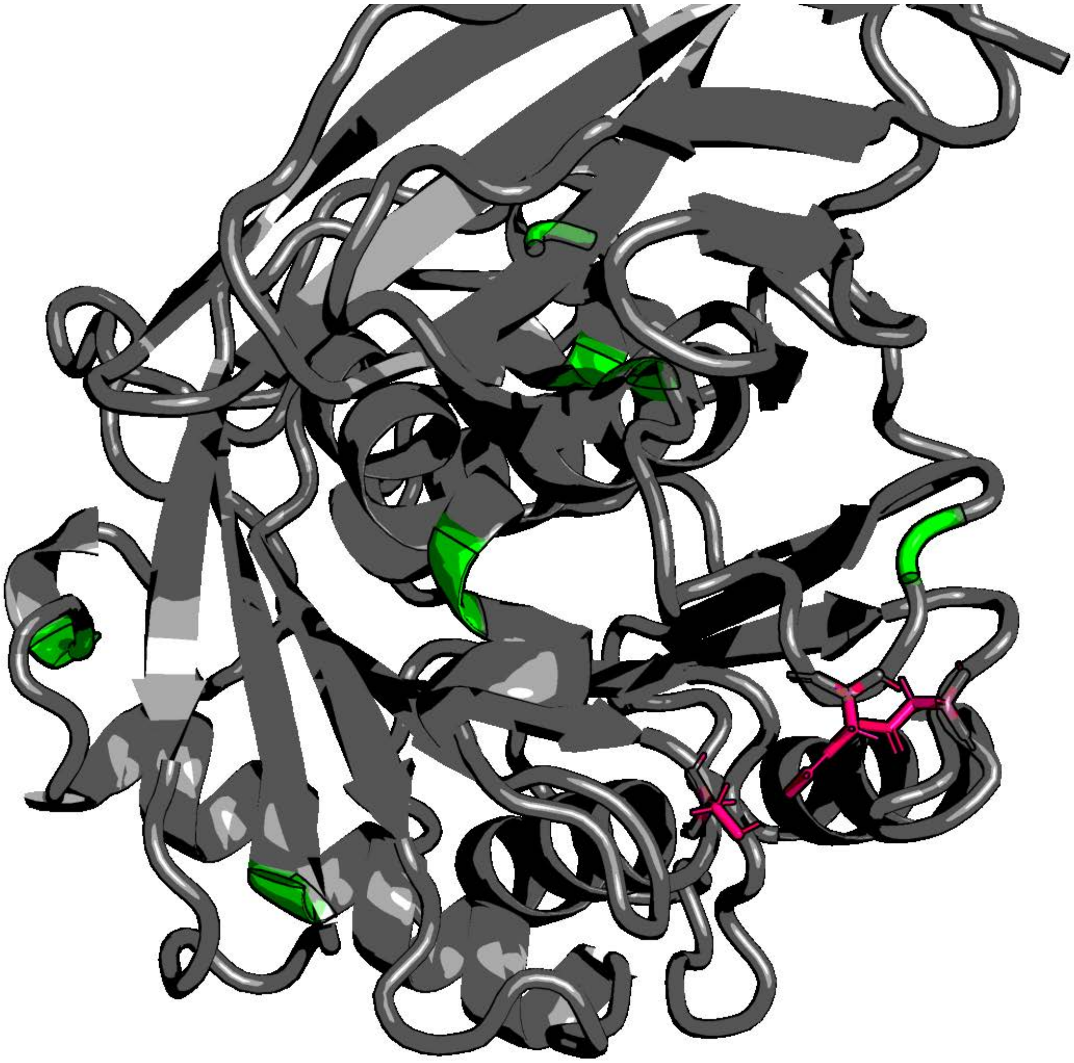

ESM018_highlights.png

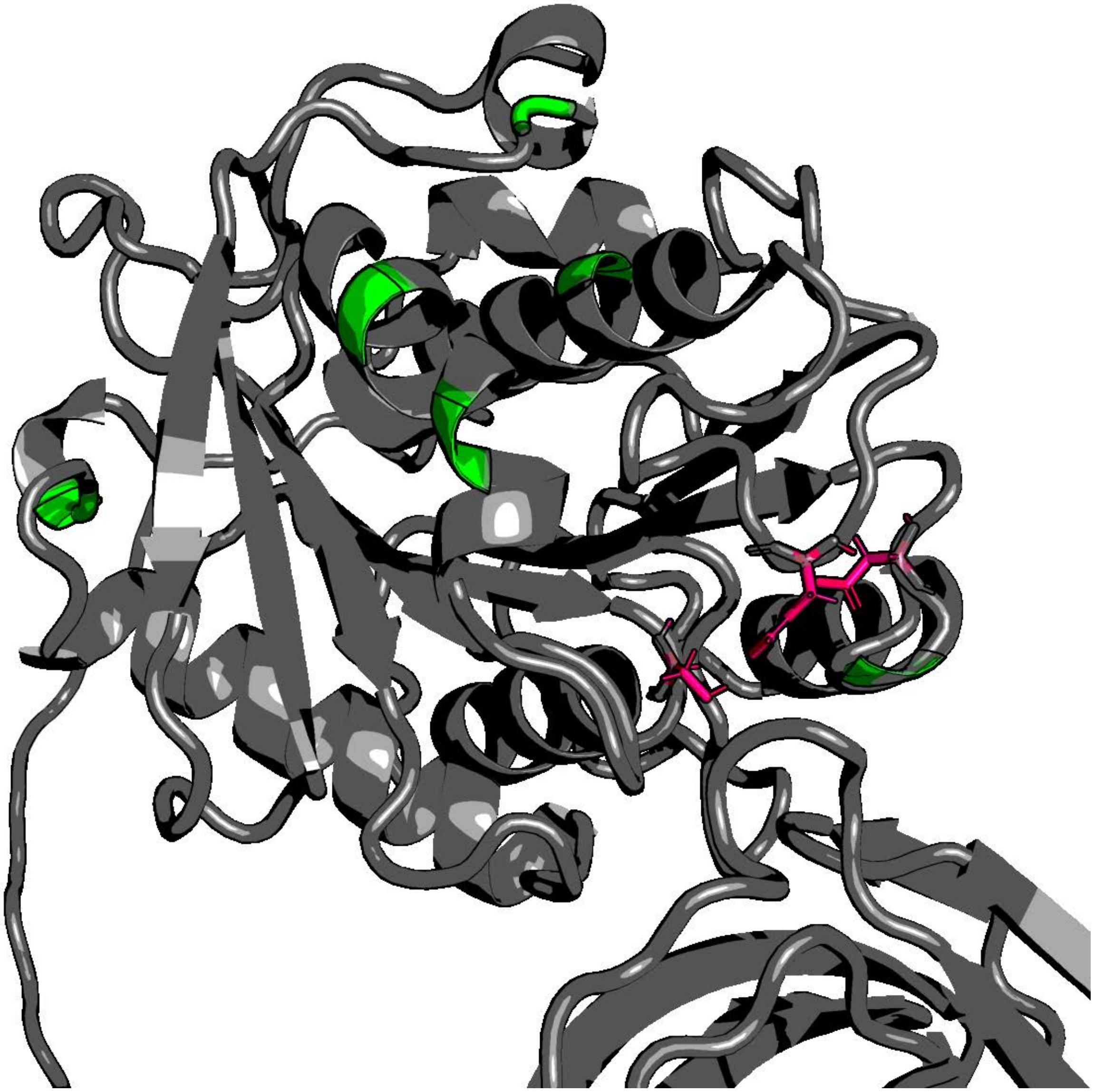

ESM021_highlights.png

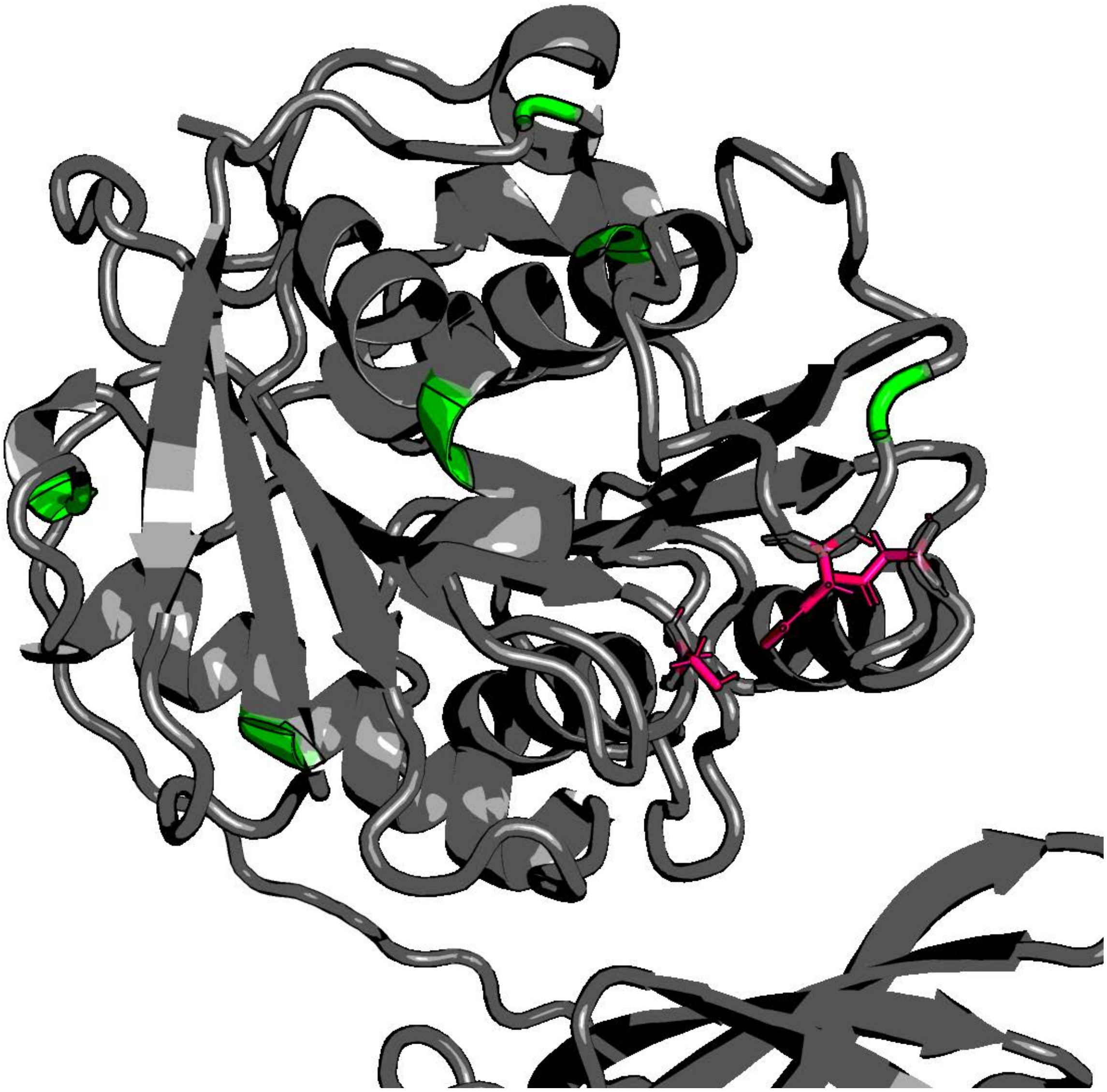

ESM023_highlights.png

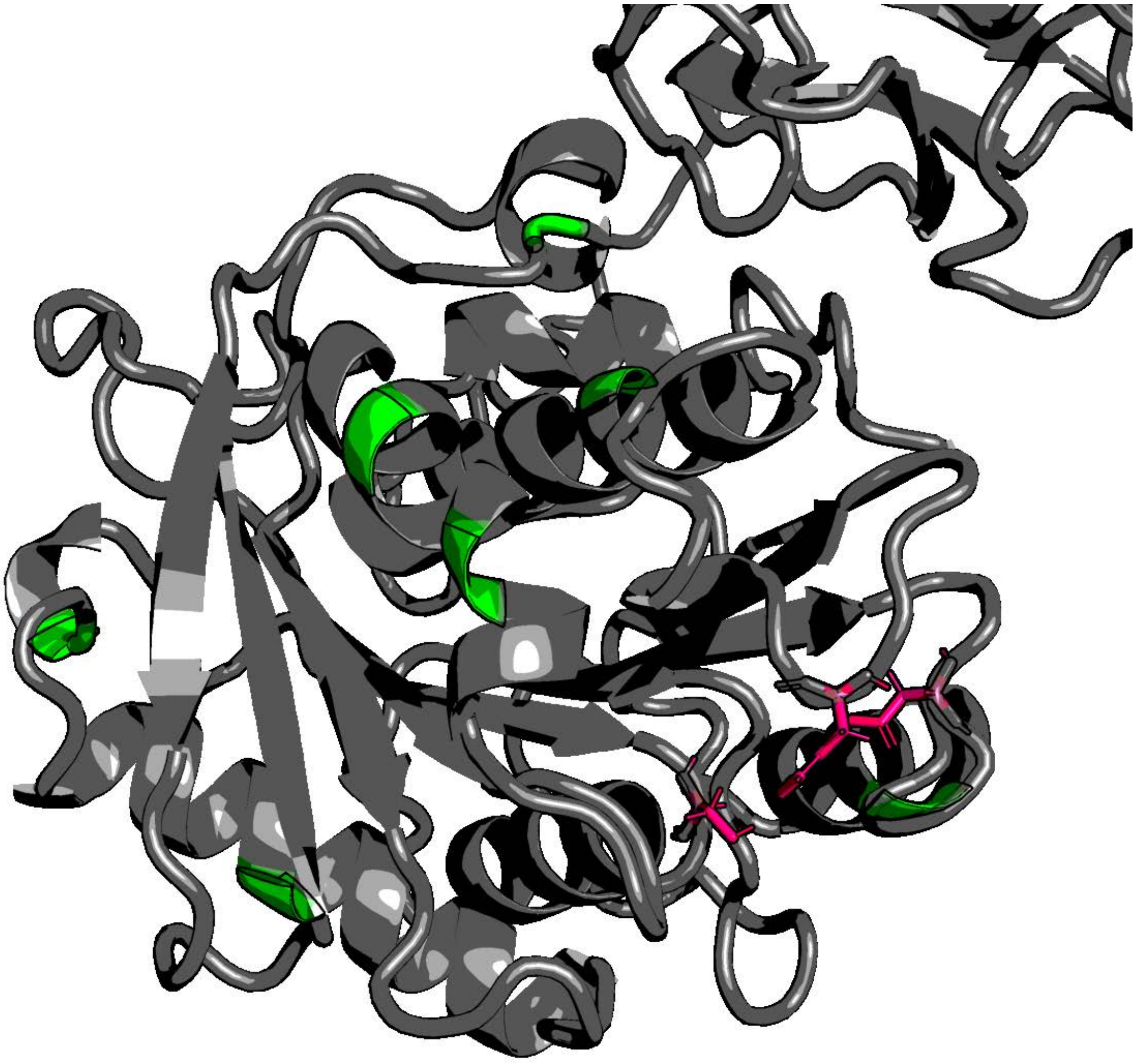

ESM026_highlights.png

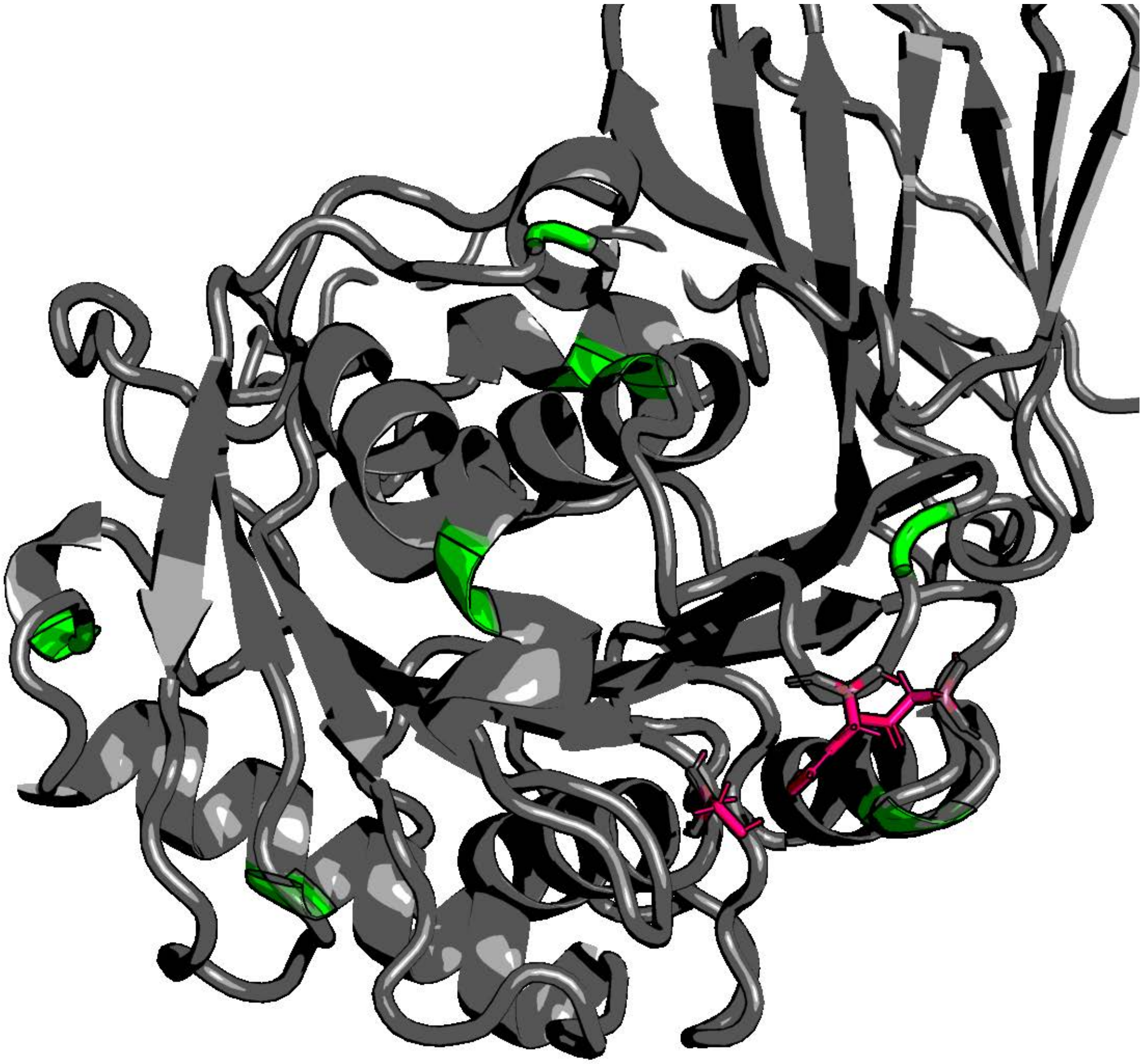

ESM033_highlights.png

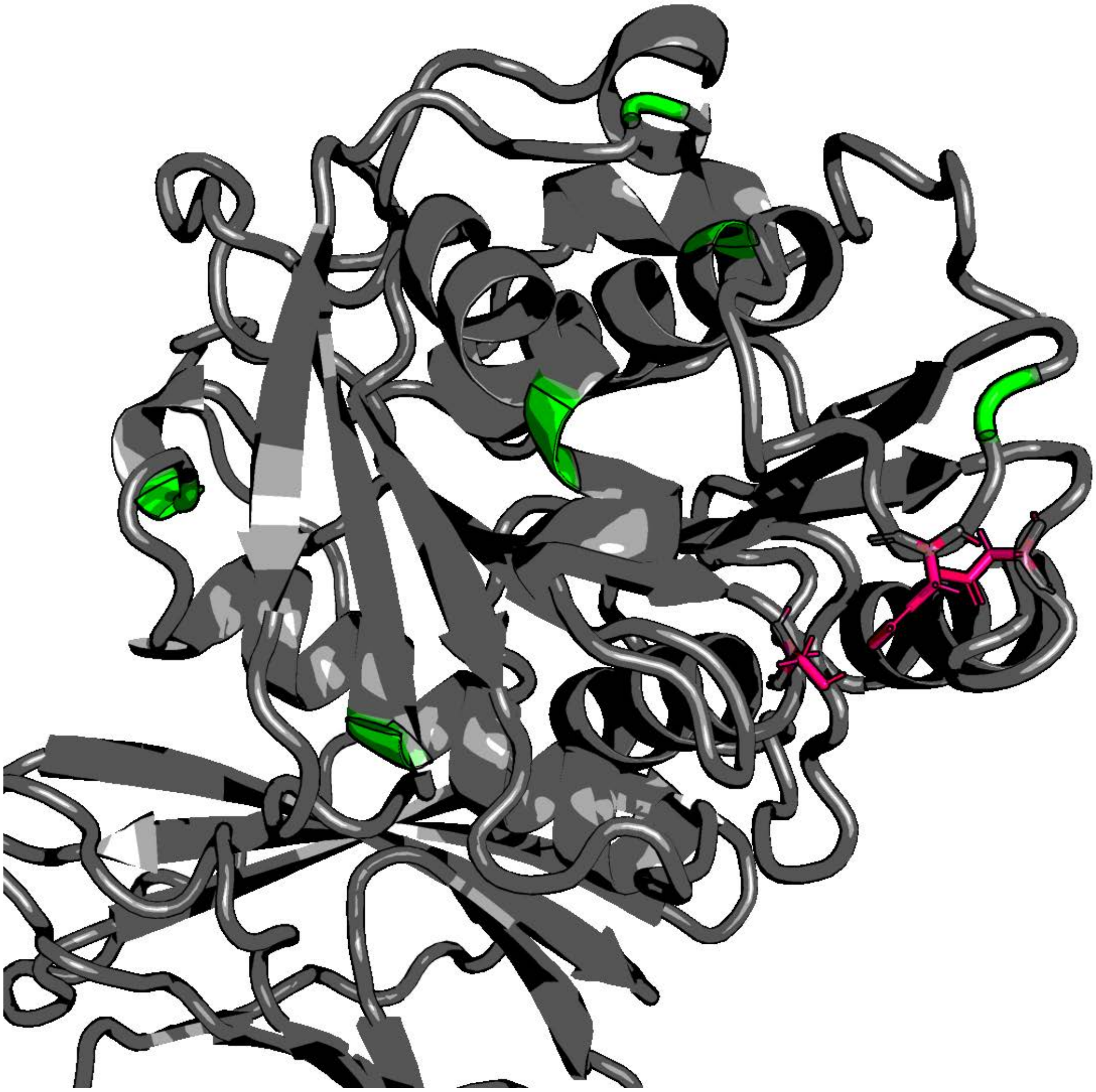

ESM035_highlights.png

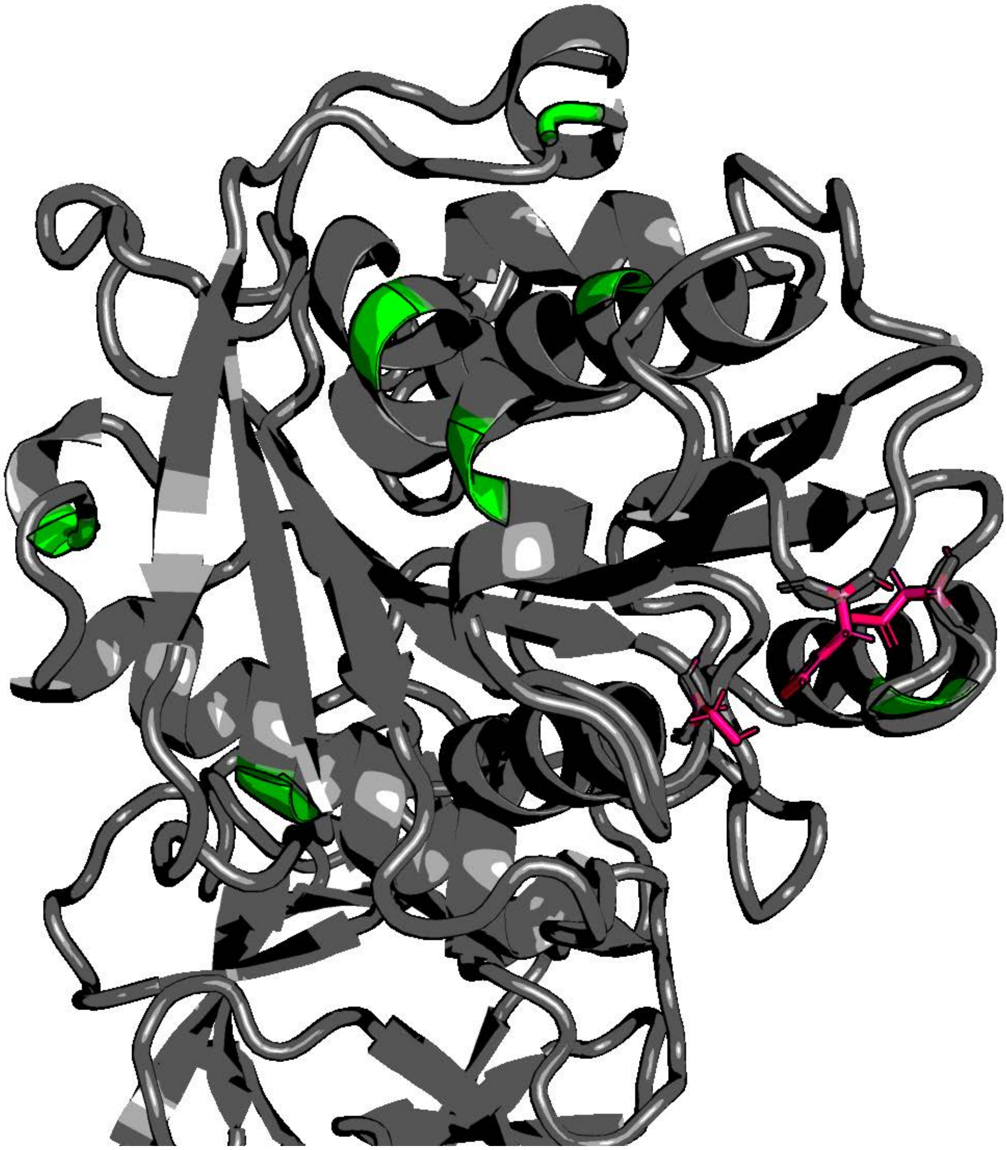

ESM038_highlights.png

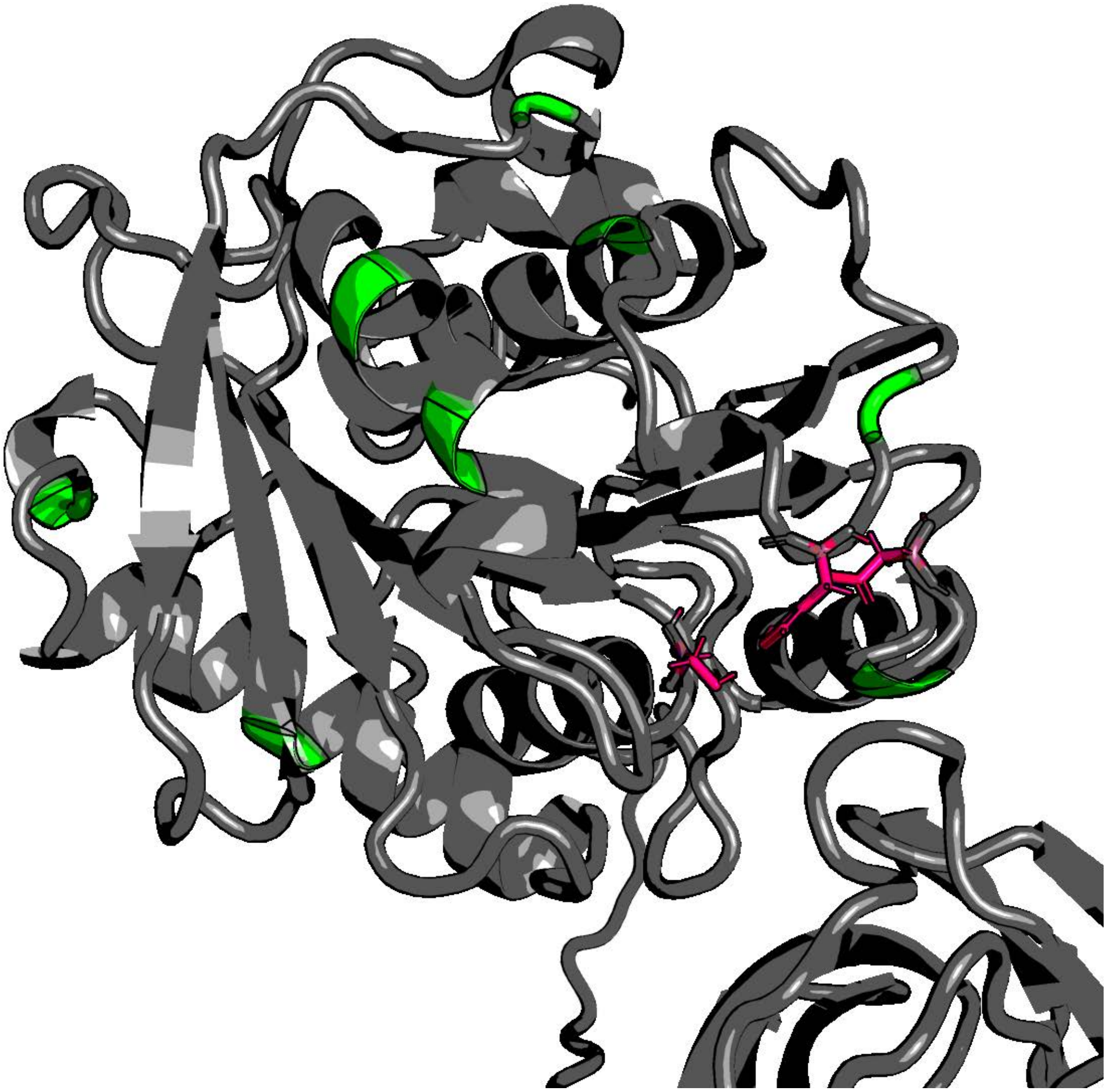

ESM043_highlights.png

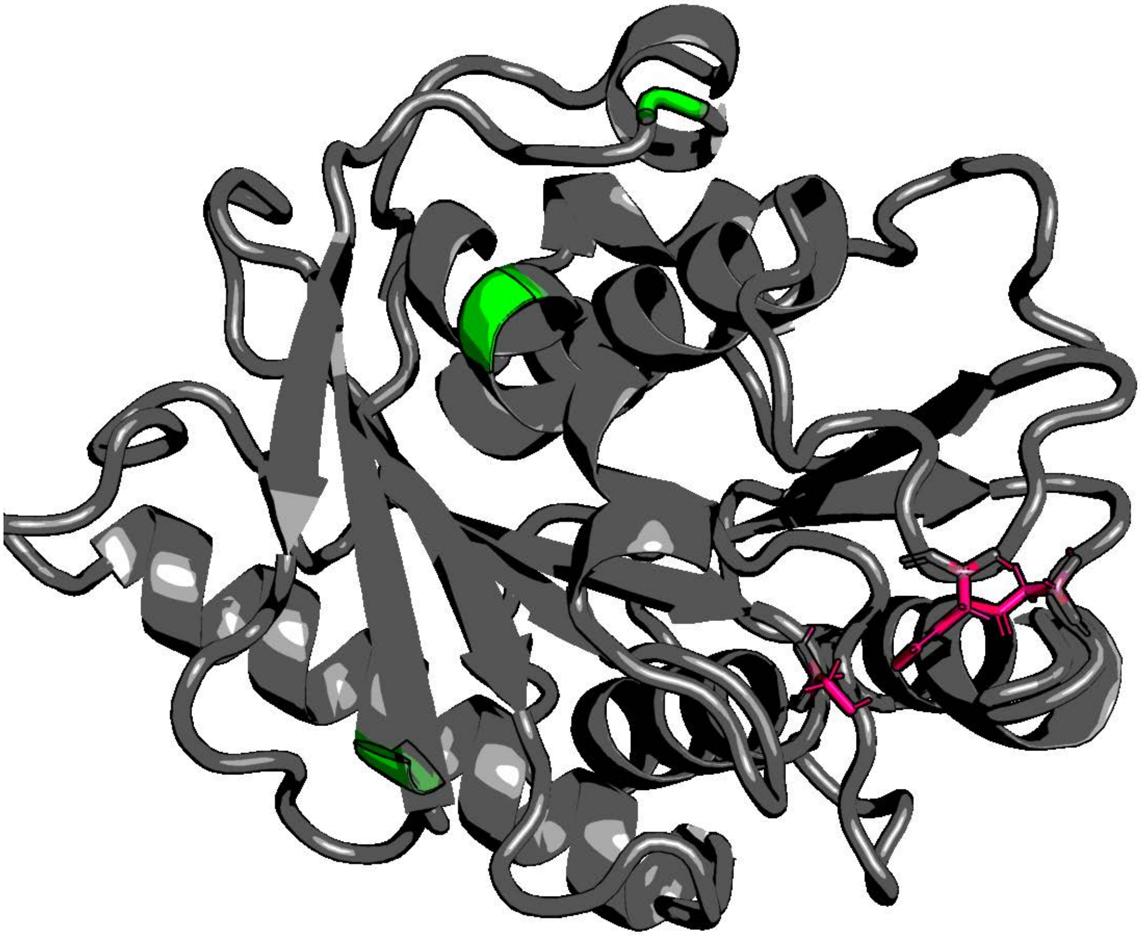

ESM045_highlights.png

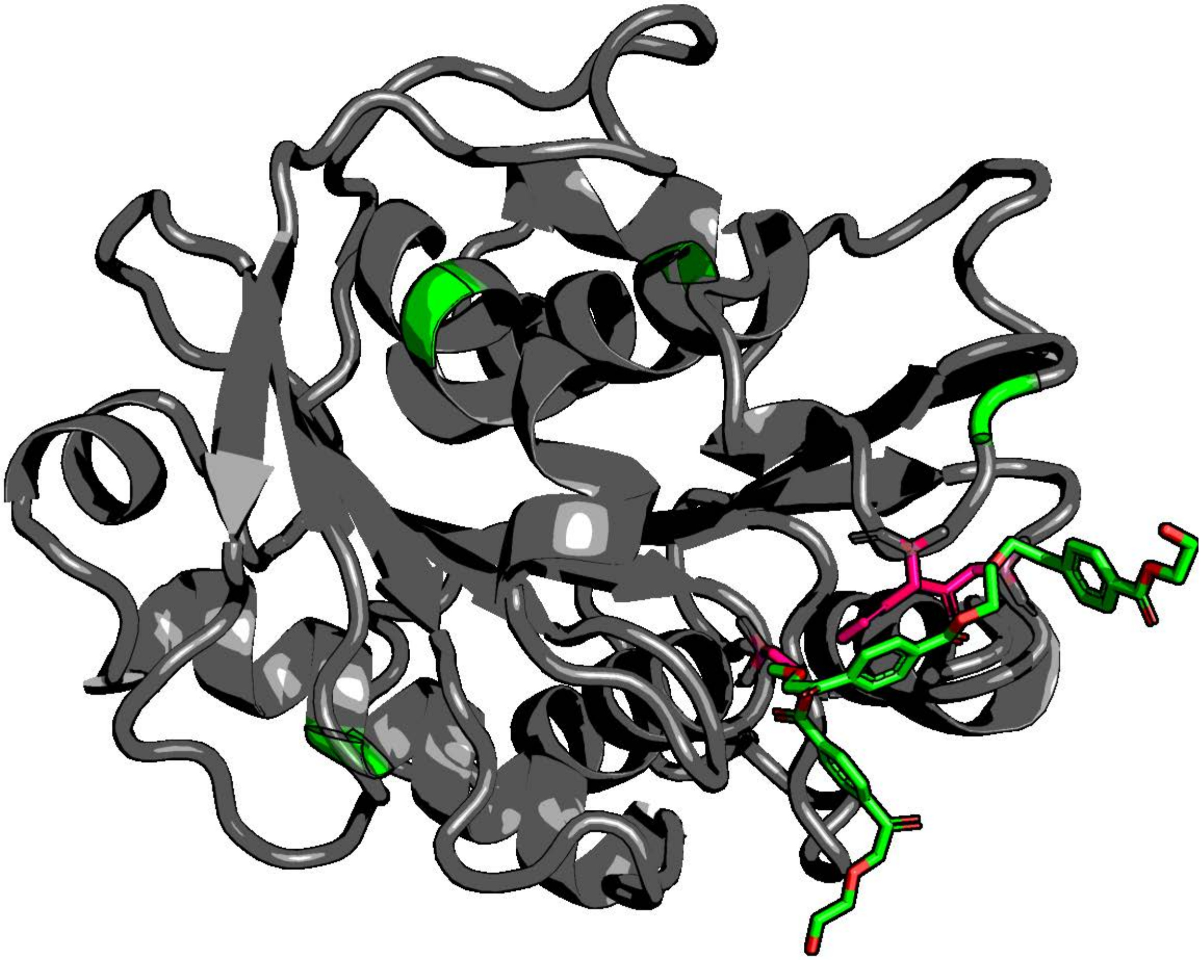

ESM047_highlights.png

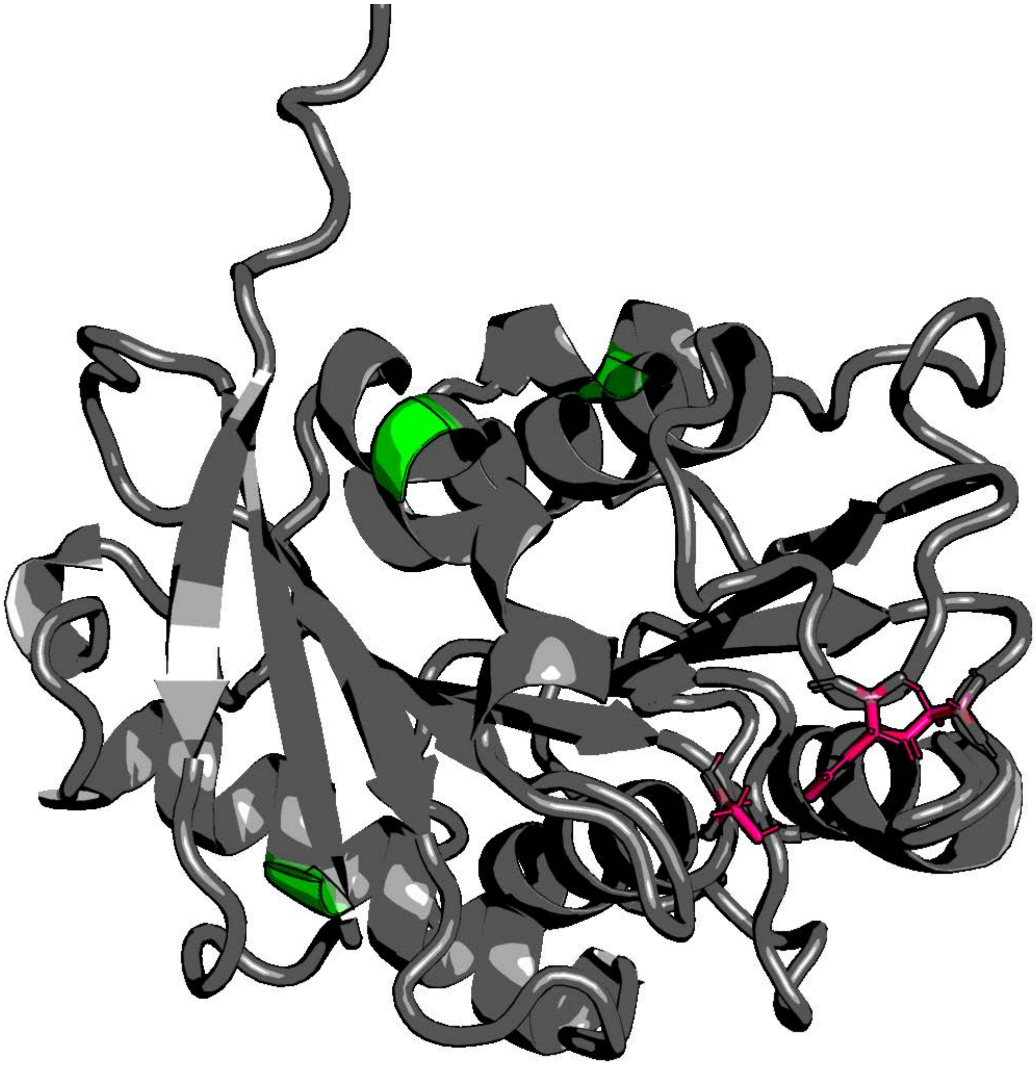

ESM050_highlights.png

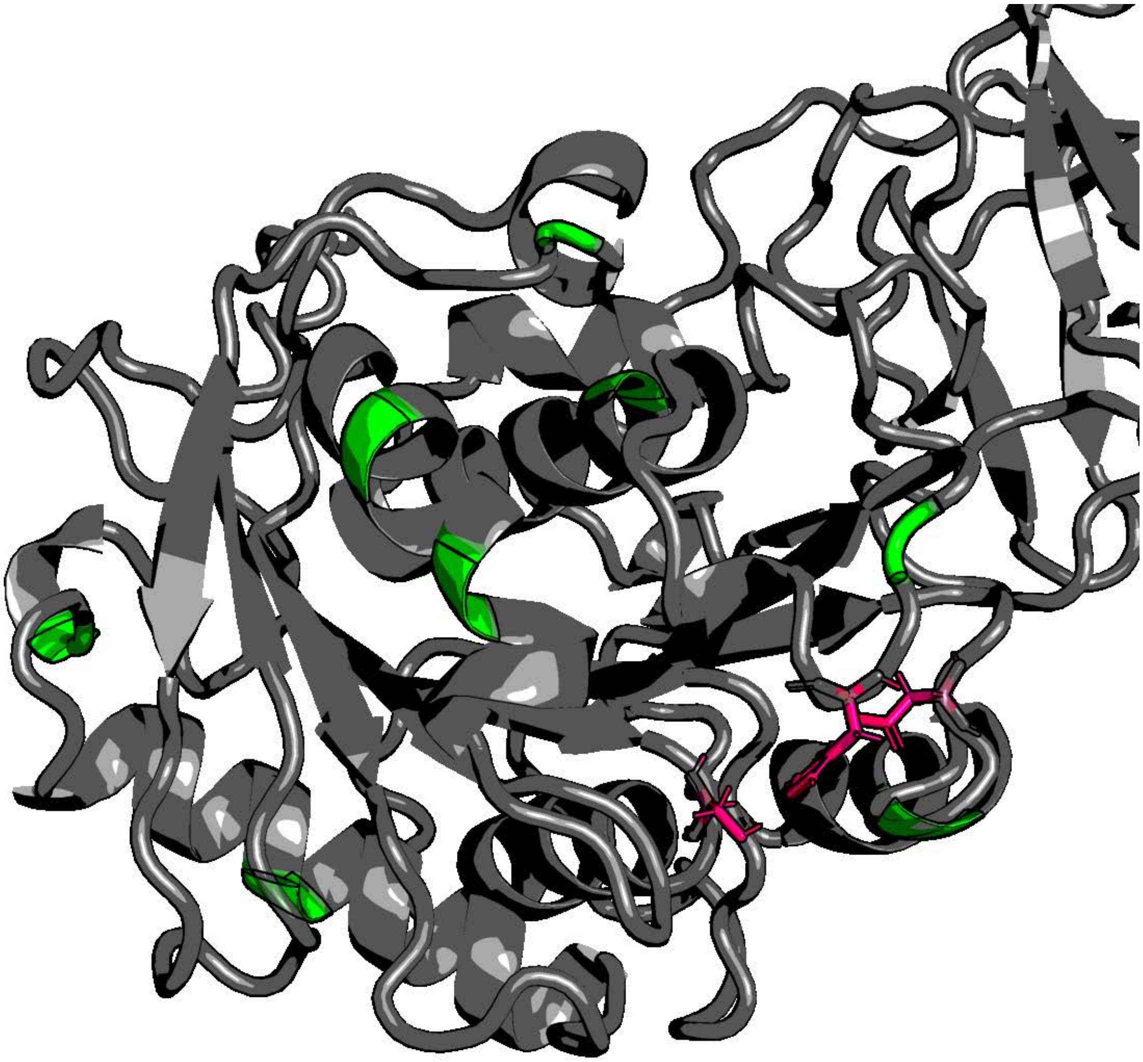

ESM052_highlights.png

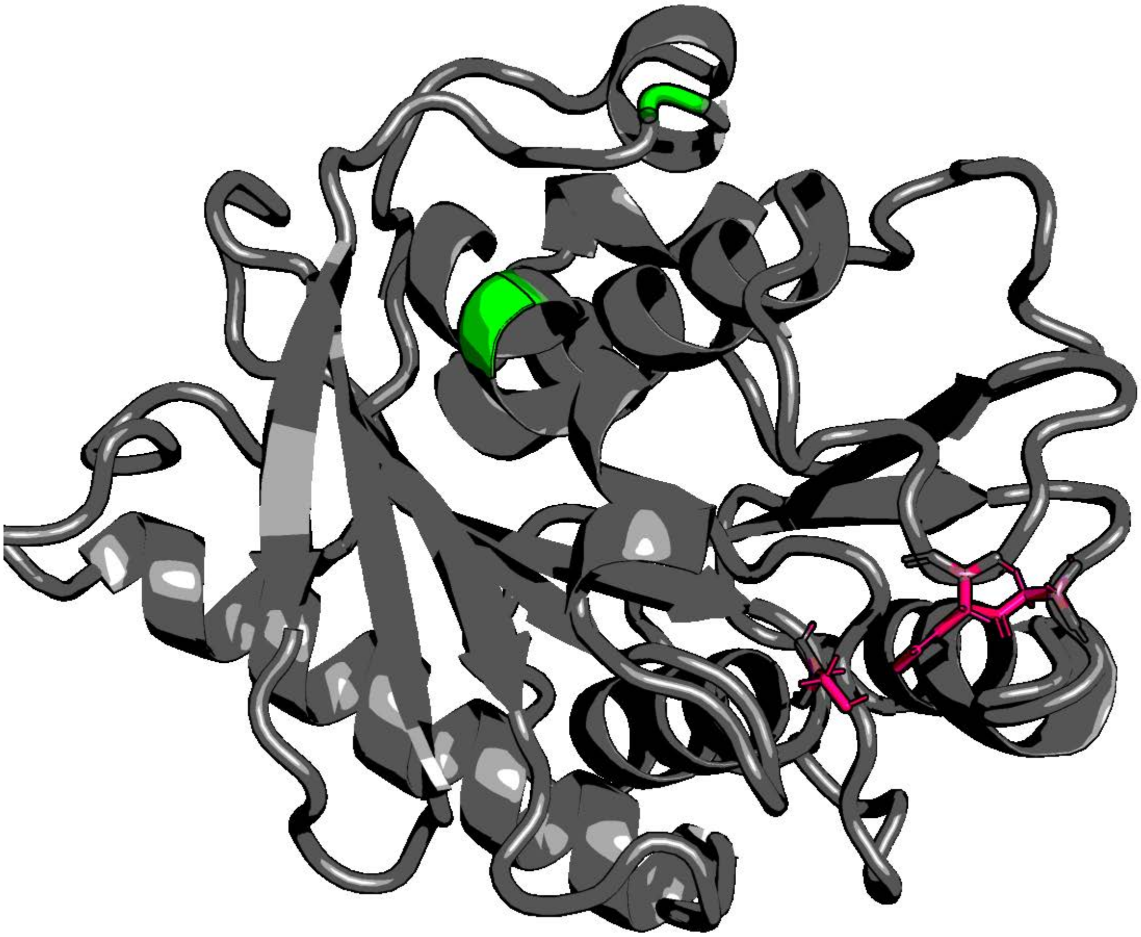

ESM053_highlights.png

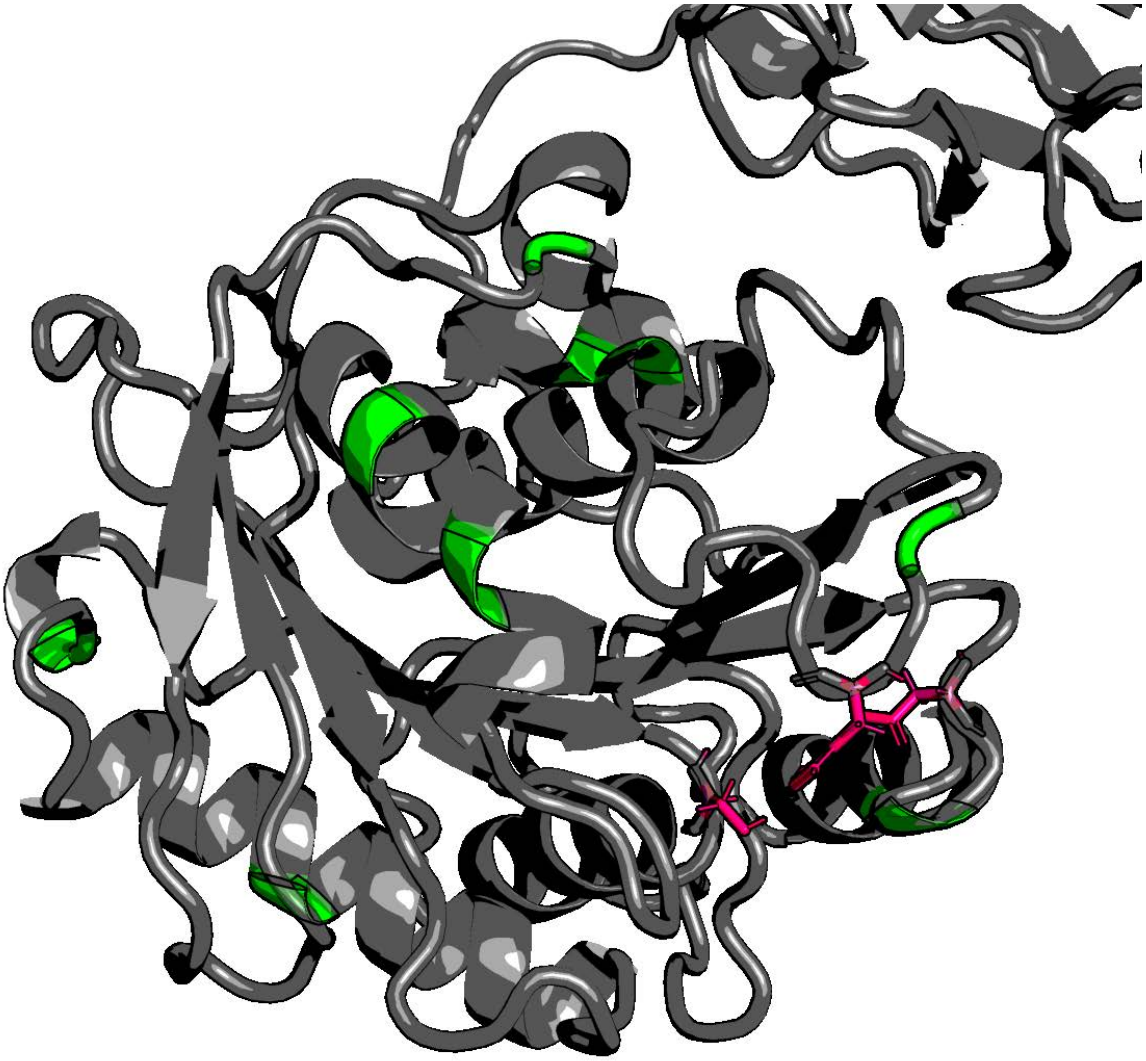

ESM058_highlights.png

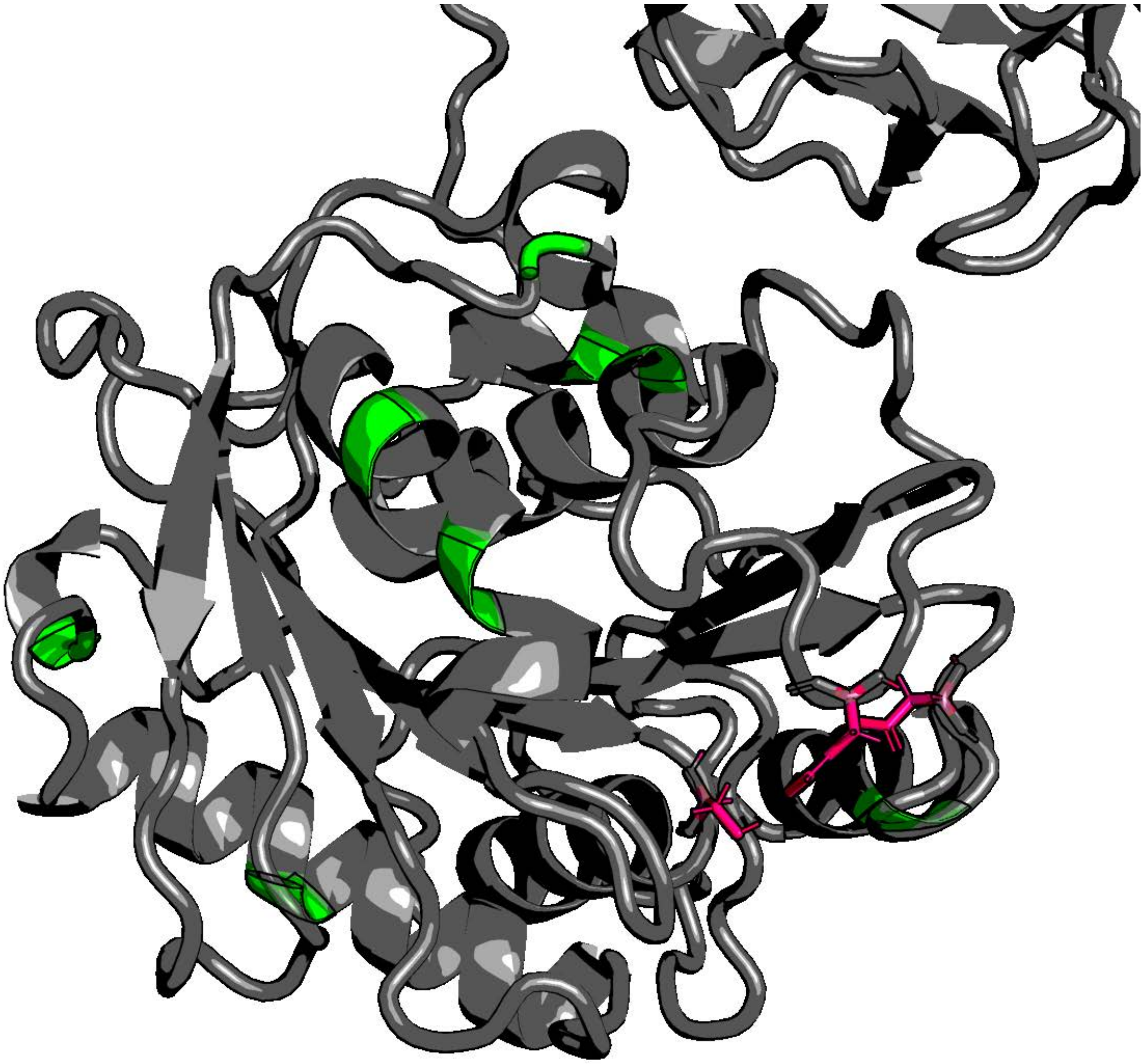

ESM059_highlights.png

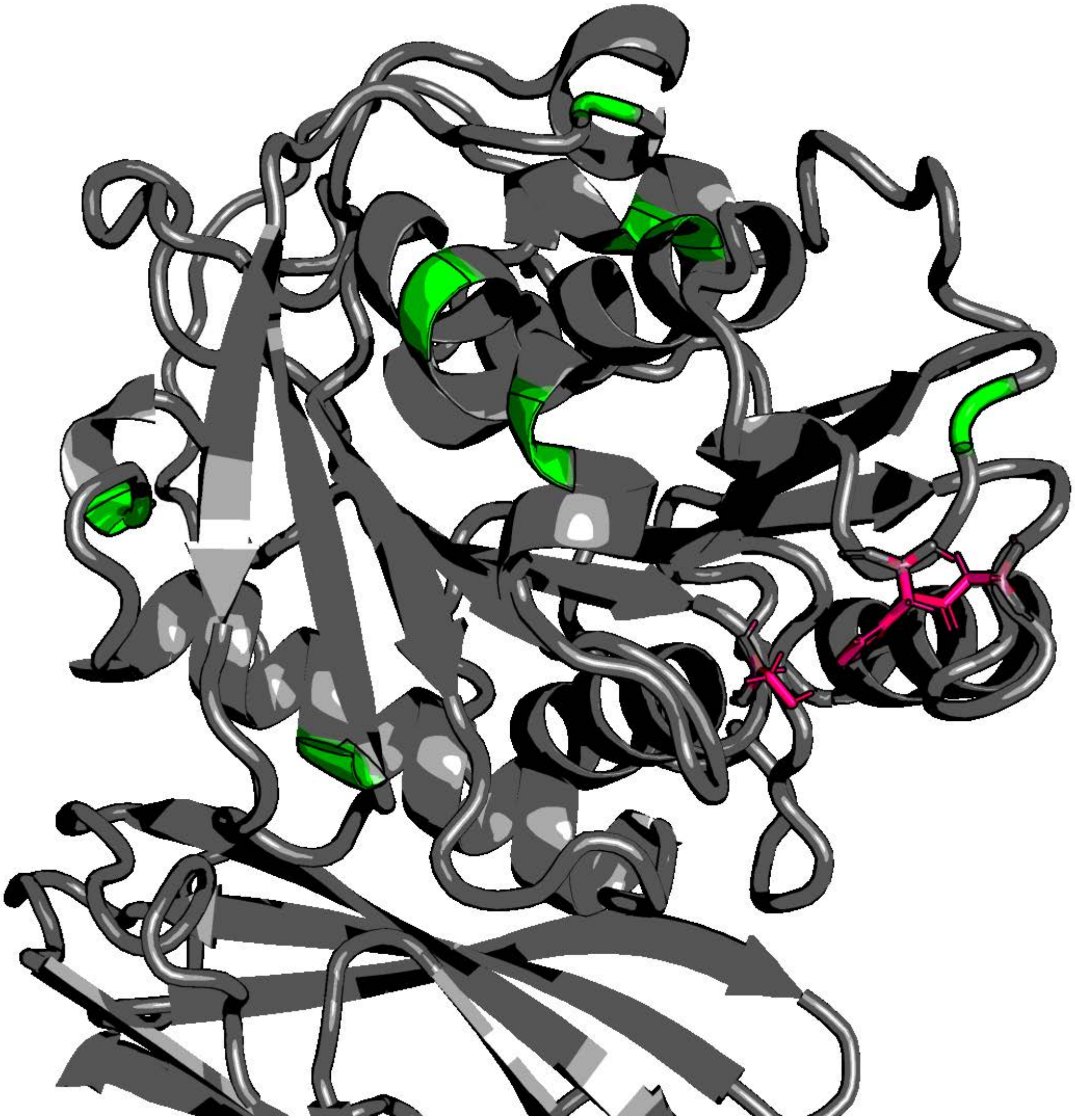

ESM060_highlights.png

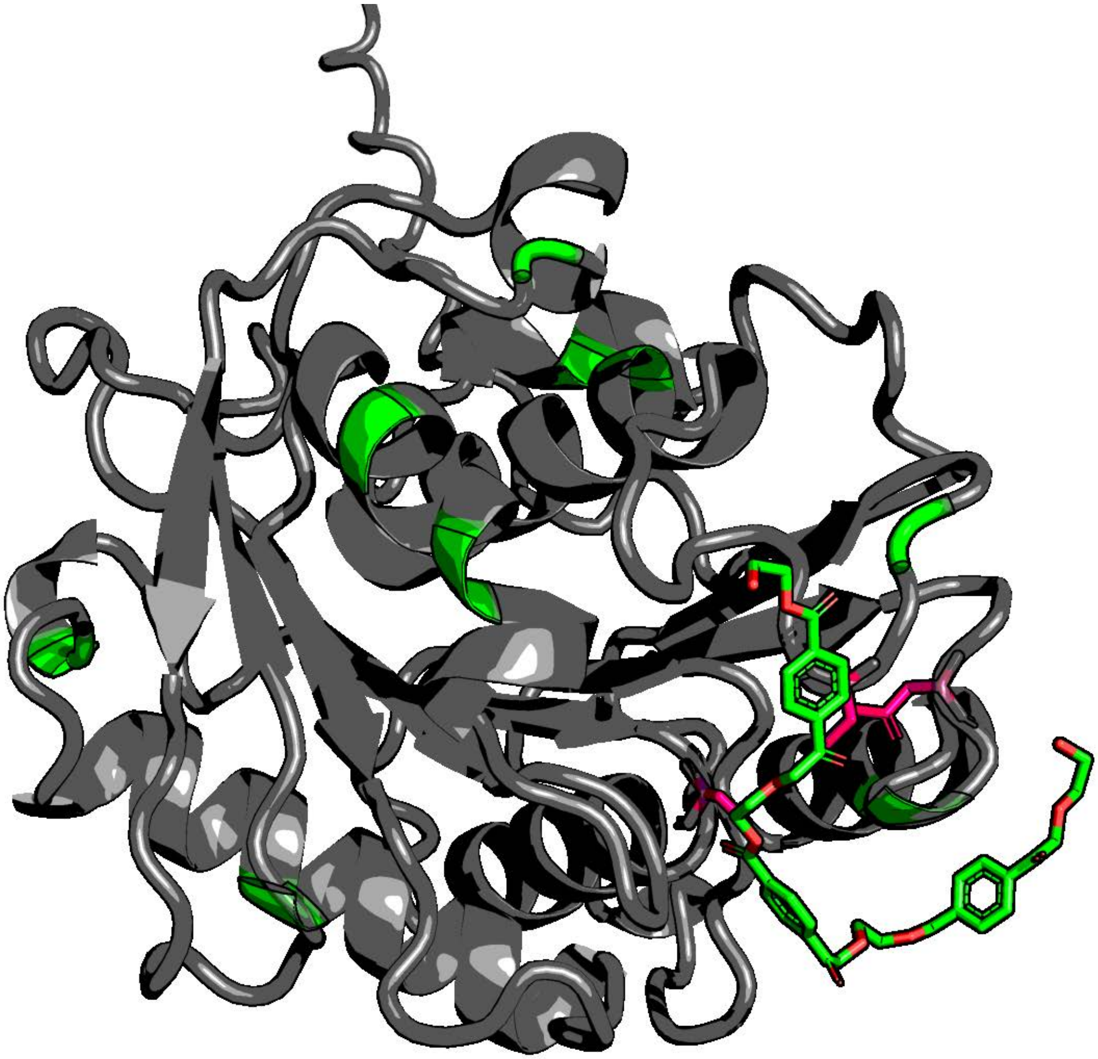

ESM061_highlights.png

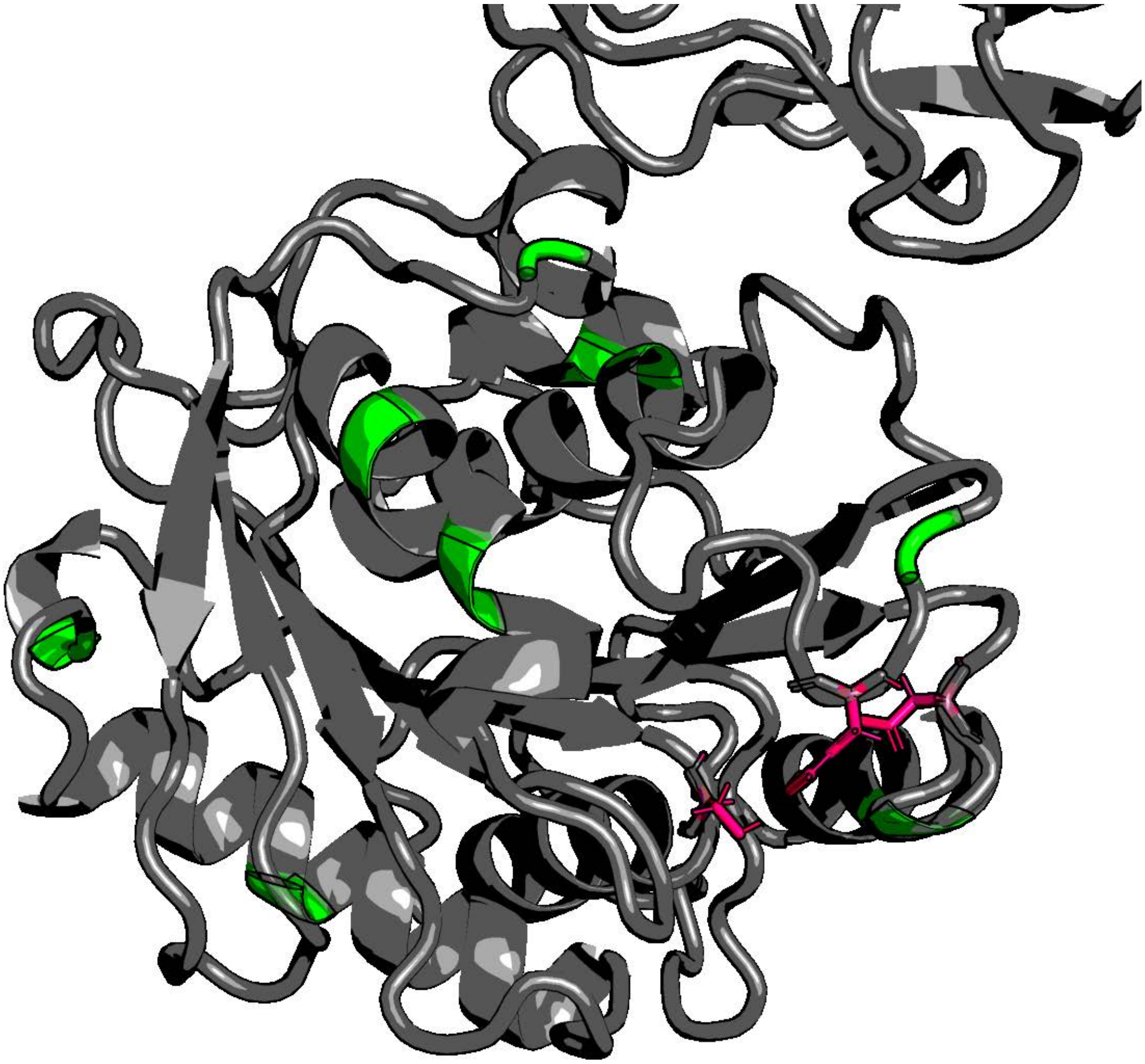

ESM062_highlights.png

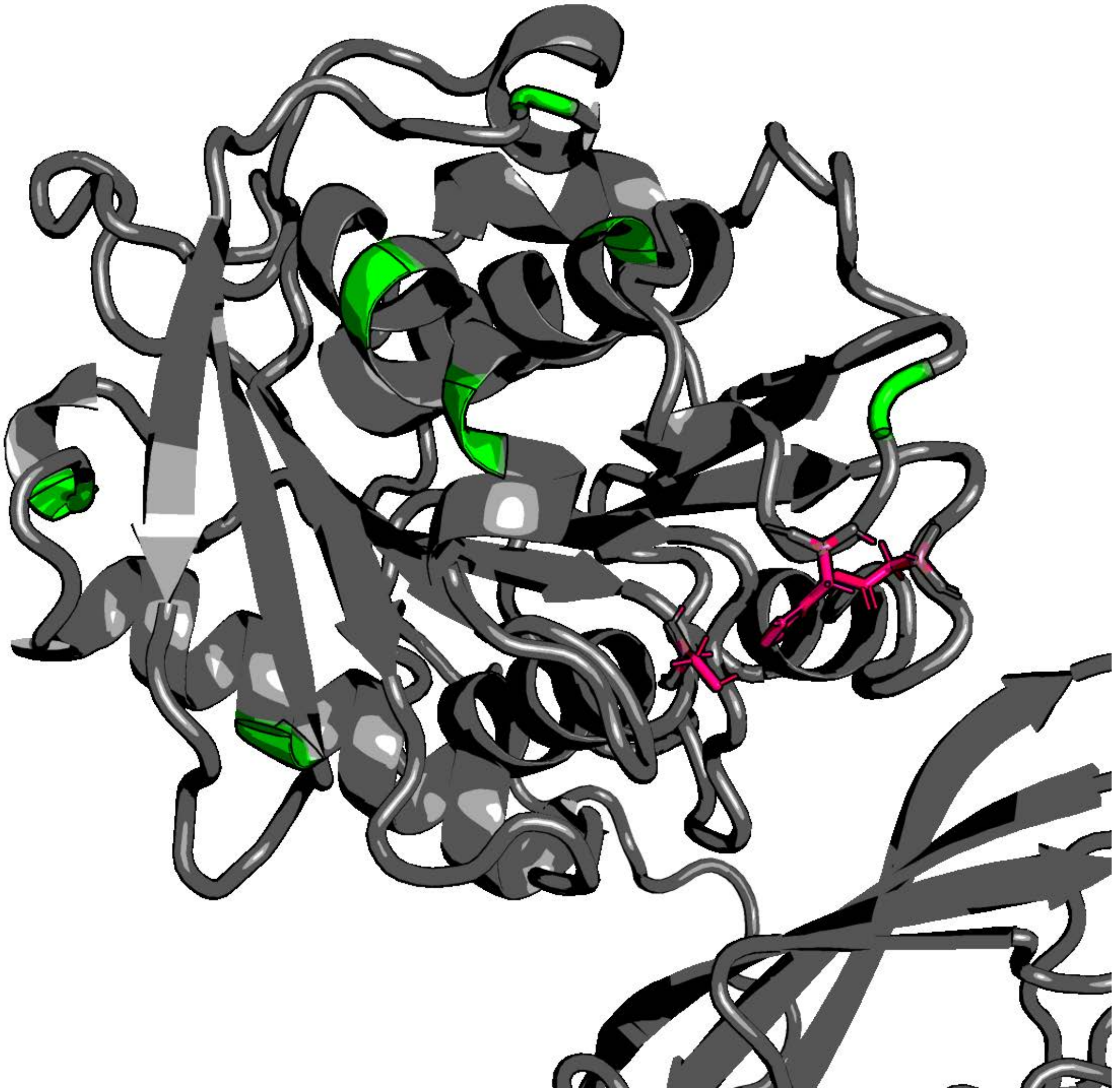

ESM064_highlights.png

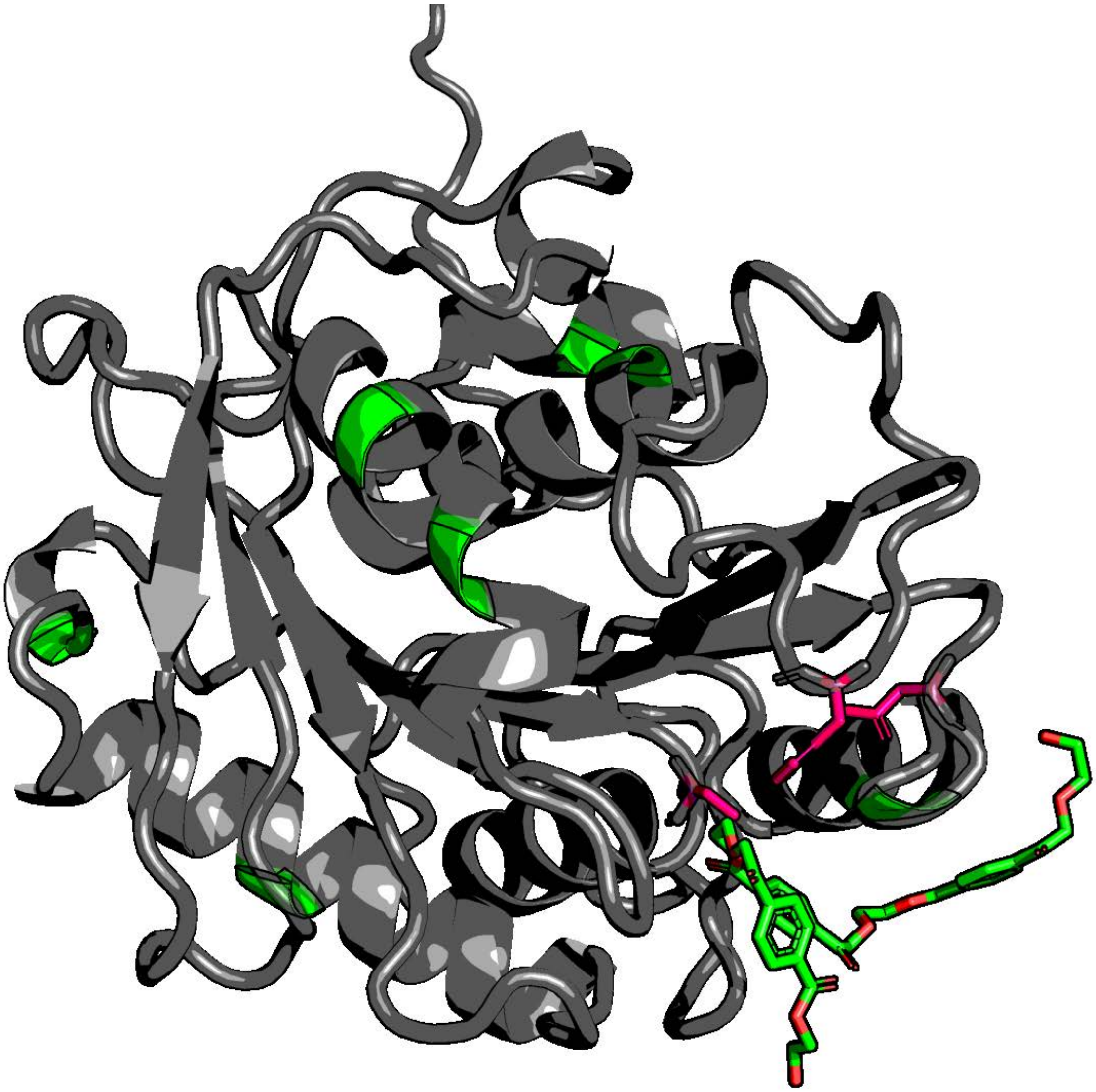

ESM065_highlights.png

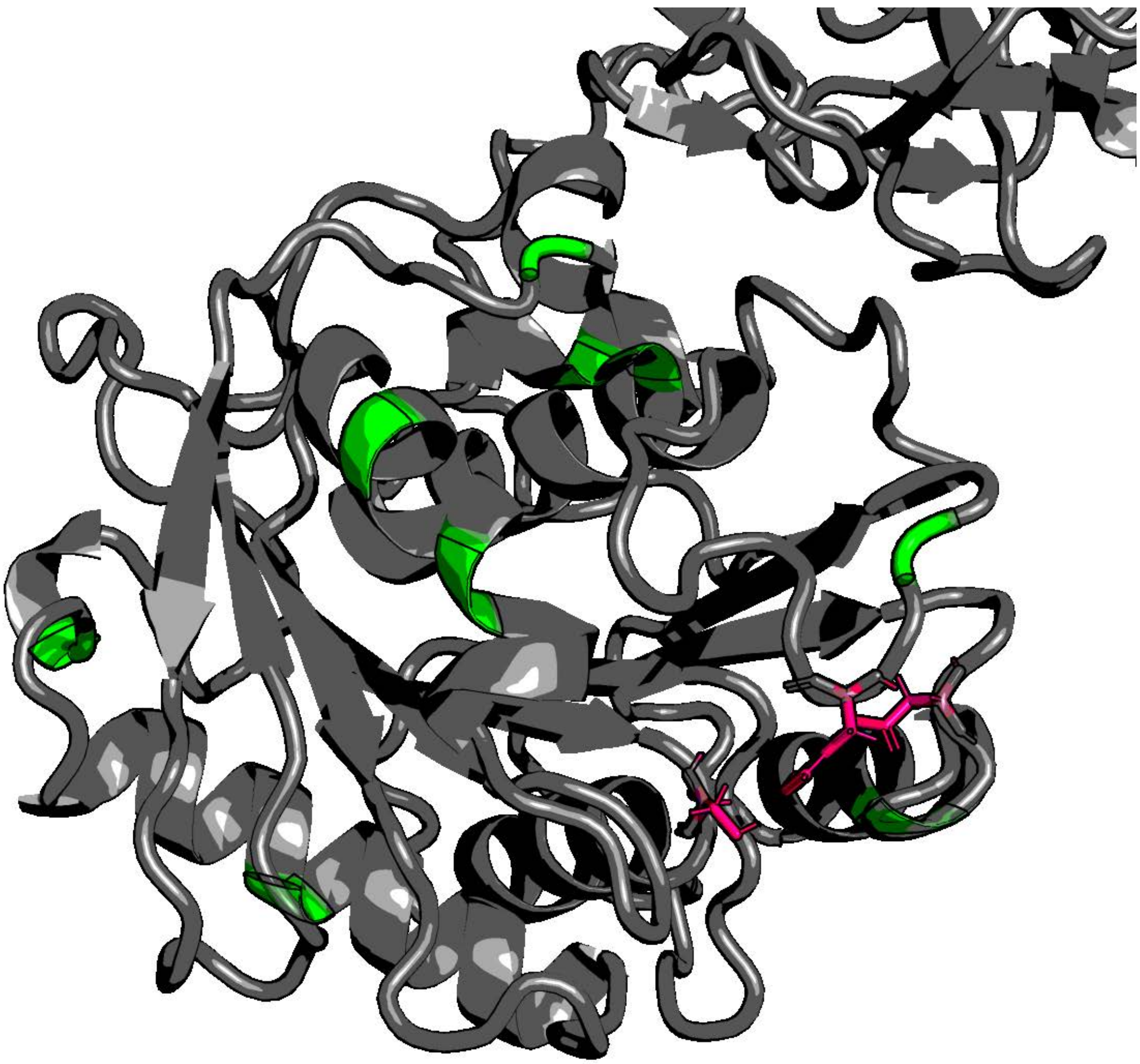

ESM069_highlights.png

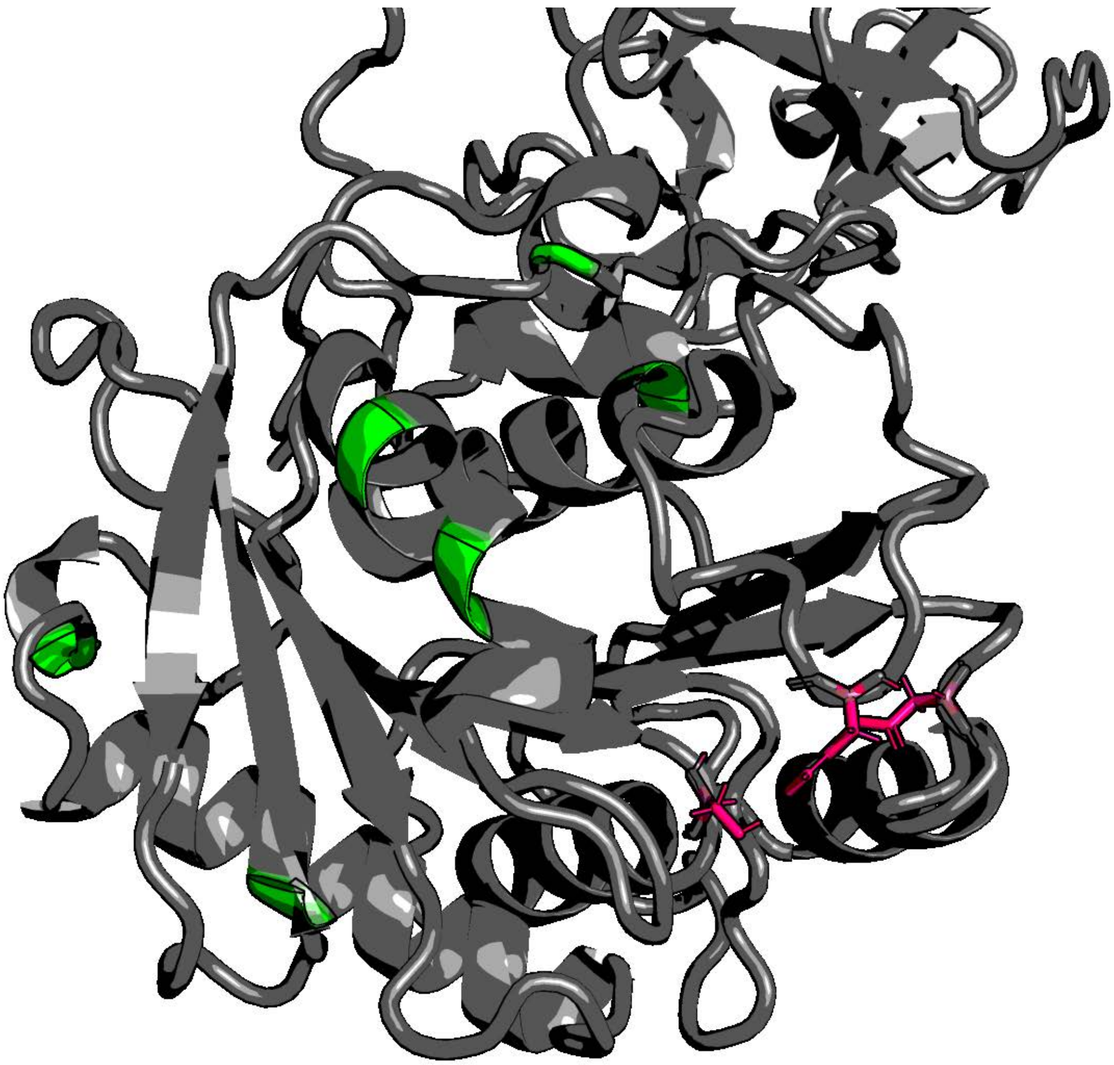

ESM070_highlights.png

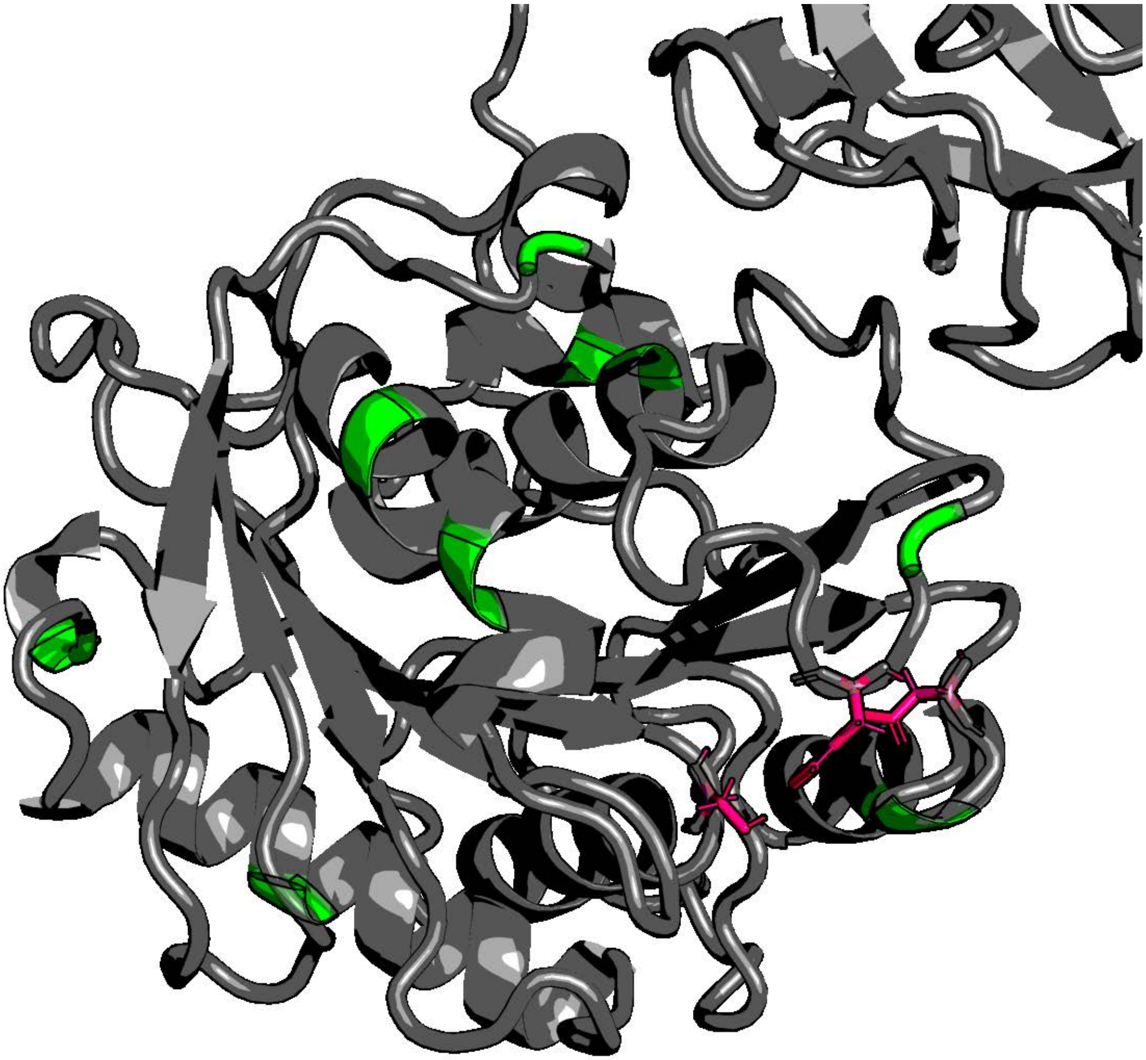

ESM075_highlights.png

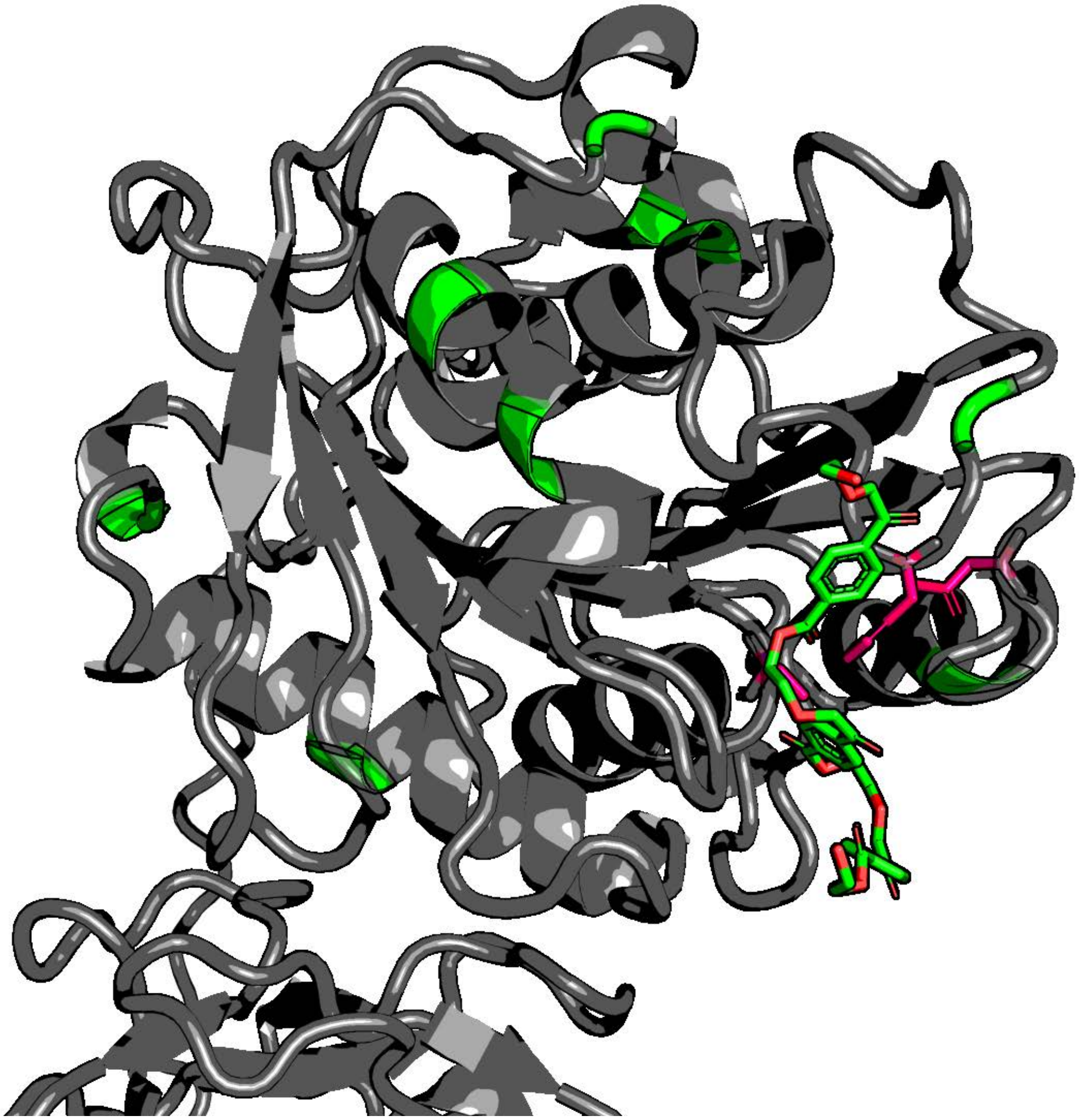

ESM080_highlights.png

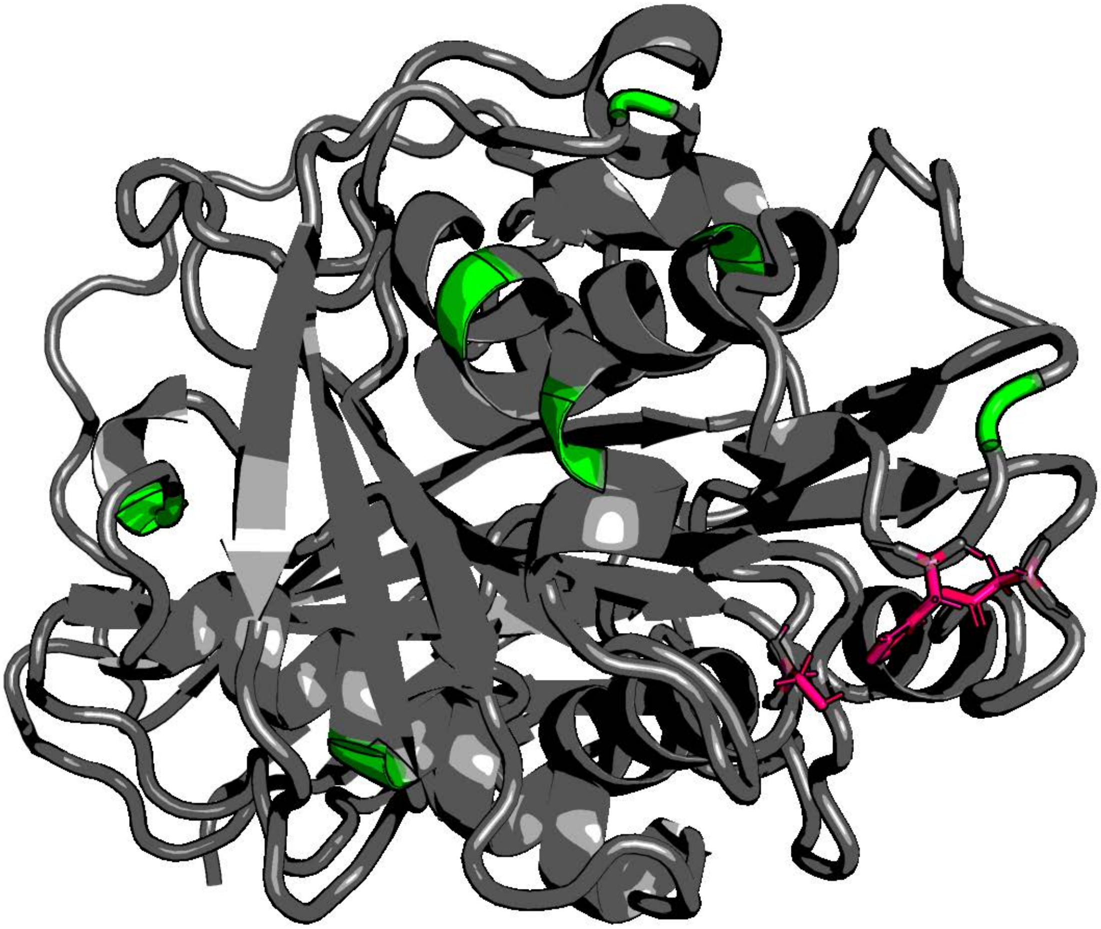

ESM081_highlights.png

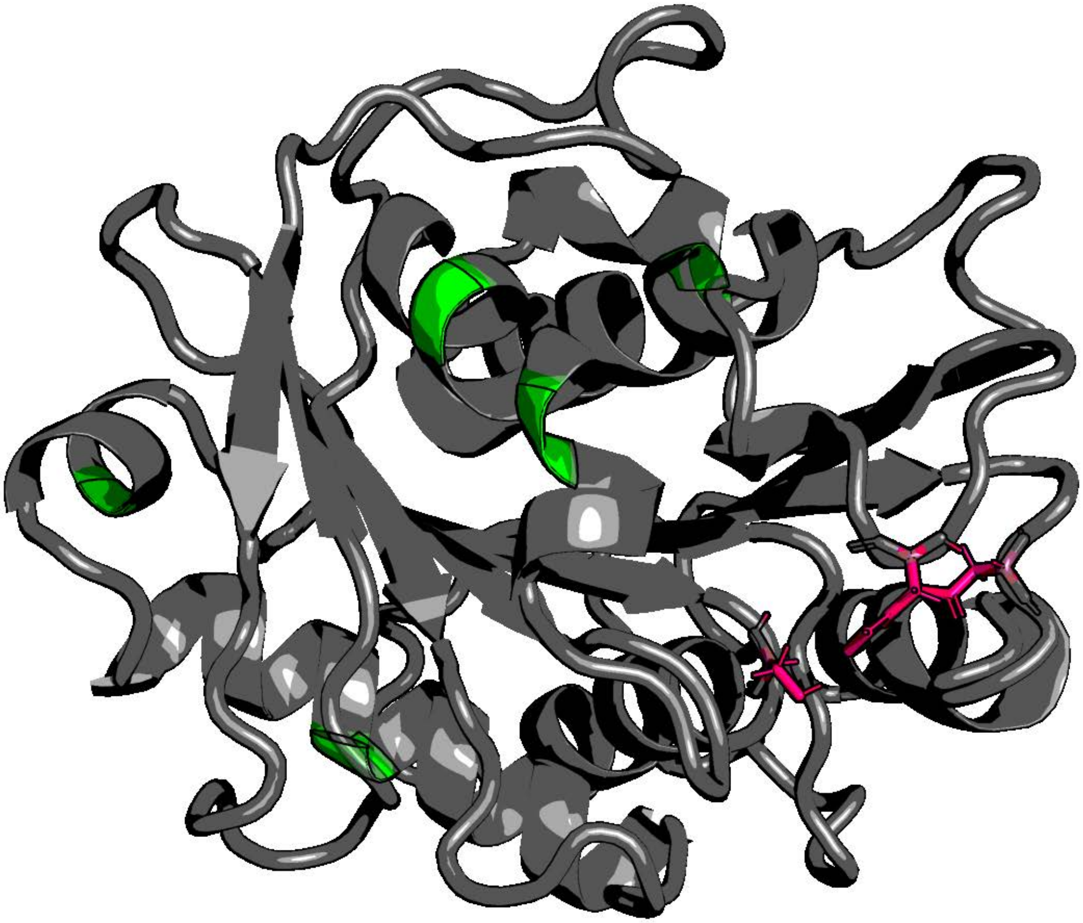

ESM082_highlights.png

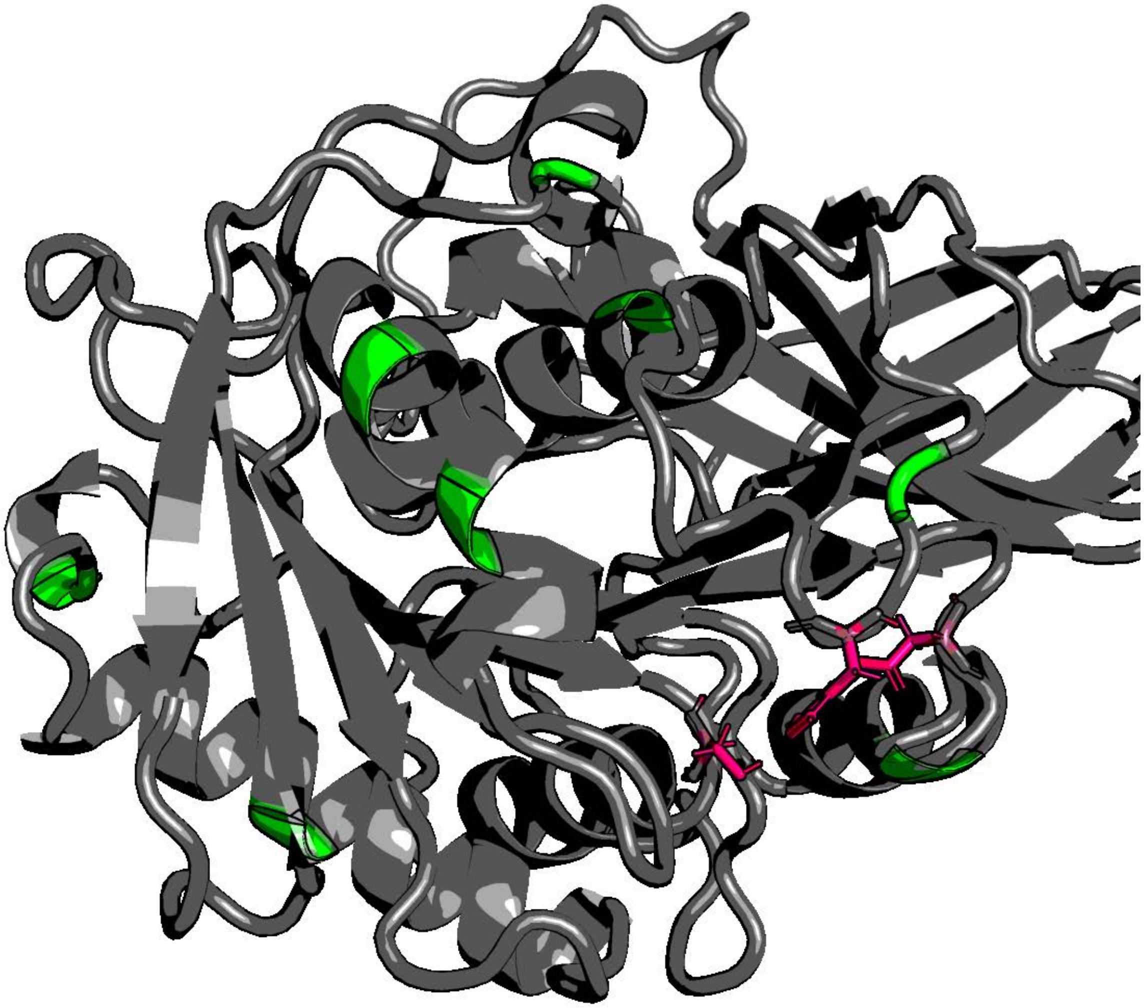

ESM084_highlights.png

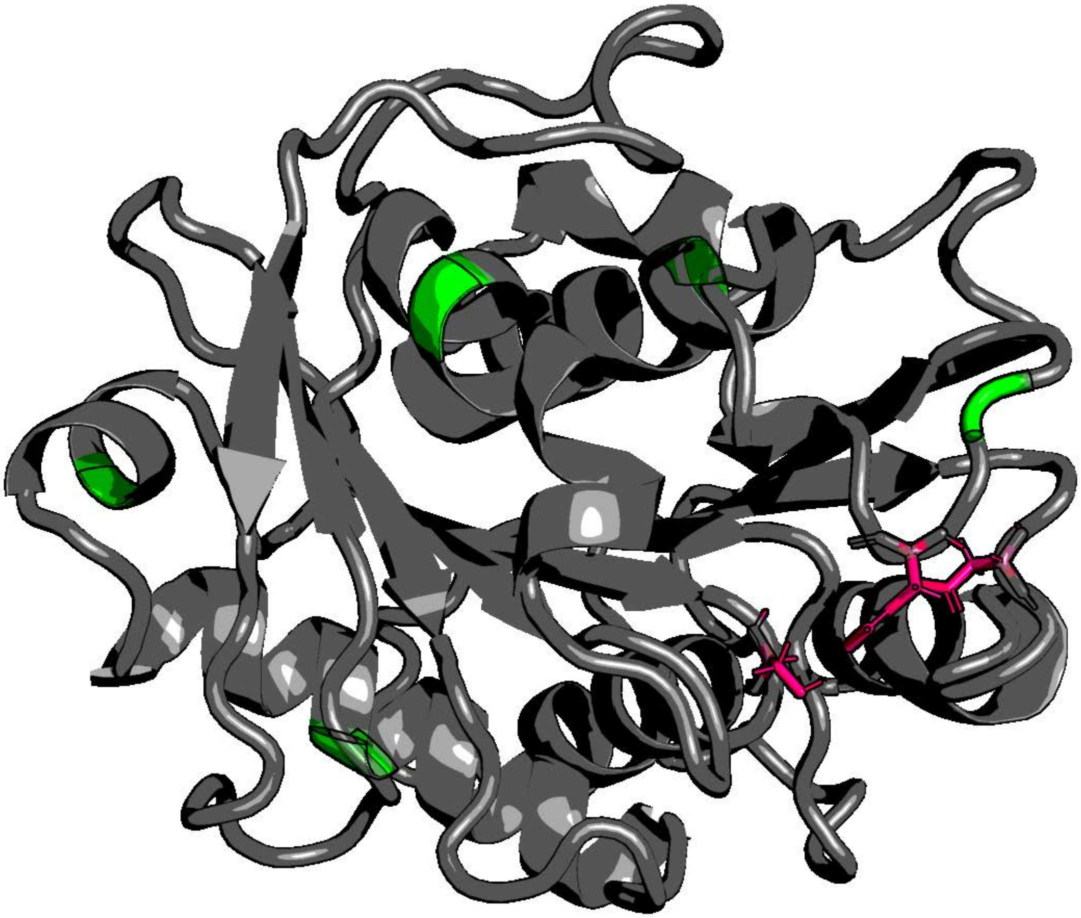

ESM090_highlights.png

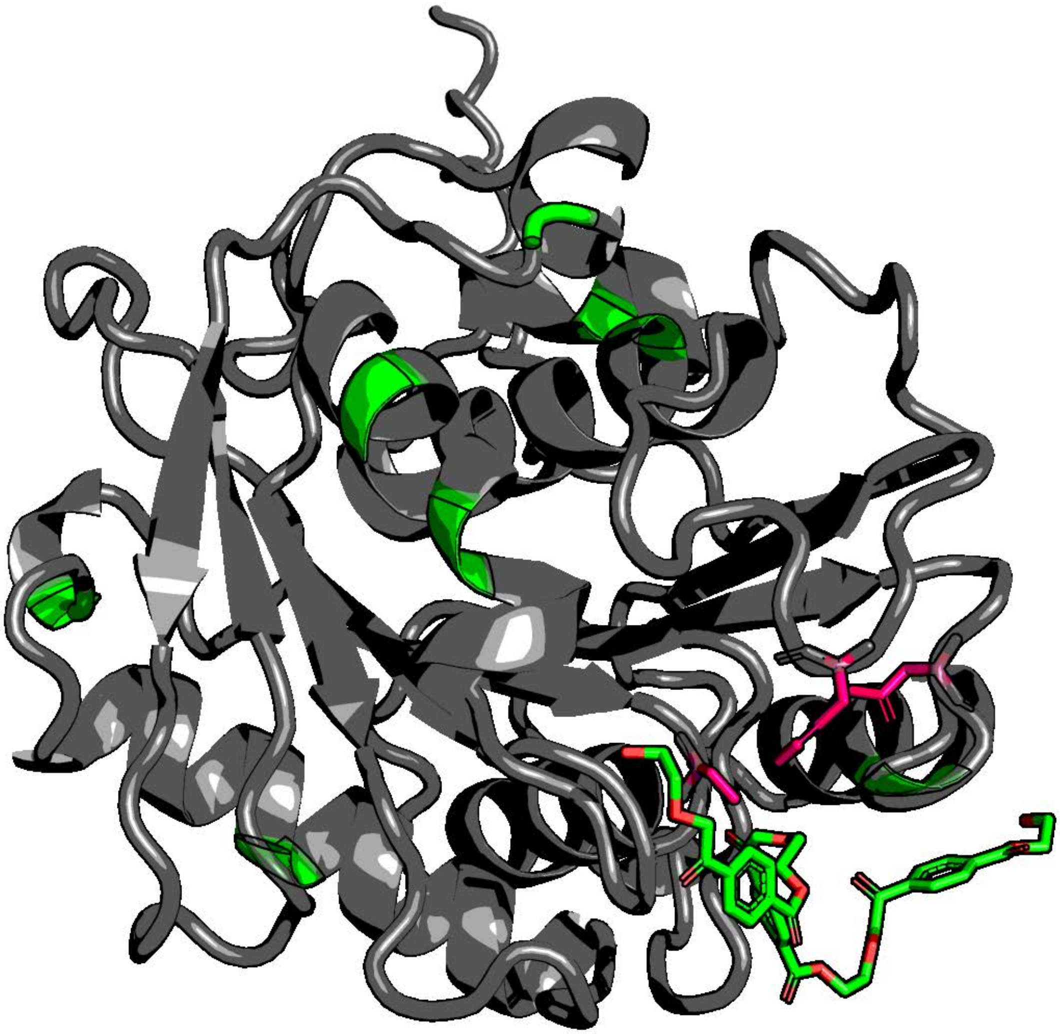

ESM091_highlights.png

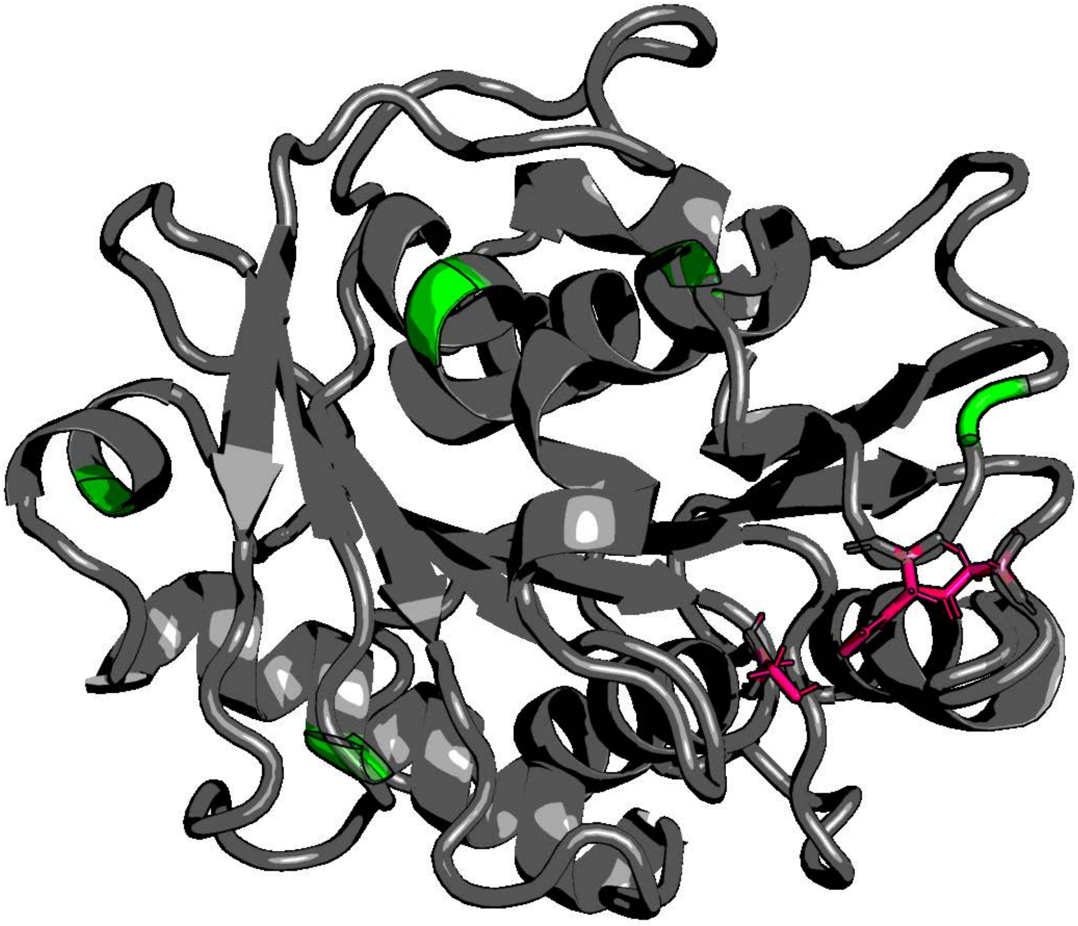

ESM093_highlights.png

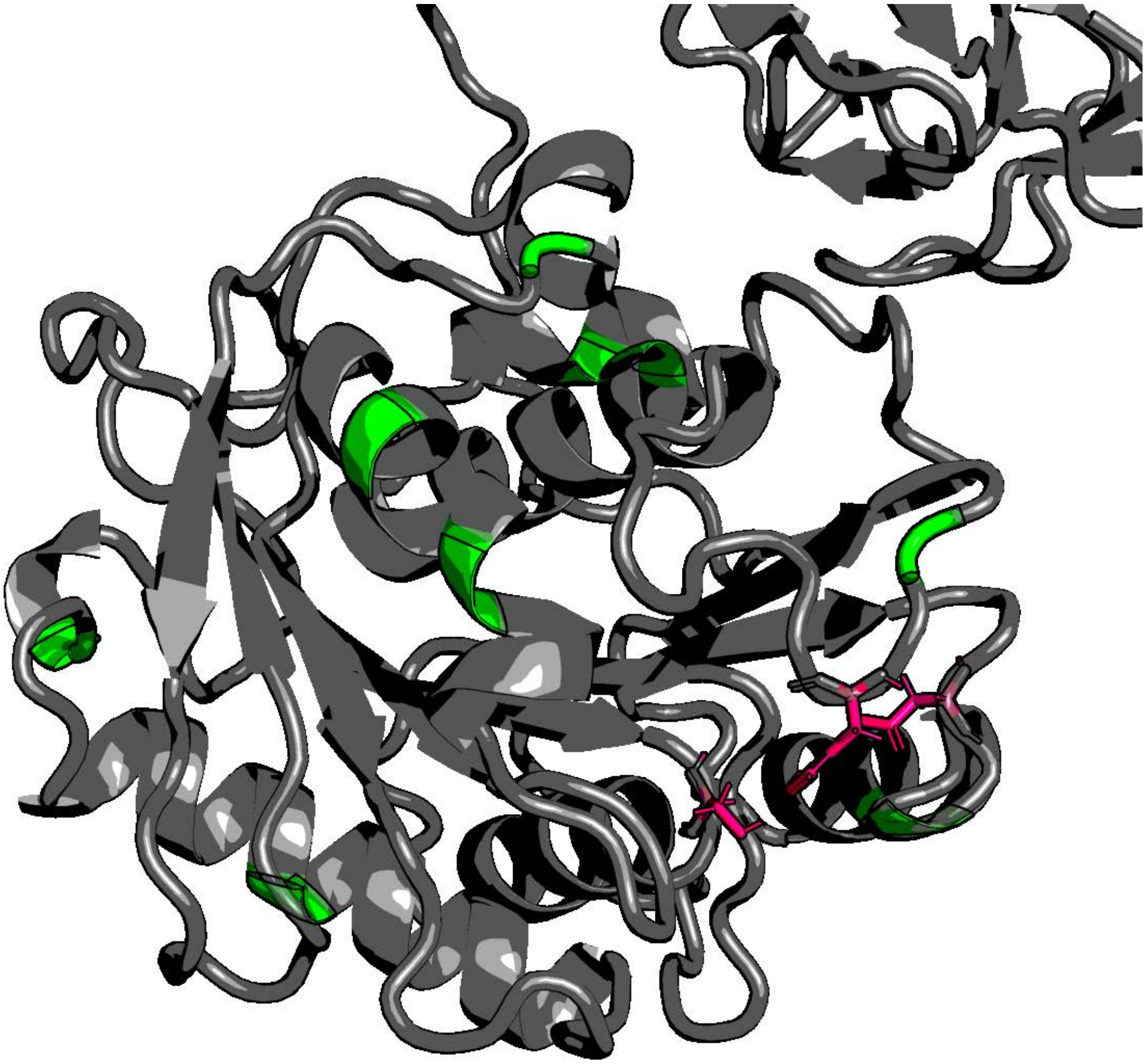

ESM095_highlights.png

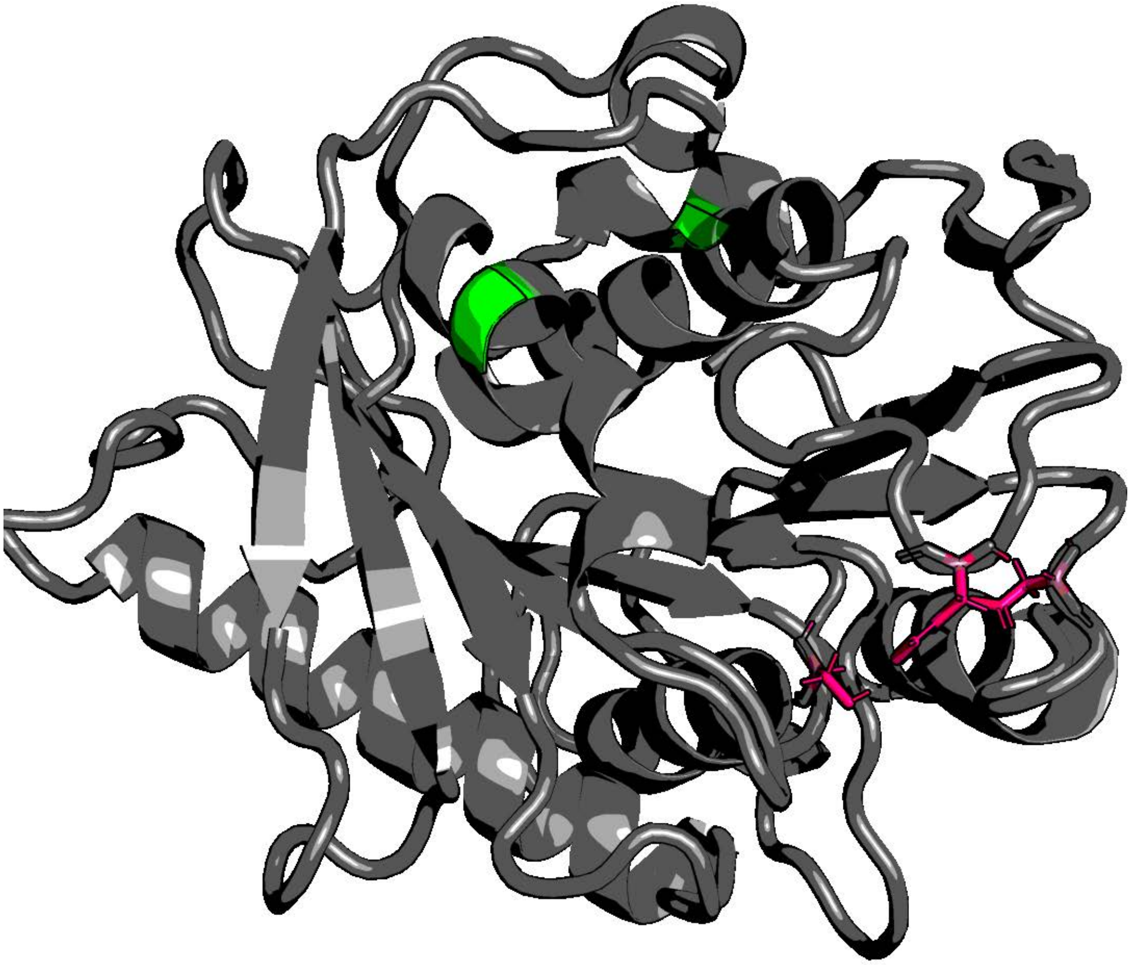

ESM096_highlights.png

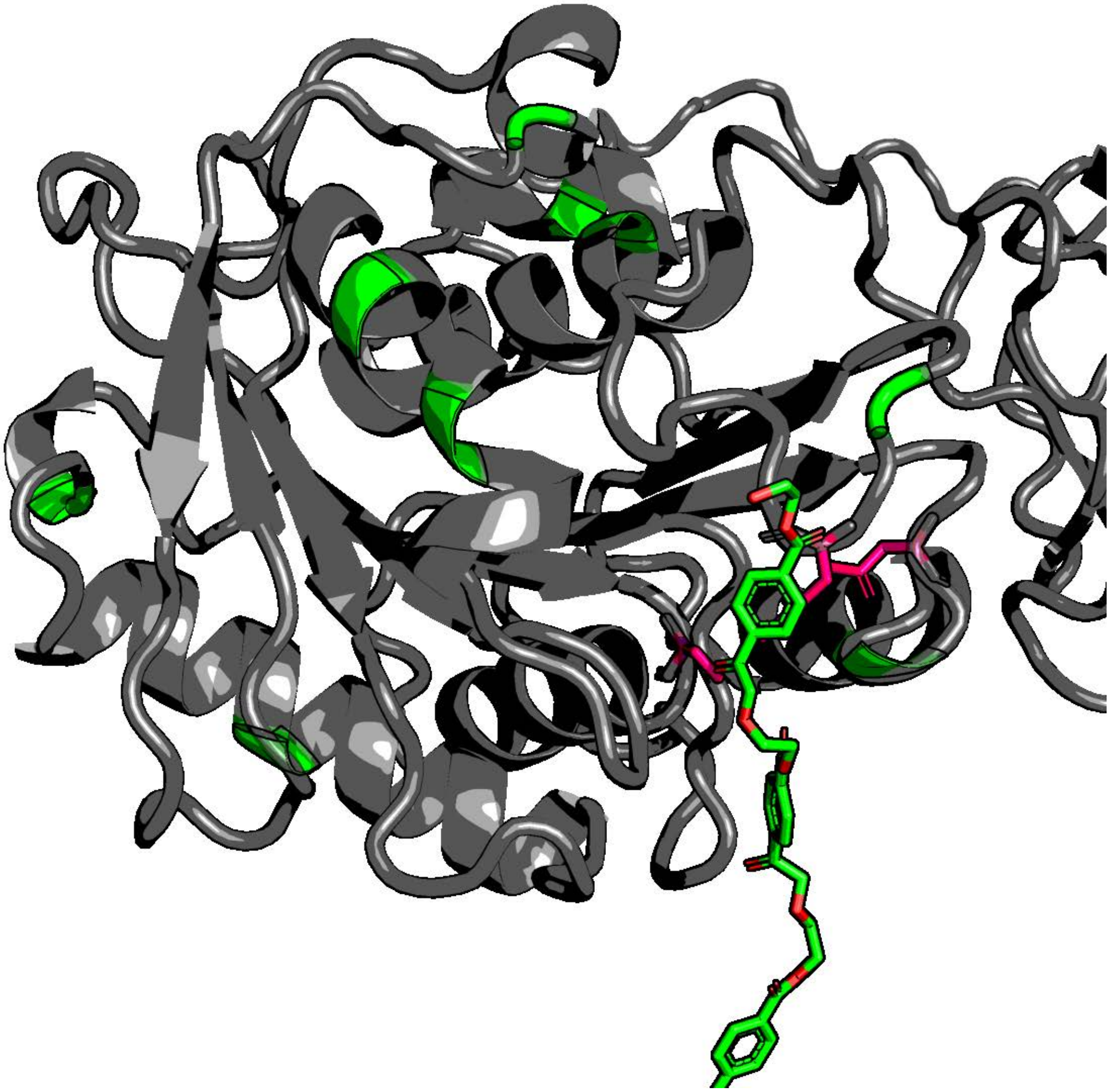

ESM101_highlights.png

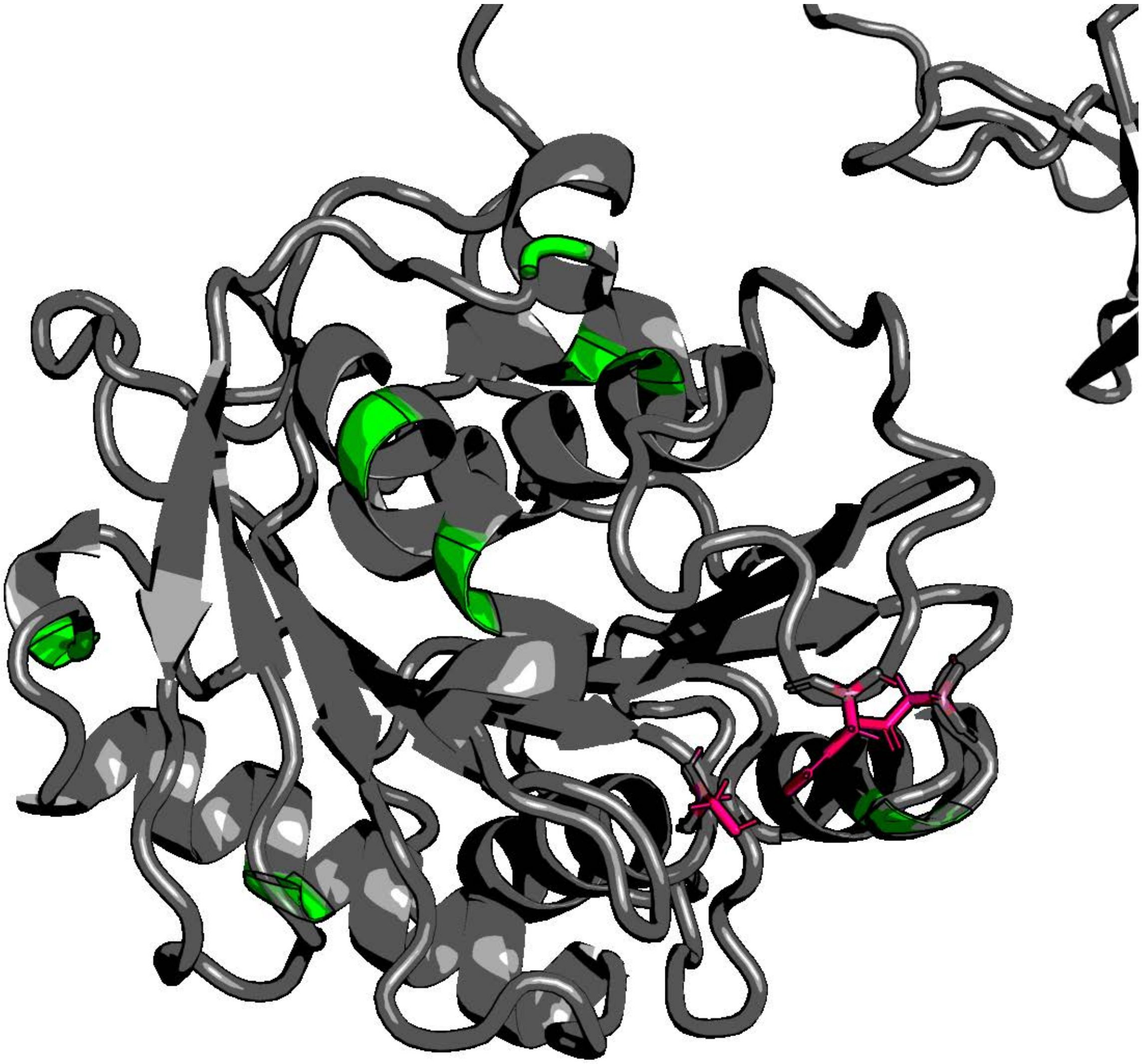

ESM106_highlights.png

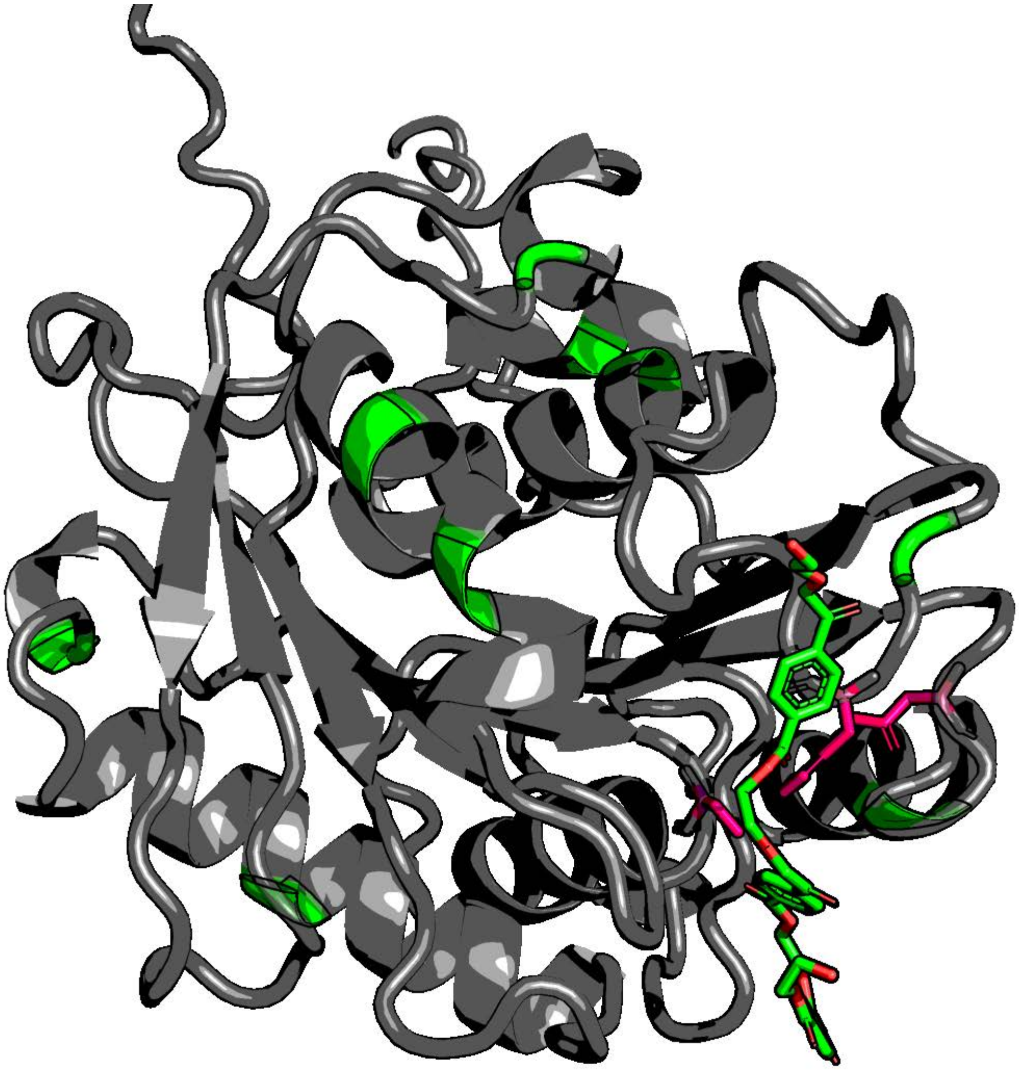

ESM121_highlights.png

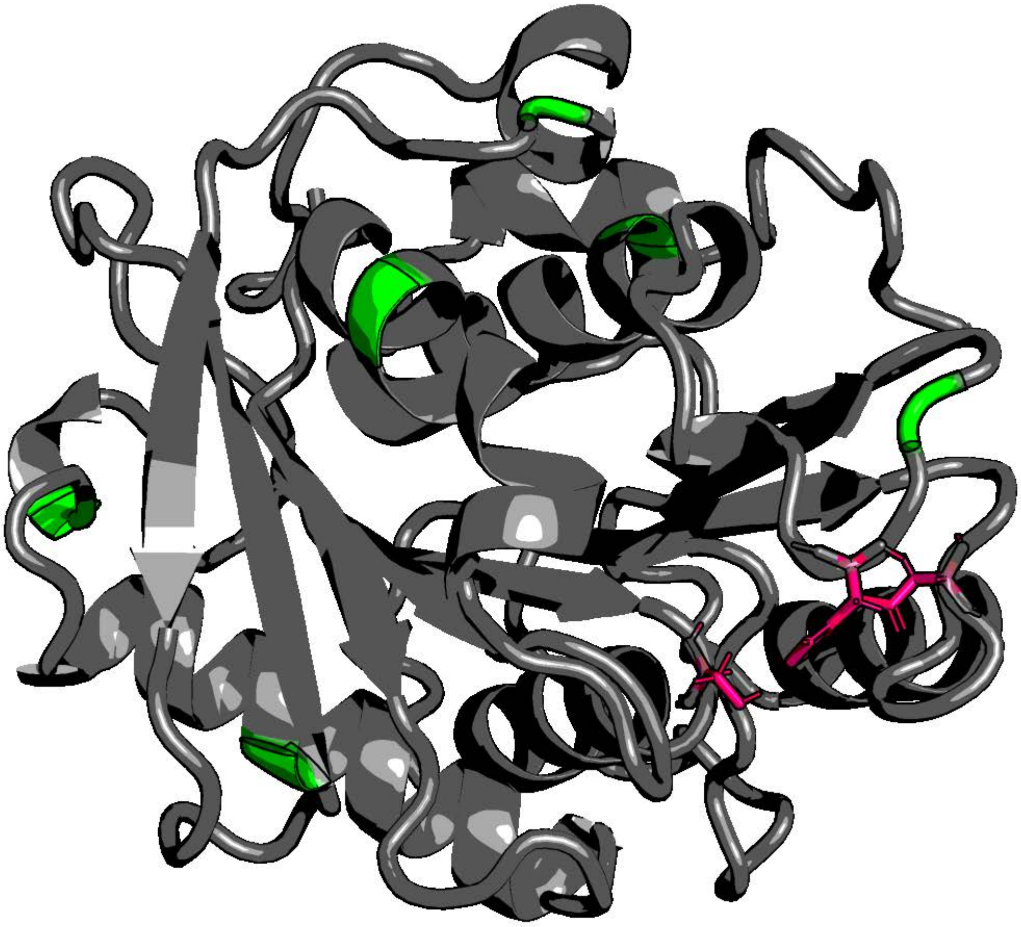

ESM128_highlights.png

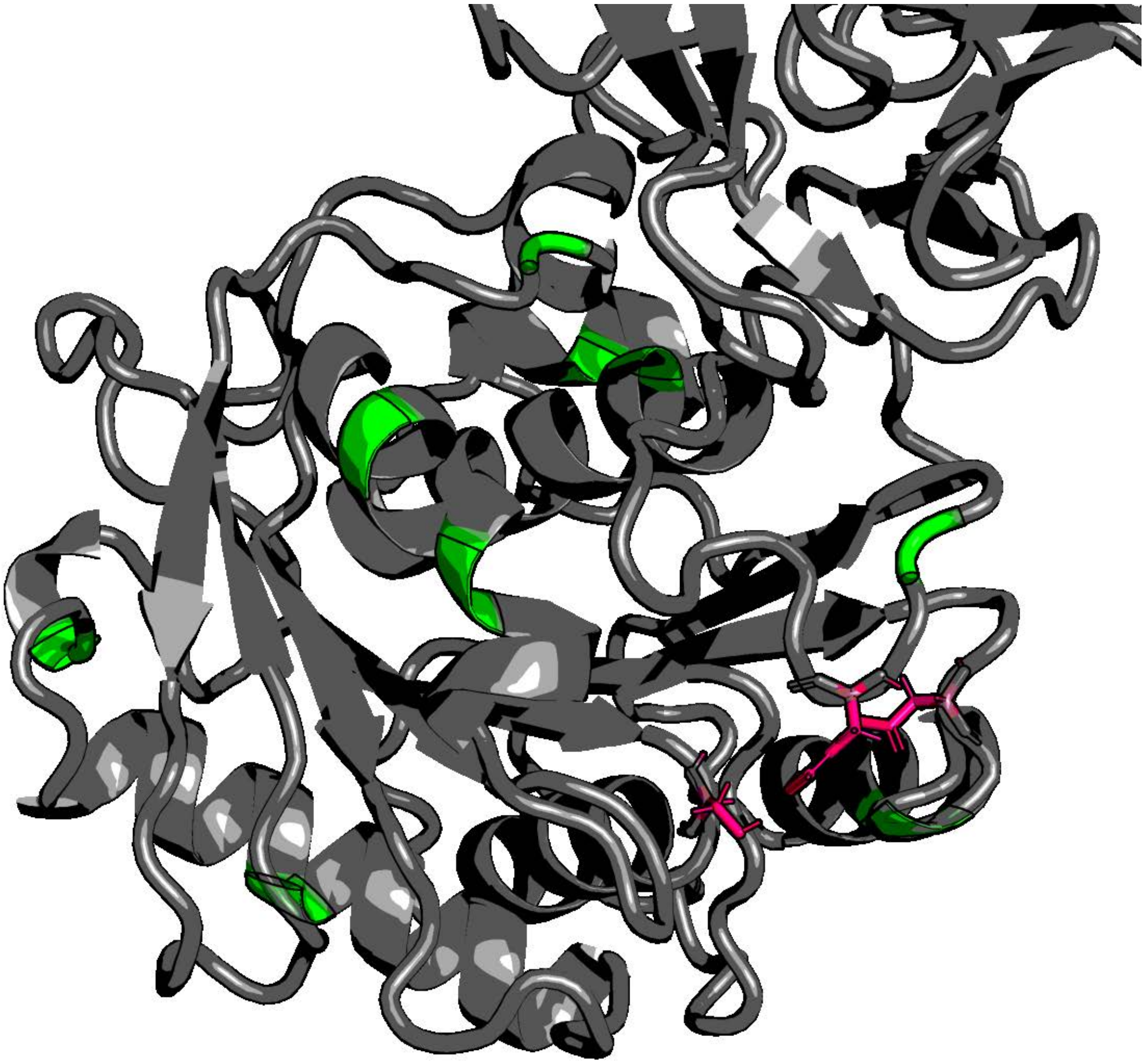

ESM130_highlights.png

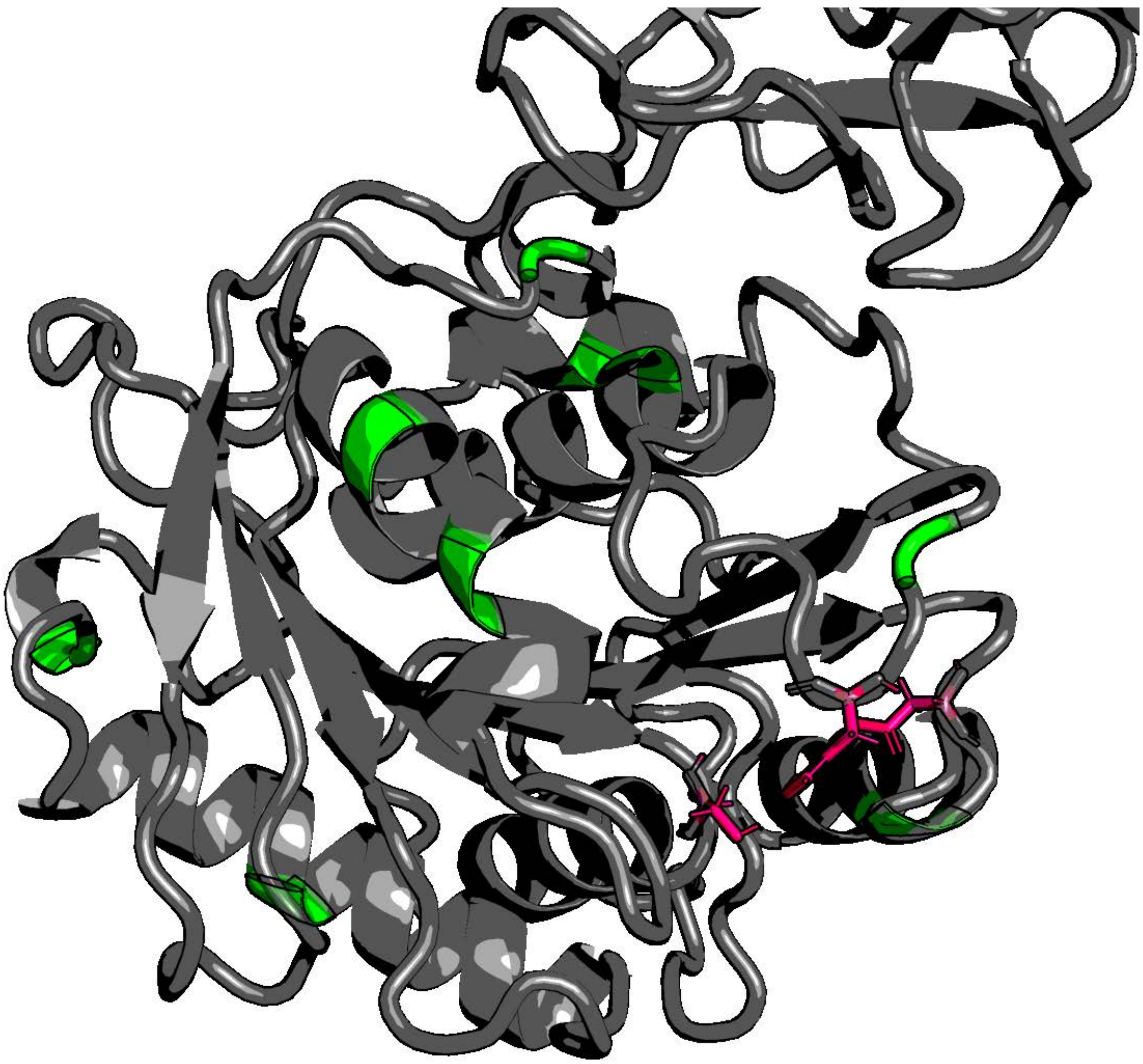

ESM132_highlights.png

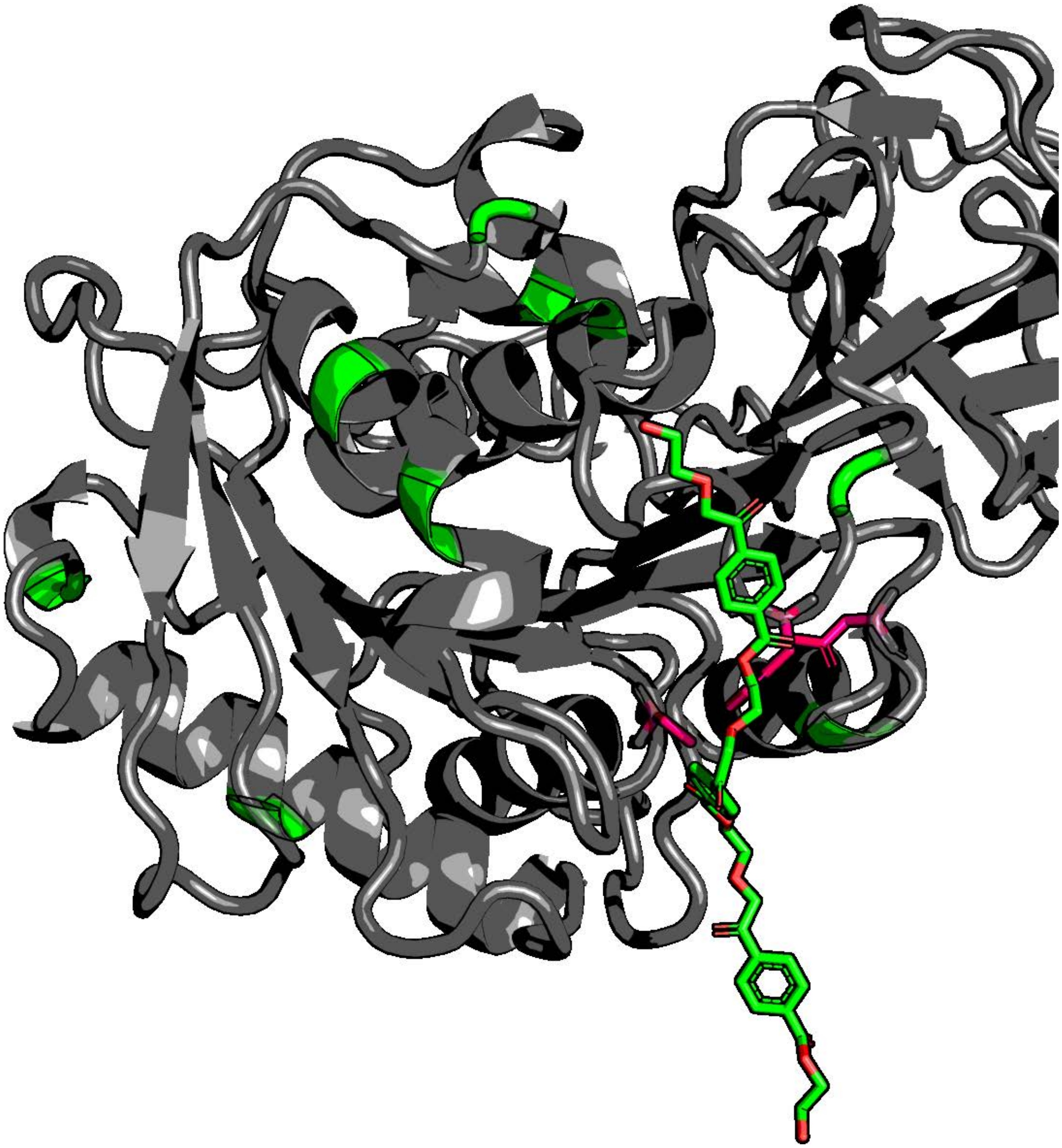

ESM135_highlights.png

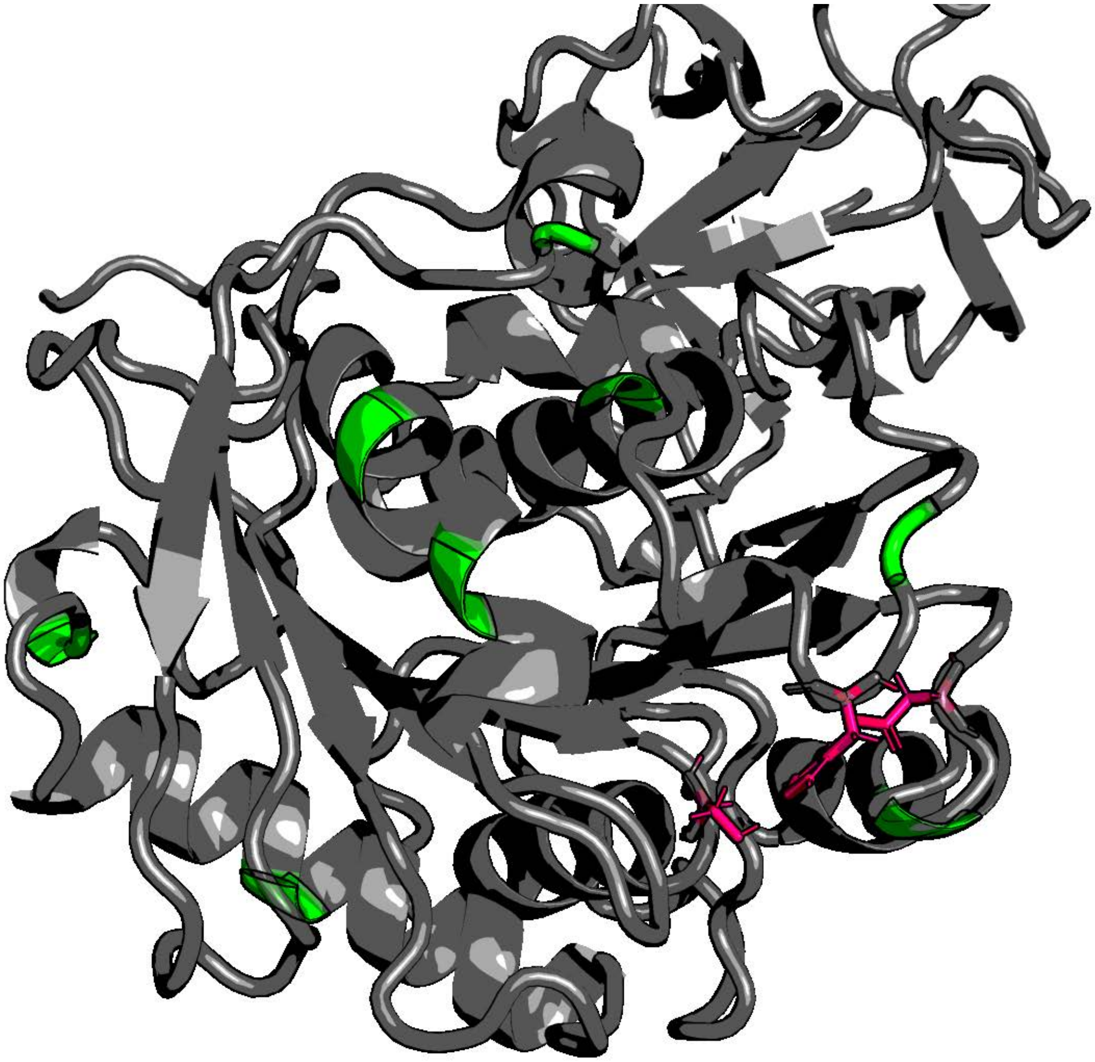

ESM136_highlights.png

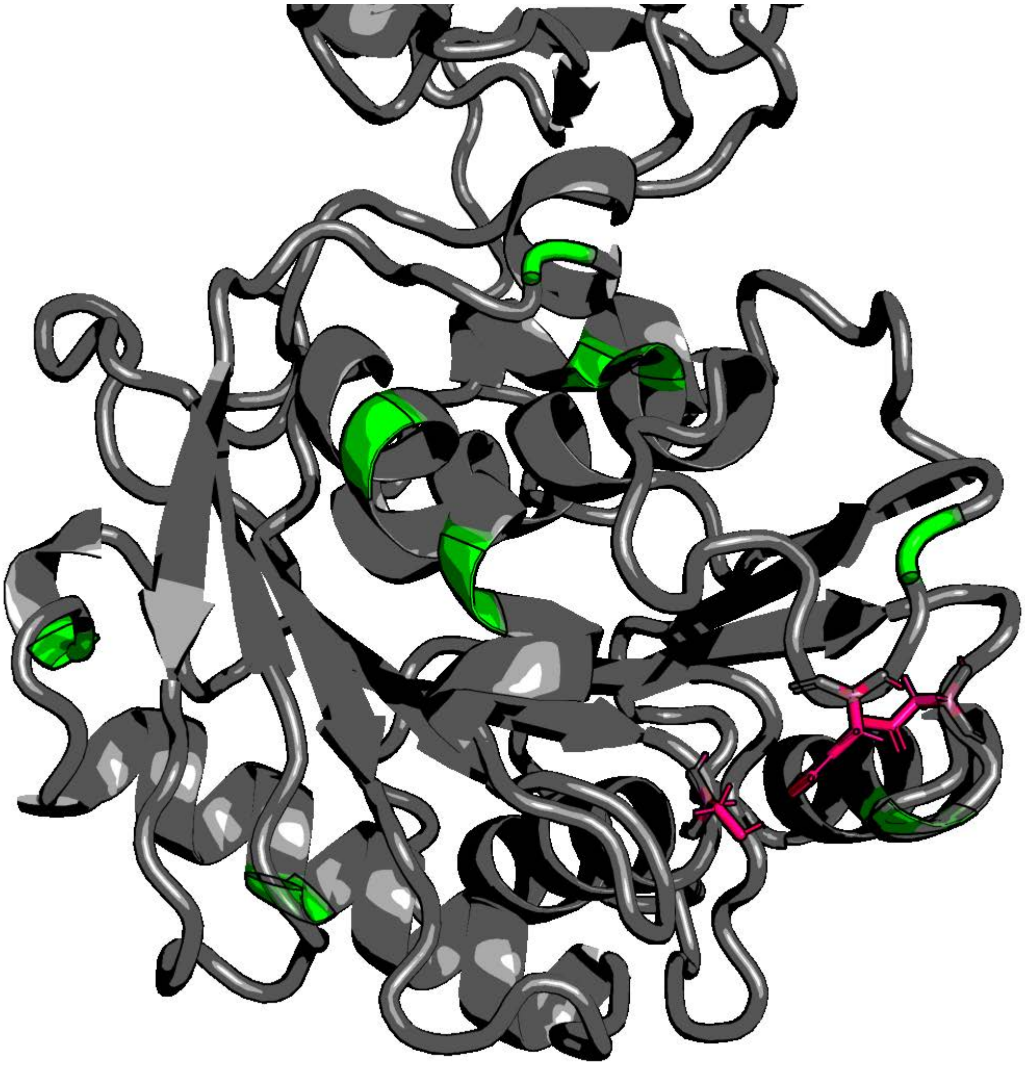

ESM137_highlights.png

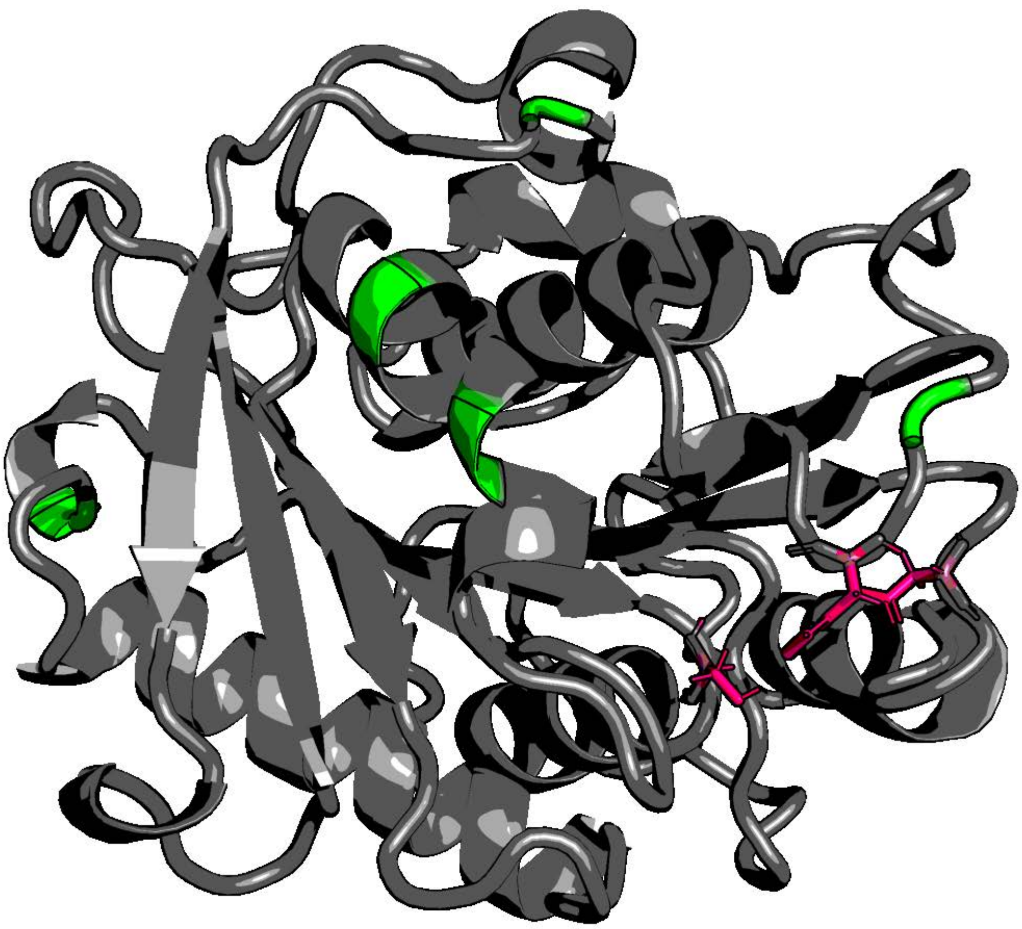

ESM140_highlights.png

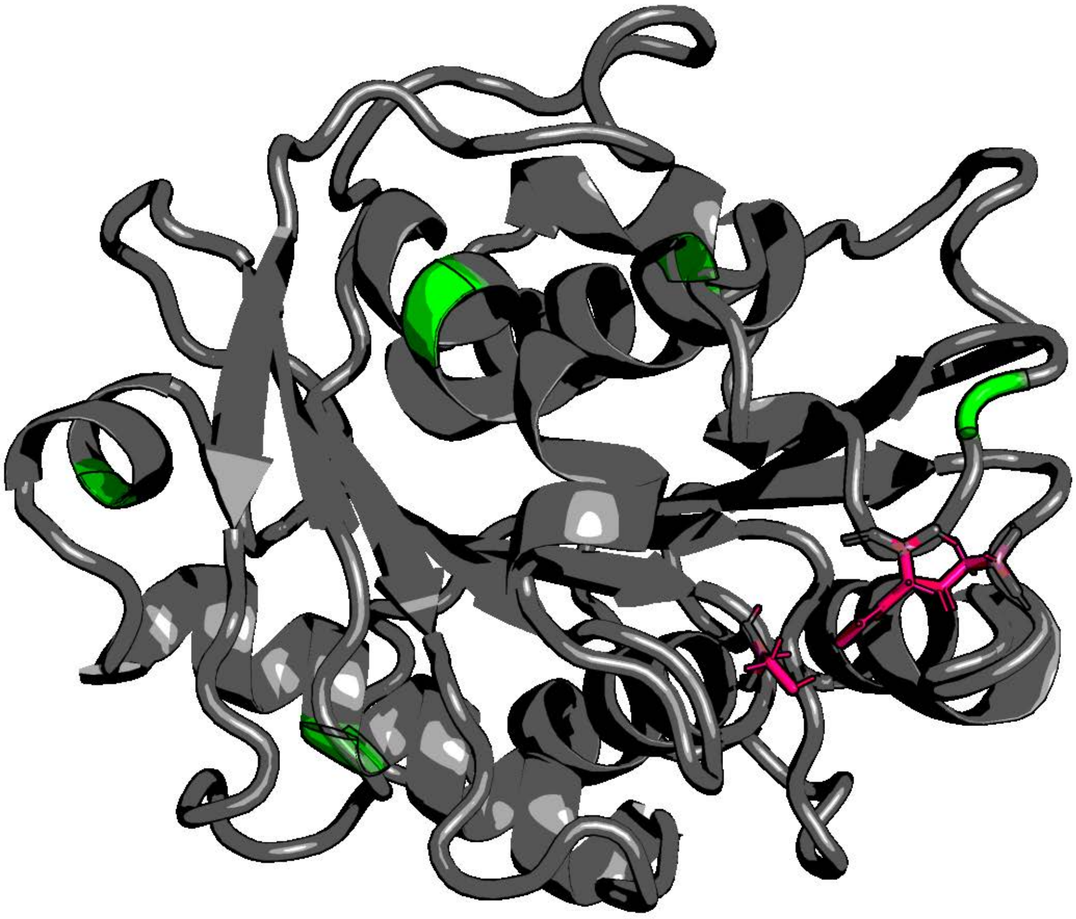

ESM144_highlights.png

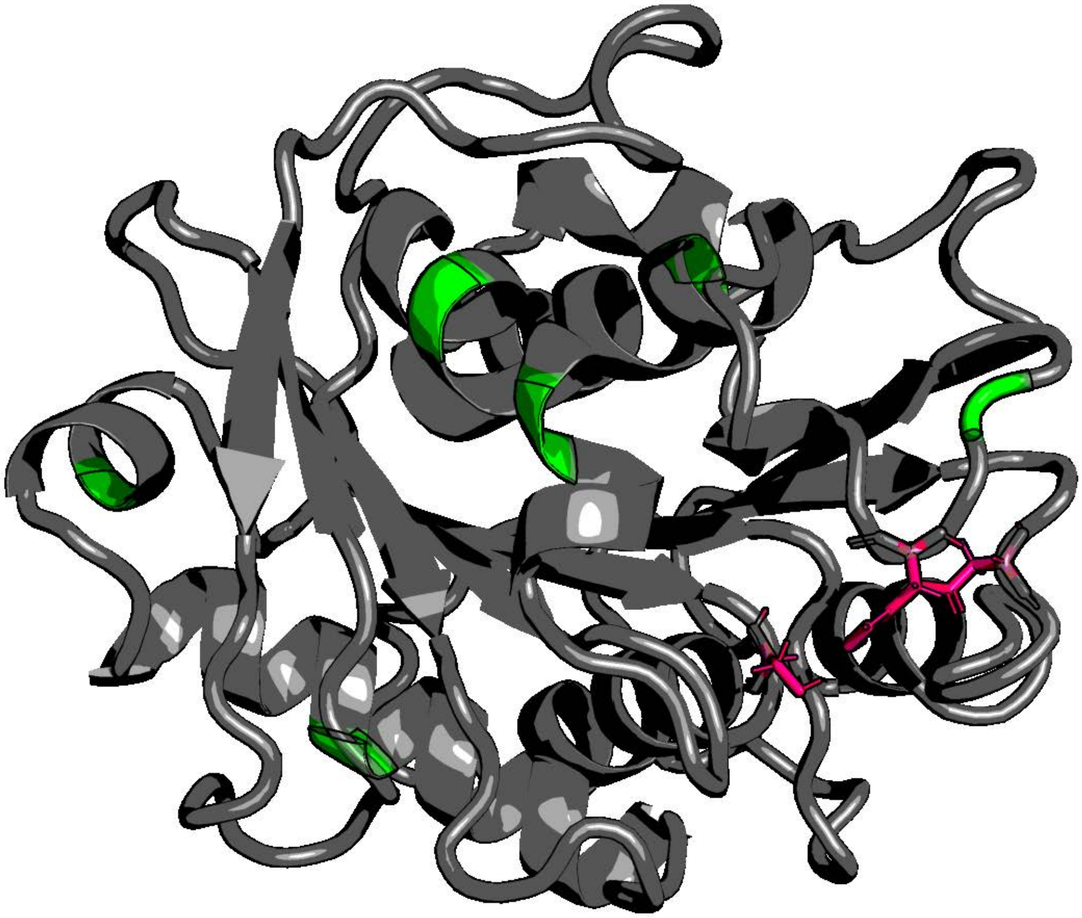

ESM147_highlights.png

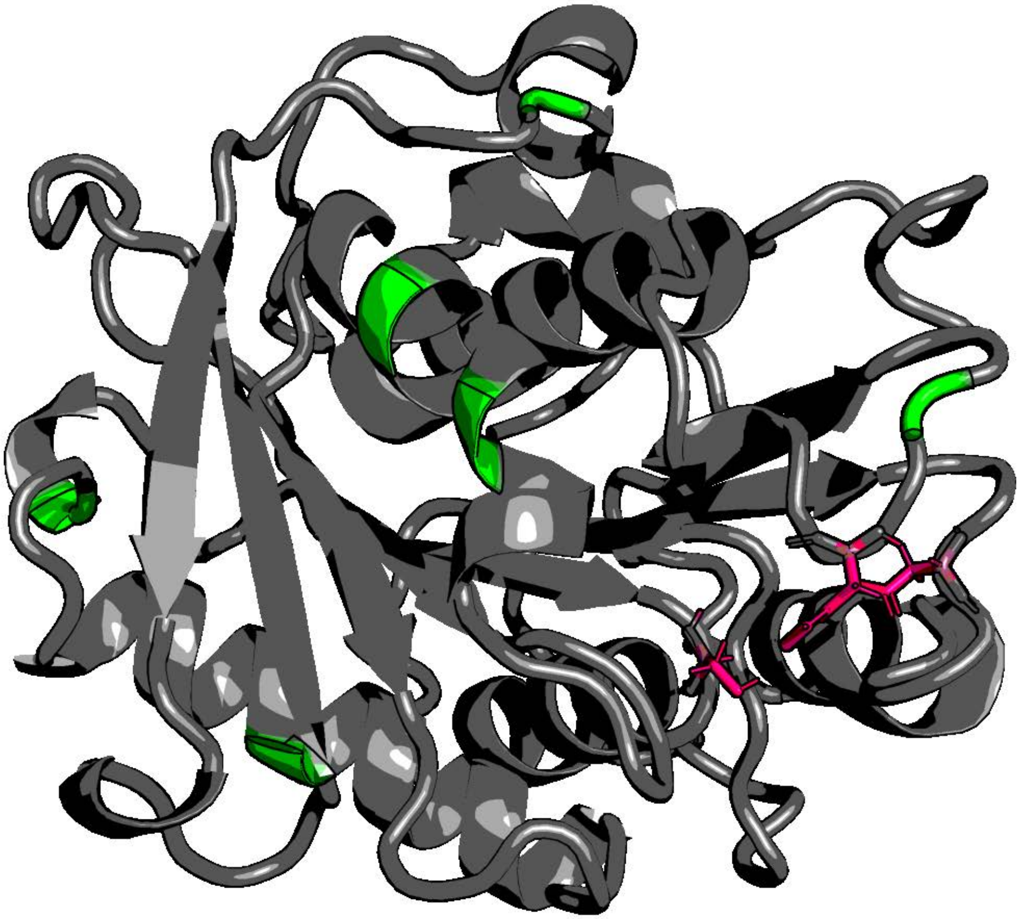

ESM156_highlights.png

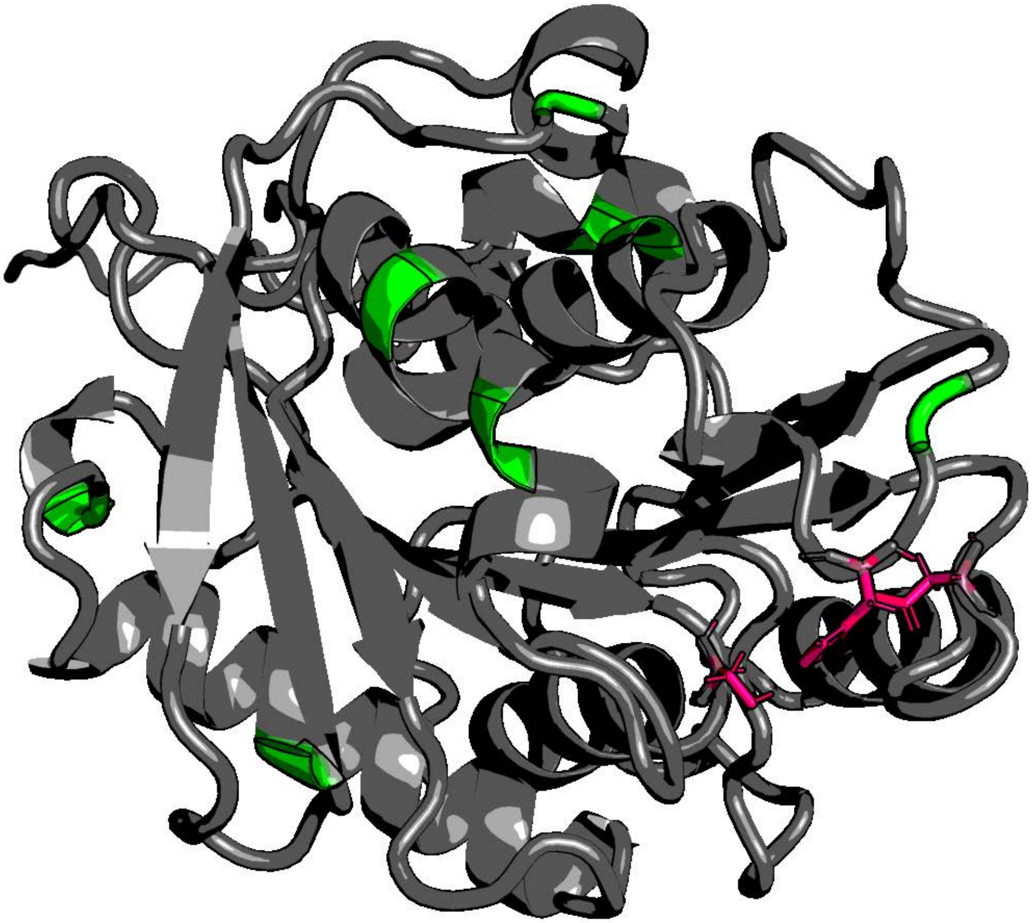

ESM157_highlights.png

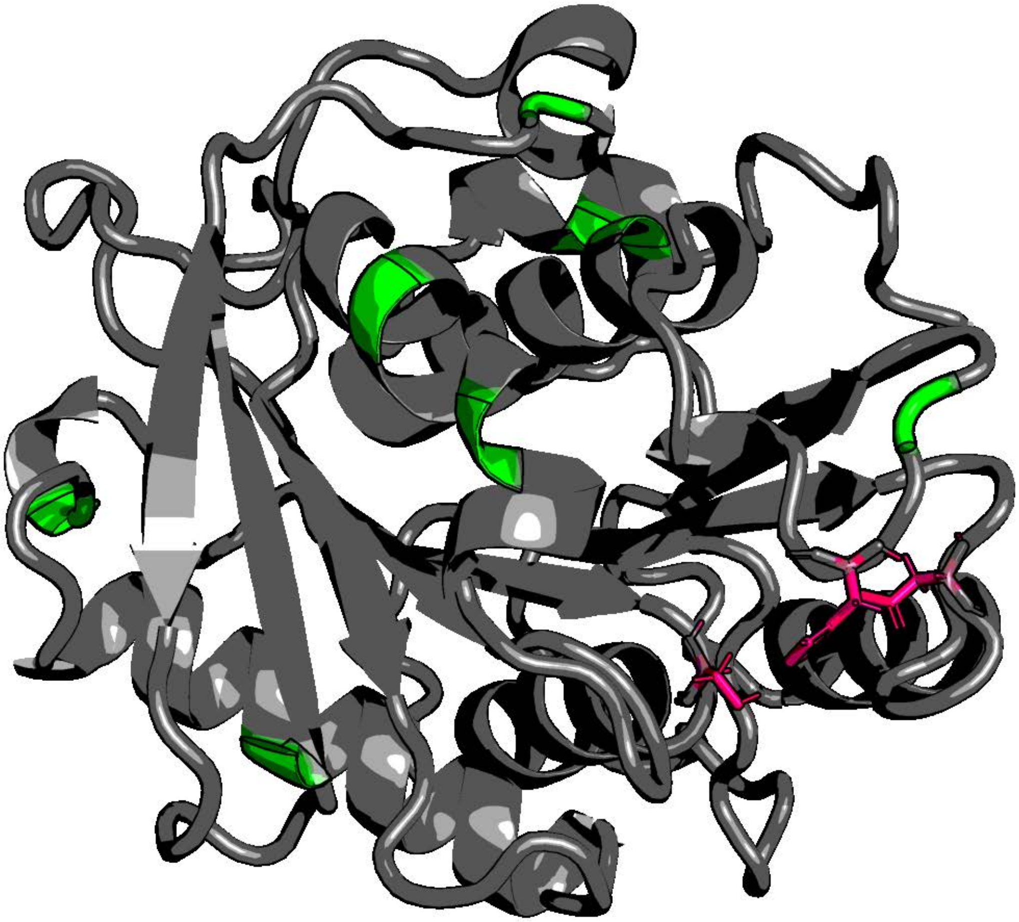

ESM159_highlights.png

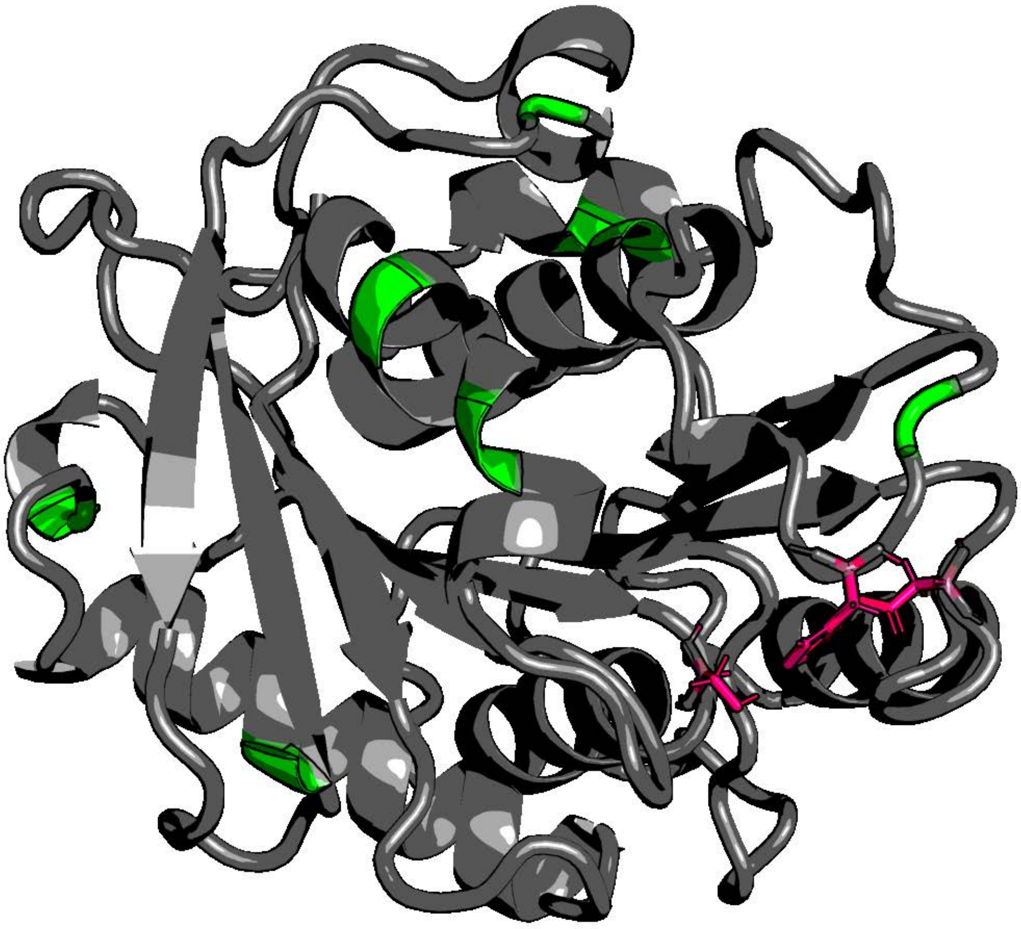

ESM160_highlights.png

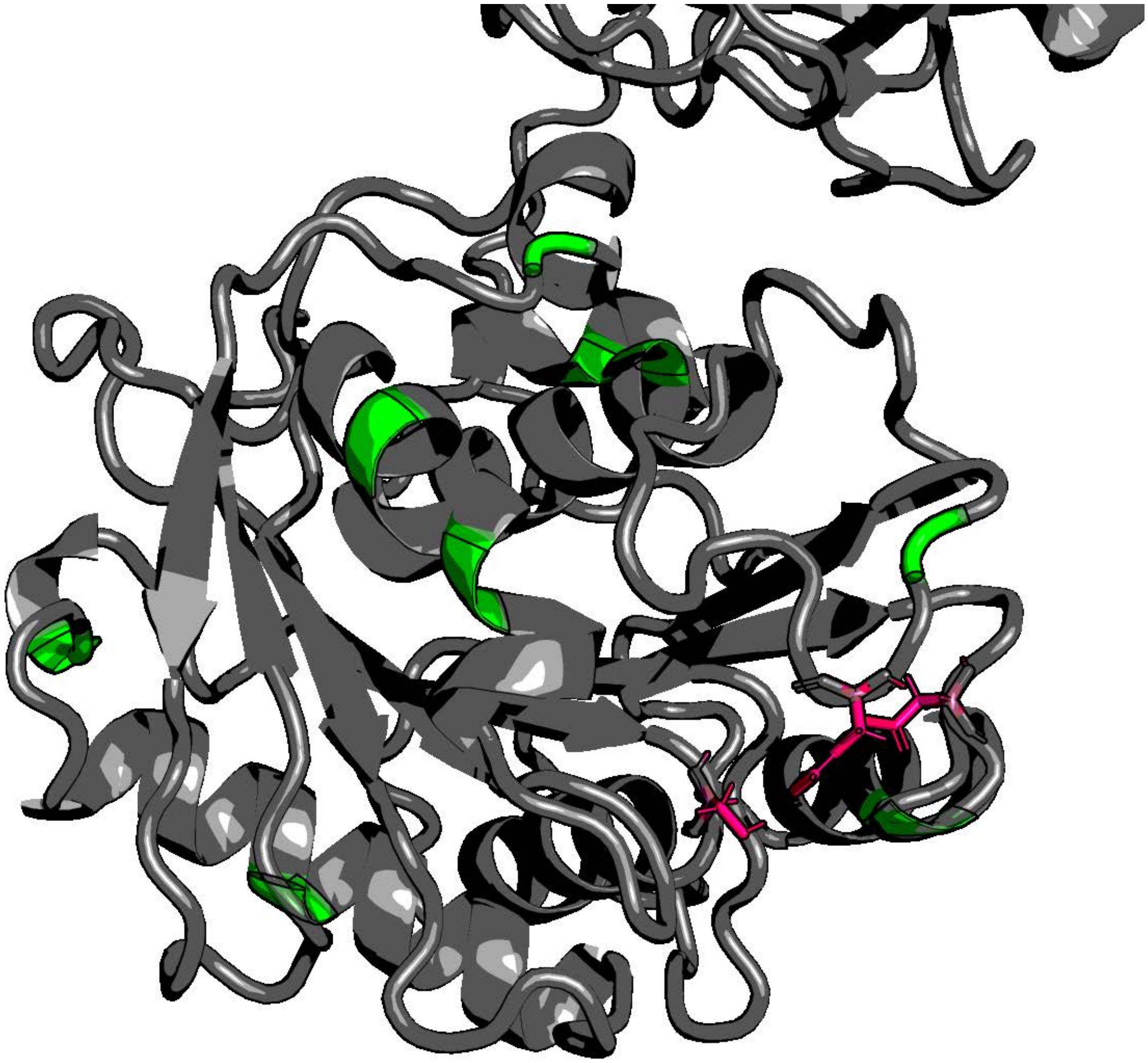

ESM161_highlights.png

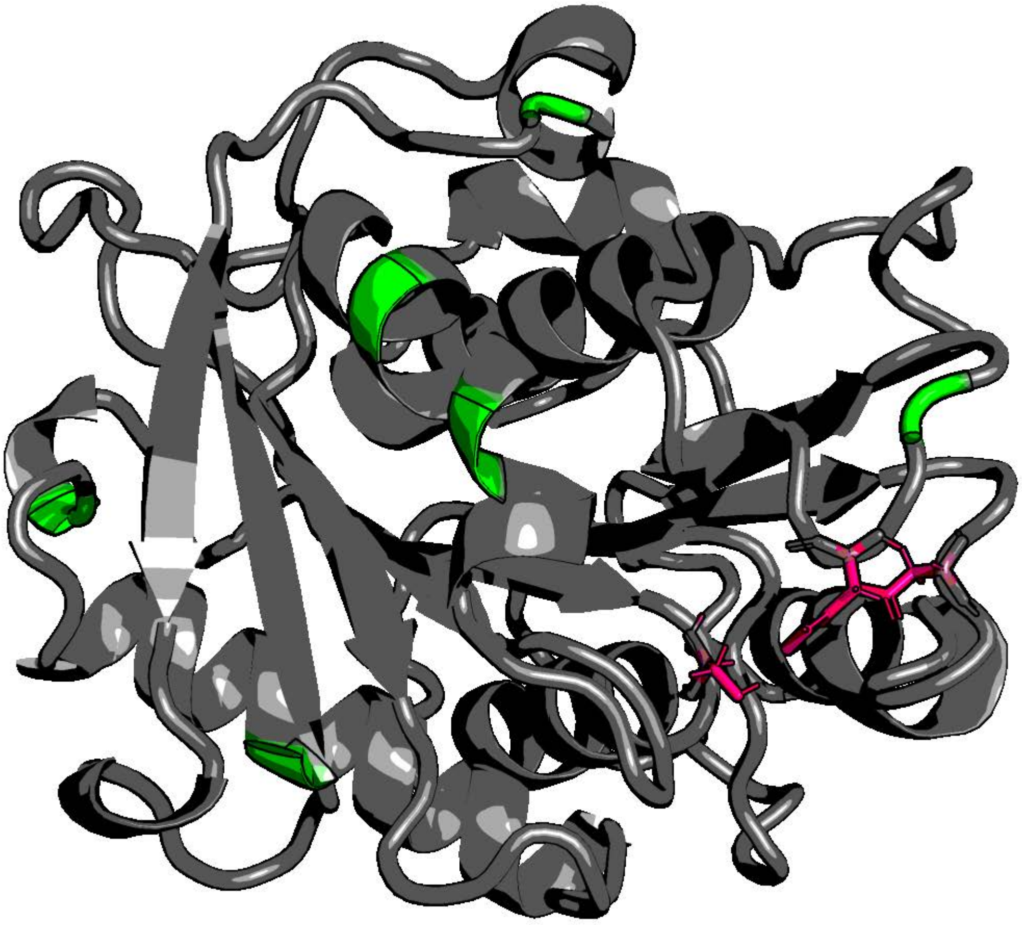

ESM167_highlights.png

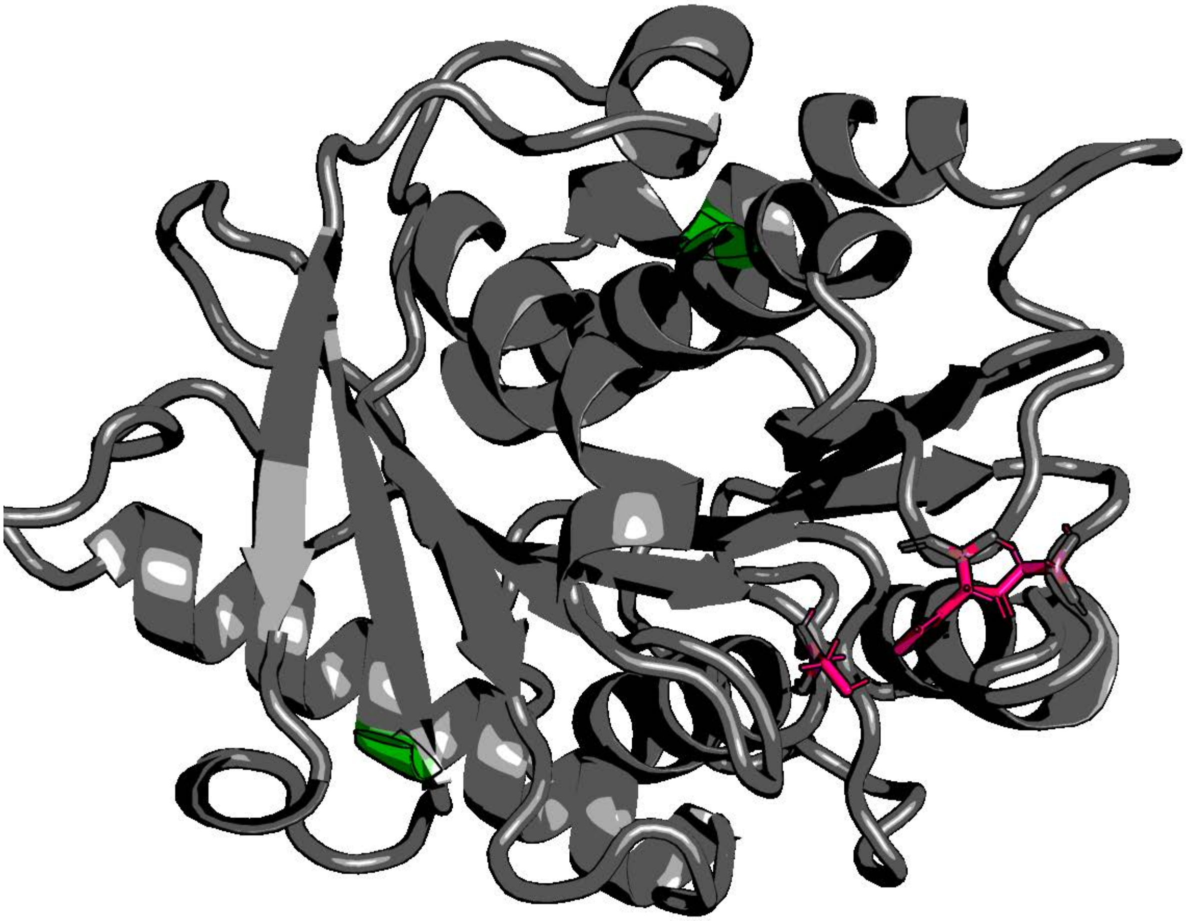

ESM169_highlights.png

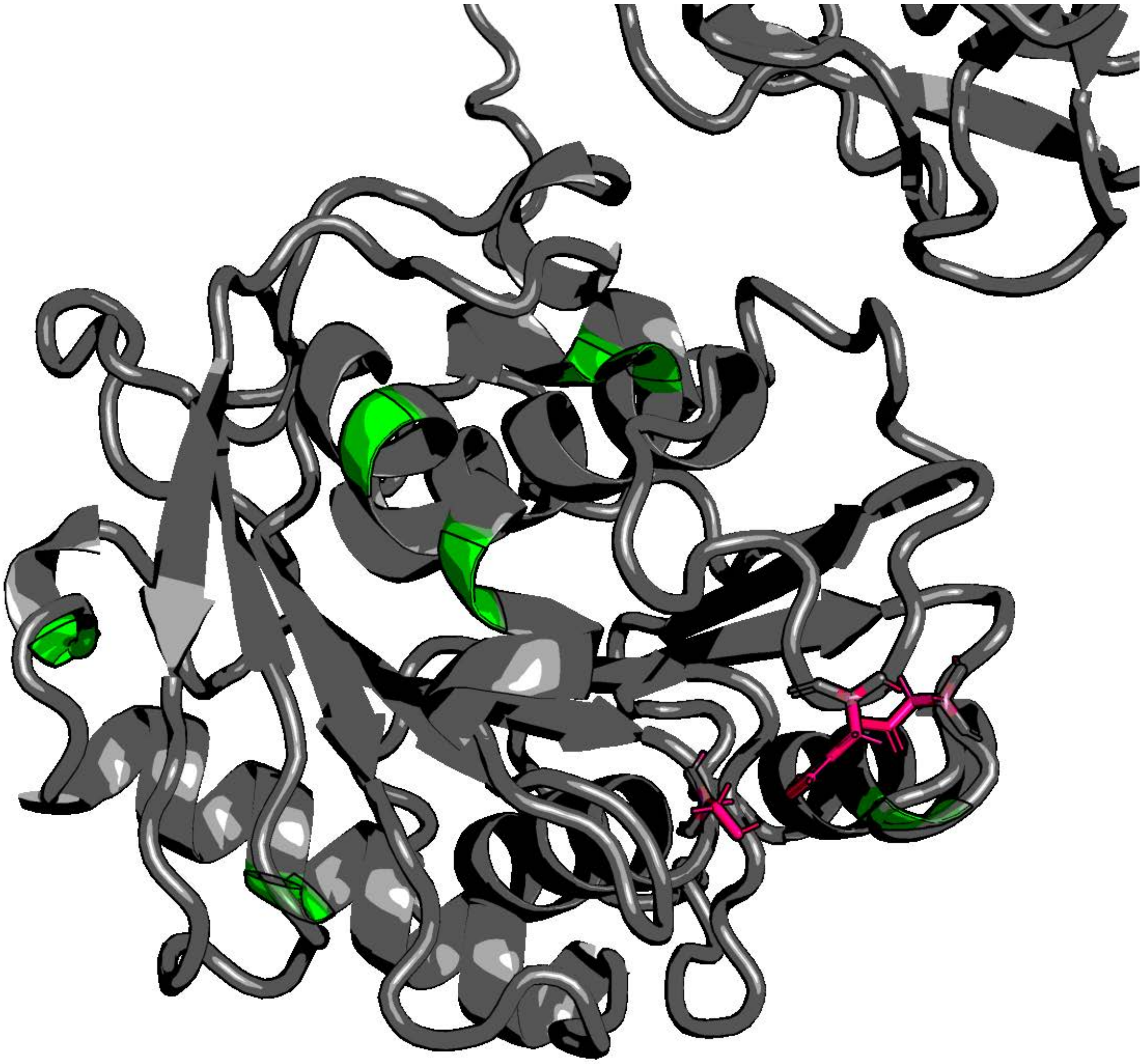

ESM173_highlights.png

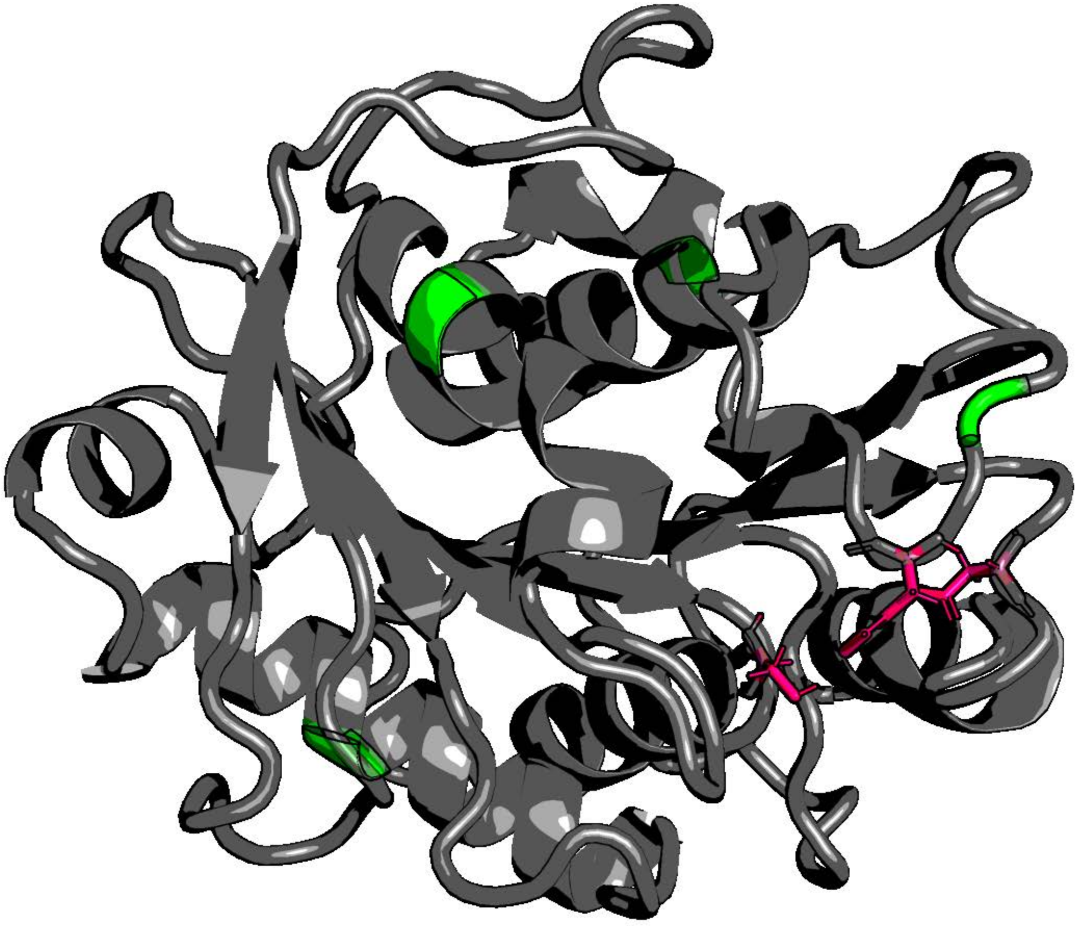

ESM177_highlights.png

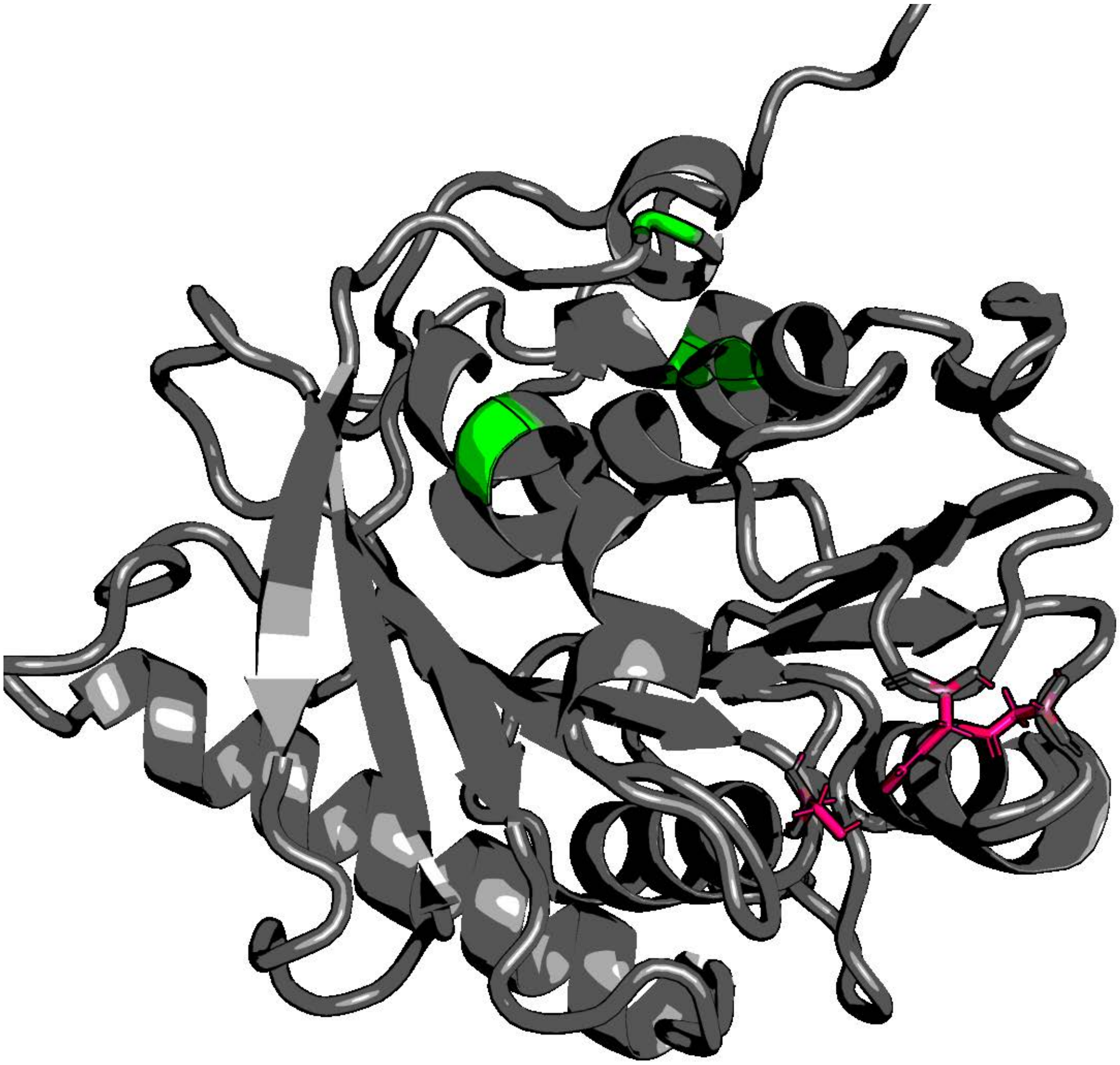

ESM186_highlights.png

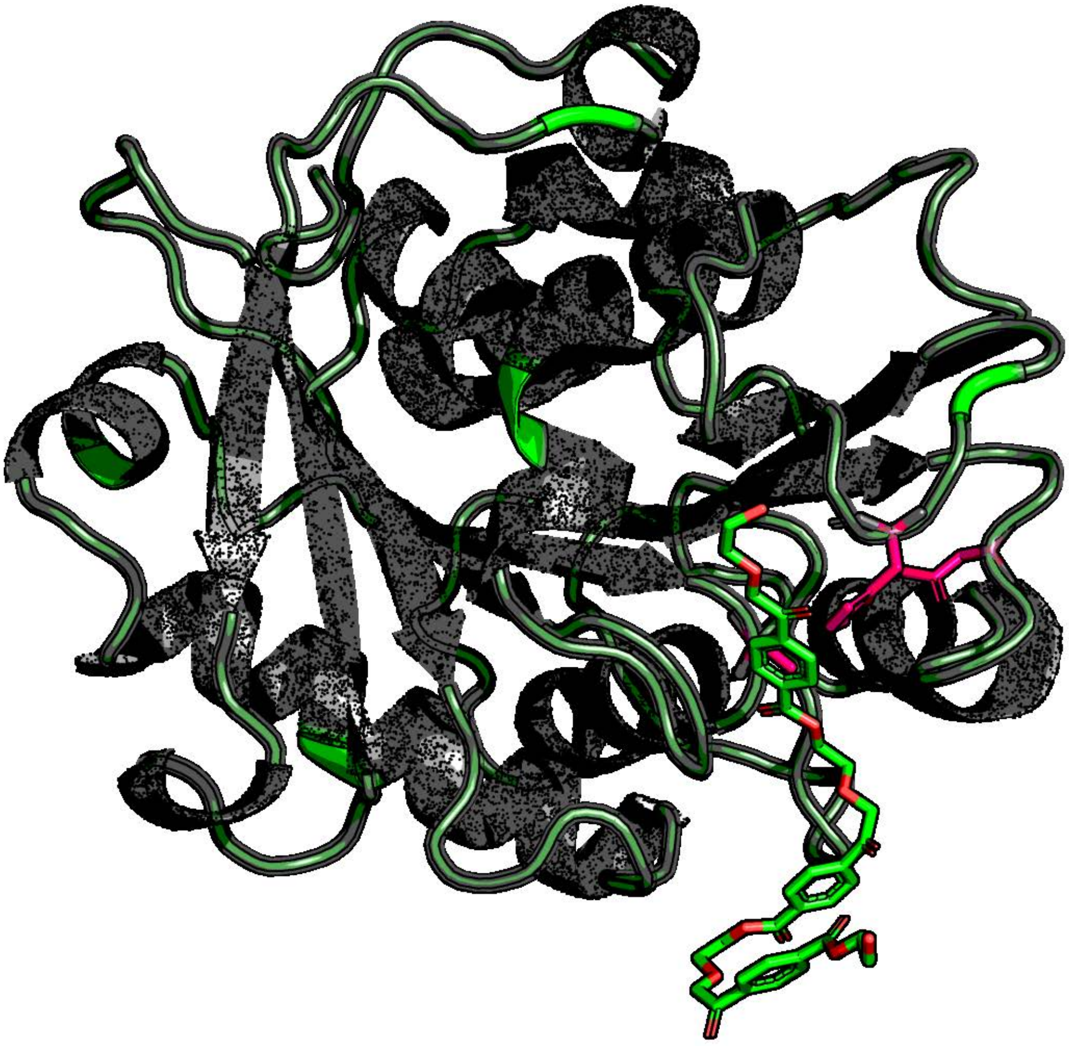

LCC-ICCG_highlights.png

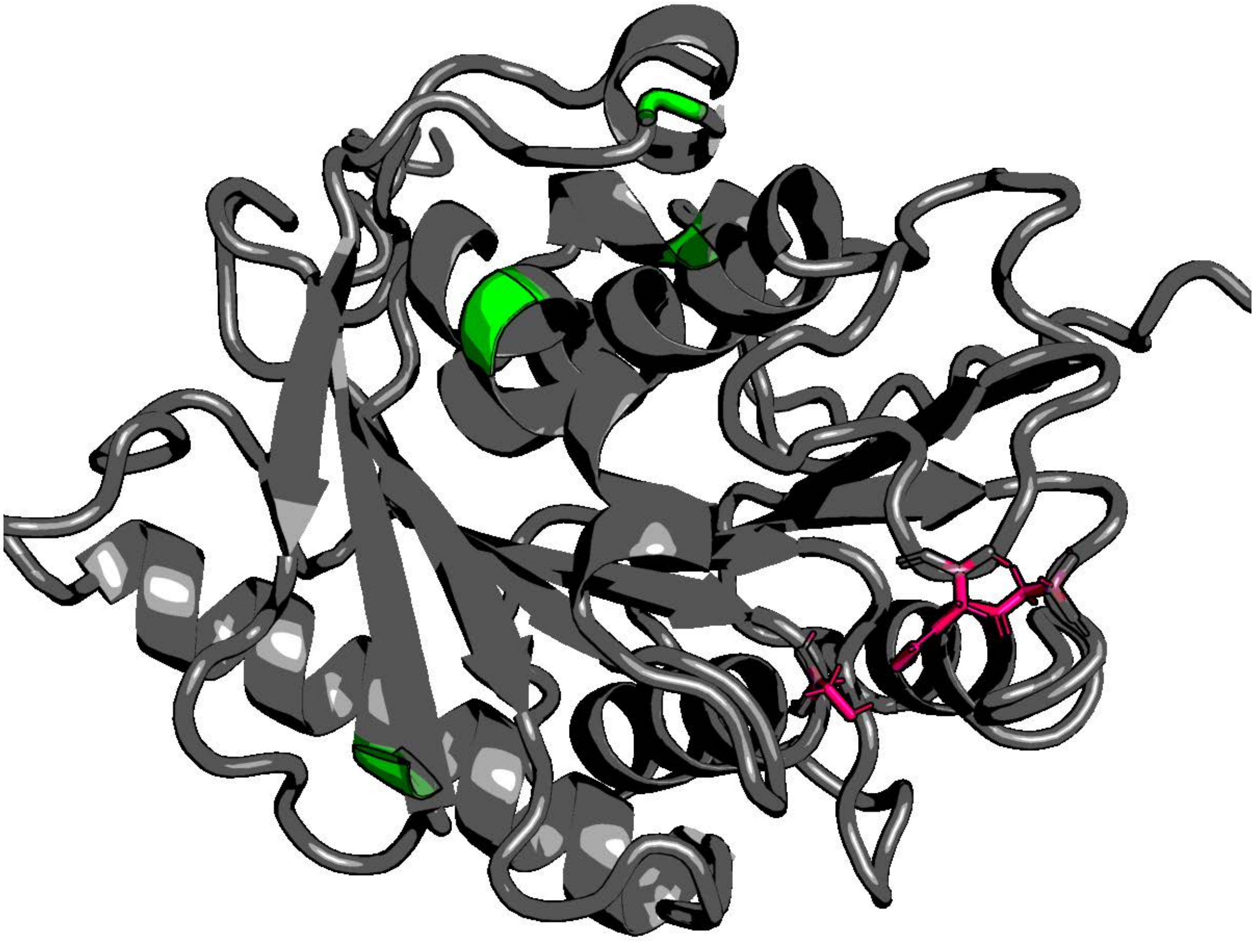

TEP004_highlights.png

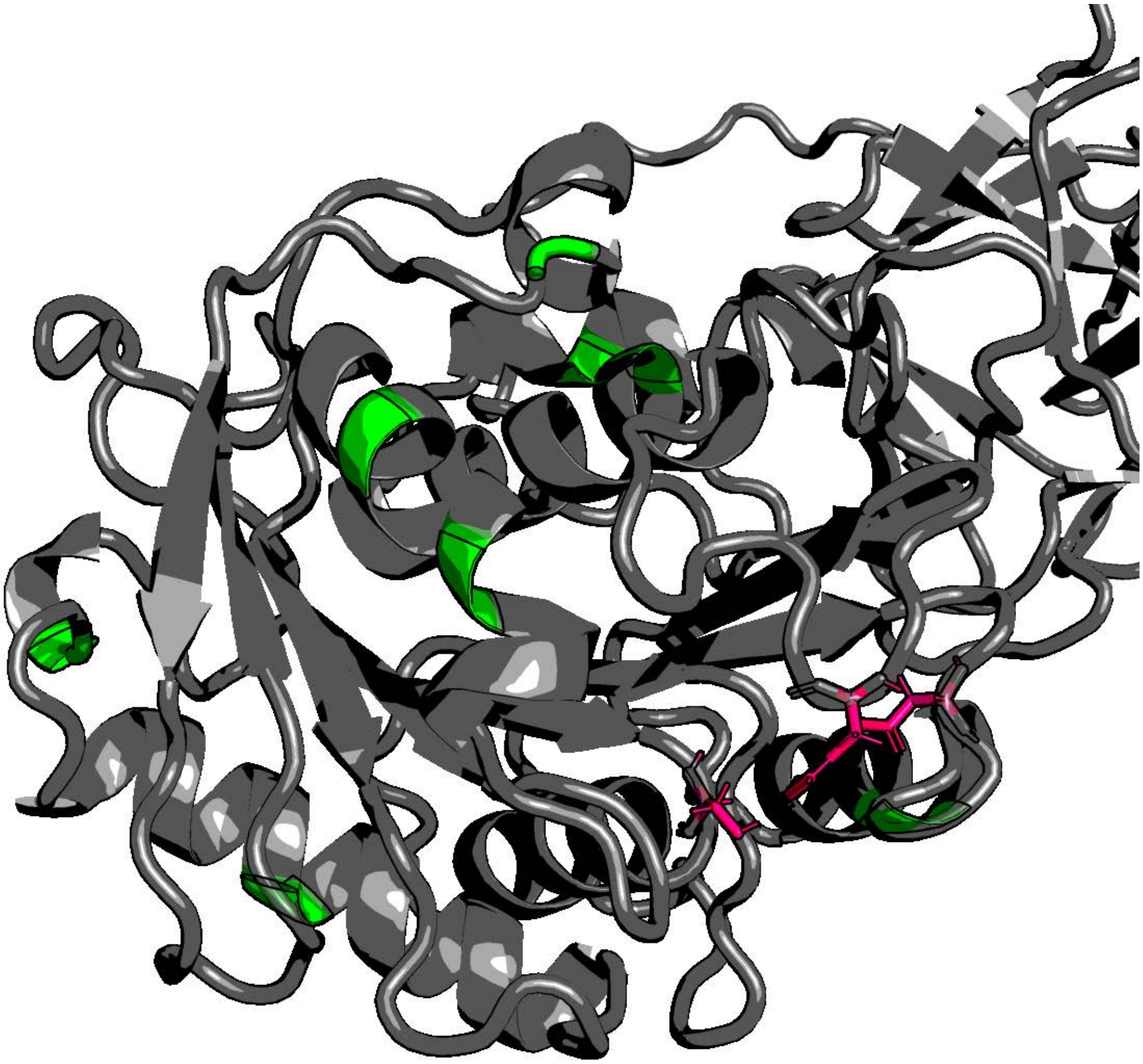

TEP010_highlights.png

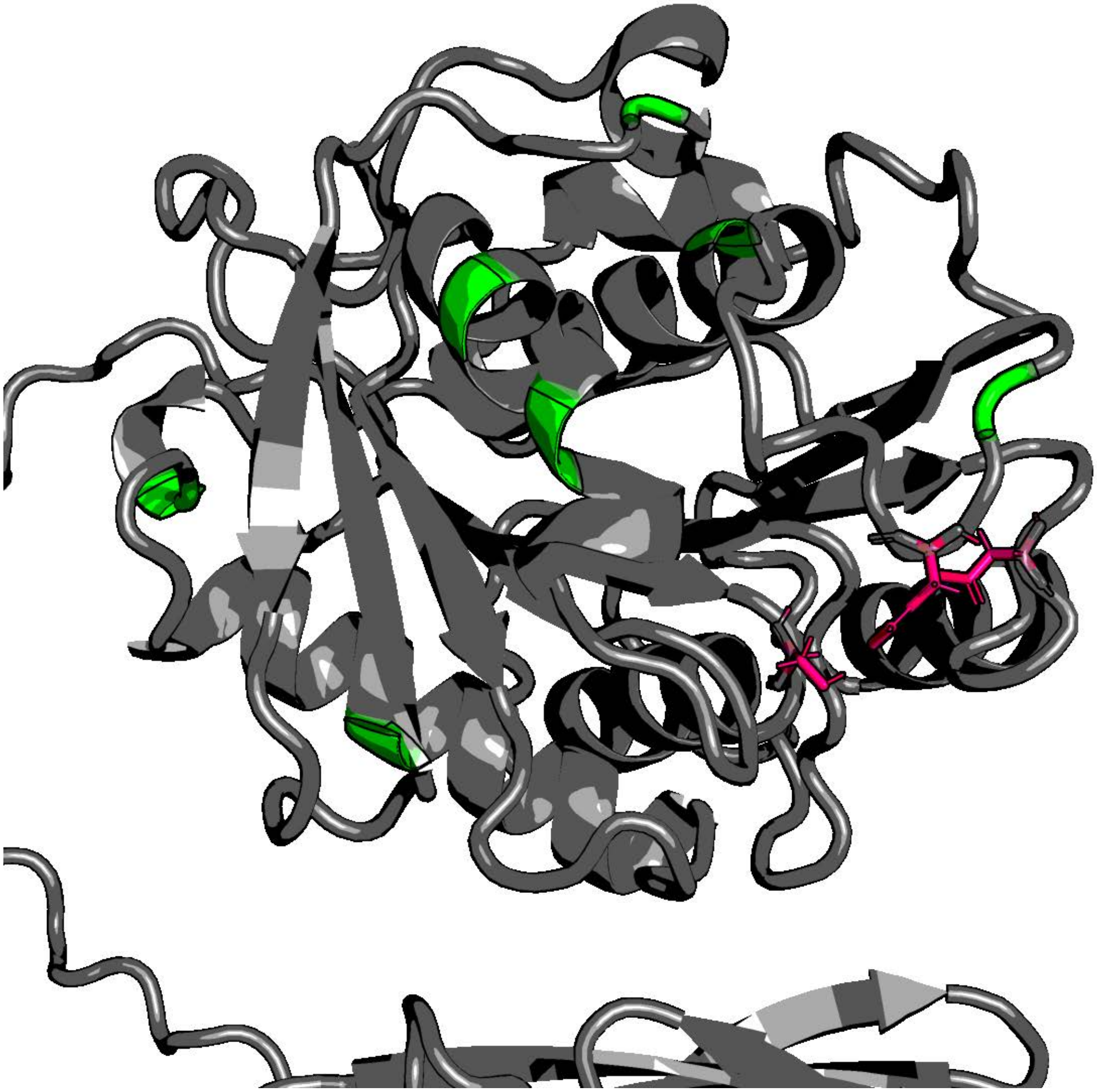

TEP013_highlights.png

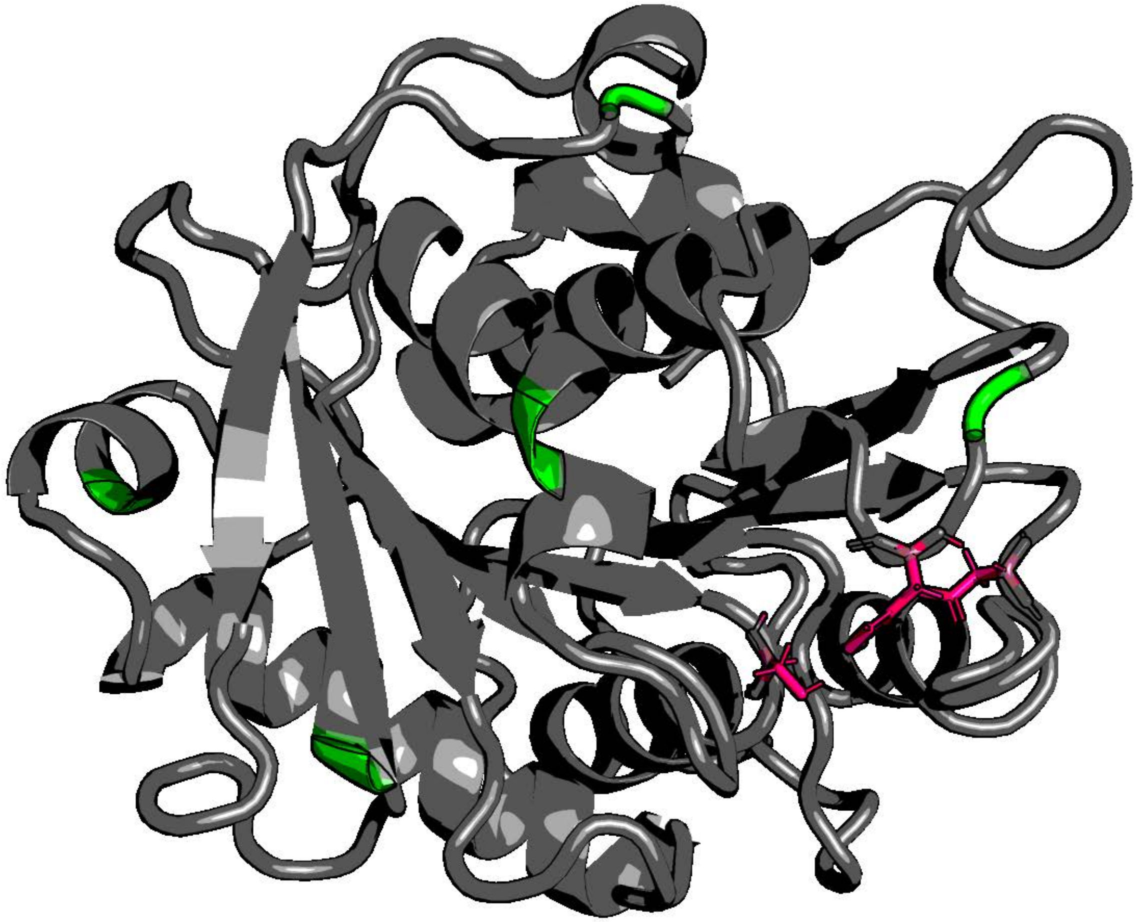

TEP014_highlights.png

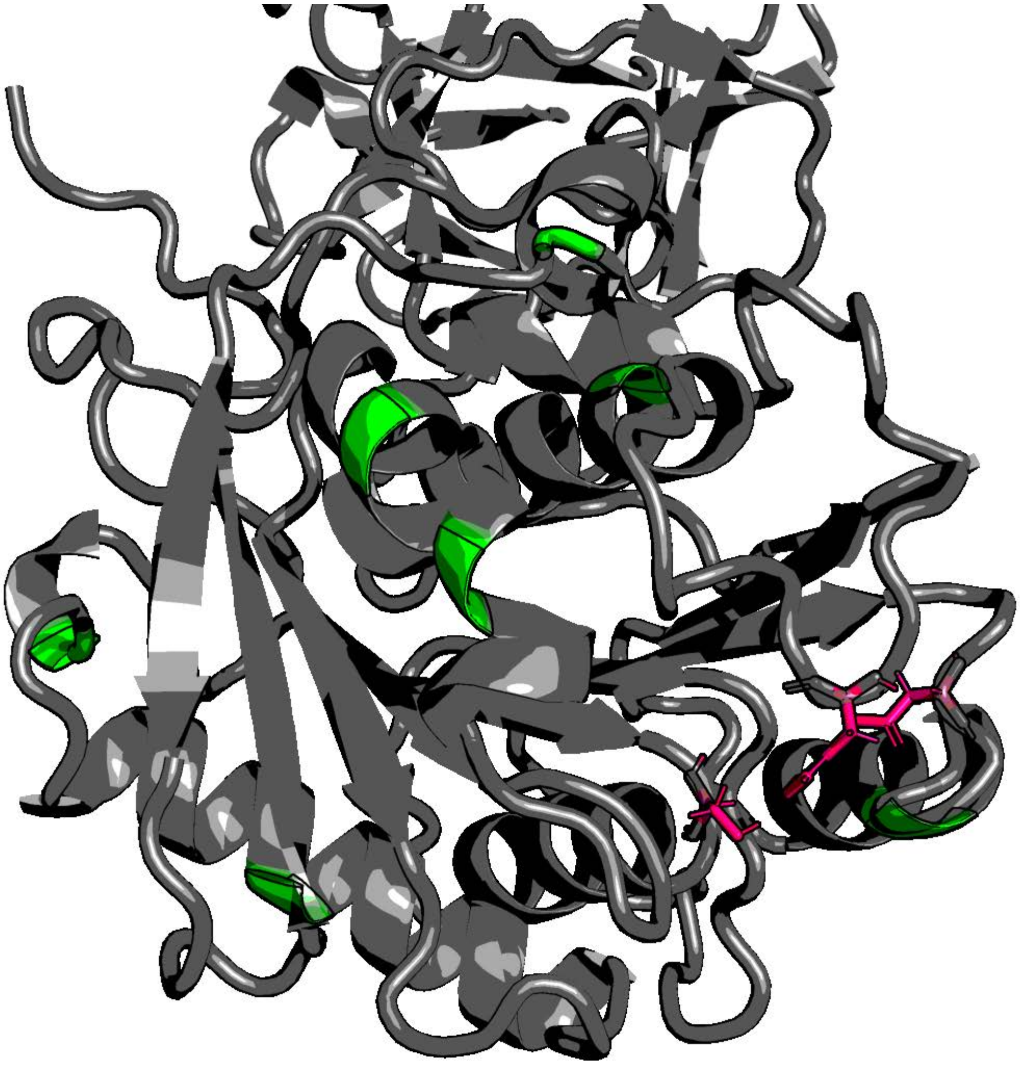

TEP024_highlights.png

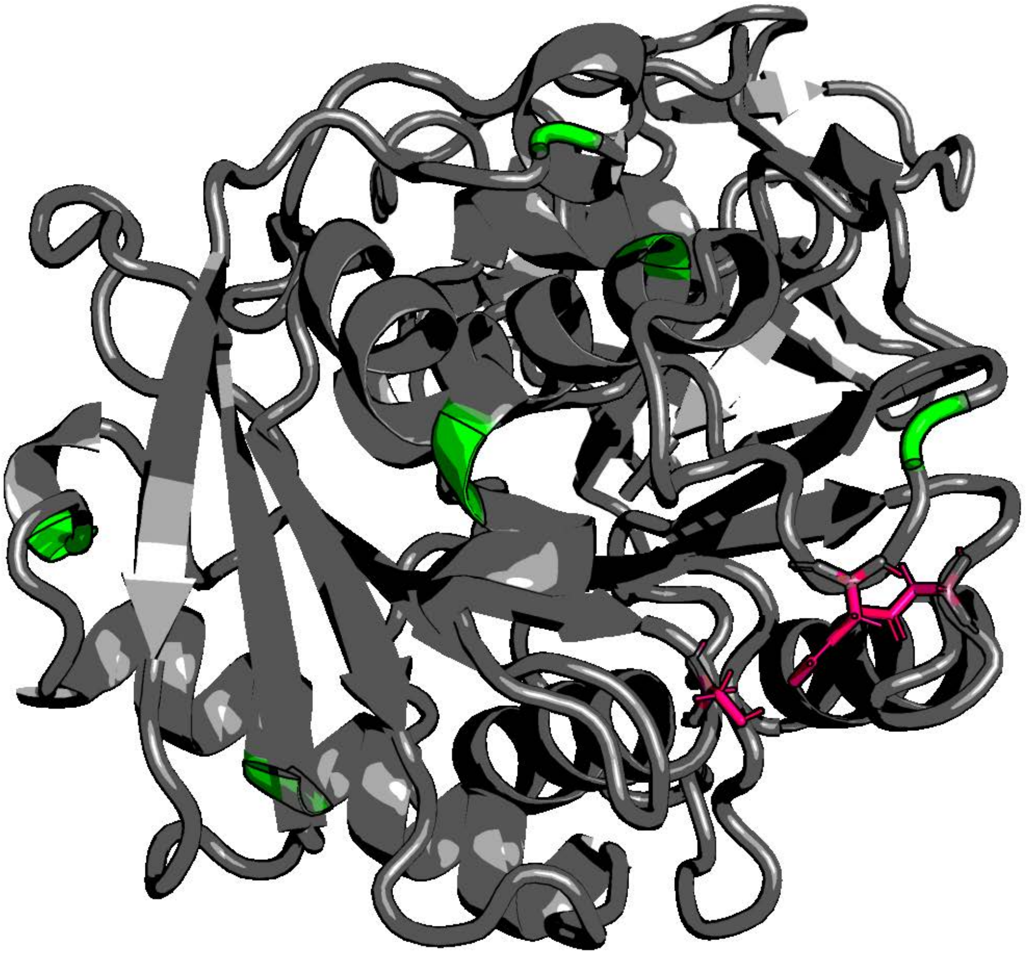

TEP029_highlights.png

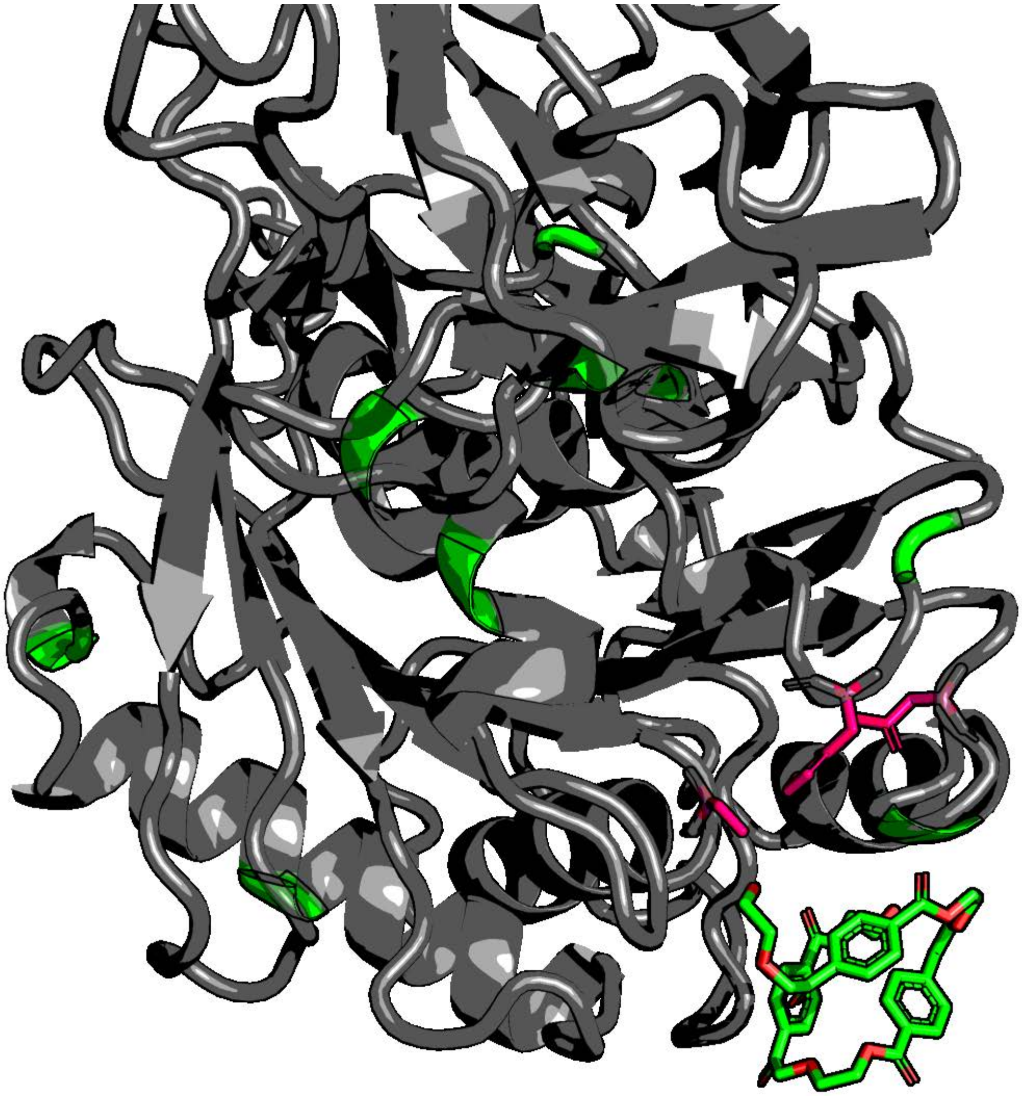

TEP054_highlights.png

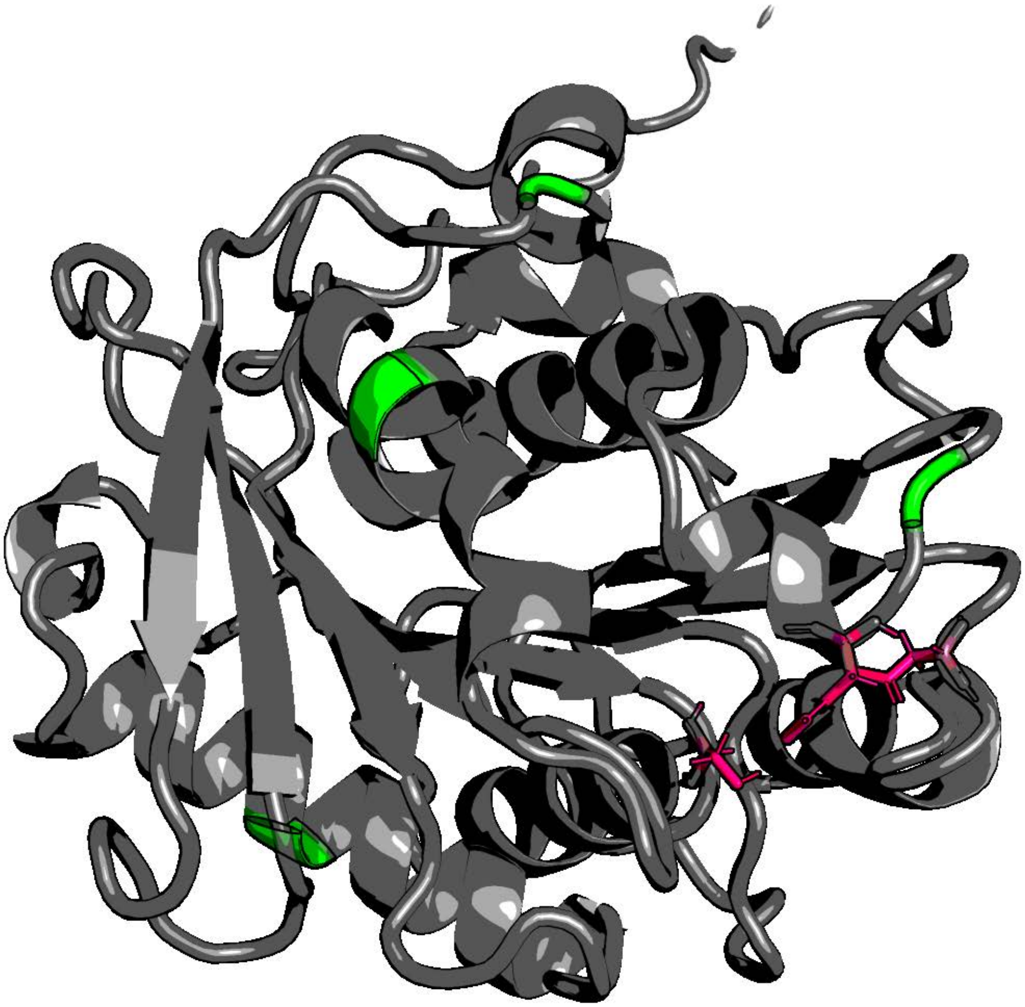

TEP067_highlights.png

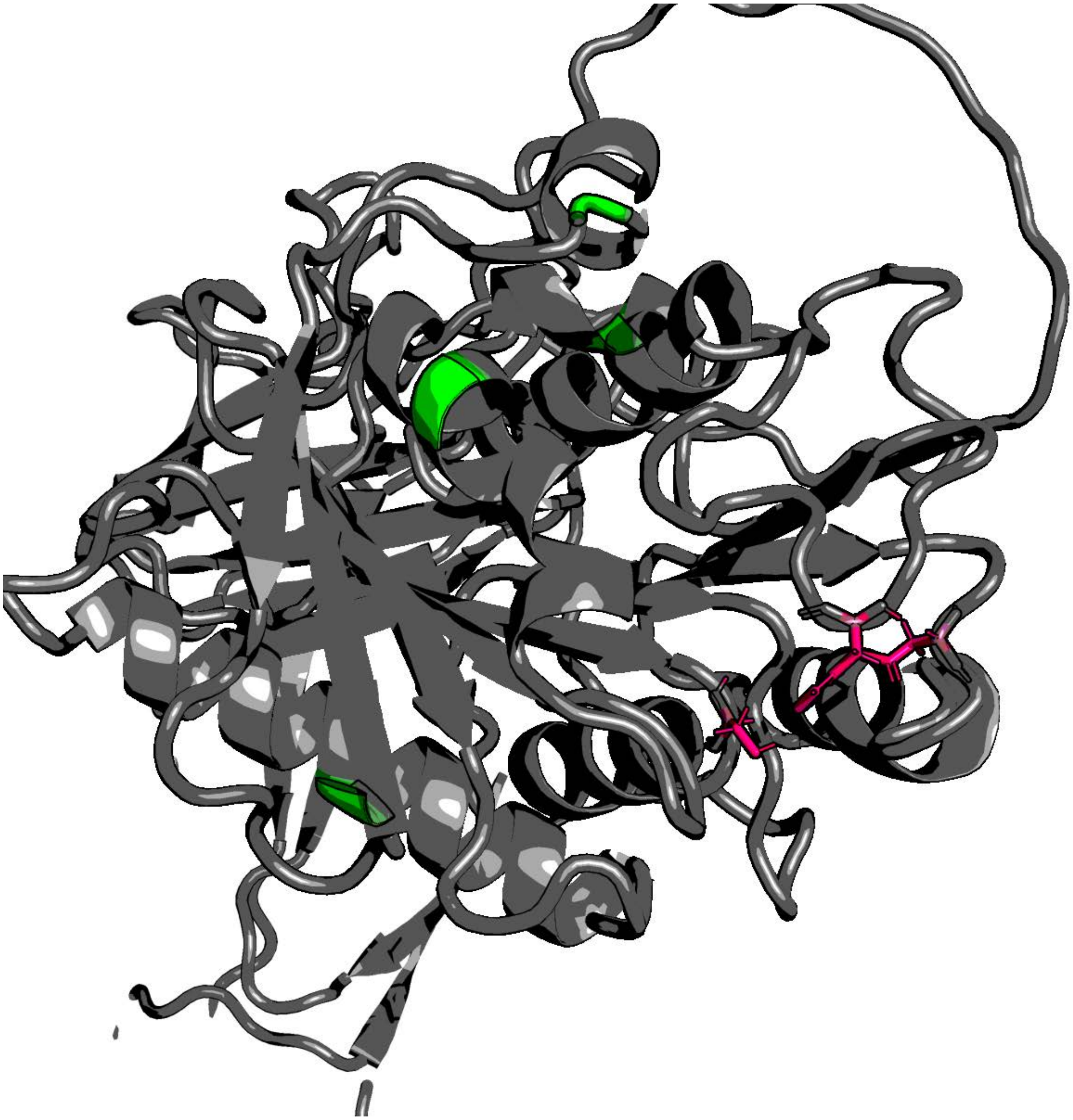

TEP080_highlights.png

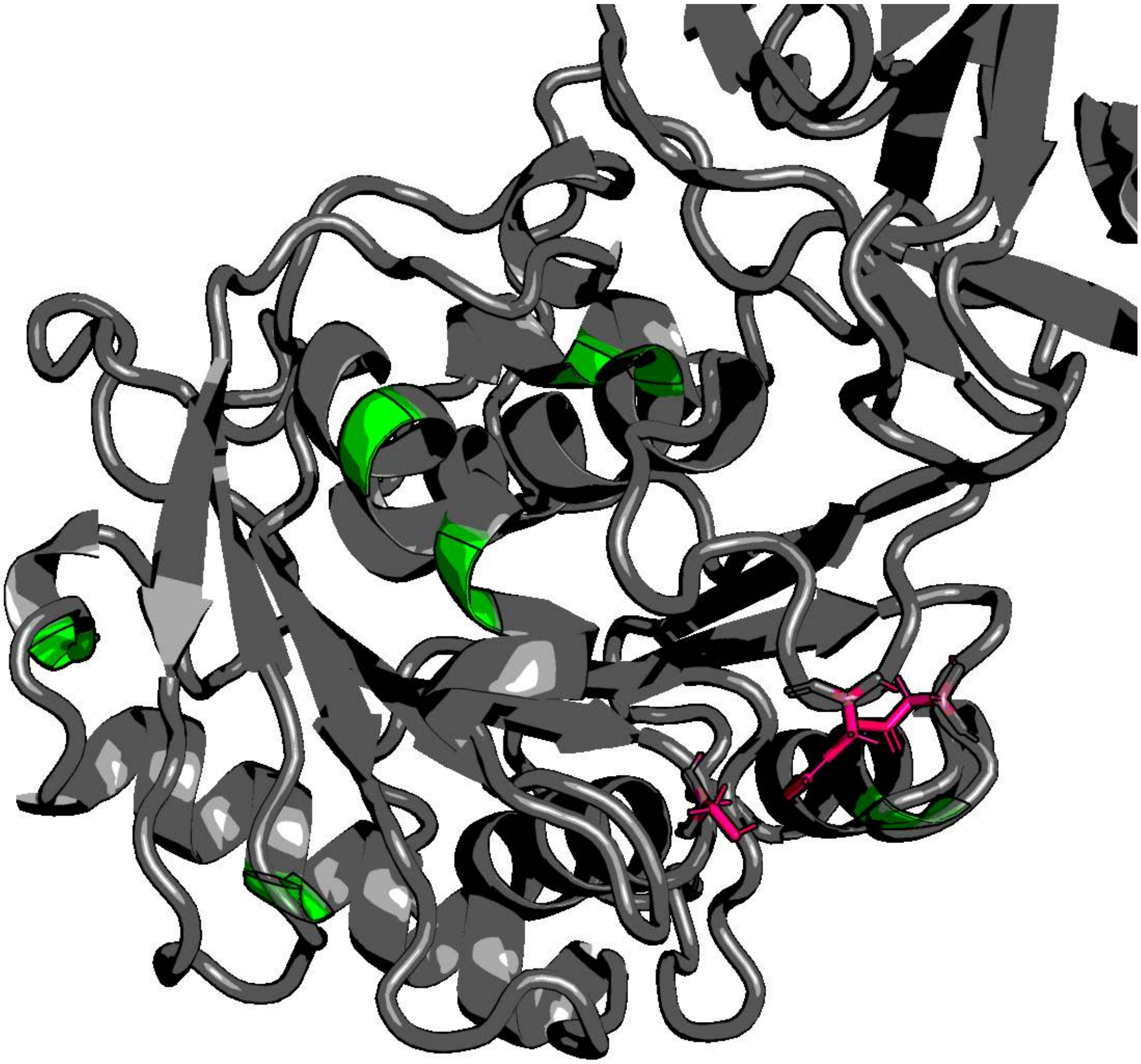

TEP082_highlights.png

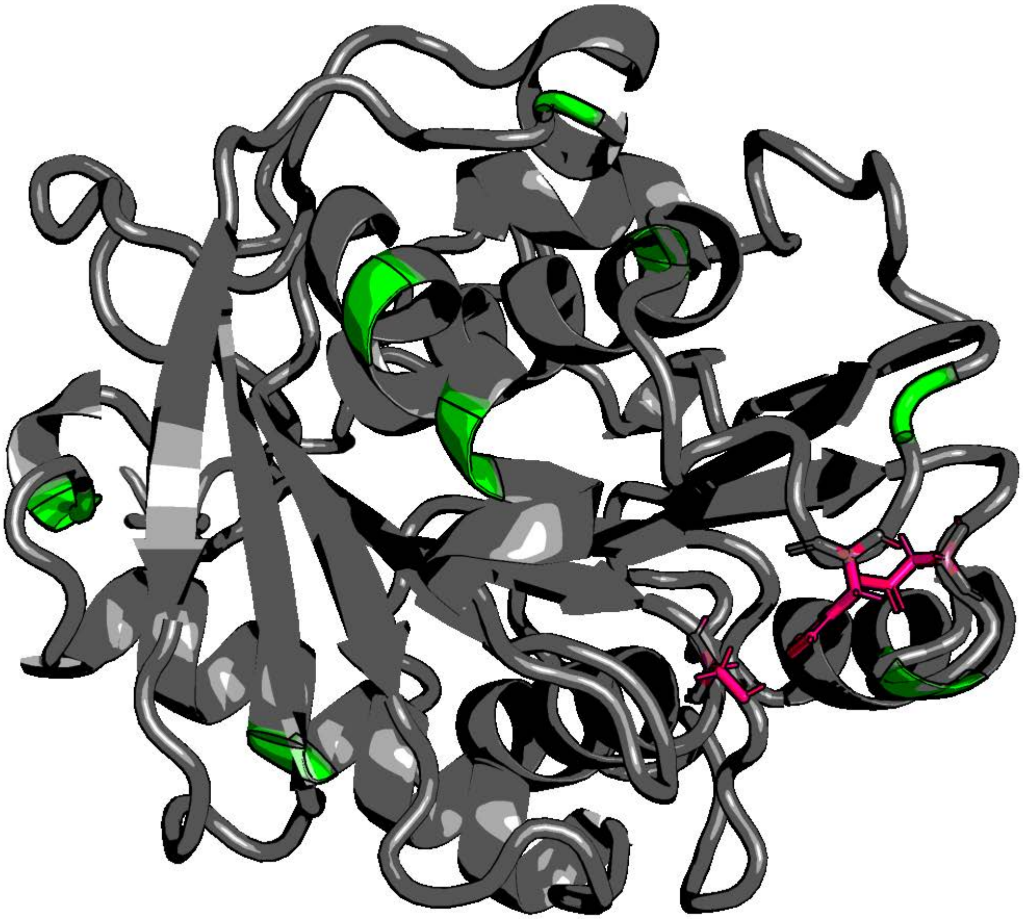

TEP083_highlights.png

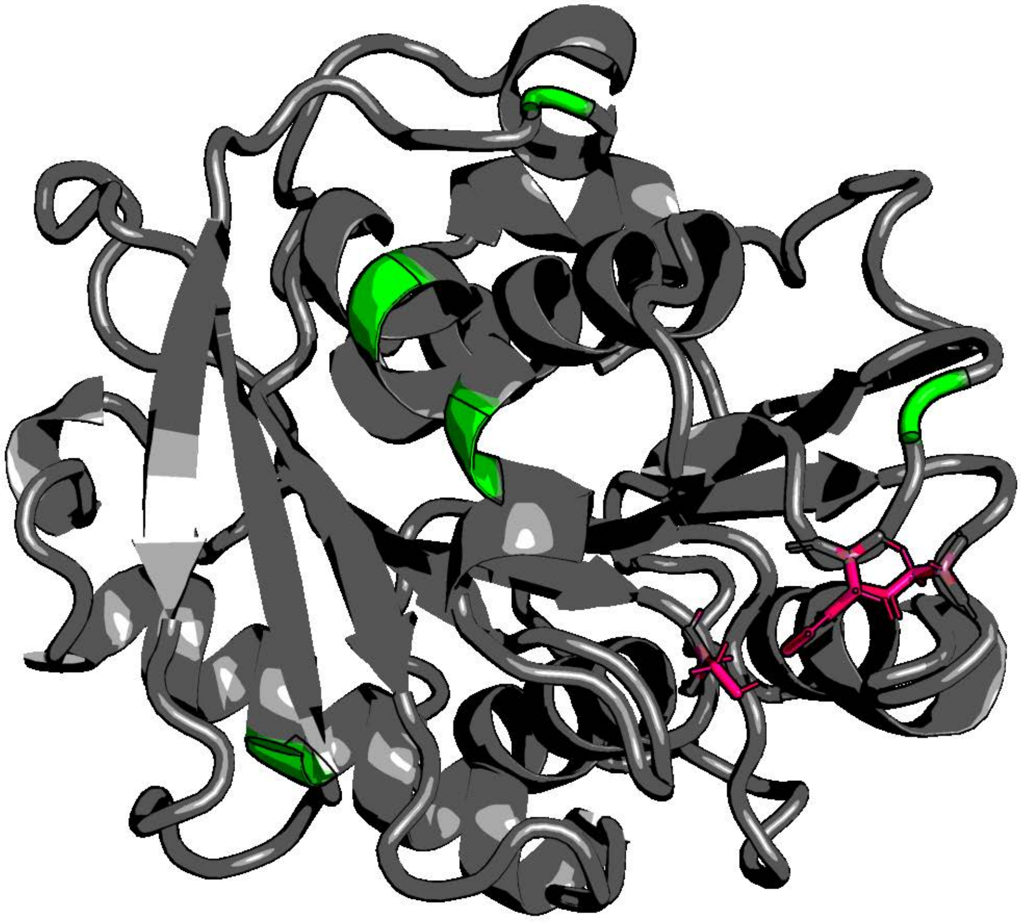

TEP091_highlights.png

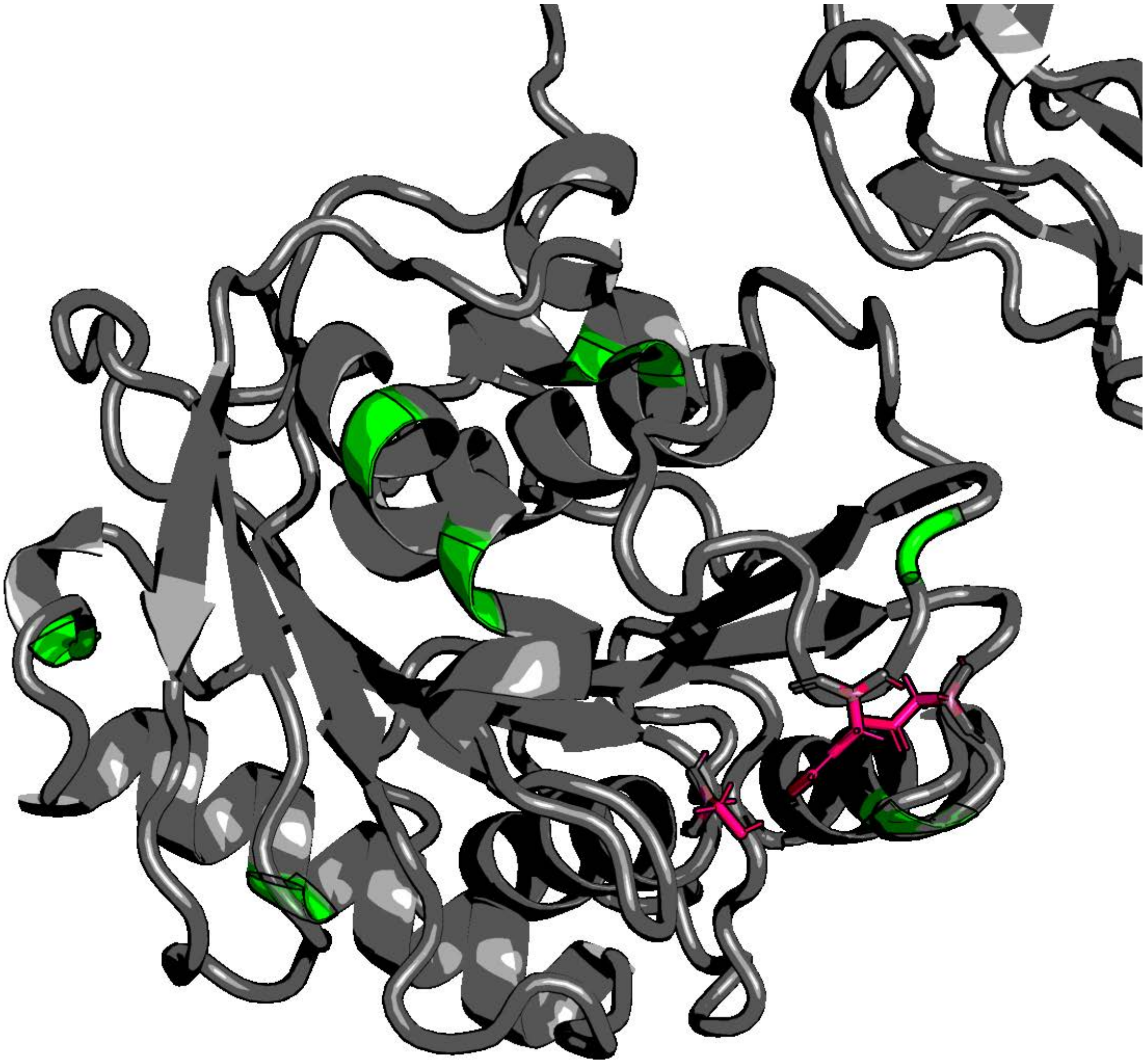

TEP092_highlights.png

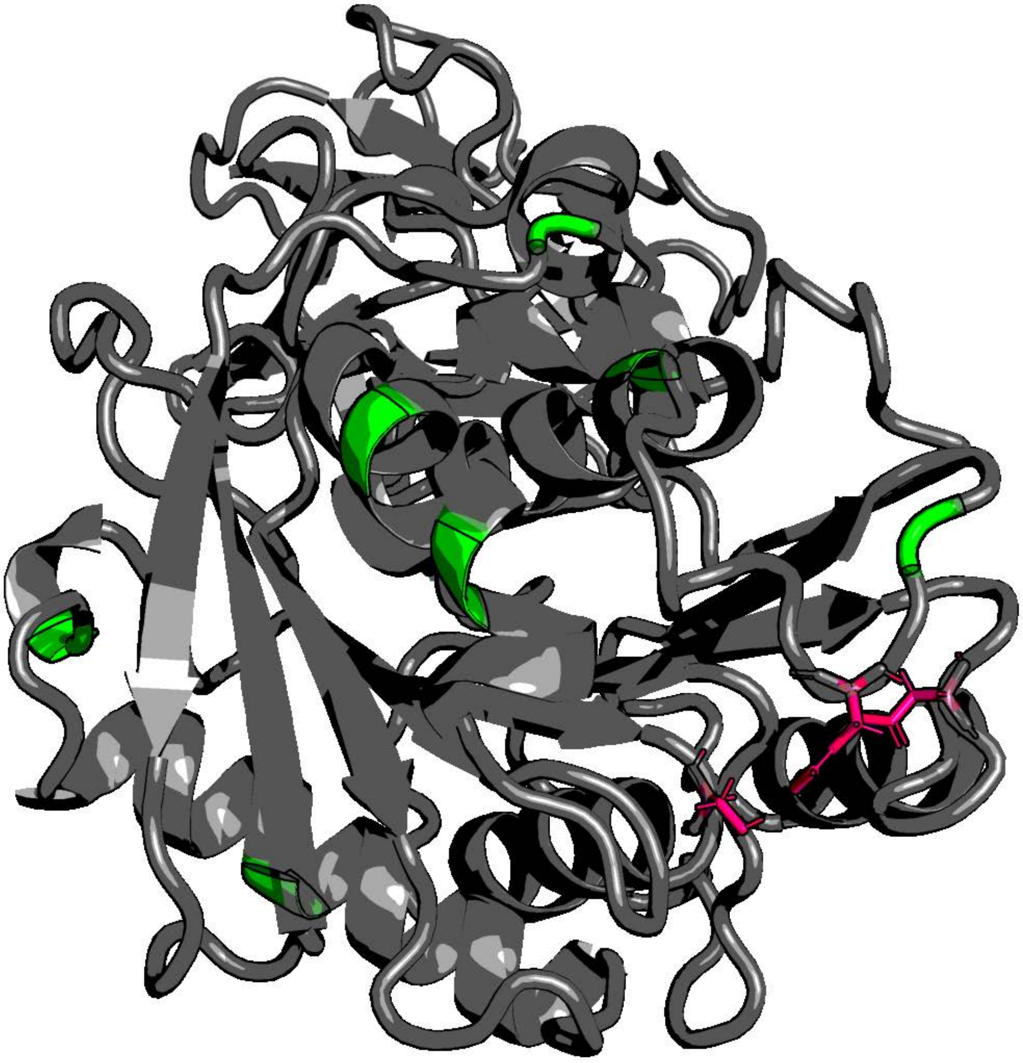

TEP093_highlights.png

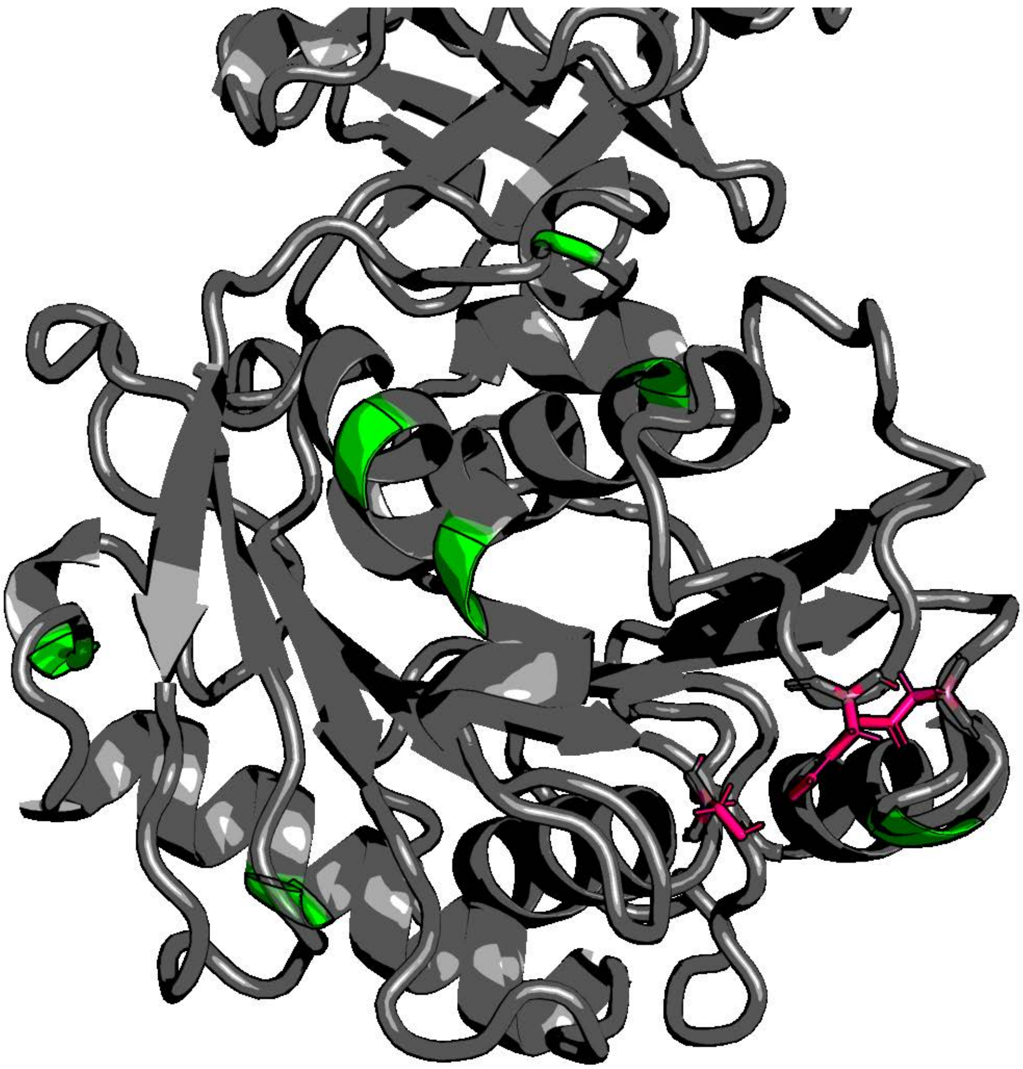

TEP097_highlights.png

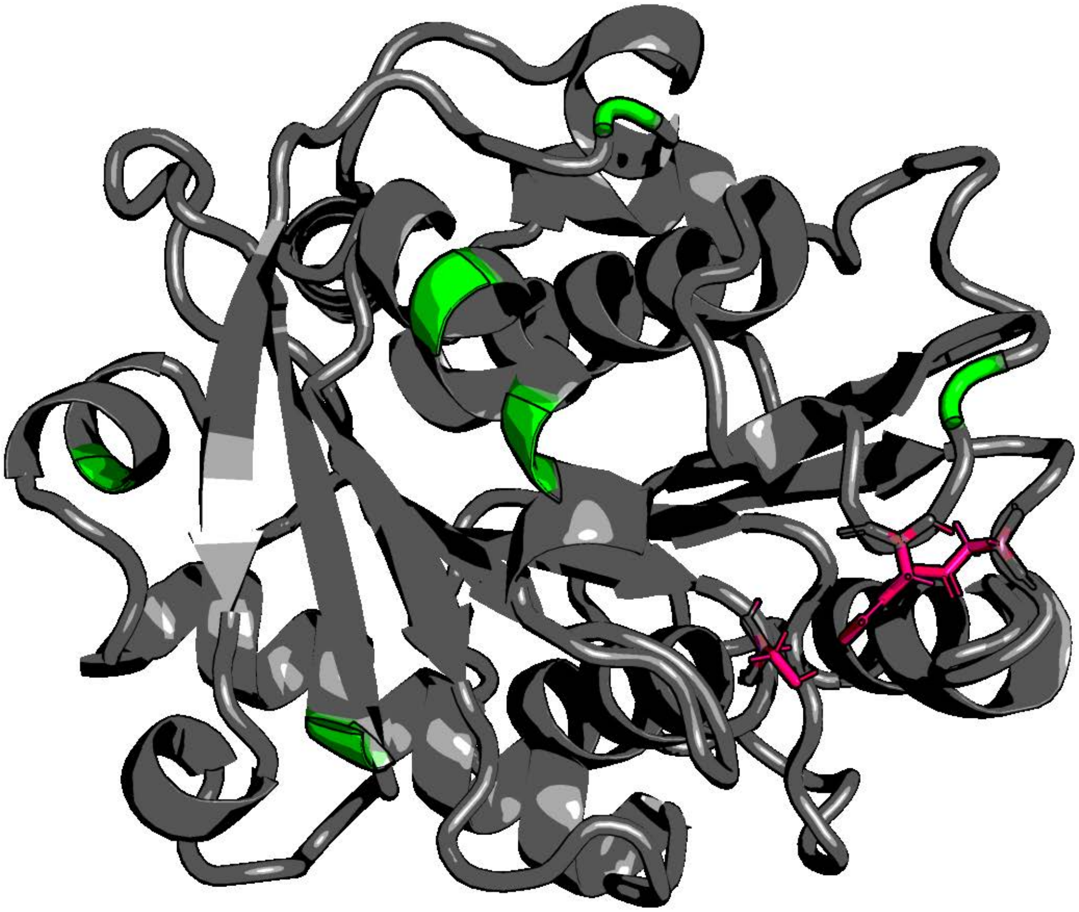

TEP101_highlights.png

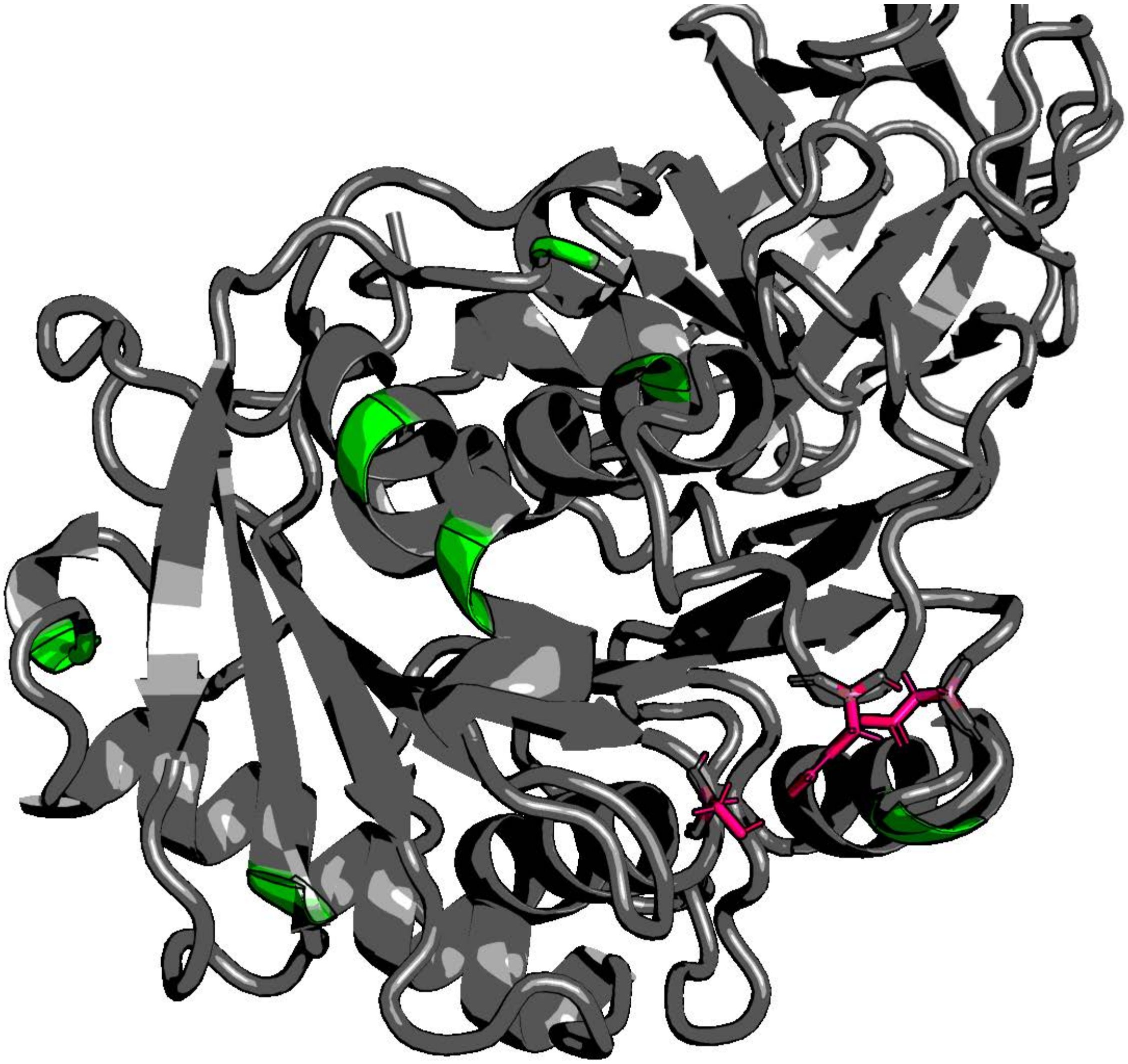

TEP102_highlights.png

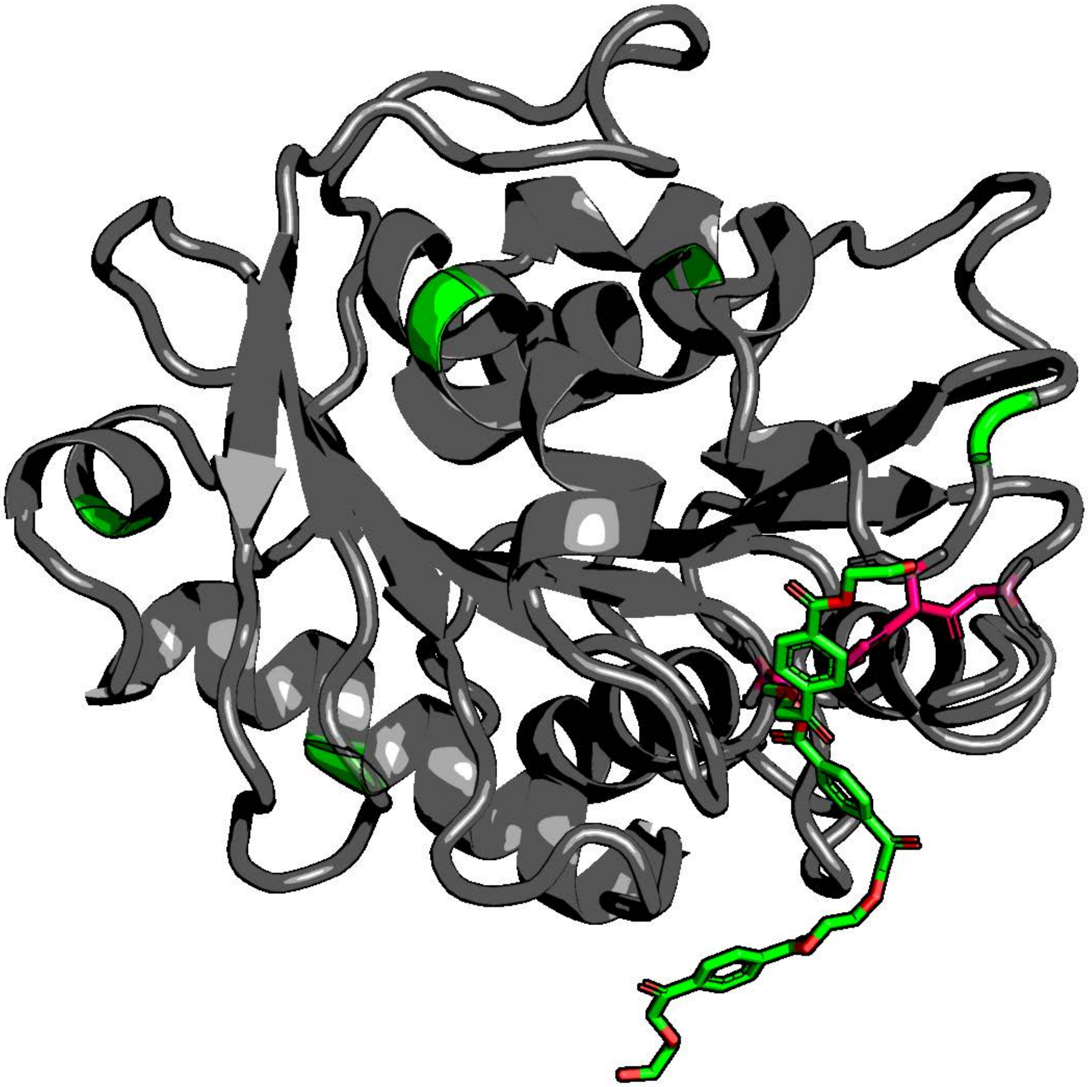

TEP103_highlights.png

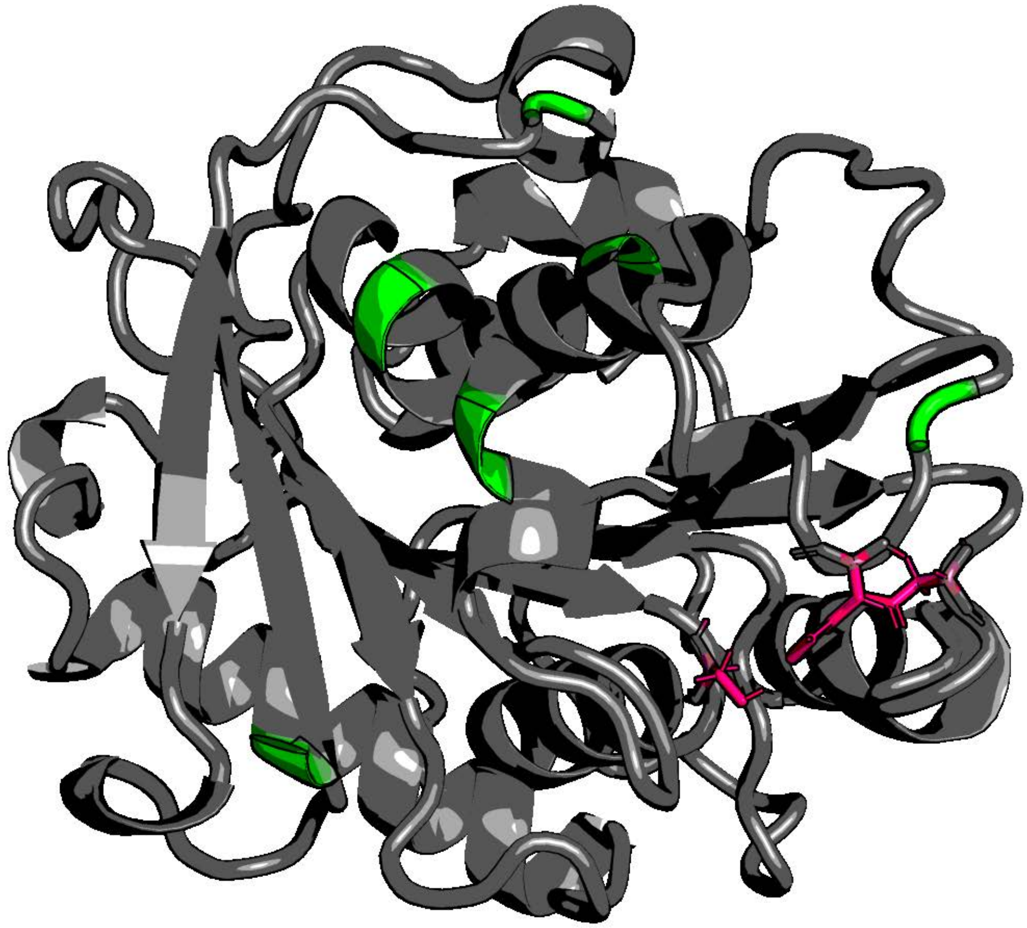

TEP105_highlights.png

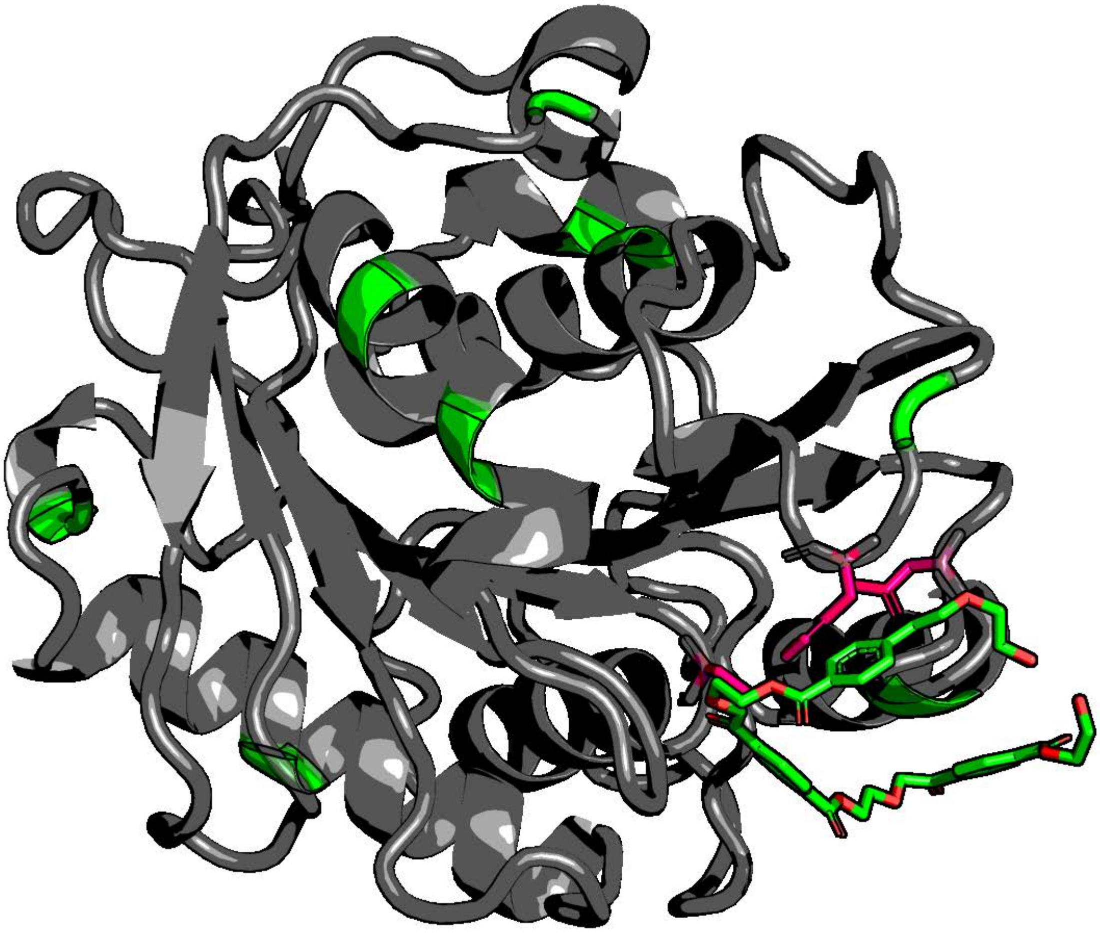

TEP109_highlights.png

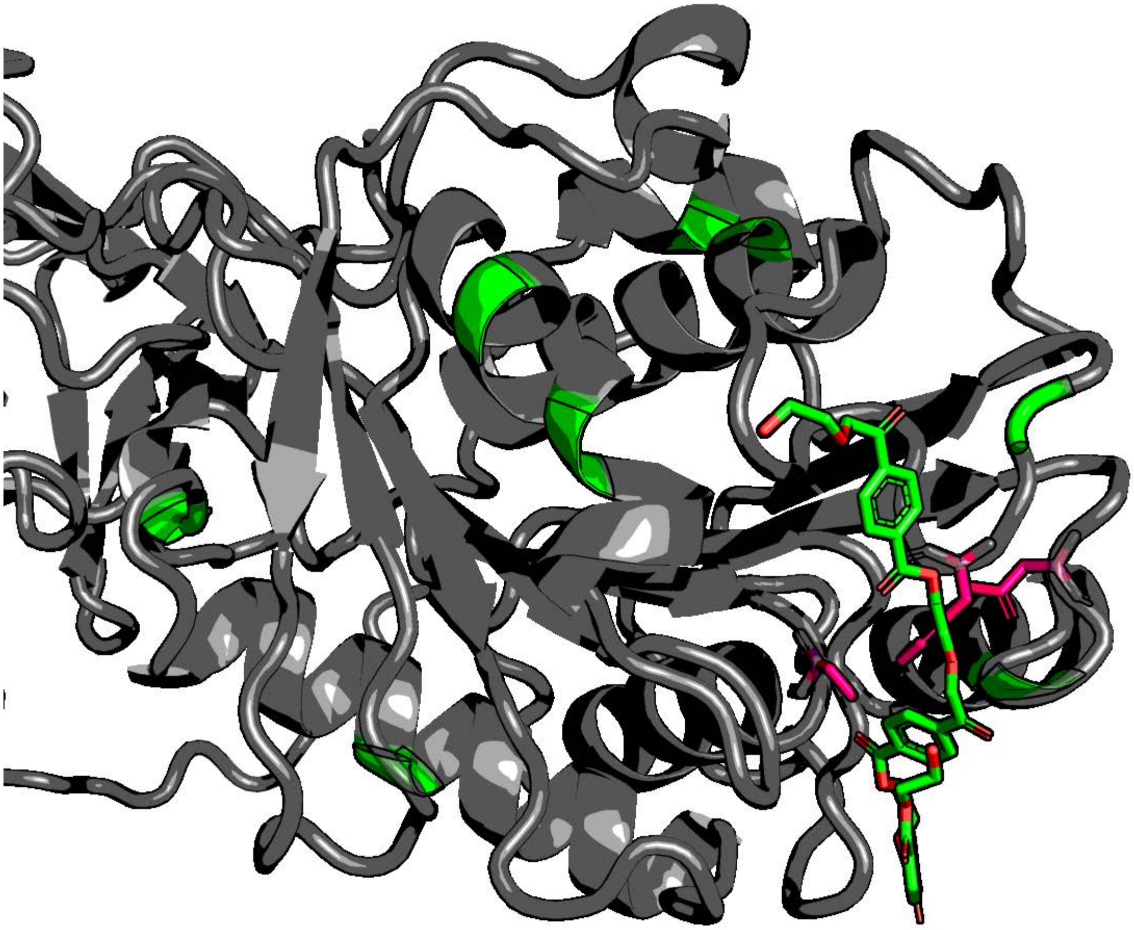

TEP111_highlights.png

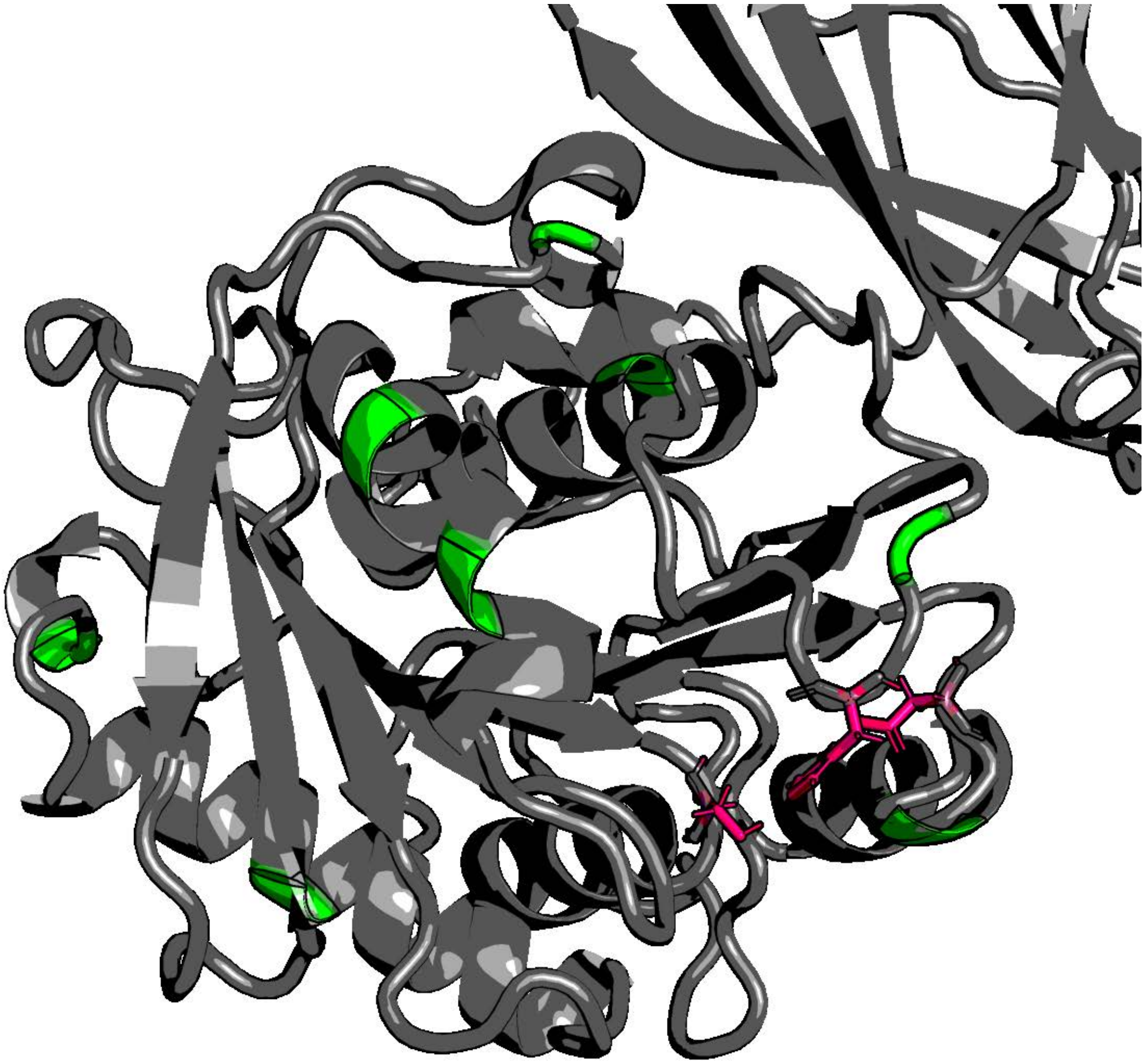

TEP114_highlights.png

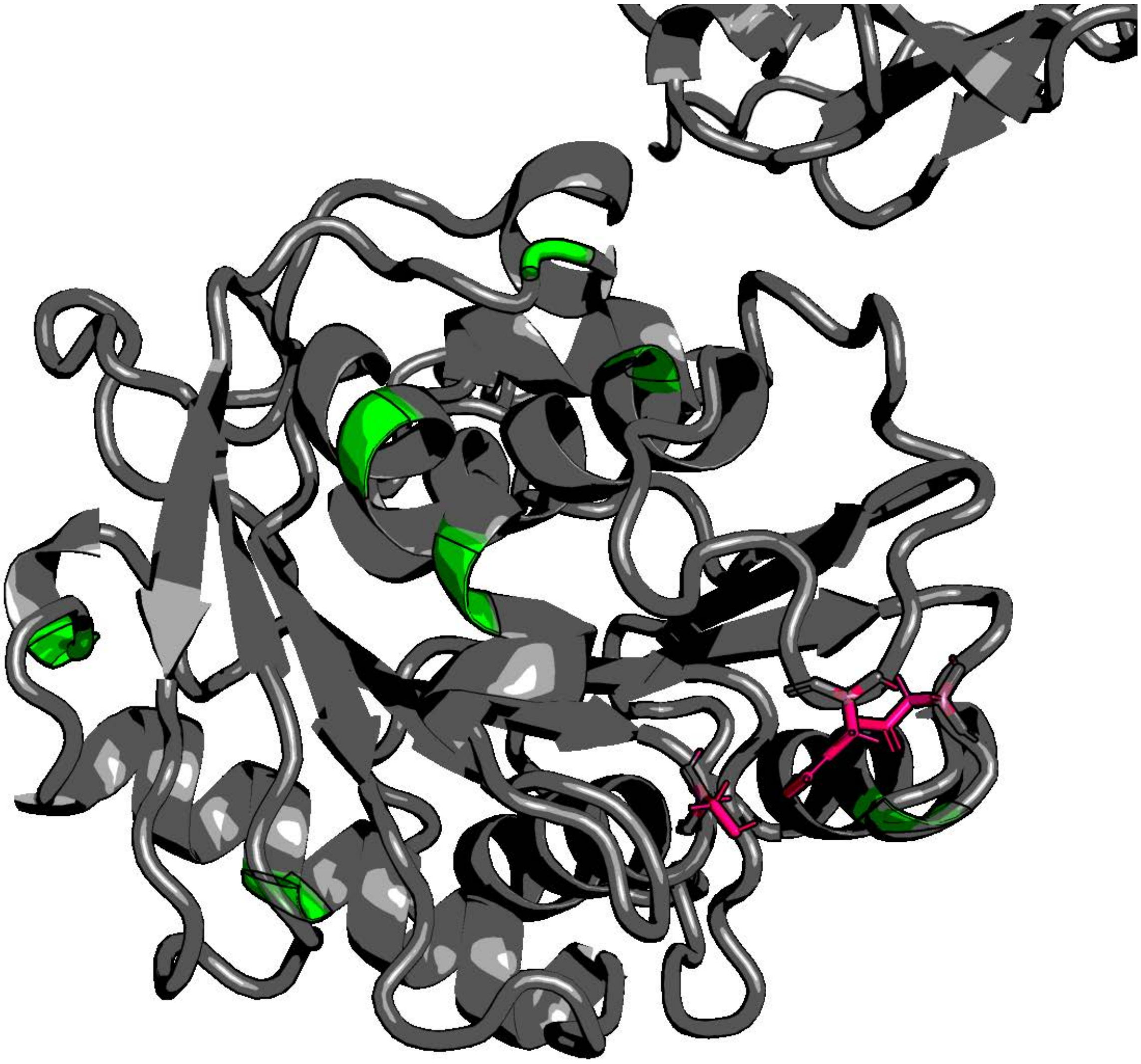

TEP115_highlights.png

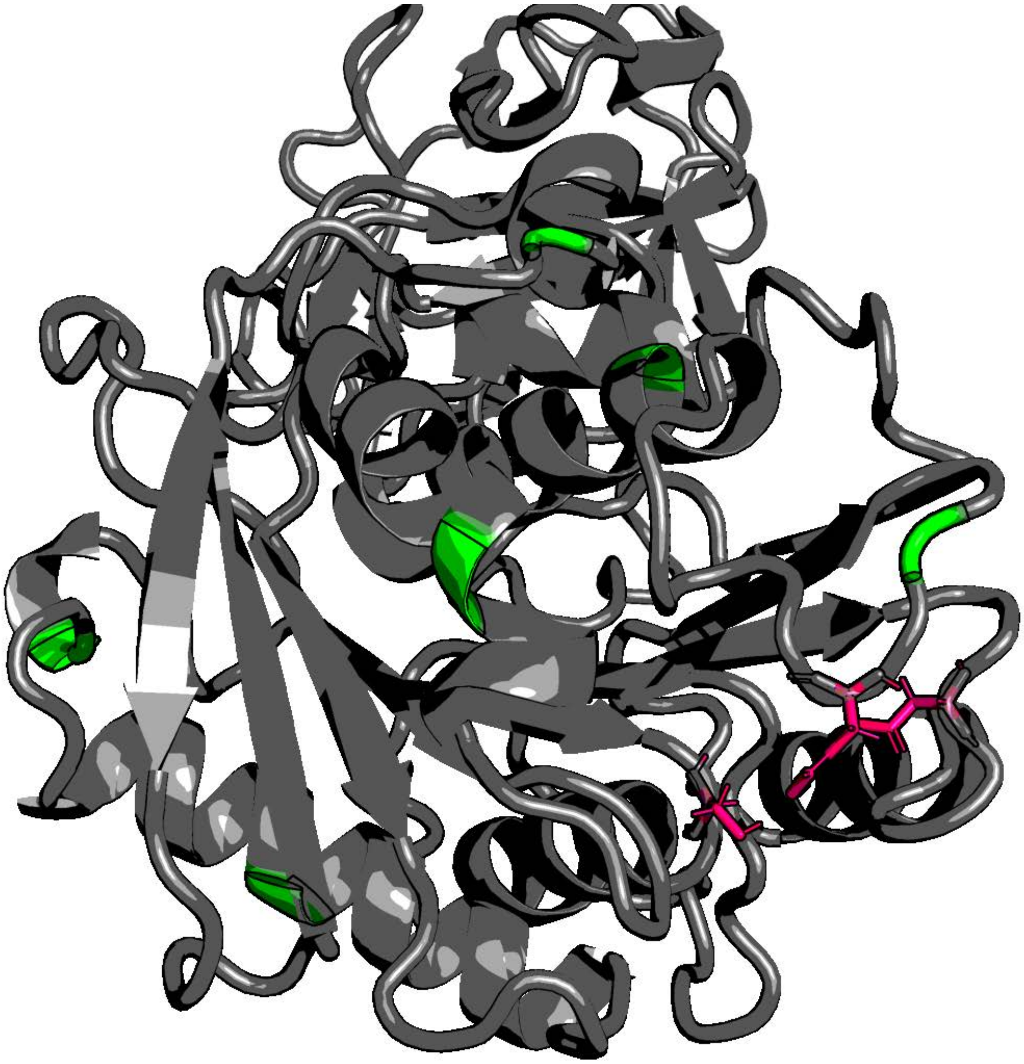

TEP131_highlights.png

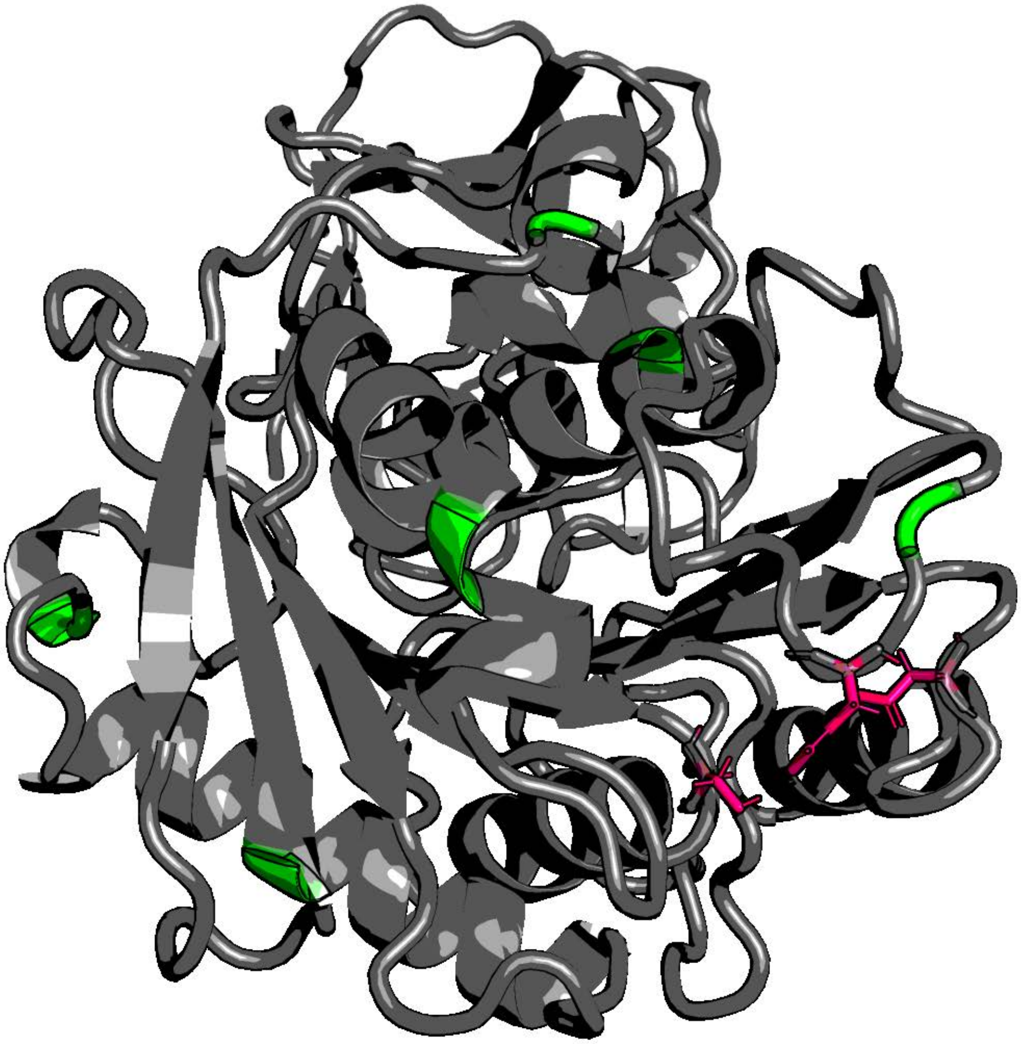

TEP163_highlights.png

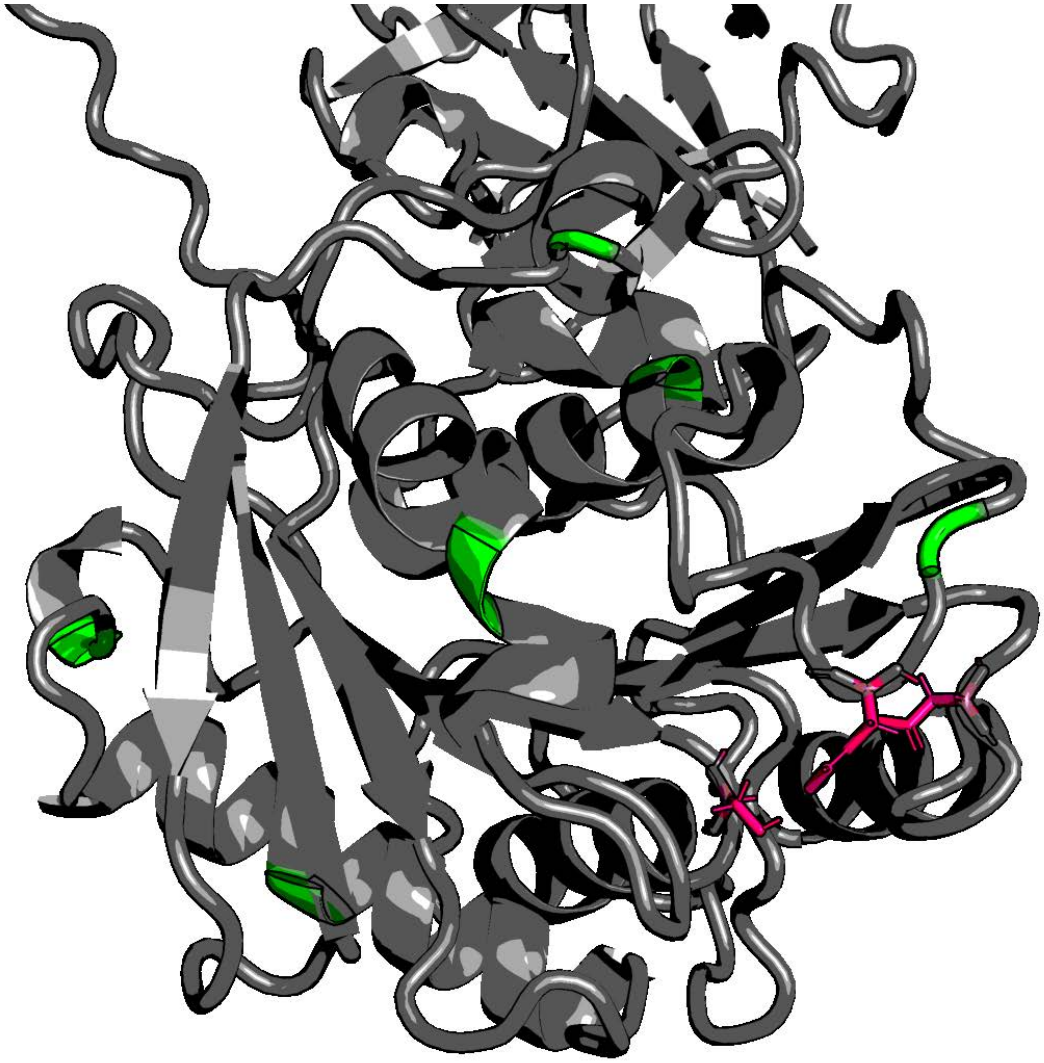

TEP164_highlights.png

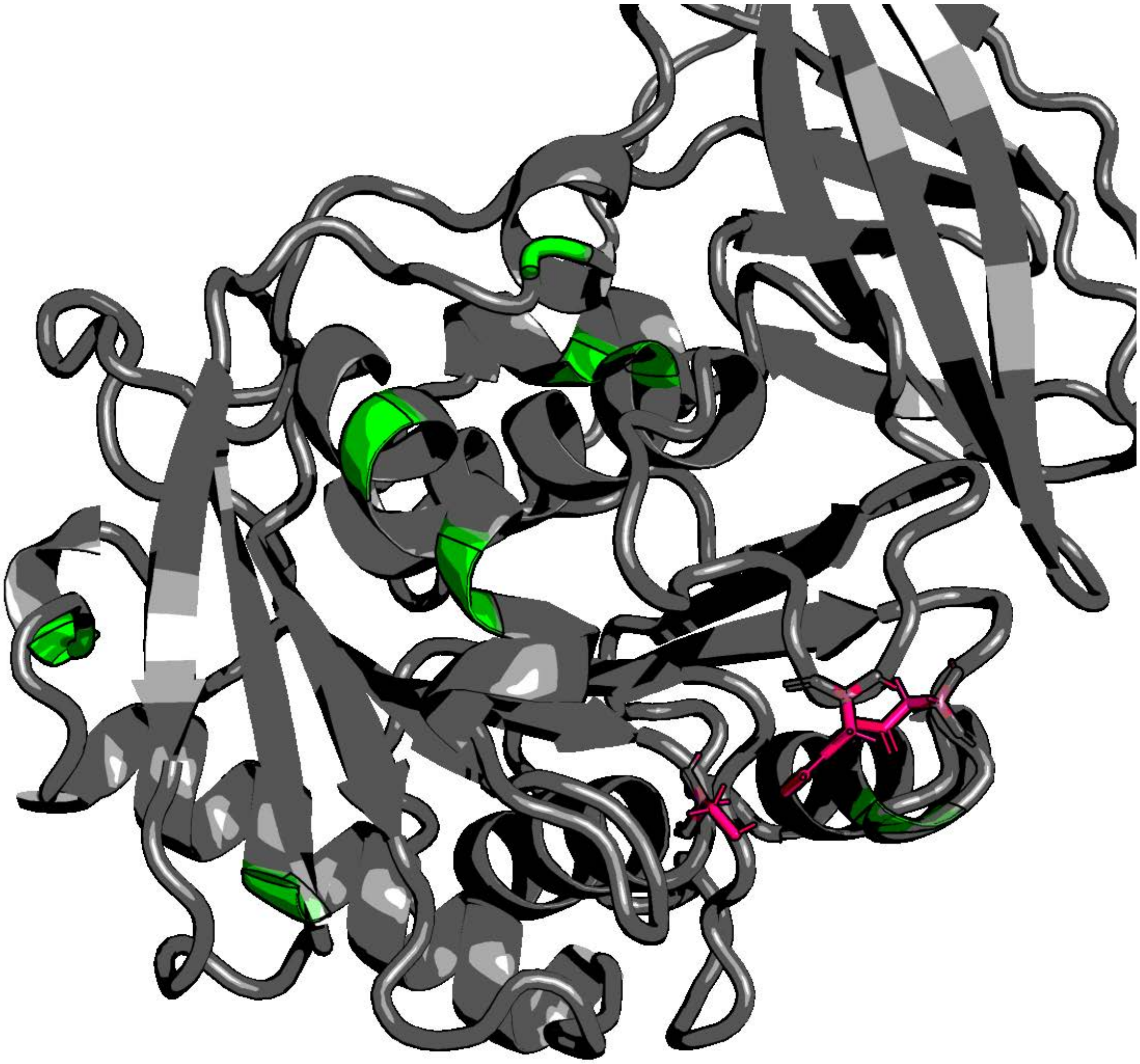

TEP165_highlights.png

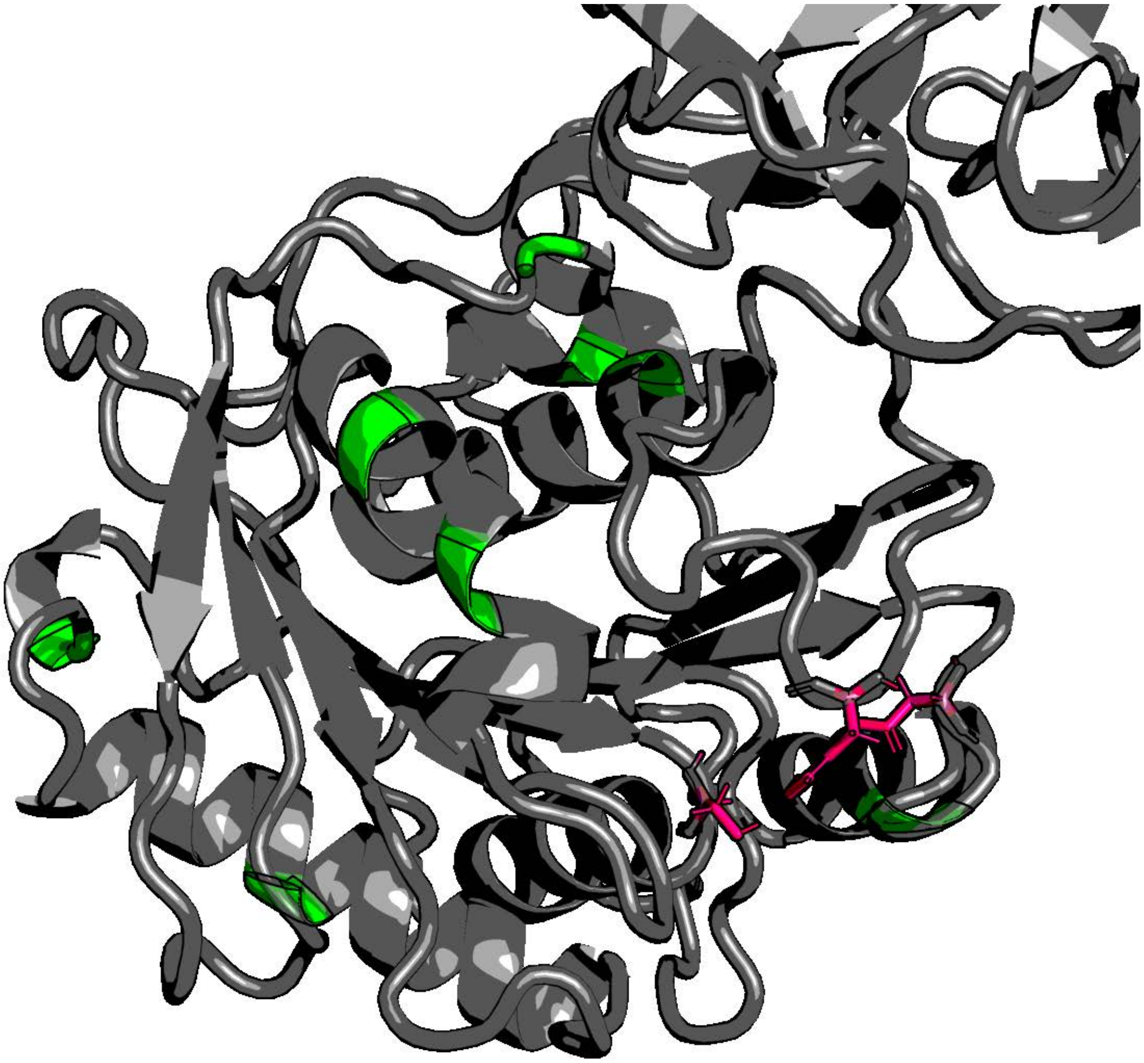

TEP168_highlights.png

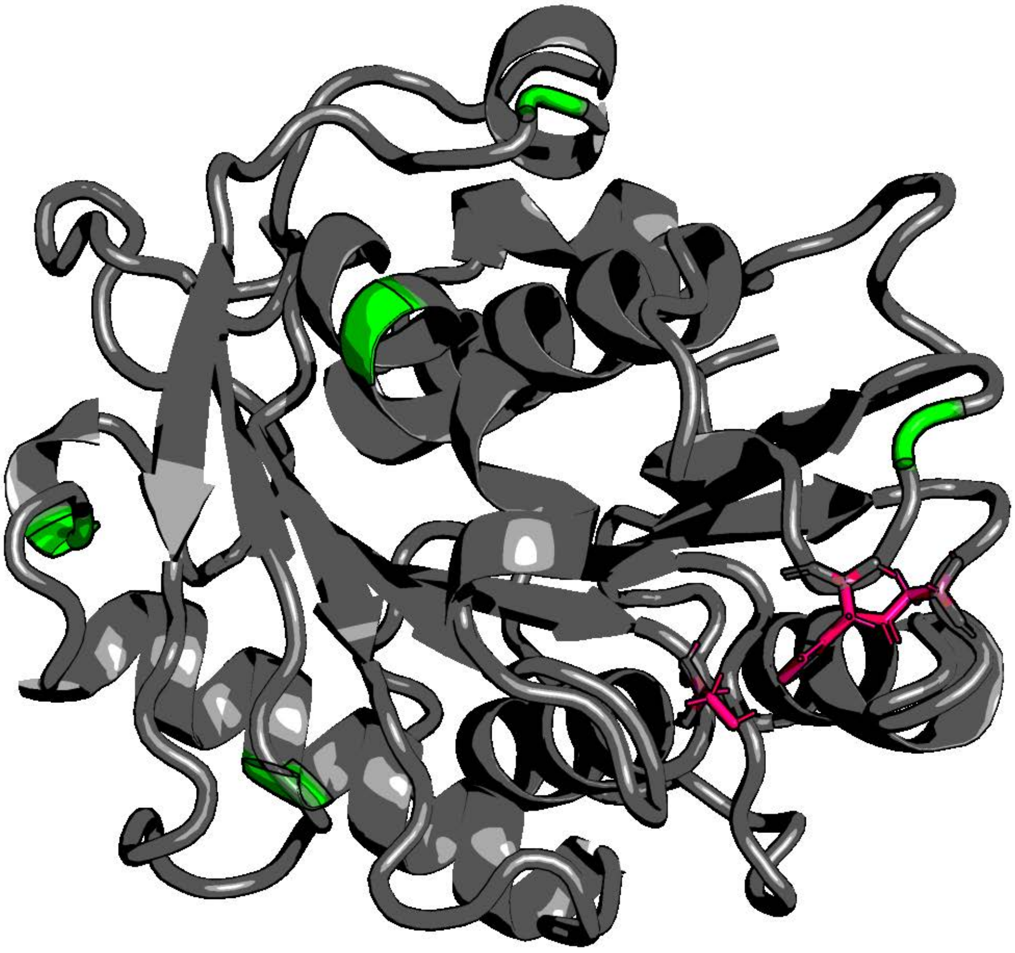

TEP178_highlights.png

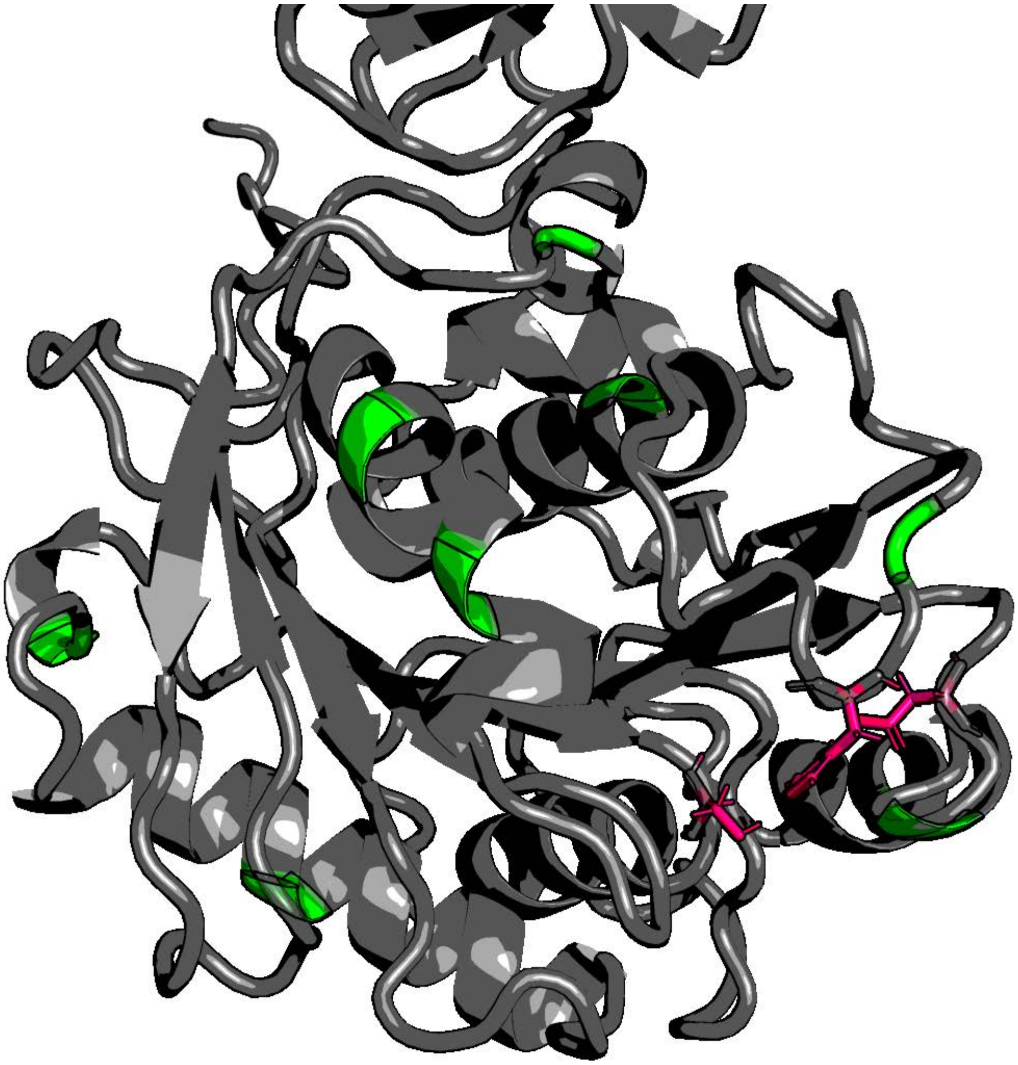

TEP181_highlights.png

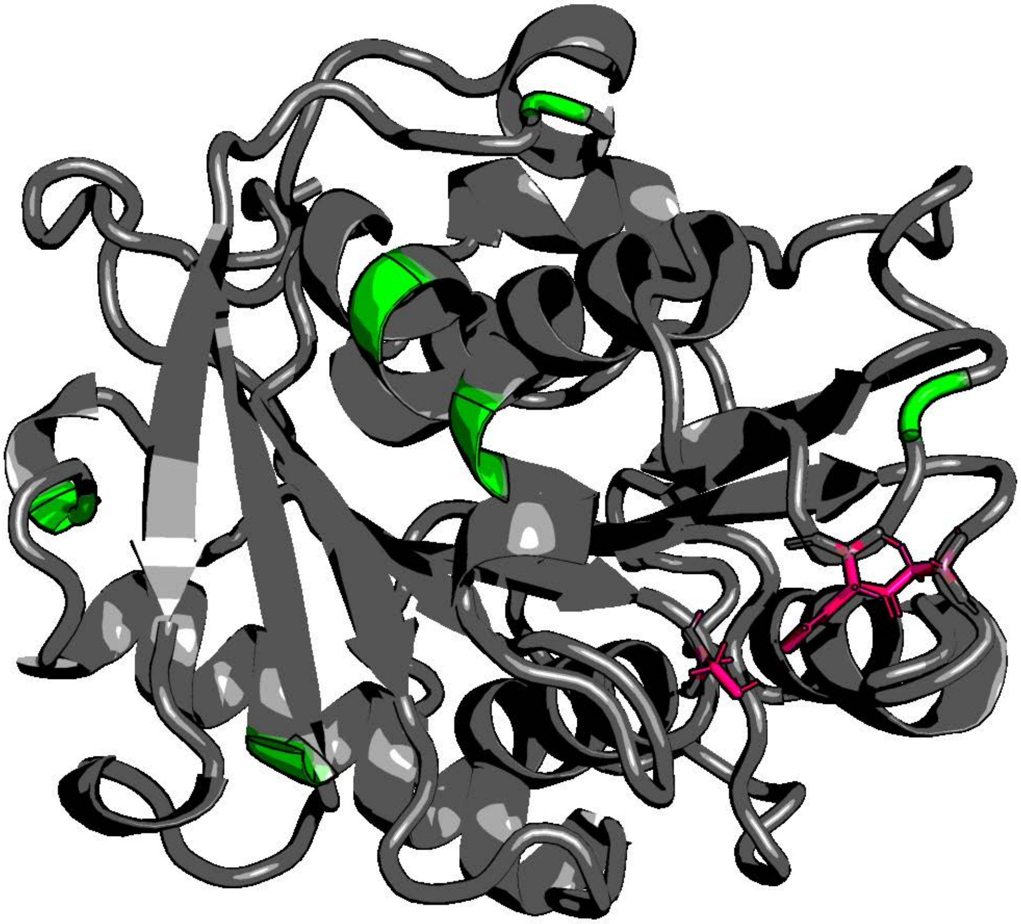

TEP182_highlights.png

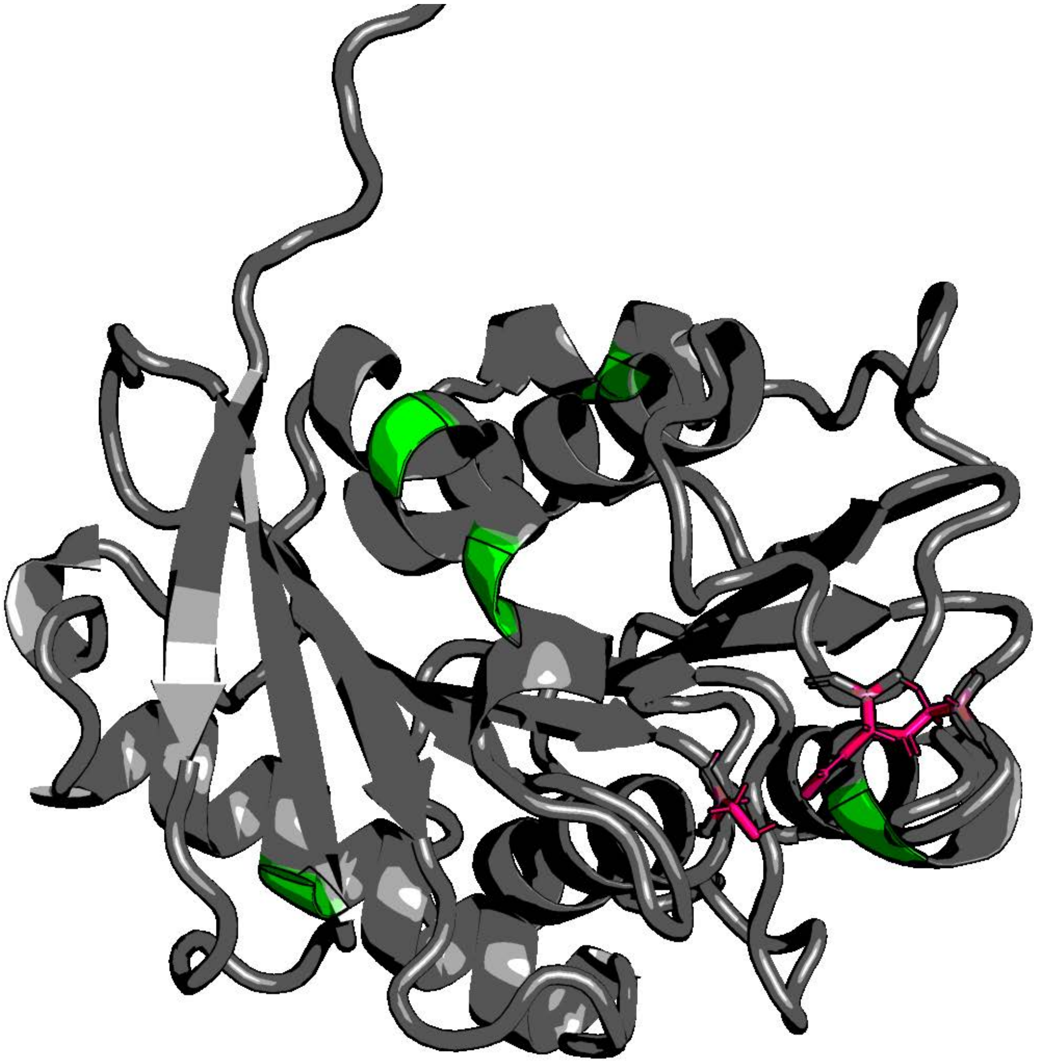

TEP189_highlights.png

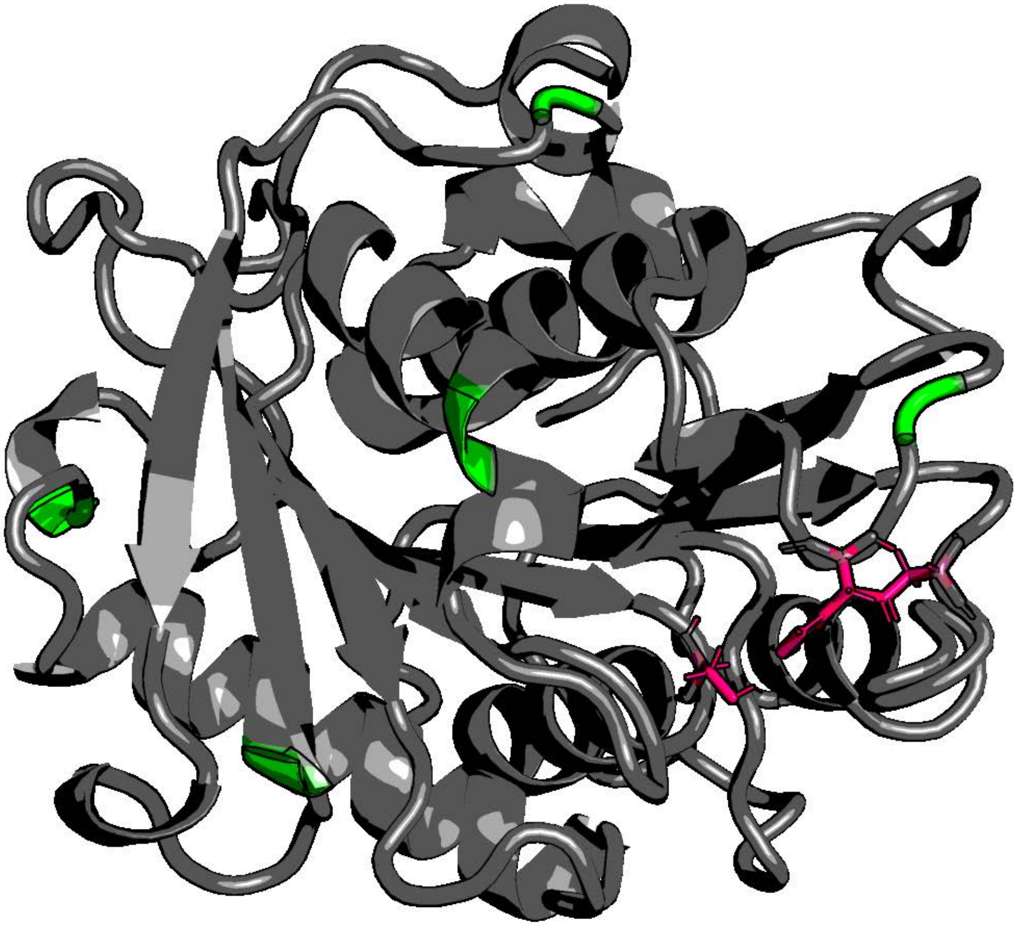

TEP194_highlights.png

Observed predicted pKa

Created on: 2025-04-15 16:06:06

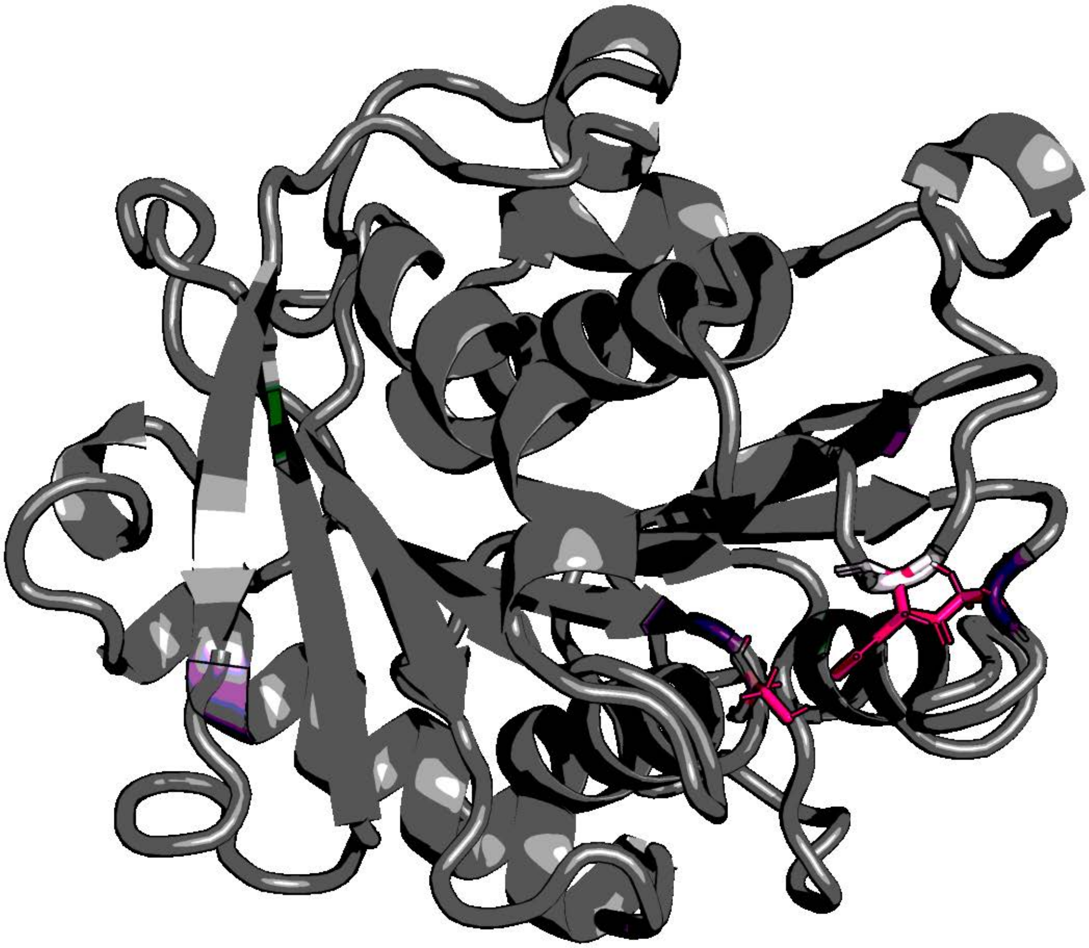

DP003_highlights.png

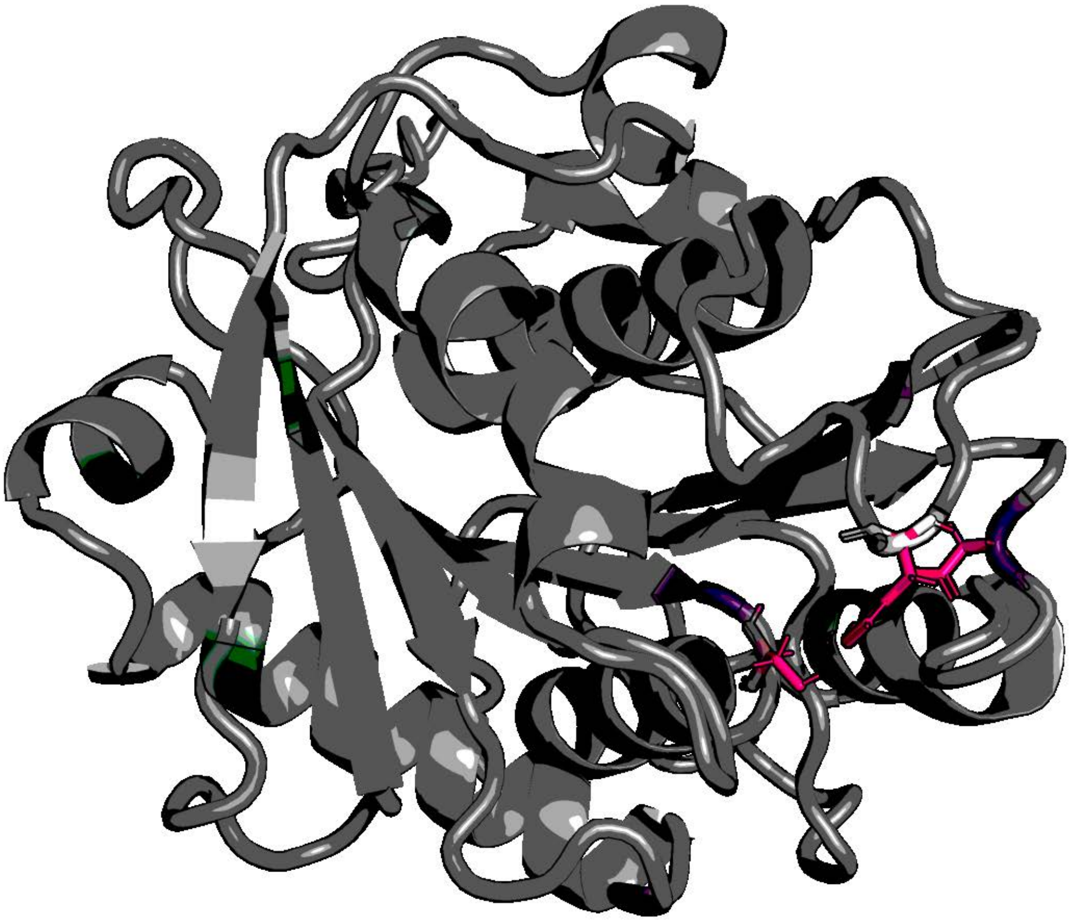

DP007_highlights.png

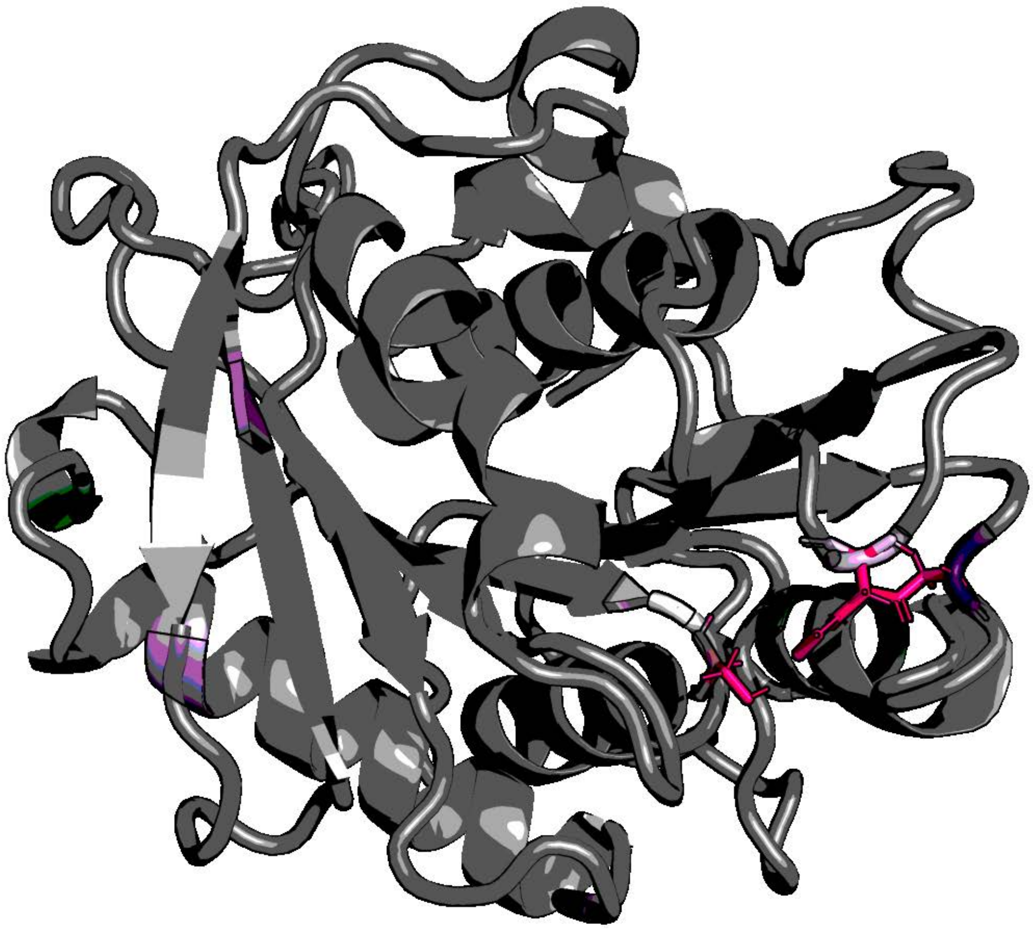

DP008_highlights.png

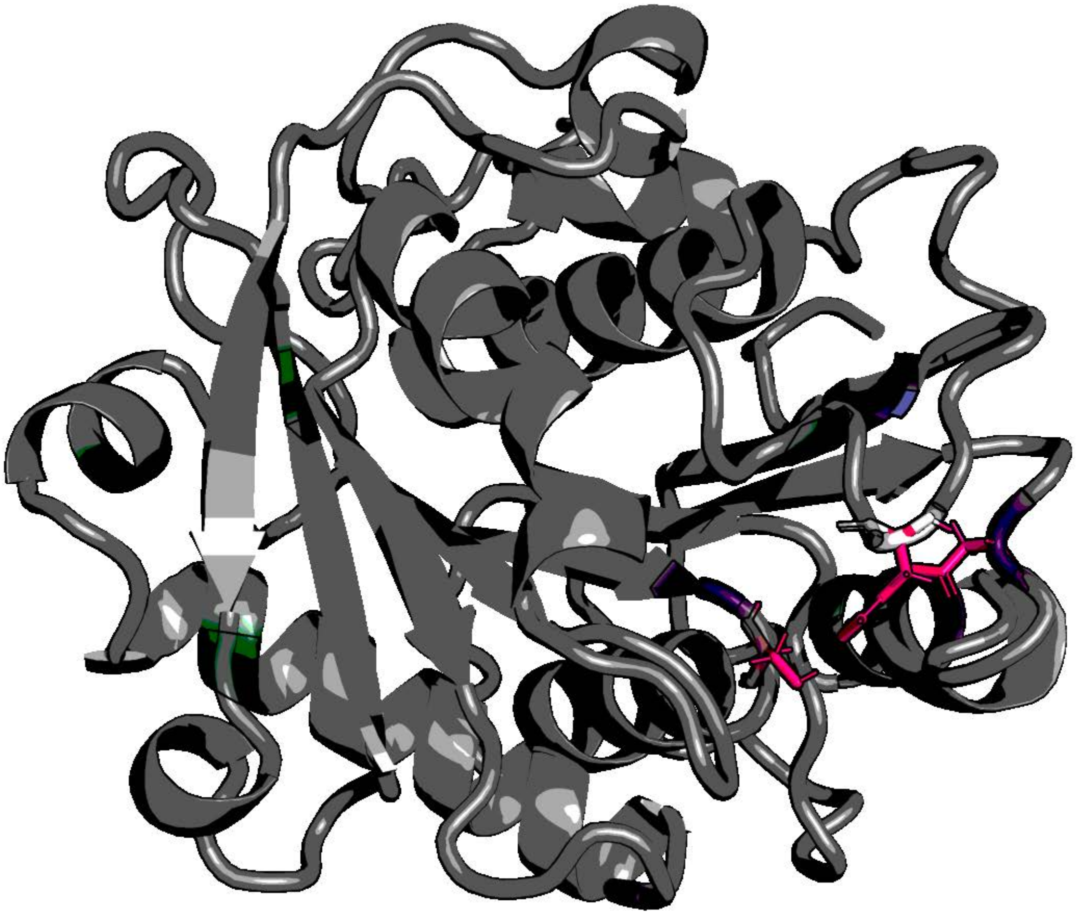

DP009_highlights.png

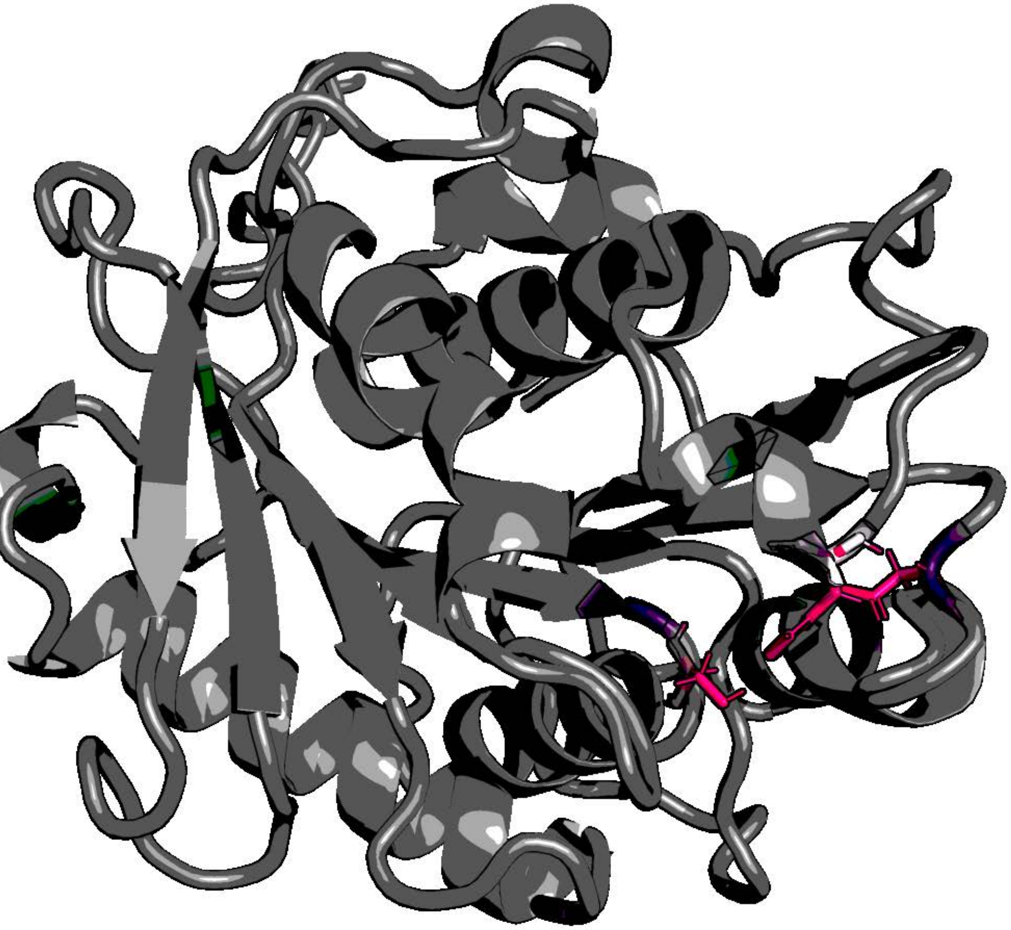

DP016_highlights.png

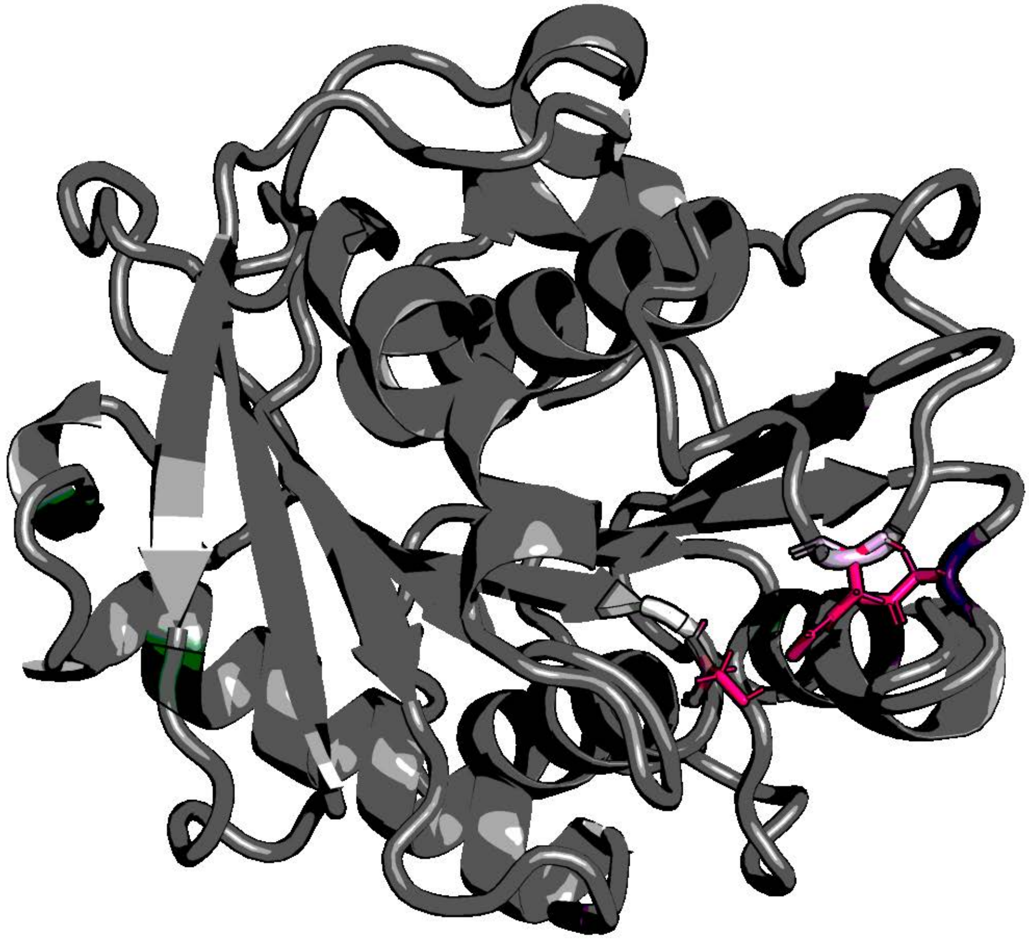

DP017_highlights.png

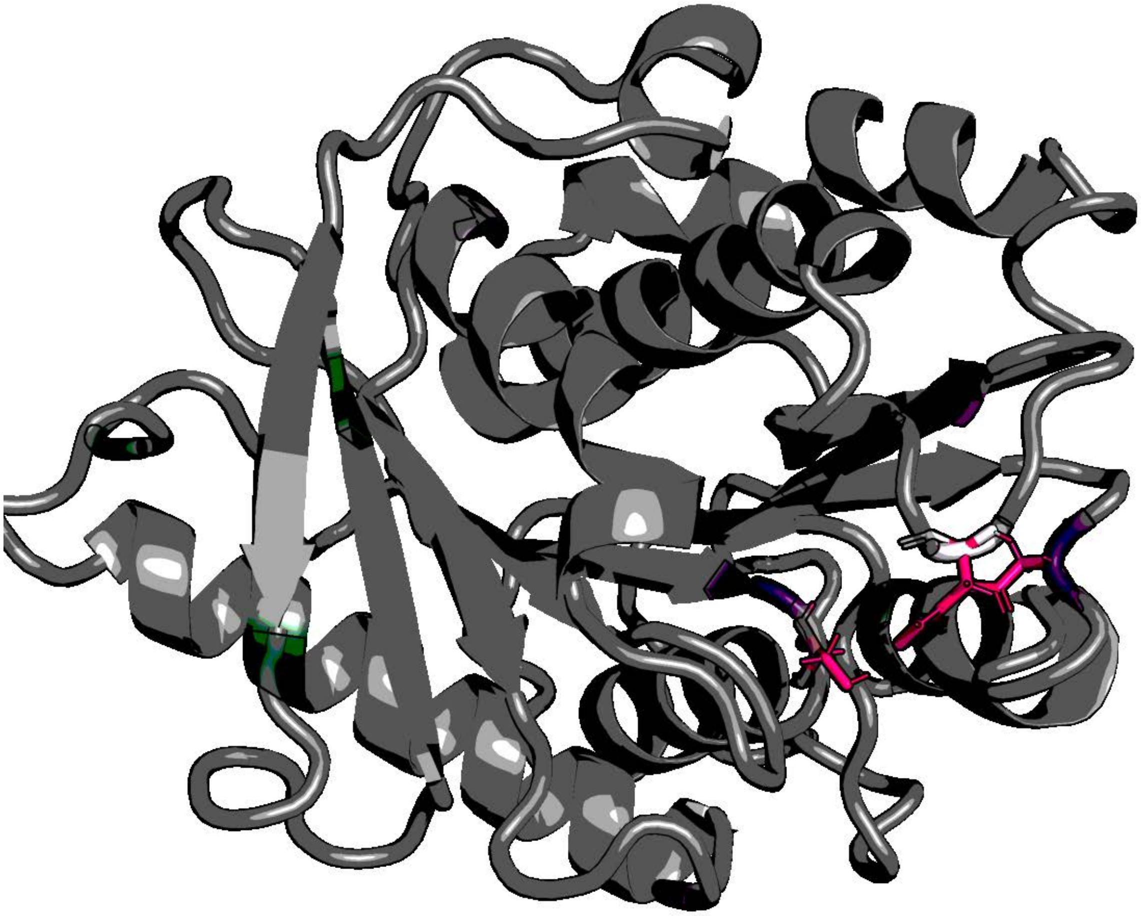

DP038_highlights.png

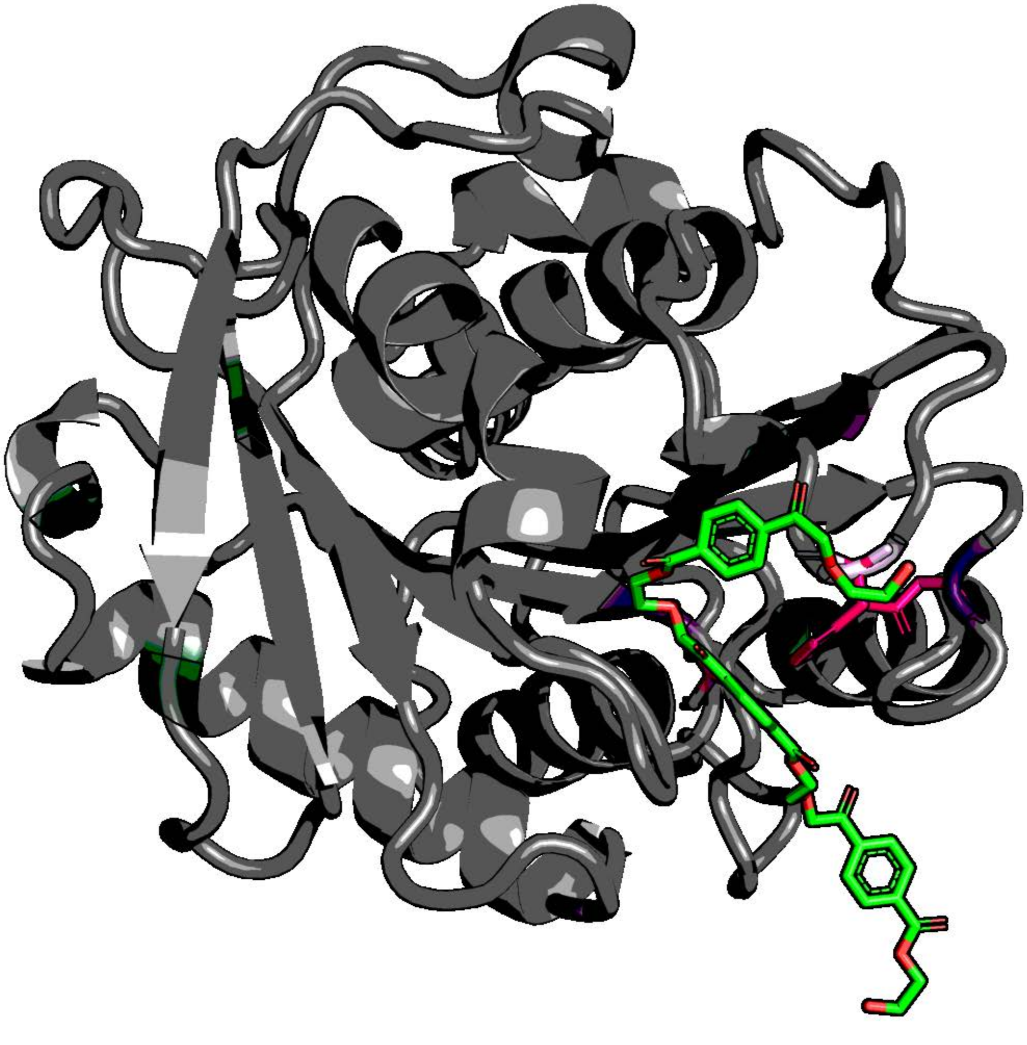

DP043_highlights.png

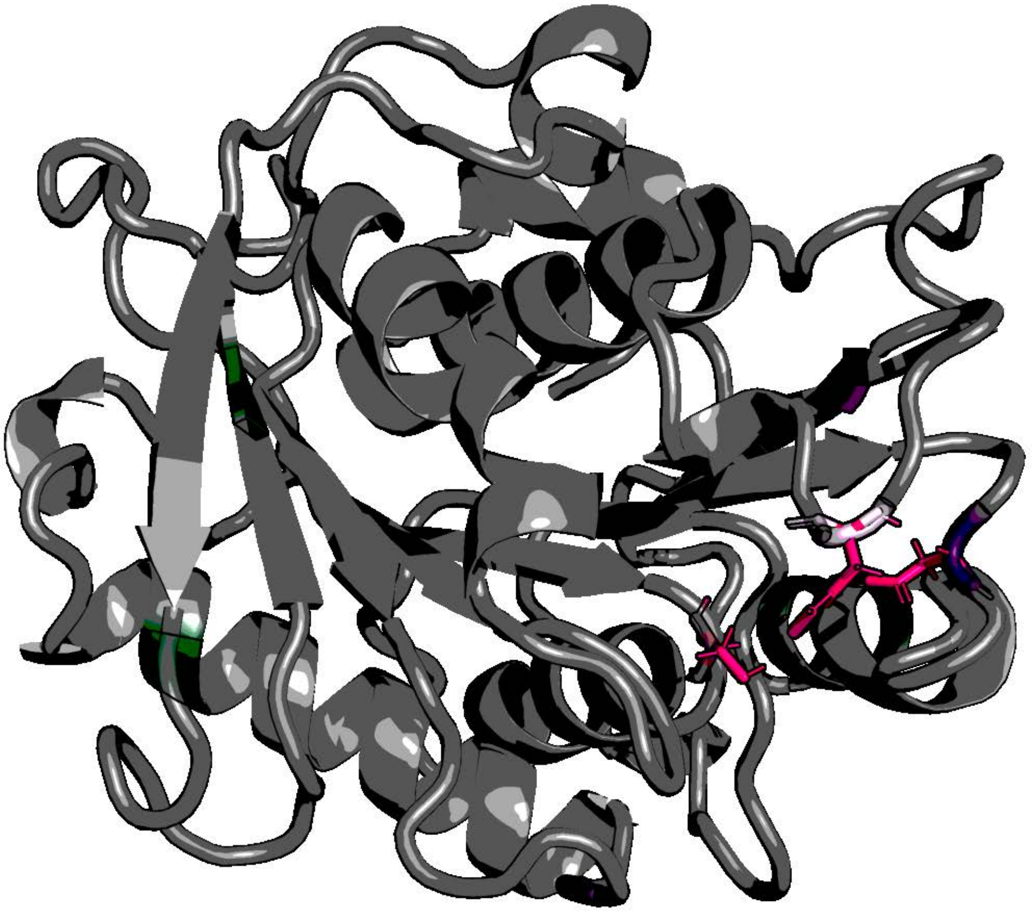

DP054_highlights.png

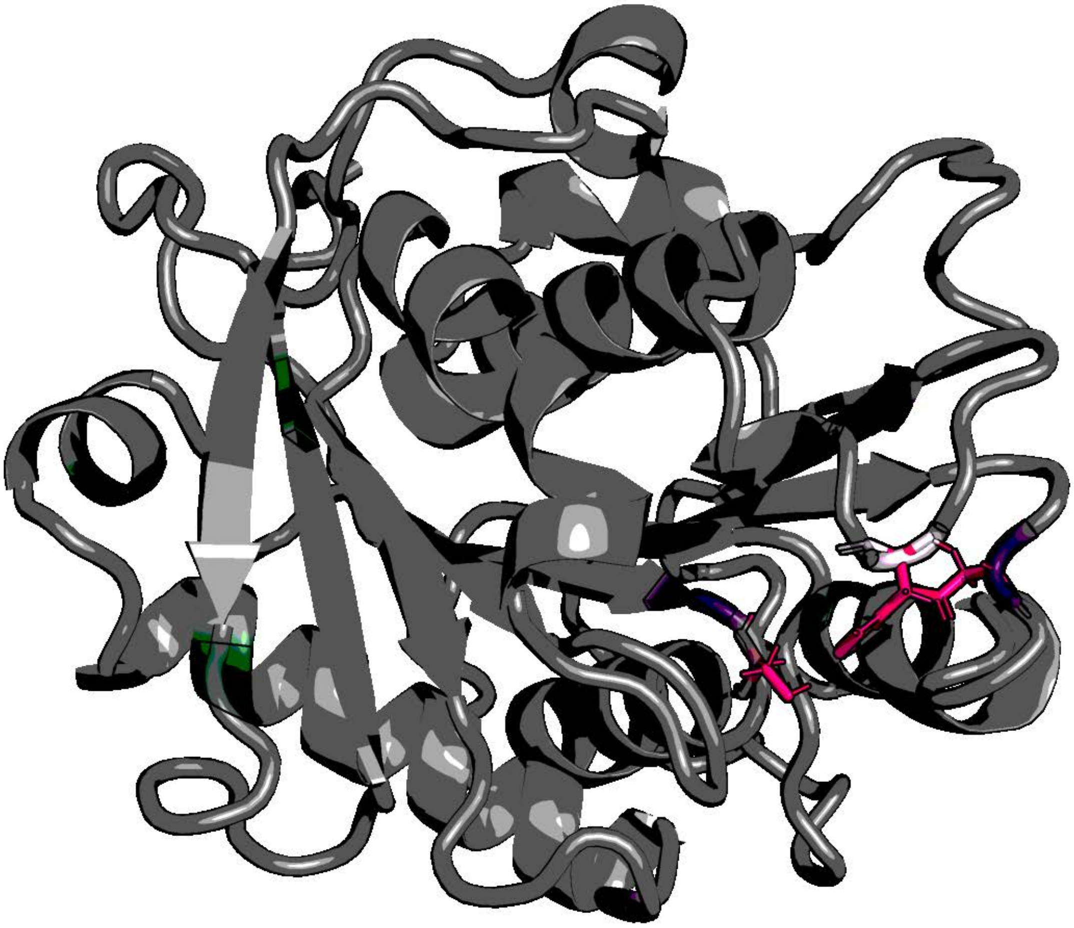

DP056_highlights.png

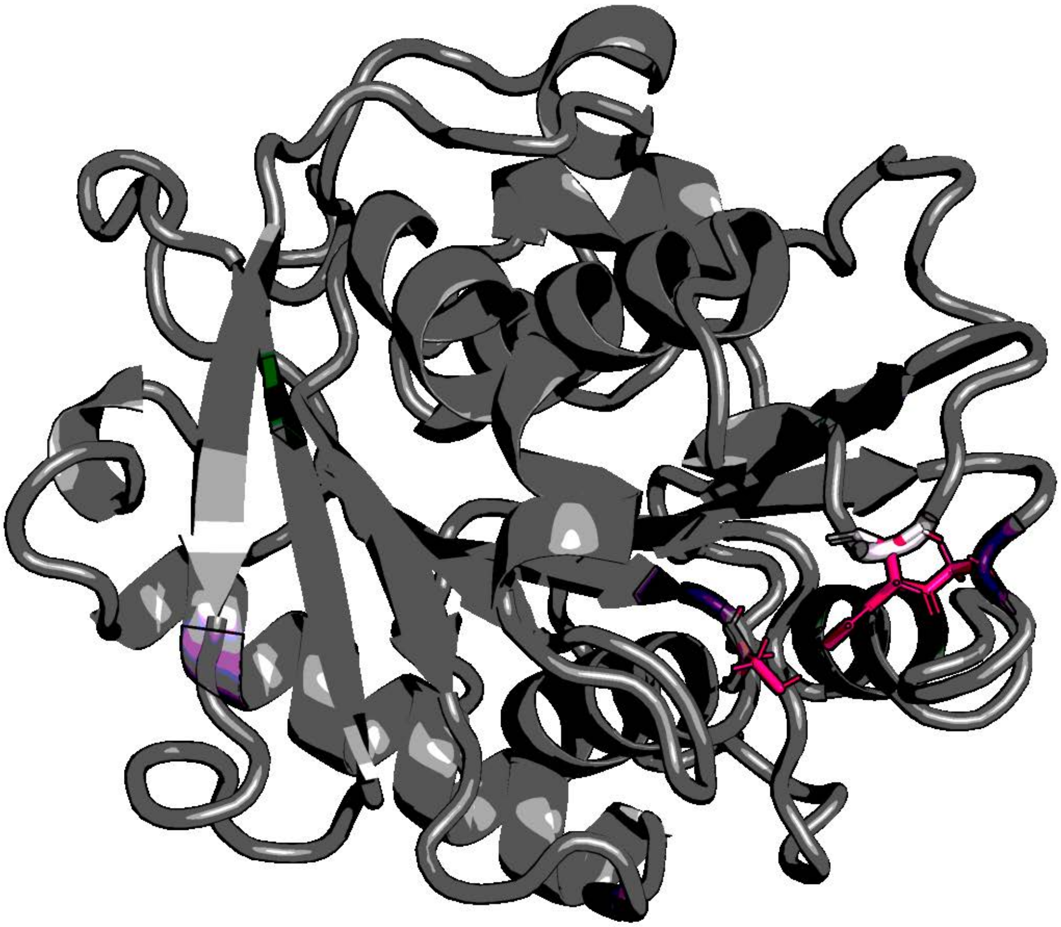

DP057_highlights.png

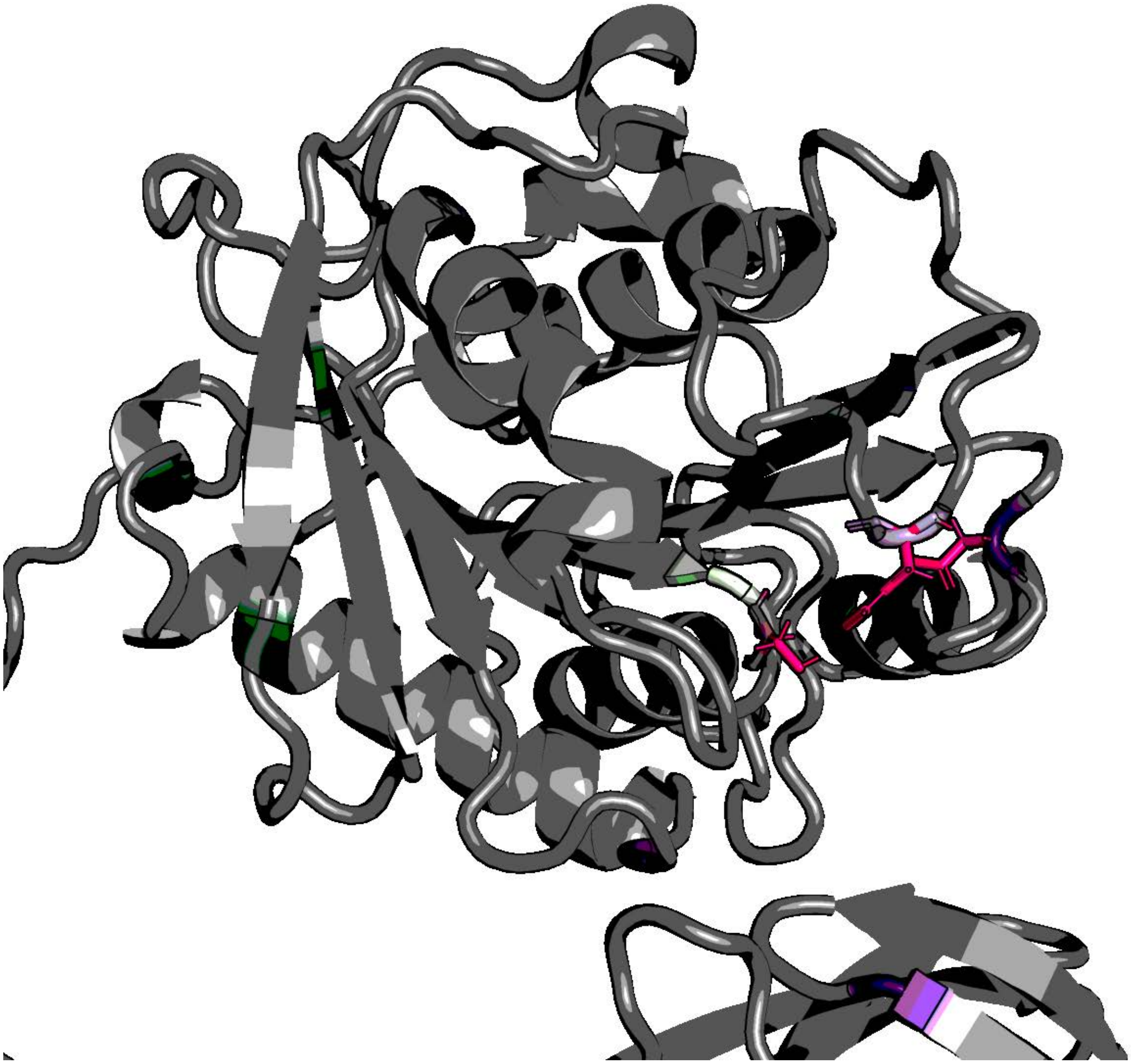

DP059_highlights.png

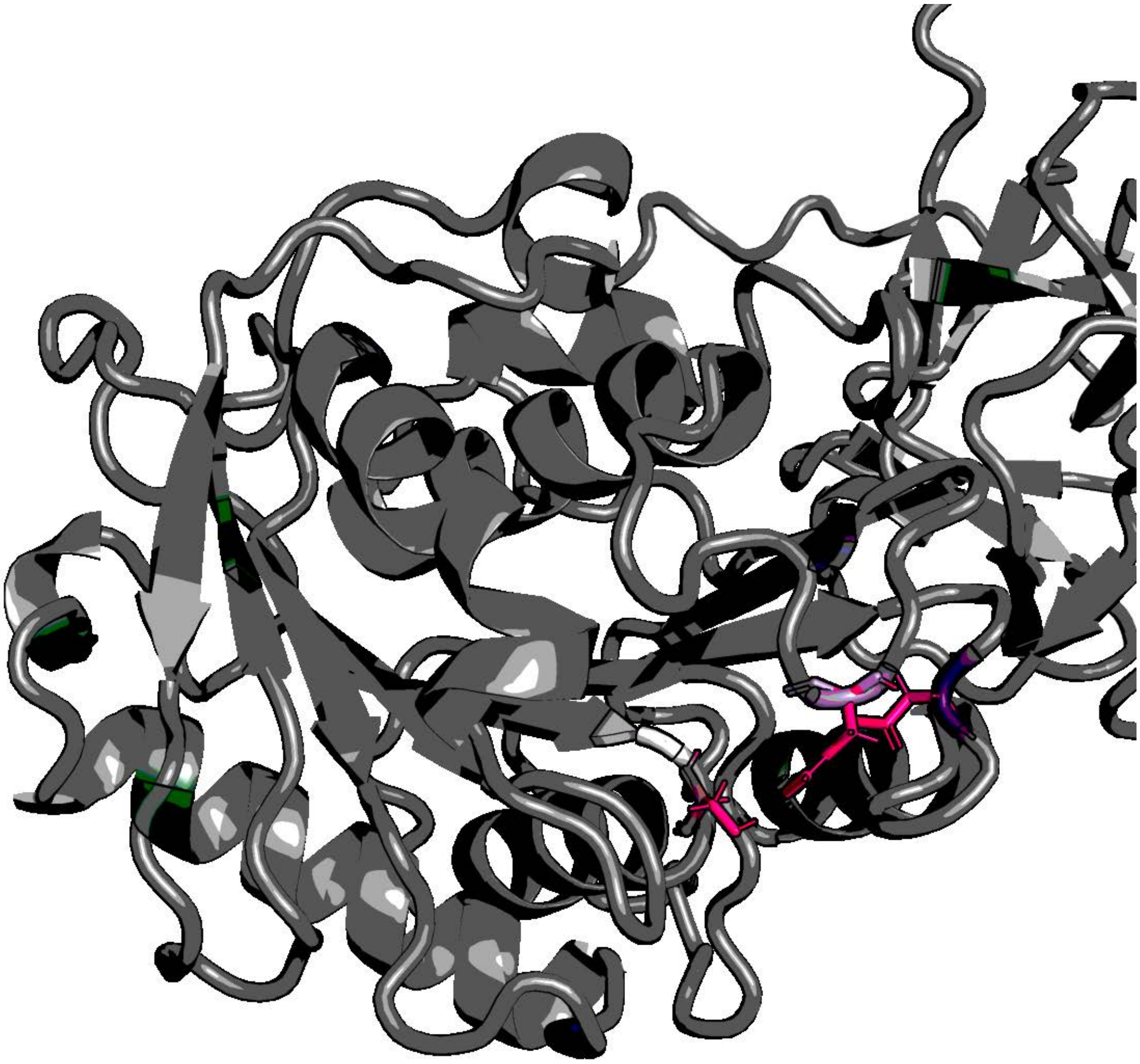

DP061_highlights.png

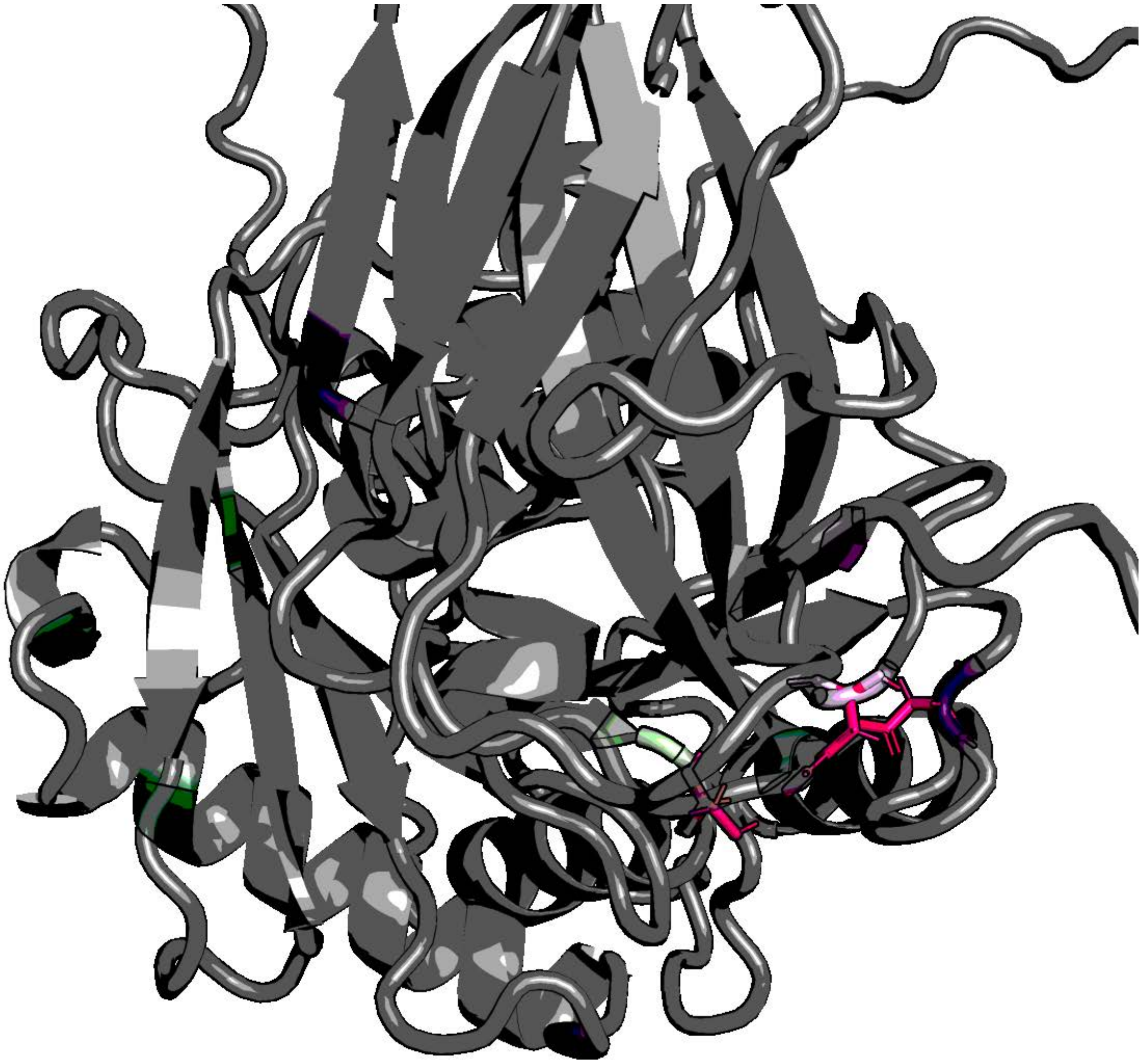

DP068_highlights.png

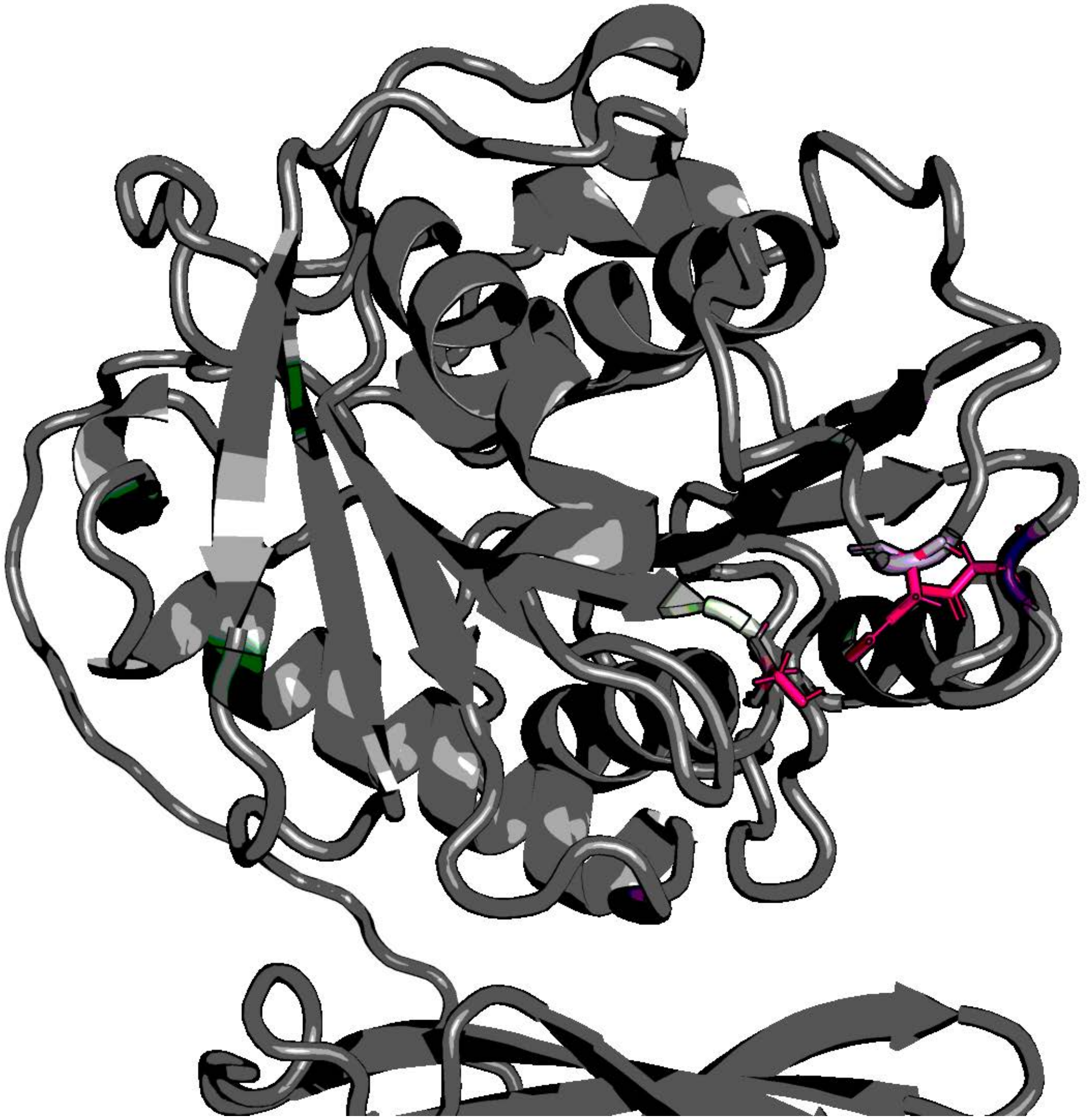

DP072_highlights.png

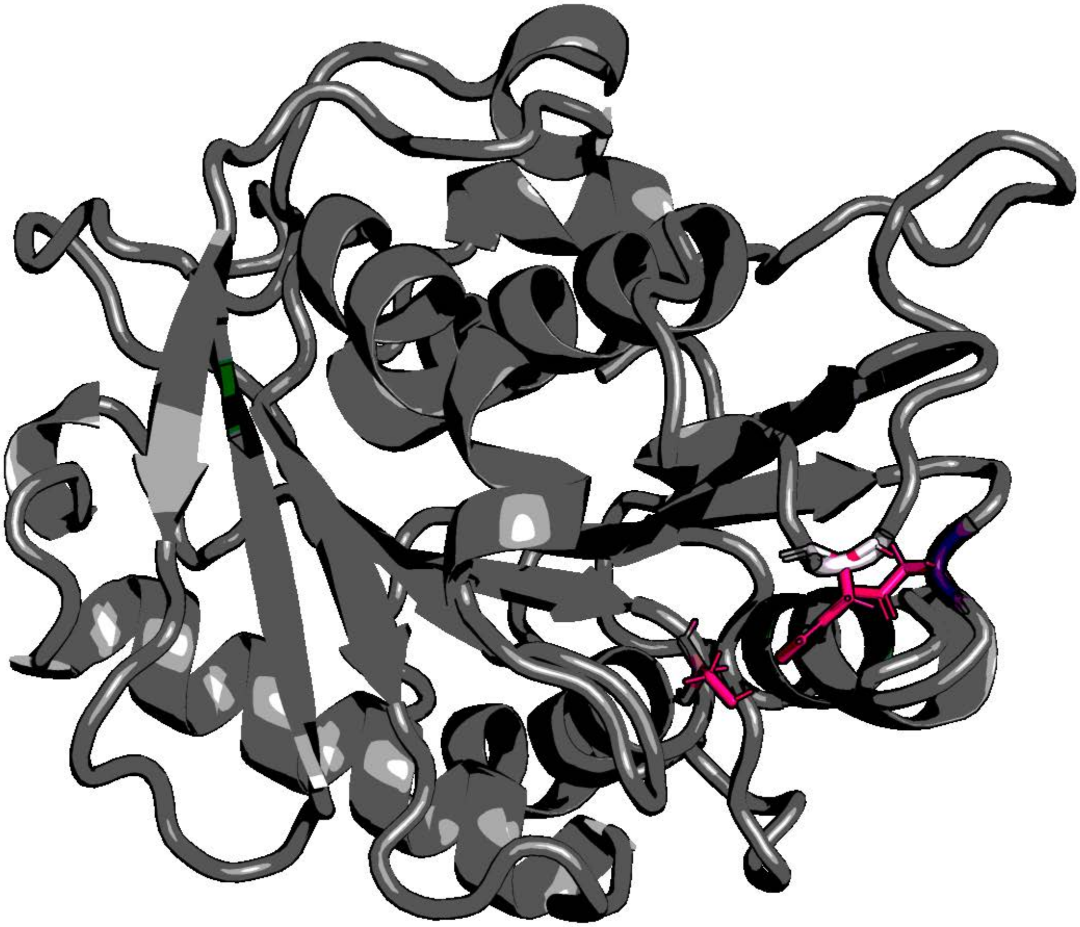

DP077_highlights.png

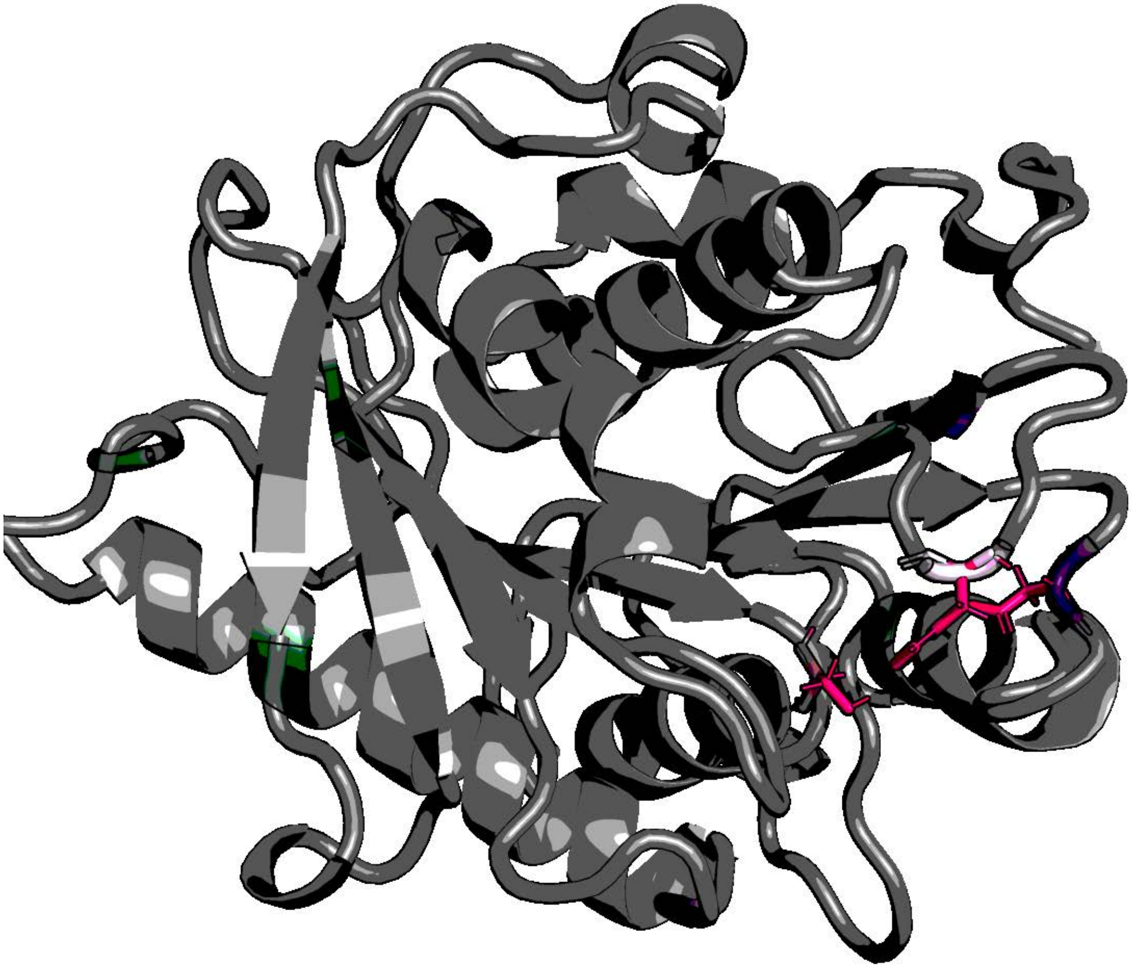

DP078_highlights.png

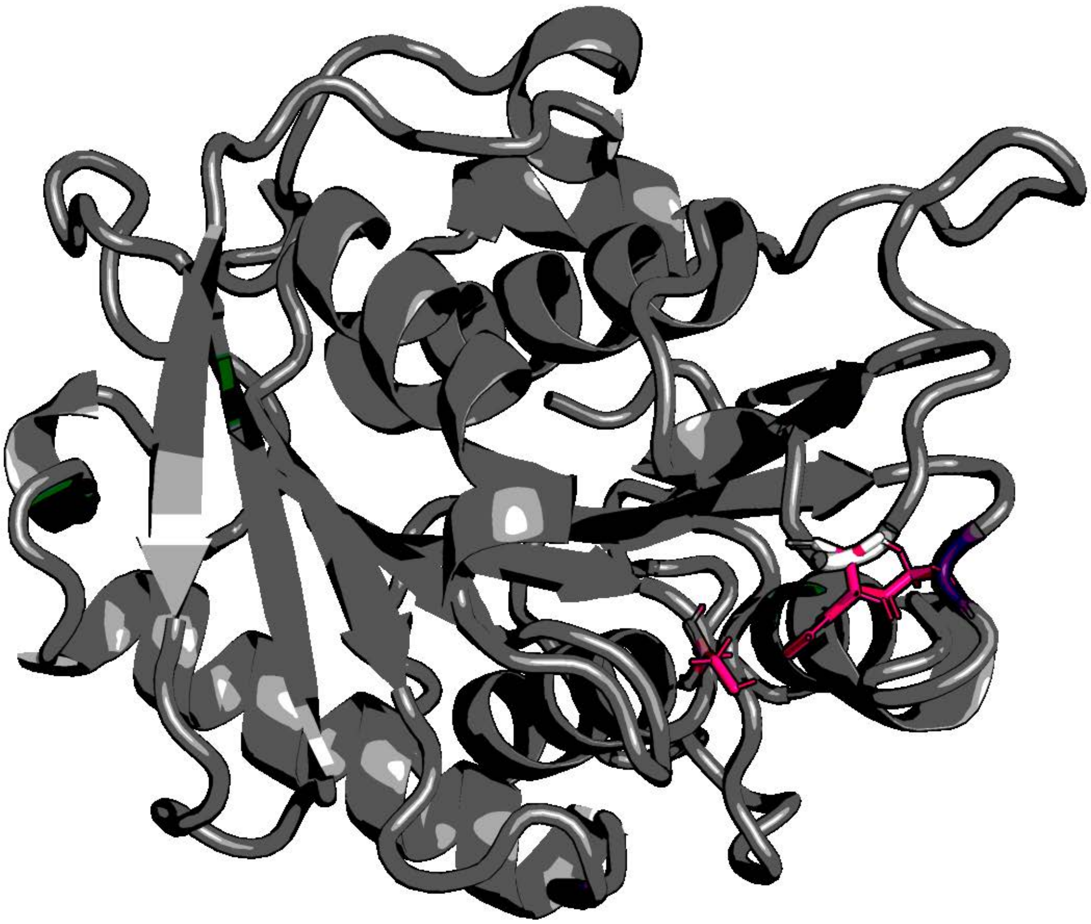

DP080_highlights.png

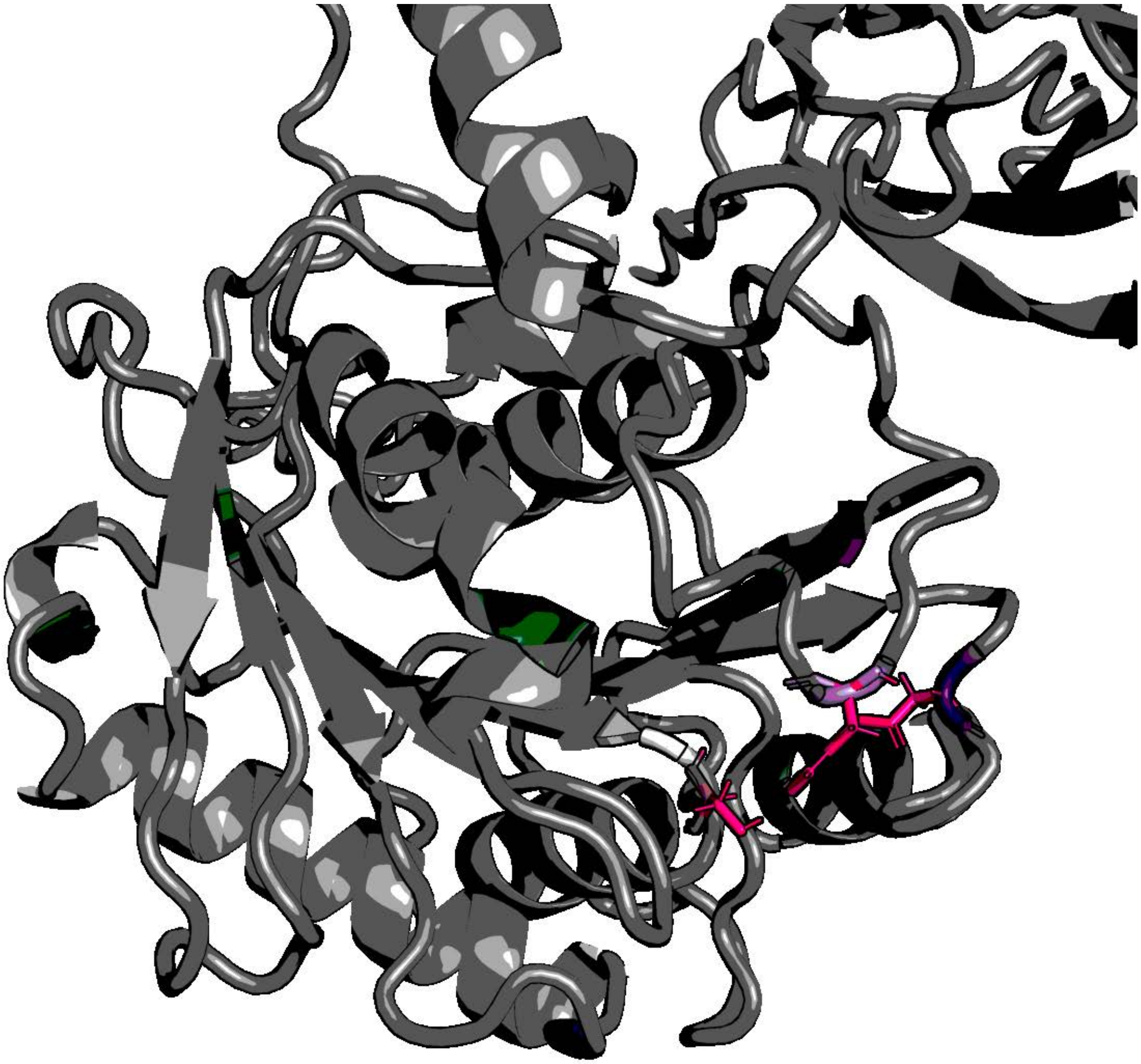

DP081_highlights.png

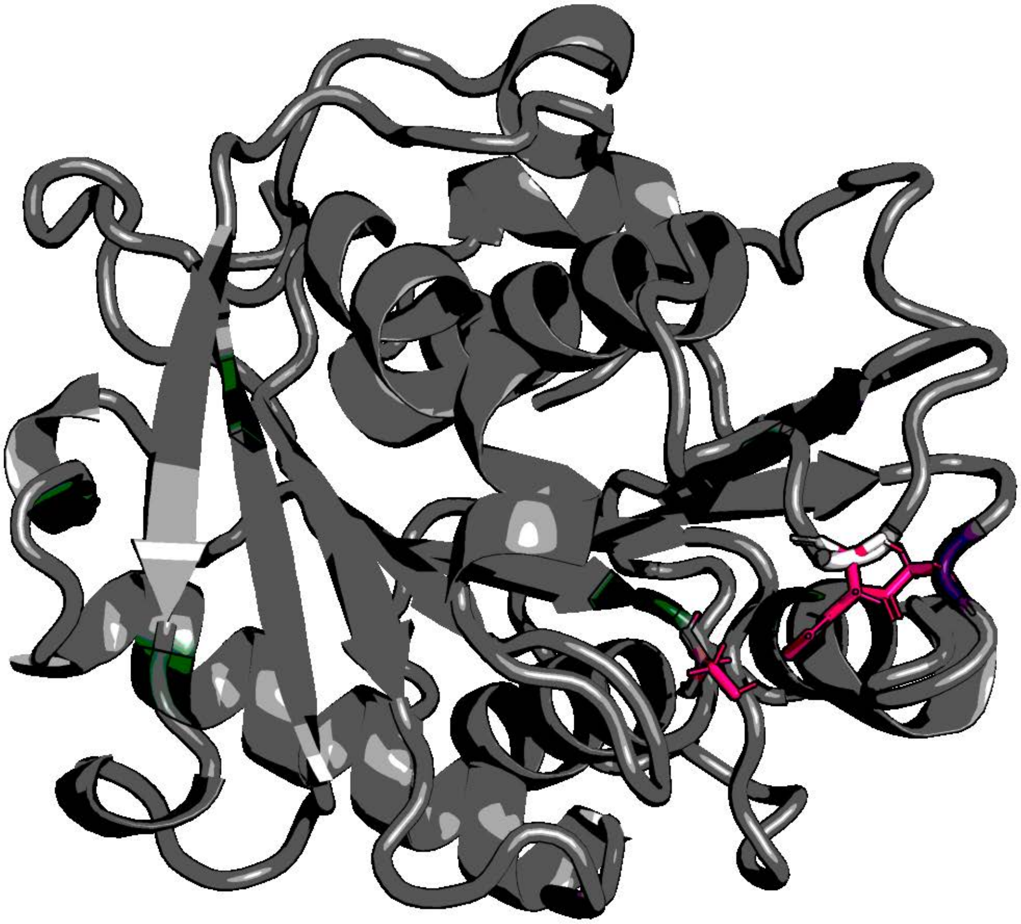

DP082_highlights.png

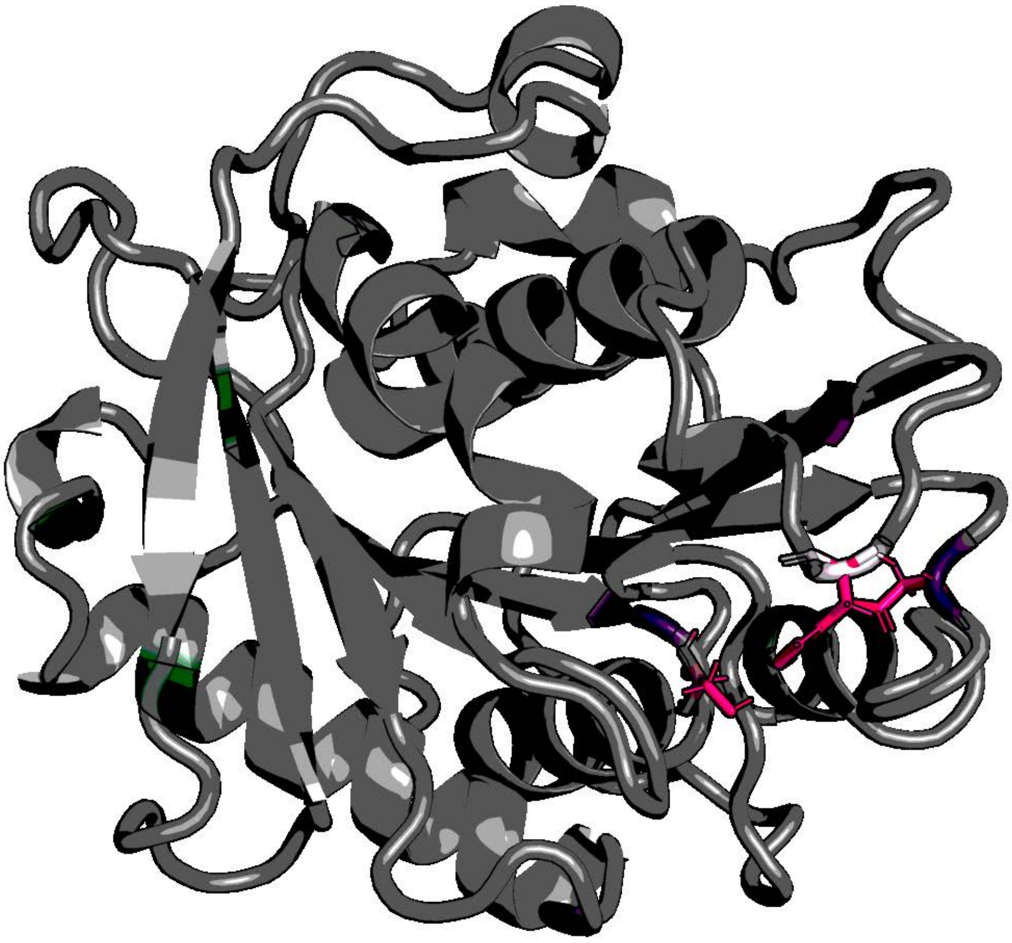

DP084_highlights.png

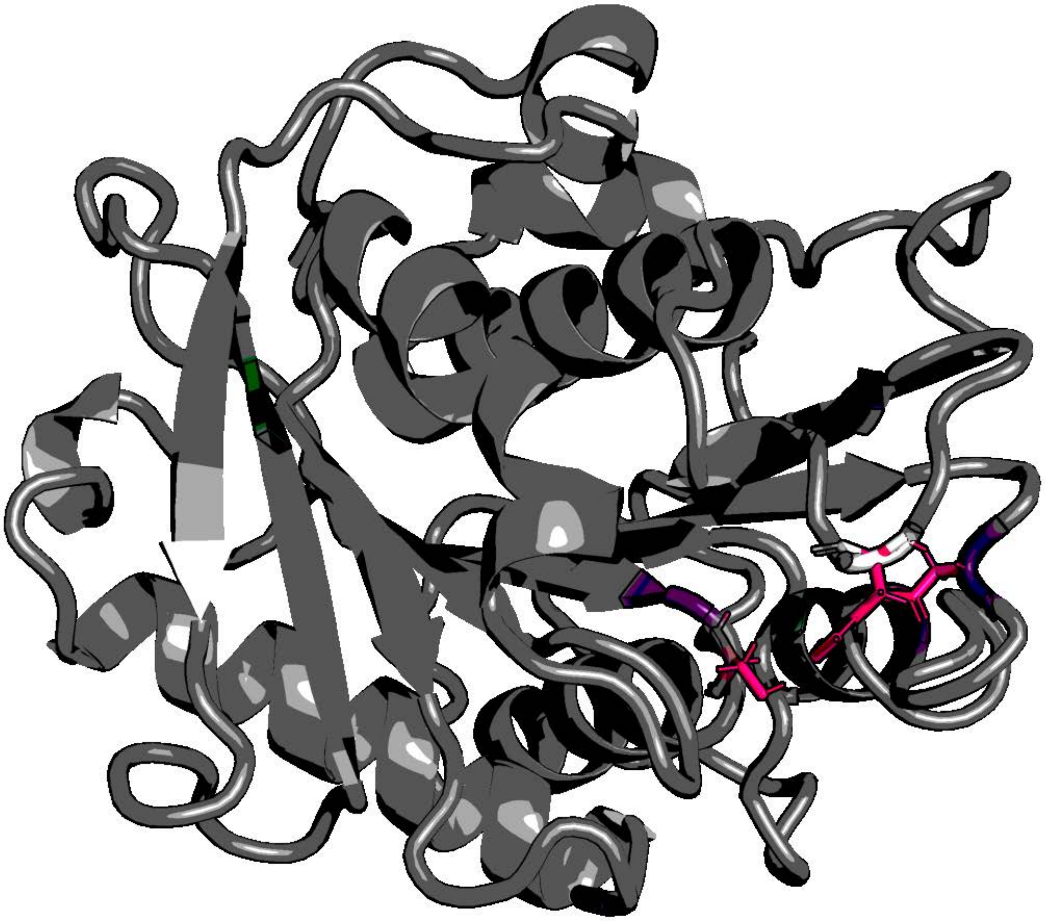

DP089_highlights.png

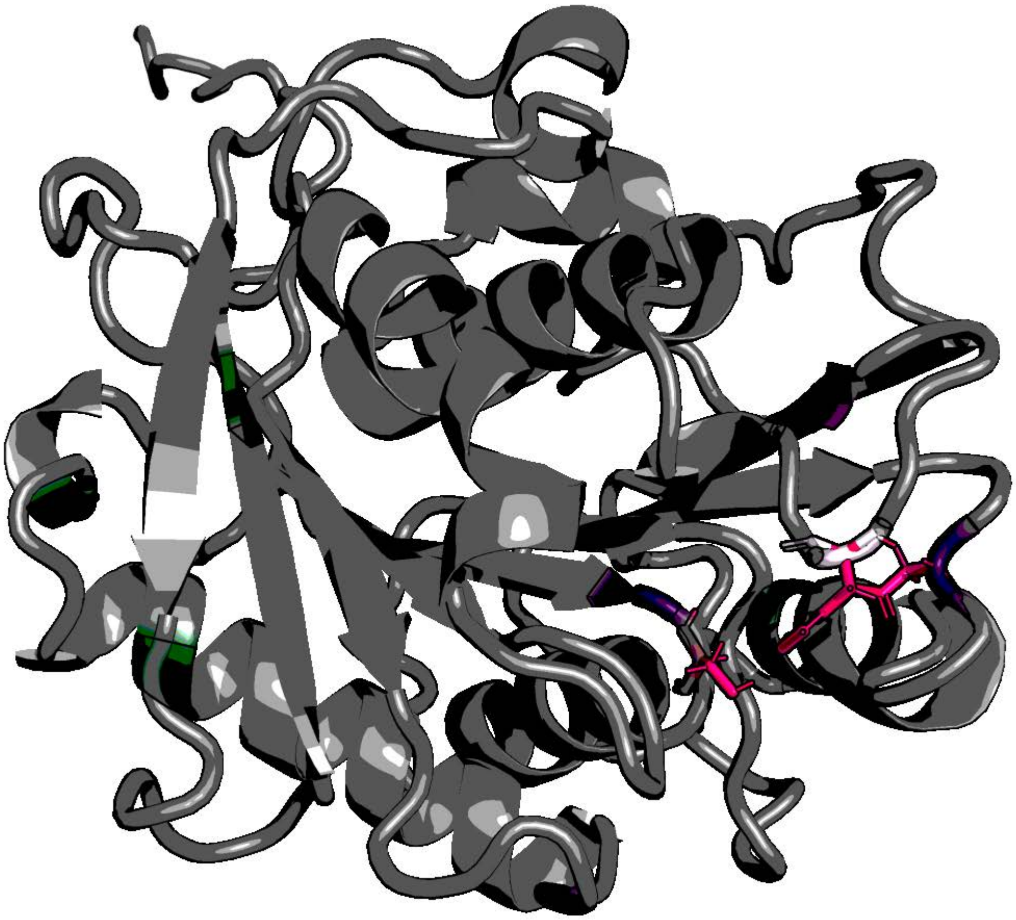

DP090_highlights.png

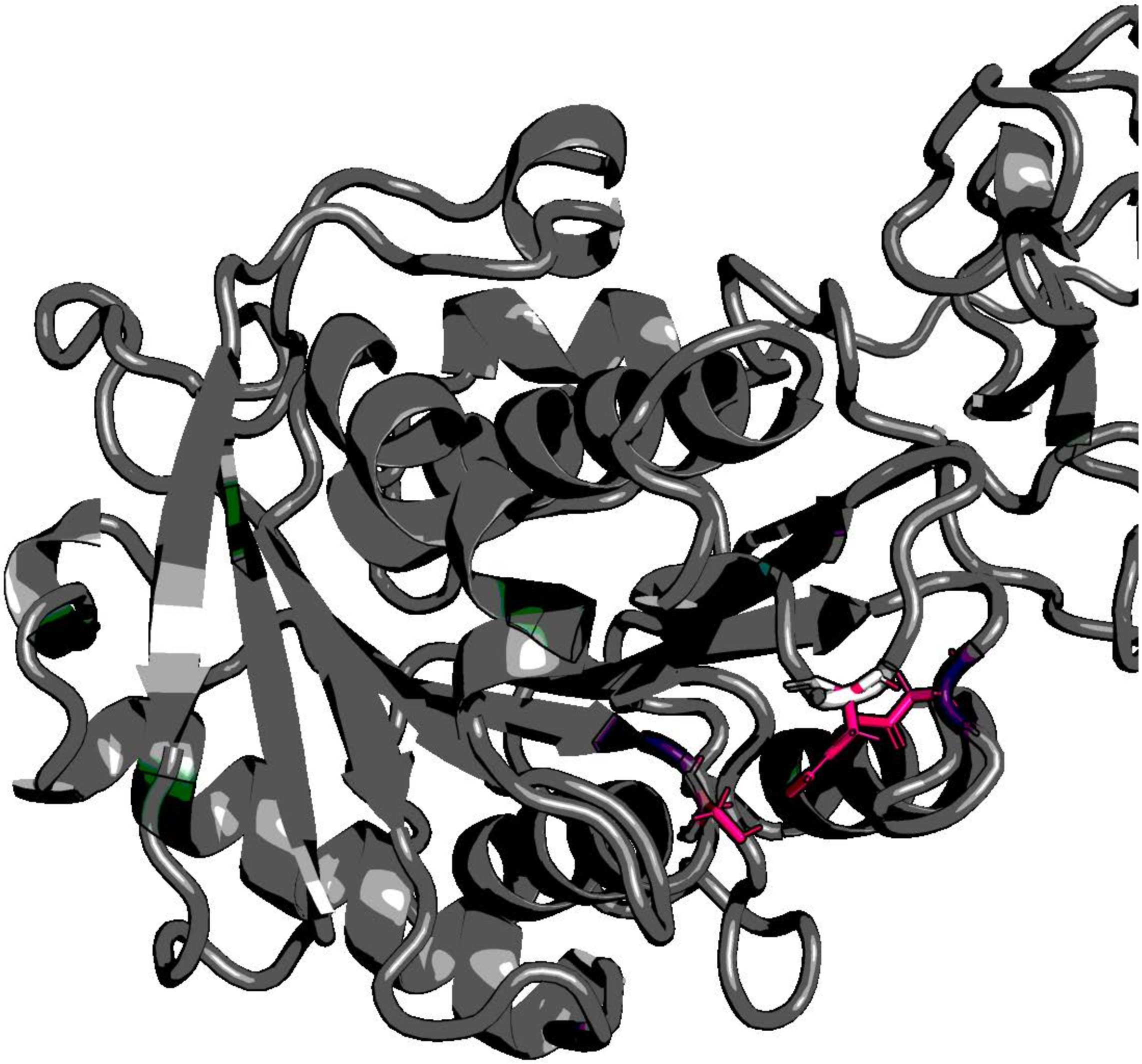

DP092_highlights.png

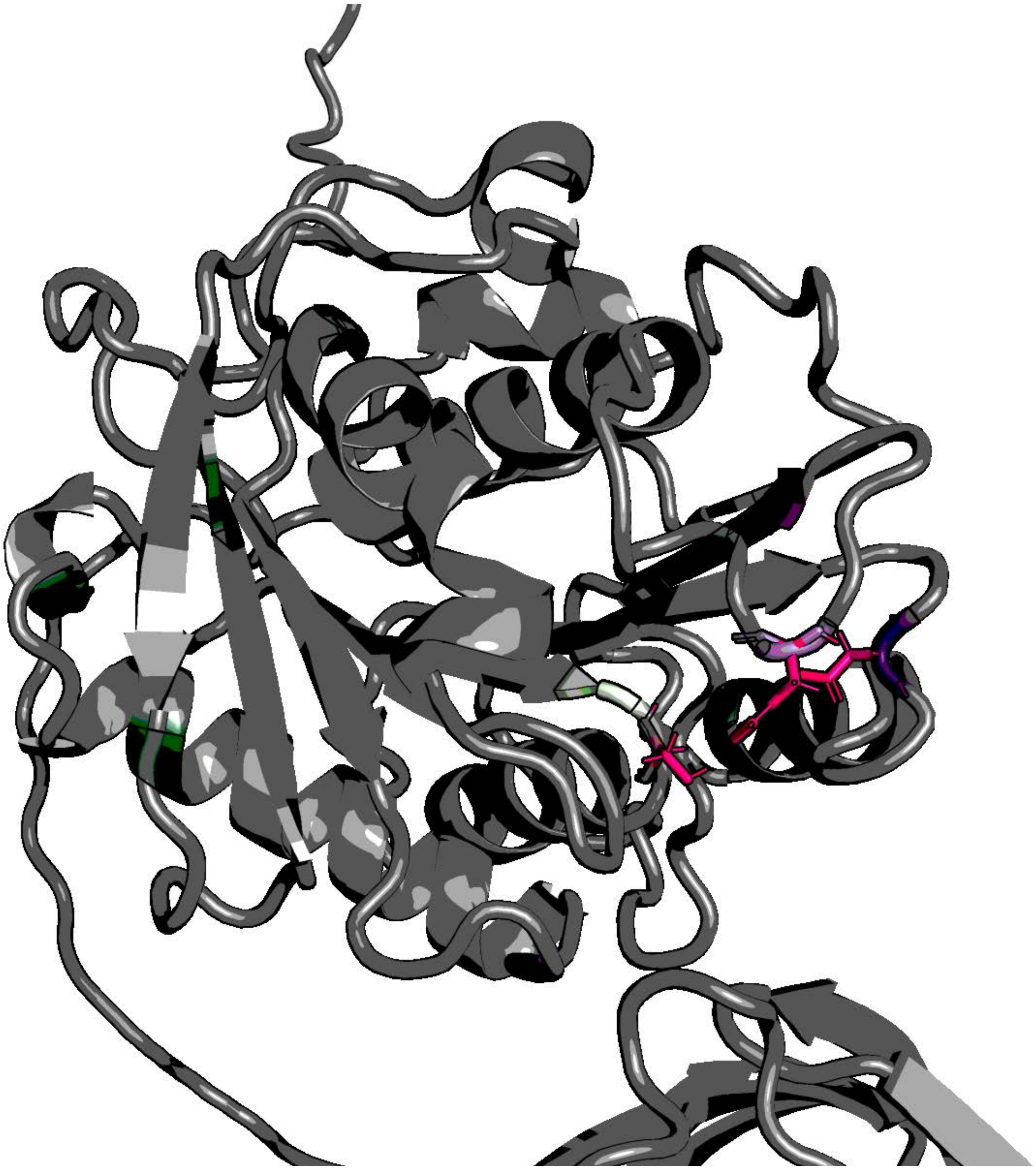

ESM006_highlights.png

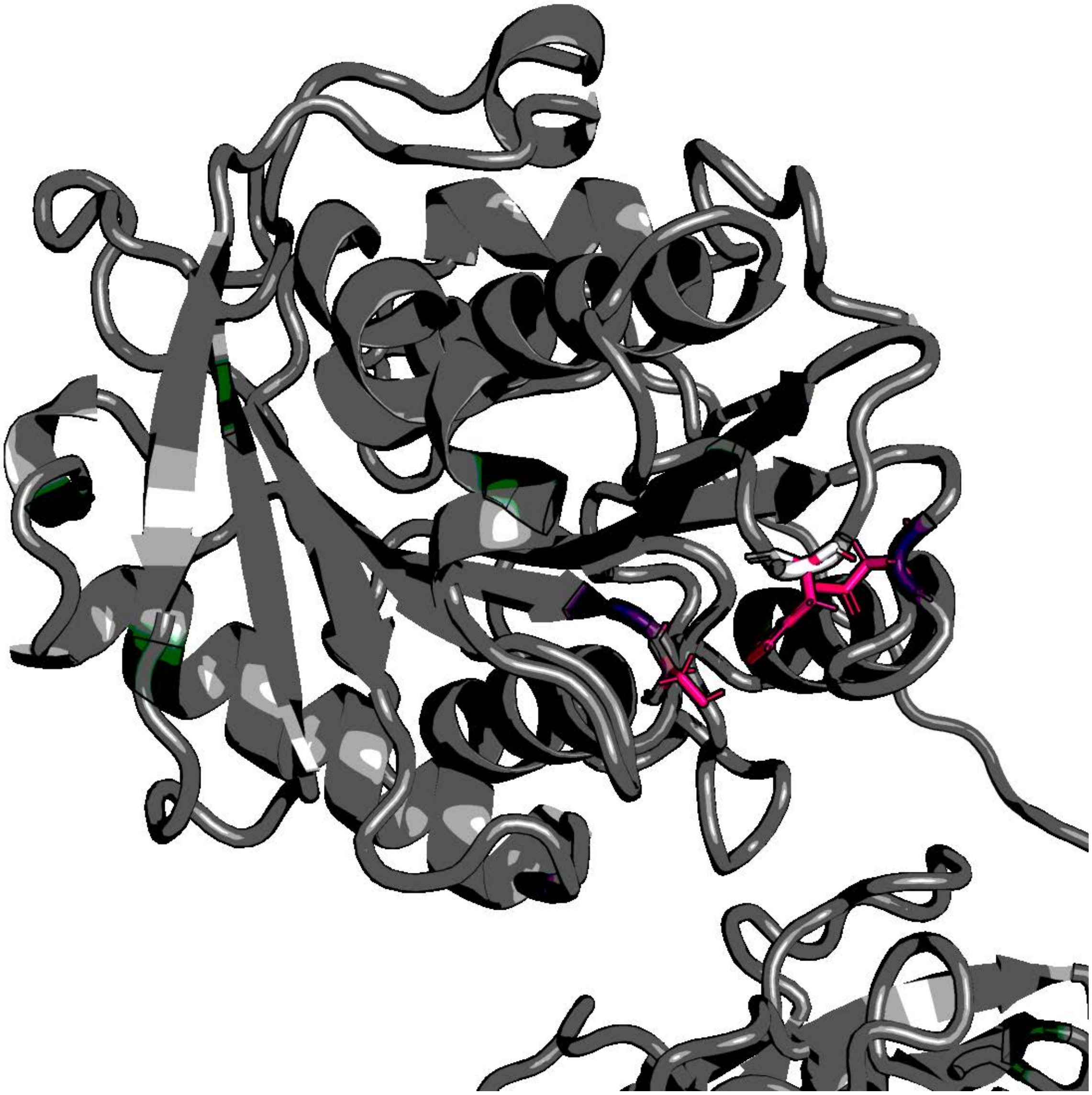

ESM010_highlights.png

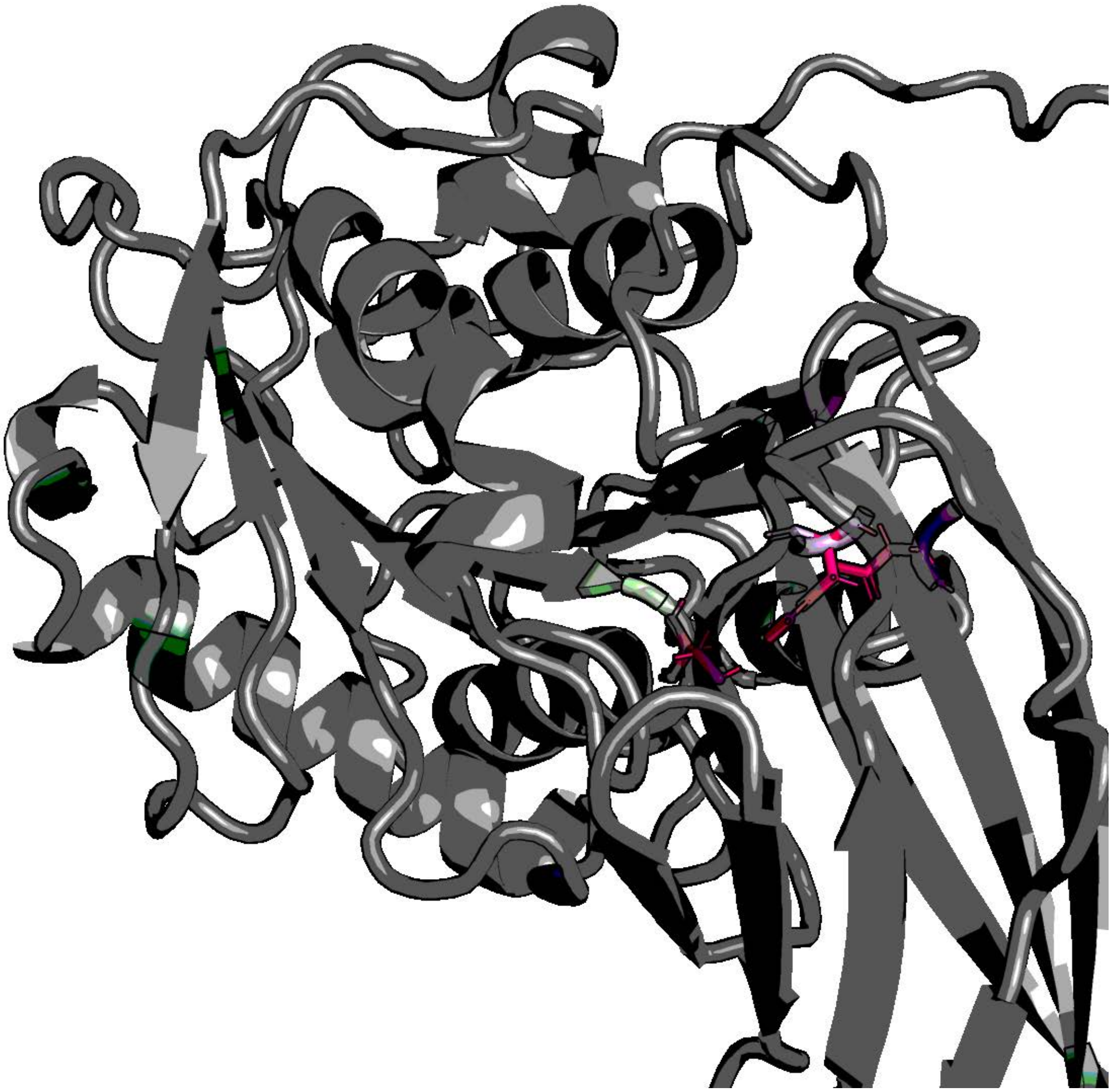

ESM012_highlights.png

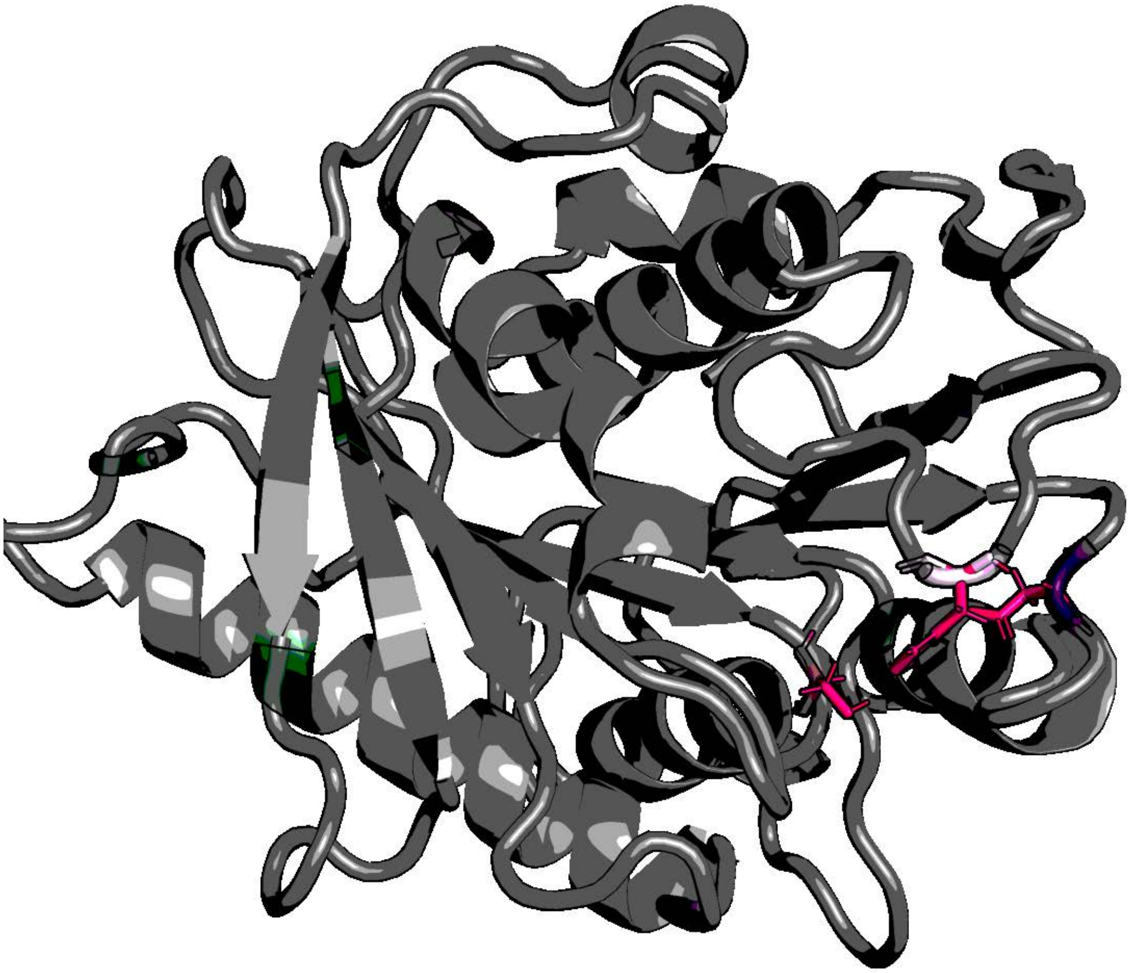

ESM017_highlights.png

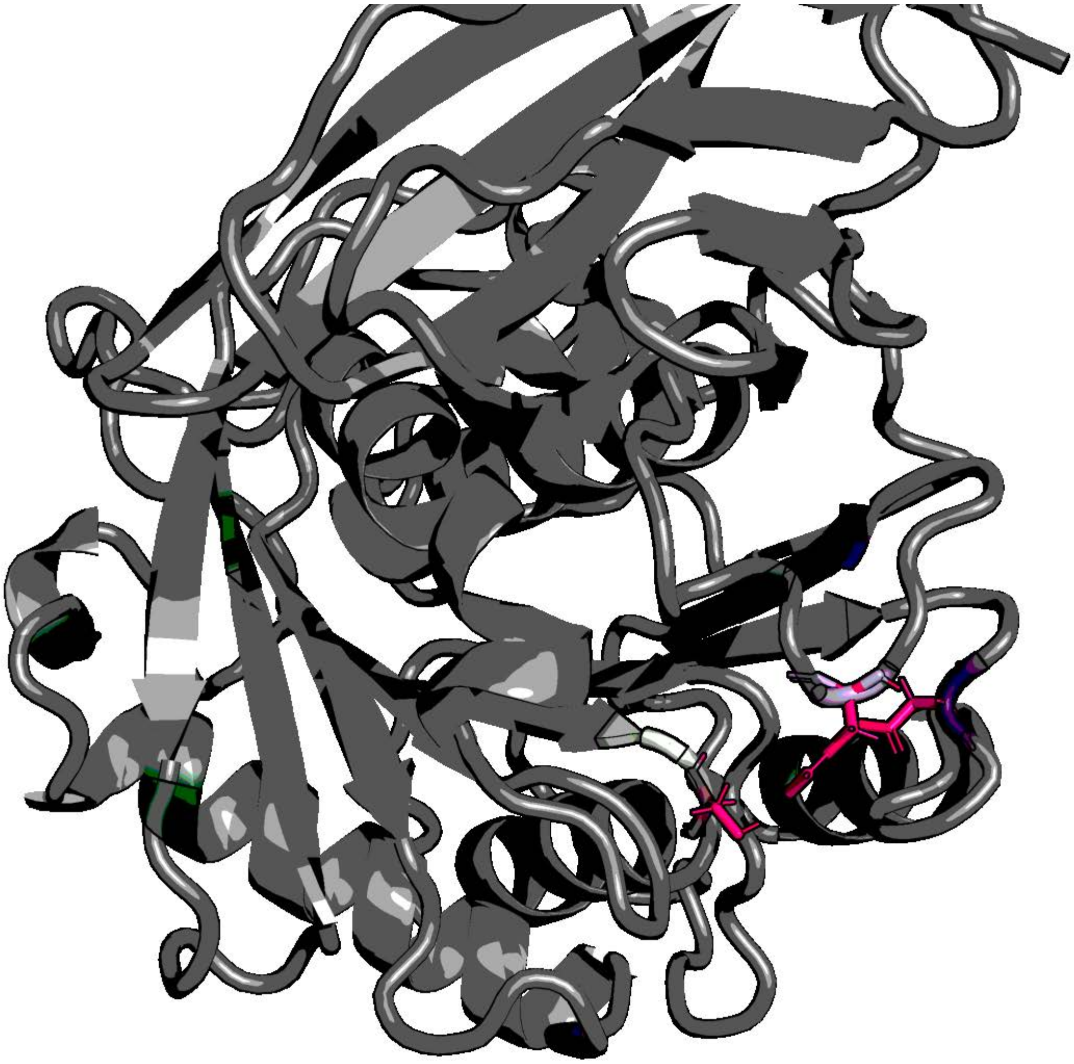

ESM018_highlights.png

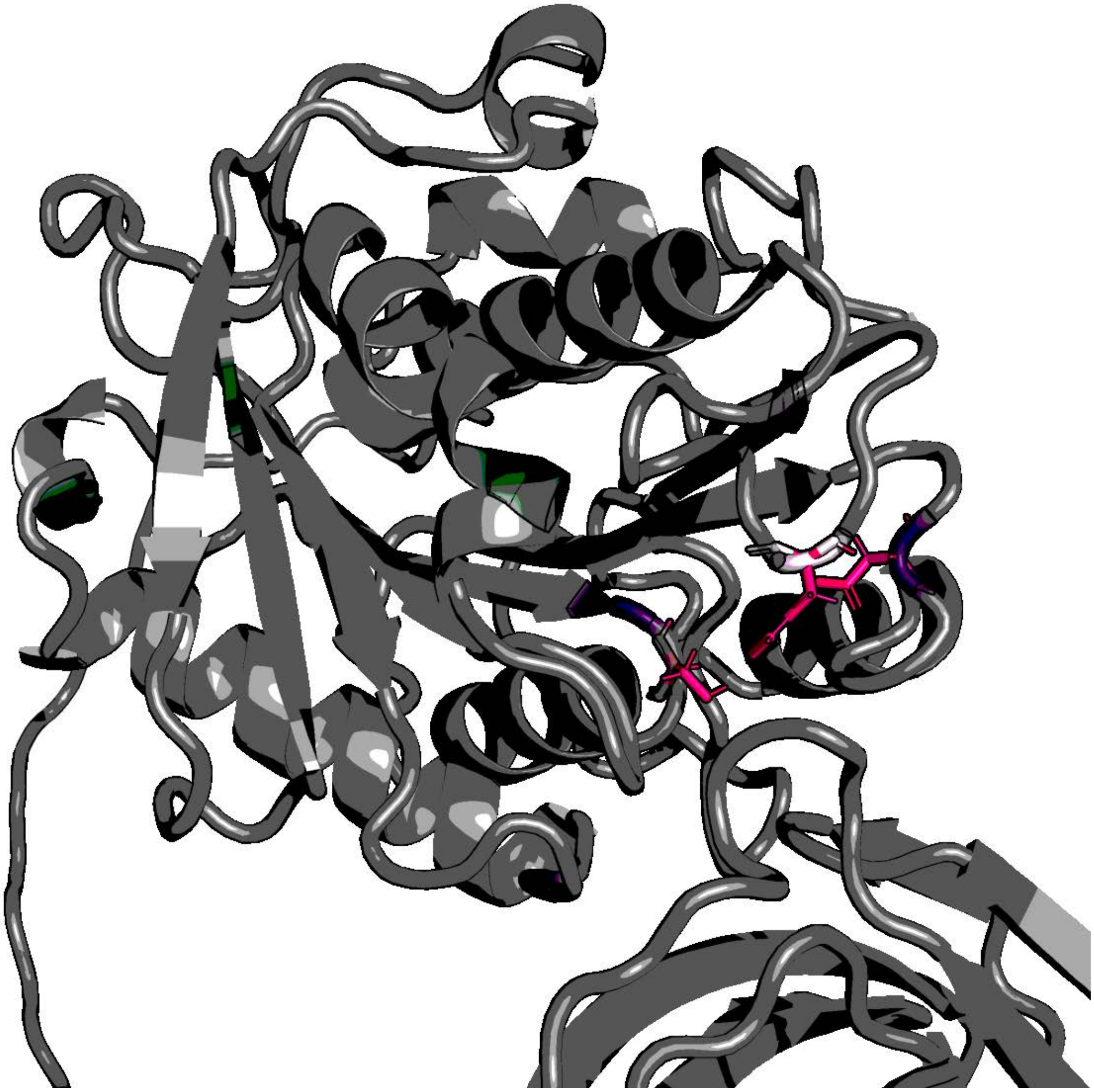

ESM021_highlights.png

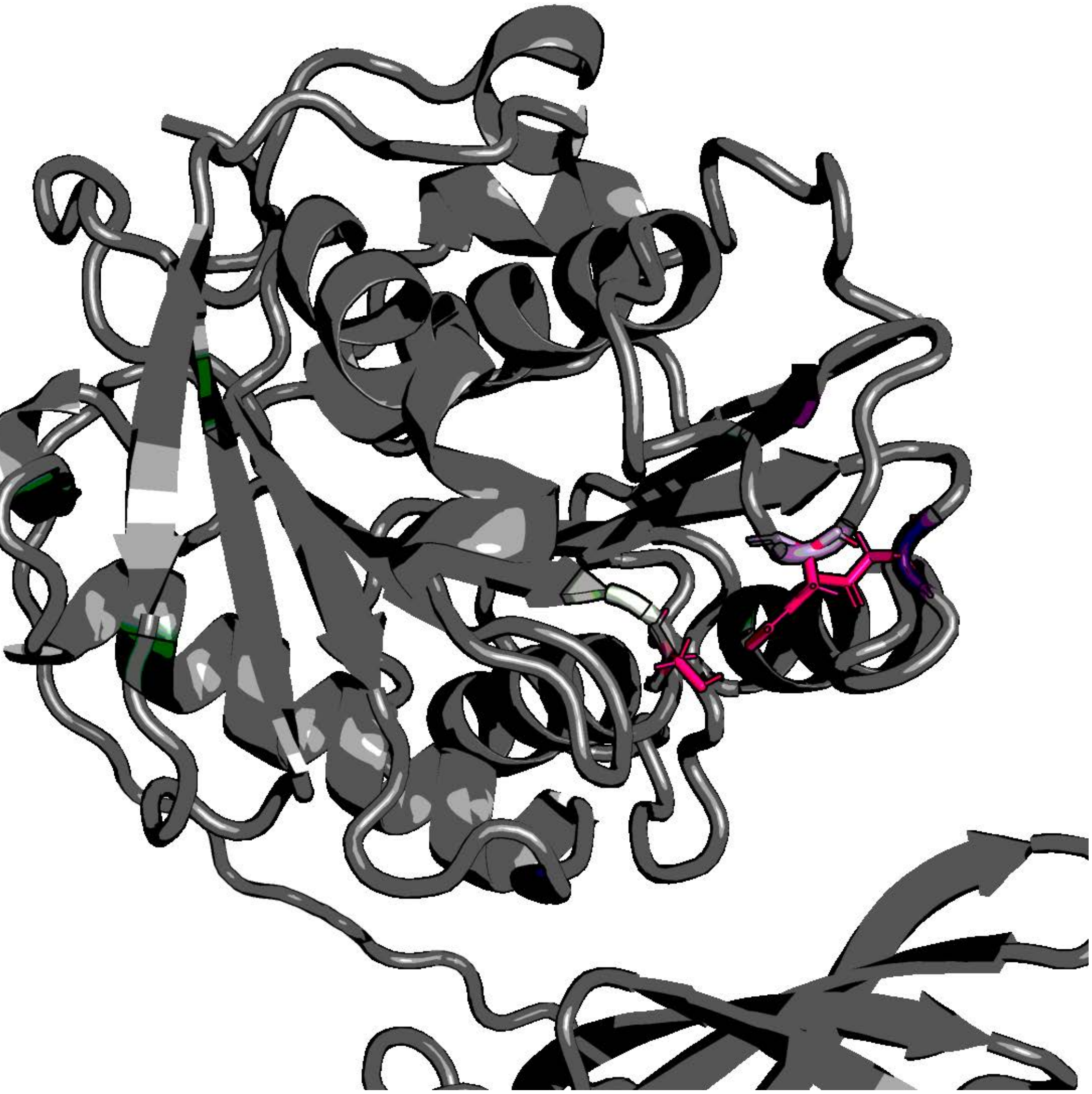

ESM023_highlights.png

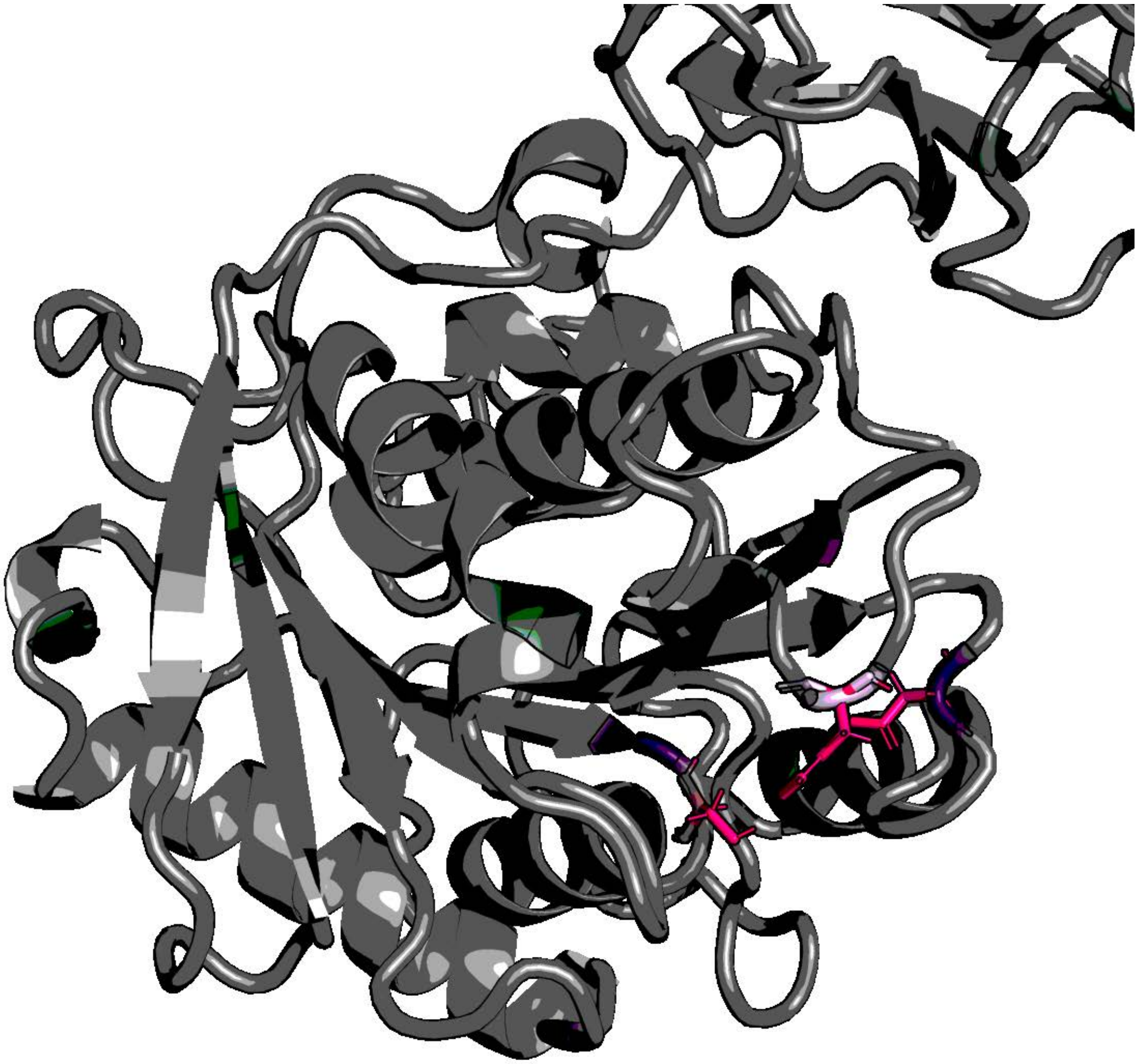

ESM026_highlights.png

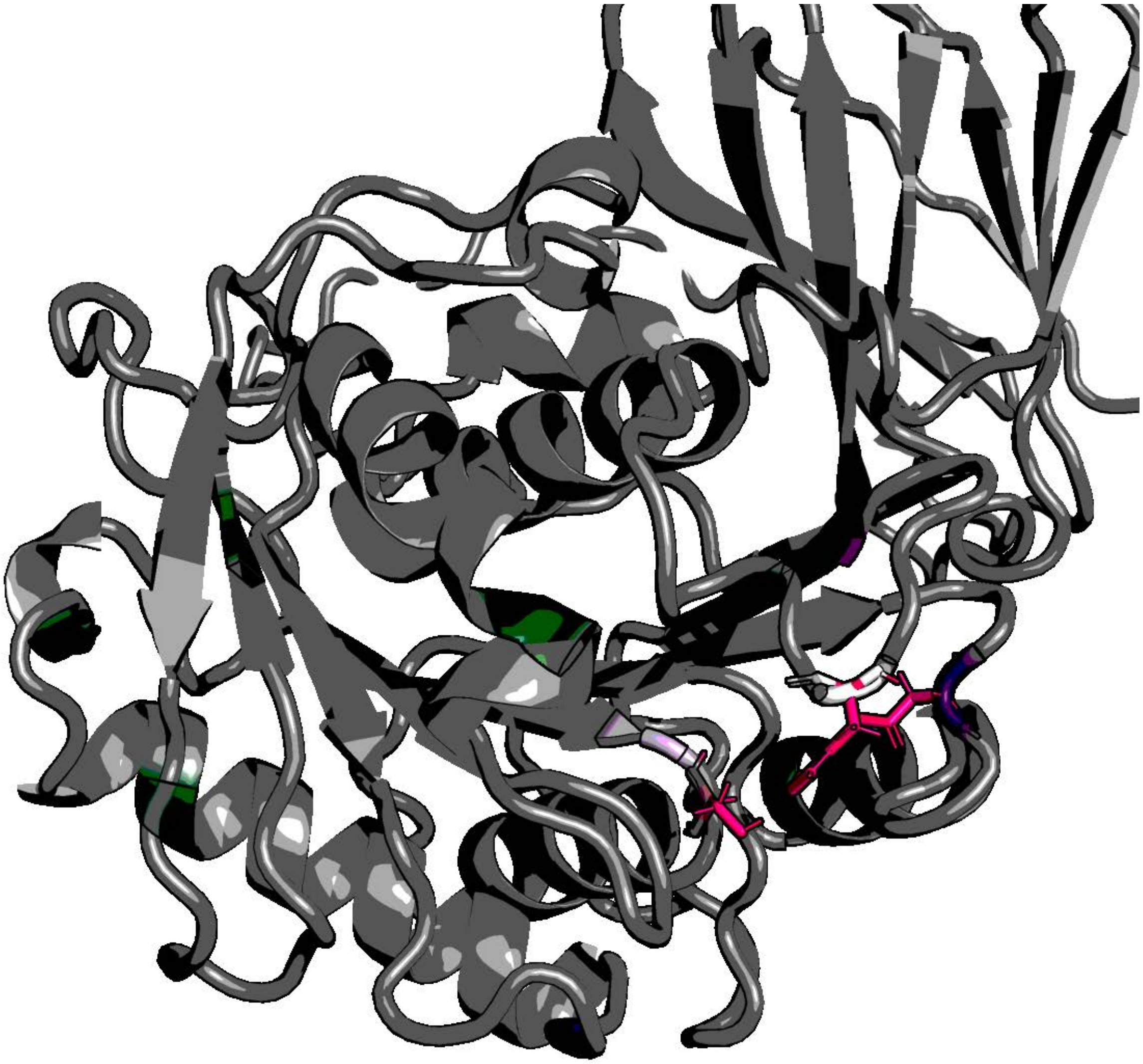

ESM033_highlights.png

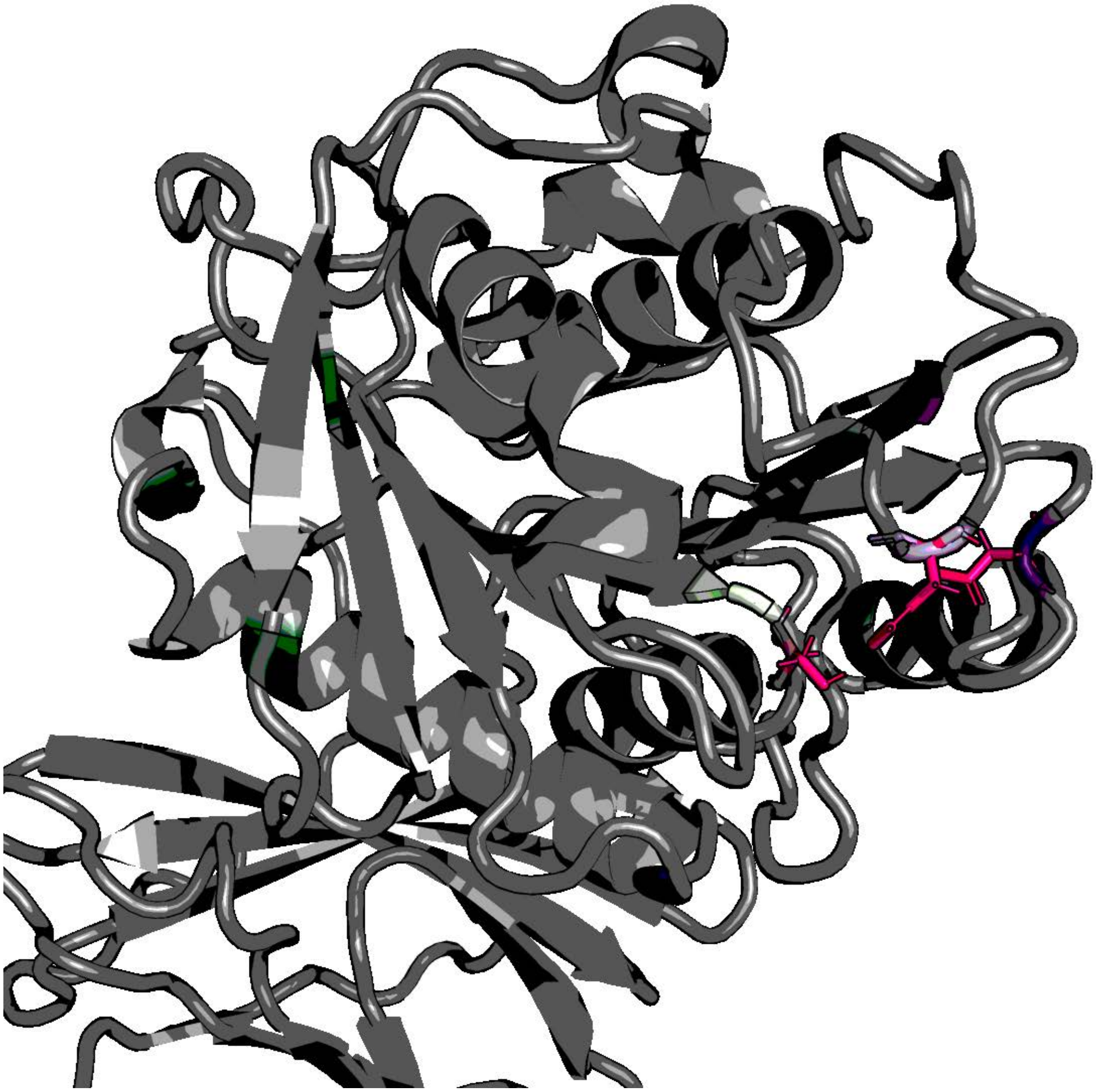

ESM035_highlights.png

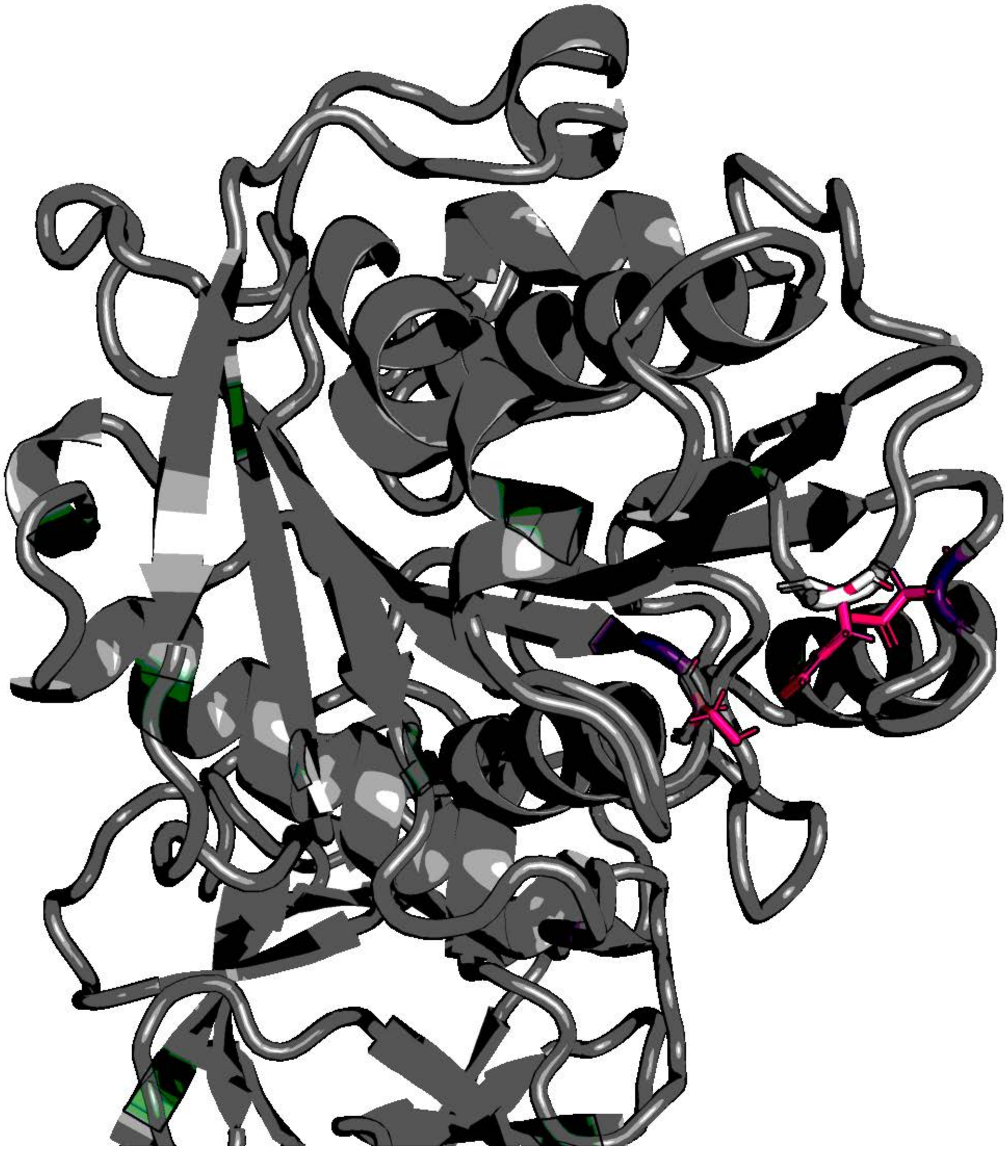

ESM038_highlights.png

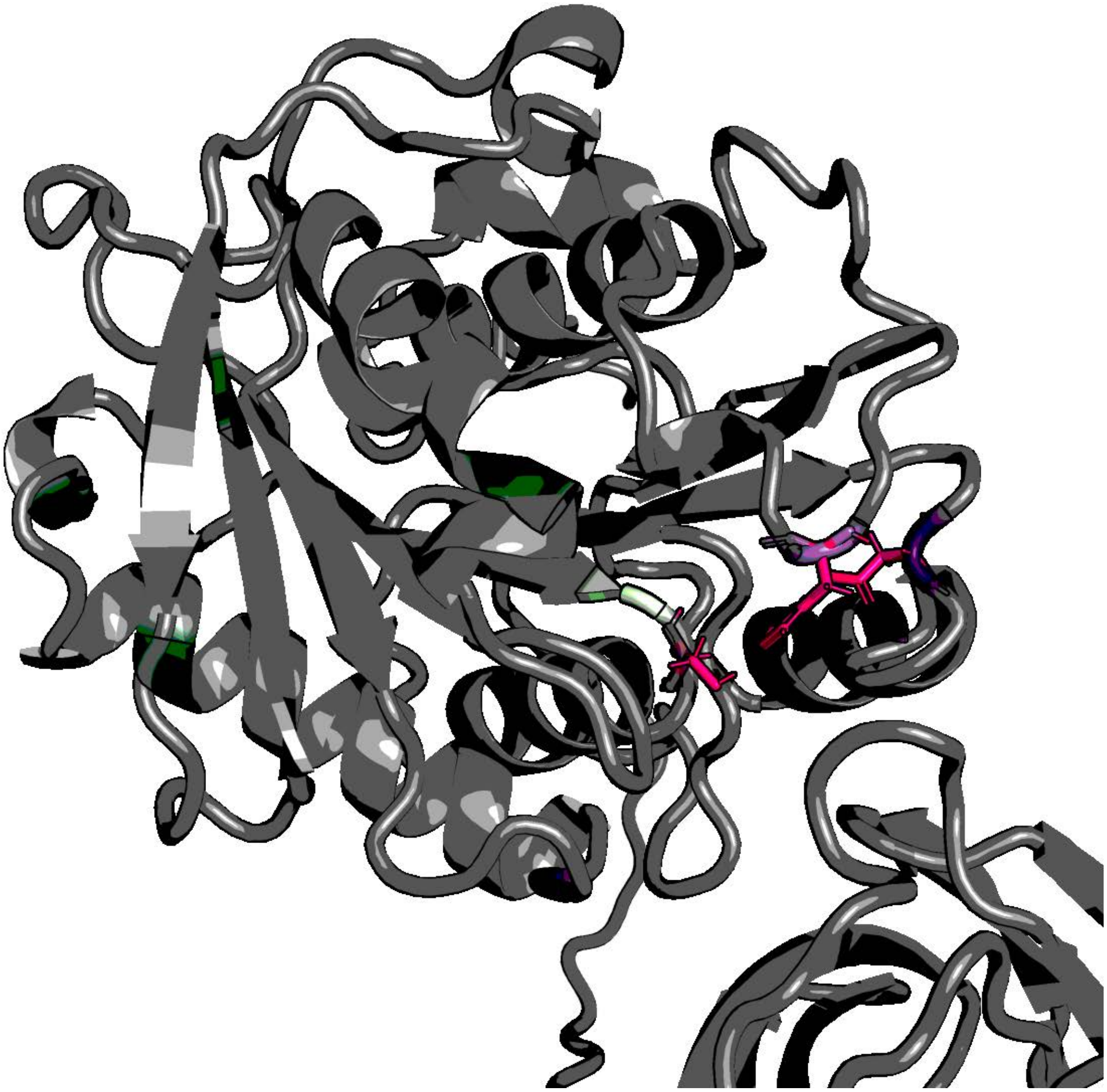

ESM043_highlights.png

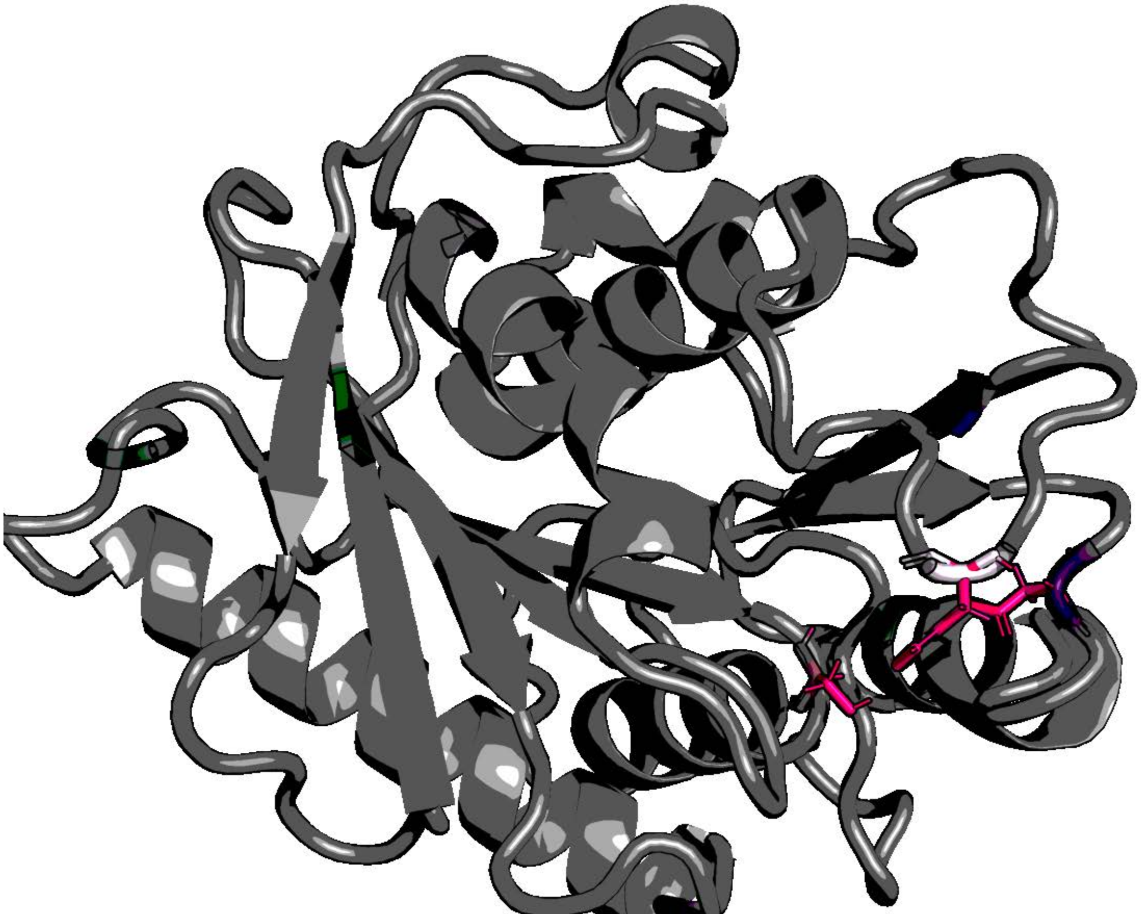

ESM045_highlights.png

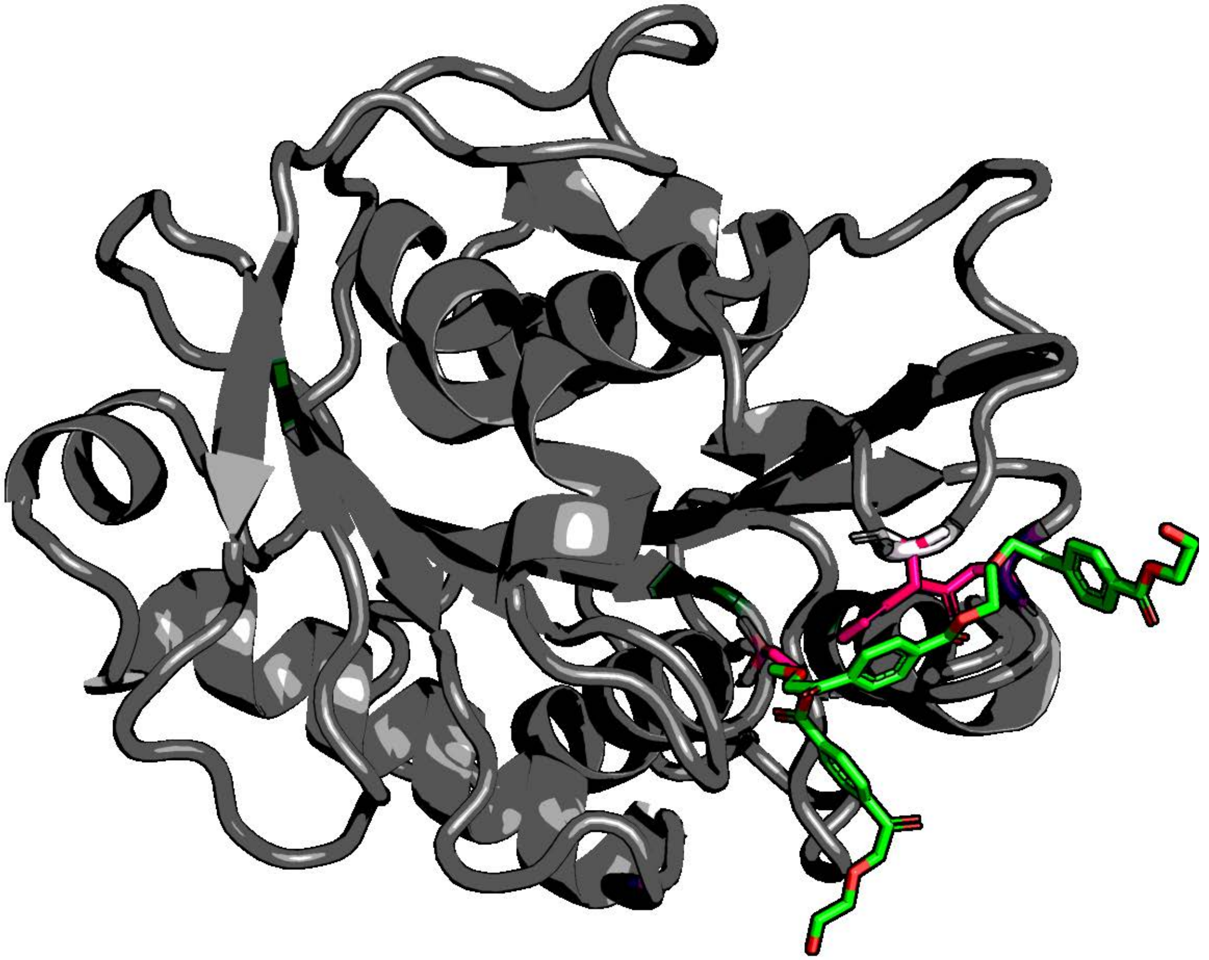

ESM047_highlights.png

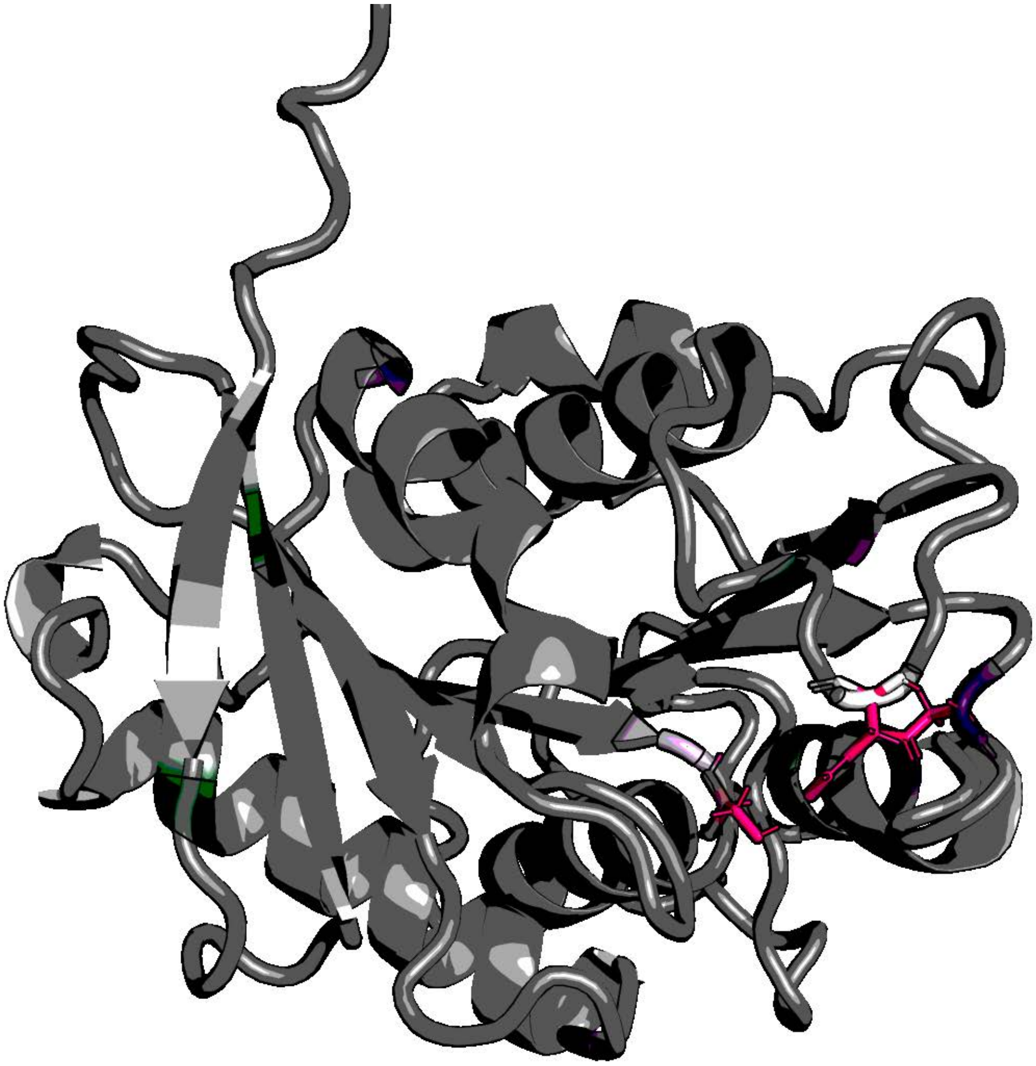

ESM050_highlights.png

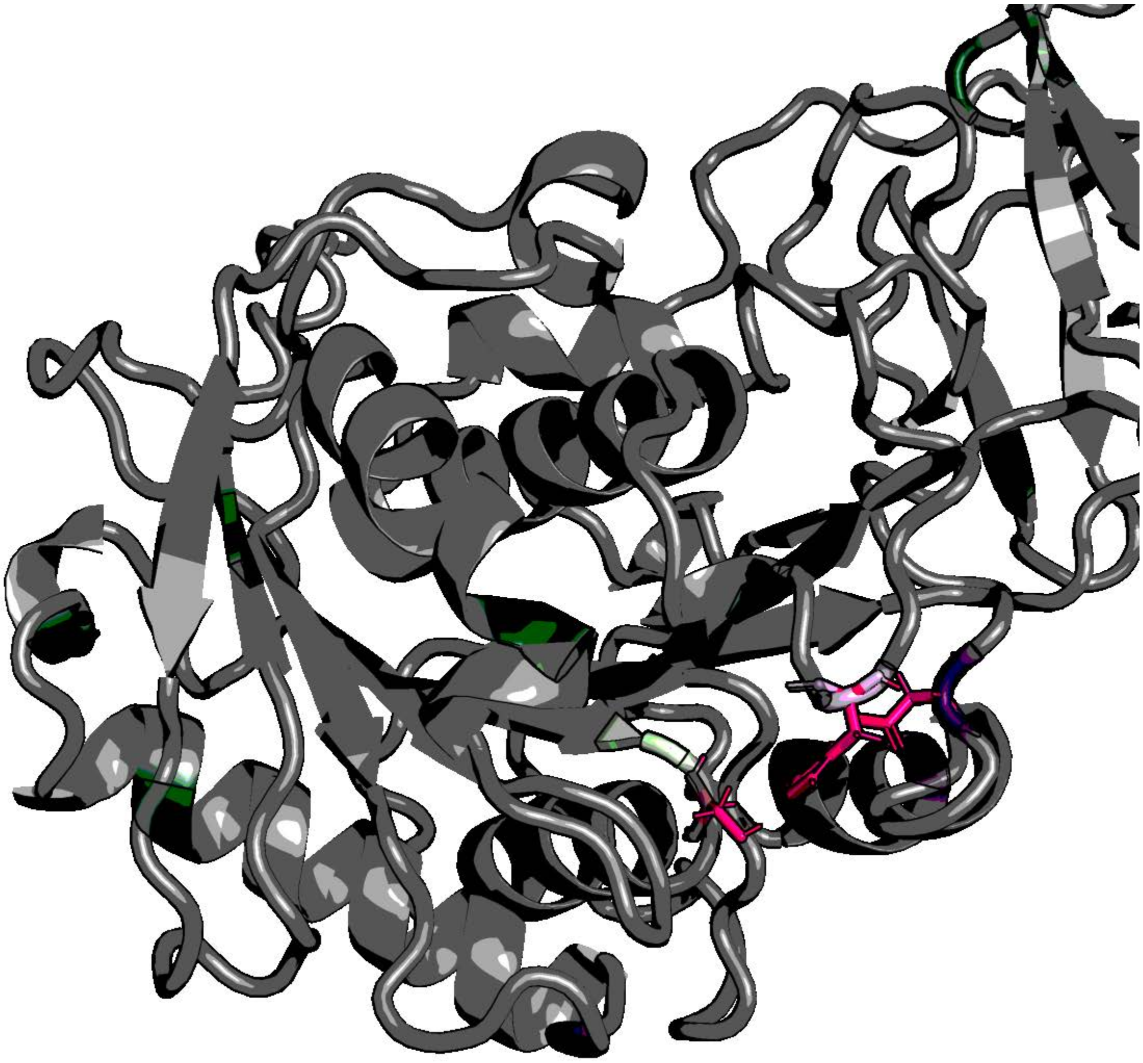

ESM052_highlights.png

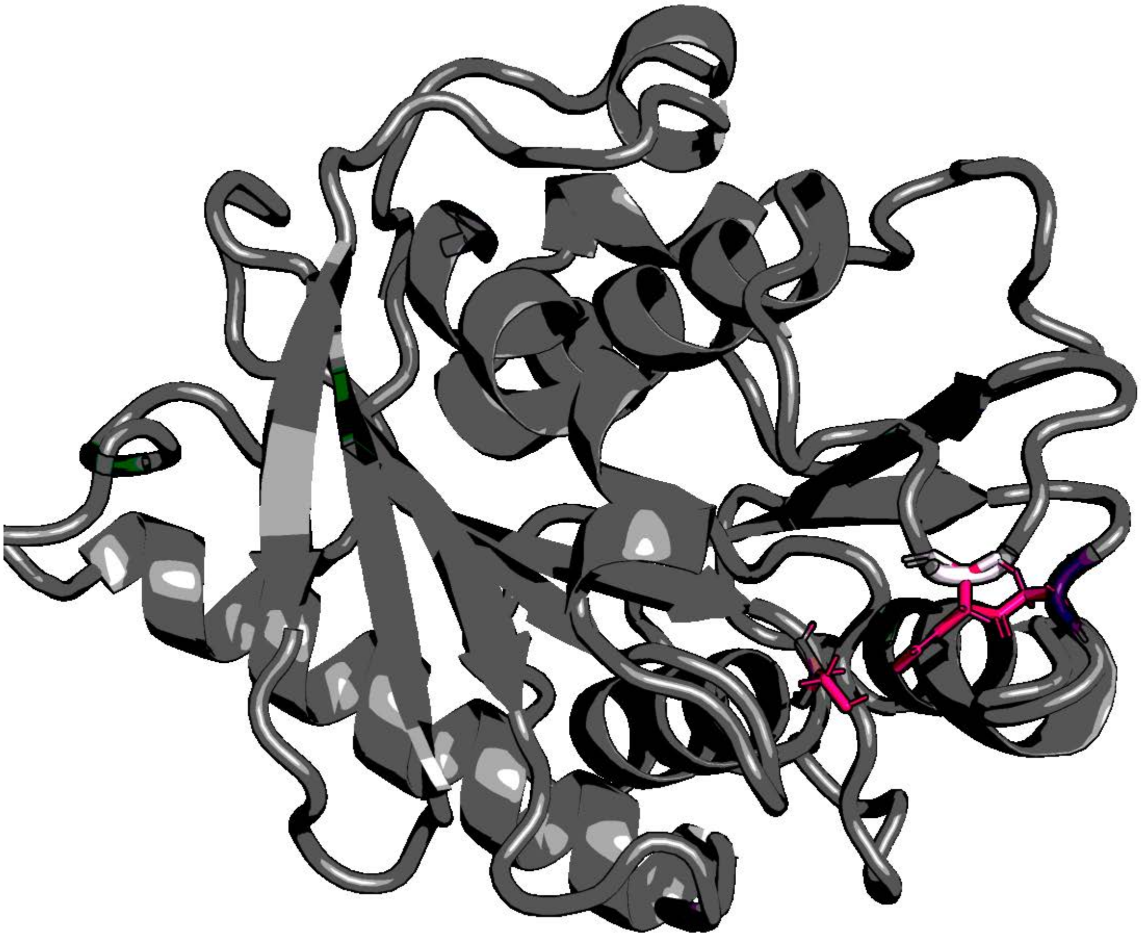

ESM053_highlights.png

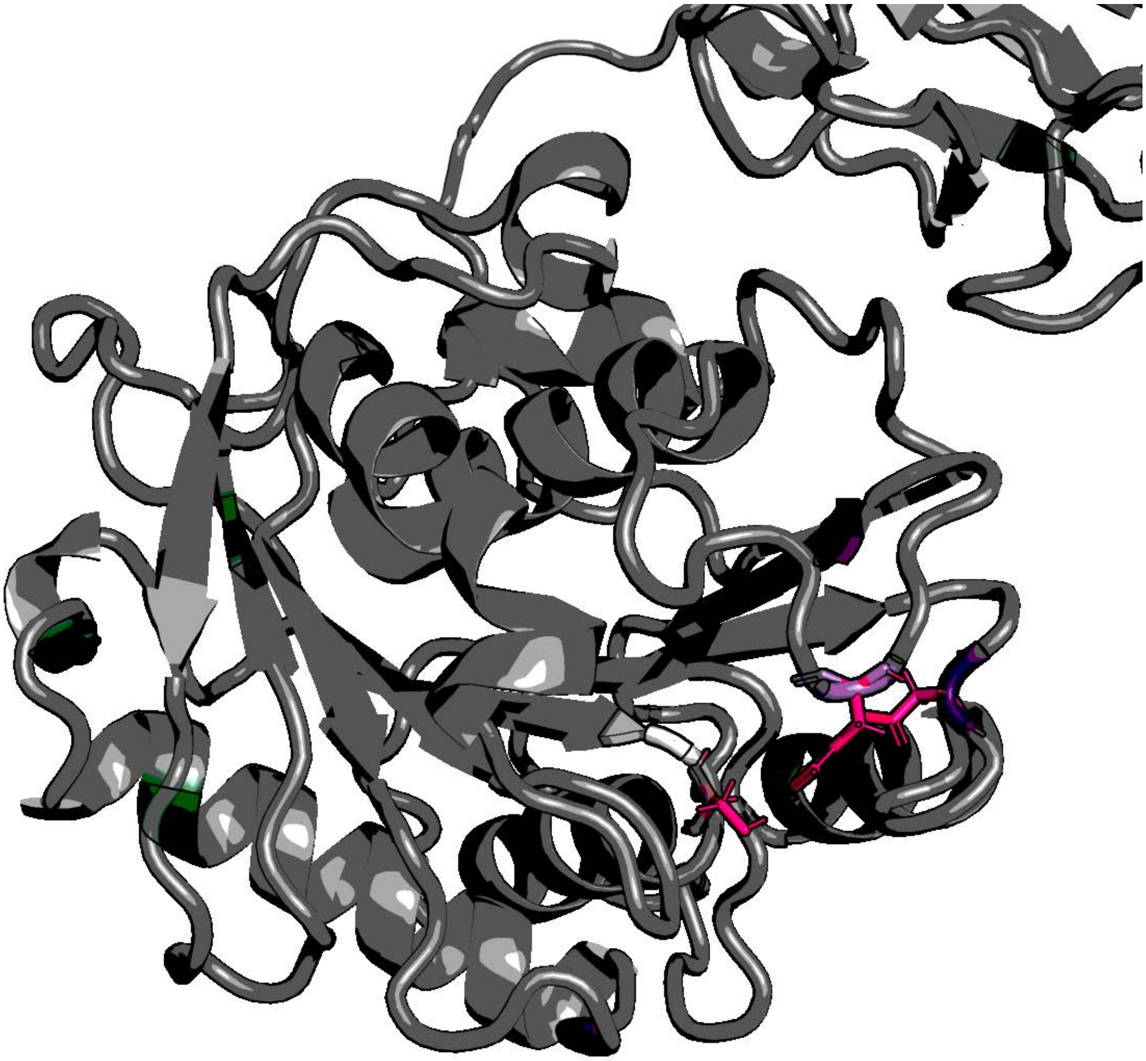

ESM058_highlights.png

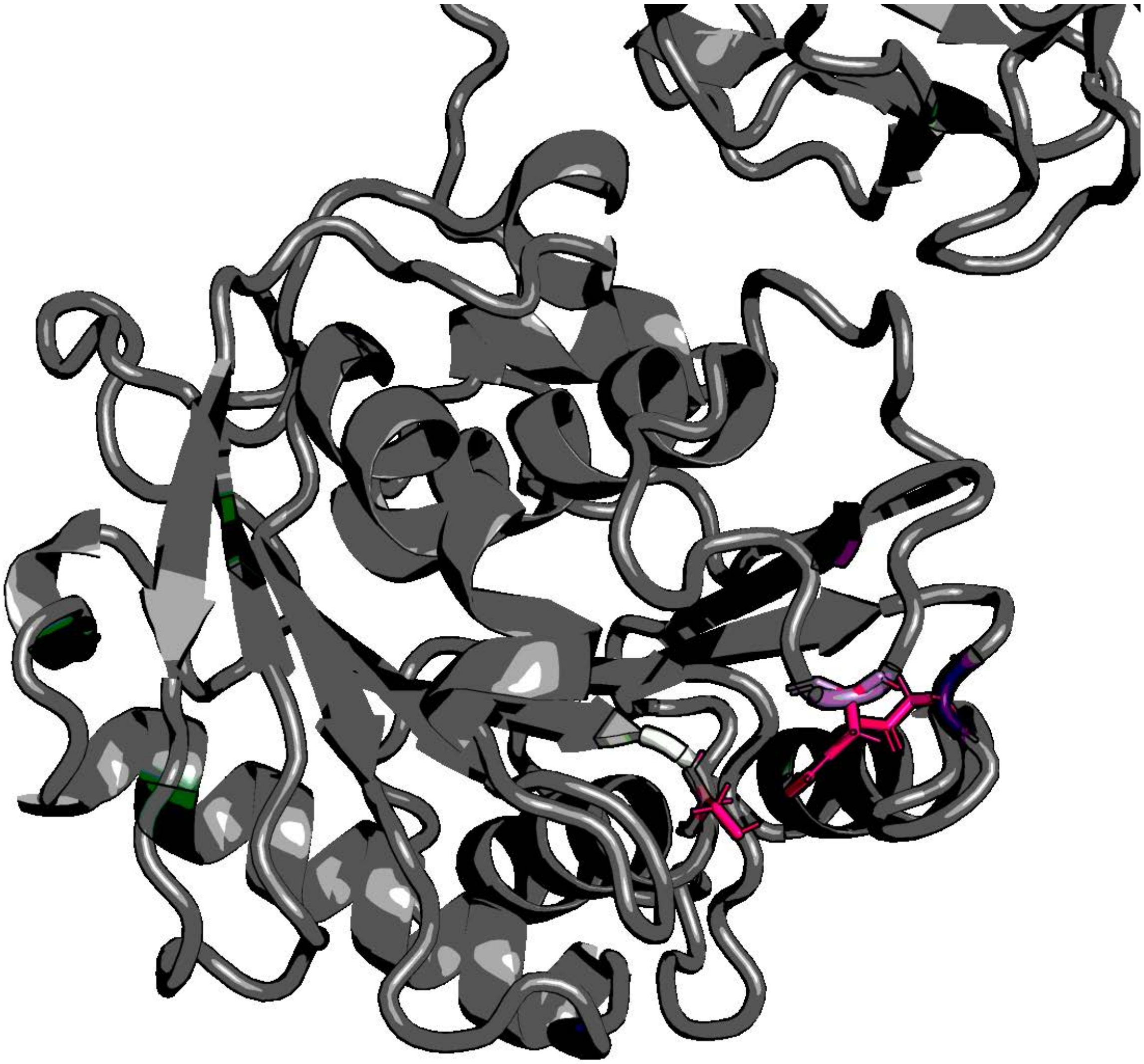

ESM059_highlights.png

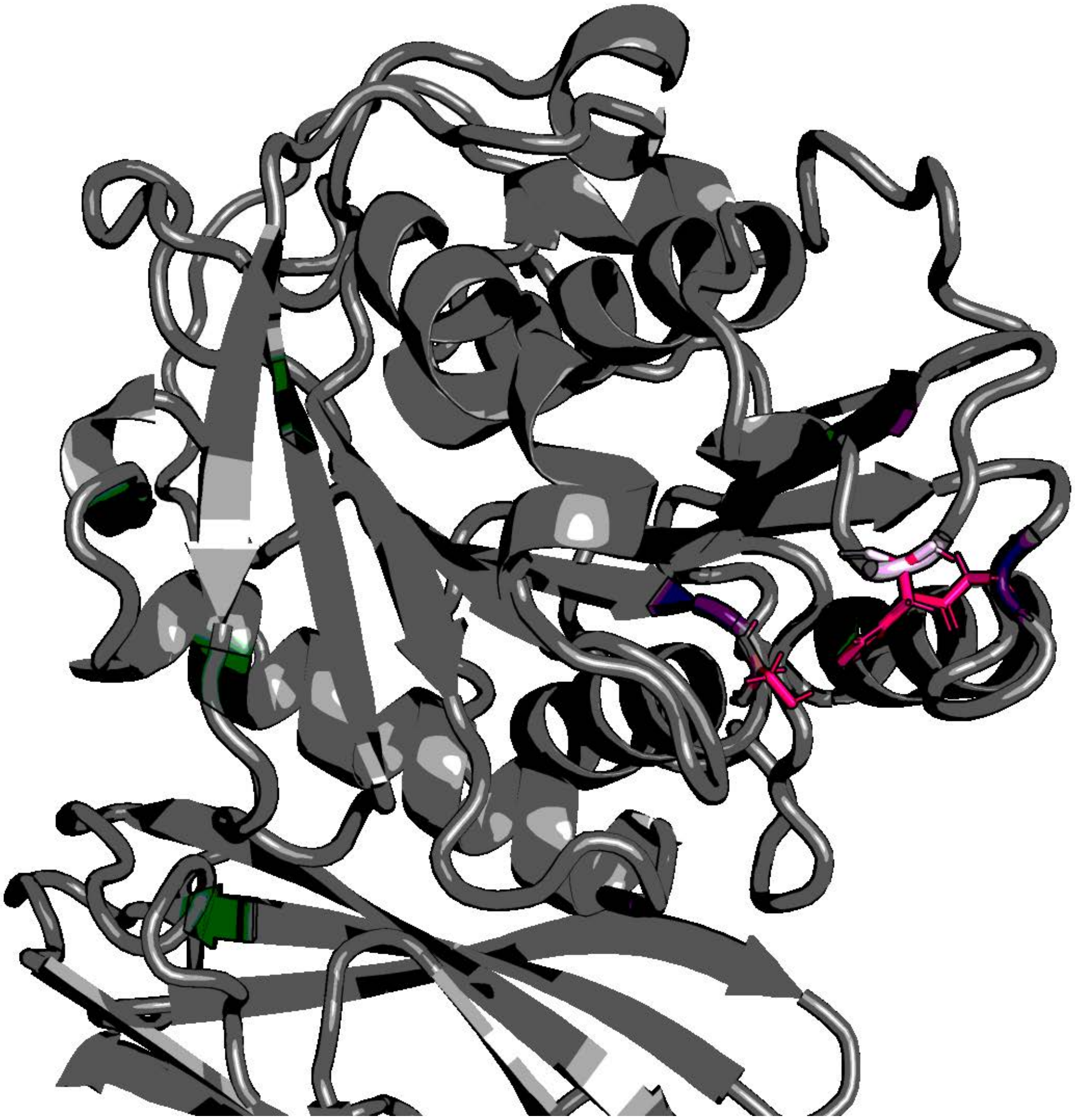

ESM060_highlights.png

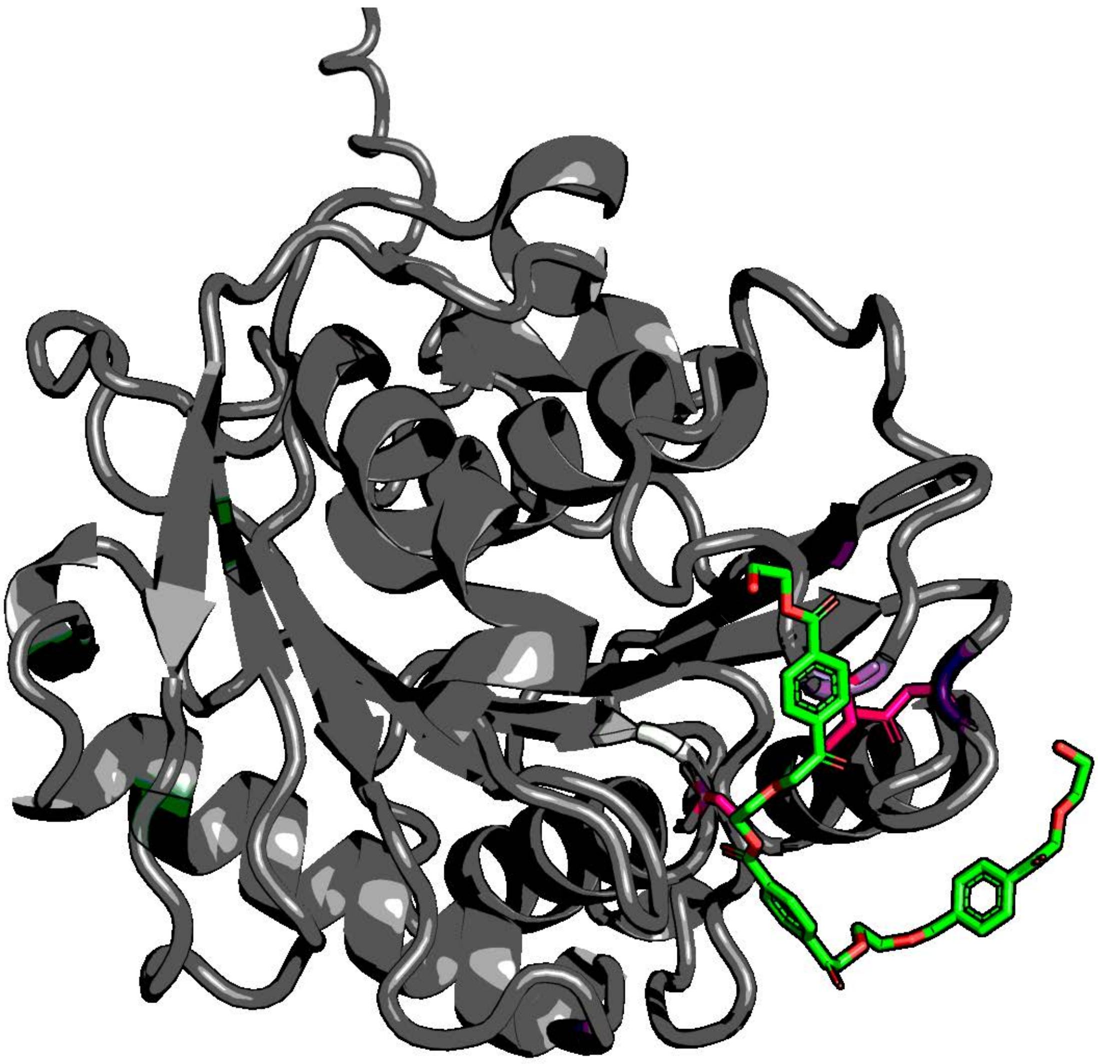

ESM061_highlights.png

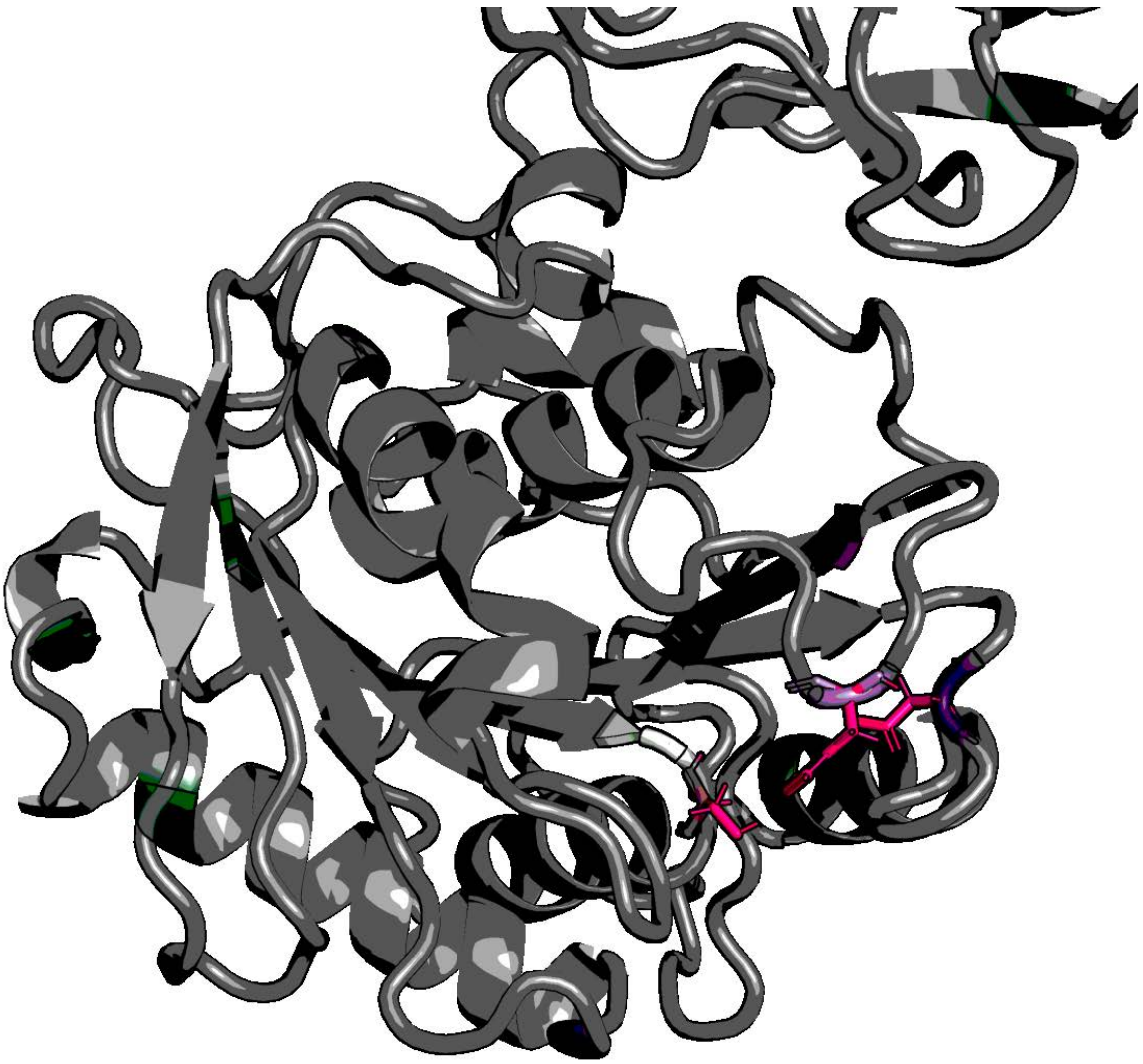

ESM062_highlights.png

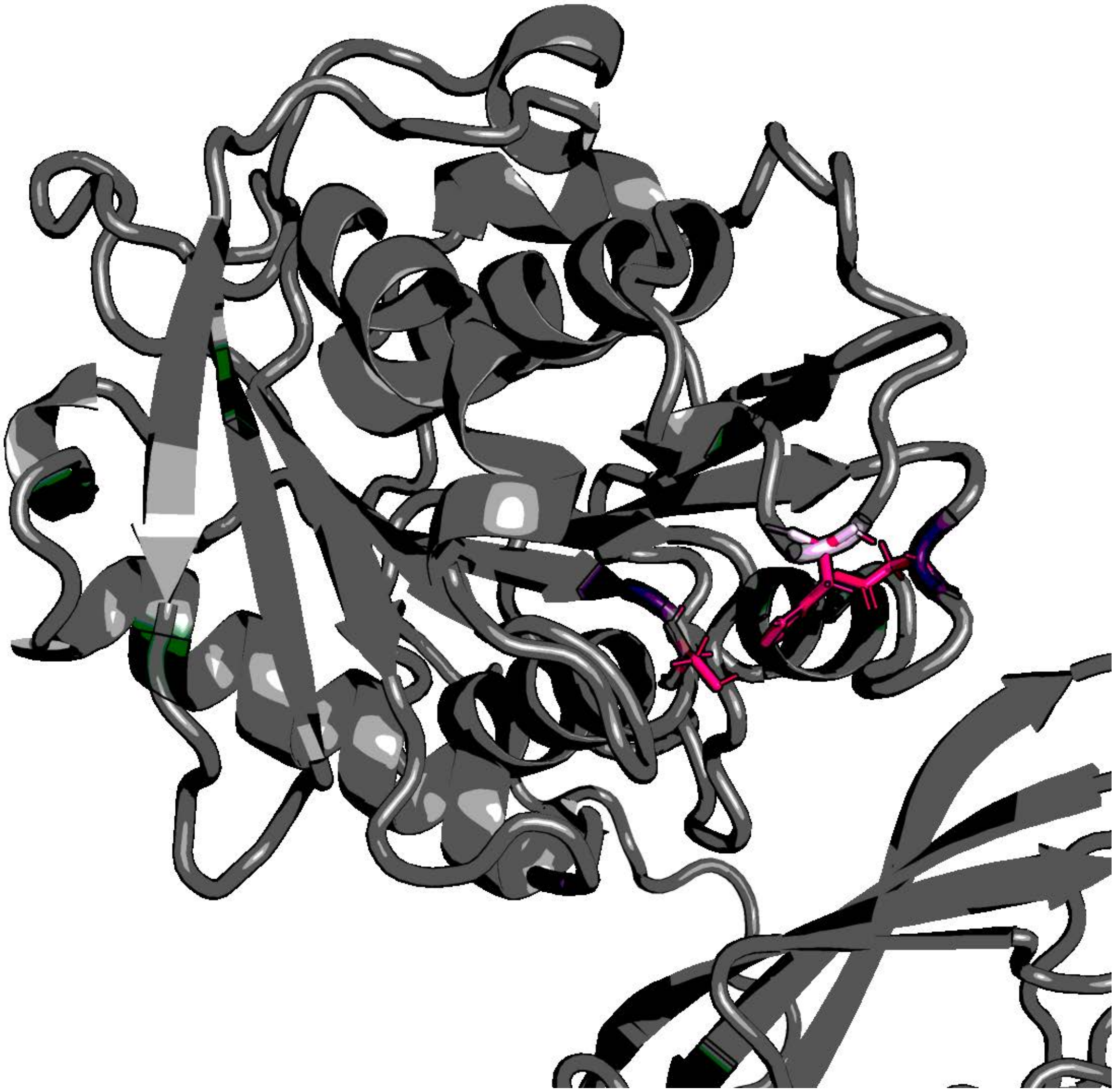

ESM064_highlights.png

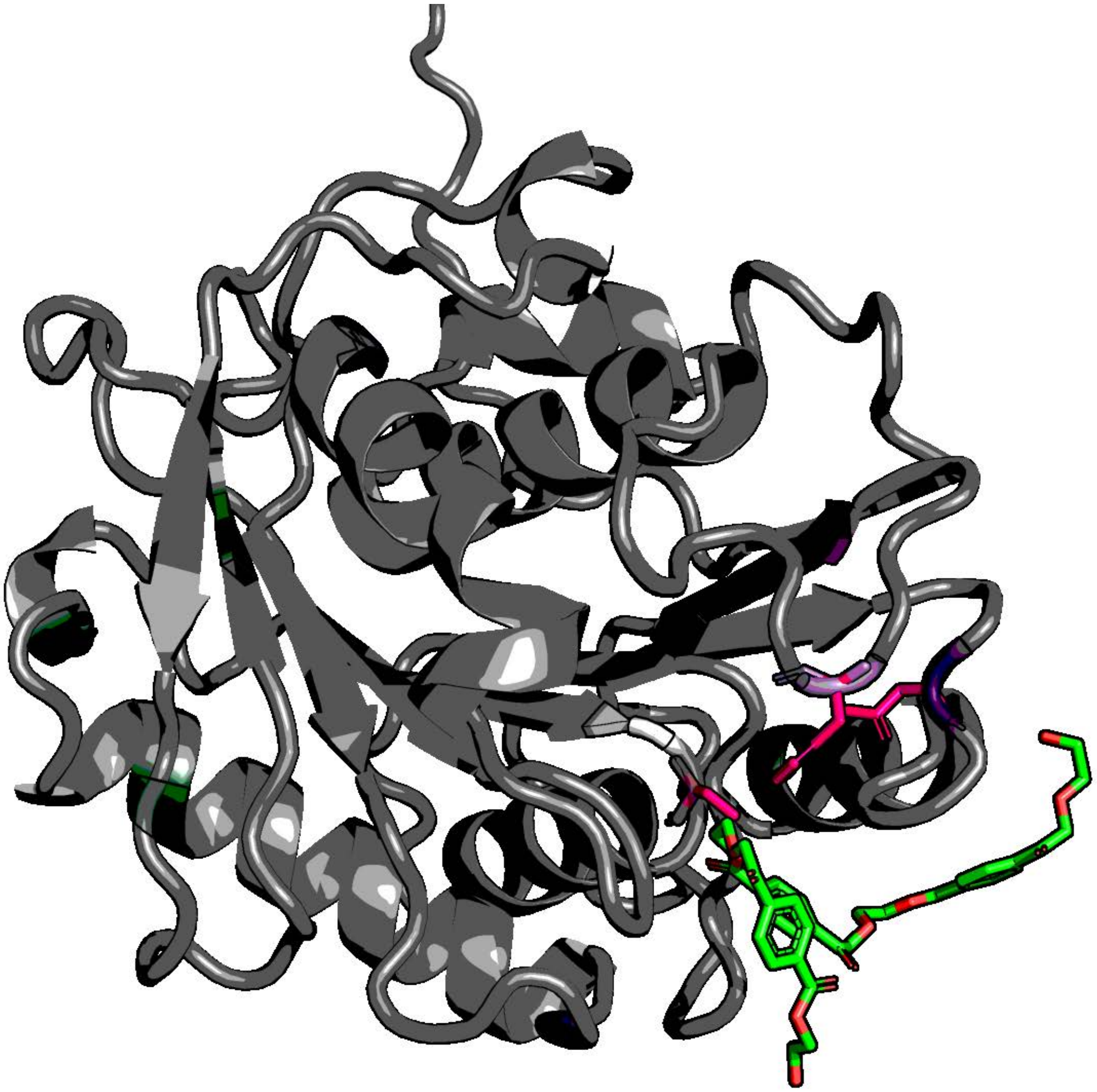

ESM065_highlights.png

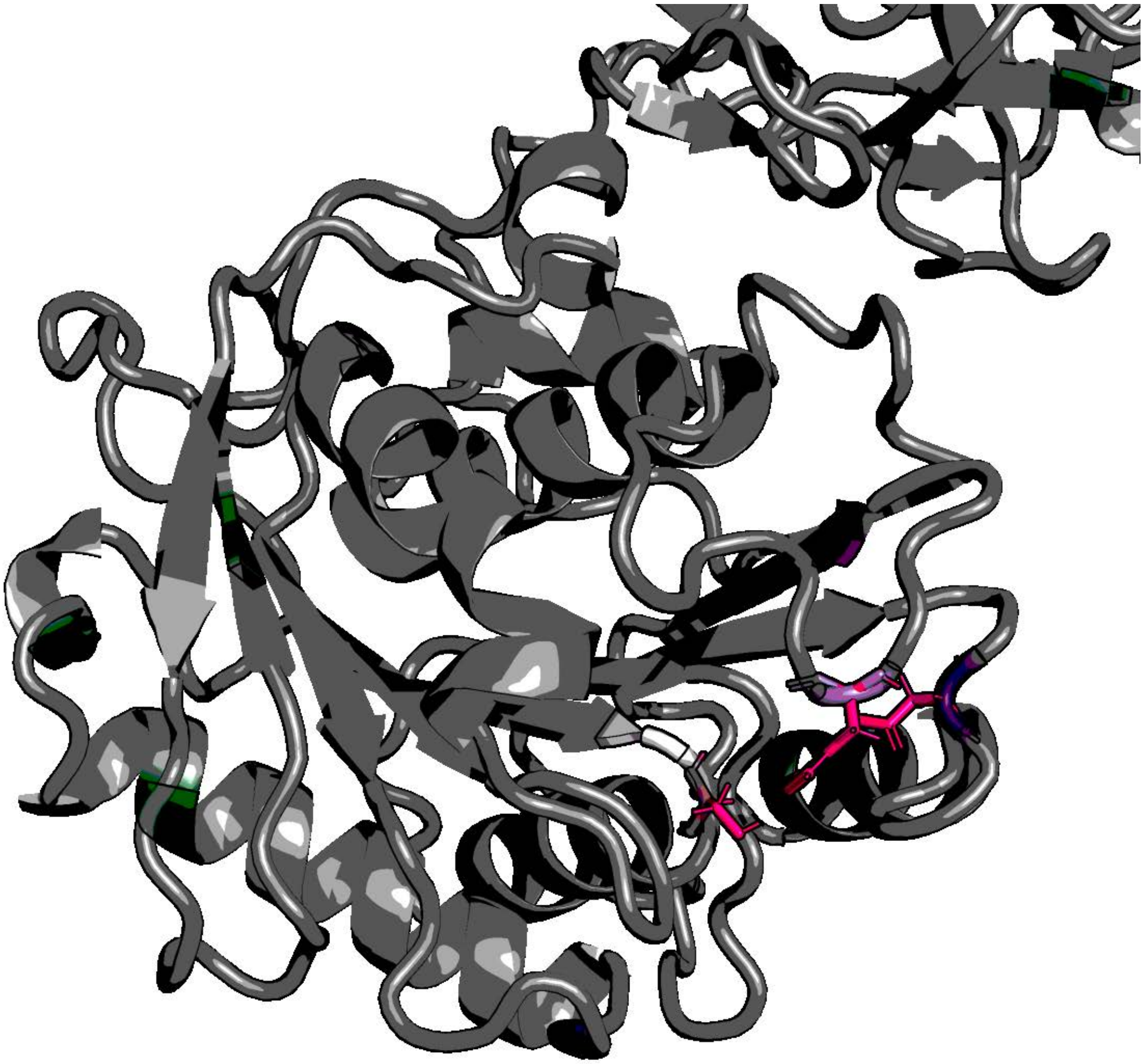

ESM069_highlights.png

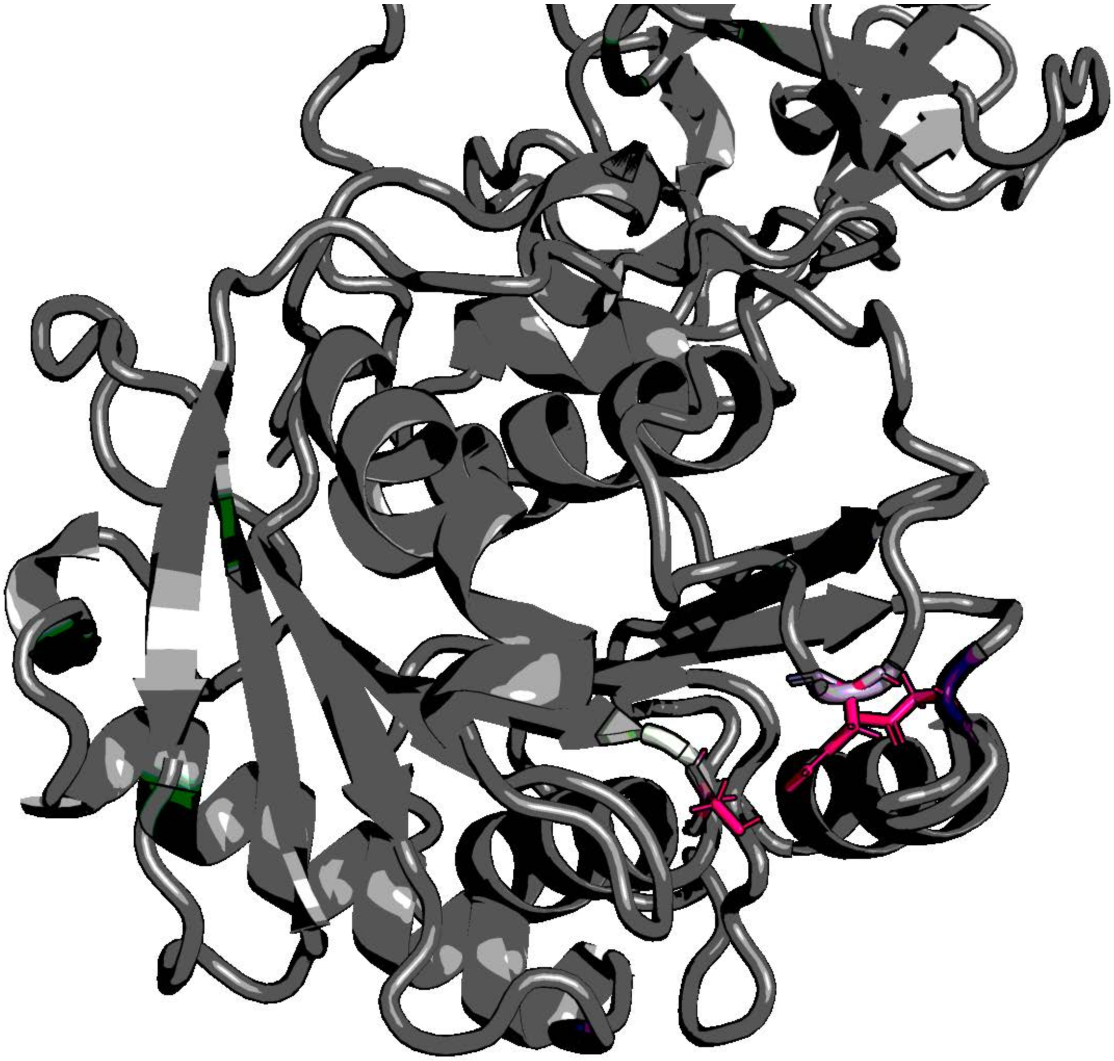

ESM070_highlights.png

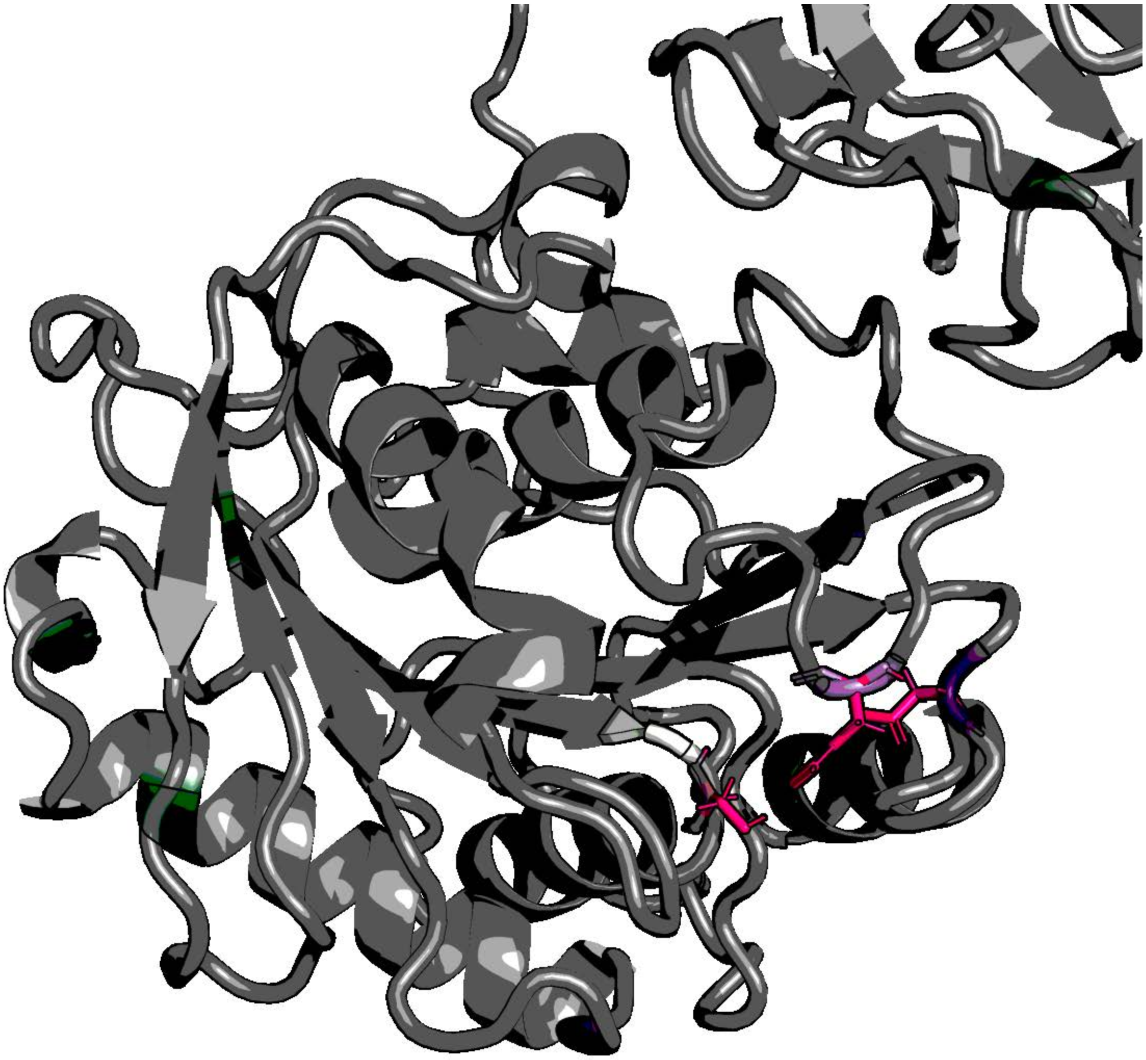

ESM075_highlights.png

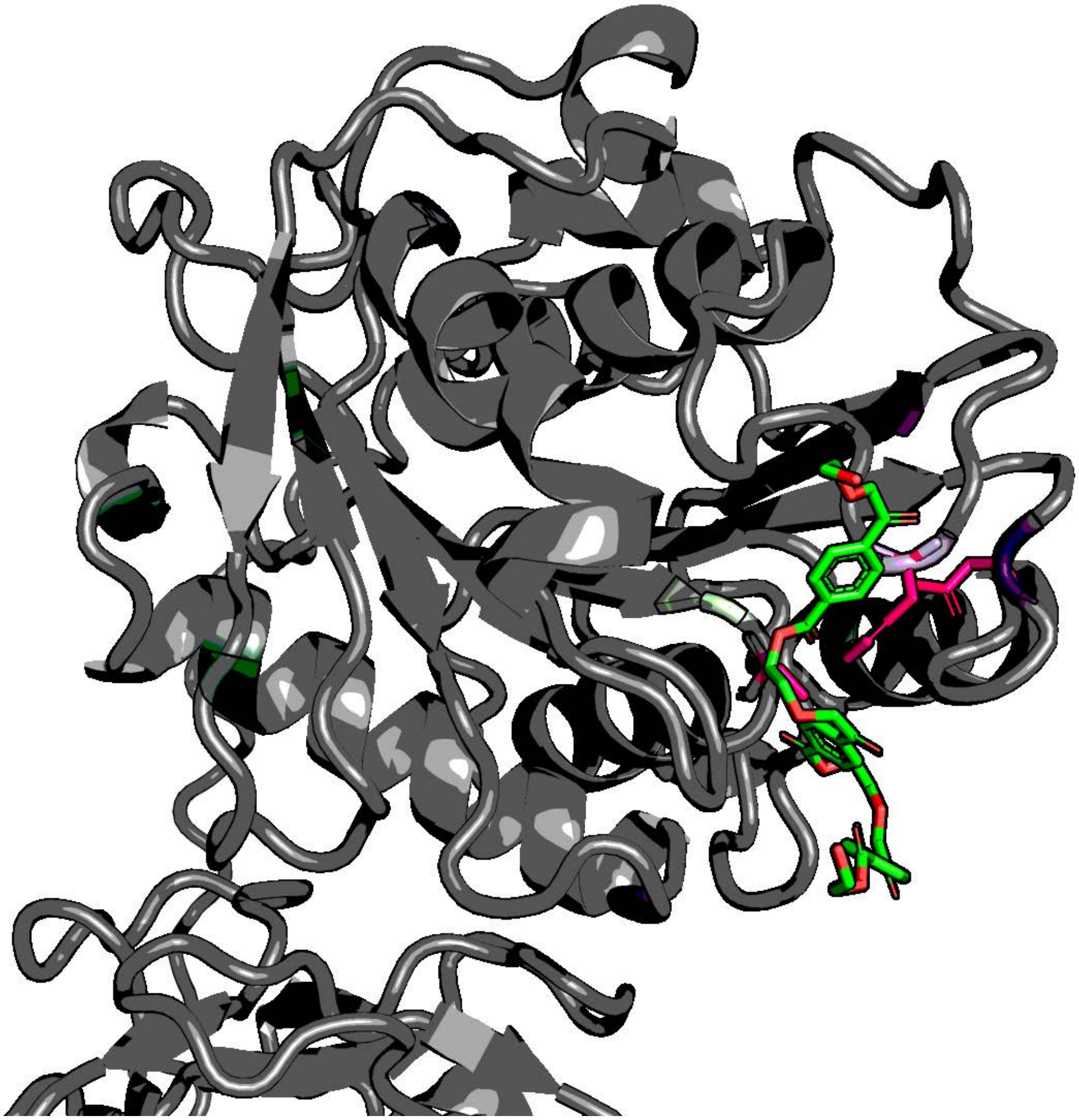

ESM080_highlights.png

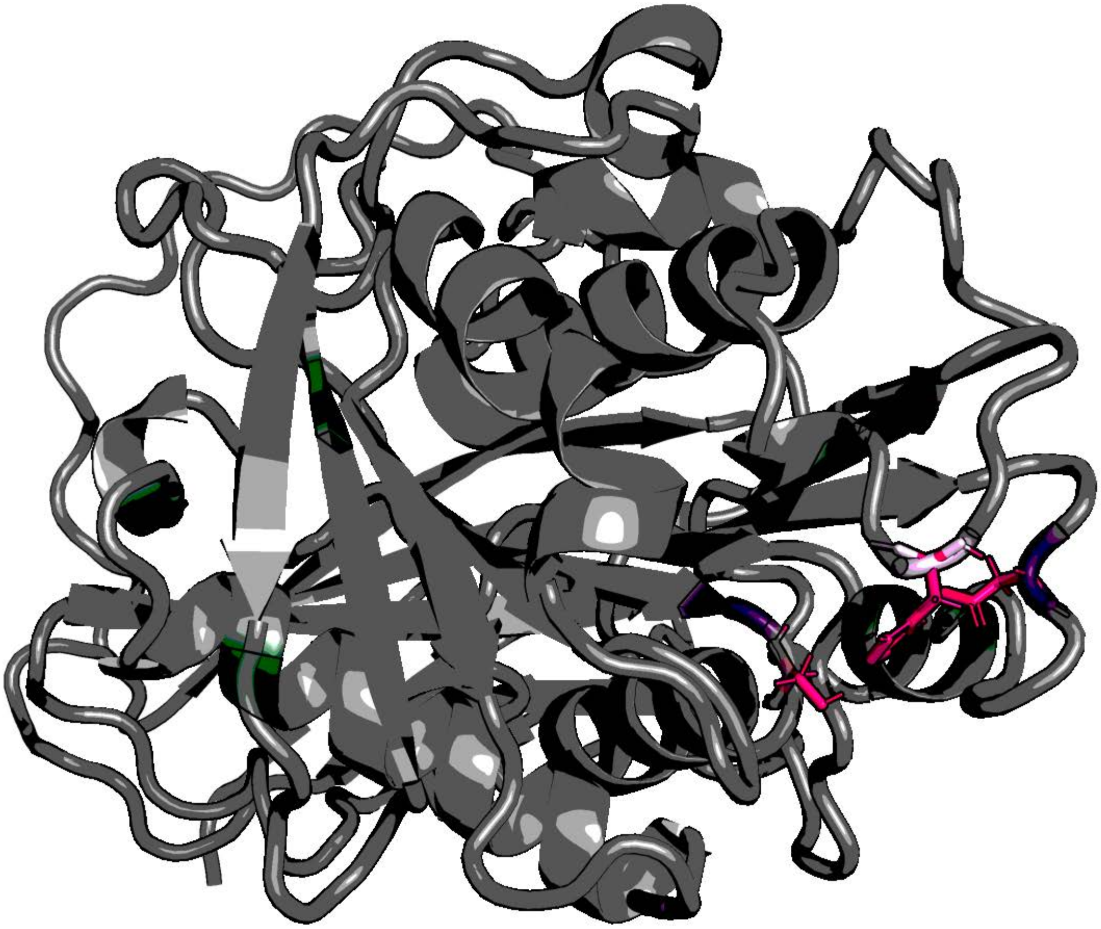

ESM081_highlights.png

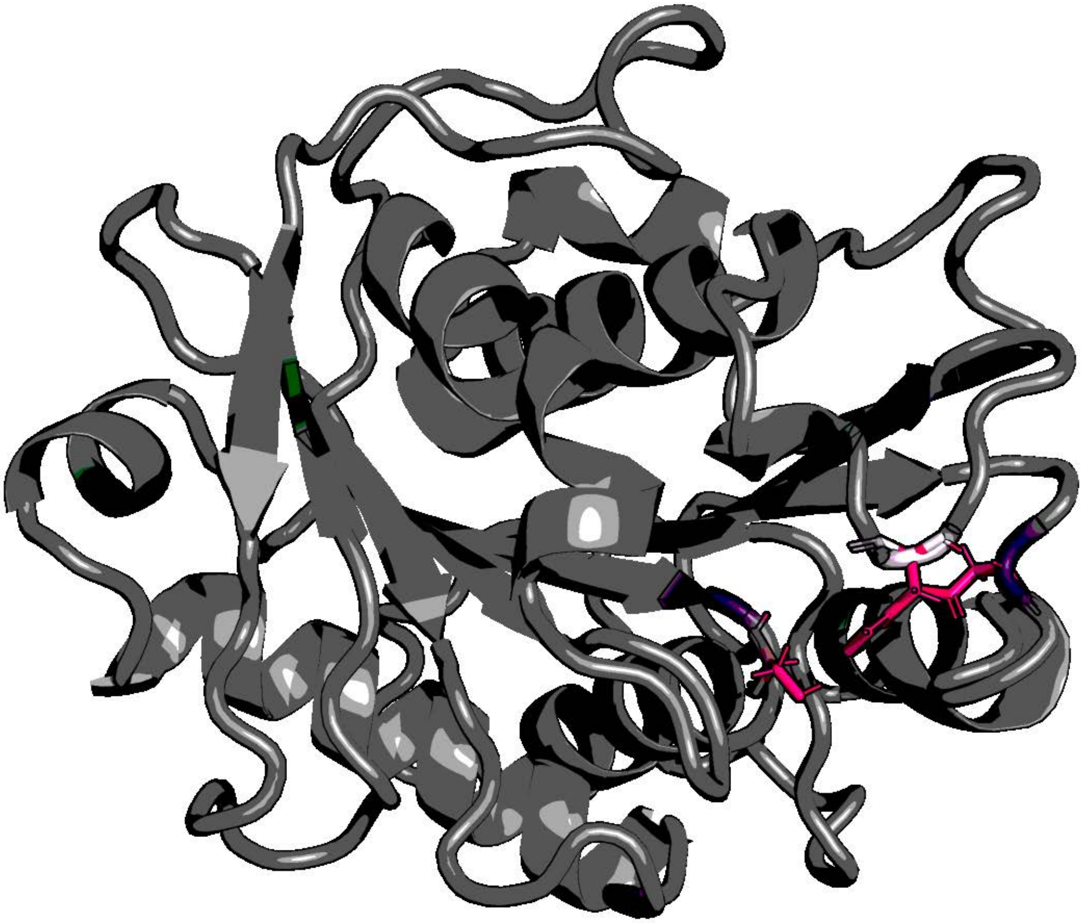

ESM082_highlights.png

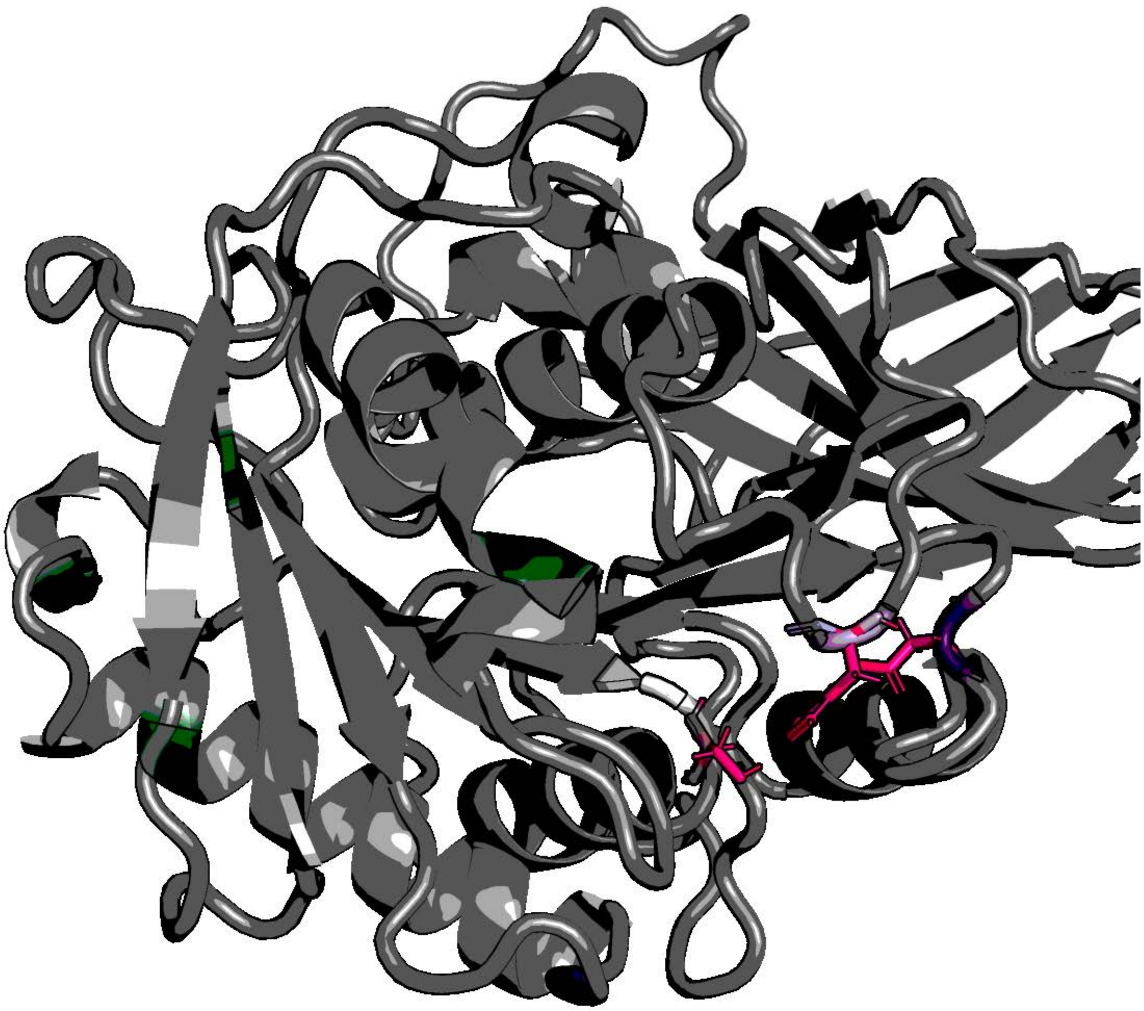

ESM084_highlights.png

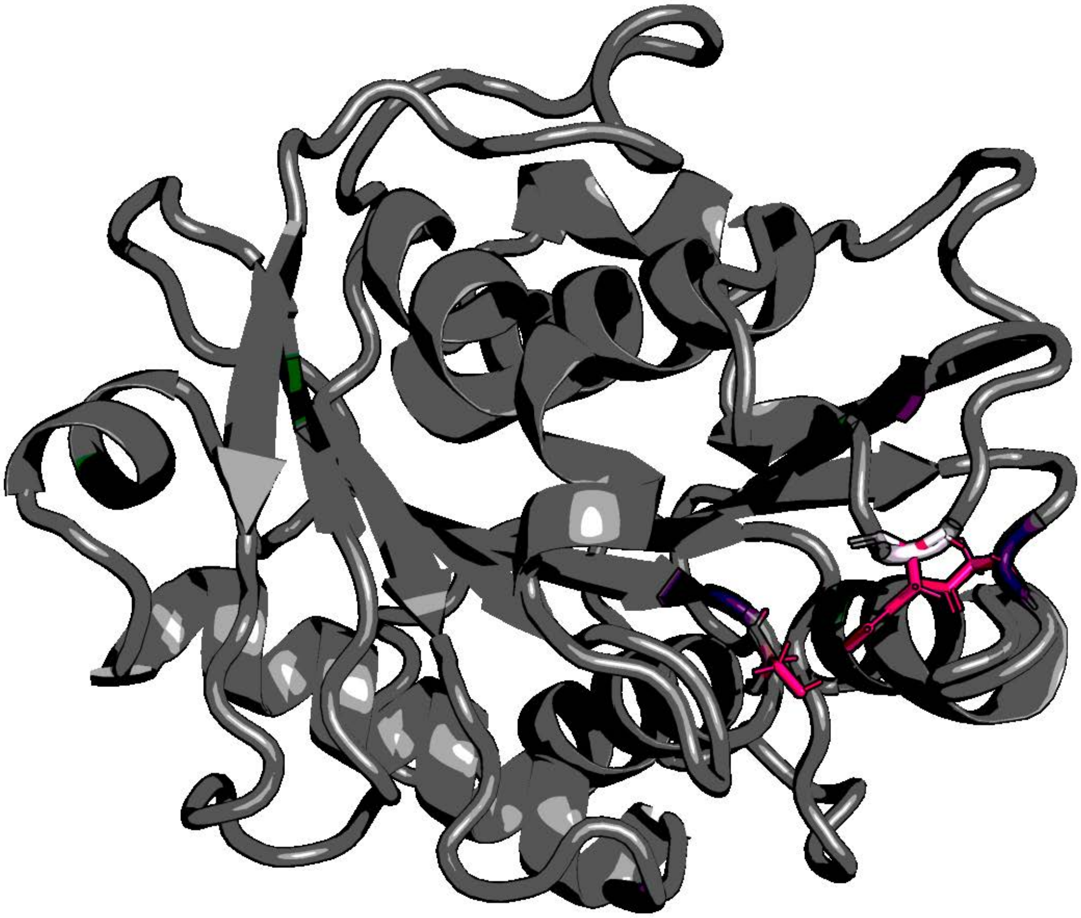

ESM090_highlights.png

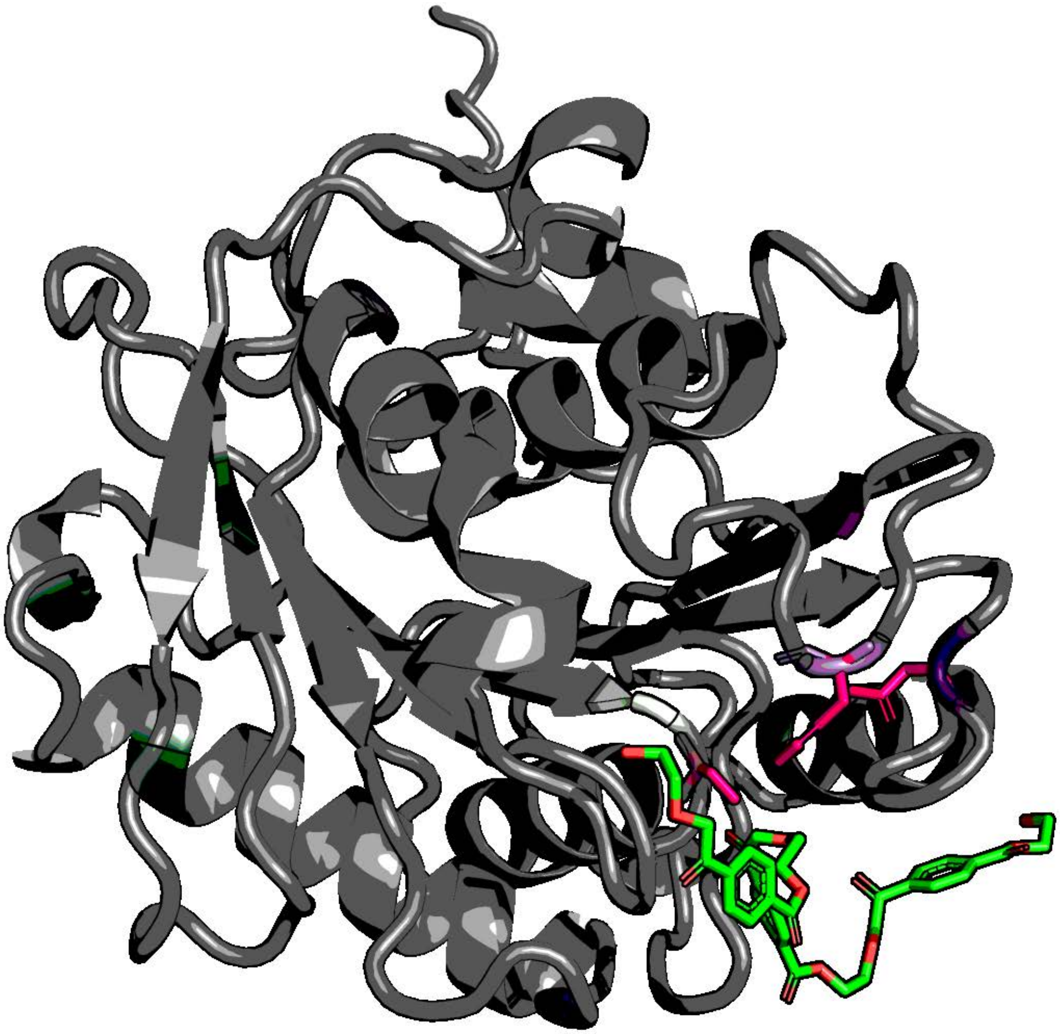

ESM091_highlights.png

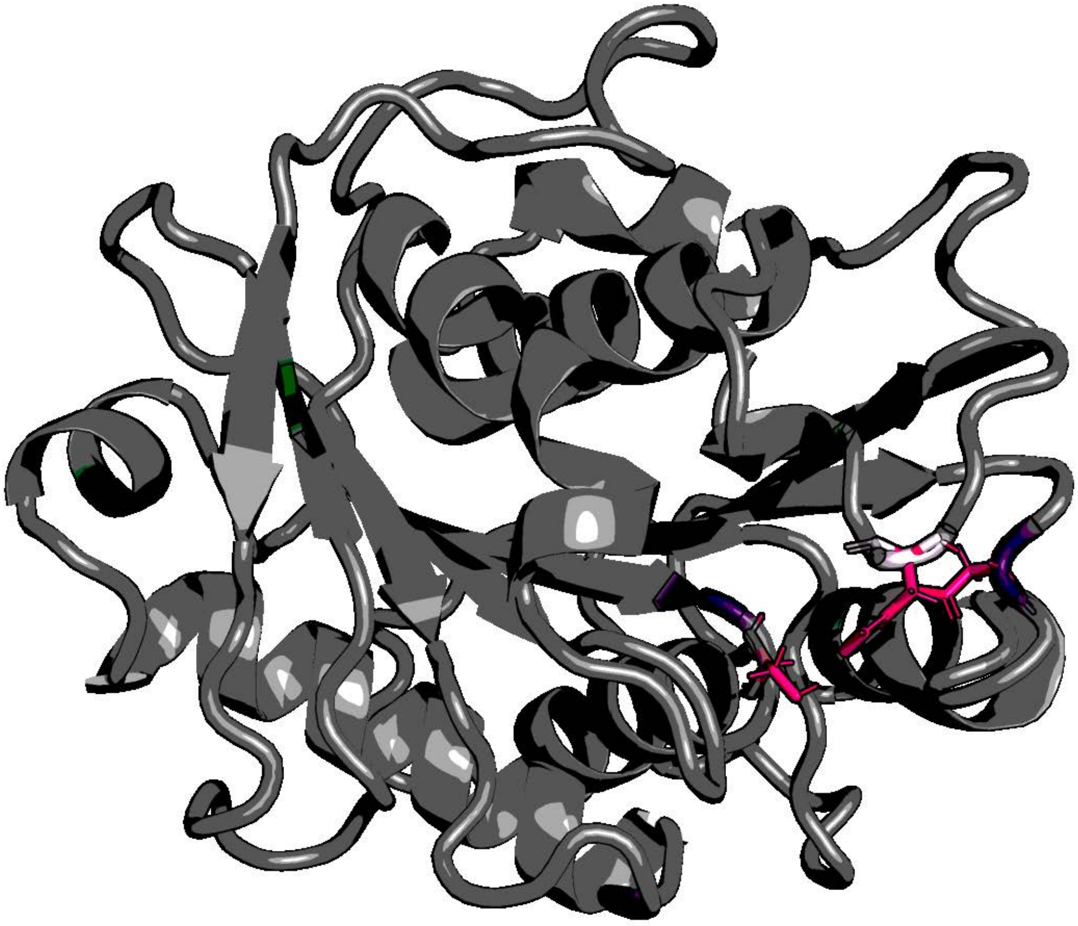

ESM093_highlights.png

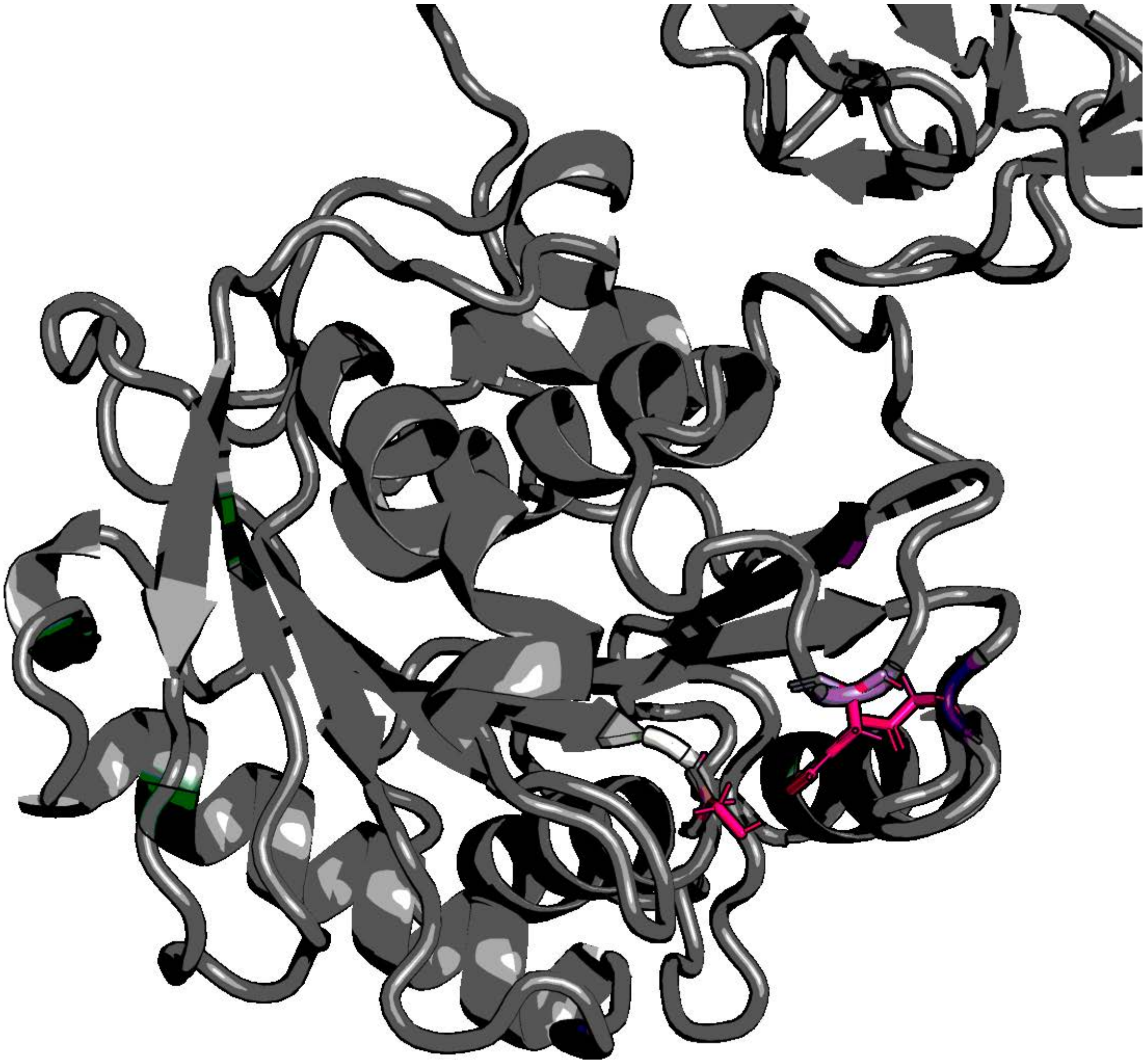

ESM095_highlights.png

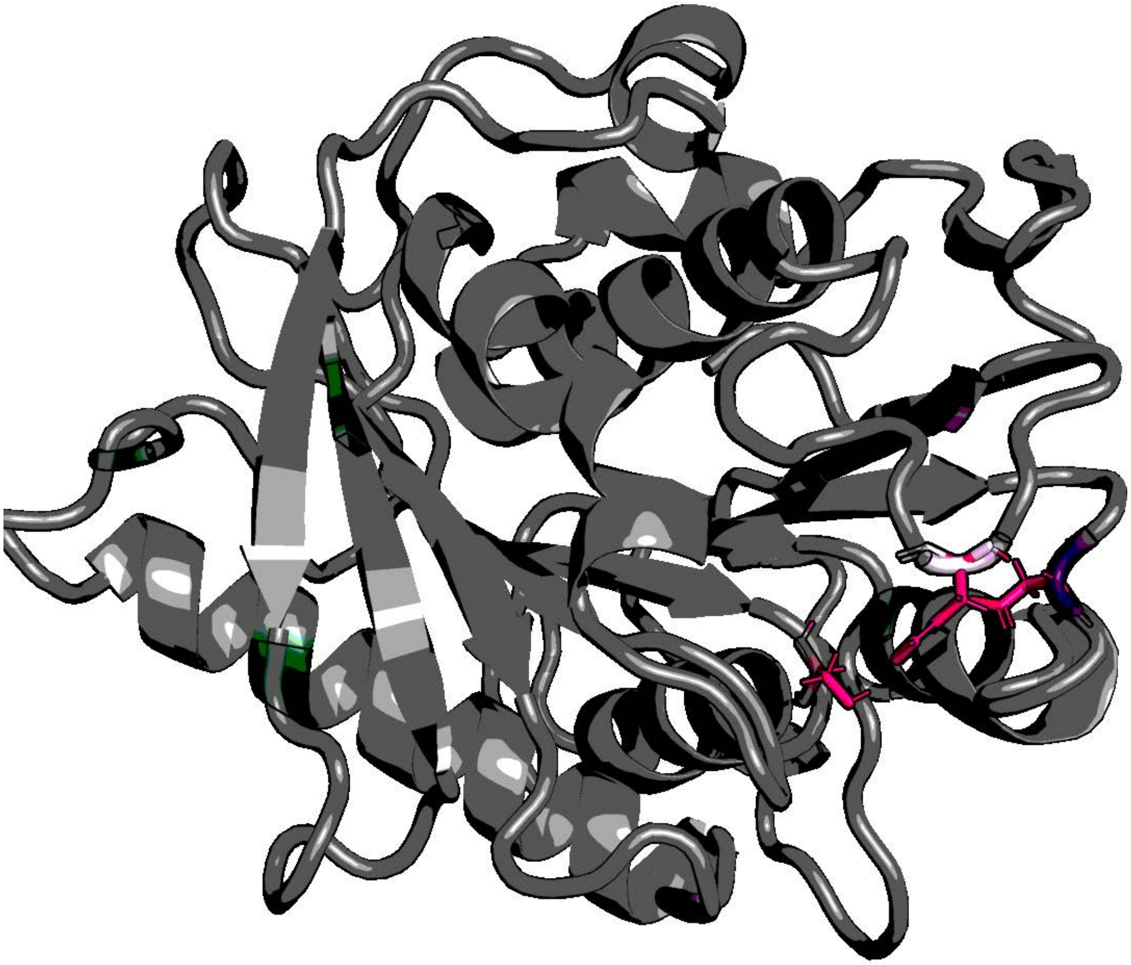

ESM096_highlights.png

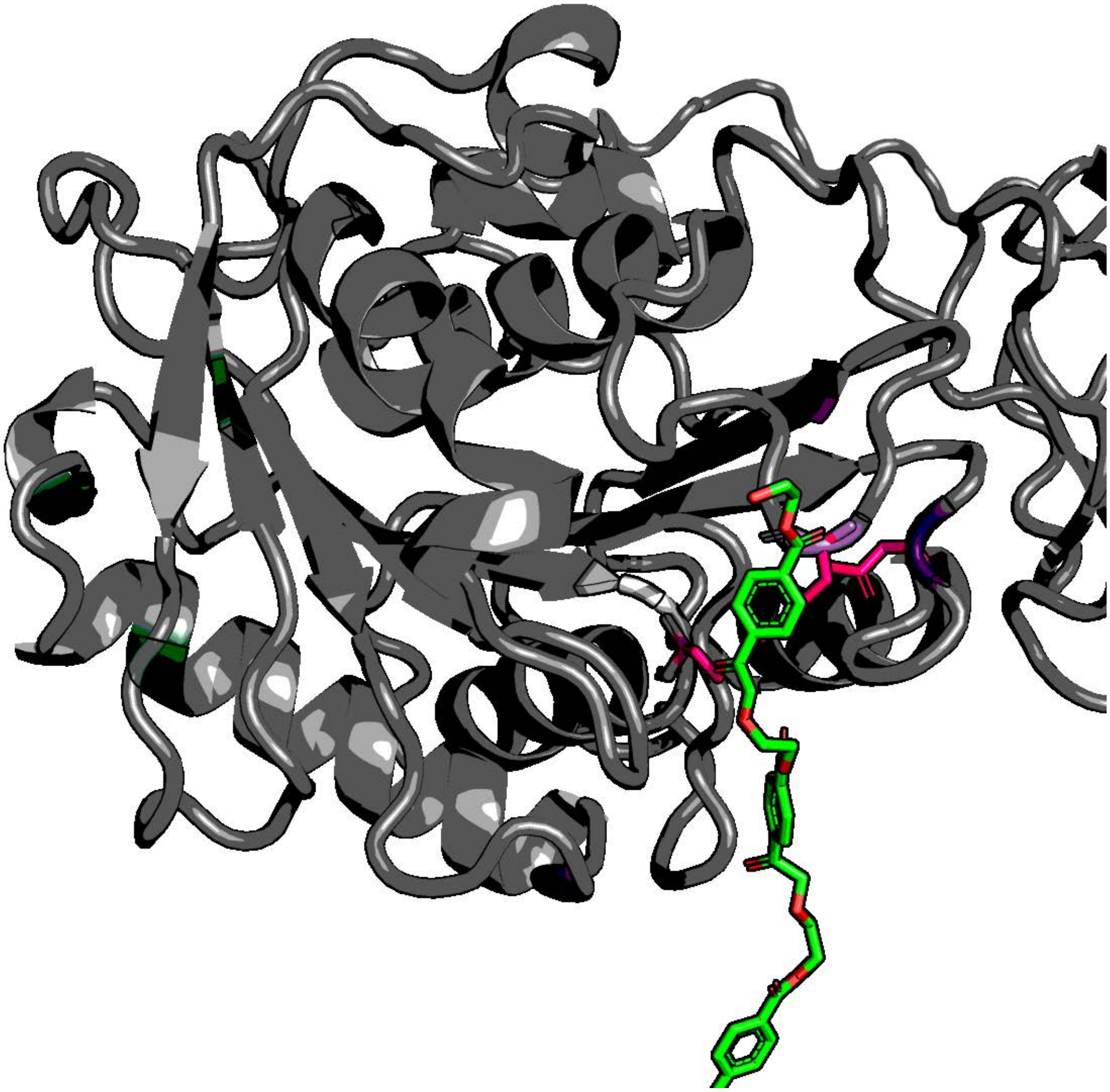

ESM101_highlights.png

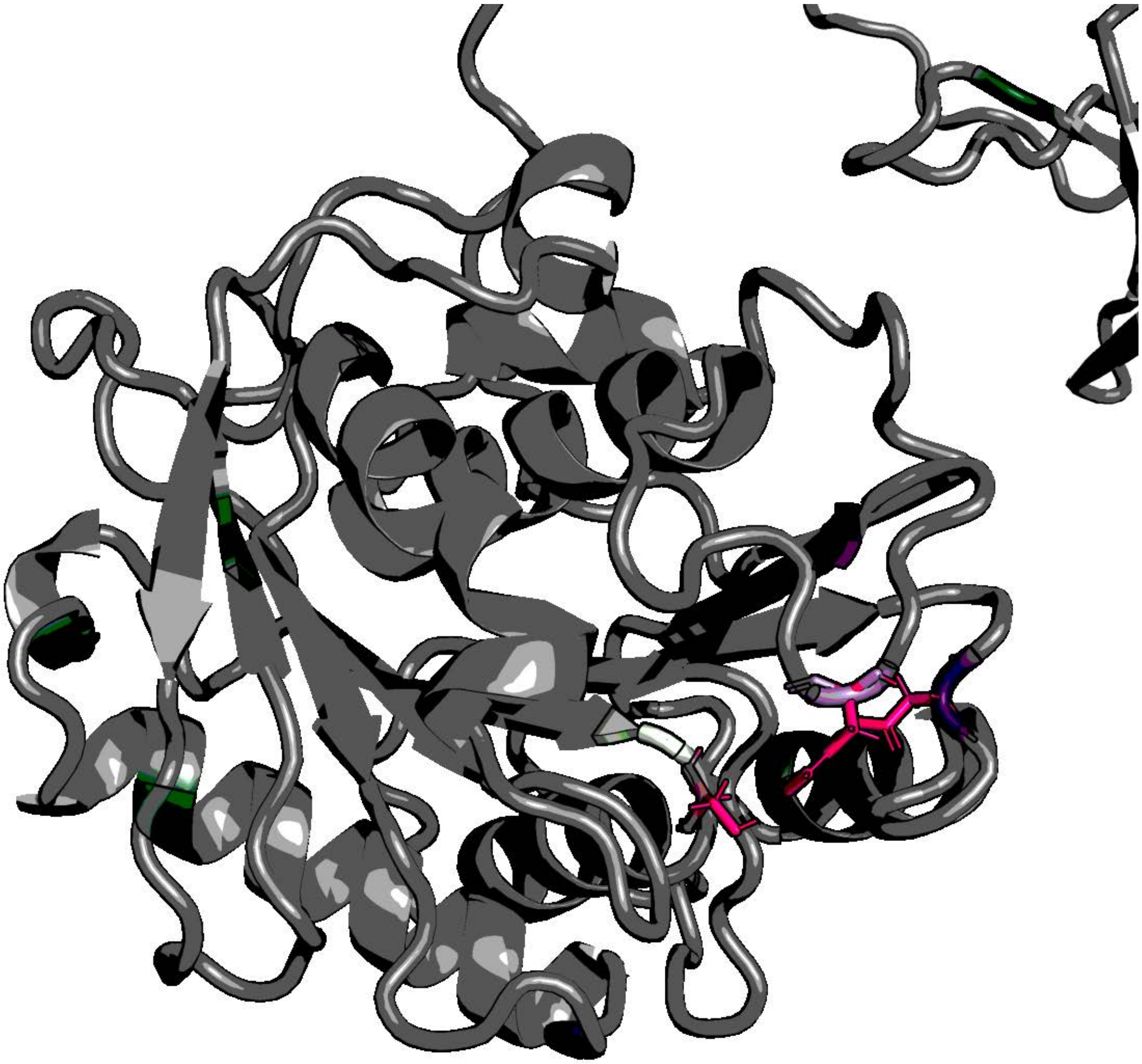

ESM106_highlights.png

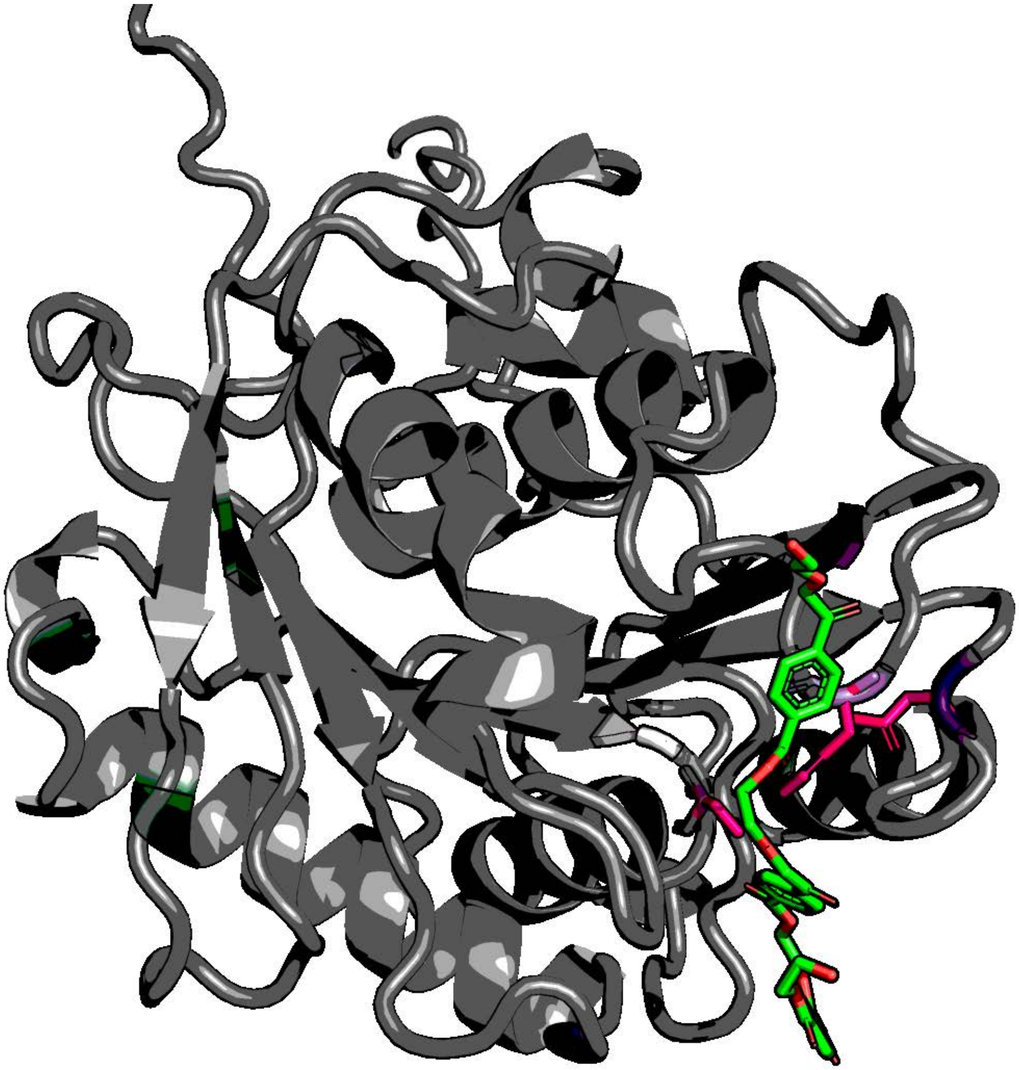

ESM121_highlights.png

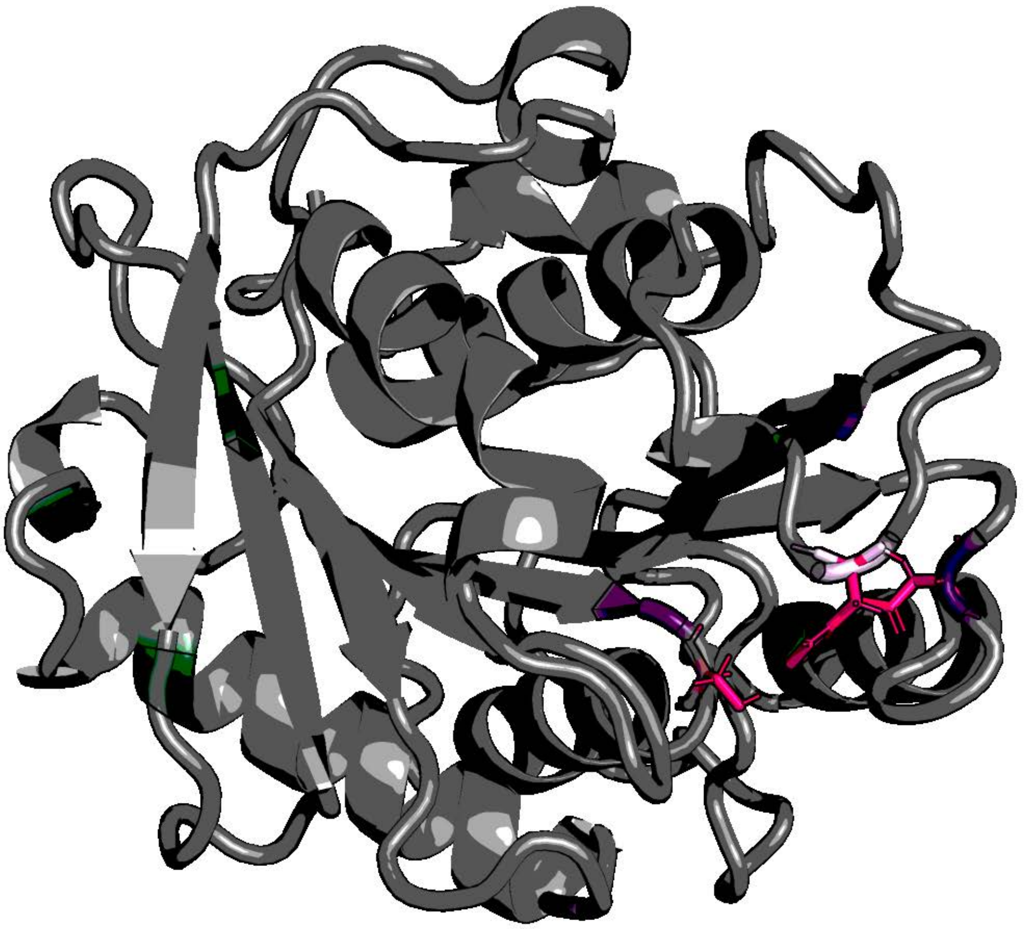

ESM128_highlights.png

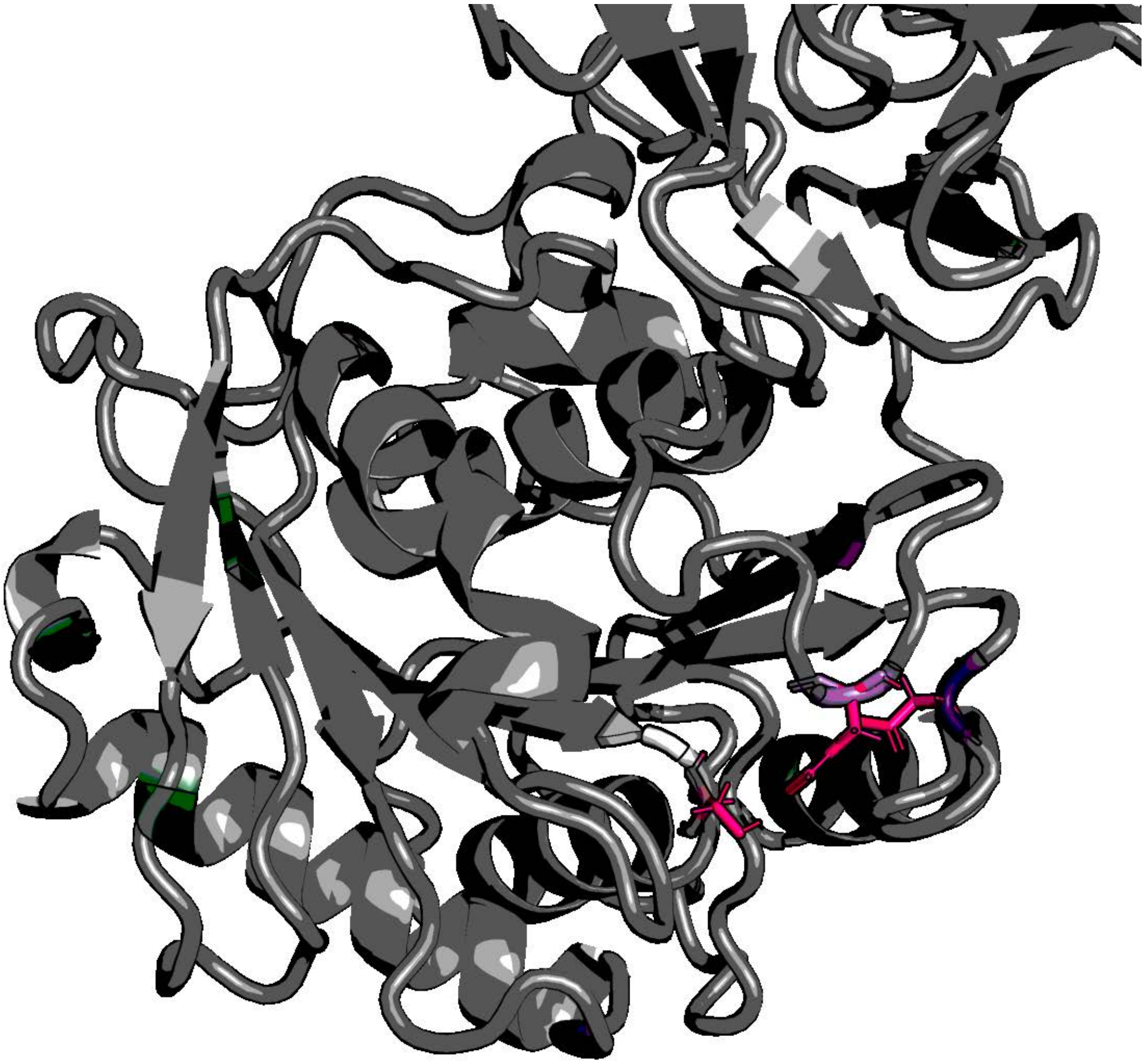

ESM130_highlights.png

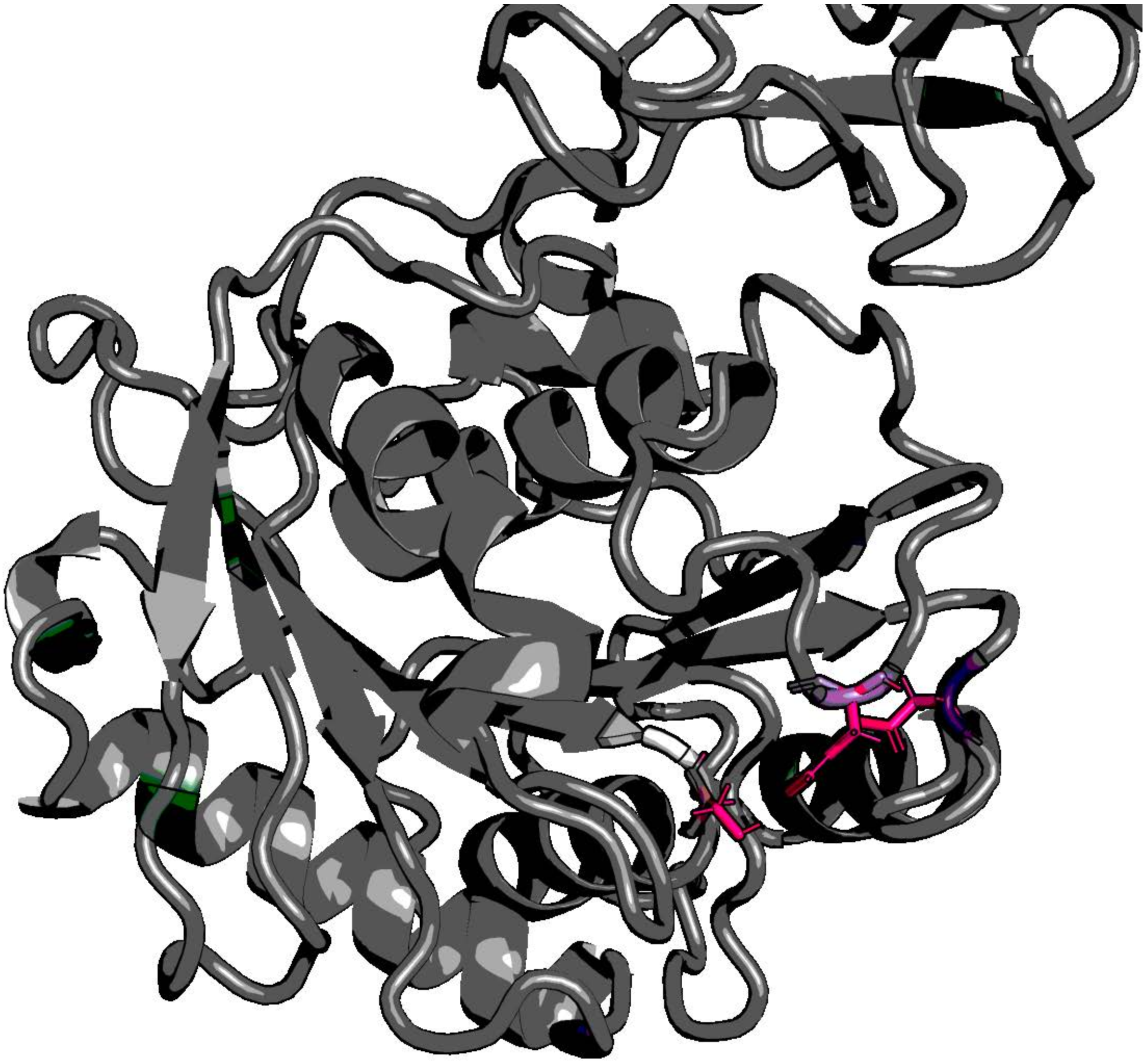

ESM132_highlights.png

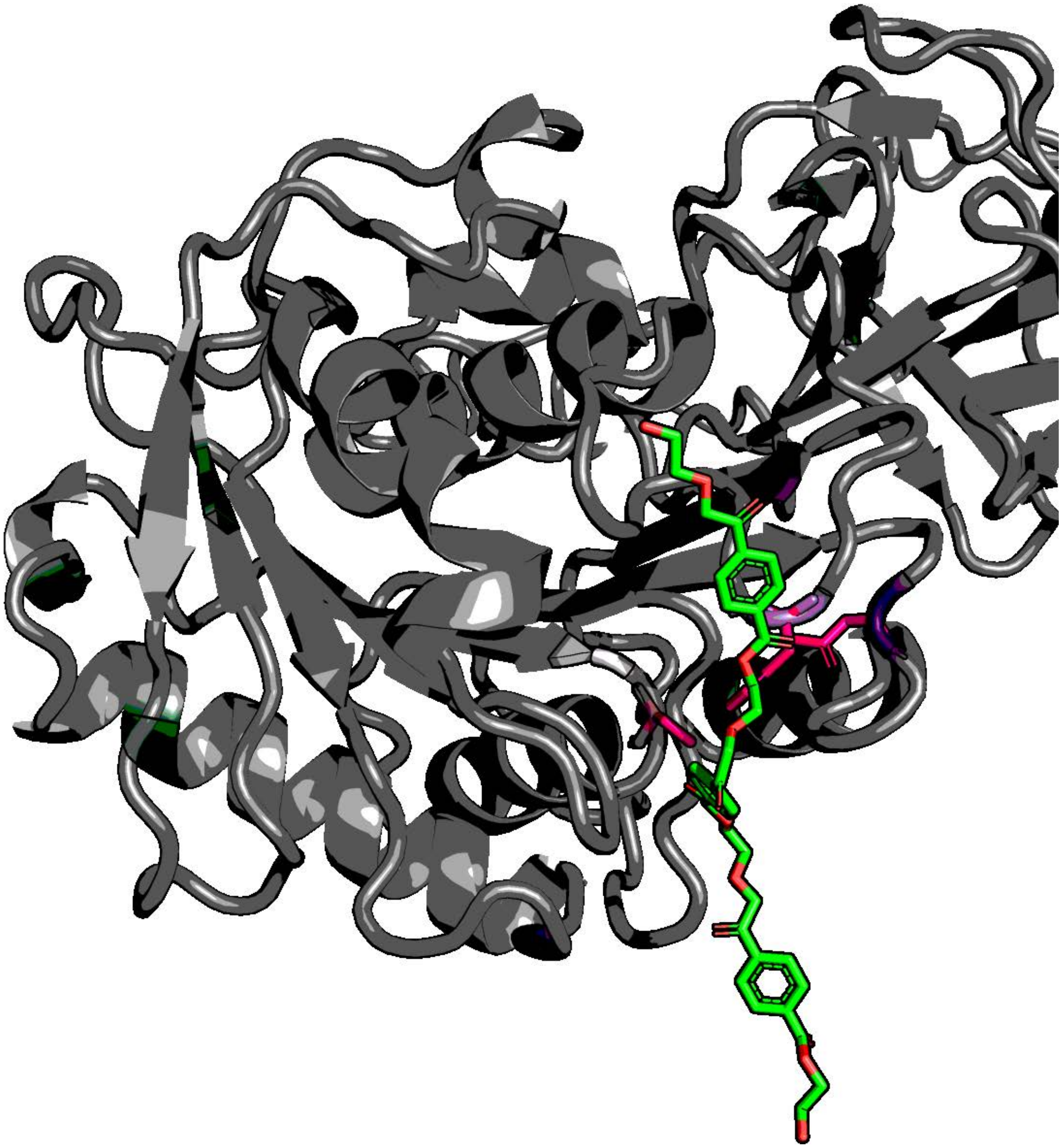

ESM135_highlights.png

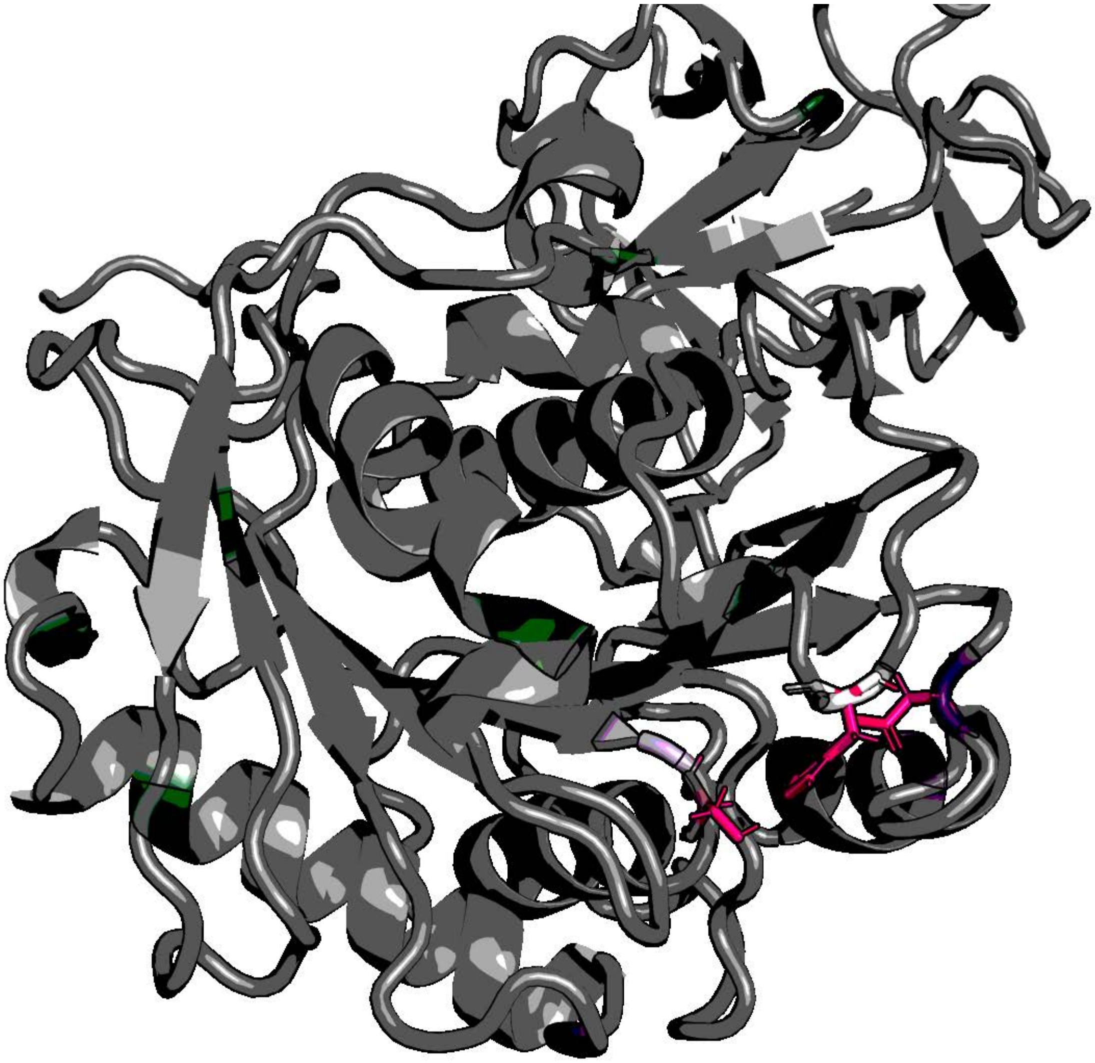

ESM136_highlights.png

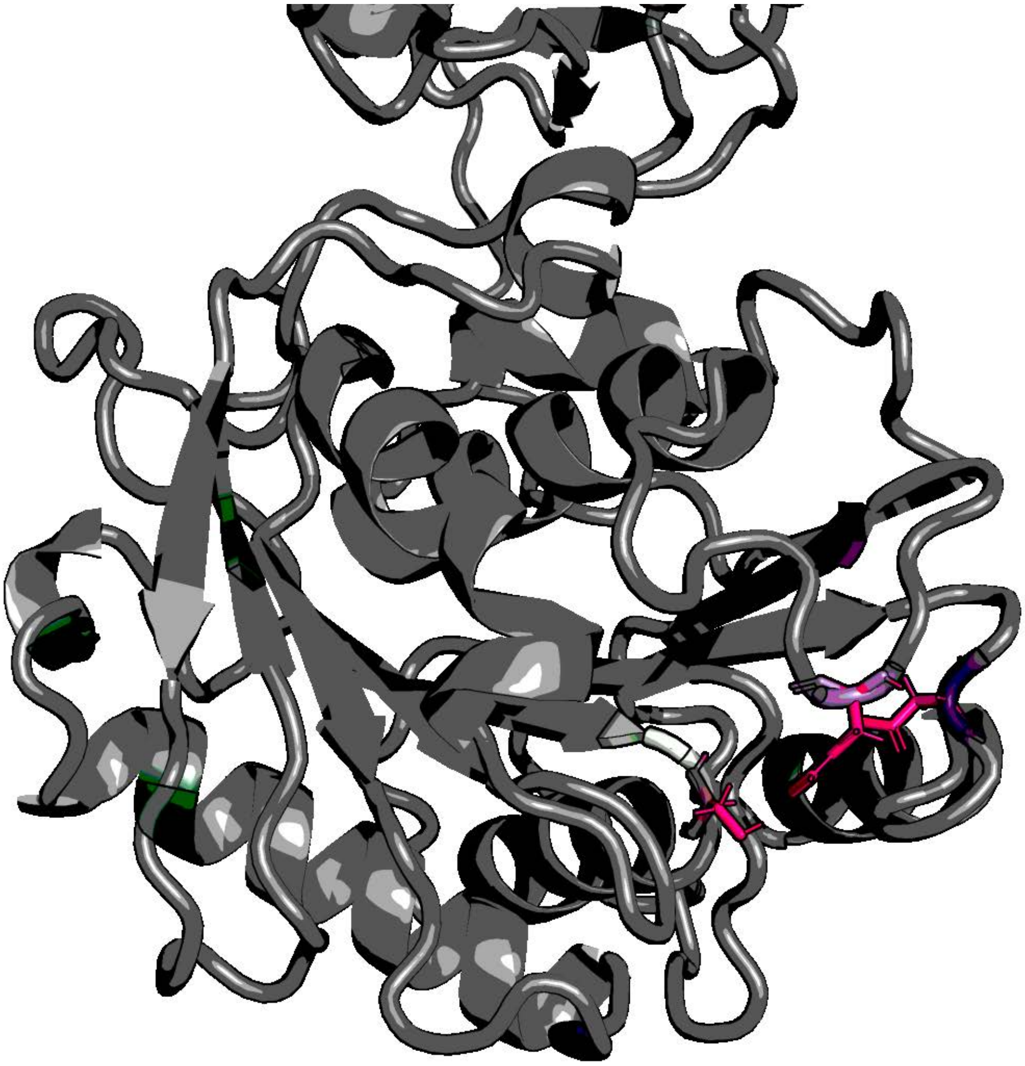

ESM137_highlights.png

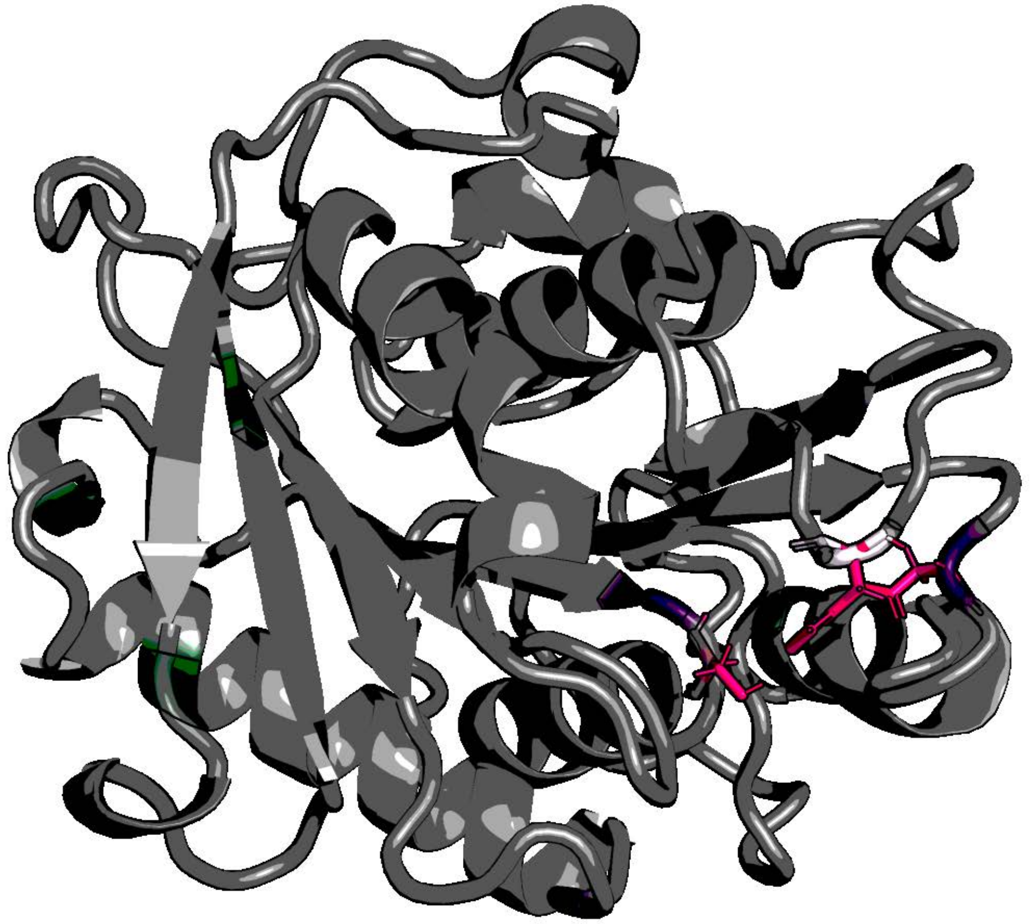

ESM140_highlights.png

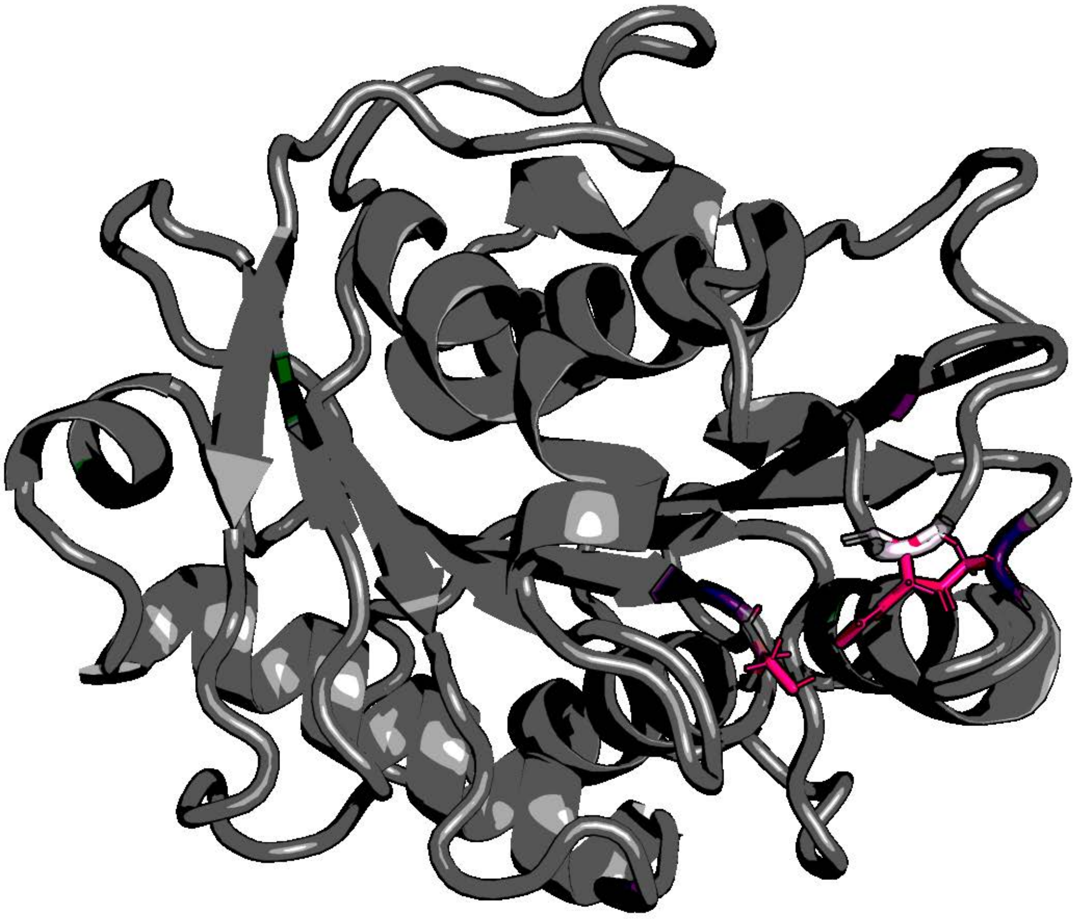

ESM144_highlights.png

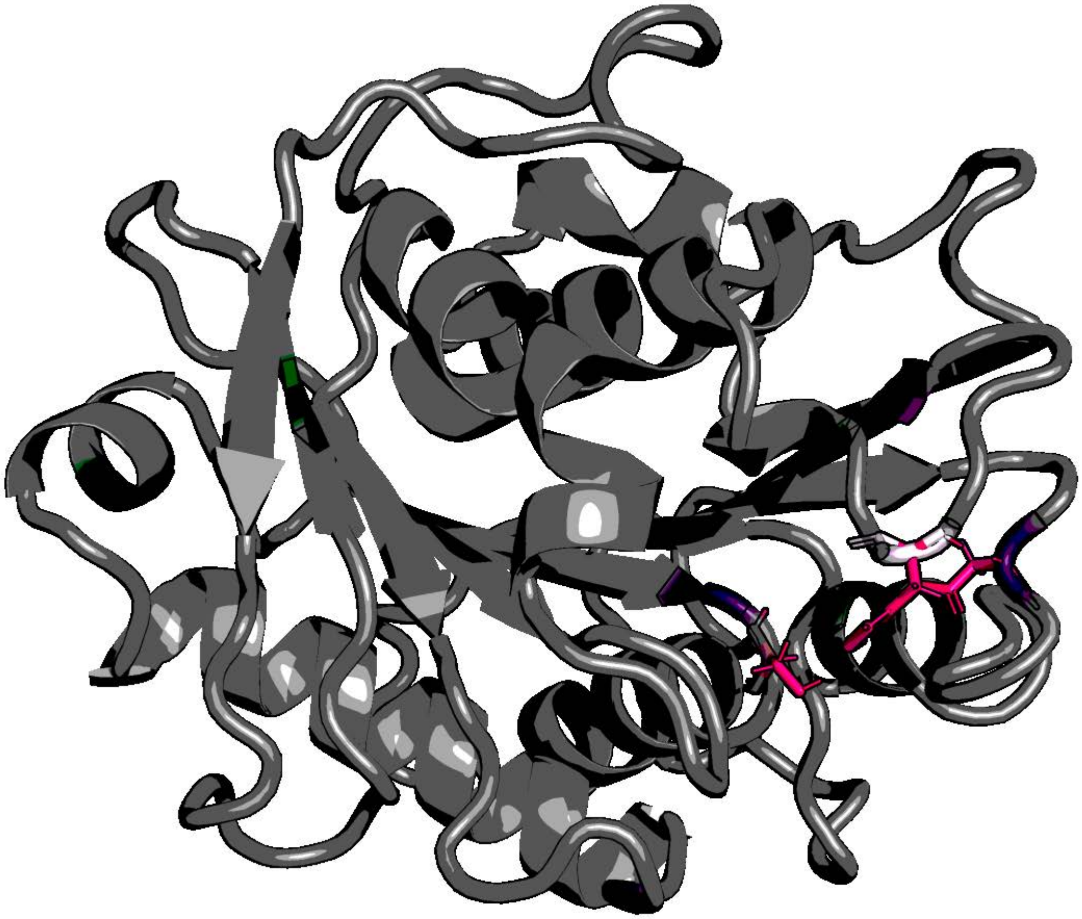

ESM147_highlights.png

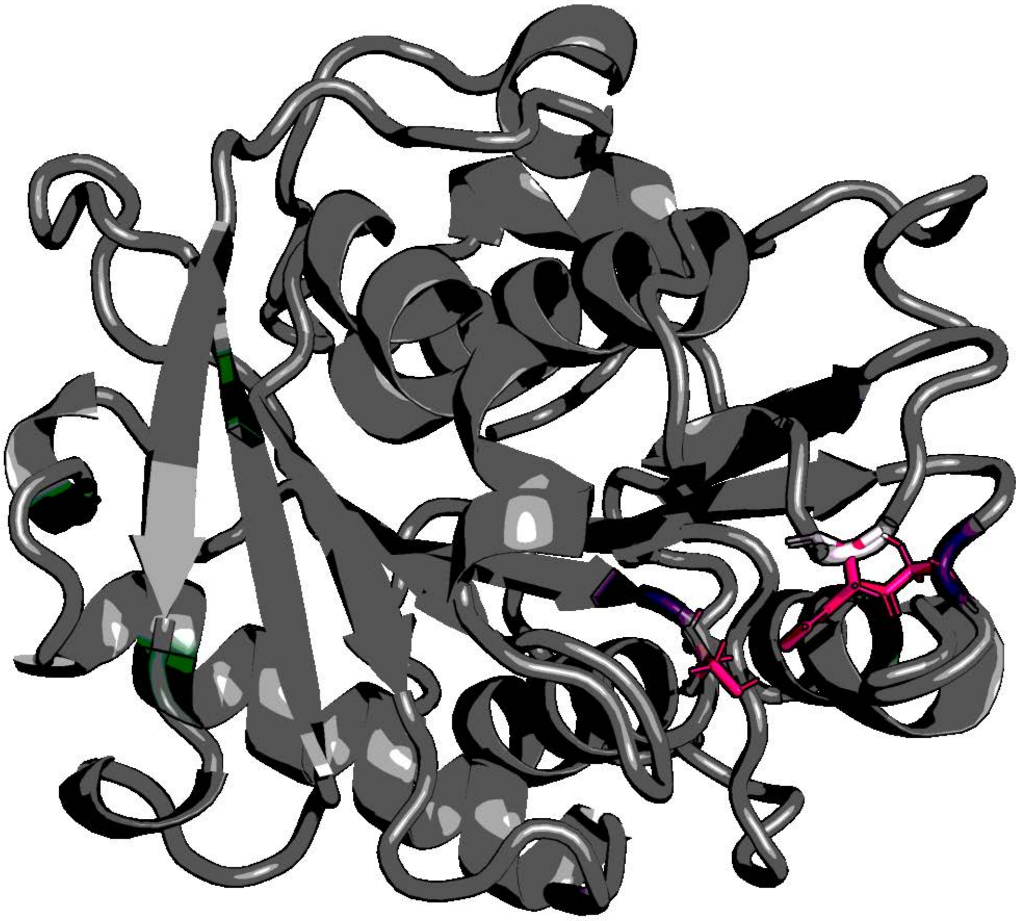

ESM156_highlights.png

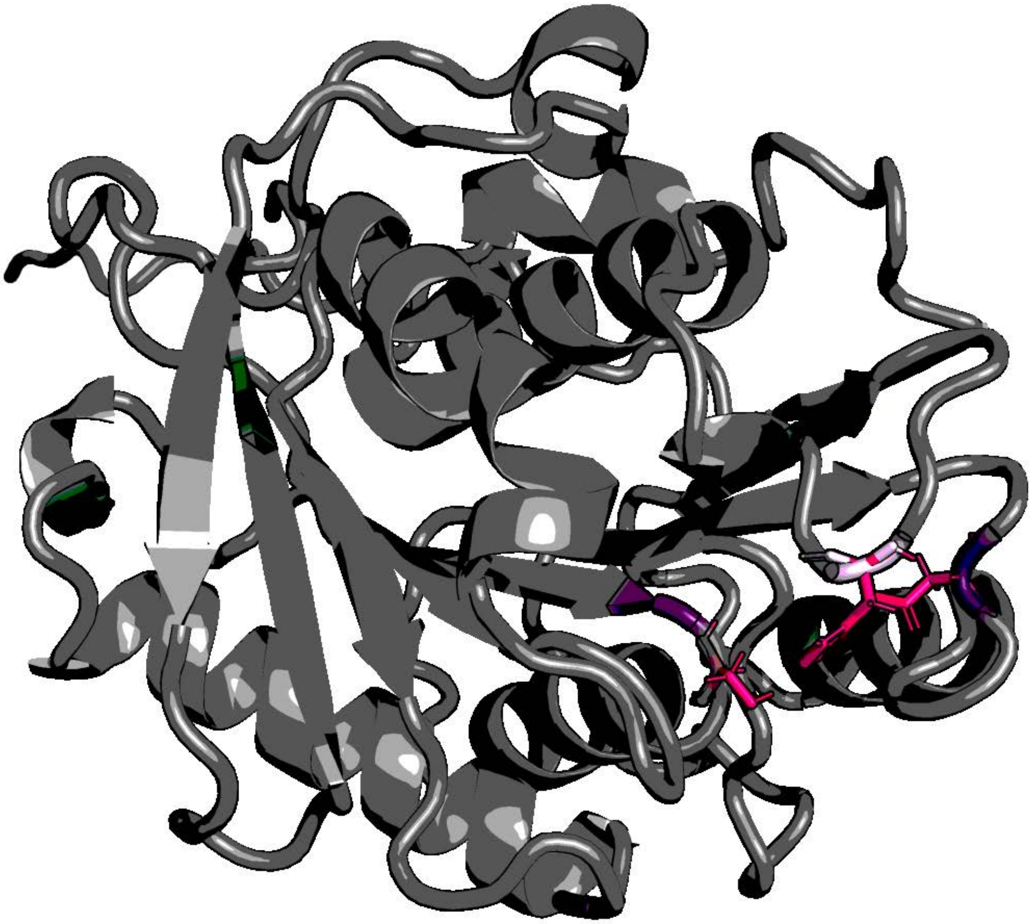

ESM157_highlights.png

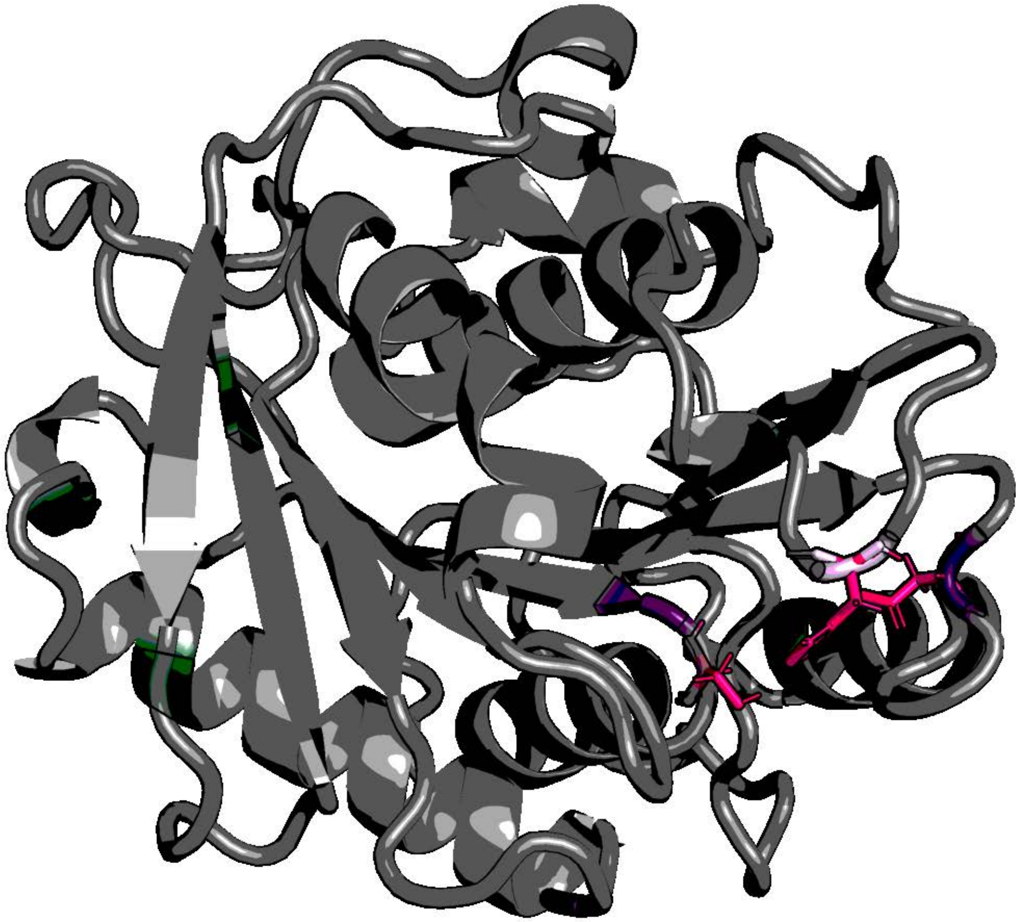

ESM159_highlights.png

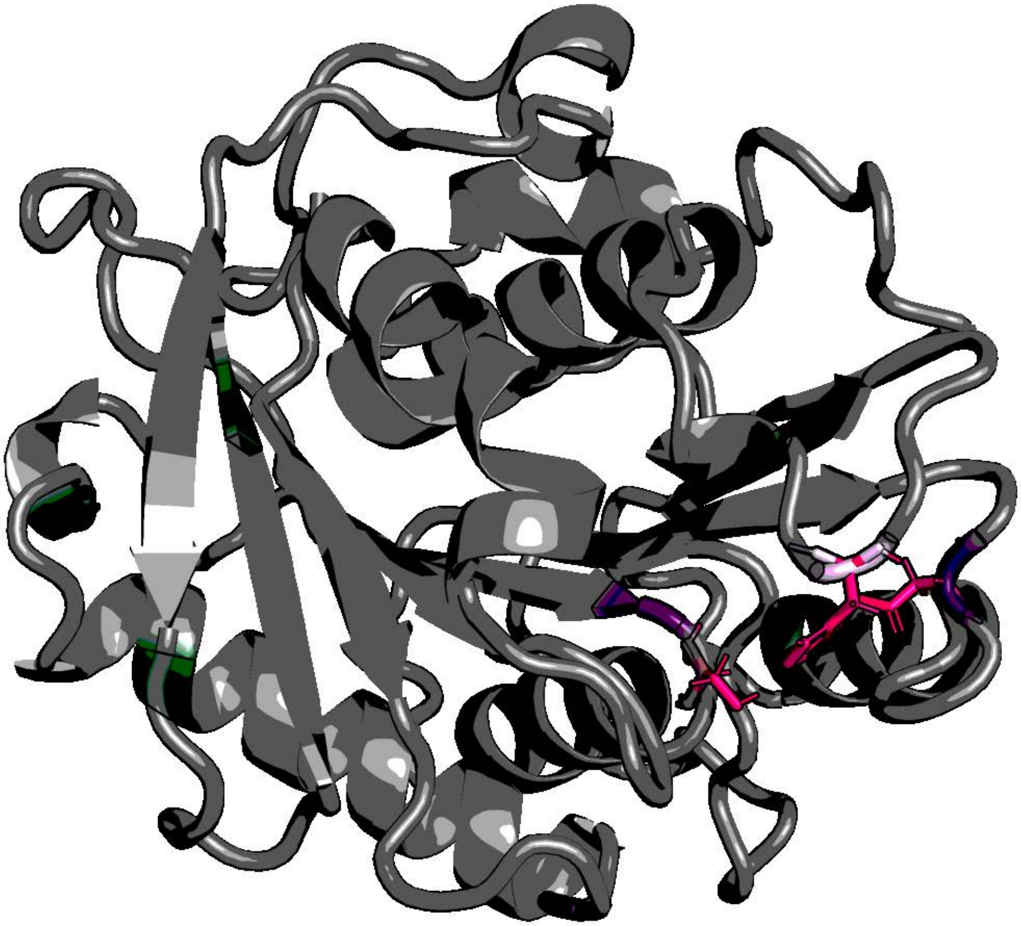

ESM160_highlights.png

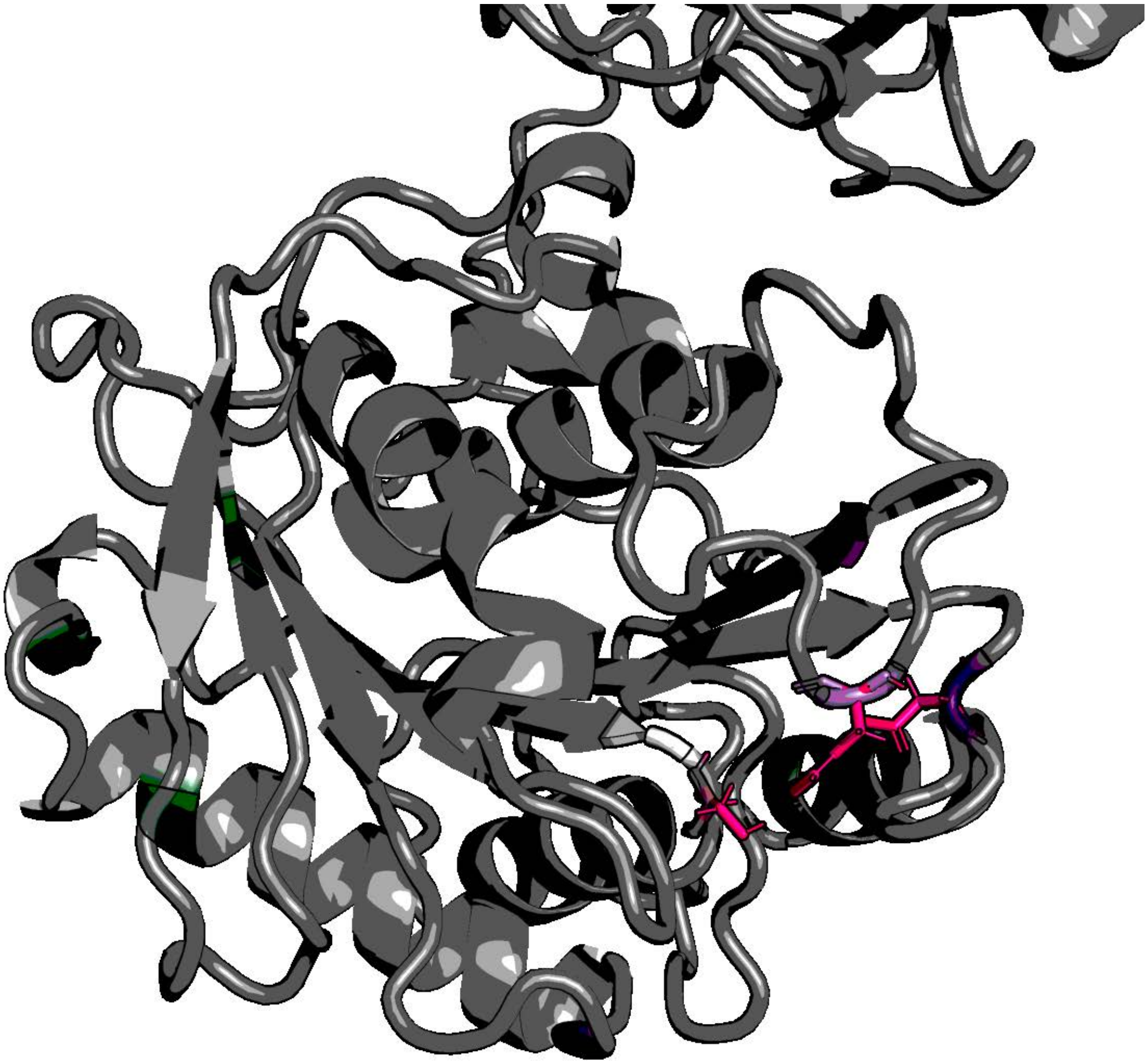

ESM161_highlights.png

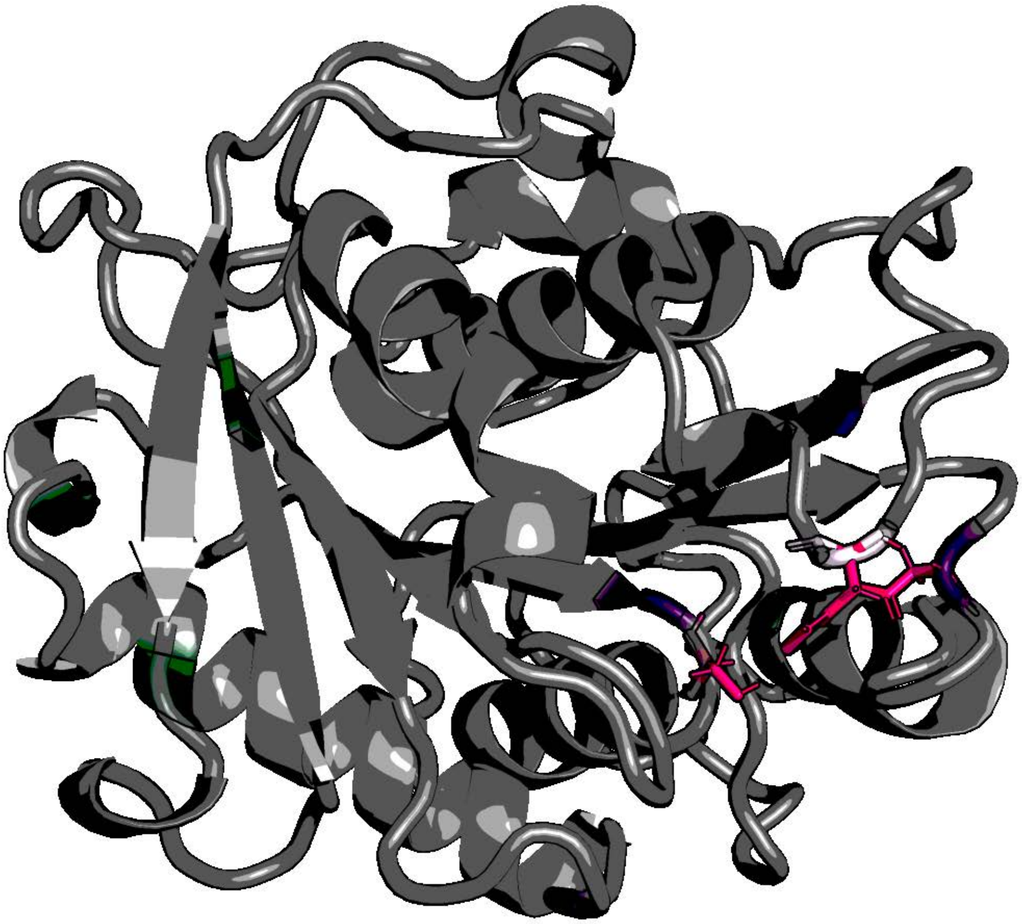

ESM167_highlights.png

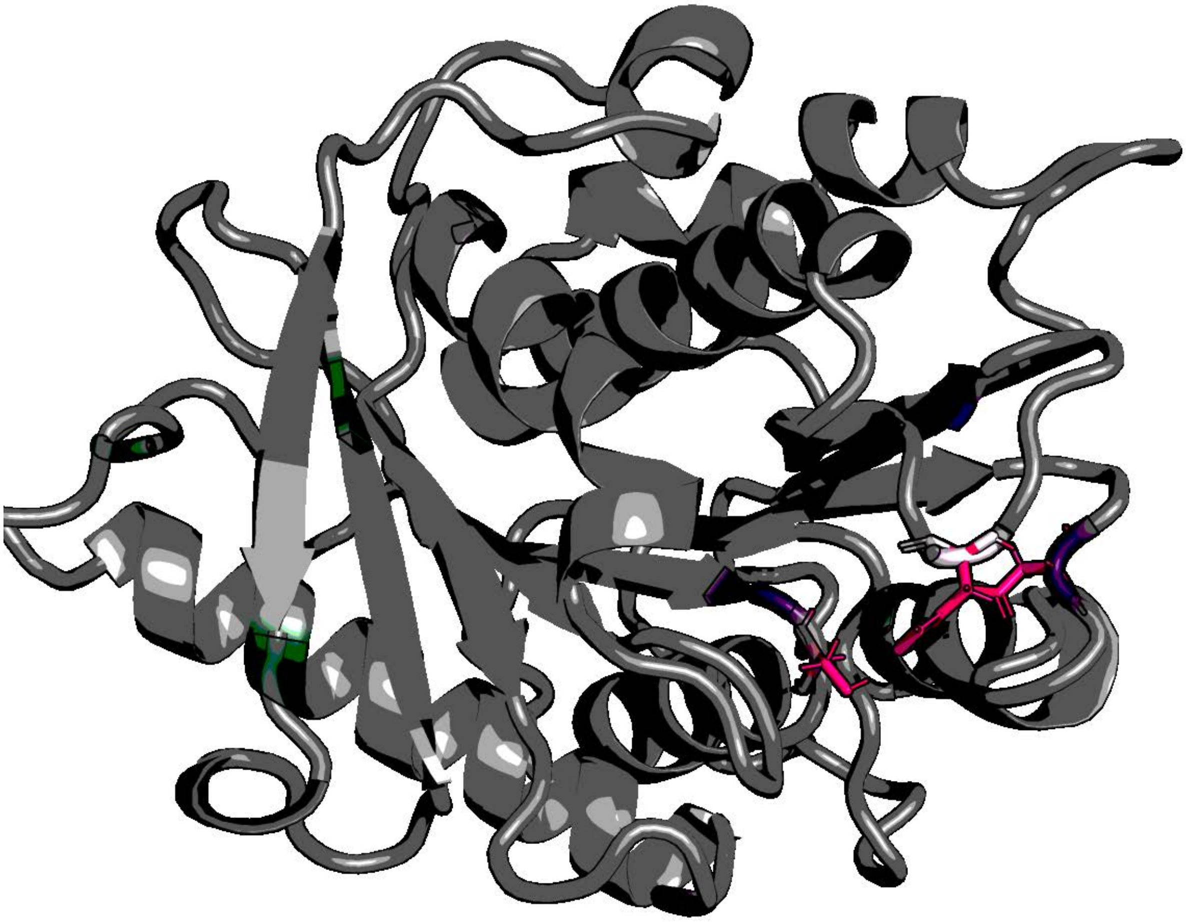

ESM169_highlights.png

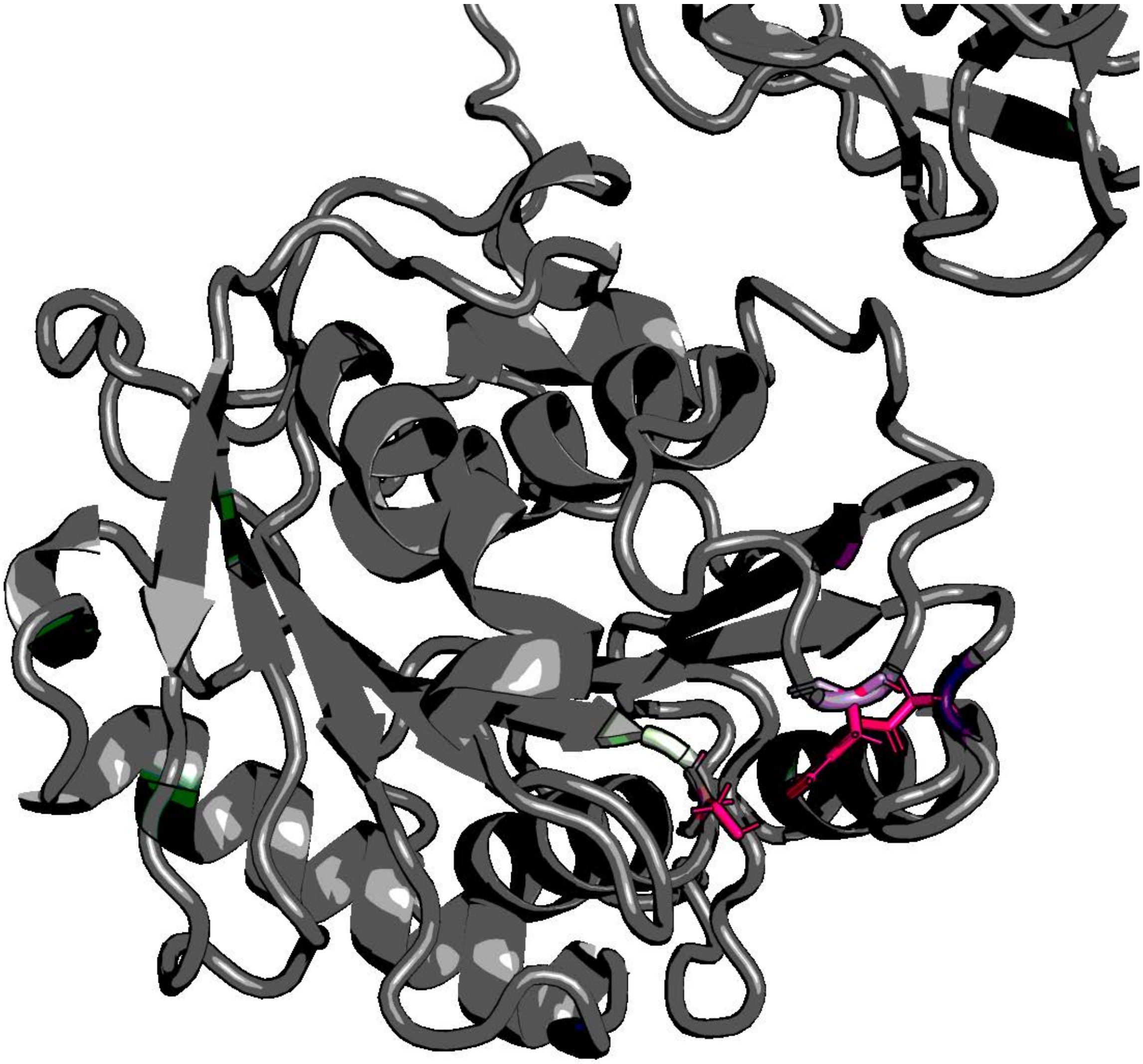

ESM173_highlights.png

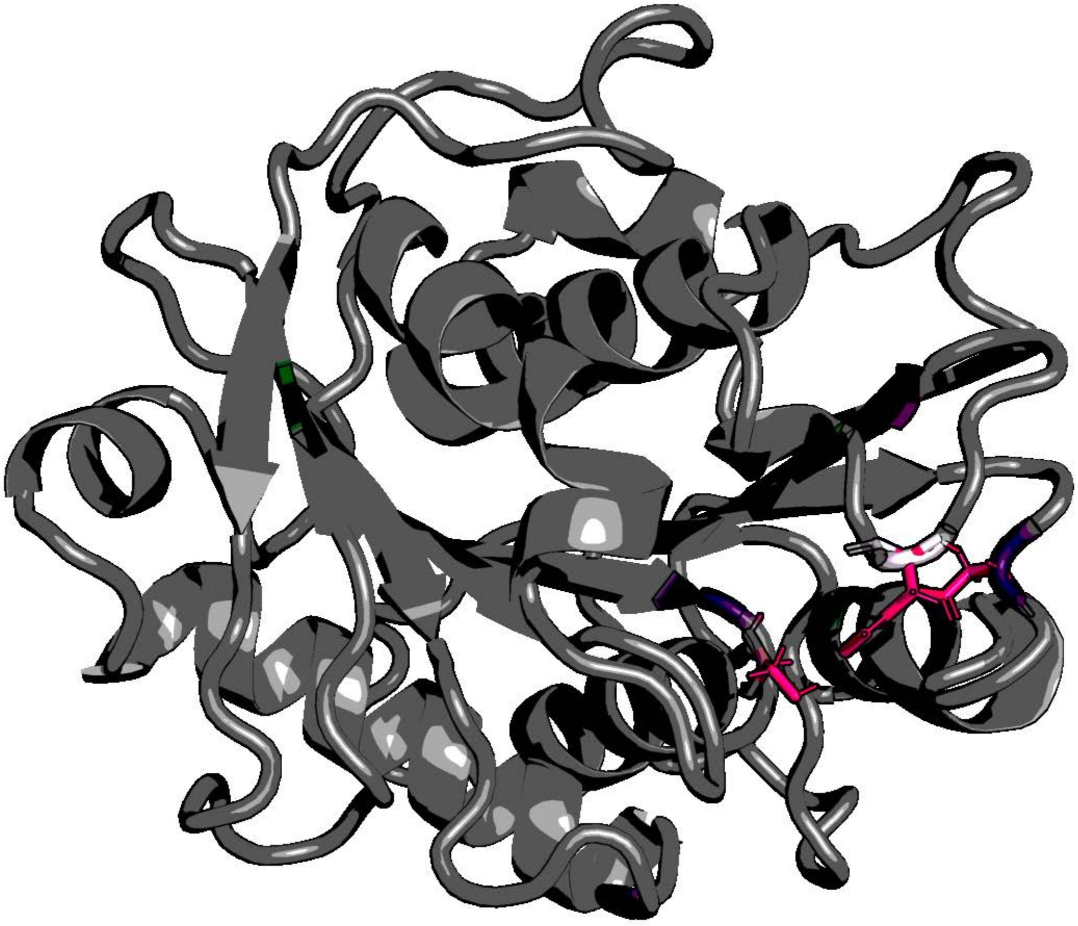

ESM177_highlights.png

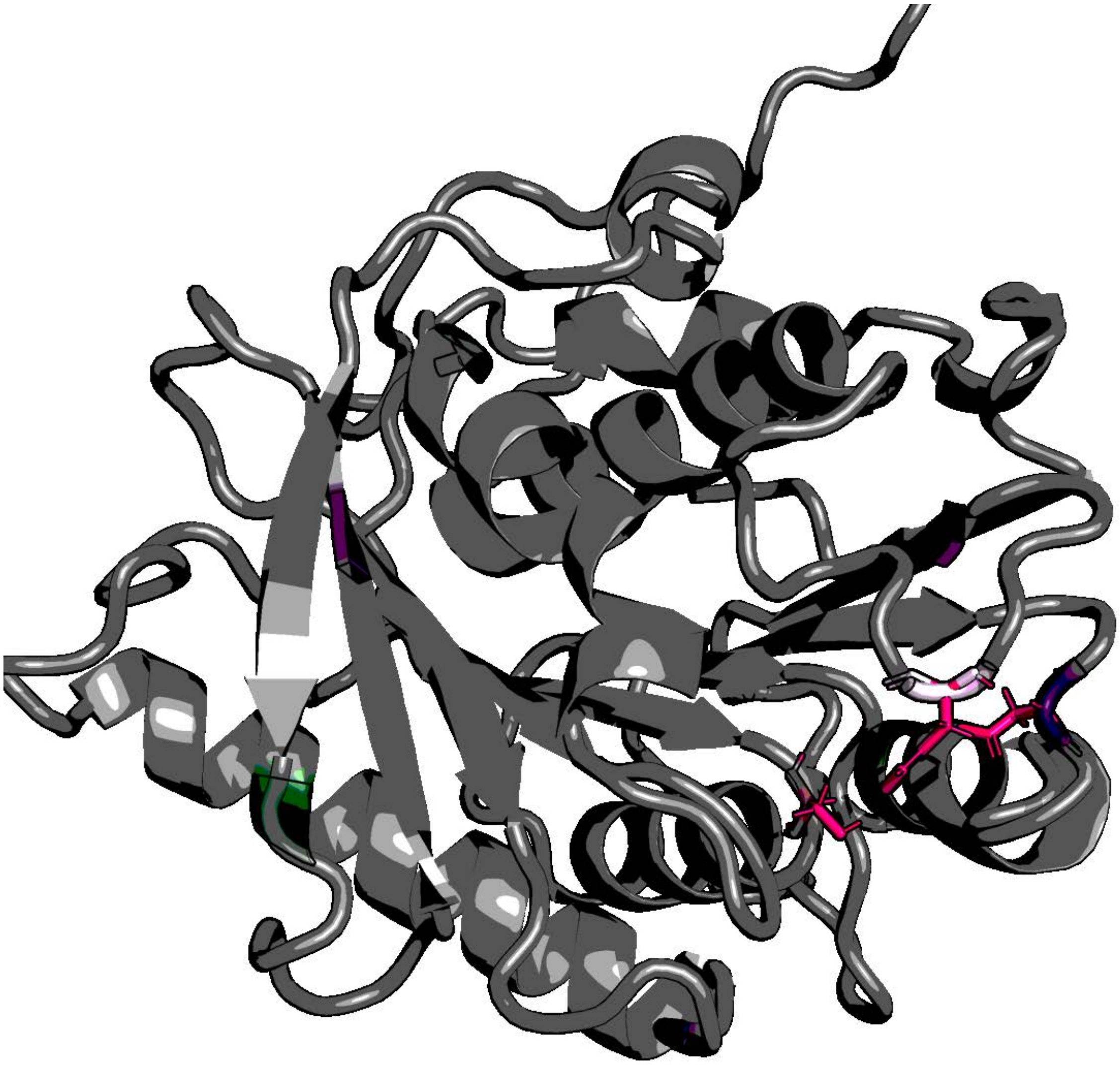

ESM186_highlights.png

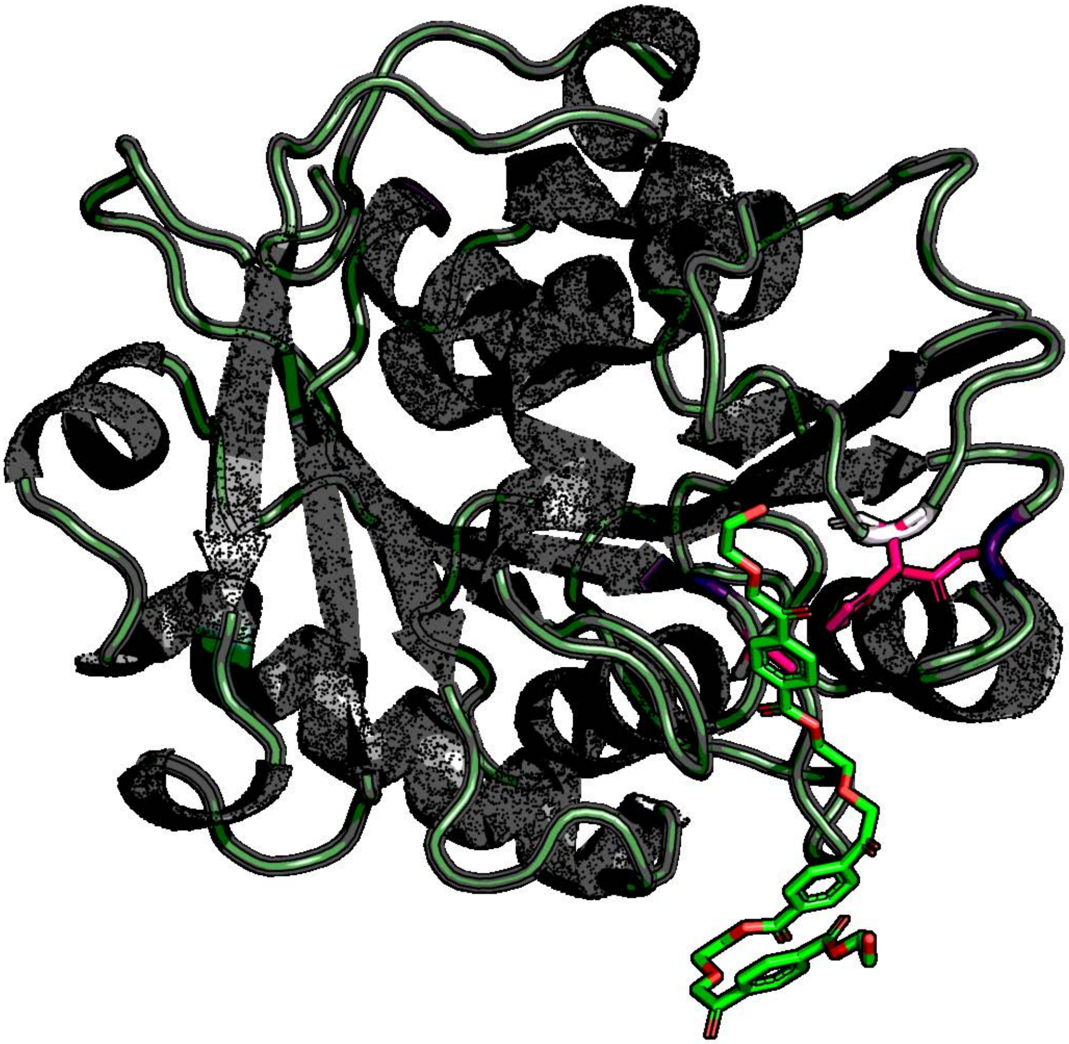

LCC-ICCG_highlights.png

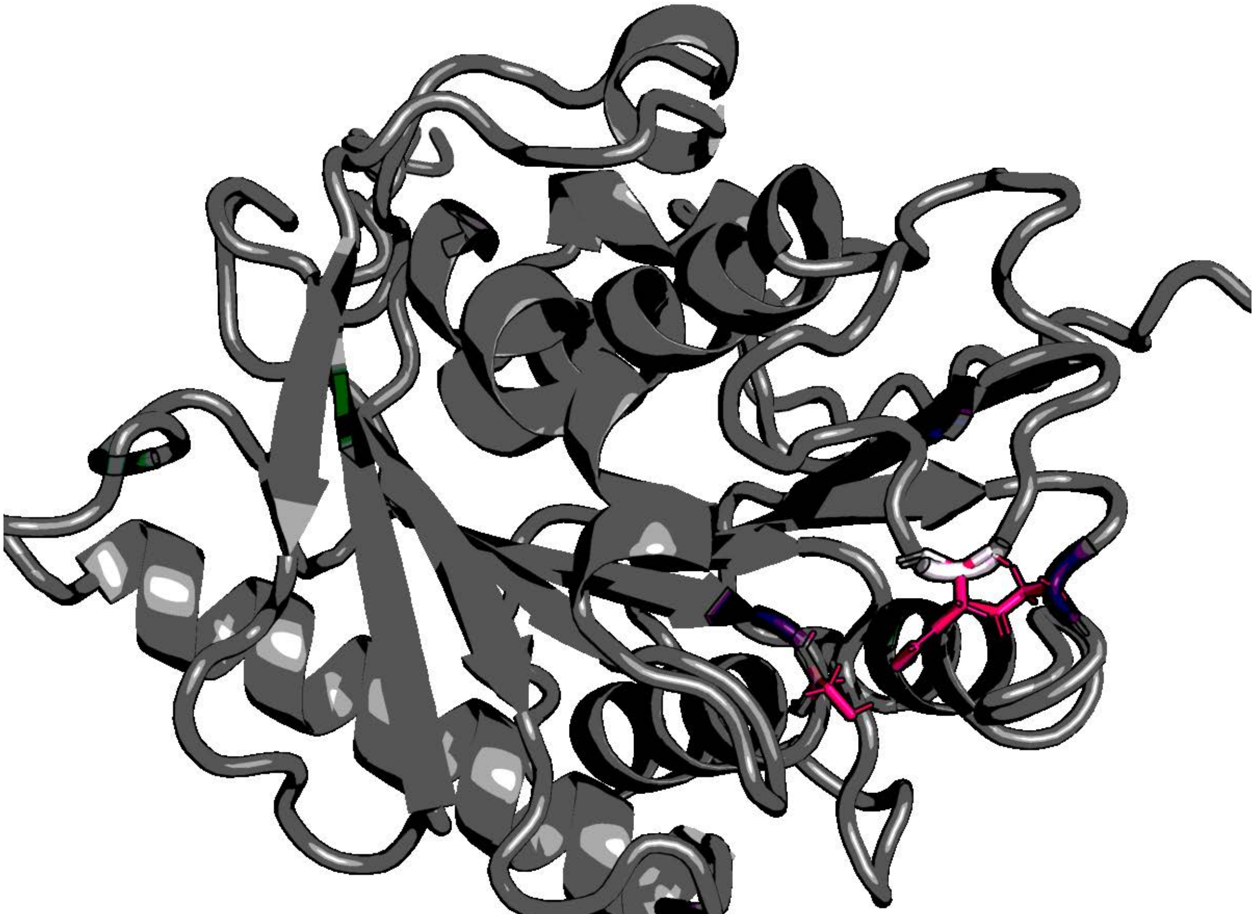

TEP004_highlights.png

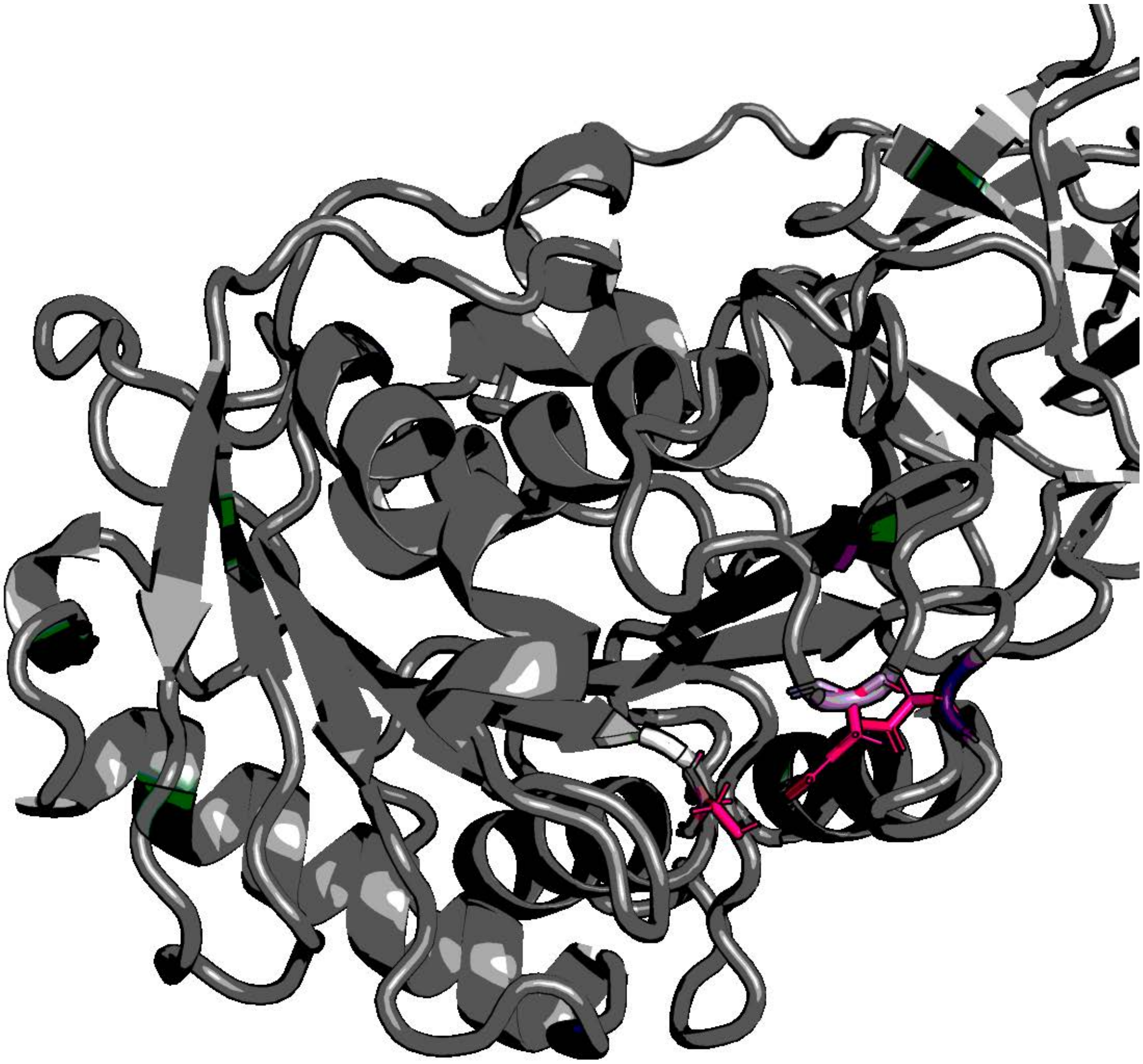

TEP010_highlights.png

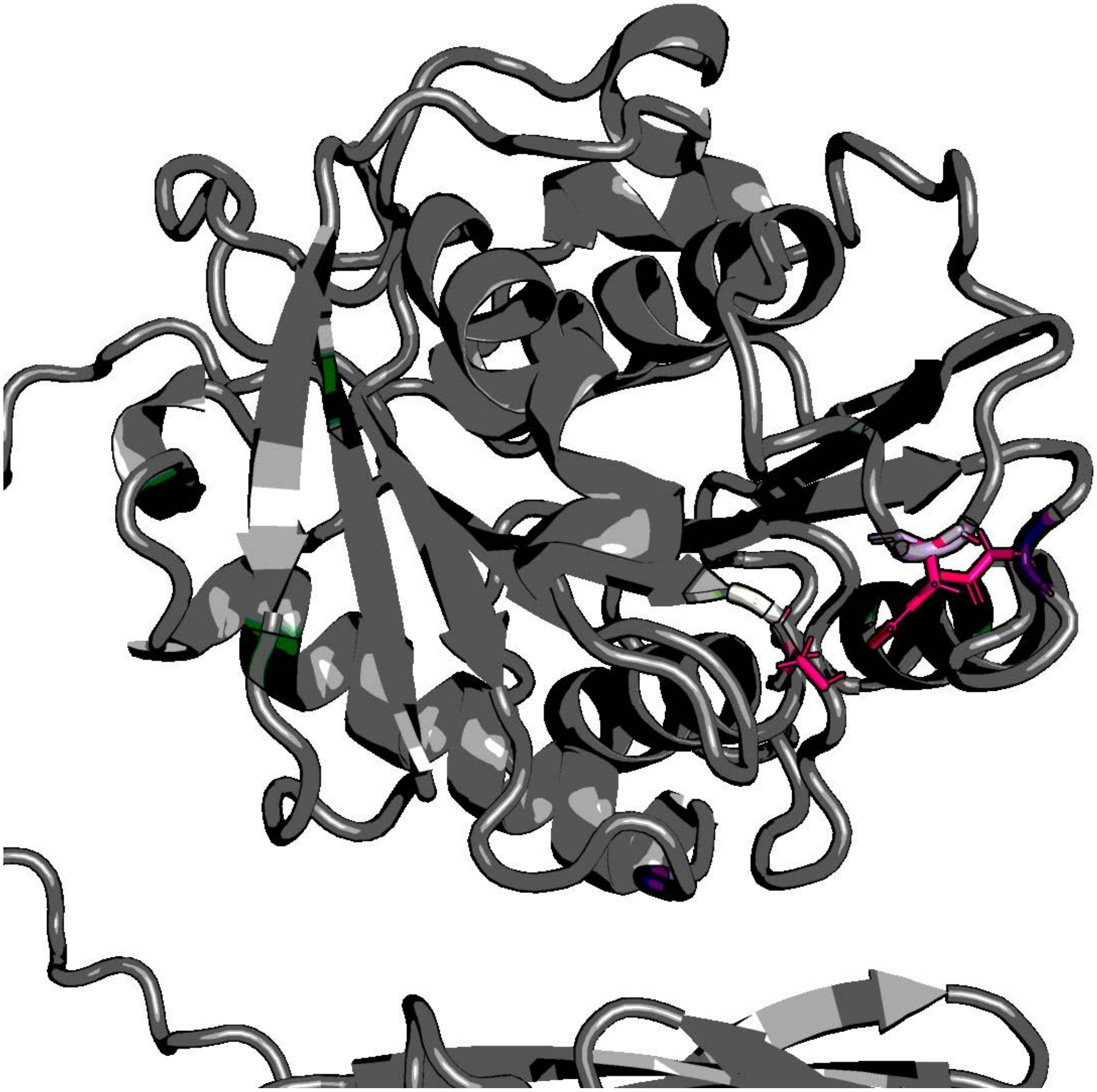

TEP013_highlights.png

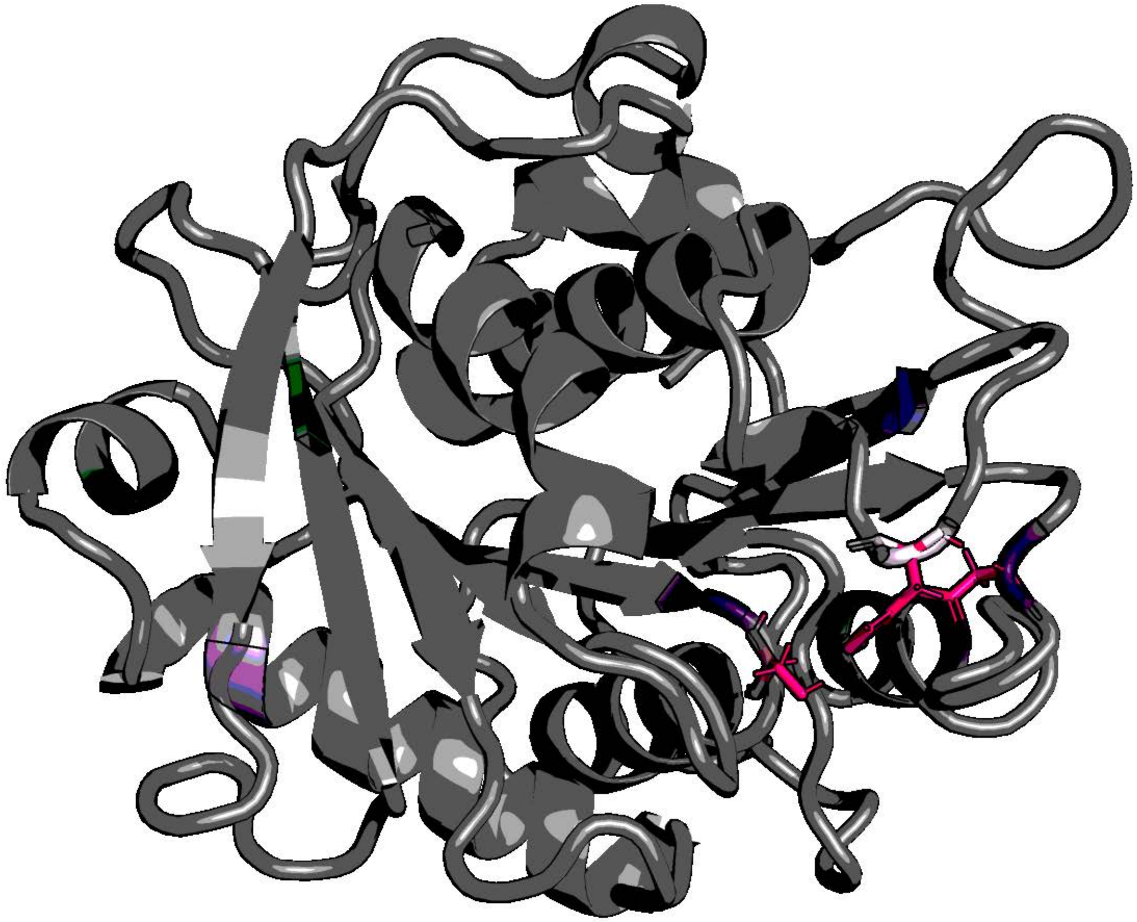

TEP014_highlights.png

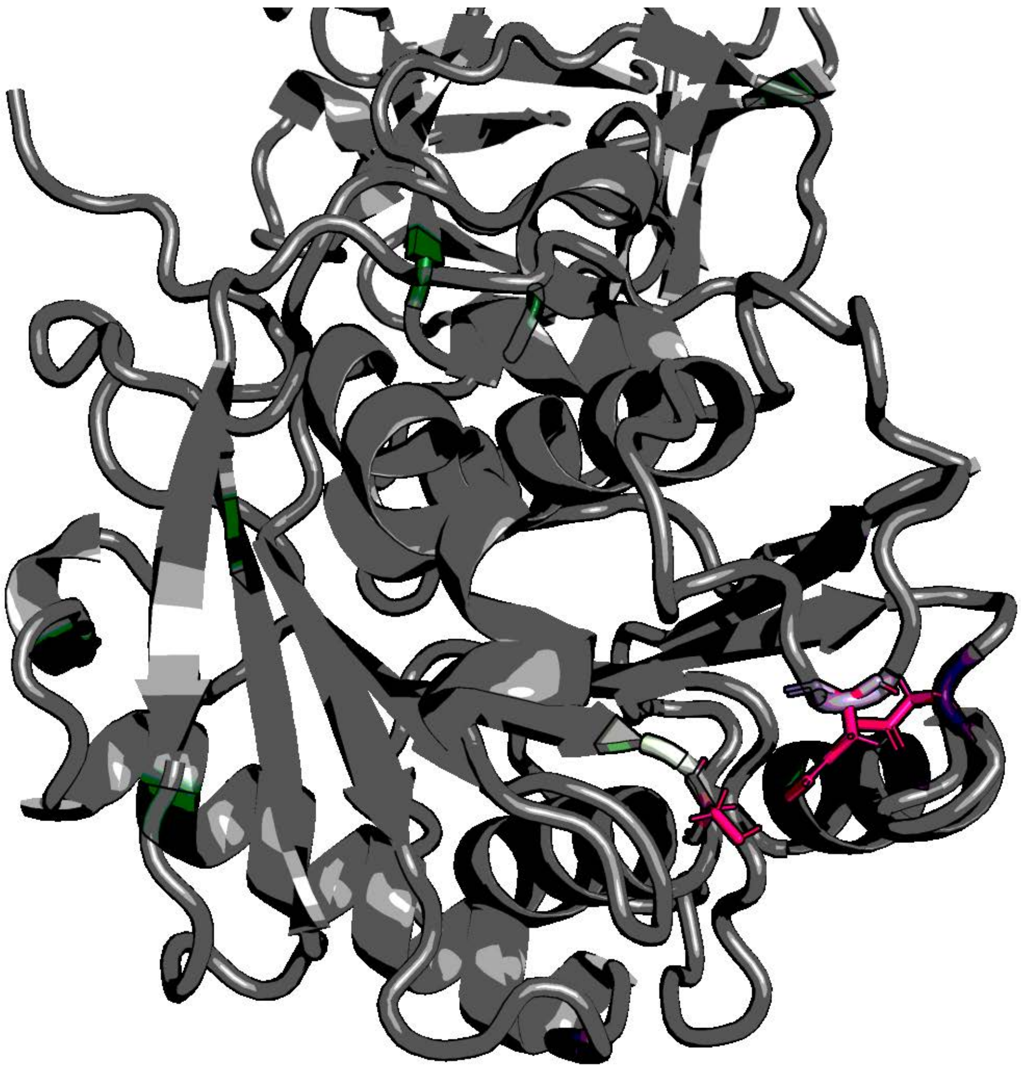

TEP024_highlights.png

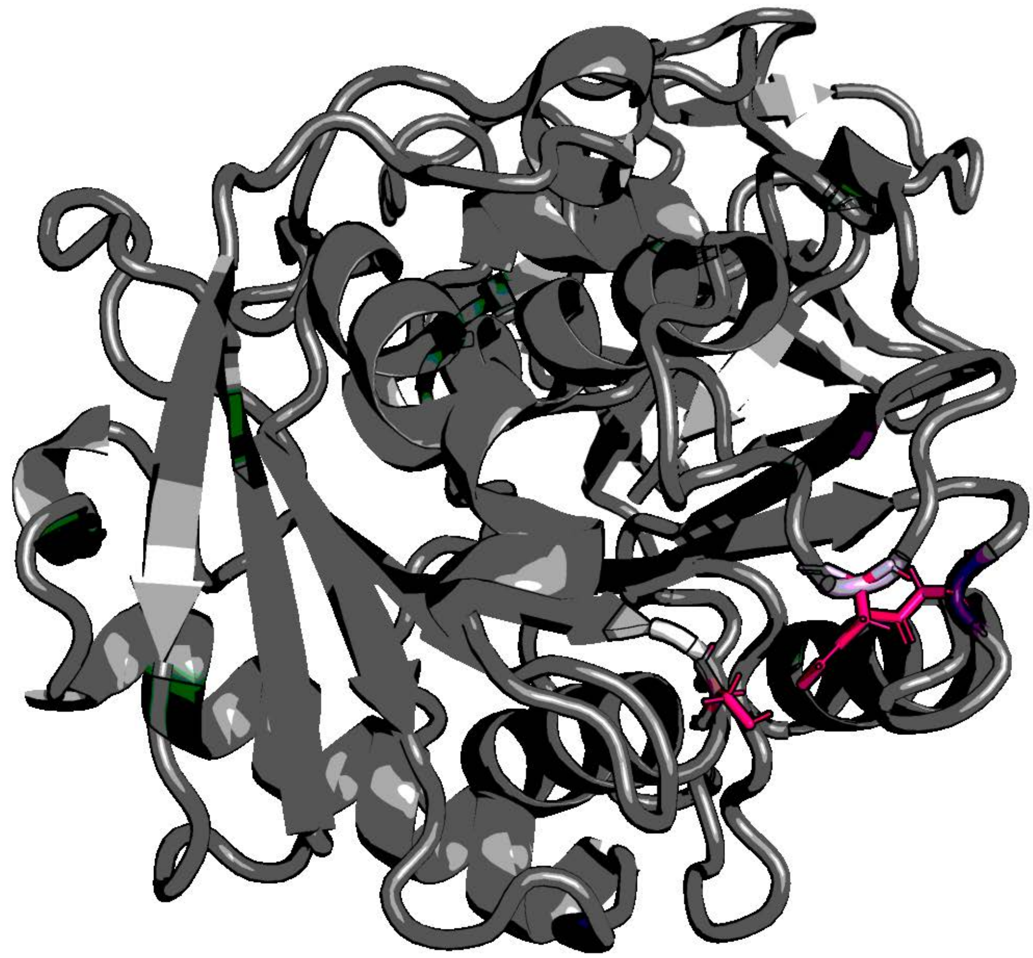

TEP029_highlights.png

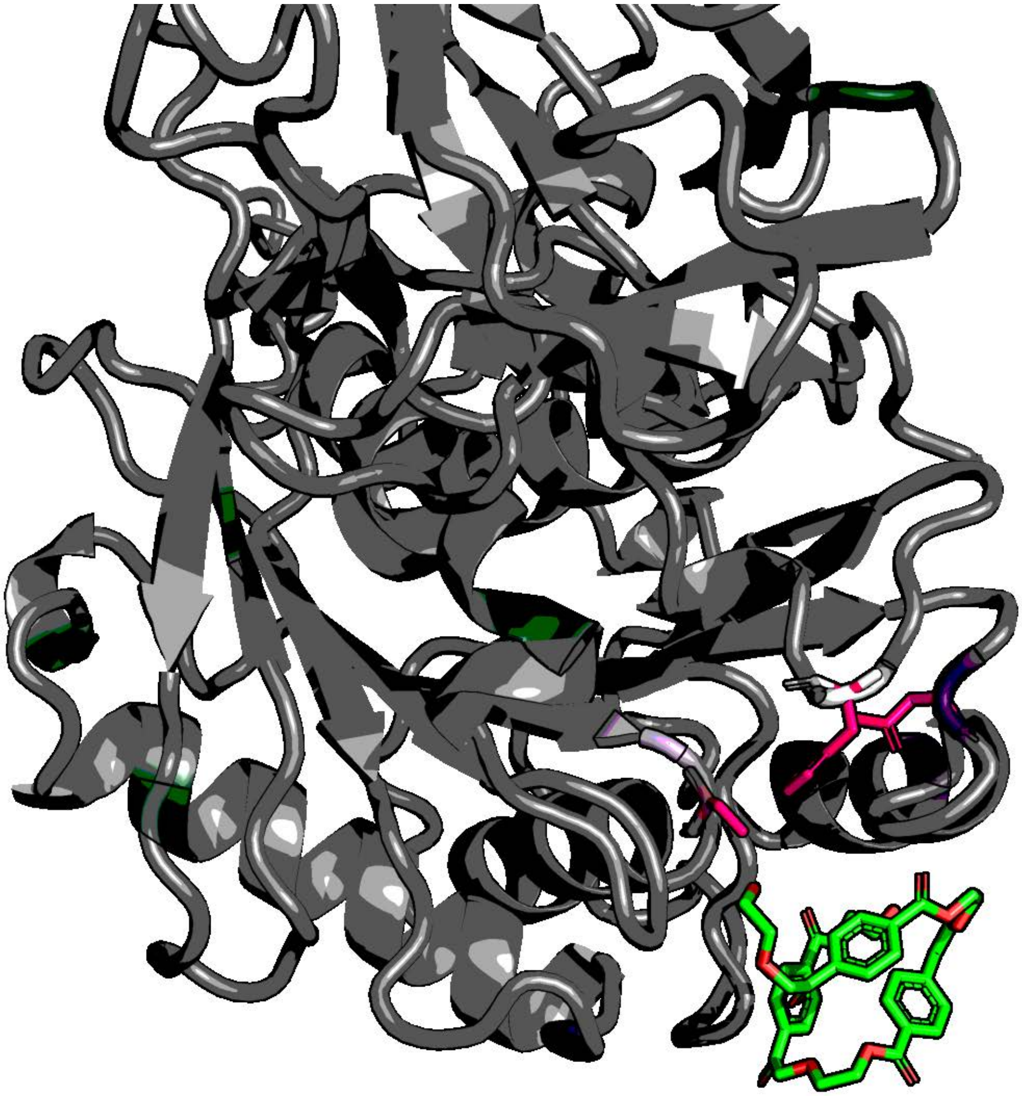

TEP054_highlights.png

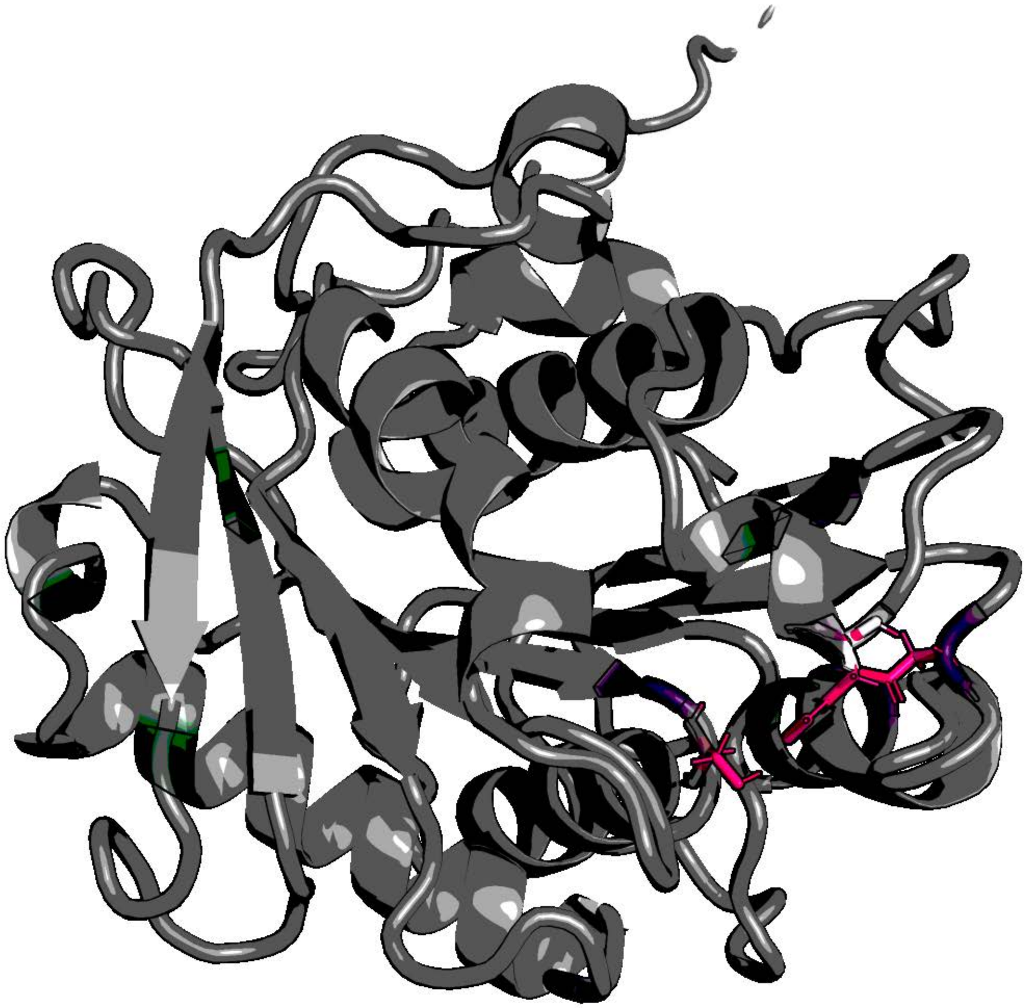

TEP067_highlights.png

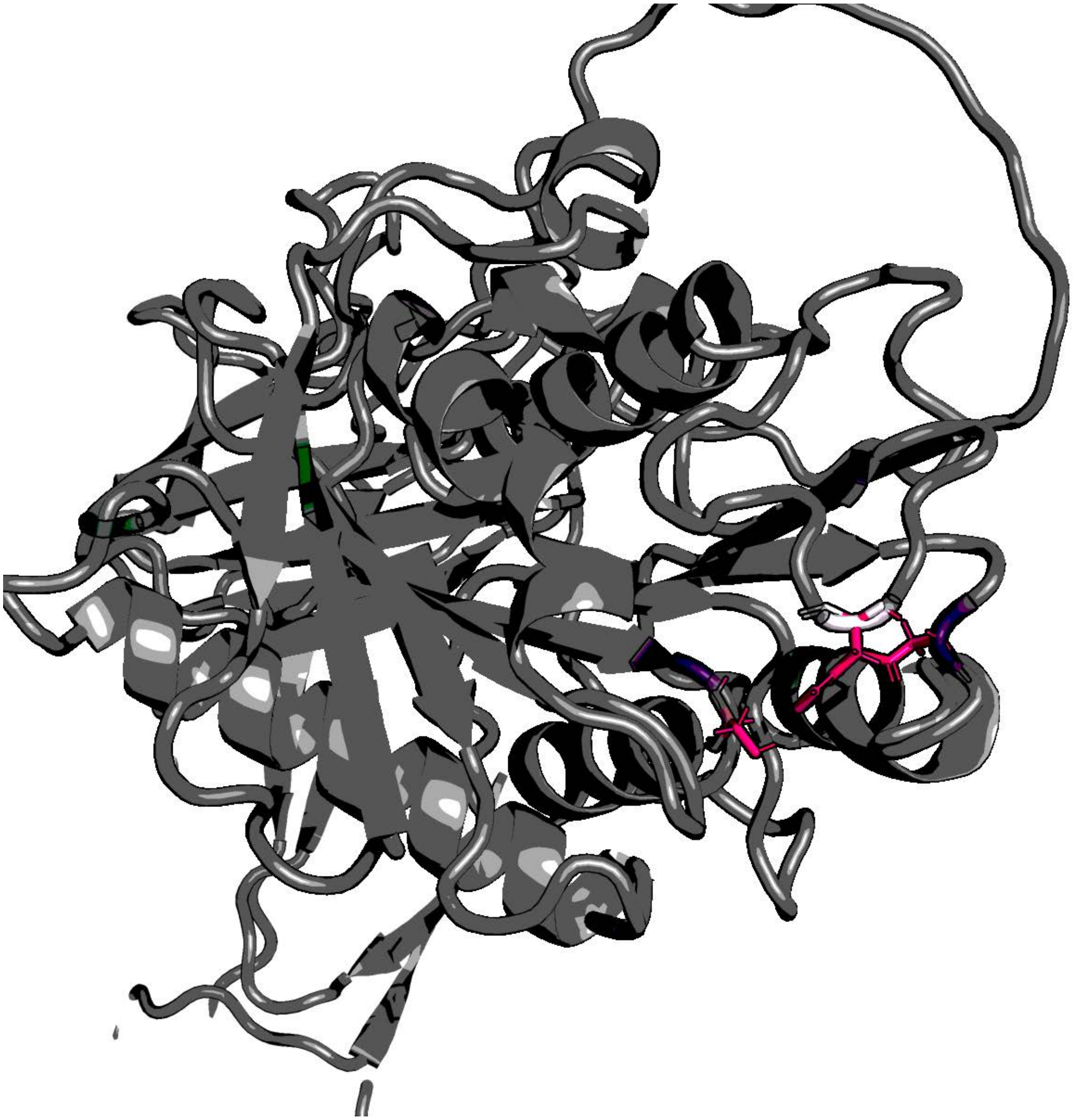

TEP080_highlights.png

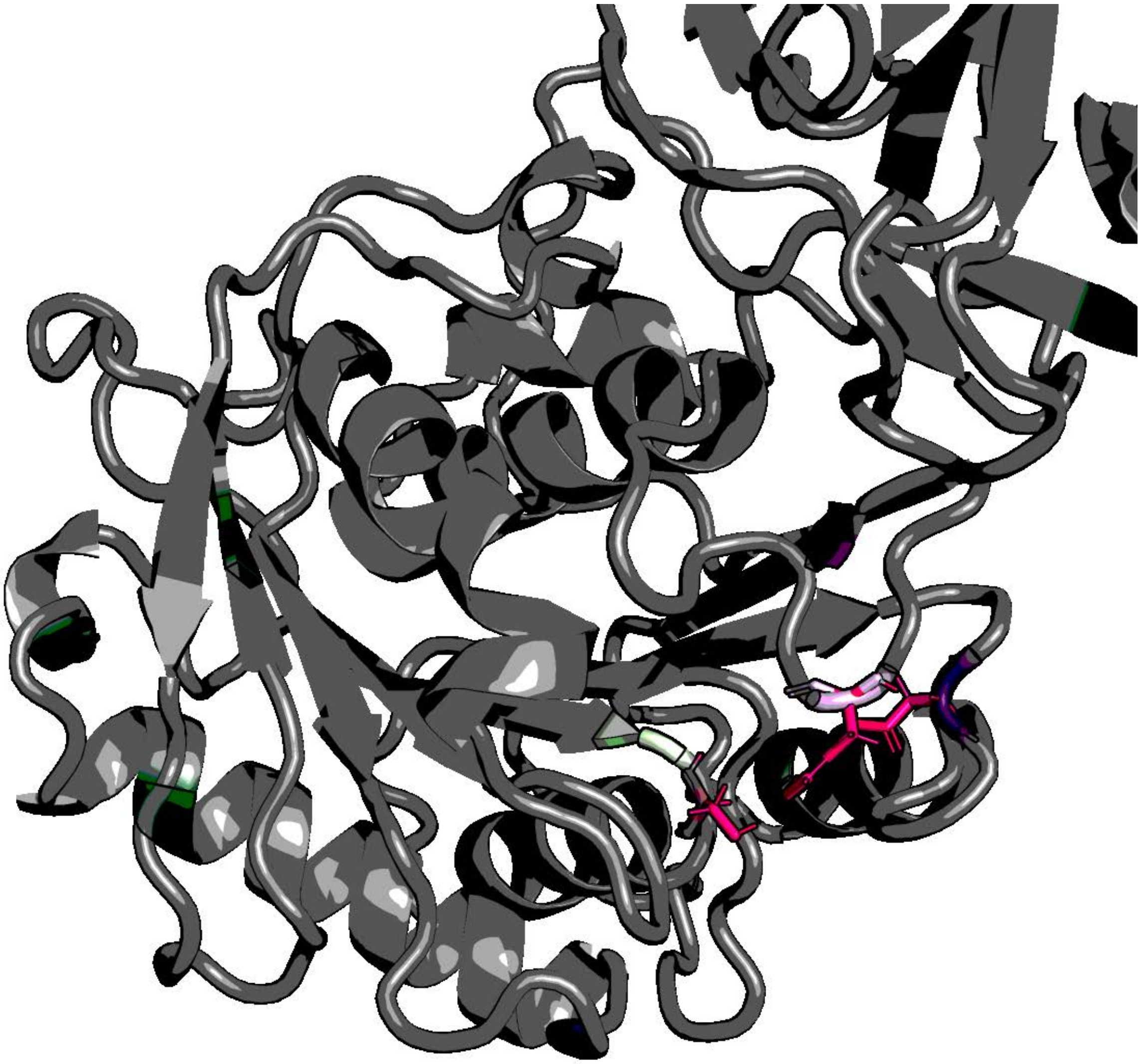

TEP082_highlights.png

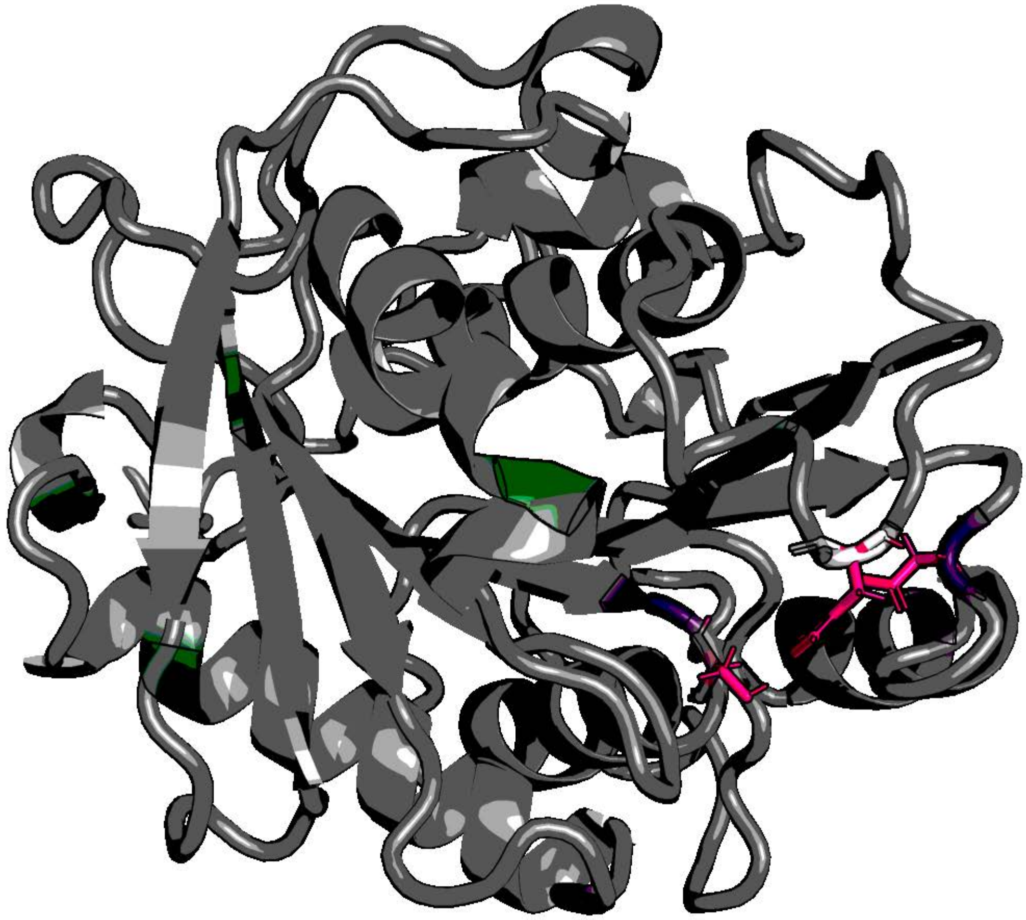

TEP083_highlights.png

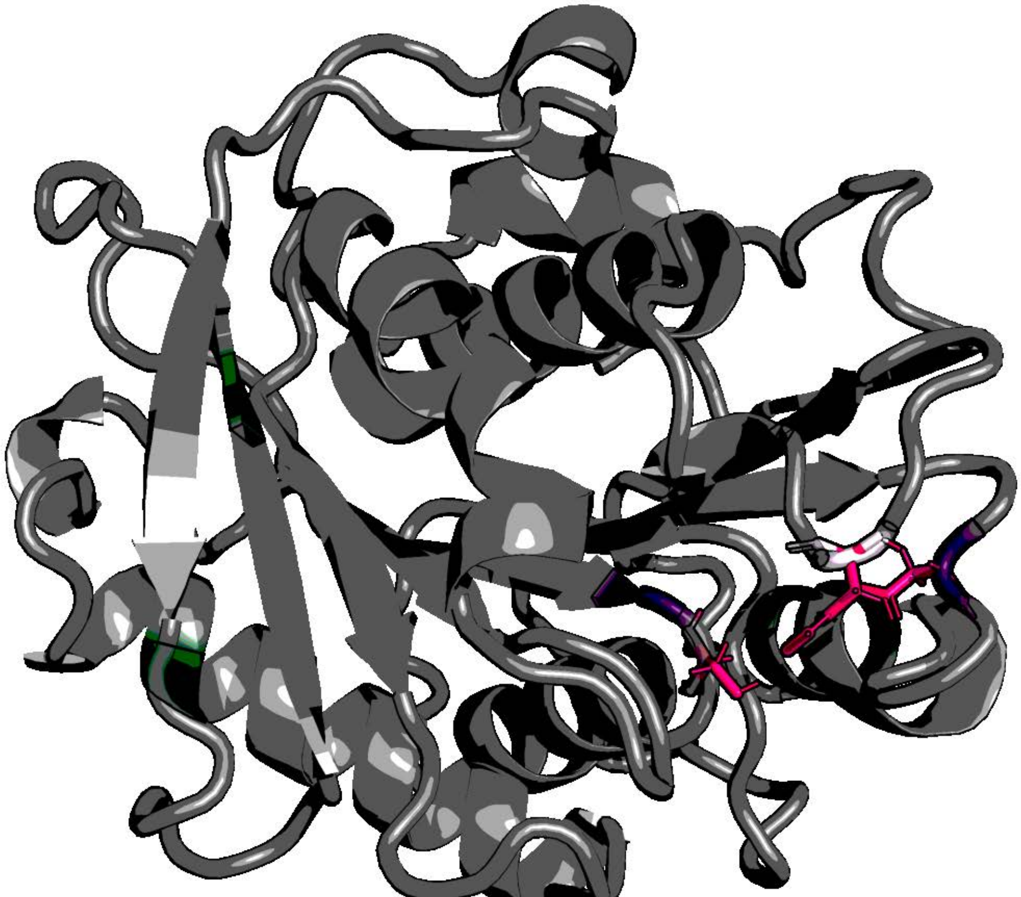

TEP091_highlights.png

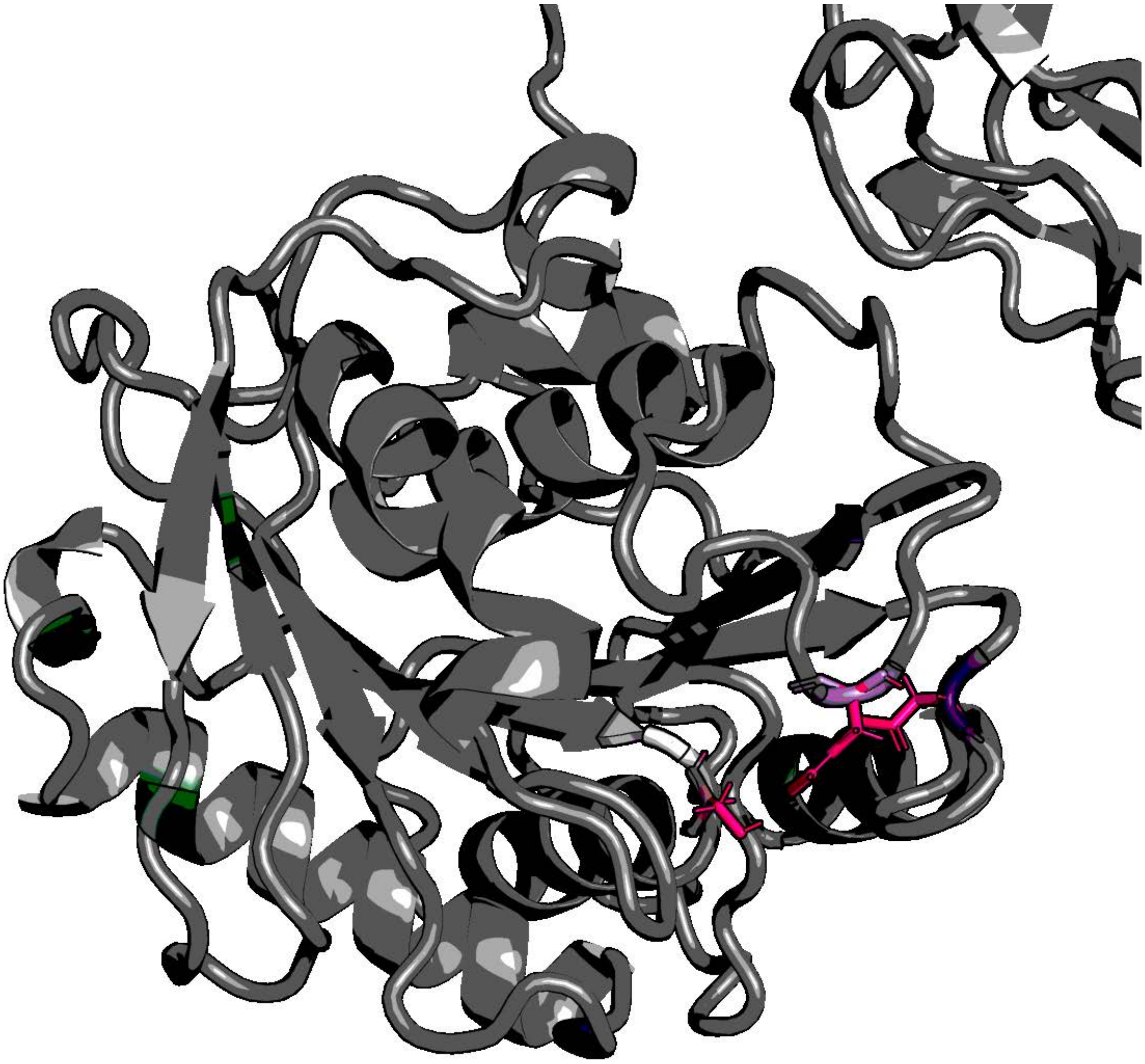

TEP092_highlights.png

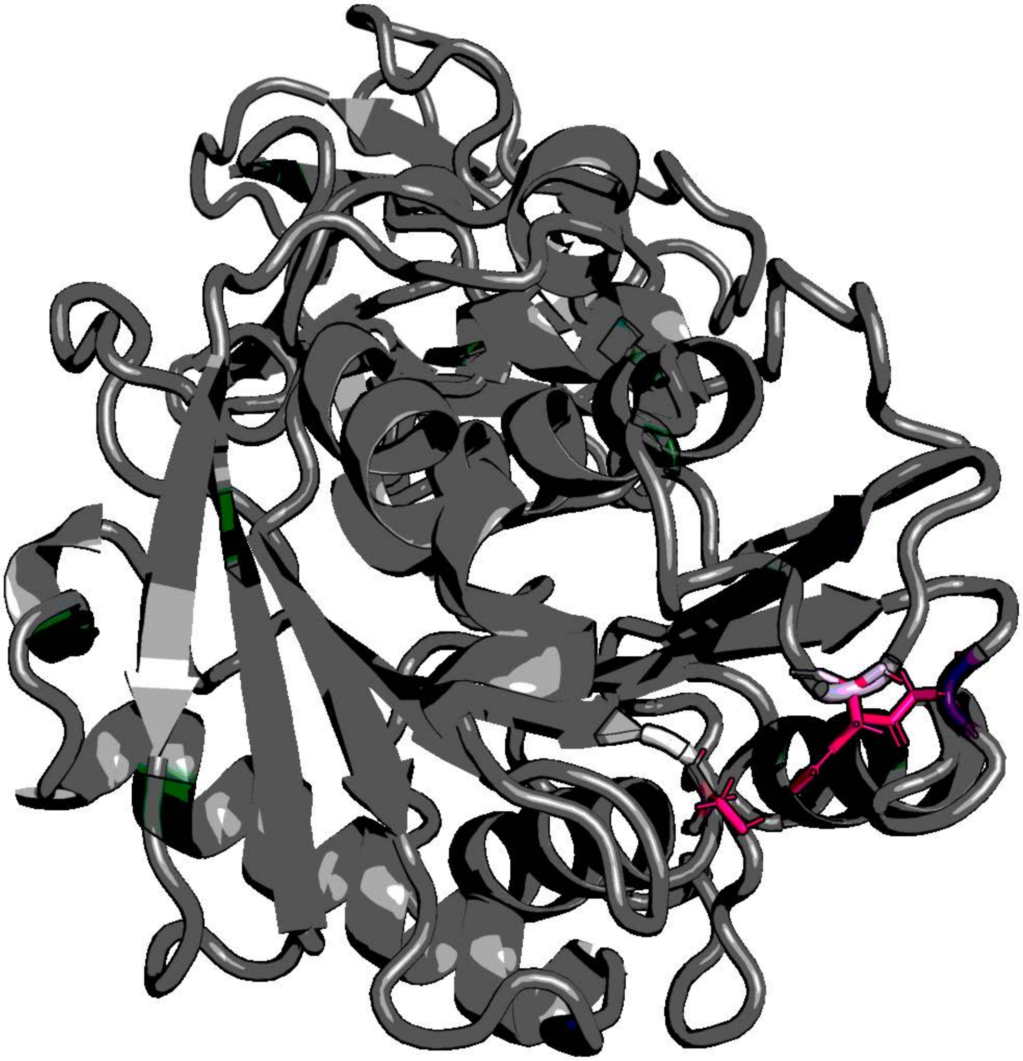

TEP093_highlights.png

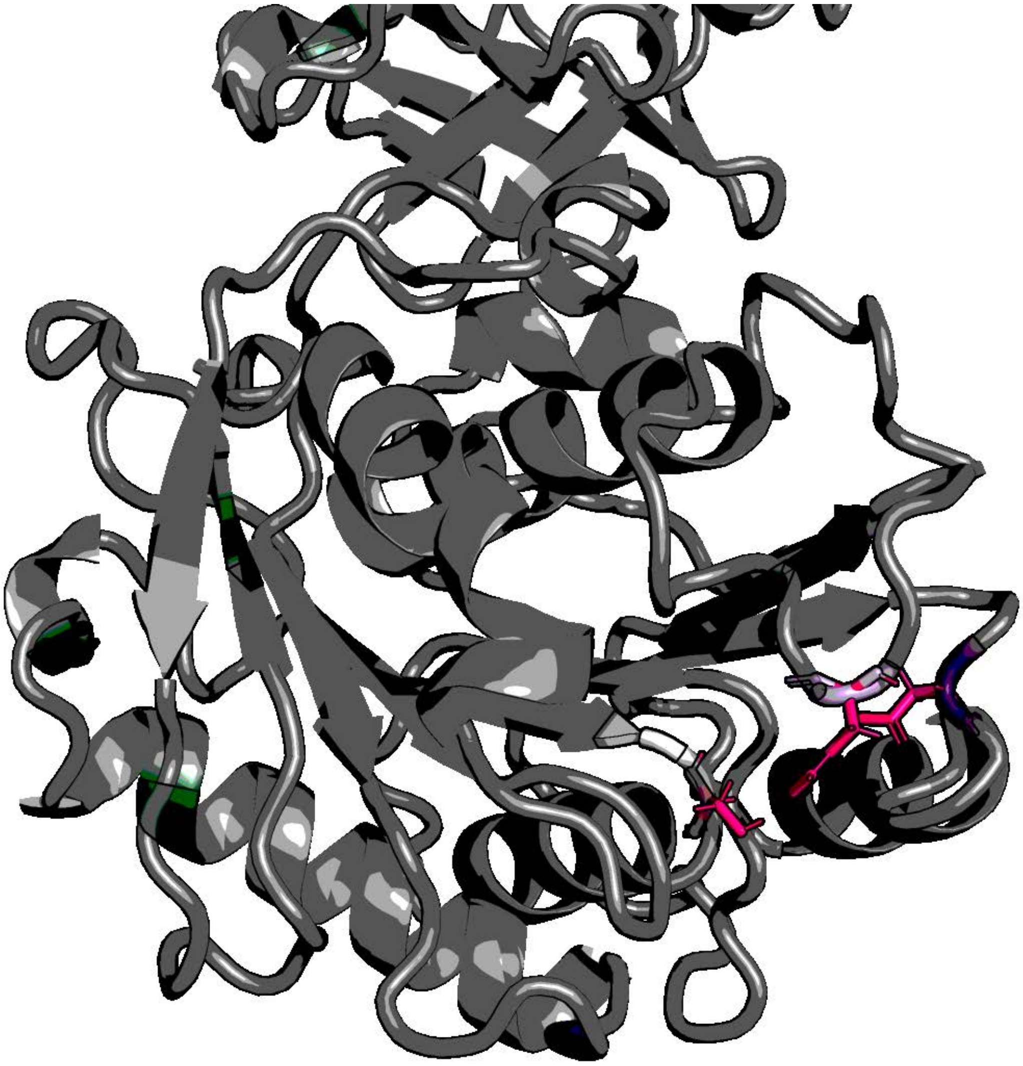

TEP097_highlights.png

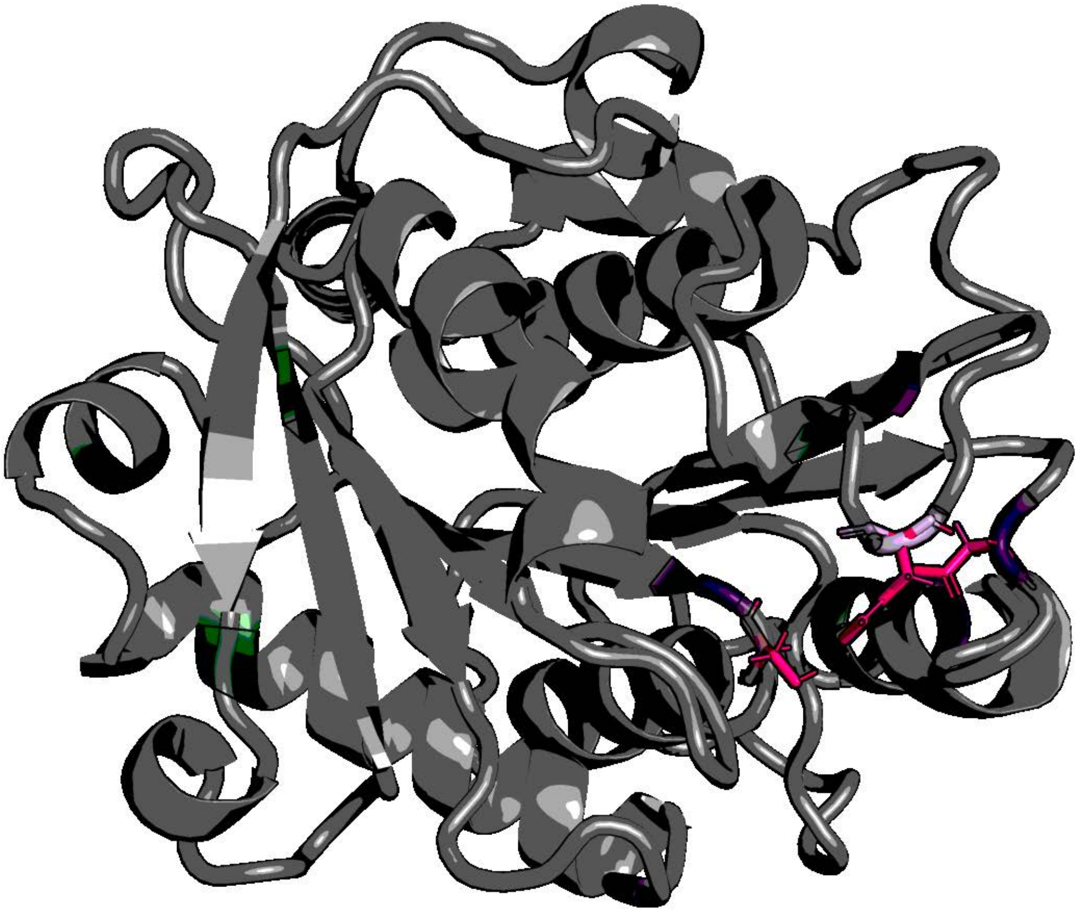

TEP101_highlights.png

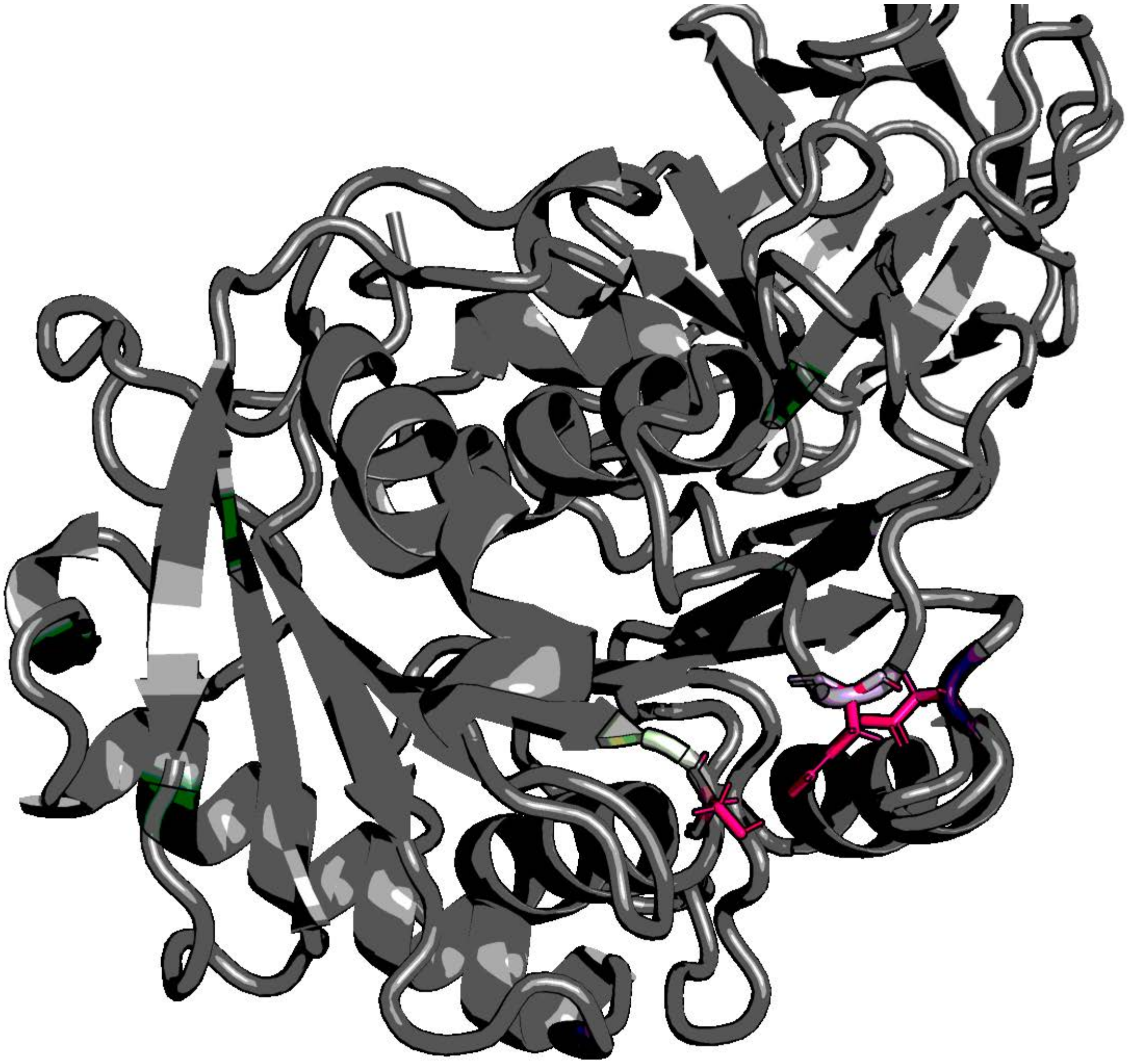

TEP102_highlights.png

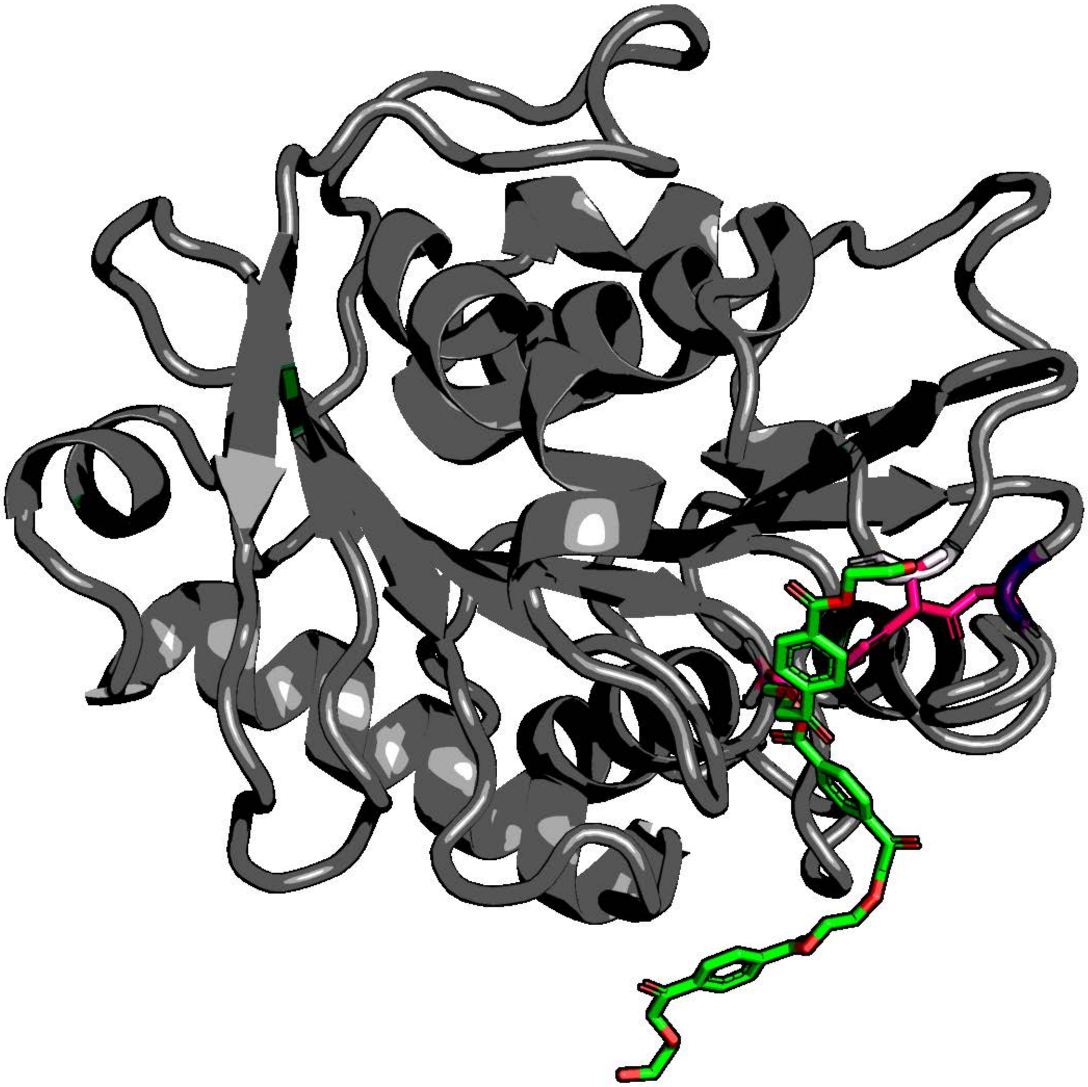

TEP103_highlights.png

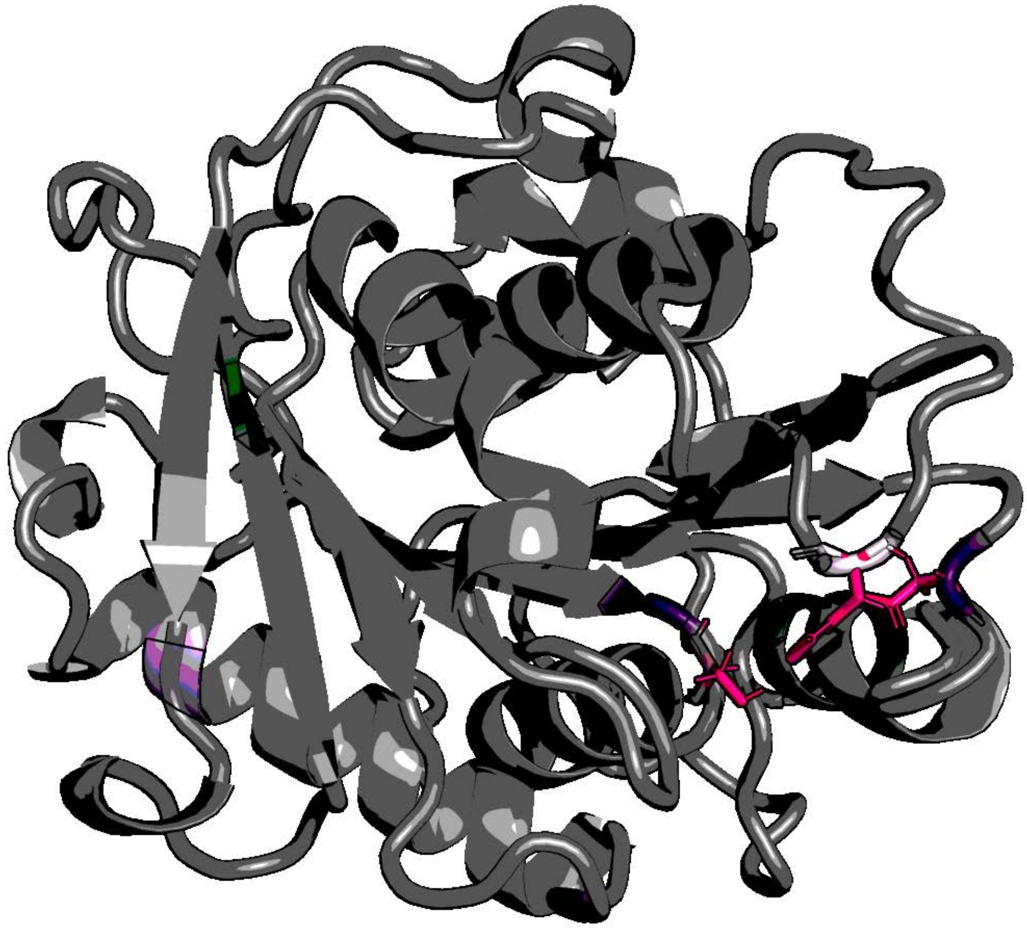

TEP105_highlights.png

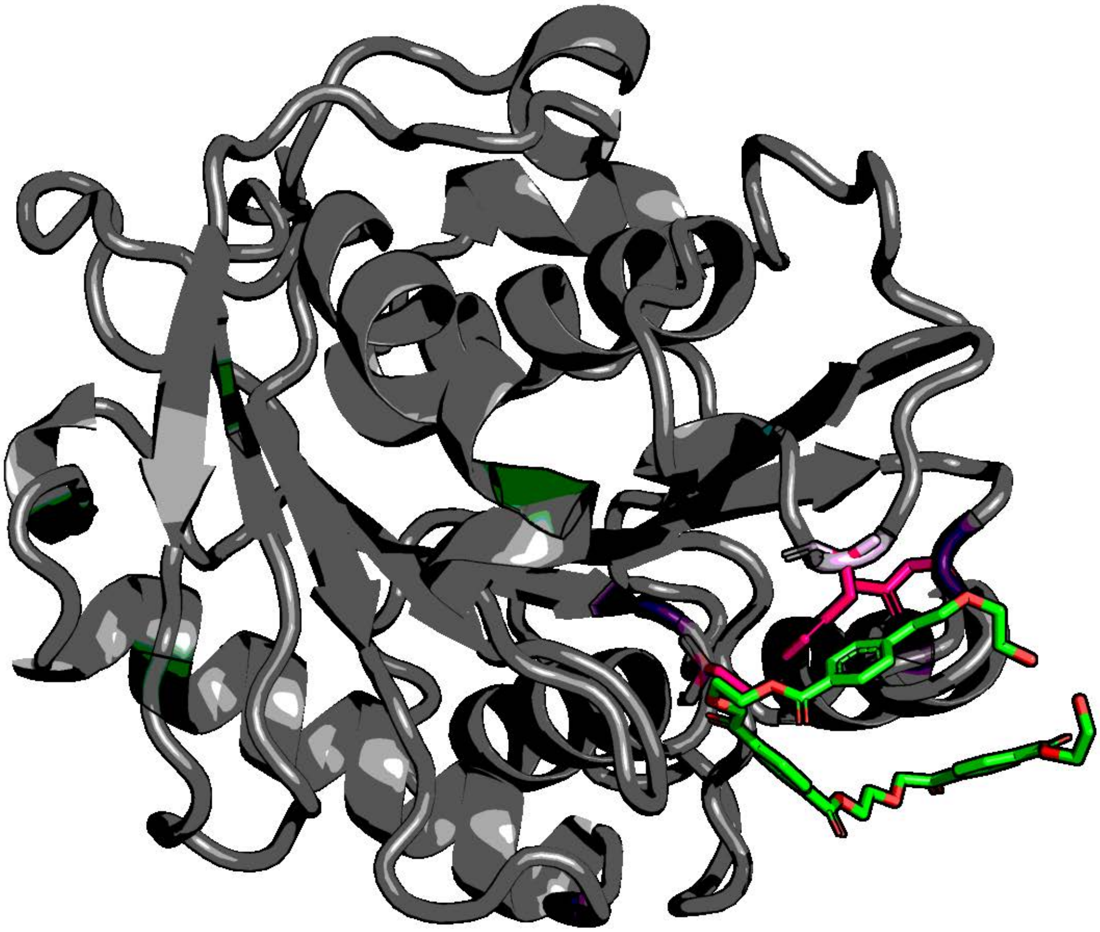

TEP109_highlights.png

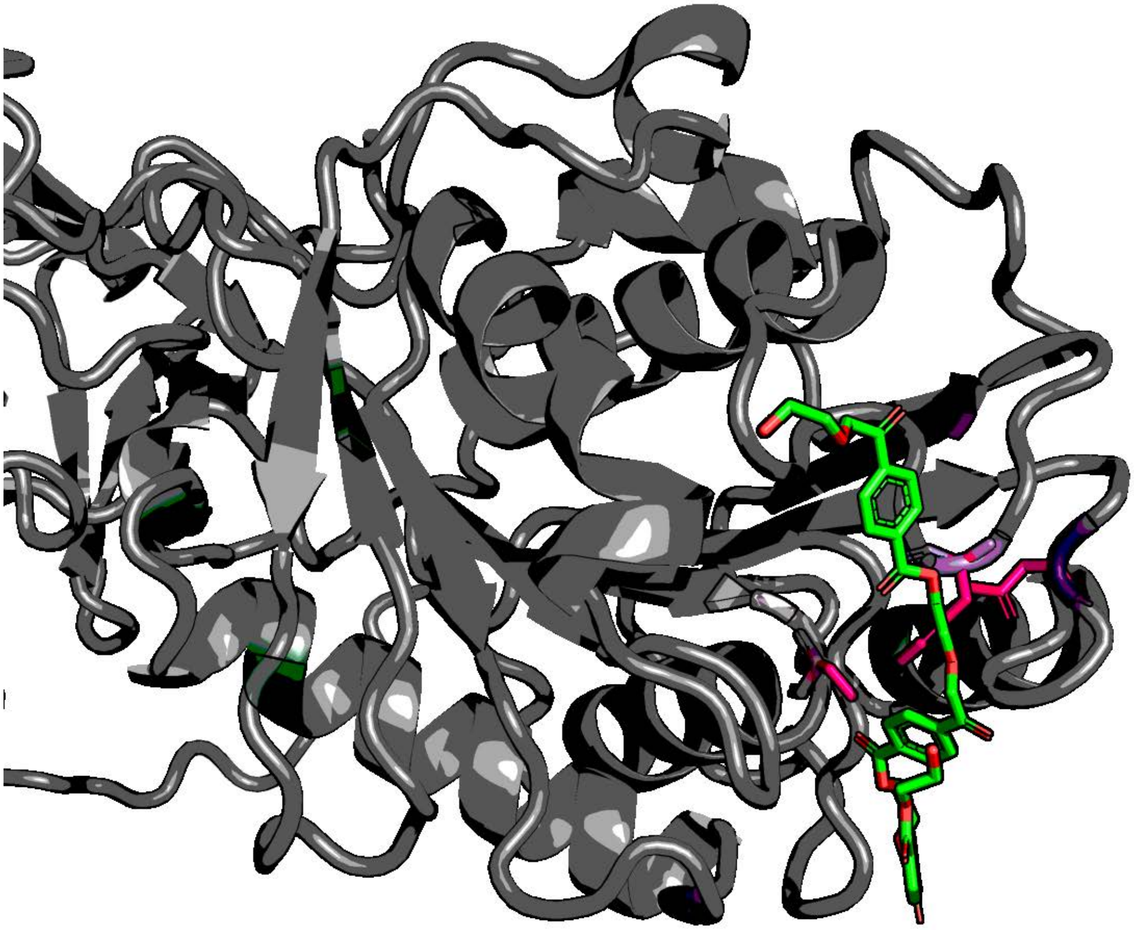

TEP111_highlights.png

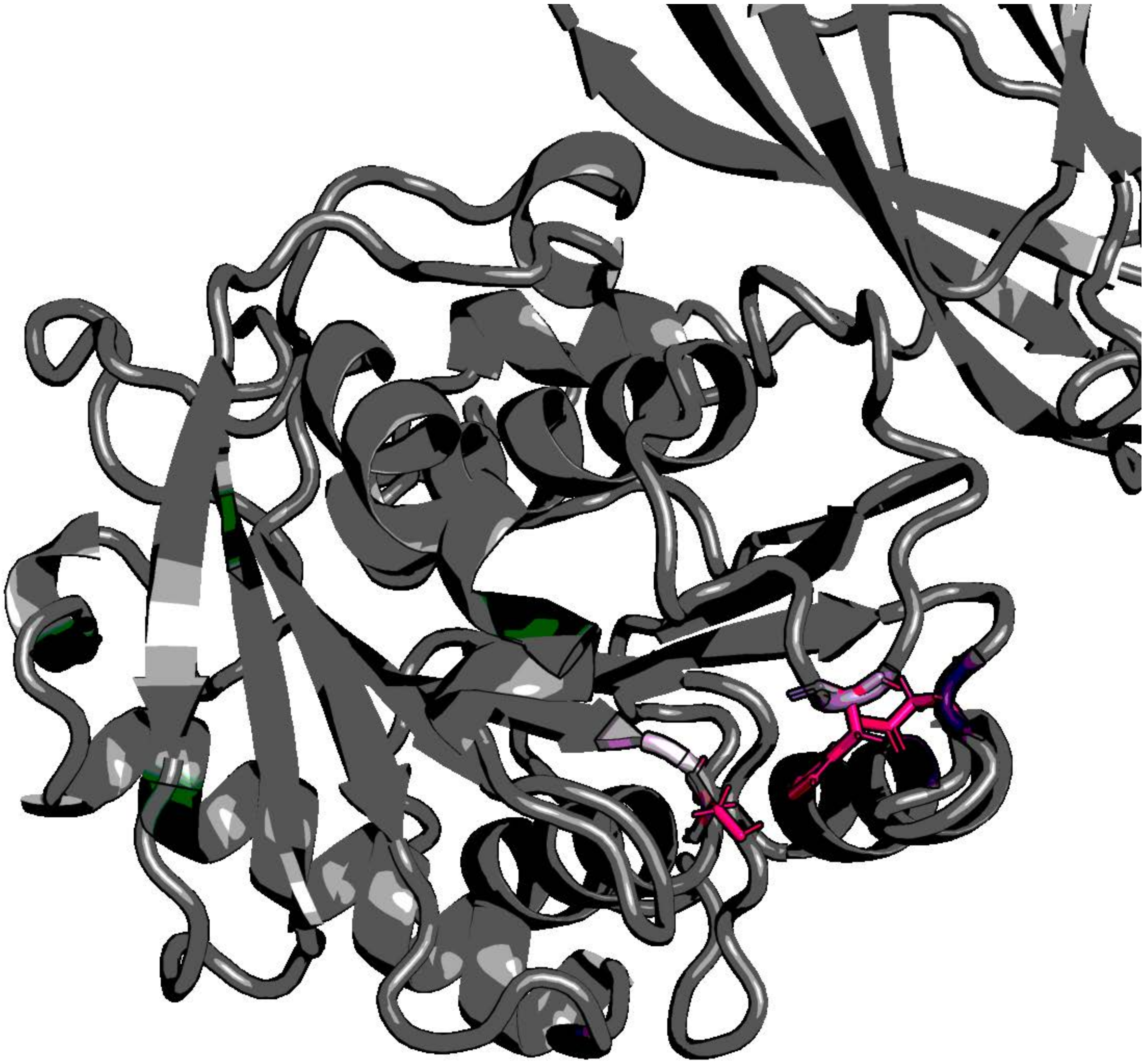

TEP114_highlights.png

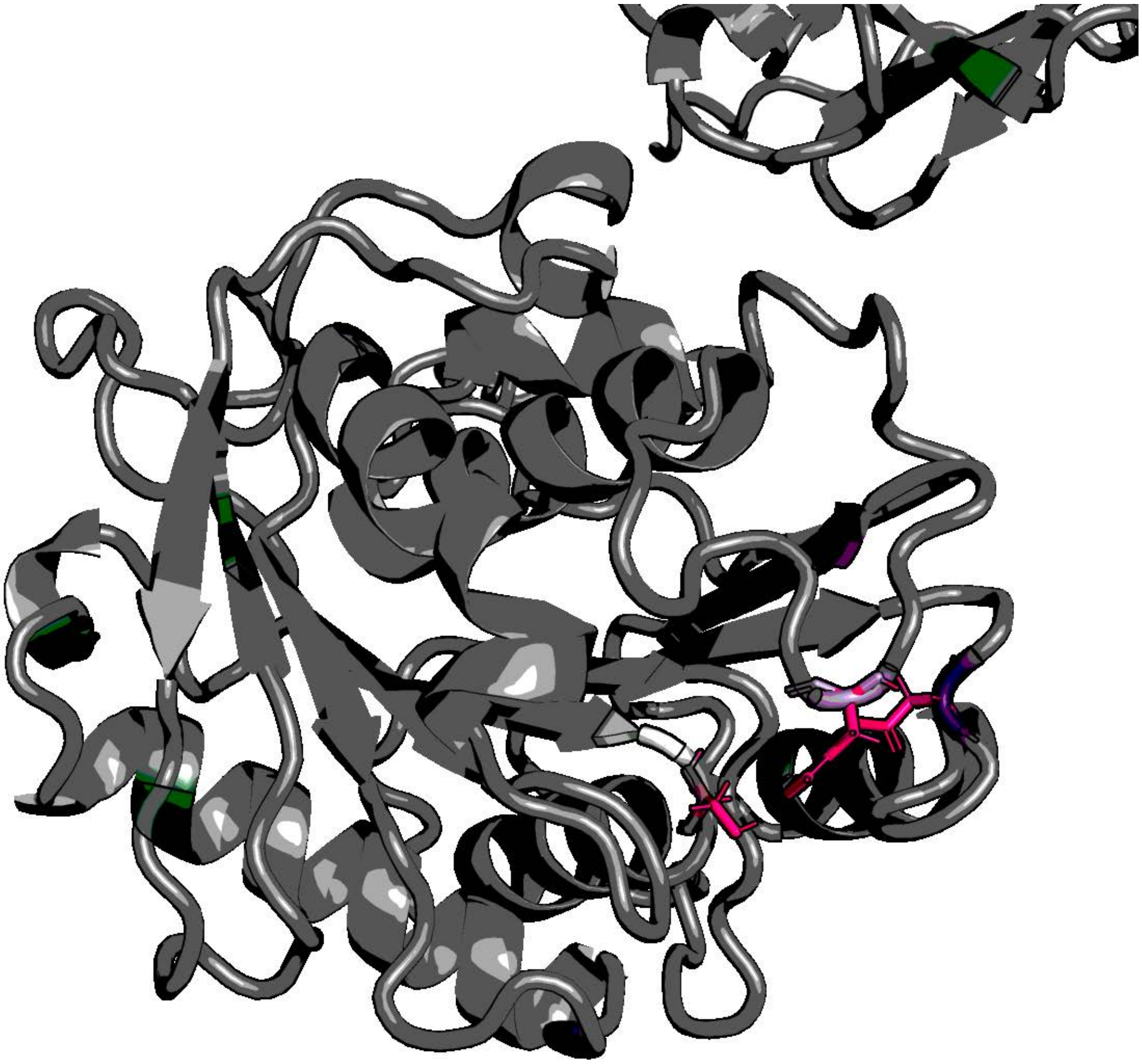

TEP115_highlights.png

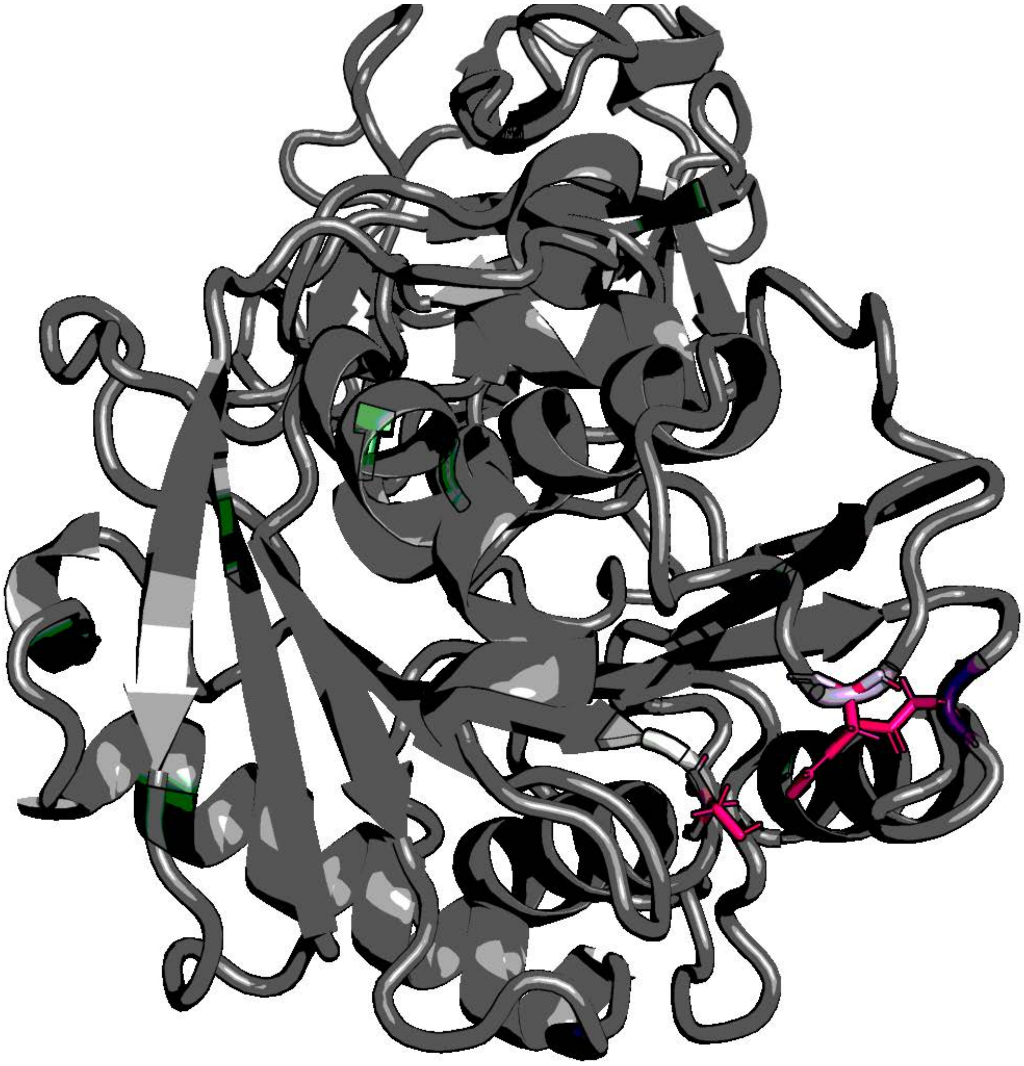

TEP131_highlights.png

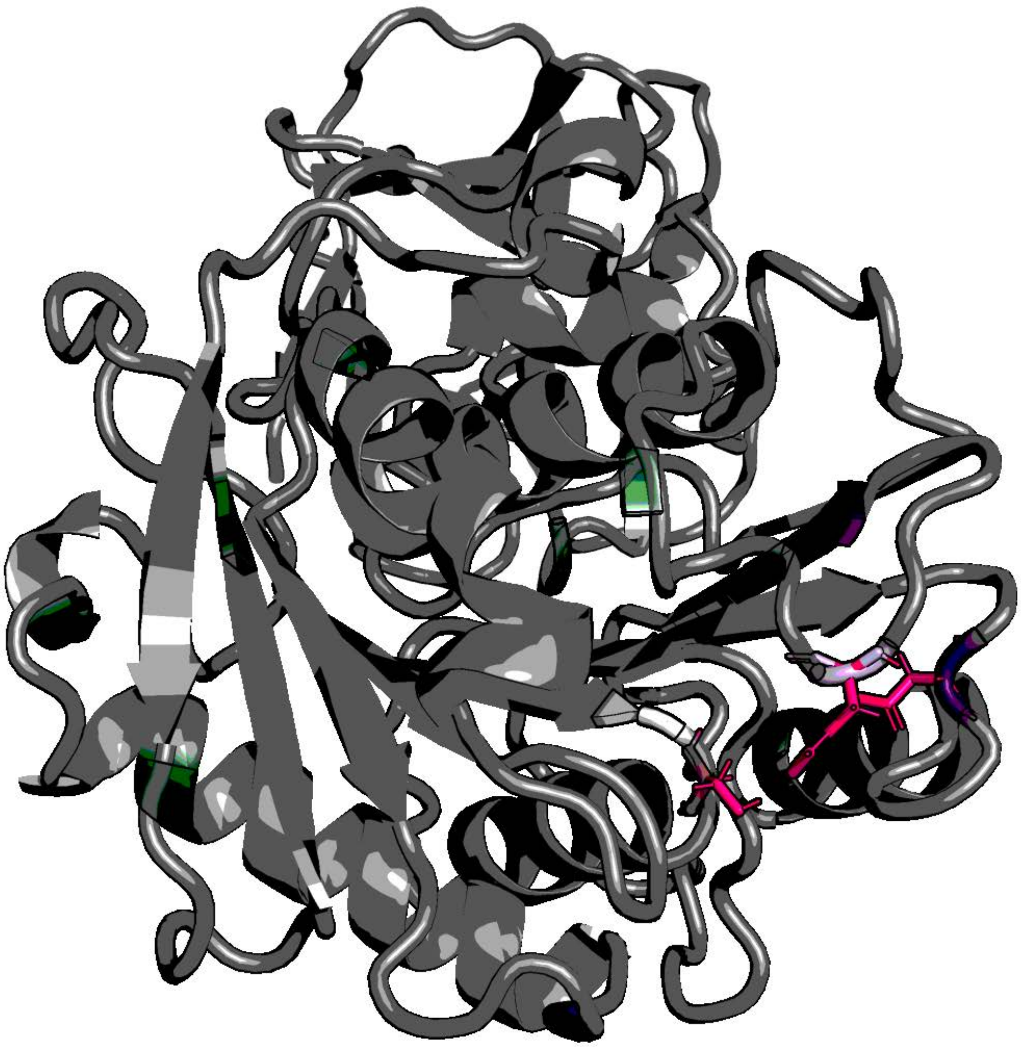

TEP163_highlights.png

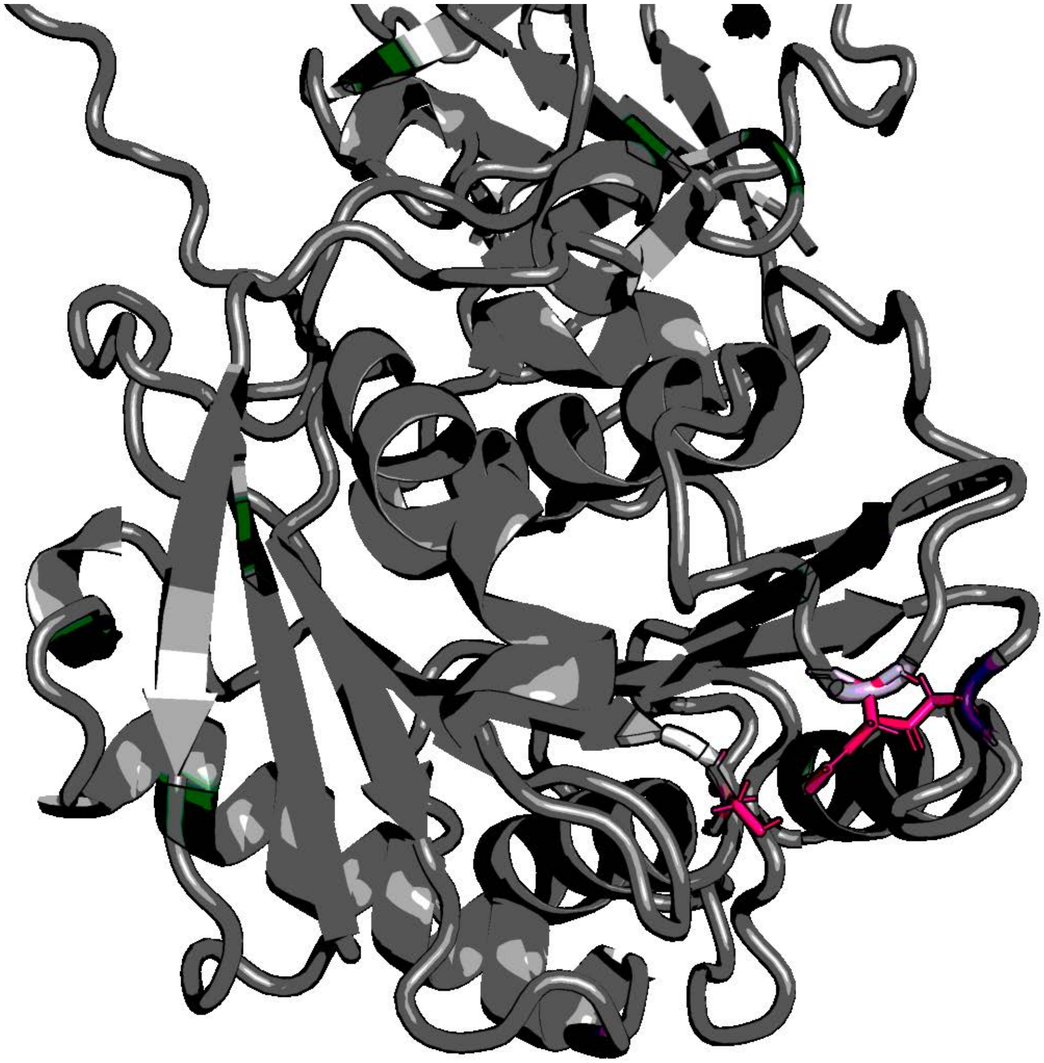

TEP164_highlights.png

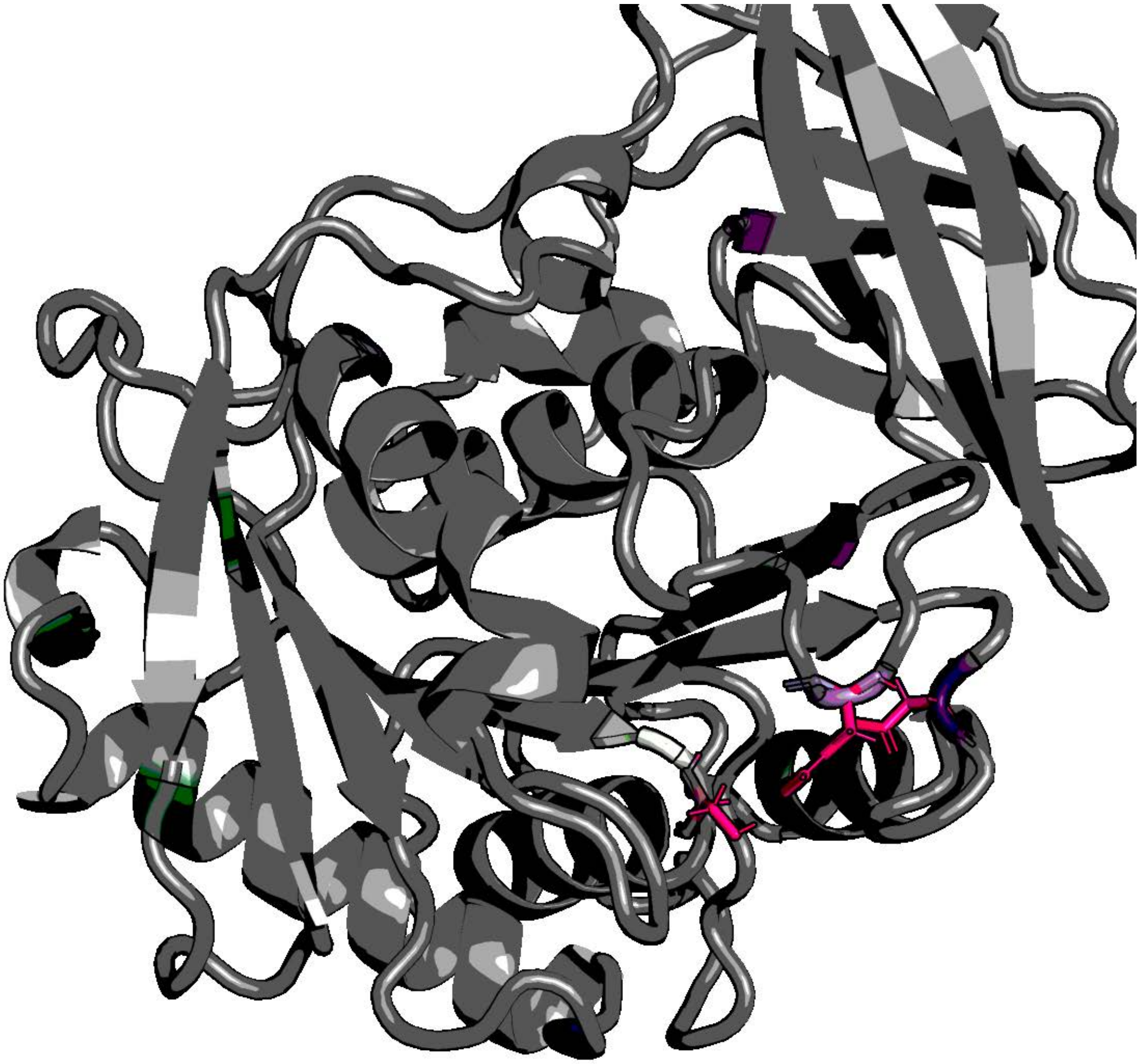

TEP165_highlights.png

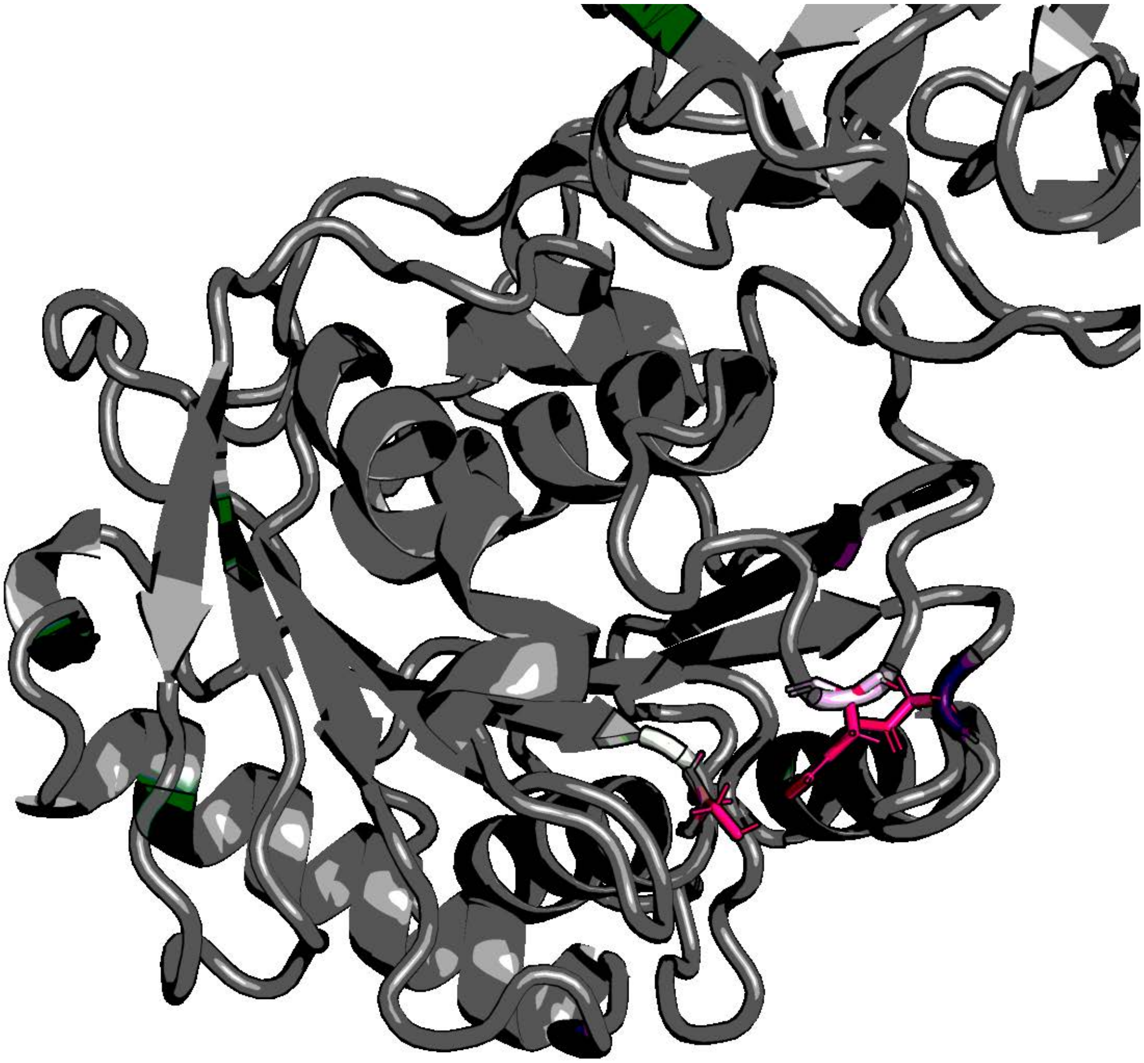

TEP168_highlights.png

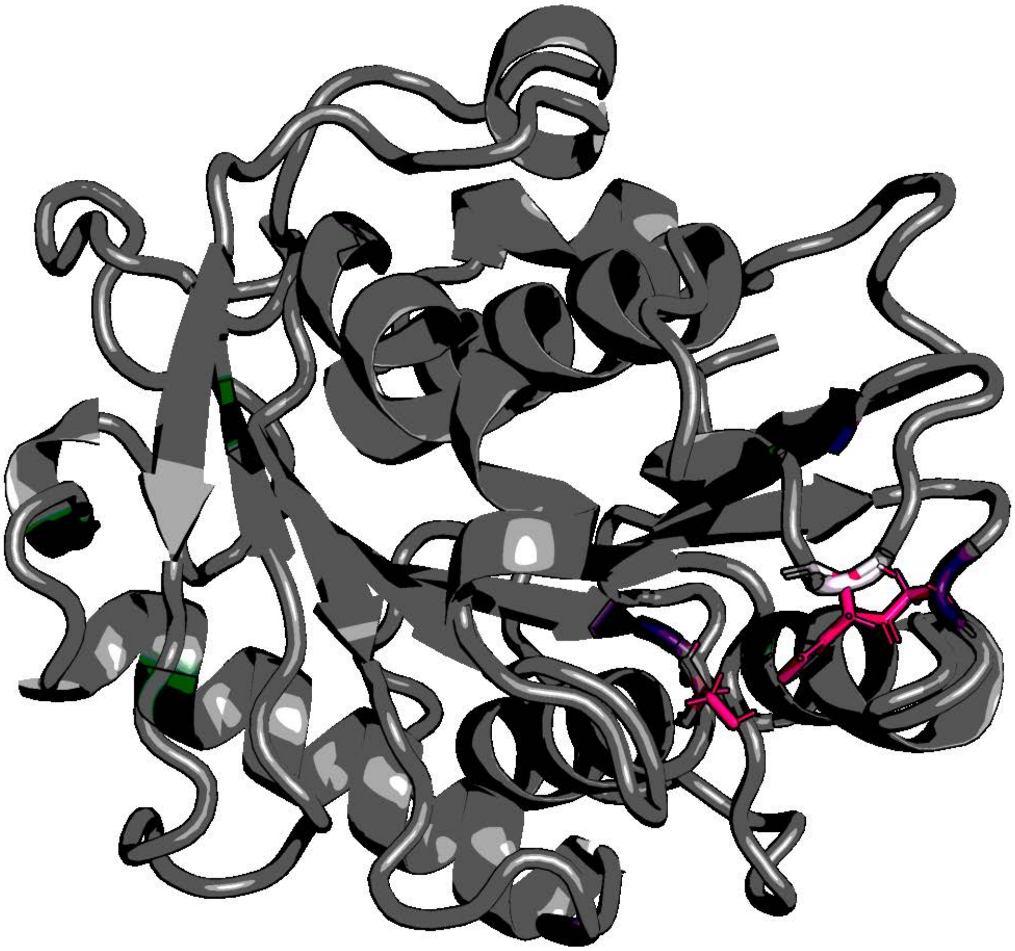

TEP178_highlights.png

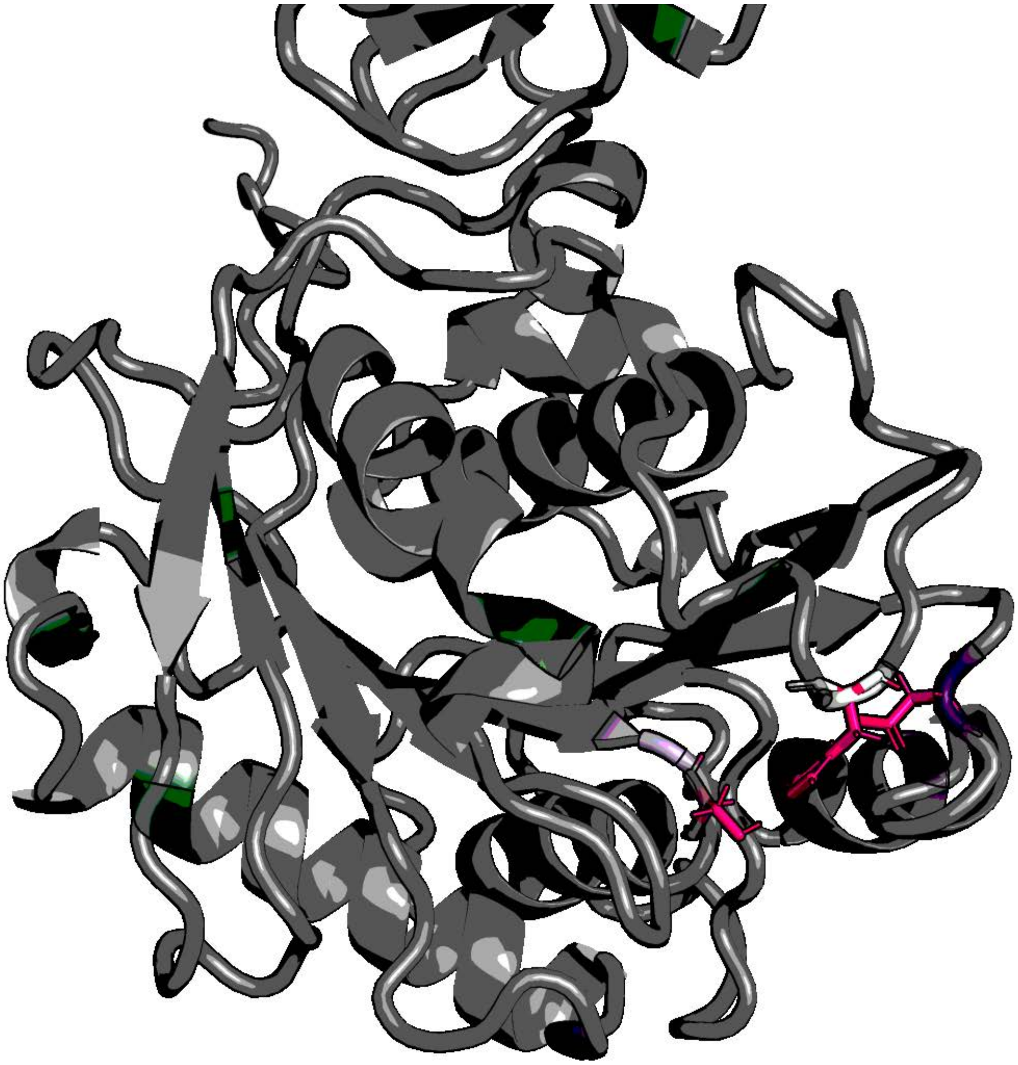

TEP181_highlights.png

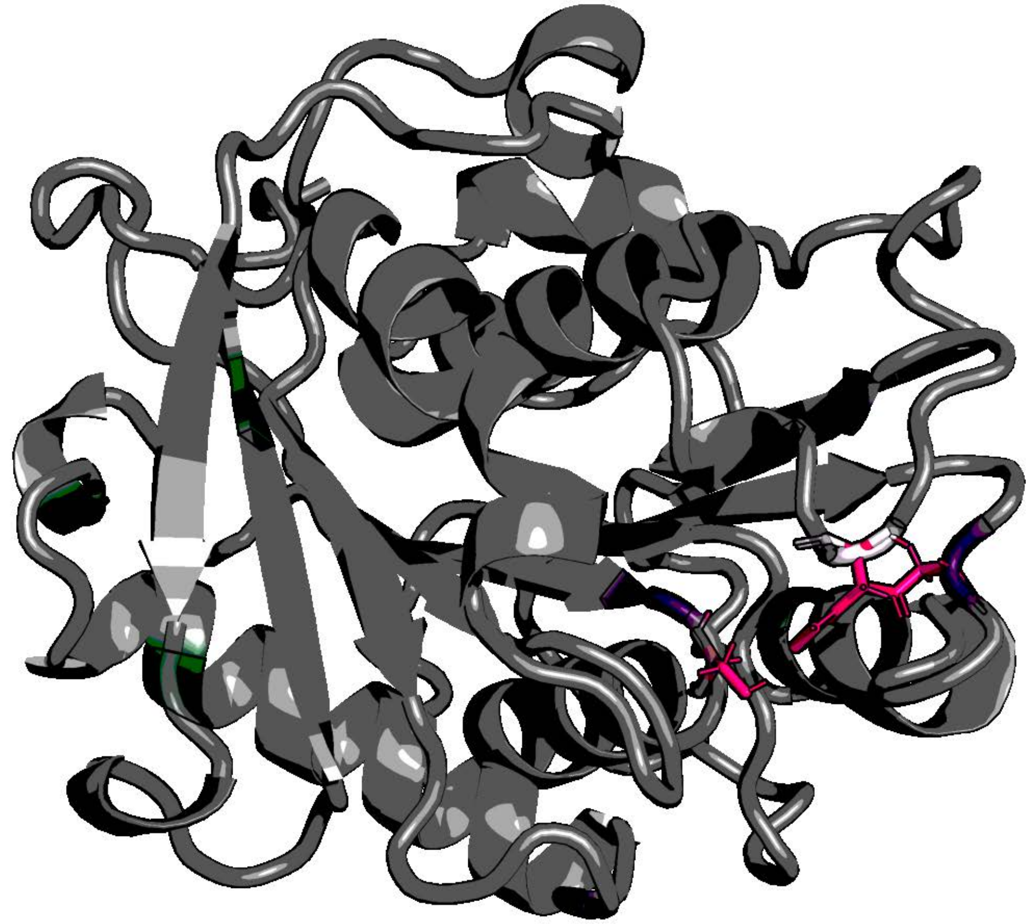

TEP182_highlights.png

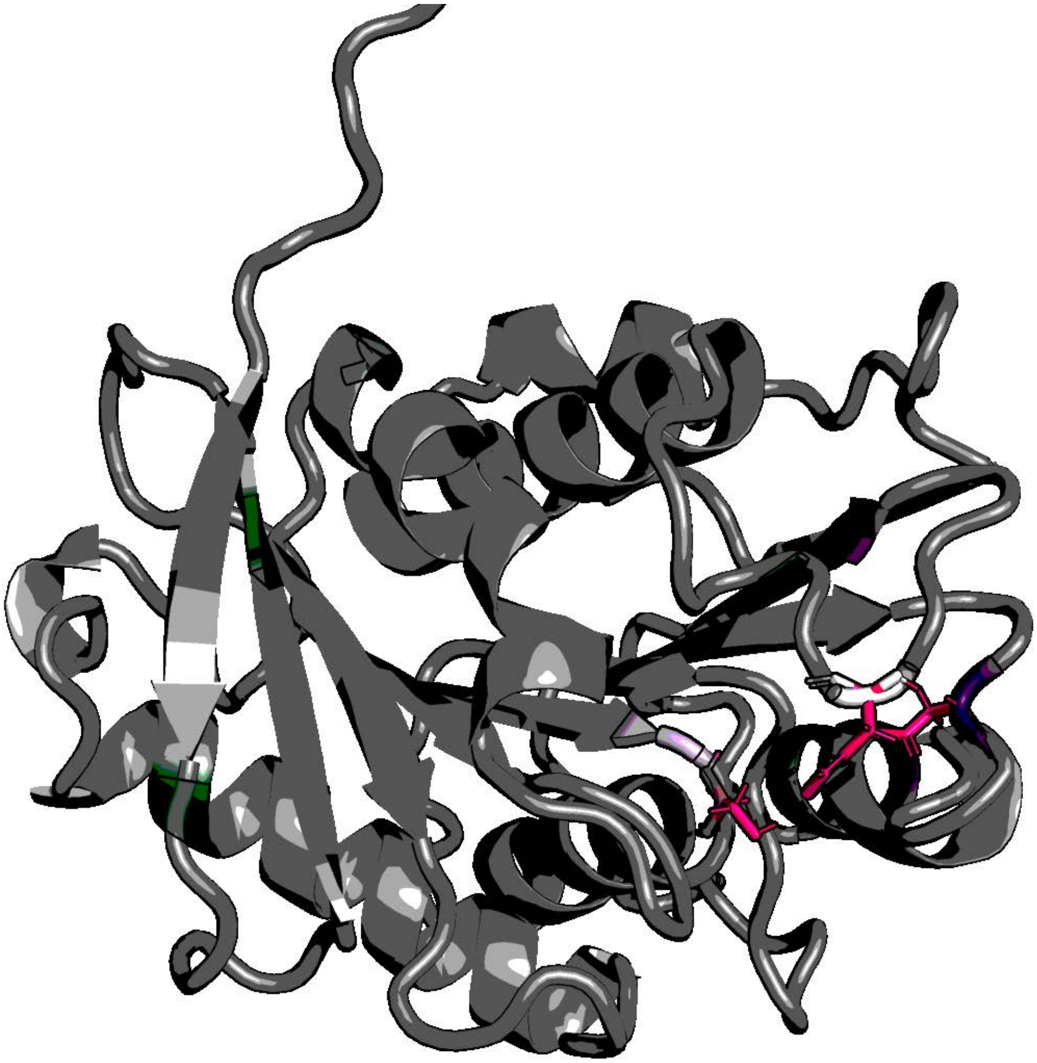

TEP189_highlights.png

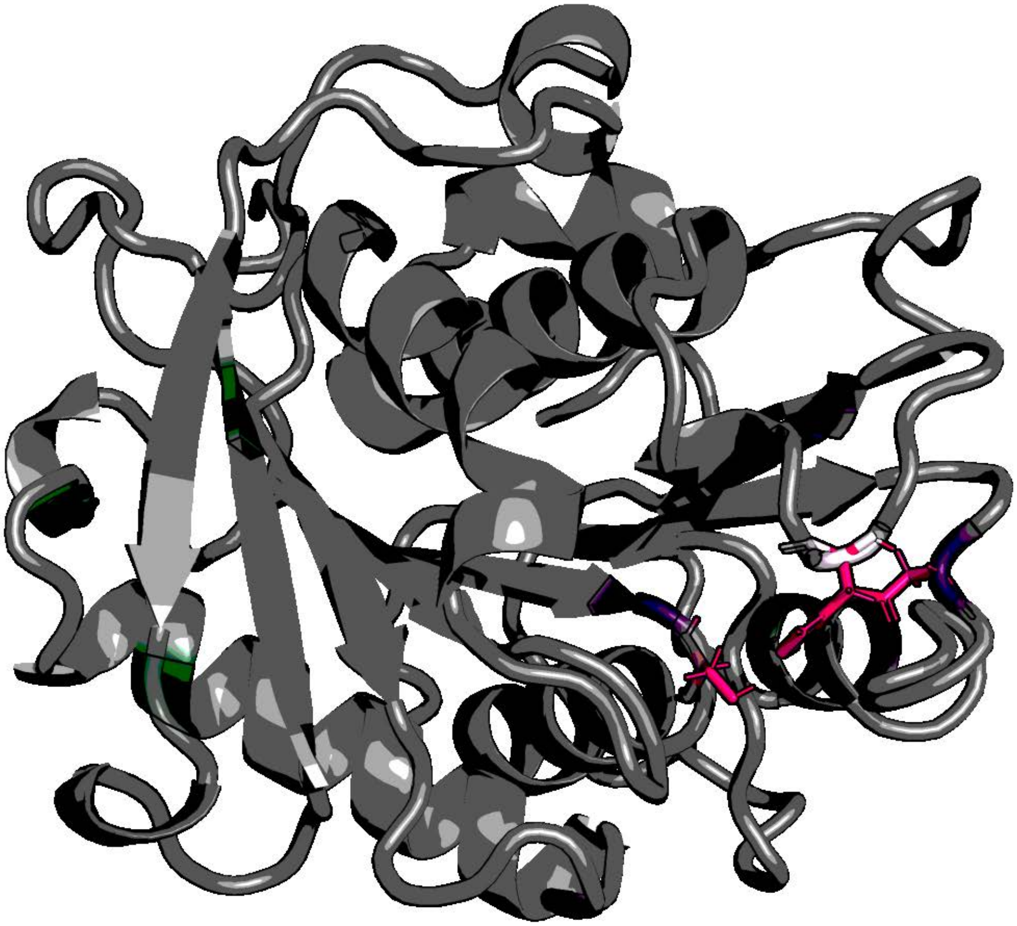

TEP194_highlights.png

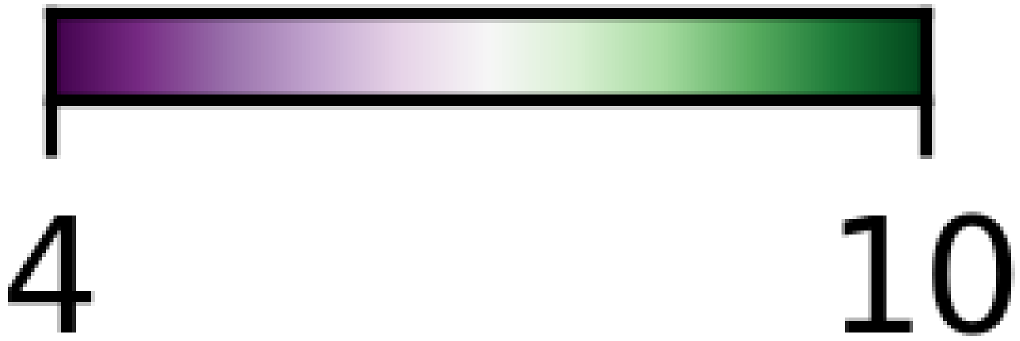

_LEGEND.png
